# iRSpot-TNCPseAAC: Identify Recombination Spots with Trinucleotide Composition and Pseudo Amino Acid Components

**DOI:** 10.3390/ijms15021746

**Published:** 2014-01-24

**Authors:** Wang-Ren Qiu, Xuan Xiao, Kuo-Chen Chou

**Affiliations:** 1 Computer Department, Jing-De-Zhen Ceramic Institute, Jingdezhen 333046, China; E-Mail: qiuone@163.com; 2 Information School, ZheJiang Textile & Fashion College, Ningbo 315211, China; 3 Center of Excellence in Genomic Medicine Research (CEGMR), King Abdulaziz University, Jeddah 21589, Saudi Arabia; E-Mail: kcchou@gordonlifescience.org; 4 Gordon Life Science Institute, Belmont, MA 02478, USA

**Keywords:** genome, DNA, recombination spots, hotspots, coldspots, trinucleotide composition, pseudo amino acid composition, web-server, iRSpot-TNCPseAAC

## Abstract

Meiosis and recombination are the two opposite aspects that coexist in a DNA system. As a driving force for evolution by generating natural genetic variations, meiotic recombination plays a very important role in the formation of eggs and sperm. Interestingly, the recombination does not occur randomly across a genome, but with higher probability in some genomic regions called “hotspots”, while with lower probability in so-called “coldspots”. With the ever-increasing amount of genome sequence data in the postgenomic era, computational methods for effectively identifying the hotspots and coldspots have become urgent as they can timely provide us with useful insights into the mechanism of meiotic recombination and the process of genome evolution as well. To meet the need, we developed a new predictor called “iRSpot-TNCPseAAC”, in which a DNA sample was formulated by combining its trinucleotide composition (TNC) and the pseudo amino acid components (PseAAC) of the protein translated from the DNA sample according to its genetic codes. The former was used to incorporate its local or short-rage sequence order information; while the latter, its global and long-range one. Compared with the best existing predictor in this area, iRSpot-TNCPseAAC achieved higher rates in accuracy, Mathew’s correlation coefficient, and sensitivity, indicating that the new predictor may become a useful tool for identifying the recombination hotspots and coldspots, or, at least, become a complementary tool to the existing methods. It has not escaped our notice that the aforementioned novel approach to incorporate the DNA sequence order information into a discrete model may also be used for many other genome analysis problems. The web-server for iRSpot-TNCPseAAC is available at http://www.jci-bioinfo.cn/iRSpot-TNCPseAAC. Furthermore, for the convenience of the vast majority of experimental scientists, a step-by-step guide is provided on how to use the current web server to obtain their desired result without the need to follow the complicated mathematical equations.

## 1. Introduction

**Figure 1.**
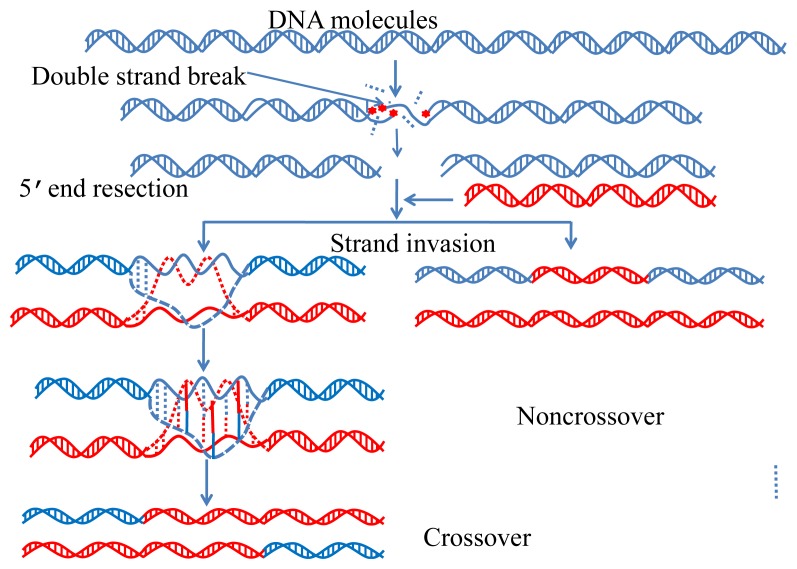
An illustration to show the process of meiosis and recombination in a DNA system. Adapted from [2].

Meiosis and recombination are two indispensible aspects for cell reproduction and growth (Figure 1). The former is a special type of cell division by which the genome is divided in half to generate daughter cells for participating in sexual reproduction, while the latter is to produce single-strand ends that can invade the homologous chromosome [1].

Recombination is initiated by double-strand breaks (or broken DNA ends); defecting in meiosis may lead to male infertility [3–5]. Meiotic recombination ensures accurate chromosome segregation during the first meiotic division and provides a mechanism to increase genetic heterogeneity among the meiotic products. Accordingly, identification of recombination spots may provide very useful information for in-depth understanding the reproduction and growth of cells.

In the past decades, a lot of global mapping studies have been performed to map double-strand break sites on chromosomes [6–13]. The following findings were observed through these studies for the meiotic recombination events. (i) They generally concentrate in 1:2.5 kilobase regions; (ii) They do not occur randomly across the entire genome but with a higher rate in some regions and lower in others; the former is a so-called “hotspot” while the latter, “coldspot”; (iii) They do not share a consensus sequence pattern.

With the rapid increasing number of genome sequences, it is important to address the following problem. Given a genome sequence, how can we predict which part of it is the hotspot for recombination, and which part is not?

Based on the nucleotide sequence contents, Liu *et al*. [14] proposed a computational method to deal with this problem. However, in their method no sequence-order effect whatsoever was taken into account, and, hence, its prediction power might be limited.

Actually, one of the most important, but also most difficult, problems in computational biology is how to formulate a biological sequence with a discrete model or a vector, yet still keep considerable sequence order information. This is as all the existing operation engines, such as covariance discriminant (CD) [15–20], neural network [21–23], support vector machine (SVM) [24–26], random forest [27,28], conditional random field [29], nearest neighbor (NN) [30,31], K-nearest neighbor (KNN) [32–34], OET-KNN (optimized evidence-theoretic k-nearest neighbors) [35–38], and Fuzzy K-nearest neighbor [39–43], can only handle vector, but not sequence, samples. However, a vector defined in a discrete model may completely lose all the sequence-order information.

To avoid completely losing the sequence-order information for proteins, the pseudo amino acid composition [44,45] or Chou’s pseudo amino acid components (PseAAC) [46] was proposed. Ever since the concept of PseAAC was proposed in 2001 [44], it has penetrated into almost all the areas of computational proteomics, such as identifying cysteine S-nitrosylation sites in proteins [29], predicting bacterial virulent proteins [47], predicting antibacterial peptides [48], identifying bacterial secreted proteins [49], predicting supersecondary structure [50], predicting protein subcellular location [51–59], predicting membrane protein types [60,61], discriminating outer membrane proteins [62], identifying antibacterial peptides [48], identifying allergenic proteins [63], predicting metalloproteinase family [64], predicting protein structural class [65], identifying GPCRs (G protein-coupled receptors) and their types [66,67], identifying protein quaternary structural attributes [68,69], predicting protein submitochondria locations [70–73], identifying risk type of human papillomaviruses [74], identifying cyclin proteins [75], predicting GABA(A) receptor proteins [76], classifying amino acids [77], predicting the cofactors of oxidoreductases [78], predicting enzyme subfamily classes [79], detecting remote homologous proteins [80], analyzing genetic sequences [81], predicting anticancer peptides [82], among many others (see a long list of papers cited in the References section of [83]). Recently, the concept of PseAAC was further extended to represent the feature vectors of nucleotides [15], as well as other biological samples [84–86]. As it has been widely and increasingly used, recently two powerful soft-wares, called “PseAAC-Builder” [87] and “propy” [88], were established for generating various special Chou’s pseudo-amino acid compositions, in addition to the web-server “PseAAC” [89], built in 2008.

Encouraged by the success of introducing PseAAC for proteins, recently, Chen *et al*. [25] proposed the pseudo dinucleotide composition or PseDNC to represent DNA sequences for identifying the recombination spots by counting some sequence effects, remarkably improving the prediction results in comparison with those by Liu *et al*. [14], without including any sequence information. However, in PseDNC, only the correlations of dinucleotides along a DNA sequence were considered, and, hence, some important sequence order effects might be missed.

The present study was initiated in an attempt to incorporate the long-range or global correlations of trinucleotides along a DNA sequences in hope to further improve the prediction quality in indentifying the recombination spots.

As demonstrated in a series of recent publications [24,42,90–92] and summarized in a comprehensive review [83], to establish a really useful statistical predictor for a biological system, one needs to consider the following procedures: (i) construct or select a valid benchmark dataset to train and test the predictor; (ii) formulate the biological samples with an effective mathematical expression that can truly reflect their intrinsic correlation with the target to be predicted; (iii) introduce or develop a powerful algorithm (or engine) to operate the prediction; (iv) properly perform cross-validation tests to objectively evaluate the anticipated accuracy of the predictor; and (v) establish a user-friendly web-server for the predictor that is accessible to the public. Below, let us elaborate how to deal with these procedures one-by-one.

## 2. Results and Discussion

### 2.1. Benchmark Dataset

The benchmark dataset *S* used in this study was taken from Liu *et al*. [14], which contains 490 recombination hotspots and 591 recombination coldspots, as can be formulated by:

(1) $$S=S^{+}\cup S^{-}$$

where subset *S*^+^ and *S*^−^ are respectively for the hot and cold spots, while ∪ represents the symbol for “union” in the set theory. For reader’s convenience, the 490 DNA sequences in *S*^+^ and 591 sequences in *S*^−^ are given in the Supplementary Information S1.

### 2.2. Formulate DNA Samples by Combining Trinucleotide Composition and Pseudo Amino Acid Components

Suppose a DNA sequence **D** with *L* nucleotides; *i.e*.,

(2) $$D=N_{1}N_{2}N_{3}N_{4}N_{5}N_{6}N_{7}\cdots N_{L}$$

where

(3) $$N_{i}\in \left\{\begin{array}{cccc} \text{A }(\text{adenine}), & \text{C}(\text{cytosine}) & \text{G }(\text{guanine}) & \text{T }(\text{thymine}) \end{array}\right\}$$

denotes the *i*-th (*i* = 1, 2, …, *L*) nucleotide in the DNA sequence. If the feature vector of the DNA sequence is formulated by its mononucleotide composition (MNC), we have:

(4) $$\begin{array}{l} D={\left[\begin{array}{cccc} f(\text{A}) & f(\text{C}) & f(\text{G}) & f(\text{T}) \end{array}\right]}^{T}\\ =\left[\begin{array}{cccc} f_{1}^{\left(1\right)} & f_{2}^{\left(1\right)} & f_{3}^{\left(1\right)} & f_{4}^{\left(1\right)} \end{array}\right] \end{array}$$

where $f_{1}^{\left(1\right)}=f(\text{A}),\;f_{2}^{\left(1\right)}=f(\text{C}),\;f_{3}^{\left(1\right)}=f(\text{G})$, and $f_{4}^{\left(1\right)}=f(\text{T})$ are the normalized occurrence frequencies of adenine (A), cytosine (C), guanine (G), and thymine (T), respectively, in the DNA sequence; and the symbol **T** is the transpose operator. As we can see from Equation (4), all the sequence order information is missed if using MNC to represent a DNA sequence. If using the dinucleotide composition (DNC) to represent the DNA sequence, instead of the four components as shown in Equation (4), the corresponding feature vector will contain 4 × 4 = 16 components, as given below:

(5) $$\begin{array}{l} D={\left[\begin{array}{cccccc} f(\text{AA}) & f(\text{AC}) & f(\text{AG}) & f(\text{AT}) & \cdots & f(\text{TT}) \end{array}\right]}^{T}\\ ={\left[\begin{array}{cccccc} f_{1}^{\left(2\right)} & f_{2}^{\left(2\right)} & f_{3}^{\left(2\right)} & f_{4}^{\left(2\right)} & \cdots & f_{16}^{\left(2\right)} \end{array}\right]}^{T} \end{array}$$

where $f_{1}^{\left(2\right)}=f(\text{AA})$ is the normalized occurrence frequency of AA in the DNA sequence; $f_{2}^{\left(2\right)}=f(\text{AC})$, that of AC; $f_{3}^{\left(2\right)}=f(\text{AG})$, that of AG; and so forth. If represented by the trinucleotide composition (TNC), the corresponding feature vector will contain 4×4×4 = 4^3^ = 64 components, as given below:

(6) $$\begin{array}{l} D={\left[\begin{array}{cccccc} f(\text{AAA}) & f(\text{AAC}) & f(\text{AAG}) & f(\text{AAT}) & \cdots & f(\text{TTT}) \end{array}\right]}^{T}\\ ={\left[\begin{array}{cccccc} f_{1}^{\left(3\right)} & f_{2}^{\left(3\right)} & f_{3}^{\left(3\right)} & f_{4}^{\left(3\right)} & \cdots & f_{64}^{\left(3\right)} \end{array}\right]}^{T} \end{array}$$

where $f_{1}^{\left(3\right)}=f(\text{AAA})$ is the normalized occurrence frequency of AAA in the DNA sequence; $f_{2}^{\left(3\right)}=f(\text{AAC})$, that of AAC; and so forth. Generally speaking, if a DNA sequence is represented by the *K*-tuple nucleotide composition, the corresponding vector D for the DNA sequence will contain 4*^K^* components; *i.e*.,

(7) $$D={\left[\begin{array}{cccccc} f_{1}^{\left(K\right)} & f_{2}^{\left(K\right)} & f_{3}^{\left(K\right)} & f_{4}^{\left(K\right)} & \cdots & f_{4^{K}}^{\left(K\right)} \end{array}\right]}^{T}$$

As we can see from Equations (5–7), with increasing the tuple number, although the base sequence-order information within a local or very short range could be gradually included, none of the global or long-range sequence-order information would be reflected by the formulation.

Actually, in computational proteomics, we have also faced exactly the same situation; *i.e.*, although the dipeptide composition, tripeptide composition, and *K*-tuple peptide composition were used by many investigators to represent protein sequences by incorporating their local sequence order information [93–97], their global or long-range sequence order information still could not be reflected. As mentioned above, to deal with this kind of problems in proteomics, the concept of PseAAC [44,45] was introduced.

Stimulated by the PseAAC approach [44,45] in computational proteomics, below let us propose a novel feature vector to represent the DNA sequence (cf. Equation (2)) by combining its TNC (see Equation (2)) and the pseudo amino acid components of its translated protein chain.

**Figure 2.**
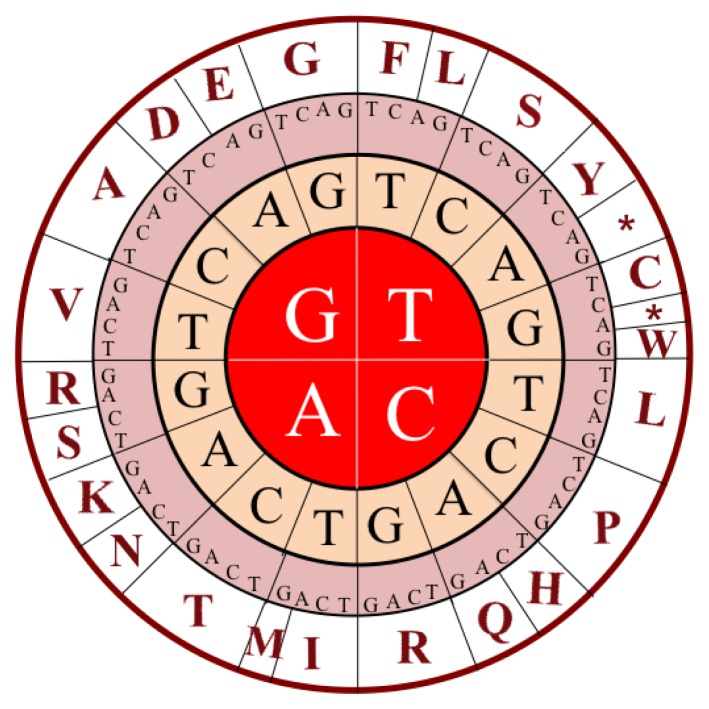
A graph to show how a DNA codon of three nucleotides is converted to an amino acid. The characters in the first three rings from the center represent four bases in DNA, while those in the fourth ring represent the single-letter codes of the 20 native amino acids in protein. The symbol * means the “Stop” sign.

As is well known, three nucleotides encode an amino acid (see Figure 2). Thus, according the conversion table from DNA codons to amino acids (Table 1), the DNA sequence in Equation (2) can be translated into a protein sequence expressed by:

(8) $$P=A_{1}A_{2}A_{3}\cdots A_{L*}$$

with

(9) $$\{\begin{array}{l} A_{i}\in \{20\;\text{native amino acids}\}\\ L*=\text{Int}\{L/3\} \end{array}$$

where the symbol “Int” is an integer truncation operator meaning to take the integer part for the number in the brackets immediately after it.

Now, according to the formulation of Chou’s PseAAC approach [44,45], for the protein chain of Equation (8), we have:

(10) $$\{\begin{array}{c} \theta_{1}=\frac{1}{L*-1}\sum_{i=1}^{L*-1}\Theta (A_{i},A_{i+1})\\ \theta_{2}=\frac{1}{L*-2}\sum_{i=1}^{L*-2}\Theta (A_{i},A_{i+2})\\ \theta_{3}=\frac{1}{L*-3}\sum_{i=1}^{L*-3}\Theta (A_{i},A_{i+3})\\ \vdots \\ \theta_{\lambda }=\frac{1}{L*-\lambda }\sum_{i=1}^{L*-1}\Theta (A_{i},A_{i+\lambda }) \end{array}\;\;\;(\lambda <L*)$$

where *θ_k_* (*k* = 1,2,3, ···, *λ*) is called the *k*-th tier correlation factor that reflects the sequence order correlation between all the *k*-th most contiguous residues along a protein chain. In this study, the correlation function in Equation 10 is given by:

(11) $$\Theta (A_{i},A_{j})=\frac{1}{6}\sum_{n=1}^{6}{\left[H_{n}(A_{j})-H_{n}(A_{i})\right]}^{2}$$

where *H_n_* (*A_j_*) (*n* = 1,2,···, 6) is the six physicochemical properties of amino acid A*_j_*; they are, respectively, hydrophobicity, hydrophilicity, side-chain mass, pK1 (α-COOH), pK2 (NH3), and PI. Note that before substituting these physicochemical values into Equation (11), they were all subjected to a standard conversion as described by the following equation:

(12) $$H_{n}(A_{i})=\frac{H_{n}^{0}(A_{i})-\langle H_{n}^{0}\rangle }{\text{SD}(H_{n}^{0})}$$

where *H_n_* (*A_i_*) (*n* = 1,2,···, 6) is the *n*-th original physicochemical property value for the amino acid A*_i_* as given in Table 2, the symbol < and > means taking the average of the quantity therein over 20 native amino acids, and SD means the corresponding standard deviation. Listed in Table 3 are the converted values obtained by Equation (12) that will have a zero mean value over the 20 native amino acids, and will remain unchanged if going through the same conversion procedure again.

By combining the *λ* correlation factors with the 64 components in TNC (see Equation (6)), the DNA sequence is formulated by:

(13) $$D={\left[\begin{array}{ccccccc} d_{1} & d_{2} & \cdots & d_{64} & d_{64+1} & \cdots & d_{64+\lambda } \end{array}\right]}^{T}$$

where:

(14) $$d_{u}=\{\begin{array}{ll} \frac{f_{u}^{\left(3\right)}}{\sum_{i=1}^{64}f_{i}^{\left(3\right)}+w\sum_{k=1}^{\lambda }\theta_{k}}, & \left(1\leq u\leq 64\right)\\ \frac{w\theta_{u-64}}{\sum_{i=1}^{64}f_{i}^{\left(3\right)}+w\sum_{k=1}^{\lambda }\theta_{k}}, & \left(64+1\leq u\leq 64+\lambda \right) \end{array}$$

where *w* is the weight factor which is determined by optimizing the outcome as will be mentioned later. The rationale of using Equation (13) to represent the DNA sequence is that the local or short-range sequence order effect can be directly reflected via the occurrence frequencies of its 64 trinucleotides, while the global or long-range sequence order effect can be indirectly reflected via the *λ* pseudo amino acid components of its translated protein chain. As three nucleotides encode an amino acid, the above approach is both quite rational and natural.

### 2.3. Use Support Vector Machine as an Operation Engine

Support vector machine (SVM) has been widely to make classification prediction (see, e.g., [24,102–105]. The basic idea of SVM is to transform the input data into a high dimensional feature space and then determine the optimal separating hyperplane. A brief introduction about the formulation of SVM was given in [103,106]. Here, the DNA samples as formulated by Equation (13) were used as inputs for the SVM. Its software was downloaded from the LIBSVM package [107,108], which provided a simple interface. Due to this advantages, the users can easily perform classification prediction by properly selecting the built-in parameters *C* and *γ*. In order to maximize the performance of the SVM algorithm, the two parameters in the RBF kernel were preliminarily optimized through a grid search strategy in this study. To obtain the optimized parameters, the search function “SVMcgForClass” was downloaded from http://www.matlabsky.com.

The predictor obtained via the aforementioned procedures is called iRSpot-TNCPseAAC, where “i” means “identify”, “RSpot” means “Recombination Spots”, while TNCPseAAC means a combination of “Tri-Nucleotide Composition” and “Pseudo Amino Acid Components.”

To objectively evaluate the quality of a new predictor, one should use proper metrics [109] and rigorous cross-validation [83] to test it. Below, let us address these problems.

### 2.4. Four Different Metrics for Measuring the Prediction Quality

In literature, the following metrics are often used for examining the performance quality of a predictor:

(15) $$\{\begin{array}{l} Sn=\frac{TP}{TP+FN}\\ Sp=\frac{TN}{TN+FP}\\ Acc=\frac{TP+TN}{TP+TN+FP+FN}\\ MCC=\frac{\left(TP\times TN)-(FP\times FN\right)}{\sqrt{\left(TP+FP)(TP+FN)(TN+FP)(TN+FN\right)}} \end{array}$$

where *TP* represents the number of the true positive; *TN*, the number of the true negative; *FP*, the number of the false positive; *FN*, the number of the false negative; *Sn*, the sensitivity; *Sp*, the specificity; Acc, the accuracy; MCC, the Mathew’s correlation coefficient. To most biologists, however, the four metrics as formulated in Equation (15) are not quite intuitive and easier-to-understand, particularly for the Mathew’s correlation coefficient. Here let us adopt the formulation proposed recently [25,29] based on the Chou’s symbol and definition [110]; *i.e.*,

(16) $$\{\begin{array}{l} Sn=1-\frac{N_{-}^{+}}{N^{+}}\\ Sp=1-\frac{N_{+}^{-}}{N^{-}}\\ Acc=1-\frac{N_{-}^{+}+N_{+}^{-}}{N^{+}+N^{-}}\\ Mcc=\frac{1-\left(\frac{N_{-}^{+}+N_{+}^{-}}{N^{+}+N^{-}}\right)}{\sqrt{\left(1+\frac{N_{+}^{-}-N_{-}^{+}}{N^{+}}\right)\left(1+\frac{N_{-}^{+}-N_{+}^{-}}{N^{-}}\right)}} \end{array}$$

where *N*^+^ is the total number of the hotspot samples investigated while $N_{-}^{+}$ the number of the hotspot samples incorrectly predicted as coldspots; *N*^−^ the total number of the coldspot samples investigated while $N_{+}^{-}$ the number of the coldspot samples incorrectly predicted as the hotspots [111].

Now, it can be clearly seen from Equation (16) that when $N_{-}^{+}=0$ meaning none of the hotspots was incorrectly predicted to be a coldspot, we have the sensitivity *Sn* = 1. When $N_{-}^{+}=N^{+}$ meaning that all the hotspots were incorrectly predicted to be the coldspots, we have the sensitivity *Sn* = 0. Likewise, when $N_{+}^{-}=0$ meaning none of the coldspots was incorrectly predicted to be the hotspot, we have the specificity *Sp* = 1; whereas $N_{+}^{-}=N^{-}$ meaning all the coldspots were incorrectly predicted as the hotspots, we have the specificity *Sp* = 0. When $N_{-}^{+}=N_{+}^{-}=0$ meaning that none of hotspots in the positive dataset and none of the coldspots in the negative dataset was incorrectly predicted, we have the overall accuracy *Acc* = 1 and *MCC* = −1; when $N_{-}^{+}=N^{+}$ and $N_{+}^{-}=N^{-}$ meaning that all the hotspots in the positive dataset and all the coldspots in the negative dataset were incorrectly predicted, we have the overall accuracy *Acc* = 1 and *MCC* = −1; whereas when $N_{-}^{+}=N^{+}/2$ and $N_{+}^{-}=N^{-}/2$ we have *Acc* = 0.5 and *MCC* = 0 meaning no better than random guess. As we can see from the above discussion based on Equation (16), the meanings of sensitivity, specificity, overall accuracy, and Mathew’s correlation coefficient have become much more intuitive and easier-to-understand.

It should be pointed out that the metrics as given in Equation (15) and Equation (16) are valid only for the single-label systems as in the current case. For the multi-label systems in which emergence has become increasingly frequent in cell’s molecular systems [112–118] and biomedical systems [43,119], a completely different set of metrics as defined in [109] is needed.

### 2.5. Evaluate the Anticipated Success Rates by Jackknife Tests

The following three cross-validation methods are often used in statistical prediction to evaluate the anticipated accuracy of a predictor: independent dataset test, subsampling (*K*-fold cross-validation) test, and jackknife test [120]. However, as elucidated by a review article [83], among the three methods, the jackknife test is deemed the least arbitrary and most objective as it can always yield a unique outcome for a given benchmark dataset, and hence has been increasingly used and widely recognized by investigators to examine the accuracy of various predictor [48,60,63,65,69,76,121,122]. Accordingly, in this study we also used the results obtained by jackknife tests to optimizing the uncertain parameters and to compare with the other predictors in this area.

## 3. Experimental Section

The results obtained with iRSpot-TNCPseAAC on the benchmark dataset S of Supplementary Information S1 by the jackknife test are given in Table 4, where for facilitating comparison the corresponding results by the iRSpot-PseDNC [25] on the same benchmark dataset are also given.

As we can clearly see from the table, the iRSpot-TNCPseAAC predictor is superior to iRSpot-PseDNC [25] in three of the four metrics as defined by Equation (16); *i.e.*, it can yield higher accuracy *Acc*, higher Mathew’s correlation coefficient MCC, and higher sensitivity *Sn*. Therefore, it is anticipated that the new predictor will become a useful tool for identifying the recombination spots in DNA, or at the very least become a complementary tool to iRSpot-PseDNC, the best existing prediction method in this area.

## 4. Conclusions

The above fact has also proved that it is indeed a feasible and promising approach to extend the concept of pseudo amino acid composition [44,45,123] developed in computational proteomics to the area of computational genomics. As shown by Equation (13) and the related equations in defining its 64 + *λ* components, each of the DNA samples investigated in this study was formulated by a combination of its trinucleotide composition (TNC) with the pseudo amino acid components (PseAAC) that were derived from the protein translated from the DNA sample according to its genetic codes. The former can better incorporate its local or short-rage sequence order information in comparison with the dinucleotide composition (DNC) used in iRSpot-PseDNC [25]; while the latter can incorporate its global or long-range sequence order effects in a more natural or logical manner. Accordingly, it is anticipated that the idea or approach by extending the Chou’s pseudo amino acid composition [44,45,123] for protein sequences to the pseudo oligonucleotide composition for DNA or RNA sequences may also be used to deal with many other genome analysis problems.

## 5. Web Server and User Guide

To enhance the value of its practical applications, a web-server for the iRSpot-TNCPseAAC predictor was established. Moreover, for the convenience of the vast majority of experimental scientists, here a step-to-step guide is provided for how to use the web server to get the desired results without the need to follow the mathematic equations that were presented just for the integrity in developing the predictor.

**Figure 3.**
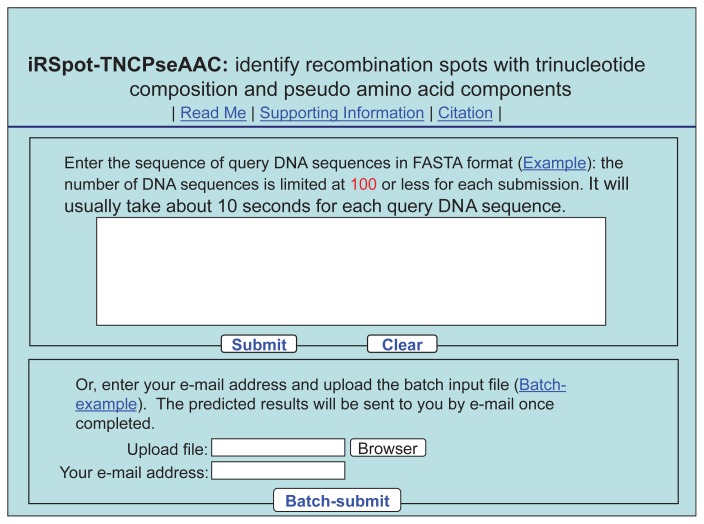
A semi-screenshot for the top page of the web-server iRSpot-TNCPseAAC at http://www.jci-bioinfo.cn/iRSpot-TNCPseAAC.

**Step 1.** Open the web server at http://www.jci-bioinfo.cn/iRSpot-TNCPseAAC and you will see the top page of the predictor on your computer screen, as shown in Figure 3. Click on the Read Me button to see a brief introduction about the **iRSpot-TNCPseAAC** predictor and the caveat when using it.

**Step 2.** Either type or copy/paste the query DNA sequences into the input box at the center of Figure 3. The input sequence should be in the FASTA format. For the examples of sequences in FASTA format, click the Example button right above the input box.

**Step 3.** Click on the Submit button to see the predicted result. For example, if you use the three query DNA sequences in the Example window as the input, after clicking the Submit button, you will see the following message shown on the screen of your computer: the outcome for the 1st query sample is “**recombination hotspot**”; the outcome for the 2nd query sample is “**recombination coldspot**”. All these results are fully consistent with the experimental observations as summarized in the Supplementary Information S1. However, no result was given for the 3rd query sample as it contains some invalid characters as warned in the output screen. It takes about a few seconds for the above computation before the predicted result appears on your computer screen; the more number of query sequences and longer of each sequence, the more time it is usually needed.

**Step 4.** As shown on the lower panel of Figure 3, you may also choose the batch prediction by entering your e-mail address and your desired batch input file (in FASTA format) via the “**Browse**” button. To see the sample of batch input file, click on the button Batch-example. After clicking the button Batch-submit, you will see “Your batch job is under computation; once the results are available, you will be notified by e-mail.”

**Step 5.** Click the Supporting Information button to download the benchmark dataset used to train and test the **iRSpot-TNCPseAAC** predictor.

**Step 6.** Click the Citation button to find the relevant papers that document the detailed development and algorithm of **iRSpot-TNCPseAAC**.

## Supplementary Information

Supplementary Information S1 The benchmark dataset *S* consists of a positive dataset *S*^+^ and a negative dataset *S*^−^. The positive dataset contains 490 recombination hot spots, while the negative dataset contains 591 recombination cold spots.

## Figures and Tables

**Table 1.** The conversion code of the 64 trinucleotides in DNA to the 20 amino acids in protein.

Trinucleotide	Amino acid
AAA	Lys (K)
AAC	Asn (N)
AAG	Lys (K)
AAT	Asn (N)

ACA	Thr (T)
ACC	
ACG	
ACT	

AGA	Arg (R)
AGC	Ser (S)
AGG	Arg (R)
AGT	Ser (S)

ATA	Ile (I)
ATC	

ATG	Met (M)
ATT	Ile (I)
CAA	Gln (Q)
CAC	His (H)
CAG	Gln (Q)
CAT	His (H)

CCA	Pro (P)
CCC	
CCG	
CCT	

CGA	Arg (R)
CGC	
CGG	
CGT	

CTA	Leu (L)
CTC	
CTG	
CTT	

GAA	Glu (E)
GAC	Asp (D)
GAG	Glu (E)
GAT	Asp (D)

GCA	Ala (A)
GCC	
GCG	
GCT	

GGA	Gly (G)
GGC	
GGG	
GGT	

GTA	Val (V)
GTC	
GTG	
GTT	

TAA	Stop!
TAC	Tyr (Y)
TAG	Stop!
TAT	Tyr (Y)

TCA	Ser (S)
TCC	
TCG	
TCT	

TGA	Stop!
TGC	Cys (C)
TGG	Trp (W)
TGT	Cys (C)
TTA	Leu (L)
TTC	Phe (F)
TTG	Leu (L)
TTT	Phe (F)

**Table 2.** List of the original values of the six physical-chemical properties for each of the 20 native amino acids.

Amino acid	Hydro-phobicity a $H_{1}^{0}$	Hydro-philicity b $H_{2}^{0}$	Side-chain mass c $H_{3}^{0}$	pK1 d $H_{4}^{0}$	pK2 e $H_{5}^{0}$	PI f $H_{6}^{0}$
A	0.62	−0.5	15	2.35	9.87	6.11
C	0.29	−1.00	47	1.71	10.78	5.02
D	−0.90	3.00	59	1.88	9.60	2.98
E	−0.74	3.00	73	2.19	9.67	3.08
F	1.19	−2.50	91	2.58	9.24	5.91
G	0.48	0.00	1	2.34	9.60	6.06
H	−0.40	−0.50	82	1.78	8.97	7.64
I	1.38	−1.80	57	2.32	9.76	6.04
K	−1.50	3.00	73	2.20	8.90	9.47
L	1.06	−1.80	57	2.36	9.60	6.04
M	0.64	−1.30	75	2.28	9.21	5.74
N	−0.78	0.20	58	2.18	9.09	10.76
P	0.12	0.00	42	1.99	10.60	6.30
Q	−0.85	0.20	72	2.17	9.13	5.65
R	−2.53	3.00	101	2.18	9.09	10.76
S	−0.18	0.30	31	2.21	9.15	5.68
T	−0.05	−0.40	45	2.15	9.12	5.60
V	1.08	−1.50	43	2.29	9.74	6.02
W	0.81	−3.40	130	2.38	9.39	5.88
Y	0.26	−2.30	107	2.20	9.11	5.63

a Taken from [98];

b Taken from [99];

c Taken from any biochemistry text book;

d Taken from [100] for C*^α^*-COOH;

e Taken from [100] for NH_3_;

f Taken from [101].

**Table 3.** The corresponding values obtained by the standard conversion of Equation 12 on the original values in Table 2.

**Amino acid**	*H*_1_	*H*_2_	*H*_3_	*H*_4_	*H*_5_	*H*_6_
A	0.62	−0.15	−1.55	0.78	0.77	−0.10
C	0.29	−0.41	−0.52	−2.27	2.57	−0.64
D	−0.90	1.67	−0.13	−1.46	0.24	−1.65
E	−0.74	1.67	0.33	0.01	0.37	−1.61
F	1.19	−1.19	0.91	1.87	−0.48	−0.20
G	0.48	0.11	−2.00	0.73	0.24	−0.13
H	−0.40	−0.15	0.62	−1.94	−1.01	0.65
I	1.38	−0.82	−0.19	0.63	0.55	−0.14
K	−1.50	1.67	0.33	0.06	−1.15	1.56
L	1.06	−0.82	−0.19	0.82	0.24	−0.14
M	0.64	−0.56	0.39	0.44	−0.54	−0.29
N	−0.78	0.22	−0.16	−0.03	−0.77	2.20
P	0.12	0.11	−0.68	−0.94	2.21	−0.01
Q	−0.85	0.22	0.29	−0.08	−0.69	−0.33
R	−2.53	1.67	1.23	−0.03	−0.77	2.20
S	−0.18	0.27	−1.03	0.11	−0.65	−0.32
T	−0.05	−0.10	−0.58	−0.18	−0.71	−0.36
V	1.08	−0.67	−0.65	0.49	0.51	−0.15
W	0.81	−1.65	2.17	0.92	−0.18	−0.22
Y	0.26	−1.08	1.43	0.06	−0.73	−0.34

**Table 4.** A comparison of iRSpot-TNCPseAAC with the best existing method.

Predictor	Test method	*Sn* (%)	*Sp* (%)	*Acc* (%)	MCC
iRSpot-PseDNC a	Jackknife	73.06	89.49	82.04	0.638
iRSpot-KNCPseAAC b	Jackknife	87.14	79.59	83.72	0.671

a From [25];

b This paper with *λ* = 5, *w* = 1.1, *C* = 32 and *γ* = 0.5 for the LIBSVM operation engine [107,108].
