# Supplementary material for: iRSpot-TNCPseAAC: Identify Recombination Spots with Trinucleotide Composition and Pseudo Amino Acid Components
Source: Int J Mol Sci. 2014 Jan 24;15(2):1746–66. doi: 10.3390/ijms15021746 (PMC3958819; doi:10.3390/ijms15021746)
Supplement: Supplementary Information S1 — The benchmark dataset S consists of a positive dataset S+ and a negative dataset S−. The positive dataset contains 490 recombination hot spots, while the negative dataset contains 591 recombination cold spots. [file ijms-15-01746-s001.pdf]

Supplementary Information

Supplementary Information S1. The benchmark dataset S consists of a positive dataset S^+ and a negative dataset S^-. The positive dataset contains 490 recombination hotspots, while the negative dataset contains 591 recombination coldspots.

(1) S^+: 490 recombination hot spots

>YYDL087C 1.67 Hot
ATGTCAACTATGTCAACGCCTGCCGCAGAACAACGCAAACTCGTCGAACAGCTGATGGGCAGGGACTTCAGTTTC
CGCCACAACAGGTATTCGCATCAAAAAAGAGACCTCGGACTACACGATCCCAAGATCTGCAAGTCATACCTTGTT
GGCGAGTGCCCCTACGACCTGTTTCAGGGCACCAAGCAGAGCCTGGGAAAATGCCCGCAGATGCATCTTACCAAG
CATAAAATTCAGTACGAGAGAGAGGTCAAGCAGGGCAAAACGTTTCCCGAATTCGAAAGAGAATATCTGGCCATT
CTATCTCGGTTTGTTAATGAGTGTAATGGCCAGATATCCGTAGCACTACAAAATCTAAAACACACCGCTGAGGAA
CGAATGAAGATTCAGCAGGTTACCGAAGAACTAGATGTCTTGGACGTGCGGATAGGCCTAATGGGACAAGAGATT
GATTCTTTAATTCGTGCAGATGAAGTCAGTATGGGTATGCTACAGTCAGTCAAATTACAAGAACTGATTAGTAAA
AGGAAAGAAGTTGCAAAACGTGTACGAAACATTACAGAAAACGTGGGCCAGAGCGCCCAGCAAAAGTTACAGGTT
TGCGAGGTGTGTGGGGCATACCTATCGCGTTTAGATACAGACAGAAGGCTTGCTGACCACTTCTTGGGGAAGATT
CATCTGGGATATGTCAAGATGAGAGAGGATTATGATCGGCTAATGAAGAATAACCGGACAACTAACGCCAGCAAG
ACAGCTACTACACTACCCGGAAGACGCTTTGTGTAG

>YBR268W 1.71 Hot
ATGTTGGCGCGCAGTTTGGGTTACAGGTTGATATCGACCAGTCGTATCTTATATAATAAACCCACGGTTAAGTCA
GTGGTGTCGTCGTGTCCTGCGGGAACATCGCTTAACTTAAACATATGGAAGAGCGGTAAAGACGCAGTGGCACTC
GAGGACAAGGAGTATCCAAATTGGTTATGGAGCGTATTGGATAGTGATCACGTTGTCGAACATGCGGCTGAGGAC
CCAGAGGGACAGGCTCTCTTAAAGAGAAGAAAGAACATAAGGAAGGCGAACAGGCAGCGAATCAAGCAAAACAAT
TTCTTGAGCCAACTTTAA

>YAL046C 1.56 Hot
ATGAAGCTCCCACAGACCATGCTACGTTCTATATCTGTGAAGCATGTCCGGTGGCCAAGGATTCTGACGGGCTCA
AAGCTTTGGTACTCAACGCAGATGGCAATGACTCCGGAGGAGAAGATGATCACCGATAAACTACAACAGGAACTG
GAACCTGAAGTGTGTAAAGTGCAAGACGTTTCCGGTGGCTGCGGATCCATGTTTGCTATCAACATAACAAGCAAG
AAGTTCAACGGACTGAGTCTCATCAAGCAGCACCAGCTGGTGAACAGAATTTTGAGGGACGATATTTCCAGATGG
CATGGCCTACAATTGACCACTAAGAAGTCAACTGGGAAGGGTCCGGCATCATCATGA

>YLR232W 2.04 Hot
ATGGGGGCTAGTAGTGGAAGAATAGGTAAGTCAATGCTGACCCAAGGCACCTTTCCTCTGGCTTCTTCAGGATGT
TTGAAATGCGATTCCACAGCACAATCGCTCCACGCATCTAGCTCAGCATTAATTGAATTCCTAGTTGACTTCGGC
ATCAAACCTAAAGAATTCCCGCATAAGTACGTCACAGGCTTATCGTCGGACGATAGTCCCATGGATTTAAATGTA
GGGATGTTGAATTCATCCCTCAGAGATTCCGGATATTCTCCGTCTAATTCCAAAGCTTTCTCCATAATTACCAGT
TTGTTTCTTTTTTCCACAGAATATTTAGGCTTAATATGTACGTATTGA

>YOR173W 2.12 Hot
ATGGGTTCCCAAGATTTAGCTAGTTTAATTGGGAGGTTTAAATACGTCAGGGTGTTAGATTCGAACCCTCATACA
AAGGTAATCTCACTACTAGGCAGCATAGATGGGAAGGATGCCGTGTTAACTGCTGAAAAGACGCATTTCATATTT
GATGAAACGGTACGTAGACCCTCTCAAAGTGGCCGTAGCACTCCGATTTTCTTTCATCGTGAAATAGACGAGTAC
TCCTTTTTAAACGGTATTACTGATCTGAAGGAATTAACTTCGAATGACATCTACTACTGGGGGTTGTCTGTTTTG
AAGCAGCATATTTTGCATAACCCAACTGCGAAGGTTAACTTAATTTGGCCCGCCTCGCAGTTCCACATCAAGGGA
TACGACCAGCAGGATTTACATGTCGTCAGAGAAACTCCTGATATGTATAGAAACATAGTTGTGCCCTTTATCCAA
GAGATGTGTACCAGCGAAAGAATGAAATGGGTAAACAACATTCTTTACGAGGGCGCCGAGGATGATAGAGTTGTT
TATAAGGAATACAGTAGCAGGAATAAAGAGGATGGATTCGTCATCTTGCCCGATATGAAGTGGGACGGAATCAAC
ATTGATTCGTTATACCTGGTCGCGATCGTGTACAGGGACGACATCAAATCTTTGAGAGATTTGAACCCAAACCAT
AGAGATTGGTTAATTAGACTCAATAAGAAGATTAAGACCATCATCCCCCAGCACTATGACTATAATGTAAACCCG
GACGAGCTAAGAGTTTTCATTCATTATCAACCTTCATATTACCATTTTCATGTCCATATAGTGAATATAAGGCAT
CCAGGAGTAGGCGAGGAGAGAGGCTCCGGAATGACAATACTGTTGGAAGATGTTATAGAAGCGTTGGGTTTCTTA
GGTCCTGAAGGTTATATGAAGAAGACGCTTACTTATGTCATTGGTGAAAATCATGATTTATGGAAAAAGGGCTTT
AAGGAAGAAGTTGAAAAGCAACTGAAGCATGATGGTATCGCAACCAGTCCTGAAAAAGGCTCTGGGTTCAATACC
AATTTGGGTTGA

>YKL087C 1.72 Hot
ATGATGTCTTCAGACCAACAGGGGAAATGCCCCGTAGATGAGGAAACCAAAAAGTTATGGCTACGAGAACATGGC
AACGAAGCGCATCCTGGTGCTACTGCCCCAGGCAATCAACTAGAGTGCTCTGCAAACCCACAAGATAACGATAAA
ACGCCTGAATATCACACCACGGTGGATCTCTCTCAGTCCAGAGAGGTTTCCACCATACCAAGGACGAATTCTGAC
AGAAACTGGATATACCCGTCGGAGAAACAATTTTACGAGGCAATGATGAAAAAAAATTGGGATCCGAACTCAGAT
GACATGAAGGTGGTTGTGCCTTTACATAACTCCATCAATGAGCGGGTTTGGAACTACATTAAAAGCTGGGAAGAC

AAGCAGGGTGGTGAAGCGTGTGGCGGTATCAAATTAACAAATTTTAAAGGTGATTCCAAAAAACTGACACCAAGG
GCCTGGTTCAGGTCCCGTATCTTGCACCTGGCCAAACCTTTTGATAGACATGATTGGCAGATAGATAGATGCGGT
AAAACCGTCGACTACGTAATCGATTTCTATTCCACCGATCTGAACGATGCAAACTCGCAGCAACAACCACTTATT
TATCTCGATGTTAGACCAAAATTAAACAGTTTTGAGGGCTTTAGATTACGGTTCTGGAAATCTTTAGGCTTTTGA

>YGR086C 1.5 Hot
ATGCACAGAACTTACTCTTTAAGAAATTCCAGGGCACCTACCGCCTCTCAATTACAGAACCCACCGCCACCACCA
TCTACAACCAAAGGTAGATTCTTTGGGAAGGGTGGTCTAGCTTACAGCTTTAGGAGAAGTGCTGCTGGAGCTTTT
GGCCCAGAATTATCCAGAAAGTTGTCTCAATTGGTTAAGATTGAAAAGAATGTTTTGAGGTCCATGGAATTGACA
GCCAACGAAAGACGTGACGCTGCTAAGCAATTGTCTATTTGGGGGTTGGAAAACGATGACGATGTTTCCGACATC
ACTGATAAATTAGGTGTCTTGATCTATGAAGTTAGTGAATTAGACGACCAATTTATCGATCGTTATGACCAATAC
AGATTGACTCTAAAGTCCATCAGAGATATCGAAGGTTCTGTTCAACCATCTAGAGACCGTAAGGACAAGATCACC
GACAAAATCGCCTACTTGAAATACAAAGATCCTCAATCACCTAAGATTGAGGTCTTGGAACAAGAATTGGTGCGT
GCTGAGGCTGAATCTTTGGTCGCTGAAGCTCAATTATCTAATATCACAAGGTCAAAGTTGAGAGCTGCTTTCAAC
TACCAATTTGACTCCATCATCGAACATTCAGAGAAAATTGCTTTAATCGCTGGTTACGGTAAGGCTCTCTTGGAA
CTATTGGACGACTCTCCTGTCACTCCAGGTGAAACCAGGCCTGCTTACGATGGGTATGAAGCCTCTAAACAAATC
ATTATTGATGCTGAAAGCGCACTGAATGAATGGACACTAGACTCTGCCCAAGTCAAGCCTACTTTAAGTTTCAAG
CAGGATTACGAAGACTTCGAACCTGAAGAAGGCGAAGAAGAGGAAGAGGAAGACGGTCAAGGCAGGTGGTCCGAA
GACGAACAAGAAGATGGACAAATTGAAGAACCTGAACAAGAAGAAGAAGGTGCTGTTGAAGAACATGAACAAGTC
GGACACCAGCAAAGTGAGTCTCTTCCCCAACAAACAACAGCTTAA

>YIL155C 2.16 Hot
ATGTTTTCGGTAACGAGAAGAAGAGCTGCCGGTGCAGCTGCTGCCATGGCCACAGCCACGGGGACGCTGTACTGG
ATGACTAGCCAAGGTGATAGGCCGTTAGTGCACAATGACCCGAGCTACATGGTGCAATTCCCCACCGCCGCTCCA
CCGCAGGTCTCTAGACGAGACCTGCTGGACCGTCTGGCCAAGACGCATCAATTCGACGTGTTGATCATCGGTGGC
GGGGCCACGGGGACAGGATGTGCCCTAGATGCTGCGACCAGGGGACTCAATGTGGCCCTTGTTGAAAAGGGGGAT
TTTGCCTCGGGAACGTCGTCCAAATCTACCAAGATGATTCACGGTGGGGTGCGGTACTTAGAGAAGGCCTTCTGG
GAGTTCTCCAAGGCACAACTGGATCTGGTCATCGAGGCACTCAACGAGCGTAAACATCTTATCAACACTGCCCCT
CACCTGTGCACGGTGCTACCAATTCTGATCCCCATCTACAGCACCTGGCAGGTCCCGTACATCTATATGGGCTGT
AAATTCTACGATTTCTTTGCCGGTTCCCAAAACTTGAAAAAATCATACCTACTGTCCAAATCCGCCACCGTGGAG
AAGGCTCCCATGCTTACCACAGACAATTTAAAGGCCTCGCTTGTGTACCATGATGGGTCCTTTAACGACTCGCGT
TTGAACGCCACTTTAGCCATCACGGCTGTGGAGAACGGCGCTACCGTCTTGAACTATGTCGAGGTACAAAAATTG
ATCAAAGACCCAACTTCTGGTAAGGTTATCGGTGCCGAGGCCCGGGACGTTGAGACTAATGAGCTTGTCAGAATC
AACGCTAAATGTGTGGTCAATGCCACGGGCCCATACAGTGACGCCATTTTGCAAATGGACCGCAACCCATCCGGT
CTGCCGGACTCCCCGCTAAACGACAACTCCAAGATCAAGTCGACTTTCAATCAAATCGCCGTCATGGACCCGAAA
ATGGTCATCCCATCTATTGGCGTTCACATCGTATTGCCCTCTTTTTACTGCCCGAAGGATATGGGTTTGTTGGAC
GTCAGAACCTCTGATGGCAGAGTGATGTTCTTTTTACCTTGGCAGGGCAAAGTCCTTGCCGGCACCACAGACATC
CCACTAAAGCAAGTCCCAGAAAACCCTATGCCTACAGAGGCTGATATTCAAGATATCTTGAAAGAACTACAGCAC
TATATCGAATTCCCCGTGAAAAGAGAAGACGTGCTAAGTGCATGGGCTGGTGTCAGACCTTTGGTCAGAGATCCA
CGTACAATCCCCGCAGACGGGAAGAAGGGCTCTGCCACTCAGGGCGTGGTAAGATCCCACTTCTTGTTCACTTCG
GATAATGGCCTAATTACTATTGCAGGTGGTAAATGGACTACTTACAGACAAATGGCTGAGGAAACAGTCGACAAA
GTTGTCGAAGTTGGCGGATTCCACAACCTGAAACCTTGTCACACAAGAGATATTAAGCTTGCTGGTGCAGAAGAA
TGGACGCAAAACTATGTGGCTTTATTGGCTCAAAACTACCATTTATCATCAAAAATGTCCAACTACTTGGTTCAA
AACTACGGAACCCGTTCCTCTATCATTTGCGAATTTTTCAAAGAATCCATGGAAAATAAACTGCCTTTGTCCTTA
GCCGACAAGGAAAATAACGTAATCTACTCTAGCGAGGAGAACAACTTGGTCAATTTTGATACTTTCAGATATCCA
TTCACAATCGGTGAGTTAAAGTATTCCATGCAGTACGAATATTGTAGAACTCCCTTGGACTTCCTTTTAAGAAGA
ACAAGATTCGCCTTCTTGGACGCCAAGGAAGCTTTGAATGCCGTGCATGCCACCGTCAAAGTTATGGGTGATGAG
TTCAATTGGTCGGAGAAAAAGAGGCAGTGGGAACTTGAAAAAACTGTGAACTTCATCAAGACGTTTGGTGTCTAA

>YDR230W 1.92 Hot
ATGTTGCATATATACATAGGAAAACGGTTAAAAGGCCCTGCTTCTACCTTCTGTTTCCCCCTCGTTCTTTTCACC
AGAACTTGTACCATTTCTTCTCAGAGGAGGCCTGAGATAAATTCTGGTTACTTTGTTCAAACTGACTCTGCTTTT
CATTTTTGGCGGCCTCCCATTGCCCCGGCAGAGATGGGTCATGAGGGACATTATGGAGCATTGGTTTTTCCTTTT
TCGCCACGGTTTCCTTTGCCAATTGTGCGATCTGGAAACTTTTTTGTCTCTTTAGCCTACATTGCTCCCATCCGA
CAATCGAGCCCAGTATCAGAGAGCTCATCGCCCAATTCGTGGCCTTAG

>YJR071W 1.54 Hot
ATGTATTCGATGGCCTTTTTGGCTTCCTCTGGCTTAGTGGCAAATTCTTCAGCAACAGTCTTTAAGTTGAACAGA
GCTCTAAATCTGTTGGCCAAAACTGTTTTGCCGGACTTGTTGACTAAGATGTCCCTTAGTTGCTCTAGAGTGCAT
TCATCGACGTTTTCTTGGAAATGTTTTTCAAAGTTAGTAGACATGTTTTTTTTTTTCTTTCTTGCGTGGTATGTT
AGAAGGACTACAGTTTATCCTAACCTAAACCTAGAGCTACCGTCAAATATACACGTGTATTCACTGGATTTGCCT
TATGTAATTTATATGATAAAAACTTTTCAGCTCATCGAGAAAAATTTTCTTTCCTCCCACGCAGGATAG

>YMR244W 1.56 Hot
ATGGTACTTTGCAAATTACTGACACCATATTTCTTACTGTCAATTTTGAGTGTCGGCGTGTTCACGGCGACCGCC
GCGCCATCGCCCAGTATTCAAATGACGGAAAATACAAATCAAGATCATCATGAGCATGCCAAGCGTGGAGGAACG
TGTGCGTTCCCTAACTACGATGGGATGGTCGCAGTACAAAAAGGTGGATCTAATGGAGGATGGGCTATGAGCCCT

```
GACCAAGAATGTTCCTACGGTTCATGGTGCCCTTACGCTTGCAAACCAGGTCAACTAATGGGGCAATGGGACCCT
TCGGCTACCACATACTCTTATCCTAAATGTCAAAATGGAGGTTTGTACTGTGATTCTAACGGTAACTTGCAAAAG
CCAAACAGTGATAAAGACTATTGTTATGATGGGAAGGGAACCGTAATAGCGAAAAACAACGCTAACAGCGGTGAC
GTTGCATTTTGCCAGACCGTGCTTCCGGGCAACGAAGCTATGCTGATCCCAACCTTAGTCGGCTCTGGGTCAAAG
CAAACGCTGGCTGTGCCTGGTACAGACTACTGGGCCTCCAGCGCGTCGCATTACTACGTAAATGCTCCCGGTGTA
AGCGTAGAGGATGCATGCCAGTGGGGTAGTAGTGCAAATCCACAGGGGAACTGGGCCCCATTTGTAGCTGGTTCC
AACATGGACGACAACCAGAACACTTTTGTAAAGATTGGATGGAACCCCGTCTACCTGGAATCGTCATGTCCGTTC
AAGAACGTTAAGCCTTCATTCGGTATTAGAATTACTTGTGATGACGAATCACAATGTGAAGGCCTACCATGCTCC
ATTGACCCAAGTTCTAATGGAGTCAACGAAGTGACAAGTTCTGGCGGTGGTTCTTCCGGGGCTGGTGGTGGAAAC
TTTTGTGTTGTCACCGCCAGAAACGGCGCCAAGGCCAACATCGAAGTTTTTGATGTTGGTAGCGGCTCATCTTCT
AAAGGCAAGAGAGAACTGAATCCGCTAGACGTTATTACCACAACGGTCACCGAGACCAAGTACAAGACAGTCACC
GTCACTGCCAAAACTTAG

>YDR517W  1.64   Hot
ATGTTTAGAATAGCTAAAAACCTCGTACGGACTTTTGAGCAGAGCGTGCAAGATACCCTGGCACTCTCGCAGGAC
TCGAGCAATCTGGACGCTTTCTTTCAGTCTATACCACCAAATTTGTTGTCTGCTCAGTTGGAATCACCCGTAGAT
GCGGTTTCTGAAGGTGTTAAGCACACCAACGTCAATGAAACGCTTTCCGGTCTCCGCATTGTTTGGGTGGACGAG
ATGCAGTTCCAACTGCAGTCCTTTTTCGACTATATAGTAGGTTTCAATGATGATCCGGTGCCGGTGGTCAGTAAT
CAACACGGATTTTCGTACCCAGATTACCGTCGTATCACATCCATCTTCAATGAACACTGTGGCCGAACTTTGAAG
GTCAACATATGGTCAGCTAAAGGAGGCACGTTCAGAGACGAGTATATCAGCATTATATCGAAGGAGAGCGACGAT
TTGGACGATGTTTCTCTAAACCATGACGAGAGGAGGCCATCTAGTGGGGAAGCGCACCAGTTCCAAGCACTCGGG
TTCAAAGTCCAATGGACGCCTTTGATAGCTTCCACTTTCACATACCATATCCTGAACGTGAACATCCCAGATGGA
CCCGCCCAATCGGCAGGGCTCATCCCAGATGAAGACTACATTATCGGCTGCCAAGATGGCCTGTTGGCCACGGGA
GGGGAGACGCTTTTACAAGATATTGTTAGATCAAGAGCAAACTACGATCTGGTCCTTTATGTCTACAACAAAGTT
TCTGACTGTGTGAGACCAATCACAGTTCATATAGGTCCCGACGGCAGGTTGGGTTGCAACGTTGGATACGGGTTT
CTTCACAGGATTCCAACAGTAAAGCACTGTCCACAACAAGCACAGCAGCAGGGGCAAGATGACAACCCTGTTCCT
GTTCCTGTTCCAGTAGAATCTGAGACGGCTTTTGTGCCATCAGCATTCACAGCACCACCGGTGCCAACAAAGAAG
AAATCAAAAAATAAAAAGGGTACCCAGCCATTGGCAATGGATGACTATTTTAATGAAGGCAGAGATAAGTCATCT
ACCGCTGCGAAGTCAGCAGAATCTGACATCCTCGCCCCACCACCACAAAAACAGTCATCCTCTGATTAA

>YLR379W  2.22   Hot
ATGATGGGCGAAACACCCAATTCCAGTAAAGTACCACGGTTGGAGGCCAGCATGGCACGTAGCCAGTACAGAGGG
TCGGAAGTCTCACTGGACACGATCCCGTACAGCGGAATCTGGCCCAGAATCAAAAAGATCAGTAGAGAAACGCCT
GTCCAGATAAGTTTCTGGTTGTATGGAACCTTCCTTTCTGGAGCAATCACTTCCGGCAGGAAAGATTCAAATGGC
TTAAACAAGTCTAGAACACGGTTGGAGGACATTTTCAAAGTAAAGCGTGAACCAGTGTCAGTTTTTCTACTTGCT
ACTATATTTAACTATGTTTTTTTCTTTTGCTTTCACCAACTGAACCTTGTAGTAAATGGAGTGAGGTTGAATTGA

>YOL085C  1.75   Hot
ATGAAGGCAAAAGACAAATATAAGGGTCGAACGAAAAATAAAGTGAAAAGTGTTGATATGATGTATTTGGCTTTG
CGGCGCCGAAAAAACGAGTTTACGCAATTGCACAATCATGCTGACTCTGTGGCGGACCCGCGCTCTTGCCGGCCC
GGCGATAACGCTGGGCGTGAGGCTGTGCCCGGCGGAGTTTTTTGCGCCTGCATTTTCCAAGGTTTACCCTGCGCT
AAGGGGCGAGATTGGAGAAGCAATAAGAATGCCGGTTGGGGTTGCGATGATGACGACCACGACAACTGGTGTCAT
TATTTAAGTTGCCGAAAGAACCTGAGTGCATTTGCAACATGA

>YDR491C  1.59   Hot
ATGGAGGCTCTCGTTGCCTTGCAGCAGACACTATCTTGGTTTCTTCTTCTAGTAGTGGTGATACTCATTTTTTTT
CTTCTTTTATCTTGCTTGACTCAATTAAGGCGACTAATAACAAAACGAATGACGAAAATGGAAAACAATAGAAAA
GTTAGGAACCAGAATCACTTTTGTAAAGTTAATATTGCGCTTGCGGAGCTCCTTACTGTTTCAACTGATGATACT
GATATGCCTTTAGAAAAGAAATCAAATTCTATTAACAGAAGGGTGCCAACATGTACGTATATATATATGTGTGCG
TGTGTATATATGTCTGACATATACATGAGTAACCGGCTAAGTAAGGTTTTTACGTTGATTGCTGCATCCATCGGT
CGTGAGTATTGTCAAATTGCCGATGCCCTGCAGGCAAAAAAACCGTTGAAAAATCGTTGGGAAAATGGGCTTGCC
CGACTGTCGGATTTAAGGGCTTGGACCCTAAAGGTTGCATGA

>YGR209C  1.85   Hot
ATGGTCACTCAATTAAAATCCGCTTCTGAATACGACAGTGCTTTAGCATCTGGCGACAAGTTAGTCGTTGTTGAC
TTTTTTGCCACATGGTGTGGGCCATGTAAAATGATTGCACCAATGATTGAAAAGTTTGCAGAACAATATTCTGAC
GCTGCTTTTTACAAGTTGGATGTTGATGAAGTCTCAGATGTTGCTCAAAAAGCTGAAGTTTCTTCCATGCCTACC
CTAATCTTCTACAAGGGCGGTAAGGAGGTTACCAGAGTCGTCGGTGCCAACCCAGCTGCTATCAAGCAAGCTATT
GCTTCCAACGTATAG

>YJR017C  1.61   Hot
ATGCCATCTGACGTAGCATCGAGGACCGGGCTGCCAACCCCGTGGACCGTCAGGTATAGTAAGTCCAAGAAAAGA
GAGTATTTTTTCAATCCAGAGACGAAGCACTCGCAATGGGAGGAGCCTGAGGGCACCAACAAGGACCAGCTACAC
AAGCACTTAAGAGACCATCCAGTGCGTGTAAGATGCCTGCACATTCTCATCAAGCACAAGGATTCAAGAAGACCC
GCATCGCATAGATCCGAGAACATTACGATATCCAAGCAAGACGCTACGGACGAACTGAAAACCTTAATCACGAGG
TTGGATGACGACTCTAAGACGAACTCCTTCGAGGCCCTGGCTAAAGAGAGATCAGATTGCTCTTCATACAAGCGA
```

GGCGGCGACCTCGGCTGGTTCGGGAGAGGCGAGATGCAGCCTAGCTTTGAAGACGCTGCCTTCCAGCTCAAGGTC
GGCGAGGTAAGCGATATCGTTGAATCAGGAAGCGGTGTTCATGTGATCAAGCGGGTAGGTTAG

>YJL077C 1.82   Hot
ATGCACATATCGCTCCCTACGAGAAACAAATCTTATTTTCGAATACGTACACGTACATATCAAATAGGCTTATAT
CATTCTGACTCTTCACCAATCAGGGACATATCAGTACTACATCTGTTAATAGCAACGTTATGCACTATTTTTTTT
CCAATTTTTTTTTCGTTGAGTAAAGTTCAGGTCGTACAGTGGCAAGGAACGACTATTTCTAAAAACTGTATTGCC
TTAACCATGTCTTTCCCCTTGAACGCTATCCCTGGCATGTATCTGATAATTGCTTTCCCTCGTCTACAAACAGTA
ATTCCCCTGCAGCGAAATACTCCTGTTCGAATTACGAAATCGGTGATAGTGAAAGGCGCAGTGAGCGTGCCACGG
ATCTCCTCTCCGATGCACTAG

>YGR239C 1.52   Hot
ATGCCCAGTGTCTGCCATACAAGCCCGATAGAAAAGATTATCCAACAAGGGCATCGCATACAGAATGATTCCTTA
ATACCGTCAAAACGCACCAAGCTGGCCCATACGGAATTAACAGCACACTATGCCACTGAAGACTCGCATGTGGAA
AAACACTTTTTGCATAATGGTAGTAATTTTGACGGCATTGACAATGTAAGGTATCAAAATCAACCCTCTCCATTA
ACCTTCATTACTCCAAACAACACTGTAGATTCCAGTGATTGGGTTCCGCAGTTCTCGTCTATGAAGATTGACGAT
TCACTAGAGTTTAGCTCAGAATATAAAAGGTTGTATTCCAATTACGAGTCACAGCAGCGCCTGAATTCAAGCAGA
CAACACTTACCTTTTAAGAATTGTATGATAAGGAAGACAAGTTGCACTTACCCACCTCAAAAGACGCTTCGACAG
CAACGCCAAGGAAACCGGGACAACCCAACGGATGCGTTTCAATTCGACGCGGAGTTCCAAGTTTTGGAGAGAGAA
ATTCAAAAAGAGCGGTACGAGCCCATAACACGTCGGGACGAGAAATGGTTCGACCAAGATCAGTCGGAACTGCAA
AGGATTGCCACAGATATAGTAAAATGCTGTACTCCACCGCCTTCGTCTGCATCCTCCTCTTCTACTCTTTCCTCC
TCTGTCGAATCAAAACTATCTGAGTCGAAGTTTATTCAGTTAATGAGAAACATTAGTAGCGGTGACGTGACTTTA
AAGAAAAATGCAGATGGAAATTCCGCAAGCGAGTTATTCTCATCCAACAACGGGGAACTGGTCGGAAACAGGCAC
ATTTTCGTCAAGGACGAAATTCACAAAGACATACTTGATTGA

>YAL054C 1.53   Hot
ATGTCGCCCTCTGCCGTACAATCATCAAAACTAGAAGAACAGTCAAGTGAAATTGACAAGTTGAAAGCAAAAATG
TCCCAGTCTGCCGCCACTGCGCAGCAGAAGAAGGAACATGAGTATGAACATTTGACTTCGGTCAAGATCGTGCCA
CAACGGCCCATCTCAGATAGACTGCAGCCCGCAATTGCTACCCACTATTCTCCACACTTGGACGGGTTGCAGGAC
TATCAGCGCTTGCACAAGGAGTCTATTGAAGACCCTGCTAAGTTCTTCGGTTCTAAAGCTACCCAATTTTTAAAC
TGGTCTAAGCCATTCGATAAGGTGTTCATCCCAGACCCTAAAACGGGCAGGCCCTCCTTCCAGAACAATGCATGG
TTCCTCAACGGCCAATTAAACGCCTGTTACAACTGTGTTGACAGACATGCCTTGAAGACTCCTAACAAGAAAGCC
ATTATTTTCGAAGGTGACGAGCCTGGCCAAGGCTATTCCATTACCTACAAGGAACTACTTGAAGAAGTTTGTCAA
GTGGCACAAGTGCTGACTTACTCTATGGGCGTTCGCAAGGGCGATACTGTTGCCGTGTACATGCCTATGGTCCCA
GAAGCAATCATAACCTTGTTGGCCATTTCCCGTATCGGTGCCATTCACTCCGTAGTCTTTGCCGGGTTTTCTTCC
AACTCCTTGAGAGATCGTATCAACGATGGGGACTCTAAAGTTGTCATCACTACAGATGAATCCAACAGAGGTGGT
AAAGTCATTGAGACTAAAAGAATTGTTGATGACGCGCTAAGAGAGACCCCAGGCGTGAGACACGTCTTGGTTTAT
AGAAAGACCAACAATCCATCTGTTGCTTTCCATGCCCCCAGAGATTTGGATTGGGCAACAGAAAAGAAGAAATAC
AAGACCTACTATCCATGCACACCCGTTGATTCTGAGGATCCATTATTCTTGTTGTATACGTCTGGTTCTACTGGT
GCCCCCAAGGGTGTTCAACATTCTACCGCAGGTTACTTGCTGGGAGCTTTGTTGACCATGCGCTACACTTTTGAC
ACTCACCAAGAAGACGTTTTCTTCACAGCTGGAGACATTGGCTGGATTACAGGCCACACTTATGTGGTTTATGGT
CCCTTACTATATGGTTGTGCCACTTTGGTCTTTGAAGGGACTCCTGCGTACCCAAATTACTCCCGTTATTGGGAT
ATTATTGATGAACACAAAGTCACCCAATTTTATGTTGCGCCAACTGCTTTGCGTTTGTTGAAAAGAGCTGGTGAT
TCCTACATCGAAAATCATTCCTTAAAATCTTTGCGTTGCTTGGGTTCGGTCGGTGAGCCAATTGCTGCTGAAGTT
TGGGAGTGGTACTCTGAAAAAATAGGTAAAAATGAAATCCCCATTGTAGACACCTACTGGCAAACAGAATCTGGT
TCGCATCTGGTCACCCCGCTGGCTGGTGGTGTTACACCAATGAAACCGGGTTCTGCCTCATTCCCCTTCTTCGGT
ATTGATGCAGTTGTTCTTGACCCTAACACTGGTGAAGAACTTAACACCAGCCACGCAGAGGGTGTCCTTGCCGTC
AAAGCTGCATGGCCATCATTTGCAAGAACTATTTGGAAAAATCATGATAGGTATCTAGACACTTATTTGAACCCT
TACCCTGGCTACTATTTCACTGGTGATGGTGCTGCAAAGGATAAGGATGGTTATATCTGGATTTTGGGTCGTGTA
GACGATGTGGTGAACGTCTCTGGTCACCGTCTGTCTACCGCTGAAATTGAGGCTGCTATTATCGAAGATCCAATT
GTGGCCGAGTGTGCTGTTGTCGGATTCAACGATGACTTGACTGGTCAAGCAGTTGCTGCATTTGTGGTGTTGAAA
AACAAATCTAGTTGGTCCACCGCAACAGATGATGAATTACAAGATATCAAGAAGCATTTGGTCTTTACTGTTAGA
AAAGACATCGGGCCATTTGCCGCACCAAAATTGATCATTTTAGTGGATGACTTGCCCAAGACAAGATCCGGCAAA
ATTATGAGACGTATTTTAAGAAAAATCCTAGCAGGAGAAAGTGACCAACTAGGCGACGTTTCTACATTGTCAAAC
CCTGGCATTGTTAGACATCTAATTGATTCGGTCAAGTTGTAA

>YJR078W 1.52   Hot
ATGAACAACACTTCCATAACCGGACCACAAGTACTACATAGAACAAAAATGAGACCACTACCAGTGCTAGAAAAA
TACTGTATCTCGCCACATCATGGGTTTTTGGACGACCGGTTACCCTTGACCAGACTGAGCAGCAAGAAATATATG
AAATGGGAAGAAATCGTTGCTGACCTGCCCTCTCTTTTGCAAGAGGATAACAAGGTGCGGAGCGTCATCGATGGG
CTGGATGTCTTGGACTTGGATGAGACGATCTTGGGCGACGTCAGGGAGCTCAGAAGAGCATATTCCATTTTGGGG
TTTATGGCGCACGCTTATATTTGGGCCAGCGGAACTCCCCGGGATGTATTACCGGAGTGTATTGCAAGGCCACTG
TTGGAGACAGCACATATTTTGGGTGTGCCACCGTTAGCTACGTACTCCTCGTTGGTGTTATGGAACTTCAAAGTG
ACCGACGAGTGCAAGAAAACGGAAACCGGGTGTTTGGACTTGGAAAATATTACAACGATAAACACATTTACGGGA
ACCGTTGACGAAAGTTGGTTTTATCTGGTCAGCGTGCGGTTTGAAAAAATCGGCAGCGCTTGTTTAAACCATGGG
TTGCAGATATTGAGAGCCATTAGAAGCGGTGATAAAGGAGACGCTAACGTGATAGACGGGTTGGAGGGCTTGGCT
GCAACGATCGAAAGGTTATCGAAAGCCTTGATGGAAATGGAGCTTAAGTGTGAACCCAATGTATTTTATTTCAAG

ATAAGACCATTTTTGGCCGGGTGGACGAATATGTCGCATATGGGGTTACCACAGGGTGTTAGGTATGGAGCGGAA
GGACAGTATCGGATCTTTTCTGGCGGGTCCAATGCGCAAAGCTCGTTGATACAGACACTGGACATCCTTTTGGGT
GTGAAACACACTGCGAATGCTGCGCATTCTTCCCAAGGAGACAGCAAGATCAATTATCTGGACGAAATGAAAAAA
TACATGCCAAGAGAACATCGTGAATTTCTTTACCATTTGGAATCGGTATGTAATATTCGCGAATACGTTTCTCGT
AACGCTTCTAACCGTGCATTGCAAGAGGCCTATGGACGTTGCATTTCCATGCTGAAAATATTTAGAGATAACCAC
ATTCAAATTGTTACCAAGTACATTATTTTGCCCTCCAATTCAAAGCAACACGGCTCCAATAAGCCGAACGTACTA
AGTCCAATCGAGCCCAATACCAAAGCAAGTGGTTGTTTAGGGCATAAAGTTGCCTCATCTAAGACTATCGGAACC
GGTGGTACAAGATTAATGCCCTTCTTAAAGCAGTGCAGGGACGAGACTGTCGCCACTGCGGACATTAAAAATGAA
GATAAAAATTGA

>YEL058W 1.79 Hot
ATGAAGGTTGATTACGAGCAATTGTGCAAACTCTACGATGACACGTGCCGCACAAAGAATGTGCAGTTCAGTTAC
GGTACGGCCGGATTCAGAACGCTGGCCAAGAATTTGGATACGGTGATGTTCAGTACTGGTATACTGGCGGTTCTC
AGGTCGCTGAAGCTTCAGGGTCAGTATGTGGGGGTGATGATCACGGCGTCGCACAACCCATACCAGGACAACGGG
GTCAAGATCGTGGAACCAGACGGATCGATGCTTTTGGCCACATGGGAGCCATATGCCATGCAGTTGGCCAATGCG
GCCTCTTTTGCCACTAATTTTGAAGAATTTCGTGTTGAGTTGGCCAAGCTGATTGAACACGAAAAGATTGATTTG
AATACAACCGTCGTGCCTCACATCGTGGTTGGGAGAGACTCTAGGGAAAGTAGTCCATACTTGCTGCGCTGCTTG
ACTTCCTCCATGGCCAGCGTCTTCCACGCGCAAGTTTTGGACCTAGGCTGTGTCACTACGCCTCAATTGCATTAC
ATTACTGATTTGTCCAACAGGCGGAAACTGGAAGGAGACACAGCGCCAGTTGCCACAGAACAGGACTACTATTCG
TTCTTTATAGGAGCCTTCAACGAGCTCTTCGCCACGTATCAGCTGGAGAAGAGGCTGTCTGTCCCAAAATTGTTC
ATAGACACAGCCAATGGTATCGGTGGTCCACAGTTGAAAAAACTACTGGCCTCCGAAGATTGGGACGTGCCAGCG
GAGCAAGTTGAGGTAATCAACGACAGGTCCGATGTTCCAGAACTGTTGAATTTTGAATGCGGTGCGGATTATGTG
AAGACTAACCAGAGATTACCCAAGGGTCTTTCTCCATCCTCGTTTGATTCGCTATATTGCTCCTTTGATGGTGAC
GCAGACAGGGTTGTGTTCTACTATGTCGACTCAGGATCAAAATTTCATTTGTTGGATGGTGACAAAATTTCCACT
TTGTTTGCAAAGTTCTTGTCTAAACAACTAGAATTGGCACACCTAGAACATTCTTTGAAGATTGGTGTTGTGCAA
ACTGCCTATGCAAACGGCAGTTCCACCGCTTACATAAAAAATACGTTGCACTGTCCCGTGTCTTGCACTAAGACA
GGTGTTAAACACTTGCATCATGAAGCTGCCACTCAGTACGATATTGGCATTTATTTCGAAGCAAATGGACATGGT
ACGATTATATTCAGCGAAAAATTTCATCGAACTATCAAATCTGAATTATCCAAGTCCAAGTTAAATGGTGATACG
TTAGCTTTGAGAACTTTGAAGTGTTTCTCTGAATTGATTAATCAGACCGTGGGAGATGCTATTTCAGACATGCTT
GCTGTCCTTGCTACTTTGGCGATTTTGAAAATGTCGCCAATGGATTGGGATGAAGAGTATACTGATTTGCCCAAC
AAGCTGGTTAAGTGCATCGTTCCTGATAGGTCAATTTTCCAAACCACGGACCAGGAAAGAAAATTGCTCAATCCA
GTGGGGTTGCAAGACAAGATAGATCTTGTGGTAGCCAAGTATCCCATGGGAAGAAGCTTTGTCAGAGCCAGTGGT
ACGGAGGATGCGGTGAGGGTTTATGCGGAATGTAAGGACTCCTCTAAGTTAGGTCAATTTTGTGACGAAGTGGTG
GAGCACGTTAAGGCATCTGCTTGA

>YNL125C 1.67 Hot
ATGTCAACGCACTCAAACGACTACTTTTCTGCTTCTTCCGGAATGGTCTCTGAGACATCGTCCGAGGTTTCTTCG
ATAAACTCTTCACAGCCTGTATCATTCTCTAAGGCTTCTATTGCTGCTCCGGTTCCATGCTCTGATCTACACAGC
ACCAAGTCGAACGATGCATCGAGAAAATTGTCTATTAGTAGGACGTTAACTAATCGGCTCAACGACATTAAAAAG
GCTGTCGATGACGACAACTTGCAGACGGAAGAAAATTCCGCAGACGTTAATAAAATATTAGAATCTAGATTTGAC
GTGGCCGATGCCATTAGGCTACAGCACAATGAGTCAGTCCAGTCAAAGTTAAACATCCCAGTCACACACACCACG
ACTGCAGGCGCCTCGTTGTCGGCACCATCTTCCTCTGCTTTCTCTGCTTCTTCTATTCAAAATGATACTACAGAA
CATAAAGCTTCCATGGACTCCAAACTCATGAGGAATAGACTATATCCGGCTTCCACGAAACACTCCGGTAAGGAT
CTTGAGGCCCAAGGAATAACCGAATTCGAGCCTGATGAACCGACTGTAAAAAAAGTATTCACCAACAAGTCTACC
GGGCAGCTGGAACTGCCCCCCGACGGTGGTTATGGCTGGGTCGTGACATTCTGTGTGTTCTTGACCATGTTTTCC
ACGTGGGGCTGCAACGCATCCTTCGGTGTCGACCTTGCCTACTACTTAAACCATGATACTTACCCTGGTGCTTCG
AAGTACGATTATGCCTTAATTGCTGGCCTAACTGTCTTTCTCGGTCAACTCTTATCCCCCCTTGTGATGGCACTG
ATGAGAATAATTGGTCTGCGGACCACCATGCTTTTTGGTGATGCTGTAATGCTTGCCGCATATCTCTTGGCCTCC
TTTACTACCAAGTTATGGCAATTGTATGTCACCCAAGGTTTTATGGTCGGTTGTTCAATATCACTGATTTTCGTT
CCAGCAACAACCGTCTTACCAGGATGGTTCTTGAAAAAAAGAGCTGTCGCAATGGGTGTCTCATTATTGGGTACC
GGTGCTGGTGGTGTCGTTTACGGTTTGGCTACAAACAAAATGCTTTCTGACTTTGGAAATACCCGGTGGTGCCTT
CGTATCATAGGCATATCGTGTAGCATAAGTGTTCTAGTTGCTATTGCGCTCTTAAAAGAAAGAAACCCTACACCT
GCCATAGGATTGAAATCGCCTCGGGCCATGTTTGAACAGCTCAAAGCAATGTTTTCATTAAAGGTTATAACTAAG
CCATTTGTGGTACTTATTGCATTATGGTTCATGTTCGCATTATTTGCCTACAATATGATGGTTTTTACTTTATCT
TCATACGCAATCTCGAAAGGATTATCATCGCACGACGCTTCCACATTGACTGCCATTTTGAACGGCTCGCAATCC
ATCGGAAGACCTCTGATGGGTTTAGCGGGAGATAAGTTTGGTAGGGCAAACGTAACGATCGTATTAACCACTTTG
TTAACAATATATATGTTTGCGTTCTGGATCCCCGCTCATACGTTTGTTCAACTCATCTTTTTTTCAATTCTAGTT
GGCTCATGCGTTGGTGTCGCCAACGTCATGAATACCGTTCTGATTGCCGATATGGTTAAACCAGAAGAGTTTTTG
CCCGCTTGGGCCTTCGTCAACTACTGTGGTGCGCCTTTCTTATTGGTTTGTGAGGTGATTGCCCAGGCATTGACG
GTGGAGAAAGATAAGAGCAATCCTTACTTACATGCACAAATTTTTTGCGGTTGCTGCTTTATTGCCGCACTAATT
TTAATTTCTATCCTTCGTGAATATTCTATCAGGATGAAATTAACGGAAAGACAAGCTATGACAAACGAGAAGTTA
AAAGAATGGAAGGCAAGCGAATACGATACCGATTCTGCCGATGAAGATTGGGGTAAATTAAAAGAAAGAAAGACT
AAATATGACCTTCTTTTAGGTCCGGGCATTAAAAAATACTTCCTAAGAATGGTATATCCAATGAAGGTCTAG

>YEL045C 2.13 Hot
ATGAAATGTCACGCGAAACGGACCCTTGCCTTTTTGGCGACGGCACTTCCCCTATCTGGAAAAAGCCGAGCATGC
ACCCGTACCCCACAAAGCTTCGCTTCCGGTTTCCGGGCAGCAGCGCCGTTTCTTTTTTCCCGCTGCTTCGCCCTT

TGTATTACTCATTGCGCACTTTTTCACTTGCCATATTCGTTCACCGGTTTTTCTTTTTATTTCTTCGTCTTTTTT
CGTCTTTTTCTTCACTGGATATACGCTTTTTGCATTTGCAATAGCACATATGTGTATATATATAAGCAAGTGTTG
AGCTTGCCTGTCAAATCCTCCATGTGTCCTTCTCGTCTATCTTGTTCTGTCTGGTATAGAGTAATACTTACATAC
ATATACGTACATTGTTTCCGCTGGCTAGTTCGTAACCACCTCCTTTCCTAG

>YJR147W 1.61 Hot
ATGGATGCAACATCGAGGATGGAGCAGCCTGATGTCTTTGTAAGCAAACTCTACCACCTGCTGCAGGGGAACGCT
TACTCGAACATAATACAATGGTCGACTGATGGCAGCAAGCTCGTCATTTGGAATCCGGACCAGTTCACCAAAGTC
ATCCTAGAGCGATTTTTTGGTATTCACACCTTTGCAGCATTCGTTAAGCAATTGAGCAAATATAACTTCCAGAAG
GCGGGCCGCCCGGACTGCGTGGAGTTTTCCAACATTCATTTTCAAAAAGATAACATTAATAGCCTCTCACTGGTT
AAAGCTCATCAGTCTGCCGCCACTCCCAATGTCGCCGCCGTCAATAATATGAATAAGCAGTGTACTTTTCACTGG
GACCCTTTCAAAGTGAACTCCATTCTAAGCAAGGCCATCGGCAAGCCTTCCTTCGAGAAATTAGTGAAAAATGTT
GACAGGCTTCAGGGCAATCTTGATGAGCTTAAGTCGACAAACGCAGATAGTCTGCGTATTATAAGGGAAATTAAC
GCCAGCTTACAGACAATCTCCTACCACCAGTTTCACGCCTACCAAACCGCTAACTTCCTTCAAGAAAATTTTGAA
GCCATCAAAAAGGTTGTATGCCCGGACTCTTGTCTACAACACCAGCAACGCCAACCAAAGCGCCCCAAACGCTAC
TCGTTGCTGTTACTAATACCAAACGCATCCGAATTGTCAGAAACACCTTTGATGCGCTTCGCCGGCGTGTTCGAA
TTTATGAACTGCTCTTTGGATACGGCCACTCAGTGGCACCCGCAGTTGCATCCCGAGGCATACGACCTTCTTTTC
GTCACCGTGTCGCCCAACATGCAACAAGAACATCTCATCTACTTCAAGAGACTGAGAAACCTACTCCCAAGCTTT
CCTGTAATAGCCATTATTAACAGACCCGTCTCCCCTCAAGATACCAGCATTGCTCCCTCCAACTACTCTCGCTAT
TATTTCCACCATTTTTTGCAGTTGGGGTTCAGTGACATCCTAGTTAGCCCCTTCACCCCAACACAACTTATAACG
CTCCTTTCCAAACATCTTCGAACGTGA

>YKR012C 1.71 Hot
ATGAACGTCGTTAGAGTCTGGCCATTTTCTACACGAACTATACCTTGTACCACTACAGTGGCAGCTTGGTGGACA
TGTTGCGTGACAGTCACAGCAACTGGGGCAGATAAAGCGACAGATGCAGATGCGGCCAGTAGTGAGACTTTAGAA
AATTTCATCTTTGTAAGTTTTATTAAAGGAATGGTATTGTTTTATGATGGGCTGTTTCGAGGAACGATGATACCA
AGAGCCAACAGATCCATCACAGCAATATGTAACACTCTGCGACAAGCATATATATATACAAAGATCCAAAGCATT
GCCTTTGAACGACGTCTATGTTTCCAAAACATTACATATAGAGAAGTGCGTGAGATACACGAAATGTACTGTAAA
TAG

>YIL136W 1.61 Hot
ATGTCATCAAGAATAATTGTCGGCAGTGCAGCATTGGCAGCTGCCATCACAGCTAGCATCATGGTCAGAGAACAG
AAGGCCAAGGGTCAGAGAAGAGAGGGCAACGTCTCCGCTTACTACAACGGCCAGGAGTACGGCAGTTCAGCACCC
CCACAGTTGGGAAAGCTACATAACATAAAGCAAGGCATAAAGGAAGATGCCTTGTCGTTAAAAGACGCGCTTCTG
GGCGTATCTCAAAAGGCTAGGGAAGAGGCTCCAAAGGTAACTAAGCGTGTGATATCACCGGAAGAGGATGCTCAG
ACACGCAAGCAGCTAGGCCAAAAAGCCAAGGATTCTTCCTCGCAAAGCATCTTCAATTGGGGGTTTAGTGAGGCT
GAAAGAAGGAAAGCCATAGCCATCGGGGAATTTGATACTGCTAAGAAGCGTTTCGAAGAGGCAGTGGATCGTAAT
GAGAAGGAGCTCTTGTCCACGGTGATGAGAGAGAAGAAGGCCGCTCTGGACAGAGCATCCATTGAGTACGAAAGG
TACGGGAGAGCCAGAGACTTTAATGAGCTTTCGGACAAGCTAGACCAACAGGAAAGGAACAGTAATCCTTTGAAA
CGCCTGTTGAAGAATAACACGGGTGACGCTAATACTGAAGAAGCCGCTGCAAGAAGTGTCCAAGGCTGGGGTGAT
ACGGCACAGGAGTTTGGTAGAGAAGAGTTGGAGGAAGCCAAGAGAAATGCTTCTTCAGAGCCAAGCGAGGCGCAA
AAACGTCTTGACGAGCTGAAGAAGATCAAGGAAAAGGGCTGGTTTGGTTACAACAAAGGGGAGCAAAGCGAGCAA
CAGATTGCTGAACGGGTAGCCAGAGGTTTAGAAGGATGGGGTGAAACAGCCGCTCAACTTTCCAAGGACGAAATG
GACGATTTAAGATGGAATTATGAGAATTCAAAGAAACAACTGGATAAGAACGTGTCCGATGCCATGGACTCGTTA
TCTAAGGCGAAGGAGGACTTGAAACAGTACGGCAGCCACTGGTGGTCTGGATGGACTTCCAAGGTCGACAATGAC
AAGCAGGCTTTAAAAGATGAGGCCCAAAAGAAGTACGATGAAGCGTTGAAAAAGTACGATGAAGCCAAGAACAAA
TTCAAAGAATGGAATGATAAGGGTGATGGTAAATTCTGGAGCTCGAAAAAGGACTAG

>YGR204W 1.69 Hot
ATGGCTGGTCAAGTGTTGGACGGCAAAGCATGCGCTCAGCAGTTTAGAAGCAATATTGCTAATGAAATCAAAAGC
ATTCAAGGTCACGTGCCTGGGTTTGCACCTAACCTTGCCATCATTCAAGTAGGCAACAGACCAGACTCAGCCACA
TATGTACGCATGAAGCGTAAGGCAGCTGAAGAGGCCGGCATTGTTGCTAATTTCATTCATTTAGATGAATCCGCT
ACTGAATTTGAAGTTCTGCGTTACGTGGACCAGCTGAATGAGGACCCACATACACACGGTATTATCGTGCAACTA
CCATTACCCGCTCATTTGGACGAGGATAGAATCACCTCGAGAGTGTTGGCAGAAAAGGACGTGGACGGGTTCGGG
CCCACCAACATTGGCGAATTGAATAAGAAGAACGGACACCCATTCTTTTTGCCCTGCACGCCCAAGGGGATCATT
GAGCTGCTTCACAAGGCCAACGTCACGATTGAAGGTTCCCGGTCCGTTGTGATCGGAAGATCTGACATTGTTGGC
TCTCCTGTTGCAGAATTGTTAAAATCTCTAAACTCCACCGTCACCATCACTCATTCTAAAACCCGTGATATCGCA
TCATACTTACACGACGCGGACATCGTAGTCGTTGCCATCGGCCAACCAGAATTTGTGAAGGGTGAATGGTTCAAA
CCAAGAGACGGCACTTCCAGTGATAAGAAAACCGTGGTAATTGATGTTGGCACCAACTACGTTGCTGATCCTTCC
AAAAAGTCCGGTTTCAAATGTGTTGGTGACGTTGAGTTCAATGAAGCAATCAAATACGTCCATCTAATCACTCCA
GTGCCCGGTGGTGTGGGCCCCATGACGGTGGCTATGTTAATGCAAAATACCTTGATTGCTGCCAAACGCCAAATG
GAAGAATCCTCGAAGCCTTTGCAGATTCCTCCCTTGCCATTGAAGTTGCTAACACCTGTTCCTTCCGATATAGAC
ATCTCCAGAGCACAACAGCCAAAGCTTATCAACCAGCTTGCTCAAGAATTGGGTATTTACTCTCATGAGTTGGAG
CTGTACGGACATTACAAGGCCAAAATTTCTCCTAAAGTCATCGAAAGGCTGCAGACGCGCCAAAATGGTAAGTAC
ATCTTGGTGTCTGGTATCACACCAACACCACTGGGAGAGGGTAAATCCACTACAACAATGGGTCTTGTCCAGGCA
CTAACGGCTCACTTGGGCAAGCCAGCCATTGCGAACGTCAGACAACCCTCCCTAGGACCCACTTTAGGTGTCAAA
GGTGGTGCTGCGGGTGGTGGTTATTCCCAAGTCATCCCAATGGACGAATTCAACTTACATTTGACTGGTGACATT

CACGCCATTGGTGCGGCTAACAACCTACTTGCTGCCGCTATTGACACTAGAATGTTCCATGAGACCACTCAAAAG
AACGACGCTACCTTCTACAACAGACTAGTGCCTAGAAAGAACGGAAAGAGAAAGTTTACTCCCTCCATGCAAAGA
AGATTGAACAGACTGGGTATTCAAAAGACCAACCCCGATGATCTAACACCCGAAGAGATCAACAAATTCGCCAGA
TTGAACATTGACCCGGACACTATTACTATCAAGAGGGTGGTCGATATCAACGACAGAATGTTAAGACAAATCACC
ATTGGTCAAGCCCCTACCGAGAAGAACCACACAAGAGTTACTGGATTCGATATCACCGTTGCTTCTGAATTGATG
GCTATTCTTGCTCTTTCAAAGGACTTGAGGGACATGAAGGAACGTATTGGAAGAGTCGTTGTTGCTGCTGACGTA
AACAGGTCTCCAGTCACTGTTGAAGATGTGGGTTGTACCGGTGCCTTAACCGCTTTATTAAGAGACGCTATCAAG
CCCAACTTGATGCAAACTTTAGAAGGTACTCCTGTCTTGGTCCATGCCGGCCCATTTGCCAACATCTCTATCGGT
GCCTCTTCTGTTATTGCTGATCGCGTGGCTTTGAAATTGGTTGGTACCGAGCCAGAGGCAAAAACAGAAGCTGGT
TATGTGGTTACTGAAGCAGGGTTCGATTTCACTATGGGTGGTGAAAGATTCTTCAACATCAAGTGCCGTTCCTCT
GGATTGACACCTAATGCTGTGGTCTTGGTTGCTACTGTTAGGGCATTGAAGTCACACGGTGGTGCTCCAGATGTC
AAACCTGGCCAACCTTTACCTTCCGCATACACTGAAGAGAATATCGAGTTTGTCGAAAAAGGTGCCGCTAACATG
TGTAAACAAATTGCCAACATTAAGCAATTTGGCGTCCCCGTCGTTGTCGCAATTAACAAGTTTGAAACTGACACT
GAAGGTGAAATAGCCGCCATTAGAAAAGCCGCTTTGGAAGCTGGTGCATTTGAAGCCGTAACCTCTAACCATTGG
GCCGAAGGTGGTAAAGGTGCTATCGACTTGGCCAAGGCCGTCATCGAAGCTTCCAACCAACCAGTGGACTTCCAT
TTCCTATATGACGTTAACTCCTCCGTTGAAGACAAATTAACTACTATCGTTCAAAAGATGTACGGTGGTGCCGCA
ATCGATATCTTGCCTGAAGCACAACGCAAGATTGACATGTACAAGGAACAAGGTTTCGGTAACTTGCCAATTTGT
ATCGCCAAGACACAATACTCTTTATCCCACGATGCAACTTTGAAAGGTGTTCCAACCGGGTTCACTTTCCCCATC
AGAGACGTCAGATTGTCTAATGGTGCTGGATACTTATACGCTCTTGCCGCCGAAATACAAACCATTCCTGGTTTG
GCTACCTATGCTGGTTACATGGCCGTGGAAGTCGATGATGACGGTGAGATCGATGGCCTGTTCTAA

>YJL175W 1.64 Hot
ATGTTTTTAACGTCACCATTTGAATCCTGTATTGTGCTGTCTTCCTTAATAGCAGGCTTATTGTTTTCATTGTCT
ACCGGGTTCGTAGGTATACTAGGAGTGTTTGCCTCATTGTTCGAAACAGAACTCTCGGTTTCGCCAAAGAGATTA
TCACTATCCTCATTATCTTGGCCAAAGACGTTCTGGGCCCTGCTTTCTTCTGTAGAAGGTGTTTCCTGGGAATCT
AGCTTATTCGCTTGCATAGTCGGCTGCTGCTTTGCCGTTACCGTTATCGCCTCACTTTCTGCATCGCGTGTGTTC
GGAACAGTTGCTTCATCCTTCAGAGACTCATCTTGTTGCTGCGATTCTTCACCAGCAGTATCTGTATTGGCAACC
CCTGCCACAGCTGCATTAGCGTTGCTATCACTATTGTTGTCATTGCCATGTTGGTCCACGTCCACGGAAGCGTTT
ACCGTGGACCCCTCACCCAGTGTATTCTCCATGCTTGCAAACCGTATCACAATTGGTCTCTGA

>YGR248W 1.78 Hot
ATGGTGAAATTACAAAGGTTTAGCGAAAAGAAAAGCCTCATACACGAATTCGGCAAGTTTATCCTTGAAAAGCAA
GAATCGGCGTTAACGGGCGACGCTGATGCAGTGTTCAATATCGCCATCAGTGGAGGATCGATGAACCAAGCGCTG
TACGAAAGTTTGGTAAATGACAAAAACATTTTTCCACATATTAAGTGGCCACAATGGAGAATCTTCTTCTGTGAC
GAAAGATTGGTTCCATTTGAGGATCCGCAAAGTAACTATGGTCAGTTCAAAAAAACAGTTTTGGACCCGCTAGTG
CATCAGGGCAACCAATTGAACTTAGGCCCCACTGTATACACTATCAACGAATCATTAATCGGTGGCGGTGAAACG
GCCAATAGAAAGATTGCCGAAGAATACGCTTCCATGCTGCCTGCATCATTCGACCTAATCTTACTCGGATGCGGA
GAAGATGGACATACATGCTCGTTGTTTCCCGGGGTTGAATTTAATTACCTTGTAGAAGAGATGGACCGCAAGGTT
TTATGGTGTAATAATTCGCCCAAGGCACCCAAGGACAGGATCACCTTTACATTAGCAGTAGTAGCCGAGGCTAAA
AGTGTGTGCTTTCTCGTTAGGGGAGCTGCTAAAAAGGCTATCATGCATGACGTGTTAATCGTAAAAAATAGCGAA
CTACCTAGTGTGCTGGTTAATGAAATGGTCGGAACCAAAGTAACTTGGTTTCTCGACGACGAAGCTGGCGCCTTG
ATTCCTGAAAACTGCTAA

>YFR026C 1.89 Hot
ATGACGCCCTATGCAGTAGCAATTACCGTGGCCTTACTAATTGTAACAGTGAGCGCACTCCAGGTCAACAATTCA
TGTGTCGCTTTTCCGCCATCAAATCTCAGAGGCAAAAATGGAGACGGTACTAATGAACAGTATGCAACTGCACTA
CTTTCTATTCCCTGGAATGGACCTCCTGAGTCATTGAGGGATATTAATCTTATTGAACTCGAACCGCAAGTTGCA
CTCTATTTGCTCGAAAATTATATTAACCATTACTACAACACCACAAGAGACAATAAGTGCCCTAATAACCACTAC
CTAATGGGAGGGCAGTTGGGTAGCTCATCGGATAATAGGAGTTTGAACGATCCGCAAACGATGCTATGGCCGGAA
AAGAAGGAAGACGAAAAAAAACTGCCAAGAAACTTTTAAAGGGGCCTGTTCGTGTACCAAAAGGTTTTGCAAGGGT
TATTTCTCTGTAAATATTTTTGGGATAAACCTGAATATATCTTATAGCTCTGGAAAGTGA

>YDL182W 1.79 Hot
ATGACTGCTGCTAAACCAAATCCATATGCTGCCAAACCGGGCGACTATCTTTCTAATGTAAATAATTTCCAGTTA
ATCGATTCGACGCTGAGAGAAGGTGAACAATTTGCCAACGCATTCTTCGATACTGAAAAAAAGATCGAAATTGCT
AGAGCCTTGGACGATTTCGGTGTGGACTACATCGAGTTAACCTCACCAGTAGCATCTGAACAATCAAGAAAGGAC
TGTGAAGCTATATGTAAACTAGGTTTAAAGGCCAAGATCCTTACACACATTCGTTGTCATATGGATGACGCCAAA
GTCGCCGTAGAGACTGGTGTCGACGGTGTCGATGTCGTTATCGGCACCTCCAAATTTTTAAGACAATATTCCCAC
GGTAAGGATATGAACTACATCGCCAAGAGTGCTGTTGAAGTCATTGAATTTGTCAAATCCAAAGGTATTGAAATC
AGATTTTCCTCTGAAGATTCCTTCAGAAGTGATCTCGTTGATCTTTTGAACATTTATAAAACCGTTGACAAGATC
GGTGTAAATAGAGTCGGTATTGCCGACACAGTTGGATGTGCCAACCCAAGACAAGTATATGAACTGATCAGAACT
TTGAAGAGTGTTGTTTCATGTGACATCGAATGCCATTTCCACAACGATACTGGTTGTGCCATTGCAAACGCCTAC
ACTGCTTTGGAAGGTGGTGCCAGATTGATTGACGTCAGTGTACTGGGTATTGGTGAAAGAAACGGTATCACTCCT
CTAGGTGGGCTCATGGCAAGAATGATTGTTGCCGCACCAGACTATGTCAAGTCCAAATACAAGTTGCACAAGATC
AGAGACATTGAAAACCTGGTCGCTGATGCTGTGGAAGTTAACATTCCATTCAACAACCCTATCACCGGGTTCTGT
GCATTCACACATAAAGCAGGTATCCATGCCAAGGCCATTTTGGCTAACCCATCTACCTACGAAATCTTGGACCCT
CACGATTTCGGTATGAAGAGGTATATCCACTTCGCCAACAGACTAACTGGCTGGAACGCCATCAAAGCCAGAGTC

```
GACCAGTTGAACTTGAACTTGACGGATGACCAAATCAAGGAAGTTACTGCTAAGATTAAGAAGCTGGGTGATGTC
AGATCGCTGAATATCGATGATGTTGACTCTATCATCAAGAACTTCCACGCAGAGGTCAGCACTCCTCAAGTACTA
TCTGCAAAAAAGAACAAGAAGAATGACAGCGATGTACCGGAACTGGCCACCATCCCCGCCGCCAAGCGGACTAAG
CCATCCGCCTAA

>YAL038W  3.27   Hot
ATGTCTAGATTAGAAAGATTGACCTCATTAAACGTTGTTGCTGGTTCTGACTTGAGAAGAACCTCCATCATTGGT
ACCATCGGTCCAAAGACCAACAACCCAGAAACCTTGGTTGCTTTGAGAAAGGCTGGTTTGAACATTGTCCGTATG
AACTTCTCTCACGGTTCTTACGAATACCACAAGTCTGTCATTGACAACGCCAGAAAGTCCGAAGAATTGTACCCA
GGTAGACCATTGGCCATTGCTTTGGACACCAAGGGTCCAGAAATCAGAACTGGTACCACCACCAACGATGTTGAC
TACCCAATCCCACCAAACCACGAAATGATCTTCACCACCGATGACAAGTACGCTAAGGCTTGTGACGACAAGATC
ATGTACGTTGACTACAAGAACATCACCAAGGTCATCTCCGCTGGTAGAATCATCTACGTTGATGATGGTGTTTTG
TCTTTCCAAGTTTTGGAAGTCGTTGACGACAAGACTTTGAAGGTCAAGGCTTTGAACGCCGGTAAGATCTGTTCC
CACAAGGGTGTCAACTTACCAGGTACCGATGTCGATTTGCCAGCTTTGTCTGAAAAGGACAAGGAAGATTTGAGA
TTCGGTGTCAAGAACGGTGTCCACATGGTCTTCGCTTCTTTCATCAGAACCGCCAACGATGTTTTGACCATCAGA
GAAGTCTTGGGTGAACAAGGTAAGGACGTCAAGATCATTGTCAAGATTGAAAACCAACAAGGTGTTAACAACTTC
GACGAAATCTTGAAGGTCACTGACGGTGTTATGGTTGCCAGAGGTGACTTGGGTATTGAAATCCCAGCCCCAGAA
GTCTTGGCTGTCCAAAAGAAATTGATTGCTAAGTCTAACTTGGCTGGTAAGCCAGTTATCTGTGCTACCCAAATG
TTGGAATCCATGACTTACAACCCAAGACCAACCAGAGCTGAAGTTTCCGATGTCGGTAACGCTATCTTGGATGGT
GCTGACTGTGTTATGTTGTCTGGTGAAACCGCCAAGGGTAACTACCCAATCAACGCCGTTACCACTATGGCTGAA
ACCGCTGTCATTGCTGAACAAGCTATCGCTTACTTGCCAAACTACGATGACATGAGAAACTGTACTCCAAAGCCA
ACCTCCACCACCGAAACCGTCGCTGCCTCCGCTGTCGCTGCTGTTTTCGAACAAAAGGCCAAGGCTATCATTGTC
TTGTCCACTTCCGGTACCACCCCAAGATTGGTTTCCAAGTACAGACCAAACTGTCCAATCATCTTGGTTACCAGA
TGCCCAAGAGCTGCTAGATTCTCTCACTTGTACAGAGGTGTCTTCCCATTCGTTTTCGAAAAGGAACCTGTCTCT
GACTGGACTGATGATGTTGAAGCCCGTATCAACTTCGGTATTGAAAAGGCTAAGGAATTCGGTATCTTGAAGAAG
GGTGACACTTACGTTTCCATCCAAGGTTTCAAGGCCGGTGCTGGTCACTCCAACACTTTGCAAGTCTCTACCGTT
TAA

>YBR222C  1.59   Hot
ATGACAAGTGCCGCTACTGTTACTGCTTCGTTCAACGATACTTTTAGCGTATCCGATAATGTCGCCGTTATTGTT
CCTGAAACGGACACTCAGGTGACCTACAGGGATCTATCCCACATGGTGGGTCACTTCCAGACCATGTTCACAAAT
CCTAATTCTCCATTGTACGGAGCTGTTTTCAGACAAGATACAGTGGCGATATCCATGCGTAATGGGCTGGAATTT
ATCGTCGCTTTCCTCGGTGCTACTATGGACGCTAAAATTGGCGCGCCCTTGAATCCCAATTATAAGGAAAAGGAG
TTCAATTTTTATTTGAATGACCTGAAATCTAAGGCGATTTGCGTCCCAAAGGGTACCACAAAGTTACAGAGTTCT
GAAATTCTAAAATCTGCCTCCACGTTTGGATGTTTTATCGTAGAGCTGGCCTTCGATGCGACCAGGTTTAGGGTA
GAGTATGATATATACTCTCCAGAGGACAACTACAAAAGGGTTATTTACCGGTCTTTGAACAACGCCAAATTTGTC
AACACAAATCCCGTTAAATTCCCTGGGTTTGCCCGTTCCAGTGACGTTGCCCTGATTTTGCATACCAGTGGTACC
ACCTCCACTCCAAAAACGGTGCCTTTGTTACATTTGAACATTGTGAGAAGCACGTTGAACATTGCTAACACTTAC
AAGCTAACGCCCTTGGACAGATCTTATGTCGTGATGCCTCTTTTCCACGTCCATGGGTTAATTGGTGTTTTACTT
TCCACTTTTAGAACTCAGGGTTCTGTTGTGGTTCCCGATGGATTCCATCCAAAGTTATTCTGGGACCAATTTGTT
AAGTACAACTGTAATTGGTTCAGTTGCGTTCCCACAATAAGCATGATTATGCTGAACATGCCCAAACCAAACCCT
TTCCCACACATTAGATTCATCAGATCGTGTTCTTCTGCTTTGGCTCCAGCAACGTTCCATAAGCTGGAGAAGGAA
TTCAATGCACCTGTCTTGGAGGCCTATGCGATGACCGAAGCATCACATCAAATGACCTCAAACAATCTGCCTCCA
GGAAAGAGAAAGCCTGGTACTGTGGGCCAGCCACAAGGAGTCACCGTCGTCATTCTAGATGACAATGACAATGTC
TTGCCCCCGGGCAAAGTCGGCGAAGTTTCCATCAGAGGCGAAAACGTCACTTTGGGGTATGCTAATAATCCAAAA
GCTAACAAGGAGAACTTCACCAAGAGAGAGAACTATTTCAGAACCGGTGACCAAGGTTATTTCGACCCTGAGGGG
TTTTTGGTCCTTACAGGCAGAATCAAAGAGCTTATCAACAGGGGTGGTGAAAAGATTTCACCCATTGAGCTCGAC
GGCATTATGCTATCGCATCCAAAGATCGATGAAGCCGTTGCATTTGGTGTTCCCGACGATATGTACGGCCAAGTA
GTTCAAGCCGCCATTGTTTTGAAGAAGGGAGAAAAAATGACCTACGAAGAACTGGTGAACTTCTTAAAGAAGCAC
CTAGCCTCTTTCAAAATTCCAACCAAGGTGTACTTTGTTGATAAGCTACCAAAAACCGCTACAGGTAAAATCCAG
AGAAGAGTTATCGCAGAAACTTTTGCTAAGAGCAGCAGAAATAAGAGTAAGTTGTAG

>YCR050C  1.96   Hot
ATGGTGGCAGTTCATAAGGTCAGGTACAACGTCATAATGATACTGGGCCCTGAACAGACACCAAACGAAAAGACA
ACGTTGGACAACTGCGGTCTTGCCCGTAGAAACTTGGTGTTGCTCAAAGCAGTCCACACAAACTGCGACAGTTGG
AACATGAACAGATATCCTCTAACCCTTCTGAAAATGGCGAACATGGCCATTTCGTGGAACACGGCACTCAAGAAA
AAAGTAAATAATGTAGCTTGGCTCTTGCTCAAATGCAATGCGCCCATGGAGCTGTGGTACACGTGTCTTAGTAAA
AATTTGTGA

>YIL046W  2.5Hot
ATGAGGAGAGAGAGGCAAAGGATGATGAGTTTCGAGGACAAGGACAAGGACGACCTTGACAATAGTAATAGTAAT
AACAGCAGTGAAATGACAGATACGGCGATGATGCCACCATTAAAGAGATTGCTTATTACGGGCAGTAGCGATGAT
TTGGCACAAGGATCATCGGGTAAGAAGAAGATGACGATGGCGACGAGGTCGCCATCGTCATCACCCGATTTGGCG
ACAAACGACAGCGGCACTAGGGTACAGCCATTGCCAGAATATAACTTCACCAAGTTCTGCTATCGGCATAACCCG
GACATTCAGTTCTCACCAACTCATACAGCGTGCTACAAGCAGGATTTGAAACGAACGCAAGAGATTAATGCTAAT
ATCGCGAAGCTACCCCTGCAGGAGCAATCCGACATCCACCACATTATCTCGAAGTACAGCAATTCCAATGACAAG
ATACGGAAGCTGATTCTGGATGGGATCCTATCGACGAGTTGCTTCCCACAGCTTTCCTACATTTCGTCACTCGTT
```

ACACACATGATCAAGATCGACTTCATCAGCATTCTGCCGCAGGAGCTGTCGCTGAAGATCTTGAGTTATCTGGAT
TGCCAATCTCTTTGCAACGCCACGAGAGTGTGCCGCAAGTGGCAGAAGCTCGCGGATGACGACAGGGTATGGTAC
CACATGTGCGAGCAGCACATAGACAGGAAATGTCCCAACTGTGGCTGGGGGCTGCCTCTTTTGCACATGAAACGT
GCGCGGATACAACAGAATAGTACAGGATCTAGCAGCAACGCAGATATCCAGACGCAAACTACGCGACCTTGGAAA
GTCATCTACAGAGAACGGTTCAAAGTGGAGTCAAACTGGAGAAAGGGCCACTGCAGGATTCAGGAATTCAAGGGC
CACATGGATGGTGTGTTAACGCTCCAGTTTAACTACAGGCTTTTGTTCACAGGCTCGTACGACTCCACCATAGGT
ATATGGGACTTATTCACGGGGAAGCTAATACGAAGGCTCAGCGGCCATTCGGACGGCGTCAAGACATTATATTTT
GACGATAGAAAGCTGATTACGGGCTCGCTCGACAAGACGATCCGTGTTTGGAACTACATAACCGGTGAATGCATT
TCCACGTATCGAGGCCACTCGGATAGCGTTCTGAGCGTAGATTCATACCAGAAGGTTATCGTTTCCGGCAGTGCT
GACAAGACGGTCAAGGTATGGCACGTGGAGTCCAGGACATGCTACACCTTGAGAGGCCACACGGAATGGGTTAAT
TGCGTCAAATTGCATCCGAAAAGCTTTTCATGTTTTAGTTGCAGTGACGATACCACAATCCGAATGTGGGATATC
AGGACCAATTCATGCCTAAAAGTGTTCAGGGGTCATGTAGGGCAGGTGCAAAAGATCATACCGCTTACCATTAAG
GATGTAGAGAATCTAGCCACCGACAACACTTCTGATGGCAGCTCTCCGCAGGATGACCCAACAATGACTGATGGT
GCAGACGAATCAGACACACCGTCGAACGAGCAAGAAACTGTCTTAGATGAAAACATACCTTATCCAACACATCTA
CTATCTTGCGGACTGGATAACACAATCAAACTATGGGACGTCAAAACCGGTAAATGCATAAGAACACAGTTTGGG
CACGTGGAAGGTGTTTGGGACATCGCCGCTGACAACTTCAGAATTATAAGTGGTTCTCACGACGGAAGCATTAAG
GTCTGGGACTTGCAAAGCGGGAAGTGTATGCACACGTTCAACGGTCGAAGACTACAAAGAGAAACTCAGCACACA
CAAACACAATCCTTGGGTGATAAAGTCGCCCCTATCGCTTGTGTTTGTATTGGAGATTCAGAATGCTTTAGTGGT
GATGAATTTGGGTGCGTAAAAATGTACAAATTCGATCTCAATGATTAG

>YLR355C 1.92 Hot
ATGTTGAGAACTCAAGCCGCCAGATTGATCTGCAACTCCCGTGTCATCACTGCTAAGAGAACCTTTGCTTTGGCC
ACCCGTGCTGCTGCTTACAGCAGACCAGCTGCCCGTTTCGTTAAGCCAATGATCACTACCCGTGGTTTGAAGCAA
ATCAACTTCGGTGGTACTGTTGAAACCGTCTACGAAAGAGCTGACTGGCCAAGAGAAAAGTTGTTGGACTACTTC
AAGAACGACACTTTTGCTTTGATCGGTTACGGTTCCCAAGGTTACGGTCAAGGTTTGAACTTGAGAGACAACGGT
TTGAACGTTATCATTGGTGTCCGTAAAGATGGTGCTTCTTGGAAGGCTGCCATCGAAGACGGTTGGGTTCCAGGC
AAGAACTTGTTCACTGTTGAAGATGCTATCAAGAGAGGTAGTTACGTTATGAACTTGTTGTCCGATGCCGCTCAA
TCAGAAACCTGGCCTGCTATCAAGCCATTGTTGACCAAGGGTAAGACTTTGTACTTCTCCCACGGTTTCTCCCCA
GTCTTCAAGGACTTGACTCACGTTGAACCACCAAAGGACTTAGATGTTATCTTGGTTGCTCCAAAGGGTTCCGGT
AGAACTGTCAGATCTTTGTTCAAGGAAGGTCGTGGTATTAACTCTTCTTACGCCGTCTGGAACGATGTCACCGGT
AAGGCTCACGAAAAGGCCCAAGCTTTGGCCGTTGCCATTGGTTCCGGTTACGTTTACCAAACCACTTTCGAAAGA
GAAGTCAACTCTGACTTGTACGGTGAAAGAGGTTGTTTAATGGGTGGTATCCACGGTATGTTCTTGGCTCAATAC
GACGTCTTGAGAGAAAACGGTCACTCCCCATCTGAAGCTTTCAACGAAACCGTCGAAGAAGCTACCCAATCTCTA
TACCCATTGATCGGTAAGTACGGTATGGATTACATGTACGATGCTTGTTCCACCACCGCCAGAAGAGGTGCTTTG
GACTGGTACCCAATCTTCAAGAATGCTTTGAAGCCTGTTTTCCAAGACTTGTACGAATCTACCAAGAACGGTACC
GAAACCAAGAGATCTTTGGAATTCAACTCTCAACCTGACTACAGAGAAAAGCTAGAAAAGGAATTAGACACCATC
AGAAACATGGAAATCTGGAAGGTTGGTAAGGAAGTCAGAAAGTTGAGACCAGAAAACCAATAA

>YAL042W 1.67 Hot
ATGAAAAGGTCCACGTTGCTGTCGCTGGACGCATTCGCTAAGACCGAAGAGGACGTACGAGTCCGCACCAGGGCC
GGCGGGCTGATCACTTTATCGTGCATCTTGACCACGTTATTTCTGCTGGTGAACGAGTGGGGACAGTTCAATTCT
GTGGTAACAAGGCCACAATTGGTGGTGGACCGTGACCGACACGCAAAGCTGGAGCTTAATATGGATGTGACATTT
CCATCGATGCCATGTGACCTGGTGAATCTCGATATTATGGACGACTCTGGAGAGATGCAACTAGACATTCTTGAC
GCAGGGTTCACGATGTCTAGGTTGAATAGCGAGGGTCGCCCCGTGGGAGATGCTACTGAGTTGCATGTGGGTGGG
AACGGCGACGGAACCGCGCCGGTTAATAACGATCCTAACTATTGTGGGCCATGTTACGGTGCCAAAGATCAGTCG
CAGAATGAGAATCTAGCACAGGAAGAGAAGGTTTGCTGCCAAGACTGTGATGCAGTGAGATCAGCATACTTGGAG
GCAGGCTGGGCTTTTTTCGACGGGAAGAATATCGAGCAGTGTGAAAGAGAGGGCTATGTCAGCAAGATTAACGAG
CACTTGAATGAAGGCTGCAGGATCAAAGGTTCTGCACAAATTAACAGAATTCAGGGGAATCTTCACTTTGCCCCT
GGAAAACCCTACCAGAATGCATATGGACATTTTCATGATACTTCTTTGTACGACAAGACTTCGAATTTGAACTTC
AACCACATCATCAATCATTTGAGCTTTGGGAAGCCGATCCAGTCCCACAGTAAGTTGTTAGGAAACGATAAGCGC
CACGGCGGCGCCGTAGTTGCCACTTCTCCCTTGGACGGACGCCAGGTGTTCCCGGACAGGAACACACACTTTCAC
CAGTTCTCGTATTTTGCCAAGATTGTCCCCACCAGATATGAGTACTTGGATAATGTTGTCATTGAGACCGCGCAG
TTCAGCGCCACTTTTCATTCCCGACCTCTTGCCGGTGGAAGGGACAAGGATCATCCAAACACACTTCACGTTAGG
GGTGGTATCCCTGGTATGTTCGTCTTTTTCGAAATGTCTCCATTGAAAGTCATCAATAAGGAACAGCACGGGCAG
ACTTGGTCGGGCTTCATCTTGAATTGTATCACCAGCATTGGTGGTGTCCTAGCTGTGGGCACTGTCATGGACAAG
CTATTCTACAAAGCACAGAGATCGATCTGGGGCAAGAAGAGCCAGTAG

>YPR039W 1.7Hot
ATGGCTTCTTTTGACTATCTGTTTCATCCCTTCGTTCCTTGCACAATCTGTCCAGACTTTCCTCTTTACAAATCA
CCAGCCTTCCCGTCTTCTTGTCTTCATCACCCACGTTTATTGTTTAATGATAAAGCCTTTTGTCCTTTATTTTTG
GTTCCTTTTCCCGCTTCGTTTACCCGGTGGTTAACCTTTCTCTTCCATCTTGTAATATATAATAACAAAATGCAT
CACCATACTTACGCACCCCATATACATGACCTAAGGGCAGCTTTAGACACAACAGCTCCCCAGAAAAAATGTCCA
AAAGAAACACTCCACCGCTCAGATCATCAGGGATAA

>YEL077C 1.52 Hot
ATGACGCTTGGTAACAGTTATGACGCTTTTAATCATGATCCTTGGATGGATGTGGTGGGATTTGAGGATCCAAAT
CAAGTAACAAATCGAGACATTTCGAGGATAGTTTTGTATTCCTACATGTTTCTGAATACCGCGAAGGGCTGTCTG

GTTGAATACGCAACTTTTCGGCAGTACATGAGGGAACTTCCGAAGAATGCACCTCAGAAGCTGAATTTTCGGGAG
ATGCGTCAGGGGTTGATTGCCCTAGGACGGCACTGCGTAGGTAGCAGATTTGAAACAGATTTGTACGAGTCGGCG
ACGAGTGAACTCATGGCCAATCATTCCGTTCAAACAGGGCGAAATATTTACGGTGTGGATTCCTTTTCGTTAACT
AGTGTCAGTGGGACGACCGCCACTTTATTGCAGGAACGAGCTTCCGAGCGCTGGATTCAATGGTTAGGCCTTGAA
AGCGACTACCATTGTTCATTCTCTAGTACTCGGAATGCGGAAGACGTAGTGGCAGGTGAGGCGGCGAGTTCAGAT
CATCACCAAAAAATCTCAAGAGTAACGCGAAAAAGGCCCCGAGAGCCCAAGAGTACAAACGATATCCTCGTTGCA
GGCCAGAAACTCTTTGGCAGCTCCTTTGAATTCAGGGACTTGCATCAGTTGCGCTTATGTTATGAAATATACATG
GCAGACACACCCTCTGTGGCAGTACAGGCCCCACCGGGCTATGGTAAGACGGAGTTATTTCATCTCCCCTTGATA
GCACTGGCGTCTAAGGGCGACGTGAAATATGTGTCGTTTCTGTTTGTACCGTACACAGTGTTGCTTGCTAATTGC
ATGATCAGGTTGGGCCGATGCGGTTGCTTGAATGTGGCCCCTGTAAGAAACTTTATTGAAGAAGGTTACGATGGC
GTTACTGATTTATACGTGGGGATCTACGATGATCTTGCTAGCACTAATTTCACAGACAGGATAGCTGCGTGGGAG
AATATTGTTGAGTGCACCTTTAGGACCAACAACGTAAAATTGGGTTACCTCATTGTAGATGAGTTTCACAACTTT
GAAACGGAGGTCTACCGGCAGTCGCAATTTGGGGGCATAACTAACCTTGATTTTGACGCTTTTGAGAAAGCAATC
TTTTTGAGCGGCACAGCCCCTGAGGCTGTAGCTGATGCTGCGTTGCAGCGTATTGGGCTTACGGGACTGGCCAAG
AAATCGATGGACATCAACGAGCTCAAACGGTCGGAAGATCTCAGCAGAGGTCTATCCAGCTATCCAACACGGATG
TTTAATCTAATCAAGGAGAAATCCGAGGTGCCTTTAGGGCATGTTCATAAAATTCGGAAGAAAGTGGAATCACAG
CCCGAAGAAGCACTGAAGCTTCTTTTAGCCCTCTTTGAAAGTGAACCAGAGTCGAAGGCCATTGTAGTTGCAAGC
ACAACCAACGAAGTGGAAGAATTGGCCTGCTCTTGGAGAAAGTATTTTAGGGTGGTATGGATACACGGGAAGCTG
GATGCTGCAGAAAAGGTGTCTCGCACAAAGGAGTTTGTCACGGACGGTAACATGCGAGTTCTCATCGGAACGAAA
TTAGTGACTGAAGGAATTGACATTAAGCAATTGATGATGGTGATCATGCTTGATAATAGACTTAATATTATTGAG
CTCATTCAAGGCGTAGGGAGACTAAGAGATGGGGGCCTCTGTTATCTATTATCTAGAAAAAACAGTTGGGCGGCA
AGGAATCGTAAGGGTGAATTACCACCAATTAAGGAAGGCTGTATAACCGAACAGGTACGCGAGTTCTATGGACTT
GAATCAAAGAAAGGAAAAAAGGGCCAGCATGTTGGATGCTGTGGCTCCAGGACAGACCTGTCTGCTGACACAGTG
GAACTGATAGAAAGAATGGACAGATTGGCTGAAAAACAGGCGACAGCTTCCATGTCGATCGTTGCGTTACCGTCT
AACTTCCAGGAGAGCAATAGCAGTGACAGGTACAGAAAGTATTGCAGCAGTGATGAGGACAGCAACACGTGCATT
CATGGTAGTGCTAATGCCAGTACTAACGCCAGTACTAATGCGATTACCACTGCTAGTACCAACGTCAGGACTAAT
GCGACTACCAATGCTAGTACTAATGCGACTACCAATGCCAGTACCAATGCGACTACCAACTCCAGCACTAATGCT
ACTACCACTGCCAGCACCAACGTCAGGACTAGTGCTACTACCACTGCCAGCATCAACGTCAGGACTAGTGCTACT
ACCACTGAAAGTACCAACTCCAGCACTAATGCGACTACCACTGCCAGCATCAACGTCAGGACTAGTGCTACTACC
ACTAAAAGTATCAACTCCAGCACTAATGCTACTATCACTGAAAGTACCAACTCCAACACTAATGCTACTACCACT
GAAAGTACCAACTCCAAAACTAGTGCTACTACCACTGCTAGCACCAACTCCAACACTAGTGCTACTACCACCGAA
AGTACCAACTCCAAAACTAGTGCTACTACCACTGCTAGCACCAACTCCAACACTAGTGCTACTACCACCGAAAGT
ACCAACTCCAACACTAGTGCTACTACCACTGCTAGCACCAACTCCAGCACTAATGCTACTACCACTGAAAGTACC
AACGCTAGTGCCAAGGAGGACGCCAATAAAGATGGCAATGCTGAGGATAATAGATTCCATCCAGTCACCGACATT
AACAAAGAGTCGTATAAGCGGAAAGGGAGTCAAATGGTTTTGCTAGAGAGAAAGAAACTGAAAGCACAATTTCCC
AATACTTCCGAGAATATGAATGTCTTACAGTTTCTTGGATTTCGGTCTGACGAAATTAAACATCTTTTCCTCTAT
GGTATTGACATATACTTCTGCCCAGAGGGCGTATTCACACAATACGGATTATGCAAGGGCTGTCAAAAGATGTTC
GAGCTCTGTGTCTGTTGGGCTGGCCAGAAAGTATCGTATAGGAGGATGGCTTGGGAAGCACTAGCTGTGGAGAGA
ATGCTGCGAAATGACGAGGAATACAAAGAATACTTGGAAGACATCGAGCCATATCATGGGGACCCTGTAGGATAT
TTGAAATATTTTAGCGTAAAAAGGAGAGAGATCTACTCTCAGATACAGAGAAATTATGCTTGGTACCTGGCCATT
ACTAGAAGAAGAGAAACAATTAGTGTATTGGATTCGACAAGAGGCAAGCAAGGGAGCCAAGTTTTCCGCATGTCT
GGAAGGCAGATCAAAGAGTTGTATTATAAAGTATGGAGCAACTTGCGTGAATCGAAGACAGAGGTGCTGCAGTAC
TTTTTGAACTGGGACGAAAAAAAGTGCCAGGAAGAATGGGAGGCAAAAGACGATACGGTCTTTGTGGAAGCGCTC
GAGAAAGTTGGAGTTTTTCAGCGTTTGCGTTCCATGACGAGCGCTGGACTGCAGGGTCCGCAGTACGTCAAGCTG
CAGTTTAGCAGGCATCATCGACAGTTGAGGAGCAGATATGAATTAAGCCTAGGAATGCACTTGCGAGATCAGATT
GCGCTGGGAGTTACCCCATCTAAAGTGCCGCATTGGACGGCATTCCTCTCGATGCTGATAGGGCTGTTCTACAAT
AAAACATTTCGGCAGAAACTGGAGTATCTTTTGGAGCAGATTTCGGAGGTGTGGTTGTTACCACATTGGCTTGAT
TTGGCAAACGTTGAAGTTCTCGCTGCAGATAACACGAAGGTACCACTGTACATGCTGATGGTAGCGGTTCACAAA
GAGCTGGATAGCGATGATGTTCCAGACGGTAGATTTGATATAATATTACTATGTAGAGATTCGAGCAGAGAAGTT
GGAGAGTGA

>YBL111C 1.64 Hot
ATGAAAGTTTCCGATAGGCGTAAGTTTGAAAAAGCAAACTTTGACGAGTTTGAGTCGGCTCTAAATAACAAAAAC
GACTTGGTACATTGTCCCTCAATAACTTTATTTGAATCGATCCCCACGGAAGTGCGGTCATTCTACGAAGACGAA
AAGTCTGGTCTAATCAAAGTGGTAAAATTCAGAACTGGTGCAATGGATAGGAAAAGGTCTTTTGAAAAAAATTGTC
GTTTCCGTCATGGTCGGGAAAAATGTACAAAAGTTCCTGACGTTTGTTGAAGACGAACCAGATTTCCAGGGCGGA
CCAATCCCTTCAAAGTATCTTATTCCCAAGAAAATCAACTTGATGGTCTACACGTTGTTTCAAGTGCATACTTTG
AAATTCAATAGAAAGGATTACGATACCCTTTCTCTTTTTTACCTCAACAGAGGATACTATAATGAGTTGAGTTTC
CGTGTCCTGGAACGTTGTTACGAAATAGCGAGTGCCAGGCCGAACGACAGCTCTACGATGCGTACTTTCACTGAC
TTTGTTTCTGGCACACCTATTGTAAGGAGTCTTCAGAAAAGCACCATAAGGAAATATGGATACAATTTGGCACCC
TACATGTTCTTGTTACTACACGTAGATGAGCTATCGATTTTTTCTGCATACCAAGCAAGTTTACCTGGCGAAAAG
AAAGTCGACACAGAGCGGCTGAAGCGTGATCTATGCCCACGTAAACCCACTGAGATAAAGTACTTTTCACAGATA
TGTAACGATATGATGAACAAAAAGGACCGATTGGGTGATGTTTAGCTACAGCGCAACGTATACGTCGACGATAC
AACAAGAACGGTTCATCGGAGCCTCGACTAAAGACGCTTGACGGACTCACTTCCGAGCGCTGGATTCAATGGTTA
GGCCTTGAAAGCGACTACCATTGTTCATTCTCTAGTACTCGGAATGCGGAAGACGTAGTGGCAGGTGAGGCGGCG
AGTTCAGATCATGATCAAAAAATTTCAAGAGTAACGCGAAAAAGGCCCCGAGAGCCCAAGAGTACAAACGATATC
CTCGTCGCAGGCCGGAAACTCTTTGGCAGCTCCTTTGAATTCAGGGACTTGCATCAGTTGCGCTTATGTCATGAA

ATATACATGGCAGACACACCCTCTGTGGCAGTACAGGCCCCACCGGGCTATGGTAAGACGGAGTTATTTCATCTC
CCCTTGATAGCACTGGCATCTAAGGGCGACGTGAAATATGTGTCGTTTCTGTTTGTACCGTACACAGTGTTGCTT
GCTAATTGCATGATCAGGTTGGGCCGACGCGGTTGCTTGAATGTGGCCCCTGTAAGAAACTTTATTGAAGAAGGT
TGCGATGGCGTTACTGATTTATACGTGGGGATCTACGATGATCTTGCTAGCACTAATTTCACAGACAGGATAGCT
GCGTGGGAGAATATTGTTGAGTGCACCTTTAGGACCAACAACGTAAAATTGGGTTACCTCATTGTAGATGAGTTT
CACAACTTTGAAACGGAGGTCTACCGGCAGTCGCAATTTGGGGGCATAACTAACCTTGATTTTGACGCTTTTGAG
AAAGCAATCTTTTTGAGCGGCACAGCCCCTGAGGCTGTTGCTGATGCTGCGTTGCAGCGTATTGGGCTTACGGGA
CTGGCCAAGAAGTCGATGGACATCAACGAGCTCAAACGGTCGGAAGATCTCAGCAGAGGTCTATCCAGCTATCCA
ACACGGATGTTTAATCTAATCAAGGAGAAATCCGAGGTGCCTTTAGGGCATGTTCATAAAATTTGGAAGAAAGTG
GAATCACAGCCCGAAGAAGCACTGAAGCTTCTTTTAGCCCTCTTTGAAATTGAACCAGAGTCGAAGGCCATTGTA
GTTGCAAGCACAACCAACGAAGTGGAAGAATTGGCCTGCTCTTGGAGAAAGTATTTTAGGGTGGTATGGATACAC
GGGAAGCTTGGGTGCTGCAGAAAAGGTGTCTCGCACAAAGGAGTTTGTCACTGA

>YJR148W 1.56   Hot
ATGACCTTGGCACCCCTAGACGCCTCCAAAGTTAAGATAACTACCACACAACATGCATCTAAGCCAAAACCGAAC
AGTGAGTTAGTGTTTGGCAAGAGCTTCACGGACCACATGTTAACTGCGGAATGGACAGCTGAAAAAGGGTGGGGT
ACCCCAGAGATTAAACCTTATCAAAATCTGTCTTTAGACCCTTCCGCGGTGGTTTTCCATTATGCTTTTGAGCTA
TTCGAAGGGATGAAGGCTTACAGAACGGTGGACAACAAAATTACAATGTTTCGTCCAGATATGAATATGAAGCGC
ATGAATAAGTCTGCTCAGAGAATCTGTTTGCCAACGTTCGACCCAGAAGAGTTGATTACCCTAATTGGGAAACTG
ATCCAGCAAGATAAGTGCTTAGTTCCTGAAGGAAAAGGTTACTCTTTATATATCAGGCCTACATTAATCGGCACT
ACGGCCGGTTTAGGGGTTTCCACGCCTGATAGAGCCTTGCTATATGTCATTTGCTGCCCTGTGGGTCCTTATTAC
AAAACTGGATTTAAGGCGGTCAGACTGGAAGCCACTGATTATGCCACAAGAGCTTGGCCAGGAGGCTGTGGTGAC
AAGAAACTAGGTGCAAACTACGCCCCCTGCGTCCTGCCACAATTGCAAGCTGCTTCAAGGGGTTACCAACAAAAT
TTATGGCTATTTGGTCCAAATAACAACATTACTGAAGTCGGCACCATGAATGCTTTTTTCGTGTTTAAAGATAGT
AAAACGGGCAAGAAGGAACTAGTTACTGCTCCACTAGACGGTACCATTTTGGAAGGTGTTACTAGGGATTCCATT
TTAAATCTTGCTAAAGAAAGACTCGAACCAAGTGAATGGACCATTAGTGAACGCTACTTCACTATAGGCGAAGTT
ACTGAGAGATCCAAGAACGGTGAACTACTTGAAGCCTTTGGTTCTGGTACTGCTGCGATTGTTTCTCCCATTAAG
GAAATCGGCTGGAAAGGCGAACAAATTAATATTCCGTTGTTGCCCGGCGAACAAACCGGTCCATTGGCCAAAGAA
GTTGCACAATGGATTAATGGAATCCAATATGGCGAGACTGAGCATGGCAATTGGTCAAGGGTTGTTACTGATTTG
AACTGA

>YBL006C 1.64   Hot
ATGAGTGGAAGTAATATGGGATACTATGACGTACTCGCAGGGCTTTCAGCGCTGGAAAAGTCATCCCAGGTAGTG
TTCAGTGCGACTGAGCTTCAACAGCTTACGCAACAATCTCATGCTACCGACAAGGGCATAGAGGGCAGTGAGAAT
AGTAAAGCGAAGGTATCCAAGCCCAAAAGAGTAGCTGTACATGGTTACCTAGGCGGTAAAGTGTCGCTCGCCGAT
GCGGCACAGGTGGAGTATGAAGTAGGCCATTCACTGCTGGGCAGTTATGTGCCACGCCAGCAGTTGGAAGCCCTG
TCAAGTGTCGACTTTTCGCACCATTTCCACCGCACATTAGAATGCAAAGCTGCTCTAGAGACACACGATGTTTTT
CTTGCCGGCGCAGGACAATTGTCTCTACCCTTCCAATCACACATAGAGAGCCCCAGGAATAGCGAGGCTAAAAGG
AAAAGAAAAGTGATAATATGCAAACGGTGTCAATCACGATTTATAGGTTCCCATAGGCGGTCTCAACTTAGAGAA
CATGCCTGCGTAGATTAA

>YCR049C 2.04   Hot
ATGGCGCTGTCCAGGAGCGTGGGGCGAGGATCAAAACTCACGTCCCCAAAAAACGACACATACTTGCTAGCATCC
TTTCGGTGGAACCTCGACCGAGACTTGCTCTTCAGGTGTGAAAGGTACTTTTGCATGTGGGCGTCCACAGGGTAC
TCCTCCTCCTGCTCCTGCTTCCCTGCCACACGTTCCGCCTCAGTCGACTCCACTCCTTCAGTCGACTCCACTGGC
TCCACCAGCGACGTGGTAGACGACCGTGGCGAAACCTCCATGGACTCCTGTGGCAGGATCACGTTATCGTACGTG
ACCGAATGCCGTTTGTTGGCTTCTGCGGAATTGAGTCTGCGGATCTTAAGAAACTCTTCGTCTTGCAACAAATCC
TTAGTCTCCGTCATTCTTGCAATCTGTTTTGGCGCTCTTGCTGCAAGCCGTGCTGAACAACCACCTGCGTGA

>YAL040C 1.54   Hot
ATGGCCATATTGAAGGATACCATAATTAGATACGCTAATGCAAGGTATGCTACCGCTAGTGGCACTTCCACCGCC
ACTGCCGCCTCTGTCAGCGCTGCCTCATGTCCTAATTTGCCCTTGCTCTTGCAAAAGAGGCGGGCCATTGCTAGT
GCAAAGTCTAAAAACCCTAATCTCGTTAAAAGAGAATTGCAAGCACATCACTCAGCGATCAGCGAATACAATAAT
GATCAATTGGACCACTATTTCCGTCTTTCCCACACAGAAAGGCCGCTGTACAACCTGACTAACTTCAACTCTCAG
CCACAAGTTAATCCGAAGATGCGTTTCTTGATCTTTGACTTCATCATGTACTGTCACACAAGACTCAATCTATCG
ACCTCGACTTTGTTCCTTACTTTCACTATCTTGGACAAGTATTCCTCGCGGTTCATTATCAAGAGTTACAACTAC
CAGCTCTTGTCCTTGACCGCGCTTTGGATTTCGTCCAAATTTTGGGACTCCAAGAATAGAATGGCCACTTTGAAA
GTCTTGCAAAACTTGTGTTGCAATCAATATTCTATAAAGCAATTCACGACTATGGAAATGCATCTTTTCAAATCA
CTCGATTGGTCCATCTGTCAGTCGGCAACATTCGACTCCTACATCGACATCTTCTTGTTCCAATCTACGTCCCCG
TTATCGCCTGGCGTTGTCCTTTCTGCCCCTTTGGAAGCTTTCATTCAACAGAAACTGGCCTTATTAAATAACGCT
GCTGGTACTGCTATTAATAAATCGTCCTCTTCTCAAGGCCCCTCTTTGAACATCAACGAGATCAAATTGGGTGCC
ATTATGTTGTGCGAGTTAGCTTCCTTCAATCTCGAATTATCATTTAAATATGATCGTTCACTAATTGCGCTGGGT
GCAATTAACCTCATCAAATTATCTTTGAACTACTATAATTCAAACCTTTGGGAAAATATCAATCTGGCTTTGGAG
GAAAACTGCCAAGACCTAGATATTAAATTGTCAGAAATCTCTAATACTTTATTGGATATAGCAATGGACCAAAAT
TCTTTCCCCTCCAGTTTCAAATCAAAATATTTGAATAGCAATAAGACATCTTTAGCAAAATCTCTCTTAGACGCA
TTACAAAACTATTGTATTCAATTGAAACTGGAAGAATTCTACCGTTCACAAGAATTGGAAACCATGTACAATACT
ATCTTTGCTCAGTCCTTTGACAGCGATTCATTGACTTGTGTTTACTCAAATGCTACTACTCCAAAGAGCGCTACG

GTTTCATCTGCGGCCACAGACTATTTCTCGGATCACACTCATTTAAGAAGGTTGACCAAAGATAGCATTTCTCCA
CCATTTGCCTTCACTCCAACCTCATCTTCATCCTCTCCATCTCCATTCAATTCCCCTTACAAGACTTCAAGTTCA
ATGACGACCCCAGACTCTGCATCACACCATTCACATTCAGGTTCGTTCTCTTCTACCCAAAATTCTTTTAAAAGG
TCACTGAGCATCCCACAAAATTCAAGCATCTTTTGGCCAAGCCCACTAACTCCCACCACCCCATCTCTAATGTCA
AATAGAAAATTATTACAAAATTTATCTGTGCGTTCAAAAAGATTATTTCCTGTTAGACCCATGGCCACTGCTCAC
CCATGCTCTGCCCCCACCCAACTGAAAAAGAGATCAACTTCCTCTGTGGATTGTGATTTTAATGATAGTAGCAAC
CTCAAGAAAACTCGCTGA

>YLR074C 1.75   Hot
ATGGGTAGATATTCGGTGAAGAGATACAAGACTAAAAGAAGAACACGAGACTTGGACCTGATCTATAACGATTTG
TCCACCAAGGAATCAGTACAGAAGCTTCTGAACCAGCCATTGGATGAAACCAAACCGGGCCTTGGTCAGCACTAT
TGCATACACTGTGCCAAATACATGGAGACTGCTATAGCGTTAAAAACGCATTTAAAGGGCAAAGTGCACAAAAGA
AGAGTCAAGGAGTTGAGGGGTGTCCCTTACACACAAGAAGTCTCTGATGCTGCTGCGGGCTACAATTTGAACAAG
TTCTTGAACCGTGTACAGGAGATCACGCAATCGGTGGGTCCGGAGAAGGAAAGTAATGAGGCCTTGCTGAAGGAG
CATTTGGACAGTACCTTGGCAAACGTCAAAACAACAGAACCTACTCTACCGTGGGCGGCTGCTGATGCCGAGGCG
AATACCGCTGCCGTAACAGAGGCAGAGTCTACTGCCTCAGCCAGTACTTAA

>YNL290W 1.61   Hot
ATGTCGACAAGTACAGAGAAGAGGAGCAAAGAAAACCTTCCATGGGTTGAAAAATACAGACCCGAAACGTTGGAC
GAAGTGTACGGACAAAATGAGGTGATCACCACAGTTCGTAAATTTGTAGATGAAGGTAAATTGCCACATCTTCTA
TTCTATGGGCCTCCAGGTACCGGTAAAACTTCTACAATTGTTGCTTTGGCACGTGAGATATATGGTAAGAATTAC
TCGAACATGGTTTTGGAGCTGAATGCATCCGATGACAGAGGTATTGATGTGGTGAGGAATCAAATTAAAGACTTT
GCCTCTACAAGACAAATCTTCTCTAAAGGGTTCAAGTTAATTATACTGGATGAAGCAGATGCCATGACCAATGCC
GCACAAAATGCGTTGAGAAGAGTTATAGAACGGTACACCAAGAATACGCGGTTCTGTGTATTGGCCAATTATGCG
CATAAACTTACACCTGCGTTATTGAGTAGGTGCACGAGATTCAGATTTCAGCCCTTGCCCCAAGAGGCCATTGAG
CGTCGGATAGCCAACGTCTTGGTGCATGAGAAGCTGAAGTTGTCTCCTAATGCGGAGAAGGCCTTAATAGAACTT
TCTAATGGGGATATGAGACGTGTGTTAAACGTTTTGCAGTCTTGCAAAGCTACTTTAGACAATCCCGACGAGGAT
GAGATCAGTGACGACGTTATCTATGAATGCTGTGGGGCACCCAGACCAAGTGACTTAAAGGCAGTATTGAAGTCG
ATACTGGAGGACGATTGGGGTACCGCCCACTACACACTTAATAAGGTACGCAGTGCCAAGGGTCTCGCGTTGATC
GACCTAATCGAGGGCATAGTGAAGATACTGGAAGACTACGAACTTCAAAATGAGGAAACAAGAGTGCATTTGCTT
ACCAAACTGGCCGATATAGAGTACTCGATATCCAAGGGTGGCAACGACCAGATTCAGGGCAGCGCGGTCATTGGC
GCCATCAAGGCCAGCTTCGAGAACGAAACTGTTAAAGCCAACGTATAA

>YLR202C 1.65   Hot
ATGCCCAACTTTCATCTTATAAATATATGCGTTTTCTACCGTTATTATAATTTTTTTCTTATTTGCCATGACATA
TCCTTTCCTTTTCGGACTATATTAGGCGGAAGAAAAATAGCAATAACAATAGTAAGAAACAATAATACGGGGTTT
TTTTTTTTTTGGGCCTACATAAGGTACTTCACCCCAATTTGGAAAGAGATAACAGAGTCTTTACCACATTATAAA
TCATGCTTTGTCGCAATACTGCCAGAACGGGCTGTAAATTCTTTCGTTTATATCATTCGAACCCTATCGAACATG
TTAAACCAATTCATATTAAACCTTTAA

>YDR234W 1.82   Hot
ATGCTACGATCAACCACATTTACTCGTTCGTTCCACAGTTCTAGGGCCTGGTTGAAAGGTCAGAACCTAACTGAA
AAAATTGTTCAGTCGTATGCGGTCAACCTTCCCGAGGGTAAAGTTGTGCATTCTGGTGACTATGTATCGATCAAG
CCGGCACACTGTATGTCCCACGATAATTCGTGGCCTGTAGCTTTGAAATTCATGGGGCTTGGCGCTACCAAGATC
AAGAATCCTTCACAGATTGTGACCACTCTGGACCACGATATTCAGAACAAATCAGAGAAAAATTTGACCAAGTAC
AAGAACATCGAAAATTTTGCTAAGAAACACCATATAGACCACTACCCTGCCGGTAGAGGTATTGGTCATCAAATT
ATGATTGAGGAGGGCTATGCTTTCCCCTTGAACATGACTGTCGCATCTGACTCGCATTCAAACACCTACGGTGGT
CTGGGGTCGCTGGGCACTCCAATAGTGAGAACAGACGCTGCAGCCATATGGGCCACGGGACAGACGTGGTGGCAG
ATCCCACCAGTGGCTCAGGTTGAGTTGAAAGGTCAATTGCCTCAGGGTGTTTCCGGAAAAGATATCATTGTCGCA
TTATGTGGGCTTTTCAACAATGATCAAGTTCTAAATCACGCCATTGAATTCACGGGTGACTCTTTGAATGCATTG
CCTATCGATCACAGACTCACTATTGCTAACATGACCACCGAGTGGGGGGCTCTTTCTGGTTTGTTCCCCGTGGAC
AAAACTTTGATCGACTGGTATAAAAACCGTTTGCAAAAGCTGGGCACCAATAATCATCCAAGGATTAATCCAAAG
ACTATCCGCGCACTAGAAGAAAAGGCGAAGATTCCGAAAGCAGACAAGGATGCACATTATGCCAAGAAACTGATC
ATCGATCTAGCCACGCTAACTCACTACGTCTCAGGTCCAAATAGTGTTAAGGTCTCCAACACCGTGCAAGATCTA
TCTCAACAAGACATCAAGATAAATAAAGCTTATCTAGTGTCATGTACAAACTCCCGTCTATCTGATTTGCAATCT
GCAGCGGATGTGGTTTGTCCTACTGGAGACTTAAACAAAGTCAACAAGGTGGCTCCAGGTGTGGAGTTCTATGTC
GCTGCTGCCTCTTCAGAAATTGAGGCTGATGCCCGTAAATCAGGCGCTTGGGAAAAGCTGCTAAAGGCTGGCTGT
ATCCCACTGCCTTCTGGTTGTGGTCCATGCATCGGTCTAGGTGCGGGATTACTGGAACCAGGTGAAGTTGGTATC
AGTGCCACAAACAGAAACTTCAAAGGTAGAATGGGTTCCAAGGATGCATTGGCTTACTTAGCTTCCCCTGCTGTA
GTCGCCGCTTCTGCCGTGCTGGGTAAGATTAGTTCTCCTGCTGAGGTATTGTCCACAAGCGAAATTCCATTCAGC
GGCGTTAAGACTGAGATAATTGAGAATCCCGTGGTTGAAGAGGAAGTTAACGCTCAAACAGAGGCTCCAAAACAA
TCCGTTGAGATATTAGAAGGTTTCCCAAGAGAGTTTTCTGGTGAATTAGTTTTATGTGATGCCGATAACATCAAT
ACCGATGGTATATATCCTGGTAAGTACACTTATCAGGATGATGTGCCTAAAGAAAAGATGGCGCAAGTTTGTATG
GAAAATTATGATGCCGAGTTCAGAACCAAGGTTCATCCAGGTGATATAGTGGTCAGTGGGTTCAATTTCGGTACC
GGTTCCTCCAGGGAACAAGCGGCCACCGCCTTATTGGCTAAAGGTATCAACTTAGTTGTTTCAGGATCTTTTGGT
AATATTTTTTCAAGAAACTCCATTAACAATGCTCTTCTGACCTTGGAAATCCCAGCATTAATCAAAAAATTACGT

GAGAAATATCAAGGTGCTCCAAAAGAACTTACAAGAAGAACTGGTTGGTTTTTGAAATGGGATGTAGCTGATGCT
AAAGTGGTCGTTACCGAAGGTTCTTTGGACGGCCCTGTGATCTTGGAGCAAAAAGTGGGTGAGCTAGGTAAGAAC
CTACAAGAAATTATTGTAAAAGGAGGCTTGGAAGGTTGGGTCAAATCCCAACTATAA

>YIL023C 1.69   Hot
ATGAAGGCGTCGCACATTTGCTCTTATTTGCTAAGCATTGCACCCCTAGTGGTGTCCCATGGAGTACATCATAAT
CGAGATCATGGCCACGAGGCAAACCATGAGTCCAAGCAAAGTTTCTTGATTTTGAAGCAAGAATCCATTTTTTAC
TCTCTAGTTTGCTTTCTGCAGAACCACCTCTTTGTCCTGGGACCCCGTTACAATGCGATTGTGGCTATCCTAATC
ATACAACTTATGCCGTGTCTTTTCGTACTGTTTGTTCCTGGACTGCGCAAGAACGATCGTGCCAGCTTGACACTG
TCCTTACTAGTTTCCTTTTCTCTAGGAACACTCCTGGGTGATATTTTATTACACGTGATACCTGAAAGTCTCAGC
GGCGTAACTGATGTCACAATGGTTGGAGGAGCCATATTTCTGGGGTTTATAAGCTTCTTGACTCTGGATAAAACG
ATGCGTATTCTGTCAGGGACGTCCAACGATGACGGCAGCATACATTCTCATTCTCATAGTCATACTCCACAACAA
ACTGCAGAGAAGAAAGCGGGCTTTAACATGTCTGCGTATCTGAATGTCATATCCGGCATTGCTCATCACATCACG
GATGGCATAGCGCTGGCTACGTCATTCTATAGTTCCACACAAGTTGGCATAATGACCAGCATAGCTGTCACTTTC
CATGAGATCCCTCATGAGCTAGGTGACTTCGCCATTCTGCTTTCCAGTGGGTTTACGTTCCCACAAGCGATAAGA
GCCCAAGCGGTGACAGCTTTCGGCGCCGTCGTTGGAACGTCCATTGGCTGCTGGATGAACGAAATCGGGAACAAC
AGCCATAAAGCAACGTCTTCATCCGCGAACGCATCTGAACTTATGCTGCCGTTCACGGCGGGCGGCCTCATATAC
ATAGCCACTACTAGCGTTGTACCGCAGATCTTACATAGCTCCGCACCCGATAGCAAGCTTCGAGAGTTTAAGAAG
TGGGCCTTGCAGCTAGTCTTCATTTTTGTAGGATTTGCCGTTATGGCGCTCATGGATGAGCATTGA

>YLR279W 1.75   Hot
ATGCGATTCAGGTCTCAACGTGCAGTGTGCAGGAGCAGGAACTATTCAATGTATTGTTGTTCCAGGGTTTTTGTT
CGCAACCCACGTCTTGACCGACGGTACCCGCAGGTGAAAATTTTAGCAAAGTTACACTTTTTTTTTCATTTTTTT
TTTTCTTTTCTCCTCCATCTAATTTCACCTGCGGTTACCGGCGGAATAACTCGCGCACCATTTCTCTGTCTCGGG
CCTCGCGTTCCTCTTTTTCGACTCGAGCGGCCTCTTCACGCTGCTCGTACTTCGCGACGCTGCGCCGGCGCTGCG
AGCGTTTCTGTTGACGGTGCCACTGTAGAAGCGCCTCCTCTCTGGACGGCATCGTGTCGTACGACGCCGCAAGTT
CGCGCGCGAGCTTAA

>YJL119C 1.61   Hot
ATGAACCACTTGACAACGGTCTTGGCCGCACGTAAAACCTGTGTGGTGCGAGAAGCAACGAATCTGTATACAGTT
GATACAGAACGTCGGATCAACAAAATGGGGCGTGATGAAGAAAAAAAGTGGCGATCCCGTGGAGGACATTGGTGC
ATGGATGATTCAATCATACAATTTACGACATTTAACCAAAGTTGCACTCTGATGGGCTCAACGACTGCTACTCGT
GCCAATAATGAAACTGAAAAGCATGAAGCCATCACTGTTTTTTTCGTGGTCTTTCTATATATAAACAAAGCCTCT
GTAAAGTTTTTTTTTTTCGCATAA

>YDR048C 1.96   Hot
ATGTCTTCTCTACTTCAACTTCTCGCAGTGTGGAGTCAAAGCTCATCAATTTCAATGAATTCAGAAGTTGCTCAG
ACAAATCAAAAATATAAAGATCATTCTTTTTTAGTGAGCCAGTCATTTTACTCGCCTTTTGTGAATTCATGCTAT
TGGAAGCTTGTCAACAGGCGTGCAGATCTTTTGAAAATCCTTTTTCTTCGAAGGAAAGGTGATATGCATTCTCAT
TTATTTTCATATACCTTCATCTTTTATTTTCTTCTATTTAAGATTTTGTCACTACATCCATTCAGAGAACCTGCA
AAAATAAATATCTGA

>YEL038W 1.58   Hot
ATGGGAGACAATTACTCGACTTACTTGCTTGATATTGAAGGGACAGTGTGCCCGATCTCGTTCGTGAAGGAGACT
TTATTCCCTTATTTCACGAATAAAGTGCCGCAATTGGTTCAACAGGATACAAGGGACTCCCCGGTTTCCAACATT
CTGTCGCAGTTTCACATCGACAATAAGGAGCAGTTGCAGGCGCATATTTTGGAGTTGGTGGCCAAGGATGTGAAG
GATCCAATCTTGAAACAGTTGCAAGGCTACGTCTGGGCTCACGGATACGAATCTGGGCAGATTAAGGCGCCCGTT
TATGCAGATGCTATTGACTTTATCAAGAGGAAGAAGCGTGTGTTTATCTACTCTAGTGGATCTGTAAAGGCACAG
AAATTGCTGTTCGGGTATGTTCAGGATCCCAACGCGCCTGCGCATGATTCGTTGGATTTGAATTCTTATATAGAT
GGTTATTTCGACATCAACACTTCTGGAAAGAAAACCGAAACACAGTCTTACGCCAACATCTTGCGGGATATCGGT
GCGAAGGCTAGCGAGGTACTGTTCTTGAGCGATAATCCATTGGAATTGGACGCAGCGGCCGGCGTTGGAATAGCT
ACTGGGTTAGCTTCCCGACCTGGTAATGCACCTGTCCCCGATGGGCAAAAGTACCAGGTATACAAGAATTTTGAA
ACTCTTTAA

>YPR151C 1.59   Hot
ATGATTTTATTAAAGAGGACAAAAATTAGGGGCGTCAGCGTCTCTTTTGTAAGCTTGCAAAGAAGGACTCACAGC
AGACTGGTGAACCCCATAAGGCAGCAACATCAACAAATTACCAAGCAAAGGAGTTCAAAGATTTTAAAGAATGCG
CATTTCTATGACTTTAGATCGCTGCCCAAAGTACCTACTACACAGTATTTGGAAGCTAGGGAGCTCACCCGCGAT
ATTCTGTATAGCGGATACAGGCCAGTAATGTACCCAGTGAAGGAAAACCCGCTATTCAGAGATAAGAAGAGAAAG
TCCTTGCAAACGTTATTAACAATGAACGAGAAAACAAACGCAGAGGCGAAGACGATCGATGAGAAAAAGCACAAG
AACATATTGTTTGGTGAACGTGGCACTGGTGGTATAATGTCAGGTGGCGTGAACGGTACATGGAAGTACAATCCC
ACCGTACCCAATGAGCTGTTGCCATTCAATTGGTGGTCTACGTCCAGTATGGGTATGGAATATTTTCCTGAATGG
AAAAACGTGCCCCCATATATGATGAGAAAGTTGAAGCCGTTTGACAAGGCACTGCAAATGCGGTTGACACACAAA
AGCAAAAAAAAAGATGAAATAA

>YOR327C 1.56   Hot

ATGTCGTCATCAGTGCCATACGATCCATATGTGCCTCCAGAGGAGAGTAACTCAGGCGCAAACCCAAATTCCCAA
AACAAGACTGCTGCTTTGAGACAAGAGATTGATGACACGGTGGGAATAATGAGAGATAATATCAACAAGGTTGCT
GAACGTGGTGAAAGGCTAACATCCATTGAGGACAAAGCTGATAACTTGGCTATCTCCGCACAAGGATTCAAGAGA
GGCGCCAACAGGGTCAGAAAGCAAATGTGGTGGAAAGATCTAAAAATGAGAATGTGTTTATTCTTAGTTGTTATT
ATTTTACTAGTGGTAATTATCGTTCCTATCGTCGTCCATTTCAGCTAA

>YER035W 2.04 Hot
ATGGGTTCCGAGACAAAACATTCTGCAAAAGTCAAGATTGTCACAAGGGAAAGTCCTCCTTCCGCCAAGGAGCAC
ATGCGCCCCACTAAAACTCAAATATTAGTTCCACCGACGCAGAGTTTGCCCAATGGCAAGAAACCAAACTTCGGT
AAGTCTACAAAACAGCGGCGAGAACCTAGGGAACGCACCTCGAAGACGGGACACGAGGACGATAAGGCAACGATG
GTCACTGTTAACATAGATGCCTTCCTACATGATAAGGCCCCTAAAAAAAAATCGTGCAAATACAAGAAGAAGAAA
ACGAGACAGTACCAGGATAGGGCGGCGGCGTCGATCGATTCGAAACCGCACGTAGCTGGTCATACGGCCTTTGCC
GGTGCTTCGTTCACAACAGATATCCCACATGAGGCAGCGCTACCCAAACCTAGTTTTGTTTGA

>YNL322C 1.89 Hot
ATGATGCGTCGCACGCTATTACATTCATTCGCTACGCTGCTACTTTCTTTGTCGTTGTGGTCAGCTGCGGTCATG
GCAGCTGTGACAACTCAGGTTACAGTGGTAACAAATGTCGCAGGGGCCCTGGTTACGGAGACCACAATATGGGAC
CCTGCCACCGCTGCTGCTGCTGCTACAACTACCGCTCAAACAGGTTTCTTCACTACGGTATTCACTACCACTAAC
GATGTCGGAACCACCGTCACTCTTACTCAGACAGTCAACAGAGCCACTATGCTACCAACCACGACGACTTCTACC
TCATCTACTGGTAAGACAACCACCACTGTTCCTACCGCAACTTCATCGTTGTCTTCGGGACTGTATTTATCTACA
GTTACCACGACAAACGATTTGGGTACCACAGTTACATTGACTCAAACGTTCACACATTCTAGCACCAGTGCTACT
TCATCCGCCTCCTCGTCTGTGTCCTCGTCTGTATCTTCGTCTGGTTCATCCTCCAGTGTAAAGACGACCACATCG
ACAGGGAGCGCAGTAGCTGAAACAGGCACCAGGCCAGACCCCTCCACAGACTTCACAGAACCTCCTGTGTCTGCT
GTCACTAGTCTATCTATTGACTCATACATTACCATCACTGAAGGTACAACCTCCACTTACACAACCACACGTGCG
CCAACGTCCATGTGGGTCACTGTTGTTAGACAGGGCAACACTATCACTGTGCAAACTACTTTTGTCCAGCGTTTC
TCCTCCCAGTACGTAACAGTCGCTTCTCCCTCCGTGGGGTCTATTGGGATGGGTACTTTAACCGGTACTGTAGGC
GTTATTAAATCTGCAATAAAGAAAACAGTTTCGCATAATGAGGCCCAGCATCTAGGTATGAGTTCGTTTACTTCA
ATTTTGGGTGGGCTATTAACGGTTTTAATTTGGTTCTTATAA

>YLR231C 2.05 Hot
ATGGAGAAAGCTTTGGAATTAGACGGAGAATATCCGGAATCTCTGAGGGATGAATTCAACATCCCTACATTTAAA
TCCATGGGACTATCGTCCGACGATAAGCCTGTGACGTACTTATGCGGGAATTCTTTAGGTTTGATGCCGAAGTCA
ACTAGGAATTCAATTAATGCTGAGCTAGATGCGTGGAGCGATTGTGCTGTGGAATCGCATTTCAAACATCCTGAA
GAAGCCAGAGGAAAGGTGCCTTGGGTCAGCATTGACTTACCTATTCTTCCACTACTAGCCCCCATCGTGGGTGCT
CAAGAAAATGAAGTTGCAGTAATGAATAGTCTCACTGCAAATTTGAATTCATTGTTAATTACGTTTTATAAACCT
ACTGAGAAAAGATTCAAGATCCTTTTTGAAAAGGGCTCCTTTCCATCAGACTATTATGCTTTCTACAACCAGTGC
AAAATTCATGGAATTTCGGAACCTGAGAATGTTTTTATTCAGATCGAGCCACGCGAGGGAGAGACTTATATCAGA
ACTCAAGATATCCTGGATACCATAGAGGTAAATCAAGATGAATTGGCGCTGGTCTGTTTGTCAGGTGTTCAGTAT
TACACGGGGCAATATTTCGATATTGGCCGAATCACCTCATTTGCCCACCAATTCCCCGACATATTGGTTGGATGG
GATTTAGCACACGCTGTAGGGAACGTCCCATTGCAACTTCATGATTGGGGTGTTGACTTTGCCTGTTGGTGTTCT
TACAAGTACTTGAATGCCGGGCCTGGTGGAATTGGTGGCTTATTTGTCCACTCGAAGCATACTAAACCAGACCCT
GCTAAGGAAAGTTTGCCAAGATTAGCTGGTTGGTGGGGCAATGATCCTGCTAAGCGATTTCAAATGCTGGAAGTA
TTCGAGCCGATTCCAGGAGCATTGGGATTCAGGCAATCTAATCCAAGCGTTATTGACACAGTGGCATTGAGAAGT
TCATTGGAATTATTTGCGAAGTTTAATGGTATTAATGAAGTTCGTAAAAGATCGTTGTTGCTGACGAACTATATG
ACGGAACTGTTGGAAGCCTCCAAGTATTACAAACACCCTTTAAGGATCGAGAAATTACCATGTTTTTTTACAATA
TTAACTCCAACAAGCACAGATGAAGAACATGGCGCCCAATTGTCACTTTACTTCGATTCTGACACTGGAAAAGAG
GATATTATGCCCAAAGTTTTCCAATATTTGCATGACCATGGTGTCATTGGCGATGCAAGAAGACCAAACGTAATT
AGATTGGCTCCTGCTCCCTTATATAATACATTTTCAGATGTATACATTGCGGTGAATGCACTAAATGAGGCGATG
GATAAGTTGTAG

>YGR180C 1.52 Hot
ATGGAAGCACATAACCAATTTTTGAAGACTTTCCAAAAAGAACGTCATGATATGAAAGAAGCTGAAAAGGATGAA
ATCCTTTTGATGGAAAACAGCCGTAGATTCGTGATGTTCCCTATCAAATACCACGAAATCTGGGCTGCTTACAAG
AAGGTTGAAGCCTCTTTCTGGACTGCGGAAGAAATCGAATTGGCTAAGGACACTGAAGATTTCCAAAAATTGACT
GATGACCAGAAGACCTACATCGGTAACTTGTTAGCCTTGTCCATTTCTTCTGACAACCTTGTCAACAAGTACTTG
ATCGAAAACTTTTCTGCCCAATTGCAAAACCCTGAAGGTAAGAGTTTCTACGGGTTCCAGATTATGATGGAAAAC
ATCTACTCTGAAGTTTACTCCATGATGGTTGATGCCTTCTTCAAGGACCCTAAGAACATCCCTCTATTCAAGGAA
ATTGCCAATTTGCCTGAAGTCAAGCATAAGGCTGCTTTCATCGAGAGATGGATTTCCAACGATGACAGCTTGTAT
GCTGAAAGACTAGTAGCATTTGCTGCAAAGGAAGGTATTTTCCAAGCTGGTAACTATGCTTCTATGTTCTGGTTG
ACTGACAAAAAGATTATGCCAGGTTTAGCAATGGCCAACAGAAACATCTGTAGAGACAGAGGTGCCTACACTGAC
TTTTCATGCTTGCTATTTGCCCATTTGAGAACCAAGCCAAACCCCAAGATCATTGAAAAAATCATTACCGAAGCC
GTGGAAATTGAAAAGGAATACTACTCAAACTCTTTGCCAGTAGAAAAATTTGGTATGGATTTGAAGAGCATTCAC
ACCTACATAGAATTTGTCGCTGACGGTCTATTACAAGGTTTCGGTAACGAAAAATACTACAACGCCGTCAACCCA
TTCGAATTCATGGAGGATGTCGCTACCGCTGGTAAGACCACCTTCTTTGAAAAGAAGGTTTCCGACTACCAAAAG
GCCAGTGACATGTCTAAGTCCGCTACCCCATCCAAGGAAATTAACTTTGATGATGACTTCTAA

>YNL104C 1.87 Hot

ATGGTTAAAGAGAGTATTATTGCTCTTGCTGAGCATGCGGCCTCCAGAGCCTCAAGAGTTATTCCTCCAGTGAAG
CTAGCCTATAAAAATATGCTTAAGGACCCTTCCTCCAAGTACAAGCCATTTAACGCTCCAAAGCTATCTAATAGA
AAGTGGCCGGATAACCGGATCACGAGGGCTCCTCGTTGGTTATCAACAGATTTGAGAGATGGTAACCAATCTCTG
CCGGATCCCATGTCAGTGGAACAAAAGAAAGAATACTTTCACAAGCTGGTCAATATTGGGTTCAAAGAAATCGAG
GTTTCCTTCCCCTCTGCATCTCAAACAGATTTCGACTTCACTAGATATGCTGTAGAAAACGCCCCAGACGATGTT
AGTATTCAATGTCTTGTCCAATCTAGAGAACACTTGATTAAGAGAACGGTGGAAGCATTAACAGGTGCTAAAAAG
GCTACTATACATACTTACTTGGCAACAAGTGATATGTTCCGTGAAATTGTTTTTAATATGTCTAGAGAGGAAGCT
ATTTCCAAGGCAGTAGAGGCCACCAAACTAGTTAGGAAACTAACTAAGGATGACCCTTCCCAACAAGCCACTCGT
TGGTCCTATGAGTTTTCCCCCGAATGTTTCAGTGATACTCCAGGTGAATTTGCTGTAGAAATTTGCGAAGCTGTT
AAGAAGGCTTGGGAACCTACCGAGGAAAATCCAATCATTTTCAACTTACCTGCTACCGTAGAAGTTGCCTCTCCA
AATGTTTATGCTGATCAGATTGAATACTTCGCTACCCATATTACTGAGCGTGAGAAGGTTTGCATCTCTACACAT
TGTCACAATGACCGTGGTTGCGGTGTCGCCGCCACAGAGTTAGGTATGCTTGCAGGTGCCGACCGTGTAGAAGGA
TGTCTCTTTGGTAATGGTGAACGTACAGGTAATGTGGACTTGGTTACTGTTGCTATGAATATGTATACCCAAGGT
GTTTCTCCTAATTTGGATTTCTCTGACTTGACCTCTGTCCTAGATGTGGTTGAGCGTTGTAATAAGATCCCAGTA
TCGCAAAGAGCACCATACGGCGGTGACTTGGTCGTTTGTGCCTTTTCCGGTTCTCACCAAGACGCCATTAAGAAG
GGTTTCAACTTACAAAACAAGAAGCGTGCTCAAGGTGAAACTCAATGGAGAATCCCATACTTGCCATTGGATCCA
AAGGACATTGGCCGTGATTACGAAGCTGTCATCAGAGTCAACTCTCAGTCTGGTAAAGGTGGTGCCGCTTGGGTT
ATTTTGAGATCTTTGGGTTTGGATCTACCAAGAAACATGCAAATCGAATTTTCTAGCGCCGTTCAAGACCATGCT
GACTCCTTGGGTAGAGAACTAAAATCAGATGAGATTTCCAAGTTATTCAAAGAGGCTTACAACTACAATGACGAA
CAGTACCAAGCTATTAGTTTAGTCAATTATAATGTTGAAAAATTCGGCACTGAACGTAGAGTGTTCACTGGTCAA
GTCAAAGTAGGCGACCAGATCGTCGATATTGAAGGTACAGGTAATGGTCCAATCTCTTCTTTGGTCGACGCCCTA
TCAAACTTGTTGAACGTGAGATTTGCCGTAGCAAACTACACAGAGCATTCTCTAGGTTCTGGTTCTTCTACGCAA
GCTGCTTCTTACATCCATCTATCGTATAGGCGTAATGCCGACAACGAAAAGGCCTACAAATGGGGTGTAGGTGTC
TCCGAAGATGTCGGTGATTCTTCAGTGAGAGCCATCTTTGCCACCATTAACAATATTATCCATTCTGGTGATGTG
TCCATTCCATCTTTGGCCGAGGTCGAAGGTAAGAATGCTGCGGCATCTGGCTCTGCATAA

>YOL039W 1.52 Hot
ATGAAGTACTTAGCTGCTTACCTATTATTGAACGCTGCTGGTAACACCCCAGATGCCACCAAGATTAAGGCTATT
TTGGAATCCGTCGGTATCGAAATCGAAGACGAAAAGGTTTCCTCCGTTTTGTCCGCTTTGGAAGGTAAGTCTGTT
GACGAATTAATTACTGAAGGTAACGAAAAGTTGGCTGCTGTTCCAGCTGCTGGTCCAGCTTCTGCTGGCGGTGCT
GCTGCTGCCTCTGGTGATGCTGCTGCTGAAGAAGAAAAGGAAGAAGAAGCCGCTGAAGAATCTGACGACGACATG
GGTTTCGGTTTATTCGACTAA

>YOR271C 1.59 Hot
ATGGCATCATCAGTCCCAGGGCCCATCGATTTGCCCGAATCCCGCTATGACTTATCCACGTATTGGGGTAGAATC
CGCCATTGCGCAGAGATTTCCGACCCCACTATGTTGTTAACTACTGAGAAGGACCTGGCGCATGCTAGAGAAATC
ATCAGTGCGTATCGTCACGGTGAACTAAAAGAGACTACTCCGGAGTTCTGGCGAGCCAAGAAACAACTGGACTCC
ACCGTGCATCCGGACACAGGGAAGACAGTTCTTTTGCCCTTCCGTATGTCTTCCAACGTGCTATCAAACCTAGTC
GTTACTGTAGGTATGCTGACGCCTGGTTTGGGTACGGCAGGGACTGTATTTTGGCAATGGGCAAATCAATCGCTA
AACGTCGCCGTTAATTCCGCTAACGCAAACAAATCTCATCCTATGTCCACGTCCCAATTGCTCACCAATTACGCT
GCCGCTGTAACTGCGTCGTGTGGTGTTGCGTTAGGTCTAAATAACCTAGTTCCCAGGTTGAAAAACATTTCTCCA
CATTCAAAGTTGATCTTGGGCCGTTTGGTGCCATTTGCCGCCGTGGTCAGTGCCGGTATTGTCAACGTCTTCCTA
ATGAGAGGCAACGAAATTAGAAAGGGTATTTCTGTGTTTGACTCTAACGGTGACGAAGTCGGAAAGTCCAAGAAG
GCTGCCTTCATGGCAGTTGGTGAAACTGCATTAAGTAGAGTGATTAACGCCACTCCAACTATGGTCATCCCACCT
TTGATTTTGGTTAGACTACAACGCGGTGTCCTCAAAGGTAAGTCTCTCGGTGTGCAAACTTTGGCCAATTTGGGG
TTGATCTCAGTCACAATGTTCTCAGCTTTGCCATTTGCACTCGGTATCTTCCCTCAAAGACAAGCAATCCATTTA
AATAAACTAGAACCTGAACTACATGGCAAGAAGGATAAGGATGGGAAGCCAATTGAAAAGGTCTATTTTAACAGA
GGTATTTAG

>YDL155W 1.7Hot
ATGCATCATAACTCACAGTCTTTGAGCTCTGGACACATCAGGAGCCCCGAGGATGAAAATGTGGCACCTATAGGT
AATCTTAAACACAGGACTGGATCCCTCAGTCATATTTCATCTGCGCACCCGAGGGTCGCACTTAGCGACGTTACC
AATATAGTTGCGACAAACTCTAGCAACAACAGCATAAGTAAGCCAAAAGTCGCCCCAATTAAAGAAAGATTGGAT
TCAGCTGCGATAATTGAGGAAGAAAGGCTGGATGCGAATAGTGTTGCACAGAGAAAAGAAGCTGATCATAACGAT
TTGTTAACGGACAGGGAACAAGAGGAACCCGTTGAAGACGACGGAGAAAGCGAAGAGGATGAAGAAGAAGACCAG
GAGCCTCTACTGTTGCAACATTATGCTAGTGATACATTGGTCTGGGAGCATGCATTTAGAACTTACTATAGAACT
ACATTAGATCCCAATGATGATGACGTGTACGATGTGGTCATGGTTGCCGAATTATCTAATGAGATATTCGAGTAT
ATGAGGAAATTGGAAGACCTGTATAAACCCAACCCGTACTACATGGATAAACAACCAGAGTTAAGATGGTCGTTT
CGAAGCACACTGATTGATTGGATCGTCCAAGTACATGAAAAATTTCAACTTTTACCTGAAACTCTATATCTCTGC
ATTAATATAATAGACAGATATCTGTGCAAAGAAGTTGTTCCTGTAAATAAGTTCCAACTTGTGGGTGCAGCCTCA
CTCTTCATTGCTGCTAAATATGAGGAAATCAACTGTCCTACAATCAAGGATTTCGTATACATGTCAGAAAACTGC
TACTCAAGGAACGACCTGCTGGACGCAGAAAGAACTATTTTGAACGGCTTAGAATTTGAATTGGGTTGGCCTGGT
CCGATGTCATTTTTACGAAGAATCAGTAAGGCAGACGATTACGAGCATGATACGAGAACACTGGCCAAATATCTA
TTGGAATCCACAATAATGGACCATCGACTGGTTTCCGCTCAACCTAGTTGGTTAGCTGCCGGTGCATACTTTCTA
AGTAAGATTATTCTGGGCCAAAATCAGTGGTCTCTGGCGCACGTCTACTATTCCAATTATACACAAGAACAAATT
CTTCCGTTGGCCACCATTATTTTAGAAAATTGCAGATATGCCTCTAAACGTCATAACGCCATATGGAGAAAATAT

TCTTCACGTCGTTATTTGCATTCTTCACAGATCGTAGCGAAGTGGATAGCATTAGCTGAACACAGAGTAGAAAGA
TCTAACTAA

>YHR210C 1.63   Hot
ATGTCAAATAATAAGGCTGGCGGTGAATATGAAGTCATCACTATTGGTGATGCAAAAAAGCTTCAAGCAACAATT
TCAGAGCTTGGCGCTACTTTGCTTGACTTGAAAGTGAACAACGAATCGATTGTTCTAGGATACCCTGATATTCAT
GGCTATATATCAGATGGCTACAACTACATTGGTGCTACTGTTGGCCGTTATGCAAATCGTATTTACAAGGGCATG
TTCAGTATGGAAGATGGACCTCATCAGTTGACAGTGAACAATTGTGGAAACACCAACCACAGTAGCATCAGCTCC
TTCCATCTCAAGAAGTATAAGGCATCCAAAGTCCAAAATCCTCTTGATGACCTTTACATTGTTGAATTTACGTTA
TTGGATGACCGCACACTGCCCAATGAATTTCCGGGAGATCTAGCTGTAAACTTAAAGTACACATTGAACGTTGCT
GACATGACCTTGGACTTGGAATACGAGGCAAAACTGGTCAGCGGTGAGGCTACTCCCATCAACATGACGAATCAC
ACGTACTTCAATTTAAACAAAACTATGGATAAAAAATCCATTAGCGGTACGGAGGTTAGGCTCTGCTCAGATAAA
TCATTGGAAGTAAGCGAAGGAGCACTAATTCCAACCGGTAAAATCGTTCAAAGAAAAATTGCTACTTTTGACTCC
TCCAAACCTACCATTCTGCAGGATGATGGCCCGATTTATGACTACGCTTTCATTGTTGATGAAAATAAGAATTTG
AAAACGACAGACTCAGTAAGTGTAAACAAGCTCGTCCCTGCCTTCAAGGCTTATCATCCGGCATCACGACTTTCA
TTGGAGGTTTCAACAACAGAACCAACTGTCCTATTTTACACAGGTGACAACTTGTGTGATGGATTTACGCCGAGA
TCTGGCTTTGCCGTTGAACAAGGCAGATATGTTGACGCAATCAACCGTGATGGATGGAGAGACTGCGTTCTTTTG
AGACGTGGTGAGGTATATACTTCTAAGACTCGATATCGTTTTGCGGTGTAG

>YGL198W 2.26   Hot
ATGTCATACGGAAGAGAAGACACTACGATTGAGCCCGACTTCATAGAACCAGATGCACCTTTGGCTGCTTCCGGG
GGTGTTGCTGACAACATAGGCGGAACTATGCAGAATTCAGGCAGCAGAGGGACGCTCGACGAGACTGTGCTGCAA
ACACTAAAGCGAGATGTGGTGGAGATTAATTCCAGACTGAAACAAGTGGTATACCCGCATTTCCCCTCATTCTTT
AGCCCCTCTGATGACGGGATAGGGGCGGCTGATAACGACATTTCAGCCAATTGCGACCTGTGGGCGCCCCTTGCG
TTTATCATATTGTATTCTCTATTTGTATCGCATGCGCGGTCGCTGTTCTCGAGCCTATTTGTGTCTAGTTGGTTC
ATTTTGCTGGTGATGGCATTGCATCTGAGACTCACCAAGCCACACCAGAGGGTGTCGCTGATTTCGTACATCTCC
ATTTCCGGGTATTGCTTATTCCCACAAGTGCTGAATGCCTTAGTCTCGCAGATACTACTTCCATTGGCCTACCAT
ATTGGAAAGCAAAATCGCTGGATTGTGAGGGTCCTGTCGCTCGTGAAACTGGTGGTCATGGCGCTGTGCCTGATG
TGGTCTGTGGCCGCCGTTTCGTGGGTTACCAAGAGCAAGACCATTATCGAGATATACCCTCTGGCACTCTGTCTT
TTTGGCATGGCCTGGTTGTCAACTATTTTATAA

>YOR376W 1.67   Hot
ATGAAAAACAGAAAATTTTCCAATCTTCTCTTACTTAGATTACGTATACTTTGCTTTAACAAGAAGCCGGCCTTT
GCTGCTACTTCTTACGCTTTCTTCTTCCGCAATTTTTCAGTGCTGATTTTCATTATGGTACCTGATGAAAAAGAA
AACGGCGCAGCAGCTGATAACAGCTTCTCTCTGCTGATTGGACGTGGGGTCGTACTATTTCTTTTTTATTGCCCC
ACCGCACTGAAAATGCATGGGCCGGTTCCGGCGCATTGGTTCTGCGATAAGAATATCGAGGCCATCCAATCAGAT
GGTCAGATAAGGTTATTAAGATCAGGCCCGTTTCCATGGAGTCATGGAACCTGCATAAGGGGAGCCTGA

>YNL072W 1.79   Hot
ATGGTACCCCCCACGGTAGAAGCATCCTTGGAGTCTCCTTACACTAAGTCGTACTTTTCACCTGTCCCAAGCGCG
CTCTTGGAACAGAATGACTCTCCAATAATAATGGGTATCGATGAAGCTGGCAGAGGGCCCGTATTAGGGCCAATG
GTCTACGCAGTAGCCTATTCTACACAGAAATATCAGGACGAAACTATAATCCCTAACTACGAGTTTGACGACTCT
AAAAAACTTACAGATCCCATCAGAAGAATGCTGTTTTCCAAGATATACCAGGACAACGAAGAACTAACCCAGATC
GGCTACGCAACTACGTGCATCACTCCGTTGGATATCTCTAGAGGAATGAGCAAGTTCCCACCGACGAGAAACTAT
AACTTGAATGAGCAGGCGCACGATGTCACAATGGCACTCATTGATGGCGTGATAAAGCAGAACGTGAAGCTCAGT
CACGTGTATGTGGATACTGTTGGACCACCAGCGTCCTATCAGAAGAAACTGGAGCAACGATTTCCTGGTGTCAAG
TTTACGGTCGCCAAGAAGGCAGACTCGCTCTACTGCATGGTCAGTGTGGCGAGTGTCGTTGCAAAAGTGACCAGA
GATATACTAGTCGAATCCTTGAAGAGGGATCCCGATGAGATCCTGGGTTCTGGATACCCCTCCGACCCGAAGACA
GTCGCATGGCTGAAGCGCAACCAGACAAGTCTTATGGGCTGGCCCGCCAACATGGTAAGATTCTCATGGCAGACA
TGCCAGACTCTGTTAGATGACGCCAGTAAGAATAGTATCCCTATCAAGTGGGAAGAACAATATATGGACAGCAGG
AAGAACGCCGCACAAAAAACCAAGCAATTGCAGCTCCAGATGGTCGCTAAGCCTGTTAGAAGGAAGAGGCTGAGA
ACCCTAGATAATTGGTACCGGTGA

>YIL040W 1.67   Hot
ATGGACGCTACTCAACCGCAATACGAACTATCCGTAGTAACACAATGCCTAAAGTCTGCCATTGACGTCATCCAA
TGGTTGATTCCTACTATTACTAAGTTCAGCCAATCACACCCGTTAGTTTTCCAACTATTGTTTATTTTCTTCACA
TTTTATGTCTTTTACAAGTTGTTGATGAACTTCATTACTCTGGTCAAAAGATTCCTGTATTTGACGTTGGTAGTA
ACATGTATCGGTATTTATATGCGTGGTTCGCAGCAGTTTTTGACTGTGGACCTGTTGAATTTCTACAATTTCGTC
ATGTCAAATAGATACTACGCATTTAAAATTTATACTCTGTTTATTAATGCCCTGGAAAGAGAAATCAACACTGTT
TATCATTTGGCGCAGATGAAAATGGAACAGTTGCTTAAATAG

>YAL034C 1.52   Hot
ATGGGATTATATTCTCCTGAATCTGAAAAGTCTCAATTAAATATGAATTACATTGGTAAGGATGATTCGCAGTCC
ATTTTCAGACGTCTGAATCAGAATTTGAAAGCAAGTAACAACAATAACGATAGTAATAAAAACGGTTTAAACATG
AGTGATTATAGCAATAATTCACCCTATGGGCGCTCGTACGACGTAAGAATTAACCAGAACTCACAAAATAATGGC
AATGGATGCTTTTCTGGCAGCATTGACTCCTTGGTTGATGAACATATAATACCATCGCCACCTTTGTCGCCCAAG
CTGGAGTCGAAAATTAGCCACAATGGCTCACCCCGCATGGCCTCTTCAGTGCTAGTGGGATCTACGCCTAAAGGC

GCTGTAGAGAATGTGCTGTTCGTGAAGCCTGTATGGCCCAATGGGTTATCAAGAAAAAGGTACCGCTACGCCACC
TACGGGTTTCTGTCTCAATACAAAATTTTCAGCAATTTGGCCCAACCATATTCTAAGAACATTATCAACCGGTAC
AACAATCTGGCCTATAATGCTAGACATAAATATTCCAAATACAATGATGATATGACTCCTCCTCCTCTGCCTTCC
TCTTCTTCTAGATTACCTTCCCCGTTAGCATCTCCGAATTTGAATAGACAAGCAAGATATAATATGAGGAAACAG
GCTCTCTACAATAACAATCTAGGAAAGTTTGAATCCGACACTGAATGGATACCACGGAAACGCAAGGTATACTCA
CCACAAAGAAGAACGATGACTACCAGTCCACATCGCGCCAAGAAGTTCTCACCCTCTGCATCCACTCCTCACACT
AACATTGCATCCATTGAGGCGATTCATGATGCTCCTCAATATATACCAAACGTCTCATGGAAAAAATTACCAGAT
TACTCTCCGCCCTTATCTACGCTTCCTACAGACAGTAACAAGTCACTCAAGATCGAGTGGAAGGGGTCTCCAATG
GACCTGTCCACAGACCCGCTGAGGAACGAGCTACACCCTGCTGAACTAGTTCTCGCTCAAACTCTAAGGTTACCT
TGTGATTTGTATCTGGATTCTAAGAGAAGGTTATTTTTGGAAAAAGTTTATAGACTAAAGAAAGGGTTGCCGTTT
AGAAGGACCGACGCCCAAAAAGCCTGTAGGATCGACGTTAATAAAGCATCAAGACTATTCCAAGCTTTCGAGAAG
GTTGGCTGGCTACAGGATTCGAATTTTACGAAGTACTTATAA

>YGR210C 1.93 Hot
ATGCCCAGGGATCCATTGATTGGTATTGTGGGTAAGCCATCTTCAGGGAAATCCACCACATTAAATTCTTTGACG
GACGCAGGTGCGGCGGTAGGAGCATTTCCCTTTACCACGATCGAGCCAAATCAGGCAACCGGCTACTTGCAAGTT
GAGTGTGCTTGCTCCAGATTTGGCAAGGAAGATCTTTGTAAACCTAACTACGGGTGGTGTAGCAAGGGTAAGAGG
CATATCCCCATCAAACTGTTGGATGTGGCAGGGCTTGTGCCTGGAGCGCATTCCGGCAGAGGGCTTGGAAATAAA
TTCTTGGATGATTTAAGACACGCAGATGCTCTTATTCATGTGGTGGACGTTAGTGGAACCACAGATGCAGAGGGG
AAGAACACAAGAGGATATGATCCATTGAACGATATAGAATGGCTGCAAGACGAAATCAGGCTTTGGGTTGAGGGG
AACCTGAAGAAAAGATGGGGGTCTATTGTGCGTAAACATACAGCAACGAAATCTAGCATAGTGGACACACTTCAA
GCGCAATTCGGTGGGTACGGTTCACATGCGCCCATGATCCAAAAAGCCCTGGACAGGTTGAAGGGACTACCACCT
TTGGAAAAAATGGGATGACGAATGGATCACTCGAGTGGTCAAGTCCTTTATGATGGAGAAATTTCCAACTGTGTTG
GCACTCAACAAGATTGACCACCCTGACGCCGATAAAAATGTTTCTAAAATCATGCTAAAGTATCCTGACACCAAA
GCTGTACTGACCAGTGCAGTGACGGAAGTTTTTTTGAGGAAATTGAAAAAGCAAGGTTTTATATTATACGAGGAG
GGCACGGAATTTGTTGACACATTTGAGGACGAGCCAGACAAACTAAAGCCACTGGACGACAAGATCCTTAACAGA
ATTGAAAATATCAGAGATTTGGTCCTGTATAGATTCGGATCTACGGGTGTTGTTCAGGTTCTGCAAGCAGCAACG
GACATCCTTGGTCTAATACCGGTGTACACTGTGAAGAACATCCAGACTTTTACGGGTGGCAACGGAACCAACGTA
TTTAGAGATTGTTTTTTGGTCAAGCGAGGAACCCCTGTTGGCAAAGTTGCCAGGTATATCATGGGTGGCGAGGTC
ACCATTGCAAGCATTGAAACCGTTGGCGGCGTGAGAGTCAGTGAAGAAAGTCTTGTTGAGCCCGGTAAGAATGAC
ATACTCGGCTTCAAGATCGCTCCAAGATCAGCATAA

>YJR079W 1.58 Hot
ATGAAGATAAAAATTGACATTATACATTTTATTAACGCCCCCCCTTTTTTTTTTTTTTTTGTTGATGCAGAAGCC
TCGCAACTTAAAGCTTTTCAGTTGTTTCTTCTTGGTCATATTTTTTATACATATATACATACATATATTTGTGAT
TTTGATGAGTTCGAGACTAAAGACCTTGCAGAGGGAAAGATAGGCGATTTGATTTCCAGACTGGAGTTCTGTTCA
AATGCGATAATTGAATCCTTGCCAAATACATTCCAATCCTTTGTCCCGGTCAAATTCAGTACGGACAAGCTTCTT
GAAGAATCGAAAGGTCTTCTCGATGTTTGA

>YLR378C 1.83 Hot
ATGTCCTCCAACCGTGTTCTAGACTTGTTTAAGCCATTTGAATCTTTCCTGCCGGAAGTGATTGCTCCAGAAAGG
AAGGTTCCATACAACCAGAAACTTATCTGGACAGGCGTTTCTCTACTGATCTTTTTGATTCTGGGCCAGATTCCG
CTGTACGGGATCGTGTCCAGTGAGACTTCCGACCCTCTGTACTGGCTACGTGCCATGCTGGCCTCCAACCGTGGT
ACTTTACTGGAATTGGGTGTTTCGCCCATCATCACTTCATCTATGATTTTCCAATTTTTGCAGGGTACTCAGCTT
TTACAAATCAGACCTGAGAGCAAGCAGGACAGAGAGCTGTTCCAAATTGCTCAAAAGGTGTGCGCTATTATTCTG
ATCTTGGGCCAAGCCCTTGTGGTCGTCATGACAGGTAACTACGGTGCCCCTTCGGACCTCGGATTGCCCATCTGT
TTGTTGTTAATCTTTCAATTGATGTTTGCATCGCTGATTGTGATGTTATTAGACGAATTGCTATCTAAGGGTTAC
GGCTTGGGTTCCGGTATTTCTCTGTTCACGGCAACCAATATTGCCGAACAAATTTTCTGGAGAGCGTTTGCTCCT
ACTACAGTCAATTCCGGTCGTGGTAAGGAGTTCGAAGGTGCTGTGATTGCCTTTTTCCATCTTTTGGCTGTCAGA
AAGGACAAGAAAAGAGCCCTTGTCGAGGCCTTTTACCGTACCAATCTACCTAATATGTTCCAAGTGTTGATGACC
GTGGCCATCTTCCTCTTTGTTTTATATTTACAAGGCTTCCGTTACGAATTGCCCATCAGGTCAACCAAAGTGAGA
GGTCAAATTGGTATCTACCCCATCAAACTCTTTTATACTTCCAACACCCCAATCATGTTGCAGAGTGCATTGACT
TCTAACATTTTCTTGATCTCTCAAATCCTTTTCCAGAAATACCCAACCAATCCATTGATTCGTTTGATCGGTGTT
TGGGGTATCAGGCCGGGCACCCAGGGCCCTCAAATGGCCTTGAGCGGGTTGGCCTACTACATCCAACCATTAATG
TCTTTATCCGAAGCTCTTCTGGACCCTATCAAGACCATCGTCTACATCACATTTGTTCTTGGTTCATGCGCAGTA
TTTTCCAAGACATGGATCGAAATCTCCGGCACTTCCCCACGTGACATTGCCAAACAATTCAAAGATCAAGGCATG
GTCATTAACGGTAAGAGAGAAACCTCCATTTACAGAGAATTGAAGAAGATCATTCCAACTGCTGCTGCTTTCGGC
GGTGCTACCATCGGTGCTCTTTCTGTTGGCTCCGACCTACTAGGTACTTTAGGTTCTGGGGCATCCATTTTGATG
GCTACTACCACCATCTACGGCTACTACGAAGCTGCCGCCAAGGAAGGTGGGTTTACTAAGAACCTCGTTCCAGGA
TTTTCTGATTTGATGTGA

>YNR017W 1.85 Hot
ATGTCGTGGCTTTTTGGAGATAAGACACCTACCGATGATGCGAATGCTGCCGTGGGCGGCCAAGATACAACCAAG
CCTAAGGAACTATCGTTGAAGCAGAGTTTAGGTTTCGAGCCAAACATCAATAACATAATATCAGGTCCTGGTGGA
ATGCATGTCGACACCGCTAGGCTGCATCCTTTGGCTGGTCTAGACAAGGGTGTGGAGTATTTAGATCTGGAAGAA
GAACAACTATCCTCGTTAGAAGGCTCACAGGGTCTGATCCCTTCCCGTGGGTGGACCGATGACCTATGTTACGGT
ACCGGTGCCGTCTACCTGCTGGGACTTGGTATCGGAGGGTTTTCTGGTATGATGCAGGGTCTGCAGAATATTCCG

CCCAATAGTCCCGGAAAATTGCAATTGAACACCGTCCTGAATCACATTACTAAGAGAGGTCCCTTCTTAGGTAAT
AATGCGGGGATTCTCGCGTTGAGCTACAATATCATCAATTCTACAATAGATGCACTAAGAGGCAAACATGACACC
GCGGGCTCCATTGGCGCTGGGGCCCTCACGGGCGCTTTGTTCAAGTCTTCAAAAGGTTTGAAACCCATGGGTTAT
TCCTCGGCAATGGTGGCCGCTGCGTGCGCCGTCTGGTGTAGTGTCAAGAAAAGACTACTTGAAAAATGA

>YJL065C 1.88   Hot
ATGAACAACGAGACTAGTGGTAAAGAAACGGCGTCTGCACCTCTGTGTTCGCCCAAGTTACCTGTAGAAAAAGTG
CAGAGAATAGCCAAGAATGATCCAGAATATATGGACACTTCGGATGACGCATTCGTAGCCACAGCGTTTGCTACA
GAATTCTTCGTCCAGGTGCTGACACATGAGTCCCTACATAGGCAACAGCAGCAGCAACAACAACAGGTACCGCCG
CTCCCAGATGAACTCACGCTGTCGTACGATGACATCTCTGCCGCAATTGTGCACTCTTCTGACGGCCATCTGCAG
TTTTTGAATGATGTGATACCAACAACAAAGAATTTGAGGCTTCTAGTGGAAGAAAACCGAGTTAGATATACTACA
AGTGTCATGCCCCCTAATGAAGTTTACTCCGCCTATGTGGTGAACGATACGGCTCCGAAGCCCAACATTGTCGAG
ATTGATCTTGATAATGACGAAGACGACGACGAAGACGTTACTGATCAAGAATAA

>YBL076C 1.58   Hot
ATGTCCGAGAGTAACGCACACTTCTCATTTCCAAAGGAGGAAGAAAAAGTTCTATCTCTTTGGGATGAAATAGAT
GCCTTTCATACTTCATTAGAATTAACAAAAGACAAACCGGAGTTTTCCTTCTTCGATGGGCCTCCATTTGCCACC
GGTACTCCTCATTACGGTCATATTCTTGCTTCCACTATTAAGGATATTGTTCCAAGATACGCTACCATGACAGGC
CACCACGTGGAAAGAAGATTCGGTTGGGATACACACGGTGTTCCAATTGAACATATCATTGACAAGAAATTAGGT
ATCACGGGTAAAGATGACGTCTTCAAGTATGGTCTTGAAAACTACAACAATGAGTGTAGATCCATTGTTATGACT
TATGCCAGTGATTGGAGAAAAACTATTGGTCGTTTGGGTCGTTGGATTGATTTCGACAACGATTACAAGACTATG
TATCCATCCTTTATGGAGTCCACATGGTGGGCTTTCAAGCAACTTCATGAAAAAGGTCAAGTTTACCGTGGATTC
AAAGTTATGCCTTATTCTACTGGCCTAACCACTCCCTTGAGTAACTTTGAAGCTCAGCAAAACTATAAAGATGTT
AACGACCCAGCTGTGACCATTGGTTTCAATGTTATTGGCCAGGAAAAAACTCAATTGGTCGCATGGACTACGACT
CCATGGACTTTACCTTCCAATTTGTCGTTATGTGTTAACGCTGATTTCGAATATGTAAAGATTTACGACGAAACC
AGAGATCGTTATTTCATCTTATTAGAATCTTTGATTAAAACCTTGTATAAGAAGCCTAAGAATGAAAAATATAAG
ATTGTGGAAAAAATTAAAGGTTCTGATTTGGTTGGTTTGAAGTATGAACCATTGTTCCCATATTTCGCTGAACAG
TTCCATGAAACAGCTTTTAGAGTTATTTCAGATGATTATGTTACTAGTGATTCCGGTACTGGTATTGTTCATAAC
GCTCCCGCTTTCGGTGAAGAGGATAATGCTGCTTGTTTGAAGAACGGCGTCATATCCGAAGATTCAGTGTTACCT
AACGCCATTGATGACCTTGGTAGATTCACGAAAGACGTTCCTGATTTTGAGGGTGTTTATGTCAAGGACGCTGAT
AAGTTGATTATTAAGTACTTAACTAATACTGGAAATTTGTTATTGGCATCCCAAATTCGCCATTCCTATCCATTC
TGTTGGAGATCCGATACCCCATTGTTATACCGTTCTGTTCCAGCTTGGTTCGTTCGTGTTAAAAACATTGTCCCT
CAAATGTTGGATTCTGTGATGAAATCTCACTGGGTTCCTAACACCATCAAGGAAAAGAGGTTCGCCAACTGGATC
GCCAATGCCCGTGACTGGAACGTTTCCAGAAATAGATATTGGGGTACTCCAATTCCTTTATGGGTTTCAGACGAT
TTTGAGGAGGTCGTCTGTGTTGGTTCTATCAAAGAATTGGAAGAATTGACCGGTGTGCGTAACATCACTGACTTG
CATCGTGATGTCATTGACAAATTGACAATTCCATCCAAGCAAGGTAAGGGTGACTTAAAGAGAATTGAAGAAGTT
TTTGATTGTTGGTTTGAATCTGGTTCTATGCCTTATGCTTCTCAACATTATCCATTTGAAAACACAGAAAAATTT
GACGAAAGAGTTCCAGCTAATTTCATCTCTGAAGGTTTGGATCAAACAAGAGGTTGGTTCTACACGTTAGCTGTC
TTAGGTACCCATCTATTTGGCTCTGTTCCATACAAGAACGTCATCGTCTCTGGTATTGTCTTAGCTGCCGATGGT
AGAAAGATGTCTAAATCCTTGAAAAATTACCCTGATCCATCCATTGTTCTGAACAAATATGGTGCGGATGCATTA
AGATTGTACTTGATAAACTCACCTGTTTTAAAAGCTGAAAGTTTGAAATTCAAAGAAGAGGGTGTTAAGGAGGTT
GTTTCAAAGGTCTTACTACCATGGTGGAACTCCTTTAAATTTTTGGACGGTCAAATTGCCTTGTTGAAAAAGATG
TCTAACATCGACTTCCAATATGATGATTCTGTGAAAAGTGATAATGTCATGGACAGATGGATTTTAGCTTCTATG
CAATCTCTAGTACAATTCATTCACGAAGAAATGGGTCAGTACAAATTATACACTGTCGTTCCAAAACTTTTGAAT
TTCATTGATGAATTGACAAACTGGTATATTAGATTTAATCGTCGTCGTTTGAAGGGTGAAAACGGTGTTGAGGAC
TGTTTGAAAGCATTAAATTCTTTATTCGATGCCTTATTCACATTTGTCCGTGCCATGGCTCCATTCACCCCATTT
TTGTCAGAAAGCATTTATTTGAGACTGAAGGAATACATCCCAGAAGCTGTCTTAGCAAAATACGGTAAGGACGGA
AGATCAGTCCATTTCTTATCTTACCCAGTGGTCAAGAAAGAATACTTTGACGAGGCCATCGAAACTGCAGTTTCT
AGAATGCAATCCGTTATCGATTTGGGTAGAAACATTCGTGAAAAGAAAACTATTTCCTTAAAAACTCCATTGAAG
ACTTTGGTCATCCTTCACAGTGATGAATCATACTTGAAGGATGTAGAAGCCTTGAAAAACTACATTATCGAGGAA
TTAAATGTTCGTGATGTTGTTATCACCTCCGATGAGGCAAAATATGGTGTTGAGTATAAAGCAGTTGCTGATTGG
CCTGTATTGGGTAAGAAGTTGAAAAAAGACGCAAAGAAGGTTAAAGATGCCCTACCATCAGTCACTTCTGAGCAA
GTACGGGAATATTTGGAAAGCGGAAAGTTGGAGGTTGCCGGTATTGAACTGGTTAAAGGAGATTTGAATGCTATT
AGAGGCTTGCCAGAATCTGCTGTCCAAGCTGGACAAGAAACCAGAACCGACCAAGACGTGTTAATCATCATGGAT
ACAAATATTTACTCTGAACTAAAGAGTGAAGGTCTCGCAAGAGAGCTGGTCAACAGAATCCAAAAATTGAGAAAG
AAGTGTGGTTTGGAAGCCACCGACGATGTTTTAGTGGAGTACGAATTAGTTAAAGATACTATCGACTTTGAAGCC
ATTGTCAAAGAACATTTTGATATGTTAAGCAAGACCTGTAGATCCGACATTGCCAAATATGACGGCTCAAAGACA
GACCCAATTGGTGATGAAGAACAATCTATTAATGACACCATTTTCAAATTAAAAGTGTTCAAATTATGA

>YEL046C 2.13   Hot
ATGACTGAATTCGAATTGCCTCCAAAATATATCACCGCTGCTAACGACTTGCGGTCAGACACATTCACCACTCCA
ACTGCAGAGATGATGGAGGCCGCTTTAGAGGCCTCTATCGGTGACGCTGTCTACGGTGAAGATGTTGACACCGTT
AGGCTCGAACAGACCGTTGCCCGCATGGCTGGCAAAGAAGCAGGTTTGTTCTGTGTCTCTGGGACTTTGTCCAAC
CAGATTGCCATCAGAACTCACTTGATGCAACCTCCATACTCTATTCTATGTGATTACAGGGCTCACGTTTACACT
CACGAAGCCGCTGGACTGGCGATCTTGTCTCAAGCGATGGTGGTTCCTGTGGTTCCTTCCAACGGTGACTACTTG
ACCTTGGAAGACATCAAGTCACACTACGTCCCAGACGACGGTGATATTCACGGTGCCCCCACCAGATTGATTTCT
CTGGAAAACACTTTACACGGTATTGTTTATCCATTGGAAGAACTGGTCCGCATCAAAGCTTGGTGTATGGAAAAT

GGTCTCAAACTACATTGTGACGGTGCCAGAATCTGGAATGCCGCTGCACAATCTGGCGTGCCATTAAAGCAATAT
GGGGAAATCTTCGACTCCATCTCCATCTGTCTATCCAAGTCTATGGGTGCTCCTATTGGGTCCGTCTTGGTTGGG
AACCTTAAGTTTGTCAAGAAGGCCACCCATTTCAGAAAACAACAAGGTGGTGGTATTAGACAATCTGGTATGATG
GCTAGAATGGCTCTTGTAAACATCAACAACGATTGGAAGTCCCAATTGCTGTACTCGCACTCTTTGGCTCATGAA
TTAGCCGAATATTGTGAGGCAAAGGGCATCCCGCTAGAGTCTCCAGCAGACACCAACTTTGTCTTTATTAACCTG
AAGGCCGCTAGAATGGACCCAGATGTCCTTGTTAAGAAGGGTTTGAAGTACAACGTTAAGCTAATGGGTGGTAGA
GTCTCGTTCCACTATCAAGTCACCAGAGATACTTTGGAAAAAGTCAAATTGGCCATCTCCGAGGCCTTCGACTAT
GCTAAAGAACATCCTTTCGACTGTAACGGACCTACCCAGATTTACCGTAGTGAATCCACCGAGGTCGACGTTGAT
GGCAACGCTATCCGCGAAATAAAAACCTACAAATACTGA

>YAL033W 1.54 Hot
ATGGTACGTTTAAAAAGTAGATATATCCTTTTTGAAATTATATTCCCACCTACAGACACCAACGTTGAGGAATCT
GTGTCGAAAGCAGACATCTTGCTTTCGCATCACAGAGCATCGCCTGCGGATGTGTCCATAAAGTCGATACTCCAA
GAGATACGACGCTCGCTGTCGTTGAATCTGGGCGACTATGGGTCTGCAAAATGTAACTCTCTCTTGCAGTTGAAA
TACTTTTCAAATAAGACGTCTACGGGGATAATCCGATGCCATCGAGAGGATTGCGACCTTGTTATCATGGCATTG
ATGTTGATGTCGAAAATTGGCGACGTCGATGGACTGATCGTGAACCCCGTCAAGGTAAGTGGACCATCAAGAAA
ATAGAGCAGTTTGCTATGAGAAGGAATTCTAAAATTCTGAACATAATCAAGTGTAGTCAATCATCACACCTCAGC
GATAATGACTTTATTATCAATGATTTCAAGAAAATTGGAAGGGAAAACGAAAACGAAAACGAGGACGATTAG

>YGR205W 1.62 Hot
ATGTGTGATAAGTCAAAAACGGTATTGGACTACACGATAGAATTCTTGGATAAATATATACCTGAATGGTTTGAG
ACGGGAAATAAATGCCCCTTGTTCATTTTCTTCTCGGGCCCACAAGGCTCAGGCAAAAGTTTTACAAGTATCCAA
ATCTATAACCATTTGATGGAAAAATATGGGGGTGAAAAATCTATCGGTTACGCCTCAATTGATGATTTTTATTTA
ACTCACGAGGACCAATTGAAACTCAATGAGCAATTCAAGAACAATAAGCTGTTACAAGGACGTGGTTTACCAGGT
ACTCATGATATGAAATTGTTGCAAGAGGTATTGAACACCATCTTCAATAATAATGAACATCCGGATCAAGATACT
GTCGTACTACCCAAGTACGATAAATCCCAATTTAAAGGAGAAGGGGACCGTTGCCCCACTGGTCAAAAAATCAAG
CTACCAGTCGATATTTTTATCTTAGAAGGTTGGTTCCTCGGGTTCAACCCCATTTTGCAAGGAATTGAGAATAAT
GATCTTCTTACTGGGGACATGGTTGATGTCAATGCCAAGCTCTTCTTCTATAGCGACTTGTTGTGGCGCAACCCA
GAAATCAAATCTTTAGGGATAGTATTCACCACCGATAACATTAACAATGTTTATGGCTGGAGACTACAACAGGAG
CATGAACTAATATCAAAGGTCGGAAAGGGGATGACCGACGAACAAGTGCATGCTTTTGTGGATAGATACATGCCC
TCCTATAAACTTTATTTGAACGACTTTGTCCGGAGTGAAAGTTTAGGTTCCATTGCTACATTGACGTTAGGAATC
GACTCCAACAGGAATGTATATTCTACGAAAACGAGATGTATTGAATAG

>YLR350W 2.44 Hot
ATGATTGACCGCACTAAAAACGAATCTCCAGCTTTTGAAGAGTCTCCGCTTACCCCCAATGTGTCTAACCTGAAA
CCATTCCCTTCTCAAAGCAACAAAATATCCACTCCAGTGACCGACCATAGGAGAAGACGGTCATCCAGCGTAATA
TCACATGTGGAACAGGAAACCTTCGAAGACGAAAATGACCAGCAGATGCTTCCCAACATGAACGCTACGTGGGTC
GACCAGCGAGGCGCGTGGTTGATTCATATCGTCGTAATAGTACTCTTGAGGCTCTTCTACTCCTTGTTCGGGTCG
ACGCCCAAATGGACGTGGACTTTAACAAACATGACCTACATCATCGGATTCTATATCATGTTCCACCTTGTCAAA
GGTACGCCCTTCGACTTTAACGGTGGTGCGTACGACAACCTGACCATGTGGGAGCAGATTAACGATGAGACTTTG
TACACACCCACTAGAAAATTTCTGCTGATTGTACCCATTGTGTTGTTCCTGATTAGCAACCAGTACTACCGCAAC
GACATGACACTATTCCTCTCCAACCTCGCCGTGACGGTGCTTATTGGTGTCGTTCCTAAGCTGGGAATTACGCAT
AGACTAAGAATATCCATCCCTGGTATTACGGGCCGTGCTCAAATTAGTTAG

>YOL021C 1.59 Hot
ATGTCAGTTCCCGCTATCGCCCCCAGACGGAAGAGACTTGCAGATGGTTTAAGCGTCACTCAGAAGGTGTTTGTA
AGGTCTCGTAATGGAGGCGCTACCAAGATCGTAAGAGAACACTATTTAAGATCGGACATCCCATGTCTTTCGAGA
AGTTGTACCAAGTGTCCGCAAATTGTCGTCCCCGATGCTCAAAATGAGTTGCCGAAGTTCATCTTGTCAGATTCT
CCCTTAGAATTAAGCGCACCCATTGGTAAGCATTACGTCGTCTTGGATACCAACGTGGTGTTACAGGCAATTGAC
CTTTTAGAGAATCCGAATTGTTTTTTCGACGTCATTGTCCCCCAGATTGTCCTAGATGAAGTGAGAAACAAGTCA
TATCCGGTGTACACGAGATTAAGAACTCTATGCAGAGATAGTGACGATCATAAGAGATTCATCGTGTTTCATAAC
GAATTTAGTGAACATACTTTTGTGGAAAGGCTTCCAAATGAAACGATTAATGACAGAAACGACAGGGCTATAAGG
AAAACCTGTCAATGGTATAGTGAGCACTTGAAACCTTATGACATAAACGTCGTTCTTGTTACCAACGATCGTTTG
AATAGAGAAGCTGCCACTAAGGAAGTGGAGTCCAATATAATAACCAAGAGCTTGGTACAGTATATCGAGTTACTA
CCAAATGCAGACGACATCAGAGACTCTATTCCTCAAATGGATTCTTTTGATAAAGATCTTGAAAGGGACACATTT
TCAGATTTCACGTTCCCGGAGTACTATTCTACAGCAAGAGTAATGGGTGGTTTGAAAAATGGCGTTTTGTACCAG
GGCAATATTCAAATATCAGAGTACAACTTTCTAGAAGGTTCAGTGAGTTTACCAAGATTTTCCAAACCAGTGTTA
ATTGTTGGGCAGAAAAACCTAAATAGAGCATTCAACGGTGACCAGGTTATCGTGGAACTGCTACCTCAATCAGAA
TGGAAGGCACCCTCATCCATTGTCTTGGATTCTGAACATTTCGACGTTAACGATAACCCCGATATTGAGGCTGGC
GATGACGATGACAATAATGAGTCCTCTTCCAATACCACCGTGATTTCCGACAAGCAGCGCAGGCTGCTTGCTAAA
GATGCTATGATAGCTCAAAGATCGAAAAAAATTCAGCCCACGGCAAAAGTTGTATATATACAAAGAAGATCCTGG
AGACAGTACGTCGGTCAACTAGCACCAAGTTCTGTTGATCCACAAAGCAGTAGTACACAGAATGTCTTCGTTATT
TTGATGGACAAATGTCTTCCAAAAGTCAGAATCAGAACAAGGCGTGCTGCTGAATTATTGGATAAGAGGATTGTC
ATTTCCATTGATTCGTGGCCCACCACTCATAAATACCCATTAGGTCACTTTGTGAGAGACCTCGGTACAATTGAA
TCTGCTCAAGCTGAAACAGAGGCCTTATTGTTGGAGCATGATGTTGAATATAGGCCTTTTTCCAAGAAAGTTCTA
GAATGTTTGCCCGCAGAAGGCCACGATTGGAAGGCCCCAACAAAACTGGACGATCCTGAGGCTGTTTCAAAGGAT
CCATTATTGACAAAAAGAAAGGATCTCAGAGATAAACTTATATGTAGTATCGATCCTCCAGGATGTGTTGATATT

GACGATGCCCTACATGCGAAAAAGCTTCCAAACGGTAATTGGGAAGTTGGTGTTCATATTGCTGATGTTACTCAC
TTCGTTAAACCGGGCACTGCCCTGGATGCGGAAGGTGCTGCAAGAGGTACTTCTGTATATTTGGTAGACAAACGT
ATTGACATGCTGCCCATGCTTCTAGGTACCGACCTGTGTTCTCTAAAACCATACGTTGATAGATTCGCATTCTCT
GTCATTTGGGAATTGGATGATAGTGCTAATATTGTAAATGTTAATTTTATGAAATCCGTCATCAGATCTAGAGAA
GCTTTCTCATACGAACAAGCACAACTGAGAATTGATGACAAAACCCAAAATGATGAATTAACGATGGGTATGAGG
GCTCTCTTGAAATTGTCTGTAAAACTGAAGCAAAAGAGACTAGAGGCAGGTGCCTTGAACTTAGCTTCTCCTGAG
GTTAAGGTCCATATGGATAGTGAGACTTCAGATCCAAATGAAGTGGAAATCAAAAAATTACTGGCGACAAATTCT
TTAGTTGAAGAATTTATGTTGTTGGCTAATATATCAGTGGCAAGGAAGATATACGATGCCTTCCCTCAAACGGCG
ATGCTAAGAAGACACGCAGCTCCGCCATCTACCAATTTTGAAATCTTAAATGAAATGTTAAACACAAGAAAGAAT
ATGTCAATTTCGTTGGAATCGTCCAAGGCCTTGGCCGATTCTTTAGACAGGTGTGTGGATCCCGAAGATCCATAT
TTTAATACATTGGTTCGTATCATGTCGACTCGCTGTATGATGGCAGCCCAATACTTTTATTCTGGAGCTTATTCT
TATCCTGACTTTAGACACTATGGTTTAGCCGTTGATATCTACACACATTTCACATCACCTATTAGACGTTACTGT
GATGTTGTGGCCCATAGACAATTAGCAGGTGCCATTGGGTATGAACCCCTAAGTTTGACTCATCGTGATAAGAAT
AAAATGGACATGATTTGCAGAAATATCAACAGAAAGCACAGAAACGCCCAATTCGCCGGTAGGGCAAGCATAGAA
TACTATGTCGGGCAAGTAATGAGGAATAACGAGTCCACAGAAACTGGATATGTTATTAAGGTATTTAATAATGGT
ATAGTCGTACTGGTTCCCAAGTTTGGCGTGGAAGGCTTGATAAGACTGGATAATTTGACTGAGGATCCTAACTCA
GCCGCTTTTGATGAGGTGGAATACAAATTAACCTTTGTGCCTACAAATTCAGATAAACCGAGGGATGTTTACGTT
TTCGATAAGGTCGAAGTTCAAGTTAGGTCGGTGATGGATCCAATTACTAGCAAGCGTAAGGCAGAATTATTGTTA
AAATAG

>YDR413C 1.54 Hot
ATGCGACTGCATTATCTCCTACGCTTGTTGTCGTTTGCCTTTCTTTGGTTTATTCTTCGTTCATTTTTGGTCAAA
TATCTGAACTTTTTTACTCTTGGCTTCTTCTTTTGTTTCTCATCCACGCCCAGAAACTTCGCGTATTTGGTGGCT
CTATTTATGCTTTGTTTGAGTACTTTTTCTGCCTTTTCTAATTTGACATTATTTAGCTGGGCTAAATATTCAAAG
TTATCATTGGGTTCCAATGTCTCCAATTCAACAGTAGTTGAGTCATCGTATATTTCCGTGATTGCTCCTTTCTTC
AAAATAGGCTTTACGCTACTTTCATTATTGTCGTTATCGCCGTCATCTTTATCAGAATCAAACCCGTGCCAGGAT
TCATCAGATTCTACCTGCAAATCCTCCGTCTTATCATTTTTCGCATCCTCTATCTCAGCTTCCAAGTTTAAACTC
TCTTTAAACGTCTTTAACTGTTCTTCCATCACCTCCTTACGTTCCTGCCGAATCTTCTGTCTTTCTTCAATTTTT
CTAAGCCTTTCCTGCTCCTTAATAAACTCTTGTGCTTTCTTTTGTCTTTGA

>YGL165C 1.64 Hot
ATGGTTACGTATCCTGTGCAGCCTTGGACAAATTTTATAATTGTATATATCTATGTATATGTATACGAATGGAAA
AATTTATCCGAATCTCGGCCCGACTGCCAGCTTGCCGGGAGAACAAACAACCGCCAATATATGTATATGTATATT
TATATAGATGTCCAGATGCATTATTGTGAATGTGAGTTATGCGAAGATACTTGTTTGTATAGCTCGTTCAACTCA
TTGATGGAGAATGGAATGTCAATATCGTTTAGTGCTGTGTTCGTAGTTGTATATGCGCTTCCTGTTTCATTGATA
CTAACAGGTTCCATAGATATACTAGGGCTTTGCTCAAGCGGATCGAGGGAAGCCAAATCAAGATTGTCAATGCTG
TCGGCCTTAATTTCTCCGTTTTGCAGAGGTGGCTTCAAACTCGGCAATGTGGTTGTCATGTCTACATCCGACGAG
CGACCATTTAGGCCCAAAAGAACGTCCAGATCTGCAATACCGAGACCATTGACATGCGCTGCTTCATCATTTGCC
CTTCTGTGGCATGACCCTCCCTTTGATTTCCGGCTGCTTTTCCTCTTGGTATGA

>YAL012W 1.96 Hot
ATGACTCTACAAGAATCTGATAAATTTGCTACCAAGGCCATTCATGCCGGTGAACATGTGGACGTTCACGGTTCC
GTGATCGAACCCATTTCTTTGTCCACCACTTTCAAACAATCTTCTCCAGCTAACCCTATCGGTACTTACGAATAC
TCCAGATCTCAAAATCCTAACAGAGAGAACTTGGAAAGAGCAGTTGCCGCTTTAGAGAACGCTCAATACGGGTTG
GCTTTCTCCTCTGGTTCTGCCACCACCGCCACAATCTTGCAATCGCTTCCTCAGGGCTCCCATGCGGTCTCTATC
GGTGATGTGTACGGTGGTACCCACAGATACTTCACCAAAGTCGCCAACGCTCACGGTGTGGAAACCTCCTTCACT
AACGATTTGTTGAACGATCTACCTCAATTGATAAAGGAAAACACCAAATTGGTCTGGATCGAAACCCCAACCAAC
CCAACTTTGAAGGTCACCGACATCCAAAAGGTGGCAGACCTTATCAAGAAGCACGCTGCCGGCCAAGACGTGATC
TTGGTTGTCGACAACACCTTCTTGTCCCCATATATCTCCAATCCATTGAACTTCGGTGCAGACATCGTTGTCCAC
TCCGCTACAAAGTACATCAACGGTCACTCAGACGTTGTGCTCGGTGTCCTGGCCACTAATAACAAGCCATTGTAC
GAGCGTCTGCAGTTCTTACAAAACGCCATTGGTGCTATCCCATCTCCTTTCGATGCTTGGTTGACCCACAGAGGT
TTGAAGACTTTGCATCTACGTGTCAGACAAGCTGCCCTCAGCGCCAACAAAATCGCTGAATTCTTGGCAGCAGAC
AAGGAAAACGTTGTCGCAGTCAACTACCCAGGTTTGAAGACACACCCTAACTACGACGTAGTGTTAAAGCAACAC
CGTGATGCCCTTGGTGGTGGTATGATCTCCTTCAGAATCAAGGGTGGTGCTGAAGCTGCTTCCAAGTTCGCCTCC
TCCACAAGACTGTTCACATTGGCCGAATCCCTTGGTGGTATCGAATCTCTATTGGAAGTGCCCGCTGTGATGACC
CACGGTGGTATCCCAAAGGAGGCCAGAGAGGCCTCTGGTGTTTTTGACGACTTGGTTAGAATCTCTGTCGGTATT
GAAGACACTGACGATCTTTTGGAAGACATCAAGCAAGCCTTGAAACAAGCCACCAACTAA

>YPL266W 1.53 Hot
ATGGGAAAGGCTGCGAAAAAGAAGTACTCCGGAGCAACTTCGTCCAAACAAGTCTCTGCCGAGAAACATTTGAGT
TCAGTATTTAAATTCAACACAGATCTAGGTCAGCATATTTTGAAAAATCCTTTGGTGGCGCAAGGTATTGTTGAT
AAGGCACAGATTAGACCCTCAGATGTTGTTTTGGAGGTTGGTCCTGGTACAGGTAACCTAACTGTAAGGATCCTC
GAACAAGCAAAAAACGTAGTGGCAGTAGAAATGGATCCCAGAATGGCTGCAGAATTAACTAAGAGGGTACGTGGC
ACACCTGTGGAGAAAAAGTTAGAAATCATGCTTGGAGATTTTATGAAGACTGAATTACCATACTTTGATATCTGT
ATTAGTAACACTCCTTACCAGATCTCATCGCCTCTGGTTTTCAAATTAATTAACCAACCAAGACCACCAAGAGTA
TCTATTCTTATGTTTCAAAGAGAGTTTGCTTTAAGATTACTGGCAAGACCAGGTGACTCATTATACTGTAGATTA
TCCGCCAATGTACAAATGTGGGCTAATGTTACACACATCATGAAAGTGGGTAAGAATAACTTCAGACCGCCACCA

CAAGTGGAATCCAGCGTTGTTAGACTAGAGATTAAAAATCCAAGACCGCAAGTGGATTACAACGAATGGGATGGT
TTGTTGAGAATCGTCTTTGTGAGGAAAAACAGAACGATTTCAGCTGGCTTCAAATCGACCACCGTGATGGACATT
CTGGAGAAGAATTATAAGACATTTTTGGCGATGAACAACGAAATGGTGGATGATACAAAGGGTTCTATGCACGAT
GTCGTCAAGGAAAAGATTGACACAGTTCTGAAGGAGACCGACTTAGGCGACAAAAGAGCGGGTAAATGTGATCAA
AATGATTTCCTAAGGCTATTATATGCTTTTCACCAGGTTGGTATCCATTTTTCATGA

>YFR034C 1.95   Hot
ATGGGCCGTACAACTTCTGAGGGAATACACGGTTTTGTGGACGATCTAGAGCCCAAGAGCAGCATTCTTGATAAA
GTCGGAGACTTTATCACCGTAAACACGAAACGGCATGATGGGCGCGAGGACTTCAACGAGCAAAACGACGAGCTG
AACAGTCAAGAGAACCACAACAGCAGTGAGAATGGGAACGAGAATGAAAATGAACAAGACAGTCTCGCGTTGGAC
GACCTAGACCGCGCCTTTGAGCTGGTGGAAGGTATGGATATGGACTGGATGATGCCCTCGCATGCGCACCACTCC
CCAGCTACAACTGCTACAATCAAGCCGCGGCTATTATATTCGCCGCTAATACACACGCAAAGTGCGGTTCCCGTA
ACCATTTCGCCGAACTTGGTCGCTACTGCTACTTCCACCACATCCGCTAACAAAGTCACTAAAAACAAGAGTAAT
AGTAGTCCGTATTTGAACAAGCGCAGAGGTAAACCCGGGCCGGATTCGGCCACTTCGCTGTTCGAATTGCCCGAC
AGCGTTATCCCAACTCCGAAACCGAAACCGAAACCAAAGCAATATCCGAAAGTTATTCTGCCGTCGAACAGCACA
AGACGCGTATCACCGGTCACGGCCAAGACCAGCAGCAGCGCAGAAGGCGTGGTCGTAGCAAGTGAGTCTCCTGTA
ATCGCGCCGCACGGATCGAGCCATTCGCGGTCGCTGAGTAAGCGACGGTCATCGGGCGCGCTCGTGGACGATGAC
AAGCGCGAATCACACAAGCATGCAGAGCAAGCACGGCGTAATCGATTAGCGGTCGCGCTGCACGAACTGGCGTCT
TTAATCCCCGCGGAGTGGAAACAGCAAAATGTGTCGGCCGCGCCGTCCAAAGCGACCACCGTGGAGGCGGCCTGC
CGGTACATCCGTCACCTACAGCAGAACGTGAGCACGTGA

>YNL173C 1.64   Hot
ATGCAATCGAGCTTACCTCAATTTACGTTCAAATGGCCCAAAGGACCCGAAGCAATTATTCTGACAGGCACGTTC
GACGACTGGAAAGGTACTTTGCCGATGGTGAAGGACCCCAGTGGCGCCTTCGAAATAACGCTGCCAGTAACGTTT
GATAGCCCTAGCAGCAAGTTTTATTTCAAGTTTATTGTTGATGGCCAATGGCTGCCAAGCAAAGACTACAAGGTG
AACATCGACGAGGGAGTGGAAAACAACTTTATTACCGAGGAAGACGTAATAAAGCAACGCGAAAATGGCTCTAGC
ACGCTGGTACCTGAAAGTGCCGGATTAGCTGTTTCAAAGAATGCCCCTCTTATCGAACCAGAAGCTGAAAAACGT
GCAAAAAAATTAAGAAAGTTCAAGATCAAGAGAGTGATCAAGACAAATAAACAAACCGGAGAAAGGTCGATATTT
TCCCAAGAAGTGGTTGAATTGCCCGATAGCGAGGATGAAACCCAGCAGGTGAACAAAACGGGCAAGAATGCGGAT
GGCTTAAGCGGTACTACAACGATAATTGAGAATAATGTTGGTGTAAACGAGGAAAAAGCAATCAAGCCGTATGAA
GAGAATCACCCCAAAGTTAATCTAGTTAAGAGTGAAGGATATGTTACGGACGGTTTGGGTAAGACGCAATCTTCT
GAGTCTAGATTATATGAACTATCGGCCGAAGATCTTGAAAAGGAAGAAGAGGAAGAGGACGAAGATAAAGGCGGC
GGTAAGGACACCAGTACAAGTGCAGACGCTGAAGCTTCAGAAGATCAAAATAAGGAACCATTAAGTAAATCCGCT
AAATTTGAAAAACCGGAAGAAAAAGTACCTGTAAGCTCAATTACAAGCCATGCTAAAGAGACTTCTGTGAAACCA
ACCGGCAAGGTTGCGACTGAGACTCAAACTTACGAGACGAAACAGGGCGCTCCTACCGCTGCCGCAAAAAAAATC
GAAGCTAAGAAAGCTACTAGACCTTCGAAACCTAAGGGCACGAAAGAAACACCAAATAAAGGTGTCCAAAAGAAC
CCTGCTAAGAATGGAGGGTTCTTTAAAAAGTTGGCCCAGCTTTTGAAGTGA

>YER119C 1.66   Hot
ATGGTAGCTAGTATTAGATCAGGTGTGCTGACACTTTTACACACGGCGTGCGGTGCTGGTATCCTGGCCATGCCG
TACGCATTCAAGCCGTTTGGATTAATTCCTGGAGTGATAATGATAGTGCTGTGTGGGGCGTGCGCTATGCAAAGC
TTGTTCATACAAGCTCGTGTAGCTAAATACGTACCACAGGGGAGGGCCTCGTTCAGTGCTCTGACGCGGTTGATT
AACCCAAATCTAGGTATTGTGTTTGACCTAGCGATCGCTATCAAGTGTTTTGGTGTGGGCGTCTCTTATATGATT
GTAGTTGGGGATCTGATGCCCCAAATAATGTCTGTGTGGACCAGAAATGCTTGGCTGTTGAACAGGAACGTGCAA
ATTTCACTGATCATGCTTTTTTTCGTGGCCCCGCTGTCTTTTCTGAAGAAGTTGAATTCGTTGAGGTACGCTTCC
ATGGTGGCCATCTCTTCTGTAGCGTACTTGTGTGTTTTGGTGTTGTTGCACTATGTAGCACCATCAGATGAGATT
CTTCGCTTGAAAGGCCGGATCTCCTACCTCCTGCCACCACAATCGCATGATTTGAACGTGCTAAACACTCTTCCA
ATTTTTGTCTTTGCCTACACATGTCACCACAATATGTTTTCAATAATCAATGAGCAGAGGTCAAGCCGGTTCGAG
CATGTGATGAAGATTCCACTGATAGCAATATCTTTGGCTCTCATCTTATATATTGCGATCGGCTGTGCTGGATAC
CTGACATTCGGCGACAACATCATTGGAAACATTATCATGCTGTACCCGCAAGCGGTCTCGAGCACCATCGGCAGA
ATAGCGATTGTGTTGTTGGTGATGCTGGCGTTCCCACTACAGTGTCATCCTGCAAGAGCTTCAATCCATCAGATT
TTGCAACACTTCGCCGAGGAAAATGTAAGTATAAGTGCTACATCGGCGGATGAACCCACTGTTGCTACCGAAAGT
TCTCCGTTGATTCGCGACAGTAGCCTTGACCTCAATGAGGTAATTGAAGAAGAATCTATTTATCAGCCTAAGGAG
ACTCCATTGAGAGGCAAGAGCTTTATTGTTATAACGTGCAGCATTTTAGTTGCATCATATCTTGTCGCCATTTCG
GTTTCCTCCTTGGCCCGTGTACTGGCCATTGTTGGGGCCACAGGCTCCACTTCTATTTCTTTCATTCTGCCCGGG
CTCTTTGGCTACAAGCTTATAGGAACTGAGCACAAGACAGCCGTGCCATTGACGACAAAGATTTTCAAATACACA
GGTCTTCTCTTGTTTATCTGGGGGCTAATAATCATGATCACCTGCCTGACCGCAGCTTTGAAGCTAAACTAA

>YPR036W 1.82   Hot
ATGGGCGCAACCAAAATTTTAATGGACAGTACTCATTTCAATGAGATCCGTAGTATAATCCGTTCGAGGTCAGTG
GCATGGGACGCCTTAGCCAGATCTGAGGAATTGAGCGAAATTGATGCGTCTACTGCAAAAGCGTTAGAATCCATT
CTGGTGAAGAAGAACATTGGTGACGGTTTATCATCTTCGAACAATGCACATTCCGGGTTCAAAGTGAATGGCAAG
ACGTTGATACCATTAATTCACTTACTTTCCACCTCAGACAACGAAGACTGCAAAAAATCTGTGCAGAACCTAATA
GCTGAATTGTTATCGTCTGACAAGTATGGAGACGATACCGTGAAGTTTTTCCAAGAAGACCCCAAGCAATTGGAA
CAATTATTTGATGTGTCACTCAAGGGAGACTTCCAGACTGTGCTAATCTCTGGGTTCAACGTGGTCTCACTCTTA
GTGCAAAATGGGTTGCACAATGTGAAACTAGTGGAAAAGCTGTTGAAAAACAACAACTTGATCAATATCTTGCAA
AACATTGAGCAGATGGACACTTGTTACGTGTGCATCAGACTATTGCAAGAACTGGCCGTGATACCAGAGTATCGT

GACGTGATATGGTTGCATGAGAAGAAGTTCATGCCCACCTTATTCAAGATCCTGCAACGTGCCACGGACTCTCAA
TTGGCCACGCGGATAGTTGCAACAAACTCCAACCACCTGGGTATTCAATTGCAGTACCACTCTTTACTATTGATA
TGGTTGCTGACCTTTAACCCAGTTTTTGCAAACGAGCTAGTCCAGAAATACTTGAGTGATTTCTTGGACCTCTTG
AAATTGGTTAAGATAACCATAAAGGAGAAAGTGTCCAGATTGTGCATATCCATCATCCTGCAATGTTGCTCCACG
CGCGTCAAGCAGCACAAGAAGGTGATTAAGCAACTTTTGTTGCTCGGCAACGCGTTGCCCACCGTACAGAGCTTG
AGCGAAAGAAAGTATTCCGACGAAGAATTGCGTCAAGACATCAGCAACCTCAAGGAAATCCTAGAAAACGAGTAC
CAAGAATTGACCTCCTTCGATGAATACGTCGCCGAATTGGACTCCAAGTTGCTGTGCTGGTCTCCACCACATGTC
GACAACGGTTTCTGGTCCGATAACATTGACGAGTTCAAGAAGGACAACTACAAGATCTTTAGACAATTGATCGAA
CTCTTGCAAGCAAAGGTCCGTAACGGCGACGTCAACGCGAAACAAGAAAAGATCATTATCCAAGTCGCCTTGAAC
GACATCACTCACGTGGTCGAGCTTCTACCAGAGAGCATCGACGTTCTCGACAAGACTGGCGGCAAAGCCGACATC
ATGGAGTTGCTGAACCATTCAGATTCTAGGGTGAAATACGAGGCCCTCAAGGCCACGCAGGCAATCATTGGATAT
ACCTTCAAATAA

>YPL177C 1.85 Hot
ATGAATTATAACTGCGAAATACAAAACAGGAACAGTAAGAATGTTGACAATCAAGTCAGTCTACCCCCTATCCAA
GTTCTATTTAACTCCATAGAAAAACGGAGTATGCCCGAGTTGGCATTTTCGAACATAGAATACTCGCATGGGAAC
CTTCGGTCTAGTACCGAGGAACAAAACTATCCTGCGCCTGTGTTACTTCCACAGCATCATTCGATTGCGTATCCC
GCAATTAATTCTGGCGGTACTAGTACTACTGCTACTCCTACTGCTTCTACAGTTGAAACGTCCAAGACGAGTAGC
AGTGCTATGGATACACAATCTCAATATGGCAGTTCAAAGAAGTCAAAATCGGCGTCCGATGATGCAAAGCCCTGC
TACAAATCCGCCCCAATATATGAAATAATCAATAAGGAAAAGGATGCGGGGGCACAATACAACAGGCCATTTTCG
GATTTTGTAGAATCTAAATCAAGGAGGAAGCAAAATTCGGGCAGGAGGTCTAACCTGCCAAAGGAAACAGTGCAG
ATACTGAATACTTGGTTGCTGAACCACCTGAATAACCCCTACCCAACTCAACAAGAAAAGAGGGAGTTGTTAATC
AAAACTGGGCTAACCAAGATTCAACTTTCTAATTGGTTCATAAATGTGAGAAGACGCAAAATATTTAGTGATTAC
TATACCCTGGTAAACTCAATTCCTAATGACAACGCGAATAATACCCCAGTGGAACGAGTTCAAAACGTCTCAGCA
TATCATAACACATTATCTGCCACAAATAATACTATGTACGATGCTACGTCAACCTGCTCCACGGATTATGAATTA
TCCAAGAGATTTGCTCATGCCCCCGTTACACGCCGTAAAAAACTAATTGATAGGCTGGAAGAATTGAAAAAGCTA
TCCAACCCTGATATGAATTGA

>YJL067W 1.61 Hot
ATGAGTAAAAAAAGGAAACGAAAATATGTCCTAATTGTCTTCGTCAACACTCACCACTTTATGCTTCACCTGGGG
ACTGGAACCCTTGGAGGTAGTGGTGGTAGTAATGTCTACCGTGCCATCGTCAAAGTAGATTTTTTCAGTTTTGAC
GGTGCTGGGTTTTGCATCATTGGAATCCTTCGTGGTACTAATGGACTGTGCCCTTCCAACAATTTTCTTGGATCC
ATCTGCAGAAGCATATTCAGCATCGTTGCCCAGATGCAAGTCGTCCCAATTCAACATGAGGTCTTCTGGAGTTCT
AGCCAGAGGCTCTACGGCAGTGCTCCTAGTCTTGATTCTTTGTTTTTGTAG

>YIL108W 1.62 Hot
ATGGTAGGCTCGAAGGATATTGATCTATTCAACCTGAGAGAGAACGAGCAAATCGTTTCTCCTTGTCTTATCGTG
CACGGAAAATGCAATAAGCAAAATGGTGCGAAGACCGTTCAAGTGCAGCATCCACAATTGCCTCCAATTACTTAC
CCCATACATAATCAGTTCTTCAAGGCCACTGTGATATTGACTCCTGGTGAGAACAAGTTGACCTTTGTTACAGAC
ACTAACACCGCTAGGACAATCGTATGTTACTACACACCACTGACGCAGAACCCTCCCGTACACCTGTGTCTGATC
TTGGCCAAGGATTCGCCTTTACAGTTTGACTCTCCTCGAGAACAGAAGGACAGAGAGGGCGGGAATGGCCTCGAA
TTGGCAATCAAGAAGCTAAGACTTGGGGCCAGACTCATGCAAGCGTACACGAACGAGCAAATGCTGAGAAACAGT
ATGGGAAATAGGACGTTTCCCTTTGTTGAAGAATTTACCTGGGACACGCTGTTCGAACGGCCTGCTATGAGAAAC
ACCATCAAAATACATGTAGTTCGTTCGGAAAAGACGGTGAAGGAAATCCAGGACCCTGACATAGCTCAACAGAAT
TCCAAAGGGAAAAACACCGGCGCCCTTTTTGGGATAGCCATGGACGCCCTTAAAAGTTACGGCGGACCCTTCACC
AATAACGAAAAGCCTGTTCAGGCAGCTTGTATGTTTCTAGACACCCACTGGGATGGTAAGTTGATTAGAGGCCAC
GCCGCATTGGGTGGGGGTGACGACAGCATTAAGCTCGCCATTTTTGGGTCGCACGGGCTTTACTCATGGCCTACT
TGTCTCGAGCAATTGGTGCCTTATTTTACTGATGAGACAAGATCCTCCACCAGTGAAGTCGCCAACGACTGTAAT
GAGTGTGGTACTTACTGGGAATGTTTGACTATCACGTTGGGAGCATTTATGCATGAGATTGGTCACTTACTGGGT
TGTCCTCATCAGGAAAGCGGTGTTATGCTAAGAGGCTATACCACTTTGAATAGGTCTTTCTTGACTAAAGAGGCT
TATTCTGTAAGAACTAATTCAACTGGTGCTAGTCCTCCCATCTTCCCCAAGGAAGAATGTACCTGGAACCGTCTC
GATACGGTAAGATTTTTGTACCACCCCTCTTTCACTTTACCACAGGATTATTACGATCCATCGTTCATGAGACCT
ACAAAATTGGGCGGATACCCCAATATAAAACACTCTGTATATCCTTTGGGTAACGGCAGTTGCCGCATATTATCG
CCCACAGGAATCTATTTGATTGAAATTATATGTGATGATTTGGCCAGGGGCCATATTGAATACTTGCCGGTTTCC
CTAGGTGGACAGGGGCCTCAACGGGAAGTTATAGTAACATTAGATGATTTACGTGCCAGGTTGCCCAAGAATGAA
CTCGCCAAGTTTGGCAATACCTTCAAATTGAAAATTCTTTCTGTTAATGCACCGGAAACCGAGTTCGACAAGTTC
CCATCTTTGTTAGATGTTCAGCCATTAGACATGTCGAAATATGGGTTTTCTAAAAATGTTCAAGGTATCAAATCA
CCACTTTATGGTCGCAGCGACGGTGGTAATGCTGTCGGCGTCGTCGCTTTTGATGTTAGATTAGTCACTGCTGTG
AGAATATACCATGGCTATGCATTAGATGGTGTGAGGTTTTATTACAAGGAAAAACCAACTGGAACAAAGGATGCT
CCCGCATCCAAGCCCTCTGTACCACCAAGAAATTATTTCTCAAAAATTACACATAGTATCAAAAATCATGCTTCA
ATAAACGAAGAAAATCTAAAAAGTGTACTCTTTGGACATGAAACACAAAATTTTACGGATGCTACACTAGAACCG
GGTGAAATAATCATTGGGTTTAATCTGAGGTGCGGAGCTTGGGTTGATGCTATCCAAATTATAACGAGTCATGGG
AGAATGACCGACATGTTTGGTAACAAAGACGGAGGTGGGTTCGCTGAATTGCAGCCACCTAATGGCCAATATATC
CTAGGTGTGACTGGCAGAGTCGGTCAATGGGTCGACGCCTTCGGAATTATTTACGGAGCTTTATAG

>YHR218W 1.87 Hot

ATGGATAGGAAAAGGTCTTTTGAAAAAATTGTCGTTTCCGTCATGGTCGGGAAAAATGTACAAAAGTTCCTGACG
TTTGTTGAAGACGAACCAGATTTCCAGGGCGGACCAATCCCTTCAAAGTATCTTATTCCCAAGAAAATCAACTTG
ATGGTCTACACGTTGTTTCAAGTGCATACTTTGAAATTCAATAGAAAGGATTACGATACCCTTTCTCTTTTTTAC
CTCAACAGAGGATACTATAATGAGTTGAGTTTCCGTGTCCTGGAACGTTGTTACGAAATAGCGAGTGCCAGGCCG
AACGACAGCTCTACGATGCGTACTTTCACTGACTTTGTTTCTGGCACACCTATTGTAAGGAGTCTTCAGAAAAGC
ACCATAAGGAAATATGGATACAATTTGGCACCCTACATGTTCTTGTTACTACACGTAGATGAGCTATCGATTTTT
TCTGCATACCAAGCAAGTTTACCTGGCGAAAAGAAAGTCGACACAGAGCGGCTGAAGCGTGATCTATGCCCACGT
AAACCCACTGAGATAAAGTACTTTTCACAGATATGTAACGATATGATGAACAAAAAGGACCGATTGGGTGATGTT
TTAGCTACAGCGCAACGTATACGTCGACGATACAACAAGAACGGTTCATCGGAGCCTCGACTAAAGACGCTTGAC
GGACTCACTTCCGAGCGCTGGATTCAATGGTTAGGCCTTGAAAGCGACTACCATTGTTCATTCTCTAGTACTCGG
AATGCGGAAGACGTAGTGGCAGGTGAGGCGGCGAGTTCAGATCATGATCAAAAAATTTCAAGAGTAACGCGAAAA
AGGCCCCGAGAGCCCAAGAGTACAAACGATATCCTCGTCGCAGGCCGGAAACTCTTTGGCAGCTCCTTTGAATTC
AGGGACTTGCATCAGTTGCGCTTATGTCATGAAATATACATGGCAGACACACCCTCTGTGGCAGTACAGGCCCCA
CCGGGCTATGGTAAGACGGAGTTATTTCATCTCCCCTTGATAGCACTGGCATCTAAGGGCGACGTGAAATATGTG
TCGTTTCTGTTTGTACCGTACACAGTGTTGCTTGCTAATTGCATGATCAGGTTGGGCCGACGCGGTTGCTTGAAT
GTGGCCCCTGTAAGAAACTTTATTGAAGAAGGTTGCGATGGCGTTACTGATTTATACGTGGGGATCTACGATGAT
CTTGCTAGCACTAATTTCACAGACAGGATAGCTGCGTGGGAGAATATTGTTGAGTGCACCTTTAGGACCAACAAC
GTAAAATTGGGTTACCTCATTGTAGATGAGTTTCACAACTTTGAAACGGAGGTCTACCGGCAGTCGCAATTTGGG
GGCATAACTAACCTTGATTTTGACGCTTTTGAGAAAGCAATCTTTTTGAGCGGCACAGCCCCTGAGGCTGTTGCT
GATGCTGCGTTGCAGCGTATTGGGCTTACGGGACTGGCCAAGAAGTCGATGGACATCAACGAGCTCAAACGGTCG
GAAGATCTCAGCAGAGGTCTATCCAGCTATCCAACACGGATGTTTAATCTAATCAAGGAGAAATCCGAGGTGCCT
TTAGGGCATGTTCATAAAATTTGGAAGAAAGTGGAATCACAGCCCGAAGAAGCACTGAAGCTTCTTTTAGCCCTC
TTTGAAATTGAACCAGAGTCGAAGGCCATTGTAGTTGCAAGCACAACCAACGAAGTGGAAGAATTGGCCTGCTCT
TGGAGAAAGTATTTTAGGGTGGTATGGATACACGGGAAGCTTGGGTGCTGCAGAAAAGGTGTCTCGCACAAAGGA
GTTTGTCACTGA

>YGR207C 1.51 Hot
ATGTCTGCAAAACAGCAACTACGTATATTAGTTCCGGTGAAAAGGGTAGTTGATTTCCAGATCAAGCCTAGAGTA
AATAAGACCTTAACTGGGATTGAAACCAGTGGAATCAAATTCAGTATCAACCCTTTCGATGATATTGCTGTCGAA
GAAGCCATCAGAATTAAAGAAAAGAACAAGAGTTTAGTAGAATCTACGCATGCAGTTTCTATTGGCTCCGCCAAA
GCTCAGGATATTCTAAGAAATTGCCTTGCTAAGGGAATAGACACCTGTAGTTTAATTGACTCTGTGGGTAAAGAA
AACATAGAGCCCTTAGCAATTGCTAAAATCTTAAAAGCTGTTGTTGAAAAGAAGGGTTCTAATCTGGTTTTGATG
GGAAAGCAAGCCATTGATGATGACTGTAACAACACCGGTCAGATGTTGGCAGGTTTATTAAATTGGCCACAGGCA
ACAAATGCGGCAAAGGTGGAATTCCTTGATAATGGTAGAGTTCAGGTAACAAGAGAAATTGACGATGGAGAAGAA
GTAATTGAAGCTTCCTTACCAATGGTTATTACTACAGATTTGAGATTGAACACTCCGCGTTACGTAGGACTGCCC
AAACTAATGAAGGCGAAGAAAAAGCCTATTGAAAAATTGGATATAGCAAAAGATTTTCCTGAAATTAATATTGAA
CCTCAGTTAAAGATAGTGTCGATGGAAGAGCCAAAGACTAAGTCACCTGGTGTGAAATTGAATTCTGTGGATGAA
TTGATTGAAAAGTTAAAAGAAGTTAAGGCCATTTAA

>YOR334W 1.52 Hot
ATGAATCGGCGTCTCCTGGTACGTTCTATATCTTGTTTCCAACCTTTGTCGAGAATAACTTTTGGAAGACCAAAC
ACGCCATTTCTTAGAAAGTATGCTGACACATCCACTGCTGCAAACACCAACAGCACCATATTGCGGAAACAGTTA
CTATCGTTGAAGCCCATTTCTGCCTCTGATTCACTGTTCATTTCGTGTACGGTATTCAATTCTAAGGGAAATATT
ATCTCAATGTCCGAGAAGTTTCCTAAATGGTCCTTTTTAACTGAACATTCCCTTTTCCCCAGAGACCTGAGGAAA
ATAGATAACTCCTCTATTGATATTATTCCAACCATCATGTGTAAGCCAAACTGTATTGTCATCAACTTATTGCAT
ATCAAAGCTCTTATCGAACGAGATAAGGTCTACGTCTTCGATACAACAAACCCTTCCGCCGCTGCCAAACTGAGT
GTACTTATGTATGACTTGGAGTCTAAGTTGTCCTCCACCAAGAATAACTCTCAATTTTACGAGCATAGAGCCCTC
GAGAGTATTTTCATCAACGTAATGAGCGCACTGGAAACAGATTTCAAGCTTCACTCACAAATCTGTATTCAAATC
TTAAATGATCTGGAAAACGAGGTCAATAGACTTAAACTGCGGCATCTTTTAATTAAGTCCAAAGATCTTACGCTT
TTTTACCAAAAAACTTTATTGATTAGAGATCTATTAGATGAACTATTAGAAAACGACGATGATTTAGCAAACATG
TACTTGACAGTTAAGAAGTCTCCTAAGGACAATTTTTCGGACTTGGAAATGCTTATAGAGACGTACTACACCCAA
TGTGATGAATACGTTCAGCAATCAGAATCTTTGATTCAGGATATCAAATCTACTGAAGAAATTGTCAACATCATA
TTGGACGCAAATAGAAATTCCTTAATGTTGTTGGAGTTGAAAGTTACCATCTACACGTTGGGATTCACAGTAGCA
TCTGTTCTGCCGGCATTCTATGGTATGAATTTAAAGAATTTCATCGAGGAGAGTGAATGGGGGTTTACTTCAGTG
GCGGTATTTTCTATTGTTTCTGCCCTTTATATCACCAAGAAAAATTTTAATTCTTTAAGATCCGTGACAAAGATG
ACCATGTATCCAAACTCACCCGCAAACTCCAGTGTGTATCCTAAAACATCAGCGTCTATTGCCCTGACAAATAAA
CTAAAGCGAAGGCGGAAGTGGTGGAAATCAACCAAGCAGCGGTTGGGAGTGCTACTTTATGGCAGCAGCTACACT
AATAAGGCTAACCTGTCGAATAATAAGATTAATAAAGGTTTTTCAAAGGTGAAGAAATTTAACATGGAGAATGAT
ATTAAGAACAAGCAGAACAGAGATATGATTTGGAAATGGTTGATAGAAGACAAGAAAAATTGA

>YIL045W 1.56 Hot
ATGGCCACCACCACGCAACCACAAAATATACTGATGGATGAACCTTTAAATCTTCCTAATAACAGTGCCCACAAC
AATAACTATGGAAACATAAATGCGAATATAAGAACTTTTGCTGGTATGAGCATGCACATGCACCCTGCCAGGCTG
AACTCTCTGGAGTTTTTGCACAAGCCCAGGAGACTATCTAATGTAAAACTGCACAGATTACCTCAGGACGAGCTT
CAAAGAAATACGGACATGAATAAGGGAATGTATTTTAATGGGAAACAAGTTCATGCCCATCACCCGTTTATAAAT
TCTGGAGCGAACTTTAACGCACATCATCAAGACGTCAGTAAATTAGGCGAGGAGGAAGACGAAATCTCTCCTCTA
TCACATGATAATTTCCAGTATGAATCCGAGGAAAATGGTAATCCTTCACCTCCCATTTACAAGAAATCTGGAGAA

CTGGTTAAGAGTTCATTAAAAAGAAGATCCAAGTCCCTACCCATTACTCCCAAATCTATATTCAACAAAACTGGC
TCTAAGAGTAAACATGTCAATTTAGATCATGTAGATACTAGGCTATTGCAAAGAAGTAAAAGTGTCCATTTCGAT
CGTGTTTTACCAATAAAGCTGTTCAATGAAAATGAGAAACCCATAGATGTTGGCAAACAAATGGTTCAACAAGAT
GTTCTGAATTTCAAGCATAAGCCTTTGACGAGACTAAGTGCCCTTAATGGCGGTAGTGATAGCGTACCTATAGAA
GATTTACTATCTGAAAACAACCAAAACGAATATGGAGATACATGGCTACAAAACCCGAAGGGTGTATTCCTATTT
GGTACAAATTCTAATAATCGTAGAAATAAAAAAAAGAAGTTTAAACTAAGTGACGATGACAGTGATATTGAAAAT
GACAATGATAGTGACGACGCTATAAACCGTTTAGTAAGGCAACAAGACAAAGACCAAGCTCATCTTGCGCACGGG
TTGAAGAATTTGTTAATAAACGATGACGACGATTATTTAGAAACAAGAACAAATTCTGCTAAATCAGGAGCCAAC
TTGTTTATTGGAAACTCTAAAAGAATTGTTGGTCTTTACAACAAGAACTTTCCAATATTAAGTGACAGGAACCGT
AAGAGTTTAAAACTTAACATATTTCTGAATCTTTCCCGTGGAAGGCCCGTTTTTTTGCAAGAAATTACGCTACTA
ACTGGCTTCCACAACATGGTTATAATTGGCAAAGTCTTTGTGAAAAACATATACTTTGATAAGAAGATTATCGTA
AGATATACATGGGATGCATGGAGAACTTTTCATGAATCAGAATGCGTGTATTTTTCTAATGCCAATGGCATCTTA
CCAGGAAGCAATATGGATATTTTTAAGTTCTCCATTGATGATATACACAATCCAAATGATAAAGATAGCAATATA
TCACAATTGGAGTTTTGTATTCAATACTTAACTTGGGGCGTTGATCGTTCTAGGAAGGAATATTGGGACAATAAT
GATTCGGCAAACTATAAAATTGACGTGGTAACGAATGAAACGAGGACAGGGCCCACAACAGACGTCAATGATAAC
TACGAGATGAAACATAGTCTTTTCAGAAACCCATTCCATTAA

>YCR052W 1.61 Hot
ATGGTAACACAGACCAATCCGGTCCCTGTTACATATCCAACGGATGCTTATATCCCCACGTATCTGCCCGATGAT
AAGGTCTCCAATCTGGCAGATTTGAAAAAATTGATAGAAATGGATTCCAGACTAGATTTGTATCTGACAAGAAGG
AGGCTGGATACGTCCATCAATTTACCTACAAACACCAAGACCAAGGACCATCCCCCCAATAAAGAGATGCTGAGG
ATTTACGTCTACAACACTACGGAAAGCAGCCCTCGCAGCGATTCTGGCACCCCAGCGGACTCAGGCAAGACTACA
TGGACACTGAGAATAGAAGGTAAGCTTCTGCACGAGTCCGCAAACGGAAAGCACCCATTTAGTGAGTTTTTGGAA
GGTGTCGCGGTCGACTTTAAAAGACTGAAACCGCTGGGCATGGGCAAGAAGAGGAAACGCGATTCGTCATTGAGC
CTTCCTTTGAATCTGCAACAACCCGAATACAATGATCAAGATAGCACCATGGGCGATAACGACAACGGCGAGGAT
GAGGACAGTGCAGAGGCAGAATCCAGGGAGGAAATTGTAGACGCACTGGAATGGAACTACGATGAAAACAACGTT
GTGGAGTTTGATGGTATCGACATCAAGAGGCAAGGCAAGGATAATTTGCGATGCAGTATAACCATCCAGTTGAGG
GGTGTCGACGGTGGAAAAGTACAGTACTCGCCCAACTTAGCTACCTTGATAGGTATGCAAACGGGCTCCGTTAAT
GACGCGGTTTATTCGATCTACAAGTACATTTTGATCAACAATCTGTTTGTTACGGAACAAACAGAGGCTCAAGAT
GGTTCCAACGATGCCGAAGACAGCAGTAACGAGAATAACAATAAAAACGGTGCTGGTGACGATGATGGCGTCGAG
GGAAGTACTCCAAAGGATAAGCCCGAATTGGGTGAAGTGAAGCTAGATTCACTCTTACAAAAGGTATTGGATACA
AACGCCGCGCACCTCCCCTTGATGAATGTTGTGCAAACCGTGAACAAACTGGTATCACCCCTACCGCCCATCATC
CTAGATTATACAATTGATCTTTCCAAAGATACCACCTATGGTGCTACCACCTTGGATGTAGATGTGTCGCACATT
CTCCACCAGCCTCAACCCCAGCCAAATTTACAAAAAGAGGAAGAAACAGATGCTGAAGACACAGCAAAACTACGT
GAAATCACAAAGCTTGCCTTGCAGTTGAACTCTAGTGCTCAAAAATACCAGTTTTTCCACGAACTGTCTTTGCAT
CCAAGAGAAACGCTGACTCACTACTTATGGTCTTCCAAGCAAAACGAGCTTGTGCTGCAGGGCGACCAATACTTC
AATGAAGATGCTGCAAGAACGAGTGACATATACAGTAACAACAACAATGACAGGTCACTAATGGGCAATATCTCA
CTACTGTACTCCCAAGGAAGACTATAA

>YCL029C 1.72 Hot
ATGGATAGATATCAAAGAAAGATAGGATGTTTCATACAAATCCCAAATTTGGGGCGCGGACAACTGAAATACGTG
GGTCCAGTGGACACGAAAGCTGGAATGTTTGCTGGTGTAGACTTACTTGCCAACATTGGTAAGAACGATGGATCA
TTCATGGGGAAGAAGTATTTTCAAACGGAGTATCCTCAAAGTGGACTATTTATTCAGTTGCAAAAAGTCGCATCA
TTGATCGAGAAGGCATCGATATCGCAAACCTCGAGAAGAACGACAATGGAACCGCTATCAATACCCAAAAACAGA
TCTATTGTGAGGCTCACTAACCAGTTCTCTCCCATGGATGATCCTAAATCCCCCACACCCATGAGAAGTTTCCGG
ATCACCAGTCGGCACAGCGGTAATCAACAGTCGATGGACCAGGAGGCATCGGATCACCATCAACAGCAAGAATTT
GGTTACGATAACAGAGAAGACAGAATGGAGGTCGACTCTATCCTGTCATCAGACAGAAAGGCTAATCACAACACC
ACCAGCGATTGGAAACCGGACAATGGCCACATGAATGACCTCAATAGCAGCGAAGTTACAATTGAATTACGAGAA
GCCCAATTGACCATCGAAAAGCTACAAAGGAAACAACTACACTACAAAAGGCTACTCGATGACCAAAGAATGGTC
CTCGAAGAAGTGCAACCGACTTTTGATAGGTATGAAGCCACAATACAAGAAAGAGAGAAAGAGATAGACCATCTC
AAGCAACAATTGGAGCTCGAACGCAGACAGCAAGCCAAACAAAAGCAGTTTTTTGACGCTGAGAATGAACAGCTA
CTTGCTGTCGTAAGCCAACTACACGAAGAGATCAAAGAAAACGAAGAGAGAAATCTTTCTCATAATCAACCCACT
GGTGCCAACGAAGATGTCGAACTCCTGAAAAAACAGCTGGAACAATTACGCAACATAGAAGACCAATTTGAGTTA
CACAAGACAAAGTGGGCTAAAGAACGCGAACAATTGAAAATGCATAACGATTCGCTCAGTAAAGAATACCAAAAT
TTGAGCAAGGAACTATTTTTGACAAAACCACAAGATTCCTCATCGGAAGAGGTGGCATCCTTAACGAAAAAACTT
GAAGAGGCTAATGAAAAAATCAAACAGTTGGAACAGGCTCAAGCACAAACAGCCGTGGAATCGTTGCCAATTTTC
GACCCCCCTGCACCAGTCGATACCACGGCAGGAAGACAACAGTGGTGTGAGCATTGCGATACGATGGGTCATAAT
ACAGCAGAATGCCCCCATCACAATCCTGACAACCAGCAGTTCTTCTAG

>YOR176W 1.6Hot
ATGCTTTCCAGAACAATCCGTACACAAGGTTCCTTCCTAAGAAGATCACAACTGACCATTACAAGATCATTTTCG
GTTACATTCAACATGCAGAATGCACAAAAGAGATCACCCACAGGAATTGTTTTGATGAACATGGGTGGCCCCTCT
AAAGTTGAGGAAACATATGATTTTTTGTATCAATTATTTGCCGATAATGACCTAATTCCCATTAGTGCTAAGTAT
CAGAAGACAATTGCTAAATATATTGCTAAGTTTCGTACCCCCAAGATAGAGAAGCAATATAGGGAAATTGGTGGG
GGCTCCCCAATCCGGAAATGGTCTGAGTATCAAGCCACTGAGGTCTGTAAAATCTTAGATAAAACCTGTCCAGAA
ACGGCGCCTCATAAGCCTTACGTGGCGTTTCGTTACGCAAAGCCGCTAACCGCAGAAACTTATAAACAAATGCTA
AAAGATGGCGTGAAGAAGGCAGTGGCCTTTTCTCAATATCCTCATTTCTCTTATTCCACTACCGGGTCATCCATC

AACGAATTGTGGAGACAGATTAAGGCATTGGACTCCGAGAGATCTATATCTTGGTCGGTTATTGATCGTTGGCCT
ACAAATGAAGGTCTAATCAAGGCCTTCTCCGAAAATATCACCAAAAAACTACAAGAGTTTCCGCAACCTGTCAGA
GACAAGGTTGTTTTATTGTTTTCCGCACATTCTCTACCCATGGATGTTGTTAACACCGGTGATGCCTACCCAGCT
GAGGTAGCTGCGACGGTTTACAACATCATGCAAAAATTAAAGTTTAAAAACCCTTATAGGTTGGTTTGGCAATCC
CAAGTTGGACCAAAACCATGGTTGGGAGCGCAGACAGCTGAAATTGCGGAATTTTTAGGCCCCAAAGTTGATGGC
CTAATGTTTATTCCTATCGCCTTTACCTCTGATCATATTGAAACATTGCATGAAATTGACTTAGGCGTCATTGGG
GAATCGGAATATAAGGATAAATTTAAGAGATGCGAATCTTTAAATGGCAACCAGACCTTTATTGAAGGCATGGCA
GATCTCGTCAAAAGCCACTTACAGAGTAACCAACTCTATTCTAATCAACTACCTCTTGATTTTGCACTTGGCAAG
TCCAATGATCCTGTAAAGGACCTTTCATTGGTATTTGGCAATCACGAATCTACTTGA

>YCL040W 1.75   Hot
ATGTCATTCGACGACTTACACAAAGCCACTGAGAGAGCGGTCATCCAGGCCGTGGACCAGATCTGCGACGATTTC
GAGGTTACCCCCGAGAAGCTGGACGAATTAACTGCTTACTTCATCGAACAAATGGAAAAAGGTCTAGCTCCACCA
AAGGAAGGCCACACATTGGCCTCGGACAAAGGTCTTCCTATGATTCCGGCGTTCGTCACCGGGTCACCCAACGGG
ACGGAGCGCGGTGTTTTACTAGCCGCCGACCTGGGTGGTACCAATTTCCGTATATGTTCTGTTAACTTGCATGGA
GATCATACTTTCTCCATGGAGCAAATGAAGTCCAAGATTCCCGATGATTTGCTAGACGATGAGAACGTCACATCT
GACGACCTGTTTGGGTTTCTAGCACGTCGTACACTGGCCTTTATGAAGAAGTATCACCCGGACGAGTTGGCCAAG
GGTAAAGACGCCAAGCCCATGAAACTGGGGTTCACTTTCTCATACCCTGTAGACCAGACCTCTCTAAACTCCGGG
ACATTGATCCGTTGGACCAAGGGTTTCCGCATCGCGGACACCGTCGGAAAGGATGTCGTGCAATTGTACCAGGAG
CAATTAAGCGCTCAGGGTATGCCTATGATCAAGGTTGTTGCATTAACCAACGACACCGTCGGAACGTACCTATCG
CATTGCTACACGTCCGATAACACGGACTCAATGACGTCCGGAGAAATCTCGGAGCCGGTCATCGGATGTATTTTC
GGTACCGGTACCAATGGGTGCTATATGGAGGAGATCAACAAGATCACGAAGTTGCCACAGGAGTTGCGTGACAAG
TTGATAAAGGAGGGTAAGACACACATGATCATCAATGTCGAATGGGGGTCCTTCGATAATGAGCTCAAGCACTTG
CCTACTACTAAGTATGACGTCGTAATTGACCAGAAACTGTCAACGAACCCGGGATTTCACTTGTTTGAAAAACGT
GTCTCAGGGATGTTCTTGGGTGAGGTGTTGCGTAACATTTTAGTGGACTTGCACTCGCAAGGCTTGCTTTTGCAA
CAGTACAGGTCCAAGGAACAACTTCCTCGCCACTTGACTACACCTTTCCAGTTGTCATCCGAAGTGCTGTCGCAT
ATTGAAATTGACGACTCGACAGGTCTACGTGAAACAGAGTTGTCATTATTACAGAGTCTCAGACTGCCCACCACT
CCAACAGAGCGTGTTCAAATTCAAAAATTGGTGCGCGCGATTTCTAGGAGATCTGCGTATTTAGCCGCCGTGCCG
CTTGCCGCGATATTGATCAAGACAAATGCTTTGAACAAGAGATATCATGGTGAAGTCGAGATCGGTTGTGATGGT
TCCGTTGTGGAATACTACCCCGGTTTCAGATCTATGCTGAGACACGCCTTAGCCTTGTCACCCTTGGGTGCCGAG
GGTGAGAGGAAGGTGCACTTGAAGATTGCCAAGGATGGTTCCGGAGTGGGTGCCGCCTTGTGTGCGCTTGTAGCA
TGA

>YIL056W 1.86   Hot
ATGAACGGTCCTCCAACATTCACTCAATATAGAATAAATAAGTTTTCTGGGAATGGAGCAACTCATAAGATTAGA
GAATTACTCAATTTTAATGATGAAAAGAAATGGAAACAGTTTTCGAGCAGAAGGCTGGAGCTGATAGACAAATTC
CAACTAAGTCAATATAAGGCAAGCGAACAAGATCAAAACATAAAGCAGATTGCTACAATATTAAGAACGGAGTTT
GGATACCCTGTAAGCTGCTCTAAGGAATTCGAAAAGTTAGTCACTGCTGCTGTGCAATCAGTGAGAAGGAATAGA
AAACGATCAAAAAAACGCTACGCTCTCAGCATAGCCAATGGCAGTGGCGGCAATGTCAACAACAGCATCTCGTCA
AATTCTACTTCTGACGACGAGATCTCTCCGTCGATCTACCAGCGCTCCAATTCGGACTTCCTGCCTAGTTCTAAT
TATGCTGCAGATTTCCAGTTTTCAAACAAGTTCCAGCCTCTCATGAGTCACCAGAGTCATAACGGAACCATTTTT
CCAACCGTGGGCACCCAAAATGACTCATCTCCATCGGTTACTTCGACCCAACAAAAGTATAACGATATAGTCACC
ATGCTAGTGCACGATCTTGTTACGAATGTCGTACCCCTATCAGAACAAGCTCTCAAGGACCCATATACTGGGCCA
AACCTGTCACATTTTGCTACGTCTTCACTCAGCCAGCAACCTAACATTACCACAAACATCCCGATAGATTCTACC
GTGCCATTTTTCCTGAGGGAAAAGTTACTCCTACAAATCCAAAGATCAAGAACTTGTCAGGACATTTCGCAGGCT
GCAGGATCCATTGATATATACGCAAATCTGGAGATTTTGGGTGAAATGTCAATCCGAATGTCTATTGCATTCGTT
ATCGAACGATTTTTCTCCAACTTGGTCTCTTCTTCAATGAAGTATATCACTGCCAAGACGTGCTCTCCTGAAAAT
CTGGCTTTGTTGTCTCAAAGACTGTTCGGTGCCGCTACAAGACATAACTTATCTCATTTCCCCGCGGCTCAGGTA
CAACTAAGGCTACTGTATTTGGTCATAGGTGGTATTGTCAAGGATTTTGGCTTCGACCCCACGCTTTACCCTTTA
AGTGAAATCATACATCATATTGTGATGGTTCAGTATCCTTTGGCTAGTTCCTGTGCGTCAGAACCACCATCATCT
TCACCTAACAAGAGGGTTAAGAGAAGCCCTCCTGTCGTTTCAAGTGACGTTATGTTGAATAATAATAACACTCTA
TCGAATAGAGCTACACTTTTAACTACGCTACCAATGAAGCCTCAATCGGCCAATAAAGATGTTAATCGCAGAGTG
ATCATTAGATTTAATGATCGCGAGCAAGCCTTTACTTTCCACCAGTTGAGCAATGGACCACCCACGGTGTCTGAA
GTTTTGGAAAACTGTAAAAATTTATTTAATATTATCAACAAGAACAAGAATTTTGGTATTTTCCATAACGATAAC
CTCTTGAACGATGAAAGTTTGGCAAAGTTATTTGATTCCTTTTCCACGAGCGAGATTCATCTCGTAATTAAGGAC
ATTTCCACCATTCCACTACAAGATGCAAAAATACCAGTGCCCATTACGTTGCCCAAAATGTCATGCATAGGTGAA
AACCCCTCTATGCCTTCTATTCCATTGGTACCACAAGAAAAAGATGATCCAAAAAAATCAAGCTTAACTGCCTTT
GATAACATCCTTAATAGGATCTCTAAATCGCCAATGAATGAAGAAAATAGCAACACTACTTTAAACACTGGCACC
TCCACCTCCAACACAAATAATAATGACCACAACGAGTCTGTGCCAGCGCCATACGTTACAAAGAACAAAAATTCT
TTTCAAAATGGCAATTTACCACAGCCGGTTTTCCAGCCTTTACTTTGA

>YCR047C 1.76   Hot
ATGTCACGTCCTGAGGAGTTGGCACCACCGGAGATTTTCTATAATGATAGCGAAGCACACAAGTACACGGGTTCG
ACCAGAGTGCAGCATATCCAGGCGAAGATGACGCTGAGGGCGTTGGAGCTTTTGAATCTGCAGCCGTGCAGTTTC
ATTCTGGATATCGGGTGCGGGTCCGGACTGTCTGGGGAGATTTTGACGCAGGAGGGAGACCATGTGTGGTGTGGT
TTGGATATATCGCCCAGCATGCTTGCGACCGGTCTTAGTAGAGAGCTGGAGGGCGACTTGATGTTGCAGGATATG
GGCACCGGGATACCGTTCCGGGCGGGCTCGTTTGACGCGGCTATTAGTATCAGTGCGATCCAATGGCTGTGCAAT

GCGGACACTTCATACAACGATCCTAAACAGCGGTTGATGAGGTTTTTCAACACATTGTATGCTGCACTGAAGAAG
GGAGGGAAATTTGTGGCCCAGTTCTACCCGAAAAACGACGACCAGGTGGACGACATACTGCAGTCTGCCAAGGTG
GCAGGGTTCAGTGGCGGGCTTGTGGTGGACGACCCAGAGTCTAAAAAGAATAAGAAGTACTACCTTGTGTTGAGC
AGTGGGGCCCCACCGCAGGGGGAGGAGCAGGTGAATTTGGACGGTGTGACCATGGACGAGGAGAACGTCAACTTG
AAGAAACAACTGCGCCAGCGCTTGAAGGGAGGCAAAGACAAGGAGTCTGCCAAGAGTTTCATTCTAAGAAAGAAG
GAGCTCATGAAAAGACGTGGGAGGAAAGTTGCGAAGGACTCCAAGTTCACCGGGAGGAAAAGAAGACACAGGTTC
TAG

>YOR268C 1.79   Hot
ATGGTTTTTGTTTTCCCCTTCCCATTTTTTTCTTATGGTTTTTCCAGTTTTCTAGAAGCCGGAAAAAAGGCAAGC
TACAAAATGTACTACGCGGAGCCAGAATTAAAAACCACGCGGACGGGACGGGCCGTGGCTTGTGACGCCGGATCC
CCGCGTATAATCCGTGTCACGCTAAAGGACAAAATCGGTTTATCCGAGCGGTTTACGGGAAGGGTCTTTTGCTAT
CTCGCAGTAGCGTGTGCCTGGTTATCCCAATACTACCACCACACCTGTGCTTTTTTTATCTTGTACGTGCATGTA
TGCGTATGTTTTCTTTTTTTCCGTTGGTGCTTATTCGCTACCGTCTCGTTGATCCATGAATCACAAACACAAGCC
GGTTCTGCTTACTATACAAAATAG

>YJL066C 1.57   Hot
ATGGGCTTTTATGAAGGCGATGACAATGATGCCAACACCAAGGCATTTAATGACAAATATATCAAAGATCAAAAG
TTCGCAACGGCACCCTTCTGGAACCTCTTTCCAAAGTTAAGGGATATTGATGAATACGATAATCCACTCCTACCA
CTGCCCTTCAATTTCAACTTCAGAGATCTTGGAGATAGTGCACTTGCCATGGCGTCCGGAATCCCTACGGTGAAG
CAGTTTGACAAGTGCGAAGAACTTAAGGGCCAGTCTGCATGGACCACCCAAGGGATTTGGAAATGTCTTGTCCCA
AGTAAGGCTATCCCCCCTCTACCACAATTGGACTTCCTCTTACCTCTGGAGGAGATCAAATCTGACAAGTCGCAC
TCTCATGGGTTGTTCTTCAATGATTTCAACCTCTTTTTGAAGTGGAGGTCGCACATGAATAGACTACAAAAACAA
AGAATCAAGACTAGGAGCACTGCCGTAGAGCCTCTGGCTAGAACTCCAGAAGACCTCATGTTGAATTGGGACGAC
TTGCATCTGGGCAACGATGCTGAATATGCTTCTGCAGATGGATCCAAGAAAATTGTTGGAAGGGCACAGTCCATT
AGTACCACGAAGGATTCCAATGATGCAAAACCCAGCACCGTCAAAACTGAAAAAATCTACTTTGACGATGGCACG
GTAGACATTACTACCACCACTACCTCCAAGGGTTCCAGTCCCCAGGTGAAGCATAAAGTGGTGAGTGTTGACGAA
GACAATTAG

>YKL190W 1.69   Hot
ATGGGTGCTGCTCCTTCCAAAATTGTGGATGGTCTTTTAGAAGATACAAATTTTGATAGAGATGAAATTGAAAGG
TTAAGGAAGAGATTCATGAAATTAGATAGAGATAGCTCAGGGTCTATTGATAAAAATGAATTTATGAGCATTCCT
GGCGTTTCGTCAAACCCTCTTGCTGGACGTATAATGGAGGTTTTCGATGCTGATAATAGTGGGGACGTGGATTTT
CAAGAGTTCATCACAGGATTATCCATTTTCAGTGGGCGTGGGTCCAAGGACGAAAAGTTAAGATTCGCCTTCAAA
ATCTACGACATTGACAAGGACGGTTTCATATCCAATGGTGAGTTGTTCATCGTGTTGAAGATTATGGTAGGTTCT
AATCTGGACGATGAACAGCTGCAACAGATAGTAGATAGGACGATAGTGGAAAACGATAGCGACGGCGACGGACGT
TTAAGTTTCGAGGAGTTTAAGAATGCTATCGAAACCACAGAAGTGGCCAAGAGTCTGACATTGCAATACGATGTG
TAA

>YJL052W 2.22   Hot
ATGATCAGAATTGCTATTAACGGTTTCGGTAGAATCGGTAGATTGGTCTTGAGATTGGCTTTGCAAAGAAAAGAC
ATTGAGGTTGTTGCTGTCAACGATCCATTTATCTCTAACGATTATGCTGCTTACATGGTCAAGTACGATTCTACT
CATGGTAGATACAAGGGTACTGTTTCCCATGACGACAAGCACATCATCATTGATGGTGTCAAGATCGCTACCTAC
CAAGAAAGAGACCCAGCTAACTTGCCATGGGGTTCTCTAAAGATCGATGTCGCTGTTGACTCCACTGGTGTTTTC
AAGGAATTGGACACCGCTCAAAAGCACATTGACGCTGGTGCCAAGAAGGTTGTCATCACTGCTCCATCTTCTTCT
GCTCCAATGTTTGTTGTTGGTGTTAACCACACTAAATACACTCCAGACAAGAAGATTGTCTCCAACGCTTCTTGT
ACCACCAACTGTTTGGCTCCATTGGCCAAGGTTATCAACGATGCTTTCGGTATTGAAGAAGGTTTGATGACCACT
GTTCACTCCATGACCGCCACTCAAAAGACTGTTGATGGTCCATCCCACAAGGACTGGAGAGGTGGTAGAACCGCT
TCCGGTAACATTATCCCATCCTCTACCGGTGCTGCTAAGGCTGTCGGTAAGGTCTTGCCAGAATTGCAAGGTAAG
TTGACCGGTATGGCTTTCAGAGTCCCAACCGTCGATGTTTCCGTTGTTGACTTGACTGTCAAGTTGGAAAAGGAA
GCTACTTACGACCAAATCAAGAAGGCTGTTAAGGCTGCCGCTGAAGGTCCAATGAAGGGTGTTTTGGGTTACACC
GAAGATGCCGTTGTCTCCTCTGATTTCTTGGGTGACACTCACGCTTCCATCTTCGATGCCTCCGCTGGTATCCAA
TTGTCTCCAAAGTTCGTCAAGTTGATTTCCTGGTACGATAACGAATACGGTTACTCCGCCAGAGTTGTTGACTTG
ATCGAATATGTTGCCAAGGCTTAA

>YOR174W 1.55   Hot
ATGTCTGTCCAAGATACTAAAGCGGTAGAGTTTTCTATGGGTCACATCAGATCATCCTCTGTATCCCTGGTAGCA
GAGGCTACTTCAAACACGAATAGTGAAGACAAATTATCTAAGGTCCAACTCTACGAAGATTTGTGCCGGTACGAG
GATACATTGAGTAAGTTGGTGGAATCTGTAGATCGTTTCAAACCAAACCTTGATATTGCCAAGGATTTGATCAGA
ACTGATGAGGCCTTGTTTGAAAACGTCAAACTGTTAGCTGAATATGACAATATCTACCGCAACTTACAGAAGATT
GACAAAGACTCTGAAGAACTAGATTCTAAAACCAGGAAGATATTGGAAATTCTAAATGAATGTCATGACGAGTTA
AAAGCACTACCGATGCTTGAACAAGTAGAGTTCGAGAAAAACACAATTTTACAGCAAAGATCGAAAATCAATTCG
ACAGAGTTGTTGGATTATGCCACCAAACTCTCCAAGTTTACCAAAATACCACCAACGTTCGACAAGGGAGCTGTG
GGACCCAACAATTTTATATGGCCCGCAGAGGATGCCCTACGAAGAGGAATGCTTGCTATGGCCTCATTACATAGT
AAAGAGCTAACTAGAATACCCGGTGAAGAAGTAGAAGAAACAGAAGTACCGACCGTACCGCCATCTCAAAGTGAA
GAACAAAAAGGGCAGATGGCCAAGAAAGAAGGTACACCAAAGACAGACTCGTTTATATTTGATGGTACTGCTAAA

GAAGTTGGTGATGAGGCAGACAATACCAAAGATAAAGAAAAGGAAGAAAATAATGATGATGCCCTAGATCTTGAT
CTTGATCTGTTTGATCCGGACGACTTCTAG

>YOR140W 1.89 Hot
ATGAGTGAAGAGGAAACGGTCTCAGCCCCAGCTCCAGCTTCTACACCGGCCCCGGCTGGAACCGATGTAGGTAGT
GGTGGAGCAGCAGCTGGAATAGCTAATGCTGGTGCTGAAGGCGGCGACGGTGCCGAGGATGTTAAGAAACATGGG
TCGAAGATGCTAGTGGGGCCCAGGCCGCCACAAAATGCGATATTCATCCATAAGCTATACCAGATTCTGGAGGAC
GAATCGCTACACGACTTGATATGGTGGACCCCTTCGGGCTTATCCTTCATGATTAAGCCTGTGGAACGGTTTAGT
AAGGCTTTGGCTACATATTTCAAGCATACTAATATCACGAGCTTTGTAAGACAGTTGAACATTTACGGCTTTCAC
AAGGTGTCGCATGATCATAGCAGCAACGACGCTAATAGTGGCGACGACGCCAATACTAATGATGACAGCAATACT
CACGATGATAATAGTGGCAACAAAAACAGTAGTGGCGACGAAAATACCGGTGGCGGTGTCCAAGAAAAGGAGAAA
AGTAACCCCACTAAAATCTGGGAATTTAAGCATAGTTCTGGTATTTTCAAGAAAGGTGACATTGAGGGACTCAAA
CACATTAAGAGAAGAGCTTCTTCCCGTAATAACTCCTCAATTAACAGTCGAAAGAATTCTAGTAATCAAAACTAC
GATATAGATTCCGGTGCAAGAGTAAGACCCTCTTCGATTCAAGACCCGTCCACCAGTTCCAATTCCTTTGGTAAT
TTCGTTCCGCAAATTCCGGGCGCCAATAACTCTATCCCCGAGTACTTTAATAACTCTCATGTAACTTACGAAAAC
GCAAACCATGCCCCGCTGGAATCCAATAACCCGGAAATGCAAGAGCAAAACAGACCTCCGAACTTTCAAGATGAA
ACACTGAAACATTTGAAAGAAATAAATTTTGATATGGTTAAGATTATAGAGTCTATGCAGCATTTCATATCTTTG
CAACACAGTTTTTGTTCACAGAGTTTCACCTTCAAGAATGTGAGCAAGAAAAAAATCGGAAAATATCGTGAAGGAT
CACCAAAAGCAACTTCAGGCATTCGAAAGCGATATGTTAACTTTCAAACAGCACGTCATGTCGAGGGCCCACCGT
ACAATAGATTCTCTTTGTGCTGTAAATGCTGCCGCTACCGCTGCCTCTGTCGCCCCTGCACCCGCGCCCACATCC
ACATCTGCATATGCACCCAAATCTCAGTATGAGATGATGGTTCCTCCAGGGAACCAGTATGTCCCGCAGAAATCT
TCCTCGACCACGAACATCCCTTCGAGATTCAACACGGCTTCAGTGCCGCCCTCGCAACTTTTGTACAATACCAAC
CGCAGTCGCAACCAACATGTGACTTATGCGAGCGAACCGGCACATGTACCAAACTTTATCAATCAGCCGATACCC
ATCCAACAACTACCTCCACAATATGCAGACACTTTTAGCACTCCACAAATGATGCACAATCCTTTTGCCTCGAAA
AATAACAACAAACCGGGCAACACGAAGAGGACCAATAGTGTTCTCATGGACCCGCTGACTCCCGCAGCGAGTGTT
GGAGTTCAGGGTCCACTAAACTATCCTATCATGAATATTAATCCTTCTGTCCGTGACTACAACAAGCCTGTCCCT
CAAAATATGGCTCCGAGTCCAATATATCCTATTAATGAACCAACAACGAGATTGTATTCTCAACCAAAAAATGAGA
AGTCTCGGGTCTACAAGCTCTTTGCCCAACGATAGAAGAAACAGTCCTCTGAAGCTGACACCGAGATCATCGTTG
AATGAAGATAGTCTGTATCCCAAACCTAGAAACTCTCTCAAATCCAGTATTTCGGGCACGTCTTTATCCTCCTCT
TTTACACTAGTTGCCAACAATCCAGCCCCAATTCGGTACTCTCAGCAGGGATTGCTTCGCTCATTAAACAAAGCT
GCTAATTGTGCTCCAGATTCGGTAACTCCATTAGACTCGTCTGTCTTGACGGGCCCTCCTCCAAAAAACATGGAC
AATCTTCCTGCTGTTTCTTCAAATTTAATAAACTCCCCCATGAACGTCGAGCACAGCTCAAGCCTATCACAAGCA
GAACCTGCACCACAAATAGAACTACCGCAGCCATCTTTGCCCACAACGAGCACCACTAAGAATACTGGCGAGGCT
GACAATAGTAAGAGAAAAGGGAGCGGTGTTTATTCTTTACTGAATCAAGAGGATAGCAGCACGTCCAGTGCTGAT
CCTAAAACTGAAGATAAGGCCGCTCCGGCACTCAAAAAGGTTAAGATGTGA

>YBR106W 2.26 Hot
ATGAATCCTCAAGTCAGTAACATCATCATCATGTTGGTCATGATGCAACTCTCCCGTCGCATTGACATGGAGGAC
CCAACCATCATCATGTACATTAGAATTTTATACTGTTCTTCCATCGGTATCTCTTGGATCATCTACCAAATGGCC
AGAAAGAGAATTGTTGCTAAAAACGACATGACTACCATGAAGTACGTCGAACCTGGTAATGCTATGTCCGGCGAA
GGTGAGAAGCTGCAAGTTACTACCGTCAGAGACTACGATTTGAAGGAAATAGACAGTGCTATCAAGTCTATCTAC
ACTGGTATGGCTATGATGGGTTTCATGCATTTGTACTTGAAATACACCAACCCATTGTTCATGCAATCCATTTCT
CCAGTGAAAAGCGCTTTGGAACACAACGAAGTGAAAATTCACCTCTTCGGTAAGCCTGCAACCGGCGATTTGAAG
AGACCATTCAAGGCTCCATCTTTGTTTGGTGGTATGGGTCAAACTGGTCCAAAGACCGACAAGAAATCTATCGAA
GAAGCTGAAAGAGCCGGTAACGCTGGTGTTAAGGCTGAATGA

>YGR175C 2.51 Hot
ATGTCTGCTGTTAACGTTGCACCTGAATTGATTAATGCCGACAACACAATTACCTACGATGCGATTGTCATCGGT
GCTGGTGTTATCGGTCCATGTGTTGCTACTGGTCTAGCAAGAAAGGGTAAGAAAGTTCTTATCGTAGAACGTGAC
TGGGCTATGCCTGATAGAATTGTTGGTGAATTGATGCAACCAGGTGGTGTTAGAGCATTGAGAAGTCTGGGTATG
ATTCAATCTATCAACAACATCGAAGCATATCCTGTTACCGGTTATACCGTCTTTTTCAACGGCGAACAAGTTGAT
ATTCCATACCCTTACAAGGCCGATATCCCTAAAGTTGAAAAATTGAAGGACTTGGTCAAAGATGGTAATGACAAG
GTCTTGGAAGACAGCACTATTCACATCAAGGATTACGAAGATGATGAAAGAGAAAGGGGTGTTGCTTTTGTTCAT
GGTAGATTCTTGAACAACTTGAGAAACATTACTGCTCAAGAGCCAAATGTTACTAGAGTGCAAGGTAACTGTATT
GAGATATTGAAGGATGAAAAGAATGAGGTTGTTGGTGCCAAGGTTGACATTGATGGCCGTGGCAAGGTGGAATTC
AAAGCCCACTTGACATTTATCTGTGACGGTATCTTTTCACGTTTCAGAAAGGAATTGCACCCAGACCATGTTCCA
ACTGTCGGTTCTTCGTTTGTCGGTATGTCTTTGTTCAATGCTAAGAATCCTGCTCCTATGCACGGTCACGTTATT
CTTGGTAGTGATCATATGCCAATCTTGGTTTACCAAATCAGTCCAGAAGAAACAAGAATCCTTTGTGCTTACAAC
TCTCCAAAGGTCCCAGCTGATATCAAGAGTTGGATGATTAAGGATGTCCAACCTTTCATTCCAAAGAGTCTACGT
CCTTCATTTGATGAAGCCGTCAGCCAAGGTAAATTTAGAGCTATGCCAAACTCCTACTTGCCAGCTAGACAAAAC
GACGTCACTGGTATGTGTGTTATCGGTGACGCTCTAAATATGAGACATCCATTGACTGGTGGTGGTATGACTGTC
GGTTTGCATGATGTTGTCTTGTTGATTAAGAAAATAGGTGACCTAGACTTCAGCGACCGTGAAAAGGTTTTGGAT
GAATTACTAGACTACCATTTCGAAAGAAAGAGTTACGATTCCGTTATTAACGTTTTGTCAGTGGCTTTGTATTCT
TTGTTCGCTGCTGACAGCGATAACTTGAAGGCATTACAAAAAGGTTGTTTCAAATATTTCCAAAGAGGTGGCGAT
TGTGTCAACAAACCCGTTGAATTTCTGTCTGGTGTCTTGCCAAAGCCTTTGCAATTGACCAGGGTTTTCTTCGCT
GTCGCTTTTTACACCATTTACTTGAACATGGAAGAACGTGGTTTCTTGGGATTACCAATGGCTTTATTGGAAGGT
ATTATGATTTTGATCACAGCTATTAGAGTATTCACCCCATTTTTGTTTGGTGAGTTGATTGGTTAA

>YER085C 1.75   Hot
ATGAATTTCAAGGAGCCGCTAGTGAATTTTTTAAAATGCGTCTTGAATAACATAAACTCGGCATTTTCGCATAGG
ATTGATCAATTGCAATTGCAGCTGTTGCGGGAAACAAATATCTTGAGGGTTTTGAACAGGGGAATAGAGAGACTG
TTTAGCGAGAATCCAAGGACCCATTCTGTTTCCCGTATCGTGGTGGAAAAAGATGTGGGCTGCAGAATTGGACGA
TATGTGGAAGGTTCTAAATATGAACTGGTAGAGTCCAGGGCGAAAGAAATACAAATATATTATGAAAGGATGGTT
TTTGAAATAACACAAGAATTGAAAGGGGACAAGCGGGTGTTTGCCTTGATTGCAAATCTTTCCCAAAGATATAGC
GCAGAGAGGAAAGAAATACGAAATGTAAAGGTGCATGGTGGGCGGCCATGTAATGAGAACGAATTCCAGGTGATT
CCGATAAGATTCAAACCTATTAATTTGGAAAGAAGAGCACGACTGATAAGAAAGAAGAAGACTATTAATTAA

>YKL186C 1.89   Hot
ATGAACACCAATAGTAATACTATGGTAATGAATGACGCAAATCAAGCACAAATAACGGCCACATTTACGAAGAAG
ATATTAGCGCATTTGGATGATCCGGACTCCAACAAATTGGCCCAATTCGTACAGCTTTTTAATCCAAACAACTGC
AGAATAATATTTAATGCTACCCCCTTCGCGCAAGCAACAGTTTTTCTGCAAATGTGGCAAAACCAGGTCGTACAA
ACACAACATGCCCTAACAGGAGTAGACTATCACGCTATTCCGGGATCCGGCACGTTGATATGCAACGTCAATTGC
AAAGTCAGATTCGACGAAAGCGGCAGAGACAAGATGGGGCAAGACGCGACTGTTCCCATTCAACCAAATAACACT
GGGAACAGAAATCGACCCAACGATATGAACAAGCCAAGACCTCTATGGGGTCCATATTTTGGCATTTCCCTGCAG
CTGATCATCGACGACCGCATATTTAGAAATGATTTTAATGGTGTAATATCGGGGTTTAACTATAACATGGTTTAC
AAACCCGAGGATTCTCTGCTAAAAATTTAG

>YDR229W 1.64   Hot
ATGCCTGACAATAATACGGAGCAATTGCAGGGATCTCCTTCAAGTGACCAGAGACTGAGGGTTGACTGGGACAAC
GGTAACCATTTTGACGTCTCTCCCGACAGATACGCGCCTCATCTATCTGAATTTTACCCAATTGTAAATAGTAAA
AGACCAGTGGCTAGCAGTGCTGGCTCAGAAAATAACGACCATCTGGACGACATGAACCATTTACGTTCATCCAAA
GTGTATTCAAAGGCCCGTCGGGCATCGAGCATAACTTCTGGTACGAGCACAATAAACGACTTGCAAACGCTAATC
ACAAAGAGAGATGTGAAAGAGACCCAGGAAGCCCTATCGACATTACTCAGGAACTCGAATGCTTATTCGGATTCG
TTATTAAAGACATCTCAGAATGGGGCAGAAATTGCCCATTCCTTAGAAAACATTGCCAAGTTGAAGGGTTGCAAT
GATGAAACGGCGGAGAAACTATTGAGTGCCAGTGGGTTATTCTACCTTTTATCTAATCATCAGCTTATAATGTCA
AAGTATTTCAATGACTTGTTGGGAGATAATTTAATTGATGATATTGATGAATTCGAATTACAGACCAAGATAATG
GAGAACAAGTTCAAAGCACAAAGCAAAGAGCAAAGTTTAAAATTGAAATTGCAGGAAAGACATAACTTCGATATC
TCGAAAAGAAAAATAAGGAATTTAATCTCATACCGAGAGAGCCTTTCTAGCTTGCAAGCAAGGTTAGATCAACTG
GAGACACTAAAGCACGATTTTTACATGGATTCATACGAACTTGTGGAAAATACATGCAACAAAGTACTCAGCAAA
GTTGCCACGGTATCTAGGGCACAAGTGGAAATTTCTGAAAATATTGCAAGGAAAGGTTGGTCTGGAGGCGGGCTA
GACGAACTACTTTGTGATGCAGACGACCCCTTCAGCAAAAAAGCAGATGGGCCATACGGGACCATCGGTGGTGAT
GGAGAGACTGCTGGAGAAGCATACAACAGCGATGAGGAAACGGGTGGGAATGACGTGGTGCTCAATGAACTACTA
GAAGGAACCAGCCAACCTTCCACATCAAAAACATCTTTACCCAAATCCAAGGGGTCCTCAACGGTATCCACACCG
AATCATTCACAGTCAAGTTCAAACAAGGACGGGGTACGAAACAATGGCGGCGGCAAAAATGGCGAAGATGAGGAT
ACAGACAATTTGATGGGAACAGAAAATTCATTTTCTTTACCGCCTACGAGAAACTCTGCCGAGGAAACCACACAA
ACGTTTAAACAATTGTCCATTAAAGAAGATAATGACAATCACAGCAGCGATACGGACGGCATGCAAGACCAGTCA
AGTAATATATAA

>YDR232W 1.82   Hot
ATGCAACGCTCCATTTTTGCGAGGTTCGGTAACTCCTCTGCCGCTGTTTCCACACTGAATAGGCTGTCCACGACA
GCCGCACCACATGCGAAAAATGGCTATGCCACCGCTACTGGTGCTGGTGCCGCTGCTGCCACTGCCACAGCGTCA
TCAACACATGCAGCAGCAGCAGCAGCCGCTGCTGCCAACCATTCCACCCAGGAGTCGGGTTTCGATTACGAAGGC
CTGATAGATTCCGAACTGCAGAAGAAAAGACTTGACAAATCGTACAGATATTTCAACAATATCAACCGATTGGCC
AAGGAGTTCCCCCTAGCTCATCGCCAGAGAGAGGCGGACAAGGTCACCGTTTGGTGTTCCAACGACTATTTAGCA
CTTTCCAAGCACCCTGAGGTATTGGACGCCATGCATAAAACTATCGACAAGTATGGTTGTGGTGCCGGTGGTACA
AGAAACATTGCTGGCCATAACATCCCCACTTTGAATCTGGAAGCCGAATTGGCCACTTTACACAAGAAGGAAGGT
GCCTTAGTTTTTTCGTCATGTTACGTAGCCAACGATGCCGTCTTATCCCTACTGGGTCAAAAGATGAAGGACTTG
GTGATTTTCTCCGACGAACTCAACCATGCGTCCATGATTGTCGGTATTAAGCATGCTAACGTAAAAAAACACATT
TTCAAACATAATGACTTGAACGAATTGGAACAACTGCTCCAGTCATACCCCAAATCCGTTCCTAAACTAATTGCT
TTCGAATCAGTATATTCTATGGCCGGTTCAGTGGCCGACATAGAAAAAATTTGCGACTTGGCCGACAAATACGGT
GCTTTGACCTTCTTGGATGAAGTACATGCGGTCGGCCTGTACGGCCCTCACGGTGCAGGTGTTGCAGAACATTGT
GATTTTGAAAGTCACCGTGCAAGTGGTATTGCTACCCCAAAGACCAATGACAAGGGCGGCGCGAAGACTGTGATG
GACCGTGTCGACATGATCACCGGCACTTTAGGTAAGTCTTTCGGTAGCGTAGGTGGCTACGTCGCAGCCTCTAGG
AAATTGATCGATTGGTTCAGATCGTTTGCACCTGGTTTCATTTTCACCACGACTTTACCACCTTCAGTTATGGCA
GGCGCTACCGCAGCAATTAGATACCAACGTTGCCACATCGACCTAAGAACCTCGCAACAGAAACATACCATGTAC
GTAAAGAAAGCTTTCCATGAGTTGGGCATTCCAGTTATTCCAAATCCTTCTCATATCGTCCCAGTGTTGATTGGT
AATGCTGATTTGGCTAAGCAAGCTTCTGACATCTTAATCAATAAGCATCAAATCTACGTACAAGCTATCAACTTC
CCTACGGTTGCTCGCGGTACCGAAAGATTGAGAATTACCCCAACGCCAGGTCACACCAACGATTTATCTGACATC
TTAATCAATGCAGTTGATGATGTGTTCAATGAGCTACAGTTACCACGTGTCAGAGACTGGGAAAGCCAAGGTGGC
TTATTGGGTGTTGGAGAGAGCGGATTTGTGGAAGAGTCTAACTTATGGACATCAAGCCAACTATCTTTAACTAAT
GACGACTTGAACCCTAATGTTAGAGACCCCATCGTTAAACAACTAGAGGTTTCTAGTGGTATCAAGCAGTAA

>YJR060W 1.71   Hot

ATGAACTCTCTGGCAAATAATAATAAGCTTTCTACTGAGGATGAGGAAATCCATTCGGCAAGAAAAAGAGGCTAT
AACGAGGAGCAGAACTACAGCGAGGCCAGGAAAAAGCAAAGAGATCAAGGCTTGTTGTCACAAGAGAGCAATGAC
GGAAATATTGACTCTGCTCTGTTGAGCGAAGGGGCTACGCTAAAAGGGACTCAAAGTCAGTATGAAAGCGGACTG
ACGTCTAACAAAGATGAGAAAGGAAGTGATGACGAAGATGCGTCAGTGGCTGAGGCTGCTGTTGCCGCTACCGTC
AATTATACAGACTTAATCCAGGGCCAGGAAGATAGTTCTGATGCCCACACATCTAATCAAACGAACGCGAATGGT
GAGCACAAGGATTCCCTTAATGGGGAAAGAGCCATAACCCCATCAAATGAAGGAGTCAAGCCAAACACCTCCCTG
GAGGGAATGACGTCTTCACCAATGGAATCTACACAGCAATCTAAAAACGACATGCTCATACCATTGGCAGAACAC
GATCGTGGGCCAGAACACCAACAGGATGATGAAGACAATGATGACGCTGACATTGATCTCAAAAAGGATATAAGC
ATGCAGCCGGGTCGTCGTGGAAGAAAACCTACTACTTTGGCCACAACAGACGAGTGGAAAAAGCAAAGAAAAGAC
TCCCATAAAGAAGTCGAAAGGCGTCGCCGCGAAAACATCAACACTGCAATCAACGTTTTAAGCGACCTCCTGCCC
GTGAGAGAATCAAGTAAGGCAGCAATATTGGCGTGTGCCGCTGAATACATTCAAAAATTGAAAGAAACCGATGAA
GCAAACATCGAAAAGTGGACGTTACAAAAATTGCTTAGCGAGCAAAACGCATCGCAATTAGCCAGTGCAAATGAG
AAACTGCAGGAAGAACTGGGAAATGCTTACAAAGAAATCGAGTACATGAAACGCGTTTTAAGGAAGGAGGGAATA
GAATACGAGGATATGCACACTCACAAGAAACAAGAGAACGAAAGAAAAAGCACTAGGAGCGATAATCCACATGAG
GCTTGA

>YDL111C 1.64   Hot
ATGTCTCTTTCGGTCGCCGAAAAGTCGTACCTATACGATTCACTAGCAAGCACGCCTTCAATTAGGCCCGATGGA
AGACTACCTCATCAATTCAGACCTATAGAAATATTTACTGACTTCCTACCAAGTTCCAATGGATCGTCCAGAATT
ATAGCTAGTGATGGAAGTGAATGTATCGTGAGCATTAAGTCCAAAGTTGTGGATCATCATGTGGAGAATGAACTA
CTTCAGGTCGATGTAGATATCGCAGGCCAAAGAGATGACGCGCTGGTAGTAGAGACAATCACTTCTTTACTGAAC
AAGGTTTTGAAGTCTGGTAGCGGAGTAGATTCCTCAAAGTTGCAATTGACAAAAAAGTACAGCTTCAAAATATTT
GTCGATGTTCTGGTCATTTCATCACATTCACACCCAGTTTCTTTAATATCCTTTGCTATTTATTCAGCGTTGAAC
TCCACATACCTACCGAAACTTATTTCAGCTTTTGACGACTTAGAGGTGGAAGAACTTCCTACGTTTCATGATTAC
GACATGGTTAAGCTTGACATTAATCCGCCTTTAGTGTTTATATTGGCCGTTGTAGGCAACAATATGCTATTGGAC
CCTGCAGCCAACGAAAGCGAAGTGGCCAATAATGGCCTAATCATTTCCTGGTCTAATGGTAAGATTACATCACCT
ATCAGATCAGTAGCATTGAATGATTCAAACGTTAAAAGCTTTAAACCTCATTTGTTGAAGCAAGGTCTTGCAATG
GTGGAGAAATACGCCCCCGACGTAGTACGATCACTGGAGAATTTATAA

>YCR068W 1.52   Hot
ATGTTGCATAAAAGCCCTTCAAGAAAGAGATTTGCTTCTCCTTTGCATCTAGGATGCATTCTAACGCTTACAGTG
CTCTGCCTTATTGCTTACTACTTTGCTTTACCGGATTATTTATCGGTAGGGAAGAGCTCATCCAGGGGTGCCATG
GATCAAAAATCTGATGGAACCTTTAGACTTAAGTCTATCTACAGGCATGGCGTCGGAGCAAACCATCGGCTACAC
CAGAGGCTGGAGGTCACTCCAGAAGTTATTTCTGCCGCTGGAATGCTGTATCAAGAAACCACAACTCAAGGACAG
GATTTCGAAGACCAAGAACCCCTATGGACTACTAATGCCGAATATGCCACCACTAACCCATTCGATTTTGAGTTT
GAGCTACGAAGGATGCCGCTATTGATGAAGCGGATGAAGGAAAGAGATCCTGAATTCATAGAGTCGTACATTTAC
GGAGAAACATACATGACGGAGGAGGAAGAACACGCCATGTGGATAGATGATGACATTGTGGCACCCAATATCACA
GATAGAGGTACAGTAGTGTCGTTAGCATTAATGTCGTCTAATGCTTACGTGAGAATACCGCAAACGGGAGACTGG
CGTAACGTCACGGAGCCGTGGAATGAAACAGAGCCGGAAGATTTTGGTTGGGATGGTGATGGTATACGTGGTCAC
GTCTTTTACAATGAAGTGGAGAATATCGTGGTACTCTCGATAAAGGGAACCAGCGCTCAGGGTTTGCCAGGGTCT
GGCGAAGATGAGACAACTGGAAACGACAAGATCAACGATAATTTGTTATTTTCGTGTTGTTGTGCAAGAGTGAGC
TACCTGTGGACTACCGTGTGCGATTGCTATGTGAAGTCGTATATATGCGATGAGTCTTGCCTGGAAAAGGAACTG
AGACGTAAGGATAGATTTTACTCTGCAGTCGTTGACATATACAAAGGCGTCCTCAAAGAATACCCCGATGCGGCC
ATATGGGTCACAGGCCACTCACTGGGAGGCGCATTGGCCAGTTTACTGGGCCGCACTTTTGGATTGCCTGCGGTC
GCATTCGAGTCCCCTGGAGAGCTACTACCTTCAAAAAGACTTCACTTACCATTCCCACCGGGACTTCCCTCATAC
ATGGAGGGTATCTGGCACTTCGGTCACAACGCAGACCCAATCTTCATGGGTACATGCAACGGAGCTAGTTCAAGT
TGCTCACTGGTGGGCTACGCTATGGAAACCGCGTGCCACACTGGTAGAGTCTGTGTCTACGATGTGGTCAACGAC
AAGGGCTGGAGTGTAAACATGTTCAACCACAGAATCCACAAAGTCATTGACGAAGTTCTCCTTGGATACGAGCAG
GCTGCCAAGTGCGTTGAACCAGAGCCCTGCGTAGATTGCTACAACTGGAAGTTTATTCCAAGCAGAGACTGGGAA
TCCTCATCGAGGCTCATTACCAAGACTAAAAGCCACGCTGCACCAACCACGACGACTCGCACCACTGCTACTACT
ACCTCTTCATCTACCTGTGTAGGCCGCAATTGGCTTGGCTTCTGCACCAAATACGAGTTGTAA

>YIL062C 1.54   Hot
ATGGAAGCCGATTGGAGGAGAATTGACATCGATGCATTTGATCCAGAGAGTGGCAGACTAACCGCTGCCGATCTG
GTACCACCATACGAAACTACTGTCACATTACAAGAATTACAACCTCGAATGAATCAATTGCGCTCGCTTGCCACA
AGTGGTGACTCTTTGGGAGCCGTTCAATTACTCACAACCGATCCTCCATACAGTGCAGATGCTCCAACAAAGGAG
CAATATTTTAAGAGCGTCCTTGAAGCATTGACACAAGTCAGGCAAGCCGATATTGGTAATGTAATCAAAAATTTG
AGTGATTCTCAGAGGGACGTGCTGGTAAAGTATCTCTACAAAGGAATGTCCGTACCTCAGGGCCAGAAACAAGGG
GGTGTCTTGCTTGCGTGGCTGGAAAGAATTACTCAAGTCAGTGGTGTCACACCTATCGTTCATTATATATCGGAT
AGAAGAACTGTATGA

>YIL099W 2.56   Hot
ATGGCAAGACAAAAGATGTTTTATAACAAATTACTCGGCATGCTCAGCGTAGGATTCGGGTTTGCTTGGGCGCTC
GAGAACATTACTATATACGAATTTGACTTTGGCAAGGGCATTCTCGATCAAAGCTACGGCGGTGTATTTTCAAAC
AACGGCCCTTCGCAAGTGCAGCTGCGGGATGCAGTCTTGATGAATGGGACAGTGGTATACGATTCAAACGGCGCT
TGGGACAGTAGTGCGCTGGAGGAATGGCTCCAGGGACAGAAAAAAGTTTCCATCGAAAAAATATTTGAAAATATT
GGGCCCAGCGCCGTGTATCCGTCTATTTCGCCTGGGGTCGTGATTGCGTCACCATCGCAAACGCATCCAGACTAC

TTCTACCAATGGATAAGGGACAGCGCGTTGACGATAAACAGTATTGTCTCTCATTCTGCGGGCCCGGCAATAGAG
ACGTTATTGCAGTACCTGAACGTTTCATTCCACTTGCAAAGAAGCAACAACACATTGGGCGCTGGCATTGGTTAC
ACTAACGATACAGTGGCTTTGGGAGACCCTAAGTGGAACGTCGACAACACGGCTTTCACGGAAGATTGGGGTCGT
CCTCAAAACGATGGGCCTGCTCTTCGAAGCATTGCCATCTTAAAAATCATCGACTACATCAAGCAATCTGGCACT
GATCTGGGGGCCAAGTACCCATTCCAGTCCACCGCAGATATCTTTGATGATATTGTACGTTGGGACCTGAGGTTC
ATTATTGACCACTGGAATTCTTCCGGATTTGATCTATGGGAGGAAGTCAATGGCATGCATTTCTTTACTTTACTG
GTACAACTGTCTGCAGTGGACAAGTCGCTGTCGTATTTTAACGCCTCAGAACGGTCGTCTCCCTTTGTTGAAGAA
TTGCGTCAGACACGCCGGGACATCTCCAAGTTTTTAGTGGACCCTGCGAATGGGTTTATCAACGGCAAGTACAAT
TATATTGTTGGGACACCCATGATTGCCGACACATTGAGATCCGGACTGGACATATCCACTTTATTAGCTGCGAAC
ACCGTCCACGATGCGCCATCTGCTTCCCATCTTCCGTTCGATATCAATGACCCTGCCGTCCTGAACACGTTGCAC
CATTTGATGTTGCACATGCGTTCGATATACCCCATCAACGATAGCTCCAAAAATGCAACGGGTATTGCCCTGGGC
CGGTATCCTGAGGACGTATATGATGGATATGGCTTTGGCGAGGGAAATCCCTGGGTCCTGGCCACGTGTACCGCT
TCAACAACGCTTTATCAGCTCATTTACAGACACATCTCTGAGCAGCATGACTTGGTTGTCCCAATGAACAACGAT
TGTTCGAACGCATTTTGGAGCGAGCTGGTATTCTCCAACCTCACGACTTTGGGAAATGACGAAGGCTATTTGATT
TTGGAGTTCAATACACCTGCCTTCAATCAAACCATACAAAAAATCTTCCAACTAGCTGATTCATTCTTGGTCAAG
CTGAAAGCTCACGTGGGAACAGACGGGGAACTAAGTGAACAATTTAACAAATACACAGGGTTTATGCAGGGTGCC
CAACACCTTACCTGGTCCTATACTTCATTCTGGGATGCCTATCAAATAAGACAAGAAGTTTTACAGAGTTTGTAG

>YBR213W 1.55   Hot
ATGGTCAAATCGCTACAGCTAGCCCATCAATTAAAAGACAAGAAAATACTACTAATCGGAGGCGGAGAAGTCGGC
TTGACAAGGTTATATAAGCTGATACCTACAGGTTGCAAGCTGACTTTAGTATCTCCTGACCTACACAAATCCATT
ATTCCGAAGTTTGGAAAATTCATCCAGAACGAGGATCAGCCCGACTACAGAGAAGACGCTAAACGCTTTATCAAT
CCGAACTGGGACCCCACGAAGAATGAAATTTACGAGTACATCCGCAGTGACTTCAAAGACGAATACCTCGACCTA
GAAGACGAGAACGACGCGTGGTACATAATAATGACCTGTATACCCGACCACCCTGAAAGCGCAAGAATATATCAC
CTTTGTAAGGAAAGATTCGGTAAGCAGCAGCTGGTCAATGTGGCGGACAAACCTGATCTCTGTGACTTTTATTTC
GGCGCCAACCTGGAGATAGGAGACCGTCTACAGATTCTGATCTCTACCAACGGCCTTTCTCCGCGCTTTGGCGCT
CTAGTAAGAGATGAGATCCGTAACTTATTCACACAAATGGGGGATCTGGCGCTGGAGGACGCTGTCGTCAAACTA
GGTGAGCTGAGAAGAGGAATTAGGCTGCTGGCACCAGACGACAAGGATGTCAAGTACCGCATGGATTGGGCCAGA
CGCTGCACAGACCTCTTCGGCATTCAGCACTGCCACAACATCGACGTAAAACGTCTGCTGGATCTTTTCAAGGTC
ATGTTTCAAGAACAGAACTGTTCCTTGCAGTTCCCTCCCAGAGAACGGTTGCTTAGCGAGTACTGCTCGTCTTGA

>YLR101C 1.67   Hot
ATGCCTTTTTTGCTTCACGTGTATATATATCAGTTGATATGCGTTTTCTATACAGACATACATGTATATACACAC
ATATATATATATACATATGTACGCAGAGATATATTAAATGGGGGTTCTAGTTTCAACAATTTGATCTTTCAGTTT
CTCGTCCCATTCCAGTTTCTTCTTCTGTATATAAGCGAAGACATCAGACATTCCAGTAGGGTCTATTTCCTGCGT
CTTGATATATGGCATACCATCCCTAGAGGTAGCAGAACCGTATTTTACGTCTTGTTTCTCAAAGTTTGGATTGGC
CAATCTAGTTACGTATACTGGGGCATTGGCAGCTTTATAACCATCAATATTGTGCCATGGAGACCCCAACAGCCT
GGCCAAATAGAACAAGCATAG

>YCR076C 1.5Hot
ATGATTGAGAATAAGGTTGAACTGGTAGCAGAACTAGTGTTGGAGTCCATCGGTAAAACTGAGGTGGTGTCACGC
CATACAGAGGGAACCAAATCATGCCAAGTGAGTTTTCGCATTAAAGATTCTCCCAGTGAGAAGGGTTCAACATCG
TTTCTCTCTGAGCTAGTGGTGATTCAGACTCTGGATGACAATGACAAGTATACTGTTGTTATTCGTCATGGTACG
TCGATCACCATGGCATGTGTGGTGGGTTACAGCGATTTTAAATTGCCCACAGAATTGAAATGGCCACTAGAAAGA
GAAAGCTTACCTGTGGAGCCAGACCTGAAACCTATAATGACACAACTTAAAAGACAAACTGCAGGTAGTGCGGAC
ATGCCAAAATTTGACGATGAGTACCAGGCACAAGCAAGACAAAACCAGGGAACAGCACCGTTGAACCCATATCCC
GGACTTACGGTAACTGAACCGAGCTTCGCTAACCCAGCAGGAGGGTATGCGGATGGTGATCTGTATCCTGTTGGC
ACAAGCCATCCGGATTGGTCTGGAGGGCTTCCCAATCCTCTGGGGAATCCAAGTAGCCAAGGCGGTATGATTTTC
GATCCAAATAGAAGACCTGCCCCAAGACGGGAAGATATGCCGCCAGGATGGATGCCTGGATCCAAGTACGACGAA
CCATTTGGTCCTGGATCTGGCGGATTTGGAGGTTCAGGATCCGGCGGATTTGGAGGGTCAGGATCTGGCTTTATT
TAA

>YDR125C 1.77   Hot
ATGCTTTTAATAAAGAGGTACTTGATGGACCCTGAAAGTTTAAGGAGGCAGATAATGAATGTATATAAATGTTAC
ATGTGGAAGAGAGCGTTCCACAGTAATAGAAGCCTACTGGAAGTTAAACGTCGTGAAAAGAGTCTCCAGAGAAAA
ATTTTGGAAAGGATTCTTAGACCCAAGGAGGAAAATGCTGTCAAAAAATCTGGATTCAAGCTATGGTCAAGCCAT
TTGAATAACCCACACAAAACTTATATGCGGCTGGAGGAATTACAGAGACGTATTATGGAAGAGGTACACGTTGAA
GGTATTAAAAAGAATGATAAGTTATTCAACGAAATAAATCAATGGCATTTCCAAAATGAAAACACAAGTACTGTT
CGAACACCAACATTGCTGATACATGGCTATGCCGCATCTTCAATGTCTTTTTTCAGAAATTATCCAGGTCTGTCC
AAGCACATCCGAAATTTATACTCAATTGACATGCCAGCGAGCGGATTGTCTTCAGTACCGTCTTTAGAGATCAAT
ACAACAACACCTCTGCCGTTAGATATCAAATTTATTGGAGAAAATAAATTTAAGGTTCCATATACAATAAATGCA
AATCACAATAAATTTGTGATTCAAATGTACGAAGATTTCTATCTCGACAGAATCGAGCAATGGCGCATTGATAAC
AAATTGGGCAAAATGAATGTTGTGGGGCATTCTTTTGGCGGATATTTATCTTTTAAATATGCTGTTAAATATCCA
AACTCGGTAAACAAACTTTGCTTGGTATCCCCACTAGGAGTGGAAAGGAATATCTGGTCTGTAAATAATAATTTC
CACTCCAACACTCTTTATACTATCGATTTTAAAAATCCAAATTCCAAGTTTTACTCCAAAAGAAATATGATTCCA
AAATACTTATTTGAACAACAATTTCACATTTTGAGAATGATGGGACCATTAGGAGCTAAGTTATGTTGGAATTAC
ATAATGGCGGCATATAGCCGAGTCCCATCGTTGGCATACAAAGAGTACATATTTGAACTGTTTTACGGTAAGGGA

GGTATACCGGAGGTTACGACAGATATTTTCAAAGCGTTATTTTCAAGATGTATCCTAGCAAAGGACCCCTTGATG
GATTCTCTGCAATATTTAAATGTTAAGAAATTATTGATAGTATATGGGCAATATGATTGGATGAATAAAAAAGCC
GGTATGTTCATGGTCAAAGAGTTGAACAATTTAAAGAACTGTCTAGAAGGAGCAAGTTATTTAGAAATACCTTCT
TCCGGTCACAATCTTTTTTTAGATAACCCAGAATCATTCAACCAATCTATAGTGTCCTTCTTATCAGACGAAACT
AAATCGCCTTGA

>YPL049C 1.82   Hot
ATGGCCGTATCAGCCCGTTTGAGAACGACTGCCGAGGATACATCCATTGCTAAATCAACACAAGATCCAATTGGT
GATACAGAAATCAGTGTAGCAAATGCTAAGGGCAGCAGCGATAGCAACATTAAGAATTCGCCAGGCGGAAACAGC
GTTGGTCAGGAGTCGGAGCTAGAGCATGTCCCTGAGGAGGATGACTCTGGTGACAAGGAAGCAGATCATGAGGAT
TCTGAGACAGCCACTGCGAAGAAGAGGAAAGCCCAACCATTGAAGAATCCGAAGAAATCATTGAAGAGGGGCAGA
GTCCCGGCGCCTTTGAATTTATCGGATAGCAACACTAATACACACGGTGGTAATATTAAGGACGGCAACTTGGCT
TCGTCTAACTCTGCACATTTTCCTCCTGTTGCTAATCAAAACGTGAAAAGCGCGCCCGCACAAGTTACTCAGCAT
TCCAAGTTCCAGCCCCGTGTCCAATACTTGGGAAAGGCCAGTTCTAGACAATCTATACAAGTGAATAATAGCAGC
AATAGTTATGGGAAACCACACATGCCCTCGGCGGGCATCATGAGCGCCATGAACCCTTACATGCCCATGAATCGC
TACATAATGTCACCATATTATAATCCGTACGGTATCCCTCCACCTCACATGCTGAACAAGCCCATAATGACACCC
TACGTGTCGTATCCATATCCAATGGGACCGCGGACCTCCATTCCTTATGCTATGCAAGGTGGCAACGCTAGGCCC
TACGAAGAAAATGAGTATAGTGCTAGCAATTACAGAAACAAGAGGGTTAACGACTCATATGATTCGCCTTTGAGT
GGCACCGCTTCCACTGGGAAAACCAGACGATCCGAGGAAGGCTCAAGAAATTCTAGCGTAGGATCAAGTGCGAAT
GCCGGTCCTACGCAACAGCGCGCGGATCTACGCCCAGCAGATATGATACCTGCTGAAGAATACCACTTTGAACGG
GATGCATTACTCTCGGCCAACACGAAAGCCAGAAGCGCAAGCACAAGCACAAGTACAAGCACAAGCACAAACCGG
GACAGGAGTTCATGGCATGAGGCAGAACCCAATAAAGACGAAGAAGAGGGTACGGACTTGGCCATCGAGGACGGA
GCGGTCCCCACTCCCACTTTTACTACGTTCCAGCGGACCTCGCAGCCGCAGCAGCAATCACCTAGTCTTCTTCAA
GGCGAAATCCGACTCTCATCGCATATTTTTGCCTTCGAGTTCCCCCTGAGCTCTAGCAATGTAGACAAGAAAATG
TTTATGAGCATATGTAATAAAGTATGGAATGAATCAAAGGAGCTGACAAAAAAATCATCATCACATCACAGAACC
GGAAAATAG

>YHR181W 1.85   Hot
ATGCTGTTAGAATTGATTTCTTATGCAGGAACCGTTTCAGGGTTTCTTTTTTTAACACTTTCAATTGCATCGGGT
TTATACTACATTAGTGAACTTGTTGAAGAACACACAGAACCCACAAGACGGTTTCTCACAAGGGCTATTTATGGT
ATAATATTAATATTAATTCTGCTACTGTTACTAGACGGTTTCCCGTTCAAACTTACACTTTTTTCCATTGCATGT
TACATAGTATATTACCAAAACTTAAAAAGTTTCCCATTTATTTCGTTAACCAGCCCAACTTTCCTATTAAGCTGC
GTATGTGTGGTCTTGAATCACTACTTCTGGTTCAAATATTTTAATGATACGGAAGTTCCCCCACAGTTCAAATTT
GATCCAAATTATATACCACGCAGACGTGCTAGTTTCGCTGAAGTAGCCTCATTTTTTGGTATATGCGTTTGGTTC
ATACCATTTGCCTTATTTGTTTCTCTGTCAGCTGGTGATTATGTGCTACCAACGACTAGTGAGCAACATATGGCT
AAGAAAAATGATGACATCACAACGAACAACCAGCCAAAATTTCGTAAAAGGGCTGTTGGGTTAGCTCGTGTAGTC
ATCAACTCGGTAAGGAAGTACATCTACTCTCTAGCCCGTGTATTCGGTTATGAGATAGAACCTGACTTTGATAGA
TTGGCTGTTTAG

>YDR037W 1.75   Hot
ATGTCTCAACAAGATAATGTCAAAGCCGCCGCTGAAGGTGTTGCTAACCTACATCTCGACGAAGCTACCGGGGAA
ATGGTCTCCAAGTCTGAATTGAAGAAGCGTATCAAGCAAAGACAAGTCGAAGCTAAAAAGGCCGCCAAAAAGGCT
GCCGCTCAACCAAAACCGGCTTCCAAAAAAAAAACAGATTTGTTCGCTGACCTGGATCCATCGCAATATTTCGAA
ACAAGATCTCGCCAAATTCAAGAATTGAGAAAGACTCACGAACCAAATCCATACCCACACAAGTTTCACGTTTCT
ATATCCAATCCTGAGTTCTTGGCCAAATATGCGCATTTGAAAAAAGGTGAAACCTTACCTGAAGAGAAGGTTTCA
ATTGCTGGTAGAATTCATGCCAAAAGAGAATCTGGCTCCAAATTGAAATTCTATGTTCTTCACGGTGATGGTGTT
GAAGTTCAATTGATGTCCCAATTGCAGGACTACTGCGACCCAGACTCTTACGAAAAGGATCACGACCTTTTGAAA
AGGGGTGATATCGTTGGTGTCGAGGGTTACGTCGGAAGAACTCAACCAAAGAAAGGTGGTGAAGGTGAAGTTTCC
GTCTTCGTTAGCAGAGTGCAATTATTGACACCATGTTTGCACATGTTACCTGCCGACCACTTTGGTTTCAAAGAC
CAGGAAACCAGATACAGAAAGCGTTATTTGGATTTGATCATGAACAAAGACGCCAGAAACCGTTTTATTACCCGT
TCTGAAATTATCCGTTACATCAGAAGATTTTTGGACCAAAGAAAGTTTATTGAAGTAGAAACTCCAATGATGAAC
GTTATTGCTGGTGGTGCTACCGCTAAGCCATTTATTACCCACCATAATGACCTTGATATGGACATGTACATGAGA
ATTGCTCCAGAATTGTTCTTGAAACAATTGGTTGTCGGTGGTTTGGATCGTGTTTACGAAATTGGTAGACAATTC
AGAAATGAAGGTATCGATATGACACATAATCCAGAATTCACCACTTGTGAGTTTTATCAAGCCTACGCTGATGTT
TATGATTTGATGGATATGACTGAATTGATGTTTTCAGAAATGGTCAAGGAGATCACTGGTTCTTATATTATCAAA
TACCATCCGGACCCTGCTGATCCAGCCAAAGAACTAGAATTGAACTTTTCTAGACCATGGAAGAGAATCAACATG
ATTGAAGAATTAGAAAAGGTATTCAACGTTAAGTTCCCATCTGGTGATCAATTACATACAGCTGAGACTGGTGAA
TTTTTGAAAAAGATTCTTGTCGACAACAAATTAGAATGTCCACCTCCACTAACTAATGCTCGTATGTTAGATAAG
CTTGTCGGTGAATTAGAAGATACATGTATCAACCCAACTTTCATTTTTGGCCACCCTCAAATGATGTCTCCATTA
GCCAAGTACTCAAGAGATCAACCGGGTCTATGTGAGCGTTTCGAGGTCTTTGTAGCTACAAAGGAAATTTGTAAT
GCCTACACTGAATTGAACGATCCATTTGACCAAAGGGCACGTTTCGAAGAACAAGCTAGACAAAAGGATCAAGGT
GATGACGAAGCTCAATTAGTCGATGAAACCTTCTGTAATGCTCTAGAATACGGTTTACCACCAACTGGTGGTTGG
GGTTGTGGTATTGATAGACTGGCCATGTTCTTGACCGACTCCAACACCATTAGAGAAGTCTTATTGTTCCCAACT
TTGAAGCCTGATGTTTTGAGAGAGGAAGTCAAAAAGGAAGAAGAAAATTAA

>YNL194C 1.52   Hot

ATGTCCTACAAAAAGTTTGTGTACTTTATTAACCTTTTCTTTCTACTAGGGGCTACATTGTTGACTTTTTTTCTG
ATACTAGCAGGTGGCAGGACCACAGGAGTGTTGAAGAATTTTTATTGGTTTCAGGCATCCACTTCGGGCTTTAAC
TCGGCGCCCTCCGTAACCAGATGGTACAATTATAACTGGTGCGGCTGGGAAAGCCGCGGAATAGCAGTCAACTGC
TCTTCTAAGATGGCAGCTCAGCCGTTTTCACCAAGGGACAACTTTGGCAGTTCCCCCCTCATGCCCTCAACTTTT
TTAAACAACAGAAATGCCTACTATTATCTTTCAAGAGTGGGCTGGGCAATGCTTCTAATCGGGCTTTTCTTTTTG
TTAATCACGTTAGTTTCGGTTATTGCCAGTTTAATCAGATACAATAGACGTACCGCCGCTCTGGCAACTGCAATG
AGCTGGATCACGCTTTTCTTCATAACCCTATCCGCTTGTCTGTACACAGGATGTTACGCTAAGGCAGTAAAGGCG
TTTCACCATGAGAATCGTGATGCTAGACTAGGTCCCAAGAATTTTGGGCTTATTTGGACCACAGTATTTTTGTTA
ATTGTAAACGCCATTTGCTGTACTATCATGGTGGCTACTCATAAAAGAAACGAGTACATCTATGACCGCAGTTTC
GCTTCTACCAAGACAGTGGACTCACAAACTCCAACTCCAGTTCCAACAAATGGAGGAATACCTTCCTCGGTGCCT
GTAACTGAGGTCCAACAATCTCAATCCCACCAGAATCATAGGTTCTTTAAGAAATTGAGAACGAAAAAGAGAACT
GTGACTAGCGCAGGCGATGAACCAGACCGGGTTCAAGAGGAACGCGTCTATACTGAACAGAATGTTCCTGTTGTA
TCATAG

>YOR348C 2.09   Hot
ATGGTAAATATACTGCCCTTCCACAAGAACAATAGACACAGCGCGGGAGTCGTCACCTGCGCGGACGATGTTAGC
GGCGACGGTAGCGGCGGCGACACCAAGAAGGAGGAGGATGTTGTCCAGGTAACGGAATCACCATCGTCCGGGTCG
CGCAATAATCATCGCAGCGACAATGAAAAAGATGACGCCATCCGTATGGAGAAAATATCTAAGAACCAGTCCGCG
TCGTCCAACGGCACCATCCGCGAGGATTTGATTATGGACGTGGACTTGGAGAAATCGCCCTCCGTCGATGGCGAT
AGCGAGCCGCACAAACTAAAACAAGGTTTGCAGTCGCGCCATGTGCAACTGATCGCGCTGGGCGGCGCCATCGGT
ACCGGTTTGCTGGTGGGGACTTCATCCACGCTTCATACGTGCGGTCCCGCGGGGCTGTTCATATCGTACATTATT
ATTTCCGCGGTGATCTACCCGATCATGTGCGCGCTGGGCGAAATGGTGTGCTTTTTGCCCGGTGATGGTTCTGAC
AGTGCCGGGTCGACGGCCAATCTGGTCACTAGGTACGTTGACCCTTCGTTGGGCTTCGCCACTGGGTGGAATTAC
TTCTACTGCTACGTCATTTTAGTGGCCGCCGAGTGTACGGCGGCATCCGGTGTGGTCGAATACTGGACCACCGCG
GTTCCCAAGGGCGTTTGGATCACGATCTTTCTATGCGTGGTTGTTATACTAAACTTTTCCGCGGTCAAAGTGTAC
GGCGAATCCGAGTTTTGGTTCGCGTCTATCAAGATATTATGCATTGTGGGACTGATCATTCTGTCCTTTATTTTG
TTTTGGGGTGGTGGCCCCAACCATGACCGTCTTGGCTTCAGATACTGGCAACATCCGGGCGCTTTTGCGCATCAC
CTCACGGGCGGATCGCTGGGTAACTTCACAGATATCTACACTGGGATTATCAAGGGCGCCTTCGCCTTCATTCTT
GGTCCGGAACTGGTGTGCATGACTTCCGCGGAATGCGCGGACCAGCGTAGAAATATCGCTAAGGCTTCGCGCCGC
TTTGTATGGAGACTGATCTTCTTCTACGTTCTGGGGACGCTGGCCATCTCCGTTATTGTTCCATACAATGACCCA
ACATTGGTAAATGCGCTGGCGCAGGGCAAACCGGGAGCCGGCTCGTCACCCTTTGTGATCGGAATTCAAAACGCC
GGGATTAAGGTTCTTCCCCACATTATCAATGGGTGTATCTTGACCAGCGCGTGGTCTGCCGCCAATGCGTTTATG
TTTGCGAGCACGAGATCACTGTTGACCATGGCGCAAACGGGACAGGCACCCAAATGTTTGGGCAGAATCAACAAA
TGGGGTGTTCCATACGTGGCTGTGGGTGTTTCCTTCTTGTGTTCTTGTTTGGCATATCTGAACGTGTCTTCATCC
ACGGCAGACGTGTTTAATTGGTTTTCCAATATCAGCACCATTTCCGGGTTTCTGGGCTGGATGTGCGGCTGCATC
GCGTACCTGAGATTCCGCAAGGCTATTTTCTACAATGGCCTCTACGACAGATTGCCTTTCAAGACGTGGGGACAG
CCTTACACCGTGTGGTTCTCTCTCATTGTTATAGGCATCATCACCATTACCAACGGGTATGCCATTTTCATCCCT
AAGTACTGGAGAGTAGCAGATTTCATTGCTGCCTACATCACCCTACCCATCTTCCTGGTTTTGTGGTTCGGCCAT
AAGCTGTATACTCGGACGTGGAGACAATGGTGGCTCCCTGTGTCCGAGATCGATGTTACTACAGGGTTAGTCGAG
ATCGAGGAGAAATCAAGAGAAATTGAGGAGATGAGATTACCCCCCACCGGTTTCAAAGACAAGTTCTTGGACGCC
TTGTTGTAA

>YCL033C 1.85   Hot
ATGAATAAGTGGAGCAGGCTGTACGTTATAACTGTACGCAGGACTTTTCCAGGGAGAAGAAACATTGTACTGACG
CAGTACTGGAATAAGAGCAAGAAAATGAGTGACGAATCGAATGACGTGAAGTGGAACGATGCCCTGACACCATTG
CAGCTGATGGTGCTGAGAGATAAGGCCACTGAAAGGCCCAACACCGGTGCGTATTTACACACCAACGAGTCCGGT
GTCTACCATTGTGCCAACTGCGACAGACCGTTGTATTCGAGCAAGGCCAAGTTCGACGCTCGTTGTGGATGGCCC
GCATTCTACGAAGAGGTATCCCCTGGAGCCATCACATATCATCGTGACAATTCTTTAATGCCTGCGAGGGTGGAG
ATATGTTGTGCAAGGTGTGGTGGACACTTGGGACATGTGTTTGAAGGTGAAGGCTGGAAACAGTTGCTAAACTTG
CCCAAGGACACCAGACACTGTGTGAACAGTGCGTCTTTAAACCTCAAGAAGGATTAA

>YNL055C 3.79   Hot
ATGTCTCCTCCAGTTTACAGCGATATCTCCAGAAATATCAATGACCTATTGAACAAGGATTTCTATCATGCTACC
CCAGCTGCCTTTGATGTGCAAACAACAACCGCCAATGGCATTAAGTTCTCATTGAAGGCTAAACAGCCTGTCAAA
GACGGTCCACTGTCTACTAACGTGGAAGCAAAGTTGAATGACAAGCAAACCGGCTTGGGTCTAACTCAAGGCTGG
TCTAACACAAACAACTTGCAAACCAAATTAGAGTTTGCCAACTTGACCCCTGGTCTAAAGAACGAATTGATCACT
TCTTTGACTCCAGGCGTCGCCAAGTCCGCCGTCTTAAACACTACGTTCACACAACCTTTCTTCACCGCAAGAGGT
GCCTTTGACTTGTGTTTGAAGTCACCAACATTTGTTGGTGACTTAACTATGGCCCACGAAGGTATTGTTGGTGGC
GCAGAGTTTGGTTACGATATCAGCGCCGGTTCCATTTCTCGTTATGCCATGGCTTTAAGTTATTTCGCCAAAGAC
TACTCCTTGGGCGCTACATTGAACAACGAGCAAATAACTACCGTTGACTTCTTCCAAAACGTCAACGCCTTTTTA
CAGGTCGGTGCTAAGGCTACAATGAACTGCAAACTACCTAACTCCAATGTCAACATCGAATTCGCCACTAGATAT
TTGCCTGATGCATCTTCCCAAGTTAAGGCTAAGGTGTCCGATTCCGGTATTGTCACTTTGGCTTACAAGCAATTG
TTAAGACCTGGCGTCACTCTGGGTGTCGGTTCCTCTTTCGATGCTTTGAAGTTGTCTGAACCTGTTCACAAGCTA
GGTTGGTCTTTGTCCTTCGACGCTTGA

>YDR233C 2.67   Hot

ATGTCCGCCTCAGCTCAACATAGCCAAGCCCAACAACAACAACAACAAAAAAGCTGTAATTGCGATTTGCTATTA
TGGAGGAACCCTGTTCAAACCGGTAAATATTTCGGTGGGTCTCTGTTAGCCTTGTTAATTTTGAAAAAAGTTAAC
TTGATCACTTTCTTTTTGAAGGTTGCCTACACTATCTTATTCACAACAGGATCTATTGAATTCGTATCCAAGCTC
TTTTTGGGACAAGGTTTAATCACCAAGTATGGTCCTAAAGAATGTCCAAATATCGCCGGCTTTATTAAGCCTCAT
ATCGATGAAGCTTTGAAACAGCTACCAGTCTTCCAAGCCCACATAAGAAAGACTGTATTCGCTCAAGTCCCTAAG
CACACTTTCAAGACCGCTGTTGCCTTATTTCTACTACACAAATTCTTCTCCTGGTTCTCTATCTGGACCATCGTC
TTTGTTGCTGACATTTTTACCTTCACTTTGCCAGTTATCTACCATTCCTACAAGCATGAAATTGATGCTACTGTC
GCTCAAGGTGTAGAAATTTCTAAGCAAAAGACCCAAGAGTTTTCTCAAATGGCTTGCGAAAAGACAAAACCATAC
TTGGACAAGGTTGAATCTAAATTGGGCCCAATTTCCAACTTGGTCAAATCTAAGACTGCTCCTGTGTCTTCTACT
GCCGGTCCTCAAACCGCCAGCACTTCTAAGTTGGCCGCTGATGTTCCATTGGAACCAGAGTCTAAGGCTTACACA
TCTTCAGCTCAAGTTATGCCAGAAGTTCCACAACACGAACCTTCCACTACCCAAGAATTTAACGTCGATGAATTG
TCTAATGAATTGAAGAAAAGTACAAAAAACTTGCAAAATGAATTGGAAAAAAACAACGCTTGA

>YJR058C 2.27   Hot
ATGGCAGTACAGTTTATACTGTGCTTTAATAAGCAGGGTGTGGTGCGGTTGGTGAGATGGTTCGATGTACACAGT
TCGGATCCTCAGCGTAGCCAGGATGCCATTGCGCAGATTTATAGACTCATATCTTCCAGAGATCATAAGCATCAG
AGTAACTTCGTAGAGTTTTCCGATTCGACGAAACTCATATACAGGAGGTATGCGGGTCTGTATTTTGTCATGGGT
GTGGACTTACTTGACGATGAACCCATATATTTGTGCCACATCCATCTGTTTGTGGAGGTGCTAGATGCATTTTTC
GGCAATGTCTGTGAACTGGATATCGTATTCAACTTTTACAAAGTCTATATGATAATGGACGAGATGTTTATTGGA
GGGGAAATACAAGAAATTTCAAAGGATATGCTGTTAGAAAGACTAAGTATTTTAGATAGACTAGACTAG

>YJR016C 2.13   Hot
ATGGGCTTGTTAACGAAAGTTGCTACATCTAGACAATTCTCTACAACGAGATGCGTTGCAAAGAAGCTCAACAAG
TACTCGTATATCATCACTGAACCTAAGGGCCAAGGTGCGTCCCAGGCCATGCTTTATGCCACCGGTTTCAAGAAG
GAAGATTTCAAGAAGCCTCAAGTCGGGGTTGGTTCCTGTTGGTGGTCCGGTAACCCATGTAACATGCATCTATTG
GACTTGAATAACAGATGTTCTCAATCCATTGAAAAAGCGGGTTTGAAAGCTATGCAGTTCAACACCATCGGTGTT
TCAGACGGTATCTCTATGGGTACTAAAGGTATGAGATACTCGTTACAAAGTAGAGAAATCATTGCAGACTCCTTT
GAAACCATCATGATGGCACAACACTACGATGCTAACATCGCCATCCCATCATGTGACAAAAACATGCCCGGTGTC
ATGATGGCCATGGGTAGACATAACAGACCTTCCATCATGGTATATGGTGGTACTATCTTGCCCGGTCATCCAACA
TGTGGTTCTTCGAAGATCTCTAAAAACATCGATATCGTCTCTGCGTTCCAATCCTACGGTGAATATATTTCCAAG
CAATTCACTGAAGAAGAAAGAGAAGATGTTGTGGAACATGCATGCCCAGGTCCTGGTTCTTGTGGTGGTATGTAT
ACTGCCAACACAATGGCTTCTGCCGCTGAAGTGCTAGGTTTGACCATTCCAAACTCCTCTTCCTTCCCAGCCGTT
TCCAAGGAGAAGTTAGCTGAGTGTGACAACATTGGTGAATACATCAAGAAGACAATGGAATTGGGTATTTTACCT
CGTGATATCCTCACAAAAGAGGCTTTTGAAAACGCCATTACTTATGTCGTTGCAACCGGTGGGTCCACTAATGCT
GTTTTGCATTTGGTGGCTGTTGCTCACTCTGCGGGTGTCAAGTTGTCACCAGATGATTTCCAAAGAATCAGTGAT
ACTACACCATTGATCGGTGACTTCAAACCTTCTGGTAAATACGTCATGGCCGATTTGATTAACGTTGGTGGTACC
CAATCTGTGATTAAGTATCTATATGAAAACAACATGTTGCACGGTAACACAATGACTGTTACCGGTGACACTTTG
GCAGAACGTGCAAAGAAAGCACCAAGCCTACCTGAAGGACAAGAGATTATTAAGCCACTCTCCCACCCAATCAAG
GCCAACGGTCACTTGCAAATTCTGTACGGTTCATTGGCACCAGGTGGAGCTGTGGGTAAAATTACCGGTAAGGAA
GGTACTTACTTCAAGGGTAGAGCACGTGTGTTCGAAGAGGAAGGTGCCTTTATTGAAGCCTTGGAAAGAGGTGAA
ATCAAGAAGGGTGAAAAAACCGTTGTTGTTATCAGATATGAAGGTCCAAGAGGTGCACCAGGTATGCCTGAAATG
CTAAAGCCTTCCTCTGCTCTGATGGGTTACGGTTTGGGTAAAGATGTTGCATTGTTGACTGATGGTAGATTCTCT
GGTGGTTCTCACGGGTTCTTAATCGGCCACATTGTTCCCGAAGCCGCTGAAGGTGGTCCTATCGGGTTGGTCAGA
GACGGCGATGAGATTATCATTGATGCTGATAATAACAAGATTGACCTATTAGTCTCTGATAAGGAAATGGCTCAA
CGTAAACAAAGTTGGGTTGCACCTCCACCTCGTTACACAAGAGGTACTCTATCCAAGTATGCTAAGTTGGTTTCC
AACGCTTCCAACGGTTGTGTTTTAGATGCTTGA

>YLR229C 1.55   Hot
ATGCAAACGCTAAAGTGTGTTGTTGTCGGTGATGGTGCTGTTGGGAAAACGTGCCTTCTAATCTCCTATACAACG
AATCAATTTCCAGCCGACTATGTTCCAACAGTGTTCGATAACTATGCGGTGACTGTGATGATTGGTGATGAACCA
TATACGTTAGGTTTGTTTGATACGGCCGGTCAAGAAGATTACGATCGATTGAGACCCTTGTCATATCCTTCTACT
GATGTATTTTTGGTTTGTTTCAGTGTTATTTCCCCACCCTCTTTTGAAAACGTTAAAGAAAAATGGTTCCCTGAA
GTACATCACCATTGTCCAGGTGTACCATGCCTGGTCGTCGGTACGCAGATTGATCTAAGGGATGACAAGGTAATC
ATCGAGAAGTTGCAAAGACAAAGATTACGTCCGATTACATCAGAACAAGGTTCCAGGTTAGCAAGAGAACTGAAA
GCAGTAAAATATGTCGAGTGTTCGGCACTAACACAACGCGGTTTGAAGAATGTATTCGATGAAGCTATCGTGGCC
GCCTTGGAGCCTCCTGTTATCAAGAAAAGTAAAAAATGTGCAATTTTGTAG

>YDR246W 1.91   Hot
ATGGCCATCGAAACAATACTTGTAATAAACAAATCAGGCGGACTAATCTATCAGCGGAATTTTACCAACGACGAA
CAGAAATTGAACAGCAATGAATACTTAATTCTTGCTAGTACACTGCACGGTGTATTCGCCATCGCGAGCCAGCTG
ACTCCGAAGGCATTACAGCTAACTCAACAAACGAACATCGAAAATACCATCCCATATATACCTTACGTGGGCATG
TCCAGCAATAGGAGCGATACAAGAAATGGAGGTGGCAATAACAACAAACACACTAATAATGAAAAACTGGGCAGT
TTTAAAGGAGATGATTTTTTCAAAGAACCATTTACGAACTGGAACAAGAGCGGATTGAGACAACTATGCACGGAT
CAGTTCACGATGTTCATATATCAGACCCTAACCGGCCTGAAGTTTGTCGCTATCAGCTCCAGCGTCATGCCCCAG
AGACAGCCAACTATAGCCACCACCGACAAGCCTGACCGACCCAAGAGTACATCCAATTTGGCCATCCAAATAGCC
GACAACTTCCTAAGGAAGGTTTACTGTTTGTATAGTGACTACGTCATGAAAGACCCATCTTACTCAATGGAAATG
CCTATAAGATCTAACCTGTTCGACGAAAAAGTCAAAAAAATGGTAGAAAACCTACAATAG

>YOR229W 1.83 Hot
ATGGCGAAAAGCAAATCCAGTCAGGGTGCGAGTGGAGCACGCCGTAAACCGGCACCTTCTTTATACCAACACATT
TCTAGCTTTAAACCACAATTCAGCACAAGAGTCGACGATGTTTTACATTTTAGCAAGACACTGACATGGAGAAGT
GAGATTATTCCAGACAAATCCAAGGGCACCTTGACGACTAGTCTTTTATATTCTCAAGGTAGTGATATTTACGAA
ATAGACACTACTTTACCATTAAAGACATTCTATGACGACGACGACGACGACAACGACGACGACGACGAAGAA
GGTAACGGCAAGACGAAGTCTGCAGCCACCCCAAATCCAGAGTATGGTGATGCTTTCCAAGATGTTGAGGGGAAA
CCATTGCGCCCAAAGTGGATTTACCAGGGCGAAACAGTGGCAAAAATGCAATACTTGGAAAGCTCAGACGACAGT
ACGGCGATCGCGATGTCCAAGAACGGCTCTTTGGCATGGTTCAGAGATGAGATCAAGGTTCCCGTTCATATCGTA
CAGGAAATGATGGGCCCTGCTACTAGATACTCTAGTATTCACTCTCTAACCAGACCAGGTTCATTAGCAGTGTCA
GATTTTGATGTGTCGACAAATATGGATACGGTAGTAAAGTCACAAAGTAACGGCTACGAGGAGGACAGCATCTTG
AAAATCATCGACAACTCCGATAGACCGGGAGATATATTACGTACAGTGCACGTTCCAGGGACCAACGTGGCTCAC
TCAGTTAGATTTTTTAACAACCATTTATTTGCATCTTGTTCAGATGACAACATTTTAAGATTCTGGGACACAAGA
ACAGCGGACAAACCGCTTTGGACGCTAAGCGAACCGAAAAATGGACGACTAACGTCCTTCGATTCCTCTCAAGTT
ACTGAAAACCTATTTGTCACTGGGTTCAGCACGGGTGTCATCAAGCTATGGGATGCGCGTGCTGTGCAACTGGCT
ACTACTGACCTCACACATAGGCAGAACGGCGAGGAACCGATCCAAAACGAAATAGCCAAGTTGTTCCATTCTGGC
GGCGATTCCGTTGTCGATATCCTGTTCTCACAAACCTCTGCAACAGAATTTGTTACGGTTGGAGGAACGGGTAAT
GTCTACCACTGGGACATGGAGTACTCTTTTTCAAGAAACGACGATGACAACGAAGACGAAGTTCGAGTGGCTGCT
CCGGAGGAACTTCAGGGTCAATGTTTAAAATTTTTCCACACAGGTGGCACAAGAAGATCGAGCAACCAATTCGGG
AAGAGGAACACTGTGGCGCTACACCCCGTAATCAATGATTTTGTTGGCACTGTTGATTCAGATAGTCTTGTTACT
GCCTACAAACCATTTCTGGCCAGTGACTTCATTGGCAGAGGTTACGACGATTAA

>YLR316C 1.65 Hot
ATGGTTAAGAAAGTTAATAATCCGCTAAAAATCGATTATCAGAACGGGATAATAGAAAATCGGCTTCTGCAAATT
AGAAATTTCAAAGATGTAAATACGCCCAAACTAATCAATGTATGGAGTATAAGAATAGATCCAAGAGACTCGAAG
AAAGTAATAGAGCTAATTCGAAACGATTTTCAGAAAAATGACCCTGTTTCATTGAGACATTTGAAAAGGATCCGC
AAGGACATCGAGACCTCCACCTTGGAAGTGGTGTTGTGTTCGAAAGAGTATATTTGCGACGAAGGTGAAATAAAC
AATAAATTGAAGAGTATTTGGGTTGGAACGAAAAAGTATGAACTGAGTGATGATATCGAAGTGCCTGAGTTTGCG
CCCAGCACAAAGGAACTGAACAATGCCTGGTCTGTGAAGTACTGGCCGTTGATTTGGAACGGCAACCCCAATGAT
CAAATATTGAACGACTATAAAATAGACATGCAGGAAGTACGAAATGAGCTATCGCGTGCGTCGACGTTGTCTGTG
AAGATGGCAACGGCAGGAAAACAGTTTCCAATGGTGAGCGTCTTTGTTGACCCATCAAGAAAGAAGGACAAAGTG
GTGGCAGAAGATGGCAGAAACTGCGAAAACTCGCTACCCATCGACCACAGTGTAATGGTGGGCATCCGTGCGGTA
GGCGAAAGACTCAGGGAGGGTGTAGACGAAGACGCAAACTCTTATCTGTGTCTTGATTACGACGTCTATTTGACC
CATGAGCCGTGCTCAATGTGCTCCATGGCCCTGATCCATTCTCGTGTGAGACGAGTGGTCTTCCTTACAGAGATG
CAGCGCACCGGGAGCTTAAAATTAACGAGTGGTGACGGGTACTGCATGAACGATAACAAACAGCTAAACTCAACA
TACGAAGCCTTTCAATGGATCGGCGAGGAATATCCTGTGGGGCAGGTTGACCGGGATGTCTGCTGCTAG

>YIL100W 2.07 Hot
ATGTATATATATATGTTGAAGTGTGTACACACGTACACGCACAAGCTACAGACGCCACGCGGCCCGGCATTCATATA
TGTACATACATTTATGCACATATATATATATATATATATGTATATGTAAATGTATATATACAGCTTGTGATTTCG
CGCGCCGAATGGGCTGAAAAGCACAGTATAGTGGAGAAGAAGAGAGAGGAGAAGGGGAGAGGGGCAATAGCAATG
GATGTGGTAGAATACAGGGAAGGCCAAGCTGTTGTTCAATGGATGCGGTTCGAGGCCCAGCGCAAGGGGGGCGCT
TCGAAGCATAGAACATTATCCGCGGAAACGGGTATTAGGGGTGAGGGTGAATAA

>YEL039C 1.79 Hot
ATGGCTAAAGAAAGTACGGGATTCAAACCAGGCTCTGCAAAAAAGGGTGCTACATTGTTTAAAACGAGGTGTCAG
CAGTGTCATACAATAGAAGAGGGTGGTCCTAACAAAGTTGGACCTAATTTACATGGTATTTTTGGTAGACATTCA
GGTCAGGTAAAGGGTTATTCTTACACAGATGCAAACATCAACAAGAACGTCAAATGGGATGAGGATAGTATGTCC
GAGTACTTGACGAACCCAAAGAAATATATTCCTGGTACCAAGATGGCGTTTGCCGGGTTGAAGAAGGAAAAGGAC
AGAAACGATTTAATTACTTATATGACAAAGGCTGCCAAATAG

>YJL100W 2.56 Hot
ATGAGTAACGAAGCTTACCAGCATGATCATACCGTAAATCCTCACCAGAAGATAGTTGTGAACAGCTACGATTGG
TTACAGTTCCGTGATGAGCAAGATCACTGTAAAAGTAAGAACCCGATAACGCACGCTTCTCCAGGGGTCGGTTCG
AATGCACAGAATTCAGACATAGCGGAGGCCCCTCAGGTATTCCACCCTTCCTATCAATCTCTGGTCAACGTACCG
TCCGAGAGCCCGCGACCTGATCAGACTTCAGGGTCCAATCCTGCCGTTGGACTGCTTCATAATGCAGAGGACAAA
GCGTCAGGGCAAGAAGAAGAAGGAAGTCAATATGAGATTCAATATTCGGTATTTCGTCCGCTGCATGCATATCCA
ACCAAGGGCCTAGCATATGAGCAGCTCCGAAGAAAAGAAGAACAAGAACAGAGGGAAAACTTCAACCATTTGGTT
TCTGACTGTATTGAGGCTGTAGAGACATTTGGACGCGAACTCGAGAGAATCCAAACTGGCTCAAGTGGGTCGTAC
TTTGTGTATGGAACACGGGCCGACGAAAGCGTTCCGGTGGGGGTCTTCAAGCCCAAGGATGAGGAACCATATGGC
CCATTTTCTCCCAAATGGACCAAATGGGCACACCGCACGTTTTTCCCATGTCTGTTTGGCAGAAGTTGTCTGATT
CCAAACCTCGGGTACATCTGTGAAAGTGCGGCTAGTTTGTTGGATAGGCGACTGGAAACGCATCTAGTACCCTAT
ACAGATACTGCATCTATAGAGTCTTTTAATTTTTATGATAATAGAAAAAAATGGGTATTGGGATACAACCTTCAG
AAGAAGAAACAAAAAAAGCTGGGTTCTTTCCAACTTTTTTTGAAGGAGTACATCAACGCCGATGAGTTCTTTCAT
AAATACCCATTACCTGGGATGTATTCAGATGTTAAACATTCATTTCATCGTAAGTCATCCGGCGAAGATATCAAT
CATAAACCAGAGACAACCAGGAACCTAACAGATGAGACAGAACCGTCCAAGCAAATAAATTCCTCTCCAATTTCG
ACGGAATCTGAAGAAAATTCGAAATTTGAATGGACAGAATCAAGTTTGAGTCAGTTCAGATTGGAACTAGAAAAA

CTAATTATCCTTGATTATATAATGAGAAACACAGATAGAGGTCTAGACAATTGGATGGTGAAACTAATCAAACTT
TCAAACAATAAGTGGAGACTCAAGTTAGCCGCCATCGACAATGGGTTGTCCTTTCCCTGGAAGCATCCTGATGAG
TGGCGTCTGTACCCATATGGCTGGTTGTATTTGCCTCTGCAACTACTAGCCAAACCATTTTCTGAGCAAATGAGA
TCTCATTTCCTACCGATATTGACAAGCACTAATTGGTGGGAAGAGTCGTACCAAGAATTCTTGGCCCTATTTAGC
AGGGACCAAGATTTTAACGTCCGAATGTGGAAGAAGCAATGGGCAGTATTAAAAGGACAGGCGTTCAACGTTGTC
GAAACATTGAAAGACCCACGACAAGGCCCCTTGGAATTGGTTAGAAGGACAAGGTGCCAGGTAATAGATGAGAAG
ATGCAAGTTCCCTGTTGCCCACCTCCAGTTTCTATTTTCAAAAACGCCATAGACGAACCTATTGGATCATATTCC
ACTTCACCAATGGTACTCCCTAGCACCCCAAGCACAATTCCATTTCATGCACATAACCAAAGCAATAGTAATCCT
GTATACTATGATTCCACTCTGCACCCATTTGCTAATAAAACAGTGATAGCAGAGCGGCTACAAATAGTCAATTCC
ACCCCCGTATTCACCTGGTGTTGA

>YMR295C 1.5 Hot
ATGATGCATTTTAGAAAGAAATCCAGTATCAGTAACACGAGTGATCATGACGGAGCGAACCGTGCCTCAGATGTC
AAGATTTCTGAAGATGACAAGGCAAGATTGAAGATGCGTACTGCTTCCGTTGCTGATCCTATTCTAGACGCTGTG
CAGGAAGCCCAACCTTTTGAACAAGCTGCTGATACCTTCCACGACAACATGAACAGACAATCTTATTTTTCCAAC
GAAGAAGGTCATGTTCTGTGTGATGTTTTCGGTCAACCCATCACACAGGCAGATATATCGAATCCAACTAGGGCA
AGAGACGAAAGACCTTTGGACACTATAAGAAGTTTCGAGTATGCTGTCTCCGGAGACCCGGTCTGGGCCCAACAG
TTGGAGACTCCAACTTACGGGTTCCGCGTAAGACCTGATTTCCCTGTGTTTGGTGCTGCTGTGACTTACGATGCG
AACGGTATGCCACAGCAAGTGGGTGGTGCTTCCAGTCAAATGTATGGTGAACAAGCAGTGTATCAGCCACAACAA
CACGTACAGACTGAGGAAAAGCAAAAGAAGAAGAAGAAGGGATTGTTTGGTAGAATGAAGAAGAAATAA

>YLR280C 1.59 Hot
ATGGCGGCAGAGGGCCCGGTGGCCAAAAGATTAATAAGTGTAATTCTAAGGTACAACTACGGCATGAACCCACTG
GGATCGTTGTCGAGTGCCAAGAAACGCGGTCACGTGAGCAAAATCGAAAGCTTGCCCGGCTTAAGCTCGCGCGCG
AACTTGCGGCGTCGTACGACACGATGCCGTCCAGAGAGGAGGCGCTTCTACAGTGGCACCGTCAACAGAAACGCT
CGCAGCGCCGGCGCAGCGTCGCGAAGTACGAGCAGCGTGAAGAGGCCGCTCGAGTCGAAAAAGAGGAACGCGAGG
CCCGAGACAGAGAAATGGTGCGCGAGTTATTCCGCCGGTAACCGCAGGTGA

>YIL160C 1.67 Hot
ATGTCTCAAAGACTACAAAGTATCAAGGATCATTTGGTGGAGAGCGCCATGGGTAAGGGTGAATCGAAGAGGAAG
AACTCGTTGCTGGAGAAAAGACCCGAAGATGTAGTTATTGTGGCTGCTAACAGGTCTGCCATCGGTAAAGGTTTT
AAAGGTGCCTTCAAAGATGTAAACACAGACTACTTATTATACAACTTTCTCAATGAGTTCATCGGGAGGTTTCCG
GAACCTTTGAGGGCTGATTTGAACTTAATCGAAGAAGTTGCCTGTGGAAATGTTCTCAATGTTGGAGCCGGTGCT
ACAGAACACAGGGCTGCATGCTTGGCAAGTGGGATTCCCTACTCGACGCCATTTGTCGCTTTAAACAGACAATGT
TCTTCAGGTTTAACGGCGGTGAACGATATTGCCAACAAGATTAAGGTTGGGCAAATTGATATTGGTTTGGCGCTG
GGAGTGGAATCAATGACCAATAACTACAAAAACGTCAATCCCTTGGGCATGATCTCCTCTGAAGAGCTGCAAAAA
AACCGAGAAGCGAAGAAATGTCTAATACCAATGGGCATTACTAATGAGAATGTTGCCGCTAATTTCAAGATCAGT
AGAAAGGATCAAGACGAGTTCGCTGCGAATTCATATCAAAAAGCTTACAAGGCGAAAAATGAGGGGCTTTTCGAA
GATGAAATTTTACCTATAAAATTACCAGATGGCTCAATTTGCCAGTCGGACGAAGGGCCACGCCCTAACGTCACT
GCGGAGTCGCTTTCAAGCATCAGGCCTGCCTTTATCAAAGACAGAGGAACCACAACTGCGGGCAATGCATCCCAG
GTCTCCGATGGTGTGGCAGGTGTCTTGTTAGCCCGCAGGTCCGTAGCCAACCAGTTAAATCTGCCTGTGCTAGGT
CGCTACATCGATTTTCAAACAGTGGGGGTTCCCCCTGAAATCATGGGTGTGGGCCCTGCATACGCCATACCAAAA
GTCCTGGAAGCTACTGGCTTGCAAGTCCAAGATATCGATATTTTTGAAATAAATGAAGCATTCGCGGCCCAAGCA
TTATACTGCATCCATAAACTGGGCATCGATTTGAATAAAGTAAATCCAAGAGGTGGTGCAATCGCGTTAGGCCAT
CCCTTGGGTTGTACTGGCGCAAGGCAAGTAGCTACCATACTAAGAGAACTGAAAAAGGATCAAATCGGGGTTGTT
AGTATGTGTATCGGTACTGGTATGGGTGCCGCCGCCATCTTTATTAAAGAATAG

>YDL197C 1.52 Hot
ATGCCAAAAAATCGTGGTGTCTTGGATGCCATCACTCGCAGTGTGATAGATGGTAGTGATCAGGAGTCGAGCTCA
TCTTTCAACTCGGACAAGGAATATGCGGCTGTGACGAAGGGCCTCAGCAGCTCAAGAGTACAAAAGAAGTCATCT
TTAAGGCAGATGAAAAGCAAAGTCAAGGAGCTTCAATCTTTAGTGAATCATTACCGAGAAAATGAAGCCGCTTTA
GTGTCTAGCGCCAAGTTATTGTCCGGCGAGATCATAGGCTATGAAATTAAGATGGCTAGTTTACATGGAAAAATG
AAAAGTATCCTGGATGAGAACAACGCGTTAAAGGAAACGCACAAGAGCTCTGCTGAAAAGAGAATCGAACTTGTT
CGACTTCCCTCTAGCAAAGAAGAACGCAACTACGATGAATACACTTTGCTTGTTAATCTCAAGAAGGAAATCTGC
GCCAAGTTACAGGACTACAAAAACGTCCAGAACACGGTGAACACCAAGTTAGATGAGATTCACACGTTCCATGAA
AAATACTACGAAGGACTAGAACTAAGTTTAGACAGCAAGGTGTTTGATGCAGAAAGCTCAAAAGAGCTGGCCAAA
GTGAGGCGAGAACTGAATAACGTGAGAAAAAATTCCGAAATCAAAGTGAATAACTTGAAAATGCAACTGCTACAA
GCAACCAAATCACTGGAGCACTTGAAGAAGCAGGCTAAAGCAAAGGATGACTATCTTAAATGTATTCCTGAGCTG
GTCGACAAAGCCAATCTCACGATGTTATCCTACAAGAAAAGCATCGCTAACCAGAGGGAGACTATCGAAGCATTA
CAAGCAGAACTATCGCAACAATCAGAAACAAAGGGGCAAATAGAAACAGAAACACAAAATCAAGTACAAATCCCC
ACCAATGTGACTTTGGTGGACCCCTTTGAGGAAAACAATCCAGAGGATTTGTTCGCTATCCAGGAACAGGAATTG
CAAGATCTAAGGTTGCATAAGAAAATGGCCGATGAGAGGAGCCGAACTACCCACTTGCATTTAGAAAGAAAAAAT
AATACCATCAAGCTACTGCAGAGTTACGTGCAATCATTAATTCAACGGCTACCACCAGCCCAGCGCAAACACCAT
TTGGGCATATTCCAAAAACTTGGTAGCGAAAAATCGTGTCCACTCGCCCCCGCTGTGGCAAGTACATATGCGCCT
CTTCTACTCTTATCCCAGCATTCGAACCATCAAGAAATTGACAACACCCCTCAACGGCTTCTATTAGCGGCTCCA
GACGGGCAGTCATATTCCGAAAAGAGCACAACACTAAACCTTGATTACTCATCAAGGAAATCCTACTTATCAAGA
CTACAACCACCACATATAGCCAACCTGAAGTCTTTGACTCTGAAAACTCTGCCAAGGGTGCCCACGGACAGTCCT

CAACTTCCTTCAAAAGACAAATCTCAAGAGACTGCAAAGAAAGACGATCGGCCAAAATTGGTGGCAAATGAACCA
GTCACACTAGACACATCAACTCCTCCTGTGGCACAAAGCTTGGCGGATTCAAAGCATTGTTCAGGACTACATAAA
TAA

>YOL082W 1.59   Hot
ATGAACAACTCAAAGACTAACCAACAGATGAACACCTCAATGGGGTACCCCTTAACCGTCTACGATGAATGTAAC
AAATTCCAACTAATTGTGCCAACTTTAGATGCAAATATCATGCTTTGGTGTATTGGACAACTTTCACTGCTGAAT
GATTCTAACGGTTGTAAGCATTTGTTTTGGCAACCGAATGACAAGTCAAATGTCCGAATCTTACTAAATAATTAT
GACTATGGTCACTTGTTCAAATACTTGCAATGTCAAAGAAAATGCTCAGTTTATATCGGAGAAGGAACGCTTAAG
AAATATAATTTGACTATTTCTACATCCTTTGACAACTTTTTGGACCTGACGCCCTCCGAGGAGAAGGAAAGCTTA
TGTAGAGAAGACGCACACGAAGATCCCGTATCTCCAAAGGCTGGTTCCGAAGAAGAGATAAGCCCTAATTCCACC
TCTAATGTGGTTGTGTCAAGAGAATGCTTGGATAACTTCATGAAACAACTCCTTAAGTTGGAAGAATCGTTAAAT
AAGTTGGAATTGGAGCAAAAGGTCACAAATAAAGAACCTAACCACAGAATAAGCGGTACGATTGACATACCAGAA
GATAGGTCCGAACTAGTGAACTTTTTCACAGAGTTGAAAACAGTCAAACAACTTGAGGACGTTTTTCAACGTTAC
CATGACTATGAGAGGTTATCACAAGAATGTGACAGTAAGACGGAAATCGCCTCTGACCATTCGAAAAAGGAAACT
AAAATAGAAGTGGAACCCCCAAATGAGCGCTCATTGCAAATCACAATGAACCAAAGGGACAATTCATTGTATTTC
CAGTTGTTTAACAACACAAACTCTGTTTTGGCAGGCAACTGCAAACTGAAGTTTACTGACGCTGGCGATAAGCCA
ACCACGCAAATTATTGACATGGGTCCTCATGAAATAGGAATAAAGGAGTACAAGGAATACCGATACTTTCCATAC
GCACTTGATTTAGAGGCCGGCTCCACTATCGAGATTGAAAATCAGTATGGAGAAGTGATCTTCTTGGGCAAGTAT
GGCTCTTCTCCAATGATTAACTTAAGGCCACCTTCAAGATTATCTGCAGAAAGTTTACAGGCATCCCAAGAGCCA
TTTTACTCCTTTCAAATCGATACGTTACCAGAACTGGATGACTCTAGTATCATCAGTACATCCATTTCACTCTCT
TATGACGGTGACGACAATGAAAAAGCCCTGACTTGGGAAGAACTCTAG

>YFL022C 1.81   Hot
ATGTCTGATTTCCAATTAGAAATTCTAAAAAAACTAGATGAATTGGATGAGATCAAGTCCACACTGGCAACTTTC
CCTCAGCACGGCTCTCAAGATGTTCTTTCCGCTTTGAACTCTTTGAAAGCCCACAACAAGTTAGAGTTTTCCAAG
GTCGACACGGTTACGTATGACTTGACCAAAGAAGGTGCTCAAATTTTGAATGAAGGTTCGTACGAAATTAAACTA
GTCAAGCTCATCCAAGAGTTGGGTCAACTTCAAATCAAAGATGTGATGTCCAAACTGGGTCCTCAAGTTGGTAAG
GTCGGTCAGGCTAGAGCTTTCAAGAACGGCTGGATCGCCAAAAACGCCTCAAACGAGCTTGAACTCTCCGCAAAA
TTGCAAAATACCGATTTAAATGAGCTTACTGATGAAACGCAATCTATTCTAGCGCAAATCAAGAACAACTCGCAT
CTGGATAGCATTGACGCCAAGATTTTGAACGACTTGAAGAAAAGAAAGTTAATTGCTCAAGGTAAAATCACAGAT
TTCAACGTTACCAAAGGGCCAGAGTTCTCGACCGACCTCACCAAATTGGAAACCGATCTTACCTCCGACATGGTC
TCCACCAATGCATACAAGGACTTGAAGTTCAAGCCTTACAATTTCAATTCTCAAGGTGTGCAAATATCTTCAGGT
GCTCTTCACCCCTTAAACAAAGTCAGAGAGGAATTTAGACAAATTTTCTTTTCCATGGGATTCACAGAGATGCCC
TCGAACCAATACGTCGAGACAGGTTTCTGGAACTTCGATGCCCTTTACGTCCCACAACAGCATCCTGCTCGTGAC
CTGCAAGACACTTTCTACATCAAGGACCCACTAACCGCTGACTTGCCCGATGACAAGACATACATGGACAATATC
AAAGCCGTTCACGAACAGGGGAGATTCGGGTCCATCGGTTATCGTTACAACTGGAAGCCAGAAGAATGTCAAAAA
TTGGTCTTGAGAACTCACTCCACAGCCATCTCTGCCAGAATGCTGCACGATTTGGCCAAAGATCCAAAGCCCACC
AGATTGTTTTCTATCGACCGTGTTTTCCGTAACGAAGCAGTTGACGCCACCCATTTGGCCGAATTCCACCAGGTG
GAAGGTGTTCTTGCCGACTACAACATTACTCTGGGTGACCTGATCAAGTTCATGGAAGAGTTTTTCGAAAGAATG
GGTGTCACCGGTTTGAGATTCAAGCCTACCTACAATCCTTACACCGAGCCATCAATGGAAATCTTTTCTTGGCAC
GAAGGTTTGCAAAAAATGGGTCGAAATCGGTAACTCTGGTATGTTCAGACCAGAAATGCTCGAGTCCATGGGTCTA
CCAAAGGATCTAAGAGTCCTTGGTTGGGGGTTATCCTTGGAAAGACCTACCATGATCAAATATAAGGTTCAAAAC
ATCAGAGAACTGTTAGGTCATAAAGTCTCTTTGGACTTTATCGAAACCAATCCTGCTGCTAGATTGGACGAAGAC
TTGTACGAATAA

>YDL124W 1.79   Hot
ATGTCATTTCACCAACAGTTCTTTACCTTGAATAATGGAAATAAAATCCCCGCAATCGCCATCATTGGGACAGGT
ACTAGATGGTATAAAAACGAAGAAACGGATGCTACCTTTTCGAACAGTTTGGTCGAACAGATTGTTTATGCTCTG
AAGTTACCTGGCATTATTCACATTGATGCTGCTGAGATCTACAGAACATATCCAGAAGTTGGGAAGGCACTTAGC
CTCACCGAAAAACCAAGAAATGCAATATTCTTGACAGACAAGTACTCACCTCAAATCAAGATGTCAGATTCCCCA
GCGGATGGACTAGATTTAGCTTTGAAGAAGATGGGCACTGACTATGTCGATCTATACCTTTTGCATAGCCCATTT
GTTTCCAAAGAGGTCAACGGGTTAAGTTTGGAGGAAGCCTGGAAGGACATGGAGCAATTGTACAAATCAGGTAAA
GCTAAAAATATCGGTGTTTCCAACTTTGCTGTGGAAGACTTGCAAAGAATTCTGAAAGTTGCGGAAGTCAAGCCC
CAAGTTAATCAAATTGAGTTCAGTCCCTTCTTGCAGAATCAAACACCAGGGATCTACAAATTTTGCCAAGAACAT
GATATATTGGTAGAAGCATACTCGCCACTAGGTCCCTTACAAAAGAAAACAGCACAAGATGACTCTCAACCGTTT
TTCGAATACGTGAAAGAGTTATCCGAGAAATATATCAAATCTGAAGCCCAAATTATCCTACGTTGGGTGACCAAA
CGTGGCGTGTTGCCTGTAACCACCTCTTCCAAACCTCAAAGAATTTCTGACGCGCAAAATTTATTCTCTTTCGAC
TTGACGGCTGAGGAAGTCGATAAGATAACGGAGTTGGGCTTGGAACATGAACCGCTAAGATTGTATTGGAATAAA
TTGTACGGTAAATACAACTACGCTGCTCAAAAAGTATAA

>YGR174C 1.72   Hot
ATGCAGTGCGCGATAACTCCTAGAGAGGCAGTAATAGCAAAACAAAGACAGTATAAGCATTATTTGGGTATGGAA
AGACCATTGTGGGTACGTTGGTTGAAAGTCTATGCAATTGGTGGCGCTATTATTGGAAGCGGGTTTCTGCTTTTC
AAGTACACAACGCCAACAGATCAACAGTTAATCAGCCAGCTTTCCCCAGAATTACGTTTACAATATGAGAGGGAG
AAAAAACTACGTCAGTCGGAGCAGCAGGCTTTGATGAAAATTGTTAAGGAAACTTCTCAAAGTGACGATCCAATT
TGGAAGACGGGCCCCCTTCAATCACCTTGGGAAAGGAACGGTGATAACGTTCAAAGCAGAGATCACTTTGCTAAG

GTGAGGGCGGAGGAGGTGCAAAAAGAAGAGTTGGCTAGGATCAGAAATGAACTGTCTCAGCTAAGATCTGAAACG
GAGGAGAAAACAAAGGAAATAGTCCAGGATAAGCAGGTTAAAAGCTGGTGGCGCTTCTGGTAG

>YER146W 1.79 Hot
ATGAGTCTACCGGAGATTTTGCCTTTGGAAGTCATAGATAAAACAATTAACCAGAAAGTGTTGATTGTGCTGCAG
TCGAACCGCGAGTTCGAGGGCACGTTAGTTGGTTTCGACGACTTCGTCAACGTTATACTGGAAGACGCTGTCGAG
TGGCTTATCGATCCTGAGGACGAGAGCAGAAATGAGAAAGTTATGCAGCACCATGGCAGAATGCTTTTAAGCGGC
AACAATATTGCCATCCTTGTGCCAGGCGGCAAAAAGACCCCTACGGAGGCGTTGTAA

>YJR057W 2.04 Hot
ATGATGGGTCGTGGCAAATTAATACTGATAGAAGGATTGGATAGGACTGGTAAAACCACGCAATGTAATATTCTT
TACAAAAAATTGCAACCAAACTGTAAACTATTGAAGTTCCCCGAAAGGTCTACCCGAATCGGAGGACTCATAAAC
GAATATTTGACGGATGATAGTTTCCAATTATCAGATCAGGCAATTCACCTCTTGTTTTCGGCAAATAGATGGGAA
ATAGTTGACAAGATAAAGAAAGATTTACTAGAAGGCAAGAACATTGTCATGGACAGATATGTTTATTCTGGAGTG
GCATATTCTGCCGCTAAGGGGACAAATGGAATGGATTTGGATTGGTGCTTGCAACCCGATGTAGGGTTGCTGAAA
CCGGATTTGACATTATTTTTAAGCACTCAAGATGTCGACAATAACGCCGAAAAATCTGGATTTGGTGACGAAAGA
TACGAAACTGTTAAGTTTCAAGAAAAAGTGAAGCAAACTTTTATGAAGCTATTGGATAAAGAGATAAGGAAAGGC
GATGAGTCAATCACGATTGTAGACGTTACTAATAAGGGCATTCAGGAAGTTGAAGCGCTTATTTGGCAAATCGTT
GAGCCTGTTTTGAGTACGCATATTGATCATGATAAATTTTCGTTCTTCTAG

>YMR265C 2.14 Hot
ATGGAAGAATTTGAAGAATTTCGAAGGAAAGGGGAGATGTCCAGCAGATGTGGCAATCACCGTGTGCTCAGAAAA
TGGAATAGTTGCGCTTGCGAGTTGGCCGTGCCCTTTGAGGTTCCTGAGCATGCGATTACGAAATTGCACATATAC
GACTTCGACAATACATTATTTGCAACGCCGGGTCCTACAGAGCAATTGTATACACGTGAGCTACTAAATTTGTTG
ACATCGAGCACGCTACCGAATGGAGGCTGGTGGAATGAGCCGGGGTTTCTGCAGGCTGCTATTGAGATCTCGAAG
ACTAAACCGAGAAGGTATTCGTGGAATGCGGATATCGTTAAATTGGCGGAGGAGTCATATTCTGCCAAGGATACT
ATCTCGATCGTTTTAACGGGCAGAGAGGAAAGTAAGTTCCATAAGTTGATTGAGCATGCTTTACAGACGGCAAGG
AGCCATTGGAAATGCTCAGAAAATGAGTTTAGGTTCAATGCAGTTTGCCTAAAAAAAAGAGCAATTTCTGAGTAC
ACTAGCAAATATAAAAAAGAGTTGATGCGGGATTTCTTAGAATATTATCCCTCTCTGCGGGAGTTGTCCATATAT
GATGATAGAATACACCAAATAGATGCATTCAAGTCTTTCTTCCATTCTTTGGATCTACCACGTCTGAAATGGTCT
GCGATCCCCGTACGACCATTCACCAAATCGCTGCCAAGAGAGCAGGAACTTGAAATGGTGATGGACATGGTACGA
AAGAACAATAGCCAAGCTCTCAGCACTTCACAAAAGTTTGATTTAAGACGGACTCCAAGACAAATTGGTTATATC
CTTTGCACGGCATCTCATCGTCTATTATCAATAGAGGTCATCAAGTACTTAAAGCGGCGTAAGGGGAGAAGAACG
TTCAGACCCAAGCTGTATGAACATCCCTTGTATATACCGTGCGCAGAACCCGGTAAAGATATCCCTGCACTTGAA
ATCGCTAAGGTATGGTCAAACAATGACACTCGCACGTTTGATTCTGAGAAGAAAGTACAGCATATTTCACAAATT
TTTTATCTGGAACAGCCTGGGAAGTGCATCGTACACTTCCAAGTCACAGACCTTGCCGTCATCGCGTCGGCCCAC
CACAACAGAAGAAAGCCATTGGAGGTTTATTTCAAAGCCACACCCGAACCAAACAGATACACTTTCACTCTCTTT
CCAGAATATATAGTCACTGGTCATTTTTACAAAAGAGACCGAATTGAAGATTTAGAAGTGGTAACAGAGCGTCTA
ATAAACTGCAAGGAAGATATTCACTGGGTTCCGTTAGACAATACAATACCAATAAAGGCATTTTTCGGGCGATTT
GCCAAGTTGGCAGCAATACCGTGTTCCAATGCTTAA

>YFR024C-A 1.64 Hot
ATGGGTATTAACAATCCTATTCCAAGGAGTTTAAAGAGTGAGACAAAAAAAGCAGCGAAGATTTTGGCTAGTTTT
GTCAAACCAAACCAAGTTTTTGGTGCGGACCAAGTCATTCCTCCAGACGTTTTGAAAAGAGCTAAAGGGTTGGCT
ATCATAACGATCTTAAAAGCAGGTTTTCTATTTTCAGGTAGAGCTGGTTCTGGTGTGATTGTCGCAAGATTAAAG
GATGGTACTTGGTCAGCTCCATCTGCCATTGCCATGGCAGGTGCTGGTGCCGGTGGGATGGTTGGTATAGAATTG
ACTGATTTTGTTTTCATTTTAAATACTCAGGATGCTGTTAAGTCGTTTTCTGAATTTGGGACCATCACGTTGGGT
GGTAACGTCTCCGTTTCTGCTGGGCCTCTTGGTAGAAGTGCAGAAGCAGCTGCATCAGCCTCCGCCGGTGGTGTG
GCTGCAGTTTTTGCCTATTCTAAGAGTAAAGGTCTATTTGCAGGGGTTTCTGTGGAAGGTTCGGCTATTATAGAA
AGAAGAGAGGCTAACAGAAAATTTTATGGTGACAATTGTACCGCGAAAATGATTTTGTCTGGTAGGATAAGACCG
CCTCCAGCAGTTGATCCATTGTTTCGTGTTTTAGAGTCCAGGGCGTTTAATTACAGACCCTCCAACGGTGGTCGT
GGCAGTTTCGATGACGACGAAGACGATTACTATGACGACGACGATTACTATAATGACATTCCTAGTTCATTCAGT
TCTACAGATGCTTCCTCCACTAGACCTAACACAAGATCCACAAGGAGAAGAGCCCAAAGCGGTTCTCGTTATACG
TTCGACGATGATGATGACGATGATGATTATGGTACTGGCTATTCAAGGAATTCAAGACTGGCTCCTACCAACAGT
GGCGGTAGTGGTGGTAAATTGGATGACCCATCTGGAGCTTCAAGTTATTATGCCAGCCACAGAAGATCAGGAACT
GCTCAGTCAAGGGCTCGTTCTTCAAGAAATCGTTGGGCTGATGATGAATACGATGATTATGATGATGATTATGAA
TCCGGGTATAGACGCGGGAATGGAAGAGATAGGACAAAAGACCGTGAAGTCGATGACTTGTCGAACAGATTTTCT
AAATCTAGAATCTCGTCCGCTTCAACACCGCAAACTAGTCAGGGCAGATTCACTGCTCCAACATCCCCATCCACG
TCTTCTCCAAAGGCCGTGGCATTATATAGCTTTGCAGGAGAAGAGTCCGGAGATTTACCATTCAGAAAGGGTGAC
GTGATCACAATTTTGAAAAAATCAGATTCTCAAAATGACTGGTGGACCGGAAGAGTCAACGGTAGAGAAGGTATA
TTCCCAGCAAATTACGTTGAACTAGTTTAA

>YAL062W 1.79 Hot
ATGACAAGCGAACCAGAGTTTCAGCAGGCTTACGATGAGATCGTTTCTTCTGTGGAGGATTCCAAAATTTTTGAA
AAATTCCCACAGTATAAAAAAGTGTTACCTATTGTTTCTGTCCCGGAGAGGATCATTCAATTCAGGGTCACGTGG
GAAAATGATAATGGCGAGCAAGAAGTGGCTCAAGGATACAGGGTGCAGTTCAATTCAGCCAAGGGCCCTTACAAG
GGTGGCCTACGCTTCCACCCATCAGTGAACCTGTCTATCCTAAAATTTTTGGGTTTTGAACAGATCTTCAAGAAT

GCGCTCACTGGGCTAGATATGGGCGGTGGTAAGGGTGGCCTGTGTGTGGACTTGAAAGGCAAGTCTGACAACGAG
ATCAGAAGGATTTGTTATGCGTTCATGAGAGAACTGAGCAGGCATATTGGTAAGGACACAGACGTGCCCGCAGGA
GATATTGGTGTCGGTGGCCGTGAAATTGGCTACCTATTCGGCGCTTACAGATCATACAAGAACTCCTGGGAAGGT
GTGTTGACTGGTAAGGGTTTAAACTGGGGTGGCTCACTTATCAGGCCGGAGGCCACCGGGTTCGGCTTAGTTTAC
TATACGCAAGCAATGATCGATTATGCAACAAACGGCAAGGAGTCGTTTGAGGGCAAACGTGTGACAATCTCCGGA
AGTGGCAATGTTGCGCAATATGCAGCTTTGAAAGTGATCGAGCTGGGTGGTATTGTGGTGTCTTTATCCGATTCG
AAGGGGTGCATCATCTCTGAGACGGGCATTACTTCTGAGCAAATTCACGATATCGCTTCCGCCAAGATCCGTTTC
AAGTCGTTAGAGGAAATCGTTGATGAATACTCTACTTTCAGCGAAAGTAAGATGAAGTACGTTGCAGGAGCACGC
CCATGGACGCATGTGAGCAACGTCGACATTGCCTTGCCCTGTGCCACCCAAAACGAGGTCAGTGGTGACGAAGCC
AAGGCCCTAGTGGCATCTGGCGTTAAGTTCGTTGCCGAAGGTGCTAACATGGGTTCTACACCCGAGGCTATTTCT
GTTTTCGAAACAGCGCGTAGCACTGCAACCAATGCAAAGGATGCAGTTTGGTTTGGGCCACCAAAGGCAGCTAAC
CTGGGCGGCGTGGCAGTATCCGGTCTGGAAATGGCTCAGAATTCTCAAAAAGTAACTTGGACTGCCGAGCGGGTC
GATCAAGAACTAAAGAAGATAATGATCAACTGCTTCAACGACTGCATACAGGCCGCACAAGAGTACTCTACGGAA
AAAAATACAAACACCTTGCCATCATTGGTCAAGGGGGCCAACATTGCCAGCTTCGTCATGGTGGCTGACGCAATG
CTTGACCAGGGAGACGTTTTTTAG

>YLR255C 1.69 Hot
ATGGGTGTGGGTGGAACGAGGATTGTATCGTTTAGACAGCCGAATTATTACCCGGTTACTCAACAAAAAGGGGCA
AGCCAGACAGGTGCAGTGGCTCAGCCGTACAGCTCGTACTGTGGTTTGCTAATGCGCTGGGCGGTTGTTGAGATA
AGAAGAAGAGGGAAAGGGAAAGGAAGAAAGAGGAAGAGAGAGAGAGAGAAAGGGCACACGAAATTCAGGATACGG
CGGAGGAGTTATCTTTATTTTATACGGTCTTGCCTTGTAAGGCCCTACTCAAGCGGGAACAAGAAAAACAGTTGT
AGCTTCCACAAGATGTTGGCTATAGAGATTGTTCTGTGCTTAAAGGCTCGCTAA

>YLR374C 1.75 Hot
ATGTTCATATTAGGCAGTGTCGGATGTGTCGAGGCGGACGAAGCCTCGCCATTATATTGTCTCTCGGCTGCCTTG
ATAAGGTTGTCAAATGACGAGATGGGAGGCAACGTGATGTGGTTCATAGCACTATTGTTTGCATTACTCATCGCT
CGCTGCACATGCCACACAAAAAACACCCACCCTGATTTCTCGAAACCAACTTTTTGTCACCAACATGCAGCGCTT
ACCAACAGTCTGTCTTCTTTATACCGTTGCTTTGTTCCAGATGGTACTGCCATGCTTCCAACGGCTACAAAAAAA
ACACCGCAACGGCGAAAAAATGGCGCAATTATTCACCGTGTAGTCATTTACCACGGCCGGGAAAGCGCGAATGGG
ATCAGCAAACAATAA

>YKL037W 1.92 Hot
ATGCAGACAATGGGCGGGGAGCACCTTCTTCTTTCACAGCTAAAGGGGTCTTTCTTTCTCCTACTTTTGGCATAC
TTTTTCAGGGGCAGAAGTCCTTATTACGCACGTTGCTACCGCCGGCTTGCTGTTACACCTGGTGCCATCACTATT
GCCATTGCCATTGCTACAGATTCAATTCCGGCGCTTGCAAAGTCCAAAGTTCTGGTGTCGGTTTGTTCTCACACA
GATCCCTGTACAGCGTCTTGTAACCTGATCCCCTTCCCCCGCCCCTTCTCGAACAGCCTGACGCGCTTCCTCTTT
TGTTTGGGCTCGGCCCGTTTTTGCATTTCCTTTCCCTGTTTTGGATTGAGTATATAA

>YOR223W 1.65 Hot
ATGTCCGCAGAACCATTATTACCCACACATAACGGATCGCAGGGAGGGGAAGTGAGGTCCCCAGATCAGAAGTTC
ATTGTGATACGGTTTTCCGACGTATCAGTAAGGGATCTGCAGTTGAACATATCAAATGTACCATTTTCGAATATA
AATACCCACTGGCTACGTCGGATGTGTAGAGAATTGAGGCCCCAACAAACACAAAAAAGAAGACTGAAGTTTATA
AGGAACGGGAGTATTCTGAATACGCATTCCAAGATTGCTGAGGAACTGACGCATTACTTCGATACTGCTAATAAC
TCTAACGTCGCAACAGGCACCAGCGTGGCACCAGAGCAGAACAACTACTATATTCATTGTATAATTGGTACAGAG
GAGCTGACACAAGCAGAGTTAGCCAACGAAGATCTAAAGGACGATGCAACTCCGTCCAATGACTCCATGACCACG
CAGGCCATCGGGTTTGATAGGCTACGATCCGTGGGATTTACTGAGCAAGAAATCGAACTGCTGAGGCAACAGTTT
CGGGCCACGTATGGAGATTTGGAGGAGGAGGAGGAGAGACTTGCTCAAAATGGCAACAGAGATGATGAGGGCCAT
GACATTAGACAGTTGGAGGAACAGTGGATGGAAAGCGGGAGTGGGACCGCTCAGGGGAATGGGGCCGGAGGCGGC
AATGAAGACAGGTTTAACTCTGTGCCGATCGCTAACATAAAGCACAACAAAGACTTGCTTTTAGGTATATGTGTC
GGATTCTTCTTCGGCGTCTTTGGTATACTATTAATGAAGTTTGATGGATTGTTCAACAGACGGCAAAAAATGGCG
ATTTTTGCCGGTGTGATTGTTAATGTTATGTTTTGTCTTGTGAGAGGTTTTTAG

>YJR115W 2.33 Hot
ATGTTCACAAATACACGTACAATATTAATATACAATAGTAAGGTGATGAACACACACACACACACACACACACAC
ACACACACACACATATATATATATATACAGGAGATCAAGTTAGTGTGAGGGGACGACTACTGAGTTTGAAGTTCTTT
AAAGTGTTAAAGTTATTTTTCCCCTCTCCCACCTCGTTGGCAACCTCTCACCCACCGCTTAGCAGCATGTCTCCG
TACATGACCATACCTCAGCAATACTTATACATAAGCAAGATACGTTCCAAGCTGTCTCAGTGCGCCCTTACTCGA
CACCACCACAGAGAACTTGATCTACGAAAAATGGTGGGCCACGCCAATATGCTGGACAGGATCCTCGACGAAATA
GACGAAATCGACAGCGAGGTAGTACTGTGTGACGCTGCCGATGGTTCTTCTACTGCAGAAGCTCATTCCGCTTCC
CCAGCATCCAGCGACTCTTCTCCTCTCACTAATAACATCCGGCCCATTAGCATTATGTGA

>YHL006C 1.82 Hot
ATGCAGTTTGAAGAGCGGTTGCAACAGCTAGTAGAAAGCGATTGGAGCCTGGATCAGTCAAGCCCAAACGTACTG
GTGATAGTCCTTGGAGACACTGCCAGGAAGTACGTAGAGTTAGGAGGACTGAAGGAGCACGTGACTACGAACACT
GTGGCAGGTCACGTGGCAAGCCGGGAGCGGGTATCCGTAGTTTTTTTGGGCCGGGTAAAGTACTTATACATGTAC
CTCACCCGGATGCAAGCGCAGGCGAATGGTCCGCAATACTCTAATGTACTCGTCTATGGATTGTGGGATCTCACC
GCGACACAAGATGGACCGCAACAGCTGCGGCTACTTAGCCTTGTGTTACGGCAGTGCCTCAGTCTGCCATCTAAA

GTTGAATTCTATCCAGAACCGCCTTCTAGTAGCGTGCCTGCCCGGCTATTGCGATTCTGGGACCATATTATCAGA
TAG

>YHR019C 5.09 Hot
ATGTCATCTTTGTACATTAAGGAGGCTACCGGTGTAGACGAATTGACCACAGCCGGTTCACAAGACCATCCATTC
AAAACCCCAGCATATGCTTTGTTCGCATCTCAACAGAAGAGCGATGCCACGGAACCTAAGCTTTTCGTGTTTAAG
ACAGAAGACAACGAGTACCAGGAAATCAGTGCTTCTGCTTTGAAGAAGGCTCGTAAGGGCTGTGATGGTTTGAAG
AAAAAGGCAGTCAAGCAAAAGGAACAGGAGTTGAAGAAACAACAAAAAGAGGCAGAAAATGCTGCCAAGCAATTG
TCTGCTTTGAATATCACCATTAAGGAGGACGAATCGCTACCAGCTGCCATTAAGACTAGAATTTATGACTCTTAT
TCCAAGGTCGGACAAAGAGTTAAGGTTTCCGGTTGGATCCATAGATTACGTTCTAACAAGAAGGTTATTTTCGTC
GTCCTCAGAGACGGATCTGGTTTCATTCAATGTGTCTTGTCCGGTGATTTGGCATTGGCTCAACAAACTTTGGAC
CTGACTTTGGAATCCACCGTTACTCTGTACGGTACCATAGTCAAATTGCCTGAGGGTAAAACCGCTCCAGGTGGT
GTTGAATTGAATGTCGACTATTACGAAGTTGTAGGTTTGGCCCCCGGTGGTGAAGACTCCTTTACAAACAAAATC
GCAGAGGGCTCAGACCCTTCTTTACTGTTGGACCAACGTCATTTGGCCTTGAGAGGAGATGCCTTGTCTGCAGTC
ATGAAAGTCCGTGCTGCTCTACTGAAAAGCGTTAGACGTGTTTATGATGAAGAACATTTGACAGAAGTTACCCCA
CCATGTATGGTGCAAACTCAAGTCGAAGGTGGTTCCACTTTGTTCAAGATGAACTATTACGGCGAGGAAGCTTAC
TTGACCCAAAGTTCCCAATTATATTTAGAAACCTGTTTGGCCTCCCTAGGTGATGTTTATACCATCCAAGAATCT
TTCAGAGCTGAAAAGTCCCACACAAGAAGACATTTGTCCGAATATACCCATATCGAAGCTGAATTGGCCTTCTTG
ACTTTCGACGATCTATTACAACATATTGAAACTTTGATCGTCAAATCCGTGCAATACGTTTTGGAAGACCCAATT
GCTGGCCCACTCGTAAAACAATTGAATCCAAACTTTAAGGCTCCAAAGGCTCCATTCATGAGATTACAGTACAAG
GATGCCATTACCTGGTTGAACGAACACGACATCAAGAACGAAGAGGGCGAAGACTTTAAATTTGGTGACGATATT
GCAGAAGCTGCTGAAAGAAAGATGACCGATACCATCGGCGTCCCAATCTTTTTGACGAGATTCCCAGTAGAAATC
AAGTCTTTCTACATGAAGCGTTGTTCTGACGACCCCCGCGTCACTGAATCCGTCGACGTTTTGATGCCAAACGTT
GGTGAAATCACTGGTGGTTCTATGAGAATCGACGACATGGACGAACTAATGGCAGGGTTTAAGCGTGAGGGTATT
GATACCGACGCCTACTACTGGTTCATTGACCAAAGAAAATACGGTACTTGCCCACATGGTGGTTACGGTATCGGT
ACCGAACGTATTTTAGCCTGGTTGTGTGACAGATTCACTGTCAGAGACTGTTCCTTGTATCCACGTTTCAGCGGT
AGATGTAAGCCATGA

>YPR045C 1.61 Hot
ATGCAGAATCCTTACGGTCACTTTACTAACAATACCACGGAGGACAGGGAAGCTTCATCTCAAGGAGGACCATTC
GGCCAAAGCCTGAATAGGCCCCTAGATTACGCCGGATCTTTCCCTTCTCTGACATACAATAATAATAATTTCATT
GCCAATCAACAACCTTCTCTGCCTTTGCCAGAACCACGTTTATCTTGGAACAACGTCAACCAGGTTTCTAACCCT
TTGATGGTAACACCGCTTCCGGGATTACAAAAACGTATGAACAAAAACATTAAAAAGAAATTACCAAGAGTATCT
AAGAAGGCATCAGCCCTTTCTAACGGGGTATCCGGCAATGTAATGAGCAACAGTAATATAGTAGGGCATGGTGCT
GTAGGTTCGGCATCGGGATGGAAAGTTGAAATGGGTGGCAGTGATGAATTAGAGAGAAGAAAGAGAAGGGCAGAA
AGATTCAGTCAGGGACCATCAGCGACGACGAATAGTAACGATAATTTGAATGAAGACTTTGCCAACCTAAATGCC
ATTAGTAGCAAATCTCATCAATATGACAAAAAGATTCATGTGGTAGGGCGTTGCCAAACTCTAGAGAAATCTTAT
CTGAGACTAACTTCAGAGCCTAATCCTGACTTGATCAGACCCCCAAATATTCTACAAAAAATGTATTGCTTGTTA
ATGGACAAATACCAATCTAAAACAGCCACTTATACTTACCTTTGCGACCAGTTTAAATCAATGAGACAAGACCTA
AGAGTGCAAATGATCGAGAACTCTTTCACTATTAAAGTGTACCAAACGCATGCACGTATTGCCTTGGAGAACGGA
GATCTTGGGGAATTTAATCAATGTCAAAATAGGATTATGGCGTTATTCGAAAACCCTACAATACCAAAAAAATCT
TATTCCGAATTTATATGCTATTCTGTTTTGTATTCGATGTTAACCGAAGACTATCCGTCGATTTCTCATTTAAAA
CTTAAGTTAATAGACGATGGCTCTTCGGAAATCTTGGAGGACGAACATGTCAAAATGATATTTGAGTTGAGCGAT
ATGAAACTAGTCGGCAATTATCACTACTTCATGAAAAACTACCTAAAACTGCACAAATTTGAAAAATGTCTAATT
AATTCATTCTTAAATCTAGAAAAGTTGATATTTTTGACCATAATTTGTAAGAGTTATAACCAGGTCAATTTAGAT
TTTGTTAAAAGTGAGTTTAATTTCAACAGTATAGAAGAGACAACAAATTTCTTGAATGAGCAAAACTTGACGGAG
TTCATATTAAACAAACAAATCACTGACAGTAATGGCAAAAGTAGTAATATCAAGATTTTGAATACCAAGGGATGT
AGGGTACAGTTGATACAAAATTATATGAAGTCTAAAAAAATTGATATAAAGGGACAAAAATGA

>YBR009C 1.51 Hot
ATGTCCGGTAGAGGTAAAGGTGGTAAAGGTCTAGGTAAAGGTGGTGCCAAGCGTCACAGAAAGATTCTAAGAGAT
AACATCCAAGGTATTACTAAGCCAGCTATCAGAAGATTAGCTAGAAGAGGTGGTGTCAAGCGTATTTCTGGTTTG
ATCTACGAAGAAGTCAGAGCTGTCTTGAAATCCTTCTTGGAATCCGTCATCAGAGACTCTGTTACCTACACCGAA
CACGCCAAGAGAAAGACTGTTACTTCTTTGGATGTTGTTTATGCTTTGAAGAGACAAGGTAGAACCTTATACGGT
TTCGGTGGTTAA

>YLR307W 1.52 Hot
ATGAAAATTTTCAATACAATACAATCTGTGCTGTTCGCAGCATTTTTTCTAAAACAGGGAAATTGCCTTGCGTCA
AATGGGAGTACCGCATTGATGGGGGAAGTAGATATGCAAACGCCCTTTCCAGAGTGGTTAACAGAATTTACTAAT
CTTACACAATGGCCTGGAATTGACCCACCTTATATTCCGCTAGATTACATAAATCTTACTGAAGTGCCAGAATTA
GATAGGTACTATCCTGGCCAGTGTCCCAAAATTTCTAGAGAGCAGTGCTCATTTGACTGCTATAACTGCATCGAT
GTTGATGATGTAACTTCGTGTTTCAAACTTTCCCAAACATTTGACGACGGTCCGGCCCCGGCGACAGAGGCATTG
CTCAAGAAATTGAGACAAAGAACCACTTTTTTTGTTCTGGGGATAAACACTGTTAATTATCCTGATATATATGAG
CATATTTTAGAGAGGGGTCATTTGATTGGTACACACACGTGGTCACATGAATTCTTGCCAAGTTTATCAAACGAA
GAAATTGTAGCCCAAATTGAATGGTCAATTTGGGCTATGAATGCCACAGGCAAACATTTCCCCAAGTATTTTAGG
CCTCCATACGGTGCAATTGATAATAGGGTTAGAGCTATAGTAAAACAGTTTGGCCTAACGGTTGTCTTGTGGGAT
CTCGATACTTTTGATTGGAAATTAATCACTAATGATGATTTCAGAACAGAGGAAGAAATACTTATGGACATAAAT

ACTTGGAAGGGAAAACGGAAAGGTTTGATCTTAGAGCACGATGGTGCACGAAGAACAGTTGAGGTTGCTATTAAA
ATCAACGAACTTATTGGTAGTGACCAATTGACAATTGCAGAATGTATTGGTGATACAGACTACATCGAACGCTAC
GACTAG

>YNR014W 1.72   Hot
ATGTCGTCCACGGATATCAAGCCTTGTGCCGTGAACATCCCTGTTTCCGCTCACATCACCTTCCACTACAAGTCG
ATCGCGGACAGAAGCAGCAGCAGGAGCAGCAGCAGCAGCTCCTGCTCCTCTGCTACATCTAAAGCCTGCTCCCCC
AGAGGCAGCTCCGTCGGCTTACCGCCTGCTTTATCAACTGACAATGAAATCGTCGAGACTGTACTTAATGTTTCC
GCTCCCGTTGTAGCAGACCCTACGCGGCCCTCATTGTTCAAGAGTAACTACACCGCAGCCTCGTGCTTGACTTCT
GACCCAACCTCGCCGTCACTACTGCCCTCTTCTAGGCGCAACTCTGTATTGCCGGCAAGCGACTTCCACCAATGC
GCGCATCACAAAAACTTTCAAAGAAGGGCCAGTGAGCCACAACTGCCCTCCTTCGATAATCGCTCGTCGTCCGAG
ATGAAAAGGTCCGTCTCGTACGCCCAGCATAGCATGATGTTCCCCATCAGTGACCAACAGGAGCCGCAAACATCC
GCGTCTCCTAACGACCACTCAGACCCGTCTTGTCCTTGCAACAGACATCACCACAGAAGAAACTCGGTCGCCGTC
AAGTTTGACAAACCATTGTATGAGAGACTAGAGACGTAG

>YGR060W 1.89   Hot
ATGTCTGCCGTTTTCAACAACGCTACCCTTTCAGGTCTAGTCCAAGCAAGCACCTACTCACAAACTTTGCAAAAT
GTCGCCCATTACCAACCTCAATTGAATTTCATGGAGAAATACTGGGCCGCATGGTACAGTTACATGAACAATGAT
GTTTTGGCCACCGGTCTAATGTTCTTTTTATTGCATGAATTTATGTATTTCTTTAGATGTTTGCCATGGTTCATC
ATCGACCAAATTCCATACTTTAGAAGATGGAAGTTACAACCAACTAAGATTCCAAGTGCTAAGGAACAACTATAC
TGTTTGAAATCCGTTCTTCTATCTCATTTCTTGGTCGAGGCCATCCCTATCTGGACCTTCCACCCAATGTGTGAA
AAATTAGGTATTACTGTCGAAGTTCCATTCCCATCTTTGAAAACAATGGCTCTAGAAATTGGTCTATTCTTCGTC
TTGGAAGATACATGGCATTACTGGGCTCACCGTCTATTCCACTACGGTGTCTTCTACAAGTACATTCACAAGCAA
CATCACAGATACGCTGCTCCATTCGGTCTTTCTGCTGAATATGCTCATCCTGCTGAAACTTTGTCTTTGGGTTTT
GGTACCGTTGGTATGCCAATTCTTTACGTCATGTACACTGGTAAATTACACTTGTTCACTCTATGTGTATGGATC
ACCCTAAGATTATTCCAAGCTGTTGACTCTCATTCTGGTTATGACTTCCCATGGTCTTTGAACAAGATCATGCCA
TTCTGGGCTGGCGCTGAACACCACGATTTGCATCATCACTACTTTATTGGTAACTACGCTTCCTCTTTCAGATGG
TGGGATTACTGTCTAGACACTGAATCTGGTCCAGAAGCTAAGGCCTCCAGAGAAGAAAGAATGAAGAAGAGAGCT
GAAAACAATGCTCAAAAGAAGACTAACTAA

>YOL086C 1.75   Hot
ATGTCTATCCCAGAAACTCAAAAAGGTGTTATCTTCTACGAATCCCACGGTAAGTTGGAATACAAAGATATTCCA
GTTCCAAAGCCAAAGGCCAACGAATTGTTGATCAACGTTAAATACTCTGGTGTCTGTCACACTGACTTGCACGCT
TGGCACGGTGACTGGCCATTGCCAGTTAAGCTACCATTAGTCGGTGGTCACGAAGGTGCCGGTGTCGTTGTCGGC
ATGGGTGAAAACGTTAAGGGCTGGAAGATCGGTGACTACGCCGGTATCAAATGGTTGAACGGTTCTTGTATGGCC
TGTGAATACTGTGAATTGGGTAACGAATCCAACTGTCCTCACGCTGACTTGTCTGGTTACACCCACGACGGTTCT
TTCCAACAATACGCTACCGCTGACGCTGTTCAAGCCGCTCACATTCCTCAAGGTACCGACTTGGCCCAAGTCGCC
CCCATCTTGTGTGCTGGTATCACCGTCTACAAGGCTTTGAAGTCTGCTAACTTGATGGCCGGTCACTGGGTTGCT
ATCTCCGGTGCTGCTGGTGGTCTAGGTTCTTTGGCTGTTCAATACGCCAAGGCTATGGGTTACAGAGTCTTGGGT
ATTGACGGTGGTGAAGGTAAGGAAGAATTATTCAGATCCATCGGTGGTGAAGTCTTCATTGACTTCACTAAGGAA
AAGGACATTGTCGGTGCTGTTCTAAAGGCCACTGACGGTGGTGCTCACGGTGTCATCAACGTTTCCGTTTCCGAA
GCCGCTATTGAAGCTTCTACCAGATACGTTAGAGCTAACGGTACCACCGTTTTGGTCGGTATGCCAGCTGGTGCC
AAGTGTTGTTCTGATGTCTTCAACCAAGTCGTCAAGTCCATCTCTATTGTTGGTTCTTACGTCGGTAACAGAGCT
GACACCAGAGAAGCTTTGGACTTCTTCGCCAGAGGTTTGGTCAAGTCTCCAATCAAGGTTGTCGGCTTGTCTACC
TTGCCAGAAATTTACGAAAAGATGGAAAAGGGTCAAATCGTTGGTAGATACGTTGTTGACACTTCTAAATAA

>YCL009C 1.68   Hot
ATGCTGAGATCGTTATTGCAAAGCGGCCACCGCAGGGTGGTTGCTTCTTCATGTGCTACCATGGTGCGTTGCAGT
TCCTCGTCGACCTCCGCGTTGGCGTACAAGCAGATGCACAGACACGCAACAAGACCTCCCTTGCCCACACTAGAC
ACTCCTTCCTGGAATGCCAACAGTGCCGTTTCATCCATCATTTACGAAACACCAGCGCCTTCTCGTCAACCAAGA
AAACAGCATGTCTTGAACTGTTTGGTGCAAAACGAACCCGGTGTCTTGTCCAGAGTCTCGGGTACGTTAGCTGCC
AGAGGCTTTAACATCGATTCGTTGGTCGTGTGCAACACCGAGGTCAAAGACCTAAGTAGAATGACCATTGTTTTG
CAAGGGCAAGATGGCGTAGTCGAACAAGCACGCAGACAAATCGAAGACTTGGTCCCCGTCTACGCCGTCCTAGAC
TATACCAATTCTGAGATCATCAAAAGAGAGCTAGTGATGGCCAGAATCTCTCTATTGGGTACTGAATACTTCGAA
GACCTACTATTGCACCACCACACTTCCACCAATGCTGGCGCCGCTGACTCCCAAGAATTGGTCGCCGAAATCAGA
GAAAAGCAATTCCACCCTGCCAACTTGCCCGCCAGTGAGGTATTAAGGTTGAAGCACGAGCATTTGAACGATATC
ACCAACTTGACCAACAACTTTGGAGGTCGTGTCGTCGACATCAGCGAAACAAGCTGTATTGTGGAGTTGTCTGCA
AAACCCACACGTATCTCTGCCTTCTTGAAGTTGGTCGAGCCATTCGGTGTCCTAGAGTGTGCAAGAAGCGGTATG
ATGGCATTGCCAAGAACTCCTTTGAAGACAAGCACCGAGGAAGCTGCCGACGAAGACGAAAAGATCAGCGAAATC
GTCGACATTTCCCAACTACCACCTGGTTAG

>YPL055C 1.63   Hot
ATGAGCGGATACACGGGAAATAATTATAGCAGATACTCATCCACTCCTCCGAGACAAAGGGGGGGTTATCACCAT
GCGAGACGAAGTAGAGGTGGAGCAGGCGGATCGTATTACAGAGGCGGTAATGCGTCTTATGGCGCGAGATACAAC
AGTGACTATGAGCAGCCGCCCCAAGAGGGAGACTTGAGACAAACCGGAGCATACTATCGAAACGGATACACAGAT
ACAAGGCCGTACTATTCAGCTAATAGCAGGCATTACCAAGCACAACCATCCCCCAGGTACAACAATGGTACAAAT
TCGTACCACCTACCGCAACGTGGCAATAGTCAAGATACGAACGGCAGGACCACGAGCGCCTCGCAAGAAGATAAT

GACGAAAAAAGAGTCAAGTCCAGATACCGGAACATGCAGGCAGATCATCCACGCCAACAACCGATGAGCGTTGGC
AGCACCAGCAGCAGAAATGGTAGTAGTGGGAACAGCAGCACTAGCAGCACCAGCAACGGGCTTCCGCCACCACCT
TCAGTATCGTCTATAACCAACAATAGATCATATCACAGCAGTGCTTATCCATATTCCAGCAGCCATACTTACAAT
AATTACCACCACCGTGAGACACCACCGCCTCCCCCATCGAATGGCTATTATGCAAAGGGTTACCCAGTACACGTA
CCAGAAAATAGAAGTAACAGCGACGGCTCTAGCAGCAGCGTCGTCAAGAAGAAAAGAATACTAGATATGAAAGAT
TCCCCCTTTATTTACTTGACAGATTTTGATAAAAATGTGAAAAAGACAAACAACACCGAAAGTGAATGTGAAAAG
GCGAGGGAAGTTTTCAAAGAGAGCGATTCCATCGATTCCGCTTTGGAAGAACTAAACCTAAAAATCAATTCTAAT
GAGCTAGAATTGCGGCTCCTAAATAACCAGTGCGACAAGCATGCATTAAATATTCAACTGACCCAAGAAAAGCTG
GACTCATTGTTATTAATGCAGTAG

>YPR037C 1.96   Hot
ATGAAACAGATAGTCAAAAGAAGCCATGCCATCAGAATAGTTGCAGCATTAGGAATCATAGGCCTGTGGATGTTT
TTCTCGTCTAATGAACTATCCATCGCTACGCCGGGCCTAATCAAGGCGAAGTCTGGTATAGATGAAGTGCAAGGG
GCGGCTGCTGAGAAGAACGACGCTCGGTTGAAAGAGATCGAGAAGCAAACCATTATGCCATTGATGGGCGATGAC
AAGGTGAAGAAGGAAGTGGGCAGGGCGTCGTGGAAGTACTTCCATACCCTGCTGGCCCGTTTTCCGGACGAGCCT
ACTCCTGAAGAAAGAGAGAAACTGCACACGTTTATTGGGTTGTATGCAGAACTCTATCCATGCGGGGAATGTTCA
TATCACTTTGTAAAGTTGATTGAGAAGTATCCCGTACAGACATCTAGCAGGACGGCTGCCGCAATGTGGGGATGC
CACATTCACAACAAGGTGAACGAATACCTAAAGAAAGACATATATGACTGTGCTACCATCCTGGAGGACTACGAT
TGTGGATGTAGTGACAGCGACGGTAAACGCGTGTCTCTCGAGAAGGAGGCTAAACAGCACGGTTGA

>YER145C 1.84   Hot
ATGCCTAACAAAGTGTTTAACGTGGCCGTTTTCTTCGTTGTGTTCAGAGAGTGCTTGGAAGCAGTGATTGTTATT
TCCGTGCTGCTATCGTTTTTGAAACAGGCAATCGGGGAACATGACCGGGCGCTGTACCGTAAATTAAGGATTCAG
GTTTGGGTCGGTGTGTTACTCGGGTTCATCATTTGTCTGGCCATCGGTGCAGGTTTCATCGGTGCGTACTATTCG
CTGCAGAAGGATATTTTCGGAAGCGCGGAAGACTTGTGGGAAGGTATCTTTTGTATGATTGCGACGATAATGATC
TCGATGATGGGTATTCCTATGTTGAGAATGAATAAGATGCAGAGTAAGTGGAGAGTGAAGATTGCGCGGTCGTTG
GTGGAGATTCCTCACCGTAAGAGAGACTATTTCAAGATTGGGTTCTTGAGTAGAAGATATGCTATGTTCCTTTTG
CCGTTTATTACTGTCTTGAGAGAAGGTTTGGAAGCTGTTGTGTTCGTTGCCGGTGCAGGTATTACCACACAGGGC
TCGCACGCCTCTGCTTACCCATTGCCCGTTGTGGTTGGTTTGATATGTGGTGGTCTTGTTGGGTATTTGTTGTAC
TATGGTGCTTCGAAATCCTCGCTGCAGATTTTCTTGATTTTGTCTACTTCGATCTTGTACCTGATCTCCGCCGGT
CTTTTCTCTCGTGGTGCGTGGTACTTTGAAAATTACAGATTCAACCTTGCCAGTGGTGGTGATGCTTCCGAAGGC
GGTGATGGTAATGGGTCTTATAACATCAGAAAGGCCGTTTACCATGTCAATTGTTGCAACCCAGAACTGGACAAT
GGGTGGGATATCTTCAATGCTTTGCTTGGATGGCAGAACACCGGCTACTTGTCATCCATGCTTTGTTACAACATC
TATTGGTTGGTTCTTATCATTGTGTTGAGTTTAATGATATTCGAAGAAAGGCGTGGTCACTTGCCTTTCACCAAA
AACTTACAATTGAAACACTTGAACCCAGGATACTGGATCAAGAACAAGAAAAAGCAGGAGTTGACGGAAGAGCAA
AAGAGACAACTGTTTGCCAAGATGGAGAACATCAACTTCAATGAAGATGGTGAGATCAATGTGCAAGAGAACTAC
GAACTTCCCGAGCAAACTACCAGTCATTCCTCGTCTCAAAACGTCGCCACAGACAAGGAAGTCCTCCACGTTAAA
GCCGACTCTCTTTGA

>YDL079C 1.61   Hot
ATGACCGATGTGTTGAGAAGCCTAGTGCGAAAGATTTCCTTCAATAACTCAGATAACCTTCAGCTAAAACACAAG
ACATCTATACAGAGTAATACTGCCCTAGAAAAGAAGAAGAGAAAGCCTGATACAATCAAAAAGGTAAGCGACGTT
CAAGTTCACCATACTGTACCTAATTTCAATAACAGTTCAGAGTACATCAACGACATAGAGAATCTTATAATATCT
AAACTGATAGACGGGGGAAAGGAGGGTATTGCAGTTGATCACATTGAACACGCCAATATCTCGGACAGTAAAACA
GATGGAAAAGTTGCCAATAAGCACGAAAATATTAGTAGCAAGCTTAGTAAAGAGAAGGTTGAGAAAATGATTAAT
TTTGATTATAGGTATATTAAAACCAAGGAAAGGACTATTCATAAACGGGTTTATAAACATGACCGCAAAACTGAT
GTCGACCGAAAAAATCATGGAGGAACTATCGACATCAGTTATCCTACGACAGAAGTGGTTGGCCATGGTTCATTT
GGTGTTGTAGTCACGACTGTAATAATTGAGACCAATCAAAAAGTTGCCATAAAGAAAGTACTACAAGATAGAAGA
TATAAAAATAGGGAGCTCGAAACTATGAAGATGTTGTGCCATCCAAATACTGTGGGTCTACAGTACTACTTTTAC
GAAAAGGACGAAGAAGATGAAGTATACCTCAATCTGGTTTTGGACTACATGCCTCAGTCGTTATACCAAAGGCTT
CGTCATTTTGTTAATTTGAAAATGCAGATGCCGCGTGTTGAAATTAAATTCTATGCATACCAACTATTCAAAGCT
TTAAACTATTTGCATAACGTTCCTCGAATCTGTCACAGAGATATAAAACCGCAAAACCTACTGGTGGATCCGACA
ACTTTTTCTTTCAAGATTTGCGATTTTGGCAGTGCCAAATGCTTGAAACCGGATCAGCCTAATGTGTCTTACATC
TGTTCAAGGTACTATAGGGCTCCCGAACTCATGTTTGGTGCCACTAATTACTCAAACCAGGTCGACGTGTGGTCA
AGCGCTTGTGTCATTGCTGAGTTGCTTTTGGGCAAGCCCTTGTTCTCTGGTGAAAGCGGTATAGATCAGTTGGTG
GAAATTATTAAGATAATGGGCATACCCACAAAGGATGAAATTTCAGGAATGAACCCAAATTATGAAGACCATGTT
TTCCCCAATATCAAGCCCATTACTTTGGCTGAAATATTCAAAGCCGAAGATCCCGATACTCTTGACTTGTTAACA
AAAACTCTGAAGTATCACCCTTGCGAAAGATTGGTACCTCTACAATGTCTATTATCAAGCTATTTTGACGAAACC
AAACGTTGTGATACCGACACTTACGTAAAAGCACAAAACCTGCGTATATTTGACTTCGACGTGGAAACTGAGTTG
GGCCATGTTCCACTTGTGGAACGGCCCGCCATTGAAGAACGGTTGAAACATTTTGTTTCTGCACCTTCATCGTCT
TTGTGA

>YNL036W 2.33   Hot
ATGAGCGCTACCGAATCTTCATCTATATTCACATTGAGTCACAACTCAAACCTACAAGATATCTTGGCCGCCAAT
GCCAAATGGGCCTCCCAGATGAACAACATACAGCCAACTTTGTTCCCAGATCACAATGCGAAGGGCCAGTCCCCT
CACACTCTTTTCATCGGCTGCTCCGATTCGCGTTACAACGAAAACTGTTTAGGTGTCTTGCCCGGCGAAGTGTTC
ACTTGGAAAAATGTTGCTAACATATGTCACTCAGAGGATTTAACTTTGAAGGCCACTTTAGAGTTTGCCATTATT

TGTCTAAAAGTTAACAAAGTTATTATTTGTGGCCACACTGATTGTGGTGGTATAAAGACATGTTTAACTAACCAA
AGGGAAGCCTTACCAAAAGTTAACTGTTCTCATCTGTACAAGTACTTAGACGATATTGACACCATGTACCATGAA
GAGTCACAAAATTTGATCCATTTGAAAACGCAACGTGAAAAATCTCATTACCTGTCGCACTGTAACGTCAAAAGG
CAGTTTAATAGGATTATTGAAAACCCTACTGTGCAAACTGCTGTACAAAATGGAGAATTACAGGTATACGGTCTG
CTTTACAACGTAGAGGACGGTCTACTGCAAACAGTTAGCACTTACACAAAAGTTACCCCAAAATAG

>YDL131W 1.56 Hot
ATGTCTGAAAATAACGAATTCCAGAGTGTCACCGAATCGACGACTGCTCCAACCACTAGTAACCCATATGGCCCA
AATCCTGCGGATTATCTATCCAATGTTAAGAATTTCCAGTTGATTGATTCAACACTAAGAGAGGGTGAACAATTT
GCCAACGCATTCTTCGATACTGAAAAAAAGATTGAAATTGCTAGAGCCTTGGATGATTTCGGTGTGGACTACATC
GAGTTAACCTCTCCCGTAGCATCCGAACAATCAAGAAAGGACTGTGAAGCTATATGTAAACTAGGTTTAAAGGCC
AAGATCCTTACACACATTCGTTGTCACATGGACGATGCCAGAGTCGCCGTAGAGACTGGTGTCGACGGTGTCGAT
GTTGTTATCGGCACCTCCAAATTTTTAAGACAATATTCCCACGGTAAGGATATGAACTACATCGCCAAGAGTGCT
GTTGAAGTCATTGAATTTGTCAAATCCAAAGGTATTGAAATCAGATTTTCCTCTGAAGATTCCTTCAGAAGTGAT
CTCGTTGATCTTTTGAACATTTATAAAACCGTTGACAAGATCGGTGTAAATAGAGTCGGTATTGCCGACACAGTT
GGATGTGCCAACCCAAGACAAGTATATGAACTGATCAGAACTTTGAAGAGTGTTGTCTCATGTGACATCGAATGC
CATTTCCACAATGATACCGGTTGTGCCATTGCAAACGCCTACACTGCTTTGGAAGGTGGTGCCAGATTGATTGAC
GTCAGTGTACTGGGTATTGGTGAAAGAAACGGTATCACTCCTCTAGGTGGGCTCATGGCAAGAATGATTGTTGCC
GCACCAGACTATGTCAGATCTAAATACAAGCTGCACAAGATCAGAGACATCGAAAACCTGGTCGCTGATGCTGTG
GAAGTTAACATTCCATTCAACAACCCTATCACCGGGTTCTGTGCATTCACACATAAAGCAGGTATCCATGCCAAG
GCCATTTTGGCTAACCCATCTACCTACGAAATCTTGGACCCTCACGATTTCGGTATGAAGAGATATATCCACTTC
GCCAACAGACTAACTGGTTGGAATGCAATCAAATCAAGAGTCGACCAATTGAACTTGAATTTGACGGATGATCAA
ATCAAGGAAGTTACTGCTAAGATTAAGAAGCTGGGTGATGTCAGACCGCTAAATATTGATGATGTAGACTCCATT
ATCAAGGACTTCCATGCAGAATTGAGCACCCCACTTTTAAAACCAGTAAATAAGGGTACAGATGACGACAATATC
GATATTTCCAATGGGCATGTTTCTAAAAAGGCAAAGGTCACCAAATAG

>YBR051W 1.62 Hot
ATGCACATTCTTTTCTTGTTTATTTTCCACTGTTTGGCATTCAAAGACTTGATTTTTTTTAAGCAATACGTACCA
TTTGCAGCGGCGGGAGGATATCCCATATCGTTTCTATTCATCAAAGTTCTGACCGCCTCAACGAATTTACTACTT
TCTTCTTCCTCTGGAGGGTCCTGGAATAAGTTATCCAAAGAGTCGCAATTACTCAAAGTCATTCTCACACACTTT
CTTGTTCCTATTTTTTTTTTCTTATTCCAATATATTATTTTATCAGAAGACAGACAGCAAGAACGACAGCCTAAG
TTTCGAGACAACGCTAAATTTGACGGACATGCGAAGACATGCCATATATAA

>YNL241C 2.22 Hot
ATGAGTGAAGGCCCCGTCAAATTCGAAAAAAATACCGTCATATCTGTCTTTGGTGCGTCAGGTGATCTGGCAAAG
AAGAAGACTTTTCCCGCCTTATTTGGGCTTTTCAGAGAAGGTTACCTTGATCCATCTACCAAGATCTTCGGTTAT
GCCCGGTCCAAATTGTCCATGGAGGAGGACCTGAAGTCCCGTGTCCTACCCCACTTGAAAAAACCTCACGGTGAA
GCCGATGACTCTAAGGTCGAACAGTTCTTCAAGATGGTCAGCTACATTTCGGGAAATTACGACACAGATGAAGGC
TTCGACGAATTAAGAACGCAGATCGAGAAATTCGAGAAAAGTGCCAACGTCGATGTCCCACACCGTCTCTTCTAT
CTGGCCTTGCCGCCAAGCGTTTTTTTGACGGTGGCCAAGCAGATCAAGAGTCGTGTGTACGCAGAGAATGGCATC
ACCCGTGTAATCGTAGAGAAACCTTTCGGCCACGACCTGGCCTCTGCCAGGGAGCTGCAAAAAAACCTGGGGCCC
CTCTTTAAAGAAGAAGAGTTGTACAGAATTGACCATTACTTGGGTAAAGAGTTGGTCAAGAATCTTTTAGTCTTG
AGGTTCGGTAACCAGTTTTTGAATGCCTCGTGGAATAGAGACAACATTCAAAGCGTTCAGATTTCGTTTAAAGAG
AGGTTCGGCACCGAAGGCCGTGGCGGCTATTTCGACTCTATAGGCATAATCAGAGACGTGATGCAGAACCATCTG
TTACAAATCATGACTCTCTTGACTATGGAAAGACCGGTGTCTTTTGACCCGGAATCTATTCGTGACGAAAAGGTT
AAGGTTCTAAAGGCCGTGGCCCCCATCGACACGGACGACGTCCTCTTGGGCCAGTACGGTAAATCTGAGGACGGG
TCTAAGCCCGCCTACGTGGATGATGACACTGTAGACAAGGACTCTAAATGTGTCACTTTTGCAGCAATGACTTTC
AACATCGAAAACGAGCGTTGGGAGGGCGTCCCCATCATGATGCGTGCCGGTAAGGCTTTGAATGAGTCCAAGGTG
GAGATCAGACTGCAGTACAAAGCGGTCGCATCGGGTGTCTTCAAAGACATTCCAAATAACGAACTGGTCATCAGA
GTGCAGCCCGATGCCGCTGTGTACCTAAAGTTTAATGCTAAGACCCCTGGTCTGTCAAATGCTACCCAAGTCACA
GATCTGAATCTAACTTACGCAAGCAGGTACCAAGACTTTTGGATTCCAGAGGCTTACGAGGTGTTGATAAGAGAC
GCCCTACTGGGTGACCATTCCAACTTTGTCAGAGATGACGAATTGGATATCAGTTGGGGCATATTCACCCCATTA
CTGAAGCACATAGAGCGTCCGGACGGTCCAACACCGGAAATTTACCCCTACGGATCAAGAGGTCCAAAGGGATTG
AAGGAATATATGCAAAAACACAAGTATGTTATGCCCGAAAAGCACCCTTACGCTTGGCCCGTGACTAAGCCAGAA
GATACGAAGGATAATTAG

>YJR075W 1.72 Hot
ATGGCCAAAACAACAAAAAGAGCCTCCAGTTTCAGGAGGTTGATGATATTCGCCATAATAGCCCTCATCTCATTA
GCATTTGGAGTTAGATACCTATTTCACAATTCTAATGCTACTGATTTACAAAAAATTCTGCAGAACTTGCCCAAA
GAGATTTCCCAAAGCATTAATAGTGCCAACAATATTCAGAGTTCGGATTCTGATCTAGTTCAACATTTTGAGAGT
TTGGCTCAGGAGATCAGACACCAACAGGAAGTTCAAGCAAAGCAATTTGATAAACAACGTAAGATCCTGGAAAAA
AAAATCCAAGACTTGAAACAAACACCTCCGGAGGCCACCCTAAGAGAACGCATAGCTATGACTTTCCCCTACGAT
TCCCATGTCAAGTTCCCAGCATTTATTTGGCAAACTTGGTCCAATGATGAAGGTCCCGAGCGTGTTCAAGATATA
AAGGGCATGTGGGAAAGCAAGAATCCGGGCTTTGCGCACGAAGTGTTGAACCATGACGTGATAAACGCACTAGTA
CACCACTACTTCTACTCCATACCGGAAATCCTAGAGACTTACGAAGCTCTGCCCTCCATCATCCTAAAGATAGAT
TTTTTCAAATACTTAATACTGTTAGTTCATGGAGGTGTTTATGCTGACATCGACACGTTCCCTGTTCAGCCAATT
CCAAACTGGATTCCTGAAGAGTTGTCGCCATCCGACATTGGGTTGATAGTTGGAGTTGAGGAAGACGCTCAAAGA

GCTGACTGGAGAACCAAGTATATCAGAAGACTTCAGTTTGGTACTTGGATTATACAAGCAAAACCTGGTCACCCT
GTTTTGAGGGAAATCATTTCTCGAATTATTGAGACCACTTTACAGAGAAAGAGGGACGACCAACTAAACGTCAAT
CTAAGGAATGATCTGAATATTATGAGTTGGACGGGTTCTGGGTTGTGGACTGACACTATTTTCACGTATTTCAAT
GACTTTATGAGAAGTGGTGTCAGGGAGAAGGTTACATGGAAATTATTCCATAACCTAAATCAACCAAAATTGCTA
AGTGATGTTCTGGTCTTTCCAAAATTCTCCTTTAACTGTCCAAACCAAATCGATAATGACGATCCACACAAGAAA
TTCTATTTCATTACTCATTTGGCATCACAATTTTGGAAAAATACTCCAAAGGTGGAGCAGAAATAA

>YDR187C 2.63 Hot
ATGAAACGGTGCGCTTGTCTAAGCAATTCACCCACAAGACACACAACCGTCGTGGTCCCATCGCCCGTGATTTCA
TCCTGCGCGGCAGCTGCCCTAGCAATCAGTACCGCTGTAGGCGACTGGATCTGCATCTCGGTCAGCAACACTTTA
CCGTCCTTGGTCAGCTTGATGTTACCAGCACCATCCACAAGCATCTTTAGCGTGCCCTTAGGGCCCAAGTTGGTC
TCTAGGACGGATTGTAAGCCCTCAGCAGATGTGACGTTAACCTTCAAAGCCGCATCCCTTCTCAACGATTCAGCC
TTCGGATTCAACAATTGCAATGACATGCTTCTACTCTACTTATATTATAATCTTCTGCTTCTCACTGCTTCCACA
CCCTTAACCTTTACCTTTTCCACACACTGCACACATACACTGTTATACATAATCCTTTCCGTCTTTCGTTCGTTT
CTTACCAGAATAACAACTACTAACACATCACCGAGCGAAAATTTTGCCACCAAAGCAAAACTCCCGTAA

>YGR155W 2.13 Hot
ATGACTAAATCTGAGCAGCAAGCCGATTCAAGACATAACGTTATCGACTTAGTTGGTAACACCCCATTGATCGCA
CTGAAAAAATTGCCTAAGGCTTTGGGTATCAAACCACAAATTTATGCTAAGCTGGAACTATACAATCCAGGTGGT
TCCATCAAAGACAGAATTGCCAAGTCTATGGTGGAAGAAGCTGAAGCTTCCGGTAGAATTCATCCTTCCAGATCT
ACTCTGATCGAACCTACTTCTGGTAACACCGGTATCGGTCTAGCTTTAATCGGCGCCATCAAAGGTTACAGAACT
ATCATCACCTTGCCGGAAAAAATGTCTAACGAGAAAGTTTCTGTCCTAAAGGCTCTGGGTGCTGAAATCATCAGA
ACTCCAACTGCTGCTGCCTGGGATTCTCCAGAATCACATATTGGTGTTGCTAAGAAGTTGGAAAAAGAGATTCCT
GGTGCTGTTATACTTGACCAATATAACAATATGATGAACCCAGAAGCTCATTACTTTGGTACTGGTCGCGAAATC
CAAAGACAGCTAGAAGACTTGAATTTATTTGATAATCTACGCGCTGTTGTTGCTGGTGCTGGTACTGGTGGGACT
ATTAGCGGTATTTCCAAGTACTTGAAAGAACAGAATGATAAGATCCAAATCGTTGGTGCTGACCCATTCGGTTCA
ATTTTAGCCCAACCTGAAAACTTGAATAAGACTGATATCACTGACTACAAAGTTGAGGGTATTGGTTATGATTTT
GTTCCTCAGGTTTTGGACAGAAAATTAATTGATGTTTGGTATAAGACAGACGACAAGCCTTCTTTCAAATACGCC
AGACAATTGATTTCTAACGAAGGTGTCTTGGTGGGTGGTTCTTCCGGTTCTGCCTTCACTGCGGTTGTGAAATAC
TGTGAAGACCACCCTGAACTGACTGAAGATGATGTCATTGTTGCCATATTCCCAGATTCCATCAGGTCGTACCTA
ACCAAATTCGTCGATGACGAATGGTTGAAAAAGAACAATTTGTGGGATGATGACGTGTTGGCCCGTTTTGACTCT
TCAAAGCTGGAGGCTTCGACGACAAAATACGCTGATGTGTTTGGTAACGCTACTGTAAAGGATCTTCACTTGAAA
CCGGTTGTTTCCGTTAAGGAAACCGCTAAGGTCACTGATGTTATCAAGATATTAAAAGACAATGGCTTTGACCAA
TTGCCTGTGTTGACTGAAGACGGCAAGTTGTCTGGTTTAGTTACTCTCTCTGAGCTTCTAAGAAAACTATCAATC
AATAATTCAAACAACGACAACACTATAAAGGGTAAATACTTGGACTTCAAGAAATTAAACAATTTCAATGATGTT
TCCTCTTACAACGAAAATAAATCCGGTAAGAAGAAGTTTATTAAATTCGATGAAAACTCAAAGCTATCTGACTTG
AATCGTTTCTTTGAAAAAAACTCATCTGCCGTTATCACTGATGGCTTGAAACCAATCCATATCGTTACTAAGATG
GATTTACTGAGCTACTTAGCATAA

>YLR314C 1.52 Hot
ATGAGTTTAAAGGAGGAACAAGTGTCCATTAAGCAGGACCCGGAACAAGAAGAGCGTCAGCATGATCAATTCAAT
GATGTTCAGATCAAACAAGAGTCGCAGGATCATGATGGAGTGGACTCGCAGTACACGAACGGTACGCAAAATGAC
GACAGCGAGAGATTTGAGGCAGCTGAATCCGATGTAAAGGTGGAACCCGGCCTCGGTATGGGCATCACCTCCTCT
CAGAGCGAAAAGGGTCAAGTTCTGCCTGACCAACCGGAGATTAAGTTCATTCGTCGTCAAATCAATGGTTATGTA
GGGTTTGCTAATTTGCCCAAGCAATGGCACAGAAGGTCCATAAAGAACGGATTCAGTTTCAATCTCCTATGTGTC
GGCCCTGATGGTATTGGTAAGACAACTTTAATGAAAACTTTATTTAATAATGATGATATTGAGGCCAATTTAGTG
AAAGATTACGAGGAGGAACTTGCAAATGATCAGGAAGAAGAGGAGGGTCAGGGCGAGGGTCACGAGAACCAATCA
CAAGAACAAAGGCACAAGGTCAAAATTAAGTCATATGAGTCGGTTATTGAAGAAAATGGTGTTAAACTGAATTTG
AACGTGATCGATACTGAAGGATTTGGTGATTTTTTAAATAATGATCAAAAATCATGGGACCCGATCATTAAGGAG
ATTGACTCTCGTTTTGATCAATACTTGGATGCGGAGAACAAAATCAACAGACATTCAATAAACGATAAAAGAATC
CATGCATGTCTTTATTTCATTGAACCTACAGGACATTACTTAAAGCCCCTGGACCTGAAGTTTATGCAATCTGTA
TATGAAAAATGTAACTTGATTCCTGTCATTGCTAAATCTGACATCTTAACTGATGAAGAGATCTTAAGTTTCAAG
AAGACCATTATGAACCAGTTAATCCAATCTAATATCGAGCTGTTCAAGCCTCCAATTTATTCCAACGATGATGCA
GAAAATAGCCATTTATCCGAAAGACTATTTAGTAGTTTACCTTATGCTGTTATTGGTTCCAACGATATTGTAGAG
AACTACAGTGGTAATCAAGTGAGAGGCCGTTCCTATCCATGGGGGGTGATCGAAGTGGACAATGACAACCACTCT
GATTTCAACCTTTTGAAAAATTTATTGATCAAGCAATTCATGGAGGAATTAAAGGAAAGGACAAGCAAGATACTA
TACGAAAACTACAGGTCTTCTAAATTGGCAAAACTTGGTATTAAGCAAGATAATTCAGTTTTCAAAGAGTTCGAT
CCAATATCTAAACAACTAGAAGAAAAAACTTTACATGAGGCAAAACTAGCCAAACTAGAAATTGAAATGAAAACA
GTTTTCCAACAAAAAGTTTCCGAAAAGGAGAAAAAACTACAAAAAATCAGAAACTGAATTGTTTGCTAGACATAAA
GAAATGAAGGAAAAATTAACTAAACAATTAAAAGCTTTGGAGGACAAGAAAAAACAGCTAGAACTTTCAATAAAT
AGTGCTTCTCCCAATGTTAACCACTCCCCCGTCCCTACAAAGAAGAAGGGATTTTTACGTTAG

>YMR262W 1.61 Hot
ATGAATAAACTTGTTGATGCCCACTGTCATGTTATAACCGACCCAGACAACACATTCTGCGGTGATGATGGAGGG
TCCCAGGGTACGTTGCGTTGCGTTATGTCATCAAACCCATATGACTGGAACAATTTGAAAAAATTGGCTGGCAGA
AGCACAAGTAAAAACGATATATGCGTGGGATTTGGTGTGCATCCTTGGTACAGCCACTTATTTTATGTGGGAAGT
CGGCGCGACAAGGTTTCTCACTATCAAGATGTACTGGAGTATAAGAATGAAGAACAGTTCGACAGTTTGGTTCAG

GTGCTTCCCGAACCTCTAGATCTTGAAGAGTATATAAAAAGAGAGTTTAACGACACTTTGGTTAGTGTGATTGGA
GAAATCGGTCTTGATAAGCTCTTCAGACTTCCTGCAAATGGTTTCTACATGCAGAACGAGAAGGCAAGGCTTACG
ACAGTGAAGGTCAAACTATCGCATCAAGAAACAGTATTCAGGCGATTTTGGCGACTGGCAAGGCACACAAGCAAG
CCCATCTCTATACACGATGTAAAGTGCCACGGGAAACTAAACGATATCTGCAATGAAGAGCTCTTGACGTATCAC
TCTGTCAAGATATGTTTACATTCGTACACAGGCTCCAAGGAAACGCTTCTGGGCCAATGGCTCAAGAAGTTTCCC
CCGGATCGTATTTTTGTGAGCCTATCCAAATGGATTAATTTAAGGACCCAGAAGAGGGAGATGCCCTGGTCAGG
AGCTTGCCCTCAACTTGCATACTTACTGAAACCGACTATCCTATTGACAATCCAGATCCGTCATACCAAAAGGCC
CTTACAGAGCAGCTGCAGTATTTGAATGCACAAATCGCACGAGCATGGGACGAGACACTGGATGCCTCACAAGCC
GCTTTGCGTGTATACGAAAACTTTCAGAAATTTATCAAGTGA

>YAL037W 1.72   Hot
ATGGATATGGAAATCGAAGATTCAAGCCCCATAGATGACCTGAAGTTACAAAAACTGGATACCAATGTTTATTTT
GGACCCTGTGAGATATTGACACAACCTATTCTTTTGCAATATGAAAATATTAAGTTCATCATTGGTGTCAATCTA
AGTACTGAAAAGATAGCGTCGTTTTATACCCAGTATTTCAGGAACTCTAATTCGGTAGTCGTGAATCTTTGCTCA
CCAACTACAGCAGCAGTAGCAACAAAGAAGGCCGCAATTGATTTGTATATACGAAACAATACAATACTACTACAG
AAATTCGTTGGACAGTACTTGCAGATGGGCAAAAAGATAAAAACATCTTTAACACAGGCACAAACCGATACAATC
CAATCACTGCCCCAGTTTTGTAATTCGAATGTCCTCAGTGGTGAGCCCTTGGTACAGTACCAGGCATTCAACGAT
CTGTTGGCACTCTTTAAGTCATTTAGTCATTTTGGAAATATCTTGGTTATATCATCACATTCCTATGATTGCGCA
CTTCTCAAATTTCTTATTTCCAGGGTGATGACCTACTATCCACTAGTGACCATCCAGGATTCTTTGCAATATATG
AAAGCAACCCTGAACATATCCATCAGTACATCCGATGAGTTCGATATTCTGAATGATAAAGAACTGTGGGAGTTT
GGCCAAACCCAGGAAATTCTAAAACGTAGGCAGACGAGCTCAGTCAAGAGGAGATGTGTCAATTTACCAGAAAAC
TCTACGATCGATAACAGAATGCTTATGGGTACCACAAAGCGAGGTCGCTTTTGA

>YDR275W 1.64   Hot
ATGTTTTTTTTCCCGAAGCTTAGAAAACTTATAGGTTCAACTGTGATCGATCACGATACCAAGAACTCATCAGGA
AAGGAAGAGATCATGTCAAATAGCCGATTAGCACTTGTTATTATCAACCACGCCTTTGATAAGGTACTATCTTTG
ACGTGGCACTGCGGGATATTATCCGAAATAAGATCAGGACTGATGTTAATGTTTGGCATTTTCCAGTTGATGTGC
TCTTTGGGTGTCATCGTATTGCTGTTGCCTATTATCATACTGGACGCGATCGATCTATTCCTCTACATGTGCAGG
TTGCTTGATTACGGTTGCAAGTTATTCCACTACAATAGATCATCATTACCGGTGGCAGATGGAAAAGAAAAGACC
AGTGGTCCCATAAGCGGAAAGGAGGAAATAGTTATTGATGAGGAGATAATTAATATGCTAAATGAATCCTCAGAA
TCATTGATCAATCATACTACAGCTGGTCTGGAATATGATATTAGTTCAGGTAGTGTTAACAAAAGTAGGCGTTTG
AATTCAACTAGTACAGTTACATTTGTGAAGCAGAATAAACTTGTTAATGAAAGGAGGGAAGACGCCTACTACGAG
GAAGAGGATGATGATTTCCTATCAAATCCGAATTATGATAAGATATCATTGATCGAAAAATCATTCACGAGTCGT
TTTGAAGTGGCTTGTGAGCAGAAAGCTGCTTAA

>YLR327C 1.79   Hot
ATGACCAGAACTAGCAAATGGACAGTCCACGAAGCAAAGTCTAACCCAAAGTATTTCACCCATAACGGCAACTTT
GGGGAGTCTCCCAACCACGTCAAGAGAGGAGGCTATGGGAAAGGCAATTGGGGCAAGCCTGGCGATGAGATTAAT
GACTTAATCGATTCTGGCGAAATTAAGACAGTCTTCAACAAGACCAGAAGGGGCTCTAACTCCCAAAACAATGAA
AGAAGGCTTTCTGATTTGCAACAATACCACATCTAA

>YJL122W 1.52   Hot
ATGCCTTCTAAAAACTCCATCAACAGACCTAAGCTGACTTCAAATTTGCACCACAAGGTGCACTCTTTAAATAAG
AAAAGAGCTCAAAGAGAAAGAGCTGGTCTCTTGAAGCCAGCTAGATCCAGCGTAAATTCGAAATCTGGGGAAATA
AAATCAGTAGCGTTGGACCTATACTTCCAAAACAAGAAGAACGAAAGTCAGAATTCCACTGCTGTGACATTACAA
AATGCTTCATCTTCTCCGGCCTCCATCACCACGAGGACACTTTCCAAAAAGAGAGCTAAGAAAATCGAGAGAAAC
TTAAAATATGCTACACAGAGAAAATTATTGGTTGACGCAAGCGCTAAGCTAGAAGACGAAATGGATATTGATCTA
GATGGCGGTAAGAAAGTGAAAGAGAATGAAAAGAAGAGTTCTTTGACTTTGGTTAAGGAAGCCTTATGGAGTGTC
ATTGACGATACTGCATCCCAGGGATTGATCATCGAAAACGGACAAGGCACCACCCTGGGCGGCCCATTTTTCCCA
TGA

>YIL065C 1.92   Hot
ATGACCAAAGTAGATTTTTGGCCAACTCTTAAGGACGCATACGAACCACTCTATCCGCAGCAGCTGGAGATTCTG
CGCCAGCAAGTCGTCTCCGAGGGGGGGCCCACCGCTACCATACAGTCAAGGTTTAACTACGCATGGGGGCTGATC
AAATCCACTGACGTGAATGACGAAAGGCTTGGTGTGAAAATCCTCACAGACATTTACAAAGAGGCCGAGTCCCGT
AGACGAGAATGCCTATATTATCTGACCATAGGTTGCTACAAACTCGGTGAATACTCTATGGCGAAGAGATATGTA
GACACTTTATTTGAGCATGAGCGTAATAACAAGCAGGTGGGCGCTTTGAAGAGTATGGTAGAGGATAAGATCCAG
AAGGAAACACTCAAGGGTGTTGTCGTCGCTGGAGGCGTACTAGCCGGCGCTGTGGCCGTGGCTAGTTTCTTCTTA
AGAAACAAGAGAAGGTAA

>YPR123C 1.61   Hot
ATGTCCATATCCATCCCTGAAGAGCTACTGTTATCAGACGATGACATACCTGACATGCCTGACATGCTAGAATCA
GAAGTTGTCTGTGCACTGGCGGAGCTTGTTGGTGTACTGCTCATCGACATAGACATACCTGACATTGAACTGCTT
CCCATCGACATACTTGCCATTGACGACATAGTCGACGCCAACGTTTTGCTCGCACTGGACATCGCTTCCATGCTC
ATCGATGACATGCTCGATAATATCGTTTTGCTGGCACTAGACATCGCATCCATGCTCATCGACGCCATACTCGAT
GCTACTGTCTTGGATGCACTAGACATGGCGTCCATATTCATGCTGCTACCCATATTCATACCTTCCATTTTGAAT
GTCAAATATAATACACTTTTTTTTATTTTCTATTCGATTCTTCCAACAAATGTAATTTGA

>YJR070C 1.69 Hot
ATGTCTACTAACTTTGAAAAACATTTCCAAGAAAACGTCGATGAATGCACTCTAGAGCAACTAAGGGACATCTTA
GTCAACAAGTCCGGCAAAACAGTTTTGGCCAACAGATTTAGAGCTCTGTTCAACTTAAAGACTGTTGCTGAAGAA
TTTGCCACTAAGCCAGAGGAAGCCAAAAAGGCCATCGAATACATTGCCGAATCCTTCGTCAATGACAAGTCTGAG
TTGTTGAAGCACGAAGTGGCCTACGTGTTGGGTCAAACCAAGAACTTGGACGCTGCTCCAACTTTAAGACACGTT
ATGTTAGATCAAAATCAAGAACCAATGGTGAGACACGAAGCCGCTGAGGCTTTGGGTGCCCTAGGTGACAAGGAT
TCGTTGGATGACCTAAATAAGGCTGCTAAGGAGGATCCACACGTTGCTGTGAGAGAAACCTGTGAACTGGCCATT
AACAGAATCAACTGGACCCATGGAGGTGCCAAGGATAAGGAAAACTTGCAACAATCCCTATACTCGAGTATTGAC
CCAGCCCCACCTCTACCATTAGAAAAGGATGCTACCATCCCAGAACTACAGGCCTTATTGAATGATCCTAAGCAA
CCTTTGTTCCAAAGATACAGAGCCATGTTCAGACTGAGAGATATCGGTACTGATGAAGCAATCCTGGCCTTGGCC
ACTGGTTTCAGTGCAGAATCCTCCCTTTTCAAGCATGAAATCGCCTACGTCTTCGGTCAAATAGGTAGTCCGGCT
GCTGTCCCAAGTTTGATTGAAGTTTTGGGCAGAAAGGAAGAAGCTCCAATGGTTAGGCATGAAGCTGCTGAAGCC
TTGGGTGCCATTGCTTCTCCAGAAGTTGTCGACGTCTTGAAATCTTACCTCAACGATGAAGTCGATGTCGTCAGA
GAATCTTGTATCGTTGCGCTAGATATGTATGATTACGAAAACAGCAACGAACTAGAATATGCTCCAACTGCTAAT
TAG

>YBR267W 1.85 Hot
ATGAGTAGCAGTGGTGTTTATACGTGTAATTCATGTGTCCTGACCTTTGATTCAAGCGACGAGCAGCGGGCCCAC
ATGAAGTCCGATTGGCATCGCTACAATTTGAAAAGACGTGTTGCACAATTGCCACCAATATCATTTGAGACATTT
GACTCCAAAGTGTCTGCAGCTGCTGCCAGTACTAGTAAGTCTGCTGAAAAGGAGAAACCTGTTACCAAAAAGGAG
TTGAAAAGAAGGGAAAAGCAAGCATTGCTCGAAAAGAAAAAGAAACTGTTGGAAATTGCCAGGGCTAATATGCTT
GAAAACATGCAAAAGAGCCAAGAGGGAAATACTCCCGACCTAAGCAAGCTCTCTTTGCAAGAAAATGAGGAGAAC
AAGGAAAAGGAAGAACCTAAGAAGGAGGAGCCTGAACAGTTGACCGAGGAAGAAATGGCGGAAAGAGTAATGCAA
GAAAACGTACGCAACAGAGTCGATATTCCACTGGAACAATGTCTATTTTGTGAGCACAATAAGCACTTCAAAGAT
GTTGAAGAAAACCTGGAACACATGTTTAGGACCCACGGGTTTTATATCCCAGAACAGAAATATCTAGTCGACAAG
ATCGGCTTGGTAAAATACATGTCGGAGAAGATTGGTCTTGGGAACATTTGTATTGTTTGTAATTACCAGGGGAGA
ACGTTGACCGCTGTAAGACAGCACATGTTGGCAAAGAGACACTGTAAAATTCCCTACGAAAGCGAGGATGAAAGG
TTGGAGATATCTGAATTCTACGATTTTACAAGCTCATACGCAAACTTTAATAGCAACACAACACCAGACAACGAA
GATGACTGGGAAGACGTGGGCAGCGACGAAGCCGGAAGCGACGACGAAGATCTGCCACAAGAGTACTTATATAAC
GATGGTATAGAGCTGCATCTACCGACAGGCATCAAAGTTGGCCACAGGTCCTTGCAAAGATACTACAAGCAAGAC
TTAAAGCCCGAGGTGATACTGACCGAAGGCCAAGGTACCCTGGTCGCTGCAGAGACGAGATCGTTCTTACCTGCC
TTCGACAAAAAGGGCGTGCAGACTCAACAGCGTGTTTGGCAAACTGAGAGGTTCGACAAGAAAAGGCTCGATAAG
AGAAGTGCCAAGTTCGTCAATAACCAACCACACTACAGAGACCAACTTCTGCAGTAA

>YGR068C 1.51 Hot
ATGTTTTCACTTAGTTCGCTATCTAGCAGCGGTGGGCATTCAGAGCAGAAGGAAAGAGAGAGAATAAGTTATTTT
GATATTAGAATAAACTCCCCGTACAAGGACATAATTTTGATCCAAGGGTCACCGTTGGAACTGTCGTCTATACCT
TTATCAGGAAACTTAGTGATTTCAGTGAAAAATGAGATCGTAGTGAAGAAAATTTCGCTGAGATTGGTAGGAAGA
TTCAAATTGGAGTTTTTGCAAGTTGGCCGATATAAGAAGAATAGCAGTAGTTTGGCAAGTCTAGTTAAGGAAAAG
CGTAAGATTTTCGAATGCTATTGGGACAACTTATTGGTTTCTTCAAAAGGGGATGTTTTAGTCGGTGGGGAAAAT
GCGGAAAATCAACATAACAGTAGTAGCGGTCGCAGTACAAGCAACCAAGATATGGACACCAGCGGCAACGCAATA
TTTCTAAGCAAGAGATCACTTTCGAGCCCTGTTTTCAACAAAATAATTCGAAGAAAGACCCATTCTTCCCACAGA
AAAATACTAGAGCTACCAGAAAATGGTGTTACAGGAACCCCTTTCGAGGGTTTAAGAGAAAACGCCCGTAGTAGA
AGCAGTAGTAGTAACACCCTCAATAACAATAGCCATAGTTATAGTAACAGAGACGGGTCTGGAAGTTCGTACTTA
TTCCTAATGAAAAGAGGCAACTACGAACTACCCTTCAATACAATGCTCCCCCCAGAAGTGTGCGAAACAATCGAG
GGACTACAAAGTGGTAGCATACTGTATTCCTTTGAAGCCATTATAGATGGACGTCAGTTATGGGATACTGATCTG
AGCGTTCATACTTCTCCTCACGGTCCAATCGGTAGCACAAGTACGAGCGGCAATGGTATGAGAACGAAGAATAAA
ATTATCATCAAAAAGTTCAAGTATCTCAGAATCCTTCGTACATTATCGATGGACAATCTTGCCATGCAAGAAGAA
ATTAGCGTGGGTAATACTTGGCGTGACAAGTTGCAATACGAAACGTCCATCCCGAGTAGGGCTGTTCCTATTGGT
AGCACAACCCCCGTGAAGATTAAGATTTTCCCCTTCGAAAAGAACATTCGTTTGGATAGGATAGAAATGGCGCTA
ATCCAATATTACGCAATGAAGGACAGCAGCGCACAAATTTATGATGATGAAATAGCCGTCATGAAAATTACTCAT
CTAGCGGACTTCGGCCCGCTGACGGACAAACTTGACGTCGATTGTCCCTTTACAATTCCAGATAACCTCAAACAA
ATAACTCAAGACTGCTGTCTGCAAGACAACCTAATCCGTGTCATGCACAAATTGCAGGTACGTATTCTTTTACAA
CGTCAAGTGGATGGCGAATACAAGAACCTGGAGATCAAGGCGCAATTACCTATGTTATTATTTATTTCGCCGCAT
CTGCCTATGAAAGGCCGTCTAGTGTTGTTTGATAAACACGATGGCAAGATCCACTTCCGCCCTGGCGAATTAGTA
CCCCTTTTTTTAACCACTTACCCCGCGCAGGGTCTGACTCCAGGCGTTGAACTGAACTCCACTACAACGGCGCAC
CTCGCGCTGCCACAACCGCCACCGAATTATCACGAGAGCACCAACGATCATCTCATGCCGGCGCTACAGCCGCTC
GGCGCCGATTCGGTGGTACTGACAGTGCCGTCATACGAGCAGGCGCAGGCGCAGGCATCGGCATCGTCTTACGTG
ACAGGGTCCGTACCCGCTTACTGCGACGATGACTGA

>YLR234W 1.5 Hot
ATGAAAGTGCTATGTGTCGCAGAGAAAAATTCTATAGCGAAGGCAGTTTCACAGATCCTAGGAGGAGGCAGATCA
ACTTCAAGGGATTCCGGCTACATGTATGTAAAGAACTATGATTTCATGTTTAGTGGGTTCCCGTTTGCCAGAAAT
GGGGCTAACTGCGAAGTTACCATGACTAGTGTTGCAGGGCACCTAACAGGCATTGATTTCAGCCATGATTCGCAT
GGGTGGGGAAAATGCGCCATCCAAGAGTTATTTGATGCGCCACTGAACGAGATTATGAATAACAACCAAAAAAAG
ATAGCAAGCAACATCAAGCGAGAAGCGAGGAATGCAGACTATCTGATGATATGGACAGATTGCGACCGGGAAGGA

GAGTACATCGGTTGGGAGATATGGCAGGAGGCCAAGAGAGGCAACAGGCTCATACAAAATGATCAAGTATACCGG
GCAGTCTTTTCGCATCTCGAAAGACAACACATATTAAATGCAGCACGAAACCCAAGTCGATTGGATATGAAGAGT
GTGCACGCTGTAGGCACGCGGATTGAAATCGATCTTCGAGCAGGTGTTACATTCACCAGACTCTTAACAGAAACG
CTACGAAATAAACTGAGAAACCAAGCCACCATGACCAAGGATGGTGCAAAACACCGCGGTGGTAACAAGAACGAC
TCACAAGTCGTATCGTATGGTACATGCCAGTTTCCAACGCTCGGCTTTGTAGTAGACAGGTTTGAAAGAATACGA
AATTTTGTTCCCGAAGAGTTCTGGTATATCCAATTGGTAGTCGAAAACAAAGACAACGGCGGAACAACAACGTTC
CAGTGGGACAGGGGCCACTTGTTCGACCGGCTGAGCGTGTTAACGTTTTACGAGACATGCATCGAAACCGCCGGC
AATGTTGCTCAAGTAGTAGACTTGAAATCAAAGCCAACAACGAAATACAGACCTTTACCTCTGACCACAGTGGAG
CTACAAAAAAACTGCGCCCGGTACCTGCGTCTGAACGCCAAACAATCACTAGACGCAGCAGAAAAGCTATACCAA
AAGGGGTTCATATCGTATCCAAGAACAGAGACTGATACTTTCCCACACGCAATGGACCTAAAATCCTTGGTCGAA
AAGCAAGCTCAATTGGACCAACTCGCTGCAGGCGGCAGAACCGCCTGGGCATCGTACGCGGCATCGCTGCTCCAA
CCCGAAAACACAAGTAACAATAACAAGTTCAAGTTTCCACGAAGCGGCTCCCATGACGACAAAGCGCATCCACCA
ATCCACCCCATCGTAAGTCTGGGGCCTGAAGCAAATGTTTCGCCAGTGGAAAGAAGAGTATACGAGTACGTGGCC
AGGCACTTTTTGGCATGCTGCTCAGAGGACGCCAAGGGCCAATCGATGACCCTTGTGTTGGACTGGGCCGTTGAA
CGTTTCTCAGCTTCAGGTCTCGTAGTCCTAGAGAGAAATTTCCTCGATGTTTACCCTTGGGCCCGATGGGAAACC
ACCAAGCAGTTACCGCGGCTTGAAATGAATGCCCTCGTAGACATCGCGAAGGCCGAAATGAAGGCGGGCACTACG
GCGCCGCCCAAGCCGATGACTGAGAGTGAACTCATTCTCCTCATGGATACAAACGGCATTGGCACAGACGCCACC
ATTGCGGAGCACATAGACAAGATCCAAGTACGTAATTACGTTAGGAGCGAGAAAGTAGGCAAGGAAACCTACTTA
CAACCCACGACCCTGGGTGTCTCACTAGTGCACGGCTTCGAGGCCATCGGCCTCGAAGACTCCTTTGCAAAGCCC
TTCCAGCGCAGAGAAATGGAGCAAGACCTCAAGAAAATCTGCGAAGGTCATGCCTCCAAGACTGATGTTGTAAAG
GACATAGTCGAGAAGTATAGGAAGTACTGGCACAAGACGAATGCCTGCAAGAATACTCTCTTGCAAGTTTATGAC
CGTGTCAAGGCATCCATGTAA

>YFR035C 2.09 Hot
ATGAGCGCGAGTGACAAAACAAAGCTCTGCAATAAGGGCATGAGCAGGACATCGCGAACCACAACTTTCGTAATC
ACACCCGCCTTCCGTGAGAGAGACGATGAGGGTGCCAATTCCCTGTGTAAAGCTTTCTTGAACACTTTCTCGAAC
CTCAAATCAGGTATGTTCAAATGTTTGTTGGGAGTTGGCGCCGTAGGTACGTTCATTTCTACGTTCCCACAGTTT
TTCTTGCTGCCTTGCCTTCTTTGCGTACGGTGTGTATGTGTATGTCTGTGTGCAAGTATTTCCTATGCTGCAAGT
GCGATTTTCTCGTTTTCTATTTTTTTTTTTTTTTGCCTCGCCTAA

>YBR219C 1.72 Hot
ATGACATTGTTAAATACCCTCAGCAACTTCGGTGGGACATGGCCGCGGTTAATCATTATGTCCATGATCAACTAC
TTCACCGTGTATCAGTGCACTATTCCTGGCACAAATAAAGTATACGTAACTCACGGCGGCAGCATGCAAGCGTGC
ACCGAGCTTTTGAATGGCACCGTGACCATCCTGCGTGACGGCTATTACATCACCAATCTCATATGTATTGTAGTC
GGACTTTTCCTATATTTTGGATATTTGAAAAGGAAAATCCTCCATTTACAAAGTCTGCCAATCAGTTCCTGGAGA
TTTTTTCATTTTTTTTTTACAATTTTGGCAGTTACGTCTCGAGCTATTTATTATAAGAGTCAGAATTGGCGCAGG
GAGTGTTAA

>YBR072W 1.75 Hot
ATGTCATTTAACAGTCCATTTTTTGATTTCTTTGACAACATCAACAACGAAGTTGATGCCTTTAACAGATTGCTG
GGTGAAGGCGGCTTAAGAGGCTACGCACCAAGACGTCAGTTAGCAAACACACCCGCAAAGGATTCTACTGGCAAG
GAAGTTGCTAGACCAAATAACTATGCTGGCGCTCTTTATGATCCCAGAGATGAAACCTTAGATGATTGGTTCGAC
AATGACTTGTCCCTGTTCCCATCTGGTTTCGGTTTCCCTAGAAGTGTCGCAGTTCCAGTTGATATTTTGGACCAT
GACAACAACTACGAGTTGAAAGTCGTGGTTCCTGGTGTCAAAAGCAAGAAGGACATTGATATTGAGTACCATCAA
AACAAGAACCAAATTTTGGTTTCTGGTGAAATTCCATCTACCTTGAATGAAGAGAGTAAAGACAAGGTCAAGGTC
AAGGAGAGCAGCTCTGGTAAGTTCAAGAGAGTCATCACTTTGCCAGACTACCCAGGTGTGGATGCAGACAACATT
AAAGCAGACTACGCAAATGGTGTTTTGACATTAACAGTTCCAAAATTGAAGCCTCAGAAGGATGGTAAGAACCAC
GTCAAGAAGATTGAGGTTTCTTCTCAAGAATCGTGGGGTAACTAA

>YLR056W 2.94 Hot
ATGGATTTGGTCTTAGAAGTCGCTGACCATTATGTCTTAGACGACTTGTACGCTAAAGTTCTGCCCGCTTCGTTG
GCAGCTAATATTCCTGTCAAGTGGCAGAAATTGCTAGGGTTGAACAGTGGGTTCAGCAATTCTACGATTTTGCAG
GAGACTTTGAACTCCAAGAATGCCGTCAAAGAATGTAGAAGGTTCTACGGGCAGGTGCCATTCCTGTTTGATATG
TCGACGACGTCTTTTGCATCGCTATTGCCTCGTTCCAGCATCTTGAGAGAATTCCTCTCACTATGGGTTATTGTT
ACGATCTTTGGTTTACTACTTTACTTATTCACGGCTAGTCTCAGCTACGTGTTTGTGTTTGACAAGTCGATTTTC
AACCATCCTCGTTACTTGAAAAACCAAATGGCAATGGAAATCAAGTTGGCAGTCAGTGCTATCCCATGGATGTCG
ATGTTGACCGTTCCATGGTTTGTTATGGAATTGAACGGCCATTCTAAACTATACATGAAGATTGATTATGAAAAC
CACGGTGTAAGGAAGCTCATTATCGAGTACTTCACTTTCATCTTTTTCACTGATTGCGGTGTGTATTTAGCGCAC
AGATGGTTGCATTGGCCAAGGGTCTACCGTGCTCTGCACAAGCCTCATCACAAGTGGCTGGTCTGCACACCTTTC
GCATCTCATTCTTTCCATCCTGTAGACGGGTTTTTGCAATCCATCTCGTACCACATCTACCCATTGATTCTGCCA
TTACACAAGGTTTCTTATTTGATTCTGTTCACTTTTGTTAACTTTTGGACTGTTATGATTCATGACGGTCAATAC
CTATCAAACAATCCTGCCGTCAACGGTACTGCCTGCCACACGGTTCACCATCTATATTTCAACTACAACTACGGT
CAATTCACCACTCTGTGGGACAGACTAGGGGGTTCTTACCGTAGACCAGATGACTCATTGTTTGATCCTAAGTTA
AGAGATGCTAAGGAGACCTGGGACGCTCAAGTTAAGGAAGTTGAACATTTCATCAAGGAGGTCGAAGGTGATGAT
AATGATAGAATCTATGAAAACGACCCAAATACCAAGAAGAACAACTGA

>YEL017C-A 1.54 Hot

ATGTTGATGAGCACGTTACCAGGTGGTGTTATCTTAGTTTTTATTCTAGTCGGTTTGGCTTGTATCGCCATCATT
TCTACCATTATCTACAGAAAATGGCAAGCTAGACAAAGAGGTTTACAAAGATTCTAA

>YJL053W 1.69 Hot
ATGAGTATTTTTTTCAAGCCGCCGATTGATATTGAGATCCTTTTCGATAATGAGGAATCTCGCAAACACGTAGAC
ATTGCAACAAGATCAAGTAATTCCAGTTACAAATCAATGAAGGAAAGCTTGCCCGTCTATGAAGATGGCGAATCC
CTTGGTGGAATTGTTACTTTGAGAGTTCGGGATAGTAAAAAGGTAGACCACTTGGGCATAAAAGTGTCCGTTATT
GGATCCATTGATATGTTAAAATCTCATGGGAGCGGTAATTCATCTTCTAAAAAAGTTACCTCTAGCACATCATCT
TCCTCTTCTAATGGTTCAGTAGATGTGCGGAAGAATTCAGTTGATCAATTTTTATGCCAAAGCTATGACCTCTGT
CCTGCTGGTGAATTACAACATTCTCAAAGTTTCCCATTCCTATTCAGAGATTTGAGTAAAAGATACGAATCTTAT
AAAGGCAAAAATGTGGATGTAGCTTACTATGTTAAGGTAACAGTTATGAGGAAGTCCACAGACATCTCGAAAATA
AAAAGATTTTGGGTCTATCTTTATAATAGTGTAACTACTGCGCCAAATACCCTTTCCGCAAATGAGACAAAGGCA
ACCACTAATGACATCGCTGGTGGTAACTATGCTGCAGATAATGCGAGTGACAACACGCAAACAAAGAGCACCCAG
GGTGAGGCGGCAGACGTTAATCAAGTTTTGCCGATATCACATTCAAATAATGAGCCCAAGCCTGTTAGATTGGAT
ATAGGTATAGAAAATTGCCTTCACATTGAGTTTGAATATGCCAAATCCCAGTACAGCTTAAAAGAAGTCATTGTG
GGGCGTATATATTTCCTTTTAACGAGACTAAGGATAAAACATATGGAATTAAGCTTGATCACAAGAGAATCTTCC
GGCCTACAAACTTCTAATGTAATGACAGATTCCACTGCTATCCGATACGAAATAATGGATGGGTCTTCAGTGAAA
GGTGAAACCATACCCATAAGATTATTCTTGAGCGGCTATGACCTGACGCCCAATATGAGCTGCAACTACTTTAAC
GTCAAGAATTATTTGAGCTTGGTTATTATCGATGAAGATGGCAGAAGATATTTTAAACAATCAGAAATAACATTG
TACAGGACCCGGTAG

>YOR331C 2.19 Hot
ATGTCGTCCTTCATCGACTCGATTAAATCGACGTCTCTTTCAAGAGCCTTGACAATCGCCTTAGGTTCCAACAAC
TTCAAAAGTGCCTCCACGATCAATGATTGCAAAATGGGCTTGTACTCGTCCCGGTTGTTGGCAATTCCTGACAAC
TTTTCCTTGGTTTCCTCGAATATCCCGTCTAGCGACTGTTCACGAGCAGAAAGAACCTTCAACCTCATTTTGTTT
GCTATCGTTGACTTAGTAATCTGTTGCGAAAGCATGGCCTTCTTCAACTTGCTCTTGAAGTTGCCGTCAATGTTG
TTGGTTTCATTTCTTACGATGTTGGTCTTCTCGATTTCGTACTCTTGGTCAGCCTTCAATTGGATTTCCTTCGCT
TTTTCTTCAGCTTCCTTTCTGATGAAAGCTTGCATCTTGTTCAATTCATCGTTCACTTGGTTTGGTGTCAAAGCA
GTAATAGCGGAGGACATGCTGTATCGGATGGTGCGTGGCCTGTTCTGTGCCTCTTTTGTAAAACAGTTACAAACA
ACTTTTCTTGCAACTGCTATTGTTCTTTGCTGA

>YPL222W 1.91 Hot
ATGGGGGAAAAAAGGACAATAATCAAAGCGCTGAAAAACTCTGCAGCGTCCCACTTCATCAAGAAGTTGACCGCA
GATACTTCCCTGTCGTCCATACAAGAGGCAATAAACGTCGTGCAGCAGTACAATGCAACGGATCCCGTGAGATTA
AAGCTTTTCCATACGCCTCGGATGGTTTCGCAGGGGGCTCATTTTGCATTTTGTTTGCCCACTAAGAAACCACAT
TACAAGCCATTACTGCTTTCGCAAAACGCACTTGATGAGTTTAATTTGGTGCAGGATCAGGACTTGGAGAAAATA
CTATCCGGTGAGAAAGTATATTATTCCGATAGTATCTTTCCCTATAGTACAGTGTACTCCGGATTTCAATTCGGC
TCATTTGCTGCACAGCTGGGAGACGGACGTGTGGTAAACTTGTTTGATCTTAAGGACAAGTGTAGCGGACAATGG
CAAACGTTTCAGTTGAAAGGTGCCGGTATGACGCCATTTTCTCGGTTTGCAGATGGGAAAGCTGTGTTAAGATCA
AGCATACGTGAATTCATAATGAGCGAGGCGTTGCACAGTATTGGAATTCCATCCACAAGAGCTATGCAATTAACT
CTGTTACCGGGGACGAAGGCGCAAAGGCGCAATCAAGAACCGTGCGCTGTTGTATGCCGTTTTGCGCCCAGCTGG
ATCCGCTTGGGTAACTTCAACCTATTCAGATGGCGGCACGACTTGAAAGGGTTGATCCAACTATCAGATTATTGC
ATTGAGGAATTGTTTGCTGGGGGGACCCAATTTGAGGGGAAACCCGACTTTAATATATTCAAAAGGGATTTTTTT
CCTGACACTGAGACGAAGATTGATGAGCAGGTGGAAAAGGATGAAACTGAGGTGAGTACCATGACCGGAGATAAC
ATTTCCACATTGAGCAAATATGATGAGTTTTTCAGACATGTAGTTAGCTTGAATGCAAACACAGTGGCCCATTGG
CAAGCGTATGGGTTTGCTAACGGTGTTTTGAATACGGATAACACCTCCATTATGGGCCTGACAATTGATTATGGC
CCCTTTGCCTTCCTGGACAAGTTTGAACCAAGCTTTACCCCGAACCATGATGACACAGCGAAGAGATATTCGTTT
GCCAACCAGCCAAGCATTATATGGTGGAACTTGCAACAGTTTGCCAAGGATCTCGCATGTCTGCTGGGTCCTGAG
GCCCGTGACCTTGAATTGTTACTTAAGGGCGAACTGAATTCTGTTGATGACGCCCTAGAGAAAACCATGATTGAA
AGGGTCCAGAAGTTGGTAGAGCTCAGTGCTAATGAGTACAAGTATGTGTTTACGACAAGGTACGCGCAGATAATG
TCTCAAAGACTCGGTGTAGATCTGGATCTGGAAAAGTGCATGTCTTCTACAAACCTCAAAGATATCGAACATGCA
GCCGAGAAGGCTAAAGAGTTCTGCGATGTTATCGTCGAACCATTGCTAGATATTCTGCAGGCCACAAAAGTCGAC
TATAACAATTTTTTTATCCATCTACAAAATTACAAGGGGCCCTTCTTCATCAAAGATAAAAGTGACACCGCAACA
CTTTTTGGAGCATTCGATGAAGAGTACTTGGGTATGTTCTTCAACTCCAAGCAACTGCAGCAAATGGCAGAAACT
GAAGAGGCCTTCGCGGCTGGTGAGAAGGTCTTTGACGCTAACGGCGAATTGCGTTTGCTCAACGAAAAAATTGCAA
GAAATACGCAATTGGACCCAAGACTATTTGACCTTGGTCCCACCCACGGAAACTGCTGCAAGAGCGTCGCTTGCC
AAGAAAGCCAACCCGTTGTTTGTCCCCAGAAGTTGGGTACTGGAGGAAGTAGTCGACGATTTAATGTACAGTCAA
AGGGATGGCCTACAAGATCCCAGTTCGGAGCTAGATACATCTGCGTTGAAGAAGCTATACCTAATGAGCGTAAAC
CCATACGACCGCACGAAATGGGATGTCACTCTGCGGCCAGAACTGGAGACAAAATGGGCTGACCTTTCCCACCAA
GATGACGCTAAGTTTATGATGCAAGCTTCCTGCAGTAGCTAA

>YOR344C 1.72 Hot
ATGAACTCTATTTTAGACAGAAATGTTAGATCTAGCGAAACTACTTTAATTAAACCTGAATCTGAATTTGATAAT
TGGTTGTCGGATGAAAATGACGGAGCTAGTCATATCAACGTCAACAAGGACTCCTCGTCAGTTCTTTCTGCATCT
TCTTCCACATGGTTCGAACCATTGGAAAACATTATCTCCTCTGCATCCAGCTCCTCGATAGGCTCTCCAATCGAA
GACCAGTTTATATCTTCCAACAACGAGGAATCTGCTCTTTTTCCAACAGATCAGTTTTTCAGTAATCCTTCCTCA
TACTCGCATTCTCCCGAGGTTAGCAGCTCGATAAAAAGAGAAGAGGATGACAATGCCCTTTCGTTGGCAGATTTT

```
GAACCGGCTTCTTTGCAATTAATGCCTAACATGATAAATACTGATAATAATGACGATAGTACCCCACTTAAGAAT
GAAATCGAGCTAAACGACTCGTTTATAAAAACAAATCTAGATGCTAAGGAAACGAAAAAGAGGGCTCCAAGAAAA
AGACTGACCCCCTTCCAAAAGCAAGCTCACAACAAGATTGAAAAACGCTACAGAATAAACATCAACACAAAGATT
GCAAGACTGCAGCAGATTATCCCATGGGTAGCAAGTGAACAAACAGCTTTCGAAGTAGGTGATTCTGTAAAAAAA
CAGGACGAAGACGGCGCAGAAACTGCCGCTACTACTCCTCTTCCATCTGCCGCTGCTACAAGCACGAAGCTAAAT
AAAAGCATGATCCTAGAAAAAGCTGTTGACTATATTCTATATCTACAAAATAACGAACGACTATACGAAATGGAA
GTTCAAAGGTTGAAAAGTGAAATCGACACTTTGAAACAAGACCAAAAATAA

>YLR211C  1.51   Hot
ATGAGTACTTTAGCGGAAGTTTATACTATTATTGAGGACGCCGAACAGGAATGTCGAAAAGGAGATTTTACAAAT
GCAAAAGCGAAGTATCAAGAAGCCATTGAAGTTTTAGGTCCGCAGAACGAGAATTTGTCTCAAAATAAATTAAGT
TCTGACGTTACGCAAGCCATTGACCTATTAAAACAGGACATTACAGCCAAAATCCAAGAATTGGAACTTTTGATT
GAAAAGCAGTCATCAGAAGAAAATAATATTGGAATGGTCAACAACAATATGCTTATTGGCTCTGTTATTTTAAAC
AACAAATCTCCGATAAATGGAATAAGCAATGCTAGGAATTGGGATAACCCAGCATATCAGGACACTTTAAGTCCC
ATAAACGATCCACTTCTTATGTCAATTTTAAACCGCTTACAATTTAATTTGAATAATGATATTCAATTGAAAACA
GAAGGAGGAAAAAATTCCAAAAACTCGGAAATGAAGATTAACTTAAGACTGGAACAATTCAAAAAGGAGTTGGTA
TTATATGAGCAGAAAAAATTTAAAGAATATGGTATGAAAATTGACGAAATTACGAAAGAAAATAAAAAATTGGCT
AATGAAATTGGGCGATTAAGAGAACGCTGGGACAGCTTAGTAGAAAGTGCGAAGCAAAGAAGGGATAAGCAAAAA
AACTAG

>YJR047C  1.52   Hot
ATGTCTGACGAAGAACACACCTTTGAAAATGCTGACGCTGGTGCCTCCGCCACCTACCCAATGCAATGTTCTGCC
TTGAGAAAGAATGGTTTCGTTGTCATCAAAGGTAGACCATGTAAGATTGTCGACATGTCCACTTCCAAGACCGGT
AAGCACGGTCACGCCAAGGTCCATTTGGTTACCCTTGATATCTTCACTGGTAAGAAGTTGGAAGATTTGTCTCCA
TCCACTCACAACTTGGAAGTTCCATTTGTCAAGAGAAGCGAATACCAATTGTTGGATATTGATGATGGCTACTTG
TCCTTGATGACCATGGACGGTGAAACCAAAGACGATGTCAAGGCACCAGAAGGTGAATTGGGTGACAGCATGCAA
GCTGCTTTCGATGAAGGTAAGGATTTGATGGTCACTATCATTTCTGCCATGGGTGAAGAAGCTGCCATCTCCTTC
AAGGAAGCTCCAAGATCTGATTAG

>YGR147C  2.12   Hot
ATGATGGTACCCAGGATTAGTGCTTCACCAGTTTTCAAGAGGATATTTCTTCGATGGGGATTTGTAACTTTACCC
ATACAGAAAACCGTATCCCATACACTGCGCAGGGACTTTAGTGCCCCTTGTAGGAGTATGGTAAAATGCCTTCTC
CTACGACCTGGGATAAGTGTGCACTCAGCACAAGACAGAAAATTTTACAGTACAGAGGAAAAAAGTAGTCAGTTT
GATGAAAATAAAAGCAAAAGTAACAATGGGAAGAAAAATGAGCCACACGGTATTAAAGGATTAATGGCTAAGTAT
GGTTATTCTGCACTGATCGTATATATCCTCTTAACTTGTGTTGATTTGCCATTATGTTTTCTGGGAGTCCACTCA
CTAGGTGAAGAGAAGATTAAGATCTATCTGAACAGAGGTAAGCAATTGATTGGGATGGGTGAGCCTGACGAAAGC
AAAGTCATTCAAGATGTTAGAAGGAAACAGGCTCATCGCGAAGCAGTGCAAGCAGAGAATGCTGACAAGGTGGAG
GACGCATCGAGGAAAACTTTCAATGAGAGATGGCAAGAGATGAAGGACAGCACGTTGTTGGCCGAATTGCTAATT
GCGTATGGCATACACAAGAGTTTGATCATCGTTAGAGTACCCCTGACGGCAGTGCTAACGCCATCCTTCGTCAAG
CTTCTGCAGAGGTTTGGCATCGACTTGATGAAGAAGCAGAAAAAGGTATTCCAAACGATGGCCTCTGGTGCCAAA
ATAAGATACAAAGGGAACAACCCTAGCGATTTCATCAAGAATGAGGGCACAGCGCTCGACATTACAAAGCGCAAA
CCAAGAACCAAAGGCCAGAAATGGTTTGATGGCCTAATGTGA

>YGL236C  1.68   Hot
ATGCTGCGTGTAACAACCTTGGCGTCTTCGTGTACTTCATTTCCCTTACAGGTCCTCAGGAGGCGGTTGACCATA
TCTTCTTTGACAAGCTTCCAACCCACAACCAAGACACAGGTCGTGGTCATCGGTGCTGGACATGCAGGCTGCGAA
GCTGCAGCTGCGTCATCTAGGACTGGTGCGCATACTACTCTCATTACACCATCGTTAACAGATATTGGTAAGTGT
TCGTGCAACCCCTCTATTGGTGGAGTGGGTAAGGGCATCCTTGTAAAGGAAATCGACGCTCTCGATGGGCTGATG
GGCAAAGTAACTGATCTCGCTGGAGTGCAATTCAAAATGCTGAACAGAAGCAAGGGTCCTGCTGTGTGGGGGCCC
AGAGCCCAGATAGACAGAGAGTTATATAAGAAATACATGCAAAGGGAACTTTCCGACAAGAAAGCACACCCCAAC
TTGTCTCTGCTGCAGAACAAAGTTGCTGACTTGATCTTGTATGACCCCGGATGTGGCCACAAGGTCATCAAAGGT
GTGGTTCTGGATGACGGTACCCAGGTTGGGGCAGATCAGGTCATAATCACTACAGGTACGTTTCTCAGTGCAGAA
ATTCACATCGGCGACAAGCGTATTGCAGCAGGAAGAATTGGCGAGCAGCCAACATATGGGATCAGCAATACTCTA
CAAAATGAGGTGGGCTTTCAGTTAGGGCGTTTGAAAACAGGCACTCCGGCTAGGCTGGCCAAGGAATCCATTGAT
TTCAGCGCTCTGGAGGTCCAGAAGGGAGATGCGTTGCCTGTCCCTATGAGTTTTCTGAATGAAACCGTGTCAGTC
GAACCCACGAAGCAACTGGATTGCTTTGGCACACATACCACACCTCAAATGCACGACTTTTTGCGCAATAACTTG
CATCAATCCATTCATATTCAGGACACGACCATCAAAGGCCCCCGCTATTGTCCCTCCATCGAAGCTAAGATTCTA
AGGTTTCCTGATAGATCTTCCCATAAGATATGGTTGGAACCAGAAGGTTTCAACTCCGACGTCATTTATCCGAAC
GGGATATCCAATTCTATGCCTGAGGATGTCCAGTTACAAATGATGAGGCTCATCCCGGGCATGGCAAACGTTGAG
ATCTTGCAGCCGGCATACGGCGTGGAATACGACTACGTAGACCCACGGCAATTAAAGCCTAGCTTGGAAACAAAA
TTGGTGGATGGACTATTCTTGGCCGGACAAATAAATGGTACTACAGGCTACGAGGAAGCCGCTGCACAGGGGATC
ATTGCAGGTATCAATGCAGGATTACTATCGCGCCAAGAACGGGAGCAATTGGTCTTGAAAAGGTCCGAAGCGTAC
ATTGGTGTGCTCATTGACGATCTAATCAATAATGGCGTCATAGAACCATATAGAATGTTTACTTCCAGATCAGAA
TTCAGAATCAGCGTAAGAGCCGATAACGCAGACTTCAGACTGACGCCCATTGGTGCTCAACTAGGGATTATATCT
CCTGTCCGTTTAAGTCAATATTCAAGAGACAAGCACCTATATGATGAAACAATACGGGCGCTTCAAAATTTCAAG
CTAAGCTCTCAAAAATGGTCTAGCTTACTACAGGCTAATATCGCGCCACAGGCTGAAAACAGATCTGCTTGGGAA
ATATTCAGATTCAAAGATATGGATCTACATAAACTCTACGAGTGCATTCCCGATTTACCCATTAATTTGCTGGAC
```

ATCCCAATGCATGTTGTCACCAAAATTAACATTCAAGGTAAATACGAACCTTACATTGTCAAACAGAACCAGTTT
GTGAAGGCATTTCAGGCAGACGAAAATATGCTGTTGCCGCAAGATTACGATTACCGTCAGTTACCCACGCTTTCC
ACTGAATGCAAACTACTCTTGAACCGCGTACAGCCATTGACCATAGGCCAAGCAAGACGGATTCAAGGTATCACT
GCGGCAGCCCTATTTGAACTCTACCGTGTGGCACGGAAGCCAAGCCAACCAGTCATGTAA

>YLR185W 1.9Hot
ATGGGTAAGGGTACTCCTTCATTCGGTAAACGTCACAACAAGTCCCACACTTTGTGTAACAGATGTGGTCGTCGT
TCTTTCCATGTTCAAAAGAAGACCTGTTCCTCCTGTGGTTATCCAGCTGCTAAGACCAGATCTTACAACTGGGGT
GCCAAGGCTAAGAGAAGACACACTACTGGTACTGGTAGAATGAGATACTTGAAACACGTTTCAAGAAGATTCAAG
AACGGTTTCCAAACCGGCTCTGCTTCTAAGGCTTCTGCTTAA

>YDL181W 1.5Hot
ATGTTACCACGTTCAGCATTAGCACGCTCATTGCAATTACAGCGCGGTGTGGCCGCAAGGTTCTACTCTGAAGGT
TCTACCGGCACCCCAAGAGGGTCAGGCTCAGAGGATTCGTTTGTTAAAAGGGAAAGGGCCACGGAAGACTTCTTC
GTTAGGCAGCGTGAGAAGGAGCAACTACGCCATTTGAAAGAACAACTGGAAAAACAACGAAAGAAGATTGATTCT
TTGGAAAATAAAATTGACTCGATGACCAAATAA

>YNL098C 1.71   Hot
ATGCCTTTGAACAAGTCGAACATAAGAGAGTACAAGCTAGTCGTCGTTGGTGGTGGTGGTGTTGGTAAATCTGCT
TTGACCATACAATTGACCCAATCGCACTTTGTAGATGAATACGATCCCACAATTGAGGATTCATACAGGAAGCAA
GTGGTGATTGATGATGAAGTGTCTATATTGGACATTTTGGATACTGCAGGGCAGGAAGAATACTCTGCTATGAGG
GAACAATACATGCGCAACGGCGAAGGATTCCTATTGGTTTACTCTATAACGTCCAAGTCGTCTCTTGATGAGCTT
ATGACTTACTATCAACAGATATTGAGAGTCAAAGATACCGACTATGTTCCAATTGTGGTTGTTGGTAACAAATCT
GATTTAGAAAACGAAAAACAGGTCTCTTACCAGGACGGGTTGAACATGGCAAAGCAAATGAACGCTCCTTTCTTG
GAGACATCTGCTAAGCAAGCAATCAACGTGGAAGAGGCGTTTTACACTCTAGCACGTTTAGTTAGAGACGAAGGC
GGCAAGTACAACAAGACTTTGACGGAAAATGACAACTCCAAGCAAACTTCTCAAGATACAAAAGGGAGCGGTGCC
AACTCTGTGCCTAGAAATAGCGGTGGCCACAGGAAGATGAGCAATGCTGCCAACGGTAAAAATGTGAACAGTAGC
ACAACTGTCGTGAATGCCAGGAATGCAAGCATAGAGAGTAAGACAGGGTTGGCAGGCAACCAGGCGACAAATGGT
AAGACACAAACTGATCGCACCAATATAGACAATTCCACGGGCCAAGCTGGTCAGGCCAACGCTCAAAGCGCTAAT
ACGGTTAATAATCGTGTAAATAATAATAGTAAGGCCGGTCAAGTTTCAAATGCTAAACAGGCTAGGAAGCAGCAA
GCTGCACCCGGCGGTAACACCAGTGAAGCCTCCAAGAGCGGATCGGGTGGCTGTTGTATTATAAGTTAA

>YLR315W 1.59   Hot
ATGAACTCTGAACAGCTGCTGCATAACTATGTTTCGGATTCGCTACTCACTACTCTGATAAGTTTTCAAGAGTTC
AAGCAGCAACTACAGTCGTACACGAGCGACGAGCAACAACTGCAACATTGGTACGAATTGCTTCAGGCCAGGGAT
GCGCGAGTGACCTCTGAGTTAGAAGCACGAATCAAGCAGTTTTTCATAACGTTGCGCTCTAGATTACTGCGATTT
CTGGAAAGCGAGCAACTATCACACTCCCTTAGCCTCGAGACACTGATCGACGCGCTGTACAAGATTAATGACCTG
TTGCAACAGCGTTTGCAGATTCTGGATGATGCTATTCAGGAAAAGACCTCGGAACTAGCAGAATTTGAAAATATG
GTTCGCTCACCGAGTGCAGGAGACAATGCCATTCCTGGCCTATTACAAATTATACAATCTTATATTAATCTATTA
GAGGAAAACTAG

>YBR025C 1.59   Hot
ATGCCTCCAAAGAAGCAAGTCGAAGAAAAAAAGGTCTTATTGGGTCGTCCAGGTAATAACTTGAAAGCCGGTATT
GTCGGTTTGGCCAATGTTGGTAAGTCTACCTTTTTCCAAGCCATCACTAGATGTCCATTGGGTAACCCAGCTAAC
TATCCATTCGCTACCATTGATCCAGAAGAAGCCCGTGTTATTGTCCCATCTCCAAGATTTGATAAGTTGTGTGAA
ATCTACAAGAAGACAGCTTCGGAAGTTCCAGCTCATTTGACCGTTTACGATATTGCTGGTTTGACTAAGGGTGCC
TCTGCTGGTGAAGGTTTGGGTAATGCTTTCTTGTCTCACATCAGATCAGTCGATTCTATCTACCAAGTCGTTCGT
TGTTTCGATGATGCTGAAATTATCCACGTTGAGGGTGACGTTGATCCAGTTCGTGATTTAGAAATTATTAACCAA
GAACTAAGATTGAAAGATATTGAATTCGCACAAAAGGCTTTGGAAGGTGCTGAAAAGATTGCCAAAAGAGGTGGT
CAATCTTTGGAAGTCAAACAAAAGAAGGAAGAAATGGATTTGATTACGAAAATCATTAAATTGCTAGAGAGTGGT
CAAAGAGTTGCTAATCACTCCTGGACTTCAAAAGAAGTTGAAATTATCAACTCCATGTTCTTGTTGACTGCTAAG
CCATGTATCTATTTGATTAATTTATCTGAAAGAGATTACATCAGAAAGAAAAACAAGCATCTGCTAAGAATCAAG
GAATGGGTAGACAAGTACTCTCCAGGTGACTTGATCATTCCATTCAGTGTTTCTCTAGAAGAAAGACTATCTCAT
ATGTCCCCAGAAGATGCTGAAGAAGAATTGAAGAAACTGCAGACAATATCTGCCTTGCCAAAGATTATCACTACC
ATGAGACAAAAGTTAGATTTGATTTCCTTTTTCACCTGCGGTCCAGATGAAGTTCGTGAATGGACCATCAGAAGA
GGTACTAAAGCTCCACAAGCTGCTGGTGTTATTCATAACGATTTAATGAATACCTTTATTTTGGCTCAAGTTATG
AAATGTGAAGATGTCTTCGAATATAAGGACGATTCTGCCATCAAGGCCGCTGGTAAGTTGATGCAAAAGGGTAAA
GACTATGTCGTTGAAGACGGTGATATCATTTACTTCAGAGCTGGTGCTGGTAAGAATTGA

>YOR374W 1.98   Hot
ATGTTCAGTAGATCTACGCTCTGCTTAAAGACGTCTGCATCCTCCATTGGGAGACTTCAATTGAGATATTTCTCA
CACCTTCCTATGACAGTGCCTATCAAGCTGCCCAATGGGTTGGAATATGAGCAACCAACGGGGTTGTTCATCAAC
AACAAGTTTGTTCCTTCTAAACAGAACAAGACCTTCGAAGTCATTAACCCTTCCACGGAAGAAGAAATATGTCAT
ATTTATGAAGGTAGAGAGGACGATGTGGAAGAGGCCGTGCAGGCCGCCGACCGTGCCTTCTCTAATGGGTCTTGG
AACGGTATCGACCCTATTGACAGGGGTAAGGCTTTGTACAGGTTAGCCGAATTAATTGAACAGGACAAGGATGTC
ATTGCTTCCATCGAGACTTTGGATAACGGTAAAGCTATCTCTTCCTCGAGAGGAGATGTTGATTTAGTCATCAAC
TATTTGAAATCTTCTGCTGGCTTTGCTGATAAAATTGATGGTAGAATGATTGATACTGGTAGAACCCATTTTTCT

TACACTAAGAGACAGCCTTTGGGTGTTTGTGGGCAGATTATTCCTTGGAATTTCCCACTGTTGATGTGGGCCTGG
AAGATTGCCCCTGCTTTGGTCACCGGTAACACCGTCGTGTTGAAGACTGCCGAATCCACCCCATTGTCCGCTTTG
TATGTGTCTAAATACATCCCACAGGCGGGTATTCCACCTGGTGTGATCAACATTGTATCCGGGTTTGGTAAGATT
GTGGGTGAGGCCATTACAAACCATCCAAAAATCAAAAAGGTTGCCTTCACAGGGTCCACGGCTACGGGTAGACAC
ATTTACCAGTCCGCAGCCGCAGGCTTGAAAAAAGTGACTTTGGAGCTGGGTGGTAAATCACCAAACATTGTCTTC
GCGGACGCCGAGTTGAAAAAAGCCGTGCAAAACATTATCCTTGGTATCTACTACAATTCTGGTGAGGTCTGTTGT
GCGGGTTCAAGGGTGTATGTTGAAGAATCTATTTACGACAAATTCATTGAAGAGTTCAAAGCCGCTTCTGAATCC
ATCAAGGTGGGCGACCCATTCGATGAATCTACTTTCCAAGGTGCACAAACCTCTCAAATGCAACTAAACAAAATC
TTGAAATACGTTGACATTGGTAAGAATGAAGGTGCTACTTTGATTACCGGTGGTGAAAGATTAGGTAGCAAGGGT
TACTTCATTAAGCCAACTGTCTTTGGTGACGTTAAGGAAGACATGAGAATTGTCAAAGAGGAAATCTTTGGCCCT
GTTGTCACTGTAACCAAATTCAAATCTGCCGACGAAGTCATTAACATGGCGAACGATTCTGAATACGGGTTGGCT
GCTGGTATTCACACCTCTAATATTAATACCGCCTTAAAAGTGGCTGATAGAGTTAATGCGGGTACGGTCTGGATA
AACACTTATAACGATTTCCACCACGCAGTTCCTTTCGGTGGGTTCAATGCATCTGGTTTGGGCAGGGAAATGTCT
GTTGATGCTTTACAAAACTACTTGCAAGTTAAAGCGGTCCGTGCCAAATTGGACGAGTAA

>YLR312C 1.67   Hot
ATGTCAGAAGAAGACGATCATTGGAATTTAGTTAGATTACGTAGATTACGTAAAGGGAGAGAAGGGGAAGAACAG
TCATCGAAGTCAGAAATATCTTTGGATAGTTTGCATGAAAGCTCCTTTGCAGGAGAGGACGACGAGGACTTCGAT
GCAGATGTCCTATCGAACACTAGCAGTGAAGAGTCTGCACAGATGAATCGTATTTACGATTTTAGAACATCTAAT
GAATTTAGTAATGCTGGAGTTAATATTGATCAAACTGGAGTTCCCACTATTTCAGAGTCATTTGATACTTTGTCC
GGCTCAAATGTTGGCGGAACGGTATTGCCAAGTATGGAGGGGTCGAAACTGAAGGATAGTACGATAAGGAATTCT
AGCACACTATCGGATCATATCATAGATAAAAGTGAGGGTAAATCTGCTAAATTGAAGATGTGGCATGTTATCATG
CTATCTTCATTGCTTTCCATGACCTTTTCATACCTCGCCCTCGAATATTCCCTGACTGGTGATGTGTTGGCAGGT
TTTAAATCACAACAGTCATTACGTAATAATGAAAGGAAGCTGTTGTACGGCAATATCGATTTTGTTGATAAAAAA
TCTTACGATTCATCAAGTGACTCTTTAAGTCAGTGGGCTCCTTCAGGAAAATACTACGTCGACTTCGACAATCAT
ATTGCATACCCATTAAAGGATGATGACCTAATGGGCTGGAGACGATACAAAACAGACTTAGTTATTTTATGGTAT
ACAACAAAAGCTCGAATGAAAGACGGTTGGCACAAGAGAATTAACAAAATAAACGGAGGAAGAATAAAGTTACAC
CTATTTCTCAAGAATTCTTTTAAATCCGCTCAAGAAAGTTTAAGGGTATTGCATAAAGAACAGAAACGCCGCTGG
AAAAGGCTCTTTGTGCTACTTCATAATAAATACAGGCAATTTTCTCCACATATTAAAAGGTATTTCGATCATTCT
TGCCAAAAAGCAAAACAATGTTGGTCGGGATCCAGATTGCAGTTGCGCAAGCTTCGTTTCAAGTCAATGAAACCA
TTCCGAGTTTTTCAGTTTAAGGTTCGCAAAGATACCAACTGGTTTGTAAAGCAGCTGAAACGGTTCGGATTGAAA
TTACAGCATTCGAGGATGTATAAAGCGATGTCAGAATGCAGGAAAAAAAATTATTTTAAGTGCAAACACTAG

>YBR266C 1.57   Hot
ATGTGTTCCAGGTTTTCTTCAACATCTTTGAAGTGCTTATTGTGCTCACAAAATAGACATTGTTCCAGTGGAATA
TCGACTCTGTTGCGTACGTTTTCTTGCATTACTCTTTCCGCCATTTCTTCCTCGGTCAACTGTTCAGGCTCCTCC
TTCTTAGGTTCTTCCTTTTCCTTGTTCTCCTCATTTTCTTGCAAAGAGAGCTTGCTTAGGTCGGGAGTATTTCCC
TCTTGGCTCTTTTGCATGTTTTCAAGCATATTAGCCCTGGCAATTTCCAACAGTTTCTTTTTCTTTTCGAGCAAT
GCTTGCTTTTCCCTTCTTTTCAACTCCTTTTTGGTAACAGGTTTCTCCTTTTCAGCAGACTTACTAGTACTGGCA
GCAGCTGCAGACACTTTGGAGTCAAATGTCTCAAATGATATTGGTGGCAATTGTGCAACACGTCTTTTCAAATTG
TAG

>YFL021W 2.13   Hot
ATGCACGTTTTCTTTCCTTTGCTTTTCCGCCCTTCCCCTGTTCTGTTCATCGCATGTGCATATATATATATAGAT
ATATATATACATTGTACACGGTGCACGGTAGTGAACATAACTATGAGCACGAACAGAGTCCCGAACCTCGACCCG
GACTTGAATTTAAACAAAGAAATCTGGGACCTGTACTCGAGCGCCCAGAAAATATTGCCCGATTCTAACCGTATT
TTGAACCTTTCTTGGCGTTTGCATAACCGCACGTCTTTCCATCGAATTAACCGCATAATGCAACATTCTAACTCT
ATTATGGACTTCTCCGCCTCGCCCTTTGCCAGCGGCGTGAACGCCGCTGGCCCAGGCAACAACGACCTCGATGAC
ACCGATACTGATAACCAGCAATTCTTCCTTTCAGACATGAACCTCAACGGATCTTCTGTTTTTGAAAATGTGTTT
GACGACGATGACGATGATGATGACGTGGAGACGCACTCCATTGTGCACTCAGACCTGCTCAACGACATGGACAGC
GCTTCCCAGCGTGCTTCACATAATGCTTCTGGTTTCCCTAATTTTCTGGACACTTCCTGCTCGTCCTCCTTCGAT
GACCACTTTATTTTCACCAATAACTTACCATTTTTAAATAATAATAGCATTAATAATAATCATAGTCATAATAGT
AGTCATAATAATAACAGTCCCAGCATCGCCAATAATACAAACGCAAACACAAACACAAACACAAGTGCAAGTACA
AACACCAATAGTCCTTTACTGAGAAGAAACCCCTCCCCATCTATAGTGAAGCCTGGCTCGCGAAGAAATTCCTCC
GTGAGGAAGAAGAAACCTGCTTTGAAGAAGATCAAGTCTTCCACTTCTGTGCAATCTTCGGCTACTCCGCCTTCG
AACACCTCATCCAATCCGGATATAAAATGCTCCAACTGCACAACCTCCACCACTCCGCTGTGGAGGAAGGACCCC
AAGGGTCTTCCCCTGTGCAATGCTTGCGGCCTCTTCCTCAAGCTCCACGGCGTCACAAGGCCTCTGTCGTTGAAG
ACTGACATCATTAAGAAGAGACAGAGGTCGTCTACCAAGATAAACAACAATATAACGCCCCCTCCATCGTCGTCT
CTCAATCCGGGAGCAGCAGGGAAAAAGAAAAACTATACAGCAAGTGTGGCAGCGTCCAAGAGGAAGAACTCACTG
AACATTGTCGCACCTTTGAAGTCTCAGGACATACCCATTCCGAAGATTGCCTCACCTTCCATCCCACAATACCTC
CGCTCTAACACTCGCCACCACCTTTCGAGTTCCGTACCCATCGAGGCGGAAACGTTCTCCAGCTTTCGGCCTGAT
ATGAATATGACTATGAACATGAACCTTCACAACGCCTCAACCTCCTCCTTCAACAATGAAGCCTTCTGGAAGCCT
TTGGACTCCGCAATAGATCATCATTCTGGAGACACAAATCCAAACTCAAACATGAACACCACTCCAAATGGCAAT
CTGAGCCTGGATTGGTTGAATCTGAATTTATAG

>YJL173C 1.64   Hot

ATGGCCAGCGAAACACCAAGAGTTGACCCCACAGAAATCTCCAACGTCAATGCTCCTGTGTTTAGGATAATAGCG
CAAATCAAATCACAACCCACTGAATCTCAGTTGATTTTACAATCGCCAACCATATCATCGAAAAACGGCAGCGAG
GTTGAAATGATTACGTTGAACAATATTCGTGTATCTATGAATAAAACATTTGAGATCGACTCGTGGTATGAGTTT
GTTTGCAGGAACAATGATGACGGCGAGCTAGGATTTTTGATTCTAGACGCTGTACTATGCAAATTCAAGGAGAAC
GAAGATTTAAGCTTAAACGGTGTGGTTGCTTTACAGAGACTATGTAAGAAATACCCAGAAATATACTAG

>YJR025C 2.68   Hot
ATGTTTAATACTACACCAATTAATATCGACAAATGGTTGAAGGAGAACGAAGGCCTTTTGAAACCACCGGTGAAT
AATTATTGCTTACATAAAGGGGGATTCACTGTGATGATTGTCGGTGGGCCTAATGAAAGAACCGGTTATCACATC
AATCCAACTCCCGAATGGTTCTATCAAAAAAAAGGATCTATGCTTTTAAAGGTTGTGGATGAGACAGACGCTGAA
CCAAAGTTCATTGATATCATCATCAATGAAGGCGATTCATATTTATTGCCAGGAAATGTTCCTCACAGTCCTGTT
CGGTTTGCTGATACTGTGGGTATTGTTGTGGAACAAGATAGGCCTGGGGGAGAAAACGATAAGATAAGGTGGTAC
TGTTCTCATTGTCGCCAAGTGGTCCACGAGAGTGAACTGCAAATGTTAGACTTAGGTACCCAAGTGAAAGAAGCC
ATTTTAGATTTTGAAAATGATGTCGAAAAGAGGACATGTTTCCATTGCAAGACGTTAAACTACGCACGCCCTCAA
TCTAATTAA

>YNR018W 1.75   Hot
ATGAAGATTTTAACCCAAGACGAAATTGAAGCTCATCGCTCACATACGCTAAAAGGTGGTATTGAAGGTGCCCTT
GCCGGATTTGCAATCTCTGCTATAATTTTCAAAGTCCTACCAAGAAGGTACCCAAAATTCAAGCCTTCGACTCTA
ACATGGTCCATAAAAACCGCCCTTTGGATCACCCCTCCCACGGTCTTGACTGCTATATGTGCGGAGGAGGCCTCG
AACAATTTCGACGCTACAATGTACGGATCCGGTTCCTCCTCGGAAGACGCACTAGATGAGCACAGAAGATGGAAG
AGTTTGAGTACAAAGGACAAGTTTGTCGAAGGTCTATCTAATAACAAGTATAAGATCATCACCGGTGCCTGGGCC
GCTTCGCTGTATGGGTCGTGGGTAATCGTGAACAAAGACCCCATCATGACCAAAGCTCAGAAGATCGTGCAGGCA
AGAATGTACGCTCAATTCATTACCGTCGGGCTGCTGCTGGCCTCCGTTGGTTTGAGCATGTACGAGAATAAGTTA
CATCCTAACAAACAAAAGGTCAACGAAATGCGCCGCTGGGAAAACGCACTGAGGGTCGCCGAAGAGGAAGAGAGA
CTCGAGAAAGAGGGAAGAAGGACCGGCTACGTTTCTAACGAAGAAAGAATAAACTCCAAGATCTTCAAGTCGTAA

>YIL047C 1.59   Hot
ATGAAGTTTGCTGACCATCTAACCGAGTCTGCCATCCCGGAATGGAGGGACAAATATATTGATTATAAGGTCGGC
AAGAAGAAGCTTCGCCGCTACAAGGAGAAGCTGGATGCCGAAGAAGAGCAATCCAGCTCCTACCGGAGCTGGATG
CCCTCTGTGTCGGTATACCAGACTGCATTTCAGCAGAGAGAGCCCGGCAAAAGTCGCAGCGACGGAGACTATCGC
TCCGGACCTGCGTTCAAGAAGGACTATTCTGCTTTGCAGAGGGAGTTCGTTGCTGACTTCATTGAAGACTGGTTG
ATCTCGTTCCAGTTGAGTAAGTGTAACGAGTTTTACCTTTGGTTGCTAAAGGAGTGCGACAAGAAGTTTGAGGTT
TTACAGTCGCAACTGCACTATTACTCGCTGCAGAAGAATTATGAGCGCGACAACTTGAACCGGTCTAGTTCGAAT
GTTGACATGTCCACGAGTCTATATGCGGCAGGGCTTGCTGGTCGTTCTGACTCCAGGGTCAATAGCATCGACAGC
GATAGCCGGTCTGTTATGTATGGGTCAATGCCCTGCACCAAAGAGGCCAAGAAGCCGCGTTTGTCGCTGCTAGCG
TACTGTCAGAAAGTTCTTAAGGACAATAGGCTGCTGCCATCGTGGCCCAAACGTGGATTTTCTCTGCTACAGGAC
TTGAGACAGGATGCGTCCTCAAGAGGCCGCGAGACGTTTGCTTTTGGCGCTTCTTTTCTGGAGACTATGACGACA
ACCCAGGCTAGAAACTTGTTAAGCAACGCTATAATCGAGTACTATCTGTACTTGCAATTGGTCAAATCGTTCCGG
GATATCAACGTCACCGGGTTCCGTAAAATGGTCAAGAAATTCGACAAAACGTGTCACACAAGAGAGCTGACCACG
TTCATGTCGTACGCCAGGACCCACTACACCCTTTTCAAGCACGCGGATGCCAACGTGCAGTTGGTGGCACAGAAG
ATGCAACAAATAACCAGTTCGCAACCAACACCCACTTCGGAGCTCTCTTCCGCCCAAAGAGACAAAGAGCCCATC
ACTTGGTTGGAAACGCAGATCACCGAGTGGTTCACCACTGCGTTGACCAATTCGCCTAAGGACAGGAAGCACAAT
ACCCATAAGTTGAAGAAATTGACCATTCAGTACTCTATCTCCGAACAAATGGTTCATAGAAACAACAGATCGATT
GTGCAGATGCTGGTTGTCGGGCTGGGCATAGGTGTGTCAATGACTTTGATCACTTACACTTTGTATTTGGGCATT
AGCTCTGAAGAAACGTCATTTACGCACAAGATTCTGTTCCCACTGTGGGGTGGTTGGTACATGGTTTTGTTGATC
GCGTTTCTCTTTCTTGTTAATTGTTTTATCTGGCATAGAACCGGGATTAATTATAGATTTATTATGCTGGGTGAG
ATTCAATCCAAGAATGGTACGCAATTTTTCAACAATGATTTTGCCACGAGTAAAATTCCGCTCAAGTTGTATTTC
TTGACATTCTTCATAGTACCTTGTGCGGTCTGTTCCATGTTAAGTTTCGCTTTGGAGAAGCTCACACCCTTAGGC
TTTTTATATATTGGTATTGTGTCTTTCTTATTTTTATGTCCCTCTGGTCTAATTCCATATTGGGACAAAGTTGTA
CACACTAGAAAATGGCTGGTGGTTACTCTTATTAGACTGATGATGTCTGGTTTTTTCCCTGTTGAGTTTGGTGAT
TTCTTTTTGGGAGATATTATTTGTTCGTTGACGTATTCTATTGCCGATATTGCCATGTTCTTCTGTGTTTACTCT
CATACGCCCAACAATCTATGTGGCTCTTCACATTCCAGAGCAATGGGTGTTTTATCATGTTTACCAAGTTATTGG
AGATTCATGCAATGTTTACGAAGATTTGCTGATTCCGGTGATTGGTTCCCTCATCTTTTGAACGCGGCGAAATAC
ACTTTGGGTATAGCGTATAATGCCACACTTTGTGCTTACAGATTGTCAGACCGTTCTGAACAAAGGAGAACCCCT
TTTATCGTGTGCGCTACGTTGAATTCGATCCTTACTTCCGCCTGGGATTTAGTTATGGATTGGTCCTTTGCTCAC
AATACTACTTCTTACAATTGGTTGCTAAGAGACGACTTGTATTTAGCTGGTAAAAAAAATTGGGAAAACGGCAGT
TATTCATTCAGTAGGAAGTTAGTTTATTACTTTGCTATGATTTGGGATATCCTCATTAGATTTGAGTGGATTGTA
TACGCAATTGCTCCTCAAACAATTCAGCAGAGTGCTGTGACGTCGTTTATATTGGCCCTTTTGGAGGTTTTAAGA
AGATTCGTTTGGATCATCTTCAGAGTCGAAAATGAACATGTTGCCAATGTTCACCTATTCAGGGTTACCGGTGAC
GCTCCTCTGCCTTATCCAATTGCGCAGGTCGGTGATGATTCTATGGATAGTAGTGATTTGGGTTCAAAAGCCTTT
TCATCACTTAACGACATACCTATCACGCCTTCACATGACAATAATCCTCACAGTTTTGCCGAACCTATGCCCGCT
TACCGTGGGACTTTCAGAAGAAGGTCCTCCGTGTTTGAGAACATTTCGAGATCCATTCCTTGGGCACATGCAACT
GATTTCCAAAGACCAACTGTCAATACCGTGGATGATAGGTCTCCCGAAACAGATAGTGAAAGTGAAGTGGAAAGT
ATTATGTGA

>YDR388W 1.64   Hot

ATGAGTTTTAAAGGGTTTACCAAGGCTGTTAGTAGGGCTCCTCAGTCCTTCCGTCAAAAGTTCAAGATGGGTGAG
CAGACAGAGGATCCTGTTTACGAGGATGCAGAACGTCGTTTTCAAGAATTGGAGCAGGAGACAAAAAAAATTAAGT
GAGGAATCGAAGAGATACTCGACTGCGGTGAATGGAATGCTGACACACCAAATTGGATTTGCCAAATCCATGGAG
GAAATTTTCAAACCTATCAGTGGTAAAATGAGTGACCCTAACGCTACCATACCAGAGGACAACCCACAAGGTATT
GAAGCAAGTGAACAGTATAGGGCAATTGTCGCAGAATTACAGGAAACATTGAAACCGGATCTGGCTTTGGTGGAA
GAAAAAATTGTTACGCCATGCCAGGAACTGTTGAAGATTATTACTTATATTCGTAAAATGGCCACCAAGAGAAAT
CACAAAAAATTAGATTTAGATAGACATCTAAACACTTATAACAAGCATGAAAAGAAGAAGGAACCAACCGCCAAA
GACGAAGAAAGATTGTATAAAGCACAAGCTCAAGTGGAAGTGGCTCAACAAGAATATGATTACTATAACGACTTG
CTAAAGACTCAATTACCAATACTGTTCAGTTTGGAAGCTGAATTTGTGAAACCATTATTTGTCTCATTTTATTTC
ATGCAATTGAACATTTTTTACACTTTGTATAATAGACTTCAAGACATGAAGATTCCATATTTTGATTTGAACAGT
GACATTGTAGAGTCGTACATTGCCAAGAAAGGCAATGTGGAAGAACAAACGGACGCCTTGACTATTACACACTTT
AAACTCGGTTACTCTAAGGCAAAATTGGAGATGACCAGAAGGAAGTATGGTGTAGCAACCGCCGAAGGCTCACCC
GTCAGTGGCGCATCAAGCGGTGTCGGATATGGTGCTGGGTATGATCCAGCAACTGCCACTTCTCCAACCCCTACA
GGATACGGTTATGGTGCCGCAGCACCAAGTTACGCCGCTCAACCTGCTGCCCAGTATGGTACTGCTGCCGCCGTT
GGTACTGCTGCTGCTGTTGGTACTGCTGCTGGTGCTGCTGCTGGCGCTGTTCCGGGTACTTATCCACAGTATGCT
GCTGCGCAATCTCCACCGCTCACTGGGCTAGGCTTCCAACAATCACCACAGCAGCAACAGGGACCACCACCGGCC
TACTCTAATCCTCTAACATCACCTGTTGCGGGCACACCAGCCGCGGCCGTAGCGGCAGCTCCTGGCGTTGAAACT
GTTACCGCATTGTATGACTACCAAGCTCAAGCTGCTGGTGATTTGTCTTTCCCTGCCGGCGCAGTTATAGAAATT
GTCCAGCGTACTCCGGACGTGAACGAATGGTGGACAGGAAGATACAATGGCCAGCAAGGTGTGTTTCCTGGGAAC
TACGTGCAACTCAACAAGAACTAG

>YFL031W 1.84 Hot
ATGGAAATGACTGATTTTGAACTAACTAGTAATTCGCAATCGAACTTGGCTATCCCTACCAACTTCAAGTCGACT
CTGCCTCCAAGGAAAAGAGCCAAGACAAAAGAGGAAAAGGAACAGCGAAGGATCGAGCGTATTTTGAGAAACAGA
AGAGCTGCTCACCAGAGCAGAGAGAAAAAAAGACTACATCTGCAGTATCTCGAGAGAAAATGTTCTCTTTTGGAA
AATTTACTGAACAGCGTCAACCTTGAAAAACTGGCTGACCACGAAGACGCGTTGACTTGCAGCCACGACGCTTTT
GTTGCTTCTCTTGACGAGTACAGGGATTTCCAGAGCACGAGGGGCGCTTCACTGGACACCAGGGCCAGTTCGCAC
TCGTCGTCTGATACGTTCACACCTTCACCTCTGAACTGTACAATGGAGCCTGCGACTTTGTCGCCCAAGAGTATG
CGCGATTCCGCGTCGGACCAAGAGACTTCATGGGAGCTGCAGATGTTTAAGACGGAAAATGTACCAGAGTCGACG
ACGCTACCTGCCGTAGACAACAACAATTTGTTTGATGCGGTGGCCTCGCCGTTGGCAGACCCACTCTGCGACGAT
ATAGCGGGAAACAGTCTACCCTTTGACAATTCAATTGATCTTGACAATTGGCGTAATCCAGAAGCGCAGTCAGGT
TTGAATTCATTTGAATTGAATGATTTCTTCATCACTTCATGA

>YDR411C 1.56 Hot
ATGGCAGGCCCAAGGAATGTGCGTACATTACATGGAAATGGAGGCCGTAATAATGATGTGATGGGCCCAAAAGAA
TTCTGGTTAAATATCCCCCCAATAACAAGAACTTTATTTACTCTAGCGATCGTTATGACGATCGTCGGCCGGTTG
AATCTAATCAATCCGTGGTACTTTATTTATGTTTGGAATTTGACGTTCAAGAAGGTTCAGATATGGAGACTTCTT
ACTTCTTGTGTAATGCTTTCGTCTCGTGCCATGCCTGCGCTAATGGAACTATATAGTATTTATGACAGGTCCTCA
CAGTTGGAGCGAGGGCATTTTGGTCCCGGTTTGTCCAATAGACGAGGACCGATGGTAACAGTAGACTATGCCTAC
TACCTGTGCTTTTGTATACTAGCCATCACTACGGCCACTACAATCATTTATGGGTCCTACTACCCTGTTGTGCTC
ACCTCTGGGTTCATATCATGCATTACATATACTTGGTCGATCGACAATGCCAACGTGCAGATCATGTTTTATGGT
TTGATTCCTGTTTGGGGGAAGTACTTCCCCTTAATTCAATTGTTTATCTCTTTCGTTTTTAATGAGGGTGATTTC
GTAATCTCATTGATCGGTTTTACTACAGGGTACCTTTATACATGCTTAGATACCCATACACTGGGGCCAATATGG
GGGATGATATCCAGAAAAGCTGATCCAACCTACGGAATCTCGCCCAATGGGAAATTCTCAACGCCATGGTGGTTT
ACTAGTCTATATGCTCGCATAACGGGTGCCCACAATGAAACTGCAACATTCAACAACAACTTTGCCAATGTGCCA
TCTTCGCAAAGAGAAACGAGAACTTTTAGTGGAAGAGGTCAACGATTGGGTACGGCCCCTGCAACATTGTCTCAA
ACCAGTGGCACAGATTCAGGCAGAGCTTCTGGAAGTCAATTAAGAAGTGGCCCATCGAATTTGAACCAATTTCAA
GGCCGCGGCCAACGAGTAGGACAAACAAACAGCCCCTCCGACTCACAATAA

>YLL065W 1.5 Hot
ATGCTACAGTATATACCACTCTCAAACTTACCCTACTCTCACATTCCACTCCATCACCCATCTCTCACCATCAGT
ACCAAATGCACTCGCATCATTATGCACGGCACTTGCCTCAGCGGTTTATACCCTGTGCCATTTACCCATAAAGTC
CATCATTATCCACATTTTAATATCTATATCTCATTCGGCGACCCCAAATATTGTATAACTGCCCTTAATACATAC
GTTATACCACTTTTGCACCATATACTAACCACTCAATTTATACACACTTATTTCAATATACCCACAAAATCACCA
CCAAAATCACCTAAACATAAAAATTATTTATCTTTCAACTTTACGAAATAA

>YER046W 1.83 Hot
ATGGGCAAAAACCATTTCTTGAAGGATTTCTCTGCTTTACCGGAGGACGTACTCATAGAAAACGAGAGAGGCATA
ACTTTACTTGGTTACCCACTATTTTCTCCCAAGATTTTACTGCCTCACGTTGACCCACCACAATTTCAAAGACTA
AACACTGAAAATGGCTCGTTAATTGCCCTGTCGAAAAATACTATATCGAACTTCATAGAACTGTATCCTATCGAT
CTGAGTACTGAACGCACTGCAGGCAGTAGCAGTTCACAGATGACAAAGTGGTTCGTACTGATGGATTACAAGGAA
AAGTACGATATTGACGATCAAGGATGGTGTTATAGCTGGAATTTCAATAACTCTAGGTGGAAATCGAAAAATGGC
TTGGTGAGAAGAAGGGTCTGGGTAAGACTACCTACGACCAGTCATGGATTAGATTAG

>YBR241C 2.5 Hot
ATGGCTGAAACTGAAAGACTAATGCCTAACGGTGGTTCCCGCGAAACAAAACCGTTAATTACGGGCCATTTAATC
TTGGGCACGATAGTTGCTTGTTTGGGGTCGATCCAGTACGGATACCACATCGCAGAGTTGAATGCGCCTCAAGAA

TTCTTATCGTGCTCCAGATTTGAGGCACCAGACGAAAACATCTCATACGATGACACTTGGGTTGGGCAACACGGA
CTCAAGCAATGCATTGCGTTAACCGATTCTCAATACGGCGCAATTACGTCTATATTCAGCATAGGCGGACTGTTT
GGGTCGTACTACGCCGGTAACTGGGCCAACCGATACGGAAGAAAGTACGTGAGCATGGGCGCCTCGGCCATGTGC
ATGGTTTCTTCTCTGCTGCTTTTCTTTTCCAACTCCTACTTGCAGTTGCTATTTGGCAGGTTCCTGGTAGGCATG
TCCTGCGGCACCGCCATTGTGATCACACCCTTATTTATCAACGAGATTGCCCCCGTGGAGTGGAGAGGCGCTATG
GGATCTATGAATCAGGTTTCTATCAATCTAGGTATCCTGCTGACCCAAACCTTGGCACTCAAGTACGCAGACTCC
TATAACTGGAGGTGGCTTCTCTTTTCCGGATCCGTGATTGCGGTAGCGAATATTTTAGCTTGGCTCAAAGTGGAC
GAATCTCCAAGGTGGCTGGTGAGCCACGGATTCGTCTCGGAAGCTGAAACTGCTCTGTTCAAGTTGCGTCCAGGA
ACGTACCAACAAGCGAAGCAAGAAATTCAGGATTGGCAGCGTAGCCATGGGCACAACCGCGACCCGGAGTCAAGC
GAAGAGACTCATTCAGGACCCACACTATGGCAATATGTTACCGATCCCTCATACAAGAAACCGCGCACAGTTATA
CTCGCCATCTTGTCGTGCCAGCAATTTTGCGGCATCAACTCTATTATCTTCTACGGTGTAAAAGTGATCGGCAAG
ATTCTACCGGACTACTCGATTCAGGTAAATTTTGCCATTTCCATCCTCAATGTCGTTGTCACTCTAGCAGCATCT
GCGATCATCGACCACGTCGGGCGCCGTCCATTATTGCTTGCCTCGACTACGGTGATGACCGCCATGTCGCTGCTG
ATCAGTGTAGGTCTCACCTTGAGCGTATCCTTTTTACTGGTCACGGCCACTTTTGTCTATATTGCCGCATTCGCC
ATCGGTCTGGGACCCATCCCCTTCTTGATCATTGGTGAGCTATCCTACCCGCAGGATGCCGCTACAGCTCAAAGT
TTTGGTACTGTGTGCAACTGGCTTGCCACCTTCATCGTCGGATACCTCTTCCCGATAGGCCACGGCCTCATGGGC
GGCTACGTATTTGCGATCTTCGCCGCCATCGCTGCCATGTTTGCTACATATGTGTACAAGCGTGTACCCGAGACA
AAGGGCAAGACTACATACAGCGAGGTTTGGGCTGGCTACTGA

>YDR036C 1.96 Hot
ATGCTCAGAAATACGCTAAAATGTGCCCAATTGTCGTCTAAATACGGATTTAAAACTACCACACGTACATTCATG
ACTACTCAACCCCAGCTAAATGTCACCGACGCACCACCTGTGCTATTTACCGTTCAAGATACAGCTAGAGTTATC
ACGCTAAATAGGCCCAAAAAGCTCAATGCTTTGAACGCCGAAATGTCAGAATCCATGTTCAAGACTTTGAACGAG
TATGCAAAGAGCGATACTACAAACTTAGTCATTTTAAAGTCATCCAACCGACCACGTTCGTTCTGTGCTGGTGGT
GATGTAGCTACTGTGGCAATATTCAATTTTAACAAAGAATTTGCCAAGTCCATCAAATTTTTTACTGATGAATAT
TCTTTGAATTTTCAAATAGCAACTTACTTGAAACCAATTGTTACCTTCATGGACGGTATCACCATGGGTGGCGGC
GTTGGTCTATCCATTCACACGCCCTTTAGAATTGCTACAGAAAACACCAAATGGGCCATGCCCGAGATGGACATT
GGTTTTTTTCCAGATGTAGGCTCAACTTTTGCTCTCCCTAGAATCGTGACATTGGCTAACTCAAACTCACAAATG
GCCCTGTATCTATGTCTTACAGGAGAAGTAGTCACAGGAGCAGACGCTTATATGCTCGGCTTAGCGTCTCATTAC
GTCAGTAGTGAAAATTTAGATGCTTTGCAGAAAAGATTAGGTGAAATTAGCCCCCCTTTTAATAACGATCCACAA
TCTGCATACTTCTTCGGGATGGTTAACGAATCCATCGACGAATTCGTATCACCATTACCAAAAGATTATGTTTTC
AAGTATTCTAACGAGAAATTAAACGTTATTGAAGCCTGTTTTAACTTGTCTAAAAATGGTACTATTGAAGACATA
ATGAATAACTTACGTCAATATGAAGGTTCTGCGGAAGGTAAGGCTTTCGCACAAGAAATCAAAACGAAATTGTTA
ACCAAGTCACCATCCTCTCTTCAAATCGCCTTGAGATTGGTGCAAGAGAATTCCAGAGATCACATAGAATCTGCT
ATCAAAAGAGACTTATACACAGCAGCTAACATGTGCATGAACCAGGACTCTTTGGTGGAATTCTCTGAAGCCACA
AAGCATAAACTTATTGATAAACAAAGGGTCCCGTATCCATGGACAAAGAAGGAACAGTTATTTGTATCTCAGTTG
ACATCTATCACATCTCCTAAACCATCGCTACCAATGTCATTACTAAGAAATACCTCGAATGTTACTTGGACTCAA
TATCCCTACCATTCTAAATACCAATTGCCTACAGAACAGGAAATCGCTGCGTATATTGAAAAGAGAACGAATGAT
GACACTGGCGCCAAAGTTACCGAAAGAGAAGTACTAAATCACTTTGCCAATGTGATTCCTTCTAGAAGAGGGAAA
CTGGGTATCCAATCGCTATGTAAAATTGTTTGTGAAAGAAAATGTGAAGAAGTTAACGATGGCTTAAGATGGAAA
TAA

>YHR096C 1.96 Hot
ATGTCGGAACTTGAAAACGCTCATCAAGGCCCCTTGGAAGGGTCTGCTACTGTGAGCACAAATTCTAACTCATAC
AACGAGAAGTCAGGAAACTCGACTGCTCCTGGTACCGCCGGTTACAACGATAATTTGGCACAAGCTAAACCCGTC
TCAAGTTACATTTCCCATGAAGGCCCTCCCAAAGACGAACTGGAAGAGCTTCAGAAGGAGGTTGACAAACAACTA
GAGAAGAAATCGAAGTCGGATTTACTATTTGTATCCGTCTGCTGTTTGATGGTTGCTTTTGGTGGGTTCGTGTTT
GGGTGGGATACTGGTACTATATCTGGTTTTGTCAGGCAAACAGACTTCATTAGGCGATTTGGCAGCACCCGTGCA
AACGGGACTACCTATCTTTCCGATGTCAGAACCGGTTTGATGGTTTCTATTTTCAACATCGGCTGCGCTATCGGA
GGTATAGTTTTGTCAAAGCTCGGTGATATGTATGGACGTAAGATTGGTCTGATGACTGTTGTCGTCATTTACTCA
ATTGGGATCATCATCCAAATCGCCTCCATTGACAAATGGTATCAATATTTCATTGGAAGAATCATCTCAGGACTG
GGCGTTGGTGGTATTACAGTTTTGGCGCCTATGCTAATTTCTGAAGTGTCGCCTAAGCAGTTGCGTGGTACTCTG
GTTTCATGTTACCAATTAATGATCACTTTCGGTATCTTTTTGGGATATTGTACTAATTTTGGTACCAAGAATTAC
TCAAACTCTGTCCAATGGAGGGTACCATTAGGCTTATGCTTTGCATGGTCTATTTTTATGATTGTTGGTATGACG
TTCGTTCCTGAATCCCCACGTTATCTGGTAGAAGTGGGAAAAATTGAAGAGGCCAAGCGGTCCTTAGCAAGAGCT
AACAAAACCACTGAAGACTCTCCTTTAGTAACTTTAGAAATGGAGAACTATCAGTCTTCTATTGAAGCTGAGAGA
TTGGCGGGCTCTGCTTCTTGGGGGGAATTGGTTACTGGTAAGCCCCAGATGTTCAGACGTACACTAATGGGTATG
ATGATTCAATCTTTACAACAGCTGACAGGTGACAATTACTTCTTTTACTATGGTACTACAATTTTCCAGGCTGTT
GGTTTGGAAGATTCATTTGAAACTGCTATTGTTTTGGGTGTTGTTAATTTTGTTTCGACTTTTTTCTCGCTATAT
ACCGTCGATCGTTTTGGTCGTCGTAATTGTTTGTTATGGGGCTGTGTAGGTATGATTTGTTGCTATGTCGTCTAT
GCCTCTGTTGGTGTTACCAGATTATGGCCAAACGGTCAAGATCAACCATCTTCAAAGGGTGCTGGTAACTGTATG
ATTGTTTTCGCATGTTTCTACATTTTCTGTTTCGCTACCACTTGGGCCCCCGTTGCCTATGTCCTTATCTCTGAG
TCGTATCCCTTAAGAGTACGTGGTAAAGCAATGTCGATTGCAAGTGCCTGTAACTGGATTTGGGGGTTCTTGATC
AGTTTTTTCACTCCATTTATTACTTCAGCAATCAATTTCTATTATGGCTATGTCTTTATGGGTTGTATGGTGTTC
GCATACTTTTATGTGTTCTTCTTTGTTCCAGAGACAAAGGGCTTAACATTAGAAGAAGTCAACGAAATGTATGAA
GAAAATGTGCTACCTTGGAAGTCTACCAAATGGATCCCACCATCTAGGAGAACAACAGATTATGACCTAGACGCT
ACTAGAAATGATCCGAGACCATTTTATAAAAGGATGTTCACTAAAGAAAAATAA

>YPR063C 1.64 Hot
ATGACTTTGAATAACGTTGCAAGGCCCGACCTTTGTGTTTCGTACAAGAAAATTGCGCCACCAAAAGGACTATAT
TCTGCCACACCAAGCATATCTGGTGTGGTTAACCAATCCATGCCTATGGCCGCTATTTTCTTGAGGAACAAGTTC
ATTGCCTGGTTCTCCCTGATTCAGAGTGTACACTACTACCTGAACACCGACGAAGACATCATCGTTGCCTATAAG
GAAAATAAGGCACCATCTCCAATGGACCAGCCTCCTGCAATCAAGCTATTCATGAGTTTAATCGGGTTATGCGTG
TGTTACATGAACTTAGTTTTCCCTCAACAAATAGCTCAACCATCGTCTAGCGGCTCTAAAGGTAATACTGAAACG
ACAATTGAGACAACTACCGAAGTAGAGACGGAAACAGCTAAGCAGTAG

>YPR038W 1.62 Hot
ATGGTTTGCTTCTCGATCTCTTTCAACCGAGCGTCGTTCTTCTCAGCAGCCGCCCCTTGCACTTCATCTATACCA
GACTTCGCCTTGATTAGGCCCGGCGTAGCGATGGATAGTTCATTAGACGAGAAAAACATCCACAGGCCTATGATT
CCTAATGCTGCAACTATTCTGATGGCATGGCTTCTTTTGACTATCTGTTTCATCCCTTCGTTCCTTGCACAATCT
GTCCAGACTTTCCTCTTTACAAATCACCAGCCTTCCCGTCTTCTTGTCTTCATCACCCACGTTTATTGTTTAATG
ATAAAGCCTTTTGTCCTTTATTTTTGGTTCCTTTTCCCGCTTCGTTTACCCGGTGGTTAA

>YGR096W 2.17 Hot
ATGTTCAAAGAGGAGGACTCGTTAAGAAAAGGGCAAAATGTGGCCGCGTGGAAAACTTTGTTGGCTGGCGCCCGTA
TCCGGGCTGCTTGCCCGTAGCATAACTGCTCCCATGGATACCATTAAGATCAGATTGCAATTGACCCCTGCAAAT
GGGCTGAAGCCCTTCGGAAGTCAGGTCATGGAGGTGGCCAGAAGCATGATAAAGAACGAAGGCATCCGGTCCTTT
TGGAAGGGTAACATACCCGGTTCTTTGCTGTACGTTACCTATGGCTCAGCGCAGTTTAGTTCATACTCCCTTTTT
AATAGATATTTAACTCCATTTGGACTAGAGGCGCGCTTACATAGTCTTGTTGTCGGAGCCTTTGCTGGCATAACC
AGTTCCATCGTTTCCTACCCGTTCGATGTTTTACGGACCCGACTAGTTGCCAACAACCAGATGCACTCAATGAGC
ATCACGAGAGAAGTGCGTGATATCTGGAAACTAGAAGGTCTCCCCGGATTTTTCAAGGGCTCCATCGCGTCAATG
ACAACCATAACTCTAACCGCATCTATAATGTTCGGAACATACGAAACAATCAGGATATACTGTGACGAAAACGAA
AAAACGACCGCCGCGCATAAAAAATGGGAACTGGCTACTTTAAATCACAGCGCTGGTACCATTGGCGGCGTCATT
GCTAAGATCATTACTTTCCCCCTCGAAACGATCCGTAGGCGAATGCAGTTCATGAATTCGAAGCATCTGGAAAAG
TTCTCCCGTCATTCGTCTGTATACGGTTCTTATAAAGGGTACGGATTCGCAAGAATCGGTTTACAGATTTTGAAA
CAAGAAGGTGTGAGTTCGCTTTACAGAGGTATTCTGGTGGCCCTTTCCAAGACAATACCCACCACTTTTGTGAGT
TTCTGGGGGTATGAAACTGCCATCCATTATTTGCGGATGTACTAA

>YJR080C 1.61 Hot
ATGATAAGCCCAAGGGTGAATACTCGAGTTTGGCAGAGATCAATTTCTCTTTTGAGCCCACAAGCGGCCAAGACG
GAGAGTAATGTCGTAACTAAAGAAAGGACATATATCGAGAACTTAAGCAAGGATATTGCGACTTCCCGTTTCAGG
CTAGTAGACGAAAATGGTAAAATTGCATCTATCACTGTTCAACCAGATATTCCAATTTGTATCAAAAAGGATTGC
CTAGTTTCCATTCACAATTTGAACCATTTATCCCTTTCCTATAAATGGCTGAATTTTTGGTCAAACCTGATTAAA
TTTCGTTCATTCAAATCTTCATTGTTTCATAGAATTATTGGATCCTCCGTTTTAGAAATTCTGGCTGCCCCAAAC
TTTCAAACATCGAGAAGACCTTTCGATTCTTCAAGAAGCTTGTCCGTACTGAATTTGACCGGGACAAAGGATTGG
AATGTATTTGGCAAGGATTCAATTATCGCATTTGAACAGAACTCCAGTCTGGAAATCAAATCGCCTATCTTTCCC
TCTGCAAGGTCTTTAGTCTCGAACTCATCAAAATCACAACTACCACGGAAATTTCAAATTCTGAATGGGAGAGGA
AATGTGTTAGTATGTGGAGGTGGGTTGGTATATTCAATTGAGTTAATTGATGAAAGCGACAAAATTCTGGTTAAT
TCCAGAAACATTTTGGCCATCAACGGCCAAAGTCAATTGGATATTGCAAATTCAGTGGAGAGACAAGAGTTACAC
GTGGAAGGCGCGTACGTCGGAGATTCAAGTAATGATACGGTGGCACCTAAATTCATTAAGAACCAAACGCTAAAA
TCTGCTTACGGACATACTGTTCAATTTTTCAAAAGAATGCGCAGTTGGATCCGTAACCAGTACGAAAAAAGATAC
ATTTATGGTGTCGACTCGTACTTTATGAAGATTAAAGGTCCAAGAACAATATTGATTCAAACGCATGAAATGACA
ACATCCAAAGATAATATTCTAACTAAACTAACGTCCAAGGGCCACGTTAAGAAGAGCAACGTGAATGATAACGGT
GTCAATTTAGAGAAACAAGTGGCTAACGATGTAAATTCAAAGATCATTGAGCTTGCCAACAGACCCTCACTGTTT
ATTGCGACCGTCTCGCAAGATGGGAGGGTTGATTTTCAAAGTACTTCGAAGTTCACATAA

>YJR049C 1.64 Hot
ATGAAGGAGAATGACATGAATAATGGCGTAGATAAATGGGTAAATGAGGAAGATGGTCGAAATGATCATCATAAC
AACAATAATAACTTGATGAAGAAGGCCATGATGAACAATGAGCAAATTGATAGAACTCAGGATATCGACAACGCC
AAAGAAATGTTGAGGAAAATATCAAGTGAAAGCAGCTCGCGCAGAAGCTCCCTGTTGAATAAAGATTCATCTCTC
GTGAACGGCAATGCAAACAGTGGCGGTGGTACGAGCATTAACGGAACAAGAGGAAGTTCTAAGAGTAGTAATACA
CACTTTCAGTATGCCTCCACGGCGTATGGTGTAAGAATGTTGAGTAAAGATATATCTAATACCAAAGTGGAACTG
GATGTGGAAAATTTGATGATTGTTACGAAACTCAACGATGTCTCACTGTATTTCTTAACAAGAGAGTTGGTAGAA
TGGGTTTTGGTACATTTTCCACGTGTGACTGTTTATGTGGATTCCGAATTGAAAAACAGCAAAAAATTTGCCGCT
GGCGAGTTATGTGAAGATAGTAAATGTAGAGAATCAAGGATCAAGTATTGGACAAAGGATTTCATCAGGGAACAT
GATGTTTTCTTCGATTTGGTAGTGACTTTGGGTGGCGACGGTACTGTTCTTTTTGTAAGTTCCATTTTTCAGAGA
CATGTACCACCCGTTATGTCGTTTTCATTAGGGTCTCTAGGATTTTTAACAAATTTTAAGTTTGAACATTTCAGG
GAGGATTTACCTCGGATTATGAATCATAAAATCAAGACAAATTTACGGTTGAGGTTGGAGTGCACAATTTATCGT
AGACACCGCCCTGAAGTAGACCCAAACACGGGGAAGAAAATATGTGTGGTGGAAAAACTAAGCACACACCACATT
TTGAACGAAGTGACCATCGATCGTGGTCCAAGTCCTTTTCTATCCATGTTAGAATTGTATGGTGACGGCTCATTA
ATGACCGTTGCGCAGGCGGACGGACTGATTGCTGCTACTCCGACTGGGTCCACGGCCTATTCTTTGAGTGCAGGT
GGGTCATTGGTATGCCCAACCGTCAATGCAATCGCTTTAACACCCATTTGTCCACATGCATTGAGTTTCAGACCC
ATCATCTTACCAGAAAGTATAAATTTAAAAGTGAAAGTCTCGATGAAGTCAAGGGCTCCAGCATGGGCGGCTTTT
GATGGGAAAGATAGAATTGAATTGCAAAAAGGTGATTTTATAACCATATGCGCCAGCCCATATGCTTTTCCAACC

GTGGAAGCCTCGCCCGATGAGTTTATTAACAGTATCAGTCGACAACTAAACTGGAATGTGAGGGAACAACAAAAG
TCCTTTACGCATATTTTGTCCCAAAAGAACCAAGAAAAATATGCACATGAGGCGAACAAAGTCAGAAATCAAGCA
GAACCTTTAGAGGTAATAAGAGATAAATACTCTCTGGAAGCAGACGCTACTAAGGAAAACAACAACGGAAGCGAT
GATGAGAGCGACGATGAGAGTGTAAACTGCGAAGCTTGCAAATTAAAGCCTTCGAGCGTCCCAAAACCTTCTCAA
GCAAGGTTTTCAGTATAA

>YHR150W 1.74   Hot
ATGAGTGAGACCAGCTCAAGTCGCAGGAGCGCGAGTAAAGACGCTGTCAAATCTTACTTCGCAGGCAAATACAAT
AAAGTGTTGGATTCTATACTGGAGGCAGAAGCAGCAATATCAAAATCGCCAACTGTAGCAGAAGATCTATCAGGA
AGCTCCTCCAGCGGAAATTCTGAGATGAGCCATCCTTCGCTAACCGCCTCCTCCGCAACAAGCCAAGGCATTAGC
AAAAAGGAACTACTCCAACAAATTGCTGGTTCATTATTCAGTACGTCCATTGAGCGCCTGAAGACTGCCCACTCA
TCAGAAGTATCAAGTACTCCGGAATATTCAGTAAATGATTCGTATGGAGAGCAAGAGTGCAGGGAGTGCGATGGT
GTTTTCAAGTGTTCAGCGCACTTTGAGGGCGCGCCCGAATATTATGACGATGAAACCGAATCAGGGCCAGCTCTA
GAACCTATGACTTCCAATAGTGAAAAAGATCCTTTTATCGATGTATTTTTGGATAAACTAATATCGAGGTTGGTC
CCTGAAAAGCTACCAGAAAGAGAGCATTTTAGTAGCAAAACCACCATTGAGCATGATCTAGACACAGGAAGGGTT
CCCGTGTTTTCAGCAACTACATTGGGTAGTAATTTCAAAAAATTGTCTAAGAAAATGGGCTCAATATTTGAACTC
CAGGACTCCATAGTTCGACTGCTGACTTGGAGAAACCCTACCGGGACGGTGACGTCGTTAATATTATTCACTTTG
ATATGTTTTAATCCAATGTACTTGGTAATTTTGCCTATATTCCGCTTTGTCTATGGTATTGTAGTTCCTGGCTAT
GTGCGTAAACATCCTTTACAAAGGAGTATATATCCTCTGAAACGGAATCACGGATCATCATTGTTATATGATGTG
TGCTATGAGGGAAAAAATGAGTACAGCTACGGTCAACAGTTTTTCTCCAAATCTTTTATGGACACTTTGGAATCA
AGAAATCAAGAAATAGACGAAATATCAGAATTAGATAAAAGAACCGAAAACACAGGGGAATTGAAACAAGGAATG
AAAGTACTAATTAATTTACGCGATATGCAAAACATGACCTCTGGTACCCTTCATGTTATTGAGGCAATCAATAGT
TTTCTTCGTAAGTCTTCTAGTTTTCAAAACGAAGAGTGCTCCACTAAACGTTTTTTTACAGGATTTCTTTTGATT
GTTTTCTTAAAAATATTATCACCATTTGTGAATTGGAGCTATGTATGTAGCATATTCGCCTGGTGCTTACTGATA
TATATGCATCCAAGAGCTCATCCAAAAATAATAAGTTTTTTCAAAACAGGAACAATGGGAAAAGAATACAAAAAC
TTGAAAAAAAGAGAACACCAGGCGCTTAACATGATATTCGATGAACAACCGGAAACTAAATTCATCGAGATTTTT
GAAATATATAAAAAGGCCTTACTGCCGAACGACTGGAAGTTTTTCCGATATTCAAATAGAATTTTCGATCCTCAA
GATCCGTATAGAAGAGCACAGCAATTCCCACCGGGAGTAGATTCTTTGGCTGATGTTATTCCCCCCACAGGCTGG
TCATTTGATCCTAATTTTGAGTGGAAAATTGACAATGATGTGGATAGGTGGGTGGTAGAACGTGGGTTGAATCTG
CCAATTACCGGCGAATTCTTATTTGATCCAATGTTCAAGAGGAGGAGACTGATACATCGTGTTATTAAGAATGCA
ACACCAGTAGCATAA

>YJL078C 1.58   Hot
ATGCTGGAGTTTCCAATATCAGTTCTGCTAGGATGCCTAGTAGCCGTCAAGGCACAAACCACGTTTCCAAACTTC
GAGAGCGATGTGCTGAACGAGCATAACAAGTTCAGAGCGCTACATGTTGACACAGCGCCGCTCACCTGGTCCGAC
ACTCTGGCCACCTATGCGCAGAACTACGCCGACCAATATGATTGTTCGGGTGTCTTAACGCATTCCGATGGCCCA
TATGGTGAGAACCTTGCCCTTGGTTACACAGACACGGGAGCGGTGGACGCCTGGTACGGGGAGATAAGCAAGTAT
AATTATTCAAATCCCGGATTTTCTGAATCCACGGGTCACTTCACACAGGTGGTTTGGAAGTCAACCGCCGAGATT
GGATGTGGTTATAAATATTGTGGTACGACATGGAACAATTATATTGTGTGCTCCTACAACCCTCCTGGAAACTAC
CTGGGTGAGTTTGCAGAGGAAGTGGAACCACTTATAAGCACTGTTTCCTCGTCCTCATCCTCGTCCTCTTCTACC
TCAACTACATCAGACACAGTCTCCACCATCTCATCCAGTATTATGCCCGCTGTAGCGCAAGGGTATACAACAACG
GTATCGTCTGCGGCTAGCAGCAGTTCTTTAAAATCGACGACCATAAACCCTGCCAAGACCGCTACCCTCACTGCG
TCCTCTTCTACCGTAATTACTAGTAGCACAGAATCAGTTGGATCCTCCACTGTCTCATCAGCCTCAAGCTCTTCT
GTCACTACTTCCTATGCTACCTCCTCGAGTACCGTCGTCTCTAGTGATGCTACTTCATCCACTACCACCACCTCA
TCGGTTGCTACATCGTCCAGTACCACTTCTTCCGACCCTACCTCGAGCACTGCTGCTGCTTCTTCTTCTGATCCT
GCCTCAAGTTCCGCTGCCGCTTCCTCCAGCGCGAGTACCGAGAACGCCGCTTCTTCTAGCAGCGCCATCTCGAGC
TCTTCATCAATGGTTTCTGCTCCTTTGAGTAGTACTCTTACTACTTCCACCGCAAGCTCCAGAAGTGTAACTTCC
AATTCAGTTAATTCTGTTAAGTTTGCAAACACAACTGTGTTTTCTGCTCAAACAACCTCTTCTGTAAGCGCCTCA
TTATCATCATCTGTAGCTGCTGACGATATTCAGGGTAGCACTTCCAAGGAGGCCACAAGCTCAGTTTCCGAACAT
ACTAGTATAGTAACTAGTGCAACTAATGCTGCCCAATATGCAACGAGACTTGGGTCATCTTCCAGAAGTTCTTCC
GGGGCCGTCTCTTCCTCAGCTGTGTCGCAATCTGTTCTGAATTCCGTTATAGCCGTCAACACCGACGTATCTGTA
ACCTCAGTTAGTAGCACAGCCCATACCACAAAGGACACCGCCACCACTTCAGTAACCGCCTCAGAAAGTATCACT
TCGGAAACTGCTCAGGCTTCAAGTTCAACAGAGAAGAATATTAGTAACAGTGCCGCCACATCGAGTAGCATTTAC
TCCAACAGTGCTTCTGTGTCAGGACACGGTGTAACATACGCTGCCGAATACGCCATTACATCCGAGCAATCCTCT
GCGCTTGCCACATCTGTGCCTGCTACAAATTGCTCTAGTATCGTGAAGACCACAACTTTAGAAAATTCGAGTACC
ACAACCATCACAGCCATTACTAAGAGTACTACAACCTTGGCCACTACTGCTAACAACTCCACAAGGGCAGCTACC
GCAGTAACCATAGATCCCACATTGGACCCTACCGACAACTCAGCTAGTCCAACCGACAATGCTAAACACACCTCT
ACATATGGATCTTCTTCCACAGGCGCATCTTTAGATAGCTTACGCACAACCACCAGTATTAGTGTCTCAAGCAAC
ACCACACAGTTAGTCTCTACCTGCACTTCCGAGAGCGATTATTCCGATAGTCCTAGCTTCGCCATCTCCACTGCC
ACCACCACTGAAAGCAATCTGATCACAAACACCATCACAGCTTCTTGTAGTACGGATAGTAATTTCCCTACCTCC
GCTGCTTCTTCTACAGATGAGACGGCCTTCACTAGAACAATCTCGACATCTTGTAGCACTTTGAACGGCGCCTCA
ACCCAAACCAGTGAGCTAACCACATCGCCTATGAAAACCAACACGGTGGTTCCAGCTTCTTCTTTCCCTTCAACT
ACAACCACTTGTCTAGAAAATGATGACACTGCCTTTTCTAGTATCTACACTGAAGTCAACGCCGCAACTATCATT
AACCCCGGAGAAACATCTTCTCTCGCTAGCGATTTCGCCACATCTGAAAAGCCAAACGAGCCCACTTCTGTCAAA
TCCACCTCAAACGAAGGCACCTCTTCCACAACAACAACCTACCAACAGACTGTTGCTACACTGTATGCCAAGCCC
TCCAGCACAAGCCTAGGTGCAAGAACAACTACTGGTAGCAACGGTCGTTCAACTACCAGCCAACAAGACGGGTCT
GCCATGCATCAGCCAACTTCCTCGATCTACACTCAACTAAAAGAAGGCACATCAACCACCGCAAAACTTTCTGCA

TACGAAGGTGCTGCAACACCTCTTTCCATTTTCCAGTGCAATAGTCTAGCTGGAACGATTGCCGCTTTTGTCGTA
GCTGTTCTGTTCGCCTTCTAG

>YDR174W 1.68   Hot
ATGACTACAGATCCTTCTGTCAAATTGAAGTCCGCCAAAGACTCCCTCGTCTCCTCCCTCTTCGAGTTATCAAAA
GCTGCTAATCAGACAGCCTCCTCTATTGTTGATTTCTACAATGCCATCGGCGACGATGAAGAAGAGAAGATAGAG
GCTTTCACCACTTTGACTGAGTCTTTGCAAACATTGACTTCTGGTGTAAACCACTTGCACGGCATCAGCTCGGAG
CTGGTTAACCCTATTGATGATGATAAAGATGCTATTATCGCTGCTCCAGTCAAGGCTGTAAGAAGAAAGATCGAA
CGTGATCCAAATGCTCCAAAGAAGCCACTGACTGTTTTCTTTGCTTACTCTGCGTACGTTCGTCAAGAACTTCGT
GAAGACAGACAAAAGGCTGGCTTGCCACCTTTATCCTCAACTGAGATTACTCAAGAAATCTCCAAGAAATGGAAG
GAATTGAGTGATAACGAAAAAGAAAAATGGAAGCAGGCTTACAACGTCGAGTTGGAGAATTACCAAAGAGAGAAA
TCCAAATACTTAGAAGCAAAGAAAAACGGTACCCTACCACCAGCTTCCTTGGAAAACGGTCCAACTCATGCGCCG
GTTCCAATTCCTTTCAGTTTACAGCACGCCGCTGAACCTCCTGTGGAAAAGAGACCTCACGATGATGATGGATCT
TCGGAAAAGAAGAAGAAGAAGAAGAAGAAGGATAAGAAGAAGGACAAATCCAACTCTTCTATTTGA

>YPL252C 2.1Hot
ATGCTGAAAATTGTTACTCGGGCTGGACACACAGCTAGAATATCGAACATCGCAGCACATCTTTTACGCACCTCT
CCATCTCTGCTCACACGCACCACCACAACCACAAGATTTCTGCCCTTCTCTACGTCTTCGTTCTTAAACCATGGC
CATTTGAAAAAACCGAAACCAGGCGAAGAACTGAAGATAACTTTTATTCTGAAGGATGGCTCCCAGAAGACGTAC
GAAGTCTGTGAGGGCGAAACCATCCTGGACATCGCTCAAGGTCACAACCTGGACATGGAGGGCGCATGCGGCGGT
TCTTGTGCCTGCTCCACCTGTCACGTCATCGTTGATCCAGACTACTACGATGCCCTGCCGGAACCTGAAGATGAT
GAAAACGATATGCTCGATCTTGCTTACGGGCTAACAGAGACAAGCAGGCTTGGGTGCCAGATTAAGATGTCAAAA
GATATCGATGGGATTAGAGTCGCTCTGCCCCAGATGACAAGAAACGTTAATAACAACGATTTTAGTTAA

>YFR025C 2.17   Hot
ATGCACTCACACCATTCACACTCCGGTGACTATAGTGCCCACGGTACGGACCCTTTGGATTCCGTGGTCGATCAA
GTGGTCAACCTCAACTTTCACACGTACTGTTTGACAGAGCACATACCAAGAATTGAGGCCAAGTTTATATACCCC
GAAGAGCAGTCATTGGGCAAGAATCCTGAGGAAGTCATAACCAAGCTAGAAACATCGTTCAAGAATTTCATGAGT
CATGCGCAAGAAATCAAGACTCGTTATGCTGATAGACCCGATGTGCGGACTAAATTCATTATAGGAATGGAGATC
GAAAGTTGTGACATGGCTCATATCGAATATGCAAAGCGACTCATGAAGGAGAATAATGATATTTTGAAGTTTTGT
GTGGGTTCGGTCCATCACGTCAACGGGATCCCTATTGATTTCGACCAACAACAATGGTACAATTCATTGCATTCC
TTCAATGATAATTTGAAACATTTTCTCCTGTCTTACTTCCAATCACAGTACGAAATGCTGATCAATATTAAACCG
TTGGTCGTGGGTCACTTCGACCTTTACAAATTATTTTTGCCCAATGACATGCTAGTAAACCAGAAATCGGGCAAC
TGCAACGAAGAAACCGGAGTTCCTGTAGCTTCACTGGACGTCATCAGTGAATGGCCAGAAATATACGATGCAGTT
GTAAGAAATTTACAATTTATAGACTCCTATGGCGGCGCAATTGAAATCAATACGTCCGCATTAAGAAAGCGCCTC
GAGGAGCCGTACCCCAGCAAAACCTTATGTAATCTGGTCAAGAAGCACTGTGGATCCAGATTTGTTCTAAGTGAT
GACGCACACGGCGTGGCGCAAGTGGGTGTGTGCTATGACAAGGTAAAGAAATACATAGTAGACGTGCTACAATTA
GAGTATATTTGCTACCTTGAGGAAAGCCAATCACCAGAGAATCTGTTAACTGTAAAGAGATTACCCATTTCGCAA
TTCGTTAATGATCCCTTTTGGGCCAATATATAA

>YFL016C 1.63   Hot
ATGGCTTTCCAACAAGGTGTATTGTCAAGGTGTTCCGGTGTCTTTAGACACCATGTGGGACATTCTCGCCATATC
AATAATATTCTTTATAGACATGCCATCGCGTTTGCATCCATCGCTCCACGAATACCAAAATCTAGCTTCCATACT
TCTGCAATCAGAAACAACGAAGCATTCAAGGACCCGTACGATACTTTAGGCTTGAAGAAATCTGCTACAGGTGCG
GAAATCAAAAAAGCATACTACAAACTGGCAAAGAAGTACCACCCGGATATCAACAAGGAACCGGATGCTGAGAAG
AAATTCCACGATTTACAGAACGCTTATGAAATTCTGTCAGACGAAACGAAGAGGCAGCAGTACGATCAATTTGGG
CCCGCTGCCTTCGGCGGCGGCGGTGCCGCTGGAGGTGCCGGTGGTGGTAGTGGCTCTCCCTTTGGTTCCCAATTT
CATGATTTCTCAGGATTCACCAGTGCAGGCGGCTCGCCATTTGGCGGTATCAATTTTGAAGACCTGTTTGGTGCT
GCATTTGGTGGTGGTGGCCGCGGTAGCGGTGGCGCAAGCAGGTCGTCATCTATGTTCAGACAATATAGGGGCGAC
CCAATCGAGATTGTCCATAAAGTGTCTTTCAAGGACGCAGTGTTTGGGTCCAAGAACGTTCAGTTAAGATTCTCT
GCGCTGGACCCTTGTAGTACCTGTTCAGGGACGGGAATGAAACCAAACACGCATAAGGTCAGTTGTAGCACTTGT
CACGGAACAGGAACCACTGTTCACATTAGGGGCGGATTTCAGATGATGTCGACTTGTCCTACTTGCAACGGTGAA
GGTACCATGAAACGGCCTCAGGACAATTGTACCAAGTGCCATGGTGAGGGTGTTCAGGTCAACAGGGCAAAGACA
ATTACGGTGGACTTGCCACATGGATTACAGGACGGCGACGTGGTCAGGATCCCTGGCCAAGGCTCATACCCTGAC
ATCGCTGTAGAGGCGGACTTGAAAGATTCAGTCAAGTTATCAAGAGGTGATATTTTGGTGAGAATTCGTGTCGAC
AAGGATCCCAACTTTTCGATAAAGAACAAGTACGATATTTGGTACGACAAGGAGATTCCTATAACCACAGCTGCA
CTTGGTGGTACTGTCACTATCCCCACTGTGGAGGGACAAAAGATCAGGATAAAGGTCGCTCCAGGGACTCAATAC
AATCAAGTGATATCCATTCCTAACATGGGTGTTCCTAAAACATCAACCATTCGCGGTGATATGAAAGTCCAGTAC
AAGATCGTTGTTAAGAAACCGCAATCGCTGGCAGAAAAATGCTTGTGGGAGGCACTGGCAGATGTCACCAACGAT
GACATGGCCAAGAAAACCATGCAACCGGGCACAGCCGCGGGTACAGCCATTAATGAAGAGATACTGAAGAAACAA
AAACAAGAAGAGGAAAAACACGCAAAAAAGGATGACGACAACACTTTGAAGAGACTAGAAAATTTCATTACCAAC
ACATTCAGGAAGATCAAAGGTGACAAAAAAAATTAA

>YHR175W 1.56   Hot
ATGGATGATAAGAAAACATGGAGTACAGTCACTTTGAGAACCTTCAATCAGCTGGTAACGTCTTCGTTAATTGGA
TACTCAAAAAAGATGGATAGCATGAATCACAAGATGGAAGGAAATGCGGGCCACGACCACAGTGATATGCATATG
GGAGATGGAGATGATACCTGTTCGATGAATATGCTATTTTCGTGGTCATACAAGAATACGTGTGTCGTCTTTGAA

TGGTGGCATATCAAGACCCTGCCTGGACTGATTTTAAGTTGTTTAGCAATTTTTGGTCTAGCCTACCTCTATGAG
TACTTAAAGTACTGTGTCCATAAGAGACAATTATCCCAGAGAGTATTGTTACCAAATAGATCTCTGACCAAGATC
AACCAAGCCGACAAAGTGTCCAATAGTATTCTATATGGTTTGCAAGTGGGATTCTCATTCATGCTCATGCTTGTA
TTCATGACTTATAATGGTTGGTTAATGTTAGCTGTCGTGTGTGGGGCAATATGGGGTAATTACAGCTGGTGTACT
TCGTATAGTCCTGAGATAGATGACAGCTCCCTCGCCTGCCATTAA

>YDR405W 1.52   Hot
ATGCCACGATTGACGGTTGGAACGAAGAATATGTTGTATCCTTTGCAAAAAACACTTGCAGTAGGAAGTTGCAAG
CCAGAGCAGGTTCCAATAAGATCACTAGCCTCAGTGGTGGAGAGTTCCTCCAAAATACTCGACAAAAGCGGCAGC
GATCGCGAAGTAGATATAAACGTAAGCGAGAAGATTTATAAGTGGACAAAGGCGGGCATAGAGCAGGGAAAGGAG
CATTTCAAAGTTGGAGGTAACAAAGTCTACTTTCCCAAGGCCAGGATCATCCTCTTGAGGCCCAACGCGAAGCAC
ACACCGTATCAAGCAAAATTCATCGTCCCAAAATCGTTCAACAAACTGGATCTCAGAGACTATCTTTATCATATC
TACGGCTTAAGAGCAATGAACATTACGACGCAGCTGCTTCATGGGAAGTTTAACCGTATGAACCTACAGACGACG
CGGTTTAGAGAACCGCAGATCAAGAAAATGACTATCGAGATGGAAGAACCCTTCATTTGGCCCGAAGAACCCCGT
CCGGACGAAAACTCCTTCTGGGACAGCACCACGCCTGACAACATGGAAAAGTACCGTGAAGAAAGACTTAACTGC
TTGGGCTCAGACGCCAACAAACCCGGCACAGCATTCGACGGAGTCGTGGGGCCTTACGAACGGGTGGCCCAGCCT
TTTATACCTCGCTTCTTAAAACGAGAGATAGACAATAAGCGAGAGCGTCACGCCGCCGAACTGCAGCGCGCAGAT
AAACTAATTGCCCTGAACAGGTACATAGAAGATCTACATTAG

>YPR183W 1.61   Hot
ATGAGCATCGAATACTCTGTTATCGTTCCCGCTTACCATGAAAAGCTGAACATCAAACCCTTAACAACCAGATTG
TTTGCCGGCATGAGCCCTGAAATGGCCAAGAAGACCGAGTTGATCTTTGTCGATGACAACTCACAGGATGGCTCC
GTTGAGGAGGTGGACGCCCTAGCTCACCAAGGCTACAACGTCCGCATCATCGTCAGGACAAATGAACGTGGTCTA
TCCTCCGCCGTGCTTAAGGGTTTCTACGAGGCCAAGGGTCAGTACCTGGTATGCATGGACGCAGACCTACAACAC
CCTCCAGAGACCGTGCCCAAGTTGTTCGAGTCTTTGCACGATCACGCCTTTACTCTGGGCACCAGATATGCCCCA
GGTGTCGGCATTGACAAGGACTGGCCTATGTACAGACGCGTCATTTCCTCCACTGCTAGAATGATGGCCAGACCT
TTGACCATCGCCTCCGACCCCATGAGCGGGTTCTTCGGTCTACAAAAGAAGTACCTCGAAAACTGCAACCCTAGG
GACATCAACTCGCAAGGTTTCAAGATTGCCCTCGAGCTTCTCGCCAAGCTTCCTCTTCCAAGAGACCCTAGAGTG
GCGATCGGCGAAGTGCCATTCACTTTCGGCGTGAGAACGGAAGGTGAATCCAAACTGTCAGGTAAAGTCATTATC
CAATACTTGCAACAACTTAAGGAGCTCTACGTCTTCAAGTTTGGCGCCAATAACCTTATCCTTTTCATTACTTTC
TGGTCCATTCTGTTCTTCTACGTTTGCTACCAGCTATACCATTTGGTCTTTTAA

>YIL043C 1.72   Hot
ATGGCTATTGATGCTCAAAAGCTTGTGGTGGTCATCGTGATCGTGGTCGTGCCTTTGCTCTTCAAGTTCATTATC
GGACCGAAGACCAAGCCTGTGCTGGATCCCAAAAGGAATGACTTCCAATCATTTCCGCTGGTTGAAAAAACCATC
TTAACGCATAATACTTCGATGTACAAGTTCGGGCTACCTCATGCTGACGACGTACTCGGTTTACCAATTGGTCAG
CATATCGTAATTAAGGCCAATATCAATGGTAAGGATATTACCAGATCGTATACGCCCACATCGTTGGATGGAGAT
ACAAAGGGAAACTTTGAATTACTAGTGAAGTCTTACCCCACAGGTAACGTTTCTAAGATGATTGGAGAGTTGAAG
ATAGGTGACTCGATCCAGATCAAGGGCCCTCGTGGGAACTATCATTATGAGAGAAACTGCCGTTCCCATCTAGGG
ATGATTGCTGGTGGTACTGGTATTGCGCCCATGTATCAGATCATGAAAGCTATTGCCATGGACCCTCACGACACT
ACCAAGGTCTCTCTAGTCTTTGGGAACGTCCATGAGGAGGATATTCTGTTGAAGAAGGAACTGGAAGCGTTGGTG
GCCATGAAGCCTTCCCAATTTAAGATAGTTTACTACTTAGACTCTCCTGACCGTGAAGACTGGACTGGTGGTGTA
GGATACATTACCAAGGATGTCATCAAGGAACACTTGCCCGCTGCTACAATGGACAACGTTCAAATTTTGATCTGT
GGTCCTCCAGCCATGGTTGCCTCAGTTAGAAGAAGTACCGTGGACTTGGGGTTCAGACGTTCCAAACCGCTTTCC
AAGATGGAAGACCAGGTGTTTGTGTTTTAA

>YOL020W 1.92   Hot
ATGACCGAAGACTTTATTTCTTCTGTCAAGCGTTCAAATGAGGAGCTGAAGGAGCGAAAATCTAACTTTGGATTT
GTAGAATACAAATCCAAGCAATTAACATCATCCTCATCACACAATTCAAACTCATCGCATCACGATGATGACAAC
CAACACGGCAAGAGGAATATTTTCCAGCGATGTGTTGATTCTTTCAAGTCTCCTCTGGACGGCTCGTTCGACACA
AGTAATTTGAAGAGGACTCTGAAACCAAGGCACTTAATCATGATTGCCATTGGGGGCAGTATAGGTACTGGTTTG
TTTGTCGGTAGTGGTAAGGCAATCGCCGAAGGTGGTCCTCTTGGTGTCGTCATTGGTTGGGCTATTGCAGGCTCT
CAAATCATCGGGACGATTCATGGGCTAGGTGAAATCACGGTGCGGTTCCCCGTTGTCGGGGCCTTTGCCAATTAC
GGCACAAGGTTTTTGGATCCAAGTATAAGTTTTGTTGTTTCGACCATATACGTGCTACAGTGGTTTTTTGTATTA
CCCTTAGAAATCATTGCGGCGGCAATGACCGTACAGTACTGGAACTCGTCTATTGACCCCGTAATTTGGGTAGCC
ATCTTTTATGCTGTTATTGTTTCCATAAATTTGTTTGGTGTACGAGGTTTTGGGGAAGCGGAATTTGCATTTTCC
ACCATAAAAGCAATAACAGTTTGCGGATTCATTATATTGTGCGTTGTCCTCATTTGCGGTGGAGGTCCCGACCAC
GAGTTCATTGGCGCCAAATACTGGCACGACCCTGGTTGTTTGGCCAATGGGTTCCCGGGCGTTCTATCTGTTCTT
GTTGTTGCTTCGTATTCTCTCGGTGGTATAGAAATGACGTGCCTGGCATCAGGTGAAACGGACCCCAAAGGCTTA
CCAAGTGCTATCAAGCAAGTGTTTTGGAGGATCTTATTTTTCTTTTTGATATCACTGACCCTGGTAGGATTCCTG
GTTCCATATACAAACCAAAACTTACTGGGTGGTTCATCTGTGGACAACTCACCTTTTGTCATTGCCATAAAATTG
CATCACATTAAAGCCTTGCCCTCCATCGTCAATGCGGTCATCCTTATCAGTGTTTTGAGTGTAGGTAACTCCTGC
ATCTTCGCTTCAAGTAGAACGTTGTGTTCGATGGCCCACCAAGGCCTTATTCCATGGTGGTTTGGTTATATCGAC
CGTGCTGGTAGGCCACTGGTTGGCATTATGGCAAATTCACTATTTGGATTACTAGCATTTCTGGTCAAATCTGGC
TCGATGTCTGAAGTTTTCAATTGGTTAATGGCCATTGCCGGCCTGGCCACGTGCATTGTCTGGTTGAGCATCAAC
CTGTCACATATCAGATTTAGACTGGCGATGAAGGCTCAAGGGAAGTCTCTGGATGAATTAGAATTTGTTAGTGCT
GTCGGGATTTGGGGATCAGCGTATTCCGCTTTAATCAACTGTCTAATCTTGATAGCTCAATTCTATTGTTCACTA

TGGCCCATTGGTGGCTGGACCAGCGGCAAAGAAAGGGCTAAAATATTTTTTCAAAATTATCTGTGTGCCTTAATC
ATGTTATTTATATTCATCGTCCACAAAATTTATTACAAGTGTCAAACAGGCAAATGGTGGGGGTTAAGGCGTTA
AAGGACATAGATTTGGAAACCGATCGTAAAGATATCGACATCGAAATAGTCAAGCAAGAAATTGCCGAAAAGAAA
ATGTACCTAGATTCCCGTCCATGGTACGTGAGACAGTTCCATTTCTGGTGTTAA

>YFL030W 2.56 Hot
ATGACTAAATCTGTAGATACGCTGCTGATCCCAGGCCCCATTATCCTAAGCGGAGCAGTCCAGAAGGCCCTGGAC
GTTCCCTCTTTGGGCCACACCTCTCCTGAGTTCGTCTCTATTTTTCAAAGGGTGCTAAAGAACACAAGAGCTGTC
TTCAAATCCGCTGCTGCCTCGAAGTCTCAGCCTTTTGTGCTTGCCGGCTCTGGTACGTTGGGGTGGGACATATTT
GCCTCGAACTTTATTCTTTCGAAGGCCCCCAACAAGAACGTGCTGGTCGTATCCACTGGGACATTTTCTGACAGG
TTTGCTGACTGTCTGCGTAGCTACGGTGCGCAAGTAGATGTTGTTAGGCCCCTCAAGATAGGCGAATCAGTTCCC
TTAGAACTGATTACTGAAAAGTTGTCGCAAAATAGCTATGGGGCTGTCACGGTTACGCACGTTGACACTTCAACG
GCAGTGCTGTCCGATCTGAAAGCCATTTCGCAAGCGATTAAGCAGACTTCGCCGGAAACGTTCTTCGTAGTCGAT
GCTGTATGCTCGATTGGATGCGAAGAGTTTGAATTTGATGAATGGGGGGTGGATTTCGCCTTGACTGCTTCGCAA
AAGGCTATTGGCGCTCCAGCGGGTCTTTCTATCTCGCTATGCAGTAGCAGATTCATGGATTATGCACTCAACGAC
AGCAAAAAATGGTCATGTACATGGGTATTTTTCCTCGTTGAGAAGATGGACACCAATAATGGAAAATTATGAGGCT
GGTAAAGGAGCTTATTTTGCAACCCCACCCGTACAACTAATTAATAGCCTTGATGTAGCCTTGAAAGAAATTCTC
GAAGAGGGGTTGCATAAGAGATGGGATTTGCACCGTGAAATGAGTGATTGGTTTAAAGACAGTTTGGTTAATGGC
TTGCAATTGACATCCGTCAGTAGGTATCCTTCTAACATGTCTGCGCACGGGTTGACAGCCGTATACGTAGCAGAT
CCTCCTGATGTCATTGCTTTTTTAAAGTCACACGGTGTAGTCATTGCCGGAGGTATTCACAAGGACATCGGACCC
AAGTATATCCGTATTGGACATATGGGTGTGACCGCTTGCAACAAGAATTTACCGTACATGAAGAACTGCTTCGAC
TTAATAAAACTAGCTCTTCAGAGGAAAAAGTGA

>YBR220C 1.72 Hot
ATGGAACCTAAGCGAAAGAGCGGGTCACTAGCCAAGCATGATTTGCCGCAATTTTATCTTTTAATTATGTTATAT
TTGGCTCAAGGCATACCTGTAGGATTGGCCTTCGGTACCGTACCGTTTCTACTGAAATCTTTAGCAAAGGAGACC
TCGTTTACATCACTGGGAATTTTCTCTATGGCTACATATCCATATTCTTTAAAGATCATATGGTCACCAATAGTA
GACTCACTGTACAACAAGCGCATCGGTAGAAGAAGATCATGGATCATTCCAGTACAATTTGTTAGTGGATTTGTG
CTATGGGCATTAGGGTGGTGCATATCACAAGGCATAATCTTCGATGGTGTCGACGATGCGTTCCATAATCGCGGT
AATGGCACTTTACACAGTGTCAGTATAAAAAATTTGACGTGGTGGTTTGGCCTGTTAGTTTTTCTGTGTGCCACT
CAAGACATCGCAGTTGATGGTTGGGCGTTGACGATTTTGTCCAAAGAATCCCTATCATATGCATCTACCGCGCAA
ACAATAGGTTTGAATATTGGTTATTTTATGTCATTTACCATTTTCCTGTCGTTGAATTCCTCTGATTTCGCCAAT
AAGTATTTCAGAAACATCCCACTGGATCACGGGTTCATTAGTCTTGGTGGGTACATGAAATTCTCGGGCATGCTT
TACATTGTAATAACCATATATATCATCTTTTGCACCAAGGAAAAACCCTACGTAGAGTATTTGCCCAAAGTGGAG
CCCATAAATACAAGTGACGGAGGGTCAAAGCCGATAAGTATTGAGTATGACGACGGTGATGTGGTGTCAACTCAG
AATACAAGCAGTATAAAGTACATTTACCGCTGCTTTATAAAAGTGTTGAAATTGAAGTCCGTAAGAAGCCTAGCC
TTCATTCACATGATTTCGAAATTTGCCTTTCAATGCAACGAAGCCGCCACAAACCTGAAACTACTAGAGCAAGGC
TTCAAAAGAGAAGACTTGGCTGTGACAGTACTCATAGACCTGCCGTTCGAAATCATATTTGGGTACTATGTTGTT
AAATGGAGCTCCGACAAGGACCCCATGATTCGTGACAATAGAAGATTAAGAAACAGCACGGGCACCAACAAGGTC
ATCAAGTTCTTAGTTGGGGATGCCGGCGTTTTAACACCATGGTTGTGGGGCTTTTTGGGCCGTCTGGCAGCCGCG
GTCTTGGGAAGTTACGTGGTGAAGCAATTCCCCAAGGATGGTGAAATATCCACGGGTTATTTTTGTCTCGTGATA
TTCCAGCACCTCTTAGGTTCCTTCATGAATACTGTCCAGTTCATTGGAATATCGGCCTTCCATACAAGAGTTGCA
GACCCCGTGCTGGGTGGCACATATATGACATTGTTAAATACCCTCAGCAACTTCGGTGGGACATGGCCGCGGTTA
ATCATTATGTCCATGATCAACTACTTCACCGTGTATCAGTGCACTATTCCTGGCACAAATAAAGTATACGTAACT
CACGGCGGCAGCATGCAAGCGTGCACCGAGCTTTTGAATGGCACCGTGACCATCCTGCGTGACGGCTATTACATC
ACCAATCTCATATGTATTGTAGTCGGACTTTTCCTATATTTGGATATTTGAAAAGGAAAATCCTCCATTTACAA
AGTCTGCCAATCAGTTCCTGGAGATGTACGTAA

>YLR213C 1.84 Hot
ATGAGAATATCAATATTACAGCTGGTGCCGGTTGTTGGCTACATCGGATTTGCACTGGGAGAATTATACAAACCC
AAAAACTCCATTTCTTGTTCTCCAAATAACCCTTGTCCCGCAGAATGGCCGTGCTGCTCGCCGTATAATGAATGT
GGCGCTGGACCGATATGTGTCGGTGGCTGCAACGTGAGGTCATCTTTCGACGAGGAAAGCTGTGCTCCTATACCT
GCATTAGTCGCCAGTCAAAAACTTGAATTTGTTAGCACACCAAAGGTTCCTAAATTTATTGTGAACTATCAACCT
AAGCCACCAATAAGAGAGAGGGAAATGGACCGAATAAGGCAAATACCAAGGTGGGTGTAGTAGAAGGGGAGCTAAAT
TCCAAGAGAATCATACACTACGCCAAATTCTTGGTCACTCCAGACAGTAAAGAGGCAGAGAAAATGTTGGAAGAT
TTCGATTTCACACATAGCGGCTACACATCAATTGAAGCGAGTAGCGGGAATATTGTTCTGGCTATGCCAAAAAAA
ACTACCGGAAGTTTGATCACATCGACCAGGTCGTTTCTATACGGCAAGGCAAGCGTGCGAATGAAAACGGCGAGG
TCGAGGGGTGTGGTGACTGCATTTGACTTAACATCCGCAATTGGGGACGAGATTGATTTTGAGTGGCTGGGTGGT
GATTTAATGACGGCCCAATCAAACTACTATTCTCAAGGGCATTTGGATTACACAAGAATGCAAAGATTTCCCGTA
GGCGCTGACACTTGGGCCACTTACCACACTTATGAGATTGATTGGGACCCGGATAGGATAATATGGTATGTTGAT
GGTAAAATAGCTCGCACAGTCCTGAAGAAGGATACCTGGGACCCCATTAGTAAAGAATATAGGTACCCACAAACG
CCGATGAGACTAGAAATCGCTGTTTGGCCAGGCGGCTCGGAAACCAACGGACCCGGTACAATAAACTGGGCAGGT
GGCCTCATAGATTGGGAAAATTCACCCGATATTATCGAAAAGGGCCAATTTACGGCGCATGTTGAACAAATCACC
GTAACGCCGTACCAAAATAAATTCACAGAACAAGTGCAGTTTTGTATAAAGGCAAAGAAAAAGGCACCAACCTTT
TCCCAAAAAGATTTGTCAAGAGTAGTCGTTTCGTATAACAGGCAAGACAGACTGAATCATCATGATGAGGGGTCT
CTCAAATGGGACTGCTTTGTTACTCCAAAGATAAATGACTGGTTGAGTTCTTGGAAACGGTCAAAATAG

>YIR036C 1.51   Hot
ATGGGCAAGGTTATTTTGATTACAGGTGCCTCCCGTGGGATTGGCCTGCAATTGGTGAAAACTGTTATCGAAGAG
GACGATGAATGCATCGTCTACGGCGTAGCAAGAACGGAAGCTGGTCTGCAGTCTTTGCAAAGAGAATACGGTGCA
GACAAATTTGTCTATCGTGTCCTCGACATCACGGACAGGTCTCGAATGGAAGCGTTGGTGGAGGAAATCCGGCAA
AAGCATGGAAAACTGGACGGTATTGTCGCAAATGCGGGGATGCTAGAACCGGTGAAGTCCATCTCCCAGTCCAAC
TCCGAACACGACATCAAGCAGTGGGAACGGCTGTTCGATGTGAACTTTTTCAGCATTGTCTCTTTGGTGGCACTG
TGTTTACCCCTCTTGAAGAGCTCGCCATTTGTAGGCAACATTGTCTTCGTCAGCTCTGGAGCCAGTGTGAAACCA
TATAACGGATGGTCGGCGTACGGCTGCTCGAAAGCCGCATTAAACCACTTTGCCATGGACATTGCCAGTGAAGAG
CCCAGTGATAAAGTGCGTGCCGTGTGTATTGCACCGGGCGTCGTTGACACGCAGATGCAGAAAGATATTAGGGAA
ACATTGGGTCCTCAGGGCATGACACCCAAGGCTCTCGAGAGGTTTACTCAATTGTACAAGACTTCGTCACTGCTG
GACCCAAAGGTGCCTGCGGCGGTACTAGCGCAACTCGTCCTGAAAGGTATTCCCGACTCTTTGAACGGTCAATAT
CTCCGCTACAACGATGAGCGACTGGGGCCGGTGCAGGGCTAG

>YJR076C 2   Hot
ATGTCCGGAATAATTGACGCATCTTCTGCATTAAGAAAAAGAAAGCATTTGAAAAGAGGTATAACCTTCACTGTG
ATGATCGTGGGCCAGTCCGGATCTGGTAGATCGACTTTTATAAATACTTTGTGCGGTCAGCAAGTTGTAGACACT
TCGACGACAATCTTGTTACCCACAGATACGTCCACAGAAATAGACTTACAATTGAGAGAGGAGACGGTCGAATTA
GAAGATGATGAAGGTGTCAAGATTCAACTTAATATCATCGATACTCCGGGATTCGGTGATTCTCTCGACAATTCT
CCATCTTTCGAAATCATTTCCGACTACATTCGCCACCAATATGATGAAATCTTATTGGAAGAAAGTCGTGTGAGA
AGAAACCCAAGATTTAAGGACGGCAGAGTTCATTGTTGTCTTTACTTAATCAACCCAACTGGCCACGGTTTAAAA
GAGATTGATGTGGAATTCATCAGACAGTTGGGATCCTTAGTTAACATCATCCCTGTGATCAGCAAATCAGATTCC
TTGACAAGAGATGAACTGAAACTGAATAAAAAATTGATCATGGAGGATATCGACAGATGGAACTTGCCAATTTAT
AATTTCCCTTTCGATGAAGATGAAATATCAGACGAAGATTACGAAACCAATATGTACCTACGTACACTTCTACCT
TTTGCTATTATTGGATCCAATGAAGTTTACGAAATGGGCGGCGATGTTGGGACAATTCGTGGCAGAAAGTATCCA
TGGGGCATACTGGACGTGGAAGATTCATCGATCTCTGATTTTGTTATCTTAAGGAATGCGTTATTGATCTCTCAT
TTACATGACTTGAAAAACTACACACATGAGATATTATATGAAAGATATAGAACCGAGGCATTATCAGGTGAATCT
GTCGCGGCAGAATCCATACGTCCAAATCTGACAAAATTGAATGGTTCGTCGTCATCGTCCACCACAACAAGGAGA
AACACAAATCCCTTCAAGCAAAGTAATAATATTAATAACGATGTTCTCAACCCAGCATCCGATATGCACGGGCAA
AGCACTGGCGAAAATAACGAAACCTACATGACACGTGAGGAGCAAATACGGTTAGAAGAAGAGCGACTAAAGGCA
TTTGAGGAGAGAGTTCAGCAAGAACTGCTGTTGAAAAGACAAGAACTGTTGCAAAGAGAAAAGGAATTAAGAGAA
ATTGAAGCCAGGTTGGAAAAAGAGGCGAAAATCAAACAGGAAGAATGA

>YGR156W 2.64   Hot
ATGACAGATCCCAGAAGAAGAACAGGCCGTCATTTCTTGACACCGGAGAATTTATCCTCTACATTACAAATCACA
AACTTACCTCCAGAATGGAACCAAGATATAATTACTTCGGTCGTGGCCGGTTCTGGTCCAGTTATAGATATAAAA
GCTAAGAATGACCCGAGAACTGGTAAACTAACCGGTGTACTGTTCGATTATTTGACTAGTAAAGATTGTAAACGC
GCTTGGGAAATTTTAAATAGAATTGAAAACTTTCCCGTAAAGATAGAGCAAATAATCCCACCAAATTATAAGGAC
CATCTTAGAGAAACAGCAAATAAAAATTCTCAAAAGCAGGTATTACAACTTAATAGAGATTCGTACCCCTTCGAG
GCGGGTTTGGAGCTACCTTTCGAAATGGTGACAGAAGTCCCCATTCCTAGGCGACCACCGCCACCACAGGCTGCA
AATAACACAAACTCTGTATCAAATAACACAAACATTCAATTCCCCGACATACTAAGTAAAGCATCTAAACACTTG
CCAAGTTTCCAAGATGGCTCGATTATTGCACCAGACAAAATTTCACAAAATTTAAGTAAAATTCCGCCGTTGCAA
CTTATTGAAATTATATCAAATTTGAAAATATTATCAAACCAAGAAAACATCCAAAAATCGCAATTAGAATCTTTC
TTAGATACTAACAGTGATATCACAATATCAGTGACCCAAGCCCTACTAGAAATGGGATTTATAGACTACAGCGTG
GTGACTAAAGTGTTGAAATCCCAAGTTGGCGAGGCCCCATCTTTGCTTTCGAGTAATAACACAAGTAATTCGAAC
ACCCCCGTAAGCGTAATTAGAAATAACACTCCGTTGCATGTACCTTCTAATGAAGTCAGCAACAATCCTAACAAT
ATGCCACTGAACGTAGCTATGCCAATGCCTATGTCGACACCACCATTTATCCCTTTACCTCTGCAACAACAACCG
TTCGGTTTTGCGCCACCGGGCCCTTTCATGCCTCCAGCTCAAGGCCCCTCCATGGGACAGCCTGTGTTGGCAAAT
CAACTCGGCCAGGTCCAGCAACAAAATATAAGTTCTACAGAAGGACCCTCTAACGCGAATAAAGCGAATGACAGC
GGCACCATTAATATGGCGAAACTGCAATTACTACCTGAAAACCAACAAGATATGATCAAACAAGTTCTTACTTTG
ACACCTGCCCAGATCCAAAGTTTACCAAGTGACCAGCAACTTATGGTGGAAAACTTTAGAAAAGAATATATAATC
TAA

>YPL048W 1.75   Hot
ATGTCTCAAGGTACTTTATATGCTAACTTCAGAATTAGAACTTGGGTCCCAAGAGGTCTTGTCAAGGCTTTGAAG
CTGGACGTTAAGGTTGTTACGCCAGATGCCGCCGCTGAACAGTTCGCAAGGGACTTTCCCTTGAAGAAGGTTCCG
GCTTTCGTGGGTCCAAAGGGTTACAAGCTAACCGAGGCAATGGCGATTAACTATTATTTAGTAAAGCTTTCACAG
GACGACAAGATGAAGACTCAACTTTTAGGTGCCGACGATGACTTAAATGCCCAAGCGCAAATCATCAGATGGCAA
TCTTTGGCCAACAGTGATCTGTGCATCCAGATTGCCAACACCATCGTTCCCTTGAAGGGCGGCGCCCCCTACAAC
AAGAAGAGCGTTGATTCTGCGATGGACGCTGTTGACAAGATCGTCGACATCTTCGAGAACAGATTGAAAAACTAC
ACCTATTTGGCTACGGAAAACATCTCCTTGGCCGACTTGGTGGCTGCCTCGATTTTCACCAGATACTTTGAAAGC
CTGTTCGGTACTGAATGGAGAGCTCAACATCCAGCCATCGTGAGATGGTTCAACACCGTTAGAGCCTCACCCTTC
TTGAAGGATGAATACAAGGATTTCAAGTTCGCTGACAAGCCATTGAGTCCCCCTCAAAAGAAGAAGGAAAAGAAG
GCCCCAGCCGCCGCTCCCGCCGCTTCGAAGAAGAAGGAGGAAGCCAAGCCAGCTGCTACCGAAACTGAAACTTCT
TCCAAGAAACCAAAGCACCCATTAGAATTATTGGGCAAGTCAACTTTCGTTTTGGATGACTGGAAGAGAAAGTAC
TCCAATGAAGACACCAGACCAGTTGCTTTACCTTGGTTTTGGGAGCACTACAACCCAGAAGAGTATTCCCTGTGG
AAAGTCACCTACAAGTACAACGATGAATTGACTTTGACTTTCATGTCCAACAACTTGGTCGGTGGTTTCTTCAAC
AGATTGTCCGCCTCTACCAAGTACATGTTCGGTTGTCTGGTAGTCTACGGTGAAAACAACAACAACGGTATTGTT

GGTGCAGTTATGGTTAGGGGCCAAGATTACGTTCCAGCTTTCGATGTCGCACCAGATTGGGAATCTTACGACTAT
GCAAAGTTGGATCCAACTAACGACGACGACAAAGAATTCATCAATAACATGTGGGCCTGGGATAAGCCAGTTTCC
GTCAATGGCGAACCAAAGGAAATTGTTGACGGTAAGGTCTTAAAATAA

>YHR018C 4.21 Hot
ATGTCAGACGGCACTCAAAAACTATGGGGTGGGAGATTCACTGGTGAAACCGATCCTTTGATGCACCTTTACAAT
GCGTCTCTTCCGTATGATTATAAGATGTATAAGGCAGATTTAGAAGGAACTAAAGTATACACAGCGGGCTTGCAG
AAGTTGGGTCTTCTAACGGAGACAGAATTGGCAAAGATCCATGAAGGTTTGGCTGAAATCAAAAAAGAATGGGAC
GCTGACAAATTTGTCCGTCATCCAAACGACGAGGATATCCATACTGCGAATGAAAGACGTCTTGGTGAACTAATT
GGCCGCGATATTGCTGGTAAAGTCCACACCGGTAGATCCCGTAATGATCAAGTTGTTACCGATTTGAGAATATAC
TGTCGTGACATTGTCAATGACACCCTCTTTCCAGCTTTAAAGGGCTTGGTTGAAGTTCTAATTAAGAGGGCCGAA
GGTGAGATAGATGTCTTAATGCCAGGCTACACACATTTACAAAGGGCACAACCTATTAGATGGTCTCATTGGTTG
AGCTCTTATGCAACATACTTCACCGAAGATTACAAGAGACTGGGTCAAATACTACACAGATTGAATCAATCACCA
CTGGGTGCAGGCGCTCTTGCTGGTCATCCTTACGGCATTGATAGAGAATTTTTGGCTGAAGGTTTGGGTTTCAAT
AGTGTAATTGGTAACTCCTTGGTTGCTGTTTCTGATAGAGATTTCATCGTGGAGTTGATGTTTTGGGGAACTTTG
TTCATGAACCATATTTCTCGTTTTGCTGAAGATTTGATTATATATTGTACAGCAGAATTTGGTTTCATACAGTTG
AGCGACGCCTATTCAACAGGTTCTTCTTTAATGCCTCAGAAGAAGAATGCAGACTCGTTAGAGTTGTTAAGAGGT
AAATCCGGTAGAGTATTTGGTGATCTGACAGGATTCTTGATGAGTTTGAAGGGTATCCCATCTACTTATGATAAA
GACATGCAAGAAGACAAAGAGCCACTATTCGATTGCTTAACAACTGTAGAGCACTCCATGCTGATTGCCACAGGT
GTTATTTCTACCTTAACTGTAAATAAGGAAAAGATGGAAGCTGCTCTCACGATGGATATGCTAGCTACCGACTTG
GCAGATTACTTGGTCAGAAAGGGTGTTCCATTCAGAGAGACTCATCACATATCTGGTGAGTGTGTCGCTACTGCT
GAAAGACTTGGTCTAAGCGGTATTGATAAATTAACCTTGGAGCAGTATCAAAAGATCGATTCGAGATTCGGACAA
GATCTTTTTGAAACTTTTAACTTTGAACAAAGCGTTGAAAGACGAGATGCTACTGGTGGAACCGCTAAATCTGCT
GTATTGAAGCAATTGGATAATTTGAAATCCCAATTAAATTAG

>YKL102C 1.59 Hot
ATGTGTGCTTTATTCGCTGAGGTTTCATCGACAGCTGCAGCCAGTTGTTCATTCGGTGTTCTCTTGTGCAGCGGC
TCAAATTGTGGCAGGCTTGTAGGCTCCGAACGCCGTAAGGTACTTTATGTTTTTAATGTCAACGTTTGCAAGAAA
ATGTCATCCTATTACTTTCTGAGGCATGATAACATTGTAATCCCTTATCTCTTGCGCTTGTTGGTATCAGATAAA
GAAGCATCAAACAAGAACCCACTCCTTCCTTTTCTCATGGATAAAGAGAGGTCGCACCATTTCGTGAGGAATATG
AATTAG

>YLR237W 1.51 Hot
ATGAGTTTCGGTAGTAAAGTCTCCAGGGCTTTGAGATTTTTGGAAATTCCTGTCAAAGACAGGGCATCTGTGAGT
TTCTTGAAGAACCCGGATTTGCAACCTATTAAGTCGGCTAACCAAACATGGGGGTTCTGGTCTAATTTTGCATAT
TGGGGTGTTATGTCCTTTTCAGTGGGTACATGGATGAGTGCCTCTTCTGCACTAGGTGTTGGATTAAGTTACCCA
GAAACCATTGGTACATTTATCGTCGGTGATGTGTTGACCATTATTTTCACATTGGCAAACTCGTGTCCTGGTTAC
GACTGGAAGGTCGGTTTCACTTTGGCCCAAAGATTCGTCTTTGGTATTTACGGTTCTGCCTTCGGTATCATCATC
AGAATTTTGATGAGTATTGTCAACTACGGGTCCAACGCTTGGGTGGGAGGTCTTTGTATCAATATGATCTTGGAT
TCATGGTCTCACCATTATTTGCACCTGCCAAACACCTTATCTTCGAAAGTTGCCATGACCACTAAAGAATTAATT
GGTTTTATCATCTTCCACGTCCTTACTGCATTCTGTTACTTAATGAAACCCTACCACATGAACTACATTTTAATT
TGGTCGTGTGTTGCTACATTTTTCTCTATGTTGGGTATGGTGATCTATTTAGCTAAACAGGCTCACGGTGTTGGA
GAATTGTTTACCTCCACTAAATCCACTGCCACTGGCTCCACCAAAGCTTGGGCTTGGGTTTATATGATCTCGTAC
TGGTTCGGTTCCGTTTCTCCAGGTTCCACCAACCAAAGTGATTACTCGAGATTTGGTTCCTCCAATTGGGCTATC
TGGGCCGGTACCATCTGTGCATTGTTAATTCCAACCACTTTAATCCCAGTTTTTGGTGTTATTGGTGCCTCCACC
TGTGACAAACTATACGGTGAACAATATTGGATGCCTATGGATATCTTCAACCATTGGTTGACAACTAACTATTCT
GCAGGTGCTCGTGCCGGTGCGTTCTTCTGTGGTCTTTCATTTGTCCTATCTCAAATGTCCTACACCATCTCCAAC
TGTGGGTTTGCCAGTGGTATGGATTTGGCCGGTTTATTACCCAAGTATGTCGACATCAAGAGGGGTGCCCTTTTT
GCCGCATGTGTCTCCTGGGCTTGTTTGCCATGGAACTTCTACAACTCTTCTTCTACTTTCTTGACTGTCATGAGT
TCTTTCGGTGTTGTCATGACTCCTATCATTTCTGTCATGATTTGCGATAACTTCTTGATCAGAAAAAGACAATAC
TCCATCACTAATGCCTTTATTCTCAAGGGTGAATACTATTTCACTAAGGGTGTTAACTGGAGAGCTATTGTTGCC
TGGGTTTGTGGTATGACCCCCGGTCTACCTGGTATTGCTTGGGAAGTCAATAATGACTATTTCCACAACACTGGT
ATTGTCAATTTCTTTTACGGTGACTCCTTCTTCTCGTTTTTGATCTCCTTTTTCGTCTATTGGGGACTATGCCTC
CTCTTCCCATTCAAAATTACTGTCAAACATGATGACAAAGATTATTATGGTGCCTTTACTGACGAAGAAGCAAGA
AAGAAGGGCATGGTTCCATACAGTGAAATTTCTGAAGAAGAAATCCGCGCTTACACATTAGGCGAAGGTTACACT
ACCGGCCACGAGTACAGACCTGAAGGCTCCGACGATGAAATACCTGAGTTGGTCAAAACTAGCTCTGAAAACACC
AATGAGTTTGAAATAGTTCATCATAAGAATAATGAAAAGCAATCCTCCACCGCCAGTGAAAAAGCTGCTTAG

>YER019C-A   1.89 Hot
ATGGCAGCTTCAGTTCCACCAGGAGGTCAGCGTATCTTGCAGAAGAGAAGACAGGCACAATCCATTAAGGAAAAG
CAAGCAAAACAAACGCCCACTTCCACCAGACAGGCTGGTTACGGTGGGTCTTCAAGCTCAATTTTGAAGTTATAT
ACGGACGAAGCCAATGGATTCAGAGTCGACTCCCTTGTCGTATTGTTCCTATCTGTCGGTTTCATCTTCTCCGTG
ATTGCTTTGCATCTATTGACGAAATTTACACACATTATATAA

>YLR236C 1.54 Hot
ATGCATACTATTTGCCTTAGAAGCCCGATCGATGAATCCTCTCCTCTTCCATATAAATCAATAAGGCAACCTCTA
GAAAACGCCCATTCCTGCCAAGCGCTTTGCAGCCTTATGGCCGTACTCTGTGCATCCGCTGCTCATCGGCTCTCT

GAAACCTTCCCTATGCGTTTGGTAGTCGCGCGTGAATATGCTAATTGGGGCGCCTTCCAACATGCGTTTACGCGG
CGCGCTGGAGCTTCTGTGGCGGCGACTTCTGCATGGTTTGATGCAGTTGCGGCTGGTACTGAAAATGCACATATG
CAGTCAGCTGAATCATGCAATTAA

>YLR235C 1.53 Hot
ATGCTAATTGGGGCGCCTTCCAACATGCGTTTACGCGGCGCGCTGGAGCTTCTGTGGCGGCGACTTCTGCATGGT
TTGATGCAGTTGCGGCTGGTACTGAAAATGCACATATGCAGTCAGCTGAATCATGCAATTAAGCGGAGAGCTTTT
TTGAAGACAAAGGGCGGCAAAAACGCCTTACATGGATGCCTTGACACGGTCATAAACTTGCAAGAGAGTATTCTT
GCAGGCATTCGTCTTGTGCCAGTACTTCCTATACTTCTCGACTATGTCCTTTACAACATCAGTCTTGGAGGCATG
ACCTTCGCAGATTTTCTTGAGGTCTTGCTCCATTTCTCTGCGCTGGAAGGGCTTTGCAAAGGAGTCTTCGAGGCC
GATGGCCTCGAAGCCGTGCACTAG

>YAL061W 2.29 Hot
ATGAGAGCCTTAGCGTATTTCGGTAAAGGTAACATCAGATTCACCAACCATTTAAAGGAGCCACATATTGTGGCG
CCCGATGAGCTTGTGATTGATATCGAATGGTGTGGTATTTGCGGTACGGACCTGCATGAGTACACAGATGGTCCT
ATCTTTTTCCCAGAAGATGGACACACACATGAGATTAGTCATAACCCATTGCCACAGGCGATGGGCCACGAAATG
GCTGGTACCGTTTTGGAGGTGGGCCCTGGTGTGAAAAACTTGAAAGTGGGAGACAAGGTAGTTGTCGAGCCCACA
GGTACATGCAGAGACCGGTATCGTTGGCCCCTGTCGCCAAACGTTGACAAGGAATGGTGCGCTGCTTGCAAAAAG
GGCTACTATAACATTTGTTCATATTTGGGGCTTTGTGGTGCGGGTGTGCAGAGCGGTGGATTTGCAGAACGTGTT
GTGATGAACGAATCTCACTGCTACAAAGTACCGGACTTCGTGCCCTTAGACGTTGCAGCTTTGATTCAACCGTTG
GCTGTGTGCTGGCATGCAATTAGAGTCTGCGAGTTCAAAGCAGGCTCTACGGCTTTGATCATTGGTGCTGGCCCC
ATCGGACTGGGCACGATACTGGCGTTGAACGCTGCAGGTTGCAAGGACATCGTCGTTTCAGAGCCTGCCAAGGTA
AGAAGAGAACTGGCTGAAAAAATGGGTGCCAGGGTTTACGACCCAACTGCGCACGCTGCCAAGGAGAGCATTGAT
TATCTGAGGTCGATTGCTGATGGTGGAGACGGCTTCGATTACACATTTGATTGCTCCGGGTTGGAAGTCACATTG
AATGCTGCTATTCAGTGTCTCACTTTCAGAGGCACCGCAGTGAACTTGGCCATGTGGGGCCATCACAAGATACAG
TTTTCTCCGATGGACATCACATTGCATGAAAGAAAGTACACAGGGTCCATGTGCTACACACACCACGATTTTGAG
GCAGTAATAGAAGCTTTGGAAGAAGGCAGGATTGACATTGATAGAGCAAGACATATGATAACGGGCAGAGTCAAC
ATTGAGGACGGCCTTGATGGCGCCATCATGAAGCTGATAAACGAGAAGGAGTCTACAATCAAGATTATTCTGACT
CCAAACAATCACGGAGAGTTGAACAGGGAAGCCGATAATGAGAAGAAAGAAATTTCCGAGCTGAGCAGTCGGAAA
GATCAAGAAAGACTACGAGAATCAATAAACGAGGCTAAACTGCGTCACACATGA

>YOR138C 2.11 Hot
ATGATGGATAATCAGGCTGTAAAGTCACTGCTAGAAATGGGTATTCCACACGAAGTAGCTGTGGACGCCCTCCAA
AGAACTGGCGGTAATTTGGAGGCAGCAGTTAACTTCATCTTTTCGAACGAGCTGCCGGAGCAAGCGGAAATGGGT
GAGGAGAATGATGGCAGTCAGCCTAGGATTTCTGAGAACAAAATAGTGGCTGGAACCAAGCCCTGCGATGTTCCG
AACAACGGGGACCAAGACATTGATATGCCGGATGTGAGTGGTGTCGATGTTGATTATGATGACGATGAAGACATC
ACGGATGAACGCAGCGGTTCTAATAGCACCAGCGGATGCAGGGTTACTGCTCAGAACTACGATCGTTATTCTATA
TCGGAGACATCCATTCCTCCCCCCTCTTATAGTATAGTACAACACAACGAGTTCAAGTCGAACGTGGGCGATCCT
ACGGTTGTGTTGCCGCTACCCTTGAATAGTCTAATTGAGAGCTATTTTGGGCTGTTTGCGTTGCTCACGGCCGTG
TATTTCCCACACGTGTTCTTGAAACCGGACTTTAAGGATTTGAACTATAGGGCAGACTGGTTCAAGGGCTCATCG
TTTACGGAGCCCAAATACAGACTTGCATATTGCGAGGCAGAAGATGGTTCCACTACGTCGGAGATCGTGCTGGCC
AGCGGACCTAATGAAGGGTTGCAACCGCATCTGCTCTGGCAGTTGCAAAAACTCATCTCCGTGGTTAACACGCGA
AAGTGTGAAAGAGCCTTTGTTTCGGCCAAGGTGTTTACGTCGTCCTTGGAACCGCAATTGAGGTCCAAGCTGGCC
GATTCCGAGCATCTTTATGAGGTGCTCCCAGCGTTTATCAAATCATTGGCAGTGGATTTGGAAATGTGTCCTGGG
ATCAGAGACCGGGAAACAAGAAGTCTCTTTATATCTTCTGCTTTGCATACTCCGAACAAGAATGAACCTCCAATG
GAGACGTTTTTGTCCTTGTTTCATTTTCTTCCTGAAGAGTACGATTCTAATCTATATAAAATGTTCAACGTTTTA
TTGTACCCCGAAGAAGAGGAAGAAGAAGAAGACGTGATTCGTGGTGGCGAGCAAGAAGAGGCGAGATATGTGGAA
CCGGAGAATACTTTGAAGGAGGTGGCACCCGTATTAACAATCCTGTTCAATGAACTAGAAACAAATACAGAATCA
GTGTCTCTACCCAATGGCGTGGATATACCGCTTGAGTTTTACCCACAGCTTTACACCAAACAGTGCAAAGACCAA
CTAATAAGACATATTATTTCTAAACGAAAGCAAGCGCGGACCAGATCCAGATGCTTGCTTCAAGAGATCAACGAG
TTAAAGAGTTACCAAGGCAAAAACATATCCACTATTCTAGAGTCGACGCTGGCGTATCTACAAACAATTCCTGAC
GATGCTAATAATGAAGCAGCAAAGCAGATCGCATCGTTGAAAGATACATTAAATTCTGCGAGAGCCGCTAAAATG
GAGGAATATAAGGACTTGGCGTCCAAACTGCATGGAGAATGGAACCTTTCACATCCAGAGACACACATAATCAAC
ACTGCAAAGCAATTGGGACTGATCGAAAATCCCTACATCCTCACAATGGCAGCACTATCGCCATATTCATACTTT
ATCAGAAGCAGAAACGGTGCATGGTCATGGATACAAAGTAATACGTTGGGAACAGAATTCAAGGTGAAAAAATGT
TCGTCGCCTTCTGTCGTTCAAGAAGCCATCAAGCATGGCACGAAGTACGCAAGTGAAACTCCGCTGATGTTCATA
TACTGTGAAGAGGGTAAAATTCCCACGGAAGAAGTAGTCGCGGAAGCGTTGAAAAGCAATAGCGGGTGCTTAAAA
TTTGCTGAGGATGACCAAAATTCATTAAAAACATTGCGTTCGCAATTTTTCGATGGTATGGAGATCCTGAACAA
GCCACCAACAATATTAACAATGGTAACGATAATGACAATGACGATGACATCGATTCTGATAATTAG

>YLR100W 1.53 Hot
ATGAACAGGAAAGTAGCTATCGTAACGGGTACTAATAGTAATCTTGGTCTGAACATTGTGTTCCGTCTGATTGAA
ACTGAGGACACCAATGTCAGATTGACCATTGTGGTGACTTCTAGAACGCTTCCTCGAGTGCAGGAGGTGATTAAC
CAGATTAAAGATTTTTACAACAAATCAGGCCGTGTAGAGGATTTGGAAATAGACTTTGATTATCTGTTGGTGGAC
TTCACCAACATGGTGAGTGTCTTGAACGCATATTACGACATCAACAAAAAGTACAGGGCGATAAACTACCTTTTC
GTGAATGCTGCGCAAGGTATCTTTGACGGTATAGATTGGATCGGAGCGGTCAAGGAGGTTTTCACCAATCCATTG
GAGGCAGTGACAAATCCGACATACAAGATACAACTGGTGGGCGTCAAGTCTAAAGATGACATGGGGCTTATTTTC

CAGGCCAATGTGTTTGGTCCGTACTACTTTATCAGTAAAATTCTGCCTCAATTGACCAGGGGAAAGGCTTATATT
GTTTGGATTTCGAGTATTATGTCCGATCCTAAGTATCTTTCGTTGAACGATATTGAACTACTAAAGACAAATGCC
TCTTATGAGGGCTCCAAGCGTTTAGTTGATTTACTGCATTTGGCCACCTACAAAGACTTGAAAAAGCTGGGCATA
AATCAGTATGTAGTTCAACCGGCATATTTACAAGCCATTCCTTCTCCGAATATTTGAATTTTTTCACCTATTTC
GGCATGCTATGCTTGTTCTATTTGGCCAGGCTGTTGGGGTCTCCATGGCACAATATTGATGGTTATAAAGCTGCC
AATGCCCCAGTATACGTAACTAGATTGGCCAATCCAAACTTTGAGAAACAAGACGTAAAATACGGTTCTGCTACC
TCTAGGGATGGTATGCCATATATCAAGACGCAGGAAATAGACCCTACTGGAATGTCTGATGTCTTCGCTTATATA
CAGAAGAAGAAACTGGAATGGGACGAGAAACTGAAAGATCAAATTGTTGAAACTAGAACCCCCATTTAA

>YJL123C 1.6Hot
ATGTCTGAAAATAAGAACTCTGAGGCTGAAGACGTATTTGAATTTTTAGATTCTTTGCCAGAAGCAAAGAATGGC
GGGAAAATGGTTAATACTGACGTAAAGGGTAGCCAAGAAGGCGTTAAGGGAGGTTCTAATTCTGTGGCGGGCAAG
ACCGGGAATGATGGAAAGAAAGGTGACGATGATATCTTTGAATTCTTAGAAGAGTTGGAGAAGAGTAACTTAAGC
TTAACTGACAAGAAAGGGGTTGAAAAGAAGGCTCCCAGTGAATCAGTTAATAATAAAGCACAAGACGAAAAAGTA
GAAGAATCAAAAGAAAACAAAAATTCCGAGCAGGACGCTCACGGAAAGGAAAAGGAACCACAGCAGCAGGAGAAA
GAAGAAGAAGAGGAGGAAGAAGAAGAAGAAGAAGAGGAGGAGGAGGAGACCCCATTGCATGACCCAATTGCTTCT
ATTTCAAACTGGTGGTCCTCCTCTGGATCCGCAAAGGTATCGAGTATTTGGAATAAAACTGCAGAACAAGCATCC
CAGATCAAAAACCGACTTGCCCAAGAACAGCTTGATTTGACTTCAAAGATAAACACGAGCACGATTACTGAAATA
GCCAGAAACTTACAGAAAATCGTTGTTGGTGAGACTGAAGAGGTGCTGCGAATTCATCTAGTCCATGATTTGGTT
AACTATCCCTCATTACAATACAATATTGAAAGCAAGTTTGATCAAGTTCTAAGCTCACAAGTTGAGGGAGGAATC
AGAATATTTGTAGATGAATGGGGCCATCCTAACAATAATGGAATTACACCTGTGGAAAAAAAGCCTTCAGTTGCA
GACGGTGAGTTAGGCAACTCTAAAAAAAAATTACAGTTTAATCTATTCGACGGCAAGGTAACAGACGGTGAGAAA
CTGGCATTTGCAAACCTGGAAAATGCAGTCAAACTCTTCAATACTGCACATGAGGAATACCAGAAACAACAGAAA
GAGGCTGATGCCACTCCAGATGACGATAGGAGTTCTATTAGCAGTAACAGCAATAAAATCAGTGATTTATTCATA
TCCATCTTACCAATTGCCATTCCGCAAAAACAAAAAGATGCCGACGGTGACTTTCAAGTGACCGACTCCAATACG
CCAGGCAATTTCAATTTCACCCTTGTATTGAAAGACATAACTAACGATATTACCACCATAACAAGATCACAAGGA
TTCCCTGTCAAATGGGTCAATTGGCTAGAAGGATCCGTGGAGAAAACAGGATCAACAGCAAGTGAGGAGAGGAAT
AAGTCATACGATCAAAAGAAACAAAAAGAAAGTGAAGACGAAGACGAAGACGATGAGATCATTGATCCTAGTGAA
TGGGTAAAGGAATGGATCGAAGATGGATTAAGCTTGTCATTTGGGGTCATGGCACAGAATTATGTCATTGACCGT
ATGGGTCTTTGA

>YKL192C 1.56 Hot
ATGTTTAGATCCGTTTGCCGCATTTCTTCCCGCGTGGCACCTTCTGCGTACCGCACTATAATGGGCCGTTCCGTT
ATGTCCAACACCATACTCGCACAAAGATTTTATTCTGCAAACTTGAGCAAAGATCAGGTTTCTCAAAGGGTCATT
GATGTTATCAAGGCGTTTGATAAGAACTCTCCCAACATTGCCAACAAGCAAATCTCCAGCGATACCCAATTTCAC
AAGGATTTGGGGTTGGACTCCTTGGACACTGTCGAGCTGCTCGTAGCTATTGAAGAAGAATTTGATATTGAAATC
CCTGACAAAGTGGCTGATGAGTTGAGAAGTGTTGGTGAAACGGTCGATTATATCGCTTCCAATCCCGACGCAAAC
TAA

>YGR192C 1.72 Hot
ATGGTTAGAGTTGCTATTAACGGTTTCGGTAGAATCGGTAGATTGGTCATGAGAATTGCTTTGTCTAGACCAAAC
GTCGAAGTTGTTGCTTTGAACGACCCATTCATCACCAACGACTACGCTGCTTACATGTTCAAGTACGACTCCACT
CACGGTAGATACGCTGGTGAAGTTTCCCACGATGACAAGCACATCATTGTCGATGGTAAGAAGATTGCTACTTAC
CAAGAAAGAGACCCAGCTAACTTGCCATGGGGTTCTTCCAACGTTGACATCGCCATTGACTCCACTGGTGTTTTC
AAGGAATTAGACACTGCTCAAAAGCACATTGACGCTGGTGCCAAGAAGGTTGTTATCACTGCTCCATCTTCCACC
GCCCCAATGTTCGTCATGGGTGTTAACGAAGAAAAATACACTTCTGACTTGAAGATTGTTTCCAACGCTTCTTGT
ACCACCAACTGTTTGGCTCCATTGGCCAAGGTTATCAACGATGCTTTCGGTATTGAAGAAGGTTTGATGACCACT
GTCCACTCTTTGACTGCTACTCAAAAGACTGTTGACGGTCCATCCCACAAGGACTGGAGAGGTGGTAGAACCGCT
TCCGGTAACATCATCCCATCCTCCACCGGTGCTGCTAAGGCTGTCGGTAAGGTCTTGCCAGAATTGCAAGGTAAG
TTGACCGGTATGGCTTTCAGAGTCCCAACCGTCGATGTCTCCGTTGTTGACTTGACTGTCAAGTTGAACAAGGAA
ACCACCTACGATGAAATCAAGAAGGTTGTTAAGGCTGCCGCTGAAGGTAAGTTGAAGGGTGTTTTGGGTTACACC
GAAGACGCTGTTGTCTCCTCTGACTTCTTGGGTGACTCTCACTCTTCCATCTTCGATGCTTCCGCTGGTATCCAA
TTGTCTCCAAAGTTCGTCAAGTTGGTCTCCTGGTACGACAACGAATACGGTTACTCTACCAGAGTTGTCGACTTG
GTTGAACACGTTGCCAAGGCTTAA

>YFL010C 2.18 Hot
ATGGCTCAAAGTAAAAGTAATCCTCCTCAAGTGCCTTCTGGCTGGAAGGCTGTTTTTGATGATGAATACCAGACT
TGGTATTATGTAGATTTATCTACGAACAGCTCTCAGTGGGAACCACCAAGGGGAACAACATGGCCAAGACCCAAA
GGTCCTCCACCAGGTGTTAACAATGAGAAGAGTTCTCGTCAACAGGCAGACCAGGCTCCTCCACCTTATTCTTCT
CAATCAACACCGCAAGTGCAAGCAGGGGCCCAAGCACAGCAACCTCGTTACTATCAGCCTCAACAGCCTCAGTAC
CCTCAATACCCTCAACAGCAGCGTTACTATCCTCAACAAGCCCCTATGCCAGCAGCAGCACCACAGCAAGCATAC
TATGGAACTGCGCCAAGCACCAGTAAAGGTAGTGGACACGGCGGTGCCATGATGGGTGGTTTACTTGGTGTTGGT
GCGGGTTTGTTAGGTGGAGCTATGCTAGAACACGCATTTGACGACCACAACTATGACGGACCAGACACTGTCGTT
GTCGAGAACAATTACTACGGTGATGATGCTGGTGGGTCCGACGGCGGATTTGATGATGCTGGTGGGTTCGACGGC
GGATTTGATGATGGATTTGACGGTAGTGACTTTTAA

>YPR150W 1.52 Hot

ATGAGGAATCGTGCTATAAAATCATTTTCCATATGTATATGCCAAATATTTATTATTTATTTCATCTTTTTTTTG
CTTTTGTGTGTCAACCGCATTTGCAGTGCCTTGTCAAACGGCTTCAACTTTCTCATCATATATGGGGGCACGTTT
TTCCATTCAGGAAAATATTCCATACCCATACTGGACGTAGACCACCAATTGAATGGCAACAGCTCATTGGGTACG
GTGGGATTGTACTTCCATGTACCGTTCACGCCACCTGACATTATACCACCAGTGCCACGTTCACCAAACAATATG
TTCTTGTGCTTTTTCTCATCGATCGTCTTCGCCTCTGCGTTTGTTTTCTCGTTCATTGTTAATAACGTTTGCAAG
GACTTTCTCTTCTTATCTCTGAATAGCGGGTTTTCCTTCACTGGGTACATTACTGGCCTGTATCCGCTATACAGA
ATATCGCGGGTGAGCTCCCTAGCTTCCAAATACTGTGTAGTAGGTACTTTGGGCAGCGATCTAAAGTCATAG

>YDR124W 2.41   Hot
ATGCGTATAGGTATGGAAGAGCTTAGAAGGGCGCTGGCGCTGCTAAATGGTCAAGGATATGAGTTTATGGTGTTC
ATAAAGGAAAAAGACCATTTAATAAGTGAGTCAATTCCAAAAAACTTGTTCTCAGAGAGTTTTATATCATCTTCA
ATTGAAAAAAACCCTCCACTCACGTTCGATCCATTGGTAAATAGGAAAAGAATAACTGTTTATGAAGAAAATATC
CAACCAGCGATCATGCTACCCTTAGATCAACCCCTACTAATACAGAATTATCTGTATACGGCTTTTAAATTATTA
AGACAGGTCCCATGCAAGGCGATTGCTAAGTTGTGGATAAAAATTATTGAGCCAAGGAAGAAAACCAGGTTTCCA
TACATTAAAGGCAATGCTGGAAGGCCGGAATGGTGGCCAAAAGATGTAGAACATAAAGAGCCGGACCACCTCCAG
AAAGCGGATCGACTGAAATTGATGTGCACGATAATTATGAATGTTCTACCGCAATCTCCCTTCAGCCTTGAGATT
TTGGACGAGCTTACAGGAGTGACCGCCGCAATGACCATTTTTAAGAAAGAAACTGTGAAGAAAGTAATCATGAAA
AATGTTTTCGAAATTGCGAAATGCTTGTGCAACAAAGATTTCAAGCAGAAGACCATATCACTTGATGATTTAAAT
GACCTAGCCCAAAAACAGAAAAAATCATACCATAGTCGTCGATTTGATGTAAATAAAATGGTTAAGGTAGAAAAA
GATTTGTTACAACAGTCAAGTGGAAGCGATTCTTCACCCTCGATGATCCTTCCTTCAAAAGAAAGTGACACAGAG
TATTTCCCCAGGGAATATCCGAAAGACCCTGTATCGGAGGATTATTCGAATAGCATATCTCGATTAGAAAATTTA
GTGGAATTTGATCCGTTATTCCTCACTAAATTGGATGAATTAACTAGTAGCGATGATTGTAAAGATTTTATTTGA

>YJL117W 1.92   Hot
ATGGCGGTTCAACAAAGAAAGAAGAAAGAGGGTAGAAAGTCCGATAAAAATGCCCCATCGGTCCCACAAGTAGAT
GCATCATTAGACAAACCACTTGATATTGATGCTCCTCCTACTATTTACAGTGTTAATTTGAAACCAGAATATGGT
ACAGCTGCATTGAATTTGTCCGCGGATTTCATCAGACAAGAACAGGCATTGGCTAATAAGTATTTGTTCTTCCAT
CCCGTCATCCTTGTTGTTCTAACCATTGGGCTGCTTATTTACTTGACGCCAAGAATTGTCTTTCCGATAAGGAAT
ACAGGATCCGTTGCTGGATGGTTCTACCAGCTGGCTCGTATTAATAAGAAGGTCGTCCTCAGCGGTTTAGTGTTC
ACCGCAATTGGTGCATCTTTCTTGTTCACCCTCCTCTCACGTGTATCTGACTCGTATTTCAAGTCGAAGATCAAC
CAACTTGTTGGTTCTAAAGGTGAGAAAGTTTTCGGCATCAATTTGAATGATTTGGTGGCAAGACATGAAACAAAG
GATCCAGTGGTGAACAATACGCATATCATCGTTTATAGAGAAACGCCCATTGCATTGATTTCGTTAGCCCCTAAC
ATGACATTAAGTACTGATGAAAATTTAGTCATGAGTGTTACTACTGTGGGTTGTCGTCGTGTATATGTAAAAAGT
GGCATTATCGAAGATTTGATTGATTGGGCTATGCTACACTCCAAAAATATTAGAAGCAGCGGCAAATACGGGGAG
ACCATGAAACTCTTGATTGATGTCTACTCATTCGATAGCACGCTTAAGGAAATTTTGAAGAAAAAAGGGTTCACT
TATATTCAAAGCATTAGAGTCTCTGAAAATCGACTACTAGGCGGTTTGTTCGGTGTGAAGAAGGAATTATGGGGT
TTGCAATTCCATTTTAAAGCCGAACACAAGGACTAG

>YJR024C 2.85   Hot
ATGTCTTCACAAGATGTTCTGATACATTCAGATGATCCGTGCCATCCAGCAAATCTCATTTGCACGCTATGCAAG
CAGTTTTTTCATAATAATTGGTGCACCGGAACTGGAGGTGGGATTTCAATAAAAGATCCTAACACGAATTATTAT
TATCTCGCCCCATCTGGGGTGCAGAAGGAGAAGATGATACCTGAGGATTTGTTTGTCATGGACGCTCAGACATTA
GAGTATTTGCGCTCTCCCAAGCTTTACAAGCCAAGCGCATGTACTCCATTGTTCTTAGCGTGCTACCAAAAGAAA
AATGCAGGTGCTATAATACATACACACTCCCAAAATGCTGTGATATGCTCCTTGCTATTTGGCGATGAGTTCAGG
ATTGCTAACATCGAACAAATCAAAGCAATTCCAAGCGGAAAGGTGGACCCTGTGACCAAGAAACCCATGGCGCTA
TCGTTTTTTGATACGCTGAAGATTCCCATCATTGAAAACATGGCGCATGAGGACGAGTTGATCGATGACTTGCAC
AAGACTTTTAAAGATTATCCAGACACATGCGCCGTAATTGTGAGGAGACATGGCATTTTTGTTTGGGGTCCGACA
ATCGATAAGGCCAAAATTTTCAACGAAGCCATCGATTATTTGATGGAGCTGGCCATAAAGATGTACCAAATGGGG
ATTCCTCCCGACTGTGGCATTGGCGAAGAGAAGAAGCATCTGAAGATGGCCAGTCCGTGA

>YPL015C 1.64   Hot
ATGTCTGTTTCTACCGCCTCTACAGAGATGTCGGTTAGGAAGATCGCTGCTCATATGAAAAGCAACCCCAACGCT
AAAGTAATCTTTATGGTGGGTGCCGGGATATCCACTTCTTGTGGGATACCAGACTTCCGATCTCCGGGAACTGGC
CTGTACCATAACTTGGCCAGGTTGAAGTTGCCGTACCCTGAAGCAGTGTTCGATGTCGATTTTTTCCAATCGGAT
CCTTTGCCATTTTACACTTTGGCTAAAGAGTTGTATCCCGGAAATTTCAGACCTTCTAAGTTTCACTATCTTTTG
AAATTGTTTCAAGACAAAGACGTTTTGAAGAGAGTATATACCCAGAATATAGACACTTTAGAAAGACAGGCCGGT
GTTAAGGATGATCTGATCATCGAGGCCCATGGCAGTTTTGCTCACTGTCATTGTATCGGATGTGGTAAAGTTTAT
CCCCCACAGGTTTTCAAATCCAAGCTGGCAGAGCATCCTATAAAGGATTTTGTCAAGTGTGATGTGTGCGGTGAA
CTGGTAAAGCCGGCAATAGTTTTTTTTGGCGAAGATCTACCGGATTCCTTTTCGGAAACTTGGTTAAACGATTCA
GAGTGGCTTCGTGAAAAAATCACGACATCTGGAAAACATCCTCAGCAGCCTCTTGTCATCGTCGTTGGCACCTCA
CTAGCCGTTTATCCCTTTGCGTCTCTGCCCGAAGAAATTCCTCGAAAGGTAAAAAGAGTTCTGTGCAATTTGGAA
ACGGTGGGCGATTTTAAAGCTAACAAGAGGCCTACAGATTTAATAGTTCATCAGTACTCGGATGAGTTTGCCGAG
CAACTCGTTGAAGAATTAGGTTGGCAAGAAGATTTCGAAAAGATACTTACGGCCCAAGGTGGAATGGGGGATAAC
TCTAAAGAGCAATTACTTGAAATTGTACATGATTTAGAGAATTTGAGTTTGGATCAATCTGAACATGAGAGTGCA
GACAAGAAGGATAAGAAGCTACAGCGTCTCAATGGCCATGACAGCGATGAAGATGGAGCAAGCAACTCCTCTTCT
TCACAAAAAGCCGCTAAAGAATAA

>YJR072C 1.85   Hot
ATGAGTCTCAGCACAATCATATGTATTGGTATGGCCGGATCCGGTAAAACAACTTTCATGCAAAGGTTAAACTCC
CACTTGCGGGCAGAGAAAACGCCACCATACGTAATCAATCTTGATCCTGCAGTATTGAGAGTCCCCTATGGTGCA
AACATCGATATCAGAGACTCAATCAAATACAAGAAAGTGATGGAGAATTACCAGCTAGGTCCGAACGGTGCCATT
GTCACCAGTTTGAATTTGTTCAGCACCAAGATTGATCAAGTTATTAGGTTGGTGGAACAGAAGAAGGACAAGTTC
CAAAACTGCATCATCGACACTCCAGGCCAAATCGAATGTTTTGTGTGGAGTGCGTCGGGTGCCATTATCACGGAA
TCTTTTGCCTCCAGCTTTCCCACAGTGATTGCATATATCGTGGATACGCCTAGAAACTCATCCCCAACTACATTC
ATGAGTAACATGCTGTACGCCTGCTCCATTCTGTATAAGACCAAACTTCCCATGATTGTCGTTTTCAACAAGACC
GACGTGTGCAAGGCAGATTTTGCTAAGGAATGGATGACGGATTTTGAGAGTTTCCAAGCAGCAATCAAGGAAGAT
CAAGACTTGAATGGTGACAATGGGCTGGGCTCCGGGTACATGAGCTCATTGGTCAATTCTATGTCACTGATGCTT
GAAGAATTCTATTCCCAGCTAGACGTCGTGGGCGTTTCCAGTTTTACCGGAGACGGATTCGACGAATTTATGCAA
TGCGTAGACAAGAAAGTTGATGAATACGACCAATACTATAAGCAAGAACGTGAAAAAGCATTGAACCTAAAAAAG
AAAAAGGAAGAGATGAGAAAGCAAAAATCATTGAATGGCCTGATGAAGGATCTAGGGTTAAACGAGAAGAGCAGC
GCTGCAGCCAGCGACAACGACAGCATAGATGCCATTAGCGACCTCGAAGAGGATGCCAATGATGGTCTTGTGGAT
AGGGATGAGGACGAAGGCGTTGAGAGAGAATACACGTTCCCAGGCGAAGAAAGAACAAAAGGTGAGGTCAACGAG
AACTCTGCTCCAGACCTACAAAGGAGATATCAGGAAGCTATGCAACAAGTAGGGAAAACGGCCAGTAGCGAGACT
GCAGAAAATATCGCGAAGTATATTAGAAACTAA

>YLR226W 1.61   Hot
ATGTCTGCTACATCTTCAAGTGGTGACGTGAAAAAATTTCAAGCTGTACCAAAACCAACGAGCAATGCATCACCA
CCACCAGCATCGAGTGGGTTTAACGCAAGGACCCTATGGCCGGATTTGATAGAAACACCAGAAAACCAGTGGGTT
TTTGAGTGCAAAGATATTATTGAAAAGATAGGCACTAATGGACCGATAGCAGTGGAAATAAAAAAAAATATGGAA
AAATGCTTAATGTACTTTTACACACTTAAAAAAAAATTAAATCTTTTTGATCACACCTATACGGCATCATGCATT
CTATTTTACAGGTATTGGTTCATATATGGAATACCTACAGCCATAACTGAATGTATTCATATATCACAGGGAATT
CTGGTCACCGCTTGCAAAACAATGGAGAATAATAGGCCTATAGAGGCCTATATTAAGGCGACTTGTGAGTTTTTG
ATGCAAAATATACCGTCTTTAAAAAGTCGAACAAATATTGATAAACTTAAATGGGAATTTAGAGATAAGTTAGTG
ACGAATGAAAAAAAAATACTATGTTTATTTGGCTTTGACTTAAATATCAGTAATCCAAAAGAATTGATTGAAGAA
GTGTTCAGTGGCTATTATAGATTCAACAGGGATCACAATCTGCCAGAAAACTTTAAAAAAGCTTTTCCAAAAATT
CTTCAAGAATCAAGAAATTTTATGGTCCAAGCTGTCACCCAGCCAGTTTCTTTATTGTGCGACGGTTATACGTTC
ATTGTACTATCTCTCATATACTGTGGTTTAGAGTATAAAAAGCTTGTAGACAAAGATTTCAGATATCCGAAAAAC
TTCTTCAAGGATAGATTCCCTATCGAAGTAACGCCGGAAAATTTTGCCAATATCTTTACCGATTACAAGTTACTG
GAAGAGAATTTTTTCAACTTAAAAAGTAATAAAGGTGCTAAATTACAAATTGATTCAAGCATGATTGATTCGGTG
ATTGACGAAAGTGGCGATGTGGAAAATGAAGTTTCAGAAATCTCTGATCCGTTTAACTACGAGTTGATTAAATCC
GGTGAGGTGAAGGAAGAGTTTTTGAATCATATTGAAACAAGGGTTAAAGATTTACTAGATAAGGCTAAACAAGAA
AGTATGAAGAGAAAGGCTAAAGATCCTATAAGAACCCCAGATGCCAAAAAACCTAAAATATAA

>YJR116W 2.44   Hot
ATGAACGCCAACTCTACCACGACAGCAATCGGCCTCACCAGCCCCTTCGAAAAGCTGTCCTTCTTCCCTCATTCG
TCTAATTTAATACTGGCTCATTTGCATGAGATCATCTTCAGCTTCGTCTTCTACCAGCTCGCATTCTCCGTTGTC
GCACCGTTCTTGAATAAAGTGGTCTTTCGCAAGCACTACACCACGATCCGTGACCCACTGCTGAAAATTGATTTC
AATGTCCACACCGTCTCGATGATCCAGGCTGTCGTCTCGAACACCGTCCTGTTGCCCACCCTCACGACGCCAATG
CATTATAATGTTGTCACCTACACAGATTCGTACAGTTCAATGGTATCTTCCCTCAGCGCAGGGTACTTTATCTGG
GACTTGACTATGTGCGTGCGTTACTTCAAGCTTTATGGTCTCGAGTTCACGGGCCACGCCATTGGCTCCGTATAT
GTGATGCTGTTGTCCTTAAGACCGTTCTGCCAACCCTGGATCGGCAGGTTCCTTATCTACGAGGCCTCCACTCCC
TTTGTGAACATCAATTGGTTCATCATGCAATGTAACGCCAAGAGCAAGAACTCTATCCCTCTGTGGTTTAATGTC
GTCAATGGCCTTTTGCTCATGACTGTGTTTTTCGTCGTCAGGATTTGCTGGGGCTCCATCGCATCCGCTCTCTTA
TTCAGGCAGATGTGGAAGGTAAGAGACGAGTTACCCAAGTTTTCTGCTGTCACGATGATGTCGCTGAACATTTTC
ATGAACCTTTTGAACGTGCTTTGGTTCAAGAAGATGATTAGAATCGCCAAGAAACTTGCAAAACCAGCCCCAACG
TCGAAGCTAGACTAA

>YLR188W 1.92   Hot
ATGATTGTAAGAATGATACGTCTTTGTAAAGGTCCAAAGTTGTTGCGAAGTCAATTCGCATCAGCAAGTGCACTA
TATTCAACCAAGTCATTGTTCAAACCTCCCATGTACCAAAAAGCGGAAATTAATTTGATTATTCCACATAGAAAG
CACTTTTTGTTGCGGTCGATTCGATTACAATCAGACATTGCGCAAGGAAAGAAGTCCACCAAGCCCACTTTAAAG
TTGTCCAATGCAAATTCAAAATCCTCGGGGTTTAAAGACATTAAACGGCTATTCGTCTTATCTAAACCGGAATCC
AAGTACATTGGTCTTGCCCTCCTTTTAATCCTCATTTCAAGTTCAGTTAGTATGGCTGTACCTTCCGTTATCGGT
AAATTATTAGACTTGGCTTCCGAAAGTGACGGCGAAGATGAAGAGGGCTCAAAAAGCAATAAGTTATATGGTTTT
ACGAAGAAGCAATTTTTCACAGCATTAGGAGCAGTATTTATAATTGGAGCAGTTGCTAATGCAAGCAGAATCATC
ATTTTAAAGGTCACCGGTGAGAGACTGGTCGCAAGATTAAGAACGAGAACAATGAAAGCTGCATTAGATCAAGAT
GCCACATTTTTAGATACTAATCGTGTCGGTGATTTGATCTCAAGATTATCATCTGATGCATCTATAGTGGCCAAA
TCGGTCACACAAAACGTCTCTGATGGAACAAGGGCAATTATTCAAGGGTTTGTCGGTTTTGGCATGATGAGCTTC
CTCTCGTGGAAATTGACTTGCGTAATGATGATTTTAGCCCCTCCTTTAGGTGCTATGGCACTGATATATGGTAGG
AAAATACGAAACCTATCAAGACAGTTACAAACCTCAGTAGGTGGGTTAACAAAAGTGGCAGAAGAGCAATTAAAT
GCTACTAGGACAATTCAAGCATATGGTGGTGAAAAAAACGAGGTTCGCCGCTATGCAAAAGAGGTTAGAAATGTA
TTTCATATTGGTCTGAAGGAAGCGGTTACTTCCGGTTTATTCTTCGGAAGTACTGGGCTAGTTGGCAACACTGCA
ATGCTGTCGTTATTATTGGTTGGAACAAGCATGATTCAAAGTGGTTCAATGACTGTCGGTGAATTATCTAGTTTC
ATGATGTATGCCGTGTATACCGGAAGTTCATTATTTGGTTTATCGAGCTTCTATTCAGAACTTATGAAAGGTGCT

GGTGCGGCTGCCAGGGTTTTTGAATTAAATGACCGTAAGCCATTGATTCGTCCGACTATTGGAAAGGATCCTGTG
TCATTAGCCCAAAAACCCATCGTTTTCAAAAACGTGTCATTCACTTATCCCACTCGGCCCAAACACCAGATTTTC
AAGGATTTGAATATTACTATCAAGCCTGGTGAACACGTTTGCGCTGTCGGTCCATCAGGAAGCGGCAAATCAACA
ATTGCGTCTTTGTTGCTCAGGTACTACGATGTGAACTCAGGATCGATCGAATTTGGTGATGAAGACATCAGAAAT
TTCAACTTGAGGAAGTATCGAAGACTAATAGGATACGTGCAACAAGAACCACTACTTTTCAATGGGACTATTCTG
GACAATATCCTCTACTGCATTCCGCCTGAAATTGCGGAGCAGGATGATCGTATTAGACGTGCTATTGGAAAAGCT
AATTGCACAAAATTTTTGGCCAATTTTCCAGATGGATTGCAGACTATGGTTGGTGCTAGAGGCGCGCAATTGTCC
GGTGGTCAAAAGCAAAGAATTGCATTAGCTAGAGCGTTTTTACTAGATCCTGCCGTCCTTATTTTAGATGAAGCA
ACCAGTGCCCTTGACTCTCAAAGTGAAGAAATAGTTGCCAAAAATCTTCAGAGACGTGTGGAAAGGGGGTTCACT
ACTATATCAATTGCACATAGGCTTTCGACGATCAAACACAGTACTAGGGTGATTGTGTTAGGAAAACATGGCTCA
GTGGTTGAAACCGGTTCATTCCGAGATCTAATCGCCATTCCTAATAGTGAACTAAATGCGCTGCTTGCCGAACAG
CAGGACGAGGAAGGAAAAGGGGGAGTGATAGATTTGGACAATAGTGTTGCCCGGGAAGTATAA

>YBR026C 1.67 Hot
ATGCTTCCCACATTCAAACGTTACATGTCGTCCTCAGCTCATCAGATTCCCAAGCACTTCAAATCGCTCATCTAT
TCAACTCATGAAGTTGAGGATTGTACCAAGGTTTTGTCAGTGAAAAATTATACGCCTAAACAAGACTTATCTCAA
TCAATTGTGTTAAAAACTTTGGCCTTTCCCATAAACCCTTCGGATATCAATCAGTTGCAAGGAGTATACCCGTCT
CGTCCAGAAAAGACATACGATTACTCCACAGATGAGCCAGCCGCTATCGCCGGTAATGAGGGTGTCTTTGAAGTT
GTTTCTTTACCTTCGGGAAGTTCCAAGGGAGATTTGAAATTGGGTGACCGAGTTATCCCATTGCAGGCAAATCAA
GGGACTTGGTCCAATTATAGAGTTTTCTCTAGTAGTTCTGATTTAATCAAGGTAAATGATTTGGATCTGTTTTCT
GCGGCAACTGTATCTGTTAATGGTTGTACCGGTTTCCAATTAGTATCAGACTATATCGACTGGAACAGTAACGGT
AATGAATGGATTATCCAAAATGCCGGTACATCTAGTGTATCAAAAATAGTTACGCAAGTAGCAAAAGCTAAAGGG
ATCAAAACATTAAGTGTTATACGTGACCGTGATAATTTTGATGAGGTAGCAAAAGTTTTGGAGGATAAGTATGGT
GCTACGAAGGTTATTTCCGAATCGCAAAACAACGACAAGACTTTTGCCAAAGAAGTATTGTCCAAGATTTTGGGT
GAAAATGCAAGGGTGAGGCTTGCCTTGAATTCTGTTGGAGGTAAATCCAGTGCATCAATAGCACGTAAGTTGGAA
AATAATGCTTTGATGCTCACTTATGGAGGAATGTCAAAACAACCTGTAACTTTACCAACATCTCTACACATTTTC
AAAGGCTTGACATCCAAAGGGTACTGGGTGACTGAAAAGAACAAAAAAAACCCCCAAAGCAAGATTGACACCATC
AGTGATTTTATCAAAATGTATAATTATGGTCACATTATTTCACCAAGAGATGAAATTGAAACTCTTACCTGGAAT
ACTAACACTACTACTGACGAACAGTTACTAGAACTAGTCAAAAAAGGTATAACTGGGAAGGGGAAGAAAAAAATG
GTTGTTTTAGAATGGTAA

>YBR132C 1.72 Hot
ATGACAAAGGAACGTATGACCATCGACTACGAAAATGACGGTGATTTTGAGTACGATAAGAATAAATACAAGACA
ATAACCACTCGAATAAAGAGTATCGAACCTAGTGAGGGATGGTTGGAACCTTCTGGGTCAGTGGGTCACATAAAC
ACGATACCCGAAGCGGGCGATGTTCACGTGGATGAACATGAGGATAGAGGGTCTTCTATTGATGATGACTCAAGG
ACTTACCTGCTATATTTCACAGAAACTCGACGTAAACTAGAAAACAGGCACGTCCAGTTGATTGCTATTTCCGGT
GTCATTGGTACGGCGCTATTCGTGGCGATCGGAAAAGCTTTATACCGTGGAGGGCCCGCCTCTTTATTATTGGCA
TTTGCTCTTTGGTGTGTTCCAATACTTTGCATTACTGTGTCTACAGCGGAAATGGTCTGCTTTTTCCCTGTAAGT
TCCCCCTTTTTGAGATTAGCAACGAAGTGCGTTGACGATTCATTGGCTGTCATGGCTAGCTGGAATTTCTGGTTT
CTTGAATGCGTACAGATCCCTTTCGAGATTGTTTCTGTTAATACAATTATACATTATTGGAGAGATGATTATTCA
GCTGGTATTCCGCTCGCCGTTCAAGTAGTTTTGTATCTGCTTATTTCCATTTGTGCAGTCAAATATTACGGTGAA
ATGGAATTTTGGTTGGCTTCTTTCAAAATTATCCTTGCACTCGGCCTATTTACATTCACGTTCATTACCATGTTG
GGTGGAAATCCTGAACATGATCGTTACGGGTTTCGTAATTATGGTGAAAGTCCATTCAAGAAATACTTTCCCGAT
GGCAATGATGTGGGGAAGTCTTCGGGCTACTTCCAGGGGTTTCTCGCTTGCTTGATTCAGGCATCGTTTACCATA
GCTGGTGGCGAGTATATTTCTATGTTAGCGGGAGAGGTCAAACGACCAAGAAAAGTATTACCCAAGGCGTTTAAG
CAGGTGTTTGTGAGATTAACATTTTTGTTTTTAGGGAGTTGTCTGTGTGTTGGGATTGTTTGTTCGCCAAATGAT
CCTGACTTGACAGCAGCAATTAATGAAGCAAGGCCTGGCGCCGGGTCTTCACCTTATGTCATTGCAATGAATAAT
CTGAAAATTAGAATATTACCTGACATTGTTAATATAGCTTTGATTACAGCCGCCTTTTCTGCTGGTAACGCTTAC
ACTTATTGCTCATCCAGAACATTTTATGGTATGGCATTAGATGGCTACGCGCCAAAAATCTTCACTAGATGCAAT
AGGCATGGTGTGCCCATTTACTCTGTGGCCATATCTTTGGTATGGGCTTTAGTGAGCCTTTTGCAACTGAATTCT
AATAGTGCGGTCGTATTGAATTGGTTAATTAACTTGATTACTGCCTCTCAATTGATTAATTTTGTCGTCCTTTGT
ATCGTCTATTTATTTTTCAGAAGGGCTTACCACGTCCAACAAGATTCGTTACCCAAGTTGCCATTCCGTTCGTGG
GGTCAACCATACACTGCTATTATCGGCCTTGTTTCATGTTCCGCAATGATTTTAATACAGGGCTACACCGTTTTC
TTTCCCAAATTATGGAACACACAAGATTTTTTGTTTTCGTATTTAATGGTGTTTATCAACATCGGTATATATGTG
GGCTACAAATTTATTTGGAAACGTGGTAAAGATCACTTCAAAAACCCACATGAAATTGACTTTTCTAAAGAGCTA
ACAGAAATTGAAAACCATGAGATTGAAAGCTCCTTCGAAAAATTTCAATATTATAGCAAAGCATAA

>YBL015W 1.91 Hot
ATGACAATTTCTAATTTGTTAAAGCAGAGAGTTAGGTATGCTCCCTATCTGAAAAAAGTTAAGGAAGCTCACGAG
CTTATTCCATTGTTCAAGAATGGTCAGTACCTTGGGTGGTCCGGTTTTACAGGAGTGGGTACTCCCAAGGCAGTG
CCGGAGGCACTGATAGATCACGTGGAGAAGAACAATTTACAAGGGAAGTTGAGATTCAACCTTTTTGTTGGAGCT
TCTGCTGGTCCAGAGGAAAACCGTTGGGCTGAACACGACATGATCATTAAGAGAGCCCCTCATCAAGTAGGGAAA
CCCATTGCAAAGGCAATTAACCAGGGTAGAATTGAGTTCTTTGATAAACATCTGTCCATGTTCCCTCAGGATCTG
ACATACGGGTTCTACACCAGGGAAAGAAAAGACAACAAAATCCTTGATTATACTATAATCGAGGCAACGGCCATT
AAAGAGGACGGGTCTATCGTCCCAGGTCCCTCTGTCGGTGGTTCTCCAGAATTCATTACAGTCAGTGATAAAGTG
ATTATTGAGGTTAACACGGCTACGCCTTCGTTCGAGGGTATTCACGATATAGACATGCCCGTGAACCCACCTTTC

AGGAAACCATACCCATATCTGAAAGTGGACGACAAGTGTGGTGTTGACTCCATCCCGGTTGATCCTGAAAAGGTT
GTTGCGATTGTGGAGTCCACCATGAGGGACCAGGTCCCACCAAATACGCCCTCTGACGACATGTCCAGGGCTATT
GCAGGTCATTTGGTCGAGTTTTTCAGAAACGAGGTAAAACATGGTAGGCTACCTGAAAACCTGCTGCCTTTACAA
AGTGGTATAGGTAACATTGCTAACGCTGTCATTGAAGGGCTTGCTGGCGCCCAATTCAAGCACTTGACTGTATGG
ACGGAAGTGCTGCAGGACTCGTTCTTGGATCTTTTCGAGAACGGATCTTTGGACTACGCCACTGCTACTTCCGTG
AGATTGACTGAAAAGGGTTTCGACAGAGCCTTTGCAAACTGGGAAAATTTCAAACACAGATTGTGTTTGAGATCT
CAAGTTGTCTCGAACAATCCGGAAATGATCCGTAGATTGGGTGTCATCGCCATGAATACCCCAGTAGAAGTTGAC
ATTTACGCGCACGCCAATTCTACAAATGTGAATGGTTCCCGTATGTTGAACGGGTTGGGTGGATCTGCTGATTTC
TTGAGAAATGCAAAGTTGTCCATCATGCATGCCCCCTCTGCAAGACCAACTAAAGTAGACCCTACCGGTATCTCT
ACCATTGTTCCTATGGCCTCTCATGTAGATCAAACTGAGCATGACCTGGACATCTTGGTCACTGACCAAGGTTTG
GCGGATCTAAGAGGTCTATCGCCTAAGGAAAGAGCCCGTGAAATCATCAACAAGTGTGCTCATCCCGATTATCAA
GCTTTGTTGACCGATTACTTGGACAGAGCAGAGCATTACGCTAAAAAGCACAATTGCTTGCATGAACCACACATG
CTAAAGAATGCTTTCAAGTTCCACACCAACTTAGCTGAAAAGGGTACAATGAAGGTCGACAGCTGGGAACCAGTT
GACTAG

>YMR245W 1.61 Hot
ATGGTCTGCTCTCTCTTAAAGTCGATGACCCTCTTCTCCTTGAAGTACTTGATCCAGCTATGGGCGCAATTCTCC
TCAAATTTTTCATTTTCCACCTTGCAGTGCGACTTTATACTCTTGCTATTCTTGGGGTCCATTGCGTCCAAGATG
TCTGCCTTGTCCAAACACTGGAAGAATGCGTCCCTGGACTCCCAGCACAACTTTCTCTGTGAACGGGTATTAGGG
GGTTGTGATTCCTTTTTGCCACCATCAAATGAAAATAAGCCCATCGCTATATTACTACTATTCCTTTCTGTATAT
TCGTGTATCTATGTTCCTGCGCAGCGCTACAATAGTACCCGTCTATGTATGTATGTATGTATGTATGTAGGCGAA
TCTTCTCCCCCATTCTACAAAGCAGCACCTACCACCGCAGCACCGTTTCACATGTCCGTCTCTTTTGTTCAGCTC
AGCTACAATCGTCAATCGCACTTTCTTCCGCGCGGGCGAGCCCGGTTTTGTTGGATTTGCTCAGGTGCCAATATA
CCATCAAGGAACGGACAGGGACCTGACAATACAATAGGCCATTTACATCACAATGCACAGGGACAGAAATTTATA
CACGATAACAATACCAACTAA

>YBR105C 1.58 Hot
ATGATCAATAATCCTAAGGTAGACAGTGTAGCGGAGAAACCCAAAGCTGTGACATCAAAGCAGTCGGAGCAAGCG
GCTTCGCCAGAACCAACACCAGCCCCCCCTGTTTCTAGAAATCAGTATCCGATCACGTTCAACTTGACTTCAACC
GCACCCTTTCATCTTCATGACCGTCATCGCTACTTACAAGAGCAAGATCTTTACAAGTGCGCTTCTAGGGATTCG
TTGTCTTCCCTGCAGCAACTCGCCCATACACCTAATGGGTCGACAAGGAAGAAATATATTGTTGAGGACCAATCT
CCCTATAGTTCGGAAAATCCAGTCATTGTGACCTCTTCCTATAACCATACGGTTTGCACAAACTACTTAAGACCA
AGAATGCAGTTTACAGGGTACCAGATATCAGGATACAAACGCTATCAGGTAACAGTTAACTTAAAGACTGTAGAT
TTGCCAAAGAAAGATTGCACGTCGCTGTCCCCTCATTTATCTGGATTTTTGTCGATAAGAGGACTCACGAACCAA
CACCCGGAAATCAGTACATATTTTGAAGCCTACGCGGTAAACCACAAGGAATTAGGGTTCTTGTCCTCTAGCTGG
AAAGATGAACCTGTTTTAAACGAATTCAAAGCCACAGACCAAACAGACTTAGAACACTGGATAAATTTCCCCTCC
TTTAGACAGCTTTTCCTGATGAGCCAAAAAAACGGTCTCAACTCAACTGACGACAATGGCACTACAAATGCAGCC
AAGAAGTTGCCTCCACAGCAGCTTCCTACTACACCAAGCGCAGACGCTGGTAACATATCAAGAATTTTTAGCCAA
GAGAAACAATTTGACAACTACTTGAACGAACGGTTTATATTTATGAAATGGAAGGAAAAATTTTTGGTACCAGAT
GCCCTATTAATGGAAGGTGTAGACGGCGCATCTTATGATGGGTTTTATTACATTGTCCATGATCAAGTTACCGGG
AACATTCAAGGGTTTTACTATCATCAAGATGCTGAAAAGTTCCAACAGCTGGAATTAGTACCATCTTTGAAAAAT
AAAGTCGAGTCCAGTGATTGTTCTTTTGAGTTTGCTTGA

>YDL071C 1.52 Hot
ATGTTAGTACGAGACATAGCCGCCGAGGTTTATTTCGCTCAATCTGTTTGTTTCAGTTTTTCCGCGTTCTCTCTT
TTTCGCTCTTTTCTGGCTACCGCGCCCGCCTTATATATAAAATCTCTTCCGAATAACAAAGTGGCCGAAACAAGC
GCCAACGTCGACCATTTTGCCACCGTGTCACTGTGCACGAGCAACGAAAAACTGGCGTTGGTTGCGCCTCACGAA
TTGCTTGAGCCTCTTCCCAATATCATCGCGCATTTATTTGAGTCATTCTCTGCCGCTTTATTATTAAAAGTCTGT
GTGCTATCAAAATGCTCTTGCTCCTGTTCACCCTCTATGCGTAAACACTTGCGCGTTTCTCCCTTTCTCTTTTAG

>YLR055C 2.13 Hot
ATGGACGAGGTTGACGATATTCTAATTAACAACCAGGTGGTGGATGACGAGGAGGATGACGAAGAGATGCTGAGT
GGGCTGGAAAACGACTCAAAGCAGGACCTCGAGGGGAATGATGACGGTGGTGAAGATGAAGAGGATGACGATGAT
GATGATGAGGACGATGATGATGACGAGGACGAACGAGAGGACGACGATGAACAGGAGGACGACGATGGTGAGGAC
GACGCCGCAAGAATGGATAAGACTGCTACACCGACGAATGAGCACCAGCATGATGAGCAAAAGGCTGCTGCTGCT
GGTGCTGGCGGTGCAGGCGATAGTGGCGATGCTGTTACTAAGATTGGATCCGAGGATGTGAAATTGAGCGATGTT
GATGGAGGAGTGGGGTCCAGGGAAGCATCTTCCTCTACACACGAAGCCTCTGCTAATGGAGAGGTTTATGAGTAC
TATAAGCACATGTTGAATGCCGCACAGATTGCGGATTCGTACAATATCTACCCCACGGCAGCCATACCCATCCAG
ACGCACGTCAATGCGTTGGCCGTGTCCAGGGGTCTCAAGTACCTGTTTTTGGGCGGTAGCGATGGATACATAAGG
AAGTACGACTTGCTGAACACGCTTGAGGGGAAACTTTCTCTAACTATCCTGCAGAAGCATTCGTTGGCTGAGTCT
ATTCAGAACGCGGGTATCTTGCAGTCGTACTGGGAAAATGAGATCCCGCAGAAAAAATCAGAAATGAAACTCTCC
GCTAATAAGACAGATTACGAGCCCAAAGTTAGCCCCGTTCATTCTTTGGAAGTCCAAAGCGAATGCCTCTTTATA
CTGAGCGGGCTACAGAATGGTGGGATTACCATGCAGGGCGTTCGCTACATGGAGGGGAGCATTGCGCACTATTTT
AAGGGCAGGAATGGACATACCCAAATCGTTAACATACTGAGATTAAACGGTCAAGAGGACAGGTTTTTGAGTGGT
TCCTGGGATAAGCGTCTTTTGGAATGGGATTTGCAGACGGGTGACATAGTTAATGAGTTTAAAAAATCAAGGTCT
GAATTGTCATCTTTGGAAATGCGGCCGCTGTACTCGTCCGTGGATGTGTCCGGTAACGTCAACAGTGGTAAAGAG
AATGAAAATGCAGATGACGATATGGATTCTCTGTTTGGTGATGAAGACGAAGACGAAAAGCAAGATGCTGGCAAC

GAACCCGTCGAGACGGGGGATGGTTCTAATGGTGAAGAGAACAAAGAACAGATATCTGAAGAATCTTTGAACATA
GTCTATGATGAATCCGTTTTTATGACCTCAGGGTTGAACGGTTCCGTGCATATTTGGGACCGACGCATGACGCAG
TCGCCAGCATTGTCTCTGGAGAGAGGTGCAGGCGTCCCACCGTGGTGTTTGTCCGCATGTTGGGGTGTAGATGGT
GATCATGTGTATGCAGGGAGAAGGAATGCCTGTGTGGAGCAGTTTGACTTGAAGATGCCCTCGAAACCTATCCAT
AATTTGAAACTGCCTTCTATTTCAGGGCCTGTCTCTTGTGTTAAAGCCATGCCTAATAACAAGCATTTACTATGT
GCATCGCGGGATAATATCAGATTGTACAACGTTGAAATTGCAGTAGATGCTTCGAATTCGACTACAAAGAGTTCT
AAAGTGCCGTTCCTCATCGTGCCGGGCCATCACGGTGGTATTATATCAAACTTATACCTCGACCCCACTTCAAGA
TTTATAATAAGCACAAGTGGCAACAGAGGCTGGCAGGGGAATTCTACGGACACGACCCTTATTTACGATATAGAC
TTAGAATAG

>YCR063W 1.61 Hot
ATGCCGCGCATAAAGACCAGAAGATCCAAGCCTGCACCTGACGGGTTCGAAAAAATCAAGCCAACCCTCACAGAT
TTCGAAATCCAACTCAGAGATGCCCAAAAGGACAAGTCGTCTAAGCTCGCAGCAAAGTCCAATGAGCAGCTCTGG
GAGATAATGCAACTCCACCACCAGCGCTCTAGATACATATATACTCTGTACTACAAGAGAAAGGCCATCTCCAAA
GACCTTTACGATTGGTTGATAAAGGAAAAGTATGCTGATAAATTGCTAATTGCCAAATGGCGCAAAACCGGGTAT
GAAAAACTGTGCTGTCTGCGCTGCATTCAAAAGAACGAAACTAACAACGGTAGCACTTGCATCTGCAGGGTGCCT
CGTGCACAGTTAGAGGAAGAAGCACGCAAAAAGGGCACACAGGTGTCCTTCCATCAGTGCGTCCACTGCGGCTGC
CGTGGATGTGCAAGCACAGACTAA

>YDL144C 1.86 Hot
ATGTCTAGAATACTAGTTATCGGGGCAGGTGGCGTTGGGGTCATTACTGCGCTTTCGTTGTGGCTTAAGAAGGAG
AGCGACGTTTCCTTGGTAGTCAGATCTGACTACGATAGGGTATTGAAGCATGGCTACACAATCGAATCATGTGAC
TATGGCCGGTTAGAAGGTTGGAGACCCCACCATATTTACAGCAGCGTCGAGGACGCTGCTTCTGCCGCAGACAAC
CAAGGATACAACTATATTGTAGTGACTACCAAGAACATCATAGATGGGCCCGTTAATTCAAGGGTGTCCAACATC
ATCAGACCTGTGCTTGAGAAGAATAAAGAGCTACATGGGCCGCAATTGACGACACACATTTTACTGGTACAGAAC
GGAATCGATATTGAGAAGGAGATATGGGCGGAATTTCCCAGAGAGCAGTATCGTTACACGGTGCTTTCTGGCATC
CAGTTAATCGGATCCACTAAGATCGGGTCAGGCCATATCAGTCAAGTGGGCCAGGACCACTTATCGTGTGGAGCT
TTTGATCCACAGGACGCTGCTGCTATCCAGGCTGCCAACGACTTCGTCAGGATGTACAGCAACGAGGGGCAAAAC
TTTGTCGAATTTGACCCTAGGGTCCGTTACTCTCGGTGGAAGAAGTTGCTCTACAATGCAGCTATCAACACATCC
ACGGCACTTGTGGGGCTAGACGTTCCGCGTTGCCTGGAGTTTGGGGTTAACAAGAAGAGCACAGAAATTGAAGTG
TTCCACCCTGCCATGAGGGAGATCATCGCCATTGCGGCGAGTGAAGGCATCATTATCGAAGAGGAGTTCATAACG
ATGTTTACAGAGATTACCAGGAAAAAGGTGTTCAAGCCATCCATGTGCGTGGACTGCGAGAAGGGCCAATTGATG
GAGCTAGAAGTCATCCTAGGCAACCCCATAAGAATCGCGAAAAGAAACGGCGTCGCTACACCCACTCTTTCTATC
TTATACAACCTACTTGTGCTAGTACAGGCCAAACTAAAGGAGCGGAAAGGCCTATTGAAGTTCGACGAAAAAACT
GCCACTCTTGTGGACGAGTAG

>YBL064C 1.54 Hot
ATGTTTAGTAGAATTTGTAGCGCTCAATTAAAGAGGACGGCATGGACCCTTCCTAAGCAGGCTCACTTGCAATCA
CAGACGATTAAAACATTTGCCACAGCACCTATTCTGTGCAAACAATTCAAACAAAGTGATCAACCAAGACTAAGA
ATAAACTCTGATGCTCCTAACTTTGATGCTGACACAACGGTTGGTAAAATCAATTTTTACGACTACTTGGGCGAC
TCTTGGGGGGTCTTGTTTTCTCACCCAGCAGATTTCACCCCTGTCTGCACCACCGAAGTCAGCGCATTCGCCAAA
TTGAAGCCGGAATTCGACAAGAGAAATGTTAAATTGATCGGGCTTTCAGTGGAAGATGTTGAGTCCCACGAAAAA
TGGATTCAAGACATCAAGGAAATAGCAAAGGTTAAAAATGTTGGTTTCCCAATAATTGGTGACACTTTTAGAAAC
GTGGCATTCCTATATGATATGGTAGATGCCGAAGGATTCAAAAATATCAATGATGGGTCACTGAAGACCGTGAGG
TCTGTTTTCGTCATCGATCCCAAGAAGAAGATTAGACTGATTTTTACCTACCCTTCCACCGTCGGAAGAAACACT
TCTGAAGTGTTAAGGGTAATCGACGCCTTGCAATTGACTGACAAGGAGGGCGTAGTAACTCCAATTAATTGGCAG
CCAGCTGACGATGTCATTATTCCTCCCTCTGTCTCCAATGATGAGGCGAAGGCTAAATTTGGTCAATTTAATGAA
ATTAAACCCTATTTAAGATTCACCAAGTCGAAATAA

>YER153C 1.96 Hot
ATGTTGACTATCACGAAAAGACTGGTGACCACCGATGTGCGGTCGCGAATACTGTTAAGCAGTTTAAACGGGAAA
ATGTCCGATGCACTGGCGCTGCTGCGTCAGCAGCAGCAGACCAGCGTGGATGTGGAGCTGCTGCACACGATGCTA
GCGCGAGCCGCTGCGCTTGCCCATGCCGACACTATAGCATACATGTGGTATCAGCATGTGATGCCACGCCGGTTG
CCAGTAGAGGGCCGCCTGCTATGTGAAATGGCTGGCGTAGCATTGTACCAGGACAGGCTCTTCTTACCCGCGCAG
TTCCTCCAGCACTACCAGGCGATGAATCGCGATCGTCGCACCAGCCCAGAAGATGAACTGATTGAGTATGAGCTT
AGACGGATTAAAGTCGAAGCGTTTGCGCGTGGCACAATGCACTCCACGGCGCTCAGGGAAAAGTGGAAGGTATTC
TTGCAGGAGATGGATACGCTACCAGGGCAGCCGCCATTAAGGCTGCGCGACTTCCCGCAAATGACCAAGGCTATG
GGCATAGCATTGATGCAGCAAGATGAGCAAGCAGCTGCCCTGGCGTTGTTTGGACGACAGCCCCTAGTGATAAAG
AACGAATGGTCACTACCGCTACTACTGGCTGGTGTCCTTTGGCATGTTCCCGGCCCAGCGCAGGCGCGACGTGTG
CTGGCGGAGTTCCGTCAAAGTTATCGCGGGCTGCCGCTGCTGGATGCCGAACTAGTGATAAAGAGAAGAGGATTT
GAAATCAACACATAA

>YGL037C 2.01 Hot
ATGAAGACTTTAATTGTTGTTGATATGCAAAATGATTTTATTTCACCTTTAGGTTCCTTGACTGTTCCAAAAGGT
GAGGAATTAATCAATCCTATCTCGGATTTGATGCAAGATGCTGATAGAGACTGGCACAGGATTGTGGTCACCAGA
GATTGGCACCCTTCCAGACATATTTCGTTCGCAAAGAACCATAAAGATAAAGAACCCTATTCAACATACACCTAC
CACTCTCCAAGGCCAGGCGATGATTCCACGCAAGAGGGTATTTTGTGGCCCGTACACTGTGTGAAAAACACCTGG

GGTAGTCAATTGGTTGACCAAATAATGGACCAAGTGGTCACTAAGCATATTAAGATTGTCGACAAGGGTTTCTTG
ACTGACCGTGAATACTACTCCGCCTTCCACGACATCTGGAACTTCCATAAGACCGACATGAACAAGTACTTAGAA
AAGCATCATACAGACGAGGTTTACATTGTCGGTGTAGCTTTGGAGTATTGTGTCAAAGCCACCGCCATTTCCGCT
GCAGAACTAGGTTATAAGACCACTGTCCTGCTGGATTACACAAGACCCATCAGCGATGATCCCGAAGTCATCAAT
AAGGTTAAGGAAGAGTTGAAGGCCCACAACATCAATGTCGTGGATAAATAA

>YHL032C 1.67   Hot
ATGTTTCCCTCTCTCTTCCGACTTGTAGTATTCTCCAAACGTTACATATTCCGATCAAGCCAGCGCCTTTACACT
AGTTTAAAACAAGAACAGAGCCGTATGTCCAAAATAATGGAAGATTTACGAAGTGACTACGTCCCGCTTATCGCC
AGTATTGATGTAGGAACGACCTCATCCAGATGCATTCTGTTCAACAGATGGGGCCAGGACGTTTCAAAACACCAA
ATTGAATATTCAACTTCAGCATCGAAGGGCAAGATTGGGGTGTCTGGCCTAAGGAGACCCTCTACAGCCCCAGCT
CGTGAAACACCAAACGCCGGTGACATCAAAACCAGCGGAAAGCCCATCTTTTCTGCAGAAGGCTATGCCATTCAA
GAAACCAAATTCCTAAAAATCGAGGAATTGGACTTGGACTTCCATAACGAACCCACGTTGAAGTTCCCCAAACCG
GGTTGGGTTGAGTGCCATCCGCAGAAATTACTGGTGAACGTCGTCCAATGCCTTGCCTCAAGTTTGCTCTCTCTG
CAGACTATCAACAGCGAACGTGTAGCAAACGGTCTCCCACCTTACAAGGTAATATGCATGGGTATAGCAAACATG
AGAGAAACCACAATTCTGTGGTCCCGCCGCACAGGAAAACCAATTGTTAACTACGGTATTGTTTGGAACGACACC
AGAACGATCAAAATCGTTAGAGACAAATGGCAAAACACTAGCGTCGATAGGCAACTGCAGCTTAGACAGAAGACT
GGATTGCCATTGCTCTCCACGTATTTCTCCTGTTCCAAGCTGCGCTGGTTCCTCGACAATGAGCCTCTGTGTACC
AAGGCGTATGAGGAGAACGACCTGATGTTCGGCACTGTGGACACATGGCTGATTTACCAATTAACTAAACAAAAG
GCGTTCGTTTCTGACGTAACCAACGCTTCCAGAACTGGATTTATGAACCTCTCCACTTTAAAGTACGACAACGAG
TTGCTGGAATTTTGGGGTATTGACAAGAACCTGATTCACATGCCCGAAATTGTGTCCTCATCTCAATACTACGGT
GACTTTGGCATTCCTGATTGGATAATGGAAAAGCTACACGATTCGCCAAAAACAGTACTGCGAGATCTAGTCAAG
AGAAACCTGCCCATACAGGGCTGTCTGGGCGACCAAAGCGCATCCATGGTGGGGCAACTCGCTTACAAACCCGGT
GCTGCAAAATGTACTTATGGTACCGGTTGCTTTTTACTGTACAATACGGGGACCAAAAAATTGATCTCCCAACAT
GGCGCACTGACGACTCTAGCATTTTGGTTCCCACATTTGCAAGAGTACGGTGGCCAAAAACCAGAATTGAGCAAG
CCACATTTTGCATTAGAGGGTTCCGTCGCTGTGGCTGGTGCTGTGGTCCAATGGCTACGTGATAATTTACGATTG
ATCGATAAATCAGAGGATGTCGGACCGATTGCATCTACGGTTCCTGATTCTGGTGGCGTAGTTTTCGTCCCCGCA
TTTAGTGGCCTATTCGCTCCCTATTGGGACCCAGATGCCAGAGCCACCATAATGGGGATGTCTCAATTCACTACT
GCCTCCCACATCGCCAGAGCTGCCGTGGAAGGTGTTTGCTTTCAAGCCAGGGCTATCTTGAAGGCAATGAGTTCT
GACGCGTTTGGTGAAGGTTCCAAAGACAGGGACTTTTTAGAGGAAATTTCCGACGTCACATATGAAAAGTCGCCC
CTGTCGGTTCTGGCAGTGGATGGCGGGATGTCGAGGTCTAATGAAGTCATGCAAATTCAAGCCGATATCTTAGGT
CCCTGTGTCAAAGTCAGAAGGTCTCCGACAGCGGAATGTACCGCATTGGGGGCAGCCATTGCAGCCAATATGGCT
TTCAAGGATGTGAACGAGCGCCCATTATGGAAGGACCTACACGATGTTAAGAAATGGGTCTTTTACAATGGAATG
GAGAAAAACGAACAAATATCACCAGAGGCTCATCCAAACCTTAAGATATTCAGAAGTGAATCCGACGATGCTGAA
AGGAGAAAGCATTGGAAGTATTGGGAAGTTGCCGTGGAAAGATCCAAAGGTTGGCTGAAGGACATAGAAGGTGAA
CACGAACAGGTTCTAGAAAACTTCCAATAA

>YOR230W 1.77   Hot
ATGCCAAAAAAGGTTTGGAAATCATCTACGCCTTCCACTTATGAACATATCTCCAGCTTAAGACCAAAATTTGTG
TCTCGTGTGGACAATGTTCTGCACCAAAGGAAGTCCTTGACGTTCAGCAACGTTGTTGTTCCAGATAAAAAAAAT
AACACTCTAACCTCAAGTGTTATATACTCTCAAGGTAGTGATATCTACGAAATTGACTTCGCTGTACCACTACAA
GAAGCCGCTTCAGAACCGGTGAAAGACTATGGTGACGCCTTTGAAGGTATTGAAAACACCTCGTTGAGCCCAAAA
TTCGTGTACCAGGGTGAAACTGTCTCCAAAATGGCCTACTTAGATAAGACTGGAGAAACTACATTATTGTCGATG
TCTAAGAACGGGTCGTTGGCTTGGTTCAAAGAGGGTATAAAGGTCCCTATTCACATTGTACAGGAATTGATGGGT
CCAGCAACCAGCTATGCCAGCATCCATTCTTTAACCAGACCTGGTGATCTACCTGAAAAGGATTTCTCATTAGCC
ATCTCTGACTTCGGCATTTCCAATGATACCGAAACCATTGTGAAGTCTCAGAGTAATGGTGATGAAGAAGACAGT
ATTTTGAAAATCATTGACAATGCCGGTAAGCCTGGTGAGATTCTACGTACTGTTCATGTGCCTGGTACCACTGTG
ACTCACACCGTAAGATTTTTCGACAATCATATCTTTGCGTCTTGTTCAGATGACAATATTCTGAGGTTCTGGGAC
ACTAGAACTTCCGACAAGCCTATATGGGTGTTGGGAGAACCAAAGAACGGAAAATTGACCTCGTTTGATTGCTCT
CAGGTTTCCAACAACTTGTTTGTTACCGGTTTCAGTACGGGTATTATCAAGTTATGGGATGCCAGAGCGGCTGAA
GCTGCCACCACTGATTTGACTTATAGACAAAACGGTGAAGATCCAATTCAGAATGAAATCGCTAACTTCTATCAT
GCCGGCGGTGATTCTGTTGTCGACGTCCAATTTTCTGCCACTTCAAGCTCTGAATTCTTTACTGTGGGCGGCACT
GGTAATATTTACCACTGGAACACTGACTATTCCTTGTCAAAGTACAATCCTGACGATACCATTGCTCCTCCTCAA
GATGCCACTGAAGAATCACAAACAAAATCTCTGAGATTCTTGCACAAGGGTGGAAGCAGAAGATCTCCAAAACAG
ATTGGAAGAAGAAACACCGCCGCTTGGCATCCAGTTATTGAAAACTTGGTTGGTACTGTCGATGACGATAGTTTA
GTCAGCATCTACAAGCCCTATACCGAGGAAAGCGAATAG

>YJL135W 1.51   Hot
ATGTTCAACCAGCTATTGTTGGAACCAAATATGTGCAAAATATCGCCAGATATTCATTTCACGAATAACAAGGCA
AAAATGTTGCAAAAAATGTTTCTTAGTACCAAATTGAACTACTGCCTTTATCATGTGACTGAACTACGCTTGGCT
GTTTTGTTTTTCCCTCACCTTGCGCTAAAGTTGTCGTCACATTATAAGGGCAGTAAGAAAAAGCAGAGTTTAAGA
AAGTTTGTTTACATACAACCATCACGTAGAGGAAATTCTTCAAACCTACCAGACCAGCGGACACAAGCCATTTGT
ATAGATAAAAAAATGGTAG

>YPL250C 1.96   Hot
ATGTCCTCTGTGGAATCTTCCCCCATCTCGCGCTATGAAGACGAGGTCTTCCCTCTTTCGTTTTCAAACGTTGCT
TTCGAGCCCCCTATGCTCTCGCATAGCCCGGACAGATCGACTTATGCCGATGACTTTTCTCAATCTTACCAGCAA

GAATTGCTAACTTTTCCGCTATCGTACCCGATTGTCGATGAGTCAGAATGCACGCACACTAAAGATAAGACGGAC
AGCAACATAATAACGAGTACTGAAGACGATTGCATGTTCGATATGGAATTTAACGGCAACGCCGCCAGTGCAGTT
GCTGCTGCTAGTAAGGAATCTAACTCTGCCTCTGGGTTTGCCTTTGCAAGTAACGATGCCTTTGCCAATGTCGCA
CAACAGAACTACAGACTGTGGTTGTCATCGGTATGA

>YLR257W 1.72   Hot
ATGGTTGACGCTAGGGGTAGTACGCCATGTTTAATTGGTGATTCTATAAGAAACGTTAATGATGGAAATAGTCTG
GATTTCCAGTATACTAATCAGTTTAATGAGGAAAGTGAGGCTTCGAGGTTACTAACGCCACAAACGAGTTCCAAT
CACGCCCTAAGCAAGATGCAGAAGGATGATGATATTCGAGATAGATCTTATACATCTGTAGCGGAATTGAATCGT
GAGGGTGCTTTGCTGACCGACGAAGTTGATTTGGAGAATGTTGATGCTTCGAAAGTTCGCAGCAATAGAGACGAC
CTAGAGGCAGAGGAGAAAAGGAAAAAACTACTCTTGTTGAAAAAAAAGCAAAGAAATAAATCCATCAACTCGGAA
AGTTTTTCCTCTCCTTCTCTGCGTGCTTCCAAATCTAATTCACTGATTACATCAACTGATCCTGTCGAAGATCAC
ATCAGTAAGTACTCTTCATCAGGCACCCCTGAAAATATTACAGGTGAGGCAGACGACGAAGACGAAGACATAATC
AGAAACTCTTACGGGCAAATGATCAAAAACAATTCCAACAGGCCACATTTGGCGAAGGGTGAATCGTACCAGTCC
GCAGAACAGGAAATAGATCATACGGCGCCTGAAAAATCAGAGAAAAGACAAGAAAGAAGCGGTAGATCCTTTGAT
AGACAAAAATCTTCGGCTGAGTTTCTAAGATCCTTATCGAGGTCAATAAGTCGTGGACCCACAAAGAACAAAACG
GTTTCGCCCTCTAAGGGAGAAGACTCTAGAATGTATAGTACCAGCAACTACTCTATATCATTAGTTGATTTGGAG
AACGGACCCAAAATTATCCCGGAAACTTTAGAAGAAGAGCAGGAGGACGCCGAAAAGGAGGGTGTTTTGATGGAA
GACGAGGGTAATGAAGAATACACCAAAGATTTGGAAGAAGCAGCTAACAAAGCTCAACCACAGTAG

>YBR261C 1.55   Hot
ATGGACGTGCCTGCAGATTCCCATATCAAATACGAAGATGCCATCGACTATTGGACGGATGTGGACGCCACTGTG
GATGGTGTTCTAGGTGGATACGGAGAAGGTACAGTAGTACCCACAATGGATGTCCTGGGATCCAATAACTTCCTG
CGTAAACTGAAGTCGAGGATGCTGCCTCAGGAGAACAACGTGAAGTACGCTGTAGACATTGGTGCGGGGATTGGA
CGTGTAAGCAAGACCATGCTTCACAAGCACGCCGCTAAGATAGACCTTGTAGAACCGGTAAAGCCTTTTATCGAG
CAAATGCACGTTGAATTGGCTGAGCTGAAAGACAAAGGCCAAATTGGACAAATATATGAAGTAGGGATGCAGGAC
TGGACCCCCGATGCCGGCAAGTACTGGCTGATCTGGTGCCAATGGTGCGTGGGACACCTGCCAGATGCAGAACTC
GTCGCATTCCTGAAAAGATGTATTGTTGGTTTGCAACCTAATGGGACAATCGTGGTCAAAGAAAATAACACACCT
ACAGATACCGATGATTTCGACGAAACGGATTCTTCGGTCACAAGGTCAGATGCTAAGTTCAGGCAAATCTTCGAA
GAAGCTGGCCTAAAGCTCATCGCCAGTGAAAGACAACGTGGCTTACCAAGAGAATTGTACCCAGTGAGGATGTAC
GCACTCAAGCCCATGCCAAACTAG

>YBR240C 2.27   Hot
ATGGTCAATAGTAAGAGGCAGCAGAGAAGCAAGAAAGTAGCGTCATCCTCCAAAGTGCCCCCCACCAAGGGGAGG
ACATTTACTGGGTGCTGGGCATGCAGATTCAAGAAACGCAGATGCGACGAGAATAGACCAATCTGTTCACTGTGT
GCCAAACATGGAGATAATTGTAGTTACGATATCAGACTTATGTGGTTAGAGGAGAACATCTACAAAGTACGCAAG
CATTCACTGATCAGTTCATTACAGGCTCGCAAATCGAAATCGAAACCATTGTGCCAGAAAATCTCAAAATCAAGG
TTTAAACAAATGACCCATTTTAGACAACTATCACCCCCGACAAGTGACTGCGAAGACAGCGTGCACGAGGCAAGT
AAGGAAACTACGCTTCCTAACGATAATACATTCACCATAAGCGTACGGAGATTGAAGATATACAATAACGCTGTG
GCCTCTGTCTTTGGTAGCATGACAAATAGAGATTATACTCAAAAGCGCATAGACAAGAAGTTGGACGAATTGCTT
AATATGGTGGAAAACGACATATCTGTGGTCAACCTCAATTGCTCCAAACACGGGCCTTACTCAGTTTTCAGGGCC
AATCCGGCTGCAGTTACCTCTGCTCTTACAGATCAGCTGCCGTCGCCGGGTCATTCGATGTCGTCAGCAGAGGAA
ACCACTACTGCAGCGCTGTCATCGCCGCCAGAAGACAGCACCTCTTTGATCGATATTATCCAAGGCAAAATTTTC
GGGATCCTATGGTTTAATTGCTACGGGAACATGATATTGAACCGACAAGAATACACGACGTGGTTCATCAATAAG
ATGAGGAACTCGTTGACCACGGAATTCATACGCTTCCTTGGGAAAATAATTGACGACCCGGACATCAATATGGCC
TCGTGCCTGTTCAAAGAGTGCATCGCTCGTTGGAGTTGTGTCGATTGGCAATCCATAGCTATAACAATGCTGGTC
ATCATTCATGGATATACCTGCCCCAACTTGACAAAGCTTCTAAGAGTGTGGTTTCTCCAGCAAAAGCTATTGAGA
TTTTCGATGTATCCGCTGGTCAACTTTATCATAAACAATACACAAGATTTAGACGTCCTGTACCATTGTAATGGA
TTGCTAGGGAATGCCGACTTATTCGAGGATCCCTACCAGGACGAGCTTACGTCGGAGTTACATGTCTTGGTCACG
GAGCGCCTAGTGAATAGTTGGAAAGATACAATATTGCAACAACTTTGCTCATGTCAAGATACCACACTTTCATGT
TCCCAACTGCGGTACTGGCAGCTACAACTGAAGTGTAATCAACAATTTTACAAGGATGTATATGCCATGCAGGAC
TAG

>YIL011W 1.52   Hot
ATGTCTTTCACTAAAATCGCTGCCTTATTAGCTGTTGCCGCTGCCTCCACTCAATTGGTTTCTGCTGAAGTTGGA
CAATATGAAATCGTCGAATTTGACGCTATTTTGGCTGATGTCAAGGCCAATTTGGAACAATATATGTCCTTGGCA
ATGAATAATCCAGACTTTACCCTACCATCTGGTGTTTTGGATGTTTACCAACATATGACCACTGCTACTGATGAT
TCCTACACCAGTTATTTCACCGAAATGGACTTTGCTCAAATCACCACTGCCATGGTTCAAGTGCCTTGGTACTCT
TCTAGACTGGAACCTGAAATCATCGCTGCTTTACAAAGCGCGGGTATCAGTATTACCTCCTTAGGTCAAACCGTT
TCCGAATCTGGTTCCGAATCTGCCACTGCTTCCTCAGACGCTTCTTCTGCTTCTGAATCCTCTTCAGCTGCTTCT
TCTTCTGCTTCTGAATCCTCTTCAGCTGCCTCCTCTTCTGCTTCTGAATCCTCTTCAGCTGCTTCTTCCTCTGCT
TCAGAATCTTCTTCTGCTGCCTCCTCTTCTGCTTCTGAAGCTGCTAAGTCTTCTAGCTCTGCCAAGTCTTCTGGC
TCTTCTGCTGCTTCATCTGCTGCTTCATCTGCTTCTTCCAAGGCCTCTTCTGCAGCTTCCTCTTCTGCAAAGGCC
TCCTCTTCTGCAGAAAAATCTACTAATAGCTCCTCCTCTGCTACCTCCAAGAACGCTGGTGCAGCTATGGACATG
GGCTTCTTCTCCGCTGGTGTTGGTGCCGCTATTGCCGGTGCCGCTGCTATGCTCTTATGA

>YJL152W 2   Hot

ATGCCGCATTTAGCCGCCGAAGCGCATACTTGGCCTCCGCATATTTCACATTCAACACTTTCGATTCCGCATCCA
ACCCCGGAACACCGGCACGTATTTCATAAAAAGGACGTGAAGAACAAAAGGAACGAAGAAAAAGGCAATAATTTA
CTCTATGTGTTATTTAGAACTACGGTGATAAAGAGCTCGTTCCGATCACTAAGTACGGCCGGAAGAGAGCTGTTG
TTTGTTGTCCATCAAGGGCACATCGGCACCGGCCTCATCGTCTTCATCATATGCTGGAGGCTGTGCTTGAGATTC
CCTCTGCAGGGTGAGCTTCCAGGTCACGGTCTACGGCGGGCGCAGTCGCATGTCTGCGTGA

>YCL035C 1.7 Hot
ATGGTATCTCAAGAAACTATCAAGCACGTCAAGGACCTTATTGCAGAAAACGAGATCTTCGTCGCATCCAAAACG
TACTGTCCATACTGCCATGCAGCCCTAAACACGCTTTTTGAAAAGTTAAAGGTTCCCAGGTCCAAAGTTCTGGTT
TTGCAATTGAATGACATGAAGGAAGGCGCAGACATTCAGGCTGCGTTATATGAGATTAATGGCCAAAGAACCGTG
CCAAACATCTATATTAATGGTAAACATATTGGAGGCAACGACGACTTGCAGGAATTGAGGGAGACTGGTGAATTG
GAGGAATTGTTAGAACCTATTCTTGCAAATTAA

>YOL060C 1.67 Hot
ATGTCGTTTTTGCCACTAAGGTCTAGGTCACGATCAGGCGCTCCTCATTGGGTATACATCATCTTATATCACATC
TTTACGATACCGAAGATATATTCACTGCCGTTGTTATCCGGATCACACGTACTCAATTCTAGGGATGTTGCCGAT
TCCGGCCATAGCGTAGGTGATGAGGCAAGCGTTACCACGTACTATATTATCTCTATCATCCTAGTGCTGCTGGGT
GGTGTATTTGCAGGATTGACGCTGGGGTTGATGGGTCAAGATGAAGTCTACCTGAAGGTAATCAGCACTTCAGGC
TCGAATTCTGAAAAGAAACTGGCCAAGCGGGTGCTTGACCTAATATCTAGGGGGAAGCATTGGGTTCTAGTCACA
CTGCTGCTTTCTAATGTTATAACCAACGAAACATTGCCTATTGTTTTGGACAGGTGTCTTGGGGGTGGTTGGCAG
GCTGTAGTGTCGTCAACTATTCTAATTGTGATCTTCGGTGAAATTATTCCGCAGAGTGTCTGTGTTAAATACGGG
CTGCAAGTTGGGGCATTCTTCTGCCCCTTTGTTCTTGTACTGATGTATCTGATGTACCCAGTCGCATATCCGATC
GCGACTCTCCTGGACTATATGCTGGGTGAAGATCATGGTACGATGTACAAAAAATCCGGCTTAAAGACTTTGGTC
ACCTTGCATAGGACCATGGGGGTGGAACGGTTGACTAAAGACGAAGTTACAATCATCTCTGCTGTTTTGGATCTA
AAGGCAAAGAGGGTTGAGGAAATCATGACTCCGATTGAAAACGTGTTCACAATGAGTGCCGATACCATCCTAGAC
GATAAAACTGTCGAAAAAATCTTCAACTCAGGGTTTTCTAGAATCCCGATTTTTTTGCCCAATGAACCAAACAAC
TTCATCGGCATGTTACTTGTCAGGGTGCTTATCTCCTACGATCCTGATGACTGCCTCCCAATCTCCCACTTCCCC
CTAGCCACGTTGCCAGAAACTTCCCCGAACACATCTTGTTTGAATATTCTGAATTACTTCCAGGAGGGGAAAGCT
CACATGTGCGTCGTTAGTAAAGAGCCGGGCTCCTCGCATGGCGCTATTGGTGTTTTAACTTTGGAGGATGTCATT
GAAGAATTGATCGGTGAAGAAATCGTGGACGAGTCTGATGTCTTTGTGGACATGCATCAGCATATTATGAGACAA
CAACCGGGGCCCTTGAGCAAAAGGCACATTACTTCCTATTTGCATCATTTGTACACTAGCTCTCACAAGGAGCAC
AAAGCTGCTGACCAAGCTGATGAATCGTCTCCACTCTTGTCTCCATCCAATTCTAATCACCCATCCGAGCACCCA
CAGCAAGATTTGAATAATAAGTCTTGGAAGCAGAAGTCAAATGATGGTTACGACAGGTCCAACGCTGTATTGTCT
CCAACTCCACAAGTGACGGAACATGGCACCATCATACCTTCAAATCTAGCGTCCAACCCGTTGAACGTAAATAAA
TCTTTTGTTACCATTAAAAAGCCTGCCAATGTTCCGAAAATCATTACTACACACACTCCTCATTCTAGCAAAGAG
CCTTCTCCAGCACCACACTCAAATGATAAATCACTCTCCGCCGAAGAACAACAACTATTAAGTGATCATGCAGAG
CTATCTCGCCAAGCTGTTCTCCATACTCAACGGTCGGGTCAACCAACTCAAGTTACTACCTCGACTAAAACCACG
AGAAATAGCCCTGATAGTATATCAATTCCAAATTCTGGCGCGAATCACGGCAACGAAAATCAAAACGTTACAATC
TCAACCTCTTACCAAAATACGAAGAATGGTATTGTGGAATCTGTCATCACAGTCAAAGGTGTTCCAAAAAACCATC
ATTGGCCCCGCAAAAGATTGGGACGAATCCAAAAGTGAATATGGAAACGAGAATATCAATCAAGAGAATTCTAAT
CGAAGTGACGATAGAGAAAGTTCAAGTTCAAATGCAAGTTTATTTTCCAGTATTAAGAATAAATTTAAGAACGAA
AACGCTAACAACAATGATCGTTCTAATTTCACCGACTCTTTGTCAAGAACTTCCAATTATGATGCCAACGGCTCC
TCGTCGACCATAAAAAGATGA

>YGL235W 1.71 Hot
ATGACCTTGTGGCCACATCCGGGGTCATACAAGATCAAGTCAGCAACTTTGTTCTGCAGCAGAGACAAGTTGGGG
TGTGCTTTCTTGTCGGAAAGTTCCCTTTGCATGTATTTCTTATATAACTCTCTGTCTATCTGGGCTCTGGCCCCC
CACACAGCAGGACCCTTGCTTCTGTTCAGCATTTTGAATTGCACTCCAGCGAGATCAGTTACTTTGCCCATCAGC
CCATCGAGAGCGTCGATTTCCTTTACAAGGATGCCCTTACCCACTCCACCAATAGAGGGGTTGCACGAACACTTA
CCAATATCTGTTAACGATGGTGTAATGAGAGTAGTATGCGCACCAGTCCTAGATGACGCAGCTGCAGCTTCGCAG
CCTGCATGTCCAGCACCGATGACCACGACCTGTGTCTTGGTTGTGGGTTGGAAGCTTGTCAAAGAAGATATGGTC
AACCGCCTCCTGAGGACCTGTAAGGGAAATGAAGTACACGAAGACGCCAAGGTTGTTACACGCAGCATCGTTCTT
TGGGGCGTTTAG

>YJL121C 1.59 Hot
ATGGTCAAACCAATTATAGCTCCCAGTATCCTTGCTTCTGACTTCGCCAACTTGGGTTGCGAATGTCATAAGGTC
ATCAACGCCGGCGCAGATTGGTTACATATCGATGTCATGGACGGCCATTTTGTTCCAAACATTACTCTGGGCCAA
CCAATTGTTACCTCCCTACGTCGTTCTGTGCCACGCCCTGGCGATGCTAGCAACACAGAAAAGAAGCCCACTGCG
TTCTTCGATTGTCACATGATGGTTGAAAATCCTGAAAAATGGGTCGACGATTTTGCTAAATGTGGTGCTGACCAA
TTTACGTTCCACTACGAGGCCACACAAGACCCTTTGCATTTAGTTAAGTTGATTAAGTCTAAGGGCATCAAAGCT
GCATGCGCCATCAAACCTGGTACTTCTGTTGACGTTTTATTTGAACTAGCTCCTCATTTGGATATGGCTCTTGTT
ATGACTGTGGAACCTGGGTTTGGAGGCCAAAAATTCATGGAAGACATGATGCCAAAAGTGGAAACTTTGAGAGCC
AAGTTCCCCCATTTGAATATCCAAGTCGATGGTGGTTTGGGCAAGGAGACCATCCCGAAAGCCGCCAAAGCCGGT
GCCAACGTTATTGTCGCTGGTACCAGTGTTTTCACTGCAGCTGACCCGCACGATGTTATCTCCTTCATGAAAGAA
GAAGTCTCGAAGGAATTGCGTTCTAGAGATTTGCTAGATTAG

>YBR265W 1.51 Hot

ATGAAGTTTACGTTAGAAGACCAAGTTGTGTTGATCACTGGTGGTTCACAAGGTCTTGGAAAGGAATTCGCCAAA
AAATATTATAATGAGGCTGAAAACACAAAGATTATTATCGTCAGTAGGTCAGAGGCTAGACTGCTGGACACATGC
AACGAAATTAGGATTGAAGCTCACCTGAGAAGGGAAACCACTGACGAGGGCCAAGTGCAACATAAGTTGGCTGCG
CCCTTGGACCTTGAGCAACGGTTATTTTACTACCCATGCGACTTGTCCTGCTACGAATCCGTGGAATGTTTGTTC
AATGCCCTGAGAGACTTGGATTTACTCCCTACACAAACGTTATGCTGTGCAGGGGGGGCTGTTCCTAAGTTATTT
CGTGGGCTAAGCGGACATGAGTTGAACTTGGGTATGGACATCAACTATAAAACAACTTTGAACGTGGCACATCAG
ATTGCCCTTGCAGAGCAAACCAAGGAACACCACCTCATCATCTTTTCTAGTGCCACCGCGCTTTACCCATTTGTG
GGCTATTCCCAGTATGCGCCTGCAAAAGCTGCAATCAAATCACTGGTAGCAATCTTAAGACAAGAACTGACGAAC
TTCCGTATCAGTTGTGTTTATCCTGGTAATTTTGAAAGCGAAGGTTTCACTGTAGAGCAGCTAACGAAACCCGAA
ATTACAAAGTTGATCGAAGGCCCCTCAGACGCTATCCCATGCAAACAAGCATGTGATATCATTGCCAAGTCGCTG
GCCAGAGGTGATGAAGACGTTTTTACAGATTTTGTCGGATGGATGATAATGGGGATGGACCTTGGGCTCACCGCA
AAGAAAAGCCGCTTTGTTCCGTTGCAATGGATTTTTGGTGTCCTATCAAACATTCTGGTCGTGCCATTCTACATG
GTTGGCTGTTCCTGGTATATCAGGAAATGGTTTCGTGAAAATGACGGCAAGAAGGCCAACTGA

>YJL151C 1.54   Hot
ATGGACAGAGACCATATTAATGACCATGACCATCGAATGAGCTATTCCATCAACAAGGACGACTTGTTGTTAATG
GTTTTGGCGGTTTTCATTCCCCCAGTGGCCGTCTGGAAGCGTAAGGGTATGTTCAACAGGGATACACTATTGAAC
TTACTTCTCTTCCTACTGTTATTCTTCCCAGCAATCATTCACGCTTGCTACGTTGTATATGAAACGAGTAGTGAA
CGTTCGTACGATCTTTCACGCAGACATGCGACTGCGCCCGCCGTAGACCGTGACCTGGAAGCTCACCCTGCAGAG
GAATCTCAAGCACAGCCTCCAGCATATGATGAAGACGATGAGGCCGGTGCCGATGTGCCCTTGATGGACAACAAA
CAACAGCTCTCTTCCGGCCGTACTTAG

>YER147C 1.76   Hot
ATGGAAAATCTTGGCGACAAACTTAGTATTTCACAAGTATACCACTTAGCACAGGAATACAGAGACCATGCATAT
TCAATTGCGAATAAAATCGGTTCTGAGGAAGGGTTGAAGCAGTATTATGGCCTCATGAACATGTCTATCCAGATG
TTTCAGCTATTGAAAACGAAATGTACGCTTTCAGTTCTGGAGGATAGTAAGGTCACGTTTGAGATGGTGGAGTTA
TTGATACAGGAGACGTATAATTTTGACCTAGCAGAGCTTTACATTTCCAGTTTGAAAGAACGGTTACAAACGCAC
CAGAGCGACACAGATTTGGTAGAAGAGATCATGCGTTGCGAGTTTCTGCTGTTACATGACTTGCCACTTATGAGA
GACAGTAAGTTCCACTATAAGATTGCACTAAGAAATTGCAATGAATTAGTCCAGTACATGGTAAATTTGCAGGAT
GAGTTGTATCAAAATTGGGCATCCGTATTCCAGTATGTCGGCGTGATGCTTTGCATTAAGCTGAAACAGCACCGC
AGGGTGAAGACGAGTTTTCATGGGCTACTCTCGCAATGTCGGGAAAAGTCACAATGGAAATGGTTTCTAAATCTG
TGTTATGTGAATTACTTGCTAAATGAGAGGTTCCCAATCCCCGAGGATGCTCTGCAGGAGCTGCGAAGCACGGAG
CTGCACACGGTGGGGCCTGAGCTATATGCATGGAAGTTGGCATTAGAGATGGTAATCCAGCTTTGTAAAGACGGG
AATATTACGGACCACTTGAACGAATTCAAAAATTTCTTTGACACAAACAAGCAATCTCTAGTCACTAATGAAGGT
AAGGGATGTGTGATCAAAATCATGCCAAGGATAGCGCTGAAAGTAGAGTTGCCTATGATTTTCCACTACAAAGAA
TTAAAGAACATACTGTTATTATTGCAAAGTGTCAGCTACATCGTGAACTGTTATGACGAAAAGGGCAATTTTTCC
AGGAAGTTCCTCCCCAAAGTCTATTCTACAACGCAGAAACTGATTAAGAACATTGCCGCTGGCGGTGTTTCGATG
AATGAGCTGGACTCACGAATCCAGACATACAAGAGCATTCTTGAATTCTGTGAGTTTTACAAAGTGTGGGAACAA
ACACTCTTGAAGGGGGCCGTAGTGACGACCGAGTCACCTAAGTTAGGGCCCTCTCCAGGCTACGTGAGGCTGCTG
CAAGCCATGAAGGTTCAGTTTGAAGGCGGCGGGGCCGTAGAAGAGTACACGCGGCTTGCACAATCTGGCGGTACA
TCCAGCGAGGTGAAGATGATATCGCTTCTGAATTGCTACACAGTGCAGGCGGCAAGAGTGAGCCGATGTTCGGGG
GACAAACAGGGCGAACTCGTAGAGCAATGCAACAAGGTGTGGCTTCAGGTGGAGAAATTGCTACAAGAAACGGAT
CTGCAGTTCAACCCCATATGGGAGTGCACAGTAACGATCCTATGGCTTTTCAGCCATTTCGAGCCGTTCAGTTGG
AACCCGCTGCCATGTAGTGACAAGCAGCGCGCCGAGTACGTGTCGAAGCTCCGCGAGTTCTACTCTAGCAACAAG
TTCGTTGCCGGAGAAGCCGTTGCGGACAACCGCTTCAAATTGAAGAAGGCTCTGTTGCTGCAAATACTGGTAAAC
TACTTAGGCGGTCGAATGCTCGAACACGATCTGGGCGAGATCTATGCGATTTCAGCAAAGTGTTTCGATATGTGC
CGCCAGCAGGGCGGCATGCGCAAGGTCCAATACGTGATCGGAATATGGCATTTAATGAATTGTACGGTGGCGATG
CGGGGAAAAGACGTCGCTCTCACGAACGCGAAGCTGGAGGCGCTGGTTAAGCAAATCACCAGTGTCAAACAATAA

>YGL166W 1.72   Hot
ATGGTCGTAATTAACGGGGTCAAATATGCCTGTGAAACGTGTATCAGGGGTCACAGGGCGGCGCAGTGTACTCAC
ACTGATGGTCCGCTACAGATGATCAGACGCAAGGGAAGACCATCGACCACATGTGGCCATTGTAAAGAGCTGAGA
AGAACCAAGAACTTCAACCCATCCGGTGGGTGCATGTGTGCCTCTGCACGACGGCCAGCTGTTGGCAGCAAGGAA
GATGAAACACGATGTCGTTGTGATGAGGGTGAACCTTGTAAATGTCATACCAAGAGGAAAAGCAGCCGGAAATCA
AAGGGAGGGTCATGCCACAGAAGGGCAAATGATGAAGCAGCGCATGTCAATGGTCTCGGTATTGCAGATCTGGAC
GTTCTTTTGGGCCTAAATGGTCGCTCGTCGGATGTAGACATGACAACCACATTGCCGAGTTTGAAGCCACCTCTG
CAAAACGGAGAAATTAAGGCCGACAGCATTGACAATCTTGATTTGGCTTCCCTCGATCCGCTTGAGCAAAGCCCT
AGTATATCTATGGAACCTGTTAGTATCAATGAAACAGGAAGCGCATATACAACTACGAACACAGCACTAAACGAT
ATTGACATTCCATTCTCCATCAATGAGTTGAACGAGCTATACAAACAAGTATCTTCGCATAACTCACATTCACAA
TAA

>YOR203W 1.54   Hot
ATGGTACCAACGATGTTCCCTCCACCAAAGGTGTTCTTATGTAGTGACACCGATTATTTAAAGCTGCAGCATACG
ATATATATACATGTGTATATATGTATACCTATGAATGTCAGTAAGTATGTATACGAACAGTATGATACTGAAGAT
GACAAGGTAATGCATCATTCTATACGTGTCATTCTGAACGAGGCGCGCTTTCCTTTTTTCTTTTTGCTTTTTCTT
TTTTTTTCTCTTGAACTCGAGAAAAAAAATATAAAAGAGATGGAGGAACGGGAAAAAGTTAGTTGTGGTGATAGG
TGGCAAGTGGTATTCCGTAAGAACAACAAGAAAAGCATTTCATATTATGGCTGA

>YER159C 2.27 Hot
ATGGCAGATCAAGTACCAGTTACAACACAACTACCACCAATAAAACCTGAACATGAGGTACCACTTGATGCTGGA
GGGAGTCCAGTAGGTAACATGGGTACCAACTCGAATAACAACAACGAGCTAGGTGATGTATTCGACAGAATAAAG
ACACACTTCCCTCCGGCCAAGGTAAAGAAAATAATGCAGACAGACGAGGATATAGGAAAAGTTTCACAAGCCACG
CCCGTAATAGCGGGCAGGTCCCTAGAGTTTTTATAGCGTTATTGGTGAAAAAAAGCGGGGAGATGGCAAGAGGA
CAAGGAACCAAGAGAATAACCGCCGAAATACTAAAAAAAACAATTTTAAACGACGAAAAATTCGATTTCTTAAGG
GAAGGTCTATGCGTAGAAGAAGGCCAAACACAACCGGAGGAAGAGAGTGCCTGA

>YLR348C 2.13 Hot
ATGTCAACCAACGCAAAAGAGTCTGCCGGTAAGAATATCAAGTATCCATGGTGGTACGGAGGTGCAGCGGGTATC
TTTGCTACCATGGTGACACATCCTCTTGACTTGGCCAAAGTCAGACTGCAGGCGGCTCCTATGCCCAAGCCCACA
CTCTTCAGGATGTTGGAGAGTATCTTGGCGAATGAAGGTGTGGTGGGGTTATATTCCGGCCTGAGCGCTGCTGTG
TTGAGGCAATGTACATACACGACGGTGAGATTCGGTGCATACGACTTGTTGAAAGAGAATGTGATCCCACGCGAG
CAATTGACTAACATGGCGTATCTTTTACCTTGCTCCATGTTCAGTGGTGCCATTGGGGGGCTTGCAGGGAATTTT
GCAGATGTTGTAAATATCAGAATGCAAAACGACTCTGCTTTGGAGGCAGCGAAGAGGAGAAACTACAAAAACGCT
ATCGATGGTGTGTACAAGATATATCGCTACGAGGGTGGACTGAAAACCTTGTTTACCGGTTGGAAGCCTAACATG
GTTAGGGGCATACTGATGACGGCAAGTCAGGTCGTTACGTATGACGTGTTTAAGAACTACTTGGTCACAAAATTA
GATTTCGACGCATCCAAGAACTATACACACTTGACAGCGTCCCTGTTGGCTGGCCTGGTGGCCACCACTGTGTGC
TCTCCAGCAGACGTCATGAAGACACGTATCATGAACGGCAGCGGAGACCATCAACCGGCTTTGAAGATCCTTGCC
GACGCTGTCCGCAAAGAGGGCCCATCTTTCATGTTCCGGGGGTGGTTGCCAAGTTTCACTAGACTGGGTCCATTC
ACAATGTTAATTTTCTTTGCAATTGAGCAATTGAAAAAACATAGGGTTGGCATGCCAAAGGAAGACAAGTAG

>YOR333C 1.56 Hot
ATGACAATACAGTTTGGCTTACACATGATGGTTGGAATAATATCAATAGAGGAGTTATCTATTTTCCTCAGGTCT
CTGGGGAAAAGGGAATGTTCAGTTAAAAAGGACCATTTAGGAAACTTCTCGGACATTGAGATAATATTTCCCTTA
GAATTGAATACCGTACACGAAATGAACAGTGAATCAGAGGCAGAAATGGGCTTCAACGATAGTAACTGTTTCCGC
AATATGGTGCTGTTGGTGTTTGCAGCAGTGGATGTGTCAGCATACTTTCTAAGAAATGGCGTGTTTGGTCTTCCA
AAAGTTATTCTCGACAAAGGTTGGAAACAAGATATAGAACGTACCAGGAGACGCCGATTCATGAGATGCTTGATT
GAAAATCACTTTCACTACGATTGCGTATACCGTTACTGTTAG

>YKL198C 1.69 Hot
ATGACAGTCTCACACAATCATTCTACGAAGATATCCCAACAACCAATCTCCTCGGTATCAGCATTTAAGTTCTTC
GGAAAGAAGCTGTTAAGTTCAAGCCATGGGAACAAGTTGAAGAAAAAGGCGTCTCTACCTCCAGACTTCCACTCT
ACAAGTACTAATGACAGCGAATCCTCCAGCCCAAAACTGCCGAATTCGTTGAAAACCTCTCGCCGTGCAAACTCT
TTCGCTCACACAACCAACAGCAAGAGATCTTTATCTTCCGCCTCAACCAAGATCCTACCTCCGGCCGGCTCCAGC
ACGTCCATCTCAAGAGGAAACAGACATTCGTCCACTTCGCGTAATCTCTCAAACTCCAAGTTCAGTAGCGAACGA
TTAGTGTACAATCCATACGGCGTCTCAACCCCAAGCACGTCACTCTCGTCCGTCTCTACCTCCATGAAGAAAGAC
CCTGATCTGGGCTTCTACCTTCACGATGGGGATTCCAAAATCCGCATGCTGCCGATCCCAATTGTGGACCCAAAC
GAGTATCTGCCCGACGAGATGAAGGAGGCAAGCATCCAGTTGAGCGATAACTTCGTCTTTGATGATGAGAATAAG
ACCATCGGATGGGGCGGTTCGTGCGAAGTGCGCAAGATCCGCTCCAAGTACCGCAAGAAGGACGTATTTGCTCTA
AAGAAGCTCAATATGATCTATAATGAAACGCCCGAGAAATTCTACAACGCTGCTCCAAAGGAGTTTATCATCGCA
AAGCAGCTAAGTCATCATGTTCACATCACAAATACTTTCCTTCTAGTCAAGGTGCCCACCACCGTCTACACCACT
CGCGGGTGGGGGTTCGTCATGGAGCTAGGTCTACGAGATTTGTTCGCGATGATACAAAAATCGGGCTGGCGCCAC
GTGGCCCTAGCAGAAAAGTTTTGTATATTCAAACAGGTGGCGTGTGGTGTCAAGTTTTGCCACGATCAGGGCATC
GCCCACCGTGATTTGAAACCGGAAAATGTACTGCTATCCCCGGACGGCGTGTGCAAGCTGACAGATTTTGGTATC
TCAGACTGGTACCACACGGATCCACACGACCTGTCCAGCCCTGTCAAGAAGTGCGCAGGGATGATCGGCTCGCCG
CCGTATGCTCCCCCGGAGGTCATGTTCTACGACTCCAAGAAGCACTACGATACGGAATTGCAACAGCCGTATGAC
CCACGCGCTCTAGACTGTTATGGCCTGGGTATTATCCTCATGACCCTCGTCAACAACGTCATTCCATTTTTGGAA
TCCTGCAGCTTCGACACTGGTTTCCGCGATTACTGTGACGCTTATGAAAACTTTATCCGTCTTCACGATCGCGCA
TTCCGCAATCGCGGCAATTACCGCCCGGGGCCGGGAATGGAGTATCACTTGGCTAGAAATTTCAAGAACGGACAT
GCATCTCGCGTGGCATGGCGGCTCGCTGACCCAGAAGCCGCCACCCGCTACACCATCGACGACCTCTTCGAAGAC
CCATGGTTCCAAGGAATTGAGACTTGTGTTGATGCCAACGACAAATATGTGTGTAAGAAACCTATTATCAAAACC
ACTACGTACGAGAATCCGCGGGGTTTCCATATCGCTACAGATGTTGCTGCAACCACACCCACCTCAAACCCATTC
CTTAAGAACCGAGTCCCCATCAGGTCAATGGTAGATATAGCCGCCCATCCTTCCCCAACGGCCACGGTTTTAGCG
TCGTCGCCTCCGCCGCCTCCTCCGGCGACTCATGTGCCAGCCGAGGCCTTATTTACACTTAGGGAAACCCCGCCG
CCCCAGCTAGCAACCCTCACGCTTAGCGAAGAACCTCCCGCAACTCCCGCACCTTCCGCACCTTCCGCACCTTCC
GCTCGCGTCCGTGGCCACTCCCCGCACAGGGTAGGTGCATCACCATCTGAACATTGTCAACAGCTTGGTCCATAG

>YPL171C 1.87 Hot
ATGCCATTTGTAAAAGGTTTTGAGCCGATCTCCCTAAGAGACACAAACCTTTTTGAACCAATTAAGATTGGTAAC
ACTCAGCTTGCACATCGTGCGGTTATGCCCCCATTGACCAGAATGAGGGCCACTCACCCCGGAAATATTCCAAAT
AAGGAGTGGGCTGCTGTGTATTATGGTCAGCGTGCTCAAAGACCTGGTACCATGATCATCACGGAAGGTACGTTT
ATTTCCCCTCAAGCCGGCGGCTATGACAACGCCCCTGGGATTTGGTCTGATGAGCAGGTCGCTGAGTGGAAGAAT
ATCTTTTTAGCCATCCATGATTGTCAGTCGTTCGCGTGGGTACAACTTTGGTCTTTAGGCTGGGCATCCTTCCCA
GACGTATTGGCAAGAGACGGGTTACGCTATGACTGTGCATCTGACAGAGTGTATATGAATGCTACGTTACAAGAA
AAGGCCAAAGATGCGAATAATCTCGAACATAGTTTGACTAAAGACGACATTAAACAGTATATCAAGGATTACATC

CATGCGGCTAAGAATTCTATCGCGGCTGGCGCCGATGGTGTAGAAATTCATAGCGCCAATGGGTACTTGTTGAAT
CAGTTCTTGGATCCACATTCTAATAAGAGGACCGACGAATACGGCGGAACGATCGAAAACAGGGCCCGCTTTACA
CTGGAGGTTGTCGATGCTCTTATCGAAACTATCGGTCCTGAACGGGTGGGTTTGAGGTTGTCGCCGTACGGCACT
TTTAACAGTATGTCTGGGGGTGCTGAACCAGGTATTATCGCTCAATATTCGTATGTTTTGGGTGAATTAGAGAAG
AGGGCAAAGGCTGGTAAGCGTTTGGCCTTTGTGCACCTCGTTGAACCACGTGTCACGGACCCATCGTTGGTGGAG
GGCGAAGGAGAATATTCCGAGGGTACTAACGATTTTGCCTACTCTATATGGAAGGGTCCAATCATCAGAGCTGGT
AATTACGCTCTTCATCCAGAAGTGGTTAGAGAACAAGTAAAGGATCCCAGAACCTTGATAGGCTATGGTAGATTC
TTCATCTCTAACCCAGATTTAGTCTACCGTTTAGAAGAGGGCCTGCCATTGAACAAGTATGACAGAAGTACCTTC
TACACCATGTCCGCGGAAGGTTATACCGACTACCCAACATATGAAGAGGCAGTAGATTTAGGTTGGAACAAGAAC
TGA

>YOR332W 2.15   Hot
ATGTCCTCCGCTATTACTGCTTTGACACCAAACCAAGTGAACGATGAATTGAACAAGATGCAAGCTTTCATCAGA
AAGGAAGCTGAAGAAAAAGCGAAGGAAATCCAATTGAAGGCTGACCAAGAGTACGAAATCGAGAAGACCAACATC
GTAAGAAATGAAACCAACAACATTGACGGCAACTTCAAGAGCAAGTTGAAGAAGGCCATGCTTTCGCAACAGATT
ACTAAGTCAACGATAGCAAACAAAATGAGGTTGAAGGTTCTTTCTGCTCGTGAACAGTCGCTAGACGGGATATTC
GAGGAAACCAAGGAAAAGTTGTCAGGAATTGCCAACAACCGGGACGAGTACAAGCCCATTTTGCAATCATTGATC
GTGGAGGCACTTTTGAAGTTGTTGGAACCTAAGGCGATTGTCAAGGCTCTTGAAAGAGACGTCGATTTAATCGAG
TCGATGAAGGACGACATTATGCGTGAGTATGGGGAAAAGGCCCAGCGCGCACCATTGGAAGAGATTGTTATCTCC
AATGATTACTTGAACAAAGACCTTGTTTCCGGCGGTGTTGTGGTCTCCAATGCATCAGACAAGATTGAAATTAAC
AACACTTTGGAGGAAAGATTGAAATTGTTAAGTGAAGAGGCATTGCCCGCCATCAGATTGGAATTGTATGGTCCT
TCCAAGACAAGAAAGTTCTTTGATTGA

>YJL211C 1.54   Hot
ATGTGTGATCTTCTTCCACACGTACCCTGCCAAGACAGTGACGACATACAAGGTGCGTTTCCTGCACTGAAAAAC
CACGCCACTGAGTGCGGATCCATACGTACAGCTAGAGCCATAGTGTGGCAGGAACCTCGTCGCGCACATAAACAC
AACTGTGTTCAGTGCCAACTCCCATTCCTCTTTGTGTTCACTCGTATTGAATGCTGCATTGAACTCGGACCAAAA
CTGTCCATACAATTCTTTATCAAGCGCTATCGAGTCGAGCTGTGCAACTCGAGACATGCTGCGTTGCCTTTAGAT
TACGTCTTCTCAGTTATCTCGGCAATCTCTCCTCGTTATGCTTTCCCTCCGTCTTCACTCATCCCTTATAATGCG
TTTCCCAGCAACCTTATCGATAGAATGGAGGGGAAATCGCATCCGTTCCACTCCTCGGATCGATACTAA

>YGL200C 2.2 Hot
ATGGCCTCATTTGCTACTAAGTTTGTCATTGCTTGCTTCCTGTTCTTCTCGGCGTCCGCCCATAATGTCCTTCTT
CCAGCTTATGGCCGTAGATGCTTCTTCGAAGACTTGAGTAAGGGTGACGAGCTCTCCATTTCGTTCCAGTTCGGT
GATAGAAACCCTCAATCCAGTAGCCAGCTGACTGGTGACTTTATCATCTACGGGCCGGAAAGACATGAAGTTTTG
AAAACGGTTAGGGACACGTCGCACGGTGAAATCACATTGTCTGCTCCATACAAGGGACATTTCCAATACTGTTTC
TTGAACGAGAACACCGGTATCGAGACAAAAGACGTGACTTTCAACATTCATGGGGTGGTATATGTGGATTTGGAC
GACCCAAACACCAATACATTGGATAGTGCTGTCAGAAAGTTGTCCAAATTAACCAGGGAAGTCAAGGACGAACAA
AGTTATATTGTTATTAGAGAAAGGACGCATAGAAATACTGCAGAGTCGACCAACGATCGTGTTAAATGGTGGTCC
ATCTTCCAATTGGGCGTCGTTATTGCAAACTCTCTTTTCCAGATATACTACTTGAGAAGATTCTTTGAGGTCACA
TCTCTAGTCTAA

>YCR061W 1.96   Hot
ATGGTGCGTTTTGTTTCAATTTTAAGTTTATTCGGCTGCGCGGCGACGCTTGTCACGGCCCATGATGACATGGAC
ATGGACATGGATATGGACATGGATATGGACATGAATATCGATACGACAACGTCTCAATCCATAGATGTCTCATCC
ACGGCTTCAATCGTCCCCGTGCCACATGAACCAAAACATTTGCATGGCCTTCCTATACTGCAATCGCCCTCGCTT
ACCCCTGCGGAGAGATTGTACTGGGAAAACTACAACACCACAACCTACTTTACTACACAGGCTGGGAATAGGTCT
GCCCTTCGCTACCACATTATTACGCTGCTCTTGGTTGCATTTGTGCTCTACCCTGTGTCCCTGGCGCTAAGCGCC
GCCCGTTCTAGGTGGTACTTACCCCTGCTGTTTGTTAATCTATGCATTTGTATTTCGTCCGTAATGGCATTGTCC
GTGTTCAAAAATACTTTCCCGGAAGAAGACTGGTATGCGCATAATATCTATGGCACCACTTCTGTGCTACTTCTC
GTTTTTATGCTTGTTCACTTCTTCGCTGCGGTGCTTTCTGTCCCCGTCTCATTAGCATCGAAAAAGGAGTACCGT
CCGGTTGACACCATCCCTCTGAATGATCTTGAATCTACGCCCGTCATGGTGAATAGTGCACGTGGCTCTCCAAGT
CCTTCTTCCAACAGAGACACGTTGTTCTCGCTCTCTTCAGACACCACGACCGCCACGGCCACCAATAATAATAAA
CGGAGACGCGCTGAAGGCGAAGACGAGGGTGATAACACCTCCAACCACGACACTTTGCGCGACGAAGACTACGAT
AATGATGACGACGAAATTGCTTCCATTGAAGCGCCACCTCTGCTTCCTCAAGACATACCCGTTTTCCGAATCTTG
TTTACCAACACGAAGTACCAGATGCTTGCCGCGCACCTCTCGTGCGTCGCCAACGTGGTCTTTCACATGCTTACC
TACCCGCTATTCATGTACATCTTTGTAGACCTAATCATCGGCTTCGCTGTAGGTAACTTGCTCGGCAAGGGCATC
CGCATCTTTAATCTCTTGGCCCACTGGATTAAGGGCGGCGTATTTTTTACTCTGGGCGTTGTCTCTTTAGCAAGA
TACTGCGGTTTCGCAGCTAAGTACGGCTGGGCATGGAACAACATCAGCTTCACCTCTCAACTCACACAAACGCGT
TCCTCCAATCTTCTTTTCCGGTTTGCTCCTGCGGGGACTTTCACCATGGAATTCGTTGAATCCTTCCTCATTTTC
TTTTACGGGTCCACCAACATCTTCTTGGAGCACCTGGCAGGAAACGGCGGCGCATGGACTGCCAAGGATTTACAG
CATGTGTCGATCGCGTTTATGTTCATAGGAACCGGATTGTGTGGGCTACTCACGGAGTACAAGCTCAACCATTGG
CGATTCGAGCATGCCCGCAAACGGCCACAGACCGATGTAGTTGCTGCCACACCGGGGTACTCTCCAAACCCGTTC
CCCGCTTTCACCATATTTTGGACTGGGATTCTGATGTCCCAGCACGCACAGTCCTCGCAATTTTCTACTACCATT
CACACGCAATGGGGATACTTGTTGTCCTATGGGTCCTTCTTCCGTCTGCTAACATTTTTGATTCTGTTTTTGGTG
CCCAACACCAACAGTGCCGCATCCAAGCCTTTCACGGAGTTGATCACCTCGTTCTGTCTCCTCTGTGGTGGTCTG
GTATTTATGGAGTCCACGGATCAGTCCATTGAAGCCATGGAATATAGGGGGTTTACCCCCATGTTCACTTTCAAC

CTCAGTGTTGGATTCGTTTCTTTGTTGATGGCTTGGGAAATGATTTATTTATTTGGAAAGACTGGCTCATCAAA
ACCAGGAAAACCAGTCTTTAA

>YCR048W 1.95 Hot
ATGACGGAGACTAAGGATTTGTTGCAAGACGAAGAGTTTCTTAAGATCCGCAGACTCAATTCCGCAGAAGCCAAC
AAACGGCATTCGGTCACGTACGATAACGTGATCCTGCCACAGGAGTCCATGGAGGTTTCGCCACGGTCGTCTACC
ACGTCGCTGGTGGAGCCAGTGGAGTCGACTGAAGGAGTGGAGTCGACTGAGGCGGAACGTGTGGCAGGGAAGCAG
GAGCAGGAGGAGGAGTACCCTGTGGACGCCCACATGCAAAAGTACCTTTCACACCTGAAGAGCAAGTCTCGGTCG
AGGTTCCACCGAAAGGATGCTAGCAAGTATGTGTCGTTTTTTGGGGACGTGAGTTTTGATCCTCGCCCCACGCTC
CTGGACAGCGCCATCAACGTGCCCTTCCAGACGACTTTCAAAGGTCCGGTGCTGGAGAAACAGCTCAAAAATTTA
CAGTTGACAAAGACCAAGACCAAGGCCACGGTGAAGACTACGGTGAAGACTACGGAGAAAACGGACAAGGCAGAT
GCCCCCCCAGGAGAAAAACTGGAGTCGAACTTTTCAGGGATCTACGTGTTCGCATGGATGTTCTTGGGCTGGATA
GCCATCAGGTGCTGCACAGATTACTATGCGTCGTACGGCAGTGCATGGAATAAGCTGGAAATCGTGCAGTACATG
ACAACGGACTTGTTCACGATCGCAATGTTGGACTTGGCAATGTTCCTGTGCACTTTCTTCGTGGTTTTCGTGCAC
TGGCTGGTGAAAAAGCGGATCATCAACTGGAAGTGGACTGGGTTCGTTGCAGTGAGCATCTTCGAGTTGGCTTTC
ATCCCCGTGACGTTCCCCATTTACGTCTACTACTTTGATTTCAACTGGGTCACGAGAATCTTCCTGTTCCTGCAC
TCCGTGGTGTTTGTTATGAAGAGCCACTCGTTTGCCTTTTACAACGGGTATCTTTGGGACATAAAGCAGGAACTC
GAGTACTCTTCCAAACAGTTGCAAAAATACAAGGAATCTTTGTCCCCAGAGACCCGCGAGATTCTGCAAAAAAGT
TGCGACTTTTGCCTTTTCGAATTGAACTACCAGACCAAGGATAACGACTTCCCCAACAACATCAGTTGCAGCAAT
TTCTTCATGTTCTGTTTGTTCCCCGTCCTCGTGTACCAGATCAACTACCCAAGAACGTCGCGCATCAGATGGAGG
TATGTGTTGGAGAAGGTGTGCGCCATCATTGGCACCATCTTCCTCATGATGGTCACGGCACAGTTCTTCATGCAC
CCGGTGGCCATGCGCTGTATCCAGTTCCACAACACGCCCACCTTCGGCGGCTGGATCCCCGCCACGCAAGAGTGG
TTCCACCTGCTCTTCGACATGATTCCGGGCTTCACTGTTCTGTACATGCTCACGTTTTACATGATATGGGACGCT
TTATTGAATTGCGTGGCGGAGTTGACCAGGTTTGCGGACAGATATTTCTACGGCGACTGGTGGAATTGCGTTTCG
TTTGAAGAGTTTAGCAGAATCTGGAACGTCCCCGTTCACAAATTTTTACTAAGACACGTGTACCACAGCTCCATG
GGCGCATTGCATTTGAGCAAGAGCCAAGCTACATTATTTACTTTTTTCTTGAGTGCCGTGTTCCACGAAATGGCC
ATGTTCGCCATTTTCAGAAGGGTTAGAGGATATCTGTTCATGTTCCAACTGTCGCAGTTTGTGTGGACTGCTTTG
AGCAACACCAAGTTTCTACGGGCAAGACCGCAGTTGTCCAACGTTGTCTTTTCGTTTGGTGTCTGTTCAGGGCCC
AGTATCATTATGACGTTGTACCTGACCTTATGA

>YOR179C 1.89 Hot
ATGGATTTACCCAAGGACAAAAGTGACAGAACTCATCAAAGAATTAATCTAAATAACAGTGGGACAGATCGAACT
AATGATTTGTACCTTCATATTGTCCAAACGTTCGGTTGCATAGAAACAACTGCAACGGAAAATGCCACGAAACTG
TTAATGCTGGGTGACGTCGAAGTAGAAATATCTGCGAGCAGCGTTTCAATTGAGTGGACACAGAAGTCAATGATA
AGCCAAACAATTGCCGATAGTATAGTAATAATGATCATCGGTTTGTGTGCAAGCGACAAGAACGTGCTATCTGAA
TCAGAATTGAAAGAGAGAAACCATAACGTTTGGAAGATCCAAGAATTGCAAAATCTGTTTCGAGAACAATTTGGA
GACAGTTTTAGCATCGATGAAGGAATAGGAAAAAAAGAAAATGTAAAGAATGGTAGCGTCACCATAGGCAAGAGT
AAAGCCACGATCGATTTCTCCACCATGAAGCTGATTGATTGTAATTCGAACCCACTAAAGGGAAGAGTGGAGAGC
ATACTAAGCATTGGCCAGAAATTAACAACTCCATTGTGCTGA

>YEL057C 1.67 Hot
ATGGCAAATGATGGGATTCAGAGGAATGACAACAGAAAGGGTTTTAAAACTGTACAGTTCTCGGCTTATTCTAAA
GAAATTGACGTCATAATGAAAAAAATATCATTCTTGGAAAGAAACATTACCCAACAACTAGATACTCTTCCTCAT
TTTCCAAAAACCTTACCACCAAATCACAAAGATTGTGTAAGTAGAAAGCACAGGGCCAGGCGAGGTTGGAGTAGT
CAGCTGAAAAACTTGCTAGGCATTTACTCCAAAGAGGAAATTTTTACTCTGGATAATTTAGCTGCAACACTGCAT
GATCAAGTTTTAAAGTTACAAGCTACACTTTTTCCCAATGCCATACTGAAACAGGTTCATCTCGACAATGCCAAC
ATAGAAAACAAGAGAATTCTGAAGGAAATTACATATAAATACCTTTCCAACGAAAACTGTAAAGAGGAGAACAAA
TTCGGAACATTCATCGTAAAGAGAATTTTTTTGGTGACTTATCACTCGGGGTTTCCGTGTTAATCAACCGTATT
GCTTTCGAATCGGCCACATCATCCATTATGGTAGTGAGAAGTTCATTTATTGAAAGTGATTTCTTTTATGAAGAT
TATTTGATTTTCGATTGCAGAGCGAAACGACGAAAAAAATTGAAGAGGAAAATTTTGTTTATTTCCACTACCATG
AATTTCAATTACCAAACCAAAGTCTAA

>YBR127C 2.24 Hot
ATGGTTTTGTCTGATAAGGAGTTGTTTGCCATAAATAAGAAAGCCGTCGAACAAGGTTTCAATGTGAAGCCTAGA
TTGAACTATAATACGGTCAGTGGTGTGAACGGTCCATTAGTCATTTTGGAAAAGGTCAAGTTCCCACGTTACAAC
GAAATTGTTAATTTGACATTGCCAGATGGAACCGTGAGACAAGGTCAAGTTTTGGAAATTAGAGGAGATAGAGCC
ATTGTGCAAGTGTTTGAAGGTACATCTGGTATTGATGTCAAGAAGACTACCGTGGAATTCACTGGTGAGAGTTTG
AGAATTCCTGTGTCTGAAGACATGTTGGGTAGAATTTTTGACGGTTCTGGTAGACCCATTGACAACGGTCCTAAA
GTTTTCGCAGAGGATTACTTGGACATTAACGGTTCTCCTATCAACCCATATGCTCGTATTTATCCAGAAGAAATG
ATTTCTACTGGTGTTTCTGCTATTGACACAATGAACTCCATTGCCAGAGGTCAAAAGATCCCAATTTTCTCCGCA
TCAGGTTTACCACACAACGAAATTGCAGCACAAATTTGTAGACAGGCTGGTTTGGTGAGACCTACCAAGGATGTT
CATGATGGTCATGAAGAAAATTTCTCCATCGTTTTTGCTGCCATGGGTGTCAACTTGGAAACCGCTAGATTTTTC
AAACAGGATTTCGAAGAAAATGGGTCTTTGGAAAGAACTTCATTATTTTTGAACTTGGCTAATGACCCTACCATT
GAAAGAATTATCACTCCAAGATTGGCCTTGACCACCGCTGAATACCTTGCTTACCAAACGGAACGTCATGTGTTG
ACCATCTTGACCGATATGTCATCGTATGCTGATGCTCTTAGAGAAGTTTCCGCTGCTAGAGAAGAAGTTCCAGGT
AGAAGAGGTTATCCTGGTTACATGTATACAGATTTGTCCACAATTTATGAAAGAGCAGGTAGAGTAGAGGGTCGT
AACGGGTCCATCACTCAAATACCTATCTTGACAATGCCTAACGATGATATTACGCATCCAATTCCGGATTTGACC

GGTTATATTACCGAGGGTCAAATCTTCGTTGACCGTCAATTACATAACAAGGGTATCTACCCACCAATCAACGTC
TTGCCTTCGTTGAGTAGATTGATGAAATCTGCCATCGGTGAAGGTATGACCAGAAAGGACCACGGTGACGTTTCT
AACCAATTGTATGCCAAGTACGCCATCGGTAAGGACGCTGCTGCTATGAAGGCCGTTGTCGGTGAAGAGGCGTTA
TCCATCGAAGATAAGTTATCTTTGGAATTTTTGGAAAAATTCGAAAAGACCTTTATCACACAAGGCGCCTACGAG
GACAGAACCGTTTTCGAAAGTTTGGACCAGGCATGGAGTTTGCTAAGAATCTACCCTAAGGAGATGTTGAATAGA
ATCTCCCCAAAGATTCTTGATGAATTTTACGATAGAGCCAGAGACGATGCCGACGAAGATGAAGAAGATCCCGAC
ACAAGAAGCTCCGGTAAGAAGAAGGACGCCAGCCAAGAAGAATCTCTAATCTAA

>YCR051W 2.08   Hot
ATGAACGCTAATATATGGGTGGCTGCTTCAGATGGTAATTTGGACCGAGTGGAACATATCCTCCGCGAGAGTAAA
GGCGCCATGACCCCGCAATCCAAGGACATTAACGGCTACACTCCAATGCATGCTGCCGCCGCATACGGCCACCTG
GATTTGCTGAAGAAAATGTGCAATGAGTACAATGGAGACATTAATGTGTTGGACAACGACGGCGATACCCCGTTG
CACCATGTGGAGGATGTGGCCACTGCCAGGTTGATCGTGGAAGAGCTGGGTGGAGACTTCACTATCAGGAATGTG
GAGGGCCAAACGCCATACGACTCGTTCGTCGAGAACGGTGAAGATGGTGAGCTAATCGAGTACATGAGGATTAAG
TCCGGCGTGGCCGATGTTCACGGAGTGGACGGCGTGCAGGGTGAGGGTGTCATCGACAGCAAATTGCTGGAAGAG
TTCAAGGACAACGTGAGATACACCTTGGAAAATGACCCTGAGGAAGGAGCCGATGAGGCCACTCTGCAACGCAGG
AGGCAGTTGGAACAGATCATTACGGGAGACAACGCTGAGGAGGAGTTGGAAAGGTACATCCGTGCTATGGTCAGA
GAGCAGATGCTGGGCCAGGGCTCCATGGCGGGTTCCGGGGACGAACCAGATTCCAAGAGAAGAAAATAA

>YGR201C 1.52   Hot
ATGTCTGACGGAACACTGTTTACTGATTTGAAGGAGAGAAAGTTGATTAGGACCATTGTGCCAAGAGGTTTAGTG
CGTTCCTTGAAGCTGGACGTTAAGCTTGCTGATCCAAGTGACGCTCAGCAACTTTACGAAAGGGAGTTTCCCTTG
CGTAAGTACCCAACTTTTGTTGGACCGCATGACGAGTGGACGCTTACAGAGGCAATGGCTATTGACTACTACTTG
ATCCATCTGTCGAGCGACAAGGAAGCTGTGCGCCAGTTGTTGGGTCCCGAAGGTGACTTCAAAACTCGTGCAGAT
ATTTTAAGATGGGAATCGCTTTCCAACAGTGATTTCTTGAATGAGGTTTGTGAGGTGTTTTTCCCTCTAATTGGC
GTAAAGCCATATAATGCCACTGAATTCAAAGCCGCAAGAGAGAATGTCGACACCATTGTTTCGCTTTACGAGAAA
AGGTTAAAGAAACAGCAATACCTTGTCTGTGATGACCACGAAACTCTTGCTGATTTAATCTCAGCGGCTGCTTTT
TCGCTAGGGTTTATAAGTTTTTTCGATGAAACATGGAGGTCTAAGCATCCAGAGGTTACGCGCTGGTTTAATCGA
GTAATTAAGTCCCGCTTTTTTGAAGGTGAATTTGAGAGTTTCAAAATGTGTGAGACTGAGATGCAACCCATTAAA
TAA

>YFR023W 1.72   Hot
ATGTATTCTATTTCAAACAAAAAACCTTCTATACTCAGTATGGTTCCCCTGAATATTTTGAAAAACCAGGATTTG
AAAGTAAAAAAAGATCAAGAAAAGAAAATATCATTTAACCCCGTAGTTACTCCTATTAGGCCAGACGACTACCAC
GAAAAAACATCCAGATCGTCAAGTTCGAGCCATTCTGATTCACCTGAATTTTTGAGAATTAACAACAACAAATCC
GGTCACAAGAATGGAAAATTGAAAAGTTTTGAAAGTAAAAAACTAGTTCCGCTATTCATTGGTGACCTCCATGAA
ACAGTTACTGAAGAAACTTTAAAAGGGATCTTCAAAAAATATCCCTCTTTTGTCTCTGCTAAAGTTTGTCTTGAT
TCAGTGACAAAAAAATCACTGGGCCATGGTTACTTAAATTTTGAAGACAAAGAAGAAGCTGAAAAGGCTATGGAA
GAATTAAATTACACGAAGGTTAATGGTAAAGAAATTAGGATTATGCCATCATTGAGGAATACAACGTTTAGAAAG
AATTTCGGCACTAATGTCTTTTTCTCTAATCTACCTCTAAATAATCCATTATTAACAACAAGAGTATTCTATGAT
ACGTTTTCTAGATATGGTAAAATTCTATCATGCAAATTAGACTCTAGGAAAGATATAGGATTTGTTTACTTTGAG
GACGAAAAAACTGCGAGAAATGTGATCAAAATGTACAATAATACCAGTTTTTTTGGTAAGAAAATTCTATGTGGA
ATACATTTTGATAAAGAGGTCAGAAGTGTTCCAAATTTTGAAACACAAAAATCACGATTAGATGCGGAAACAATT
ATTGAAAAAGAGCAATCTTTGAATGAGAAACACTCCAAGGGGAATGATAAGGAATCTAAAAATATTTACTCTTCA
TCCCAAAATTCTATATTCATCAAAAACCTTCCAACAATAACTACAAGGGATGATATTTTAAATTTCTTCAGTGAA
GTGGGTCCAATTAAGTCAATTTATCTATCCAATGCCACTAAAGTTAAATATCTTTGGGCATTTGTTACATATAAA
AATAGCAGTGACTCAGAAAAGGCAATTAAACGCTACAATAATTTTTATTTCAGAGGTAAAAAGCTTTTAGTAACC
AGGGCACAGGATAAAGAAGAAAGAGCCAAATTTATAGAATCTCAAAAAATATCTACACTTTTCCTGGAAAATTTA
AGCGCTGTCTGCAATAAGGAATTTCTTAAATATTTATGTCACCAAGAAAATATTAGACCGTTCAAGATTCAGATA
GATGGATATGACGAAAATTCTTCTACATATTCTGGGTTCATCAAATTCAGGAATTTTGAAGATGCTACGAGAATA
TTTAACTTTTTGAATAACCGTTTAGTAGGAGGAAGCATCGTCACGACATCTTGGGAAAGACAAAATAATGCACCT
AAATATCATGATGGTTATGGAATGCGCAATATACACACCTCTTCTCATCCACAAATTACCCCATATTACCAGTAC
TCACATGCGAATAGTTTGAACTCCCCACACATGAGAGATCTTTCGTCAATGAATAGCAGCACAAGATCACTAATA
AAGAATAAAAATTTCAATAAGAAAGTTTTGGAAACATTCGAAAAGCAAGTAAGAAGAGGTATAGATTTCATGAGA
TTTCCAAGTGCCACTAGAGACGAAAATGTGCACGGAATAGCTGAGTATATATTCGACACTTATTGGAATCGCGAT
GTGTTAATATTGGACAAATTTTTATCGTTGCTAAATAGTAGTCCATATCATGAAGGTGTATTGCAAAAACAAATT
GAAGAGGCTGCAAGCTCGTTAGGATTTAAAAGATGA

>YBL093C 1.92   Hot
ATGGCTTCTAGAGTGGACGAAACTACAGTCCCCTCATACTACTATTACGTGGATCCGGAAACTACATATACGTAC
CAACAACCAAATCCTCTACAGGACTTGATATCGGTGTATGGCTTGGATGACATCTCCAGGCAAGTGGCAAGAACA
AATTTGGACGGCACTAAAGCCGTGAAGCTAAGAAAATCTTACAAGAACCAGATAGCAGATCTTTCAGGTAAATTC
TCCACCATACCGACCAGAGAAAATGGTAAAGGTGGTCAAATAGCACATATTCTTTTCCAAAATAACCCAGACATG
ATGATACAACCACCTCAGCAGGGTCAAAACATGTCAGAGCAACAATGGCGCGAACAGCTGCGCAATAGAGACATA
GCATTGTTTCAGCCTCCAAATTTCGATTGGGACCTTTGCTCTTCGGTACTATCGCAGTTTGAAAGGTCATATCCA
AGCGAGTTCGCAAACCAGAACCAAGGGGGAGCCCAAGCGCCGTTTGATATAGACGACTTGGCGTTTGATCTAGAC

GGTACAGGAAAAAGCCAATCCGGCTCAAATTCAGGTAACAATAGTAAGAAAAGGAAGAACAAATCTAGTGGAAGT
TCGATGGCTACACCAACACATAGTGACAGTCATGAGGATATGAAAAGAAGGAGGCTGGAGTAG

>YDL183C 1.69 Hot
ATGATACGTTCAATATTTATACCGCCAACTAGTATAACTGCCACTTCTCGACTGTTCTACGGAATGAGGGCGTAT
TCAACAAAACTTGAAAAGGCTTCACTCCAAAAATATCTGCATGATCCTGTTAAAGTGACTGTGATACCAATAACG
GACAAAGAATCATTCATTTACTATAAGCATACCGATAATCTCTTTAATAGTCAGTCTAGAATATTAAAAGCAGAA
AAGTGGATCGTTGAGAAATCAGCAAAGCTTTGGAGAAAACTGAAAAAGTCCCCTAAATCGTATAATAAAAAAATA
GTGTCCATGGTGCAGTCTCTGTTAAATAGCACACCCTGGTCAGAAAATAGTCTGTTAACCATACCAAGTGAAAGC
TATATATTGAAGAGAATTAAGGGAGAGAAAGATAAGACCCAAGAGATTCGACTCACATTGAAAGATTATACCGTA
AAGGCAGAACAGGTCGATACTCAACCCTTACACGTGTATTATCCCCCTGGAATTTCTAGCCCTGATGAGTGCTTG
AGACAAATGAAAAAGCTCTACCAAGAAGGCTTAATTTATCACAAAAAATGGACTTTATATTGCCTTTTAGGTCTC
CCGCTGACAATTCCTCTAATTTTAATACCGCTTATACCAAATGTTCCAGGGTTCTACCTATCTTATAGAGCTTAT
GTTAATATCAAAGCTTACCTTGGTGCTAAGCACTTGAAGAGTCTACTGGAAAGTTCAAAACAAAATTTAGAATTC
AGGGAACTGTTGGGATATACGGAAGTATATAAACGTGGGACCAGCAGCCGTACGCAGGGCAATCAAAAGGAATCA
AAAGGTGCACCAGAATTACTACTTAACAAGAAAACCCTTCCGTTGATCTTAGATTTCTTAGAGGTTCATGAACTA
GAAAGCGACTTGAACAAAGTCATCCTTCAAGAATCGAAATCTCAAGAGAAAAACAAAATTTAA

>YNL304W 1.55 Hot
ATGTCCCAGAGAAAGCGATACTCATTGAACGTCGTCACGTCCCCGTCGATACCGTCACCGACACCCAGTGCACCG
ATACGGACTAACGAATCCAATTGGGAAGCTGCTTCGCCAGCGAGTGCTGCATCTTCATTTCTCCCGAATGTTCAT
CATGGTGGCACGGTTTTGAATCCTGGGTTGGGCATAATGAGGTCACCTTCGTTGAACAAGTCTGGTGCGTTTGGC
CGGTCCGGCAGCAGTGGTTCCAGTACAGTAATAGAACCGTCCAACATAAAGTTGCTGCTAATTGGAGATGCAAAC
GTAGGGAAGACAGCTATGATTTTGAGCTATTGCCGGGAACTGTTGACACGAGCGGAAATGTCACGATCGGCGCGA
CTGAGGCACCAGCAGCAGCAACAGCATAAAGACTTAGGATTGAAGAAAACTGTGGTGAACCATAGATTAAGCATG
AAAGAGAAAAGAAAACGGTACAGCTCTAACGATTTTGAAAAGGAGTTCAAAGATATTAATCATTTTGCAGACGAA
ACAAGCGATTTCGGAAACCCGAACATCGGTGATGATAACAACCACGAAATGGCGGATCCTAATGAGATTGTCATC
GAAACCAGAAGTACTATCGGAATAGACATCAAGACGAACTTGGTCAACATAGATAACCGGTTTTTAATGTCATA
CTGTGGGACACTGCGGGACAAGAACGGTACCAAAACGCAATCATTCCATCACTGTACAAGAAGACTAATGCGGTG
ATATTAACTTACGATATAACAAATGCGAAGTCCTTCCAAAGCTGTATGGAGCGGTGGATAGTTCAGGCGTTGGAA
AACTTTTCTTCCCAGGATTTGTTAAAAGCAAGATTTTTTCTGGTGGGTAACAAGATTGACCTTTATAAGGAAAGG
CAGGTAACCCATTATGATGTGGTGCAAATGGTTCAGGAAATGCAACTGAAACATGGTATTAAAATTTCGGGAAAC
TTTGAAGTAAGCTGTAAATGGGTCAACGTGGTGGAGAGAACCATGAATATGATAATACTAGACCTGGTTGAGAAC
GGATGTTTCGAAAACAATGATCCTTGTGTGTCGATTACAACATCTGACGACGTCCAGGGACATGAGCAAGAATTC
CACGATACAGTAGAAGAGCCTTTCAATTTCACTCGGCAACGTCAACACCAACTTGAAAAAAATAACACAGTGGAT
ATTACAAAACCTAATGACGACATTGCAAATAATCAATCAATTTGTTGCGTTTAG

>YGR208W 1.77 Hot
ATGTCAAAGTTTGTTATCACCTGCATAGCTCATGGAGAAAATCTCCCAAAAGAAACCATCGACCAGATTGCGAAA
GAAATTACTGAAAGCTCGGCGAAAGATGTATCAATCAATGGTACCAAGAAACTATCGGCCAGAGCTACCGATATA
TTCATTGAAGTCGCAGGATCGATTGTTCAAAAAGACCTCAAGAATAAGCTGACGAACGTCATTGACAGTCATAAT
GATGTTGATGTTATTGTTTCTGTCGACAATGAATATCGTCAAGCCAAAAAGCTCTTTGTCTTTGACATGGATTCA
ACATTAATCTACCAAGAGGTCATCGAATTGATTGCCGCTTATGCTGGTGTTGAAGAACAAGTGCACGAGATCACA
GAAAGAGCCATGAACAATGAACTTGATTTCAAAGAGTCTTTAAGAGAACGCGTTAAATTATTGCAAGGCCTCCAA
GTCGATACACTATATGATGAAATAAAACAAAAGCTAGAGGTCACCAAGGGTGTGCCAGAACTATGCAAGTTCCTT
CACAAAAAAAATTGCAAGCTCGCTGTTTTAAGCGGTGGTTTTATTCAGTTTGCCGGTTTTATCAAGGATCAGTTA
GGTTTAGATTTTTGTAAGGCAAACTTGTTGGAAGTTGACACTGATGGAAAATTAACCGGTAAAACACTGGGTCCT
ATCGTAGACGGACAGTGCAAGAGTGAAACCCTTCTGCAACTATGTAATGACTACAATGTTCCAGTTGAAGCAAGT
TGTATGGTGGGTGACGGTGGTAACGACCTGCCAGCCATGGCTACCGCCGGGTTTGGGATCGCATGGAACGCCAAG
CCAAAGGTGCAGAAAGCTGCACCTTGTAAGTTGAATACCAAGAGCATGACTGACATTTTATACATTCTTGGTTAC
ACCGATGATGAAATATACAATAGACAATGA

>YLR297W 1.69 Hot
ATGGTCGAAGGTGATTTTGTCGATGAGCAATCGAACATTGCCTTACTAAGCTCCAAATCCATGTGTGGTGATCAT
CATAGCGTCAAGAATAGCATAGGGGATGAGATCTTTAAATTATTGACAAAGATTTTAAATTCGGATGAGAAAGCA
AGCGGAGATGTGCACACACTCGTTTCTGGGACCCCGGATCTCTCAAATTTTAACCTCGATAATGAACCACTGGAA
AACATACTCGCTGTTTTCATAATATCTTTTATTATTGTAGTGGTTGGAGTTTTACTACTTGGGTTGATTGGCATG
ATATTCATATCTCTCCGTTCAGGCAGCAGTAATGATAAGAAACTACAGTCCAACGATGAAGAAAAGCAAGCACTT
GCTGAAAAAGCATGA

>YJL088W 1.89 Hot
ATGTCAACCACAGCATCCACGCCTTCATCTTTACGTCATTTGATTTCTATAAAAGATCTTTCTGATGAAGAATTC
AGAATCTTAGTACAAAGAGCTCAACATTTCAAGAATGTTTTAAAGCAAATAAAACGAATGATTTCCAATCCAAC
CATCTGAAACTATTGGGTAGAACTATAGCCTTAATATTTACTAAAAGATCAACTAGAACGAGAATTTCGACCGAA
GGTGCAGCCACCTTCTTTGGTGCCCAACCGATGTTTTTAGGTAAAGAGGATATTCAGCTTGGTGTCAATGAATCA
TTTTACGATACCACCAAGGTTGTATCATCTATGGTTTCATGTATTTTTGCCCGTGTGAACAAACATGAAGACATA
CTTGCTTTTTGCAAGGATTCCTCTGTACCGATCATCAACTCTCTATGTGACAAATTCCACCCTTTGCAAGCAATT

TGTGATCTTTTAACAATAATCGAAAACTTCAATATATCTCTAGATGAAGTAAATAAGGGAATCAATTCAAAATTG
AAGATGGCATGGATTGGTGATGCCAATAATGTCATAAATGATATGTGCATCGCATGTCTGAAATTCGGTATAAGT
GTCAGTATTTCCACTCCCCCCGGTATTGAAATGGATTCCGATATTGTCGATGAAGCAAAGAAAGTTGCTGAGAGA
AACGGTGCGACATTTGAATTAACACACGACTCTTTAAAGGCCTCCACCAATGCCAATATATTAGTAACCGATACT
TTCGTTTCCATGGGTGAAGAATTTGCGAAACAGGCCAAGCTGAAACAATTCAAAGGTTTTCAAATCAATCAAGAA
CTTGTCTCTGTGGCTGATCCAAACTACAAATTTATGCATTGTCTGCCAAGACATCAAGAAGAAGTTAGTGATGAT
GTCTTTTATGGAGAGCATTCCATAGTCTTTGAAGAAGCAGAAAACAGATTATATGCAGCTATGTCTGCCATTGAT
ATCTTTGTTAATAATAAAGGTAATTTCAAGGACTTGAAATAA

>YJL069C 1.58 Hot
ATGACAATGGCAACAACCGCAATGAATGTTAGCGTACCTCCACCAGATGAAGAAGAACAGTTACTAGCCAAATTT
GTATTCGGTGATACAACAGATTTACAAGAAAATCTCGCTAAATTTAATGCCGATTTCATTTTCAATGAACAAGAA
ATGGATGTTGAAGACCAGGAGGATGAGGGATCTGAATCAGATAACAGTGAAGAAGATGAAGCGCAGAACGGTGAA
TTAGACCATGTTAACAACGATCAGTTATTTTTTGTGGACGATGGTGGTAACGAAGATAGTCAAGATAAGAATGAA
GATACCATGGACGTGGATGACGAAGATGACTCTTCAAGCGATGATTATTCTGAGGACTCTGAAGAAGCTGCTTGG
ATAGATTCGGATGATGAAAAAAATTAAAGTGCCTATTCTTGTTACCAATAAGACAAAGAAATTAAGAACCTCCTAT
AACGAGTCCAAAATCAACGGTGTTCATTACATCAACAGATTGAGATCTCAGTTTGAAAAGATATATCCAAGGCCC
AAATGGGTGGACGATGAATCTGATTCTGAATTAGATGACGAAGAAGACGATGAAGAAGAAGGCTCTAATAATGTT
ATTAATGGAGATATTAATGCTCTAACGAAGATCCTGTCTACTACTTACAATTATAAAGACACTTTATCGAATTCC
AAACTATTACCCCCTAAGAAACTTGACATTGTGCGTCTTAAAGATGCAAATGCATCACACCCGTCTCATTCCGCC
ATACAATCTCTTTCATTCCATCCTTCTAAGCCTCTACTGTTAACGGGTGGGTATGATAAGACCTTAAGAATATAT
CATATTGACGGGAAAACGAACCATCTAGTGACAAGTTTGCACCTGGTTGGGTCTCCAATACAAACATGTACTTTC
TATACTTCTCTATCAAACCAAAATCAACAAAATATATTCACCGCCGGTAGAAGAAGATATATGCATTCCTGGGAC
TTATCCTTAGAAAATTTAACTCATTCACAGACTGCCAAAATCGAAAAAATTTTCCAGATTGTATGGACATGAGTCC
ACTCAAAGATCATTTGAGAATTTCAAAGTTGCACATTTGCAGAATTCACAAACAAATTCTGTCCATGGAATTGTC
CTTTTACAGGGTAATAATGGTTGGATTAACATTTTACATTCCACTTCCGGACTATGGTTAATGGGCTGCAAGATA
GAAGGTGTGATAACGGATTTCTGTATCGATTATCAACCTATTTCACGTGGTAAGTTTAGGACCATTTTAATTGCT
GTAAACGCGTATGGTGAAGTGTGGGAATTTGACTTGAATAAGAATGGCCATGTTATAAGAAGATGGAAAGACCAA
GGTGGCGTGGGTATAACAAAAATCCAAGTTGGTGGTGGGACAACAACTACTTGTCCGGCTCTTCAAATAAGTAAG
ATAAAGCAAAATAGGTGGCTTGCGGTAGGTAGTGAGAGTGGATTTGTTAATCTTTACGATAGAAATAATGCTATG
ACCTCGTCGACCCCTACACCTGTCGCCGCCTTGGACCAGCTAACAACTACCATTTCTAATCTACAATTTTCCCCT
GATGGTCAAATACTGTGCATGGCATCCCGTGCAGTCAAGGATGCTTTGAGACTAGTTCATCTGCCTTCTTGTAGC
GTGTTCAGCAACTGGCCTACCAGCGGGACGCCTTTGGGTAAAGTTACCAGTGTCGCATTTTCGCCATCTGGTGGG
CTACTGGCCGTGGGTAACGAACAAGGTAAAGTGAGGCTCTGGAAATTAAACCACTACTAA

>YFL068W 1.7Hot
ATGATGCCTGCTAAACTGCAGCTTGACGTACTGCGGACCCTGCAGTCCAGCGCTCGTCATGGAACGCAAACGCTG
AAAAACTCCAACTTTCTCGAGCGCTTCCACAAAGACCGTATCGTCTTTTGCCTCCCATTCTTCCCGGCACTTTTT
CTCGTCCCAGTTCAAAAAGTACTGCAGCACCTCTGTCTTCGATTCACGCAAGTTGCTCCATACTTTATAATACAA
CTCTTTGATCTGCCTTCCAGACATGCGGAAAACTTGGCTCCCTTGCTTGCCTCTTGTCGAATCCAATACACTAAT
TGTTTCTCTTCTTCTAGTAATGGCCAGGTACCAAGCATAATTTCTCTGTATCTGAGAGTAGATCTCTCCCCTTTT
TACGCTAAAAAATTTCAAATACCCTACAGGGTCCCCATGATATGGCTCGATGTCTTCCAAGTATTCTTTGTATTC
CTCGTCATTTCGCAGCATTCTCTCCACAGCTAG

>YDL169C 1.88 Hot
ATGGAGGATAAGGAGAAACTTATTGTATACTCAAATACCAGTTCGGTATTTACATATAGTGCAGAAATAAGGCCA
AATTTTAAAATATCTGTATCTCAAAGTCAAGGTTTTGCCTGGAACCAGGACTTATTCGCCACACAATACCAACAA
TCATATAAGGTGGTATATGATGCACACGAGGACAACTTTGATGAGTTAATTTTGAAGATCAAGGGCAAATTAAAG
ACAAAATCTAATAAAAGAGCTAAAATGAAGTCTAAGACTAAATTAACAAGGACTGCAAAGCAGAGAAGGGAAAGC
CCCGTTTGTGAAAGGGACGAGAGTGACGAGGATAATGATAGTGATCACTACCAAAGAATACAAGTGCTAGATGGC
CATGAATTTCCGAGAGCAAATCGGTATAAATCTGTATGGGCTCATGACGTCCACTCGAATGAAGATAGTACAAGC
GACGGGGAAAGCAATCATGATATAGATATGATTGGCGGCACTGGCACGTCGTATGCAGGAGCAGCCATAATGGAT
AGGCCACGCAGGAAATCTGAGAGAAGCATAAGCTTTGTGGAGGACTCCAAGACAGGCGATTACAGGTATCAAACC
GGGCAGGTGGATGTTGTAGAGGTGGACTCGGACACCCCTGAAAATAATCACCTAAAATGGTTAATTAAATAA

>YIL054W 1.85 Hot
ATGGCCCCGAAGGCTTTTTTTGTCTGTCTTCCTTGGGTCTTACCCCGCCACGCGTTAATAGTGAGACAAGCAGGA
AATCCGTATCATTTTCTCGCATACACGAACCCGCGTGCGCCTGGTAAATTGCAGGATTCTCATTGTCCGGTTTTC
TTTATGGGAATAATCATCATCACCATTATCACTGTTACTCTTGCGATCATCATCATTAACATAATTTTTTTAACG
CTGTTTGATGATGGTATGTGCTTTTATTGTTCCTTACTCACCTTTTCCTTTGTGTCTTTTAATTTTGACCATTTT
GACCATTTTGACCTTTGA

>YAL039C 1.88 Hot
ATGGGTTGGTTTTGGGCAGATCAAAAAACTACGGGCAAAGATATTGGTGGGGCAGCAGTATCATCCATGTCAGGG
TGCCCAGTCATGCACGAGTCGTCGTCGTCGTCGCCACCATCCTCTGAGTGCCCCGTTATGCAGGGAGATAACGAT
AGAATAAACCCGCTGAACAATATGCCGGAGTTGGCAGCATCCAAACAGCCTGGCCAAAAGATGGACTTGCCCGTT
GATCGGACCATCTCCAGCATCCCCAAGAGTCCAGACAGTAACGAGTTCTGGGAGTATCCTTCTCCACAACAGATG

TACAATGCTATGGTTAGAAAGGGCAAGATTGGCGGTAGCGGCGAAGTCGCCGAAGATGCAGTGGAGTCCATGGTG
CAGGTCCACAACTTTCTAAATGAAGGGTGCTGGCAGGAAGTGCTCGAATGGGAAAAACCGCACACAGATGAAAGC
CACGTGCAGCCTAAGTTGCTGAAATTCATGGGGAAACCGGGCGTATTGAGCCCTCGTGCTCGCTGGATGCACCTG
TGCGGCCTACTGTTTCCGTCCCATTTTAGCCAAGAACTACCATTCGACAGGCACGACTGGATTGTACTCCGAGGC
GAGCGCAAAGCGGAACAACAACCTCCAACCTTCAAGGAAGTTAGATACGTCTTGGATTTCTACGGAGGGCCCGAC
GACGAAAACGGAATGCCTACTTTCCACGTGGATGTCCGTCCTGCCCTAGATAGTCTAGACAATGCTAAGGACCGG
ATGACCCGTTTCTTGGACCGGATGATCTCGGGTCCGTCCTCTTCGTCCTCCGCCCCTTAA

>YIL054W 1.85 Hot
ATGGCCCCGAAGGCTTTTTTTGTCTGTCTTCCTTGGGTCTTACCCCGCCACGCGTTAATAGTGAGACAAGCAGGA
AATCCGTATCATTTTCTCGCATACACGAACCCGCGTGCGCCTGGTAAATTGCAGGATTCTCATTGTCCGGTTTTC
TTTATGGGAATAATCATCATCACCATTATCACTGTTACTCTTGCGATCATCATCATTAACATAATTTTTTTAACG
CTGTTTGATGATGGTATGTGCTTTTATTGTTCCTTACTCACCTTTTCCTTTGTGTCTTTTAATTTTGACCATTTT
GACCATTTTGACCTTTGA

>YJL189W 1.56 Hot
ATGGCTGCTCAAAAGTCTTTCAGAATCAAGCAAAAAATGGCTAAGGCTAAGAAGCAAAACAGACCATTGCCACAA
TGGATCAGATTGAGAACCAACAACACTATCCGTTACAACGCTAAGAGAAGAAACTGGAGAAGAACCAAGATGAAC
ATCTAA

>YPL223C 1.69 Hot
ATGTCCAATCTATTAAACAAGTTTGCTGATAAGTTGCACGGCAACGATCATGATGAACGTTACGAAGACGACAAT
GACGACCAGACTAGACAACAGCGTCATGAAAAACATCAACAGAGGGAATTCAGGAATCAAGGATCCAAGGCCGAT
CCCTACGGCGAAGAAAACCAAGGGAATTTCCCTCAACGCCAGCAGCCACAGTCTAATCTAGGCGGTAACACGCAG
TTTGGCGGTAACGACTTCCAGCAACAAACTACTGACTACACTGCCGGCACTGGTGGTGGCACTTATACCCAAACT
TACCGCGAAACTAACACTCAAGGTCAGTTGGACGACGATGAAGACGATGACTTCTTGACTTCGGGCCAACAGCAA
AAACAAGGTCGTACAAGAGGTGCTCAAAGTAACCGCTACCAATCCTCTAATATCGGCAGCGGTAGACGCGATCTG
TCTGGGTCAGGAAACGATGAATATGATGATGATAGTGGGAACCAAGGCGTCTGGTAG

>YAL044C 2.07 Hot
ATGTTACGCACTACTAGACTATGGACCACCCGCATGCCCACTGTGAGCAAATTGTTTTTGAGAAACAGCTCCGGC
AATGCCCTAAACAAGAATAAACTACCATTTTTGTACTCATCCCAAGGACCTCAAGCCGTGAGGTACACTTCCCAA
CATGAGTGGATAGCTGTGCATCAGGACAAGACTGCCTTTGTCGGAATTACAAAATACGCCACTGATGCCTTAGGG
GACGCTACCTATGTTGAGTTGCCAGAAGTGGGCACTGAGATTGCCCAAGGTGAGTCGCTAGGGTCCATTGAGTCC
GTCAAGTCAGCCTCCGAGATCTACCAGCCTGCCGATGGTACCGTAGAGGAAATTAACACTAATCTTGAGGAAAAT
CCAGGTGTGGTGAACGAAGATCCTATGGGTGACGGCTGGCTAGTCAAAATGAAGCTTGGTGAGGGCGTTAATGTG
GAACAGGTCGAGGGTCTAATGTCCTTAGAACAGTACGAAAAGACACTGGTTCATGATGACTGA

>YCL030C 3.74 Hot
ATGGTTTTGCCGATTCTACCGTTAATTGATGATCTGGCCTCATGGAATAGTAAGAAGGAATACGTTTCACTTGTT
GGTCAGGTACTTTTGGATGGCTCGAGCCTGAGTAATGAAGAGATTCTCCAGTTCTCCAAAGAGGAAGAAGTTCCA
TTGGTGGCTTTGTCCTTGCCAAGTGGTAAATTCAGCGATGATGAAATCATTGCCTTCTTGAACAACGGAGTTTCT
TCTCTGTTCATTGCTAGCCAAGATGCTAAAACAGCCGAACACTTGGTTGAACAATTGAATGTACCAAAGGAGCGT
GTTGTTGTGGAAGAGAACGGTGTTTTCTCCAATCAATTCATGGTAAAACAAAAATTCTCGCAAGATAAAATTGTG
TCCATAAAGAAATTAAGCAAGGATATGTTGACCAAAGAAGTGCTTGGTGAAGTACGTACAGACCGTCCTGACGGT
TTATATACCACCCTAGTTGTCGACCAATATGAGCGTTGTCTAGGGTTGGTGTATTCTTCGAAGAAATCTATAGCA
AAGGCCATCGATTTGGGTCGTGGCGTTTATTATTCTCGTTCTAGGAATGAAATCTGGATCAAGGGTGAAACTTCT
GGCAATGGCCAAAAGCTTTTACAAATCTCTACTGACTGTGATTCGGATGCCTTAAAGTTTATCGTTGAACAAGAA
AACGTTGGATTTTGCCACTTGGAGACCATGTCTTGCTTTGGTGAATTCAAGCATGGTTTGGTGGGGCTAGAATCT
TTACTAAAACAAAGGCTACAGGACGCTCCAGAGGAATCTTATACTAGAAGACTATTCAACGACTCTGCATTGTTA
GATGCCAAGATCAAGGAAGAAGCTGAAGAACTGACTGAGGCAAAGGGTAAGAAGGAGCTTTCTTGGGAGGCTGCC
GATTTGTTCTACTTTGCACTGGCCAAATTAGTGGCCAACGATGTTTCATTGAAGGACGTCGAGAATAATCTGAAT
ATGAAGCATCTGAAGGTTACAAGACGGAAAGGTGATGCTAAGCCAAAGTTTGTTGGACAACCAAAGGCTGAAGAA
GAAAAACTGACCGGTCCAATTCACTTGGACGTGGTGAAGGCTTCCGACAAAGTTGGTGTGCAGAAGGCTTTGAGC
AGACCAATCCAAAAGACTTCTGAAATTATGCATTTAGTCAATCCGATCATCGAAAATGTTAGAGACAAAGGTAAC
TCTGCCCTTTTGGAGTACACAGAAAAGTTTGATGGTGTAAAATTATCCAATCCTGTTCTTAATGCTCCATTCCCA
GAAGAATACTTTGAAGGTTTAACCGAGGAAATGAAGGAAGCTTTGGACCTTTCAATTGAAAACGTCCGCAAATTC
CATGCTGCTCAATTGCCAACAGAGACTCTTGAAGTTGAAACCCAACCTGGTGTCTTGTGTTCCAGATTCCCTCGT
CCTATTGAAAAAGTTGGTTTGTATATCCCTGGTGGCACTGCCATTTTACCAAGTACTGCATTAATGCTTGGTGTT
CCAGCACAAGTTGCCCAATGTAAGGAGATTGTGTTTGCATCTCCACCAAGAAAATCTGATGGTAAAGTTTCACCC
GAAGTTGTTTATGTCGCAGAAAAAGTTGGCGCTTCCAAGATTGTTCTAGCTGGTGGTGCCCAAGCCGTTGCTGCT
ATGGCTTACGGGACAGAAACTATTCCTAAAGTGGATAAGATCTTGGGTCCAGGTAATCAATTTGTGACTGCCGCC
AAAATGTATGTTCAAAATGACACTCAAGCTCTATGTTCCATTGATATGCCAGCTGGCCCAAGTGAAGTTTTGGTT
ATTGCCGATGAAGATGCCGATGTGGATTTTGTTGCAAGTGATTTGCTATCGCAAGCTGAACACGGTATTGACTCC
CAAGTTATCCTTGTTGGTGTTAACTTGAGCGAAAAGAAAATTCAAGAGATTCAAGATGCTGTCCACAATCAAGCT
TTACAACTGCCACGTGTGGATATTGTTCGTAAATGTATTGCTCACAGTACGATCGTTCTTTGTGACGGTTACGAA
GAAGCCCTTGAAATGTCCAACCAATATGCACCAGAACATTTGATTCTACAAATCGCCAATGCTAACGATTATGTT

```
AAATTGGTTGACAATGCAGGGTCCGTATTTGTGGGTGCTTACACTCCAGAATCGTGCGGTGACTATTCAAGTGGT
ACTAACCATACATTACCAACCTATGGTTACGCTAGGCAGTACAGTGGTGCCAACACTGCAACCTTCCAAAAGTTT
ATCACTGCCCAAAACATTACCCCTGAAGGTTTAGAAAACATCGGTAGAGCTGTTATGTGCGTTGCCAAGAAGGAG
GGTCTAGACGGTCACAGAAACGCTGTGAAAATCAGAATGAGTAAGCTTGGGTTGATCCCAAAGGATTTCCAGTAG

>YER158C  1.82   Hot
ATGTTACAGCAGGGTTCTTCCTCGAGGAGGAGTTTGCATGGTAATGATTTTCACACGCTCACTTCTCCTTCAAGG
AGAGACTCTTTGAGTATTCCTAGGGCTGTGGACGCTAGATCTGCGTCTACGATTGATCTGTTCTACATACCGGAT
GCTACCGTCTCAAGGAGGCACTCTACTCTGGTGGCTAATAGATCAGACAATAATGGTAATGGCGCACCGATGCGC
CAGTACAATAAACCTAACTTTGCTTCCTCCTCGACTTCATCTCTGCCTTCAACTAGAAATCGCCCCTCTCGCTAC
GATAACATGAATATGAATATGAATATGAATATGAACATGAACATGAACATGAACATGAACATGAACAACCATACT
ACCAGTGATCACAATGCGCACCCGCAATATCGTTGTAGACCAAACCCGTCCAGACGCCACTCCTTAATGACCATT
CCCGAAAAGTACTCTGGTAGTCGCTACAGTCTCCGATCATCCCCGCCTACCTATTCTAATCCACGTGTTAGGAAG
GAACTTACTCCTTTCCAATTACAAAGAAAGCAGATGAAAAGCGCTTTTCAATTCCCCAACGGTGAAAATTTCACG
CCAAGAAACCAGATAGCAAGGCTTCCGCCATCTTCGACTTTTCCCGACTCTCCTTCTAGTTCATCGTTGCCTTTG
ACCCAGACTGGAGGCCCATCTTCCGCAGATAACGATTCCATTGCCACTGGCACGAACAATCGTTCTCCTCAACAA
ACGAAAGCTGCTGATGCTAATCAGAAATCTGAATCCGAGTCTCCAAAGGCAATTAGATCAAATTCTAAAAAGATA
TCTCGTTTCTTCAGGAAAATCTGGTCGTCCAAGTCATCAAATTCTGCAGATTCCGTAGAAGAGAATAGCAAAACA
AAGCAGAAAAGGAAAAATCCGGAGAGAGTTGTACCAGAACCGATAACGTCCCTTGACCAACCGGTTGAAATTATA
AAACAGAGTTTTTCAACCGTTAACAATCACGAAACGGCTGTACCTTCAATTAAGGACTCTGGCATAGTACAAGAA
TTGACAGCACTCGGAGATAATAACAGGATACCAGTTCTTCCACCCCCTAGGTCACCAAACAGACCTACACTGTCT
GATAAACGAACTACAAAGTTATACTACTGTAGTCAAGATTCGTCTAATGAAGATATTGCTCCAGAGGAAAAGAGC
ACCGTTTTCTTAAAGCGTTTGCAGGATGAATGGTCTACCGTGTATTTAAACAAATTGCCTTTGACTGCTTCTGTG
CCATCTTCTCTCTCTACCACTACTGATGCTGCGAACTCTTCGTTCATAAACTCTTCAATATCATCGCCGGCTCCT
TCTTCTTCGTCCTCGTCATCGTTGGTTTCCCGTGGACCAATGCAATCCATATCTTCTTCGCCAACACCTGCCCCT
TCTTCAGGGTCATCGAAATCTAAAAATGCCGTCAAATCCTTACGCTTTGCGGATGAGATTTATGTAAACGATACG
TGGTCCGCCGCGGACTATTGCAGGTGTGATAATACCTTTTTGAATAATTTCTTTAAAGGAAAGAGCCAAGACATT
ACCAATCCTTCTACTTTTGTCGGTAATAATCTCTCATCCACAAAAAATATATCCAATATTGAAATCAAAATGGAG
GTGAATGAGTTCAAAAGAAAGGAAATGCGCGTTCACCAGGATAGCGCTAAGTATACACATTACTACTTATAA

>YHL025W  1.81   Hot
ATGGGTGTCATCAAGAAGAAAAGATCGCACCATGGAAAGGCTTCGCGCCAACAATACTACTCTGGGGTACAGGTT
GGGGGAGTAGGCAGCATGGGCGCCATAAACAATAACATCCCGTCGTTGACGAGCTTCGCGGAGGAAAACAACTAT
CAGTACGGATACAGCGGCTCCAGTGCCGGCATGAATGGCAGATCGCTTACGTACGCGCAGCAACAGCTTAATAAG
CAAAGACAGGACTTCGAACGTGTACGACTTAGACCAGAACAGCTCAGCAATATCATACATGACGAGAGCGACACG
ATATCGTTCCGATCCAACCTTTTGAAGAACTTTATAAGCTCGAACGACGCATTTAACATGCTGAGTTTGACCACG
GTACCGTGCGACAGAATTGAGAAGTCCAGATTGTTCAGTGAAAAAACAATAAGATATCTCATGCAAAAACAACAC
GAAATGAAAACACAAGCGGCAGAGTTGCAAGAAAAGCCTCTGACGCCACTTAAATACACAAAACTTATAGCTGCG
GCAGAGGACGGAAGCCGTAGCACAAAGGATATGATAGATGCTGTCTTCGAGCAAGATAGTCATTTGAGGTACCAG
CCGGACGGCGTGGTCGTACATCGTGACGATCCTGCGCTGGTGGGTAAACTCCGCGGAGATCTCCGCGAAGCGCCG
GCGGACTACTGGACGCATGCTTATAGGGATGTTTTGGCGCAATACCACGAGGCCAAGGAGCGTATCAGGCAGAAG
GAAGTAACTGCAGGTGAAGCACAGGACGAAGCCAGCTTGCAGCAGCAACAGCAGCAAGATTTGCAGCAACAGCAA
CAAGTAGTGACTACAGTTGCCTCGCAAAGTCCTCATGCAACTGCAACGGAAAAGGAGCCAGTACCCGCCGTGGTT
GACGACCCACTGGAGAACATGTTCGGAGATTATTCCAATGAGCCGTTCAACACCAATTTCGACGATGAATTTGGA
GATCTTGATGCTGTATTTTTTTAA

>YJL134W  1.72   Hot
ATGGTAGATGGACTGAATACCTCGAACATTAGGAAAAGAGCCAGGACTCTCTCTAACCCCAATGACTTTCAAGAG
CCTAATTACTTGCTGGATCCCGGTAATCATCCCTCAGATCATTTTCGAACTCGAATGTCCAAATTTCGGTTTAAT
ATTAGAGAGAAGCTGTTAGTGTTTACCAACAATCAATCATTCACATTAAGCCGCTGGCAAAAGAAGTACCGTTCT
GCGTTTAATGATCTCTACTTTACTTATACTTCCTTAATGGGATCGCATACCTTCTATGTTCTGTGTTTACCTATG
CCCGTGTGGTTTGGATATTTTGAAACAACAAAAGATATGGTTTATATCTTGGGATATTCTATCTACTTGAGTGGT
TTTTTTAAAGATTACTGGTGCTTGCCCAGGCCTAGAGCACCTCCATTACATCGAATTACGTTAAGTGAATATACA
ACGAAGGAATATGGTGCTCCAAGCTCCCATACAGCAAATGCAACAGGAGTGAGTCTCTTGTTTCTCTACAACATC
TGGAGGATGCAAGAATCTTCTGTCATGGTCCAACTATTGTTGTCATGTGTGGTTTTATTTTATTATATGACTTTG
GTTTTCGGTAGAATATACTGTGGGATGCATGGCATATTAGATTTAGTAAGCGGTGGGCTCATTGGAATAGTGTGT
TTCATTGTTAGGATGTATTTCAAGTACAGGTTTCCGGGTTTACGCATTGAGGAGCATTGGTGGTTTCCTTTGTTT
AGTGTGGGATGGGGTCTTCTTCTTTTGTTTAAACATGTTAAGCCCGTAGACGAATGTCCTTGCTTCCAAGATAGT
GTTGCGTTCATGGGCGTTGTGTCAGGTATTGAATGCTGTGATTGGTTGGGCAAAGTGTTTGGAGTCACCCTGGTG
TACAATTTGGAACCTAACTGTGGCTGGCGGTTAACCTTAGCCAGGCTGCTGGTGGGCCTACCGTGCGTTGTTATC
TGGAAGTACGTGATCAGCAAACCGATGATCTACACGTTATTGATCAAAGTTTTCCATCTGAAGGATGACAGAAAC
GTTGCGGCAAGAAAAAGACTGGAGGCCACGCACAAAGAAGGTGCAAGCAAGTACGAATGTCCATTATATATTGGA
GAGCCCAAGATTGACATTCTAGGTAGATTTATTATCTATGCTGGCGTTCCATTCACCGTTGTAATGTGCAGCCCC
GTCCTATTTTCCCTCTTAAATATAGCATAA

>YGR159C  1.69   Hot
```

ATGGCTAAGACTACTAAAGTAAAAGGTAACAAGAAGGAAGTTAAGGCTTCCAAACAAGCCAAAGAAGAAAAAGCT
AAGGCCGTCTCTTCCTCTTCCTCCGAATCTTCATCCTCATCTTCATCTTCATCTGAATCTGAATCTGAGTCTGAG
TCTGAATCTGAATCTTCATCTTCATCTTCATCCTCTGATTCTGAATCCTCTTCTTCATCGTCTTCTGACAGCGAA
AGTGAAGCTGAAACCAAGAAGGAAGAATCCAAGGATTCCTCTTCCTCTTCCTCTGACTCTTCTTCCGACGAAGAA
GAAGAAGAAGAAAAAGAAGAAACCAAGAAGGAAGAATCAAAAGAATCTTCTAGCTCTGATTCATCCTCATCTTCA
TCTTCTGATAGCGAAAGCGAAAAGGAAGAGTCTAACGATAAGAAACGTAAATCTGAGGACGCCGAAGAAGAAGAA
GACGAAGAGTCTTCCAACAAGAAGCAAAAAAATGAAGAAACCGAAGAACCAGCTACTATTTTCGTTGGTAGACTA
TCGTGGTCTATTGATGACGAATGGTTGAAGAAGGAATTCGAACACATCGGTGGTGTCATTGGTGCCAGAGTTATT
TATGAAAGAGGTACCGATAGATCTCGTGGTTATGGTTACGTTGATTTTGAAAACAAATCTTATGCTGAAAAGGCC
ATTCAAGAAATGCAAGGTAAGGAAATTGATGGTAGACCAATCAACTGTGATATGTCCACAAGCAAGCCAGCTGGT
AACAACGATCGTGCCAAGAAATTCGGTGATACCCCATCTGAACCATCTGACACTTTGTTCTTGGGTAACTTATCT
TTCAATGCTGACAGAGACGCTATTTTCGAATTATTCGCTAAACACGGTGAAGTTGTTTCCGTCCGTATCCCAACA
CATCCAGAAACTGAACAACCAAAAGGTTTCGGTTATGTTCAATTCTCCAACATGGAGGACGCCAAGAAGGCTCTA
GACGCTTTACAAGGTGAATACATTGACAACAGACCAGTTAGATTAGACTTCTCTTCTCCAAGACCAAACAACGAT
GGTGGTCGTGGCGGTAGCCGTGGTTTTGGTGGTCGTGGCGGTGGTCGTGGCGGTAACCGTGGATTCGGTGGTCGT
GGTGGCGCTCGCGGTGGCCGTGGCGGTTTCAGACCATCTGGTTCTGGTGCTAATACTGCTCCATTGGGCAGATCA
AGAAATACCGCTTCTTTCGCTGGTTCAAAGAAAACATTTGATTAA

>YEL037C 1.54 Hot
ATGGTTAGCTTAACCTTTAAAAATTTCAAGAAGGAAAAAGTTCCTTTAGATCTGGAACCTTCAAACACAATTTTA
GAGACCAAGACCAAGCTTGCTCAATCCATTTCTTGTGAAGAATCTCAAATAAAACTGATCTACTCGGGTAAAGTG
CTACAAGATTCCAAAACCGTATCGGAATGCGGGCTAAAAGATGGGGACCAAGTTGTCTTCATGGTTTCTCAAAAA
AAGTCCACGAAGACCAAAGTAACAGAACCTCCAATTGCTCCTGAGAGCGCCACTACCCCCGGAAGAGAAAATTCT
ACAGAAGCATCCCCCAGTACGGATGCTTCTGCAGCTCCTGCAGCCACTGCTCCCGAAGGCTCACAACCGCAAGAA
GAACAAACCGCCACTACAGAACGTACTGAATCTGCCTCTACACCGGGATTCGTGGTGGGAACCGAGAGGAACGAG
ACCATCGAGAGAATCATGGAAATGGGCTACCAAAGAGAGGAAGTCGAACGAGCCTTGAGAGCAGCCTTTAATAAT
CCAGATAGAGCGGTGGAATATCTACTGATGGGTATTCCAGAAAATCTGCGTCAACCGGAACCACAGCAACAAACA
GCCGCCGCAGCGGAACAACCATCGACAGCCGCCACCACTGCGGAACAACCGGCTGAAGACGACTTATTTGCACAA
GCTGCCCAAGGCGGTAATGCTTCATCCGGTGCGCTTGGCACAACTGGAGGAGCTACAGATGCTGCGCAAGGTGGA
CCTCCAGGTTCCATTGGCCTCACTGTAGAAGATTTACTATCGTTGAGACAGGTCGTTTCAGGTAACCCAGAAGCT
TTAGCCCCATTGTTGGAAAACATAAGTGCTAGATATCCTCAATTACGTGAACATATCATGGCAAACCCAGAAGTG
TTTGTGTCCATGTTGCTAGAAGCCGTGGGTGACAATATGCAAGATGTTATGGAAGGTGCGGATGATATGGTGGAA
GGAGAGGATATAGAAGTTACAGGAGAGGCTGCTGCTGCAGGACTGGGACAAGGTGAAGGTGAAGGTTCTTTCCAA
GTTGACTATACCCCCGAAGACGATCAAGCTATTTCGCGCCTCTGTGAATTGGGCTTTGAAAGAGATCTTGTTATC
CAGGTGTATTTTGCGTGCGATAAAAACGAAGAAGCTGCAGCAAATATTCTATTCAGCGATCATGCCGACTGA

>YPL068C 1.66 Hot
ATGCATATGCAACTCAGGAAAAGAAAAAGGGTTGACTATTCAGGGAGAAATCAGACGTCCGATCCTCCTTCAACC
ACAACGGCAGCGGTACCATCCATTATTGTCCCTAAGAAGCGTAAAGTAGTCGCACAAAACATGGTTAGTCCAGCT
ATTCGAGCCACAACCACAACACTGGGCACCTCAAATATAATAATACCTAAGCCTCTGCAGCGCCCTAAATTCCAC
AATAGCGCCAGCCTATCTTCACCTGATGATGATCCCGAAAAGATCTCTGTATTGGAAGTTCAGAAAAATTTGTCC
AATCTCATCAAAAGACAACAAAGATTATTTTATAAAGATATACATAAGCCGACTCTCGCCGGCTTGAAAAATTTC
GAAATGTTAAGGCTGCCCAATGATCTAAAACTCCTACAAAACATCGTAAACCTATTATATTCATTCGAGCAATTA
AACAGTGACTCGAAGACAAGACCAGTCACTACTTCCAAATTAAAGGCCTCCTCTCAAGCGCACTCAGACAAATTG
AAAAAAATGCTAGCTGAGAGGAAACCTCCATTTAGCCACCCCAGTCACAGCGGCACTGCCTACCACAATGACATC
ATCCATGAGATAGCGAATTTGCATTCTATTAACCTGGTAGATTTGATTAATTTGGAAGTATATAATAACAATTGC
CATACAAACAACACAGCGTTGCAAACTACGGCGAATTCGCTGACACTTAATAGCATTATCAAAAAGCTTGACAAG
CCAATACTGAAAGAAAGAAATAATTCTCTGGTATGGCCCCATAAAAGCAGATTTAAAGCAAAAAGGAACCAACCT
TCGCCTGGCCAATCTTTAATTAACAATACAGATATAACTCTCTACAACGATGTATAG

>YEL059W 1.85 Hot
ATGAGTCTTTCTTTCCTTCTCTTTTCCCCTTTTCTCCCCCCCTGTTTCTCGTCCATCTCTATCTGTCTATCTGTC
TTATCTACTGTTAGTTTTTTTTTCGCTTTCACGATACCGCATTACGTACTGCGCTGCGGAAGTGTGGATGAGTGG
CATATACACTCTTCTGCAGAGGATTTTCGTACACAACGGTGTGTGTGTGCTGTGAAGCTGTCTGCTTCCTTACTT
GGCTGTTTGCTTGCTTGTGCGTCATGGTCACTTTTGTTAGAAGTGAGCCGCATAAAATGGCATGTCGGCACAGCC
TACAGTTAA

>YGR177C 5 Hot
ATGGAAGATATAGAAGGATACGAACCACATATCACTCAAGAGTTGATAGACCGTGGCCATGCAAGACGTATGGGC
CACTTGGAAAACTACTTTGCTGTTTTGAGTAGGCAGAAAATGTACTCGAATTTTACTGTTTACGCGGAATTGAAT
AAAGGTGTTAATAAGAGACAACTAATGCTTGTCTTGAAAGTATTACTTCAAAAATACTCAACTCTTGCGCATACA
ATCATTCCTAAGCATTATCCTCATCATGAAGCGTACTACTCTAGCGAAGAGTACCTTAGTAAACCTTTTCCACAG
CATGATTTCATAAAGGTGATTTCTCATCTTGAATTCGATGACTTGATTATGAATAATCAACCAGAATACAGAGAA
GTCATGGAGAAAATCTCAGAACAGTTCAAAAAGGATGATTTCAAAGTCACCAATAGGTTAATCGAATTGATTAGC
CCTGTAATCATACCTCTGGGTAATCCGAAGAGGCCTAATTGGAGATTGATTTGTTTACCAGGTAAGGATACTGAT
GGGTTTGAAACGTGGAAAAACTTCGTTTATGTCACTAACCACTGCGGCTCCGACGGTGTCAGTGGATCGAATTTT
TTCAAAGATTTAGCTCTACTCTTTTGTAAAATCGAAGAAAAAGGGTTTGATTATGATGAAGAGTTCATCGAAGAT

CAAGTCATCATTGACTATGATCGAGACTACACTGAAATTTCTAAATTGCCAAAACCGATTACGGATCGTATTGAC
TACAAGCCAGCATTGACTTCATTACCCAAATTCTTTTTAACAACCTTCATTTATGAACATTGTAATTTTAAAACC
TCCAGCGAATCTACACTTACAGCTAGATATAGCCCCTCTAGTAATGCTAATGCTAGTTACAATTACTTGTTGCAT
TTCAGTACTAAGCAAGTAGAACAAATCAGAGCTCAGATCAAGAAAAATGTTCACGATGGGTGCACCCTAACACCC
TTCATTCAAGCGTGCTTTCTTGTAGCCCTGTATAGACTGGATAAGCTGTTCACAAAATCTCTTCTCGAGTATGGG
TTCGATGTGGCTATTCCAAGCAACGCAAGAAGGTTTTTACCAAACGATGAAGAGTTAAGAGATTCTTATAAATAC
GGCTCCAACGTTGGAGGTTCGCATTACGCCTATCTAATCTCCTCATTCGACATTCCCGAAGGTGACAATGACAAG
TTTTGGAGTCTTGTCGAATACTACTATGACCGCTTTTTAGAATCGTACGACAACGGTGACCACTTGATTGGTCTG
GGGGTCCTACAACTTGATTTTATCGTTGAAAACAAGAATATAGACAGCCTTCTTGCCAACTCTTATTTGCACCAG
CAAAGAGGCGGTGCAATCATCAGTAATACAGGACTTGTCTCGCAAGATACGACCAAGCCGTACTACGTTCGGGAT
TTAATCTTCTCGCAGTCTGCAGGCGCCTTGAGATTTGCGTTCGGCCTAAACGTTTGCTCCACAAACGTGAATGGT
ATGAACATGGACATGAGCGTGGTTCAGGGCACTCTACGGGATCGTGGCGAATGGGAATCGTTCTGCAAGCTCTTC
TACCAAACCATCGGCGAATTTGCGTCGCTTTAA

>YLR416C 1.53 Hot
ATGAAACATGTGAAACCCTTTTTCCTCTTCGTTGATCCTAAAAAAAGCGCGTCTGGAAAACAAGGCGAAGCTTCC
TCACATGGAAAAGGCTTCTTAATATGTCGCTACGCCTCATTCCCATTGCATACAGAAAGCAAAGTAGTGCGTTTT
GCGAACGTCACAGATCAAAAGTTTGGCCATGCATTGTCATTCTTTTTTTCTTACATACACTGCACCAGACATGTC
GGTTCCGCCCCGCTAACGGTTAGTCAGCCACACATTGACGTACACTGTGAACAGCCTATTTCTTTCCATGTATCT
CAGTGCCCAGCTTATGAGAACTGTCACAGCCTCCCACTTGACCCTCAGAGCCCTCTCCACTCCCCCCCTCTTTCA
ACATCGCCAGATAGCCGCCGTTGA

>YGR176W 5.88 Hot
ATGCATCGACTACGAGCGTATTGTGTTATATCAGCTCTGTCCGATACACTGCTTGCCTTTTGTACGAGCTCGGCC
GAGCTATACGAAGGCCCGCTACGGCAGTATCGCATTAAAGCGACGCAAATTCGCCGATGGTTTGGTAGAAGAGCT
TGCAGAACGATTCCCATTCGCCACGATCCCGTAGAGTGCCCTGAACCACGCTCATGTCCATGTTCATACCATTCA
CGTTTGTGGAGCAAACGTTTAGGCCGAACGCAAATCTCAAGGCGCCTGCAGACTGCGAGAAGATTAAATCCCGAA
CGTAGTACGGCTTGGTCGTATCTTGCGAGACAAGTCCTGTATTACTGA

>YJR022W 1.64 Hot
ATGTCAGCCACCTTGAAAGACTACTTAAATAAAAGAGTTGTTATAATCAAAGTTGACGGCGAATGCCTCATAGCA
AGCCTAAACGGCTTCGACAAAAATACTAATCTATTCATAACCAATGTTTTCAACCGCATAAGCAAGGAATTCATC
TGCAAGGCACAGTTACTTCGAGGCAGCGAGATTGCTCTTGTTGGCCTCATAGATGCAGAAAATGATGACAGTCTA
GCTCCTATAGACGAAAAGAAGGTCCCAATGCTAAAGGACACCAAGAATAAAATCGAAAATGAGCATGTAATATGG
GAAAAAGTGTACGAATCAAAGACAAAATAA

>YER152C 2.22 Hot
ATGAAATACAAGGAAATCAATTTCTTCAAGGGCCATCCGAGCTCGAGGTTGCTGCCTCGAGAAGCAGTAATTCAA
GCGACTGCGGCTATATTGGGGCCCGAGACCAGGGAGTACGATAACGACCCCTATAACAGGCATCCGCTGACGTAC
GGTTCGGACGAAGGTGCCCTGTGGGTGCGAGAGCAGATTTGTACGTTTCTGAATGATCAGCTGTTTAAGTTCGAA
AATGGGGCTCGGAGCAGGACACGGGCAGACTATTTGAATCTGAATAGCGGCGCTTCGTATGGCATGCTGAACATC
CTTCTGCAAACAACCTTGCCACATAACGGGTATACCAGGCAGGCGTTCATCATCACGCCAACATATTTCTTGATC
AACAATTGCTTCACAGATGCGGGATTCAAGGGGAAAATGACCGCCATCAACGAGCAGGGCCACGACTCGATTGAT
TTCGAGTCGTTGATTTCTGCCCTTGAGCAGCACGAGGCGGAGCCGCAGCCCCATAGTACCACAGAGATGATTCAG
GGGCCAAAGTTGACCAAGAAGGTCTACAGGTACGTTATGTACTGCATCCCGACGTTTGCAAACCCATCGGGAAAC
ACATACTCGCTTGAGACCAGACGCAGACTTATCGACATCGCTCGGAAGTACGACATGCTGATAATCACTGATGAC
GTGTACGATATTCTAGATTACACGACGCCCTCAGATGAGCTGCCCTCTCCGCCCCTAAGGATGGTGCACATAGAC
AGAAGTACAGCGCCCTCCGGTGAGGACTCGTTCGGGAATACAGTGTCCAACGCAACTTTCTCCAAGCTGATCGCC
CCTGGGCTCAGATTTGGATACCATGAGTCAATCAACGCGAATCTCGCCAGACAGCTATCTAAAGGTGGTGCAAAC
GTCTCTGGCGGAACTCCCTCACAACTGAACTCCATGATCGTGGGTGAGATGCTGCGTAGTGGTGCCGCCCAGAGA
TGCATTGCACATCTGAGATCCGTATACTCCGAGAGGGCCACTGTCTTGACCTCGGCGCTTAAGAAATACATGCCC
CTAGGAACCGAGATTATGCCATTGAAGGGCGGCTATTTTACTTGGATCACTCTCCCACCAGCGTACAATGCCATG
GAGATATCCACTATTCTTGCCAAGAAATTTAATGTCATCCTTGCCGACGGCTCCAATTTCGAGGTCATCGGCGAT
GAGAAAAAACTGGGGTCAGTCATGCTTTAGGCTTTCTATTAGTTTCTTAGAAGTTGATGATATCGACAGGGGCATT
GAGCTGTTTGGTGCTGTTTGCAAATCTCATGCGATCACCAATAACATAACTATGTAG

>YAL060W 1.54 Hot
ATGAGAGCTTTGGCATATTTCAAGAAGGGTGATATTCACTTCACTAATGATATCCCTAGGCCAGAAATCCAAACC
GACGATGAGGTTATTATCGACGTCTCTTGGTGTGGGATTTGTGGCTCGGATCTTCACGAGTACTTGGATGGTCCA
ATCTTCATGCCTAAAGATGGAGAGTGCCATAAATTATCCAACGCTGCTTTACCTCTGGCAATGGGCCATGAGATG
TCAGGAATTGTTTCCAAGGTTGGTCCTAAAGTGACAAAGGTGAAGGTTGGCGACCACGTGGTCGTTGATGCTGCC
AGCAGTTGTGCGGACCTGCATTGCTGGCCACACTCCAAATTTTACAATTCCAAACCATGTGATGCTTGTCAGAGG
GGCAGTGAAAATCTATGTACCCACGCCGGTTTTGTAGGACTAGGTGTGATCAGTGGTGGCTTTGCTGAACAAGTC
GTAGTCTCTCAACATCACATTATCCCGGTTCCAAAGGAAATTCCTCTAGATGTGGCTGCTTTAGTTGAGCCTCTT
TCTGTCACCTGGCATGCTGTTAAGATTTCTGGTTTCAAAAAAGGCAGTTCAGCCTTGGTTCTTGGTGCAGGTCCC
ATTGGGTTGTGTACCATTTTGGTACTTAAGGGAATGGGGGCTAGTAAAATTGTAGTGTCTGAAATTGCAGAGAGA
AGAATAGAAATGGCCAAGAAACTGGGCGTTGAGGTGTTCAATCCCTCCAAGCACGGTCATAAATCTATAGAGATA

CTACGTGGTTTGACCAAGAGCCATGATGGGTTTGATTACAGTTATGATTGTTCTGGTATTCAAGTTACTTTCGAA
ACCTCTTTGAAGGCATTAACATTCAAGGGGACAGCCACCAACATTGCAGTTTGGGGTCCAAAACCTGTCCCATTC
CAACCAATGGATGTGACTCTCCAAGAGAAAGTTATGACTGGTTCGATCGGCTATGTTGTCGAAGCCTTCGAAGAA
GTTGTTCGTGCCATCCACAACGGAGACATCGCCATGGAAGATTGTAAGCAACTAATCACTGGTAAGCAAAGGATT
GAGGACGGTTGGGAAAAGGGATTCCAAGAGTTGATGGATCACAAGGAATCCAACGTTAAGATTCTATTGACGCCT
AACAATCACGGTGAAATGAAGTAA

>YFL032W 1.79 Hot
ATGAGGGTTGTAAGGCAAAGTGGCTCAGCATTAAGTAGGGCTAAAAAGAGTCGCAAATATACATCGTACCGAGTG
ATGAACGTGGTTTTGAACACCTTGTTCTCTTTTGTTCTCGCTCCCTACATTCACTATATATTAGAAGAAATTTCA
CCTCAATGGACAACTCGAGAAATGAATACAGAAATATGTTTTTTAGCGAAATTTTCCTTTCTTCTTGTCTTCTTG
TTTTATTTAAACTTCCAAGGCTTTAACTCAGTGTCAAACATAACAACCTCCTCCTCCCCCACCTACGACAACAAC
CGCCACTATGGAAATGACTGA

>YDL085W 1.54 Hot
ATGCTGCCCAGACTTGGTTTTGCGAGGACTGCTAGGTCCATACACCGTTTCAAGATGACCCAGATCTCTAAACCT
TTTTTCCATTCCACTGAAGTTGGTAAGCCCGGACCACAGCAGAAGCTATCGAAATCTTACACTGCGGTATTCAAG
AAATGGTTTGTCAGAGGTTTAAAGTTAACCTTTTACACGACGTTGGCCGGCACATTGTATGTGTCATACGAGCTG
TACAAAGAATCGAACCCACCCAAACAGGTTCCCCAATCGACCGCTTTTGCTAATGGTTTGAAAAAGAAGGAGCTG
GTTATTTTGGGTACAGGCTGGGGCGCCATATCTCTTTTGAAGAAATTAGACACGTCTTTGTATAACGTGACCGTG
GTGTCGCCAAGAAGCTTCTTTTTGTTCACACCGTTATTACCCTCAACGCCTGTGGGTACGATAGAGATGAAGTCT
ATTGTCGAACCGGTTAGATCGATCGCTAGAAGAACGCCTGGAGAAGTTCACTACATTGAGGCGGAAGCGTTGGAC
GTTGATCCAAAGGCCAAAAAAGTAATGGTGCAATCGGTGTCAGAGGACGAATATTTCGTTTCGAGCTTAAGTTAC
GATTATCTTGTTGTTAGTGTAGGCGCTAAAACCACTACTTTTAACATTCCCGGGGTCTATGGCAATGCTAACTTC
TTGAAAGAGATTGAAGATGCTCAAAATATTCGTATGAAGTTAATGAAAACCATAGAACAGGCAAGTTCATTTCCT
GTGAACGATCCGGAAAGGAAGCGATTATTAACGTTCGTGGTTGTTGGAGGGGGCCCTACGGGGGTTGAATTTGCC
GCCGAACTGCAAGATTACATCAATCAAGATTTGAGGAAGTGGATGCCCGACTTAAGTAAAGAAATGAAGGTTATC
TTAATTGAAGCCCTGCCTAATATCCTAAACATGTTCGATAAGACGTTGATCAAGTATGCCGAGGACCTTTTTGCC
AGAGATGAAATTGACTTGCAAGTGAATACTGCCGTGAAAGTCGTAGAGCCAACCTATATACGCACTCTGCAAAAC
GGCCAAACAAACACGGATATCGAATACGGGATGCTGGTTTGGGCCACGGGAAATGAACCAATCGATTTTTCAAAG
ACACTGATGAGTAGAATACCGGAGCAAACTAATAGGCGTGGTCTGTTAATTAATGACAAGTTGGAGCTTCTCGGT
TCTGAGAATTCGATTTATGCAATTGGTGATTGTACCGCACACACGGGTTTCTTTCCCACGGCACAAGTTGCACAT
CAGGAAGGCGAATACTTGGCCAAGATCTTGGATAAAAAATTACAGATAGAACAATTGGAATGGGACATGCTCAAC
AGTACCGATGAAACTGAGGTATCACGTCTACAAAAAGAGGTTAATTTGAGGAAATCTAAGTTGGATAAGTTCAAC
TACAAGCATATGGGTGCCCTTGCGTACATCGGCTCTGAAACCGCAATTGCAGATTTGCATATGGGCGACTCATCA
TACCAGTTGAAAGGTATGTTTGCCTTCTTGTTTTGGAAATCCGCTTATTTGGCCATGTGTCTCTCTATCAGGAAT
AGGATTTTAATTGCCATGGACTGGACCAAAGTTTACTTTCTTGGAAGGGATTCCTCCGTGTAG

>YCL028W 1.5Hot
ATGGATACGGATAAGTTAATCTCAGAGGCTGAGTCTCATTTTTCTCAAGGAAACCATGCAGAAGCTGTTGCGAAG
TTGACATCCGCAGCTCAGTCGAACCCCAATGACGAGCAAATGTCAACTATTGAATCATTAATTCAAAAAATCGCA
GGATACGTCATGGACAACCGTAGTGGTGGTAGTGACGCCTCGCAAGATCGTGCTGCTGGTGGTGGTTCATCTTTT
ATGAACACTTTAATGGCAGACTCTAAGGGTTCTTCCCAAACGCAACTAGGAAAACTAGCTTTGTTAGCCACAGTG
ATGACACACTCATCAAATAAAGGTTCTTCTAACAGAGGGTTTGACGTAGGGACTGTCATGTCAATGCTAAGTGGT
TCTGGCGGCGGGAGCCAAAGTATGGGTGCTTCCGGCCTGGCTGCCTTGGCTTCTCAATTCTTTAAGTCAGGTAAC
AATTCCCAAGGTCAGGGACAAGGTCAAGGTCAAGGTCAAGGTCAAGGACAAGGTCAAGGTCAAGGTTCTTTTACT
GCTTTGGCGTCTTTGGCTTCATCTTTCATGAATTCCAACAACAATAATCAGCAAGGTCAAAATCAAAGCTCCGGT
GGTTCCTCCTTTGGAGCACTGGCTTCTATGGCAAGCTCTTTTATGCATTCCAATAATAATCAGAACTCCAACAAT
AGTCAACAGGGCTATAACCAATCCTATCAAAACGGTAACCAAAATAGTCAAGGTTACAATAATCAACAGTACCAA
GGTGGCAACGGTGGTTACCAACAACAACAGGGACAATCTGGTGGTGCTTTTTCCTCATTGGCCTCCATGGCTCAA
TCTTACTTAGGTGGTGGACAAACTCAATCCAACCAACAGCAATACAATCAACAAGGCCAAAACAACCAGCAGCAA
TACCAGCAACAAGGCCAAAACTATCAGCATCAACAACAGGGTCAGCAGCAGCAACAAGGCCACTCCAGTTCATTC
TCAGCTTTGGCTTCCATGGCAAGTTCCTACCTGGGCAATAACTCCAATTCAAATTCGAGTTATGGGGGCCAGCAA
CAGGCTAATGAGTATGGTAGACCGCAACAGAATGGTCAACAGCAATCCAATGAGTACGGAAGACCGCAATACGGC
GGAAACCAGAACTCCAATGGACAGCACGAATCCTTCAATTTTTCTGGCAACTTTTCTCAACAGAACAATAACGGC
AACCAGAACCGCTACTGA

>YFR027W 1.52 Hot
ATGAAAGCTAGGAAATCGCAGAGAAAAGCGGGCAGTAAACCAAATCTTATCCAGTCTAAATTGCAAGTTAATAAT
GGTTCGAAATCGAATAAAATAGTCAAGTGTGATAAATGTGAGATGTCATATTCCTCGACATCAATAGAAGATCGC
GCCATCCACGAGAAATACCACACTTTACAGCTGCATGGACGTAAATGGTCGCCGAATTGGGGTTCTATAGTATAC
ACAGAGCGAAACCATTCAAGGACGGTGCATCTATCAAGATCGACAGGGACAATAACGCCATTGAACTCCTCACCT
TTGAAAAAAAGTAGTCCGTCTATTACCCATCAGGAGGAGAAGATTGTATATGTGAGACCAGATAAGTCGAATGGT
GAAGTCCGAGCCATGACGGAGATAATGACACTAGTGAATAACGAGCTGAATGCGCCACACGATGAGAATGTCATT
TGGAACAGTACCACAGAAGAAAAAGGCAAAGCGTTTGTATACATAAGAAATGACAGGGCGGTCGGAATAATAATT
ATAGAGAACCTTTATGGGGGCAATGGTAAAACATCTAGTCGTGGACGTTGGATGGTTTATGATTCTAGAAGATTG
GTACAGAATGTGTACCCCGATTTTAAGATTGGCATATCGAGAATTTGGGTGTGCAGGACAGCAAGGAAGTTGGGT

ATCGCAACCAAATTGATTGACGTTGCAAGAGAAAATATTGTTTACGGTGAAGTTATTCCTAGGTACCAGGTAGCA
TGGTCGCAACCCACAGACAGCGGTGGAAAACTGGCTAGCAAATACAACGGCATTATGCATAAATCAGGCAAGTTA
CTATTGCCGGTATACATATGA

>YJR114W 1.56 Hot
ATGGGCACGGACTTCAGTGCCTCCCATTGTGCCAACTGCTCTGCAGTTGGCTTTATCTTGGAGCCCGCCTGTGAA
CCCTGTAAGAGGTCTACTTTGGACTGGCCCGGTGGAGCCAGCGAGGTCTCTGGCAGGTATTCTTTTGCAGAGAAC
TCTTCCCTTAGCTCGTTGATCGCTTCAAGCCACTCGTCGACCCCTTTCTCTGCCGATGGTTCGCTAGCTGGTGTT
CGTACTGACGACTGGAAACGAGCAACACATGTCCCACTTTGCCACAGGAGACGTGGCCTGACTAACATATACCTC
CTCGCACAATGCAGCATTATATTCACCTCAGATCCGTATCTATCTATTTTTCTTCTGAAAACGATAGCTGCTGTC
TTTTCTGTACGTGTATAA

>YJL026W 2.12 Hot
ATGCCTAAAGAGACCCCTTCCAAAGCTGCTGCCGATGCATTGTCCGACTTGGAAATCAAAGATTCCAAGTCCAAC
CTTAACAAGGAATTGGAGACATTGAGAGAGGAAAACAGAGTAAAGTCAGACATGCTTAAGGAGAAATTGAGCAAG
GACGCTGAAAATCACAAGGCTTACTTGAAATCTCATCAAGTTCACCGTCACAAACTTAAGGAAATGGAAAAGGAG
GAACCTTTGTTGAATGAAGACAAGGAGAGAACTGTTCTTTTCCCTATCAAGTACCATGAAATCTGGCAAGCCTAC
AAGCGTGCCGAAGCTTCTTTCTGGACCGCTGAAGAAATTGATTTGTCTAAGGATATCCATGACTGGAACAACAGA
ATGAACGAAAACGAGAGATTTTTCATTTCCAGAGTTCTTGCCTTTTTCGCCGCTTCTGACGGTATTGTTAATGAA
AACTTGGTTGAAAACTTCTCCACCGAAGTCCAAATTCCAGAGGCAAAGAGTTTCTACGGTTTCCAAATCATGATT
GAAAATATTCACTCTGAAACTTACTCCTTGTTGATCGATACTTACATCAAGGACCCTAAAGAAAGTGAATTCTTG
TTCAATGCCATTCACACCATCCCAGAAATCGGTGAGAAGGCCGAATGGGCTTTAAGATGGATTCAAGACGCTGAC
GCCTTGTTTGGTGAAAGACTAGTTGCCTTTGCCTCCATTGAAGGTGTCTTTTTCTCCGGTTCCTTTGCCTCCATT
TTCTGGTTGAAAAAGAGAGGTATGATGCCCGGTTTAACCTTTTCCAACGAATTGATCTGTAGAGACGAAGGTTTG
CACACCGACTTTGCATGCTTGTTGTTCGCCCATTTGAAGAACAAACCAGACCCAGCCATTGTTGAAAAAATTGTC
ACCGAGGCTGTGGAAATTGAACAAAGATACTTCTTGGACGCCTTACCAGTTGCTTTGCTAGGTATGAACGCTGAC
TTAATGAACCAATACGTTGAGTTCGTCGCCGACAGACTGTTGGTTGCTTTCGGTAACAAGAAATACTACAAGGTC
GAAAACCCCTTCGATTTCATGGAAAACATCTCCTTGGCCGGTAAGACCAACTTCTTCGAAAAGAGAGTTTCTGAC
TACCAAAAGGCTGGTGTTATGTCCAAGTCGACTAAGCAAGAAGCCGGTGCTTTCACCTTCAACGAAGACTTTTAA

>YHR126C 1.61 Hot
ATGAAGTGCACTTTAGTTTCCACATTGTTTGCCATCACCAATATTCTAGTTGCACATGCACAAGTAAGCAACTCC
TCAGACACGTTGGACGTACAATTTGCGAATAGTACGAACTCGTACATAGAAGGAAAATTTAATTCGACTGATGAA
GCCTTCAACAGCAGCGCATCTTGGTCCTTAGCAGCTCAGCAGAAAAAGATATCTAATGCAGCTGTATATGATGTG
GGTGGTTGGAATGGCTCATTGTATCGTTCCAATAGAAGCGCTGTTGCAGATCATCAACCCGGCAAAAAGCAAGAT
GCCGCTATTTCACAGATCAGTGATGGTCAAATCCAAGCCACTGCGTCTGGACCTGAGACTACCGCTGCTACTACC
CCAAGTAGTACCGCAAATGTCTCTGTCTATGAAGGTGCTGGTATGAAGGTTGAATCCAAGAACATGGGTTATATA
GTTGGAGTAGCAGCGCTATTATTTTTATAA

>YPL042C 1.65 Hot
ATGTATAATGGCAAGGATAGAGCACAAAACTCCTATCAGCCAATGTACCAAAGGCCTATGCAGGTACAAGGACAA
CAGCAAGCTCAATCGTTCGTTGGAAAGAAAAACACAATCGGAAGTGTGCATGGAAAAGCCCCGATGCTAATGGCC
AATAATGATGTTTTTACTATTGGACCTTATAGGGCAAGAAAAGATAGAATGCGGGTATCTGTCTTAGAAAAGTAC
GAAGTTATTGGCTACATTGCTGCGGGCACATATGGTAAAGTTTACAAAGCGAAAAGACAAATCAACTCCGGTACC
AATTCCGCTAATGGTTCTAGTCTGAATGGTACCAATGCGAAAATTCCGCAGTTTGACAGCACGCAACCAAAATCA
AGCTCTTCAATGGACATGCAGGCAAATACAAACGCATTAAGAAGAAACTTGTTAAAGGATGAAGGAGTGACCCCC
GGAAGAATACGAACTACGAGGGAAGATGTATCCCCGCACTATAATTCCCAAAAACAAACCCTCATTAAAAAACCG
CTGACGGTATTTTATGCCATTAAAAAGTTCAAGACAGAGAAGGATGGCGTCGAACAATTGCATTATACGGGAATA
TCTCAGAGTGCCTGTAGAGAAATGGCATTATGTCGAGAATTGCACAACAAGCATTTAACCACATTAGTGGAAATT
TTTTTGGAAAGGAAATGTGTCCATATGGTATACGAATATGCGGAGCATGATCTGCTACAAATTATCCACTTCCAT
TCCCATCCCGAAAAAAGGATGATACCACCAAGAATGGTTCGGTCTATTATGTGGCAGCTTTTAGACGGCGTATCG
TATCTTCATCAAAATTGGGTGCTTCATCGAGATTTGAAACCCGCAAATATAATGGTGACCATAGATGGATGTGTT
AAAATTGGTGATTTAGGTTTGGCCAGAAAATTTCATAATATGCTGCAAACCCTCTATACTGGGGATAAAGTGGTT
GTCACTATATGGTACCGTGCACCTGAGTTGCTATTGGGAGCACGGCACTATACCCCTGCGGTTGATTTATGGTCC
GTTGGCTGCATTTTTGCAGAACTGATAGGATTACAGCCCATATTTAAAGGTGAAGAAGCTAAACTAGACTCTAAA
AAGACTGTTCCATTTCAAGTGAATCAACTACAGAGAATTTTGGAAGTTCTTGGCACTCCCGATCAAAAAATTTGG
CCTTATTTGGAGAAGTATCCAGAATATGATCAAATTACGAAGTTTCCAAAGTATAGGGATAACCTTGCTACATGG
TATCATTCCGCGGGAGGAAGGGACAAGCATGCTTTAAGCTTACTTTACCACTTGTTAAATTATGATCCAATTAAA
AGAATAGATGCATTTAATGCGTTGGAACATAAGTACTTCACAGAAAGTGATATTCCTGTTAGTGAAAATGTATTT
GAAGGTCTAACTTACAAATACCCGGCAAGAAGAATTCACACGAACGATAATGACATCATGAATCTTGGATCAAGA
ACGAAAAACAATACACAAGCTTCAGGAATCACCGCAGGTGCCGCTGCAAATGCGTTAGGTGGGCTTGGTGTTAAC
CGTAGAATTCTGGCCGCGGCAGCAGCAGCCGCTGCTGCGGTGTCAGGAAACAATGCATCAGATGAGCCATCTCGA
AAGAAAAACAGAAGATAG

>YJL079C 2.26 Hot
ATGAAACTTTCTAAATTATCGATCTTAACCTCCGCCTTAGCCACGTCTGCATTGGCCGCTCCTGCCGTTGTTACT
GTCACTGAACACGCCCATGAGGCTGCAGTCGTTACTGTGCAAGGCGTAGTTTATGTCGAAAACGGCCAAACACGT

ACAACTTACGAAACACTAGCTCCTGCTTCCACTGCCACTCCAACTTCTACAGCTACAGCTTTGGTTGCTCCCCCT
GTCGCTCCTTCCTCTGCTTCCAGCAATTCCGATGTGGTCTTGTCTGCTTTGAAGAACTTAGCCTCTGTTTGGGGT
AAAACTACCGATTCAACGACCACGTTGACATCTTCTGAATCTACATCGCAATCGTTGGCTCAAGCTACTACGACT
TCTACACCCGCTGCTGCTTCCACCACTTCCACACCCGCTGCTACCACCACCACTTCTCAAGCCGCTGCTACTAGC
TCAGCATCGTCTTCGGATAGTGACCTGTCAGATTTTGCCTCTTCTGTGTTGGCTGAACACAACAAGAAGAGAGCT
TTGCACAAGGACACACCAGCTTTGTCCTGGTCCGATACTTTGGCCTCCTACGCTCAAGACTATGCTGACAACTAT
GATTGCTCCGGCACTTTGACCCATTCTGGCGGTCCATACGGTGAAAACTTGGCTTTGGGTTATGACGGCCCAGCT
GCCGTCGACGCTTGGTACAATGAAATTTCCAACTACGACTTCTCGAATCCAGGCTTTTCAAGTAACACTGGCCAC
TTTACTCAAGTCGTTTGGAAGTCCACCACCCAAGTTGGTTGTGGCATCAAAACCTGTGGCGGTGCATGGGGTGAC
TATGTCATCTGTAGTTACGACCCCGCAGGAAACTACGAAGGCGAATACGCCGATAATGTCGAGCCCCTAGCTTAA

>YDR050C 1.81 Hot
ATGGCTAGAACTTTCTTTGTCGGTGGTAACTTTAAATTAAACGGTTCCAAACAATCCATTAAGGAAATTGTTGAA
AGATTGAACACTGCTTCTATCCCAGAAAATGTCGAAGTTGTTATCTGTCCTCCAGCTACCTACTTAGACTACTCT
GTCTCTTTGGTTAAGAAGCCACAAGTCACTGTCGGTGCTCAAAACGCCTACTTGAAGGCTTCTGGTGCTTTCACC
GGTGAAAACTCCGTTGACCAAATCAAGGATGTTGGTGCTAAGTGGGTTATTTTGGGTCACTCCGAAAGAAGATCT
TACTTCCACGAAGATGACAAGTTCATTGCTGACAAGACCAAGTTCGCTTTAGGTCAAGGTGTCGGTGTCATCTTG
TGTATCGGTGAAACTTTGGAAGAAAAGAAGGCCGGTAAGACTTTGGATGTTGTTGAAAGACAATTGAACGCTGTC
TTGGAAGAAGTTAAGGACTGGACTAACGTCGTTGTCGCTTACGAACCAGTCTGGGCCATTGGTACCGGTTTGGCT
GCTACTCCAGAAGATGCTCAAGATATTCACGCTTCCATCAGAAAGTTCTTGGCTTCCAAGTTGGGTGACAAGGCT
GCCAGCGAATTGAGAATCTTATACGGTGGTTCCGCTAACGGTAGCAACGCCGTTACCTTCAAGGACAAGGCTGAT
GTCGATGGTTTCTTGGTCGGTGGTGCTTCTTTGAAGCCAGAATTTGTTGATATCATCAACTCTAGAAACTAA

>YAL013W 1.99 Hot
ATGAGTCAGCAAACACCACAGGAAAGTGAACAGACCACAGCGAAAGAACAGGACCTTGATCAAGAGAGCGTGTTG
AGCAACATTGACTTCAATACGGATTTGAATCACAATTTGAATTTATCGGAATACTGTATATCCAGTGACGCAGGA
ACAGAGAAGATGGATAGCGACGAGGAGAAGTCGTTGGCCAATCTGCCGGAGTTGAAATACGCTCCCAAGCTATCC
AGCCTGGTGAAGCAAGAGACGCTCACCGAGAGCTTGAAAAGACCACACGAAGATGAGAAAGAGGCGATAGATGAG
GCCAAGAAGATGAAAGTGCCGGGAGAGAACGAGGACGAAAGCAAGGAAGAGGAAAAGAGTCAAGAACTGGAAGAG
GCAATTGACAGCAAGGAGAAGAGCACCGACGCCAGGGACGAGCAAGGGGACGAAGGTGATAATGAGGAGGAAAAC
AACGAGGAGGATAATGAAAACGAAAACGAGCATACAGCACCGCCTGCGCTGGTGATGCCCTCCCCCATCGAAATG
GAGGAACAGAGGATGACTGCGCTGAAGGAAATCACCGACATCGAGTACAAGTTCGCGCAATTGCGCCAAAAACTA
TATGACAATCAATTGGTGCGGTTGCAAACGGAGCTGCAGATGTGTCTGGAAGGGTCACACCCGGAATTGCAGGTC
TACTACTCGAAGATTGCCGCGATCCGTGACTACAAGCTACACCGAGCGTACCAGCGACAGAAGTACGAGCTTTCA
TGCATCAACACAGAAACAATCGCTACCAGGACATTCATTCACCAGGACTTCCACAAGAAGGTCACCGACCTGCGA
GCCAGGCTGCTGAACAGAACCACGCAGACCTGGTACGATATCAACAAGGAGCGCCGCGATATGGATATAGTCATC
CCAGATGTCAATTACCACGTCCCCATCAAACTTGATAACAAGACGCTGAGCTGTATCACGGGCTACGCCAGCGCA
GCACAGCTGTGCTATCCCGGCGAGCCCGTGGCAGAGGACCTCGCTTGCGAAAGCATCGAGTACCGCTACAGAGCC
AACCCGGTGGACAAACTCGAAGTCATTGTGGACCGAATGAGGCTCAATAACGAGATTAGCGACCTCGAAGGCCTG
CGCAAATATTTCCACTCCTTCCCGGGTGCTCCTGAGTTGAACCCGCTTAGAGACTCCGAAATCAACGACGACTTC
CACCAGTGGGCCCAGTGTGACCGCCACACTGGACCCCATACCACTTCTTTTTGTTATTCTTAA

>YHR219W 1.61 Hot
ATGGACTTGAATCAAAGAAAGGAAAAAAAGGGCCAGCATGTTGGATGCTGTGGCTCCAGGACAGACCTGTCTGCT
GACACAGTGGAACTGATAGAAAGAATGGACAGATTGGCTGAAAATCAGGCGACAGCTTCCATGTCGATCGTTGCG
TTACCGTCTAGCTTCCAGGAGAGCAATAGCAGTGACAGGTGCAGAAAGTATTGCAGCAGTGATGAGGACAGCGAC
ACGTGCATTCATGGTAGTGCTAATGCCAGTACCAATGCGACTACCAACTCCAGCACTAATGCTACTACCACTGCC
AGCATCAACGTCAGGACTAGTGCGACTACCACTGCCAGCATCAACGTCAGGACTAGTGCGACTACCACTGAAAGT
ACCAACTCCAACACTAATGCTACTACCACTGAAAGTACCAACTCCAGCACTAATGCTACTACCACTGCCAGCATC
AACGTCAGGACTAGTGCGACTACCACTGAAAGTACCAACTCCAACACTAGTGCTACTACCACCGAAAGTACCGAC
TCCAACACTAGTGCTACTACCACCGAAAGTACCGACTCCAACACTAGTGCTACTACCACTGCTAGCACCAACTCC
AGCACTAATGCCACTACCACTGCTAGCACCAACTCCAGCACTAATGCCACTACCACTGAAAGTACCAACGCTAGT
GCCAAGGAGGACGCCAATAAAGATGGCAATGCTGAGGATAATAGATTCCATCCAGTCACCGACATTAACAAAGAG
TCGTATAAGCGGAAAGGGAGTCAAATGGTTTTGCTAGAGAGAAAGAAACTGAAAGCACAATTTCCCAATACTTCC
GAGAATATGAATGTCTTACAGTTTCTTGGATTTCGGTCTGACGAAATTAAACATCTTTTCCTCTATGGTATTGAC
ATATACTTCTGCCCAGAGGGAGTATTCACACAATACGGATTATGCAAGGGCTGTCAAAAGATGTTCGAGCTCTGT
GTCTGTTGGGCTGGCCAGAAAGTATCGTATCGGAGGATGGCTTGGGAAGCACTAGCTGTGGAGAGAATGCTGCGA
AATGACGAGGAATACAAAGAATACTTGGAAGACATCGAGCCATATCATGGGGACCCTGTAGGGTATTTGAAATTT
TTTAGCGTAAAAAGGGGAGAGATCTACTCTCAGATACAGAGAAATTATGCTTGGTACCTGGCCATTACTAGAAGA
AGAGAAACAATTAGTGTATTGGATTCGACAAGAGGCAAGCAAGGGAGCCAAGTTTTCCGCATGTCTGGAAGGCAG
ATCAAAGAGTTGTATTATAAAGTATGGAGCAACTTGCGTGAATCGAAGACAGAGGTGCTGCAGTACTTTTTGAAC
TGGGACGAAAAAAAGTGCCGGGAAGAATGGGAGGCAAAAGACGATACGGTCTTTGTGGAAGCGCTCGAGAAAGTT
GGAGTTTTTCAGCGTTTGCGTTCCATGACGAGCGCTGGACTGCAGGGTCCGCAGTACGTCAAGCTGCAGTTTAGC
AGGCATCATCGACAGTTGAGGAGCAGATATGAATTAAGTCTAGGAATGCACTTGCGAGATCAGCTTGCGCTGGGA
GTTACCCCATCTAAAGTGCCGCATTGGACGGCATTCCTGTCGATGCTGATAGGGCTGTTCTACAATAAAACATTT
CGGCAGAAACTGGAATATCTTTTGGAGCAGATTTCGGAGGTGTGGTTGTTACCACATTGGGTTGATTTGGCAAAC

GTTGAAGTTCTCGCTGCAGATAACACGAGGGTACCGCTGTACATGCTGATGGTAGCGGTTCACAAAGAGCTGGAT
AGCGATGATGTTCCAGACGGTAGATTTGATATAATATTACTATGTAGAGATTCGAGCAGAGAAGTTGGAGAGTGA

>YJR021C 1.54 Hot
ATGGTCGCTAGAGGTAGAACAGACGAGATATCTACAGATGTTTCAGAGGCTAATTCTGAACACTCGTTGATGATT
ACAGAAACATCATCACCGTTCAGATCTATATTCTCCCACAGTGGGAAAGTAGCGAATGCAGGCGCTTTAGAGGAA
TCGGACAAGCAGATACTGGAATGGGCAGGTAAACTGGAATTGGAGAGTATGGAGCTGAGGGAAAACTCTGACAAG
CTAATTAAAGTTTTAAATGAAAATTCTAAGACATTATGTAAATCGCTAAACAAGTTTAACCAGCTACTGGAACAA
GATGCTGCTACGAACGGAAACGTGAAAACCTTAATAAAGGATTTGGCTTCCCAGATTGAAAACCAACTGGACAAA
GTGTCAACAGCAATGTTATCGAAAGGCGACGAGAAGAAGACAAAGTCGGATTCCAGCTACAGGCAGGTATTAGTC
GAAGAAATAAGCAGGTATAATTCCAAGATAACGCGACACGTAACTAACAAGCAGCATGAGACGGAAAAGTCGATG
AGGTGCACCCAGGAGATGCTCTTCAACGTCGGCAGCCAACTGGAGGACGTGCATAAAGTGCTCCTGTCTCTATCC
AAAGACATGCACAGTCTACAAACCCGCCAGACTGCTCTAGAGATGGCGTTTCGAGAAAAGGCAGATCACGCCTAC
GATCGCCCGGATGTGTCTCTGAATGGCACCACACTCCTGCACGATATGGACGAAGCCCACGATAAGCAGCGTAAG
AAGTCCGTGCCGCCACCAAGAATGATGGTCACAAGGTCCATGAAACGCAGAAGATCCAGCTCCCCAACCCTATCC
ACCAGCCAAAACCACAATAGCGAAGACAATGATGACGCTAGCCATCGGCTGAAGCGTGCGGCCAGGACCATTATT
CCCTGGGAGGAACTAAGACCCGACACTCTGGAATCTGAGCTGTGA

>YML121W 1.65 Hot
ATGTCGTCAAATAATAGGAAGAAACTGCTTCTGATGGGCCGGTCCGGCTCCGGTAAATCGTCAATGAGGTCGATC
ATCTTTAGTAACTACTCCGCTTTTGACACTAGGAGATTGGGTGCCACCATTGATGTAGAGCACTCCCATTTGAGA
TTTCTTGGGAATATGACTCTAAATCTGTGGGACTGTGGTGGGCAGGACGTGTTTATGGAGAATTATTTCACCAAG
CAAAAAGACCACATTTTCCAGATGGTGCAGGTGTTAATTCACGTTTTTGATGTAGAGTCAACTGAAGTTCTCAAG
GATATTGAAATATTTGCAAAAGCTTTGAAGCAATTAAGGAAGTACTCTCCCGACGCCAAAATTTTTGTTCTTCTG
CATAAGATGGATCTTGTTCAGTTGGATAAGAGAGAGGAGCTGTTCCAAATCATGATGAAAAACCTGAGTGAAACG
TCTTCGGAATTTGGGTTTCCCAATCTGATAGGTTTTCCTACTTCGATTTGGGATGAGAGTTTATACAAGGCATGG
TCGCAGATTGTATGCTCGCTAATACCCAATATGTCCAACCATCAAAGTAATTTGAAGAAGTTTAAGGAGATTATG
AACGCCCTTGAAATTATTCTTTTCGAAAGAACAACTTTCTTAGTGATATGCTCCAGTAATGGCGAAAATAGTAAT
GAAAATCATGATAGTTCGGATAATAATAATGTCTTGCTAGACCCGAAGCGATTCGAAAAGATATCCAATATAATG
AAAAACTTCAAGCAGAGTTGCACGAAATTGAAGAGCGGATTCAAGACTTTAATATTGAACAACAACATCTACGTC
AGCGAGTTATCGTCCAATATGGTGTGTTTTATAGTGTTGAAAGATATGAATATTCCACAAGAATTAGTATTGGAA
AACATCAAAAAAGCCAAAGAGTTTTTCCAATGA

>YPR061C 1.54 Hot
ATGCTACACCATAAGTTCGTATACCCATTTTTATTCAAGTGGCACTTATCATGTGTAGAAAAGTGTCCCCCACAA
ATCACTTTTATAGCTAAGTATGCTACAGCGAACGATAAAAATGGCAATAGAAAACTTACGATAAGGGATGAACAA
TGGCCTGAGTTGGCAGATCCAACTCCCTATGATATTTTTGGCATTCCAAAGGCCGGATCTGGAAATCCTAAACTG
GACAAGAAGTCGTTAAAAAAAAAATATCATCGTTATGTAAAATTGTACCACCCTGACCATTCCGATAACATTCAA
ATATTTAGCTCAGAAAAGGTTACCAACAGTGATAGTAAATCACCGCTGCTGCTAACATCAAGCGAAAAACTACAT
AGATTTAAAGTCATCTCTCAAGCATATGATATTCTTTGTGACCCAAAGAAAAAGATCGTATATGACACAACGAGG
CAAGGCTGGACCACATCGTATTCACCACGTTCTAACGTTAATACTGAAAATTACCAATATGCCGGCTCTTATGGC
TACCACTCTAACGCGCAGTATGAATACTGGAACGCTGGGACTTGGGAAGACGCAAATAGCATGAAAAACGAAAGA
ATTCAAGAAAACATCAACCCATGGACCGTTATTGGCATAATTTGTGGCCTAGCTATATGCATCGAAGGGACTGCG
TTGTTAGCCAAAATCCAGGAGTCTCTGAGCAAGGCCGAATTTACTCATGACGAAAGTGGATTACATTTGATTCAG
TCATACACGAATTATGGTCTTGATACTGACAAATTTTCCAGATTGAGGCGGTTCTTATGGTTTAGAACTTGGGGA
CTTTACAAGTCGAAAGAGGATTTAGATAGAGAAGCCAAGATCAATGAAGAAATGATACGCAAACTGAAAGCAGCT
AAATGA

>YGR078C 2.34 Hot
ATGGACACACTGTTCAACTCCACGGAAAAGAATGCCAGAGGCATACCGCAGGCTCCATTCATCGAAAATGTCAAC
GAAATAATTAAGGACCCAAGCGACTTCGAATTGTGCTTCAATAAGTTCCAAGAACGGCTTTCTAAGTACAAATTT
ATGCAGGAATCTAAATTGGCTACGATAAAGCAGCTGAAGACTAGAATCCCGGACTTGGAAAACACATTAAAAATA
TGCCAAAGTCTGCGCAATCACAGCGACGAGGGCGATGAGAGCGATGAACCTATCCTGTTGCACTACCAATTGAAC
GACACGCTCTACACAAAGGCTCAGGTTGATATACCGGAAGATCGGGCGGATTTGAAAGTTGGGTTGTGGCTTGGC
GCAGACGTGATGCTGGAATATCCCATCGACGAGGCCATAGAGTTGTTAAAGAAGAAACTCGCGGATTCCGAACAG
TCTTTAACGGTGTCCACCGAGGACGTAGAGTTTTTGAGGGAAAATATCACGACAATGGAGGTCAATTGCGCGCGA
TTGTATAACTGGGACGTTCAGAGAAGGCAAGACTTGAAGCAGGCTCAAGAAGGGACTAAAAACCTCAAGATATAA

>YJL120W 1.64 Hot
ATGACATTCGCAACCCAAGTTGGCGAAGTCAGAAGCAAGGATACTGGGAGCTATAATTGGTTTGACCATTTTTTC
TTGTGTGTTTACCTCGCTCTTGGAATTAGCAAATGGCCTTCTTGCATGAAATTGTATCGAGTTTGCTTTATTTTT
CTTTTTACGGGCGGATTCTTTCTATTCTGGCTTTCCTATAACAGAGATCATGAAAGAAGTTCCAGCTTACGGATC
AAGAAAGTACCTATACATATACAAAAATCTGATTACTTTCCCAGCTCGACTTGGATAGCTGTTCTTGTTTTCTCT
TGGCGACACATTTTTTTGTTTCTGA

>YAL043C 1.6Hot

ATGTCATCTGCAGAGATGGAACAATTGTTACAGGCCAAGACACTGGCCATGCACAACAATCCAACGGAGATGCTG
CCCAAGGTGCTCGAAACTACGGCATCCATGTACCACAACGGTAATCTCAGCAAGCTGAAGTTGCCTTTGGCCAAG
TTTTTTACACAGTTAGTTCTAGACGTGGTGTCGATGGACTCTCCAATTGCGAATACTGAGAGACCGTTTATTGCT
GCTCAATATCTGCCACTACTTCTTGCTATGGCGCAATCCACCGCGGACGTACTAGTGTACAAGAATATCGTGCTT
ATTATGTGCGCTTCATACCCGCTGGTGTTGGATCTGGTTGCTAAGACATCAAACCAGGAAATGTTTGATCAGTTG
TGTATGCTGAAGAAGTTCGTGCTCTCGCACTGGAGAACTGCATATCCTTTGCGTGCCACCGTTGACGATGAAACG
GATGTCGAACAATGGCTGGCGCAGATTGACCAAAATATCGGCGTGAAATTAGCGACCATCAAGTTCATATCTGAG
GTCGTGCTGTCGCAAACTAAATCACCCAGCGGCAACGAGATTAATTCATCTACCATCCCGGATAACCACCCTGTG
TTGAACAAACCGGCTTTGGAGAGCGAGGCTAAGAGGCTTCTTGATATGTTGCTAAACTACCTAATTGAGGAACAG
TACATGGTCTCGTCCGTTTTCATTGGTATCATCAATTCTTTATCCTTCGTCATCAAAAGAAGGCCGCAGACAACA
ATAAGAATTCTTTCCGGGCTGTTGCGTTTCAACGTCGACGCCAAGTTTCCCCTAGAGGGCAAGTCTGACTTGAAC
TACAAACTATCCAAGAGATTTGTTGAAAGGGCGTACAAGAACTTTGTGCAATTTGGGCTAAAAAATCAAATCATT
ACAAAATCCCTCTCATCCGGATCAGGGTCATCGATCTACTCCAAGCTGACCAAGATTTCTCAAACTTTACACGTT
ATTGGCGAAGAGACCAAGAGCAAGGGAATTTTGAACTTCGACCCTTCCAAGGGCAATAGCAAGAAAACGTTGTCC
AGGCAGGACAAACTAAAATACATCTCACTATGGAAAAGGCAATTATCCGCGTTATTGTCTACTCTAGGGGTGTCC
ACAAAGACCCCCACGCCTGTGTCCGCACCTGCAACGGGCTCTTCAACCGAAAACATGCTTGATCAACTGAAGATA
TTGCAAAAATACACCCTCAACAAGGCTTCACACCAGGGCAATACTTTTTTCAACAACTCACCCAAACCAATCAGC
AACACCTACTCATCTGTGTACTCATTGATGAACAGTTCGAACTCCAACCAGGATGTGACCCAGCTACCCAATGAC
ATACTTATCAAGCTGTCCACAGAGGCCATCTTGCAAATGGACAGCACGAAACTGATCACCGGATTGTCTATCGTT
GCTTCGAGGTACACGGATTTAATGAATACGTACATCAATTCTGTACCGTCCTCGTCATCATCAAAGAGGAAATCC
GACGATGATGACGACGGCAACGACAATGAAGAAGTTGGAAACGATGGCCCAACGGCTAATAGCAAGAAAATCAAA
ATGGAAACAGAACCACTAGCGGAGGAACCAGAGGAGCCCGAAGACGATGACCGAATGCAGAAGATGCTTCAAGAA
GAGGAAAGCGCCCAAGAAATCTCAGGAGATGCCAACAAATCAACTTCTGCCATTAAGGAGATCGCACCCCCCTTT
GAACCTGACTCATTGACGCAGGATGAAAAACTAAAGTACCTCTCAAAGCTGACCAAGAAACTGTTTGAATTATCC
GGTCGCCAGGATACTACCCGGGCCAAATCTTCGTCTTCCTCCTCCATATTACTGGACGATGACGACTCCTCGTCA
TGGTTACACGTCTTAATCAGATTGGTTACGAGAGGAATCGAAGCACAAGAGGCCAGTGACCTGATTCGTGAAGAA
CTGCTTGGCTTCTTCATCCAGGATTTCGAGCAACGTGTCAGTCTGATCATTGAATGGCTCAATGAAGAATGGTTC
TTCCAAACCTCGCTGCATCAAGATCCCTCTAACTACAAAAAATGGTCCTTAAGAGTTCTCGAGTCTCTGGGTCCA
TTCCTTGAAAACAAACACAGACGATTCTTCATCAGACTTATGAGCGAACTGCCCAGTCTTCAAAGCGATCATCTT
GAGGCACTGAAGCCTATCTGCCTGGATCCGGCAAGAAGTTCCCTTGGTTTCCAAACGCTAAAGTTTCTCATTATG
TTTAGACCCCCAGTGCAGGACACTGTTCGCGACCTGCTGCATCAGCTAAAGCAAGAAGATGAAGGCTTACACAAG
CAGTGCGATTCACTGCTTGACAGGCTAAAATGA

>YBL095W 1.6Hot
ATGTCCAGAACTATTCCATTTCTATTTAAATTAGTCAACAGGGCAGTAATTTTGCCTACGGCAGGTTTTACATTA
GGAGTTGGTGCGTTTGTAAAGGCGTGGCCCGATGATGCCGGTGTTCTATCATTGAATGATCCGCAAACGCCAGCG
GAGTTGATTAGTGCGACCAAGAGCCGCCAACCTATGGAGCTGCAGAGGGTTGACATCCTCGCTCAAATCGAGAAA
AGCGAGGTTTACAACAAGTTGGCCCAGGATGAGAAGATGCACCATGTCTTATTCAGTGAGAAAATACCAAGCGGG
CATAGGGAATATCATGTAGGACAAGGCCTCTTGTTCGGCAAGGGGAAGCTTGAAATTGATCCTTTGGTGTTCCAT
GATGTGAATCACGGTGAATTAACCGTGATTTATCACTTAGGTGCTGAGTTAGGGAATCGAGACGGTAACGTCCAT
AAGGGCTTGTTGTCATTGTTGCTGGATGAAGCATTGTGCTATTGTGGCTTCCCTTTGTTGCCTAGTAAAAGAGGT
GTAACTGCAAGGCTGTCGCTAGAGTTTTTTGAGGACATTCCTGTAGATACTACGATTATACTAAAAGCAAACGTC
AAAGAGATTAAAGGCAGAAAGTGTATCATTGAGGGACACTTGGAACAGTTTCCGTTGGAAGTTTCTTCTCGAAAT
GGAACTAGAAGTTGGAACTTACCACATATTTGGGGTTTCAACCATAAGCAGGAGATGGCAAAAAAATTTGCCAAG
GCCAATTGTATTCTCGTTGAGCCTACTTGGTTCAAATATTTTAAATGGCTTGATATGTTTTGA

>YIL053W 1.7Hot
ATGCCTTTGACCACAAAACCTTTATCTTTGAAAATCAACGCCGCTCTATTCGATGTTGACGGTACCATCATCATC
TCTCAACCAGCCATTGCTGCTTTCTGGAGAGATTTCGGTAAAGACAAGCCTTACTTCGATGCCGAACACGTTATT
CACATCTCTCACGGTTGGAGAACTTACGATGCCATTGCCAAGTTCGCTCCAGACTTTGCTGATGAAGAATACGTT
AACAAGCTAGAAGGTGAAATCCCAGAAAAGTACGGTGAACACTCCATCGAAGTTCCAGGTGCTGTCAAGTTGTGT
AATGCTTTGAACGCCTTGCCAAAGGAAAAATGGGCTGTCGCCACCTCTGGTACCCGTGACATGGCCAAGAAATGG
TTCGACATTTTGAAGATCAAGAGACCAGAATACTTCATCACCGCCAATGATGTCAAGCAAGGTAAGCCTCACCCA
GAACCATACTTAAAGGGTAGAAACGGTTTGGGTTTCCCAATTAATGAACAAGACCCATCCAAATCTAAGGTTGTT
GTCTTTGAAGACGCACCAGCTGGTATTGCTGCTGGTAAGGCTGCTGGCTGTAAAATCGTTGGTATTGCTACCACT
TTCGATTTGGACTTCTTGAAGGAAAAGGGTTGTGACATCATTGTCAAGAACCACGAATCTATCAGAGTCGGTGAA
TACAACGCTGAAACCGATGAAGTCGAATTGATCTTTGATGACTACTTATACGCTAAGGATGACTTGTTGAAATGG
TAA

>YKL035W 1.85 Hot
ATGTCCACTAAGAAGCACACCAAAACACATTCCACTTATGCATTCGAGAGCAACACAAACAGCGTTGCTGCCTCA
CAAATGAGAAACGCCTTAAACAAGTTGGCGGACTCTAGTAAACTTGACGATGCTGCTCGCGCTAAGTTTGAGAAC
GAACTGGATTCGTTTTTCACGCTTTTCAGGAGATATTTGGTAGAGAAGTCTTCTAGAACCACCTTGGAATGGGAC
AAGATCAAGTCTCCCAACCCGGATGAAGTGGTTAAGTATGAAATTATTTCTCAGCAGCCCGAGAATGTCTCAAAC
CTTTCCAAATTGGCTGTTTTGAAGTTGAACGGTGGGCTGGGTACCTCCATGGGCTGCGTTGGCCCTAAATCTGTT
ATTGAAGTGAGAGAGGGAAACACCTTTTTGGATTTGTCTGTTCGTCAAATTGAATACTTGAACAGACAGTACGAT
AGCGACGTGCCATTGTTATTGATGAATTCTTTCAACACTGACAAGGATACGGAACACTTGATTAAGAAGTATTCC

GCTAACAGAATCAGAATCAGATCTTTCAATCAATCCAGGTTCCCAAGAGTCTACAAGGATTCTTTATTGCCTGTC
CCCACCGAATACGATTCTCCACTGGATGCTTGGTATCCACCAGGTCACGGTGATTTGTTTGAATCTTTACACGTA
TCTGGTGAACTGGATGCCTTAATTGCCCAAGGAAGAGAAATATTATTTGTTTCTAACGGTGACAACTTGGGTGCT
ACCGTCGACTTAAAAATTTTAAACCACATGATCGAGACTGGTGCCGAATATATAATGGAATTGACTGATAAGACC
AGAGCCGATGTTAAAGGTGGTACTTTGATTTCTTACGATGGTCAAGTCCGTTTATTGGAAGTCGCCCAAGTTCCA
AAAGAACACATTGACGAATTCAAAAATATCAGAAAGTTTACCAACTTCAACACGAATAACTTATGGATCAATCTG
AAAGCAGTAAAGAGGTTGATCGAATCGAGCAATTTGGAGATGGAAATCATTCCAAACCAAAAAACTATAACAAGA
GACGGTCATGAAATTAATGTCTTACAATTAGAAACCGCTTGTGGTGCTGCTATCAGGCATTTTGATGGTGCTCAC
GGTGTTGTCGTTCCAAGATCAAGATTCTTGCCTGTCAAGACCTGTTCCGATTTGTTGCTGGTTAAATCAGATCTA
TTCCGTCTGGAACACGGTTCTTTGAAGTTAGACCCATCCCGTTTTGGTCCAAACCCATTAATCAAGTTGGGCTCG
CATTTCAAAAAGGTTTCTGGTTTTAACGCAAGAATCCCTCACATCCCAAAAATCGTCGAGCTAGATCATTTGACC
ATCACTGGTAACGTCTTTTTAGGTAAAGATGTCACTTTGAGGGGTACTGTCATCATCGTTTGCTCCGACGGTCAT
AAAATCGATATTCCAAACGGCTCCATATTGGAAAATGTTGTCGTTACTGGTAATTTGCAAATCTTGGAACATTGA

>YJL153C 1.85 Hot
ATGACAGAAGATAATATTGCTCCAATCACCTCCGTTAAAGTAGTTACCGACAAGTGCACGTACAAGGACAACGAG
CTGCTCACCAAGTACAGCTACGAAAATGCTGTAGTTACGAAGACAGCTAGTGGCCGCTTCGATGTAACGCCCACT
GTTCAAGACTACGTGTTCAAACTTGACTTGAAAAAGCCGGAAAAACTAGGAATTATGCTCATTGGGTTAGGTGGC
AACAATGGCTCCACTTTAGTGGCCTCGGTATTGGCGAATAAGCACAATGTGGAGTTTCAAACTAAGGAAGGCGTT
AAGCAACCAAACTACTTCGGCTCCATGACTCAATGTTCTACCTTGAAACTGGGTATCGATGCGGAGGGGAATGAC
GTTTATGCTCCTTTTAACTCTCTGTTGCCCATGGTTAGCCCAAACGACTTTGTCGTCTCTGGTTGGGACATCAAT
AACGCAGATCTATACGAAGCTATGCAGAGAAGTCAAGTTCTCGAATATGATCTGCAACAACGCTTGAAGGCGAAG
ATGTCCTTGGTGAAGCCTCTTCCTTCCATTTACTACCCTGATTTCATTGCAGCTAATCAAGATGAGAGAGCCAAT
AACTGCATCAATTTGGATGAAAAAGGCAACGTAACCACGAGGGGTAAGTGGACCCATCTGCAACGCATCAGACGC
GATATCCAGAATTTCAAAGAAGAAAACGCCCTTGATAAAGTAATCGTTCTTTGGACTGCAAATACTGAGAGGTAC
GTAGAAGTATCTCCTGGTGTTAATGACACCATGGAAAACCTCTTGCAGTCTATTAAGAATGACCATGAAGAGATT
GCTCCTTCCACGATCTTTGCAGCAGCATCTATCTTGGAAGGTGTCCCCTATATTAATGGTTCACCGCAGAATACT
TTTGTTCCCGGCTTGGTTCAGCTGGCTGAGCATGAGGGTACATTCATTGCGGGAGACGATCTCAAGTCGGGACAA
ACCAAGTTGAAGTCTGTTCTGGCCCAGTTCTTAGTGGATGCAGGTATTAAACCGGTCTCCATTGCATCCTATAAC
CATTTAGGCAATAATGACGGTTATAACTTATCTGCTCCAAAACAATTTAGGTCTAAGGAGATTTCCAAAAGTTCT
GTCATAGATGACATCATCGCGTCTAATGATATCTTGTACAATGATAAACTGGGTAAAAAAGTTGACCACTGCATT
GTCATCAAATATATGAAGCCCGTCGGGGACTCAAAAGTGGCAATGGACGAGTATTACAGTGAGTTGATGTTAGGT
GGCCATAACCGGATTTCCATTCACAATGTTTGCGAAGATTCTTTACTGGCTACGCCCTTGATCATCGATCTTTTA
GTCATGACTGAGTTTTGTACAAGAGTGTCCTATAAGAAGGTGGACCCAGTTAAAGAAGATGCTGGCAAATTCGAG
AACTTTTATCCAGTTTTAACCTTCTTGAGTTACTGGTTAAAAGCTCCATTAACAAGACCAGGATTTCACCCGGTG
AATGGCTTAAACAAGCAAAGAACCGCCTTAGAAAATTTTTTAAGATTGTTGATTGGATTGCCTTCTCAAAACGAA
CTAAGATTCGAAGAGAGATTGTTGTAA

>YBR214W 2.14 Hot
ATGGCTTCTACTTCGAACACGTTCCCTCCAAGCCAAAGCAATTCTTCCAACAACCTTCCAACTTCTAGACATGCA
TCCATTGTGGAGATGCTGTCTACTCCGCCATTGCTGCCCCACGTTCAAGTGAACGATACAGACGACAAGGAACAA
CCAGAAGAGTCCACGCCGCCAACAGCAACAGCGGCAGCGCCTGGTCCAGGTTGTGCTGCTACACCCGCCCCCTTG
CGCGATGAAAAGCCTCAATTCAAACTATCAGCTGTTCCTATGACCCAGACGCCATCGCAGTGTCTGTCATGTGTG
CACGCCCAAAAATGGCAACACATACCGTTGTCGCAATTGATCGAACAGAATAAGCTTATTTTTGTTCCAGGTTCC
ATATCGGTAGAGGAGGCATTCAACACTTTGATCAAGTATCATCTAAACTCTATTCCCGTGGAATCTTTCCCCGGC
GACATGAACTGTTTTACTTTCGACTACAATGACCTTAATTCGTACCTTTTGCTGGTTTTGAATAAAATCACCGTC
AGCAACAAACAGCTTACTGCAGATTGCCAGAATGGAAAACCCGTACCAGTGGGCGAAATGGTGAAACTAACTCCT
AAGAATCCGTTCTATAAGCTGCCGGAGAATGAGAGTTTGTCCACGGTGATGGGGATTCTCGGTTCGGGTGTCCAT
CGTGTAGCGATAACAAACGAAGAGATGACCAAAGTTAAAGGTATTTTGTCCCAACGTCGTTTGATAAAGTATCTT
TGGGACAACGCTAGATCTTTTACAAGCTTAGAACCTCTATTAAACTCCTCATTACAAGATTTGCACATCGGTGTT
CTCAATATTCAATCTAAGCCAACTTCAAGACAATCCCGTGTCATCTCTATTCAAGGCGAGGAGCCCTTAATCATG
GGCCTTTATAAAATGCATGTGGAAAGAATTTCTTCCATTGCAGTCATCGACAAACAAGGTAATTTGCTAGGTAAC
ATATCGGTAACGGACGTAAAACATGTCACAAGAACCTCCCAATACCCCTTACTACATAAGACATGTCGCCATTTC
ATTTCTGTCATTTTAAACTCCAGAGGATTAGAAACGGGTAAAGATTCTTTCCCCATTTTTCACGTATACCCCAGT
AGTTCACTAGCAAGGACTCTTGCCAAGTTAGTTGCTACAAAGTCTCATAGACTATGGATCGTACAACCGCCGGAG
TCGTCAACCTCGGCGTCATCCACAAATTTAACAGCTGCTAATACTGCAGCCAATGCAGTTTCTGCCACCGCTCAA
TCGTCTGCTAATGGTGCGACTCCAATGTCAAAGTCATCCTCTTCAACATCCCTCAACTCCCACTCTCCGTTGATG
ACAGCAATGGAAGACCCACCATCCCCACGTTCTTCGGCCATCGCGATCCCACCACCAAGTCCCGCTTCCTCAACA
AACACACCAAACCTATTCGAAAAGGAATATAGAACGGGTAAGTTGATCGGTGTGGTCTCATTGACAGACATCATC
AACTTATTAGCAAGGAAACAAACAGGGAATAAAGAAGTGGATCCGCAATCCGCAAGAAGACAGAGAGGCAGTATC
GCCATGTGA

>YFR033C 2.22 Hot
ATGGGCATGTTGGAACTAGTTGGTGAGTACTGGGAACAACTAAAGATAACCGTTGTGCCTGTTGTGGCCGCGGCC
GAAGATGACGATAACGAGCAGCATGAAGAAAAGGCAGCAGAAGGAGAAGAAAAAGAAGAAGAAAATGGGGATGAA
GATGAGGATGAAGACGAAGACGAAGATGATGATGATGATGACGACGAAGATGAGGAAGAAGAGGAAGAAGTCACT
GATCAGTTGGAAGATTTGAGAGAACATTTCAAGAACACGGAGGAGGGTAAGGCCCTTGTGCACCACTACGAGGAG

TGTGCTGAGAGAGTCAAGATACAGCAACAACAACCCGGCTACGCGGATCTTGAACACAAGGAGGACTGTGTGGAG
GAGTTTTTCCATCTACAGCACTATTTGGACACTGCCACGGCACCTAGATTATTTGACAAATTAAAGTAG

>YIL123W 1.56 Hot
ATGAAATTCTCAACTGCCGTTACTACGTTGATTAGTTCTGGTGCCATCGTGTCTGCTTTACCACACGTGGATGTT
CACCAAGAAGATGCCCACCAACATAAGAGGGCCGTTGCGTACAAATACGTTTACGAAACTGTTGTTGTCGATTCT
GATGGCCACACTGTAACTCCTGCTGCTTCAGAAGTCGCTACTGCTGCTACCTCTGCTATCATTACAACATCTGTG
TTGGCTCCAACCTCCTCCGCAGCCGCTGGGATAGCCGCTTCCATTGCTGTTTCATCTGCTGCCTTAGCCAAGAAT
GAGAAAATCTCTGATGCCGCTGCATCTGCCACTGCCTCAACATCTCAAGGGGCATCCTCCTCCTCCTCCTCCTCC
TCGGCAACTTCTACCCTAGAAAGCAGCTCTGTTTCTTCATCTAGTGAAGAAGCTGCTCCAACATCTACTGTCGTG
TCAACTTCTTCCGCAACCCAATCTAGTGCTTCTTCTGCCACTAAATCTAGTACTTCTTCCACTTCACCATCTACT
TCTACTTCTACTTCCACTTCTTCTACTTCCTCTTCCTCTTCCTCCTCCTCCTCCTCTTCTTCTTCTTCTTCTGGC
AGTGGTAGTATCTACGGTGATTTGGCCGACTTTTCAGGCCCAAGTGAGAAATTCCAAGACGGCACTATTCCATGT
GACAAATTCCCATCTGGTCAAGGTGTCATTTCTATTGACTGGATTGGCGAGGGTGGATGGTCCGGTGTGGAAAAC
ACCGACACTTCCACTGGCGGTTCATGCAAGGAGGGGTCCTACTGTTCCTACTCCTGCCAACCAGGTATGTCTAAG
ACCCAATGGCCATCCGATCAACCATCTGACGGTAGATCTGTCGGGGGTTTGTTGTGTAAAAATGGTTATTTGTAC
CGTTCTAACACTGACGCGGATTACTTATGTGAATGGGGTGTCGAGGCTGCCTATGTTGTTTCTAAACTAAGCAAG
GGTGTCGCCATTTGCAGAACCGACTACCCGGGCACTGAAAACATGGTTATCCCAACCTATGTTGAAGGGGGTAGC
TCTTTGCCATTGACCGTTGTTGACCAAGATACTTACTTTACTTGGGAAGGCAAAAAGACATCTGCTCAATACTAC
GTTAATAACGCCGGCGTCTCAGTTGAAGATGGGTGTATCTGGGGTACTTCTGGATCTGGTATTGGTAACTGGGCA
CCATTAAACTTTGGTGCTGGCTCCACTGGTGGAGTGACATACTTATCATTGATTCCTAACCCAAACAACAGCGAC
GCATTGAACTACAACGTCAAGATAGTTGCTGCTGATGATTCATCCAATGTCATCGGTGAATGTGTTTACGAAAAT
GGTGAGTTCTCTGGCGGTGCTGACGGGTGTACCGTCTCTGTTACTTCCGGTAAAGCTCATTTCGTCTTATACAAT
TAA

>YEL047C 1.76 Hot
ATGTCTCTCTCTCCCGTTGTTGTTATTGGAACCGGTTTGGCCGGGCTGGCTGCTGCCAATGAATTGGTTAACAAG
TATAACATCCCTGTAACCATCCTCGAAAAGGCTTCCTCGATCGGTGGGAACTCTATCAAGGCCTCCAGTGGTATT
AACGGTGCTTGCACCGAGACTCAACGTCACTTCCACATCGAGGACTCCCCACGCTTATTTGAAGATGACACCATC
AAGTCTGCTAAAGGTAAAGGTGTCCAAGAATTAATGGCTAAGTTGGCCAATGATTCTCCCCTGGCTATTGAATGG
TTGAAAAACGAATTTGATTTGAAATTGGACCTATTGGCTCAATTGGGTGGCCACTCTGTGGCAAGAACTCACAGA
TCGTCTGGGAAGTTGCCTCCAGGTTTCGAAATTGTTTCTGCCTTATCTAACAATTTGAAGAAATTAGCTGAGACT
AAACCAGAGTTAGTTAAGATTAACTTAGACAGTAAAGTCGTAGACATCCATGAAAAGGATGGCTCCATTTCTGCT
GTAGTGTACGAGGACAAGAATGGCGAAAAGCACATGGTGAGTGCTAACGATGTCGTTTTTTGTTCTGGAGGGTTT
GGCTTTTCTAAGGAAATGCTTAAAGAATACGCACCCGAACTGGTGAACTTGCCAACAACAAACGGGCAACAAACA
ACTGGTGATGGTCAAAGGCTTCTGCAGAAGTTAGGCGCTGATCTGATTGACATGGACCAAATTCAAGTTCATCCA
ACTGGGTTCATTGATCCAAATGACCGTAGCTCAAGCTGGAAATTCTTGGCTGCCGAATCCTTAAGAGGTCTTGGT
GGTATCCTATTAAACCCTATTACCGGTAGAAGATTTGTCAACGAATTGACCACAAGAGATGTAGTCACTGCAGCT
ATTCAAAAGGTTTGTCCTCAAGAGGATAACAGAGCACTATTGGTTATGGGCGAAAAAATGTACACAGATTTGAAG
AATAATTTAGATTTTTACATGTTCAAGAAACTTGTACAGAAATTGACATTATCTCAAGTTGTTTCTGAATATAAT
TTACCAATCACTGTCGCCCAATTATGCGAGGAATTGCAAACATACTCTTCCTTCACTACCAAGGCTGATCCGTTG
GGACGTACCGTTATTCTCAACGAATTTGGCTCTGACGTTACTCCAGAAACTGTGGTTTTTATTGGTGAAGTAACA
CCGGTTGTCCATTTCACCATGGGTGGTGCTAGAATCAATGTCAAGGCTCAAGTCATTGGCAAGAACGACGAAAGG
CTACTAAAAGGCCTGTACGCGGCCGGTGAAGTTTCTGGCGGTGTTCATGGCGCCAATAGGTTGGGTGGTTCAAGT
TTGTTAGAATGCGTTGTCTTTGGGAGAACCGCAGCTGAATCTATTGCCAATGACCGCAAGTAA

>YIL057C 1.82 Hot
ATGACGAAAAAGGATAAGAAAGCAAAGGGTCCTAAGATGTCCACCATCACTACAAAAAGTGGTGAGTCCTTAAAG
GTTTTTGAGGATTTGCATGATTTTGAAACATATTTAAAGGGTGAGACGGAAGATCAAGAGTTCGACCATGTCCAT
TGCCAACTGAAGTACTATCCACCCTTTGTCCTGCATGATGCGCATGATGATCCGGAAAAGATCAAAGAGACTGCC
AATTCGCACTCTAAGAAGTTTGTTCGCCATTTACACCAGCATGTTGAGAAGCACCTGCTAAAGGACATCAAAACC
GCTATCAACAAGCCAGAATTGAAATTCCACGATAAGAAAAAGCAGGAATCCTTTGACCGGATTGTTTGGAATTAT
GGCGAAGAAACGGAGTTGAACGCCAAGAAATTCAAGGTGTCTGTCGAAGTTGTATGTAAACACGATGGCGCAATG
GTAGATGTTGATTACAAGACAGAACCCTTGCAGCCACTCATCTAA

>YBL045C 1.56 Hot
ATGCTAAGAACAGTAACTTCAAAGACTGTATCTAACCAGTTCAAGAGGTCTTTGGCTACAGCTGTAGCAACCCCC
AAGGCCGAAGTAACGCAATTATCTAACGGTATAGTTGTAGCCACTGAGCATAATCCTTCCGCTCACACAGCCTCT
GTCGGTGTTGTCTTCGGCTCCGGTGCTGCCAACGAAAACCCTTATAACAACGGGGTTTCCAATTTATGGAAGAAC
ATCTTTCTATCCAAAGAAAACTCTGCTGTTGCCGCCAAGGAAGGTTTGGCATTGTCTTCCAATATCTCCAGAGAC
TTCCAATCTTACATTGTATCTTCTTTGCCAGGTTCTACCGATAAATCACTAGACTTCTTGAATCAGTCTTTCATC
CAACAAAAGGCTAATTTGCTATCTTCTTCTAACTTCGAGGCCACGAAGAAATCTGTCTTGAAGCAAGTTCAAGAT
TTTGAAGAAAACGACCATCCCAACAGAGTTTTGGAACATTTACACTCCACCGCCTTCCAAAACACTCCATTATCT
TTGCCTACTAGAGGTACTTTGGAGTCCTTGGAGAATTTAGTTGTTGCTGATCTGGAATCTTTTGCCAATAACCAT
TTCTTGAATTCAAATGCTGTTGTTGTTGGTACCGGTAATATCAAACATGAGGATTTAGTAAATTCCATCGAATCC
AAAAACCTAAGTTTGCAAACCGGTACCAAGCCCGTACTCAAGAAAAAGGCCGCTTTCTTGGGTTCCGAAGTTAGA
TTGAGAGACGACACCTTGCCAAAGGCGTGGATTTCGCTGGCTGTGGAAGGTGAACCTGTCAATTCACCAAATTAT

TTTGTTGCTAAATTAGCCGCCCAAATTTTCGGCTCATACAACGCCTTCGAACCCGCTTCTAGATTACAAGGTATC
AAATTGTTAGACAACATACAGGAGTACCAATTATGCGACAATTTCAATCATTTCTCTCTTTCTTACAAAGATTCC
GGTTTATGGGGGTTCTCTACGGCCACGAGAAACGTTACCATGATTGACGACCTCATCCATTTCACTTTGAAACAA
TGGAACAGATTGACCATTTCTGTCACTGATACTGAAGTTGAACGTGCCAAATCGCTGTTGAAATTACAATTAGGG
CAGTTATACGAATCTGGTAATCCCGTCAATGACGCTAACTTGTTAGGCGCAGAAGTCCTAATCAAGGGCTCCAAA
CTGTCTCTGGGTGAGGCTTTCAAGAAAATTGATGCAATCACAGTCAAAGATGTTAAGGCTTGGGCCGGTAAGAGA
TTATGGGACCAAGATATTGCTATTGCTGGTACAGGCCAAATTGAAGGGTTATTAGATTACATGAGAATCAGAAGT
GACATGTCCATGATGAGATGGTAA

>YEL036C 1.51 Hot
ATGAAGTATAATAACAGAAAACTCTCGTTCAACCCTACCACAGTAAGTATCGCTGGAACGTTGCTTACGGTGTTC
TTTCTCACAAGACTCGTGCTTTCGTTCTTCTCGATATCGCTATTCCAGCTGGTAACTTTCCAAGGAATCTTCAAG
CCCTATGTTCCAGATTTTAAAAATACTCCCAGCGTAGAGTTCTACGACCTACGAAATTACCAAGGCAACAAAGAT
GGTTGGCAACAGGGTGACCGCATCTTGTTTTGCGTGCCACTGAGAGATGCTTCTGAGCATCTTCCCATGTTTTTT
AACCATCTGAATACCATGACATATCCGCACAACCTGATTGATCTGTCGTTTTTGGTGAGTGACTCTTCGGATAAC
ACCATGGGCGTGTTACTATCCAATCTACAGATGGCGCAATCGCAACAAGACAAGTCCAAAAGGTTTGGTAACATC
GAAATCTATGAGAAGGACTTCGGCCAAATCATTGGTCAGTCCTTTTCTGATCGTCACGGGTTTGGCGCCCAGGGC
CCCAGAAGAAAGCTGATGGCCAGGGCTCGTAATTGGCTTGGGTCCGTGGCCCTGAAACCATACCACTCCTGGGTT
TACTGGAGAGACGTGGATGTCGAAACCATCCCCACTACCATCATGGAGGATCTAATGCATCACGACAAGGACGTC
ATTGTCCCCAACGTCTGGAGACCTTTACCAGACTGGCTGGGAAACATTCAACCCTACGATTTAAATTCATGGAAG
GAATCAGAAGGGGGTCTTCAATTGGCGGATTCCCTAGACGAAGATGCTGTCATTGTGGAAGGGTACCCAGAGTAC
GCCACTTGGAGACCTCACCTGGCATACATGAGAGACCCAAACGGTAATCCAGAAGACGAAATGGAATTGGACGGT
ATTGGAGGGGTCTCGATTCTCGCGAAAGCTAAAGTATTCAGAACAGGCTCACACTTTCCTGCATTCTCCTTTGAA
AAGCACGCAGAGACAGAAGCATTCGGTAGGCTCTCCCGCAGGATGAATTACAATGTCATCGGCTTGCCCCATTAT
GTCATCTGGCACATCTACGAACCTTCCAGCGACGACTTAAAGCACATGGCTTGGATGGCTGAAGAGGAGAAGCGG
AAACTAGAAGAAGAGAGAATTCGCGAATTTTACAACAAGATCTGGGAGATTGGATTTGAAGATGTCAGAGACCAA
TGGAACGAAGAAAGAGATTCAATCTTAAAAAACATTGACTCCACTTTGAACAATAAAGTCACTGTAGACTGGTCT
GAAGAGGGAGATGGCTCGGAGCTGGTGGACTCAAAGGGTGACTTTGTCTCACCAAACAACCAACAACAACAACAA
CAGCAACAACAACAACAACAGCAGCAGCAGCAACAGCAACAGCAACAACAGCTCGACGGCAACCCGCAGGGGAAA
CCTCTTGATGACAACGACAAGAACAAGAAAAAACATCCTAAAGAAGTTCCATTAGACTTCGACCCTGATAGAAAC
TAG

>YNL244C 2.38 Hot
ATGTCCATTGAGAATCTGAAATCATTTGATCCTTTCGCCGACACAGGAGACGACGAAACCGCCACTTCAAACTAT
ATTCATATTCGTATCCAACAGAGAAATGGTAGAAAAACTTTAACTACGGTGCAAGGTGTCCCAGAGGAATATGAT
TTAAAGAGAATTCTTAAGGTCCTAAAGAAGGACTTTGCATGTAATGGTAACATTGTCAAGGATCCAGAAATGGGG
GAGATTATTCAGTTGCAGGGTGACCAAAGAGCAAAGGTTTGCGAATTTATGATCTCCCAACTGGGATTGCAAAAG
AAGAACATTAAAATTCATGGGTTTTAA

>YCR025C 1.71 Hot
ATGTCGTCATTTGGACATCGAGCATTTTTCAGCTGTTGTCCTTTACTAGAAGGAGAAACTTTGCATTCAATAAAA
TTGCGCAAAGAGGCAACATACCAAACTCAATCCGGGTGGCCGGTTCGATGGCCGATTCGGTATCCGCGCGCACTT
CCCCTAGAATCCCACGTGCACCCATACCATATGATCGTTAAATTACCGCACCCACGCACCCAACCGCGGCTGTTT
CTATGGCTGTCGTTGCATTGGACAGGAATTCCTTTTTTGGGAAAGCCGGGAATAGGTCGCAATCGGGTAAAATCA
AAACAATTTTCGCATTTCCAACAGAGTAAAATCAAAACAACTATGAGAAAAAAAACAACAAACAGAAAGCAGAAA
GCAGAAAGATGTCAATATAGACTTGTCACTGCGTAA

>YDL086W 2.08 Hot
ATGTTGATTACTGAGACCTTTCATGATGTGCAGACATCATACGGCACCACTCTGCGTATATACGTATATTCTCCT
AAGATTGCAGGCTATCCACAAGCTAAGTTTCCTGGGGTGATTCTGTACAGTGAAATTTACCAAGTGACGGGTCCG
GTTCGTCGTTTTGGTCAAAGAATTGCGTCTGAAGGTTACGTTGTGGTGGCACCTGCCATATACCACAACTTTATG
GGCCCTGAAGCGTTGCCCTATGACGTGCAGGGTACCGATATTGGTAACGAGTACAAGATCAAGAAGCCGTTAGAA
TCATACGATGAGGACAACAAACTGTGTTGCGATCTACTTTTCCAGTTACCCCAATTCGATGGTAAGAGAATTGGT
TCCACAGGAATGTGTTTAGGTGGCCATTTGGCTTTTAGAGCGCTCCTGGACAAAAGGGTTACCTGCGCAACCTGC
TTCTTCCCTACCGACATCCATTCCAGAACCTTAGGTCTTGGCCAGAACGATAACTCTTTGGAACGTGTTTCGAAG
GAGTTGGGTAATAACCAAGAAATGGTTTTGATCTTCGGAACTGCGGACACCCATGTCGACCCGCAAGGCCGCGAT
CTAATCAGAAAGACCCTCAGAGACCACGGAGTAAAGTTTACGTTCTTGGAAATCCTGGCTGCCCAGCATGCGTTC
ATCCGTGACGAGTTCAGCAAGGGCAGGTTCGACTCCGCTATCACTCAAAGTTGTCTCGGCTTCTTGTTCGAGCAG
TTCAACAGAAAATTGAGAATCGATTTGGGTGAATTTGTCGATGACAATACACCTTTGGAGCACGTTTGTTAA

>YBL082C 1.67 Hot
ATGGAAGGTGAACAGTCTCCGCAAGGTGAAAAGTCTCTGCAAAGGAAGCAATTTGTCAGACCTCCGCTGGATCTG
TGGCAGGATCTCAAGGACGGTGTGCGCTACGTGATCTTCGATTGTAGGGCCAATCTTATCGTTATGCCCCTTTTG
ATTTTGTTCGAAAGCATGCTGTGCAAGATTATCATTAAGAAGGTAGCTTACACAGAGATCGATTACAAGGCGTAC
ATGGAGCAGATCGAGATGATTCAGCTCGATGGCATGCTGGACTACTCTCAGGTGAGTGGTGGAACGGGCCCGCTG
GTGTATCCAGCAGGCCACGTCTTGATCTACAAGATGATGTACTGGCTAACAGAGGGAATGGACCACGTTGAGCGC
GGGCAAGTGTTTTTCAGATACTTGTATCTCCTTACACTGGCGTTACAAATGGCGTGTTACTACCTTTTACATCTA

CCACCGTGGTGTGTGGTCTTGGCGTGCCTCTCTAAAAGATTGCACTCTATTTACGTGCTACGGTTATTCAATGAT
TGCTTCACTACTTTGTTTATGGTCGTCACGGTTTTGGGGGCTATCGTGGCCAGCAGGTGCCATCAGCGCCCCAAA
TTAAAGAAGTCCCTTGCGCTGGTGATCTCCGCAACATACAGTATGGCTGTGAGCATTAAGATGAATGCGCTGTTG
TATTTCCCTGCAATGATGATTTCTCTATTCATCCTTAATGACGCGAACGTAATCCTTACTTTGTTGGATCTCGTT
GCGATGATTGCATGGCAAGTCGCAGTTGCAGTGCCCTTCCTGCGCAGCTTTCCGCAACAGTACCTGCATTGCGCT
TTTAATTTCGGCAGGAAGTTTATGTACCAATGGAGTATCAATTGGCAAATGATGGATGAAGAGGCTTTCAATGAT
AAGAGGTTCCACTTGGCCCTTTTAATCAGCCACCTGATAGCGCTCACCACACTGTTCGTCACAAGATACCCTCGC
ATCCTGCCCGATTTATGGTCTTCCCTGTGCCATCCGCTGAGGAAAAATGCAGTGCTCAATGCCAATCCCGCCAAG
ACTATTCCATTCGTTCTAATCGCATCCAACTTCATCGGCGTCCTATTTTCAAGGTCCCTCCACTACCAGTTTCTA
TCCTGGTATCACTGGACTTTGCCTATACTGATCTTTTGGTCGGGAATGCCCTTCTTCGTTGGTCCCATTTGGTAC
GTCTTGCACGAGTGGTGCTGGAATTCCTATCCACCAAACTCACAAGCAAGCACGCTATTGTTGGCATTGAATACT
GTTCTGTTGCTTCTATTGGCCTTGACGCAGCTATCTGGTTCGGTCGCCCTCGCCAAAAGCCATCTTCGTACCACC
AGCTCTATGGAAAAAAAGCTCAACTGA

>YGL035C 1.69   Hot
ATGCAAAGCCCATATCCAATGACACAAGTGTCTAACGTTGATGATGGGTCACTATTGAAGGAGAGTAAAAGCAAG
TCCAAAGTAGCTGCGAAGTCAGAGGCGCCAAGACCACATGCTTGTCCTATCTGTCATAGAGCTTTTCACAGACTG
GAACATCAGACGAGACACATGAGAATTCATACAGGTGAGAAGCCTCACGCGTGTGACTTCCCCGGATGTGTGAAA
AGGTTCAGTAGAAGCGATGAACTGACGAGACACAGAAGAATTCATACAAACTCCCACCCTCGAGGTAAAAGAGGC
AGAAAGAAGAAGGTTGTGGGCTCTCCAATAAATAGTGCTAGTTCTAGTGCTACCAGTATACCAGATTTAAATACG
GCAAATTTTTCACCGCCATTACCACAGCAACACCTATCGCCTTTAATTCCTATTGCTATTGCTCCGAAAGAAAAT
TCAAGTCGATCTTCTACAAGAAAAGGTAGAAAAACCAAATTCGAAATCGGCGAAAGTGGTGGGAATGACCCATAT
ATGGTTTCTTCTCCCAAAACGATGGCTAAGATTCCCGTCTCGGTGAAGCCTCCACCTTCTTTAGCACTGAATAAT
ATGAACTACCAAACTTCATCCGCTTCCACTGCTTTGTCTTCGTTGAGCAATAGCCATAGTGGCAGTAGACTGAAA
CTGAACGCGTTATCGTCCCTACAAATGATGACGCCCATTGCTAGCAGTGCGCCAAGGACTGTTTTCATAGACGGT
CCTGAACAGAAACAACTACAACAACAACAAAATTCTCTTTCACCACGTTATTCCAACACTGTTATATTACCAAGG
CCGCGATCTTTAACGGATTTTCAAGGATTGAACAATGCAAATCCAAACAACAATGGAAGTCTCAGAGCACAAACT
CAGAGTTCCGTACAGTTGAAGAGACCAAGTTCAGTTTTAAGTTTGAACGACTTGTTGGTTGGCCAAAGAAATACC
AACGAATCTGACTCTGATTTTACTACTGGTGGTGAGGATGAAGAAGACGGACTAAAGGACCCGTCTAACTCTAGT
ATCGATAACCTTGAGCAAGACTATTTGCAAGAGCAATCAAGAAAGAAATCTAAGACTTCCACGCCCACGACAATG
CTAAGTAGATCCACTAGTGGTACGAATTTGCACACTTTGGGGTATGTAATGAACCAAAATCACTTGCATTTCTCC
TCATCATCTCCTGATTTCCAAAAGGAGTTGAACAACAGATTACTGAACGTTCAACAACAGCAGCAAGAGCAACAT
ACCCTACTGCAATCACAAAATACGTCAAACCAAAGTCAAAATCAAAATCAAAATCAAATGATGGCTTCCAGTAGT
TCGTTAAGTACAACCCCGTTATTATTGTCACCAAGGGTGAATATGATTAATACTGCTATATCCACCCAACAAACC
CCCATTTCTCAGTCGGATTCACAAGTTCAAGAACTGGAAACATTACCACCCATAAGAAGTTTACCGTTGCCCTTC
CCACACATGGACTGA

>YCL011C 1.54   Hot
ATGGAGAGAGAGAGCTAGGGATGTATGGAAATGATAGGAGTAGATCAAGATCACCTGTACGTCGTCGTTTGAGCGAC
GACAGAGACAGGTACGATGATTATAACGATAGTAGCAGTAATAATGGTAATGGCAGTCGTCGTCAGAGACGCGAC
CGAGGCTCCCGTTTCAATGATCGGTACGATCAGAGTTATGGTGGCAGCCGCTACCACGATGATAGGAACTGGCCC
CCTCGCCGAGGAGGCCGTGGCAGAGGAGGAAGCAGATCATTCAGAGGGGGACGCGGTGGCGGTAGGGGTCGTACT
TTAGGTCCAATTGTTGAAAGAGACTTAGAAAGGCAATTTGACGCGACCAAGAGAAATTTTGAAAATAGTATCTTC
GTGAGAAACTTGACTTTTGATTGTACCCCTGAAGACCTTAAGGAATTGTTTGGTACAGTGGGCGAAGTTGTGGAG
GCTGACATTATCACATCAAAGGGCCATCACCGTGGTATGGGGACTGTGGAATTTACCAAAAACGAATCTGTCCAA
GATGCCATATCGAAGTTTGATGGTGCCCTCTTTATGGACCGGAAACTAATGGTAAGACAGGATAATCCTCCTCCT
GAAGCTGCCAAGGAATTTTCTAAGAAAGCTACTAGGGAAGAAATAGATAATGGGTTTGAAGTGTTCATCATCAAT
TTACCGTACTCTATGAATTGGCAATCCTTAAAAGATATGTTTAAAGAATGTGGTCATGTCTTGCGTGCCGATGTA
GAATTGGATTTCAACGGATTTTCAAGAGGATTCGGTTCTGTCATTTATCCTACTGAGGATGAAATGATTAGAGCT
ATCGATACATTCAACGGCATGGAAGTAGAAGGTAGAGTTTTGGAAGTTAGAGAAGGGCGTTTCAACAAGAGAAAG
AACAATGATCGTTATAATCAAAGGCGTGAGGACCTTGAAGATACCAGAGGTACTGAACCAGGTCTTGCGCAGGAT
GCCGCTGTCCACATTGATGAAACTGCAGCAAAATTTACTGAAGGTGTCAATCCAGGAGGGGATAGAAACTGTTTC
ATTTATTGTAGTAATTTACCATTCTCAACAGCAAGAAGCGATTTATTCGACTTGTTTGGGCCTATCGGCAAAATC
AATAACGCGGAATTGAAACCACAGGAAAATGGTCAACCAACTGGTGTTGCTGTTGTAGAATATGAAAATTTAGTA
GATGCAGATTTTTGTATTCAAAAATTAAATAATTATAATTATGGTGGTTGTAGTTTACAGATCTCTTATGCTAGA
CGTGATTAA

>YLR080W 1.56   Hot
ATGACTACAAGAAAGACAGCTTCCTCTCTTCAGCTTTTAGGGAAAATCACAGGAACAAAAGCTGGAACTAAGCAA
AAGAAGATGAATTTCATTAATGGACTGATATGGTTATATATGTGTGTGTGGATGGTACACGGAAAAGTGACGCAG
AAGGATGAATTGAAATGGAATAAAGGATACTCGCTACCAAATTTGCTAGAAGTGACAGATCAGCAAAAAGAACTT
TCACAATGGACTTTGGGTGACAAAGTAAAACTTGAAGAAGGGAGGTTTGTTTTAACTCCTGGAAAGAACACAAAG
GGTTCACTTTGGTTGAAACCTGAATATTCAATAAAGGATGCAATGACAATAGAGTGGACGTTTAGAAGTTTCGGG
TTCAGAGGCAGCACAAAGGGGGGTCTTGCATTTTGGCTGAAGCAAGGAAATGAGGGAGATAGTACCGAGTTATTT
GGTGGAAGTTCGAAGAAGTTTAATGGTTTGATGATATTGTTACGATTAGACGATAAGTTGGGAGAGAGCGTGACA
GCGTATTTGAATGACGGAACAAAAGATCTTGATATTGAATCCTCACCGTACTTTGCGTCATGTCTGTTCCAATAC
CAGGATTCCATGGTACCATCAACATTAAGATTGACTTACAATCCACTAGATAATCACTTGTTAAAGTTGCAAATG

GACAACAGAGTGTGTTTCCAGACAAGGAAAGTTAAATTTATGGGCAGCAGCCCATTTAGGATTGGAACAAGTGCT
ATCAACGATGCATCCAAAGAATCGTTTGAAATCTTGAAAATGAAGCTTTATGACGGAGTTATAGAGGATTCGCTA
ATTCCTAACGTGAATCCTATGGGACAACCCAGAGTGGTTACTAAAGTGATCAATTCTCAAACTGGTGAAGAGAGT
TTCAGGGAAAAGATGCCATTTTCTGATAAGGAAGAAAGTATAACGAGTAACGAGCTTTTCGAAAAGATGAACAAG
TTGGAGGGGAAAATCATGGCAAATGATATCGATCCATTACTCCGCAAGATGAACAAGATTGTGGAGAATGAACGT
GAACTGATTCAACGTTTAAGACCACTGTTAGATCTGAAGAAAACAGCCATAAGTGACGATAGTTTCCAAGATTTT
CTTTCGATGAACGCAAACCTGGACAGATTGATAAAAGAACAAGAAAAAATTCGACAAGATGCCAAGCTGTATGGC
AAGCAGACCAAAGGTCATGATGAGATATTTTCCAAAATAAGTGTATGGTTGGCACTGCTGATTTTCATTATGATC
ACATTGGCGTACTACATGTTTAGAATTAACCAAGACATCAAGAAGGTCAAACTTCTGTAA

>YLR212C 1.59   Hot
ATGGGTGGAGAAATTATTACTTTGCAAGCAGGTCAATGCGGGAACCACGTTGGTAAGTTTCTCTGGTCTCAATTG
GCGAAAGAACACGCTATAGGCACCGATGGGCTATCTCAGCTTCCTGATTCCAGTACGGAAAGAGATGATGACACA
AAGCCTTTCTTCCGCGAAAACAGTAGGAATAAGTTTACGCCAAGGGCTATTATGATGGATTCCGAGCCTAGTGTC
ATCGCCGACGTGGAGAACACATTTCGTGGGTTTTTCGACCCAAGAAATACCTGGGTAGCTTCTGATGGCGCTAGC
GCTGGTAATTCTTGGGCCAATGGGTATGATATAGGAACTCGTAACCAGGATGATATTCTTAACAAGATCGACAAG
GAGATTGATTCTACCGACAATTTCGAAGGTTTCCAGTTGCTGCACTCAGTAGCCGGAGGGACCGGTTCAGGGCTC
GGATCCAACCTCTTAGAAGCGCTGTGTGATCGATATCCTAAAAAAATACTCACGACATATTCTGTGTTCCCTGCA
AGATCTTCTGAGGTTGTTGTTCAATCTTATAACACTATTCTGGCTTTGAGAAGACTGATAGAAGATAGCGATGCA
ACGGTGGTGTTTGATAACGCTTCGTTGCTGAATATTTCCGGTAAAGTGTTTAGAAATCCGAACATCGATTTACAA
CACACGAATCAATTGATATCGACCATAATATCTTCAGTAACGAACAGTATACGGTTTCCGAGTTACATGTATTCA
TCCATGTCCAGTATTTACTCTACCTTGATTCCTTCTCCTGAACTTCATTTCCTAAGCCCTAGTTTCACTCCATTT
ACATCAGATTATATTCACGATGATATAGCGCATAAAGGCCATTCCAGTTATGACGTCATGTTAGATTTATTAGAC
CCTTCCAATTCTCTTGTCTCTACCGCGATGAACAATCCAACATATTTTAACGTTTATAATACAATCATTGGTAAT
GTAGAACCTCGTCAGATATCGCGTGCTATGACCAAATTACAACAACGCATCAAATTCCCCTCATGGTCCTCTTCT
GCGATGCACGTTAACATTGGAAGAAGATCGCCATACTTGCCTTTACAACCAAATGAAAACGAAGTTAGCGGCATG
ATGTTAAGCAATATGTCTACCGTGGTGAACGTCTTTGAGAATGCGTGCAATACTTTTGACAAAGTATTTGCCAAG
GGCGCATTTTTAAATAATTATAATGTAGGCGACTTGTTCCAATCAATGCAGAACGTTCAAGATGAATTCGCCGAG
TCAAGAGAAGTAGTACAAAGCCTGATGGAGGATTATGTAGCTGCAGAACAAGATTCGTACTTGGACGATGTACTT
GTTGATGATGAAAATATGGTTGGCGAGTTGGAAGAGGACCTGGATGCCGACGGTGATCATAAATTAGTATAA

>YFR038W 1.57   Hot
ATGAGCAGATGCTCAAACGCGGCGCTGATGACTGTTGTTGAGGATGCTGTTGGGGCCAGGGTGGCTGCAAGGACG
AGAAACATGAGCAATGGCGTCAATTACCGGGAGAAGGAAGTCAATGACCTAACGGCTGACATTTCAGATTCAGAT
TCAGATTTGGATTCAGAGGACAATAAACATGGCAAAGGCGACAATGACACTGCCCCCATATGGTTACAAGATGAT
GTGCATTCAGACGAAGATATTCAGCTGGACTCAGAGGACGATTCCGATACAGAAGCTGTTCAAGCCCAAGTGGTG
GATAAATTGGCAAAAGACACGAAATCAGAGCAAAAATCCTTGGATGATGAGCTCTCCGAGATGGATACCAAGACG
GTTTCTTTAAAACTTAAGAAACTAAACGAGTTTGTGAGGCAGAGTCAGGTATATTCCAGTATAATCGCCGATACT
TTACTACATCGATCTAATGAAGTGGCTAACGCTAATACAAAAGATAACAGCAACTCTGATGATGAAGAACATTCT
TCAAAAAAACGTAAAACAAAAAAAAAATCAATAACAGATTTCTTCAAAAAACAGAAAAAGAACGAAGATACAACC
ACCCAAAACGGCGCACCAGACGATGCAGCAATAAAGCAACCTCGCCTATTGAAAAACTGTATTTTAAAACCTTAC
CAACTAGAAGGTCTTAACTGGCTGATCACTCTTTATGAAAATGGCCTGAACGGAATTCTTGCCGATGAAATGGGT
CTTGGTAAGACTGTTCAAAGTATTGCTCTTTTGGCGTTCATATATGAAATGGACACAAAGGGTCCTTTTCTAGTG
ACTGCACCATTAAGCACCCTAGATAATTGGATGAACGAATTTGCAAAATTCGCTCCTGATCTACCAGTTCTAAAA
TATTATGGGACCAATGGTTATAAGGAACGTTCTGCCAAGTTGAAAAATTTCTTTAAACAACACGGCGGCACGGGG
ATCGTCATCACATCATATGAAATTATTTTAAGAGATACCGACTTAATCATGAGTCAAAATTGGAAATTTTTAATT
GTTGATGAAGGCCACCGTCTGAAAAATATAAACTGTAGATTGATTAAAGAGTTAAAAAAAATCAATACTTCCAAT
AGACTGCTACTAACAGGGACGCCTTTACAAAATAACCTAGCTGAATTATGGTCACTTTTGAATTTCATAATGCCC
GATATCTTTGCCGATTTCGAGATATTCAATAAATGGTTTGACTTCGACAGCTTAAATTTAGGCTCCGGTTCCAAC
TCTGAAGCATTGAATAAGTTAATCAACGATGAACTACAGAAAAATTTGATTTCCAATTTACACACAATTCTGAAA
CCATTCCTTTTGAGAAGATTGAAAAAAGTTGTTTTGGCTAATATTCTGCCGCCAAAGAGAGAATATATCATAAAT
TGTCCGATGACGTCTGCACAGGAAAAATTCTATAAGGCTGGATTGAATGGAAAGTTGAAAAAAACCATGTTCAAA
GAATTAATCAAGGATTTTTTCACATTGAACGATGAATACATCGGCCACGTTTCTAATCGGTCCATACGAGATTTC
ATCAATTATAAACTATCTGGTAATGAAACTTCAAATACAGACAATAAAATCAATCCAACCTTGTTGCAAATGGAC
AAACTATATAAGAAGAACCTGCAAATGGAAATTTCAAATAAGAAATTGCAAAATATGATGATGCAATTGAGACAA
ATCATTGACTCAACTTTTTTGTTTTATTTCCCATATTTACACCCAGAAGACTTGACTTTGGAAACTCTTTTAAAG
ACGTCTGGGAAATTACAAATCTTACAAAAATTAATCCCCCCATTAATATCTGAGGGGCACAAAGTGCTTATTTAT
TCTCAGTTTGTTAACATGCTCGATTTGATCGAAGATTGGTGTGATTTAAATTCGTTCGCGACATTTCGAATTGAT
GGTTCAGTAAACAATGAAACAAGAAAGGATCAACTCGAGAAGTTTAATAGCTCGAAAGATAAGCATAACATTTTC
CTATTGTCCACAAGGGCGGCAGGACTGGGTATTAATCTAGTCGGGGCAGATACAGTTGTCTTGTTTGATAGTGAT
TGGAATCCACAGGTGGATTTACAAGCCATGGACAGGTGTCACCGTATTGGCCAAGAATCACCGGTTATAGTATAT
AGGTTGTGTTGTGATAACACCATTGAACATGTTATATTGACCAGAGCGGCCAATAAGCGAAATTTGGAAAGAATG
GTTATCCAGATGGGAAAATTCAATAACCTGAAAAAATTGGCGTTAAATGAAGGTTCTTTTTTGAAGGCAAACAAA
GCGGGTGTTAATGTCACTAATAAGGACTTAGTCCAGGAGTTATCTATGCTTTTAATGAGCGATGAATCCAATATA
GGCTTTGAAAATGGGGGACAGAAAGAAAATAAGGCTACTGAGGGCCAACTGACTGATAAGGAGGTTGAAGAATTG
ACCAACAGATCTCTTGAAGCGTACAAGGCCAACAGAGTCGTAGATCTGCCGCACGTCAAGCTATTTGAAACAACC
TCCGGTCTTTAA

```
>YIL118W  1.62   Hot
ATGTCATTTCTATGTGGGTCAGCTTCAACGTCAAATAAACCGATCGAAAGAAAGATCGTTATTTTGGGCGACGGT
GCCTGTGGTAAAACTTCGTTGCTGAATGTTTTCACCAGAGGTTATTTTCCCGAAGTTTATGAGCCTACTGTTTTT
GAAAACTATATCCATGATATTTTCGTTGACAGTAAACATATCACGCTATCGTTGTGGGATACTGCGGGCCAAGAG
GAATTTGACAGGTTACGATCCTTGTCTTATTCAGATACGCAATGTATAATGTTATGTTTCAGTATTGATTCACGC
GATTCTTTAGAAAATGTCCAAAATAAATGGGTGGGTGAAATCACTGATCATTGTGAAGGCGTCAAGTTAGTCTTA
GTTGCACTAAAGTGTGACTTAAGAAACAATGAAAATGAATCTAACGCAATCACACCGAACAATATCCAACAGGAT
AACAGCGTTTCTAACGACAACGGAAATAACATAAATAGCACCTCAAACGGCAAAAACCTGATAAGTTATGAAGAA
GGTCTAGCTATGGCTAAAAAGATCGGTGCGCTACGTTATTTGGAATGTAGCGCTAAGCTGAATAAAGGTGTCAAC
GAAGCTTTCACAGAAGCCGCAAGAGTTGCTTTAACCGCGGGCCCAGTAGCAACCGAAGTGAAAAGTGACAGTGGA
TCCAGCTGTACCATTATGTAA

>YNL305C  1.89   Hot
ATGTCAGGTCCTCCACCTCCTTACGAAGAGCAAAGCTCACATCTTTACGGCCAGCCTGCGAGCAGCCAGGATGGA
AACGCTTTCATCCCGGAAGACTTTAAATATTCCACGGTGGTTATCTCGTGCGAACCCATCATTCGCCAGCGGTTT
ATGCACAAGGTTTACTCTCTGCTGTCATGCCAGTTACTGGCCAGTTTGTCGTTCTGCTACTGGGCTAGCGTTTCC
ACTTCTTTGCAGAACTTTATCATGTCGCATATCGCCCTCTTCTACATCTGCATGGTGGTATCGCTAGTATCGTGC
ATTTGGCTGGCAGTAAGTCCTCGTCCCGAAGACTACGAGGCCAGTGTTCCCGAACCGTTGCTCACGGGCAGCAGC
GAAGAGCCGGCACAAGAGCAAAGGCGTCTGCCTTGGTACGTCTTGTCCTCGTACAAGCAAAAGCTCACGCTGCTC
TCCATTTTCACTCTTTCCGAGGCTTACTGTCTGTCGCTGGTAACCTTAGCGTACGATAAGGATACAGTACTTTCG
GCTCTGCTGATCACCACCATCGTCGTAGTCGGCGTTTCCTTGACCGCACTATCAGAGAGATTCGAGAACGTGCTG
AATTCCGCAACGTCAATATACTATTGGTTAAACTGGGGTCTATGGATTATGATCGGAATGGGTCTCACGGCCTTG
CTTTTCGGCTGGAATACCCACTCTTCCAAGTTTAATCTGCTTTACGGCTGGCTTGGTGCCATTTTATTTACCGCG
TATCTTTTCATCGATACGCAACTGATTTTTAGGAAAGTGTATCCTGATGAAGAAGTTAGATGCGCCATGATGCTT
TACCTGGACATCGTCAATTTGTTTTTGTCTATTTTAAGGATTTTGGCCAACTCTAACGACGACAATTAA

>YDR051C  1.75   Hot
ATGTGTGAAGAGAATGTTCATGTTAGTGAAGATGTTGCTGGCAGTCACGGTTCTTTTACGAATGCCAGACCTAGG
TTAATTGTACTAATAAGGCATGGGGAAAGCGAATCAAATAAAAACAAGGAGGTCAATGGTTATATTCCTAACCAT
CTGATTTCTTTAACGAAAACAGGTCAAATCCAAGCTAGACAAGCTGGTATTGACTTATTACGTGTTTTAAACGTA
GACGATCACAACTTGGTGGAGGATTTGGCTAAGAAGTATATTAAAGATGAAAGTAGCAGGAGAACTTTACCGCTG
AAGGACTATACCAGGCTGAGTAGAGAAAAAGACACAAACATAGTTTTTTATACATCACCCTATAGAAGAGCAAGG
GAAACATTGAAAGGTATTTTGGACGTCATCGATGAATATAATGAATTAAACAGTGGTGTTCGTATATGTGAAGAT
ATGAGATATGATCCACATGGTAAACAGAAACATGCATTTTGGCCGAGAGGACTTAATAATACTGGTGGTGTTTAC
GAAAATAATGAAGATAATATTTGTGAAGGGAAGCCTGGAAAATGTTATCTGCAATATCGGGTTAAGGATGAGCCA
AGAATAAGGGAACAAGATTTTGGTAATTTCCAAAAAATCAATAGCATGCAGGACGTTATGAAGAAGAGATCTACG
TATGGTCATTTCTTCTTCAGATTCCCTCATGGAGAAAGTGCGGCAGATGTATATGACAGAGTCGCCAGTTTCCAA
GAGACTTTATTCAGGCACTTCCATGATAGGCAAGAGAGAAGACCCAGAGATGTTGTTGTCCTAGTTACACATGGT
ATTTATTCCAGAGTATTCCTGATGAAATGGTTTAGATGGACATACGAAGAGTTTGAATCGTTTACCAATGTTCCT
AACGGGAGCGTAATGGTGATGGAACTGGACGAATCCATCAATAGATACGTCCTGAGGACCGTGCTACCCAAATGG
ACTGATTGTGAGGGAGACCTAACTACATAG

>YOL053W  2.11   Hot
ATGTGGGGGTTATGCAAGAAGCATTTTCCGAGCAATAAAATTCAAGTGCAGGAGAGAAACAAAGCATTAAAACCA
AAGAAGAGTGGCAGCGAACATAAGACAAAGCAACTGTTTCCTGTATTCAATTGCAAGAAGAAGGAAAAAGGGGTA
ATGATACGGTTTGCGATTCTACGTAATGCTAACACATCTCTACTGTCCGCGAGAAGTATATGCTTATTTACCCAG
GCGCCTACCTATTGTCATGTAAGGTTGAATACCTTGAATAAAAGCATTACAACTAAAAGAAATTCATTGACAGAG
TCGAAAAGGCATGTCCATGATGGCAAGCATTTTTTTACGACGCCACATCAACAGCAACAGACGAAGTTGGGGGAA
ATTGAAGAGGGACACAGTCCTAACATAAAAGGAGAGGACTTGAGAAGCATTGGTCAAGCAATAACACATCAGCGA
AACAAGCGCCGAAAGCAAATATGGTCCGCCATATTTGGTGGTATATTCGGTGTGATATTAGGATACTCACTAATC
TATAGAGTGATATATTTAAAGGAACAAAGTTTCTTACCATTGTTTCCATCATCCAAGATACGTAAACTGAGTACC
AGAGATTTGAAAAAGGTTGACGTAAACCAAGTACAGAAGCTTTCTAAATTAAGGGTTTTAGAGATATTATCCGGT
CATGACATGATCAAGGAACAATACGGAGTACCATTGCTTGATAAAGATGGCAATTCACCCACGTTAAATGAATTC
AGCATGTGGTGCGAAGATCAAGATCCATGTGTAACTGGTATTGTAATGGAGCCAGACGATAAAAGGGATAGTTCG
CACACTTGGTATAGAATACCCTTTGTTTGCAAATGGAGGATAACGCATCGGCCAATAAGTATACGGGGAACCATC
GACGATTTACTGAACCGCATTGGTTTGGAAACAGCCGATTTATTTGAGATTATATCGCCGGAGAGGGTATACGGG
TCGTTCAAGTACGAATACCCACTGCAGGGGGACTCACATGCATTACACCTTTGGTTTCATGGCGAAATCGAACTG
GACGATGATTCTTTGATTGTATATAATGGAAAATACCACGTTGATGTTAAATTGCAAGAAATCGACCTCTTCAGA
CGTGAAAAGAACGGACAACTAATACAGTACGTTCTATACAAAAACGAAGCGGGCGACAAGTAA

>YBL016W  2.12   Hot
ATGCCAAAGAGAATTGTATACAATATATCCAGTGACTTCCAGTTGAAGTCGTTACTGGGAGAGGGTGCATACGGT
GTGGTATGTTCTGCAACGCATAAGCCCACGGGAGAAATCGTGGCAATAAAAAAGATCGAACCATTCGATAAGCCT
TTGTTCGCATTACGTACGCTGCGTGAAATAAAGATCCTGAAGCACTTCAAGCACGAAAATATCATAACAATCTTC
AACATTCAACGCCCTGACTCGTTCGAAAACTTCAATGAGGTCTACATAATTCAAGAGCTAATGCAGACAGATTTA
CACCGTGTAATCTCCACCCAGATGCTGAGTGACGATCATATACAATATTTTATATACCAAACCTTGAGAGCAGTG
```

AAAGTGCTGCATGGTTCGAACGTCATCCATCGTGATTTAAAGCCCTCCAACCTTCTCATAAACTCCAACTGTGAC
TTGAAAGTATGTGATTTCGGTTTAGCAAGAATCATTGACGAGTCAGCCGCGGACAATTCAGAGCCCACAGGTCAG
CAAAGCGGCATGACCGAGTATGTGGCCACACGTTGGTACAGGGCGCCAGAGGTGATGTTAACCTCTGCCAAATAC
TCAAGGGCCATGGACGTGTGGTCCTGCGGATGTATTCTCGCTGAACTTTTCTTAAGACGGCCAATCTTCCCTGGC
AGAGATTATCGCCATCAACTACTACTGATATTCGGTATCATCGGTACACCTCACTCAGATAATGATTTGCGGTGT
ATAGAGTCACCCAGGGCTAGAGAGTACATAAAGTCGCTTCCCATGTACCCTGCCGCGCCACTGGAGAAGATGTTC
CCTCGAGTCAACCCGAAAGGCATAGATCTTTTACAGCGTATGCTTGTTTTTGACCCTGCGAAGAGGATTACTGCT
AAGGAGGCACTGGAGCATCCGTATTTGCAAACATACCACGATCCAAACGACGAACCTGAAGGCGAACCCATCCCA
CCCAGCTTCTTCGAGTTTGATCACTACAAGGAGGCACTAACGACGAAAGACCTCAAGAAACTCATTTGGAACGAA
ATATTTAGTTAG

>YJR073C 2.33 Hot
ATGAAGGAGTCAGTCCAAGAGATCATCCAGCAACTCATCCACAGTGTCGATTTACAGTCTTCCAAGTTCCAGCTG
GCCATTGTGTGCACGATGTTCAATCCTATCTTTTGGAACATCGTTGCAAGAATGGAATACCACAAGCATTCTCTC
ACCAAGATGTGTGGTGGGGCCAGAAAGGGCTGTTACATGTTGGCGGCGACCATATTTTCGCTAGGTATCGTCAGA
GACATGGTGTACGAGTCTGCATTGCGTGAACAGCCTACGTGTTCTCTGATCACGGGCGAGAACTGGACCAAGCTG
GGTGTGGCTCTCTTTGGTTTGGGGCAAGTGCTTGTTTTGAGTTCCATGTACAAGCTGGGTATCACAGGGACGTAC
TTGGGTGACTATTTCGGCATCCTGATGGATGAGAGAGTCACCGGCTTCCCCTTCAACGTTTCCAACAACCCCATG
TACCAGGGTTCCACTTTGTCCTTCTTGGGCATAGCCCTTTACAAAGGAAAGCCTGCGGGGCTGGTTGTTTCTGCC
GTAGTTTACTTCATGTACAAGATCGCTCTTCGTTGGGAAGAACCTTTTACTGCCATGATCTACGCTAACCGTGAT
AAGGCCAAAAAGAATATGTAA

>YCL010C 1.56 Hot
ATGGACGGATATTGGGATGTTGTAGTGTCGTCCTTGCAAGACATATACAATGCCAACGAGGTCATTCCCTTTGAT
GATGAGCTGCAAACAAAAAAACTAAATTTCCTTAATATGTCAAAGGATCAGCTGCAATTGCATCTCAACACTTTC
CAAGAACACATGGAAAACGTCAACAGAGTGCACCGAATCCTTGACAATGTACGTTCCAATTTGTCGTTGATGTTG
AATCAGTCACGAGAGGAAAAGAGTGAGGAAAATACTGAAGATGCAGAAGAAGGAGAAGGGACACGCATGGCATTG
TCGCAGGGCAAGAAAGCTGTGGGGAAAGTTGGCAGATCGTACTGGACCAGCGAATACAACCCAAATGCACCTATT
TTGGTGGGCTCAGAGGTTGCCTATAAGCCCAGAAGGGGCAGTGCCGACGGCGAATGGATACAGTGTGAAGTACTC
AAAGTCGTAGCAGATGGTACACGATTTGAAGTGAGAGATCCTGAACCTGATGAATTGGGCAACTCAGGCAAGGTG
TATAAATGTAATCGGAAAGAACTACTGTTGATCCCGCCAGGCTTTCCCACTAAGAACTACCCGCCAGGTACTAAG
GTTCTGGCAAGATATCCTGAAACGACCACTTTTTATCCTGCAATTGTCATCGGAACCAAGAGGGACGGCACTTGC
AGGCTTCGATTTGATGGTGAAGAGGAAGTGGACAAAGAGACGGAAGTGACTCGCCGTCTAGTGCTGCCTTCGCCA
ACGGCTTTGGCAAACCTAGCAAGGAAATAG

>YNR041C 1.59 Hot
ATGTTTATTTGGCAGAGAAAGAGTATTTTACTAGGGAGGTCCATTCTGGGCAGCGGCCGAGTTACCGTTGCTGGA
ATCATTGGAAGCAGCAGAAAGAGATATACGTCTTCTTCCTCCTCCTCCTCCTCCCCTTCCTCGAAAGAATCAGCA
CCTGTGTTTACGTCGAAAGAGTTGGAAGTTGCAAGAAAGGAAAGATTGGATGGACTGGGACCGTTCGTGTCTCGG
CTGCCTAAGAAATGGATACCGTATGCGGAGCTGATGAGGCTAGAGAAACCCGTGGGCACGTGGCTGTTATATTTG
CCCTGCAGTTGGTCTATACTTATGGGAGCCATGATGCAAGGTGCTACATTGAGCGCAACAGCTGGTATGTTGGGT
ATTTTCGGTGTAGGTGCATTGGTAATGAGAGGTGCTGGCTGCACCATCAACGATTTTTTGGATAGAAAGTTGGAT
CAAAGAGTTATCAGGTCCGTCGAGAGGCCTATCGCCTCTGGTAGAGTGAGCCCCCGTCGGGCGTTAGTATTCTTG
GGGGCTCAAACTCTTGTTGGAATGGGCGTTCTATCTTTGCTTCCTGCGCAGTGTTGGTGGCTAGGCCTTGCATCG
TTACCTATTGTGTTCACATACCCTTTGTTTAAGCGGTTTACGTACTATCCTCAAGCTGCATTAAGTGCATGTTTC
AATTGGGGAGCGTTACTTGGGTTTCCTGCCATGGGTGTAATGAGCTGGCCTACAATGATACCTTTGTACCTGAGC
AGTTACTTGTGGTGTATGACTTACGATACTATATACGCACACCAGGATAAAAAGTTTGACATCAAGGCTGGAATC
AAGTCTACCGCTTTGGCTTGGGGTCCACGCACAAAGTCTATCATGAAGGCCATGTCCGCGTCTCAAATCGCACTG
TTAGCTGTCGCCGGCTTGAACAGTGGGTTGTTATGGGGGCCTGGGTTCATTGGTGGACTAGGAGTCTTCGCTTAT
AGACTCTTCAGCATGATAAAGAAAGTGGATCTTGACAATCCTAAAAACTGCTGGAAGTATTTTAATGCAAATATC
AACACCGGACTTTACTTTACGTACGCCTTGGCAGTCGACTACATCTTGAGACTGTTTGGATTCTTGTAG

>YOL047C 1.72 Hot
ATGTCGACTAGACCCCAACCTGATTGGTATTACCATAGACATCCATATGCCTCTACGCCACTGGCAGAAGGGGAG
GAACCACAATTACTCCCCATCCAAGACCAAGGGAACCATAAGAAATCAAAAATTTGGATGGCATACAAGGCTCCG
ATAGTTCGATGGTACAAGAATGCTATGCTTGTTAAAGACAATTTTTGGAAAGACCTAGAGTCGTCGCACCAGATC
ATCTGGTATCCGTATAAAGGAATTAGCGAATCCGTGGGAAACTCGGATTATTTGCATCTATTTTTCCTAATATTT
GGATACTACCTGCTGAATCTCCTGCTTATTGTTGCGTTTACGTCAATCCTTGCGTGGTCCTTGTTAGTGTGCATC
TATTTGCCCTTTCTCGGACTCTTTGCGTTACCTCTGGCTTATATGCAAACTATTCTGATTTCCACAACGCTATGT
AATTCTATGGTGAAGGGTACCGATTTCGTTCTTTTCACACGTATTTATGGAGTGACGTTCGCAAGAAAGGATTA
ACAGAGTTGAGTGAAGCCTGTGAAACGATAAGTTTCACGCCCTTTGTCTACAGAAGGTCACATCGTTTGGGTGGC
CTCTTTTCGAAACGGTTTTACCTCGTCTCTTTGCCGCAGTTTTTCATTTTTTTCTTCTGGTACATCTTCATAGCC
TTTATGTTTCTTTTATTGTTGCTCGTTCCAATAGTGGGTCCAATTACAATCAATATGCTACCATTTAGCCCCGGA
ATGGGATTTTACTATTTTGAGCCTTATTTTGTTGACGTGCTACATTTGGATTCGCGCAAACTTTCCAAAGTTTAC
TACAAGGGCTTTGCCAAGTGGCTCCTTTACTCGATATCCAGCGGTTTATTAGAGTCTATTCCCATTCTGGGTGGG
TTGTTCATTGGAACAAACGCAGTAGGTGCTTCGCTTTGGATAGTTAAAGAGATAAAAGACCGGGACCAGCCAGCC

GTACCTCCTTCCCCTCCTGCGGAACCAGAAGAGCCTACTGTAGGATCCTATGCACCTCCAATCCAACAATCTATA
GCTCACATTAATCCGCCATGA

>YJL118W 1.85   Hot
ATGGCTTCATGCTTTTCAGTTTCATTATTGGCACGAGTAGCAGTCGTTGAGCCCATCAGAGTGCAACTTTGGTTA
AATGTCGTAAATTGTATGATTGAATCATCCATGCACCAATGTCCTCCACGGGATCGCCACTTTTTTTCTTCATCA
CGCCCCATTTTGTTGATCCGACGTTCTGTATCAACTGTATACAGATTCGTTGCTTCTCGCACCACACAGGTTTTA
CGTGCGGCCAAGACCGTTGTCAAGTGGTTCATTATTGTCGACCCACTGATAAATTCTATTCTAATCAATTATCTC
ATTGATAGGCTCTGCACTCTTGGGCATGCTGTATTGCGAGTGAAAAAGCGCAAGACCGAGGAGAGACAACCCTGT
TCTCCCATTATACAACACACACACGTTAAGAGACGCAAACGGCCTCGTCTGAGGATCGTAGCCATCAAGAGGAAG
CGGAGGCGTCGAAGACCACATCGCATCGAGCGTCCACTCTCAAATATGTACCCAATAATGGAAATCCAGATGGTG
GCTGTTCCACTTGCTCTTCCCAGTCCAACTGCACTGGTGCATTATCAGCAGCAGCAACAGCAGCTCCCGCAACAT
CATCCCTGGTACGACCTCTCGCTTTCTGAAGAAGCCCTCTCGACCTGCTGTTGTAGTTGA

>YDR297W 1.72   Hot
ATGAACGTAACATCGAATGCAACTGCAGCCGGTTCCTTTCCACTAGCATTTGGTCTCAAGACCTCATTTGGGTTT
ATGCACTATGCCAAGGCCCCTGCCATTAATTTACGCCCCAAGGAATCCTTGCTGCCGGAAATGAGTGATGGTGTG
CTGGCCTTGGTTGCGCCGGTTGTTGCCTACTGGGCGTTGTCTGGTATATTCCATGTAATAGACACTTTCCATCTG
GCTGAGAAGTACAGAATTCATCCGAGCGAAGAGGTTGCCAAGAGGAACAAGGCGTCGAGAATGCATGTTTTCCTT
GAAGTGATTCTACAACATATCATACAGACCATTGTTGGCCTTATCTTTATGCACTTCGAGCCGATCTACATGACT
GGGTTTGAAGAAAATGCCATGTGGAAGCTTCGTGCAGACCTTCCTCGGATTATTCCAGATGCCGCTATTTATTAC
GGCTATATGTACGGAATGTCCGCTTTGAAGATCTTTGCAGGCTTTTTATTCGTTGATACATGGCAATACTTTTTG
CATAGATTGATGCATATGAATAAGACCTTATACAAATGGTTCCACTCTGTTCATCATGAACTATACGTGCCATAT
GCTTACGGTGCTCTTTTCAACAATCCTGTTGAGGGCTTCTTGTTAGATACTTTGGGAACCGGTATTGCCATGACG
TTAACTCATTTGACTCACAGAGAGCAAATCATTCTTTTTACCTTTGCCACCATGAAGACTGTCGATGACCACTGT
GGGTATGCTTTGCCACTTGACCCATTCCAATGGCTTTTCCCTAATAACGCTGTCTATCACGATATCCACCACCAG
CAATTTGGTATCAAGACGAACTTTGCTCAACCATTTTTCACTTTCTGGGACAATTTGTTCCAAACTAACTTTAAA
GGGTTTGAAGAATATCAAAAGAAGCAAAGACGTGTCACCATCGACAAGTACAAAGAGTTTTTGCAAGAGAGAGAA
TTGGAAAAGAAGGAGAAACTCAAAAACTTCAAAGCTATGAATGCTGCTGAAAATGAAGTAAAGAAAGAGAAATAA

>YOL058W 1.73   Hot
ATGTCTAAGGGAAAAGTTTGTTTGGCTTATTCTGGTGGTTTAGATACCTCCGTCATTTTGGCTTGGCTACTAGAC
CAAGGCTACGAAGTTGTAGCTTTCATGGCTAATGTAGGGCAAGAAGAAGATTTCGATGCCGCCAAGGAAAAGGCC
TTGAAGATCGGTGCCTGCAAGTTCGTTTGTGTGGATTGTCGTGAAGATTTTGTCAAGGATATTCTATTCCCAGCT
GTACAGGTCAACGCTGTGTACGAAGACGTTTATCTGTTGGGTACCTCTTTGGCAAGACCTGTTATTGCCAAAGCC
CAAATTGACGTCGCTAAACAGGAGGGCTGTTTCGCGGTCTCTCATGGTTGTACCGGTAAAGGTAATGATCAAATC
AGATTCGAATTGTCATTTTACGCTCTGAAGCCAGACGTTAAGTGTATTACACCATGGAGAATGCCTGAATTTTTC
GAAAGATTTGCTGGCAGAAAGGATTTGTTAGACTATGCTGCACAAAAGGGTATTCCCGTCGCCCAAACCAAGGCC
AAGCCATGGTCTACTGACGAAAACCAAGCCCACATTTCTTACGAGGCAGGTATCTTGGAAGACCCAGATACCACC
CCACCAAAGGACATGTGGAAATTGATCGTCGATCCAATGGATGCTCCGGACCAACCACAAGATTTGACCATTGAC
TTTGAACGTGGTCTTCCAGTCAAGTTGACCTACACCGACAACAAGACTTCCAAGGAAGTTTCCGTTACCAAGCCT
TTGGATGTTTTCTTGGCCGCATCCAACTTAGCAAGGGCCAACGGTGTTGGTAGAATCGATATTGTAGAAGATCGT
TACATTAACTTGAAATCCAGAGGTTGTTACGAACAGGCTCCATTGACTGTTTTGAGAAAAGCTCATGTTGATTTG
GAAGGTTTGACTTTAGACAAAGAAGTCCGTCAATTGAGAGACTCATTCGTCACACCAAACTACTCCAGATTGATA
TATAACGGTTTCCTACTTCACCCAGAGTGTGAGTACATCAGATCTATGATCCAACCATCCCAAAATAGCGTTAAC
GGTACTGTCAGGGTTAGACTGTATAAGGGTAACGTCATCATTCTGGGCAGATCTACAAAGACTGAAAAGTTGTAC
GATCCGACAGAATCCTCTATGGATGAGTTGACCGGTTTCTTACCTACCGATACCACCGGTTTCATTGCCATCCAG
GCCATTAGAATTAAAAAATACGGTGAATCCAAAAAAACCAAAGGTGAAGAGTTGACTTTGTAA

>YEL044W 1.96   Hot
ATGAGCGGTAGTAGGGGCAATAGCAGCAATAGTAGCGTCAGTAATAATAGTAATAATAACAATAATAATGACGGG
GGCGACGAGAGATTGCTGTTTCTAAGAAGCGTGGGCGAACGCAATGAAATTGGCTTTCCCTCTAGATTCAAGTCG
GCGCATTACAAGAAACCGACAAGAAGACACAAATCAGCGAGGCAGTTGATCTCGGACGAAAACAAGCGGATCAAC
GCCTTGTTGACCAAGGCTAACAAAGCTGCAGAGAGTTCTACTGCTGCTAGGCGACTTGTGCCCAAAGCGACGTAC
TTTAGCGTGGAAGCGCCACCGTCTATCAGGCCTGCCAAGAAGTACTGCGATGTTACTGGGTTGAAGGGCTTCTAC
AAGTCGCCTACGAACAACATTCGGTATCACAACGCAGAAATCTATCAGTTAATCGTAAAGCCGATGGCCCCCGGC
GTCGATCAGGAGTACTTAAAATTGAGAGGGGCCAACTTCGTTCTAAAATAG

>YFR017C 1.56   Hot
ATGACAGATCCCCACTTGAACACGCCCCAAGTGAGCACGTCACCCACATTTGAAAGATCACAGGACTTCCTCAAC
ATCGACGAACCGCCCTGTGCACAGGAAACACCTAGTGTTTCTACATTCAACCTCCCGGGTCCAAGCGCTCCCGCT
CAAAGCGTAGACAAGCCAGTCCCCATGATTAGACGGCGGTCTACCAATTATATGGACGCACTAAATTCCAGGGAA
CAAGCCCGGGAACGCGAAAGCAGTATCGGGGAGCACGCCCCGGGAGCCGAACGTAGGAGTAGCGGACCCATGGAT
TTCCAGAATACTATCCACAATATGCAATACAGGACCACTAACGACTCTGACCTGAGCCACGCTGGCGTGGATATG
GGTGACTCCATCTCCCATACACCGATCTGTTCTCGTGCTGGCAACAGACCCATTTTCAAAAACTCGTACCTTGAC
AACAACAGCAATGGTAACAGCGCAAGAGTCCCACACGGCTCTCCTCCACAGTTGGGCACGCGTAGAAAGTCGTCT
TTTAAGTACGAGGACTTTAAGAAGGACATCTATAACCAGCTTCACATGTTTGGAGAGAAGTAA

>YER115C 1.53 Hot
ATGGCTGTTTCTAATATATGGCAATCGTACTCTTCCTCCAATTTACATTGGATTTATCCTCTGTATACCAATAAC
TGTTCACAAAATGTAAAGTCATCATTTACAGCAGAAATATTATTGAAAAGAAGATGTAATGATATTCAAGATATA
CTTAATGACAGGATGATTGAATTGTTGCTACAGGGCGCGTGTGACCCAAATAAGCAGCAGAATTACCTTCAGGGG
ATGAGTCCAAGTAGGAAGAAGAAAACCCACGTTAAGAAATTTTTGAAAAAGCAAAAAAAATCAAGGAAACCAATC
ACTCTGGAGCATGGTTGCTTGTCAGGACCCGTGACTCTACGTTTCGGAAATTTTGCAGGAATCAGAGATTTGAGA
GGTACCAGGTGCCCGCTCCACGGCATAAAACATGGTGTCCATCCAAAACCCGGGGAGAGGTGTGCATGTCAGCAA
GCTACCCTCTTTCCAAGTCCACTAGCTCGGTTTAGTTGTGACCAGTCTGCTGTACTTGGTTGCGCTGCCTCCTCC
ACACGGGTCTATGACAGTATAGCCGATGAGTTTTCATCCCTATATTTTTAA

>YFL066C 2.15 Hot
ATGGCAGACACACCCTCTGTGGCAGTACAGGCCCCACCGGGCTATGGTAAGACGGAGTTATTTCATCTCCCCTTG
ATAGCACTGGCGTCTAAGGGCGACGTGAAATATGTGTCGTTTCTGTTTGTACCGTACACAGTGTTGCTTGCTAAT
TGCATGATCAGGTTGGGCCGACGCGGTTGCTTGAATGTGGCCCCTGTAAGAAACTTTATTGAAGAAGGTTACGAT
GGCGTTACTGATTTATACGTGGGGATCTACGATGATCTTGCTAGCACTAATTTCACAGACAGGATAGCTGCGTGG
GAGAATATTGTTGAGTGCACCTTTAGGACCAACAACGTAAAATTGGGTTACCTCATTGTAGATGAGTTTCACAAC
TTTGAAACGGAGGTCTACCGGCAGTCGCAATTTGGGGGCATAACTAACCTTGATTTTGACGCTTTTGAGAAAGCA
ATCTTTTTGAGCGGCACAGCCCCTGAGGCTGTAGCTGATGCTGCGTTGCAGCGTATTGGGCTTACGGGACTGGCC
AAGAAGTCGATGGACATCAACGAGCTCAAACGGTCGGAAGATCTCAGCAGAGGTCTATCCAGCTATCCAACACGG
ATGTTTAATCTAATCAAGGAGAAATCCGAGGTGCCTTTAGGGCATGTTCATAAAATTTGGAAGAAAGTGGAATCA
CAGCCCGAAGAAGCACTGAAGCTTCTTTTAGCCCTCTTTGAAATTGAACCAGAGTCGAAGGCCATTGTAGTTGCA
AGCACAACCAACGAAGTGGAAGAATTGGCCTGCTCTTGGAGAAAGTATTTTAGGGTGGTATGGATACACGGGAAG
CTGGGTGCTGCAGAAAAGGTGTCTCGCACAAAGGAGTTTGTCACTGACGGTAGCATGCAAGTTCTCATCGGAACG
AAATTAGTGACTGAAGGAATTGACATTAAGCAATTGATGATGGTGATCATGCTTGATAATAGACTTAATATTATT
GAGCTCATTCAAGGTGTAGGGAGACTAAGAGATGGGGGCCTCTGTTATCTATTATCTAGAAAAAACAGTTGGGCG
GCAAGGAATCGTAAGGGTGAATTACCACCAATTAAGGAAGGCTGTATAACCGAACAGGTACGCGAGTTCTATGGA
CTTGAATCAAAGAAAGGAAAAAAGGGCCAGCATGTTGGATGCTGTGTCTGCTGA

>YFR036W 1.75 Hot
ATGATCAGAAGGGCCCCTACCACCTTGCAGCTCAGTCACGACGACGTAACCTCTCTGATCGATGACCTGAACGAG
CAGAAACTCAAGCAGCAGCTGAATATCGAGAAGACAAAATACTTCCAAGGAAAAAATGGCGGATCGCTGCACTCC
AATACAGACTTTCAGGACACATCGCAGAATATCGAAGACAACAATAACGATAACGATAACGATATCGATGAAGAT
GACGACATGTCATCTTACAACGACAAAGCAGCCTCGGTAGCGCACACCAGAGTCCTCAATTCCTTGCATCTGTCC
ACCGACAGCAATACCGCCCACGAGACGTCCAATGCAAACGACAACCACAACCCCTTCTACATCCGCGAGGAATAA

>YHR217C 1.66 Hot
ATGTCCCTACGGCCTTGTCTAACACCATCCAGCATGCAATACAGTGACATATATATACACACACCACACCCACAC
CCACACCCACACCCACACACACCCACACACACACACCCACACACACCCACACCCACACCACACCCACACCCACAC
ACACCACACCCACACACCACACCCACACCCACACCACACCACACCCACACACCACACACCACCCTATCTAACCTG
TCTCTTAACCTACCCTCACATTACCCTACCTCCCCACTCGTTACCCTGCCCCACTCAACCATACCACTCCCAACC
ACCATCCATCTCTCTACTTACTACTACCATCCACCGCCCATCATAACCGTTACCCTCCAATTACCCATATCCAAC
TCCACTACCATTACCCTGCTATTACCCTACCATCCACCATGTCCTACTCACTGTACTGTTGTTCTACCCTCCATA
TTGAAACGTTAA

>YBR128C 1.55 Hot
ATGCATTGCCCAATTTGCCACCATAGAGCGCATGTAGTGTACTGTGCACATTGTATCAATACGAGCCCAAGTCTG
CTACTGAAGCTAAAACTAGATTTAATTTTATTAAAGGATGAGAATAAAGAACTTAACGGGAAAGTCGAACAAATA
TTAAACGAGGCCATGAACTATGACCAGTTAGATATCAAGAGGATGGAGAAAAAGAAAGATCCCCTGATGAATAGT
CTCATGAAGTTAGATGTTTTACGAATGAAAAAGAATAATAACCTGATTAGGCACAGGATAGAACAGCTAAATGAG
CGGATTTATAGTAAAAGGAATCACATCAGCGAATTGAAAGTAGAAATTGACAATTATAAATGTTATAAGGTGGGT
ACTGGTACGGACAAATTAAGAGAGCAAGTGGAAATTAGTGATGCAAAAAATAAGCTGGCACAAGTATCGAAGATT
TGTGAATCGGCTAGAGATTACAAGCTCAATCTACTAAATAATTGGTTTGTGATCCAGAAGCTGCAAGATAATTTC
CAAATACCTTTCGCTATAGCTTTTCAGCCACTAATATCTTTGAAAAACTTCCGTATTTTACCTCTTGCTATAACA
AACGATTCCATCAACATCATGTGGAAGTATATTAGCTTCTTCTCAGACATTCTTATGATTAAACTTCCCTACACA
AATAAAATCTGTGAACAACCCATGTTTGAGTTTTCCGATAGCATACAGACAGTAGTACAAAGGTTGATCAAGCTT
ATCATAAATATTTTACAGATATGTAGACATTTGAAACTCGTACCTTCAACACCCATGGATATCCCATGGCTACTG
GACCAGTACGATGTGGATGGGCTGTTCTATAATATGGTGAAACGGAACAAGATGAAGTGTAGGTCCGTCTCGCTA
TATTGGACTTTTGGGATGTTGTACTCGATGGTTTTGGATAACATGAATAATCCACAAAGAGGACATCCTGCAAGG
CGGACCGCACCACCTCCAACAGTCACAGGACCTCATGACCGATGGTACGTGGTAGGCTAG

>YPL111W 2.13 Hot
ATGGAAACAGGACCTCATTACAACTACTACAAAAATCGCGAATTGTCCATCGTTCTGGCTCCATTCAGCGGCGGT
CAGGGTAAGCTTGGTGTCGAGAAGGGCCCTAAATACATGCTTAAGCATGGTCTGCAAACAAGCATAGAGGATTTG
GGCTGGTCTACGGAATTAGAGCCCTCAATGGACGAGGCCCAATTTGTGGGAAAGTTGAAAATGGAGAAGGACTCC
ACAACTGGGGGTTCCTCTGTTATGATAGACGGTGTCAAGGCTAAAAGAGCAGATTTGGTTGGTGAAGCCACCAAG
TTGGTGTACAACTCCGTGTCGAAAGTGGTCCAGGCGAACAGATTCCCCTTGACCTTGGGTGGTGATCATTCAATA

GCCATTGGTACTGTATCCGCGGTTTTGGACAAATACCCCGATGCTGGTCTTTTATGGATAGACGCCCACGCTGAT
ATAAACACCATAGAAAGCACCCCCTCTGGAAACTTGCACGGCTGTCCCGTGTCATTCCTAATGGGTTTGAACAAG
GATGTCCCACATTGTCCCGAGTCGCTCAAATGGGTTCCAGGCAACTTGAGCCCAAAAAAGATCGCGTATATTGGG
TTGAGAGATGTTGATGCCGGAGAAAAGAAAATCTTGAAAGATCTGGGTATCGCCGCCTTTTCCATGTACCACGTT
GACAAATACGGCATCAACGCTGTCATTGAAATGGCAATGAAAGCCGTGCACCCAGAAACAAACGGTGAAGGTCCC
ATTATGTGCTCCTATGACGTCGATGGTGTAGACCCATTATACATTCCTGCTACAGGTACTCCAGTGAGAGGTGGG
TTGACCTTGAGAGAAGGTCTTTTCTTGGTGGAAAGATTGGCCGAATCCGGTAATTTAATTGCGCTAGACGTTGTT
GAATGTAACCCTGATCTGGCTATTCATGATATCCATGTTTCAAACACCATCTCTGCAGGTTGCGCCATTGCAAGG
TGTGCATTGGGTGAAACCTTATTGTAG

>YPL187W 1.61   Hot
ATGAGATTTCCTTCAATTTTTACTGCAGTTTTATTCGCAGCATCCTCCGCATTAGCTGCTCCAGTCAACACTACA
ACAGAAGATGAAACGGCACAAATTCCGGCTGAAGCTGTCATCGGTTACTTAGATTTAGAAGGGGATTTCGATGTT
GCTGTTTTGCCATTTTCCAACAGCACAAATAACGGGTTATTGTTTATAAATACTACTATTGCCAGCATTGCTGCT
AAAGAAGAAGGGGTATCTTTGGATAAAAGAGAGGCTGAAGCTTGGCATTGGTTGCAACTAAAACCTGGCCAACCA
ATGTACAAGAGAGAAGCCGAAGCTGAAGCTTGGCATTGGCTGCAACTAAAGCCTGGCCAACCAATGTACAAAAGA
GAAGCCGACGCTGAAGCTTGGCATTGGCTGCAACTAAAGCCTGGCCAACCAATGTACAAAAGAGAAGCCGACGCT
GAAGCTTGGCATTGGTTGCAGTTAAAACCCGGCCAACCAATGTACTAA

>YIL153W 2.22   Hot
ATGTCTCTGGATCGTGTAGATTGGCCGCACGCCACCTTCTCTACGCCGGTGAAGCGTATCTTCGATACGCAGACA
ACGCTGGACTTCCAATCGTCACTAGCCATTCACAGAATCAAGTATCATCTTCACAAGTACACTACGTTGATATCG
CACTGTTCTGACCCCGACCCGCACGCTACTGCGTCGTCGATCGCCATGGTTAACGGGCTGATGGGAGTGTTGGAC
AAGCTGGCACACCTGATCGACGAGACACCGCCTCTTCCAGGTCCTAGAAGGTATGGTAACCTAGCATGCCGCGAG
TGGCATCACAAGCTGGATGAACGTTTGCCTCAGTGGCTGCAGGAAATGCTTCCCTCGGAATACCATGAGGTGGTG
CCTGAATTGCAGTATTACCTGGGCAACAGCTTTGGTTCGTCGACAAGATTGGACTACGGCACGGGCCACGAACTT
TCGTTCATGGCCACGGTCGCAGCACTGGACATGCTGGGGATGTTTCCGCACATGAGGGGGGCAGACGTTTTTCTG
CTGTTCAACAAGTACTACACGATCATGAGGAGGCTGATCCTGACGTATACGCTGGAGCCCGCTGGGTCACATGGC
GTGTGGGGGCTTGATGACCATTTCCACTTGGTGTACATCTTGGGCTCGTCTCAATGGCAGCTGCTTGATGCGCAA
GCACCCTTGCAACCAAGAGAAATACTGGATAAGTCTCTGGTGCGCGAGTATAAGGACACCAACTTCTACTGCCAA
GGCATAAACTTTATTAATGAGGTGAAAATGGGGCCCTTTGAAGAGCATTCCCCCATCCTATACGATATTGCCGTC
ACCGTTCCACGCTGGTCCAAAGTGTGTAAGGGGCTTCTAAAAATGTATTCTGTAGAGGTTCTGAAGAAGTTCCCC
GTGGTGCAACATTTCTGGTTCGGCACAGGCTTCTTTCCCTGGGTCAACATTCAAAACGGCACTGATCTTCCAGTC
TTTGAAGAAAAGGAGGAAGAGAGTATAGAACAGGCTAACGCAGGATCGCCCGGTCGAGAACAGACTTCAACACGA
TTCCCAACGTCCACGTCAATGCCTCCCCCAGGCGTTCCGCCGTCAGGTAACAATATCAACTATTTACTAAGCCAT
CAAAACCAGTCGCACCGGAACCAGACTTCTTTTTCAAGAGATAGACTACGTAGATAA

>YOL059W 1.96   Hot
ATGCTTGCTGTCAGAAGATTAACAAGATACACATTCCTTAAGCGAACGCATCCGGTGTTATATACTCGTCGTGCA
TATAAAATTTTGCCTTCAAGATCTACTTTCCTAAGAAGATCATTATTACAAACACAACTGCACTCAAAGATGACT
GCTCATACTAATATCAAACAGCACAAACACTGTCATGAGGACCATCCTATCAGAAGATCGGACTCTGCCGTGTCA
ATTGTACATTTGAAACGTGCGCCCTTCAAGGTTACAGTGATTGGTTCTGGTAACTGGGGGACCACCATCGCCAAA
GTCATTGCGGAAAACACAGAATTGCATTCCCATATCTTCGAGCCAGAGGTGAGAATGTGGGTTTTTGATGAAAAG
ATCGGCGACGAAAATCTGACGGATATCATAAATACAAGACACCAGAACGTTAAATATCTACCCAATATTGACCTG
CCCCATAATCTAGTGGCCGATCCTGATCTTTTACACTCCATCAAGGGTGCTGACATCCTTGTTTTCAACATCCCT
CATCAATTTTTACCAAACATAGTCAAACAATTGCAAGGCCACGTGGCCCCTCATGTAAGGGCCATCTCGTGTCTA
AAAGGGTTCGAGTTGGGCTCCAAGGGTGTGCAATTGCTATCCTCCTATGTTACTGATGAGTTAGGAATCCAATGT
GGCGCACTATCTGGTGCAAACTTGGCACCGGAAGTGGCCAAGGAGCATTGGTCCGAAACCACCGTGGCTTACCAA
CTACCAAAGGATTATCAAGGTGATGGCAAGGATGTAGATCATAAGATTTTGAAATTGCTGTTCCACAGACCTTAC
TTCCACGTCAATGTCATCGATGATGTTGCTGGTATATCCATTGCCGGTGCCTTGAAGAACGTCGTGGCACTTGCA
TGTGGTTTCGTAGAAGGTATGGGATGGGGTAACAATGCCTCCGCAGCCATTCAAAGGCTGGGTTTAGGTGAAATT
ATCAAGTTCGGTAGAATGTTTTTCCCAGAATCCAAAGTCGAGACCTACTATCAAGAATCCGCTGGTGTTGCAGAT
CTGATCACCACCTGCTCAGGCGGTAGAAACGTCAAGGTTGCCACATACATGGCCAAGACCGGTAAGTCAGCCTTG
GAAGCAGAAAAGGAATTGCTTAACGGTCAATCCGCCCAAGGGATAATCACATGCAGAGAAGTTCACGAGTGGCTA
CAAACATGTGAGTTGACCCAAGAATTCCCATTATTCGAGGCAGTCTACCAGATAGTCTACAACAACGTCCGCATG
GAAGACCTACCGGAGATGATTGAAGAGCTAGACATCGATGACGAATAG

>YFR037C 2.64   Hot
ATGAGCGACACTGAAAAGGATAAGGATGTCCCTATGGTAGACTCGCACGAAGCGACCGAGGAGCCACCCACCACA
AGCACCAACACGCCATCTTTCCCTCACTTAGCACAGGAACAGGCGAAGGAGGAATCTGCCACATTGGGAGCAGAA
GTAGCTCATAAGAAAATCAATTACGAGCAAGAAGCACAGAAACTGGAGGAGAAGGCCCTTAGGTTCCTGGCAAAG
CAAACTCACCCGGTGATTATTCCGTCGTTTGCCTCTTGGTTTGATATTTCGAAGATCCACGAGATCGAAAAAAGA
TCCAATCCCGACTTTTTCAACGATTCATCAAGGTTCAAGACACCAAAGGCATATAAGGACACAAGAAATTTTATC
ATCAACACGTACCGTCTTTCGCCGTACGAATATTTGACCATTACCGCTGTGAGAAGAAATGTTGCCATGGATGTT
GCCTCGATAGTGAAGATTCACGCGTTCTTGGAAAAATGGGGCTTAATCAATTATCAGATTGACCCCAGGACCAAG
CCCAGTCTTATTGGGCCAAGTTTTACGGGCCACTTCCAAGTGGTTCTGGACACTCCGCAGGGGTTAAAGCCATTT
TTACCAGAGAATGTGATCAAGCAAGAAGTGGAAGGAGGAGATGGAGCGGAACCACAAGTCAAGAAGGAATTTCCC

GTTAATCTCACAATCAAGAAGAACGTTTACGATTCTGCACAAGACTTCAATGCATTACAAGACGAAAGTAGAAAC
TCCAGGCAGATTCACAAAGTTTACATTTGCCATACATGCGGTAACGAGTCAATCAATGTGCGCTACCACAATTTG
CGTGCACGGGACACCAACCTGTGCTCCCGTTGTTTCCAAGAGGGTCATTTCGGTGCCAACTTTCAATCTTCAGAT
TTCATCAGATTAGAAAACAACGGAAACTCGGTTAAAAAAAACTGGTCAGACCAGGAGATGCTACTATTGCTGGAG
GGTATTGAAATGTATGAAGACCAGTGGGAGAAGATTGCTGACCACGTGGGTGGGCACAAGCGTGTAGAAGACTGC
ATTGAAAAATTCCTAAGCTTACCGATCGAGGACAACTACATCCGAGAAGTTGTTGGTTCAACGCTGAATGGTAAG
GGTGGCGACAGCCGCGATGGTAGTGTGTCCGGTTCGAAGTTGATGGAATGCGTGAATGATGCTGTCCAGACGCTA
CTGCAAGGCGACGACAAATTGGGTAAGGTCTCTGATAAGTCGAGAGAGATCTCCGAAAAGTACATTGAAGAAAGC
CAAGCGATAATCCAAGAGTTAGTCAAGCTGACCATGGAGAAATTAGAGAGCAAGTTTACAAAGCTGTGCGATCTA
GAAACGCAACTGGAGATGGAAAAACTGAAATATGTGAAGGAATCTGAAAAGATGCTGAACGACCGATTATCACTG
AGTAAACAGATTCTTGACCTGAACAAGTCGCTGGAGGAGTTGAATGTGTCGAAGAAACTGGTACTGATCTCGGAG
CAAGTAGACTCGGGCATACAACTAGTGGAGAAGGACCAGGAGGGCGATGACGAAGACGGCAATACGGCCACAGGA
CATGGCGTGAAACGTGTAGGCAAGGAAGGCGAGGAAGTAGGCGAAGGCGACTCCATTGCAAAATTGCAGCCCCAG
GTGTACAAACCGTGGTCATTGTAA

>YGR160W 1.56 Hot
ATGACACCACCGATGTGTTCGAATTCCTTCTTCAACCATTCGTCATCAATAGACCACGATAGTCTACCAACGAAA
ATAGTAGCTGGTTCTTCGGTTTCTTCATTTTTTTGCTTCTTGTTGGAAGACTCTTCGTCTTCTTCTTCTTCGGCG
TCCTCAGATTTACGTTTCTTATCGTTAGACTCTTCCTTTTCGCTTTCGCTATCAGAAGATGAAGATGAGGATGAA
TCAGAGCTAGAAGATTCTTTTGATTCTTCCTTCTTGGTTTCTTCTTTTTCTTCTTCTTCTTCTTCGTCGGAAGAA
GAGTCAGAGGAAGAGGAAGAGGAATCCTTGGATTCTTCCTTCTTGGTTTCAGCTTCACTTTCGCTGTCAGAAGAC
GATGAAGAAGAGGATTCAGAATCAGAGGATGAAGATGAAGATGAAGATTCAGATTCAGACTCAGACTCAGATTCA
GATTCAGATGAAGATGAAGATGAGGATGAAGATTCGGAGGAAGAGGAAGAGACGGCCTTAGCTTTTTCTTCTTTG
GCTTGTTTGGAAGCCTTAACTTCCTTCTTGTTACCTTTTACTTTAGTAGTCTTAGCCATCTTATTTTATCCTGCC
TGGGTTGAGTGA

>YAL036C 1.96 Hot
ATGTCTACTACAGTTGAAAAAATCAAAGCTATCGAAGATGAAATGGCCCGTACCCAAAAGAACAAGGCCACATCT
TTCCATTTGGGTCAACTGAAGGCCAAGCTGGCCAAACTGAGAAGAGAATTGTTGACCAGTGCTTCATCCGGCAGC
GGTGGTGGTGCTGGTATTGGTTTTGATGTGGCTAGAACTGGTGTGGCCAGTGTGGGGTTTGTCGGGTTCCCGTCG
GTGGGGAAATCTACATTACTGTCCAAGTTGACTGGTACTGAGTCTGAAGCAGCTGAGTACGAGTTTACCACCCTG
GTTACCGTCCCCGGTGTCATTCGTTATAAAGGTGCCAAGATCCAAATGTTGGATTTACCTGGTATTATCGATGGT
GCTAAGGATGGTAGAGGTAGAGGTAAGCAAGTTATTGCCGTGGCAAGAACCTGTAACCTGTTATTTATCATCCTA
GATGTGAACAAACCCTTGCATCATAAGCAAATCATTGAGAAGGAACTGGAAGGTGTGGGGATTCGTCTGAATAAA
ACTCCGCCAGATATCTTGATCAAAAAAAAAGAGAAAGGTGGTATTTCCATCACAAACACAGTCCCATTGACCCAT
CTGGGGAATGACGAAATCAGAGCCGTTATGAGCGAGTACAGAATAAATAGCGCTGAGATTGCCTTCAGGTGTGAT
GCCACTGTGGATGATTTGATTGATGTTTTGGAAGCTTCGTCAAGAAGATACATGCCTGCCATCTATGTGTTAAAC
AAGATTGATTCTCTGTCAATAGAGGAATTGGAATTACTTTACCGAATTCCTAATGCCGTGCCTATTTCGTCTGGT
CAAGATTGGAACTTGGACGAGCTGTTGCAAGTCATGTGGGATAGACTAAATCTAGTCCGTATTTACACTAAACCA
AAGGGCCAAATACCAGATTTTACCGACCCTGTGGTGCTAAGATCAGACCGTTGCAGTGTCAAGGATTTTTGTAAC
CAAATTCATAAATCTTTAGTGGACGACTTTAGAAATGCTCTGGTTTACGGTAGCAGTGTCAAACATCAACCTCAA
TACGTGGGGTTGAGTCACATTTTGGAAGACGAAGATGTTGTTACCATCTTGAAAAAGTGA

>YOL093W 1.55 Hot
ATGTCCAATGATGAGATAAACCAGAACGAGGAAAAGGTGAAAAGAACACCACCTTTACCGCCTGTGCCAGAAGGC
ATGTCTAAAAAGCAATGGAAAAAAATGTGTAAAAGACAGAGATGGGAGGAGAATAAAGCCAAATACAATGCGGAA
CGCCGTGTAAAGAAGAAGAGACTTCGCCATGAAAGAAGTGCGAAGATTCAAGAATACATTGACAGGGGAGAAGAG
GTCCCGCAGGAATTGATTAGAGAGCCTCGTATCAATGTGAATCAGACTGATTCTGGGATAGAGATCATACTTGAC
TGTTCTTTTGATGAGTTAATGAACGACAAGGAAATAGTAAGCTTGTCTAACCAGGTCACCAGGGCATACTCCGCG
AATAGAAGGGCGAACCATTTTGCAGAAATAAAGGTGGCCCCATTCGACAAAAGACTAAAGCAAAGGTTTGAAACT
ACTCTGAAAAATACCAATTATGAAAACTGGAACCATTTTAAATTCCTACCAGACGACAAAATCATGTTTGGAGAT
GAGCATATAAGTAAGGACAAAATAGTGTATTTAACGGCCGATACAGAGGAAAAGCTGGAGAAGCTGGAACCCGGG
ATGCGATACATTGTTGGCGGCATTGTAGACAAGAATCGTTATAAAGAATTGTGTCTGAAGAAGGCCCAAAAGATG
GGCATTCCTACGCGAAGATTGCCCATTGACGAGTACATCAATCTCGAAGGCAGAAGAGTGTTGACGACAACGCAC
GTCGTGCAACTCATGCTGAAATACTTTGATGACCATAACTGGAAAAATGCCTTTGAAAGCGTTTTACCACCCAGA
AAGCTGGATGCGGAAGCAAAATCCGCAAGCTCTTCGCCAGCTCCAAAGGACACATAG

>YDR073W 2.14 Hot
ATGAGCAGTGAAATTGCCTACTCGAATACGAACACCAACACTGAAAACGAGAACCGCAATACTGGCGCTGGCGTA
GATGTAAATACAAATGCAAATGCAAATGCAAATGCAACTGCAAATGCAACTGCAAATGCAACTGCAAATGCAACT
GCAGAGCTGAACCTCCCCACGGTCGATGAGCAAAGACAGTATAAGGTACAACTGCTATTGCATATCAACAGCATA
TTACTTGCTAGAGTTATTCAGATGAATAATAGTTTACAAAACAATCTACAGAACAATATAAATAATAGCAATAAC
AATAACATCATCAGGATACAGCAACTTATATCTCAGTTCCTTAAAAGGGTTCATGCCAATCTTCAATGCATATCT
CAGATAAACCAAGGAGTGCCCTCAGCGAAACCACTGATCCTCACGCCTCCTCAGCTAGCCAACCAGCAGCAACCT
CCACAGGATATTCTTTCTAAACTCTATCTTCTCTTGGCAAGAGTGTTCGAGATATGGTAG

>YGL148W 1.56 Hot

ATGTCAACGTTTGGGAAACTGTTCCGCGTCACCACATATGGTGAATCGCATTGTAAGTCTGTCGGTTGCATTGTC
GACGGTGTTCCTCCAGGAATGTCATTAACCGAAGCTGACATTCAGCCACAATTGACCAGAAGAAGACCGGGTCAA
TCTAAGCTATCGACCCCTAGAGACGAAAAGGATAGAGTGGAAATCCAGTCCGGTACCGAGTTCGGCAAGACTCTA
GGTACACCCATCGCCATGATGATCAAAAACGAGGACCAAAGACCTCACGACTACTCCGACATGGACAAGTTCCCT
AGACCTTCCCATGCGGACTTCACGTACTCGGAAAAGTACGGTATCAAGGCCTCCTCTGGTGGTGGCAGAGCTTCT
GCTAGAGAAACGATTGGCCGTGTCGCTTCAGGTGCCATTGCTGAGAAGTTCTTAGCTCAGAACTCTAATGTCGAG
ATCGTAGCCTTTGTGACACAAATCGGGGAAATCAAGATGAACAGAGACTCTTTCGATCCTGAATTTCAGCATCTG
TTGAACACCATCACCAGGGAAAAAGTGGACTCAATGGGTCCTATCAGATGTCCAGACGCCTCCGTTGCTGGTTTG
ATGGTCAAGGAAATCGAAAAGTACAGAGGCAACAAGGACTCTATCGGTGGTGTCGTCACTTGTGTCGTGAGAAAC
TTGCCTACCGGTCTCGGTGAGCCATGCTTTGACAAGTTGGAAGCCATGTTGGCTCATGCTATGTTGTCCATTCCA
GCATCCAAGGGTTTCGAAATTGGCTCAGGTTTTCAGGGTGTCTCTGTTCCAGGGTCCAAGCACAATGACCCATTT
TACTTTGAAAAAGAAACAAACAGATTAAGAACAAAGACCAACAATTCAGGTGGTGTACAAGGTGGTATCTCTAAT
GGTGAGAACATCTATTTCTCTGTCCCATTCAAGTCAGTGGCCACTATCTCTCAAGAACAAAAAAACCGCCACTTAC
GATGGTGAAGAAGGTATCTTAGCCGCTAAGGGTAGACATGACCCTGCTGTCACTCCAAGAGCTATTCCTATTGTG
GAAGCCATGACCGCTCTGGTGTTGGCTGACGCGCTTTTGATCCAAAAGGCAAGAGATTTCTCCAGATCCGTGGTT
CATTAA

>YDL125C 1.51 Hot
ATGGAGCCATTGATATCGGCACCGTACCTAACAACAACAAAAATGTCTGCTCCTGCTACGCTTGATGCTGCCTGT
ATTTTTTGCAAGATTATTAAAAGCGAAATTCCATCCTTCAAATTGATTGAAACAAAGTACTCGTATGCTTTCTTG
GACATCCAACCTACTGCTGAAGGTCATGCTTTAATCATTCCTAAGTACCATGGTGCGAAGTTGCATGACATCCCG
GACGAATTCCTTACCGATGCTATGCCGATTGCCAAGAGACTGGCCAAGGCAATGAAGTTGGACACTTATAATGTG
TTGCAGAATAATGGTAAAATTGCGCATCAAGAAGTCGACCACGTCCACTTCCATTTGATTCCTAAGAGAGATGAG
AAAAGTGGTTTGATTGTAGGGTGGCCAGCCCAAGAAACGGACTTCGATAAGTTGGGCAAGCTACACAAGGAATTG
CTTGCCAAACTAGAAGGCTCCGATTAG

>YLR349W 1.61 Hot
ATGGCACCACTGAACATGGAGCAAGGTAAAAGATACGCCATGTTAGTCAATTGCTCGCGTGGGATCACATTCTCT
TTCAACAAGTCGTATGCACCGAATCTCACCGTCGTGTATGTACATTGCCTCAACACAGCAGCGCTCAGGCCGGAA
TATAACCCCACCACACCTTCATTCGCCAAGATACTCTCCAACATCCTGAAGAGTGTGGGCTTGGGCATAGGAGCC
GCCTGCAGTCTGACTTTGGCCAAGTCAAGAGGATGTGTCACCATGGTAGCAAAGATACCCGCTGCACCTCCGTAC
CACCATGGATACTTGATATTCTTACCGGCAGACTCTTTTGCGTTGGTTGACATGCCGTCCAGCTGTTGCGTTATC
TTTTCGACACTTTCAGAGCAAGAGAAACGAACCTCTACACATAAATATGCATACGATTATGTATATCCATATATA
TATATACACTACAAGCCGACCGCCCGACCGTTTCCTGCCCCCAACATTGCAGCGTAA

>YER149C 1.69 Hot
ATGCATAAATTTGATCTAGAGCTCTCGAGAAGAGCGAACCCTTTGCTTTTTAGTGCAGAACGATACGAAGAGTAT
CCATTGAAATACGATGAATTGAAACAGTACCTTCTCTCCCAAAATCCGTCACATCCGCATCACAATAGTAGACCA
TACACTTCCATTGACTATTTTGACTATCTTCTGTATAGAAGTAAAAATGACGAGTCAGAAATAGACCTTGACAAA
AAGCTAGTGTCTGAGTTCGCCCTATACTACGTTCAAAAAGAGCACTTGAACTCTGATGATCTAAATCCCACACTC
AATGAGTTGCTGAAGCTGCAGCCGAAGTCTGCGGATTGGTATGAAATGATGCTGAGGATCCTTGAATCCATTAAC
ACTACAGGGATAGACCAGCTTACGAAGGAAAACAACAACAGTTTCCCAAATTCTAAAAGAGCGAGGTCTTCCACT
AATATGGGTGGCACAGATAAATTTAACAAGGGTGCTTACCATACTGACAAAGCTGATGACGATAAAAATGAAATA
CTTCAAGAATTAACGTCCTTCTTGATGTCAAACTCCATTCAGAAAGGCATTGATATCAAGCCAATACCATTAGAT
GACCCTGTGAAGTTCTTGAAAAATGGGATAAATTCTATCTTGGATACCTGCGTCAGTCTAGAAAAAAATACGTCA
TCACCACCTATTTCGCCTAACGCCGCTGCAATTCAAGAGGAAGACTCCTCTAAAAAATTAGAAGAACTAGAAACG
GCATTTAGTGATTTGCAACTGGCACACAATTTCTTGACTAAGCAATTCGAGAATGACCGTGCCGAATATGTACAA
GATATCGAGAAGCTAACGCGGACTAACAGAGAATTACAAGATAAGTTATTAAACAACCACTCGAATTTGAGTAAA
ACTGAAAAGAAATTACACGAATTGGAGCAGGAAAATAAAGAACTTGAAAAAGCCAACAACAAGCTGAATTCAAGC
AGACATAATTTCGGCATGTCATCACCAGCGAGTTCGCCGGTGACTTGGGATCCAAGTTCACCTTCCTCTGTAGGA
AGCCCCACAAGCGGTTCTGGATCACGTTCACTTTCAATAATGACAAGTGAGTTTAAAAAAGTGCTTACGAGTACA
CAAAGAAAGTATGAAAAAGAATTGTCGGATGAGCGTGAACATAGATTCAAGCTGGAAAGGGAGCTTGCTTTGCTG
AAAAATGCAGAGGCAAACACCTCGCTGGCGCTTAATAGAGACGATCCACCAGATATGCTATGA

>YJR074W 2.7Hot
ATGAAGATTGAAAAGGCTTCTCATATTTCACAACCGGTGCAACTTTCCACATGCACTCTCATTGACACCTACCCT
GGTCATCAAGGAAGCATGAACAACAAGGAAGTCGAATTATACGGCGGCGCCATCACTACTGTCGTCCCACCTGGG
TTTATAGACGCTTCTACTCTAAGAGAAGTCCCCGATACACAGGAGGTGTACGTCAATTCCCGTCGCGATGAAGAA
GAATTCGAAGATGGACTAGCCACCAATGAAAGCATTATCGTGGATCTCTTGGAGACGGTCGACAAGAGCGATCTG
AAGGAGGCTTGGCAGTTCCACGTGGAAGACCTGACGGAGTTGAACGGGACCACCAAATGGGAAGCTTTACAAGAA
GACACTGTTCAGCAAGGAACCAAGTTCACCGGACTCGTTATGGAAGTAGCAAATAAGTGGGGAAAACCAGATCTG
GCACAGACCGTTGTCATCGGCGTAGCGTTGATCAGATTAACCCAGTTTGACACGGATGTGGTCATCTCCATTAAT
GTACCTTTGACAAAGGAGGAGGCGTCGCAAGCAAGCAACAAAGAGTTGCCTGCAAGATGTCATGCTGTTTACCAA
TTGTTGCAAGAAATGGTACGGAAGTTTCACGTTGTGGACACGTCGCTGTTTGCTTAA

>YBL055C 1.61 Hot

ATGTGGGGCATCTTATTGAAATCCTCGAACAAAAGTTGTTCTAGGCTCTGGAAACCAATTTTGACACAATATTAT
AGCATGACATCAACTGCCACGGATTCTCCGCTGAAATACTATGATATTGGATTGAATCTGACAGATCCTATGTTT
CACGGTATTTACAATGGTAAGCAGTACCATCCAGCAGATTATGTAAAATTATTAGAGCGTGCTGCTCAGAGGCAC
GTGAAAAATGCCCTTGTTACAGGATCATCCATAGCGGAGTCTCAAAGTGCCATTGAGTTGGTAAGCAGCGTCAAA
GATCTTAGCCCGTTAAAGCTGTATCACACAATAGGTGTTCACCCATGTTGCGTTAACGAGTTTGCGGATGCTAGT
CAAGGGGACAAGGCTAGCGCTTCTATTGATAACCCTTCCATGGATGAAGCATATAATGAGTCTCTATATGCTAAA
GTTATTAGTAATCCATCTTTTGCACAGGGTAAACTGAAAGAGCTTTATGATTTGATGAATCAGCAAGCAAAACCT
CATGATACAAGTTTCAGGTCAATTGGTGAGATCGGGTTGGACTACGACAGATTTCACTATAGTTCTAAAGAGATG
CAAAAGGTTTTTTTTGAAGAGCAACTGAAAATAAGTTGTTTGAACGATAAACTCAGCAGCTATCCATTGTTTCTG
CATATGAGAAGCGCATGTGACGATTTTGTTCAAATATTGGAAAGATTTATTGCCGGGTTCACTGATGAGAGGGAT
ACCTTTCAGCTACAAAAGTTAGGTGCATCGTCGTCTAGCGGGTTTTACAAATTTCATCCAGATAGAAAACTAGTG
GTCCATTCATTCACTGGTTCTGCGATAGATTTGCAGAAATTACTGAACTTATCACCCAACATCTTCATAGGAGTA
AACGGTTGCTCGTTGAGAACCGAGGAAAATCTAGCTGTTGTAAAGCAAATACCAACAGAAAGGTTGTTATTAGAG
ACAGATGCTCCGTGGTGTGAGATTAAAAGAACGCATGCATCTTTCCAGTACTTAGCCAAATACCAGGAGGTTAGG
GATTTCGAATACCCTGCATTCAAGTCCGTTAAGAAAAATAAGCTCGCTGACAAGTTGAATGCAGAAGAACTTTAC
ATGGTCAAGGGCCGTAATGAGCCTTGTAATATGGAACAAGTAGCAATTGTCGTATCGGAAGTCAAGGACGTGGAT
CTGGCTACTTTGATAGATACCACGTGGAAGACGACCTGTAAGATATTTGGAGAGTAA

>YJL116C 1.54 Hot
ATGAAAATTTCCGCAGCTTTAATATTGTCTTCCCTTTCTTCTGTCGCATTTTCTGCCCCTGCACCTGCTCCAGCG
GACAGTCACCATGAAGATCATCACAAAGATGAAAAACCAGCGGTTGTCACTGTCACTCAATACATAGATTCCAAT
GCCGCTACTAGTACTGTAGAATCTGCTGCTACTACCACTACATTGTCCTCATCTGAGAAGGATACCTCTGAACAG
AAGCGTGATGGCGGATTCCAAGATGGTACTGTCAAATGTTCGGACTTCCCTTCTGTAAACGGTATAGTTTCCTTG
GACTGGCTAGGATTTGGTGGATGGGCCTCTGTCATGGACATGGATGCCAACACTTCGTCCGAATGTAAGGATGGC
TACTACTGTTCTTATGCATGTGAACCTGGAATGTCAAAGACTCAATGGCCTTCTGACCAACCAAGCGATGGTAAA
TCTGTTGGTGGTCTTTACTGTAAAAATGGTTACTTGTACCGTACCAACACTGATACCAGCGATTTATGTTCTACG
GATGAAACATCTGCTAAGGCCATTAACAAAAAGTCTGACTCCATTGCTCTATGTAGGACGGATTACCCAGGATCA
GAAAACATGGTGATTCCCACAGTGGTTGATGGTGGAGATTCACAACCAATTTCAGTCGTTGATGAAGACACTTAT
TATCAATGGCAGGGTAAAAAGACTTCTGCTCAGTACTATATTAACAACGCCGGTGTATCTGCAGAAGATGGGTGC
ATTTGGGGTACTTCTGGTTCGGATGTCGGCAACTGGGCTCCACTAGTGTTAGGTGCTGGTTCCACTAATGGAGAA
ACATACTTGTCGTTGATTCCAAACCCCAACAGTAACCAAGCTGCCAACTTTAACGTTAAAATAGTTGCATCCGAT
GGCGCTAACGTTCAGGGCAGCTGTGCGTATGAAGATGGCTCTTTCACCGGAGATGGTTCCGATGGTTGCACAGTT
TCTGTTTTATCTGGATCTGCTGAATTTGTTTTCTATTAA

>YLL018C 1.52 Hot
ATGTCTCAAGACGAAAATATTGTCAAAGCTGTTGAAGAATCCGCAGAACCTGCTCAAGTTATTCTTGGGGAAGAT
GGTAAGCCATTGTCCAAGAAGGCCTTGAAGAAATTGCAGAAAGAGCAAGAGAAACAGAGAAAGAAGGAGGAAAGA
GCTCTCCAGTTGGAAGCTGAAAGAGAAGCCCGTGAAAAGAAAGCCGCTGCCGAAGACACCGCAAAGGACAACTAC
GGTAAGTTGCCATTGATCCAGTCTCGTGACTCTGACAGAACTGGTCAGAAGCGTGTCAAGTTTGTTGACTTGGAT
GAGGCTAAGGATAGCGACAAAGAAGTCCTCTTCAGGGCAAGAGTCCACAACACCAGACAACAAGGTGCAACATTG
GCCTTTTTAACTTTAAGGCAACAAGCTTCCTTGATCCAAGGTCTAGTAAAGGCCAACAAGGAAGGTACCATCAGC
AAAAACATGGTCAAATGGGCTGGTTCATTGAATTTGGAGTCCATTGTCCTTGTCAGAGGTATTGTCAAGAAGGTA
GATGAGCCAATCAAGTCTGCTACTGTGCAAAACCTGGAAATTCACATTACCAAGATTTATACCATTTCCGAGACT
CCAGAAGCATTGCCAATCCTTTTGGAAGATGCCTCCCGTTCCGAAGCTGAAGCTGAAGCTGCAGGTTTGCCCGTG
GTCAACTTGGACACCAGATTAGACTACCGTGTCATTGACTTGAGAACCGTCACCAACCAAGCTATTTTCAGGATT
CAAGCTGGTGTTTGTGAGTTGTTCAGAGAATATTTGGCCACAAAGAAATTTACCGAAGTACACACACCAAAACTG
TTGGGTGCACCAAGTGAAGGTGGTTCCAGTGTGTTTGAGGTGACATACTTCAAAGGGAAGGCCTACCTAGCTCAA
TCTCCACAATTTAACAAGCAACAATTGATTGTGGCCGACTTTGAAAGAGTTTACGAAATCGGGCCTGTGTTCAGG
GCTGAAAACTCCAACACCCACCGTCACATGACCGAGTTTACTGGTTTGGACATGGAAATGGCTTTCGAAGAACAT
TACCACGAAGTTTTGGACACGTTGAGTGAGTTGTTTGTGTTTATTTTCAGTGAATTGCCCAAGAGATTTGCTCAT
GAAATTGAGTTGGTACGTAAGCAATACCCTGTTGAAGAATTCAAGTTACCTAAAGATGGTAAGATGGTTCGTCTA
ACATACAAAGAAGGTATTGAAATGCTAAGAGCTGCCGGTAAGGAAATTGGTGATTTTGAAGACTTGAGTACCGAA
AATGAAAAGTTCTTGGGTAAGTTGGTTCGCGACAAATACGACACCGACTTTTACATCCTAGACAAGTTCCCCTTG
GAGATCCGTCCCTTCTACACAATGCCCGACCCAGCCAACCCTAAGTATTCTAACTCGTATGATTTCTTCATGAGG
GGTGAAGAAATCTTGTCCGGTGCACAACGTATCCACGACCATGCTCTATTACAAGAAAGGATGAAAGCCCATGGT
TTGTCTCCTGAGGACCCAGGTCTAAAGGACTACTGTGACGGCTTCAGCTATGGGTGTCCTCCACACGCCGGTGGT
GGTATCGGTTTGGAAAGAGTTGTTATGTTCTATTTGGATTTGAAAAATATCAGAAGAGCTTCATTGTTCCCAAGA
GATCCAAAGAGATTAAGACCATGA

>YBL033C 1.57 Hot
ATGACCATAGATAACTACGACAACAGTAAACAGGATAGCAGCAAATACGAGGTTAGTGGTACGGGTGATGGCAGG
AACGGCGATGGCGGCTTGCCTCTAGTACAATGTGTCGCAAGAGCTCGTATCCCAACCACACAGGGTCCGGATATC
TTTTTACATCTTTACAGTAACAACAGGGACAACAAGGAACATCTAGCCATTGTGTTTGGTGAAGACATACGGTCG
CGCTCGCTATTCCGTAGAAGACAGTGCGAGACGCAACAAGATAGAATGATCAGGGGCGCTTATATTGGCAAACTG
TATCCCGGCAGAACTGTGGCAGACGAAGACGATAGACTCGGATTAGCTCTGGAGTTTGATGATAGTACAGGTGAG
TTATTAGCTTCCAAAGCCACTACATGGGACGCCCATAACGACACGCTGGTACGGATCCATTCTGAATGTTACACC
GGTGAAAACGCATGGAGCGCCCGTTGTGATTGTGGTGAACAATTCGATAGGGCCGGTAGGCTTATCGCTTGCGAC

CACGAACCCACAAGCAACATCAAAGGTGGAAACGGCCATGGTGTTATCGTGTATCTAAGACAAGAGGGTCGTGGC
ATCGGGTTAGGTGAGAAACTCAAGGCCTACAACCTGCAAGACTTAGGTGCTGACACAGTGCAGGCCAACTTAATG
CTGAAACATCCCGTGGATGCTAGGGACTTCTCGCTGGGTAAGGCTATTTTGCTGGATCTTGGCATCGGTAATGTC
AGGCTGCTGACAAATAACCCGGAAAAGATAAAACAGGTAGATCATGCGCCTTACCTTAAGTGCGTTGAACGAGTG
CCTATGGTACCCATACACTGGACAAACTCCAGTGAAGGCATAGACTCCAAGGAGATAGAAGGTTATCTCAGGACC
AAGATAGAAAGAATGGGTCATTTACTAACGGAGCCTCTGAAACTTCATACAAACCCTCAACCTACTGAGACAAGT
GAAGCCCAAAACCAAAACCGTATGAACTCTGCGTTGTCATCAACATCGACGCTGGCAATATAA

>YJL043W 1.9Hot
ATGTTTGTTGATTATTCCGGACTGGAGCGTTACACGGATATAAATGCATCTTTTGGTAAGTTGGTCAACACTTAC
TGCTGTTTTCAGCGCTGTGAAGCCATCAGCGAACAATTGGAGATTCTGAAAAGTCTAGTACCAAAGTGCCACGAC
ATTGTGGCACTTACTGACGAAGACTTTGCTAGTGGCCGTACTGCCGGACTGACGCAGAAATTGTTTGCTATGGCG
ATGACCCTACACCAGATCACCGACTGTATCGACCTCCTGCAGAAATGCAACACCATAATACCCATCGAGATCGCC
AACCCGGCTAGCTTTGAAAGCGGCGCCGCGACGGCGCCGCTACGTCAAAGCTATGCTAGGCTCTTGGATGACTGG
TCCCACTACATGGGCCCATCAACGGTAAAACACACAGGCTGTACCAACCGACCCAAGTGGAGATTTCCCTGGCAA
CAGTCACGGACTATCATCATCCCCATGCTGTTTATTGGCGAAACTGCAATGAGCACCAGGGACCTAAGGTCTGTG
CTGCATGATTGTGAGATCAGGCACGCGAGCGAAATGCCGCTGCAGCTACTTTGGACGTCGTCACCGGAGTTGGTG
TACGCCACACCGCATGTTGACGACTACGACATTTGGTCGCGCTACGGGTCTGATTACAACATGCAGATAGAGGAC
GAAGACGAAGCATCAAAAGGGCGACAGCGCAAGTGTGTGGTGCAGTTGGAGGCACTGTTGGGTGCGCTACCTACT
ACGGACCCGTTGTTCCAATGGTAG

>YDL196W 1.67 Hot
ATGCCCTCCGAGTCAAGGAAAAGATTGTCTTCCAAAAAAGGTGCAACTGTGCGTTTAGAATTGGTGGAGAATTAT
GTGATATGCTTCTTCACCGTCCTCTGCTTCTGCCTAATACCACACTCATCAATTGACTGGAGATCTGGCCTATCT
TGCTACTACTTCATAGATTTTTTTTTTTTTCATCTATCCCCTTCCATCCCCTTCTGGTTCTACCCCTTCAGCGTG
AAAAATCACCATACACGATCAATCCGCCCGAGAACGAAAAGTGAAAAGAACAAACAAGTCGTGTCAGATCCATTC
CTCTATTCCTGTAGTCGGGTTGCATTCTGA

>YIL044C 1.61 Hot
ATGTCGACGTCAGTCCCAGTCAAGAAGGCATTAAGTGCTCTTTTACGCGATCCAGGAAACAGTCATTGTGCCGAC
TGTAAGGCGCAACTACATCCACGCTGGGCTTCCTGGTCACTTGGTGTTTTCATTTGCATTAAATGTGCTGGTATA
CATAGATCATTGGGAACGCACATTTCGAAAGTGAAATCTGTTGATCTGGATACATGGAAGGAAGAACATTTGGTG
AAACTGATACAGTTCAAAAACAATTTAAGAGCTAACTCATACTACGAAGCTACTTTGGCTGATGAATTGAAACAG
AGGAAAATCACTGATACGAGCAGTTTACAAAATTTCATCAAGAATAAATATGAATATAAGAAGTGGATTGGTGAC
CTCTCCAGTATTGAAGGGTTAAATGATTCTACTGAACCAGTTCTGCATAAGCCGTCTGCGAATCATTCGTTGCCT
GCTTCGAATGCAAGATTGGACCAGAGCTCAAATTCATTACAAAAGACGCAAACACAACCACCTTCCCACTTATTA
AGCACCTCAAGGAGTAATACGAGCTTATTAAACCTACAGGTTTCATCGCTTTCTAAGACTACATCTAATACAAGT
GTAACTAGTTCTGCAACAAGCATAGGTGCTGCTAATACCAAGACTGGCAACAGAGTCGGTGAATTTGGGCAAAGG
AACGATTTGAAAAAATCCATTTTATCTTTATACTCAAAACCTTCTGCCCAGACTCAAAGCCAGAACTCATTTTTC
ACTTCCACGACACCTCAGCCTTGTAATACTCCGTCACCATTCGTGAACACTGGAATTACCGCTACAAATAATAAT
AGTATGAATTCAAATTCCAGTTCTAACATTTCATTGGATGATAACGAGTTATTTAAGAACGTCTGGAGTTGA

>YDR475C 1.92 Hot
ATGGTGGTACGAGATCAAGATGAGGCGTTGAGAAATTCTTATAAATATGTTAAGCTGTATGTCAGGCAGGACCAG
CTGGAGGATACGGTGGACATACTCGCTAAGCAGGACGAGGACAAGTCCAACAATGATGACAGGCGCAGTCTAGCC
TCGATATTGGACTCGAGCAGTTCCGTCAAAAAGAAGGGGAAAGGCAGCAACGAAAAGTACCTACCATGCGTATCT
TTCAACACAGTTCCCAGATCAAGGGTATCTTCGCCCTTAGATGAAGAGAAAAGAGAATTTCCTGGCGTGCAGATC
TCTGCAGATTATACAATGGAGGAATATTATGACGACGAAAGTGGCTTTACCTCGGACAACAATGCCGACTACTTC
TCCGGGAATAGCTATTCGAGTAGGCGAGAGGGCAGTGCTAGTCCCGGCAGGTATTCTTCTCCACCTCCTGCCTCT
AAAAGAAACATCAAAATAGGTAAGATGTTTAAGATTTCTGAAAATGGTAAAATTGTAAGGGAAGACTATCCTACT
ACACCCACCGATATCAATGACGCTCTTGTGATTAGTAGGGCGTATGCCAATTGGAGACAACTATGGATCAAGAAG
AAAAATCAGATAGATCATCGATTAGAACAAAAGCGCGATTTCTTCAATTACCCAACAATACTGTTTCCTCCAAAC
AAAAAAAAGTCGTCTGAGGGCGCTACTCCCACAATAAAATTCAATCCTCCCATAGAAGACGGCTTTACGCCTTTG
ACTAAGTCTCAAAAGCGGAAAGAGCGTGTTTTGAGCGAAAAGGTTGGGTTCCCGAACACTCCTCGAACGATATTG
TGCCACATCAGCGGCAGGAAACATACTTGGGTAGCACTAGATTGGGCGCTACGAACTTTGATTCAAAATACAGAC
CACATTGTTGTGTTGGCAAACCTGCCCCGCCTGACTAAGAATAATTTCGAAGACAATGATTCGATGAGTGAGCGA
AAAAGAATGCTAATGATGATGGATGATAGCAGGAGTGTTAGCTCAGCAAGAAGATCACGATCCAGATCGAGATCA
CGATCCATATGTACAAGAAGGGCACTTTCTCTGGGGCCTGAGGAATCTGACAACAAATTAAAGCACCAAAATTTT
ATCGAGTGGACATCTGGTTATACGCAGAACGAAATAGAACGTAAATTGCAAGATCTTTTTGATTATGTTACACTC
ATAATTCCGCAAGATCGTTCTGTCAAGGTCACTGTCGAGATCCTAATTGGCAAGACTAAAAAAACGCTTCTTGAG
GCAATAAACATCTATTTACCTGATTTCTTTGTATCTAGCACTTTGAGGTGGGAGAGAACAGACAGCTTAGTTCGT
TGGAAATCGAACTTTCTGACAGATAAACTGTGTACTAATTTTCCGATTCCAACGTTTATTGTACCGGCAAAGAGA
ATGTTCGATTTGGAAATAGATTTGCAAAAAGAGTTTAAAGAACCGGAGGTAACGAAACAAAAAAATACTAGCGGT
CCAAAGCCTGGGTTCAGCCATTCTAAATCAGCAGATGCTAGCATTCCAACTATCTCAAATATCAAACGGAAGCAA
GATAACGATTATTCTATAGATTCACTATGTTATGCCCCAGAAGCTAATGGTGCTAACAATAGTAGCCGAGAGGAA
GCATCTGATGATGAGCTTAACGCTTTTAAGGATGATGAGAATGATGTGATGTCCGTGAAATCCTTAACCTCTAAC
ATTTCGGTAAAGGAAAAGTTATGTACCATGGCAAGGAAACGTAGAAAGAGTATGGCTCAGCAGTTAAATGACGCC

GATCATGATTCATCAATACCACCGGGACAGAGGCATTTGAAAAAACTGAATATTATTTTGGAATCTTCATTAAAG
TTTTCATTAGAAATCGATAGCATCACTGATAGTATTGAAAATGGGGATGTAGACGAAAAGAGAGCACACTCTATG
GAGTCGGGCTTTGAAGAACTGAAAAGAGTCATCACAGGTGGTGCTCCTCCAAGGCATGTGGCTACCCCGCAGAGG
TCCATGCTTGATGTTTTGGACAATCCAAGTTCATCTAGATCCAAATCCAAGTCAAGATCAAGCTCTAAGTCAAGG
ATAAGAGACAAATCAAAACCAAGCTCTCCTACTGCTACCGATATAAACTCAAGTGCGAGTGCGTCGAGATCACGT
TCTCCGCAAATCAAGTTCGCGTCGAGTGTCAAGAATGTAGATGGTAATGCTGCACTGGGTGCTATTAAATCTAGG
CACTCGCTAGACAGCCCAGGGGACCAGCAACAACAACATCATCATCACCATCATCGTGACACAGATCAGTTGTCC
GTTCCAGGCCTTCCCCATTTGGCACCTTCAAAGTCATATAGTGTGAGTTCTGGTAATAAGGATTCATCTTTGAGG
AAAGTCAGTAGCTCATCTTCGCTCAGAAAAGTAAAATCTAATGATTCGAACTCTGGTAAACGTATTAAAAAACCG
GTCGTAACTTCAGCACACTTGAAACCAAGCTCAGGCGGTGGTGGTTTATTCTCTTTTTTTAAGAGTAAATCGAGA
TCCCCGTCATCTTTCAGAAAGGAAGATGAGAGTAAAAATACGCCTAAGCGTGGCGGATTGTTTGGGTTCGGAAGG
CTGTAG

>YPL170W 1.88   Hot
ATGTCCTTCATTAAAAACTTGTTATTTGGAGGTGTTAAAACAAGTGAGGATCCAACCGGGCTCACAGGTAACGGG
GCCTCAAACACAAACGATTCTAATAAAGGTAGTGAACCGGTAGTAGCGGGTAATTTCTTTCCTAGGACGCTTTCC
AAATTTAACGGCCACGACGATGAAAAAATATTTATTGCTATTAGGGGCAAAGTATACGACTGCACAAGAGGGAGG
CAGTTTTACGGTCCAAGCGGGCCATACACTAACTTTGCAGGCCATGATGCGTCGCGTGGTCTTGCATTGAACTCC
TTCGATCTGGACGTTATTAAAGATTGGGATCAGCCTATCGATCCCTTAGATGATCTGACAAAAGAACAGATTGAC
GCACTGGATGAGTGGCAAGAGCATTTTGAGAATAAGTACCCATGCATTGGTACTCTGATTCCGGAGCCTGGCGTG
AACGTATGA

>YDL170W 1.67   Hot
ATGAATTATGGCGTGGAGAAGCTGAAATTGAAATATTCGAAGCATGGGTGTATTACTTGCAAGATCAGAAAGAAG
AGATGCTCTGAAGATAAACCTGTGTGCCGGGACTGTCGTCGATTAAGTTTTCCCTGTATCTATATATCAGAATCT
GTTGATAAGCAGTCTTTGAAAAAAATAAAGGCAGATATACAGCACCAGTTGATCAGCAAGAAAAGAAAGCATGCT
CCTGACAGTGCTCAGAAAGCGGCCGTGGCCACCCGTACTCGACGGGTCGGTAGCGACGAACAGGATAACCAGGTA
TATTTGTCAAAACCGCTGGAAGATTGCATTTCTCAGAAGCTGGACTCTATGGGATTGCAGCTGTACAACTACTAC
AGGTCTCACCTTGCAAACATAATTTCCATCGCGCCCATGAACCAGAACTACTATCTGAACATTTTCTTGCCAATG
GCCCACGAGAATGATGGTATATTGTTTGCTATTCTTGCTTGGTCAGCTAATCATTTGTCTATATCATCATCAAAT
GAGCTGCGAAAGGATGAAATATTTGTCAACTTGGCGAACAAGTATACATACATGTCGTTATCGCATCTAAAGACA
AACGAGGGCTCAAGTGCCTGTGCTAAACTGGGGTTTCTATATTCACTAGCACAAATCCTAATCTTATGTGGTTCA
GAGATATGTCAGGGTGATGTGAAATTTTGGAAGATATTATTGAATATAGGAAAAAATTTGATCGAAAACCATGTG
GGCAAGGATGTCTCACGGATACTAACCACTACTACAGAAGAACCCTCCCTAGAGGAGAGGATAATCTTCCCCAAT
TTCAATTCCGTTGTTAAGTACTGGCTGATTGTGAATTTTATATATCATGATATTTTGAATTTCAATACAACGTCC
TTCCCGATCGAACAATACGAGAAATTCTTCCAAAGGGATCAGAACTCTTTACCCAGCTCAGCCAACTTTATTGAG
TCAATAGACTCGCCAATTGAGGAAATAGACCCTTTAATTGGCATCAACAAGCCTATTTTACTACTATTGGGACAA
GTCACAAATTTGACGAGGTTTTTGCAGACTATGGAACAGGAAGAAATGCTAGAGCATGGCGATAAAATTTTGAGT
TTGCAAGTCGAGATTTATAAATTACAACCTTCGCTTATGGCGCTGGAACATTTGGATGATGAAAAGAAATTTTAC
TATTTAGAACTATTCGAGATAATGAAAATTTCTACCTTAATGTTCTTTCAATTAACTTTATTAAAGATTGACAAA
GATTCGCTGGAACTACAGATACTGAGAAACAAATTGGACTCGAAGCTAGACAAGGTCATCGGAACTTTTTTGGAA
GGATCGTTATGCTTCCCACTTTTCATTTACGGTGTATGCATTCAGGTAGAAGATATGGAGAAAAAAATAGATTTG
GAAGCCAAATTTGACGACATTTTAAAACGATATAAATGTTACAATTTCCAAAATGCTAGATTATTGATACGGAAA
ATTTGGCAAAACGAGGCTGATGGCATCAGTGAGCATGACCTGGTCCACATGATTGACGAATTAGATTACAATATT
AATTTTGCCTGA

>YIL121W 1.88   Hot
ATGGCAGGAGCAACATCAAGTATAATTCGGGAAAATGATTTTGAGGACGAGCTTGCGGAGAGTATGCAGTCATAT
AATAGAGAAACTGCAGACAAATTGGCTTTGACTAGGACCGAGAGTGTAAAGCCAGAACCGGAGATAACCGCTCCG
CCTCACTCACGCTTTTCCCGTTCTTTCAAGACAGTTTTAATAGCTCAGTGCGCTTTCACTGGGTTTTTCTCCACA
ATAGCAGGTGCCATCTACTATCCAGTTCTGAGCGTTATAGAAAGAAAATTCGATATTGACGAGGAATTGGTGAAT
GTCACTGTTGTAGTATATTTTGTATTTCAGGGTCTTGCCCCCACATTCATGGGCGGGTTTGCCGATTCACTGGGC
AGGAGACCGGTGGTGCTTGTCGCAATCGTCATTTATTTTGGTGCCTGCATCGGTCTTGCTTGTGCTCAAACGTAT
GCTCAGATCATTGTGCTAAGATGTCTACAAGCCGCCGGTATTTCACCCGTGATTGCGATTAACAGCGGAATAATG
GGAGATGTTACTACTAGAGCCGAGCGCGGCGGGTACGTTGGATATGTTGCTGGATTTCAAGTTCTAGGTTCTGCG
TTCGGAGCCCTTATCGGTGCCGGATTATCATCTAGATGGGGATGGAGAGCCATCTTTTGGTTCTTAGCAATTGGA
TCTGGCATTTGCTTCCTAGCCTCGTTTTTAATTTTGCCAGAAACAAAGAGGAACATATCCGGGAATGGTTCTGTC
ACCCCAAAATCATACTTAAATAGAGCTCCCATTCTTGTTTTGCCAACAGTAAGAAAATCATTGCATTTGGATAAT
CCAGATTACGAAACATTGGAACTGCCCACGCAACTAAATCTACTGGCGCCCTTCAAGATTTTGAAAGCTTATGAA
ATTTGTATACTTATGCTAGTCGCTGGATTACAATTTGCCATGTATACTACGCACCTTACGGCGTTATCCACAGCT
TTGAGTAAACAATATCACTTGACTGTTGCAAAGGTAGGATTATGCTACCTTCCCTCCGGTATCTGCACATTATGT
AGTATTGTCATTGCTGGAAGATATTTGAATTGGAATTACAGGCGCAGATTAAAATATTACCAAAATTGGTTGGGC
AAGAAGAGATCAAAGCTTTTAGAAGAACACGACAACGACCTTAATTTGGTGCAACGTATCATAGAAAATGATCCC
AAATACACCTTTAATATCTTCAAGGCGAGGTTGCAACCCGCATTTGTTACCTTGCTTTTAAGCAGTAGCGGATTT
TGTGCGTACGGATGGTGCATTACTGTCAAAGCCCCTTTAGCGGCCGTTCTTTGCATGAGTGGATTTGCGTCGCTG
TTTTCCAATTGCATTTTGACTTTTTCAACAACTCTAATAGTTGATCTTTTTCCCACGAAGACATCTACGGCTACG
GGATGTTTAAACCTTTTCAGATGCATTCTATCTGCTGTGTTTATAGCTGCATTGAGTAAAATGGTAGAAAAAATG

AAATTCGGAGGTGTTTTTACATTTTTGGGGGCTTTAACCTCCTCGTCATCGATCCTCTTATTTATTCTCTTGAGA
AAAGGTAAAGAATTAGCCTTTAAGAGGAAAAAGCAAGAACTGGGAGTAAATTAA

>YOR224C 1.64 Hot
ATGTCTAACACTCTATTTGATGATATATTCCAAGTCTCGGAAGTTGATCCCGGTCGTTATAACAAAGTCTGCCGT
ATCGAAGCCGCATCTACCACTCAGGACCAATGCAAACTAACCTTGGATATAAATGTTGAATTGTTTCCCGTCGCC
GCACAAGATTCTTTGACAGTGACTATTGCATCCTCTTTAAACCTCGAAGACACCCCAGCTAACGACTCTTCTGCG
ACAAGAAGCTGGAGACCTCCACAGGCTGGTGACAGATCCCTTGCAGATGATTATGATTACGTCATGTATGGTACC
GCTTACAAGTTTGAGGAAGTAAGCAAGGATCTAATTGCCGTTTACTACTCATTCGGTGGCCTCTTAATGAGATTA
GAAGGTAACTATAGAAATTTGAATAACTTGAAGCAAGAGAACGCTTATCTTTTGATTCGTCGTTAG

>YIL042C 2.23 Hot
ATGTGGAAGATTATGCGTTCATGGAAATGTGGGGGAATGCGCTGGGCACATCGCCAGCGACCCTCACACGAGCTG
CTGTCGCAACTGTCGTTCGACCAGCATTACAAGATTAGGTCGAACATTGAGCTGCTGATACAGGACTACGCTAGT
AAACCAATTGCGCCCCTAAACTACGAGTATTTCCTGCAATACAGGCCGCCGCTGACCAAGAAGGAGGAGTACATG
CTGACGATCAAGACAATCAACCTGTTGCTTTCATTGACGTGTAAACGGCTTAATGCCATTCAGAGGCTACCGTAT
AATGCGGTAATCAACCCTCATATTGAGAGAACCAACTCTTTGTATTTGAAAAGCTTGCAAACGTTGCTATCAATC
GCATACCCGTACGAGTTGCACAATCCTCCCAAGATACAGGCCAAGTTCACAGAACTGCTGGACGACCACGAGGAC
GCCATTGTGGTATTGGCAAAAGGTCTTCAGGAAATCCAATCGTGTTACCCAAAGTTTCAAATTTCGCAGTTCTTG
AACTTCCACCTGAAGGAGAGAATCACGATGAAGCTGCTTGTGACGCACTATTTGTCGTTGATGGCACAGAACAAA
GGTGACACGAACAAAAGGATGATTGGAATCTTGCACCGGGACCTCCCCATAGCACAATTGATCAAGCACGTTTCT
GATTATGTCAATGATATATGCTTTGTGAAGTTCAACACTCAGCGGACCCCGGTCCTAATTCACCCGCCATCACAA
GACATCACATTCACGTGCATTCCGCCCATTCTGGAGTATATTATGACAGAGGTATTCAAGAACGCTTTTGAAGCT
CAGATAGCTCTTGGAAAGGAACATATGCCCATAGAGATCAATCTTTTAAAGCCAGACGATGACGAGCTGTACTTA
CGGATACGGGACCATGGTGGTGGGATCACGCCCGAGGTAGAGGCCCTCATGTTCAACTACTCTTACTCTACACAT
ACCCAACAATCAGCTGACTCTGAGTCTACAGATCTGCCCGGCGAGCAAATCAACAACGTCTCCGGCATGGGCTTT
GGTCTACCAATGTGCAAGACGTACCTAGAACTGTTTGGGGGCAAGATTGACGTTCAAAGTTTACTTGGCTGGGGC
ACGGACGTGTACATCAAGCTCAAAGGCCCTTCTAAGACTGCACTACTCTCCAAAAAGTAA

>YFR032C 1.58 Hot
ATGACAGAACAAGTTAACAATGACACTACCAGTGACACTACCACTACCATTACCACAGTTTACATTTCGAACCTG
CCATTCACGGCGAGTGAGCGAGACCTACATGCCTTCCTAAACAACTATGGCGCCAGTTCAGTGTTGATTCCAACG
CAGACCGTCCGCAGATTCAGCAAAAGGCACAATAGCAACCCTCGCAAACCACTAGGCATAGCATTTGCCCAGTTC
GCAAACAATACTCTGGCTTTGAAGGCCATTCAAGACTTGAACGGAACCGTTTTCCAAAATCAGAAACTATTTCTA
AAGTTACACGTTCCTTACGAGGCAGACTCTACTCCAGACACGGATGTAAAGAAGCCCAAAGAAAAAAATAAGGTT
AAGAAGACACCAGAGACTGCCGCGGATACGGTTTACTGTCACGACCTGCCAGACGATATAACCGACAGTGAGATC
CGTGAGTTGTTCCAGCTCTACTCGCCTCAAGAAATCTGGATTTACAGGTCCAAGGTGTACAGGAGAAAGTGTATT
CCTTTTGCTCCACACCAAATTACTGCCGCTTTGGTCACTTTGCAATCTGAAACCCCAATTGGGGACATTTGTGAC
AGTGTGGCCAAGACAGCTACTTTGAGGGGCAAATCGATAATAGTGAAGCCCGCTTACGTCTCTAAGATTCAAGAA
ATTAAACAATTGGTCAAGGACAACTTAACCAATGCGCGCGACCCACCTCCAGCAGCATTGGCAGAGCCAGCGCCA
GCGCCAGCGCCAGTAGAGCCAGCGGAGCAAGTGCAGGAAGGGCAAGACAATGCGGAAACCAATGATGTCCCACCT
CCGCCTGCTTCGTCTTCCGATCGTCCAACCGTGGCTGCTACTTAG

>YOL126C 1.79 Hot
ATGCCTCACTCAGTTACACCATCCATAGAACAAGATTCGTTAAAAATTGCCATTTTAGGTGCTGCCGGTGGTATC
GGGCAGTCGTTATCGCTGCTTTTGAAAGCTCAGTTGCAATACCAGTTAAAGGAGAGCAACCGGAGCGTTACCCAC
ATTCATCTGGCTCTTTACGATGTCAACCAAGAAGCCATCAACGGTGTTACCGCCGACTTGTCTCATATAGACACC
CCCATTTCCGTGTCGAGCCACTCTCCTGCAGGTGGCATTGAGAACTGTTTGCATAACGCTTCTATTGTTGTCATT
CCTGCAGGTGTTCCAAGAAAACCTGGCATGACTCGTGATGACTTATTTAACGTGAATGCTGGTATCATTAGCCAG
CTCGGTGATTCTATTGCAGAATGTTGTGATCTTTCCAAGGTCTTCGTTCTTGTCATTTCCAACCCTGTTAATTCT
TTAGTCCCAGTGATGGTTTCTAACATTCTTAAGAACCATCCTCAGTCTAGAAATTCCGGCATTGAAAGAAGGATC
ATGGGTGTCACCAAGCTCGACATTGTCAGAGCGTCCACTTTTCTACGTGAGATAAACATTGAGTCAGGGCTAACT
CCTCGTGTTAACTCCATGCCTGACGTCCCTGTAATTGGCGGGCATTCTGGCGAGACTATTATTCCGTTGTTTTCA
CAGTCAAACTTCCTATCGAGATTAAATGAGGATCAATTGAAATATTTAATACATAGAGTCCAATACGGTGGTGAT
GAAGTGGTCAAGGCCAAGAACGGTAAAGGTAGTGCTACCTTATCGATGGCCCATGCCGGTTATAAGTGTGTTGTC
CAATTTGTTTCTTTGTTATTGGGTAACATTGAGCAGATCCATGGAACCTACTATGTGCCATTAAAAGATGCGAAC
AACTTCCCCATTGCTCCTGGGGCAGATCAATTATTGCCTCTGGTGGACGGTGCAGACTACTTTGCCATACCATTA
ACTATTACTACAAAGGGTGTTTCCTATGTGGATTATGACATCGTTAATAGGATGAACGACATGGAACGCAACCAA
ATGTTGCCAATTTGCGTCTCCCAGTTAAAGAAAAATATCGATAAGGGCTTGGAATTCGTTGCATCGAGATCTGCA
TCATCTTAA

>YBR231C 1.96 Hot
ATGCCAGAAGTAGAGACAAAGATTATACCAAACGAAAAGGAGGACGAGGACGAGGACGGCTACATAGAAGAGGAG
GACGAGGACTTCCAGCCTGAAAAAGATAAATTAGGTGGTGGTAGCGACGATAGTGATGCCAGCGATGGCGGTGAC
GACTATGATGACGGTGTGAACAGAGATAAGGGTAGAAATAAAGTGGACTATTCGCGTATAGAGAGCGAGAGCGGC
GGACTGATCAAGACTAGAAGAGCTCGGCAAGCTGAGGAAGAATATGCGAAAACACATAAGTATGAATCGTTAACT
GTTGAATCGATCCCTGCAAAAGTAAACAGCATCTGGGAGGAGCTACAGGAAGCTAGTAAGAACCGTCTTTTGAGC

AGTTCAGGGAAAGTCGGCTCTGTTCTCGACGGTTCCAAGGAGGCTAGGTCGACAACGGCCGCGCAACAGGAGGAC
AAAATCCTCATCGAAAGAAACTACAAGTTTGCTGGTGAAACTGTCCATGAGAAAAAATGGGTTTCACGCAGCAGT
GCGGAGGGTCAAGAATATCTAAACAGTTTGAAATTTAAGCAGCAGGCGCCTGCTGCTCCTGTTCAACTGGAAAAA
GCAGTTAGGACCAAGTCCAATGAAAGCCGGCAACACTTGCGACGGCCACTGAAAAGGCCTCCATTGTTGGAGCAG
ATCATTTCTGGCGGGCTACGACCCAAGCTAACGACATTAGAAAAATCTCAGCTGGACTGGGCTAGTTATGTAGAC
CGTGCTGGACTTAACGACGAATTGGTGCTGCACAACAAGGATGGGTTCTTGGCCCGGCAAGAATTTCTGCAGCGG
GTCGGGTCTGCAGAGGACGAGAGATACAAAGAATTGCGCCGGCAACAACTTGCTCAGCAGTTGCAGCAGGATAGC
GAAGCTTCATAG

>YGL199C 1.92 Hot
ATGGAGATGTACGAAATCAGCGACACCCTCTGGTGTGGCTTGGTGAGTCTCAGATGCAATGCCATCACCAGCAAA
ATGAACCAACTAGACACAAATAGGCTCGAGAACAGCGACCGCGCATGCGATACAAATAGAGAATACAATATGATA
AACGCAAGGGGCGCCCACAGGTCGCAATTGGCTGAAATGTCGTTATCAGCCGCCCCTATCCCGTCATCAGAGGGG
CTAAAGAATGAGGGGAAATGCGGGTATACCACTTGTTTCAGTCTGGAATTAATCTCCACCACATCTCGCTTTAGT
GTTTGCAGCACAGTCTCGTCGAGCGTCCCTCTGCTGCCTGAATTCTGCATAGTTCCGCCTATGTTGTCAGCAACA
CCCCCGGAAGCAGCCAAAGGTGCATCTGGTTCTATGAAGTCGGGCTCAATCGTAGTGTCTTCTCTTCCGTATGAC
ATTTTGCCTGCCCTCTTGTGA

>YDL139C 1.52 Hot
ATGAAAACCAATAAGAAAATTTCTAAAAGAAGAAGCCTGAAAAATCTCCATGGTGCCTTAAAAGGACTATTGAAA
GAGTCCGGCAAGAAGAGTGAGTCCAAAATACGAAAACATAGCGATTGCAATCCTGTTCATCGAGTATATCCGCCA
AATATCGAAAAACGGAAGACCAAAAAGGACGATGGAATTTCACGGCCAATTGCTGAAAGAAATGGTCATGTGTAT
ATAATGTCCAAGGAAAACCACATCATCCCTAAACTAACCGATGATGAAGTCATGGAGCGCCACAAACTAGCAGAC
GAAAACATGAGAAAAGTGTGGTCCAACATCATAAGCAAATACGAGTCTATTGAAGAACAAGGAGACCTCGTAGAC
CTGAAAACTGGTGAAATAGTTGAAGATAATGGGCATATCAAGACGCTGACGGCCAACAACAGCACGAAAGATAAA
AGAACAAAATACACAAGCGTGCTCAGAGACATTATCGACATCAGCGACGAGGAAGATGGTGATAAAAATGATGAA
TATACTCTTTGGGCTAACGATAGCGAGGCAAGCGATTCAGAGGTGGATGCTGACAACGACACTGAAGAGGAAAAA
GACGAGAAATTGATAGACGCAGATTTCAAGAAGTACGAGGCCAAACTCTCGAAAAGGATATTACGAGATTAA

>YKR048C 2.18 Hot
ATGTCAGACCCTATCAGAACGAAACCCAAGTCGTCGATGCAAATCGACAACGCCCCTACGCCTCACAATACCCCA
GCATCGGTTCTGAATCCGAGCTATTTGAAAAACGGCAACCCAGTTAGGGCCCAGGCACAGGAGCAGGATGACAAG
ATCGGCACCATCAACGAGGAGGACATCTTGGCTAACCAACCCCTGTTGTTACAGTCCATCCAGGACAGACTTGGC
TCGCTGGTGGGCCAGGACAGCGGGTATGTGGGGGGTCTTCCCAAGAACGTTAAGGAAAAGCTGCTGAGCTTGAAG
ACTTTACAAAGCGAGCTATTCGAAGTTGAAAAGGAATTTCAGGTGGAGATGTTTGAGCTAGAGAACAAGTTTCTG
CAGAAGTACAAGCCCATTTGGGAGCAGCGGTCCAGGATCATTTCAGGACAAGAGCAACCCAAGCCGGAACAGATC
GCAAAGGGCCAAGAGATTGTGGAGTCACTCAATGAGACGGAGTTGTTGGTGGACGAAGAAGAGAAGGCCCAAAAT
GATTCCGAGGAGGAACAGGTGAAGGGAATTCCCTCATTCTGGCTAACCGCATTAGAGAACTTGCCCATCGTTTGC
GACACGATCACTGACCGCGATGCGGAAGTTCTGGAGTACCTGCAAGATATTGGTCTGGAATACTTGACGGATGGT
AGACCCGGTTTCAAGCTGTTGTTCAGATTTGATTCTTCCGCCAACCCATTCTTCACCAACGACATTCTGTGCAAG
ACCTACTTCTACCAGAAGGAGCTCGGTTACTCCGGGGACTTTATCTACGACCACGCAGAAGGCTGCGAGATCAGT
TGGAAGGACAACGCCCACAACGTCACTGTGGACCTAGAAATGCGCAAGCAGAGAAACAAGACCACCAAGCAGGTG
AGGACCATCGAGAAGATCACTCCCATAGAATCCTTTTTCAACTTCTTCGACCCTCCCAAGATCCAAAACGAAGAC
CAAGACGAGGAGTTGGAGGAAGACCTCGAGGAACGTCTAGCTTTAGACTACTCCATCGGGGAACAACTTAAGGAC
AAGCTAATTCCTAGAGCCGTGGACTGGTTTACCGGCGCAGCCTTGGAATTCGAGTTCGAGGAGGACGAAGAAGAA
GCGGACGAGGACGAAGACGAAGAAGAAGATGATGATCACGGCTTGGAGGATGACGACGGGGAATCTGCCGAAGAG
CAAGACGATTTTGCTGGCAGGCCGGAACAGGCTCCTGAATGCAAGCAGTCATAA

>YDR275W 1.64 Hot
ATGTTTTTTTTCCCGAAGCTTAGAAAACTTATAGGTTCAACTGTGATCGATCACGATACCAAGAACTCATCAGGA
AAGGAAGAGATCATGTCAAATAGCCGATTAGCACTTGTTATTATCAACCACGCCTTTGATAAGGTACTATCTTTG
ACGTGGCACTGCGGGATATTATCCGAAATAAGATCAGGACTGATGTTAATGTTTGGCATTTTCCAGTTGATGTGC
TCTTTGGGTGTCATCGTATTGCTGTTGCCTATTATCATACTGGACGCGATCGATCTATTCCTCTACATGTGCAGG
TTGCTTGATTACGGTTGCAAGTTATTCCACTACAATAGATCATCATTACCGGTGGCAGATGGAAAAGAAAAGACC
AGTGGTCCCATAAGCGGAAAGGAGGAAATAGTTATTGATGAGGAGATAATTAATATGCTAAATGAATCCTCAGAA
TCATTGATCAATCATACTACAGCTGGTCTGGAATATGATATTAGTTCAGGTAGTGTTAACAAAAGTAGGCGTTTG
AATTCAACTAGTACAGTTACATTTGTGAAGCAGAATAAACTTGTTAATGAAAGGAGGGAAGACGCCTACTACGAG
GAAGAGGATGATGATTTCCTATCAAATCCGAATTATGATAAGATATCATTGATCGAAAAATCATTCACGAGTCGT
TTTGAAGTGGCTTGTGAGCAGAAAGCTGCTTAA

>YMR264W 2.08 Hot
ATGGAGGATTCGAGATTGCTTATCACTTTGATTCTTGTGTTTGGAGTTATATTTCTGAAAAAATTCTTCCAAAGT
AATCAGCATCCCTCAGCACAACGCTTATCCGCTACAGGTGTAAACGCACACGGACGTCCTCAGGGCTCCACGCAG
AATGCCTTGAGAAGGACTGGTAGAGTCAATGGAGGTCACCCCGTGACTACTCAGATGGTGGAAACAGTGCAAAAT
CTAGCCCCTAACTTACATCCTGAGCAAATTAGGTATAGTTTGGAAAACACAGGCTCAGTCGAGGAAACAGTGGAA
AGGTACTTGCGTGGTGATGAATTCAGCTTTCCACCTGGGTTTGAGCCCTCCAGGGCGCCAATGGGGGCCAATGCG
GCTGTTGATAATAACGCTGCTGGTGGCGGAGAGTTTAACGATCCCAGAAAGAAGAACATGATTTGCGCTGAGAAT

CTACTCGATAAATTCCATGTGGATCTCAATGAAGATATGAGTAACTTAAGCTTTAAGGACTTAGACATTGAAGAG
AGAAAGAGATTGTTGGTTTGGCAAGCCAGAAAGAATTTGGAGACAAAATTGCAAAGTGATAAAGATTTGCAAAGT
TTGCTGACTTGA

>YJL063C 2.08 Hot
ATGACAGTGGGTATTGCTAGGAAACTCTCTAGGGACAAGGCGCATAGGGATGCGCTGCTAAAAAACCTTGCGTGC
CAGTTGTTTCAGCATGAATCTATAGTGTCAACGCATGCCAAATGTAAAGAGGCTTCCAGGGTCGCAGAGCGGATC
ATTACCTGGACAAAGAGGGCAATAACGACAAGTAATAGTGTTGCACAGGCGGAATTAAAGTCCCAAATTCAGAGC
CAATTGTTTTTAGCAGGAGACAACAGAAAGTTGATGAAGCGGTTGTTCAGCGAGATCGCACCGCGGTACTTGGAA
AGGCCCGGTGGGTACACGCGTGTGCTGCGGTTAGAGCCTAGGGCTAACGACTCAGCGCCGCAATCCGTCTTGGAA
CTAGTCGACTCTCCCGTGATGTCCGAATCGCATACGGTGAACAGGGGCAATTTAAAGATGTGGCTGTTAGTCAAG
TCAGTCATCAACGATGACGCCAACCAGCTACCTCATAACCCACTCACATTGCAGAATCTTCACAAAGTGGCCAAG
TTTAAAGCAGAAGCGCAGCTACATGGTGAAATTATGCTCATCAAGCAGGTCTTACTCAAGGAAATGTCCCTTCCC
TATGATGAGGCACTCGAAAACGAAAGGACGCAGGCACTTTTGAAGGAGGTCTACTCTTCGTCATTGCCAAAGAAG
ACGAAGAAACCCTCCTCCTATGTCATGGTTCCCAGACCTTAA

>YGR059W 2.27 Hot
ATGAAGTCAAAAGGGAGTCGGTTGTCAACAGACTGTCCTGTCGAATTTCCCAAGATAGTCTCTGGATTCGCTGAG
GAAGTGAAAATACGTAGACAAAGTTCCCAAGGACAGTACGCCGTCGATTCACATCCTCCGAAAAGCCCTGAACTG
AAACACAGAAGACAGAGGTCATCCTCTTTTGTTAACGGTAAATGCAGGAACAGGGACCTTCCATTGTTAGATAAC
AAGAAAGCACAAGAAATAAACACCAATTCACACGGCCAAGACATCGGTATCAAAAACTTACCACGTCAACGTGAG
TTGTTGAATGCCAAAAATGGAATTGATTTTACGCTGATGGTGGCGGGTCAAAGTGGATTGGGTAAAACCACGTTT
ATCAATTCCTTATTTTCTACTTCTTTAATTGATGATGACATCAAAGAAAACAAACCTATTATTCGTTATAAAAGC
ATTGTAGAAGGAGATGGAACACACCTTAATTTCAACGTCATCGATACACCTGGTTTTGGTAACAATATGGATAAT
GCATTTACGTGGAGGACAATGGTTAACTATATTGATGAAGAAATAAGATCGTACATTTTCCAAGAAGAACAACCT
GATAGGACAAAAATGGTTGATAATAGAGTCCATTGTTGTTTGTACTTTCTGAGACCTTCAAATAAGGGAATTGAT
ACTTTAGACGTCGTAACAATGAAAAAATTAGCGAAGAGAGTGAATTTAATCCCGGTTATTGCTAAATCAGATTTG
CTAACGAAAGAGGAATTGAAAAACTTCAAAACACAAGTTAGAGAAATAATAAGAGTACAAGATATCCCTGTATGT
TTCTTTTTCGGCGATGAAGTTTTGAATGCAACACAAGATATTTTTCAAAAATATCCATTCAGTATAATTGCATCT
AATGAGTACATTTTTAATGAAAAGGGCGAAAAAGTTAAAGGAAGACAATACAAATGGGGCGCTGTTGACATTGAA
AATGAAAAGTACTGTGACTTCAAAATCTTGCAAAAGACGATTTTTGATTGGAATTTAATTGATCTTGTAGAAAGT
ACCGAGGATTATTATGAAAAATGCAGATCTGAAATGCTAAGAACTAGGCTATTAAAGGCCAGAGATTGCTTAACA
ACGAAAAGTGTTGACATAACGGAAGAACAAAGGAAATTTTTGGAGGAAGAAATGAACTTCGATGAAATCGAGGAA
AACAAACTCAAAAATTACAAGTGCTATGAAATAATTAATAAAACGGTCATGGATAAGGTGGCTACAGAATGGGAT
CCTGAATTTATAACTAGACAATTAGAAGCTAAGAAAAAATTCAACGAGCTGTCCAACAGAGAAATTTCAAAATTT
CGAGACTGGAAAAAGAGCCTATTCATGGAACAAGAGAATTTTAACCAAGAGATTGAACAATTGAATCACAAGTTG
GAAAACTTACAACTGGAATGTCAGGACTTGGAATACAAGCTGTTAATCGGAAAAAGTTCCAACAGCCATTCCACA
GATAGTGCTACTTTAGTAAACGTTCACATCAAAAGGTAG

>YDR231C 1.96 Hot
ATGCGTTGGTGGCCGTGGTCAAATCAGACAGAAGATCAAAAGCAGCAGCAACAACCGCAGGGTAAAGCAGATGGT
GATCGCGTGCTGACAAATTACTCGCGGGGTCAAAAGATCCTCTTAGAAGACACTCCACCCAAGTTTGCCGATGAC
TTGTCCAATTCTCAGTTGGCCAAGAAGCAAGAACGGGCTACTTTGAAAGAGGCTTGGGACTCGATCAGATGGAGT
GATTTTAGTTTGCAGAAGCTGACTTCAATCCCGTGCTTCAGAGACGCGGGCATGCTCGGGTTTTCCAGCATGTTC
TTGATGGGATCTATTATATTTATATACCATAAGAGCCCGACTAAGGCCACGAATTGGGCGATGAGCTCTCTGATA
CTGGGCTCGATTGTCGGATGGGAGCAATGTAGGCTAAAGAGACAAAAAAGTTTCCAGATCGCACAATTGGCAAAG
GAAACCGTGGCGAAAAAGGAAAAACCAATGCTCCATAATGTCCCTCATGACCCATCTCTGCCGGGGCAATGGGAG
GCCGCCAAAAATGAAAAGCAGAGTCAGTTTGAACAAAGTAACCAGAATTTATCTCAGGCCTCCTCTGAGAAGAAA
TGGTACAAGTTCTGGTGA

>YAL020C 1.68 Hot
ATGAGTTGTGTGTATGCGTTTGGGTCTAATGGGCAAAGGCAACTGGGACTGGGGCACGATGAGGATATGGATACC
CCACAGAGGTCTGTGCCAGGAGATGATGGAGCAATAGTCAGGAAGATAGCGTGCGGTGGGAACCACAGCGTGATG
CTGACAAATGACGGGAATCTGGTAGGATGTGGAGATAACAGACGGGGAGAACTGGATAGTGCGCAAGCACTGCGG
CAGGTGCATGACTGGAGGCCCGTGGAAGTACCGGCACCCGTGGTGGATGTGGCGTGCGGCTGGGACACGACAGTT
ATTGTGGATGCTGATGGCCGTGTATGGCAGAGAGGAGGCGGTTGCTACGAGTTCACTCAGCAACATGTGCCATTG
AATTCCAACGATGAGCGCATCGCAGTATACGGATGTTTCCAGAACTTTGTGGTGGTGCAAGGCACCCGAGTATAC
GGCTGGGGCAGCAACACAAAGTGTCAATTGCAAGAGCCCAAATCCCGATCACTGAAAGAGCCCGTATTGGTGTAC
GATACCGGGTCTGTGGCCGTAGACTACGTGGCCATGGGCAAGGACTTCATGGTCATAGTGGACGAGGGCGGCCGC
ATAGTGCACGCATCCGGTCGCCTGCCCACTGGGTTCGAGCTCAAACAACAGCAAAAAAGACACAATCTAGTGGTA
CTGTGCATGTGGACCTCGATCCACCTGTGGAATGCGCGCCTCAATACGGTAGAGTCGTTTGGTAGGGGCACACAT
TCCCAACTCTTCCCGCAAGAGCGCCTAGACTTCCCTATTGTCGGTGTTGCAACCGGGAGTGAGCACGGTATTCTA
ACTACTGCTAATCAAGAAGGCAAGTCTCACTGTTACAATGTATACTGCTGGGGCTGGGGAGAGCATGGCAACTGC
GGCCCGCAAAAGGCGTCCCAGCCTGGACTGCAGCTCGTGGGCCAATACTCTGGAAAACCTCGCGTGTTTGGCGGA
TGTGCCACCACGTGGATCGTGCTCTAG

>YGR079W 1.75 Hot

ATGAGCAGTGCCGCAAATGAGGGATGTGTTTATTTGTTCATTGTCGTTTTGCGATTATCTTCGTTTTCATGTGTT
AACTCATTCATTCATTCATTTACTCGTTCACGCACACGCAGCAGTTATTCCCTCGACGAGCGTTCACTTGTATCA
TATAGCATAGTTTACGTAGCAATGAATAAAAGCAGCAAGGCATTTCAGGTACCGAATAAGGTCATCACCAAGGAG
GATATTACTCCCTTGTCCAGATCGCACACAAAGAAAGCGGACACTCGCGGGACAGCAGATGGTAAAAACACCACA
GCCAGTGCAGTCGAGGCCACCCCAATCATCATCACCACTGCTCGTTCGATAGATACTGCCGGTTCACTATCTGAG
AACGCCACCGAGGACGATGGCACCCAAAACGGAGATTTACACGATGATGACGATGATGACGACCTCGAAAGTACG
CTCGGCTATAGTTCTGAGCCTGATCCTCTCTTCTCCCCATGCCACCAGCCTTCCTTCACCAACAGCACATTTAGT
TACAGTGCGGACAATGAACTACCCATGGAGGAAAATCACAACAAGAATAATTTCCACGATAGCAGCGAGAGCAGT
ATCTTTCTACCACAGATACAGCAATCATTCTTTTTCGGCGACAATAGCAAGAGTGACGCAAATAATACGGACTTC
TGGAAGGAAGTAAATGGCACTGCAGAAGAGGCCATTTGCCTGCAGGAGACAAGACAAAGAAAATGCTCCCTCGTG
GCTCTGCATCCAGGCGACGCCACCACGAGTTCGAACGACACATTGGGAATAGAGGACTTCATCAAAGATGATATC
AACAGTGCTGAAGCCATGGAACCCTCCCCATCTTCATCACCATCTTCCTCCCTTCTTGATAATTTGGACTACAAT
ATCAAGTTATTGTGCTACAGAGACAATGAGGGCAAGTTCACCTTGAAAAAAAGGAAGTTTTTGAAGAATTCCCTG
CGCTCATCCTCTGCAATTTCTAAAAAGTGGAAACCCTTGTCTAAAAGAGATAAGCTGTTGAAGAGGGCCATAAGA
AGAAAGAGTGGTGTTTGCCAGACGTTATCAGCTGGTTTTGGTATCGGTGAATTCATGTTGTAA

>YCR046C 2.44   Hot
ATGTGGAGCAGGAACGTCAGATTGCTTGGATCATGGACAAGGTCCTACATGGTCCCCGCCACCAAGAGAAAAACC
ATCCCCGTGTACCCACCTGTGCAGCGCATAGCTTCGTCGCAGATTATGAAGCAGGTGGCCCTCTCAGAAATAGAG
TCTCTGGATCCCGGGGCCGTTAAGAGGAAGCTCATCAGTAAAAAGAACAAGGACCGCTTGAAGGCAGGCGACGTG
GTCCGGATTGTGTACGACTCGTCCAAGTGCTCGTACGACACCTTTGTTGGCTACATCCTTTCCATAGACCGCAAA
CAACTGGTGCAAGACGCCTCGTTGCTGTTGCGGAACCAGATAGCCAAGACGGCCGTCGAGATTAGAGTGCCATTG
TTTTCGCCGCTGATCGAGAGAATCGACTTGCTAACCCCCCACGTCTCGAGCAGACAAAGAAACAAACACTACTAC
ATCAGAGGTACAAGGTTGGATGTCGGCGACCTCGAGGCAGGTCTAAGAAGAAAGAAATAG

>YBL113C 1.55   Hot
ATGGACTTGAATCAAAGAAAGGAAAAAAAGGGCCAGCATGTTGGATGCTGTGGCTCCAGGACAGACCTGTCTGCT
GACACAGTGGAACTGATAGAAAGAATGGACAGATTGGCTGAAAATCAGGCGACAGCTTCCATGTCGATCGTTGCG
TTACCGTCTAGCTTCCAGGAGAGCAATAGCAGTGACAGGTGCAGAAAGTATTGCAGCAGTGATGAGGACAGCGAC
ACGTGCATTCATGGTAGTGCTAATGCCAGTACCAATGCGACTACCAACTCCAGCACTAATGCTACTACCACTGCC
AGCATCAACGTCAGGACTAGTGCGACTACCACTGCCAGCATCAACGTCAGGACTAGTGCGACTACCACTGAAAGT
ACCAACTCCAACACTAATGCTACTACCACTGAAAGTACCAACTCCAGCACTAATGCTACTACCACTGCCAGCATC
AACGTCAGGACTAGTGCGACTACCACTGAAAGTACCAACTCCAGCACTAATGCTACTACCACTGCCAGCATCAAC
GTCAGGACTAGTGCGACTACCACTGAAAGTACCAACTCCAGCACTAATGCTACTACCACTGCCAGCATCAACGTC
AGGACTAGTGCGACTACCACTGAAAGTACCAACTCCAACACTAATGCCAGTACCAATGCGACTACCAACTCCAGC
ACTAATGCTACTACCACTGCCAGCACCAACGTCAGGACTAGTGCTACTACCAATGCGACTACCAACTCCAGCACT
AATGCTACTACCACTGCCAGCACCAACGTCAGGACTAGTGCTACTACCACTGCCAGCACCAACGTCAGGACTAGT
GCTACTACCACTGCCAGCATCAACGTCAGGACTAGTGCTACTACCACTGAAAGTATCAACTCTAGCACTAATGCT
ACTACCACTGAAAGTACCAACTCCAACACTAGTGCTACTACCACCGAAAGTACCGACTCCAACACTAATGCTACT
ACCACTGCTAGCATCAACGTCAGGACTAGTGCGACTACCACTGAAAGTACCAACTCCAACACTAGTGCTACTACC
ACCGAAAGTACCGACTCCAACACTAGTGCTACTACCACTGCTAGCACCAACTCCAGCACTAATGCCACTACCACT
GCTAGCACCAACTCCAGCACTAATGCCACTACCACTGAAAGTACCAACGCTAGTGCCAAGGAGGACGCCAATAAA
GATGGCAATGCTGAGGATAATAGATTCCATCCAGTCACCGACATTAACAAAGAGTCGTATAAGCGGAAAGGGAGT
CAAATGGTTTTCCTAGAGAGAAAGAAACTGAAAGCACAATTTCCCAATACTTCCGAGAATATGAATGTCTTACAG
TTTCTTGGATTTCGGTCTGACGAAATTAAACATCTTTTCCTCTATGGTATTGACATATACTTCTGCCCAGAGGGA
GTATTCACACAATACGGATTATGCAAGGGCTGTCAAAAGATGTTCGGGCTCTGTGTCTGTTGGGCTGGCCAGAAA
GTATCGTATCGGAGGATAGCTTGGGAAGCACTAGCTGTGGAGAGAATGCTGCGAAATGATGAGGAATACAAAGAA
TACTTGGAAGACATCGAGCCATATCATGGGGACCCTGTAGGATATTTGAAATATTTTAGCGTAAAAAGGAGAGAG
ATCTACTCTCAGATACAGAGAAATTATGCTTGGTACCTGGCCATTACTAGAAGAAGAGAAACAATTAGTGTATTG
GATTCGACAAGAGGCAAGCAAGGGAGCCAAGTTTTCCGCATGTCTGGAAGGCAGATCAAAGAGTTGTATTATAAA
GTATGGAGCAACTTGCGTGAATCGAAGACAGAGGTGCTGCAGTACTTTTTGAACTGGGACGAAAAAAAGTGCCGG
GAAGAATGGGAGGCAAAAGACGATACGGTCTTTGTGGAAGCGCTCGAGAAAGTTGGAGTTTTTCAGCGTTTGCGT
TCCATGACGAGCGCTGGACTGCAGGGTCCGCAGTACGTCAAGCTGCAGTTTAGCAGGCATCATCGACAGTTGAGG
AGCAGATATGAATTAAGTCTAGGAATGCACTTGCGAGATCAGCTTGCGCTGGGAGTTACCCCATCTAAAGTGCCG
CATTGGACGGCATTCCTGTCGATGCTGATAGGGCTGTTCTACAATAAAACATTTCGGCAGAAACTGGAATATCTT
TTGGAGCAGATTTCGGAGATGTGGTTGTTACCACATTGGCTTGATTTGGCAAACGTTGAAGTTCTCGCTGCAGAT
AACACGAGGGTACCACTGTACATGCTGATGGTAGCGGTTCACAAAGAGCTGGATAGCGATGATGTTCCAGACGGT
AGATTTGATATAATATTACTATGTAGAGATTCGAGCAGAGAAGTTGGAGAGTGA

>YLR187W 1.76   Hot
ATGAAAAGGATTTTCTCTGGTGTGAAGTCCCCAAAGTTATCTGCTCCCCCAAAAGTTTTCAAAAATGATGAGAGC
CCATCCACTCCGAGCTCTCCCAAATTCGACCAAGGGCTTCGAAGTTTATCAGCATCAGCTTCAAGACTCTTCAGT
AACTCAATATCCACTCCTGGGAGCCCTACGTTAGATCTTCCGCAAGAACACTCTATTAATGGTGATATCTCACCA
GAACTAGTACCAATCGTTACTTTACTCTCTGCACAGGCTCATAGGAGATACCATTATGGTATATTCTTGATATTA
CATGATTTGAAGACAGACGGAACACCTGCCGCGCGTCAGTGGGAAGAATGCTATGGTGTGTTGCTAGGAACTCAG
CTGGCTTTATGGGATGCAAAAGAATTATCAGATTCCAAGAATAATAAGAACACATCAACCATGAAAAAAGCCGCA
TCGAGACCTTCTTTTATCAATTTTACAGATGCTTCTGTGAGGAGTTTGGACGCTAATGATCAGGTAATCATTGCA

TCAGAGAACGAAAAGACAAAGAAAGATCTAGACAATGTTCTTGTTGTTTCAACAACTTTAAAAAACAGGTATTTC
TTAAAATTCAAAAATTCAAAATCATTCAAAACTTGGAACGCAGCAATTAGGCTTAGTTTGTTCGAGTTTACGGCG
TTGCAAGAGGCCTACACGGGCTCATTTTTGTCAAGCAGAGGTGTTAAACTTGGTGATATCAAAGTTGTTATGGCA
GATACAAAATTCACTTATGAGGACTGGGTTAGCGTAAGATTCGGGACAGGTATGCCATGGAAACGTTGTTATGCT
GTTATTTCTCCACAGTCTGGCAAAAAAAAGAAAAATTCGAAAGGTTCAATATGTTTCTATGAAAATAACAAAAAA
ACCAAAAAATCAAACATAATGACAACGGTAGTAGATGCACGGGCTTTATATGCCGTTTATCCTTCCTCGCCGATA
TTAATCGACACATCTACAATCATAAAATTGGAAGGTTTTGTCTCTTTTGACAAAAGTGAGGAACCTCAAGAAACC
AACCTATTTATCATGCCTGAAAAGCATCAAGGTGTTCCGGGATATGACACTATTATTCGCTTCTTAATCCCAGCT
ATGAACGCGTTCTACTTATATGGTAGGCCAAAGGGTTTGATTGCCAACAGAACTGACCCAGACTCCCTGCTGTTT
GCCTTACCCACTCTTCCGCATATATATTATTTGCAGGTGGATGACGTTTTATCATTAACAAAAGACAAAAATTAC
ATACATTGGAGTGCTGCTGATTGGAGGAATAACATTGTTCAAGTATTACAGAAAAAATTAAGTAAGGGCTATAAA
GGCTGTGGTAATAAAACTGTATCAGTTTCATCTGGGATGATGAAGTCGCCGGCAATTAGTTCAGCTGAATTATTT
GAAGGGTACGATTCTCTCCCTGAAAGACAAATGGAAAGTCCGCAAAAATCTAAGATGAAGTCTCCTACATTAGCA
TCGACCGATGATATTAATTCCGCTTCTGCTTCCGTAAACTCACATGCTACCTCAGTAAAACAAACTGAATTATTT
GTGACTGATAATTCTTCAAAAATCAATGACTCTGTGTCGGCGCAGTCCAGTGTCACTACTAACTTCAAAGATACT
TTTACCACCCCAATGACATCAGGAATGCTCAACCATGAAAATTCAGAGAGAAGCTTTGGTTCAGGATTAAAACTC
AAAATCACTGATTCAAATTTGGAAAATATGGAAGACGTTGAAGCGAAATCTGCTAACGAATTCTCTACGACACCA
GAAGACAAACATATTCACCTCGCCAATGCAGCCGAATTATCCGCCCTTTACGACAAATATTCCACATCTCCGTTT
GGTAAATCGGAGGCTAATTCAAGCCCTAAACCTCAAAAATTGGAAGTTAAAGATCGGTCAAAAAATGAAAACAGA
AGCCCCTATGAGAGATACGTAGGTACATCCGCTGAAAGCAAGACATTTGAAATAGGTAACGTCAGAGAGTCGAAA
AGTACGATAAATACTTCCCTTTCTTCGCCTTTGAGGGTAGAAGATAGTAGACGTTCAAAAAATGAGGACCTGGGA
TCTTTGAAAGAGTTTGAAGAGTTATCTCAAAAGATTAGCAATATGGGAATGGCAAATATTTCTTCAGAAGCTTTA
AGTGACACAGCAGAAAACAGTTCTTTTGTTACGGACCTGAACTTGAATATCAATAACAGTTCATCGGTCAATCTC
AATGAAGAACAACGTGTGCCGGATTTTGGGGAGGAAAACGTATTTGATCCAGATTATATGGAACAAAACCAAATG
TTGGAAACAGAAAGTAGGTACACTACGGATGAGTTTGACTTTTCAGATAATCAGGATGCAGCATCCAGTAATTAT
AGCAATGGGCAAACCAACCGAACAGTAACTGAAACCCTTTCTGCCAGTGACAGAAATGACAAGATCCCACATTCT
TCTTTATTTACAAACTTAAACCAGCTTACTTCGAATGGAGGAAACTATCAGGATAGGGAAGATTTCTCTGGTGAT
CAAATAAACAAACCTCAACAATCTCAACCACTTCATGTGAAAGGACCACAAACTTCGTCATTTGGTTACAGAAAT
TCTTCAGCCAATAGCTCGCAGCCTCAAGCACCATACCCGGTTGGTCGCCCGTTAGGTAAGATAAGAACGGGTCCG
CTGACCGTCCAGCCTATGCAACAAGGTGGAAACTCATCAATGTATTCTTTCCAATCTTCGCAACATCGGTTTCAT
TCGTCGCAACAGCGCCAAAACCAATCACTTTCCTTCAGAAACAATACCTATGGAAGTGGTAACAATCAAAATACA
TTCCATCCATCGCCGCAACTGCAACAACAGCCTCAAAATATGAGATATTTGAATAATAAGTTACCAATAAATGAT
AGATCTCCAATACCGCAAACACAGCACCATGTACCAGATGGCCGTCCCAGCCTCCATATAAATACCACTAACCGC
ACAAATCCGCTTACTGCACAAAGTGGATTTTCTCAATTTATGCCTCCCAATAGCACATCAACCAACCCATATTCC
AGCTGA

>YAR042W 1.57 Hot
ATGGAACAACCTGATCTATCGTCTGTGGCCATCAGTAAGCCGCTGCTGAAGTTGAAACTTCTCGACGCCCTTCGC
CAGGGAAGTTTCCCCAACCTACAAGATCTCCTAAAGAAACAATTCCAGCCGCTAGACGACCCAAACGTCCAACAA
GTGCTCCATCTCATGCTCCACTATGCCGTGCAAGTCGCCCCCATGGCTGTCATAAAGGAAATCGTCCATCATTGG
GTCTCAACTACAAACACCACTTTTCTAAACATCCATCTTGATCTAAACGAACGGGACTCCAACGGCAACACCCCA
TTGCACATCGCCGCCTACCAGTCCCGCGGTGATATCGTAGCCTTCCTCCTGGACCAACCAACCATCAACGACTGC
GTGCTCAACAACTCCCACTTGCAGGCCATCGAAATGTGCAAGAACCTAAACATCGCGCAGATGATGCAGGTGAAA
CGCTCCACATACGTTGCAGAGACCGCCCAGGAATTCAGAACAGCTTTTAACAACAGGGACTTCGGCCACCTAGAA
TCTATCCTCTCCAGCCCTCGAAACGCAGAACTGCTCGACATCAACGGTATGGACCCGGAGACTGGCGATACCGTT
CTGCACGAATTCGTCAAGAAAAGAGACGTCATCATGTGCCGTTGGTTGCTTGAACACGGTGCTGACCCCTTCAAG
AGAGACCGCAAGGGCAAACTGCCCATCGAGCTCGTTAGGAAAGTCAATGAAAACGACACCGCCACCAACACCAAG
ATCGCCATCGACATCGAACTGAAAAAACTATTGGAAAGGGCCACCAGGGAGCAAAGTGTCATCGACGTCACAAAC
AACAACTTGCACGAGGCCCCCACTTACAAAGGCTACCTGAAAAAATGGACCAACTTCGCTCAAGGCTACAAATTG
CGTTGGTTCATCCTTAGTAGCGATGGGAAACTATCCTACTACATCGATCAGGCCGACACTAAGAATGCCTGCAGG
GGCTCCCTAAACATGTCTTCGTGCTCTCTGCATTTGGATTCGTCTGAAAAGTTGAAATTCGAAATTATCGGCGGT
AACAACGGTGTTATCAGGTGGCATTTAAAGGGGAACCACCCCATCGAGACAAATAGATGGGTTTGGGCCATCCAG
GGCGCCATAAGATACGCAAAGGACAGAGAAATTTTGCTGCACAATGGCCCCTATTCGCCTTCTCTGGCCTTAAGC
CATGGCTTGTCATCCAAAGTGTCCAATAAAGAAAACTTGCATGCAACTTCAAAACGGTTGACCAAGAGCCCGCAT
CTGTCCAAATCCACACTGACACAAAACGATCACGATAATGACGATGACAGCACTAACAACAACAACAACAAAAGT
AATAATGATTATGACGATAATAATAATAATAATAATAATGACGATGATGATTATGATGATGATGATGAAAGTAGA
CCCCTCATAGAACCATTACCGTTGATTTCATCCAGAAGCCAAAGCTTAAGCGAAATCACTCCCGGTCCACATTCT
AGGAAGTCTACAGTCTCGTCTACAAGGGCAGCCGATATACCATCAGATGATGAGGGTTACTCTGAGGACGATTCT
GATGACGACGGTAACTCCTCTTACACAATGGAAAACGGCGGTGAAAACGATGGCGACGAAGATCTAAATGCCATT
TATGGTCCCTATATTCAAAAACTACACATGCTACAAAGATCCATTTCCATCGAGTTGGCATCTTTGAACGAATTG
CTGCAAGATAAACAACAACACGATGAGTACTGGAACACCGTCAACACTTCTATTGAAACCGTCAGCGAATTTTTC
GACAAATTAAATCGGTTGACCTCTCAAAGGGAAAAAAGAATGATTGCCCAAATGACCAAGCAACGGGATGTTAAC
AATGTTTGGATTCAATCGGTAAAAGATCTGGAAATGGAACTGGTTGATAAAGACGAAAAATTGGTTGCCTTGGAT
AAAGAACGGAAAAAATCTGAAAAAAATGCTTCAAAAAAAAATTGAACAATCAACCACAGGTTGAAACTGAGGCTAAT
GAAGAATCCGATGATGCAAATTCAATGATAAAAGGATCCCAAGAATCAACAAATACCCTTGAGGAAATCGTAAAA
TTTATCGAAGCAACAAAGGAAAGTGATGAGGATTCTGACGCCGACGAATTTTTCGACGCAGAAGAAGCTGCTTCC
GACAAAAAAGCCAATGATTCGGAAGACTTAACCACAAACAAGGAGACTCCAGCTAATGCGAAACCACAAGAAGAA

GCTCCTGAAGACGAGAGCCTTATTGTGATCAGTTCTCCACAGGTGGAAAAGAAGAACCAACTATTAAAAGAGGGA
TCATTCGTCGGATATGAAGACCCAGTGAGAACCAAACTGGCTTTAGACGAAGATAATCGTCCCAAGATTGGTCTC
TGGTCTGTTTTAAAGTCTATGGTCGGTCAAGACTTAACCAAACTAACTCTACCGGTATCGTTCAATGAGCCAACA
TCCTTACTACAGAGAGTATCTGAAGATATTGAGTATTCTCATATTCTTGACCAAGCTGCCACTTTTGAAGACTCC
TCTTTAAGAATGCTATATGTAGCTGCCTTTACTGCATCAATGTACGCATCTACCACTAACAGAGTGTCTAAACCA
TTCAACCCCTTACTCGGTGAAACTTTTGAATATGCCAGAACTGATGGTCAGTACCGATTCTTCACCGAACAAGTC
TCTCACCACCCACCTATCTCTGCTACTTGGACAGAATCGCCCAAATGGGATTTTTACGGTGAATGTAATGTTGAT
TCGTCATTCAATGGGCGCACGTTCGCCGTGCAACATTTAGGATTATGGTACATTACTATCCGGCCTGATCATAAT
ATTAGTGTTCCCGAGGAAACTTATTCCTGGAAAAAACCAAATAACACTGTTATCGGTATTTTAATGGGGAAACCA
CAAGTAGACAACAGTGGGGACGTCAAAGTCACAAACCATACCACAGGCGACTATTGTATGCTGCATTACAAAGCC
CATGGCTGGACCTCAGCCGGTGCATATGAAGTCAGAGGTGAAGTATTCAACAAGGACGATAAAAAATTATGGGTT
CTTGGTGGGCATTGGAATGATTCCATTTACGGGAAAAAAGTAACTGCTAGAGGCGGAGAACTGACATTAGACAGA
ATAAAAACGGCAAATTCTGCCACGGGAGGACCAAAACTAGATGGGTCTAAGTTTCTGATATGGAAAGCAAATGAA
AGGCCTTCAGTGCCATTTAATTTAACGTCGTTTGCATTGACTTTGAATGCTTTGCCACCCCACTTGATACCATAT
TTAGCACCCACAGATAGTCGTTTAAGGCCCGATCAAAGGGCTATGGAAAATGGTGAATACGATAAAGCTGCCGCG
GAAAAGCATCGTGTTGAAGTAAAACAAAGGGCAGCAAAAAAAGAAAGGGAACAAAAAGGAGAAGAATACAGACCT
AAGTGGTTTGTCCAGGAGGAGCACCCCGTTACCAAAAGTCTATACTGGAAATTTAATGGAGAGTATTGGAACAAA
AGAAAAAATCATGACTTTAAAGATTGTGCTGATATTTTCTAA

>YGL056C 1.52 Hot
ATGCCTCAAAATACAAGACACACGTCCATCGTAGAAATGCTTTCTACCCCACCTCAATTACCGAATTCCACAGAT
TTAAATAGTTTGAGCGAACAGACAGACAAGAACACTGAAGCAAACAAAAGCGACACAGAATCACTACATAAATCT
ATTTCGAAATCGTCATCTTCATCTTCTCTCTCCACGCTGGATAACACGGAATACTCAAACAATAATGGCAACTCC
TTGTCCACCTTGAATTCACAGAATCTGTTATCTGTTCATAGGCAGGAGTGGCAACACACTCCTCTGTCAAATTTG
GTGGAACAGAACAAATTGATTTTCATCAGAGGTTCTATTTCCGTAGAGGAAGCATTCAACACGCTGGTCAAGCAC
CAGCTAACTTCTTTGCCTGTGGAGAATTTTCCGGGAGACATGAATTGTTTAACATTCGACTATAATGATCTCAAT
GCGTACCTTCTTCTTGTATTAAACAGGATCAAGGTGAGCAACGACAAGATAACCTCAGACTGCCAGAATGGTAAG
TCCGTGCCCGTGGCGGAGATAGTAAAGTTGACACCTAAGAATCCATTCTACAAATTGCCCGAAACGGAAAACTTA
TCGACGGTAATAGGCATACTAGGCTCAGGTGTGCATCGTGTTGCCATCACGAACGTGGAAATGACACAGATTAAA
GGTATACTTTCCCAACGTCGATTGATTAAGTATCTGTGGGAGAATGCGAGATCATTTCCCAACTTGAAGCCTCTC
TTGGACTCTTCCCTGGAGGAGCTGAATATTGGTGTACTAAATGCAGCCCGTGATAAACCTACATTCAAGCAGTCA
CGTGTCATATCCATCCAAGGTGACGAGCATCTAATTATGGCTCTGCATAAAATGTATGTCGAAAGAATCTCTTCC
ATTGCGGTAGTTGACCCGCAGGGAAACTTGATTGGTAATATTTCAGTAACAGACGTCAAACACGTCACCAGAACT
TCACAATATCCTTTATTACACAATACATGTCGTCATTTTGTCTCGGTTATTTTGAATTTAAGGGGCCTAGAGACC
GGTAAGGACTCATTTCCTATTTTCCATGTGTATCCAACAAGTTCCCTGGCAAGAACGTTTGCCAAATTGGTCGCT
ACCAAATCTCACAGATTATGGATAGTACAACCAAATGACAATCAACCAACAGCATCTTCCGAAAAATCCTCATCT
CCATCACCAAGTACTCCACCCGTAACGACATTGCCATCGCTTGCGTCGTCATACCATTCGAACACGCAATCTTCC
AGGATGGCCAACTCTCCTGTTCTAAAATCTTCAGATACATCAAACAATAAAATAAATGTAAATATAAATTTGAGT
GGTCCCTCACCTTCTCAACCACAGTCTCCATCGGCCACAATGCCACCACCTCAAAGTCCAAGTAATTGTCCCGCA
TCTCCAACTCCAGCACATTTCGAAAAGGAGTATAGAACAGGTAAATTGATAGGTGTTGTGTCATTGACTGACATA
TTAAGTGTTTTGGCAAGAAAACAAACGCATCATAAGGAAATTGATCCGCAGATGGCAAGAAAGCAAAGAGGGCAT
ATAGGCTGA

>YBR107C 1.64 Hot
ATGCCTTATACTTGGAAGTTTTTAGGAATCAGCAAGCAACTTTCACTGGAAAATGGTATAGCAAAGTTGAACCAA
TTGCTAAACCTGGAAGTAGATTTAGACATTCAAACCATTCGAGTACCCAGTGATCCTGATGGTGGTACCGCCGCT
GACGAGTATATCCGTTACGAAATGAGGTTGGACATCTCTAATTTAGATGAGGGCACTTACTCTAAGTTCATTTTC
TTGGGGAACTCAAAAATGGAGGTGCCGATGTTTTTGTGCTATTGCGGGACAGATAACCGCAATGAAGTGGTATTG
CAGTGGCTGAAGGCAGAGTACGGTGTCATTATGTGGCCGATAAAATTTGAACAGAAGACCATGATAAAGTTAGCT
GACGCCAGCATTGTGCACGTAACGAAAGAGAACATCGAACAAATTACCTGGTTTAGCTCAAAGTTATATTTTGAA
CCAGAAACACAAGACAAAAACTTGCGGCAATTCAGTATAGAAATACCGAGAGAAAGCTGTGAGGGTTTGGCTCTT
GGCTATGGTAATACTATGCACCCGTATAACGATGCAATTGTACCCTACATTTATAATGAAACGGGGATGGCGGTG
GAAAGATTACCGTTGACTTCCGTCATATTGGCAGGGCACACGAAAATAATGAGAGAATCCATTGTCACTTCAACA
CGAAGTCTCAGGAACAGAGTTCTAGCAGTTGTACTCCAATCGATTCAGTTTACCAGCGAGTAA

>YBR264C 1.59 Hot
ATGGAAGCAACCATCAAAGTGGTACTGCTAGGAGATTCATCAGTGGGGAAAACCAGTATAGTGACTAGGCTCAAA
TCAGGTAAGTTTCTAGCAAAACATGCGGCTACGATAGGTGCGGCGTTCATCACCAAGACAATCGAGGTTCCTTCT
AACGACTCCTCTACGGAGAAACGTATCCATATGGAGATATGGGACACGGCGGGTCAGGAACGGTATAAATCACTG
GTGCCAATGTATTATCGAGATGCGAATATTGCTTTGATTGTATTCGAATTGGGAGACGTATCCAGTCTGCAATGT
GCAAAGACATGGTTTCAAGATTTACAGGACCGTGCTCAGGGAACGCAGGTAATTATCGTGGGCAATAAGTACGAT
TTAGTCTGCGAAGAGCATTCAGGGGAAGTGACTATACCGGCGGAGTTACAGGGTCTGCCGTATGTGGCCGTAAGT
GCAAAGACAGGGTACAATTTCGATACATTGAATAAAATAATAATCAGTTTGGTTCCCGAAAGTCAATTCAAGACA
TTGTCAAAGAACAATGAACAGGGAAATATACTGGAAATAAATAAAAAAAAAAAGCGGCAGTGGCTGTATATGTTAA

>YBR230C 2.27 Hot

ATGTCTGCAACTGCTAAACACGACAGTAACGCTAGCCCAAATTCTGACTCTGAAGACGGTCACCACCACAACAAT
AAGAAGGAATGCGCTATCGAATATCTGAAGGCACGGTTAAATAGTGCATCAGCAGTCGCTTGCGGCTACCTCCAA
GCTTTTGTCAGTAAGACGCAAGACTTTGCCAAAGTATGCTTTTTAGAACTTCAGAATCCCGTTGTCTTGGTCAAC
TTGTTGCTGCATTCTTCAGTGGTATGTTATTTATGTAACGGGTATGCGAACCACAACGCCAGATTCTTGAAGGGG
AAACCTAACTCTACAGTCTTAGCAACAACCGCCGGCGCTCTGGGTCTTTTGACGCTGGACGGTATAATTTCAAAG
AAATACTACTCCAGATACGACAAGAAATAA

>YOR175C 1.52 Hot
ATGTACAATCCTGTGGACGCTGTTTTAACAAAGATAATTACCAACTATGGGATTGATAGTTTTACACTGCGATAT
GCTATCTGCTTATTGGGATCGTTCCCACTGAATGCTATTTTGAAGAGAATTCCCGAGAAGCGTATAGGTTTAAAA
TGTTGTTTTATCATTTCTATGTCGATGTTTTACTTATTCGGTGTGCTGAATCTAGTAAGTGGATTCAGGACCCTG
TTTATTAGTACCATGTTTACTTACTTGATCTCAAGATTTTACCGTTCCAAGTTTATGCCACACTTGAATTTCATG
TTTGTTATGGGTCATTTGGCAATAAATCATATACACGCCCAATTCCTTAACGAACAGACTCAAACTACCGTTGAC
ATTACAAGTTCACAAATGGTTTTAGCCATGAAACTAACTTCTTTTGCATGGTCGTACTATGATGGTTCATGCACT
AGCGAAAGCGATTTCAAAGATTTGACTGAGCATCAAAAATCTCGTGCTGTCAGAGGTCATCCACCCTTATTAAAG
TTCCTGGCATATGCATTTTTCTATTCAACGTTGCTAACTGGCCCAAGTTTCGATTATGCCGATTTTGACAGCTGG
TTGAATTGTGAGATGTTCCGTGACTTGCCTGAAAGCAAAAAGCCTATGAGAAGACACCACCCTGGTGAAAGAAGA
CAGATTCCAAAGAATGGTAAACTTGCATTATGGAAAGTTGTTCAAGGTCTTGCTTGGATGATTTTAAGTACACTA
GGAATGAAGCACTTCCCCGTAAAATACGTTTTGGACAAAGATGGCTTCCCAACGAGATCTTTTATATTCAGAATC
CATTACTTATTCTTGCTTGGTTTCATCCATAGATTCAAGTACTACGCTGCCTGGACTATTTCGGAAGGATCTTGT
ATTTTGTGCGGTTTGGGTTATAATGGTTATGATTCAAAGACACAAAAGATCAGATGGGATCGTGTCAGAAATATT
GACATTTGGACCGTAGAAACGGCGCAGAATACGCGTGAAATGTTGGAAGCATGGAATATGAATACTAACAAGTGG
CTAAAATACTCTGTTTATTTACGTGTCACAAAGAAGGGCAAAAAACCTGGTTTCCGCTCAACTTTGTTTACTTTC
CTAACTTCCGCATTTTGGCATGGTACCAGACCTGGGTACTATCTGACTTTTGCGACAGGGGCTTTGTACCAAACA
TGTGGTAAAATCTACAGACGCAATTTTAGACCAATTTTCTTGCGAGAAGATGGTGTCACTCCTTTGCCTTCTAAA
AAAATCTACGATTTAGTTGGCATATATGCAATTAAACTAGCATTTGGTTACATGGTGCAACCATTTATTATCCTT
GATTTGAAGCCATCTTTAATGGTATGGGGCTCTGTTTATTTCTATGTTCATATTATTGTTGCTTTCTCATTTTTC
CTATTCAGAGGACCATATGCTAAACAAGTTACTGAATTTTTTAAATCCAAACAACCTAAAGAAATATTCATTAGA
AAACAAAAGAAGTTGGAAAAAGATATTTCTGCAAGCTCTCCAAACTTGGGTGGTATATTGAAGGCAAAGATTGAA
CATGAAAAGGGAAAGACAGCAGAAGAAGAAGAAATGAACTTAGGTATTCCACCAATTGAGTTAGAAAAGTGGGAC
AATGCTAAGGAAGATTGGGAAGATTTCTGCAAAGATTACAAAGAATGGAGAAATAAAAATGGTCTTGAAATAGAA
GAGGAAAACCTTTCTAAAGCTTTTGAAAGATTCAAGCAGGAATTTTCTAACGCTGCAAGTGGATCAGGTGAACGT
GTGAGAAAAATGAGTTTTAGTGGTTACTCACCAAAGCCTATTTCAAAAAAGGAAGAGTAG

>YKL039W 1.56 Hot
ATGAGAGTATATCAGTTTTGCCGACCTTTCCAATTATTCACCTGCTTCTTATGCTACCTTCTAGTGTTCGTAAAA
GCTAACAAGGAAAAGATAAGTCAAAAGAATTATCAAGTATGCGCAGGTATGTATTCCAAAGAAGATTGGAAGGGC
AAAATAGACCCGTTTATATCATTCAATCTTAAAAAAATAAGTGGATTGAGTGATGAGTCTGATCCAGGCTTAGTC
GTTGCAATCTATGATTTCCAAGACTTTGAGCACTTAGGTGTGCAATTGCCGGATGAAGAAATGTATTATATTTGT
GATGATTATGCAATTGATATCGGGATTTGTGAAGAGGAGAATCGTGATGAGTTTATTGTTCAAGATGTTGTATAT
GATCCATATACTTCCACAAATAGATCTTTGGCCAATCCAATCATGACGTTTTCTCAAAATGAGGTCGGCCTTCAT
GATACTAGATACCCAATAAAAGAAACCGGATTTTATTGTGTTACTGCGTTCAGATCATCGACGTCTACCAAGTTC
AACGCTGTGGTGAATTTCAGGAATGCTTATGGTCAGCTTGCGGGCACCGAGATCAACAAATTGCCACTATATGGT
CTTTTGGCGGTCGCATACGTTGTGGCGATGGCGTTATATTCCTTCGCCTTCTGGAAGCACAAGCATGAATTGTTA
CCATTACAAAAGTATTTGCTGGCCTTTTTTGTATTTTTAACCGCAGAAACCATATTTGTATGGGCTTACTATGAT
TTGAAAAATGAAAAAGGTGATACTGCGGGAATAAAAGTTTATATGGTGTTCTTGTCGATATTGACGGCTGGTAAA
GTTACATTTTCATTTTTCCTATTATTGATTATCGCTTTGGGCTACGGTATCGTGTACCCTAAGTTGAATAAAACG
TTGATGAGGCGTTGCCAAATGTATGGTGCGTTAACTTATGCAATTTGCATTGGATTTTTGATCCAAAGTTATTTG
ACCGACATGGAGGCACCATCTCCACTAATCTTGATCACCTTGATTCCTATGGCGCTTGCATTAATCATATTTTAC
TACATGATTATTAGATCCATGACAAAAACAGTTATCTATTTGAAAGAGCAAAGGCAGATCGTAAAATTGAACATG
TACAAGAAGCTACTATACATCATTTATGCGTCCTTTTTGAGTGTACTAGCTGGTAGTATAGTTTCGTCATTTATT
TACGTTGGCATGAACACTATTGATATGATTGAGAAGAACTGGAGGTCAAGGTTCTTCGTTACTGATTTCTGGCCT
ACGTTAGTGTATTTCATTGTTTTCGTTACCATCGCGTTCTTATGGAGACCAACAGATACTTCATACATGCTAGCC
GCATCCCAACAACTACCCACAGACCCAGAAAATGTCGCGGACTTTGACTTGGGCGATTTGCAATCGTTTGACGAT
CAAGATGACGCAAGTATCATCACTGGTGAGCGTGGCATAGACGAGGATGACCTCAATTTGAATTTTACTGATGAT
GAAGAAGGACACGATAATGTAAATAACCATAGCCAAGGCCACGGGCCAGTGTCTCCCTCTCCAACAAAATAA

>YHL050C 1.84 Hot
ATGGCAGACACACCCTCTGTGGCAGTACAGGCCCCACCGGGCTATGGTAAGACGGAGTTATTTCATCTCCCCTTG
ATAGCACTGGCGTCTAAGGGCGACGTGGAATATGTGTCGTTTCTGTTTGTACCGTACACAGTGTTGCTTGCTAAT
TGCATGATCAGGTTGGGCCGATGCGGTTGCTTGAATGTGGCCCCTGTAAGAAACTTTATTGAAGAAGGTTGCGAT
GGCGTTACTGATTTATACGTGGGGATCTACGATGATCTTGCTAGCACTAATTTCACAGACAGGATAGCTGCGTGG
GAGAATATTGTTGAGTGCACCTTTAGGACCAACAACGTAAAATTGGGTTACCTCATTGTAGATGAGTTGCACAAC
TTTGAAACGGAGGTCTACCGGCAGTCGCAATTTGGGGGCATAACTAACCTTGATTTTGACGCTTTTGAGAAAGCA
ATCTTTTTGAGCGGCACAGCCCCTGAGGCTGTTGCTGATGCTGCGTTGCAGCGTATTGGGCTTACGGACTGGCC
AAGAAGTCGATGGACATCAACGAGCTCAAACGGTCGGAAGATCTCAGCAGAGGTCTATCCAGCTATCCAACACGG
ATGTTTAATCTAATCAAGGAGAAATCCAAGGTGCCTTTAGGCACTAATGCTACTACCACTGCCAGCACCAACGTC

AGGACTAGTGCTACTACTACTGCCAGCATCAACGTCAGGACTAGTGCGACTACCACTGCCAGCATCAACGTCAGG
ACTAGTGCGACTACCACTGAAAGTACCAACTCCAACACTAATGCTACTACCACTGAAAGTACCAACTCCAGCACT
AATGCTACTACCACTGCTAGCACCAACTCCAGCACTAATGCTACTACCACTGAAAGTACCAACGCTAGTGCCAAG
GAGGACGCCAATAAAGATGGCAATGCTGAGGATAATAGATTCCATCCAGTCACCGACATTAACAAAGAGCCGTAT
AAGCGGAAAGGGAGTCAAATGGTTTTGCTAGAGAGAAAGAAACTGAAAGCACAATTTCCCAATACTTCCGAGAAT
ATGAATGTCTTACAGTTTCTTGGATTTCGGTCTGACGAAATTAAACATCTTTTCCTCTATGGTATTGACATATAC
TTCTGCCCAGAGGGCGTATTCACACAATACGGATTATGCAAGGGCTGTCAAAAGATGTTCGAGCTCTGTGTCTGT
TGGGCTGGCCAGAAAGTATCGTATCGGAGGATGGCTTGGGAAGCACTAGCTGTGGAGAGAATGCTGCGAAATGAC
GAGGAATACAAAGAATACTTGGAAGACATCGAGCCATATCATGGGGACCCTGTAGGGTATTTGAAATTTTTTAGC
GTAAAAAGGAGAGAGATCTACTCTCAGATACAGAGAAATTATGCTTGGTACCTGGCCATTACTAGAAGAAGAGAA
ACAATTAGTGTATTGGATTCGACAAGAGGCAAGCAAGGGAGCCAAGTTTTCCGCATGTCTGGAAGGCAGATCAAA
GAGTTGTATTATAAAGTATGGAGCAACTTGCGTGAATCGAAGACAGAGGTGCTGCAGTACTTTTTGAACTGGGAC
GAGAAAAAGTGCCAGGAAGAATGGGAGGCAAAAGACGATACGGTCTTTGTGGAAGCGCTCGAGAAAGTTGGAGTT
TTTCAGCGTTTGCGTTCCATGACGAGCGCTGGACTGCAGGGTCCGCAGTACGTCAAGCTGCAGTTTAGCAGGCAT
CATCGACAGTTGAGGAGCAGATATGAATTAAGTCTAGGAATGCACTTGCGAGATCAGCTTGCGCTGGGAGTTACC
CCATCTAAAGTGCCGCATTGGACGGCATTCCTGTCGATGCTGATAGGGCTGTTCTACAATAAAACATTTCGGCAG
AAACTGGAATATCTTTTGGAGCAGATTTCGGAGGTGTGGTTGTTACCACATTGGCTTGATTTGGCAAACGTTGAA
GTTCTCGCTGCAGATAACACGAGGGTACCGCTGTACATGCTGATGGTAGCGGTTCACAAAGAGCTGGATAGCGAT
GATGTTCCAGACGGTAGATTTGATATAATATTACTATGTAGAGATTCGAGCAGAGAAGTTGGAGAGTGA

>YPR124W 1.55   Hot
ATGGAAGGTATGAATATGGGTAGCAGCATGAATATGGACGCCATGTCTAGTGCATCCAAGACAGTAGCATCGAGT
ATGGCGTCGATGAGCATGGATGCGATGTCTAGTGCCAGCAAAACGATATTATCGAGCATGTCATCGATGAGCATG
GAAGCGATGTCCAGTGCGAGCAAAACGTTGGCGTCGACTATGTCGTCAATGGCAAGTATGTCGATGGGAAGCAGT
TCAATGTCAGGTATGTCTATGTCGATGAGCAGTACACCAACAAGCTCCGCCAGTGCACAGACAACTTCTGATTCT
AGCATGTCAGGCATGTCAGGTATGTCATCGTCTGATAACAGTAGCTCTTCAGGGATGGATATGGACATGAGTATG
GGAATGAACTATTATCTGACTCCCACATATAAAAACTATCCAGTTTTGTTTCACCATTTGCATGCAAACAATAGT
GGTAAGGCTTTCGGTATTTTCTTATTATTTGTTGTGGCTGCTTTCGTCTACAAACTACTGCTTTTCGTTAGTTGG
TGCCTTGAAGTTCACTGGTTTAAAAAATGGGACAAGCAAAATAAATATTCCACTTTACCTTCAGCAAACTCCAAA
GACGAAGGAAAACATTATGACACAGAGAATAATTTTGAAATTCAAGGTTTACCTAAGCTGCCAAATTTATTAAGC
GATATATTTGTTCCATCTTTAATGGATCTCTTTCATGACATTATAAGGGCGTTCTTAGTATTTACCTCTACGATG
ATTATTTATATGTTGATGCTTGCTACCATGTCTTTTGTTTTAACATACGTTTTTGCTGTAATTACTGGGTTAGCT
TTATCGGAAGTCTTCTTCAATAGATGCAAAATAGCCATGCTAAAGAGGTGGGACATCCAAAGAGAAATTCAGAAA
GCGAAGAGCTGTCCTGGCTTCGGTAACTGCCAATGTGGTAGACATCCCGAACCCAGCCCTGATCCAATTGCTGTT
GCCGATACCACTTCCGGAAGTGATCAAAGTACTCGCCTGGAAAAGAACAACGAATCTAAAGTTGCGATTTCCGAA
AATAATCAAAAGAAAACACCTACACAAGAAGAGGGATGTAATTGTGCCACAGACTCAGGAAAGAATCAAGCAAAC
ATAGAGCGCGACATCCTTGAGAATTCCAAGTTGCAGGAACAGTCCGGGAATATGGATCAAAACTTACTTCCGGCC
GAAAAATTCACTCATAACTAA

>YCL031C 1.54   Hot
ATGGGTATTGAAGACATTAGCGCCATGAAGAACGGGTTTATAGTGGTGCCGTTCAAATTACCGGATCACAAGGCA
CTACCCAAAAGCCAGGAAGCTTCGTTGCATTTCATGTTTGCTAAAAGACACCAGAGTTCAAATTCCAACGAGTCT
GACTGTTTGTTTTTGGTCAACCTTCCATTATTATCTAACATAGAGCACATGAAGAAATTTGTCGGGCAGCTCTGT
GGGAAATACGATACAGTATCGCATGTAGAGGAACTACTATATAACGATGAATTTGGATTACATGAAGTAGATTTA
TCGGCATTGACCTCCGATCTGATGTCCTCCACTGACGTCAACGAGAAGAGATACACACCAAGAAACACGGCGCTA
TTAAAATTTGTTGATGCTGCAAGTATAAATAACTGCTGGAATGCTTTGAAAAAATACTCGAATTTGCATGCCAAA
CATCCAAATGAACTATTTGAATGGACATATACGACTCCATCATTCACAACTTTTGTTAACTTCTACAAACCACTG
GATATTGATTATTTGAAAGAAGATATTCATACACATATGGCAATTTTTGAACAGCGTGAAGCTCAAGCACAAGAG
GATGTTCAAAGTTCTATAGTGGATGAGGATGGATTCACATTAGTTGTAGGAAAGAACACCAAGTCATTGAATTCC
ATAAGAAAGAAAATATTAAACAAAAATCCATTATCCAAACATGAAAATAAGGCCAAGCCAATTTCAAATATAGAT
AAAAAGGCAAAGAAAGATTTCTATAGATTTCAGGTCAGAGAACGTAAAAAACAGGAGATCAATCAACTGTTAAGT
AAATTTAAGGAAGATCAAGAAAGAATCAAGGTAATGAAAGCTAAGAGAAAATTCAATCCATACACTTAA

>YFL011W 1.58   Hot
ATGGTTAGTTCAAGTGTTTCCATTTTGGGGACTAGCGCCAAGGCATCCACTTCTCTAAGTAGAAAGGATGAAATT
AAACTAACCCCTGAAACAAGGGAAGCTAGCTTGGACATTCCATACAAACCCATTATTGCATACTGGACGGTGATG
GGTCTCTGTCTGATGATTGCCTTTGGTGGATTCATTTTTGGTTGGGATACAGGAACCATTTCAGGGTTTATTAAC
CAAACAGATTTCAAGAGAAGGTTTGGTGAGTTACAAAGGGACGGCAGTTTTCAACTATCAGATGTCAGGACAGGG
CTAATTGTCGGTATCTTCAACATAGGTTGTGCTTTAGGTGGCCTAACGCTGGGACGCCTGGGCGATATTTATGGG
CGTAAAATCGGCTTAATGTGTGTTATACTGGTGTATGTTGTTGGTATCGTGATCCAGATTGCTTCCTCTGACAAA
TGGTATCAATATTTTATTGGTAGAATTGTTTCTGGAATGGGTGTTGGAGGTGTTGCTGTGCTGTCGCCAACTTTG
ATCTCAGAAATTTCCCCAAAGCACCTAAGAGGCACTTGTGTCTCTTTTTACCAGCTAATGATTACCCTTGGAATT
TTCTTGGGCTACTGTACCAATTATGGTACAAAGAAATATTCAAATTCAATACAGTGGCGGGTTCCCTTGGGTTTG
TGTTTTGCGTGGGCAATCTTTATGGTGATTGGAATGGTTATGGTTCCGGAATCGCCCAGATATTTAGTAGAAAAA
GGTAAGTATGAAGAAGCTAGAAGGTCTTTGGCCAAATCAAACAAGGTCACAGTTACTGATCCAGGCGTTGTTTTT
GAGTTTGATACTATAGTTGCAAATATGGAATTAGAAAGGGCTGTTGGAAATGCCAGTTGGCACGAACTCTTCTCA
AATAAAGGAGCAATTCTACCAAGGGTAATAATGGGAATCGTTATCCAGTCACTGCAACAGCTTACTGGCTGTAAT

TATTTTTTCTACTACGGCACGACCATTTTCAATGCTGTTGGAATGCAAGACTCTTTCGAGACTTCCATTGTCCTT
GGGGCTGTTAATTTTGCTTCTACATTTGTTGCACTATACATTGTGGATAAATTTGGGCGTCGAAAATGTTTATTG
TGGGGGTCTGCCTCGATGGCAATTTGTTTCGTCATATTCGCCACCGTTGGCGTCACTAGATTATGGCCACAAGGG
AAAGACCAACCTTCTTCGCAAAGTGCTGGTAATGTTATGATCGTTTTTACTTGTTTCTTCATTTTCTCTTTTGCC
ATTACTTGGGCTCCTATCGCCTATGTCATTGTGGCAGAAACTTATCCATTAAGAGTTAAAAATCGTGCCATGGCC
ATTGCGGTTGGTGCGAACTGGATGTGGGGTTTCTTGATTGGATTTTTCACACCCTTTATCACTAGATCCATAGGA
TTTTCTTATGGCTATGTTTTCATGGGTTGCTTAATCTTTTCGTACTTCTACGTTTTCTTCTTTGTTTGCGAAACA
AAGGGATTAACTCTGGAGGAAGTTAATGAAATGTACGAAGAAAGAATAAAGCCATGGAAGTCCGGAGGTTGGATT
CCCAGTTCTAGAAGAACACCACAACCAACAAGCAGTACACCATTAGTTATTGTTGATAGTAAATAA

>YDR382W 1.62   Hot
ATGAAATACTTAGCTGCTTACTTATTATTGGTTCAAGGTGGTAACGCTGCCCCATCCGCCGCTGACATCAAGGCC
GTCGTCGAATCTGTCGGTGCTGAAGTCGATGAAGCCAGAATCAACGAATTGTTGTCCTCTTTGGAAGGTAAGGGC
TCTTTGGAAGAAATCATCGCTGAAGGTCAAAAGAAGTTCGCTACTGTTCCAACTGGTGGTGCTTCTTCTGCTGCT
GCCGGTGCTGCCGGTGCTGCTGCCGGTGGTGATGCTGCTGAAGAAGAAAAGGAAGAAGAAGCTAAGGAAGAATCT
GATGATGACATGGGTTTTGGTTTATTCGATTAA

>YLR081W 1.61   Hot
ATGGCAGTTGAGGAGAACAATATGCCTGTTGTTTCACAGCAACCCCAAGCTGGTGAAGACGTGATCTCTTCACTC
AGTAAAGATTCCCATTTAAGCGCACAATCTCAAAAGTATTCTAATGATGAATTGAAAGCCGGTGAGTCAGGGTCT
GAAGGCTCCCAAAGTGTTCCTATAGAGATACCCAAGAAGCCCATGTCTGAATATGTTACCGTTTCCTTGCTTTGT
TTGTGTGTTGCCTTCGGCGGCTTCATGTTTGGCTGGGATACCGGTACTATTTCTGGGTTTGTTGTCCAAACAGAC
TTTTTGAGAAGGTTTGGTATGAAACATAAGGATGGTACCCACTATTTGTCAAACGTCAGAACAGGTTTAATCGTC
GCCATTTTCAATATTGGCTGTGCCTTTGGTGGTATTATACTTTCCAAAGGTGGAGATATGTATGGCCGTAAAAAG
GGTCTTTCGATTGTCGTCTCGGTTTATATAGTTGGTATTATCATTCAAATTGCCTCTATCAACAAGTGGTACCAA
TATTTCATTGGTAGAATCATATCTGGTTTGGGTGTCGGCGGCATCGCCGTCTTATGTCCTATGTTGATCTCTGAA
ATTGCTCCAAAGCACTTGAGAGGCACACTAGTTTCTTGTTATCAGCTGATGATTACTGCAGGTATCTTTTTGGGC
TACTGTACTAATTACGGTACAAAGAGCTATTCGAACTCAGTTCAATGGAGAGTTCCATTAGGGCTATGTTTCGCT
TGGTCATTATTTATGATTGGCGCTTTGACGTTAGTTCCTGAATCCCCACGTTATTTATGTGAGGTGAATAAGGTA
GAAGACGCCAAGCGTTCCATTGCTAAGTCTAACAAGGTGTCACCAGAGGATCCTGCCGTCCAGGCAGAGTTAGAT
CTGATCATGGCCGGTATAGAAGCTGAAAAACTGGCTGGCAATGCGTCCTGGGGGGAATTATTTTCCACCAAGACC
AAAGTATTTCAACGTTTGTTGATGGGTGTGTTTGTTCAAATGTTCCAACAATTAACCGGTAACAATTATTTTTTC
TACTACGGTACCGTTATTTTCAAGTCAGTTGGCCTGGATGATTCCTTTGAAACATCCATTGTCATTGGTGTAGTC
AACTTTGCCTCCACTTTCTTTAGTTTGTGGACTGTCGAAAACTTGGGACATCGTAAATGTTTACTTTTGGGCGCT
GCCACTATGATGGCTTGTATGGTCATCTACGCCTCTGTTGGTGTTACTAGATTATATCCTCACGGTAAAAGCCAG
CCATCTTCTAAAGGTGCCGGTAACTGTATGATTGTCTTTACCTGTTTTTATATTTTCTGTTATGCCACAACCTGG
GCGCCAGTTGCCTGGGTCATCACAGCAGAATCATTCCCACTGAGAGTCAAGTCGAAATGTATGGCGTTGGCCTCT
GCTTCCAATTGGGTATGGGGGTTCTTGATTGCATTTTTCACCCCATTCATCACATCTGCCATTAACTTCTACTAC
GGTTATGTCTTCATGGGCTGTTTGGTTGCCATGTTTTTTATGTCTTTTTCTTTGTTCCAGAAACTAAAGGCCTA
TCGTTAGAAGAAATTCAAGAATTATGGGAAGAAGGTGTTTTACCTTGGAAATCTGAAGGCTGGATTCCTTCATCC
AGAAGAGGTAATAATTACGATTTAGAGGATTTACAACATGACGACAAACCGTGGTACAAGGCCATGCTAGAATAA

>YMR244C-A   2.18   Hot
ATGGGCTTATTTTCATTTGATGGTGGCAAAAAGGAATCACAACCCCCTAATACCCGTTCACAGAGAAAGTTGTGC
TGGGAGTCCAGGGACGCATTCTTCCAGTGTTTGGACAAGGCAGACATCTTGGACGCAATGGACCCCAAGAATAGC
AAGAGTATAAAGTCGCACTGCAAGGTGGAAAATGAAAAATTTGAGGAGAATTGCGCCCATAGCTGGATCAAGTAC
TTCAAGGAGAAGAGGGTCATCGACTTTAAGAGAGAGCAGACCATCAAGAGAATCGAGCAGGAAGCTAAACAGAGG
GAACGAAATCAGTGA

>YJL027C 2.08   Hot
ATGATGGTAACGCGCAAACACTACCGCTATATATACCTACAAAACTCTCATTCTCTCATTTCCTGCTTCGTTCAT
TTCGAGTTTCCAAGGGTATGGTACGGTGCTATCTGCCCGTGCTTCCCCTCCTTTGCTTTGCTAAGAAAAATTTTC
TTTTGTCAGCAGCAACAACACGCTACGCTCTGCGCTGTGCTACGAAGTGGTCTCTGTGGCAACGGGGACATTGTA
CCCATGCCCGCTCGCAGGGAGGTCTGGGTGTGGGGCGTGTGCGACCTCGTTGCCATGGCGATCGCGCGTGGGTGT
GGTCTTTCCCCAAACGGTTGCCCGTTGCTCCGGATTTCGCATAGTTGTCGCGTAAACAAAAAACACGAGAGAGGG
CGGACGGCGCTGAATAGTGGAAGATCGCGCGATGTCAAGTAA

>YDR044W 1.59   Hot
ATGCCTGCCCCTCAAGATCCAAGGAATCTTCCAATTAGACAACAAATGGAAGCTCTTATCCGTCGCAAACAAGCT
GAAATCACGCAAGGTTTGGAATCCATCGATACTGTTAAGTTCCACGCTGATACTTGGACCCGTGGTAACGATGGT
GGTGGTGGTACCTCTATGGTTATCCAAGACGGTACAACTTTCGAAAAAGGTGGTGTTAATGTCTCCGTTGTTTAT
GGTCAATTGAGCCCAGCGGCCGTTTCAGCCATGAAAGCTGATCATAAGAATCTGCGTCTACCAGAAGATCCAAAG
ACTGGTTTGCCAGTTACCGACGGTGTCAAGTTCTTCGCTTGTGGTTTAAGTATGGTCATTCATCCCGTTAACCCA
CACGCTCCAACCACGCACTTAAACTACCGTTACTTCGAAACTTGGAACCAAGATGGGACCCCACAAACTTGGTGG
TTTGGTGGTGGTGCTGATTTGACACCTTCTTACTTATACGAAGAAGACGGTCAATTATTCCACCAACTGCACAAG
GATGCCTTGGACAAGCACGACACTGCTTTGTACCCACGTTTCAAGAAATGGTGTGATGAGTACTTCTACATTACC
CACCGTAAGGAAACACGTGGTATTGGTGGTATATTCTTTGACGATTATGATGAACGTGACCCACAAGAAATATTG

AAGATGGTTGAAGACTGTTTCGATGCTTTCTTGCCATCCTACTTGACTATCGTCAAGAGAAGAAAAGATATGCCA
TATACAAAGGAAGAACAACAATGGCAGGCCATTAGACGTGGTAGATACGTTGAATTCAACTTAATCTACGATAGA
GGTACCCAATTCGGTTTGAGAACCCCAGGCTCTAGAGTTGAGTCAATTTTGATGAGTTTGCCTGAACATGCTTCA
TGGTTATACAACCACCACCCTGCTCCTGGTTCCAGAGAAGCTAAATTACTAGAAGTTACCACCAAACCAAGAGAG
TGGGTTAAATAA

>YPL067C 1.79 Hot
ATGCAACAAGATATCGTCAACGATCACCAGGAGGAGGCCCAAGGGTGGAAGTGGGAACAAATCAAAGAGATAATT
GAATCGGGGGAGCTTGCCCGTTTGAAACGAAGTCGCCAGATGACTGACAAATACCACGAGCATAAGAAAAGGACT
GCTGGCCTGGACATGAACCAGTACGTTTTGCAAAAACTAGGCTGGTCGCTAGACGAACCGCAGCTCGAAAATGCT
GCCGCGAAGGCTTTTAGTTCTTCTACTCTATATGCTGTGCGTGCCAACGATTTCCCGTATAACTTCGAGCCTGGT
GTTGTACACCTTGTTCTCTGGTCCAAGGTTGCCCTGCCAGTACATTCCCCAGATAAGGCCGTGCGCGAAGCGGCC
CGCGCTCGCATGAATGCATTTTTGCAGGCACAGCCGCTACTGCGTCCACTTCTAAGCTCGGGTCACGTGGCCTGG
TTTGTCAACTACCCGGAACTACAAAGTGTTGCCCGGATTTTCCACGCTCACGTGCTGCTTTTCTTCCCACGGGAA
CGCTATTCCGCGGAACAGGTAAAAACGACCGTTGATGATATCCTTTCACACGGTTTCGAGCCACTTGCCTGA

>YBL032W 1.85 Hot
ATGTCACAGTTCTTCGAAGCTGCTACTCCCGTTGCAATTCCCACAAACAATACCAACGGCGGCTCCAGTGATGCC
GGCAGCGCCGCCACTGGCGGCGCCCCCGTTGTTGGCACCACCGCTCAACCCACCATCAATCACAGGCTTTTGCTG
TCATTGAAAGAGGCTGCCAAGATCATTGGCACTAAGGGCTCCACCATCTCACGCATAAGAGCTGCAAACGCCGTC
AAGATCGGTATTTCTGAAAAGGTGCCCGGTTGCTCTGACAGGATCCTGTCCTGTGCTGGGAACGTAATCAATGTG
GCCAATGCCATTGGTGATATTGTTGACGTGCTTAACAAACGGAATCCCGAAAATGAGGACGCAGCTGAGGGCGAA
GCGGAAGAGCACTACTACTTCCACTTTTTGAACCATATTTTACCAGCTCCCTCAAAGGACGAGATCAGAGATCTG
CAGCAACTGGAGGACATCGGTTATGTGAGGCTCATTGTGGCCAATTCCCATATCTCATCGATTATCGGGAAAGCA
GGCGCCACCATCAAGTCCCTGATCAATAAGCACGGCGTTAAGATCGTGGCTTCCAAGGACTTCTTACCTGCTAGC
GACGAGAGAATTATCGAGATCCAGGGTTTCCCAGGATCCATCACCAATGTACTTATCGAAATTAGCGAGATCATC
TTGAGTGACGTTGACGTCAGATTCAGCACAGAAAGATCTTATTTCCCTCATCTGAAAAAGTCCTCCGGTGAGCCA
ACTTCCCCTTCTACCTCATCTAACACTAGGATCGAATTGAAGATTCCAGAACTGTATGTAGGCGCCATTATTGGC
CGTGGAATGAACAGAATTAAGAATTTGAAAACTTTCACAAAAACCAATATTGTCGTGGAAAGGAAGGATGACGAT
GATAAAGACGAAAATTTTAGAAAATTCATAATCACAAGTAAATTTCCTAAGAATGTCAAACTTGCTGAGTCCATG
CTTTTGAAGAACCTGAATACTGAAATTGAGAAACGTGAAAACTACAAGAGAAAATTGGAAGCTGCCGAAGGAGAT
GCCACTGTTGTTACTGAACGCTCTGATTCTGCTTCTTTCTTGGAAGAGAAGGAAGAACCTCAAGAGAATCATGAT
AACAAAGAGGAGCAGTCGTAG

>YCL034W 1.92 Hot
ATGGGGTTTCTTTCGGATCATCCGCATACAGCTATCACCGAGACGATCTTTCGAATTGTCTCTTCAAGGGACTAT
ACGCTAGAAGTGGAGTTAGCTCCCTTGATTCAGCTTATCAAAGCAGATCACAACGATTATAACTATACCGTGAAC
CAAGAAGAAGCTGCGCGAGCACTTAGAAAGAAAATAAAGTATGGGAACCGGCTGCAGCAGTCCAGGACTCTGGAC
CTCTTGGATCTGTTCATCTCACAAGGCGTGAAGTTTACAGTCATGTACAATGATGACAAGCTGCTGCAAAGGCTG
AGAGGGATGGCTACGAACTCTGAAAACAGCGGGTCCGGTGAAAAGTATGAGCCTAGGATTATTAAGAAGTGCGCG
GCGTATGCTATCTCGTGGCTAAACTATATTACGCAGAACAACCTTGAGAATGCGAGGGCGTATTCAGGTCTTTAT
CAATTGGGTCAAACAGTCAAACAGAGGTACTCGAAGAGCAGCCGTTCCCGCCGTAGCGGCGGAGGCTCTGGCGGA
CGGTCTAACTTCATGGATGATAGCGCCGACGACACTTTGTACCAGTCCAACTCGCTGACTAGTGCCGATAGATTG
TATAGGATTCCACAGATCAACATGAACAAAGAGGCACCCAGGATTCGGCTCATAATCAGCGATGCTCTCGCGTCT
GCTGTGTCGTTACAGAACTCGCTGATCGGGCTGCCCAAGGGCAAGTTCTCCACAGACGATGAAGAGGCCACGTCC
AAGTTCATCCAGGCAAGGGCGATCAGAAGGAAAGTTCTTAGATATCTGCAATTGGTCACGGAGGGTGAGTTTCTG
GGTAGCTTAATTCATGCCAATGATGAACTGGTGGCTGCGTTGACTGCATACGACGACCGAAGCGCTCAGGACGAT
AGCAGCGATGAAAGTGATCACGGCTCGTACGATGACGGTATTTATGATGAAAACGAGCAGGATAACAGCAGGTAT
ATTGATAGCGAGTCCTCAGAGGAAGAGAGCTTGTCTTCTTATCAGCCGTCCACGATCTCAAACCCTTTCGGTGAT
CATAACAAAATTTGA

>YLR099C 1.54 Hot
ATGTGGACAAACACTTTCAAATGGTGCAGCAAAACTGAGAAGGAGACCACTACTGCAGACGCCAAGGTGTGTGCT
AGCGTGCAGGGCCTGAAGGCCCTGCAGCAGCAAATTATGGATAGCACCACTGTGCGTGGGTCCGTTAATAACACG
ATGACTCCAGGCGGGATTAACCAGTGGCATTTCCACAATAAGCGTGCGAACAAAGTGTGCACGCCTACAGTACTA
ATCCATGGATACGCTGCCTCGTCGATGGCGTTCTACAGGACGTTTGAGAACCTGTCAGACAACATTAAAGATCTA
TATGCCATCGACTTGCCAGCCAATGGTGCCTCAGAGGCGCCAGCATTGCAAGTGAACAAAACCAAGAAGATCAAG
TCTCTCAGGTTTAAGCATATAGAAGATGACGTAGTAATCCCTGTGATAGAGAAGCGCCCCCCAGCAGAGGACATC
AAGTCACACCTGGAGCAGTACGAAAGCTACTTCGTAGACAGGATAGAGCAATGGCGGAAGGATAACAAGCTTCGC
AAAATAAACGTGGTGGGCCATTCATTCGGAGGATATATTTCATTCAAATACGCCCTCAAGTACCCTGATTCCATT
GAGAAACTGTGTCTCATATCTCCTCTTGGTGTGGAAAACAGTATACATGCTATCACCCACAAATGGGAGCCCAAC
ACCACCTATCCACTTACGTTTACCGACCCATCGTCCCGATATTATACAAGAAAGCTGAACGTACCGCGGTTTATC
TTTGAAAACCAGCTAAACGTTTTGAAGTGGATGGGACCCATAGGCTCCAAATTGTGCTCGAACTACATCTCCACC
GCATACGTAAAAGTTCCGGATCAGATATATAAGGACTATCTTTTGCACTCATTTGTCGGGAAGAACCAGACGGTG
CAACCACAGACAATCAAGGTGTTCACCCATTTGTTTGAAAGGAATCTGATCGCTAGAGATCCGATAATAAACAAC
GTTCGTTTTTTGAACCCAGCAACGCCTGTCATGTTCATGTATGGCGAGCACGATTGGATGGATAAATATGCGGGC

TACTTGACTACTGAATCAATGCTAAAAAACAAGGCAAAGGCTAGCTACGTTGAAGTCCCTGATGCTGGCCATAAT
CTTTTCCTGGACAACCCGCAGCACTTTGCCTCTTCTTTAGTCTCGTTCCTGTCAAAGTAA

>YGL162W 2.17   Hot
ATGTCCACAAGCATTACAGTAAGAAATAGAGATCGATCGCTACCACCGCTATTGCTCCCCAATGTTTCCCTGCTA
GAAAAAGATATACGGCGTAAAGGCACCCAAAATGTGGGCATCACCGATCCAGAACTCTTGTCGACCACTTGGACG
AGGAAGCGGGCTTTTCCGACTGACGAGCTTTTAGGAGGCTATAAGAGATTAAAGCCTGCTGCCGCTGACAGTAAT
GAGTGCGCTATTGGTATTGCCACGGTGACGCCGCCGCCAACGCTCCCCGTAAGTGCGATTGTTCCCCCTCCACAA
AATTACACTCCACCATTGTTTGAGTATCATCCTCATGCTCTTGCTTCCATGGTCAATGAAGACGCTAATGCGTCA
TGCACTCAAATGTCTATAATTTCGCGCTCAACGAGCAACTCGACAACCTCCTCTGCCACATCTACTAGTTCAATT
TCCAAGAGACAAAGAAGTGGCCCAAGTTGTGACAAATGTCGTTTGAAAAAAATAAAATGTAATGCGAAAATTGAG
ATTTTGCTTCAAGACGATACTATAATGCCAATGATCTCGAACAAGTTGCGGTACGTCTTGACTCCTGACGATATT
CGGCTATATCGAGGCACGCTGTTGCGGAATATTGCCATACCGGATGATGTCATTGAGGGTACAGGCTCACGCAAG
TTGATTAAGCATATTGATAAGTTGGTTTTGCTCACACCTTGTTTACCATGCATTAAGAAAAAGCATTCTTCTTCT
TCCACTAATTTCCCAAAAAATGATAAATGTACTTTTTCAAAAGGCTTTACCAGAGCTGACATAAACATTTCATCC
AAAATCTCCTTAAAATTTAAGGATAAAACCATTTACGACATAACCTATGATGACTATAAAAGCATTGATTTTTAG

>YIL101C 1.8Hot
ATGAAATATCCCGCTTTTAGCATTAACAGTGACACGGTACACTTAACGGACAACCCCCTGGACGACTATCAGCGT
CTCTACCTCGTCAGCGTACTGGATAGGGACTCGCCGCCGGCCAGTTTCAGCGCCGGACTCAATATTAGAAAGGTT
AACTATAAATCCTCCATTGCTGCGCAGTTCACACATCCAAACTTCATTATCAGTGCTCGGGACGCAGGGAACGGG
GAAGAGGCGGCGGCGCAGAACGTTCTGAACTGCTTCGAGTACCAGTTTCCCAACCTACAAACAATTCAGAGCCTA
GTCCACGAACAAACGTTGCTATCGCAGCTCGCTAGCTCCGCCACTCCTCACTCCGCCTTGCATCTGCACGATAAA
AATATTCTGATGGGTAAGATCATACTACCTTCCCGGTCTAACAAAACACCAGTCTCAGCATCACCCACCAAGCAA
GAAAAGAAGGCTCTGTCAACAGCATCGCGTGAAAATGCCACCTCCTCGCTGACAAAGAATCAGCAGTTCAAGTTG
ACCAAAATGGACCATAATCTGATCAATGACAAGCTCATCAACCCTAACAACTGCGTAATATGGTCGCATGACTCC
GGCTATGTGTTCATGACGGGCATCTGGCGGCTGTATCAGGATGTTATGAAGGGGCTTATAAACTTGCCCAGAGGT
GACAGCGTTTCCACTAGCCAACAGCAGTTCTTTTGCAAGGCTGAATTCGAGAAAATTCTATCTTTTTGCTTTTAC
AATCACAGTTCGTTCACTTCTGAGGAATCTTCAAGCGTACTGTTGTCGTCCTCCACCTCTTCGCCGCCAAAGAGA
CGGACATCCACAGGGTCTACCTTCTTGGACGCCAATGCGTCTTCTTCATCTACTTCTTCAACGCAGGCAAACAAT
TACATCGATTTTCATTGGAACAACATCAAGCCCGAACTGCGGGACCTTATTTGCCAGTCTTACAAAGACTTCCTG
ATCAATGAACTTGGTCCTGACCAAATAGACTTGCCCAACTTAAATCCGGCAAATTTTACGAAAAGAATAAGAGGT
GGTTACATCAAAATTCAGGGTACTTGGTTGCCGATGGAAATCTCAAGGTTGCTATGCCTGCGGTTTTGTTTTCCG
ATCAGATACTTTCTGGTGCCTATTTTTGGTCCAGATTTCCCCAAAGATTGCGAATCGTGGTATTTGGCTCATCAA
AACGTTACATTTGCTAGTTCTACCACTGGTGCAGGTGCTGCTACTGCTGCTACTGCTGCTGCTAATACAAGTACC
AATTTTACGTCTACTGCAGTGGCAAGACCTAGGCAGAAGCCCAGACCCAGGCCAAGACAAAGATCTACTTCCATG
TCCCATTCCAAAGCGCAGAAACTTGTTATTGAGGACGCCTTGCCCTCATTCGACTCATTTGTAGAAAACTTGGGC
CTTTCCTCAAATGACAAGAACTTCATTAAAAAAAAACAGCAAAAGGCAAAAGTCGTCCACATACACCTCTCAAACT
TCTTCTCCAATAGGGCCAAGAGATCCAACGGTGCAAATTTTGTCAAACCTAGCATCCTTCTACAACACCCACGGT
CACCGGTATTCATATCCGGGAAATATCTATATACCACAGCAAAGATACTCTTTACCCCCACCAAACCAGCTCTCG
TCGCCACAGCGGCAGCTAAACTATACTTACGACCACATTCACCCAGTTCCTTCTCAGTACCAGTCTCCAAGACAC
TACAACGTCCCCTCTTCACCAATCGCGCCTGCTCCTCCCACTTTCCCTCAACCTTATGGTGATGATCACTATCAT
TTTTTGAAATATGCTAGCGAAGTTTATAAGCAACAAAACCAACGACCAGCTCATAATACAAATACCAATATGGAC
ACCTCTTTTTCGCCAAGGGCGAACAATTCACTGAACAATTTCAAATTTAAAACAAACTCAAAACAATAA

>YLR081W 1.61   Hot
ATGGCAGTTGAGGAGAACAATATGCCTGTTGTTTCACAGCAACCCCAAGCTGGTGAAGACGTGATCTCTTCACTC
AGTAAAGATTCCCATTTAAGCGCACAATCTCAAAAGTATTCTAATGATGAATTGAAAGCCGGTGAGTCAGGGTCT
GAAGGCTCCCAAAGTGTTCCTATAGAGATACCCAAGAAGCCCATGTCTGAATATGTTACCGTTTCCTTGCTTTGT
TTGTGTGTTGCCTTCGGCGGCTTCATGTTTGGCTGGGATACCGGTACTATTTCTGGGTTTGTTGTCCAAACAGAC
TTTTTGAGAAGGTTTGGTATGAAACATAAGGATGGTACCCACTATTTGTCAAACGTCAGAACAGGTTTAATCGTC
GCCATTTTCAATATTGGCTGTGCCTTTGGTGGTATTATACTTTCCAAAGGTGGAGATATGTATGGCCGTAAAAAG
GGTCTTTCGATTGTCGTCTCGGTTTATATAGTTGGTATTATCATTCAAATTGCCTCTATCAACAAGTGGTACCAA
TATTTCATTGGTAGAATCATATCTGGTTTGGGTGTCGGCGGCATCGCCGTCTTATGTCCTATGTTGATCTCTGAA
ATTGCTCCAAAGCACTTGAGAGGCACACTAGTTTCTTGTTATCAGCTGATGATTACTGCAGGTATCTTTTTGGGC
TACTGTACTAATTACGGTACAAAGAGCTATTCGAACTCAGTTCAATGGAGAGTTCCATTAGGGCTATGTTTCGCT
TGGTCATTATTTATGATTGGCGCTTTGACGTTAGTTCCTGAATCCCCACGTTATTTATGTGAGGTGAATAAGGTA
GAAGACGCCAAGCGTTCCATTGCTAAGTCTAACAAGGTGTCACCAGAGGATCCTGCCGTCCAGGCAGAGTTAGAT
CTGATCATGGCCGGTATAGAAGCTGAAAAACTGGCTGGCAATGCGTCCTGGGGGGAATTATTTTCCACCAAGACC
AAAGTATTTCAACGTTTGTTGATGGGTGTGTTTGTTCAAATGTTCCAACAATTAACCGGTAACAATTATTTTTTC
TACTACGGTACCGTTATTTTCAAGTCAGTTGGCCTGGATGATTCCTTTGAAACATCCATTGTCATTGGTGTAGTC
AACTTTGCCTCCACTTTCTTTAGTTTGTGGACTGTCGAAAACTTGGGACATCGTAAATGTTTACTTTTGGGCGCT
GCCACTATGATGGCTTGTATGGTCATCTACGCCTCTGTTGGTGTTACTAGATTATATCCTCACGGTAAAAGCCAG
CCATCTTCTAAAGGTGCCGGTAACTGTATGATTGTCTTTACCTGTTTTTATATTTTCTGTTATGCCACAACCTGG
GCGCCAGTTGCCTGGGTCATCACAGCAGAATCATTCCCACTGAGAGTCAAGTCGAAATGTATGGCGTTGGCCTCT
GCTTCCAATTGGGTATGGGGGTTCTTGATTGCATTTTTCACCCCATTCATCACATCTGCCATTAACTTCTACTAC
GGTTATGTCTTCATGGGCTGTTTGGTTGCCATGTTTTTTTATGTCTTTTTCTTTGTTCCAGAAACTAAAGGCCTA

TCGTTAGAAGAAATTCAAGAATTATGGGAAGAAGGTGTTTTACCTTGGAAATCTGAAGGCTGGATTCCTTCATCC
AGAAGAGGTAATAATTACGATTTAGAGGATTTACAACATGACGACAAACCGTGGTACAAGGCCATGCTAGAATAA

>YOL052C 2.29 Hot
ATGACTGTCACCATAAAAGAATTGACTAACCACAACTACATTGACCACGAACTATCAGCCACTTTAGACTCAACG
GATGCGTTCGAGGGTCCCGAGAAGTTGCTGGAAATCTGGTTCTTCCCTCACAAGAAGTCCATCACGACCGAAAAG
ACATTAAGAAATATTGGCATGGATAGATGGATCGAGATTTTGAAATTAGTGAAATGCGAAGTTCTTTCCATGAAG
AAGACTAAAGAACTGGATGCCTTTTTGTTGAGTGAGTCTTCCCTCTTCGTCTTCGATCACAAATTGACGATGAAG
ACGTGCGGTACTACAACCACATTGTTCTGTCTCGAAAAGCTTTTCCAGATCGTTGAGCAAGAGTTATCGTGGGCT
TTCCGCACAACACAAGGGGGCAAGTACAAACCATTTAAAGTGTTTATTCTAGACGATGTTTCCTTTTCCCCTGT
AAGCAAGCCGCTATCCATCAAAACTGGGCTGACGAAGTCGACTATTTGAACAAATTTTTCGACAATGGTAAAAGT
TATTCCGTGGGAAGAAATGACAAGAGCAACCACTGGAACCTGTACGTCACCGAGACGGACCGCTCCACACCTAAG
GGAAAGGAGTACATCGAGGATGACGACGAAACTTTCGAAGTACTGATGACGGAGCTGGACCCAGAATGCGCTAGT
AAGTTTGTTTGCGGGCCTGAGGCATCCACAACCGCTCTCGTGGAGCCAAACGAAGATAAGGGCCACAACCTCGGC
TACCAAATGACTAAAAATACAAGGCTTGACGAAATATATGTCAACTCGGCCCAAGACTCCGATTTATCATTTCAC
CACGATGCATTTGCGTTCACGCCATGTGGATACTCATCCAATATGATTCTCGCTGAAAAATACTATTACACCCTG
CACGTGACTCCGGAAAAGGGTTGGTCTTACGCCTCTTTCGAAAGTAACATACCCGTATTTGACATTTCCCAAGGG
AAGCAAGACAACTTGGACGTTCTTCTACATATTCTGAACGTTTTTCAACCAAGAGAGTTCTCGATGACCTTTTTT
ACCAAAAATTATCAGAACCAATCCTTCCAAAAACTACTAAGCATCAACGAGTCACTGCCCGACTACATCAAGTTA
GACAAAATTGTTTATGATCTGGACGACTACCACCTTTTCTATATGAAATTGCAGAAGAAAATATGA

>YPL037C 1.82 Hot
ATGCCAATTGACCAAGAAAAATTAGCTAAGCTACAAAAGTTGTCTGCTAACAACAAAGTTGGTGGTACTAGAAGA
AAGCTTAACAAGAAGGCAGGCTCTTCTGCCGGTGCCAACAAGGATGACACCAAGTTGCAAAGTCAATTAGCTAAG
TTGCACGCTGTCACCATTGACAACGTCGCCGAAGCCAACTTTTTCAAGGACGACGGTAAGGTCATGCACTTCAAC
AAGGTCGGTGTCCAAGTTGCTGCTCAACACAACACTTCTGTATTCTACGGTCTACCACAGGAAAAGAACTTGCAA
GATTTGTTCCCAGGTATTATCTCTCAATTGGGCCCTGAAGCCATCCAAGCCTTGTCTCAATTGGCTGCCCAAATG
GAAAAGCACGAAGCCAAGGCTCCAGCTGATGCTGAAAAGAAGGATGAAGCTATTCCAGAGTTAGTTGAAGGTCAA
ACTTTTGATGCTGACGTCGAATAA

>YER034W 1.5 Hot
ATGGATGCATTCAGCTTAAAGAAGGATAATCGAAAAAAATTTCAAGATAAACAGAAATTGAAAAGAAAACATGCC
ACACCCAGTGATAGAAAGTACCGGCTATTGAACCGCCAAAAAGAAGAGAAAGCTACCACAGAGGAGAAAGATCAA
GACCAAGAACAGCCCGCCCTGAAGTCAAACGAGGACAGGTACTATGAGGACCCGGTACTCGAGGACCCGCATTCT
GCAGTCGCCAATGCAGAGTTGAACAAGGTGCTAAAAGACGTCCTCAAAAATCGGCTCCAGCAGAACGACGACGCC
ACAGCCGTCAATAATGTTGCTAATAAAGATACTTTGAAAATCAAAGACCTCAAGCAGATGAATACGGATGAGCTC
AATCGTTGGCTCGGACGGCAGAATACAACATCGGCTATAACAGCGGCTGAGCCCGAATCATTAGTCGTTCCCATT
CACGTACAAGGTGATCATGATCGTGCGGGCAAGAAGATCAGTGCCCCTTCGACCGATCTACCGGAAGAACTAGAG
ACCGATCAGGATTTCCTTGATGGACTGCTCTAA

>YJL101C 1.98 Hot
ATGGGACTCTTAGCTTTGGGCACGCCTTTGCAGTGGTTTGAGTCTAGGACGTACAATGAACACATAAGGGATGAA
GGTATCGAGCAGTTGTTGTATATTTTCCAAGCTGCTGGTAAAAGAGACAATGACCCTCTTTTTTGGGGAGACGAG
CTTGAGTACATGGTTGTAGATTTTGATGATAAGGAGAGAAATTCTATGCTCGACGTTTGCCATGACAAGATACTC
ACTGAGCTTAATATGGAGGATTCGTCCCTTTGTGAGGCTAACGATGTGAGTTTTCACCCTGAGTATGGCCGGTAT
ATGTTAGAGGCAACACCAGCTTCTCCATATTTGAATTACGTGGGTAGTTACGTTGAGGTTAACATGCAAAAAAGA
CGTGCCATTGCAGAATATAAGCTATCTGAATATGCGAGACAAGATAGTAAAAATAACTTGCATGTGGGCTCCAGG
TCTGTCCCTTTGACGCTGACTGTCTTCCCGAGGATGGGATGCCCCGACTTTATTAACATTAAGGATCCGTGGAAT
CATAAAAATGCCGCTTCCAGGTCTCTGTTTTTACCCGATGAAGTCATTAACAGACATGTCAGGTTTCCTAACTTG
ACAGCATCCATCAGGACCAGGCGTGGTGAAAAAGTTTGCATGAATGTTCCCATGTATAAAGATATAGCTACTCCA
GAAACGGATGACTCCATCTACGATCGAGATTGGTTTTTACCAGAAGACAAAGAGGCGAAACTGGCTTCCAAACCG
GGTTTCATTTATATGGATTCCATGGGTTTTGGCATGGGCTGTTCGTGCTTACAAGTGACCTTTCAGGCACCCAAT
ATCAACAAGGCACGTTACCTGTACGATGCATTAGTGAATTTTGCACCTATAATGCTAGCCTTCTCTGCCGCTGCG
CCTGCTTTTAAAGGTTGGCTAGCCGACCAAGATGTTCGTTGGAATGTGATATCTGGTGCGGTGGACGACCGTACT
CCGAAGGAAAGAGGTGTTGCGCCATTACTACCCAAATACAACAAGAACGGATTTGGAGGCATTGCCAAAGACGTA
CAAGATAAAGTCCTTGAAATACCAAAGTCAAGATATAGTTCGGTTGATCTTTTCTTGGGTGGGTCGAAATTTTTC
AATAGGACTTATAACGACACAAATGTACCTATTAATGAAAAAGTATTAGGACGACTACTAGAGAATGATAAGGCG
CCACTGGACTATGATCTTGCTAAACATTTTGCGCATCTCTACATAAGAGATCCAGTATCTACATTCGAAGAACTG
TTGAATCAGGACAACAAAACGTCTTCAAATCACTTTGAAAACATCCAAAGTACAAATTGGCAGACATTACGTTTT
AAACCCCCCACACAACAAGCAACCCCGGACAAAAAGGATTCTCCTGGTTGGAGAGTGGAATTCAGACCATTTGAA
GTGCAACTATTAGATTTTGAGAACGCTGCGTATTCCGTGCTCATATACTTGATTGTCGATAGCATTTTGACCTTT
TCCGATAATATTAACGCATATATTCATATGTCCAAAGTATGGGAAAATATGAAGATAGCCCATCACAGAGATGCT
ATCCTATTTGAAAAATTTCATTGGAAAAAAATCATTTCGCAACGACACCGATGTGGAAACTGAAGATTATTCTATA
AGCGAGATTTTCCATAATCCAGAGAATGGTATATTTCCTCAATTTGTTACGCCAATCCTATGCCAAAAAGGGTTT
GTAACCAAAGATTGGAAAGAATTAAAGCATTCTTCCAAACACGAGAGACTATACTATTATTTAAAGCTAATTTCT
GATAGAGCAAGCGGTGAATTGCCAACAACAGCAAAATTCTTTAGAAATTTTGTACTACAACATCCAGATTACAAA
CATGATTCAAAAATTTCAAAGTCGATCAATTATGATTTGCTTTCTACGTGTGATAGACTTACCCATTTAGACGAT

TCAAAAGGTGAATTGACATCCTTTTTAGGAGCTGAAATTGCAGAATATGTAAAAAAAAAATAAGCCTTCAATAGAA
AGCAAATGTTAA

>YIL152W 1.69   Hot
ATGAGTCACAAGAGAAGGGGCCTGGTAATATATCAGGATCAGAAGCAGCAACAACAACACCCCCCGGGGCAGTCC
CTAAGCTCTATATCCTGGTCTCCAACCAGGCGACCTCATCACCCTTTAAAACAACAAAGCACCAACAGTTTCTCA
GAAATACTATCGAAATCAAGTGTGCAACCGAACGTGCAACATGACGGTAATCATATGCCCATTTCATTGCTAGTT
CTGAAACAAGAACATCATAAGCAGCAGCAGCAGCAGCAGCAGCGCCAGAATATCCGATCACAAAACTCTACGCCT
CCCCTGCGACAGTTGGTTCAGGAGTCGCAGTGGACGAGCTCCGCAAGTAATAGTTCTCTAAAGAAACAGGAAAAG
CAGCCGCAAACATTCTACAACACTGACTCTAAACTTGTTTCTCAACTGCATAGCTCCGTGAAGGACCTGGATGCC
ATAATCCAGACGCATAAACCAAAGTTTGACACAATTATTCGCGATTTCTCTCAAGCAACCATACTGTCCTCAAAC
GAGTTACTCATCAAACTTCCGAAGGACCAAACAATAATATTGCATAGCAGAGCGCCAAAAATTAACGCAGAATGG
CTACAGAACAAGGTTAATGACCCTAGTGCCTCGTTAGTGATCGATTCAAGATCTTTTCTAACCTTGTGTAACAAT
ATAAAATGGTACCTGCATTGGAAATTTATATGA

>YCR079W 1.75   Hot
ATGCGGCTGGGGAATGCTTATGCGTATTGCAAGCCGTCGCAAAATGTTGGGCTGAAGCTGGACTTGTTACGGGGT
CTTCCAGGGTACGTGGGTCACGCCACGAGTAGGATCAACCGGTTAGAGAACCAGGACAATTACTCCATTAAAATG
ATGCGGTCGTGGCCTAATGCGTATGGAAGTGCCCTGAACTGCTCTGTATTCGATGGCCATGGGGAGAAAGGTGCG
CAACTGTCACAGTTGTTAGCCGATAAATTGTGCAGTAGCCTGGATTTCCCTGAACCCTCCTGGGACAAGCAGGAC
TTGAAGAAGTTGGTACAGGAATATGCAAGAAGGTTTCCCGAGGGAAATTACTGGAAACATAAGCTGTCCACGTTT
GAAAAATTCTACAACAAGTTTATAAAGAACTGCAACTCGAAACAAGAACTGCTGCTGATGAAGGAGGGCGATAGT
GCGATCCTTGGACAAAATGGTGGCAGGATGATTTTTGATAAAATGGGGAATATTATTGACAAAATTGCACTGCTC
ACTGAGCTAGACCGTCTGCGATTGTTCTACGGGTTTGCCAGGTTCGATCTCGACCAATGCTGCGGCCTGGGCACC
GCAGCGGGGTCCACAGCGTCGTCCATATTTCTATATCCGTATGATGATCCGAATGCACCCATTGATGAGGGGAAA
GACGATGACTCGTGGATCATATCGCACTCTGGATTGCTCAAACTCATTGTCACGCAGGTGGGCGACTCTAAGATC
ATCCTCTGCGATCAAGACGGGATTGCACACGCATTAACAACAACTCACCACATCAATTCCAGTAGGGAACGACAC
CGTTTGAGCATAGACCCTTCTCGATTGGACCCAGACGCCTTTGGAGAGACAAGGTTTTTGAATAATTTCGCCAAC
ACTAGATCGTTTGGTGATGTCGCTGGGAAACCGTATGGTATATCTAGTGAGCCGGATATTTTCTCGTTTCTTGTC
GGAAATACCTTGCATTTGCCGCGATCTGAAAGGTCGAAGCTGCCCTTCAATGGTGATGAATGCTTCCTGGCGCTT
GTCACCGACGGCATCACCAATAAACTTGCAGACCAAGAGGTTGTTGACCTCATCACCTCTACGGTGAACTCCTGG
GGACTGAAAAAGGCGACTCCTCAATTTGTTGCAGAGGAGACAATCAAATTTATTCAGGCAATAGCCACTAAACAC
TCAGACAACGCCACTTGCGTGGTGGTTAGGCTTTCCAATTGGGGCAACTGGCCCAATGTTGACAGAACTGGCCCC
CAAAGAGAAACCAAACTAATGAATGCACAATCTAACGAAACAAAATTAAACTAA

>YDR188W 2.61   Hot
ATGTCATTGCAATTGTTGAATCCGAAGGCTGAATCGTTGAGAAGGGATGCGGCTTTGAAGGTTAACGTCACATCT
GCTGAGGGCTTACAATCCGTCCTAGAGACCAACTTGGGCCCTAAGGGCACGCTAAAGATGCTTGTGGATGGTGCT
GGTAACATCAAGCTGACCAAGGACGGTAAAGTGTTGCTGACCGAGATGCAGATCCAGTCGCCTACAGCGGTACTG
ATTGCTAGGGCAGCTGCCGCGCAGGATGAAATCACGGGCGATGGGACCACGACGGTTGTGTGTCTTGTGGGTGAA
TTGCTTAGACAAGCGCACCGTTTCATTCAAGAGGGCGTGCATCCGCGGATCATCACTGATGGGTTTGAGATTGCG
CGGAAGGAGTCCATGAAATTTTTGGACGAATTCAAGATCAGTAAGACGAACCTTTCCAATGATAGAGAGTTCCTT
CTGCAAGTGGCCCGGTCGTCGCTGCTAACCAAGGTGGACGCTGACTTGACGGAGGTCTTGACGCCCATTGTAACG
GATGCGGTGCTAAGCGTGTACGACGCGCAGGCTGACAATTTGGATTTACACATGGTTGAGATCATGCAGATGCAG
CACTTGTCTCCTAAAGATACCACTTTTATTAAGGGTTTGGTATTGGACCACGGTGGTAGGCATCCTGACATGCCC
ACCCGTGTGAAGAACGCGTATGTTTTGATTTTGAACGTATCTTTGGAATACGAGAAGACTGAAGTTAACTCTGGG
TTCTTTTACAGTTCTGCGGACCAAAGGGACAAGTTGGCCGCTAGTGAGAGGAAGTTTGTGGATGCCAAGTTGAAG
AAGATCATCGACTTGAAAAACGAAGTTTGTGGCATGGATCCAGACAAGGGTTTTGTTATCATTAACCAAAAAGGC
ATTGACCCCATGTCTTTAGACGTGTTTGCCAAACACAACATCTTGGCTTTGAGAAGGGCCAAGAGACGTAACATG
GAAAGATTGCAATTGGTCACTGGCGGTGAAGCTCAGAACTCTGTGGAAGACTTGTCGCCTCAGATTCTTGGGTTT
TCTGGCTTGGTCTACCAAGAAACCATAGGCGAGGAAAAATTCACATACGTTACAGAGAACACTGACCCCAAGTCT
TGCACCATCTTAATCAAGGGCTCCACTCATTATGCCCTCGCTCAAACAAAGGATGCGGTGAGAGATGGTCTCAGA
GCTGTGGCAAACGTTCTCAAGGACAAAAACATCATTCCAGGCGCTGGTGCGTTCTACATCGCCCTTTCGAGATAC
CTAAGATCTGCCAACATGAACAAGTTGGGTGCCAAGGGTAAAACAAAGACAGGTATTGAGGCGTTCGCAGAAGCA
TTGCTGGTAATTCCAAAGACTTTGGTGAAGAACTCAGGATTCGACCCATTGGACGTGCTCGCAATGGTGGAGGAC
GAGTTGGATGACGCTCAGGATTCTGACGAAACGAGATATGTTGGTGTGGACTTGAACATAGGTGATTCTTGCGAC
CCTACCATCGAGGGTATTTGGGATTCCTACCGCGTACTAAGAAATGCTATTACCGGTGCTACAGGTATTGCCAGC
AACTTGTTATTATGCGATGAATTGTTAAGGGCAGGTAGATCCACTTTGAAAGAAACACCACAGTAA

>YJL055W 1.69   Hot
ATGACAATGGAAAAAAAATGGAGGTAATAGCAGCCGTGGTGGCCAAGTAGGCGGCAAGTCTGTGTGTGTTTACTGC
GGGTCTTCATTTGGCGCTAAGGCGCTATACTCAGAAAGTGCAGAAGAATTAGGAGCCCTTTTCCATAAGCTGGGA
TGGAAATTGGTATACGGTGGAGGCACTACTGGTTTGATGGGCAAGATAGCAAGGTCTACGATGGGACCTGATTTA
AGCGGACAGGTTCACGGTATCATTCCAAATGCACTTGTGTCTAAGGAAAGGACAGACGAGGATAAAGAAGATGTT
AATAAAGCATTGTTGGAGTCTGTAGAAAATCATAAGGGCGCCACTCCTATTTCTGAAGAGTATGGGGAAACAACG
ATTGTACCAGATATGCATACGAGAAAAAGAATGATGGCAAATTTGAGTGACGCGTTTGTTGCTATGCCTGGTGGA
TACGGGACTTTTGAAGAAATCATGGAATGTATCACGTGGTCGCAACTGGGGATTCATAATAAACCAATTATCTTG

TTCAATATCGATGGGTTCTATGACAAATTATTGGAGTTCCTCAAACACTCTATTCAAGAACGGTTCATCAGTGTG
AAGAATGGTGAAATCATTCAAGTTGCCTCCACTCCGCAGGAAGTTGTTGATAAAATAGAGAAGTACGTCGTTCCA
GAGGGCCGTTTCAATTTGAATTGGAGCGACGAAGGTCACGCTCACGAGGATTGTGCTAAATAA

>YDR476C 1.67   Hot
ATGTGGGATTCGTTAATTGTAAGCATCAACGATACGCACAAGTTGGGGCTGGAGGATTGTCTCGCCGTTTTTGGC
CACGTTCCCATTACCAAGGCCGTCAAGCATGTCAGACTGACAGAGATCGACACGCAGACGTCCACGTTTACGCTC
AAGTTCCTTCACACAGAAACGGGTCAGAACATCGAGAAGATTATCTACTTCATTGACAATGACACGGGCAACGAT
ACCCGCACTGCTACCGGAATCAAGCAAATCTTCAACAAGATGTTCCGCATCGCTGCGGAGAAGAGGAAGCTGTCG
CTCATCCAGATCGACACTGTTGAGTACCCCTGCACTCTAGTAGACTTGTTGATCCTGGTAGGCGTGGCGCTACCG
CCATTGTGCTACCTCTATCGTCCTGCACTGCACGCTATCTTTTTCCTGGTCCCTAATCCTGTGGGCTCCACCTTA
GAGGCGTGGCTGGATAGTGATCTTGTGCTGCGACTCATCATTGTTGCCGAGTTCCTGACTCACGCCCTCGAGACG
CTCATTTTCGTCGTCCCTAGGCTCAAGTACTACCGTGTTCCTGGCGAGTTCGTGCCCGAGTGGCTTCTTCTAGGT
CTCCTCGAAGGTTACGGTCCTGCGAGACGTCTAGATACTAAGGCTCGCACTCTTGGCGAAGGGTCAGTTAATTAG

>YPL127C 1.61   Hot
ATGGCACCCAAGAAATCCACTACCAAGACCACAAGTAAGGGCAAGAAGCCTGCAACCAGCAAAGGCAAGGAGAAA
TCAACTTCCAAGGCCGCTATCAAGAAAACCACGGCAAAAAGGAGGAAGCTTCCTCCAAGAGTTACAGGGAGTTG
ATCATTGAAGGGCTCACGGCTTTGAAGGAACGTAAGGGATCCAGTCGTCCGGCACTCAAGAAGTTTATCAAGGAA
AACTACCCGATCGTCGGATCCGCAAGCAACTTTGATTTGTACTTCAACAATGCCATAAAGAAGGGTGTGGAGGCC
GGCGATTTTGAACAGCCAAAGGGACCCGCTGGTGCTGTGAAACTGGCCAAGAAGAAATCTCCAGAAGTAAAGAAA
GAAAAAGAGGTCAGTCCAAAACCCAAGCAAGCCGCCACTTCTGTGAGTGCAACCGCATCAAAAGCGAAAGCCGCA
TCCACGAAGCTAGCGCCAAAGAAAGTAGTGAAAAAAAAATCGCCTACTGTTACCGCCAAGAAGGCCTCTTCGCCT
TCTTCATTGACCTACAAGGAAATGATCCTTAAAAGCATGCCTCAACTTAATGACGGTAAGGGCTCCAGCCGTATC
GTTTTAAAGAAGTATGTCAAGGACACTTTCTCCTCCAAGTTGAAAACAAGCTCAAATTTTGACTATCTGTTCAAT
AGCGCTATCAAGAAATGTGTTGAAAACGGCGAGTTAGTGCAACCAAAGGGCCCCTCCGGCATTATTAAACTAAAC
AAGAAGAAGGTCAAACTCTCCACGTAA

>YAL022C 1.52   Hot
ATGAGTACTAGTGCGGACACTGATACCATCAAGAAGCCAATCCTTGCGGTGCCAGAGCCTGCACTGGCCGATACG
CATTCAGAGGAGATATCACGCTCTGGAGAAGAACATGAATCAGAGAACAACGAGCACTCAGATGAAGAAGGCGAT
AATTATTCTGAAAGAGAGCAATCTGTGTCAACCGAACCACTGGATACATTGCCGTTAAGAAAAAAGTTGAAAAAT
CTTTCATATATTACATTTTTCGCCATAGGAATAGGTCTTTTATGGCCGTGGAACTGTATCCTCAGTGCCTCGCAA
TATTTTAAGCACGATATTTTCAAAGACACCTCCATTTGGGCAAAGATCTTCACAAGCTCTATGATGTCCTTTTCT
ACCATATCGTCAATGCTGTTCAACATCTACTTGGCCAAAAGACAGTACAAATACTCGAGAAGGGTCATAAACGGG
CTTGTGTGGGAGATTATTGTCTTTACTGTCATGTGCTTCTTTACAATTTTGCATTTCCTTTTACCTAAATGGTTC
AATTTCATGTTTATAATGATGCTTGTAGTGATCAGTTCCATGGGGACAGCCATGACACAGAATGGTATCATGGCC
ATAGCCAACGTCTTCGGTTCCGAGTACAGTCAAGGTGTCATGGTGGGGCAAGCCGTTGCTGGTGTCCTGCCCTCC
CTAGTGCTTTTTGCCCTAGCTTTCATCGAGAACTCTTCTGTGTCTACTACGGGCGGGATTCTTCTATACTTTTTT
ACCACAACACTCGTGGTCACCATTTGTGTGGTCATGTTCAGCGTGAGCAAAATCAGTCGGAAAGTGAACGAGAAT
TGGAATGTGGAAGACGGACATATCACTGATGTGTTGTTAGGGTCTCTGCGCTCCAATGAGGAGGAAATCCGTATT
GTTGGCCGCATTGACCAAATGGAGGATGAAGACCACCGCCGCACCAACGGCACTCGCGACGACAACGACGAAGGT
GAGGAACTCCAACTAAAAGTGCCTTTCGAGGTCTTATTTGCCAAACTAAAGTACCTGGTTCTTTCCATATTCACC
ACGTTTGTCGTAACTCTTGTTTTTCCTGTATTTGCGTCTGCCACCTACGTGACAGGGCTTCCTTTAAGCAACGCA
CAGTACATACCTCTCATATTCACGCTGTGGAACCTAGGCGACCTTTACGGAAGAGTCATTGCCGACTGGCCCATG
TTCCGTGACCAGAAATTTACGCCACGCAAAACCTTCATCTACTCATTGTTGCGGGTGGCCGCAATACCACTGTTC
TTGATGTTCACAGCCATCACCTCCTCTTCAAGCGGCGATGAAGAGCACAATGGCTCCGTAATCGTTGACTTGTGT
TACATGCTACTGCAGTTCCTTTTCGGCGTAACCAACGGGCACGTCATCTCTATGAGTTTCATGAAGGTGCCAGAG
CAACTGGACAACGACGACGAGAAGGAAGCGGCCGGTGGATTCACTAATATTTTCGTTTCTACAGGACTAGCTCTG
GGCAGCATCATAAGCTACGTATTCGTCTTCATAATTGACTTTATTATCAGGTAG

>YNL245C 1.94   Hot
ATGGGGTCGGGCGATTTGAATCTGTTGAAATCATGGAATCCCAAGCTTATGAAAAATAGAAAAAAAGTATGGGAG
ACCGAACAGGATCTAATTACCGAACAGCAGAAGCTTAACACGCGATTGAAAGAAATTGAAAAAGAACGAGAACTA
AATGAACTGCTGAACGAATCGAGCAAGGATAAGCCGGAGACTCTCAAGAATGACTTGGCTTTGAAGAAGTCGGGC
TTGGAATGGATGTATCAGGATGCAAAACTGAGTGACGAAAAGGAAGACTATTTATTAGGTAAGAAAAAACTAGAT
TCATCTATACTAAACCAACCTGCAACACCGCCTGTAAGGGCGGCCACTACTATTTCCGCTTCTGGTGCCGCAACA
TCCATAAGTTCGCAGAAGAAGAAGTCAAAATTGCTTAAAGATGATCCAATGAGTAAGTTTAAGGTAACTAAACAA
CAAAGAAGAACTCCAGACTCAACAAAAAAAAAGAGCAATGTCGCAACGAGGGAAACCGTTGTCCAAGCCCGCTCCG
GACCTAGACTACTAA

>YIL152W 1.69   Hot
ATGAGTCACAAGAGAAGGGGCCTGGTAATATATCAGGATCAGAAGCAGCAACAACAACACCCCCCGGGGCAGTCC
CTAAGCTCTATATCCTGGTCTCCAACCAGGCGACCTCATCACCCTTTAAAACAACAAAGCACCAACAGTTTCTCA
GAAATACTATCGAAATCAAGTGTGCAACCGAACGTGCAACATGACGGTAATCATATGCCCATTTCATTGCTAGTT
CTGAAACAAGAACATCATAAGCAGCAGCAGCAGCAGCAGCAGCAGCGCCAGAATATCCGATCACAAAACTCTACGCCT
CCCCTGCGACAGTTGGTTCAGGAGTCGCAGTGGACGAGCTCCGCAAGTAATAGTTCTCTAAAGAAACAGGAAAAG

CAGCCGCAAACATTCTACAACACTGACTCTAAACTTGTTTCTCAACTGCATAGCTCCGTGAAGGACCTGGATGCC
ATAATCCAGACGCATAAACCAAAGTTTGACACAATTATTCGCGATTTCTCTCAAGCAACCATACTGTCCTCAAAC
GAGTTACTCATCAAACTTCCGAAGGACCAAACAATAATATTGCATAGCAGAGCGCCAAAAATTAACGCAGAATGG
CTACAGAACAAGGTTAATGACCCTAGTGCCTCGTTAGTGATCGATTCAAGATCTTTTCTAACCTTGTGTAACAAT
ATAAAATGGTACCTGCATTGGAAATTTATATGA

>YJR112W 1.82 Hot
ATGGTTAACTCACATGGAATACGGTATATTCGGTTGAAGCAGGTCTTTAATCGTGCATTGGACCAATCGATTTCG
AAATTACAGAGTTGGGACAAGGTCAGTTCATGCTTCCCGCAATATGTGAATAGCAAGCAGGGCGCTATAAACGTC
GCCAATTGTCAGCGCCAATTAACGGAATTCTGGACGGAGCTTTGTCAACGAGAGTTTAAAGAAATTATGGAAGAA
CGAAACGTCGAGCAGAAACTAAATGAGTTGGACGAACTGATTTTGGAGGCTAAAGAACGTTACACGGATCGTGAT
CAAGATGAAGTAAACAAAGGGCCCGCGATCGATGAGCTCTCCAGCAAAGAGCTTGTGGAATGCCATTTATACAGC
CAACGGATGCATGCCATACACGAGATAGACGAGCGGCTTGCGAAAGTGAATGAGATGAACGACCAGTTAGCCCAG
GAGTTGAAAGACTTAGAGACGCAAGTAGAGGTAGAGAAAAACGAAATTGGCAAGATGTACGATGAGTACCTGGGC
TCCCACACGGACCAACCGGCCAACGTGCTGCTGGTACAGAGCCTCAATGATATGGTCTTGGAATTGAAGGAAAAC
TATTGA

>YPL088W 1.95 Hot
ATGGTTTTAGTTAAGCAGGTAAGACTCGGTAACTCAGGTCTTAAGATATCACCGATAGTGATAGGATGTATGTCA
TACGGGTCCAAGAAATGGGCGGACTGGGTCATAGAGGACAAGACCCAAATTTTCAAGATTATGAAGCATTGTTAC
GATAAAGGTCTTCGTACTTTTGACACAGCAGATTTTTATTCTAATGGTTTGAGTGAAAGAATAATTAAGGAGTTT
CTGGAGTACTACAGTATAAAGAGAGAAACGGTGGTGATTATGACCAAAATTTACTTCCCAGTTGATGAAACGCTT
GATTTGCATCATAACTTCACTTTAAATGAATTTGAAGAATTGGACTTGTCCAACCAGCGGGGTTTATCCAGAAAG
CATATAATTGCTGGTGTCGAGAACTCTGTGAAAAGACTGGGCACATATATAGACCTTTTACAAATTCACAGATTA
GATCATGAAACGCCAATGAAAGAGATCATGAAGGCATTGAATGATGTTGTTGAAGCGGGCCACGTTAGATACATT
GGGGCTTCGAGTATGTTGGCAACTGAATTTGCAGAACTGCAGTTCACAGCCGATAAATATGGCTGGTTTCAGTTC
ATTTCTTCGCAGTCTTACTACAATTTGCTCTATCGTGAAGATGAACGCGAATTGATTCCTTTTGCCAAAAGACAC
AATATTGGTTTACTTCCATGGTCTCCTAACGCACGAGGCATGTTGACTCGTCCTCTGAACCAAAGCACGGACAGG
ATTAAGAGTGATCCAACTTTCAAGTCGTTACATTTGGATAATCTCGAAGAAGAACAAAAGGAAATTATAAATCGT
GTGGAAAAGGTGTCGAAGGACAAAAAAGTCTCGATGGCTATGCTCTCCATTGCATGGGTTTTGCATAAAGGATGT
CACCCTATTGTGGGATTGAACACTACAGCAAGAGTAGACGAAGCGATTGCCGCACTACAAGTAACTCTAACAGAA
GAAGAGATAAAGTACCTCGAGGAGCCCTACAAACCCCAGAGGCAAAGATGTTAA

>YHL020C 1.93 Hot
ATGTCTGAAAATCAACGTTTAGGATTATCAGAGGAAGAGGTAGAAGCGGCTGAAGTACTTGGGGTGTTGAAACAA
TCATGCAGACAGAAGTCGCAGCCTTCAGAGGACGTCTCACAAGCTGACAAAATGCCGGCAAGTGAGTCGTCTACG
ACGCCGCTAAACATTTTGGATCGCGTAAGTAACAAAATTATCAGTAACGTAGTGACATTCTACGATGAAATAAAC
ACCAACAAGAGGCCACTGAAATCAATAGGGAGGCTGCTAGACGATGACGATGACGAGCATGATGATTACGACTAC
AACGACGATGAGTTCTTCACCAACAAGAGACAGAAGCTGTCGCGGGCGATTGCCAAGGGGAAGGACAACTTGAAA
GAGTACAAGCTGAACATGTCCATCGAGTCTAAGAAGAGGCTTGTAACGTGCTTGCATCTTTTAAAGCTGGCCAAT
AAGCAGCTTTCCGATAAAATCTCGTGTTTACAGGACCTTGTTGAAAAGGAGCAGGTGCATCCTTTGCACAAGCAA
GATGGAAATGCTAGGACGACCACTGGAGCTGGCGAGGACGAGACATCGTCAGACGAAGACGACGACGATGAGGAG
TTTTTTGATGCCTCAGAGCAGGTCAACGCCAGCGAGCAGTCTATTGTGGTGAAAATGGAGGTGGTCGGCACAGTC
AAGAAAGTCTACTCGCTGATATCGAAGTTCACAGCAAATTCGCTGCCGGAGCCCGCAAGATCTCAGGTTCGGGAA
AGTCTTCTAAACTTACCCACAAATTGGTTCGACAGCGTCCACAGTACATCACTGCCGCATCATGCTTCGTTTCAT
TATGCCAACTGTGAAGAACAAAAAGTGGAGCAACAGCAACAGCAACAGCAACAGCAGCAGCAGCAGCAACTTTTG
CAGCAGCAACTCCTGCAACAGCAACAGCAAAAAAGGAACAAGGATGGCGACGACTCAGCCTCGCCGTCCTCCTCC
GTAACTGCGAATGGGAAAGTACTCATTCTCGCCAAAGAATCCCTGGAAATGGTGAGAAATGTCATGGGCGTAGTC
GACTCCACGTTGGGCAAGGCTGAAGAATGGGTGAAGCAGAAACAGGAGGTAAAAGAAATGATCAGGGAGCGTTTC
TTGCAACAGCAGCAACAGTACAGGCAGCAACAGCAGAAGGATGGCAATTACGTAAAGCCCTCTCAGGACAACGTG
GATAGCAAGGACTAA

>YNL056W 2.27 Hot
ATGAAATACATACCCCCATTAAATTTTTCGCCAGTGGTGAGCACGGATGTATCGTTATATCGATCGGGATACCCG
ATGCCGTTAAATTATAGTTTCATCAAGCATCAGTTACACTTGAAGACCATAATATATATTGGCGATAAAGATAGG
CCTCTCGAAGAATATCAAAGCTTCCTGGAGTCAGAAAAGATAAAATACTATCACATATTTATGGATTCAAGCCGC
GATGAAGGAATTCAAGAAAGAATGAACCAGGTCTTGCACTTGGTGCTGGATGTCAGAAACTATCCCATCTTAGTG
CATTCCAATAAGGGCAAGCACCGTGTTGGTGTAGTTGTGGGAATCATAAGAAAGTTGTTACAGGGATGGTCCACT
GCAGGTATATGCCAAGAGTACGGGTTATTTTCTGGTGGGATGAAAGATGGAGTAGATTTGGAGTTTATCACAATG
TTCGAAACCAATCTGAAAATACCGAGAAACGTTATCCCTGGCTTTGCTAAACACTGCTTGTATTTGAATGAGCTA
GAGGCTGCGGAAGGAAGTGATGATGAGTCAGGAAGTGAGTCTATTCTCACTGCCAAGCAACCAATATGA

>YGR161C 2.08 Hot
ATGATCGCTACCTCCAGAGCCGTAAACATGAATAAAGAATCAAAACACAAGAAGGCTGTTGCCAAACCATGCAGA
GAGAGACAAACTTCAGTTACAAGAGCCATGAGACCAGCTGTAGCACGTGATCCTCGCAGACTTTCGACGTCGTCG
TCTCCTTCTTCGTCACCAATGTCAGCACAGAGAAGGCTTTCGAGGGAAGAAATAATAAACGAAATGGAAAAGGAG
CAAGACGCTATTGTAGTAAGACTTCTACGGGAAATTGAAACTTTAAAAGAAGAAAACTCTAGGTTAAAAAATCAA

CTGCACCATCCGGTCCCGGCTAGAAGGTCGTCTCCATTTTTCGAAGGCGAGTCTGCCATCCTAGATGATGATGAC
TGCAATTATGGTTACACCCTCGACACTCCAAAGCTAAAGCTCACAGATGGTGCATCCAGACACACCGTACTTCCC
TTAACACCCAAGGACTCCATGACCCACATTTCCCATTCCGCCAGAAGGTCAAGCCGGAACGCTTCCATATCTAAC
GGAACAAGCATCTCAGACACGATTTTCCCCATTGAGACTAAAATCCACTCAGCACCAACAACAAACAGAAACCTT
CCCTCCGCCGATCTTCCACATCACACTCTTCTTCCACGTTCTCTAAGCGGCATTTCGTCTAGCGATTTAACCGAA
TCCGGTGCTCTTCTTCATGACAGGAGAAGGCGTTCTTCCAATTACAGTCTCGATGGCTCAAACTCTTTAAAGGCT
GATCTCATGGCAAAGAGATTCCAAACTGGTTCATTGAAATAG

>YDR046C 1.82 Hot
ATGTCAGATCCTATAGTAACGTCTTCCAAAATGGAAAAAAGTGCAGAGTTTGAAGTAACAGACTCTGCTTTATAT
AATAACTTCAATACATCAACAACAGCTTCACTAACTCCGGAGATTAAGGAACATTCTGAGGAATCTCGCAATGGG
TTAGTTCACAGATTCGTCGACTCATTCAGAAGAGCCGAAAGCCAACGTTTAGAAGAAGACAATGACTTGGAGGAT
GGTACCAAATCGATGAAATCTAATAACCACTTAAAAAAGTCAATGAAATCTAGACATGTTGTAATGATGTCTTTA
GGTACAGGGATAGGAACAGGTCTTCTAGTTGCCAACGCCAAAGGTTTGAGTCTTGCAGGCCCGGGGTCTTTAGTC
ATCGGTTACGTTATGGTGTCCTTCGTCACGTATTTTATGGTCCAAGCGGCTGGTGAGATGGGTGTCACTTATCCG
ACGCTTCCAGGTAACTTCAATGCATACAATTCAATCTTTATTTCAAAGTCGTTTGGGTTCGCGACCACTTGGTTA
TTTTGCATTCAGTGGTTAACTGTGCTACCGTTGGAATTGATTACTTCTTCCATGACCGTCAAGTACTGGAATGAC
ACAATTAATGCTGATGTATTCATTGTCATTTTCTATGTGTTTTTATTGTTTATCCACTTTTTTGGTGTTAAGGCA
TACGGTGAAACGGAATTTATCTTCAATTCCTGTAAGATATTGATGGTTGCAGGATTCATTATTTTTATCAGTCGTC
ATCAATTGTGGGGGAGCCGGTGTGGACGGCTATATTGGCGGCAAATATTGGCGCGACCCTGGTTCATTTGCAGAA
GGCAGCGGTGCCACTCGTTTCAAAGGGATATGTTATATTCTAGTATCTGCATATTTCTCCTTCGGTGGTATTGAA
TTATTTGTTCTTTCCATCAATGAGCAGTCTAACCCTAGAAAATCCACTCCTGTAGCCGCGAAGAGAAGTGTATAT
CGTATTTTGATCATTTATTTGCTGACAATGATTCTAATTGGGTTCAATGTTCCTCACAATAACGATCAATTGATG
GGTTCCGGTGGCTCCGCAACGCACGCTTCACCATATGTCTTGGCAGCCTCTATTCACAAGGTTAGGGTCATCCCC
CATATCATTAATGCTGTTATTTTGATATCAGTCATCTCTGTTGCGAACTCTGCCCTTTATGCCGCACCGAGATTA
ATGTGTTCCTTGGCTCAACAGGGCTATGCTCCAAAATTTTTGAACTATATTGATAGGGAGGGAAGACCTTTAAGG
GCCTTGGTAGTGTGCTCTCTTGTTGGTGTCGTTGGCTTCGTCGCCTGTTCTCCACAGGAAGAGCAAGCATTTACA
TGGTTGGCAGCCATTGCAGGTTTGAGTGAACTTTTCACATGGTCAGGCATTATGCTTTCACACATTCGATTCAGA
AAAGCGATGAAAGTCCAAGGAAGATCACTTGATGAAGTCGGCTATAAGGCCAATACCGGTATATGGGGTTCATAC
TATGGGGTTTTTTTCAATATGCTTGTCTTTATGGCCCAATTTTGGGTCGCACTGTCTCCAATAGGAAATGGCGGA
AAGTGTGATGCTCAGGCATTCTTCGAGAGTTATCTGGCTGCCCCGCTATGGATATTCATGTATGTTGGATACATG
GTTTACAAAAGAGATTTTACCTTTTTAAACCCACTGGACAAGATCGACTTGGACTTTCATAGAAGAGTTTACGAC
CCTGAAATAATGAGGCAGGAGGACGAAGAAAATAAAGAAAGGTTAAAGAACTCTTCAATATTCGTCAGAGTCTAC
AAATTTTGGTGTTAG

>YAL069W 1.54 Hot
ATGATCGTAAATAACACACACGTGCTTACCCTACCACTTTATACCACCACCACATGCCATACTCACCCTCACTTG
TATACTGATTTTACGTACGCACACGGATGCTACAGTATATACCATCTCAAACTTACCCTACTCTCAGATTCCACT
TCACTCCATGGCCCATCTCTCACTGAATCAGTACCAAATGCACTCACATCATTATGCACGGCACTTGCCTCAGCG
GTCTATACCCTGTGCCATTTACCCATAACGCCCATCATTATCCACATTTTGATATCTATATCTCATTCGGCGGTC
CCAAATATTGTATAA

>YIL041W 2.13 Hot
ATGTCGTTTAATGCCTTCGCCAGCTCTTTGAGCAAGAAATTACAGGAAATATCCACAAGTGTTTCCGAAAAAACC
CAGGAATTGCCCAGTTTGGCGCAATCCACCCAAAGAATGGTCCAGGAACGTTTGGGCCAGGTGACGGACATCTCC
CAATTGCCAAGAGAGTACACAGAGCTGGAAGATAAGGTTGACACCATCAAACTGATTTACAACCACTTCTTGGGT
GTAACTGCTATCTACGAAAACGGATCGTACGATTACCCTAAATACATCAACGAATCCGTCAACGAGTTTTCAAGA
AGCGTGGCTTCCAAGTTGACAGAGTTGACTCATGCTACATCTGCGTCTGAGGCACAAAACATCTTAGTTGCTCCA
GGCCCCATCAAAGAACCCAAGACGTTGAACTACGCCCTCAGTAAAGTGGCTTTGAACTCCAGTGAATGTTTGAAC
AAGATGTTCCCCCACCGAGGAACAACCCTTGGCTTCGGCACTCTTGCAATTCAGTGATGTGCAGGCTAAGATTGCT
CAAGCTAGAATTCAACAAGATACCTTGATTCAAACCAAATTCAATAAAAATTTGAGGGAAAGGCTCTCTTTTGAG
ATCGGTAAGGCCGATAAGTGCCGCAAGGATGTCCATTCCATGAGATTAAGATATGACGTGGCAAGAACTAACTTG
GCAAACAACAAGAAGCCAGAAAAGGAAGCTTCCTTGAGAGTCCAAATGGAGACTTTGGAAGACCAGTTTGCTCAA
GTCACTGAAGACGCTACTGTGTGCTTGCAGGAGGTTATTTCTCACGCTAACTTCAGTGAAGATTTGAAGGAATTG
GCCAAGGCTCAAGCTGAGTATTTTGAAACCTCGGCTGGCCTAATGAAAGAGTTCCTATCCAACTCATTTGCGGAA
GAGCCGGAAGCAAAGCCTGAGGTTGCAGAAGAAGAAAAGCCACAGACAGCTATCTCTATGAATGACGAAGACGAC
GCTTAA

>YPL092W 1.96 Hot
ATGGTTGCCAATTGGGTACTTGCTCTTACGAGGCAGTTTGACCCCTTCATGTTTATGATGGTCATGGGTGTCGGC
ATTTCATCGAATATTCTATATAGCTTCCCATATCCTGCAAGGTGGCTAAGAATATGCTCCTACATCATGTTTGCT
ATCGCTTGCCTTATTTTCATTGCTGTGCAGGCACTACAAATATTACATTTGATTGTCTATATTAAGGAGAAAAGC
TTCAGAGAATATTTTAATGACTTTTTCAGAAATATGAAGCACAATTTATTTTGGGGTACTTATCCCATGGGGTTA
GTTACAATTATAAATTTCTTAGGAGCACTCTCGAAAGCGAACACGACGAAGAGCCCCACTAATGCCAGAAATTTG
ATGATATTTGTTTACGTCTTGTGGTGGTATGATCTCGCAGTCTGTCTAGTAATAGCGTGGGGTATCTCGTTTCTC
ATCTGGCATGACTATTACCCTTTGGAAGGGATTGGGAATTATCCTTCATATAATATCAAAATGGCATCCGAAAAC
ATGAAAAGTGTATTGCTACTGGATATCATTCCGCTGGTTGTCGTCGCTTCAAGTTGTGGAACATTCACAATGTCA

GAAATATTCTTCCATGCGTTTAATAGAAACATTCAACTGATAACGTTGGTCATATGTGCCTTAACGTGGCTGCAT
GCCATTATCTTCGTCTTCATACTGATTGCGATATACTTCTGGAGTCTTTATATTAATAAGATACCACCAATGACA
CAGGTTTTCACCTTATTCCTGCTTTTGGGCCCGATGGGCCAAGGAAGTTTTGGAGTCTTATTGCTTACAGATAAT
ATAAAAAAATATGCGGGCAAATATTACCCAACAGATAACATTACAAGAGAACAAGAGATATTGACTATTGCAGTT
CCATGGTGTTTCAAAATTCTAGGCATGGTTTCTGCTATGGCATTGCTCGCTATGGGCTATTTTTTCACCGTGATT
TCTGTCGTTTCAATCCTGTCGTACTACAATAAAAAAGAGATTGAAAACGAGACAGGAAAAGTGAAGAGAGTTTAT
ACCTTCCACAAAGGTTTTTGGGGGATGACTTTCCCGATGGGTACTATGTCTTTAGGAAACGAAGAGTTATATGTG
CAGTATAACCAGTACGTTCCCTTATATGCATTTAGAGTCCTAGGAACCATATACGGCGGTGTTTGCGTTTGTTGG
TCAATTCTATGCCTTTTATGCACATTGCATGAGTATTCTAAAAAGATGCTGCATGCTGCCCGTAAATCTTCATTA
TTTTCAGAGTCAGGTACGGAAAAGACGACAGTTTCTCCGTATAACAGCATTGAAAGCGTGGAAGAATCAAACTCG
GCTCTAGATTTTACGCGTTTAGCATAA

>YIL154C 2.04   Hot
ATGCAGAAGAGCATATTGCTGACTAAACCTGACGGAACACAATCGAATCTACACAGCATCAAAACGGAAACGCCC
ACCACGGTGGAGTTCGACTCGGAGCAGATGGAAAGGGGCCACAGGGAAAGAGGTCGTAGCAAGAAGAAAAGAGGC
GAACGAGACTCAAACGTGAGCAGTCTGTCGCGGTCGAGAAGCAGGGCCAGTAGCCGCAGCAGAGTAAGGGAGGAA
GAGTTCCTCAAGTGGACCGTGTTGAGGCAGGACCCCTCGATGCGGTTGAGGGTCGTGGATGTGGATTCTGAAGAG
GAAGGTGAGGGCAACGACGAAGATGACGACGACGGCGACGGCGACGATATGGACGAGGAAGAGTCCGATGAAGAG
CAAGTGAGCGATATAGAGAACGATTTAGAGATTGACGAGGAGTTCCACTACGATCTGGGCATGAAAGTGTTACCC
AACTTTTGTACCAGCATAAACGAAGTGCTAGACTCCAGCAAGCCCTGGATAGCCAAGTACGAGATCAGCATCCGT
GGCCACGAAAACGAAGACGTGTCTCTGGAGCAACTCGACGGAGGCTACGTCAGAGCCATGCAACTACTCACCAAG
GGTGCCGGCGCAGAGGCCGGGAACCAAAGGTCCTTCATCCTCTACACGGACCTGAGCAGCGAGTCCACCTACGCC
CTGACCTATCTCATGGGCGCAGCTGTCAACCAGGGAGACACCGTCTACATTGTCCACTGGGAGCCCTCGAAGCCC
ACGGACGACTCCCAGATGTTCGCCAACGTTGCCAGAATCAGAAAGCACGTCATGCACCTGTTTGACTGCGTCGCG
GGCGTGCTCGACGACCTCGACGTCGTCGTCCTCTCCTTGACCCATCCGTACCCAAAACACCTCCTCAACGAGATG
ATCCACGGCCTCAAGCCAGTCGCCCTGTGCTGCTCCCTCTCGGTCATCCTGTCCACTCTGCAGAACTTCGTCTGC
TCTGTGCCCATCCTCGCGGTTAGAAAGAAGCTGAAACGTGCCAAGCGCAAGGGCATCAGCGAGTGA

(2) S^- : 591 recombination cold spots
>YPR115W 0.78   Cold
ATGTCTGACTATTTCACGTTTCCTAAGCAGGAGAATGGCGGTATCTCGAAGCAGCCTGCGACACCAGGTTCAACT
CGGAGCTCTTCTAGAAATTTGGAGTTACCGAAGAACTATCGTTCATTTGGTGGTAGTAGCGATGAACTGGCATCT
ATGTATTCCGCCGACTCCCAGTACTTGATGGATATGATTCCTGATTCATTGACGCTGAAAAATGAGCCAGCATCT
GGAAATACGCAAATGAACGGGCCAGATGGCAAAGAGAATAAGGACATAAAACTTGACGAGTATATATTACCCAAG
ACAGACCCAAGGTCACCCTACTATATTAACATGCCTATACCTAAAAAATTGCCGAAGAGTGAAGGTAAGGCGAGG
GCTAAACAAAAGGTTAACAGGGCTGATCCTTCTGATTTAGATGTGGAGAACATTTACGAAACTTCCGGGGAGTTT
GTTAGAGAGTATCCGACAGATATTTTGATTGATAGGTTTCACAAATGGAAGAAAATCTTGAAAAGTTTGATCGCT
TACTTCAGAGAAGCTGCCTACTCTCAGGAACAAATTGCAAGGATCAACTACCAGATGAAAAATGCCGTAAAATTT
GCTTTTCTTACTGATTTGGAAGATGAAACAAACAAACTGGTTGATCCAAGTATATCTAAGTTGCCTACTAAGAAA
CCTCAGCCTGTCCCATTGGCCGCTCAAAAATTGGACAGCAAGTACGACACAGATGTGGAACAGCCGCAGTCAATC
CAATCAGTACCTTCGGAAGAGGTAGCTTCAGCTTCTTCTGGCTTCATGAAATTTGGGTCAGGATCCATTCAAGAT
ATTCAAGTGATTTTGAAGAAGTACCATTTATCTTTGGGTAGTCAACAATATAAGATCTCTAAGGAAATCTTGGCA
TATATTATACCCAAATTGACCGACTTGCGTAAGGATTTGACCACAAAGATGAAAGAAATTAAAGAATTAAATGGA
GATTTCAAGACTAACATAGGCGAGCATATCAAAATAACAAGCAGGCTTCTGAATAAATATATTGCTTCAGTGAAG
TTATTAGATGAAGCATCCACTTCGGGCGATAAGCAAGGTGAAAAATTAAAACCAAAACACGATCCATACCTTTTG
AAATTACAACTAGATTTGCAGCTGAAAAGACAATTACTCGAAGAAAACTATCTTCGAGAAGCTTTTCTGAATTTA
CAATCAGCAGCACTACAACTGGAGAAAATAGTATATTCTAAAATTCAATCCGCATTACAGCGCTACTCAGCGTTA
ATTGATTCAGAAGCACGATTAATGATCAAAAATTTGTGCCATGAATTGCAGCAAGGTATTCTATCTAGACCACCG
GCTGTAGAGTGGGATAATTTTGTCTCTCATCATCCTACCTGTCTGATGAATTTAAAATCGACAGACCCGCCACCG
CAACCCAGAAGATTATCTGATATTGTTTATCCTAACATGAAATCGCCGCTGGCCAAATGCATTAGAGTAGGATAT
CTACTGAAGAAAACAGAGTCTTCTAAAAGTTTTACTAAAGGTTATTTTGTGTTGACTACAAATTATCTTCACGAA
TTCAAAAGCAGCGATTTCTTTCTAGACAGCAAGTCTCCCCGTTCAAAAAATAAACCTGTTGTTGAACAATCAGAC
ATTAGCCGTGTGAATAAGGATGGGACTAACGCTGGCAGTCACCCATCGAGCAAAGGGACACAGGATCCGAAACTG
ACGAAGAGAAGAAAGGGTTTGAGTTCTTCGAATCTATATCCTATCTCTAGTTTGTCTTTAAATGATTGCTCATTA
AAGGATAGTACTGACTCAACTTTTGTTTTACAAGGGTATGCTAGTTATCATTCACCAGAGGACACATGTACAAAA
GAATCAAGCACGACCTCAGATTTGGCATGTCCAACAAAGACGCTTGCTTCAAATAAGGGCAAACATCAACGTACG
CCTTCAGCTCTCTCGATGGTGTCCGTTCCGAAATTTCTAAAGAGTTCTTCCGTGCCCAAGGAACAAAAAAAGGCT
AAAGAAGAAGCTAACATCAATAAGAAGTCAATTTGCGAGAAGCGCGTTGAATGGACGTTTAAAATATTTTCCGCA
AGTCTTGAACCAACTCCAGAGGAATCAAAAAATTTCAAGAAATGGGTTCAGGACATAAAAGCGCTTACAAGTTTC
AATTCTACTCAGGAGAGGTCGAACTTCATTGAAGAAAAGATTCTGAAAAGTAGAAACCATAATAATGGAAAGAGC
TCTCAAAGGTCCAAAAACAGTACATATATCACTCCAGTAGACAGTTTTGTTAACCTTTCTGAAAAAGTAACGCCA
TCTTCCTCTGTTACCACGTTAAACACTAGGAAGAGGGCAAATCGTCCTCGTTATATAGACATCCCAAAAAGCGCA
AATATGAATGCAGGCGCTATGAATTCCGTATACAGGTCAAAAGTCAATACACCAGCCATTGATGAAAATGGTAAT
CTGGCCATAGTGGGAGAAACGAAAAACAGTGCTCCACAAAATGGTATGAGTTACACAATTAGAACCCCTTGCAAA
TCGCCTTATTCACCTTATACTGGGGAAGGTATGCTTTACAACCGGTCAGCAGATAACTTAATGGCATCCTCTTCG
CGAAAAGCATCGGCTCCCGGAGAGGTACCTCAAATAGCTGTTAGTAATCATGGGGATGAAGCCATTATTCCGGCA
AGCGCGTATTCAGATAGTAGTCATAAATCGTCTCGTGCCTCCAGTGTTGCATCCATTCACAACCAACGGGTTGAT

TTTTATCCTTCGCCATTAATGAACTTACCAGGTGTTTCACCCAGTTGTCTAGCATTGGATGGTAATGCAAACGGA
TATTTTGGAATACCATTGAATTGCAATTCGGAAGCAAGACGAGGGTCAGATCTTTCACCTTTTGAGATGGAGTCT
CCATTGTTCGAGGAAAACAGAACTCAAAACTGCAGTGGCTCTCGCAAAAGTTCAGCTTGTCATATTCCCCATCAA
TGTGGTCCAAGGAAAGAAGGTAATGACTCACGATTGATCTACGGCAATGAGAAGGGCGCCTCACAATCACGTCTA
ACGTTGAAGGAACCGCTTACATCTAAAGGTGTTGAAGCTCCTTATAGCAGTTTGAAAAAGACATATAGTGCGGAA
AACGTCCCACTAACATCTACTGTATCGAATGATAAGTCTCTTCACTCGCGCAAGGAGGGAAGTACTAATACTGTA
CCTGCTACATCCGCGTCAAGTAAGTGA

>YDR163W 0.82 Cold
ATGACCACATCACACAGACCACAGTTAGAAGCAAGAAGCGGTGCAAAAGCGGCCGCTTATACACCAACAGGCATC
GAGCATGCCAGATTACTACCAGGACATACAACATTAAAATACAGGAAATTTAAAGAGGAGGAAAATCTTAGAGCA
AACTGTGCGCAAGAAGATAGGAGCAACGATAAATCTTTAGAGGAGGCAGTAATGAACGAAGAGAAACAGGATGTT
GTGGGGAGTGGGAACCTCCAAGAAACCCGCAGTGAGAAAGACCAAAAAGACTCGTTGCAAGAGCTGCTGGTTACT
CAAAAAAATAAAGTGGAAGATAAAGCAGAGCTTGAAGGAAATGAACAATTAAAGGGAGGAAATTCGTCTAGGCGA
TCGTGGAGAAAGGGCACGGCGTTTGGACGCCATAAAGTAACCAAAGAGACAAATATTAAGGAACATGCAACAAAA
AAATCAGCGTCCGGATACATTAACGATATGACAAAATCTGAATATCATCAAGAATTTCTCCATAAGCATGTTAGA
TAG

>YLR115W 0.77 Cold
ATGACTTATAAATACAATTGCTGTGATGATGGATCAGGAACCACCGTTGGTTCAGTGGTACGATTTGATAACGTC
ACCTTGTTAATCGATCCCGGTTGGAATCCATCTAAAGTATCGTATGAGCAATGCATCAAGTACTGGGAAAAAGTG
ATACCGGAAATTGATGTAATAATACTATCACAACCAACAATTGAATGTTTAGGTGCTCATTCCCTTCTGTACTAT
AATTTTACCTCGCATTTCATATCCAGAATTCAAGTTTACGCAACTCTTCCTGTTATCAACTTAGGGAGAGTTTCC
ACCATAGATTCATATGCATCTGCAGGTGTTATAGGTCCATATGACACAAATAAACTCGATCTTGAAGATATTGAG
ATATCTTTTGATCATATTGTACCTTTGAAGTACTCGCAATTGGTAGATCTACGATCAAGATACGATGGATTGACT
TTACTGGCGTACAATGCTGGTGTGTGTCCAGGTGGTTCTATTTGGTGCATTAGCACATACTCAGAGAAATTGGTT
TATGCAAAGCGATGGAACCACACTAGAGATAACATACTAAATGCTGCTTCTATATTGGACGCCACTGGTAAGCCC
TTATCAACTTTGATGAGGCCCTCTGCAATAATTACTACTTTGGACAGGTTTGGCTCCTCTCAACCGTTTAAAAAA
CGTTCAAAAATCTTTAAAGATACATTAAAGAAAGGTTTGAGTTCGGATGGGTCCGTTATAATACCGGTAGATATG
AGTGGCAAATTTCTGGACCTGTTCACACAAGTTCATGAGTTATTATTTGAAAGCACAAAGATCAATGCGCACACA
CAAGTACCTGTACTTATTCTGTCTTACGCGAGAGGAAGAACTCTAACATATGCCAAATCAATGCTCGAGTGGCTG
TCCCCTTCACTACTTAAGACATGGGAAAACAGAAATAATACTTCTCCATTTGAAATTGGATCACGGATCAAAATC
ATCGCACCAAACGAATTGAGTAAATATCCTGGTTCAAAGATTTGCTTCGTATCTGAGGTAGGAGCTTTGATTAAT
GAAGTGATAATTAAAGTTGGTAATTCTGAGAAGACAACCTTGATTTTGACCAAGCCGAGCTTTGAATGTGCGTCG
TCTTTAGACAAGATACTAGAAATTGTTGAACAAGATGAAAGAAATTGGAAAACATTTCCTGAAGATGGTAAATCA
TTCCTCTGCGATAATTACATTTCAATAGACACCATAAAAGAAGAACCCTTGAGTAAAGAGGAAACAGAGGCGTTT
AAAGTACAGCTTAAAGAAAAGAAAAGGGACAGAAATAAGAAAATTCTACTGGTGAAAAGAGAGTCTAAGAAACTG
GCTAACGGCAACGCAATCATAGATGATACCAACGGAGAGAGGGCAATGAGAAATCAAGACATTTTAGTGGAAAAC
GTCAATGGAGTACCACCGATTGATCATATTATGGGGGGTGACGAAGATGATGATGAAGAGGAAGAAAACGACAAT
TTGTTGAATTTACTCAAGGATAACTCTGAGAAATCAGCAGCGAAAAAAAATACGGAAGTTCCTGTAGACATTATA
ATTCAACCAAGTGCCGCTTCAAAACATAAAATGTTCCCATTTAACCCCGCTAAAATCAAGAAAGATGATTATGGT
ACTGTTGTAGATTTTACGATGTTTTTACCTGATGATTCTGACAATGTTAATCAAAATAGCAGGAAAAGACCTCTC
AAAGACGGTGCTAAAACCACGAGCCCTGTAAACGAAGAAGATAACAAAAATGAAGAAGAAGATGGTTATAATATG
AGTGATCCAATTAGCAAAAGGAGCAAACACCGTGCTTCTCGTTACTCGGGATTTTCTGGAACCGGAGAAGCAGAA
AATTTTGATAACCTTGACTATTTAAAGATAGACAAAACGCTCTCCAAAAGAACAATCTCAACTGTAAATGTCCAA
TTGAAATGCTCCGTAGTAATCCTAAATTTACAGAGTTTAGTTGATCAAAGGTCTGCATCTATTATATGGCCATCG
CTAAAATCTAGAAAAATAGTTCTTTCCGCCCCCAAACAGATTCAAAATGAAGAAATTACGGCAAAACTTATCAAG
AAAAATATTGAAGTGGTCAACATGCCATTAAATAAGATAGTGGAATTCAGTACAACAATAAAGACCCTGGATATC
TCCATTGACTCCAACCTCGATAACCTTTTGAAATGGCAACGTATCAGTGATAGTTATACAGTTGCCACTGTCGTT
GGCAGGCTGGTTAAGGAGAGCCTACCGCAAGTGAACAATCATCAAAAAAACAGCGTCAAGGAGTAAACTAGTCTTG
AAACCATTACACGGTTCATCTAGGAGCCATAAAACAGGTGCGTTGTCAATCGGTGACGTTAGGTTAGCACAATTA
AAGAAACTTCTCACAGAGAAAAACTACATTGCAGAATTTAAAGGGGAAGGTACACTTGTCATAAATGAAAAAGTA
GCAGTCCGCAAAATCAATGATGCAGAAACTATTATCGATGGTACGCCTTCCGAACTTTTTGATACTGTCAAAAAA
TTGGTTACGGATATGTTAGCAAAAATCTAG

>YDL063C 0.78 Cold
ATGGGCAGATCAAAGAAAAGATCAAGAGCATCTTCGTCTCGATTAAATCCACTACGCAAAGCTGGGTCAAATGAC
AATAATAAAGATACCAACGTCGTTAATAAAAAACTCCAGCCGCTATTGCAGAATTTGTCAAGTGTGGTGCCTAAT
GATAGAAGTATTGCTTTGAGTTCCATCAGTGTTCTTTGTGAGGATGCACATATGAGACAACTTTTACTGAAAGAG
AAATTAGTTCCTATCATTTTGAATAAATTGCTGAACGATTCTAATTCGGACATAGTTGTGGAGTCGTTCGGCTTA
TTAAGGAACTTGTCTTTGGAAGAAGGTTATGATGTGTCAATCTACCTTTGGAGATCTGATATATGGACAAGTATA
ACAAGTAATTTTGGCAGGATTGTTGAATCGTTATCCGCTTTGCAAGCAGCAGAACAGCAACCGCAGCTAAAACCT
GCAGGAAAATCCAAAATAGAATCTAAAAGGTTGTTATTCGATTTTGCCGATAACTTACTGTCGTTAGTGGTGGCT
CTGTCTAATGGATCCGACGATATTCTTAATGAGATATTAACGGAAAGTAAAATAAATGAAATTTTTCAGGTCATT
TCACAGCTATTAAAATATGGTGTCGAGAAACTGCCAATTAATCTGTTCAATACCACTCTTGATCTCATTTATGAT
TTGAGTTCCGAATCATTTGAATTTATTGATCATGTTTCCAACAATGAACTTCTATCACAGTTCTTAAATGGGCTA
TCTCCGGCTTTACATCCACAAGCTAATGAATTAACAAAAGTTCTAATTGAAGGTATCCATTGCCAATTCCTTGAC

ATGAAAATAACTTATGATCAATGTAACAAAATGATCCACTCTGTATGTCATTCCATTAATAACATTGATCCAGTA
CAGTTAGTTAATGACATTAATAACCCTGTAGAAATTGGACCTGCTACTAGTAAAGATGAAAGCAGTAAGGTTATA
ACTAAGATCAAGGATTACAATGCGAAACGTAACGAATCCATGATAAAATTGCAAAGCATTGAAATAGCAATTGAT
CTTATCACCGCAATCATTGAGATAGTTGCATCCAAATATGAATCACCAGAATCACAGGAAGTAGCAATTCCAGAG
GAGCTAATCAATACCTTAACAAATTTCTTACCTCACGTTTTCATGATTTTGAAAGATACCTTCACCTCGCGAATA
TTGATCGGATGGAACAATCTGATCTGGCTCTTTGTGTCTCTATCTCTAACAGAGCTCTCTGGAGAGTTGTTAACC
ACCTTATGGAGTTATGTTACACAGTTGGACAGCCAAGATGATTTGAGTATTAAAATAGGAAGAATGGGCTGTATT
TGGGCGCTTCTGAAACTCATCTTTCCTGATGGAGCGTTTGAAAGTGAGAATCGAGCATTGATCAATGTCCAAATG
CTGAACAATTCAGGATTCGCTAGGGGAATTATTGAAGAGTTCCAAAATAATAATGACCTTGAGTTACAACAAAAA
TGTATTAATGTACTAAGCACATATGCTATGATTCAGGGACAAATTGATGCAAATAAGGAGATTGGGCAGTTCTTC
ATACAAACTTTAACACAGTTGAATGTTCGCCCTGAAATTTTGATAGAAATGACAAATTCGCTTTTCCAAATTTAC
GGGGATGCCTCTTACGACTATAATGAACCAATATTTGTAAGGGGTGGATTTTTATCTATTCTAAAAGACCAAGTT
GTACCAAATTTGAGGCAGCAATTCAAAATGGTTGATAAAAACAAAAATCCCGAACTAAAAGAAAGATGCCATGAT
TGCTTTACTACGTTGGATAGTTTCATTCACTACAAAATGAATGAAAATTCTACTAACCAATAG

>YIR009W 0.8Cold
ATGGTTGAACCAGCAAGGAAAAAGCAGAGGATTGATCGCGATACTCATCACACTGTGGCTGAACCAGTAACGGAA
GCTAAAAATACGCTGTACGTGAGCCAGCTTAATGAAAAAATTAATATGCAACGACTGCGAGTAAACCTTTTTTTG
CTCTTTGCTACGTTTGGAGAGGTACTGAAGGTTAGCATGAATTTCAAGAAACAGCGGGGCCAAGCCTTTATCACC
ATGAGAACCATCGACCAGGCCAGTCTGGCCCAGATTTCATTAAACGGCGAGCGATTCTTTGGTAAGCCTTTGAAA
GTTGAGTTCAGCAAGAGTGAAACTAAAACACTATGA

>YOR018W 0.68 Cold
ATGTTTTCATCATCATCTCGACCTTCAAAAGAGCCATTACTATTTGACATCAGACTTAGAAATTTGGACAATGAT
GTTTTACTGATAAAAGGCCCCCCTGATGAGGCATCTTCTGTCCTACTATCTGGAACTATAGTATTGTCAATTACT
GAGCCAATTCAAATCAAGTCTTTGGCTTTGAGACTTTTTGGTAGGTTGAGACTAAATATTCCAACGGTTTTACAA
ACTGTTCATGGGCCGCATAAGCGATACTCAAAGTTTGAGAGAAACATATATTCTCATTTTTGGGATGATTTTAAT
ATAAAAAGTTATTTCCAAAACTTGTACGATAATCATAATAATGGTAAAATAACAATTTCTAGTAAATCCTCAACA
AATTTAGCAGCATTGCCAAAGAGAAAAAGAGCCCTTTCTACTGCATCATTGATATCAAGTAATGGGCAGACAAGC
GCAAGCAAAAACTATCACACCTTAGTAAAAGGTAACTACGAATTCCCTTTCAGCGCGATTATTCCTGGGTCATTA
GTGGAAAGTGTAGAAGGCCTACCAAATGCTGCCGTCACTTATGCTCTGGAAGCTACTATCGAGAGACCTAAGCAG
CCCGACTTGATCTGTAAAAAACATCTAAGAGTTATTCGAACGTTAGCTATAGATGCAGTCGAGTTATCTGAAACA
GTATCAGTGGATAACTCATGGCCTGAAAAAGTCGATTATACGATCTCCATTCCAACTAAGGCAATTGCCATTGGC
TCTTCCACCATGATCAATATTTTAATTGTTCCTATATTAAAAGGATTGAAGTTAGGCCCTGTTAGGATCAGTTTG
GTGGAAAATTCCCAGTATTGTGGTAGCTATGGAGGGGTTATCAACCAAGAAAGAATGGTGGCTAAATTAAAACTA
AAAGATCCCCTGAAGCACGTTGCCCAAATAAAGAAGAAGAGGAGCCTAAATGAAGCTGCCGACGAAGGGGTTGAT
ACGGACACAGGGGAATTTCAAGATAAATGGGAAGTTCGAGCTTTATTAAACATACCTGCAAGCCTGACTAAATGC
TCCCAAGACTGTCGCATTTTATCTAATATCAAAGTCCGTCATAAGATCAAGTTCACTATAAGTTTACTCAATCCG
GACGGTCATATTTCAGAATTGCGTGCGGCACTGCCTGTCCAATTATTCATTTCACCGTTTGTTCCAGTCAATGTA
AAGACCTCCGATGTTATTGAAAGAACGCTCAAAACGTTTGGACCCTCATATCAAGTAACAAGTCAGCACGATAAT
TCATTCAGCAGCAAAAACTTTGTAGACGATAGTGAAGAAGATGTGATTTTTCAAAGATCTGCTTCTGCGTTACAA
TTGTCTTCAATGCCAACCATAGTATCTGGCTCTACTTTAAATATCAATAGTACTGATGCAGAGGCTACCGCAGTC
GCTGACACAACTATGGTAACTAGTTTGATGGTACCTCCCAACTACGGCAATCACGTTTACGATCGAGTGTATGGC
GAGGTAACTAATGAAGACGAAACTTCAGCATCAGCTTCTTCAAGTGCCGTCGAATCACAGGCAATTCACAATATT
CAAAACCTATATATATCGGATAGTAACAATAGCAATAATCCTATTTTGGCACCAAATCCTCAAATCAAGATTGAA
GATGATAGCCTAAATAATTGTGACTCTCGAGGGGACAGCGTTAACAATAGTAACCTGAATCTGGTTAATAGTAAT
CTAACAATTAGTGAAAATTGGAACAATAACTCTCCTTCTGCAAATAGATATAATAACATCATTAATGCTGGATTG
AATAGTCCTTCACTGACACCAAGCTTTGCACATTTATCTAGGCGTAACTCATATAGTCGCCAAACATCTTCTACA
TCGCTGAAGAACGATTTGGAACTGACAGATTTAAGCAGAGTTCCCTCGTATGATAAAGCAATGAAATCTGATATG
ATTGGTGAGGATCTTCCACCGGCTTATCCCGAGGAAGAACTTGGAGTTCAAGAAAATAAAAAAATTGAACTAGAA
AGGCCACAAATTCTTCATCACAAGTCTACATCCTCTTTGTTGCCACTTCCAGGCTCGAGCAAGAGTTCCAATAAT
CTGAAAAGATCGTCTAGTAGGACACATTTATCCCACTCTCCATTACCAAGGAATAATAGCGGATCCTCAGTATCA
TTGCAGCAGTTGGCGAGAAACAACACAGATAGTTCATTTAATCTAAATCTCTCTTTCACTTCAGCAAAAAGCAGC
ACAGGAAGCAGACATTTTCCGTTTAATATGACAACATCTTTCACTAGTAATTCAAGTTCCAAGAACAATTCACAT
TTTGATAAAACTGATTCTACATCTGACGCTAATAAGCCAAGAGAAGAGGAAAACTATACGAGCGCAACCCACAAT
CGAAGGTCACGCTCATCGTCAGTTCGGAGCAACAATAGCAACTCACCATTAAGACAAGGAACAGGTTCATTTGCT
AATTTAATGGAGATGTTCACAAAACGGGATCGCTCATAG

>YIR029W 0.69 Cold
ATGAAGTTTTTTAGTTTGGCAGATGAGGCAGAATTTAAGTCAATAATCATTTCAAAAAACAAAGCAGTAGATGTT
ATAGGATCTAAGTTAGGTGGTCAAGTGGTTTCTTTTTCAGATGAATGGTTTGCTTCTGCTGAAAATTTAATTCAG
CCAACTGCTCCTATAAGGGATCCTACCAGATTTGTTCATTCAGGCGCATGGTACGACGGCTGGGAAACACGAAGA
CATAATGAGATGGAATACGATTGGGTTATCATCAAGATGGGAGTCGCTGCTGCCCATATTATTGGTGGTGAAATT
GACACTGCGTTTTTCAATGGTAATCATGCACCATTTGTATCAATAGAGGCGCTCTATGACGAGGGCGAAGAAGGT
AATATAGTGGAAGATGACTCGCGTTGGGTTGAGATTGTTGAGAAATTTGAGTGTGGTCCTTCGCAAAGGCATCTT
TTCGTTAGAGGCAACGGCCTTACTAAAGAAAGGTTTACTCACATCAAGCTGAAGATGTACCCCGATGGAGGAATC
GCCAGATTTAGATTATACGGAAGAGTTGTTCCTCCAGAACTCAAAACGAAGGATCATATAATTGACTTGGCTTAT

GTTTGCAATGGTGCTGTCGCTTTAAAGTATTCTGACCAACACTTCGGTTCAGTTGATAATTTGCTACTGCCAGGT
CGAGGTCATGATATGTCGGACGGCTGGGAAACCAAAAGATCGAGACAACCAGGTCATACTGATTGGGCGGTAATT
CAACTGGGCAGGGAGTCTTCTTTCATTGAGAAGATTATTGTTGATACGGCACACTTCAGGGGGAACTTCCCTCAG
TTCATTACCGTGGAAGGTTGCTTGAAGGAGTCAGAATCAAGTGAGAACACTGGTGAAGGAACATGGGTAGAGCTA
GTTGGTAAGTCAAAGACGGGGCCTGACAAAGAACATGTGTATGAAATACGTAAGAGTATAAGAGTCTCCCATGTC
AAATTAACGATTATTCCAGATGGAGGAGTGAAAAGAATAAGAGTTTGGGGGTACTGA

>YKL048C 0.78 Cold
ATGTCACCTCGACAGCTTATACCGACATTAATTCCGGAATGGGCACCATTATCCCAGCAATCGTGCATAAGAGAG
GATGAGTTAGATAGTCCCCCGATAACGCCTACGAGCCAGACATCTTCATTTGGTTCTTCTTTTTCTCAACAGAAA
CCAACCTATAGTACAATTATAGGAGAAAATATACACACGATCCTGGATGAAATTCGACCATATGTGAAAAAAAATA
ACTGTTAGTGACCAAGATAAGAAAACTATAAACCAATATACGCTAGGAGTCTCTGCAGGAAGTGGACAATTTGGT
TATGTACGAAAAGCGTACAGTTCTACTTTAGGCAAGGTTGTTGCTGTCAAGATTATACCAAAAAAACCTTGGAAT
GCCCAGCAATATTCAGTAAATCAAGTAATGAGGCAAATCCAGCTTTGGAAGAGTAAAGGAAAAATAACGACAAAT
ATGAGTGGTAATGAGGCTATGAGACTTATGAATATCGAAAAATGTAGGTGGGAAATTTTTGCGGCTTCAAGACTT
CGAAATAATGTTCATATTGTGCGACTAATAGAATGCTTGGACTCTCCTTTCAGCGAATCTATCTGGATAGTCACT
AATTGGTGCAGCCTTGGTGAACTACAGTGGAAACGTGACGATGATGAAGATATTTTACCGCAATGGAAAAAAATT
GTGATTTCAAATTGTAGTGTTTCTACATTTGCCAAAAAAATCCTGGAGGATATGACAAAAGGGTTGGAATATTTG
CATTCTCAGGGTTGTATTCATCGTGATATCAAACCGTCCAATATTTTATTGGATGAAGAAGAAAAAGTAGCGAAA
CTTTCTGATTTTGGAAGTTGTATTTTCACTCCCCAATCATTACCTTTCAGCGATGCTAATTTTGAAGATTGTTTT
CAGAGGGAATTGAACAAAATTGTTGGTACTCCGGCATTTATTGCACCAGAGCTATGTCATTTGGGCAATTCCAAA
AGAGATTTTGTGACGGATGGCTTTAAGTTGGATATTTGGTCATTGGGAGTGACACTATACTGCTTACTGTACAAC
GAGCTGCCATTTTTCGGGGAAAATGAATTCGAAACCTACCACAAAATCATCGAAGTATCATTGAGTTCCAAAATA
AATGGTAATACTTTAAACGATTTAGTCATTAAAAGGTTATTGGAGAAAGACGTTACTTTACGCATAAGTATTCAG
GATTTAGTAAAGGTTTTGTCGCGTGACCAGCCCATAGATTCTAGGAATCACAGTCAAATTTCATCGTCCAGTGTG
AACCCCGTAAGAAACGAAGGTCCTGTAAGAAGATTTTTTGGTAGGCTACTGACTAAAAAAGGAAAGAAAAAGACC
TCAGGAAAAGGGAAAGACAAGGTATTGGTATCTGCAACTAGTAAAGTAACACCTTCGATACATATCGACGAGGAA
CCGGATAAAGAATGTTTTTCGACTACGGTCCTTAGATCTTCGCCAGACTCGAGCGATTATTGTTCATCGTTAGGG
GAGGAAGCCATTCAGGTTACGGATTTCTTAGATACTTTTTGTAGGTCAAATGAAAGCTTACCTAATTTGACTGTG
AATAATGATAAGCAGAATTCGGACATGAAAACTGACAGAAGCGAGTCATCCTCTCATTCGTCATTGAAAATCCCA
ACACCTATCAAAGCCATGATAAGACTAAAGAGTTCCCCTAAAGAGAACGGAACAGAACCCATATTAATTGCTCA
CAGGACAAACCGAGTTCCCCACTAATGGATAGGACTGTTGGAAAGCGCACGGTTAACAATTCAGGGGCTAGAAAG
CTTGCTCATTCAAGTAATATTCTCAACTTTAAAGCATACATAAATTCGGAAGATAGTGACATTCGAGAAACAGTA
GAAGATGTAAAAACGTATCTGAACTTTGCAGATAATGGTCAAATATAG

>YBR298C 0.72 Cold
ATGAAGGGATTATCCTCATTAATAAACAGAAAAAAAGACAGGAACGACTCACACTTAGATGAGATCGAGAATGGC
GTGAACGCTACCGAATTCAACTCGATAGAGATGGAGGAGCAAGGTAAGAAAAGTGATTTTGATCTTTCCCATCTT
GAGTACGGTCCAGGTTCACTAATACCAAACGATAATAATGAAGAAGTCCCCGACCTTCTCGATGAAGCTATGCAG
GACGCCAAAGAGGCAGATGAAAGTGAGAGGGGAATGCCACTCATGACAGCTTTGAAGACATATCCAAAAGCTGCT
GCTTGGTCACTATTAGTTTCCACAACATTGATTCAAGAGGGTTATGACACAGCCATTCTAGGAGCTTTCTATGCC
CTGCCTGTTTTTCAAAAAAAATATGGTTCTTTGAATAGCAATACAGGAGATTATGAAATTTCAGTTTCCTGGCAA
ATCGGTCTATGTCTATGCTACATGGCAGGTGAGATTGTCGGTTTGCAAATGACTGGGCCTTCTGTAGATTACATG
GGCAACCGTTACACTCTGATCATGGCGTTGTTCTTTTTAGCGGCTTTCATTTTCATTCTGTATTTTTGCAAGAGT
TTGGGTATGATTGCCGTGGGACAGGCATTGTGTGGTATGCCATGGGGTTGTTTCCAATGTTTGACCGTTTCTTAT
GCTTCTGAAATTTGTCCTTTGGCCCTAAGATACTATTTGACGACTTATTCTAATTTATGTTGGGCGTTCGGTCAA
CTTTTCGCTGCTGGTATTATGAAAAATTCCCAGAACAAATATGCCAACTCAGAACTAGGATATAAGCTACCTTTT
GCTTTGCAGTGGATCTGGCCCCTTCCTTTGGCGGTAGGTATTTTTTTTGCACCAGAGTCTCCATGGTGGCTGGTT
AAAAAAGGAAGGATTGATCAAGCGAGGAGATCACTTGAAAGAACATTAAGTGGTAAAGGACCCGAGAAAGAATTA
CTAGTGAGTATGGAACTCGATAAAATCAAAACTACTATAGAAAAGGAGCAGAAAATGTCTGATGAAGGAACTTAC
TGGGATTGTGTGAAAGATGGTATTAACAGGAGAAGAACGAGAATAGCTTGTTTATGTTGGATCGGTCAATGCTCC
TGTGGTGCATCATTAATTGGTTATTCAACTTACTTTTATGAAAAAGCTGGTGTTAGCACTGATACGGCTTTTACT
TTCAGTATTATCCAATATTGTCTTGGTATTGCTGCAACGTTTATATCCTGGTGGGCTTCAAAATATTGTGGCAGA
TTTGACCTTTATGCTTTTGGGCTGGCTTTTCAGGCTATTATGTTCTTCATTATCGGTGGTTTAGGATGTTCAGAC
ACTCATGGCGCTAAAATGGGTAGTGGTGCTCTTCTAATGGTTGTCGCGTTCTTTTACAACCTCGGTATTGCACCT
GTTGTTTTTTGCTTAGTGTCTGAAATACCGTCTTCAAGGCTAAGAACCAAAACAATTATTTTGGCTCGTAATGCT
TACAATGTGATCCAAGTTGTAGTTACAGTTTTGATCATGTACCAATTGAACTCAGAGAAATGGAATTGGGGTGCT
AAATCAGGCTTTTTCTGGGGAGGATTTTGTCTGGCCACTTTAGCTTGGGCTGTTGTCGATTTACCAGAAACCGCT
GGCAGGACTTTTATTGAGATAAATGAATTGTTTAGACTTGGTGTTCCAGCAAGAAAGTTCAAGTCGACTAAAGTC
GACCCTTTTGCAGCTGCCAAAGCAGCAGCTGCAGAAATTAATGTTAAAGATCCGAAGGAAGATTTGGAAACTTCT
GTGGTAGATGAAGGGCGAAACACCTCATCTGTTGTGAACAAATGA

>YNL267W 0.79 Cold
ATGCATAAAGCATCCAGTTCAAAGAAAAGCTTTGATGATACTATTGAACTAAAAAAAAAATGAACAATTACTGAAA
CTGATCAACTCTAGTGAATTCACCCTTCACAATTGTGTGGAGTTACTGTGCAAACACTCCGAAAATATTGGTATA
CATTACTATTTATGTCAGAAATTGGCCACATTTCCTCACAGCGAACTACAGTTCTATATTCCCCAACTAGTACAG
GTCCTCGTCACCATGGAGACAGAATCAATGGCTTTAGAAGATTTACTATTAAGGTTGAGGGCGGAAAACCCTCAT

TTTGCACTACTGACGTTTTGGCAGTTACAAGCGTTACTAACGGATTTATCTACTGACCCCGCTTCTTATGGCTTC
CAAGTGGCTAGAAGAGTCTTAAACAACTTACAAACCAATCTCTTTAACACAAGTTCAGGTAGCGATAAAAATGTT
AAAATACATGAAAACGTTGCACCGGCCTTAGTTCTTTCCTCTATGATAATGTCGGCTATAGCATTCCCCCAATTA
AGCGAAGTGACCAAACCATTAGTGGAATCTCAAGGTAGAAGACAAAAAGCTTTCGTTTTTAAGTTGGCTAGAAGT
GCAATGAAAGATTTTACCAAGAACATGACCTTGAAAAATACCCTACTAAACAAGAAAACTTCCAGATCCAAAAGA
GTTAGCTCAAATCGCAGTTCAACTCCGACTTCTCCAATAGATTTAATAGATCCAATAAAAACTAAAGAAGATGCA
TCCTTCAGGAAATCCAGACATAGTGAGGTAAAATTGGATTTCGACATTGTGGATGATATAGGTAACCAGGTCTTT
GAAGAAAGAATCTCATCTTCAATCAAACTCCCAAAACGTAAACCTAAGTATTTGGATAATTCATACGTTCATAGG
ACATATGATGGCAAAAATATAAACAGGGACGGAAGCATTTCAAATACCGCAAAGGCTCTCGATGGAAATAAAGGT
GATTATATTTCTCCAAAGGGACGTAATGATGAAAATAATGAGATTGGTAACAATGAAGATGAAACTGGTGGTGAA
ACGGAGGAGGACGCGGACGCTTTAAACTCTGATCACTTCACCAGTTCTATGCCAGATCTGCATAATATTCAACCA
AGGACTTCTTCTGCTTCATCTGCTTCTTTAGAAGGGACACCTAAGTTAAACAGGACCAATTCCCAACCCCTTTCG
CGCCAGGCATTCAAAAACAGTAAAAAAGCGAATTCTTCTTTGAGTCAAGAAATTGACTTGTCGCAATTATCGACC
ACTTCAAAAATAAAAATGTTAAAAGCAAATTATTTTCGTTGCGAGACACAATTTGCCATTGCGCTAGAAACAATA
TCTCAAAGGCTAGCTCGAGTACCGACAGAAGCTAGATTGAGTGCCTTACGAGCCGAGTTGTTTCTATTAAACAGG
GACCTACCAGCGGAAGTAGATATCCCCACTTTATTGCCTCCAAATAAAAAAGGAAAGTTACATAAATTAGTGACC
ATTACTGCTAATGAGGCACAAGTTTTGAACTCTGCAGAAAAAGTTCCATATTTACTCCTGATAGAATACTTGAGA
GACGAATTTGATTTTGACCCAACAAGTGAAACCAATGAAAGATTATTAAAGAAGATCAGTGGTAATCAGGGAGGC
TTGATATTTGATTTAAATTACATGAATAGAAAAGAAAACAACGAAAATAGAAATGAAAGTACTCTTACTAGCAAC
AACACTCGATCTTCGGTATATGATAGTAACTCGTTCAACAACGGGGCCTCCCGCAATGAAGGTCTATCCAGTACC
TCAAGAAGTGATTCAGCGTCCACAGCTCATGTTAGGACTGAAGTCAATAAAGAGGAAGATTTAGGTGATATGTCA
ATGGTAAAAGTCAGGAACAGAACGGATGACGAAGCGTATAGAAATGCTTTAGTAATACAGAGTGCCGCAAATGTT
CCAATTTTACCTGATGATAGTCAAGACAGAAGCCCAGAGTTGAACTTTGGCTCAAACTTGGACGAAGTACTCATC
GAGAATGGAATTAATAGCAAAAATATACATAGCCAAACTGACGCTTTAGCAGACCAGATGAGAGTTTCAGCTGTT
ATGTTAGCACAATTGGATAAGTCGCCACAACAGTTATCAGAAAGTACCAAACAAATCCGCGCTCAAATTATTTCA
TCAATGAAGGAGGTGCAGGATAAATTTGGTTACCATGATTTGGAAGCCCTTCATGGAATGGCAGGTGAGAGAAAA
TTGGAAAACGATTTAATGACGGGTGGTATTGACACTTCATATCTAGGTGAAGATTGGGCTACCAAGAAAGAGAGG
ATACGTAAAACTTCGGAATATGGCCATTTCGAAAACTGGGATTTATGTTCTGTAATCGCCAAGACGGGTGATGAT
TTGAGACAGGAGGCGTTTGCATACCAGATGATTCAAGCGATGGCCAATATTTGGGTTAAAGAAAAAGTTGACGTT
TGGGTTAAAAGAATGAAAATTTTAATTACTAGTGCGAATACGGGACTTGTGGAGACCATCACAAATGCTATGTCT
GTGCATAGTATTAAAAAGGCTTTAACCAAAAAAAATGATTGAAGATGCAGAACTAGATGACAAGGGTGGTATTGCC
TCTTTGAATGATCACTTCCTTAGAGCTTTTGGTAATCCTAATGGATTTAAGTATAGAAGAGCACAAGACAACTTT
GCTTCTTCGTTAGCCGCATATTCTGTCATTTGCTATCTCTTGCAGGTTAAAGATAGACACAACGGTAACATTATG
ATCGATAACGAAGGCCATGTAAGTCACATCGATTTTGGATTTATGCTATCAAATTCACCCGGCTCAGTGGGCTTT
GAGGCCGCACCATTCAAATTAACTTACGAATATATTGAACTGCTAGGCGGAGTAGAGGGAGAAGCGTTTAAAAAG
TTTGTTGAACTAACTAAAAGTTCGTTCAAGGCTCTGAGAAAGTATGCTGATCAAATCGTATCAATGTGTGAGATT
ATGCAAAAGGACAATATGCAGCCTTGTTTCGATGCTGGCGAACAAACAAGTGTACAACTACGACAAAGGTTCCAA
TTGGACTTATCAGAAAAAGAAGTTGATGACTTCGTAGAAAATTTCTTGATAGGTAAATCTTTGGGTAGTATTTAC
ACCAGAATATACGACCAATTTCAACTTATTACACAGGGTATATATAGCTGA

>YDR239C 0.75   Cold
ATGTTTGATGGCTTTAGCAACAATAAAGGTAAACGAAGGTCTTTTTTTCGGTTTGGTTCTGAAAGTAAAAACAAT
GACTCTGAAAAATCCGTTAGAAAGCCATCGGTGCCCTCTACAATAAAGAAATCAACAAATATCGCACGAACTTCG
ACGGCTGAAACTTCTGCCCCTGATATACCGCCAAGAAGTCCAAATAGGAATGCTCATAGCAGGAGCCATTCTATC
CAGGCACCATTACAAAAGGAAACATTAAAGAATACTAATCCATTTTTGAATGCTGAGGATACCTTAGGAGATTCT
CTTGAATTAACACAGTCCAAGGAAGCGTCAGGAAACGATCACAAAGGAATTGAATATTTACAAGAAAACAACATC
ATTGGGCAGAGAACCAACCCCTTCACAACAAGCGCAAATTCAAATGCACATTTCTCCAAGATAAAACGTTCGAGG
CCTCCACCACCGCCTATGGACATGAAGTCAATAACAACGTCAATAAGCAACAATACTACTAAGGAAGAAATAGAG
AGTAATAATGATTCTGAGCGGGATAGTATTGCAATCTCATCCACCCACAATCAACATAGGCGGCAGCGTTCAGAA
GCAGAAAAGTTGGTAGATGATATTGAAAATTACATTAATGAACACAAGGTAAGTTCCGGTAGCTCACTATCCCTA
GACACGTCTGAAAACTCAGATACTAAAGCATCGCAAGATAAGTTGCCAGTAGATGTCATGGAAGCTCCCATTTTG
AGAAATGTCAGCGCGGAGAGTTCCTTAAGCTACGTAAAACCACTTATTGTGGATAATGAAGAAGTAAATAAAGCA
AGTAACGGAAATTTAGTACAATCAGATCATCTAAAAGAATTTTCTTCGAATCTGGATGATGGGGATGATAAATTT
TCCTTTAGTACATCGGCTAGTGGTAAATCCACCAAAAGCCTTCAGCAAGTTTCTAAAGACGAATCTAGTGGTTTT
AAAGCTGCACATTTTGATTTTGCATATAAATCAAATGAGCACCTTGGGAGTGATGGAAGTATTGCAAGCGCCAGA
AAGCCTCTGCGGATAACTAATGAGATTGATAGCGGTTCTTCCAACGAGGATGATGATGATGGTCTTCAGGAAAAA
GGTTTTGTGGATAGTGAAAGTAAAGCATTTATAAATTACGCAAGTGATCAAGGCTCATCTATAAAAAATGACGTC
TCAACACAAGAGCCTGAGTTACCAAGTCATCGTAGAATATTTAGGGTGGTTAATGAAGATCGCCCTAGTTTCTAT
TTAAATAGTGTAAACGATACGGGCAGTCTAACCGACAAGCATTCTTTTGACACTGCATCATCTGGTGAGTATGAT
GCCAAGTCAAACTTCTCTAGCCAGTCAGGACTTTCTATATCAAAAGGCTCTAAATCAACTGTTTTGGCTGCTCTT
GACTCAAACGGAAACACAAAATCATCAAATAAAACATCCGAGTTAAACAGTTTAAACTCCATTTCAGAAAGTTTA
GTGCCGGCTGCTCACTCGTTCAATGAACACACAGTTACTATCCCTGCAACGGTGGATTTACCAAATCCGGTACAT
GATGCCCCAAGCGAAAGATCTGTCAAATGTTCACCGTTAACATCTGTCGTATCGAACAAATCGGAAAAGTCCGTG
CCTTTGGTTTCTAGTTACGTAGAAGAACTGCGACTAAAGTATTACAAGACTTCTAACTTTTTACAAGCGCCTCCT
AACTTGCCGGTTGCTCTAAAACAAAAAAATAATTTGATTCAACCCAAGAATATTAAGGTCAAGTTGAGGACAAGT
TCTAAGCAAATTGGTATCAAGCATGGTAAAGTTAAACAGAAATTACTGGCGTTGGAGACAAGAAATGAAGAATCA
GATGGAACCGCTACAGGGCTGAAAAATAAAATCAATGTTGACCACACAAAGGAATTCCATAAATTGTTAGGGAAG

GAAAATGAAACTGGCTCTATTTCAAAAAAAGAAGGCACTGACGCAGAACAAGCCGAAGACTACCTCAAGGACATA
CCCGGTGATGAAGCATATAACAGTGATGATATAATGGCGCCTTTACGAGAAAAAAGAGGGCAAAACGGTTCTGTT
GATTCCGTGTCAAGATCTAACACGGTAGTCAGCTACTACACAAGGTCTCAGAACAGAATGAGAAGTGGAACACTG
GATAATGATTACGTGAACAGACAAAAGCTTCCTACACATATCTCTCTTCAAGATTATCGTGATGCTAATGCTAGA
AGTAATATATCGCGTCAGGACTCTGTCTCCACAACGAACTCTGATGTGGTAGACCTCAGCTATTCTCTGGGGCAT
GGCTTGCGTGTGGCAAACCCTGATTCAGACCCAGAATGA

>YOL143C 0.76 Cold
ATGGCAGTTAAAGGATTAGGCAAACCAGATCAAGTTTATGACGGTTCCAAAATCAGGGTCGGTATCATTCATGCC
CGTTGGAACCGTGTCATTATTGACGCTCTTGTGAAAGGTGCTATTGAAAGAATGGCTTCCCTTGGTGTCGAGGAA
AATAACATAATAATTGAAACTGTTCCTGGATCCTATGAATTACCTTGGGGGACAAAAAGGTTTGTTGACAGACAA
GCGAAGTTGGGAAAGCCGCTAGATGTTGTAATTCCTATTGGTGTACTTATCAAAGGTAGTACAATGCACTTTGAA
TACATTTCAGATTCCACCACTCACGCATTGATGAACTTACAAGAAAAAGTCGACATGCCCGTCATTTTTGGCCTC
TTAACTTGCATGACTGAAGAACAAGCCCTGGCCAGAGCTGGCATCGATGAAGCGCACTCCATGCACAACCATGGT
GAAGATTGGGGTGCTGCAGCAGTGGAAATGGCTGTTAAGTTCGGTAAAAATGCTTTTTGA

>YOL154W 0.76 Cold
ATGAAGTTCTCTTCCGGCAAATCTATCATCTTTGCAACTATTGCTTCTCTAGCTTTGAGTGCTCCTGTCACTTAC
GACACCAACTCTACTGCTGAGTTACAATCTCCTTCATCTCAAGAAATTCTGGGTTGGAGTCACGCAACTTTTCCT
ACCATTTACCAAACCTGTAATGAGACGAACGCAAGAATGTTGAATGCAGCTTTTAAGGATACCGCTGAAATCACC
GCTTATGGTAAAGATAGACTTTTGAACTATGGTGTCGATGACGTTTACTACAAAAGATGGTTTGGTAATGGTAGT
ATTTTCACCGTCATGGGTGTCTTTGAGCAATTGATGGAGGCTTCCAAGGGTGCCATGCTCATGAGATGTGATGAT
ATTGATGGCTTGTGTGCAGCTAATCCAAACTATTACGCTGGTCATCACCGTCAATCTGCTCCAGCTGAAACTGTT
ATTTGTGATTACTTCTACACTTCCAAAAAGCCACTATCAACAATTTGTTTCGAAGGTACTATTGTCGATGTCGGT
CCAAAACATTATGCAGGTATTGATATGTTACATCGTTACTTGCACGTCCCTACCATGAGTATGGATGGATATGTT
GGCGAGTACGCGGAAACTCTTGAAGAAGTTGTGGACTACACCCAGAACAATGCTACTTACGCAGTTAGAAACACC
GACAACTATCTTTACTATCTCGCTGACGTTTACAGTGCTTCTGTTATACCTGGTGGCTGTCTAGGTAACTTGTAA

>YDL118W 0.81 Cold
ATGTTTGAATCGTTTGTTAATGAAGGCACAGCATTCCTCCTCTTTGCCAAAGATGAGCGCATAAGGTTCAAACAT
GATAAATATAAGGCGCTGCCTATTGATGTTCGTAATGCTGAAGGTAATGTACCTCTCCATAGCTGTAGAGGGCTG
TCTATCTCTTTCAAATTTTTCCATAATGTAGCTTTCTTGTCTTGCTGGATTCTCGTCTTCAAAAGGTCTAAAGGT
TGCAACGCAACAGCAGATGTTAGCCCACCGAAAAAGCCCCCGATCAAATGCGATGAATTTCTTGGCTTAGTTGCT
TGCTCAGTCATGTATGCTGCTTCACAAATTGTGTACCTTCTAGTTGTTCCCGCTGTGCTATTTGCTTTTCTTTTG
GTATAA

>YGR099W 0.79 Cold
ATGGTTTTAGAAACGCTGAAACAAGGGCTTGACAGCAGTCAAATACATGAAGCTTTAATACAACTAGATTCCTAT
CCTCGCGAACCGGTGGATCTAGATGCCTCGATGGTTCTCATAAAATTCGTAATTCCAGTTTATCCATCACTACCA
GAAAGATCGAAGGTGATATTGAGACGGCTAGCTTCTAAATCGTTTACATTTTTATGCCAAATTGTCACCTTCTCA
AGGACAATAAGTGGCCGCGATGGACTGCAAGAGATACGCATATACCAAGAAATTTTAGAAGACATCATCAGTTTT
GAACCGGGATGTTTGACTTTTTATTTAAAGGCAAGCACTACTAGCAAAGCAGACCGTGATAGCATTAAGGCCCTT
TTCTTTGGGAGCAAGTTATTTAATGTGTTGGCCAACCGAATTGATATGGCGAAATATTTGGGATACCTTCGGCTT
CAGTGGAAATTTCTACTTGAGAGTAATGAAACAGATCCACCTGGGTTTTTAGGGGAATGGTTAGTTTCATCATTT
TTGCTTAACCCAGTGCTAGCGGCAGATATGTTATTAGGCGAATTATTTTTATTGAAAGAGTCGTATTTCTTTTCT
TTTCAGAAAATCATATCTGCTAGTAGTCTGATAGATCAAAAGAGGCTGATTGCTAAGTTTTTATTACCATATATT
CAAGTTATCGTGACTTTAGAAAATTTGAACGATGTTAGGAAAATTCTGCGGCGGTTTGATCTCGATAAAATCATA
AGTCTGTCCGTATTATTTGAAATACAGTCGCTGCCATTGAAGGAAGTGATAGTACGTCTGATGAGCAACCACTCT
TCCACGAAGTTTGTTAGCGCTTTGGTAAGTAAATTTGCTGATTTTACAGATGAGGAAGTAGACACAAAGACATGT
GAATTGCTTGTACTCTTTGCAGTGCACAATCTCAATCATTCCCAGAGAGAGGAAATAGCACACGATGAGCGGTTT
TTGAATGGCGTGACCAAACATTTAGGTAGCAATGAACGTGAGGCAAGGGAGCGTGCGATGTTTATTGCCAAGCTT
TTATCAGGCGGTCATCTGAAGTACGAAAGTGATTTTAAAATCAATATACCGAATGTCAAATTCGAAAGCAATAGC
GATGATAAAATCATTGATTTCCAATCTTTAAAAAATCCATCCATATGCAATACCCAAACTGATGTGGGTAAGGAT
AAGATTACTGAGGTCTCGGGTCATGTTCAAAGCCTTACATTGGACTGCAGTGATAGCGATGATGAAGATGAGAAC
GATGAGCGGGAGATTGTTAAACGAATTGTATTTTTAAAAGATTTAATGAAAGAATACGAAAAAACTGGGGAATCT
CGAAAAGCTCCATTGATACCACTTCTGAAGCAAACAGTGAAATTGATACGGCAAAAGGCGGATTTTCAGTTAGAG
GTTGGCTATTATGCACAAGGTATTCTGTCAAGCATTGTGTGCTTGAATAATGAATTTGATGAACCGCTTTTCGAG
CAGTGGAGAATAAACGCTCTAACAAGCATACTGGTCGTCCTTCCAGAGAAGGTGAATGGCGCTATAAATATTCTA
TTCAATTCAGAACTGTCGTTACAGCAAAGGATGTCACTATTGTCGGCTCTGGGTCTTTCTGCAAGAGAGTTGAGA
GGGCTTGATGATCCTACTATTGTCAAGCCCAAGTTTGATTTTCCAACAAATCGTTTACCGTGGGATGATCAAAGC
CATCATAATAGTAGGCTTGTTGAAGTTCAAGAGTCAACAAGTATGATAAAGAAGACGAAAACAGTATGGAAATCT
AGAAAACTAGGCAAGGATCGAGAAAAAGGAACGCAAAACCGCTTTAGAAAATATGCTGGTCTATTCTTCTATCCG
CTAGCACACGGTTGGCTTAATGGAATTGACGTAGGCACATACAATCAGCTTTTTAAGTCGCATTATTTAACAACA
CTGCGTATCATATACTCTTGCGCTAACCCGGTCCACGATTTTGAATCCATGACGGAGTTGATGAATCATATAATT
AGTTCAGCCATAGAGGAAGGAATTTCTCTCAATAAAGGTTAG

>YNL233W 0.76 Cold

ATGTCGGATAGTATTTCAGATTCAAAGTCCTCAGAACTTTTAAATTCTACTTTTTATTCGTCGACGTCGATAAAT
ACACTTGATCATGCTAGAACCTTTCGAAATTCTCTGATATTGAAGGAGATATCAGATCAAAGTCTAAATTCATCA
ATAAAGCCATGCGAATCCGTCCTTGATAGGGATGTTGAAAGCTCAGTTCTGCAGCGGTCGTTTGGAGAAAGTAAT
GCCCGAGACTCAGAAGTACAAACGGTAAATATGACAACTTCCCCCTCATTAAGTGCATTGGCTGATATTCTGAAC
GAAAGATCCAAATATGCTGACCAAAAAACTAGAAAGGCACAAAATATTGAGTCTTCCATAATAGAAGAAGAAGAA
GAGGCCGAAGAGCAAAACAACTCGATCAATTATCACGAGGATATAACTGGATCCAGGCTGTCTGTACGAGAGGAA
GCAAATGAAAACTTGGCGATGACCTCTCCAAACCTTATTGACATTGACGGATCCAATAGTATTCAAGTTGCCCCT
TTGAGTTTACCGTCTTTCGAAGAACCTGACTTTTTGTCCACTCCTAGAGTAAAACCTGATTCTCAGGGACCTCGG
AGCAAAGTTAGTACACGACGTACTATTCTCGAACGAGACAACAATTTACCAGTTAAGCGGGAGGAAAATACTATC
ATTAATTCAGAAACTGAAAGCACAACACATAGCGCACCATTTTTAAAAGAGGATCCGAAACCCTCGCCTCCTTCG
AGTAAATTATACAACCCAAAAGTTCGTTTAAACAAAGCAGAAGCGCGAAAGTATACTGATTCTTCTGCCCAAAGG
ACTACTTCAGCAGGCTCGGTACTTGAAGACACTTCTATGCATAAAAAGAAAAAGAGTATATTTTCATTTCTGAAG
AAGAAAGAACCGAAACCAGTTATTGGTAATAATAGTGTAACGAATGAAAAAAAATAAGATGTCTTCATCGAGCACT
TTCTCAATGAATATTCAAACCTCTTTAAAAACGCCTGAAAAACTAAAGAAGAAGTCTCACAGTAGCAGTAGCATT
TTCAATTCCTTCCTAAAGGGAAAAATTGAAACATCAGACTCACCTCGAAAGGAACCAATGAGGCAAAAAAAAAGA
ACCCCAAAATCCAAGGACAAAAAGCAAGATACTGAACAAATAATTGACGCCGCATCAGTGTTATCCACTGAATCA
CCTCTACTACGAAAAAACCACGACGATACTCCCGTGAAAATAGATCACGTAACGAGATCAATTGATCAGAGAAAA
CCTACTCCTTTAAACATGGATCTCATATTGGGAGGTGATAAACAGATAAATACCCCGCTACAAGAACACGTAAGA
GAAGATGATGATGCCAAAAATGATCTTCAATTACCAACCAAGGATAATTTTTTATCACTGGACTACGAAGCACCC
TCACCAGCATTTTCCAAACACGATACAGGTGAGGTTCTTTTCCCAAAATTTTTGGATAATCATGAAGTCGACTCG
ATCGTTTCTTTAGAAAGAACAAGATCTACAAAGTCTAACAAGAGAAGTTCAATGAACTCGCAAAGAAGGTCATTG
ACAGATACGCTATCGATCAAAGCGCAAAGTGAGGGAATGTTCATCACTGAGGCATCTTCTGTTGTATTATCTACT
CCAGACTTGACGAAGTCTCCTGCTAGCAGCATTTTAAAGAATGGAAGGTTCGAATACTCAGATAATTTCTCCAGA
GAACACTCTTACGAAGGTACCACGAATGAGGACTTTTTAGATATCAAGGACGATAGTGGTCCACTAAAAAAAGAT
GACATATTTTTAGAGTCTATTGAACAAAAATTTGACCAGCTAGTGATGGCCTCTGATGAAGAAAAAACTGAAGTT
GAAAGGGATGTACCAAAACCAAGAGAAGAACCTCTGAAGAAGGATTCTGAAAGGCAAAGCGTTTTTGCAGATGAT
GATAACGAGTTAATTTCTGATATAATGGAGTTTGCAAGTTTTATTAACTTCGGTGATGATGATCTCAATCTAGAC
CTAGACTTGGGAGATACAACGGCCTCATATGCAACAGAAACCCCTGAACCTGTTGGAAATGATGAAGTAAATCGC
TCTGGTACTTTTGATACACGAAATAACAAAGAGGATAGTTACAAAGAAAGAGAAACTCAATCTTATTCCGCTGCT
GGAGCTACTACATACGGAGATGAGCGTCAAGGTCAATTACATACATTTGAACAAGACGGAAGTGAAATAAATGAC
AACGAATTTGAGAATGAAGATTTCAACAAACACATAGAACAGCCCATAGAAGTTACTCCAAGAAATAATGCATAT
TTACCAGAATTCGAACCCAATAGACCTGTTTCAATGTCCTTTAAAGGACTAAAAGCCCCAAGAATGAATACTTCT
TTTATTGACTCCATGACGCCTGATTCTCCCGTAAAATCAGACTTAACAAGTCTAGGTGAGGTGTATGTAAACAGT
AATAATGACCAGGGTGTCCGGTTTTCGTCCCAGATAATACTTTACGACACTTATGGAGAATTCGAATATGATAGA
CATCCAGAAATTTCCACTTGTAACCAGCTTACTCCACAACTGGCTCAAATGATCAAATTGGAATTAAATGAGTTA
AAGAGCGCAATGGAAGTACACGATGATTCGCGATGTTACACACATTTTTATTAG

>YLR182W 0.79 Cold
ATGGCGTTGGAAGAAGTGGTACGATACTTAGGACCTCACAATGAGATCCCTTTGACACTCACTAGAGACTCGGAG
ACTGGCCATTTCCTCCTGAAACATTTTCTGCCCATTTTGCAGCAATATCATGACACGGGGAATATTAACGAGACC
AACCCCGATAGTTTCCCCACTGATGAGGAAAGAAATAAATTACTGGCACATTATGGAATTGCTGTAAATACAGAC
GACCGAGGTGAGTTATGGATAGAGCTGGAAAAATGTTTACAATTATTAAACATGCTAAATTTATTCGGTTTGTTC
CAGGATGCATTCGAATTTGAGGAGCCTGAAACAGATCAGGATGAAGAAGATCCCAGCCATTCGAAACTACCAGAA
AACAAGACCAAAAGTGAAAATTCTAAGGATAATATCAGCTCAAAGAGGATTAATAATTTACAAGATATGAGCCTG
GATTCTGATGCACACAGAGAATTAGGCTCTCCTTTAAAAAAACTAAAAATAGATACCTCTGTAATAGATGCTGAG
AGTGACTCCACTCCGAATACTGCCAGAGGCAAGCCTAACGATGATATTAATAAGGGCCCTAGCGGCGACAATGAA
AATAATGGCACTGATGACAATGACAGAACCGCTGGACCTATCATAACATTCACTCATGACCTAACTTCTGACTTT
TTAAGCAGTCCACTGAAAATCATGAAAGCACTACCTTCTCCAGTTGTAAATGATAATGAACAGAAGATGAAACTA
GAGGCATTCTTACAACGGTTGTTATTTCCAGAAATTCAAGAAATGCCTACATCCCTTAATAATGACAGCAGTAAT
AGAAATTCAGAAGGGGGGGAGCTCAAACCAACAACAACAGCACGTATCATTTGATAGCCTTTTGCAAGAGGTAAAC
GACGCTTTTCCTAATACTCAATTAAATCTTAATATTCCTGTAGATGAGCATGGAAACACACCATTACATTGGCTG
ACTTCAATAGCAAACCTGGAATTAGTGAAACACCTGGTTAAGCATGGTTCAAACAGATTATATGGTGATAATATG
GGGGAGTCATGCCTAGTGAAAGCTGTCAAATCAGTAAATAATTACGACTCTGGTACTTTTGAGGCACTTCTAGAT
TATTTATATCCATGTTTAATTTTGGAAGACTCAATGAATAGAACAATTTTGCACCATATCATTATTACGTCTGGT
ATGACTGGCTGTTCAGCAGCTGCGAAATATTATTTAGATATTTTAATGGGATGGATTGTCAAGAAACAAAATAGA
CCCATTCAAAGTGGTACTAACGAAAAAGAAAGCAAACCGAATGACAAAAAATGGGGAAAGAAAGGACTCTATACTA
GAAAATTTGGACCTGAAATGGATAATAGCAAACATGCTCAATGCGCAGGACTCTAATGGCGATACTTGCTTGAAC
ATTGCAGCAAGATTGGGAAACATTTCAATAGTAGACGCTTTATTAGATTACGGTGCTGACCCATTTATTGCAAAC
AAATCAGGGTTGAGACCCGTGGATTTTGGGGCAGGTACTTCAAAATTACAAAATACGAACGGCGGTGACGAAAAC
TCGAAGATGGTCTCTAAGGGTGATTACGACGGTCAAAAAAATGGAAAGGCCAAAAAAATAAGGTCTCAACTGTTG
AAAAACCCACCTGAAACAACTTCGTTAATTAATGATGTCCAAAATTTACTGAATTCAATCTCGAAAGATTATGAG
AATGAAACAGTGCAATATAATGAGAAATTAGAAAAACTACACAAGGAATTGAACGAACAGCGAGAAGAATTAGCT
AATTCTAGAGAACAACTAGCAAATGTGAAGCAATTGAAAGATGAATACTCACTAATGCAAGAGCAATTGACCAAT
TTAAAAGCAGGAATTGAAGAAGAAGAGGAAAGTTTTAGGGAAGAGAGCAAAAAGCTAGGAATAATTGCAGATGAA
AGTTCAGGTATTGATTGGGACTCTAGCGAATATGATGCAGATGAACCCTTCAAAGTAGAGTTCCTTTCAGATTTC
TTGGAGGATAAATTACAAAAGAATTATGAAGGCGATATTTCCAAACTATTAGAAGCGGAGTCGAAAGAGCAGATA
ATGGAACAGATACGAAATCAACTTCCCGCAGAAAAAATCCAATCGATGCTTCCACCAACGGTCTTATTGAAGGCA

AGAATAAATGCATACAAGCGGAATGATAAACACCTAACCAATGTATTGGATACAATCAGCACCAAACAATCAGAA
TTAGAGAATAAATTTAGAAGAGTGTTATCTTTATGTTTGAAAATCGACGAAAATAAAGTTGACAATATGCTTGAT
GGTTTGTTACAGGCCATATCTTCTGAAGACCCACAAGACATTGACACTGACGAAATGCAAGATTTTTTAAAAAAG
CATGCTTCATGA

>YOR002W 0.76   Cold
ATGGCCATTGGCAAAAGGTTACTGGTGAACAAACCAGCAGAAGAATCATTTTATGCTTCTCCAATGTATGATTTT
TTGTATCCGTTTAGGCCAGTGGGGAACCAATGGCTGCCAGAATATATTATCTTTGTATGTGCTGTAATACTGAGG
TGCACAATTGGACTTGGTCCATATTCTGGGAAAGGCAGTCCACCGCTGTACGGCGATTTTGAGGCTCAGAGACAT
TGGATGGAAATTACGCAACATTTACCGCTTTCTAAGTGGTACTGGTATGATTTGCAATACTGGGGATTGGACTAT
CCACCATTAACAGCATTTCATTCGTACCTTCTGGGCCTAATTGGATCTTTTTTCAATCCATCTTGGTTTGCACTA
GAAAAGTCACGTGGCTTTGAATCCCCCGATAATGGCCTGAAAACATATATGCGTTCTACTGTCATCATTAGCGAC
ATATTGTTTTACTTTCCTGCAGTAATATACTTTACTAAGTGGCTTGGTAGATATCGAAACCAGTCGCCCATAGGA
CAATCTATTGCGGCATCAGCGATTTTGTTCCAACCTTCATTAATGCTCATTGACCATGGGCACTTTCAATATAAT
TCAGTCATGCTTGGCCTTACTGCTTATGCCATAAATAACTTATTAGATGAGTATTATGCTATGGCGGCCGTTTGT
TTTGTCCTATCCATTTGTTTTAAACAAATGGCATTGTATTATGCACCGATTTTTTTTGCTTATCTATTAAGTCGA
TCATTGCTGTTCCCCAAATTTAACATAGCTAGATTGACGGTTATTGCGTTTGCAACACTCGCAACTTTTGCTATA
ATATTTGCGCCATTATATTTCTTGGGAGGAGGATTAAAGAATATTCACCAATGTATTCACAGGATATTCCCTTTT
GCCAGGGGCATCTTCGAAGACAAGGTTGCTAACTTCTGGTGCGTTACGAACGTGTTTGTAAAATACAAGGAAAGA
TTCACTATACAACAACTCCAGCTATATTCATTGATTGCCACCGTGATTGGTTTCTTACCAGCCATGATAATGACA
TTACTTCATCCCAAAAAGCATCTTCTCCCATACGTGTTAATCGCATGTTCGATGTCCTTTTTTCTTTTTAGCTTT
CAAGTACATGAGAAAACTATCCTCATCCCACTTTTGCCTATTACACTACTCTACTCCTCTACTGATTGGAATGTT
CTATCTCTTGTAAGTTGGATAAACAATGTGGCTTTGTTTACGCTATGGCCTTTGTTGAAAAAGGACGGTCTTCAT
TTACAGTATGCCGTATCTTTCTTACTAAGCAATTGGCTGATTGGAAATTTCAGTTTTATTACACCAAGGTTCTTG
CCAAAATCTTTAACTCCTGGCCCTTCTATCAGCAGCATCAATAGCGACTATAGAAGAAGAAGCTTACTGCCATAT
AATGTGGTTTGGAAAAGTTTTATCATAGGAACGTATATTGCTATGGGCTTTTATCATTTCTTAGATCAATTTGTA
GCACCTCCATCGAAATATCCAGACTTGTGGGTGTTGTTGAACTGTGCTGTTGGGTTCATTTGCTTTAGCATATTT
TGGCTATGGTCTTATTACAAGATATTCACTTCCGGTAGCAAATCCATGAAGGACTTGTAG

>YPL021W 0.78   Cold
ATGTTATATAACAAAGAGCAGGGAACAAGCGGCGCTAGTAGCTCTGGTCGTAGAACGAAATTCCACTTCGATCGT
TTTGTACAAATGGTTCTGTTCATTGCCGCTAATCCCAATTATTGCTGTTCAGTAGCCAGTATCCCGAAATCTGGT
GTCACGCCAGATTTAAAAAGAGCAGATATTCTTGAACAGAAGATCAAAAGCTTAAATAGTGCGCTGAGTCCAAAA
TTGAAGGAAGAAAGTCGTCTTGGTGGGCCTTTGCATAACCCCTCTATTCTACCAGCCCCATCTTTTTCATCACTA
CCAATATCATCCAATGGAAAGAAATCTTTAGCAGGATATAGACCTAAAAGTAGGAAGAAACAAACTATACTCCCC
AACGGTCAACCAAAAGAGTGCGCCACTTGTGGTGACACCTGGACTTCTCAGTGGAGAAGTGGACCTAATGGAAAT
GTTGAGCTATGTAGTCGATGTGGCATAGCATATAGGAAAAAAATGGAGAAAAAAAATACGATCGCAACAATCCTCC
GATGATGGTACTAAAAATTTTATATTTAAAAATAAATAG

>YJR053W 0.75   Cold
ATGTCAATTAGGCCTCTCACGTTAAACGGTTTAGATGAGCCAGAAACCTCTTTTGAAGAACTGAATACAACTCTA
CCTCGCTTTCAATCCCATGAAACATTAACTTTGGAAGAAAACGTGCCACCATTGAGTACATCAACTTATATACCG
CCTCCATCCTCGGTAGGTACTTCTGACACTGGCACGGTATTTTCCAATAGTACCAGCGCGTTTTGGTCTAATAAG
CAAGCAGACGATGATCAGGACATGGAGGTAGACCAGGATGATGAATTTCTAAATGACTTCCAGGAATTCCAAAAT
AAAAAGGATGATTTTGACGATGCTATTAAAACAAATTTTCATTTGAGAAATGGATGTCGAACTGGCCCTTTCAAA
AACGACATTTTCGCAGAAGAGTTTGATAGAAAACTGAGTTTGGAAGATAAACCAAGGCTAAAGCAACCTAGATCA
ATGATGGAATTGAAACCAAAAAGAAAACTATCAAATAGCGTCACTTCAAGAAACTTAAGGTCTGGTAATTCTGTA
AGATTTAAGAAATCTATGCCTAACTTAGCTTTAGTTAATCCAGCTATCAGAGAAGAAGAAGAGGACGAAGAACGA
GAAAGAGAAGATCAACGAGAGTTTAATTACAAGATTGATAACGATACGCAGGACACAATTTTGGCGAAATTCAGT
TCAGATGATGAAGGTGACTTTCTGACAGGATTTGAAGAATTAGAGGGTGAAGCAATTGATGAAACGATTTCTTCG
AACGATAAAGAAAGTGCCGACCATCCGCGTTTCTTAAAGAAGTCATCCTCATCTTTGCCATTAAAAATATCACCT
GCACAATATGATATAGTAAAGCATGACGAATTATTAACTCCAGGTCTTCATAGGCGACAACGAGATTGGAATACC
CAGCAGGAACTGGACTCTTTTAAAGAAAAACGATCAGTAAGACACTGTTCTAATCAAAATGTACAACTCAATGGT
CCAGCTAAAATCAAAACAATAAAACAGCAAATTGATCATAATACTCCAATGAAGAAAGGTTCTATGATATATAAC
CCGAAAACTATGAAGTGGGAAGGAAATGAAAACGTCTTAAGCAAATTTAGTGATGTAGACACGGCAAACAGGAAA
GCTTTATTGATAAAAAACAAGCTACAACGAGATGCAGATTCTAAGAAGCAAAAATATTCTGATCTACAGCATGCA
AGAGCCACTTCGAGAAATCAAAAGGTCATCGGGAACATGATTTTAGATGAACAAAATTTAAGATGGGTTAGTGTT
TCAGAGGAAGAAGCAGACCCTTTTGCAGGCATTCCTGAGATAAACTTACCACCAGTTGGCAAAAGTATGAAAAAA
CGTAGCTCCTCACCATTTTTGCGTTCCAAAAGCCAAGTTAATACGCCATTTGTTTCAAATGACAATGATGGCGTT
TATCAAAGTACGGCTGCTCAAGCAAGACTCCGTAAATACCATTCAATGAGGACACTTAACGGTACAACAGAAACT
CCAGAAATAAGCTCTACTTTCCATTTGAGTTCAAGAGCATTGGAAAAATTTTACCACGAGGAGAACAGGTGGTGT
AAAAAGTTAGCCTCATGGTTTATACCCCGAGATGAGACCATTATCAGTGTTGATGAGGAAACAATCATGGATGAA
AGTACGGTCAATAGCAAGAGAAAATCCTATATGTATGAAATCAGGAACATGGTAATCAATTCGACAAAAGATTAG

>YOL023W 0.82   Cold
ATGCTCAGAAGACACGGATTATTCTGGTTAAAGACATGTCCCCGTCTAAATGTTCTTCTAAATCAATCCATACCG
ATACCTCATTTGCTTCATAGCCGTGATATTTGCCAGCAAAGATGGTATGCAAAAGGGAAAAGAAGGAACCAGATT

TCCAAGAAAGAGTTAAAGCCTCTCAATTTTTCTATTCCGAATTATATTTCTGTGAATAAATTGGCCAATTTGTTA
AATTGTCGTGTGGAAAGATTAATCAAAGATTTGACAGCATTAGGTTTCGAAAATATTACAACCACGTATATATTA
TCAAAAGAATACGTCGAATTGATTTTACAAGAATACAACTTTGCCCTTCCGAATCTGTCAACGTCTACAAATTTA
GATAATGTTTATGATGAGCTAAAATCTCCTGTAAACCCAAAATTATTAACCAAAAGAGCCCCCGTGGTAACTATA
ATGGGCCATGTTGATCATGGTAAGACCACTATCATAGACTATCTAAGGAAATCTTCTGTTGTGGCTCAAGAACAT
GGTGGAATCACGCAACACATTGGTGCCTTTCAAATAACAGCTCCGAAGTCTGGGAAAAAGATCACATTTTTAGAC
ACCCCTGGCCATGCTGCCTTTTTGAAAATGAGAGAAAGAGGTGCAAATATTACTGATATTATTGTTTTGGTGGTA
TCTGTAGAAGATTCTCTGATGCCCCAGACATTAGAAGCCATAAAACATGCCAAAAATTCAGGAAATGAAATGATA
ATTGCAATAACGAAAATAGATAGAATTCCACAGCCAAAAGAACGTGAAAAAAAAATTGAAAAAGTTATAAATGAT
TTGATTGTTCAAGGAATACCAGTAGAGAAAATTGGCGGAGACGTTCAAGTGATCCCGATAAGTGCCAAAACTGGT
GAGAACATGGACCTTTTAGAGGAATCAATAGTCCTATTAAGTGAAGTAATGGATATAAGAGCTGAAAACTCCCCT
AAAACTATCGCAGAGGGTTGGATTATCGAAAGTCAAGTTAAAAAACAGGTCGGAAATGTGGCGACCGTCTTGGTT
AAGAAGGGCACTTTACAAAAAGGGAAAATTCTAATTTGTGGTAATACATTCTGCAAGATCAAAAATTTAATTGAT
GATAAGGGCATACCGATTCTTAAGGCAACACCATCATATGCTACAGAGGTTCTTGGTTGGAAAGATGTACCCCAT
GTTGGAGATGAGGTCATTCAGGTAAAGAGCGAAGCGATCGCTAAAAAGTTCATCAGTAAAAGGCAAGATCTTATC
GAAGTTCAAAAAAACTCTAGTATCGTTGAAAAATTAAATGAAGAAAGGGCCCTTGCCAAAGAGCAACACCTAAAT
AAGGAACTGGAACATGAGAATACTGTCCAGGAACACGAACAAAATACTGGTCCTAAACTTATTAATTACATCATC
AAATGTGATGTCTCTGGCTCAGCAGAAGCGGTTTCAGAAAGCATTTCCTCTCTAGGAAATGACGAAGTGAGATGT
AATGTTATCTCTTCTTCTGTTGGTATACCTACGGAAAGTGACTTAAAGATGGCGCAAATAACGGAGAGTACAATT
CTATGCTTCAATTTGGGGAATTTGCCGAGTGAGGTGATCAACAATCGTGCTGGCATCAAAATAAAACAGTACAAT
GTTATCTATAAGCTAATTGAAGACGTCACTGAAACACTAACTGAAAATCTAAAGCCCATCTTTGAAAAAAAGATC
GTTTCAACTGTTGACGTTCGTGAAACTTTTGACTTTAGGTTAAAGAAAAAAATTATTAGGATTGCAGGTTGCAAA
GTAAACAACGGTGTCATCAAAAAGAATTCATTGGTGCAGGTAGTTAGAGGTCCTAACGAAGATGTAATTTTTGAT
GGGAAAATCTCTACTTTGAAGCACAATAAAGATGACGTAGCAGAAGTTTCAAAAGGGCATGAATGTGGAATCACT
TTTGAGAGTGGATTCGAGGGTTTCAAACCAGGTGATAAGATCCTAGTTTATGAAAACGTGCGAGTTCCTCGTTAC
TTGTAA

>YDL216C 0.81   Cold
ATGTCACTTTCAAATAAGACTGTAAAGGAATTACGACAACTACTAAAGGAAAGGTACACCGTGGAAGATGAACTA
ACTGAATCTATAGCGCTATCAAGTATGCGATTCAAGCCGTCGCAAGAACCTGAATTTCATGCGCTAAGCCAATCT
TCCTTGCTCAAAACTAAACTAAAACAACAAAGTAGTACAGATATTCCGAGTTACACCCACGTACTTATATCAAAA
TTATCATGTGAAAAGATCACCCATTATGCAGTTCGTGGAGGAAATATTGAAATCATGGGCATATTAATGGGGTTC
ACATTAAAAGATAATATTGTTGTAATGGATTGTTTCAATCTTCCCGTAGTAGGGACAGAAACTCGCGTAAATGCA
CAATTGGAGTCATACGAATATATGGTTCAATATATCGACGAGATGTATAATCATAATGACGGTGGTGATGGGCGA
GATTACAAGGGCGCTAAACTGAATGTTGTTGGCTGGTTCCATTCACATCCTGGTTACGACTGTTGGTTAAGTAAT
ATAGATATCCAAACCCAGGATTTGAACCAGAGATTCCAAGATCCATATGTCGCAATTGTGGTCGATCCCTTGAAA
AGTCTGGAAGATAAAATTTTAAGAATGGGTGCATTTAGGACAATAGAAAGTAAAAGTGATGATAATAGTGCCACT
TCATACTATGAACTAGAAACAATCATATTTGATTCAGAATTGAACCGAGCGCTATTTGAAACAAAGTTAAACTTA
CATTGCGTAATAGAGGATGATGAGTCAGAACAAATATCATTAAATAGATTGATCGATTCAATGAAGCAGTATAGC
TATTTGATGGATTCCAAAAATGTTAGAACAAGGATTAAGTTGGCTACTACCAGTGAACGTGTTAGCAACGAAAAT
AAAAAAAACATTGACTATCAAAACCGTTCAACAAGGTCTCAGTTCTGCCTCAATACGCAAAGGGGGGATAGCACT
GAAACAAGCTCCTTTGGAAGCATGTTTTCAGGTGATAATACTAGTGACGTAGATATGGAAGATAGAAACCTTACC
GAGTTCGACAGTACTGACACTAGTCTGTGCATAAACGGCGAACCGAGCATTCATGTAAATCGCGTGGAAAGAAGC
AGCAGGTCTACCGACAATTTTCACAATTCTAAAAAACGCATGAACAGCAATCAGGAAAGATGTCATGATGAGGGC
AATGATATGTTACAGAGAAATGTCTTGGAGACAGATTATGCAAGAGCAAAAAATCGAATACTTGCGTCAAAGATA
AAACAATATGAGCGGTTACGTTTTTACAAGGACACCTTTACATTATGA

>YIL002C 0.72   Cold
ATGAGACTCTTCATCGGTAGAAGATCACGGTCGATTGTAATATCTTCCAACAACTACTGTTTATCCTTCCAGCGT
TTGCGCAGCATACCAGGTGCCAGTTCTCAGCAGCGTCAATTAAGTAAAACTCCTAGCGTAACAATCAAGTCCTAC
CCTGACACAGATCTTTCAAGTGATAGTAACTATCTCGAAGTCAAAAGTTGCATATTTAATGGATTATTAGGGCTC
GTTTGTCTTAATGGTGATATATATGTTGCCGTCATATCTGGTGTTCAGAATGTTGGGTTTCCTCGATGGAAACTG
ATAGATCATCAAGTTAGGCCTTCGGAGAGCATTTATAAAGTATTGGATGTTGACTTTTACAGCTTAGAAAATGAT
GTTTTCGACTACCTCCTCTGCGAGAGATCAGAGCAAAACTATGATAAATTGATTCATGAGCATCCTTGCGGCCCC
CTGAAAAAACTGTTTAGTGATGGAACATTCTACTATTCTAGAGATTTCGACATCTCTAATATCGTGAAAAACCAT
GGTTTATCACATAACTTAGAATATACCGTAGATAATCAAGATTTATCATTTATTTGGAATGCTAACTTAGCAAGT
GAGGTAATCAACTGGAGAAGCAAGATTTCAAATGAGGAGAAACAACTTTTTGCCAACGCAGGGTTCTTAACATTT
GTAATTCGGGGTTATTGCAAGACAGCATTAATAGAAGACGGCCCAAATACAGCCTCTATAACCATTATTTCCAGA
ATTTCGACTGAGAGCAAGCAGGATACGCTAGAATTGGAGGGTATTAGTGAAGATGGGAGAGTGTCGCTATTTGTT
GAGACAGAAATTGTAGTAACCACAGAAAAGTTTATTTTTTCTTATACTCAAGTGAACGGAAGCATTCCTCTTTTT
TGGGAATCTGTAGAAAGTCAACTGTTGTATGGAAAAAAAATTAAGGTAACAAAAGATTCTATTGAAGCTCAGGGA
GCTTTTGACAGGCATTTTGATAATTTAACCTCAAAGTATGGTGTTGTGAGTATCGTTAATATCATAAAACCTAAA
AGCGAATCACAAGAAAAGTTAGCACTGACGTACAAGGATTGTGCTGAATCAAAGGGAATAAAAATAACAAATATA
GAATACAGCTCAAGCGTTCTGACAAAGTCTCCCCACAAATTACTTTATTTATTAAAACAGGATATTTACGAGTTT
GGAGCGTTTGCCTATGATATCTCTAGGGGCATCTACTTTGCAAAACAAACGGGGGTATTAAGAATAAGCGCATTT
GATTCAATTGAAAAACCTAACACAGTTGAAAGGTTAGTGAGCAAGGAGGTACTTGAATTAACCACAAATGAAATT
GATGTATTTGAATTGACTTCCCCATTCTTGGACGCACATGATAAGCTATGGTCAGAAAACTATTACTGGCTTGAT

CGAACGTATACTAAGCACACGAAGAATTCTGGTAAATATACAAAGGTTTATTCGAAGTTATTTGGTTCCCGAGTT
AGATTATACGACCCGCTCCATATCTACATCTCTCAGTATTTAAAGCAATTAAGGTCAAAATATACGTTTGAGAAA
GACATCTCAATTTTTGCAGGTACATTTAATATAAGTGGTAAAATTCCAAAAGATGATATAAAAGATTGGATATTT
CCAAAATCAATGTCCAAGGAGGATGAAATGGCTGATCTTTATGTTATCGGATTAGAAGAAGTGGTAGAACTTACC
CCAGGTCATATGCTTGCTACCGATCCATATGTCCGACAATTTTGGGAGAAGAAGATATTAACACTATTAAATGGG
CCCGGACGCAAAAAAAAATATATCCGTTTATGGAGTACACAGTTGGGTGGTATTTTACTATTATTATTCATGAAT
GAAACGGAATATTCCAAGGTAAAGCATATTGAGGGGGATGTTAAAAAAACCGGGTTTGGAGGAATGGCTTCTAAT
AAAGGAGCTGTCGCGGTTAGTTTTAAATACTCAGCTACAAGATTTTGCGTACTAGTATCTCATTTAGCGGCAGGC
TTAGAAAATGTTGAACAAAGGCATAATGATTATAAGACGATTGCGAAAAGCATACGATTTTCTAAAGGATTACGA
ATAAAGGATCACGATGCAATTATTTGGATGGGCGATTTCAATTACAGAATATTGATGTCAAACGAAGATGTTAGA
CGAAAAATTGTTTCTAAAGAATATGCCAGCCTATTTGAAAAGGATCAACTTAATCAACAAATGATCGCAGGTGAG
TCTTTTCCATATTTCCATGAAATGGCGATTGATTTCCCACCTACCTATAAATTCGACCCAGGAACTAAAAACTAC
GACACGAGCGAAAAAAATGAGAATACCAGCATGGACTGATAGGATTTTAAGTAGGGGAGAAGTACTAGAACAGTTA
GAATATAAATGCTGTGAAGATATACTATTTTCCGATCATCGGCCCGTATATGCCATATTTAGGGCTAGAGTGACT
GTAGTGGATGAACAAAAAAAGACTACTTTGGGTACGCAAATCTACGAGAAGATTATGGAAAGGCTAGAGGGATTG
GATGATGATGAGAAAATTGCAGTTTTGAGTGATGATGCATTTGTTATCGAAAGTTTTGAGGGCAGTGATTCGATA
GCTGGCCCTACGCATTCTCCGACACCAATTCCCGAGCCCAAAAGAGGTAGAAAGTTACCACCACCTAGTTCGGAT
TTAAAAAAATGGTGGATTGGAAGTGGGAAGCAAGTTAAAGTAGTTCTTGACGTAGACCCGGCCGTTTACATGATT
AATCCTAAAAGAGATCCCAATCCATTCGTTGAGAACGAAGATGAGCCACTTTTTATAGAAAGGTAA

>YOR156C 0.8 Cold
ATGGCAAGTGTCATGTCAAATAATAATAATAATAATAATAATAATAATGCCAGTTATATGTTTACGAACCCTTTG
TCCAACACAGGTGGTGGACTCATAAATGAAATAAAAGATGCAATAAATGAAATGGAACAATTAAAGGTTCTAGAG
TTAAAGCAAATATGCAAATCTCTGGATTTATCCATAACGGGCAAAAAGGCTGTCTTACAAGACCGAATAAAACAA
TTCTTAAGAAAATCCTGTGATATTGGACATATAGATCCGTGGAGGCCTAAGGCTATTAAGATTCTTATCGCGAAA
GTAAGGATAAACTCATCATTACCAAAATACTCTACCCTTTGGGAAACTTTAAAGACGGGTGCGTTCAAGCATCCT
GTAGCATCGGGGCAACTACCCGTGACAGCTCTCCAGAGTACTGCATTGCCACCTTATTCCCAACAACAGGCCTTA
GCATATTCTTTTACCTCACCGTTTTACAAACCAATAGTACAAATACCGGATGCCAATAAAAAATTGAAGCAATCT
GCAGGAAGAGGTTGTACAAAAATGAAGTTCAAGGTAAGTAAATCTAATCATGATCTTTTGAAATCTAACAAGAGT
TATAAACTGTATTTGTTTTCTGGCTTTTCAATACCGTTTATATATGAAACTGTGGGCCATGAGGCAATAGATTTC
CCGTATCCCTGTGAACTAGTGTTTAATGGCACCAAACTAGAGGACAACGTGAAAGGTTTAAAGAAACAAAATGGG
ACAGGTAATCCTGCAAATTTAACGCCTTATCTTAAAGTACCGACCGAGATGAATCACCTAGACTTACATTATCTC
AATATTGATAAAGAGTACTCTATAAGTTGCTTTATAGTGGAAGTTTTTTCACCTGAAGCTCTTCTGGGGAAAATT
CTGAAAAGGCCCAAAATTATCAAGCAAGCCACCACAGCATACATCAAAAGAACCTTGAATGAGCAAGATGATGAC
GATATTATTACAACCTCCACGGTGCTGAGTTTGCAATGTCCTATATCTTGTACAAGGATGAAGTATCCGGCGAAG
ACTGACCAATGTAAGCATATCCAATGTTTTGATGCTCTCTGGTTTCTTCACTCTCAGTCACAAGTCCCTACATGG
CAGTGTCCGATTTGTCAACACCCTATAAAATTCGACCAGTTGAAGATTTCCGAATTTGTAGATAATATTATTCAA
AATTGCAATGAAGATGTTGAGCAAGTTGAGATATCTGTTGATGGCTCATGGAAACCGATTCATAATAGTTCGGCC
GTTATAACGGATACTGTTAACCAAAATCACAGTGTGAAAAACGAGAACCAAGGCACTGTCAAACAAGAGCAAGAT
TATGACAGCCGAAATGCCTTCGATACTAATTTGCGTAATGGTTCAAACCACAACGAACCCGAAATAATATCTCTG
GATAGCTCAGATGATGAAGCCTTTATTCCGGCTAGTAAAAGCTTCCCAACGCACGTGAACCCCCGTAATGACCAA
TTAAGAGCAGATATATTTCCCTCAGAATCGGAAGGATCCAGTGACTACAACCCCAATCATACATCAACGCCGAAA
GGTTCTCCAACTATGGACCAAGATAATTATCAAGATGCTTTTCAAATGAGGTCATTTTTGAACCAAGGCGCTACT
ACAAATATAAATGACACACCGACGAACAATAGCAGTATTAATTCTTTCGTTACGGCGACGAATGGTGACAGCAGA
ATATTCTACAATAGAGGGCCATCAACGCCACTATTACCGGCTGTTCTACAAAATTTGACTAATCAAACGGAAGCG
CAGAGAAACCCCTACGGACCTAATTACAATACCACAGCTCAAGATCGTAATCTTTTGGGAATCGAAGGTGATTTG
CCTCCAATCCCCCCTGTGGACCCAAATTCAGAAGCAGAAACTGAACTACCAACAAGGACCACATCGGCGGCACAT
CTCCCTCCATATATACACGTGAGCACCAGCGGCCATGGGGATGATGGAAAAATTAGGAAGAGAAGGCATTCTAAT
GTTTCTATCTACATTCCGAAGAATCCATACGCCACCCTTATGAAACGGAGACCACAGGCAAATCACGCCATAATG
AATAAAACTCTGGCCCAAACTAATGACTTTAACACATCGGCTCAAGATAACTCGGAAGTGGTCGATTTAACATCA
GATTGA

>YDL005C 0.76 Cold
ATGGTAGTACAAAATAGCCCAGTTTCGTCTGTTCACACAGCCAATTTCTCTGAACGGGGGAGCAATACCAGAACA
ATGACGTATAAAAATAAGCTTACTGTTTGCTTCGATGACATCCTAAAAGTTGGAGCAGAAATGATGATGCAACAA
CAGTTAAAAAATGTACAACTAGATTCTTATCTCGTCAATGGTTTCAGTCAATCTCAGCAAAAATTATTGAAAGAG
AAGGTTAAATTATTTCATGGAATTTTAGATGATTTAGAGACTTCTCTAAGCCAGAGTTCTTCATATTTAGAAACT
CTTACTGCACTCGGAAAAGAAAAGGAGAAAGAAAGAGAGGAAGCTGAAAAAAAGAGGGCTGAACAAGAAAATATG
AGGAAGGTTAGAGAACAAGAAGAATTAAAAAAGCGACAAGAACTCGAAGAAGCCTCCCAACAGCAGCAACTCCAG
CAAAATTCAAAGGAAAAAAATGGCCTTGGTTTGAACTTCTCCACAACAGCACCTGCGAACACAACGGATGCCAAC
GGTTCCAAAGAGAATTATCAAGAACTTGGATCTCTTCAATCATCCAGTCAAACACAACTTGAGAACGCAAATGCT
GCTAATAACGGTGCCGCATTTTCTCCCTTAACCACTACTCGAATCCAAAGCCAGCAGGCCCAACCCTCGGATGTA
ATGTTCAACGATTTAAACTCTATGGATATTTCGATGTTTTCAGGCCTTGACAGTACTGGTTTTGACTCCACAGCC
TTCAATGCCACGGTGGACGAAACCAAAGGCTTTGACGATAATGACTCAGGTAACAACTACAATGACATAAATATT
TCTTCTATTGAAAACAACATAAATAATAATATCAATAGCACCAAGAACGGCAAGGATAACAACAACGAAAGCAAC
AAAAATAACAACGGCGATGAAAAGAATAAGAACAACAACGAGGACAACGAGAACAACAACAACAGCAGTGAGAAG
AACAACAATAATAATAATAATAACAATAACAACAATGACGATAACGGCAACAACAACAACAACAACAGTGGGAAC

GACAATAACAATACCACCAACAACGACAGCAATAACAAGAACAATTCTATCACTACCGGAAATGATAATGAAAAT
ATAGTTAATAATGATTTACCAACGACCGTTGTTTCCAATCCTGGAGATAACCCTCCTCCAGCCGACAACGGTGAA
GAATACTTGACATTAAATGATTTCAACGACCTTAATATTGACTGGTCGACCACTGGAGATAATGGCGAATTAGAC
CTCAGCGGCTTCAATATATAG

>YOL116W 0.8Cold
ATGGCAAGTAACCAGCACATAGGAGCTTCAAACCTAAATGAGAATGAGGCTATATTAACCAACCGCGTTGCTGAG
CTGGAAAGGCGTATGTCGATGTTTGAGGGTATATTTCACGCGTTAAGTAACCGTCTCGATCTTCACTTTAAAAAA
TATGATGTAGTGGTAAACTCCCAACAGCAACAAATCAACGAACTGACCGCGTTTTTATCAACATTGCTGAATGAC
CAACAACGCCACGCTGAAATTCTCAGTGAAAAATTAAGCGGAACGTTGCATGGGGTGTCAGCTACGTCAATATCC
TTAAGCCAAACTCTTGACCCACAAGGCTTCACTGATGGAACTACGGCACCAGGAGCTCCTAGGAATTATACTTCA
GTGCCTATGAATAATGATCAAACTGCTCATCCGCAAAATGAAGGAGCTGTTAGTAATGAAACACTTTTTGAGGAC
ATTTTGAATGGAAATTCACAAGAAAATGATAAGAGTCAACAACAAACTAACAGCTCAAATTCTATAAGCCAAGAA
AATAATAGCACCAACCCTTCAGTGGACACTCGGTTCAACAAGCCGCAAAATTATAATTCCAATTTAGTCCCATCC
TTGGAAGAGTATTCAGCAAATCCACCTAACAATGATGGTGGCCAAAGTCAAGGACTGTACATAAGCAGCAACTCT
TCTCAATCACGGCAGTCTCCTAATCTCCAGAAAGTTTCTCCTAACCATGAAAATGCGGTTGAATCAAATGCACAA
GAGAGCGTGCCGACATTTGAGGAGGAACAGTATGAGACTAAAACAGGATTGAAACGGAAACGAATAGTCTGCACA
AGACCCTTCGAATTTATCAAGTCACCACACTCTGTGATGGAGGTTTGGAAGGAGTATACAGAAGGTGTTAACGGG
CAGCCTTCTATAAGGAAAATGGAAGCTCTTTATCAAACGGCATGGAGGCGAGATCCAGCAGTAAATAAAAGATAT
TCGAGAAGAAAGGTTCTTTGGAAAGCCATTCAAACTGGCCTTAATCGTGGGTATTCATTAAACTATGTTGTTGAA
ATATTAGAAAACTCAAGATATGTTAATGATAAACAGAAGGTTAAACAACCTATTGGTTGGTTATGCCACAGTTCT
CATATTCCAGAGACTTTGAAGTGA

>YOL163W 0.78   Cold
ATGATAGCGTGGTCCCTAGTGGCTACGTTGCAATGCAAAATGACAGGGAAGAGTTCTTTCTATACATGTAGAGCA
CTTATGGGCCTATTTGAAGGTGGTTTTGTGGCTGATTTGGTTCTCTGGATGTCGTACTTCTACAGCTCTTCAGAA
TTGTCAATAAGACTGTCGTTCTTCTGGGTGACACTATCTCTAACACAAATCATCACTTCAATTGTGGCCTTCGGG
GTTTTTCACATGAGAGGGATAGGTGGCATGGCAGGGTGGCAATGGCTTTTTTTGATAGAAAGAATTTTCACTTTA
GTCATCGGTATCAGTGCATACTTCTTGATGGTACCCTCCGTAGTTCAAACGAAGAAACCTTGGAGCAAGAAAGGA
TGGTTCACTGAGAGGGAAGAAAAAATCATCGTAAACAAGATTCTGAGAGATGATCCGACAAAAGGGGATATGAAC
AATAGGCAAGGTATGTCACTTAAAATGTTATGGCAGGGGATAACAGATTACTATATATAG

>YGL130W 0.81   Cold
ATGGTTTTAGCAATGGAAAGTAGAGTGGCACCGGAAATTCCTGGGCTCATTCAACCTGGGAATGTCACGCAAGAC
TTGAAGATGATGGTCTGTAAATTATTGAATTCCCCAAAACCTACGAAAACATTCCCTGGTTCCCAGCCTGTGTCC
TTTCAGCATTCTGATGTGGAAGAGAAGCTGCTTGCGCATGATTACTACGTTTGTGAGAAAACAGATGGTCTGCGG
GTGTTGATGTTTATAGTGATAAATCCTGTGACGGGTGAGCAAGGATGCTTTATGATTGATAGGGAAAATAACTAT
TATCTGGTTAATGGATTTAGGTTTCCCAGATTACCCCAAAAGAAGAAAGAAGAGCTGCTAGAGACTCTTCAAGAT
GGCACCTTATTAGATGGTGAACTTGTCATACAAACTAACCCAATGACAAAATTACAAGAGTTGCGTTATTTAATG
TTCGATTGTCTTGCTATCAATGGTAGATGTCTCACACAATCACCAACAAGTTCTAGACTAGCCCACCTTGGAAAA
GAATTTTTTAAACCATACTTCGATTTAAGAGCAGCGTACCCTAATCGTTGTACTACTTTTCCGTTCAAAATTTCC
ATGAAACATATGGATTTCAGTTACCAATTAGTAAAAGTTGCTAAAAGTTTAGATAAACTACCACATCTTTCTGAT
GGTCTGATATTTACTCCTGTGAAGGCACCTTACACTGCCGGCGGAAAAGATTCATTGTTATTAAAATGGAAGCCA
GAACAAGAAAACACCGTGGACTTCAAATTGATTTTAGATATCCCAATGGTGGAGGATCCTTCTTTGCCTAAAGAT
GATCGGAACAGGTGGTATTACAATTATGACGTTAAGCCAGTTTTCAGCTTATATGTCTGGCAAGGCGGAGCTGAT
GTCAATTCACGTTTAAAACATTTCGACCAGCCTTTCGATAGGAAGGAATTTGAAATATTAGAAAGAACATACAGA
AAATTTGCAGAGTTGAGCGTTTCAGATGAGGAATGGCAAAATTTGAAGAACCTAGAACAGCCATTAAATGGTAGA
ATAGTAGAGTGCGCAAAAAACCAAGAGACTGGGGCGTGGGAAATGTTAAGATTCAGGGATGATAAGTTAAATGGT
AATCATACATCGGTGGTCCAGAAAGTTTTGGAGAGTATCAACGATTCAGTTTCATTGGAGGACCTCGAGGAAATT
GTTGGTGATATTAAAAGGTGCTGGGACGAGAGAAGAGCAAATATGGCTGGTGGTAGTGGGAGACCACTACCGTCT
CAAAGTCAAAATGCGACATTATCTACCTCTAAGCCAGTCCATTCACAGCCCCCAAGTAATGATAAGGAGCCAAAA
TATGTAGACGAGGATGATTGGTCGGATTAG

>YGL009C 0.78   Cold
ATGGTTTACACTCCATCCAAGGGTCCAAGAACTCTTTACGATAAGGTTTTTGATGCACATGTTGTCCATCAAGAT
GAAAATGGTTCCTTTTTGTTGTATATCGACAGACACTTGGTTCATGAAGTCACCTCTCCACAAGCTTTCGAAGGC
CTAGAAAATGCCGGCAGAAAGGTCAGAAGAGTTGATTGTACTCTAGCTACTGTAGATCATAATATTCCAACTGAA
TCAAGAAAGAATTTCAAATCACTAGACACATTTATCAAGCAAACTGATTCGCGTTTACAAGTCAAAACTTTAGAG
AATAATGTTAAACAGTTCGGCGTTCCATATTTCGGTATGAGTGATGCCAGACAAGGTATCGTCCACACCATCGGT
CCCGAAGAAGGTTTCACTTTACCAGGTACCACTGTTGTTTGTGGTGACTCTCATACCTCTACTCACGGTGCCTTT
GGTTCGCTGGCCTTTGGTATTGGTACTTCTGAAGTTGAACACGTCTTGGCCACTCAAACAATTATCCAAGCTAAA
TCGAAAAACATGAGAATTACTGTTAATGGTAAGCTATCACCTGGTATCACATCTAAAGATTTGATTTTGTATATT
ATTGGTCTGATAGGTACTGCAGGTGGTACTGGTTGCGTTATTGAATTCGCAGGTGAAGCCATTGAAGCTCTTTCT
ATGGAAGCACGTATGTCCATGTGTAACATGGCCATCGAAGCTGGTGCAAGAGCAGGTATGATTAAACCAGACGAA
ACTACTTTCCAATACACCAAAGGCAGACCTTTGGCTCCAAAAGGTGCCGAATGGGAAAAGGCTGTTGCCTACTGG
AAAACTTTGAAAACAGACGAAGGTGCCAAATTTGACCACGAAATCAACATTGAAGCTGTCGACGTGATTCCAACT
ATTACATGGGGTACTTCTCCTCAAGATGCCTTACCAATTACTGGTTCCGTACCAGATCCAAAAAATGTTACAGAC

CCAATAAAGAAATCTGGTATGGAAAGAGCCTTAGCCTATATGGGTTTGGAACCTAACACACCGCTAAAGAGCATC
AAAGTAGATAAAGTCTTTATTGGATCTTGTACCAATGGCCGTATTGAAGATTTAAGAAGTGCTGCAGCTGTAGTT
AGGGGTCAAAAATTGGCTAGTAACATCAAATTAGCTATGGTTGTTCCGGGATCTGGTTTGGTCAAAAAACAAGCC
GAAGCAGAAGGTTTGGACAAGATTTTCCAAGAAGCTGGTTTTGAATGGAGAGAAGCTGGTTGTTCAATATGTTTA
GGTATGAACCCTGATATTTTGGATGCCTATGAACGTTGTGCTTCTACTTCCAACAGAAATTTCGAAGGTCGTCAA
GGTGCCTTGTCTCGTACACATCTAATGTCCCCAGCCATGGCGGCTGCTGCAGGTATCGCAGGTCACTTCGTAGAT
ATTAGAGAATTCGAATATAAGGATCAAGACCAAAGTAGTCCAAAGGTTGAGGTCACTTCCGAAGATGAAAAAGAG
CTTGAAAGTGCTGCTTACGATCACGCTGAACCTGTCCAACCCGAAGACGCTCCCCAAGATATTGCCAATGATGAA
TTAAAAGATATTCCAGTGAAGTCAGATGATACACCTGCTAAACCTAGCTCTTCCGGCATGAAGCCATTTTTGACT
TTGGAAGGTATTAGCGCACCATTAGATAAGGCCAATGTTGATACAGACGCTATTATCCCAAAGCAATTTTTAAAG
ACAATTAAGAGAACGGGTTTGAAAAAAGGGTTGTTTTATGAATGGCGTTTCCGTAAAGACGATCAAGGTAAGGAT
CAAGAAACCGATTTTGTATTAAATGTTGAACCTTGGAGGGAAGCTGAAATATTGGTTGTTACTGGTGACAATTTC
GGATGTGGTTCTTCCAGAGAACACGCACCATGGGCTCTAAAAGACTTTGGTATAAAATCCATCATTGCACCTTCT
TACGGTGATATTTTCTACAACAACTCTTTCAAGAACGGCTTATTACCTATTAGATTGGATCAACAAATAATCATT
GACAAATTGATCCCTATTGCTAATAAGGGTGGTAAGCTCTGCGTCGATTTACCAAATCAAAAAATTTTGGATTCC
GATGGCAATGTGCTCGTTGATCATTTTGAAATAGAACCTTTCAGAAAGCACTGCCTAGTTAACGGTCTTGATGAT
ATTGGTATTACATTGCAAAAGGAAGAATACATTTCCAGATACGAAGCTTTGAGAAGAGAGAAATACTCATTCTTG
GAAGGTGGATCAAAATTATTAAAATTTGACAACGTTCCAAAAAGAAAAGCTGTTACAACTACTTTCGATAAAGTC
CACCAGGATTGGTAG

>YOL118C 0.79   Cold
ATGTCTTTTAGAAAGAAAAAACTCAAACCTCCAGCGGGTAGTCAATTTATTATAAACGACTCCATAATGAGCTAT
ATTGACCGCACGAAAACACTAATAAGGATGATTGGATGCAAGAACCAGTACATAAAAGCACGTATGAAAGATAAG
ACCTTCTTCTATACGAAGCAATTCCGTACAGCCAAAAACAAATTTTTCTTTCATCTGTACCATTGGGAGGCCACT
CATATTAACGTTGACCACTATATATGTACATGTCATCCCATTTTTTGGGGCTCTATAGGTCAGAAACTCAGGAGA
TCCGCCTGA

>YNL183C 0.71   Cold
ATGTCTTCATTAACTCGATTGCTACAGGAAAAACGAAAAAATGAAACTTCGAACTCATCACCGAGAACTTCTGCT
GATACGTTGACCACGACGCCCGAGTCACAATCGCTTGACCTTCACTCTAGGAATAAGAGTAGTAGTCATATTGGT
AGCGTGAGTAATAGCAGCAGTAGTGATCGTAATAGGGCTAACGTGCCAGTTCCTGGCAGTGTCACAACTGTTACT
CAAATATACTCAGAGGAAGATTCATCATCAACAGCCGGTTCCTCTCTCGACGATAGGAATCAGTTTTCCTCCAGC
TTCCTTAATGCAAACTTTGCTCACACTGCATCATTCTACGGCACAAGTGCACAAAGTAGAGATAGATTTGGATCC
TTAATAAACGATCAAGGTACTGCAGGACTTTCCTCACATGGTGGTTCTTTTGCTGCACAGAACAGAATCACAAGT
AGATTATCTACCACGTCACATACTTCAGGAAGGGCCATTCCTTCTTTATCATCAAGTATACCTTACTCCGTGCCA
AATTCTAACAAAGATAACAATAGCTCGAATAGCAACAGCTCCTCTTTGTCTTCTTCGTGGCTCGAAACATATGCA
GGCGGTATGCCCAATAATATTTCTGCAATCGATTCAAATGTTATATCCTCCCCAAAAGTAGATAGCGTGGAACCA
CGTTTTGTTATCTCCAAACAAAAACTCCAAAAAGCATCGATGGATAGTAATAATGCAAATGCCACTCAATCAAGA
TCTATATCCCGTTCAGGTTCATTCTCTTCACAGCTTGGGAATTTTTTCTTTTCCAAAAACTCAAAAGAATCTTCA
AATTCTAACTCTGCAGGCATGTCTTTTTCTGCCAATTCCAATGGTCCTTCTCCAAACATTAAGAATCCCAATGTT
ACCAATGGTTCGACGCCTATCCCGAAGCCTATCCGCGCAAGACAAAGCAGCATATATTCTGCATCAAGACAGCCA
ACTGGTTCCTATACTGATAACTTTTACGGTTCCCCCTCATCTGTACATGACCACTTACCTCCAAGTCAAAGTGTT
CCAAGGTCACAACATTCTTCCATCGGTGATTTAAAGAGGTTCTTCAAAAAAAGTTCTAACAGTAACCTTTCCTCG
AATAGTAACAACGTGATCCCGAACGGATCACCTCTTTCTAGCGGAATTGCCGTTCCGTCACACTCTCATTCATCG
TCTCATTTTGCCGCAGGGAATAACTCATATTCCACTAGTTATAACGGAAATGGAGATACTATTTATTCTCATAGT
CACGGCGGTTCAGGAATTCCGTTCTCAAAAAGGTACATCAAAACCGGTGCCGATTTAGGCGCTGGAGCAGGAGGT
TCTGTCAAATTAGCTCAAAGAATTTCAGATAACAAAATATTTGCAGTGAAGGAGTTTAGAACTAAATTTGAAAAT
GAATCCAAAAGAGATTATGTCAAGAAAATTACATCAGAATACTGTATTGGTACAACTTTGAATCATCCCAATATC
ATTGAAACAATTGAAATCGTGTATGAAAATGATAGAATTCTCCAAGTTATGGAATATTGTGAATACGATTTATTT
GCTATTGTAATGAGTAATAAAATGTCCTATGAAGAGATTTGTTGTTGCTTCAAACAAATACTTACTGGTGTACAA
TATTTGCATAGTATCGGCTTAGCTCATAGAGATCTAAAGCTAGATAATTGTGTTATAAACGAAAAAGGTATTGTA
AAATTAATCGATTTTGGTGCTGCTGTGGTATTTTCATATCCATTCTCAAAAAATCTTGTGGAAGCAAGTGGAATT
GTTGGAAGTGATCCATATTTAGCACCTGAAGTCTGTATATTTGCGAAGTATGACCCACGTCCCGTAGATATTTGG
TCTTCTGCAATCATTTTTGCATGTATGATACTGAAGAAATTTCCATGGAAAATTCCGAAATTAAGGGACAATTCA
TTCAAACTATTTTGTTCCGGACGTGATTGCGACTCCTTAAGTAGCTTGGTTACAAGAACTCCTGATCCACCTTCC
TACGATGAATCGCACTCAACAGAAAAGAAAAAACCCGAAAGCTCTTCCAATAATGTATCAGATCCTAATAATGTT
AATATCGGGCCACAAAGATTGTTGCACTCATTACCAGAAGAGACTCAGCATATTGTTGGACGTATGATTGATTTG
GCGCCCGCATGCAGAGGAAATATCGAAGAAATCATGGAAGATCCTTGGATCCGTAGCATTGATATGTGCCACTTG
GTTGAAGATGGATTATCGTTCAAAGTGGTTAGAGGAGAAGACCATCATCACACACAAGTGGATCAAAGTGAAGCA
CATATTGCAGGCCTAGAAAAGAAAAAGAAAAAGCAAAATAATCAATAA

>YDR237W 0.81   Cold
ATGCAAAGGTTTTCACTAGTCACTCACAGATCATTCTCTCACTCCTGTGTGAAGCCCAAATCTGCATGCTCTTTG
GTCAAACCAGTTCATCACTTGGTGAAAATTGATAAGTCAAAGTTATCCCCTAGATTTCCAGAATTGAAATATGAC
AAAAGTGATATTAGGTCACCTGGATTTAAACCAAAAGACACTCATGCAGATAGACTCAATGACCATTACCTTAAC
ACTTTACAATCTGATTTGTTATTGATTAATTATTCACATAACGCTGCGGTGGTAAAGGGTCTAAAACAAAGAGCG
TGGAGTGGTGATTCGCCATACCACTTGAACAGACCACCGAAAAATCCTCGAGGTTCGAAAGCTCAACTACCTGAC

ATTCATCCTATCAAATGGAGTAACATTCCTGGGCTAGAAAGTGTTGTAATAAACTGTTTTGTTAGGGAAGCGAGA
GAAAATCAGCTATTGGCTATCACCGCTGCGTTACAATTACAACAAATTACTGGATGTAAACCTCATCCAATTTTT
TCCAAAAATGATGTTCCAACCTGGAAACTGAGAAAAGGTCATCAGATGGGAGCTAAAGTCGAACTAAAGGAAAA
GAAATGTCTCAGTTCTTAAGCACATTGACCGAAATCGTCCTACCAAGAATCAGAGAATATAAAGGTATAAGCAAC
CAGTCTGGGAACAGATTTGGTGGGATTAGTTTCGGCCTGACGGCTGAAGATATCAAATTCTTCCCTGAAATCGAT
GCAAACCAGGACTCATGGCCTAAAACTTTTGGTATGCACATAAATATAAACACCTCAGCTCAATTAGATTACCAG
GCGAGAACTTTGTTGAGTGGGTTCCAGTTCCCATTTTTCGGAGAGGAGAAATAA

>YOL144W 0.8Cold
ATGGATAGTGTAATTCAAAAAAGAATTTTTGTCGGAAATATTTTCCATAATGCAGATGATTGTTATTCAGAACTA
CTAGATCGATTTGGAAAATTTGGTGATTGCCAAGATTTTCAGTTTGAAAAACACAACCACTTTGCATTTATCGAT
ATAAGGTTTAATGATGAGGCAGATTTTAACAAGCTAAGAAAGAGCTTTAATAATGTTAAGTTTAAAGGGAACATT
TTGAAGGTTGATGAAGCAAAGCCGAATTGGGAAAGCACCTGGGCGGTACAACATGCAAAGGACCTTAAAGAAGAT
ATTATACTAAATGCTAAAATGAAAAAAAAGAATTGGCAACATTATAAGAAAATGGAAAATGTAGCGAAAAGTTGG
AAAGATCATAAGGAAGTTATTGCTGGCAGAATGAGGGAGGCGCCGAGAAAAAGGAGTCAATTGAGGAACATTACA
TTCAGGATCAATGTCAATGGTTCATTGAAAGTTTACAAATGCTATAAGACTAAATTATGGGGTTATGAAAGGAAC
AAAGAATTGAATGACCTTGTGTACAAGTTTACAAATAATTTCTGGAAAAACGGATACAACCACATTGTCGATAGA
TTGGATTACAGTCGTGCTGTTAAAACTGTTCGATTCAAAAATGGGCTGAAACAGTTAACAGTGTCAAAAGATGAA
AACGTTTGCAGTGGAGAGATGGACAGCGATGAGAATATGTCAGAAGAGGAGAAGGAGAAAAATAATGTTATTCTT
AATGACCTATTAAAGGACTTTGACTTCGATAAGCCAATGACGTTGAATGATTCGGACGAGGAACTTCTGACAGAG
CAACGTAAAGGAGAGGAAGAAGAAGAAGAAGAAGAAGAAAAAGAAGTAAACGCTCCCGAATACGAGAATGTTAAT
AAGACGAAGGACCAGAGCACCCTACCACAAGAAAAACCAGAGGAAAGGAAAGAACAGGACGAGGGCGACGGGCAG
GAGGATAATGAATTTATTCCGACTTTCACCAAGGAAATTGGTCAAGGTACAATAAGCAACACTGAGACGTTAAGA
AACCTATTCAACCCCAACGAGGCAGAACCTGTATCACAATTCAAATTGATTGAAGATTCTGATAATGATATTGAT
CATGCGAAAGATGTTGATGTGAACCAACTGGAGGAAGAAGTAAGTAAATCATCTGACACCCTAGGTTTGACATCG
GCTCCCGTACCACATGTATCTAGAGATAAAGATAACAAAAATTTCTTGTTTTTCCCTCATTTGCAATCGCCATTT
TTAGTAGGACAAACACAGTTGAGTAAAGTAAGGGCTCCTGGAAGAGAAACAATGCTATCGAACTGGGATGAGGAG
TTCTGGGCTAACAGAGGTAATTGGACCCGTGATATGAGGCGCAAAATGAAAGATGCATTGAAGCACCGTAAGAGG
AAACAATCAAAGAGCGGGCTTCTTCTATAG

>YJR096W 0.78 Cold
ATGGTTCCTAAGTTTTACAAACTTTCAAACGGCTTCAAAATCCCAAGCATTGCTTTGGGAACCTACGATATTCCA
AGATCGCAAACAGCCGAAATTGTGTATGAAGGTGTCAAGTGCGGCTACCGTCATTTCGATACTGCTGTTCTTTAT
GGTAATGAGAAGGAAGTTGGCGATGGTATCATTAAATGGTTGAACGAAGATCCAGGGAACCATAAACGTGAGGAA
ATCTTCTACACTACTAAATTATGGAATTCGCAAAACGGATATAAAAGAGCTAAAGCTGCCATTCGGCAATGTTTG
AATGAAGTCTCGGGCTTGCAATACATCGATCTTCTTTTGATTCATTCGCCACTGGAAGGTTCTAAATTAAGGTTG
GAAACTTGGCGCGCCATGCAAGAAGCGGTTGATGAAGGATTGGTTAAGTCTATAGGGGTTTCCAACTATGGGAAA
AAGCACATTGATGAACTTTTGAACTGGCCAGAACTGAAGCACAAGCCAGTGGTCAACCAAATCGAGATATCACCT
TGGATTATGAGACAAGAATTAGCAGATTACTGTAAATCTAAAGGTCTCGTCGTCGAAGCCTTTGCCCCATTGTGT
CACGGCTACAAAATGACTAATCCAGATTTATTAAAAGTTTGCAAAGAGGTGGACCGTAATCCAGGTCAAGTTTTG
ATTCGTTGGTCTTTACAACACGGTTATTTACCACTACCGAAGACTAAAACTGTGAAGAGGTTAGAAGGTAACCTT
GCAGCCTACAACTTTGAACTGTCAGACGAACAGATGAAATTTCTTGATCATCCTGATGCTTATGAGCCTACCGAT
TGGGAATGCACAGACGCGCCATAA

>YGL186C 0.72 Cold
ATGAATAGGGACAACATGGACACAACAAAGCGCAAAGAAGATCATACAAAGCATACTACTGATGTAATAGAATTT
TACGAAGAAGGAACCGCAGCTTCTTCTTTAAACATAGCCACAGAAAAGGCCAACTCAAGTCCGTCCATTTTGAGA
AGAATTATCAACAGGGCGGCATGGCTTTCTAAGAAAGTGGACGCAATGGGAGTAGAGTCTACCGGTATTCAGAGG
ATATCTCCCTATGAACGAGGAACATCAAAGAAACAATTTTTGCATGTCGCGGGGCTATGGTTGAGTGCCACAGGT
GGTTTGTCTTCCATGTCGTCGTTTTTATTAGGCCCTCTGCTTTTCGGTTTAAGCTTTAGAGAATCCGTAGCCTCC
AGTTTAATATCGGTAACAATTGGTTGCTTAATTGCTGCCTATTGCTCGATCATGGGACCGCAGTCCGGTTGCAGA
CAGATGGTAACAGCTAGATACCTGTTCGGATGGTGGTTTGTTAAATTAGTTGCATTAGCTTCCATTATCGGTGTC
ATGGGTTGGTCTGTGGTTAATTCCGTGGTTGGGGGTGAAATGTTAGCTGCTATTTCTAATGATAAAGTACCCCTA
TGGGTAGGGATTGTCATAGTGACCGTATGCTCCTTTTTAGTTGCCATTTTCGGTATCAAACAAGTCATTAAAGTG
GAAACTTACTTATCCGTACCCGTCCTGACAGCATTTTTACTACTGTACATTTCATCAAGTGATAAATACAGCTTC
GTCAATGCATACGTTTCAAAGGGAAATTTGGACTCTTCCACCCGAAAGGGCAACTGGATGAGTTTTTTTTCATTA
TGCTATAGTATTACGGCTACTTGGGGGTCTATTACAGCAGACTATTATATATTGTTCCCAGAAGATACACCATAT
ATTCAAATTTTTTGCCTAACGTTTTTTGGAACATTTTTGCCTACCTGTTTCGTCGGAATCTTAGGTCTCTTACTA
GCTTCTGTTGCCATGTCGTATAAGCCTTGGTCAGTCGAGTACGATTCCCATGGAATGGGTGGTCTACTTTGGGCA
GGTTTTCAGCGTTGGAACGGCTTCGGAAAGTTCTGCGTGGTAGTACTGGTATTCAGTTTGGTATCCAACAACATC
ATCAATACGTATTCGGCAGCATTCTCCATCCAACTTTCTAGTGTGTTTTGTGCAAAAATCCCTCGCTGGTTCTGG
TCTATTGTTTGTACTATAATTTGTTTGGTATGCGCATTAATAGGTCGTAATCATTTTAGTACTATTTTGGGAAAT
TTCCTACCAATGATAGGCTACTGGATTAGCATGTACTTTATCTTATTATTCGAAGAGAATTTAGTATTCAGAAGG
TTTTTCTTACATTTGTACACCAAAGAGTTTCCTACAGTTACAGGAGAAATAAATGGACCGGAACTTGTTGGATCT
TCAAAAGAAGTGGAGAAAGATGCAGTTACTAATATCCATTTGTTAAAAAGAAAACATAAGGTGACTAAACATCGT
TACAATTGGGACAAATGGGAAGATTATGAGGTTTTAACGCACGGTTATGCAGCAACATTTGCGTTTATTGTAGGA
GTTGCTGGTGTGGTAGTTGGTATGGCGCAAGCCTATTGGATTGGCCCAATTGCTGCCAAATTTGGTGAATATGGT

GGTGATGTCGCAATGTGGTTAAGTATGGCGTTCTCAGGTGTTGTATACCCTCCTTGTAGATACTTAGAGCTACGT
AAATTTGGTCGTTAA

>YDR264C 0.73   Cold
ATGGTGAACGAATTAGAGAATGTGCCACGGGCGTCGACCTTGACGAATGAAGAGCAAACGGTTGATCCTAGCAAT
AACGACTCACAGGAAGATATTAGTCTGGGTGATTCTAACGAAATTACTTCGTTGGCATCATTAAAGGCTATCCGA
TCAGGAAATGAAGAGGAAAGCGGGAACGAGCAGGTGAATCATAATGATGAGGCCGAAGAAGATCCTCTGTTAACT
CGCTACCATACCGCTTGCCAAAGAGGTGATCTAGCGACTGTCAAAGAAATGATACATGGAAAGTTGCTCGAAGTT
AATAACGACGGTGACTCTACGGAACATATCACTGGTTTGCATTGGGCAAGTATTAATAACAGACTTTCTGTAGTG
GATTTTCTAGTTTCACAAGGCGCTGATGTCAATGCAAGGGCTGGTGCTTTGCATGCCACACCATTACATTGGGCT
GCAAGGTATGGGTACGTGTACATTGTCGATTTTTTGCTTAAGCATGGAGCTGATCCCACCATGACCGATGACCAA
GGTTTCAATCTACTACACTTATCTGTGAACAGCTCAAATATAATGCTAGTTCTATACGTTTTATTCAACGTTGTC
AGCAAAGGTCTTTTAGATATAGATTGCCGAGACCCAAAGGGCAGAACTTCACTACTATGGGCTGCTTACCAGGGA
GATTCCCTAACTGTAGCAGAATTGTTGAAATTTGGTGCCAGCATAAAGATCGCAGATACAGAAGGATTCACCCCA
CTGCACTGGGGCACTGTAAAAGGGCAACCACATGTACTAAAATACTTGATTCAAGATGGTGCAGATTTTTTCCAA
AAAACGGATACCGGAAAAGATTGCTTTGCTATTGCTCAAGAAATGAACACTGTGTACTCACTTAGAGAGGCTCTA
ACACATTCTGGATTCGACTACCATGGTTATCCCATTAAAAAATGGTTCAAAAAAAGTCAACATGCTAAGCTCGTT
ACATTCATAACGCCATTTCTATTTCTAGGAATCGCATTTGCTCTTTTCTCTCATATTAACCCTTTGTTTGTAATC
ATTGTCCTTTTTTTATTGGCCATTGCCACTAACAAAGGCTTGAACAAATTCGTTCTGCCGTCATATGGACGAATG
GGTGTCCACAACGTTACCTTGTTAAGGTCACCTTTGTTATCCGGTGTTTTTTTTGGCACTTTACTTTGGGTGACT
ATCGTTTGGTTTTTCAAAGTTATGCCTCGAACATTTTCTGATGAACAATATACTAACATCCTAATGCTGGTAATA
CTTGTTTCTGTATTTTACCTTTTTGGGCAGCTTGTTATTATGGACCCAGGATGTTTGCCAGAAGAAACTGATCAT
GAGAACGTGCGACAAACAATCTCGAACTTGCTCGAAATCGGTAAATTTGATACGAAGAACTTTTGTATCGAAACT
TGGATTAGAAAACCTCTAAGAAGCAAATTTTCGCCCTTAAACAATGCTGTGGTTGCAAGATTTGATCATTACTGC
CCTTGGATCTTTAATGATGTCGGACTCAAAAATCACAAAGCATTTATATTTTTTATTACATTAATGGAAAGTGGT
ATTTTCACATTTCTTGCACTTTGCCTGGAATATTTTGATGAACTTGAAGATGCACACGAAGATACATCCCAGAAA
AATGGCAAATGTTTCATCCTAGGCGCTTCAGATCTCTGTTCGGGATTAATATATGATAGATTTGTCTTTCTGATA
TTATTATGGGCTTTACTCCAATCTATTTGGGTTGCAAGTTTGATCTTCGTCCAAGCCTTTCAAATATGTAAAGGA
ATGACCAATACAGAATTCAATGTTTTGATGAAAGAGAGTAAGTCCATTGGCCCCGATGGGTTATCTTTCAATGAA
AACTTCAATACTACTCCAGAAGGATTTGCGCCGTCGATTGATCCGGGTGAGGAGAGTAATGATACTGTACTAGCA
CCAGTCCCAGGCTCAACGATAAGGAAACCACGCACTTGTTTTGGCGTTTGTTACGCGGTGACTGGTATGGATCAG
TGGTTAGCGGTTATAAAAGAAACCATTGGCATCAAGGATAGCACCGGACATAATGTGTATTCGATTACATCAAGA
ATACCAACAAATTACGGTTGGAAAAGAAATGTTAAAGACTTTTGGCTTACAAGCGATATAAATGCGCCTTTGTGG
AGGCGAATTTTGTATCCTCCTTCGGGCTCTAAAGCTTTGCTCAACGGGATAGAAGTTGACTATTTTAAGCTTTAC
AAATTGCCAAATAAGGATGTTGAACAAGGAAATGATATGGTATGA

>YAL010C 0.81   Cold
ATGCTACCCTATATGGACCAAGTACTAAGGGCATTTTATCAGAGCACCCATTGGAGTACGCAAAATAGCTACGAG
GATATAACGGCCACATCGAGAACATTATTAGATTTCCGAATTCCCTCAGCAATACACCTGCAAATTTCCAACAAA
TCTACTCCCAATACATTCAATTCTTTAGATTTTTCTACGAGGTCCAGGATAAATGGTTCTCTGAGTTATTTATAC
TCCGATGCACAGCAATTGGAGAAATTCATGCGCAACTCTACTGATATCCCATTACAAGATGCCACCGAAACATAC
AGACAATTGCAACCAAACCTCAATTTCAGTGTTAGTAGTGCGAATACGTTGAGTAGTGACAACACCACAGTCGAC
AATGACAAGAAATTACTACATGACTCGAAATTTGTTAAAAAATCCCTTTATTATGGTAGAATGTACTACCCCAGC
TCTGATTTAGAAGCAATGATAATAAAACGACTAAGTCCACAAACCCAATTTATGCTTAAGGGTGTCAGTAGTTTC
AAAGAAAGCTTAAACGTTTTAACGTGCTATTTTCAAAGAGATTCTCACCGCAATTTACAGGAGTGGATATTTTCC
ACCAGTGATCTATTATGTGGTTATAGAGTATTACACAATTTCCTTACCACGCCTTCCAAGTTTAACACCTCACTG
TACAATAATTCTTCGTTGTCGCTTGGTGCTGAATTTTGGTTAGGGTTAGTAAGTTTAAGCCCCGGTTGTTCGACA
ACTTTAAGATATTACACACATTCTACAAACACAGGACGACCACTAACTTTGACATTATCTTGGCAACCATTATTC
GGCCATATATCCTCCACATATTCGGCCAAGACAGGGACAAATTCTACTTTTTGCGCGAAGTATGATTTTAATCTT
TATTCGATTGAATCAAATCTTTCATTTGGGTGCGAATTTTGGCAAAAAAAGCATCATTTGCTTGAAACCAATAAA
AACAATAATGATAAATTAGAACCAATCTCCGACGAATTGGTTGATATAAATCCAAACAGCAGAGCGACTAAACTA
CTGCACGAAAATGTACCGGATCTGAATTCAGCTGTTAACGATATTCCTTCTACACTAGATATACCTGTTCACAAA
CAAAAGCTATTAAATGATTTAACTTATGCATTCTCGTCGTCATTAAGAAAAATCGATGAAGAAAGATCTACCATC
GAAAAATTTGATAACAAAATAAATAGTTCCATTTTTACCAGTGTTTGGAAATTAAGCACGTCATTACGTGACAAG
ACTTTAAAACTATTATGGGAAGGCAAATGGAGGGGATTTTTAATATCTGCCGGGACAGAGCTGGTATTCACTAGA
GGCTTTCAAGAAAGTTTATCCGATGATGAAAAGAATGATAATGCAATATCTATATCAGCAACTGATACAGAAAAC
GGCAATATACCAGTTTTCCCGGCAAAGTTTGGCATACAATTCCAGTACTCCACATGA

>YNL329C 0.67   Cold
ATGAAGGCATCGCTTACGTTTAGTCTCTCCGGAATATACGCCCCCTGTAGCATTTCTAGGGATATATATCTCGAA
TACGGCGATAAAAAAGCTGAATGTTTATATGGGACAATTAGATTGCCTCAGTACGGTCCAGGTTGCACGCCTGGT
AAAATTGTACATTGTGTCCTGGATGATTCACTGCCCTTCTGTAGCATTGTTGTACCTTCCAAACTTTTTGGGTTT
ATGCCGACACAGCCCACTATGGATTTTTGCTATTTTGAACCCATATTAGACAATGTAGTACCTGTACTGGATAGC
GTAACGTTTCTGATTAATGAACAATTGTACTCAAAACTAATGGACCTACCGCAAGAGATGCAACAAATACAATTT
TTGCATTACAAATATAACATCAACTCCATGGAAACCGTGGTTCATTCTCGAGATATTTTAACATCTGGTCTTTGC
CAAATTTTAAACTGCTCACCTTTCCCGCAGGGCCTTGTTGATTTTACAGAAACCCAATTGATATTAGTAAATGAT
ACTGAGCAGAAACTAAGTGCTCTAAAATATGCTAACGAAGATGAAGAATATGCATTACCTAAAATAGGGACAAAT

TCTGCTTTATCAATTGATCTTGAATCTCTCCCCTGCACAATTTCAAGAGATCTTTTGCGACCTGCACCTCACATC
AATGATGATAATTCAATATATGCCTTTACAGACGCTGAAACTCTACTGCGCCTGGATGTCACAAGTGGATCGTTT
ATAACAGTGTCAAATATGGGTTGCGTCAGGCTAGTTAAGCTGTTTGTTTTACTACTCCCCAACGGCTTTAAAAAA
AGAACAATATACGCACCTCCCAAAATAATAGCAAGCTTTCCAGACTGCAGCGTCGTGACAATATCAAAGTCCAAT
ATAGGCCATACAGATATTCCCATTGCTAATCAAGTTTTCATCTCAAGAGTTGGAGGCTGGCTTCAATCTCAGAAA
TGCTTTCAAAATATTATTCTAACAACCTTGAAAAAGTTTTTAGCGAGAGCAAAAGGATTCTTTGTCAAAATGAT
TTAATCCCAATCGCTTTTGATTCAAGTATGGCTGATCTCAATATAGCAGAGGAAAATGATGAGTCTGATGACGAA
GATGAGCTAGGGCAGTACTACAAGAACGATTCCCTTGTATGGTTCTTTGTCACAAGCGCAGAGCTCGATTGCTTC
TCTAAAGATAATTCTCATTTTATTATTGATCCCAATCGGACGAAGCTAATAACAACTAATATCACTAACAGAAGA
CCATTACCCTTGAGTAGATCCAATCTTCAGAGATATTACGGTTTTGCTGAAACTTTTTACTATGATTTGCATATC
TTCCCCTATGTAAGACAATTGGTTAACATATTAGAAACTTCCTTCAATTGTTCTCAAAGGGGAATCACCCTAAAT
GCCTCTGTGTTACTTCATTCCACTACAAACAATGTGGGCAAGGCTACAATGGTGAGATTTGCTTCAAAATATCTT
GGTATACATCTACTGGAGATAGATTGCCTTTCGCTCACTTCAAATTCAAGACAACTGGATTCAACATCTAAGATT
ATTGGTTACATTAGGGCTAAATGTGAAAATGTCCTACCGTACGCATCACCTGCTGTGATTTTTTTGGCTCATTTG
GATTCAATTTTGCTTGACGTAAACGCTAATCAAGATCCAGAGGCAATCAAATTACAAAAATCTATAAACTTCGAA
ATGTCTAAACTTCTGGACGATTTTACGTTTAAATTCCCAGGAACTACATTTGTTGGCTCCGTAAATAACATAGAT
AACGTGCCTTCCAGCTTTAGATCACATATGAGATTTGAGATTCTCGTACCCGTTCCGTCTGAAGCACAGAGACTG
CGTATCTTTCAGTGGTACTTATCTTCACATGAACTAAACAGAGATGTTCAACAAAAGGTTCCAGTATCTTACATG
GATAATATTTCATTTTCATCACTCTCTTCATATTCTGCTGGTTTAACTCCTTTAGACATCAAATCAATTGTGGAA
ACGGCACGAATGACGGCTACTGCGCGTTTTTACCAAGAATCAAAGAAGTGTGGATGGCTTCCGCAATCAATTTTG
ATCACTCAGGAGGATTTATCAAAAGCTACTTCGAAAGCTAGGAACGAATTCTCGGTTTCGATTGGTGCCCCACAA
ATCCCTAACGTAACTTGGGATGATATAGGTGGTATTGATTTTGTTAAAGGTGAAATATTGGACACAATAGACATG
CCGCTAAAACATCCTGAATTATTTACCTCAGGTATGAAAAAGAGAAGCGGTATTCTTTTTTATGGTCCACCGGGT
ACAGGTAAAACTCTAATGGCTAAGGCCATTGCAACAAATTTTTCTTTAAATTTTTTTAGTGTTAAAGGCCCTGAA
CTGTTGAATATGTACATTGGTGAGAGTGAAGCTAATGTGCGCAGAGTGTTTCAAAAGGCGAGAGAGGCTAAACCT
TGTGTCATATTTTTCGATGAAATCGATTCAGTAGCACCCAAACGTGGTAATCAAGGTGATTCGGGTGGTGTTATG
GATCGTATCGTTTCACAGTTACTAGCAGAGTTAGATGGTATGAGTACCGACGCTGACGGTGTATTTGTTATCGGA
GCAACAAACAGACCAGACTTATTGGACGAAGCACTACTAAGACCAGGACGATTCGATAAATTGTTATATTTAGGC
ATTCCGGATACGGATACCAAACAATTGAATATTTTAGAGGCTCTGACTCGCAAATTCGTGCTCGACAACGACGTA
AAGCTTATTGAGTTGGCAAAGCTATGTCCATTTAATTACACCGGGGCAGATTTTTATGCTCTCTGCTCAGATGCA
ATGCTTAACGCCATGTCAAGAATTGCACGCATGGTAGAAAAAAAAGTTTCTCAGCATAACGAATTGACGGGAGAG
AATATTTCTACACGTCGCTGGTTTGATAAGATTGCGACAAAAGAGGATACTAAGGTTGTCGTAAAAATGGAAGAC
TTCTTGAAAGCACAAGAGCAGCTCACCCCAAGTGTGTCGCGGGCTGAACTGAATCATTATGAAGCGGTGAGAGCT
AATTTTGAAGGTGCTTAA

>YDL037C 0.77   Cold
ATGAGCCAACAGAATATACTACATTATGATATGGATGTTACTTCCGTATCATGGGTCAAGGATAACACATACCAG
ATTACTATTCATGTCAAAGCCGTAAAAGATATCCCTTTGAAATATTTATGGTCTTTAAAGATTATTGGTGTCAAC
GGTCCCTCAAGCACCGTACAACTTTACGGCAAAAATGAGAAGACATACCTAATCAGTGATCCAACAGATTTTACA
TCCACTTTTCAGGTTTATGCTTACCCTTCTTCTGACGGTTGCACAGTTTGGATGCCAAATTTCCAAATCCAGTTC
GAATATCTACAAGGTGATGCTGCTCAGTACTGGCAAACTTGGCAATGGGGGACAACCACCTTCGATTTATCGACA
GGTTGTAACAATTATGACAATCAAGGCCACTCACAAACGGATTTCCCTGGATTTTATTGGACCTACCAGTGCAAG
GGAAACAATGATGGCACTTGTACAAAAGCCTCGTCTTCATCCATTACTACTTCATCCATTACTACTTCATCCACT
ACTACTTCATCCACTACTACTTCATCCACTACTACTTCATCCTCTACTACTTCATCCTCTACTACTTCATCCTCT
ACTACTTCATCCTCTACTACTTCATCCTCTACTACTTCATCCTCTACTACTTCATCCTCTACTACTTCATCCTCT
ACTACTTCATCCTCTACTACTTCATCCTCTACTACTTCATCCTCTACTACTTCATCCTCTACGAAAACTTCCACT
ACTACGTCATCTACTGTGAAAAGCTCTTCCACTACATCCATTGATTTTACTACTTCCGTTGACTCACATACTTCA
TCAAGCGTAGCCGATATTTATCGTTCAAGAACATCTACTGATGTCACCACATTAGCAGCTTCAACTAGTCCTTTC
AGCAGTTTCACTTCAAGTGACTCTAGTAGTTCAAGCGATGTAACCAGTTCAACCATTCAAACTACTTCTGTTGAT
CCAACCACTTAA

>YFL025C 0.82   Cold
ATGGGTATCAGGAGATTAGTTAGTGTTATAACGCGGCCCATAATTAATAAAGTTAACTCATCTGGTCAATACTCG
AGAGTACTCGCTACGCGTGAAGACCAGGATAAAGCTTCCCCCAAATATATGAACAATGATAAGATAGCAAAAAAA
CCTTACACATATCGCCTTTTCTCTATTCTTGGCATTTTAAGCATTTGCTCGCTTTTATTGATATCGCTACTGAAA
CCTTTTAATGGTGCAGATGCTCCTCAGTGTGAATCTATCTATATGTTTCCATCTTACGCAAGGATCGATGGATTT
GACGAGAGGTATACACCACTGGCACACAAGTATCATCTTTACTTGTATCGAGAACAAAGCGTAGACCGAGAACCT
TTAAATGGTGATGAGTTACAACTTGATGGTATCCCTGTACTTTTTATACCCGGAAATGCAGGTTCTTTTAGGCAA
TGCAGATCGATTGCTTCCGCTTGCTCCAATATATATTTTGATTCCAATACCAGGGCGACTCTGCGTAACGAAAAT
GTGCGAAATTTGGACTTCTTTACGGCAGATTTCAATGAAGATTTCACTGCGTTTCATGGTGAAACAATGCTTGAT
CAAGCTGAATACCTAAATGATGCCATTAAGTACATCTTGTCTCTTTACGAAAGGACCCCTGATTACCCACATCCA
AAACCGCAGTCAGTTATTATCGTGGGACATTCCATGGGCGGTATTGTATCCAGGGTCATGCTAACTTTGAAGAAC
CACGTACCGGGAAGTATAAGCACCATATTGACCTTATCATCACCTCATGCTGCTTCACCAGTAACCTTTGATGGT
GACATTTTAAAGCTTTACAAAAATACAAACGAATATTGGAGAAAGCAGTTATCCCAAAACGATTCTTTTTTCTCT
AAGAACATTTCATTGGTTTCCATTACAGGCGGTATATTGGATACTACTTTACCTGCAGATTATGCATCTGTAGAA
GATTTGGTTAGCTTAGAAAATGGGTTCACCAGCTTCACAACTACTATTCCAGATGTTTGGACTCCAATTGATCAT
TTAGCCATTGTTTGGTGCAAGCAATTAAGAGAGGTACTAGCGCGCCTTTTATTGGAAAGTATAGATGCCTCCAAA

CCTGAAAAAGTTAAGCCATTGAATCAGAGATTGCAAATCGCTAGAAAACTATTGCTTTCAGGGTTTGAAGACTAC
TCATGGATGAATAGTAAGTTAAATTATCCTCAAGAAAACTTACAAGAATTTAGTGACAACTTCTTTTCTGATTAC
GCTACTTTGGAAATGAACGATGTCCTAGATTTTGAAATGTTTAACCTGGAAAAGTGGCACAACAATTATACAAAA
ATAAATATTCCGAGTAACATCTCCTCCACTGAACACCTGCATTTCACATTATTAACTAGTCTCGATATGCCTATG
ATCTATTTCTGTACAGAGTCCAGGGTGAATCTAAGCTGTATAACAGCTGTTGATAGTATTCTAACAGTGCCCAGG
TCTTCAAAAGATACTCAGTTCGCAGCTGATTCTTCTTTTGGAGAAGCAAAAAATCCGTTTAAAGCCGTTAGTGTT
GGCAAGAATATACTACAGAAGTACGACTACTTGATGATAAGCAAACCCACTTACGGTGAATTTTCAGAACAAGAA
GGCATGGAGGACAATCAGGGGTTTCTTTTGGCATTACTGCGCAACGTCTCCAATGTACAAATAGTCAATACTACA
CCTTCTCAAATATTGCTCTTTGGAGAACAACTACACTTGGATGGCAAAGACATCGAGCAGGTTATAAGTTTCAGT
AATTTGTGGGATTCTTTATTATCGTACAAATTAGAAACCAAGATTGAGGCAAGCAACGAAGGTATTGCTTCCGAA
GAGACATTGTTTCAGCCTTTCATAAGGCAATGGGTTTACGAACCTTTCGAGTCTAAATGGCATCTAAATATTATA
AACAAAAGTCTCGATATAAATATGCATAATGTAGCACCGTTTATCCCATTAAATGAAAGCGAACCGAGATCGCTA
CAATTAAGTTTTTTATTCCACCCGGAATGTCCTTAGAAGCTAAAATGACTATTAATTGGTCTTTAACTTTGAAA
ATGCTGTTTATCAGGTACAGATTAGCACTAGCATCTTTTCCAGTCGCGTTTATAGCACTCGTACTGTCATACCAG
TTCTACTGGTACAACAAGACATCTGAGTTTCCTTCCTTTGATAGCACTTTGGGATATATTTTAAGAAAGCACGGA
ATATTAATGTTTTTTACGCTGTTCTTAGCTTCGCCTGTGGTTAATAATAAGTTGGTACAACGAATACTTTATTTA
TTGGATCCTGTCGGTTTAAATTATCCATTTCTGCTATCCGAGAGAAACATGCATGCAAATTTTTATTATTTGGGG
ATCAGAGATTGGTTTATGTCAACTATTGGAATTCTCTTTGGGGTTATGACAGTTGGATTACTAGCACTAGTTTCT
AAGATTTTTGGATCCTTGGAAATTTTAGTAATATTCCTTCAACGGAAGTTATCAAAGAAAAACACTGAAGATAAA
GAGGCTTTTGATACTATTGAACATAAAGCGTATGGTAAGGGAAGATTAATGGCATCTGTGCTTTTATTACTGTTG
GTATTTTTTCACATACCATACCAGATGGCGTTTGTTATCTCATTAGTAATCCAAATTGCTACCTGCATTAGAGTA
GCGCTGTTGAAACTATCAAACAACGAGCAGAAATTGAACTTACTAAATTATAATATGACGTTGCTTCTGCTTTTA
TTATTTGTTAGTGCCATTAACATCCCTATCATTATTGTTTTCCTGCATAATGTTGCCATCAAATGGGAAACTTCA
TTTAGATCCCATCACAATATACTAGCAGTGGCACCAATTATTTTTTTGGTTGGAAATAATTCTATTTTCAAAATG
CCAAACAGCGTACCTTTAGACACGTGGGATGGTAAAGTTACCATCATTTTATTTGTGTACTTAACCGTTTTCAGT
TTCATTTATGGAATAAGAAATCTGTACTGGATACACCACTTGGTGAACATAATATGTGCCTGGTTGCTATTTTTC
GAAACAATACATTAG

>YDR293C 0.68 Cold
ATGTCTAAAAATAGCAACGTTAACAACAATAGATCCCAAGAGCCAAATAACATGTTTGTGCAAACCACAGGAGGT
GGTAAAAACGCCCCAAAGCAGATTCATGTTGCACACAGACGTTCCCAAAGTGAGTTGACAAATTTGATGATTGAA
CAATTCACTTTGCAGAAGCAGTTGGAGCAAGTTCAAGCACAGCAGCAACAGTTGATGGCTCAGCAACAGCAATTG
GCACAACAGACAGGACAATACCTGTCAGGAAATTCTGGCTCTAACAATCATTTCACGCCTCAACCGCCTCACCCT
CATTACAACTCAAACGGTAATTCACCTGGTATGAGTGCAGGTGGCAGCAGAAGTAGAACTCACTCCAGGAACAAC
TCCGGATATTATCATAATTCATATGATAACAATAACAATAGCAATAATCCTGGGTCTAACTCACACAGAAAGACG
AGTTCACAATCCAGCATATATGGCCATTCCAGAAGACATTCTTTAGGTCTAAATGAAGCGAAAAAGGCTGCTGCG
GAAGAACAAGCTAAAAGAATATCTGGGGGTGAAGCAGGCGTAACTGTGAAGATAGATTCTGTTCAAGCTGATAGT
GGCTCAAATTCTACTACAGAACAATCTGATTTTAAATTTCCACCACCACCAAATGCTCATCAGGGCCATCGTCGC
GCAACTTCAAACCTATCACCTCCCTCTTTCAAATTTCCTCCAAACTCTCACGGGGATAATGACGATGAATTCATA
GCAACCTCTTCAACGCACCGCCGTTCAAAGACAAGAAACAATGAATATTCTCCAGGCATTAATTCCAACTGGAGA
AACCAATCACAGCAACCTCAACAGCAGCTTTCTCCATTCCGCCACAGAGGATCTAATTCAAGGGATTACAATTCC
TTCAATACCTTAGAACCTCCTGCGATATTTCAGCAGGGACACAAACATCGTGCCTCTAATTCATCAGTTCATAGT
TTCAGTTCACAAGGTAATAATAACGGAGGTGGACGTAAGTCCCTATTTGCACCCTACCTTCCCCAAGCCAACATT
CCAGAGCTAATCCAAGAAGGGAGACTAGTAGCTGGTATATTAAGAGTTAATAAAAAGAATAGATCGGATGCCTGG
GTCTCTACAGATGGCGCTCTTGATGCGGATATTTACATTTGCGGCTCCAAAGATCGTAATAGAGCACTTGAAGGT
GATTTAGTCGCGGTAGAACTATTAGTTGTGGACGATGTTTGGGAGTCCAAGAAAGAAAAGGAAGAAAAGAAGAGG
AGAAAGGATGCCTCTATGCAACACGATCTAATTCCTTTGAACAGTAGTGACGATTACCACAACGATGCATCTGTT
ACTGCTGCAACAAGCAACAATTTTCTATCTTCTCCCTCCTCGTCTGATTCGCTAAGCAAGGATGATTTATCCGTC
AGAAGAAAGAGGTCATCTACTATCAATAATGATAGTGATTCCTTATCATCTCCTACCAAATCAGGAGTAAGGAGA
AGAAGTTCATTGAAACAACGTCCAACTCAAAAGAAAAATGACGATGTTGAAGTTGAAGGTCAGTCATTGTTATTA
GTTGAAGAAGAAGAAATCAACGATAAATATAAGCCACTTTACGCAGGCCATGTCGTTGCTGTTTTGGACCGTATC
CCTGGTCAGTTATTTAGCGGTACATTAGGTTTGTTGAGACCATCCCAACAAGCTAATAGCGACAATAACAAACCA
CCACAAAGCCCAAAAATTGCTTGGTTCAAGCCTACTGATAAGAAGGTGCCATTAATTGCAATTCCTACAGAATTA
GCTCCAAAGGACTTTGTTGAAAACGCTGATAAATACTCCGAAAAGTTATTCGTTGCCTCTATTAAACGTTGGCCA
ATCACATCTTTGCATCCATTTGGTATTTTAGTTTCCGAACTTGGAGATATTCACGATCCTGATACTGAAATTGAT
TCCATTTTAAGGGATAACAATTTTCTTTCGAATGAATATTTGGATCAAAAAAATCCGCAAAAAGAAAAACCAAGT
TTTCAGCCGCTACCATTAACGGCTGAAAGTCTAGAATATAGGAGGAATTTTACGGACACTAATGAGTACAATATC
TTTGCAATTTCCGAGCTTGGATGGGTGTCTGAATTTGCCTTACATGTCAGGAATAACGGAAATGGTACCCTAGAG
CTGGGTTGTCATGTTGTTGATGTGACCAGCCATATTGAAGAAGGCTCCTCTGTTGATAGGCGTGCGAGAAAGAGG
TCCTCTGCGGTGTTCATGCCACAAAAACTTGTCAATTTATTACCACAATCGTTCAACGACGAACTGTCGTTGGCC
CCTGGCAAGGAATCAGCCACGCTGTCGGTTGTTTACACTCTAGACTCATCTACTTTAAGGATTAAATCTACTTGG
GTAGGCGAATCTACAATTTCCCCCTCAAACATCTTGTCTTTAGAACAATTAGACGAAAAATTATCTACTGGAAGT
CCCACTAGCTACCTCTCTACTGTACAGGAAATTGCTAGATCATTTTATGCTAGAAGAATAAATGATCCAGAAGCT
ACATTACTTCCCACCCTGTCCTTATTGGAAAGCTTGGATGACGAAAAAGTTAAGGTTGACTTGAACATCCTGGAT
AGAACTTTAGGCTTTGTTGTAATTAATGAGATTAAAAGAAAGGTCAACTCCACTGTTGCAGAGAAAATTTACACC
AAACTTGGTGATCTAGCTCTTTTGAGAAGGCAGATGCAACCCATTGCAACCAAGATGGCGTCATTTAGAAAGAAA
ATTCAAAATTTTGGTTACAATTTTGATACCAATACGGCGGATGAATTAATCAAAGGGGTGCTAAAAATTAAAGAT

GACGATGTTAGAGTCGGAATTGAAATTTTACTGTTTAAAACCATGCCAAGAGCTAGATACTTTATTGCTGGCAAA
GTAGACCCGGACCAATATGGGCATTATGCCTTGAACCTACCTATCTACACACATTTCACAGCGCCAATGAGAAGA
TACGCTGATCATGTCGTTCATAGGCAATTAAAGGCCGTTATCCACGATACTCCATACACCGAAGATATGGAAGCT
TTGAAGATTACCTCCGAATATTGTAATTTTAAAAAGGACTGTGCTTATCAAGCACAGGAACAAGCAATTCATCTA
TTGTTGTGTAAAACAATCAACGACATGGGAAATACTACAGGACAATTATTAACAATGGCTACTGTCTTACAAGTT
TACGAGTCCTCCTTTGATGTATTTATTCCAGAATTTGGTATTGAAAAGAGAGTTCATGGAGATCAACTACCTTTG
ATCAAAGCTGAGTTTGATGGTACCAATCGTGTCTTGGAATTGCATTGGCAGCCCGGCGTAGATAGTGCAACTTTT
ATACCAGCAGATGAAAAAAATCCAAAATCCTATAGAAATTCCATTAAGAACAAATTCAGATCCACAGCCGCTGAG
ATTGCGAATATTGAACTAGATAAAGAAGCGGAATCTGAACCATTGATCAGCGATCCATTGAGTAAGGAACTCAGC
GATTTGCATCTAACAGTACCAAATTTAAGGCTACCATCTGCAAGCGACAACAAGCAAAATGCTTTAGAAAAATTC
ATTTCTACTACTGAAACCAGAATTGAAAATGATAACTATATACAAGAAATACATGAATTGCAAAAGATTCCTATT
CTATTGAGAGCTGAGGTGGGGATGGCTTTGCCATGTTTAACCGTCCGTGCATTAAATCCATTCATGAAGAGGGTA
TAA

>YDR258C 0.73   Cold
ATGTTAAGACAAGCTACAAAAGCACCAATACAAAAATATTTACAGAGAACGCAACTATTAAGGCGAAGTACTCCA
CGAATATACACTATTGTACAGTGTAAAAGATCTATTTGCAGTTTTAATGCAAGACCTCGAGTAGCCAATAAACTT
CTATCAGATATAAAAACAAATGCATTAAATGAAGTCGCTATTTCAACCTGCGCCCTCAAGAGCTCATATGGATTG
CCCAATTTCAAAAGAACGTACGTCCAAATGAGGATGGATCCCAATCAGCAACCGGAAAAGCCAGCTTTAGAACAA
TTTGGTACAAACTTGACAAAATTAGCGCGCGACGGTAAATTAGACCCTGTCATTGGCAGAGACGAAGAAATCGCA
AGAGCTATTCAAATTTTATCAAGAAGAACAAAAAATAATCCATGTTTGATCGGTCGAGCTGGTGTCGGTAAAACC
GCCCTTATTGATGGGTTAGCTCAAAGAATTGTTGCTGGGGAGGTCCCTGACTCGTTGAAAGATAAAGATCTAGTA
GCTTTGGATTTGGGATCTTTGATTGCTGGCGCCAAATATAGAGGTGAATTCGAAGAACGTTTAAAAAAGGTTTTA
GAAGAAATTGACAAGGCCAATGGTAAGGTTATTGTTTTCATTGATGAAGTTCATATGCTGCTTGGCTTAGGTAAA
ACTGATGGTAGTATGGATGCTTCTAACATCTTAAAGCCAAAACTGGCCCGTGGGCTACGTTGTATCTCTGCAACC
ACTCTTGATGAATTCAAAATCATTGAAAAAGATCCGGCTTTATCGAGAAGGTTTCAGCCTATCTTATTGAATGAG
CCGAGCGTCTCCGATACGATTTCTATACTAAGAGGTTTAAAAGAAAGGTACGAGGTTCATCATGGTGTAAGGATC
ACTGATACAGCTTTAGTGTCTGCTGCAGTTCTTTCAAATCGTTATATAACAGACAGGTTTCTACCTGATAAAGCC
ATTGATCTAGTTGACGAAGCTTGTGCTGTACTACGTTTACAACATGAATCCAAACCGGATGAAATACAGAAACTG
GATCGTGCTATTATGAAAATACAAATAGAACTGGAATCTTTAAAGAAAGAGACCGACCCTGTTTCCGTGGAAAGA
AGAGAAGCGTTGGAAAAGGACCTAGAAATGAAAAATGATGAATTAAACAGATTAACAAAAATATGGGACGCTGAA
AGAGCTGAAATTGAATCTATCAAAAATGCTAAGGCAAATTTGGAACAAGCAAGAATTGAGTTGGAAAAGTGTCAG
AGAGAGGGTGATTACACTAAAGCATCCGAGTTGCGATATTCCAGAATTCCTGACTTGGAGAAGAAGGTAGCTTTA
AGCGAGAAGAGTAAGGACGGAGATAAAGTAAATTTACTTCATGATTCAGTTACTTCTGACGATATTTCTAAGGTT
GTTGCCAAAATGACTGGTATTCCTACAGAAACGGTGATGAAAGGCGACAAGGACCGCCTACTGTATATGGAAAAT
TCTTTAAAAGAAAGAGTCGTTGGCCAAGACGAGGCCATTGCTGCTATTTCTGATGCTGTACGTCTTCAAAGGGCT
GGTTTGACCAGTGAAAAGAGACCTATTGCCAGCTTTATGTTCTTAGGTCCTACTGGTACTGGTAAAACAGAATTG
ACCAAGGCTTTAGCTGAATTCCTATTTGATGACGAATCAAATGTGATTCGTTTTGACATGTCAGAATTTCAAGAG
AAGCATACTGTCTCTCGTTTAATCGGTGCACCTCCAGGTTACGTCTTAAGCGAATCTGGTGGCCAATTAACTGAG
GCTGTCAGAAGAAAGCCTTATGCTGTCGTTTTATTTGATGAATTTGAAAAAGCGCACCCCGATGTTTCCAAACTA
CTGTTGCAAGTGTTGGATGAAGGTAAGCTGACCGATTCCCTAGGCCATCATGTTGATTTCCGTAACACGATCATT
GTGATGACCTCAAACATTGGACAAGATATTTTATTAAACGACACAAAGTTGGGTGATGATGGTAAGATTGACACC
GCAACCAAGAATAAAGTTATCGAAGCAATGAAAAGATCCTATCCACCTGAGTTCATCAATCGTATTGATGATATT
CTAGTTTTCAATAGGCTATCCAAGAAAGTTTTGAGGTCAATTGTTGATATTAGAATTGCAGAAATTCAAGATCGT
TTGGCCGAAAAAAGAATGAAAATTGATTTAACTGATGAAGCGAAGGATTGGCTAACTGACAAAGGCTACGATCAA
TTATATGGTGCAAGGCCATTGAATAGATTAATTCATAGACAGATTTTGAACTCCATGGCAACTTTCTTATTAAAA
GGTCAAATTAGAAACGGCGAAACTGTTAGAGTGGTGGTTAAAGATACTAAGCTTGTCGTGCTACCAAATCATGAA
GAAGGCGAAGTTGTTGAAGAGGAAGCTGAAAAGTAA

>YGR025W 0.79   Cold
ATGACTGGCTATGAGCCCTTTAAGTTTTTCTCTATCCGCATGTCTTCAATTAATTCTCCATCAGTGATTTTTAAA
ACAATAAAAACTTTCAATCCGGGTAAAACTGAAGAATATAGAGCGAAAAAAGCGTATGTGCAGGACAAGGGATAC
TTACGCCAAAAAAGACCATGTCCATTTTATTATAGGATACCCACTTGCACGGCGTCAAAGTACTCAATTGGCTGT
GCTTCTTCAAAAAAACTAACATACACAAGAACCAAATTTAAATTCAACTTGGTTACCCAAAGAGAGAATTCATTA
TAG

>YNL215W 0.81   Cold
ATGGACAGTGAAGCAAGTGACATTGAGGCAGAGTTATCCGACAGCGTCTCAGCTGGTGGGGAAGAATACATTGAC
GATGATGATTATACGGAAGATATTGACGATCAGATAGTCACAGCCAAATCATCTAGAAGAACAGCACGTAGGAGT
GTACCTAAGGGTGTAAGAACCTCAAAACGCATTAGAGATAAAGAACTTTCTGTGGAGGTTGATGAAGATTATGAT
GAGGAGGAAGATGTATTGAGCCCTTCTAAGAAGCGCCATCTACACACCAGATCAATGGACAAAAGGCAAGTTGCC
GCAACAGCTTCTGAAAAATCTGATATTGGTGATAGTAAAGGTAATGATGGCGAAATAGAAGACGGTATATTGGAA
GAAGAAGAGAGCTTAGAGAAGGAACTGAATAGAGGTGGAGGAAAGGAGGTAGAGAAAAGTGAAGAATCTTACTAC
GCCCAAAACGATGTTGGACAGAAGGGGGAGGAAGAACAAGACGGGGAATCTGGAGGCTATGAAGATAATGAACCC
AGTATAAGTAAAGAGTCAGACGAATTGGTCTCTGTGGTAAACGGTAACGGTAACGAAGAGGATGATGAAGTGGAG
GCAACAAAAGAAAATACAACAGATTCTACTAGAAGTACTACGACTCGAAGCAAGATGCTGTTAGATTTGCTGGAA
GATGGTGGGTCTAAAAAGAAATTAACAGATGAAGAAATCCAGCTGCGAAGAGCCGAAAATGCACGTAAAAGGAAG

AACCTTAGCGAGAAGAGACTGGAAGAGGAGAAGCAGGACACAATCAATAAGCTTTTGAAGAAAAGGGCCGGTAAG
TCTAGGAGCCACTTGCCAAATGATGATGAAAAGAATGATGGCTCTTCAAGTTTCGTGAAACCACGCAGGCCTTAC
AATAGCGAGGGTATGACAAGAATTCTGCGGAGATATGAAGAAGACTTATTTTGTACATTTTAA

>YKL220C 0.68 Cold
ATGCATTGGACGTCCATCTTGAGCGCTATTTTGCTTTTCTGCCTGTCAGGAGCAAGAGCATCACCTGCAAAGACA
GTTATTCGTAACAAGGTGCCCTTGCTTGTCACTAATGCCTGCACGAGGATTTTTCAAAAAGTTACTTGGGAGTAT
ACCAGCAAGTCTAAGCGTTCATCACCAGTATGTTCTTACGAACCAGCGTTTCAATCAATGCTGTATTGTATCTAT
GAAACGTTGGATGAAAAAGGTTATTCGAATAAAACCTTAGAAAAAACATTTAGTACCATCAAAAAAAACTGTGCA
AGTTATAGTGATGCTCTTCAGAACATGACTAATTCTGAGTTTTATGACGTTCTAAACAACGGAACGAGGCATATG
ACGCCCTATGTCAAAGGCAGTGCAAACCTAACATATCCAGTTGAAATGGATACACAGTTAAGAAAAGCCTACTAT
CATGCATTGCATGGTTTCTATGCTAACTTAGATGTCGGAAACATATATGGTGGCATTATATGTGCTTATTTTGTC
GCTATCATGGCTTTTGCAGGCGTTCTTCATTGCATGAATTACACTCCCTTTAAAACTGTTCTATTGAAGCAAAAG
CTTGTGGGATATGTAAGGGGATACCTCACTCTACCTACCATTGGAAGCAAACATGCGTCAGATTTCTCTTATTTT
AGAATATTCACAGGATATTTACCTACAAGATTAGAGGGTATCATTATTCTTGGATATCTCGTGCTTCATACCGTT
TTTTTGGCATACGGTTATGAATACGATCCTGAAAATATAATATTCAAGTCTCGTAGAGTTCAAGTTGCTCGATAT
GTGGCAGACAGAAGTGGTGTACTCGCATTCGCACACTTTCCATTAATAGTTCTTTTCGCGGGAAGAAACAACTTT
CTCGAGTATATTTCTGGGGTTAAATATACTTCCTTCATTATGTTTCACAAATGGTTGGGAAGAATGATGTTTTTG
GATGCTATGATTCATGGCTCTGCATATACTAGTTATACGGTAGCGAATAAAACTTGGGCAACAAGTAAAAACCGA
TTATATTGGCAATTCGGGGTGGCAGCACTTTGTTTAGCTGGCACAATGGTTTTCTTTTCCTTTGCAGTATTCAGG
AAGTATTTTTATGAAGCCTTTCTTTTTCTTCATATCGTCCTTGGTGCAATGTTCTTTTATGCATGTTGGGAGCAT
GTTGTTAGTTTAAGTGGCATTGAGTGGATATACACTGCTATTGCGATTTGGATCGTTGACCGGATTATTAGAATT
ATCAAAGCTTCTTATTTTGGTTTCCCCAAAGCTTCCCTACAACTAATCGGGGATGATCTCATTCGTTTAACAGTT
AAAAAACCGGCAAGGCCATGGAGGGCCAAACCTGGGCAATATGTTTTCGTTTCGTTTTTACATCCACTGTACTTC
TGGCAGTCACATCCATTTACTGTTTTGGATTCAGTAAGCAAGAATGGTGAACTGGTTATTATCCTGAAAGAAAAA
AAGGGAGTAACAAGACTTGTCAAAAAGTATGTGTGTCGCAATGGAGGTAAGACATCTATGAGACTAGCTATAGAA
GGTCCATATGGTTCTTCATCTCCGGTCAATAATTACAATAATGTATTGTTACTCACTGGAGGTACCGGTTTGCCT
GGACCTATTGCACATGCAATTAAACTTGGAAAGACGTCAGCGGCTGCTGGAAAACAATCTGTAAAATTAGTGATT
GCAGTTAGAGGATTCGACGTACTCGAGGCTTATAAGCCGGAGTTGATGTGTTTAGAAAATCTGAATGTACAGCTT
CACATCTACAACACAATGGAAGTCCCATCATTAACCCCTAGTGATAGTTTAGATATTTCTCAACAGGATGAGAAG
GCTGATGAAAAAGGCACTGTTGTGGCAACTACTTTAGAAAAGTCTGCCAATCCACTTGGTTTTGATGGTGTTGTT
TTCCATTGCGGGCGACCAAATGTTAAGGAACTTCTACATGAAGCGGCTGAATTGAGTGGCTCATTATCTGTGGTT
TGCTGTGGACCTCCTATTTTTGTTGACAAAGTGAGGAATGAAACCGCAAAAATAGTTTTAGATAAGTCTGCAAAA
GCCATTGAGTACTTTGAAGAGTATCAATGCTGGTGA

>YGL240W 0.79 Cold
ATGGACCCGATTGGAATAAACAAAGTCTTGGACCATTTAGCACCCTCAGAGCTTATCAAACCTGTTAAGAGTTGC
CATAATAAACCTTCAGTGTTGGTGTTGGATGACAGGATTGTGGATGCGGCAACCAAAGATCTCTACGTGAATGGG
TTCCAAGAAGAGATTCAGTATCAGAATCCTACACCGGAGAACTTGCAACATATGTTCCACCAAGGTATTGAAATA
TTGGATTCGGCAAGGATGATCAACGTGACACATTTAGCGCTTTGGAAGCCTTCTTCTTTTAAGTTGGGGAATCCT
GTGGACTTTGCTCTAGATGATAACTACGATACGTTTTGGCAGAGCGATGGAGGGCAACCACACCAATTGGACATT
ATGTTTAGTAAGAGAATGGACATCTGTGTCATGGCAATATTCTTTTCGATGATTGCGGACGAGTCCTATGCTCCA
AGCTTAGTGAAAGTTTACGCGGGGCATAGTCCTTCTGATGCGAGGTTTTATAAGATGCTTGAAGTGAGAAATGTG
AATGGCTGGGTGGCACTAAGGTTTCTAGATAATAGGGAGGACGACCAGCTGCTGAAATGTCAATTCATCAGGCTT
TTGTTTCCAGTCAACCATGAAAACGGAAAAGATACACATCTGAGAGGCATAAGGCTATATGTTCCATCTAATGAG
CCACATCAAGATACCCATGAGTGGGCACAGACCCTCCCAGAAACTAACAATGTCTTCCAGGATGCTATATTACGT
TAA

>YCL052C 0.79 Cold
ATGGTGACAAGACATAGAGTGACTGTACTCTACAATGCCCCTGAGGATATCGGTAATCATATGCGCCAAAATGAC
ACTCATTTGACTGTTCGTGGAGGTTCTGGTGTGGTTTTACAACAAAGGTGGCTATTAGAGAGGACTGGAAGCTTG
GATAAATCCTTTACGAGAATCACTTGGAGGCCCAGAGCGGACTTGGCTAGAAGTTTAAGCGTTATAGAAAATGAA
CTGAGTGCTGGCTTTTCAGTTTACTCAAATTCTTCGGATGTGCCGGAAAGGTTTATTACTAACCCAGTCTACAAT
TCATTTCACAGTGAGAAGTTTGACATAGAGCAGTACTTGCCTCCCGAAGTAGATTTGAATCTGTCATGGAATCCA
GAAGATTTTACATATGATATATCAGTGGAGCCCACACAAATCCAAATTGTTGAATATCGTCTGTTGAAACAGGGT
GAAGAATTTACAATTGCAAGAGTGAAAGATGAGAAACTCGAAGTAGGTGTATTCTTTGTGGATGCAAGTGATGAA
AGTGATGTCGATATTGGTGGAATACGTTGTAATTGGAGGATGGACGATGGTAAAATGGAAAGATGTCAGAAAACA
TCCTTATTGTATAAACAGGGCCATATCGCATACAATCACTCGACGACTACGACATCACTATATCTGAATGAACCT
ATCGGTTTGCATCCAAAAATCATGATTGATCTCACAGATTTCGAAGAACGCCCTAAATGCATGTATCTAATGCAC
CTGCAATTGCCGTTAGAATTATTTATCGATAAATTCCAATCCTCTCCCTTACTACTTTTTGGAGAAGACGACTTA
GAATTACCAGAATACTCTCTTCGAGATAAGGCATGGGGTTCTGAAAGTATCTTTGAATTGAAAGCCGGCACAATG
AATGAAGTGACATTGCATACTAGATATATTGAGCCTTCTAATAATAAAGGGGATAAATTAGAAGTTTCATTTGAT
CCAGAAGTTATATTAGCCTGCGACACAGGTGACAATAAAGTTTCCCGTAATCCATTTTATAAAAAAGGTCTAGGA
TATGAATCTCTCTTTACAGACGATACTACATTCCGCCATTTGAACTCGACAACTCTTCTAGTACCAATTCCAAGG
CCTGACACAAAGGATTATTCCAAGATCAAAAATGGTACGTTACTATGCTTACTCATCTCCATCATATACATTTTC
TCCAAGGTATTTGGTAACAACAAGAAGAAAAGATCAGTAAAACGGGAATAA

>YLR128W 0.72 Cold
ATGAGTAATAATAAAATAAAAAGAAAGGATGCAAGCCCGGAACAAGAGGCAATTGAATCATTCACCTCATTAACC
AAATGTGACCCCAAGGTATCCAGGAAGTACCTGCAGCGTAATCACTGGAACATCAATTACGCTCTCAATGATTAT
TATGACAAGGAAATAGGGACGTTTACTGACGAGGTTTCAACTGTGGCTCATCCACCCGTATACCCGAAGGAGCTA
ACACAGGTATTTGAACACTATATCAATAACAACTTGTTTGATATAGACTCACTGGTCAAGTTCATTGAGGAGCTA
GGCTACAATCTTGAAGATTTAGCGACATTATGCTTAGCCCATTTACTGGGGTACAAGAAACTAGAAGAACCCTTA
AAACGAGAGGACTTCCTGTCAACATGGTTTATGCAAGGCTGTTCCACCATTTCAGACATGCAAGAGTGCATCAAG
ACGTTAGATGTTAAATTACATGAAGACTTACAATATTTCACACAGATTTATAACTATGCTTTCAATTTGATTCTG
GACCCAAATCGAAAGGATATAGATACAGACGAAGGCATCCAGTATTGGAAGCTATTTTTCCAACCAGAGTACCCA
GTGCGTATGGAACCAGATTTGCTTGAGGCATGGTTCCGTTTCCTTCGCGATGAGGGGAAAACCACTATAAGTAAA
GACACCTGGCGTATGCTGCTCCTCTTTTTCAAACGATACCCCACTATTCAGAAAATAATAAGCGATTACGACGAA
ACTGCAGCCTGGCCATTTATTATCGATGAGTTCTATGAGTGCTTACAGGATCAGCAATAA

>YJL214W 0.76 Cold
ATGACTGATCGTAAAACCAACTTGCCAGAAGAACCGATTTTCGAAGAGGCAGAAGATGATGGCTGCCCTTCGATA
GAAAATTCTTCACATCTGTCAGTACCTACAGTGGAGGAAAACAAGGACTTTTCCGAGTATAATGGGAAGAGGCA
GAGGAAGTTGTTGTTCCAGAAAAGCCTGCTTCAGCCTATGCTACTGTTTCTATCATGTGTTTATGTATGGCTTTC
GGTGGATTTATGTCCGGTTGGGACACAGGTACGATTTCTGGTTTCGTCAATCAGACTGATTTTTTAAGAAGATTT
GGTAATTATAGCCATTCCAAGAACACTTACTACTTATCTAATGTGAGAACTGGGTTGATTGTGTCCATCTTCAAT
GTGGGAAGCGCCATTGGCTGTCTTTTCTTGTCTAAATTGGGTGATATTTACGGCCGCTGCATGGGTTTGATTATA
GTTATTGTCGTTTATATGGTTGGTATTGTCATTCAAATTGCCTCTATAGATAAGTGGTATCAGTATTTTATTGGA
AGAATTATCGCTGGTATAGGTGCTGGTTCCATTAGTGTTCTTGCCCCGATGCTTATTTCGGAAACTGCGCCAAAG
CATATCAGAGGTACGTTGCTAGCTTGTTGGCAATTGATGGTGACTTTCGCAATTTTCTTGGGTTATTGTACCAAT
TATGGTACCAAGACTTACTCGAATTCTGTCCAGTGGCGTGTTCCGCTTGGTCTATGTTTTGCATGGGCTATTATT
ATGATTGGTGGTATGACGTTTGTTCCGGAATCTCCTCGGTTTTTGGTGCAAGTCGGTAAGATTGAGCAAGCTAAA
GCTTCTTTTGCCAAGTCGAACAAGCTTAGTGTTGACGATCCTGCTGTGGTTGCAGAGATTGATCTTCTTGTTGCT
GGTGTGGAGGCAGAAGAAGCAATGGGAACTGCTTCATGGAAGGAATTATTTTCGAGAAAGACTAAAGTTTTTCAA
CGTTTAACGATGACAGTCATGATTAACTCTCTGCAGCAACTAACCGGTGACAACTATTTCTTCTACTACGGTACT
ACTATCTTCAAATCTGTCGGTATGAATGACTCTTTTGAGACTTCAATTGTCTTGGGTATTGTGAATTTTGCTTCT
TGCTTCTTTTCACTTTATTCTGTTGATAAGTTGGGCCGTCGTAGATGTCTTTTACTTGGAGCAGCCACCATGACG
GCGTGCATGGTTATTTACGCCTCCGTTGGCGTCACAAGACTATATCCGAACGGTAAAAGTGAACCATCATCTAAA
GGTGCTGGTAATTGTACGATTGTTTTCACGTGTTTTTACATTTTCTGCTTTTCCTGCACCTGGGGACCTGTATGT
TATGTGATTATTTCTGAAACATTTCCATTAAGGGTGAGATCCAAGTGTATGTCCGTTGCAACAGCGGCCAACTTA
TTGTGGGGGTTCCTAATCGGGTTTTTCACTCCTTTTATTACTTCGGCAATTAATTTCTACTACGGTTACGTTTTC
ATGGGTTGCTTAGCGTTTTCATATTTTTACGTCTTTTTCTTTGTTCCAGAAACAAAAGGTCTAACTTTAGAAGAA
GTTGATGAGATGTGGATGGACGGTGTATTACCTTGGAAATCTGAATCCTGGGTACCAGCTTCTAGAAGGGATGGT
GATTATGATAACGAAAAATTACAGCATGACGAGAAACCCTTCTACAAAAGAATGTTTTAG

>YLR012C 0.78 Cold
ATGAAGACTGTCTATTACAAAGAAATCACCTACCAGCAGTATCTTCAACTGCAACCTGAACAACAGGAAAAATAC
CTGGCTTTATGTCAAAAAGATTTTGAACAAGAGACTGAAAGAATTGCATTTGATCGTCAAGGAGGTGTGCCTGGT
ATTGCTAGGAAATTTGCACAAGAAGAAGTTGCTTGGTTCGATCGCGTTACAACATGGTCATATATGAATGCTTAT
ATTCCGAGCTACAGAAGAAGAAGAAACCTTTTAAAGATTGATATGTTAAAGATGTCTAATGCTGAAGAATACTGA

>YOR377W 0.8Cold
ATGAATGAAATCGATGAGAAAAATCAGGCCCCCGTGCAACAAGAATGCCTGAAAGAGATGATTCAGAATGGGCAT
GCTCGGCGTATGGGATCTGTTGAAGATCTGTATGTTGCTCTCAACAGACAAAACTTATATCGAAACTTCTGCACA
TATGGAGAATTGAGTGATTACTGTACTAGGGATCAGCTCACATTAGCTTTGAGGGAAATCTGCCTGAAAAATCCA
ACTCTTTTACATATTGTTCTACCAACAAGATGGCCAAATCATGAAAATTATTATCGCAGTTCCGAATACTATTCA
CGGCCACATCCAGTGCATGATTATATTTCAGTATTACAAGAATTGAAACTGAGTGGTGTGGTTCTCAATGAACAA
CCTGAGTACAGTGCAGTAATGAAGCAAATATTAGAAGAATTCAAAAATAGTAAGGGTTCCTATACTGCAAAAATT
TTTAAACTTACTACCACTTTGACTATTCCTTACTTTGGACCAACAGGACCGAGTTGGCGGCTAATTTGTCTTCCA
GAAGAGCACACAGAAAAGTGGAAAAAATTTATCTTTGTATCTAATCATTGCATGTCTGATGGTCGGTCTTCGATC
CACTTTTTTCATGATTTAAGAGACGAATTAAATAATATTAAAACTCCACCAAAAAAATTAGATTACATTTTCAAG
TACGAGGAGGATTACCAATTATTGAGGAAACTTCCAGAACCGATCGAAAAGGTGATAGACTTTAGACCACCGTAC
TTGTTTATTCCGAAGTCACTTCTTTCGGGTTTCATCTACAATCATTTGAGATTTTCTTCAAAAGGTGTCTGTATG
AGAATGGATGATGTGGAAAAAACCGATGATGTTGTCACCGAGATCATCAATATTTCACCAACAGAATTTCAAGCG
ATTAAAGCAAATATTAAATCAAATATCCAAGGTAAGTGTACTATCACTCCGTTTTTACATGTTTGTTGGTTTGTA
TCTCTTCATAAATGGGGTAAATTTTTCAAACCATTGAACTTCGAATGGCTTACGGATATTTTTATCCCCGCAGAT
TGCCGCTCACAACTACCAGATGATGATGAAATGAGACAGATGTACAGATATGGCGCTAACGTTGGATTTATTGAC
TTCACCCCCTGGATAAGCGAATTTGACATGAATGATAACAAAGAAAATTTTTGGCCACTTATTGAGCACTACCAT
GAAGTAATTTCGGAAGCTTTAAGAAATAAAAAGCATCTCCATGGCTTAGGGTTCAATATACAAGGCTTCGTTCAA
AAATATGTGAACATTGACAAGGTAATGTGCGATCGTGCCATCGGGAAAAGACGCGGAGGTACATTGTTAAGCAAT
GTAGGTCTGTTTAATCAGTTAGAGGAGCCCGATGCCAAATATTCTATATGCGATTTGGCATTTGGCCAATTTCAA
GGATCCTGGCACCAAGCATTTTCCTTGGGTGTTTGTTCGACTAATGTAAAGGGGATGAATATTGTTGTTGCTTCA
ACAAAGAATGTTGTTGGTAGTCAAGAATCTCTCGAAGAGCTTTGCTCCATTTACAAAGCTCTCCTTTTAGGCCCT
TAG

>YNL027W 0.81   Cold
ATGTCATTCAGCAACGGAAATATGGCTTCCTACATGACTAGTAGTAATGGAGAGGAGCAAAGTATAAACAACAAA
AACGATATAGACGATAACAGCGCTTACAGACGTAATAATTTCAGGAATAGTAGCAATTCAGGATCACATACGTTC
CAATTATCGGACTTGGACTTAGATGTGGATATGAGGATGGATTCCGCCAATTCATCGGAGAAAATATCAAAGAAC
CTTTCCTCTGGCATCCCGGACTCATTTGATTCTAACGTGAACAGCTTGCTGTCTCCGTCAAGCGGTTCTTATTCT
GCAGATTTGAATTACCAAAGTCTATACAAACCAGATCTTCCACAGCAACAGTTACAACAGCAACAGTTACAACAG
CAACAGCAACAGCAACAGCAACAACAACAGCAACAGCAGAAGCAAACACCAACTTTAAAAGTCGAACAATCTGAT
ACATTTCAGTGGGACGATATCTTAACACCTGCTGATAATCAACATCGGCCATCCCTCACAAACCAGTTTTTATCT
CCAAGATCTAATTACGATGGTACCACTAGGAGCTCGGGCATTGACTCCAATTATAGCGATACAGAATCAAACTAT
CATACGCCTTATTTGTATCCACAGGACTTAGTTTCTTCACCTGCGATGTCTCACTTAACCGCGAATAACGATGAT
TTTGACGATCTTTTGAGCGTCGCATCTATGAACTCAAATTATTTACTGCCCGTAAATTCACATGGTTATAAACAT
ATTTCAAACCTTGATGAGTTGGATGACTTGCTATCTTTAACATATTCAGACAATAACCTTTTATCAGCATCAAAC
AACAGTGATTTCAACAACAGTAATAACGGAATTATTAATACCGCTGACACTCAAAACAGTACCATTGCCATTAAT
AAAAGTAAAGTCGGTACTAACCAAAAGATGTTATTGACTATTCCAACTTCTTCCACACCTTCTCCTTCCACTCAT
GCTGCCCCGGTAACACCCATCATTTCTATACAGGAATTCAATGAGGGACACTTCCCAGTTAAAAACGAAGATGAC
GGAACGTTACAACTAAAAGTTCGGGATAACGAAAGTTATAGCGCTACTAACAATAACAATCTACTTCGTCCAGAT
GATAATGATTACAACAACGAAGCTCTCAGTGATATTGACCGCTCCTTCGAAGATATCATTAACGGTCGAAAATTG
AAACTTAAAAAATCAAGAAGAAGATCTTCTCAAACTTCCAATAATAGTTTCACTAGCAGGAGATCCTCGAGATCA
AGAAGCATATCTCCCGATGAAAAAGCTAAATCTATAAGTGCAAATAGGGAAAAGTTATTGGAGATGGCAGATCTT
TTACCATCCAGTGAAAATGACAATAATCGGGAGCGCTATGACAATGATAGCAAAACTAGCTATAACACCATTAAC
AGTAGCAATTTTAATGAGGATAATAATAATAACAATTTGTTAACAAGTAAACCAAAAATTGAATCGGGCATTGTC
AATATTAAAAATGAACTAGATGACACTAGCAAAGATCTTGGCATACTTCTAGACATAGATAGCTTGGGCCAATTT
GAGCAGAAAGTTGGTTTCAAAAATGATGATAATCACGAAAATAACGATAATGGCACATTTTCTGTTAAGAAAAAT
GACAATCTAGAAAAACTAGACAGTGTAACAAATAATAGGAAAAATCCTGCGAATTTTGCTTGTGATGTATGTGGT
AAGAAGTTTACAAGACCTTATAACTTAAAGTCACACTTAAGAACGCATACAAATGAAAGGCCATTTATTTGTTCT
ATTTGTGGTAAGGCGTTTGCACGTCAACATGATAGAAAGAGACACGAAGATTTGCATACAGGTAAGAAAAGGTAT
GTATGCGGTGGTAAGCTGAAGGACGGTAAACCCTGGGGTTGTGGCAAAAAGTTTGCAAGAAGTGATGCTCTTGGT
AGGCATTTTAAAACTGAAAGTGGTAGAAGATGCATCACTCCCTTGTACGAAGAAGCCAGACAGGAGAAATCGGGA
CAAGAGAGTTAA

>YDR294C 0.8Cold
ATGAGTGGAGTATCAAATAAAACAGTATCAATTAATGGTTGGTATGGCATGCCAATTCATTTACTAAGGGAAGAA
GGCGACTTTGCCCAGTTTATGATTCTAACCATCAACGAATTAAAAATAGCCATACATGGTTACCTCAGAAATACC
CCATGGTACAACATGTTGAAGGATTATTTGTTTGTGATCTTTTGTTACAAGCTAATAAGTAATTTTTTTTATCTG
TTGAAAGTTTATGGGCCGGTGAGGTTAGCAGTGAGAACATACGAGCATAGTTCCAGAAGATTGTTTCGTTGGTTA
TTGGACTCACCATTTTTGAGGGGTACCGTAGAAAAGGAAGTCACAAAGGTCAAACAATCGATCGAAGACGAACTA
ATTAGATCGGACTCTCAGTTAATGAATTTCCCACAGTTGCCATCCAATGGGATACCTCAGGATGATGTTATTGAA
GAGCTAAATAAATTGAACGACTTGATACCACATACCCAATGGAAGGAAGGAAAGGTCTCTGGTGCCGTTTACCAC
GGTGGTGATGATTTGATCCACTTACAAACAATCGCATACGAAAAATATTGCGTTGCCAATCAATTACATCCCGAT
GTCTTTCCTGCCGTACGTAAAATGGAATCCGAAGTGGTTTCTATGGTTTTAAGAATGTTTAATGCCCCTTCTGAT
ACAGGTTGTGGTACCACAACTTCAGGTGGTACAGAATCCTTGCTTTTAGCATGTCTGAGCGCTAAAATGTATGCC
CTTCATCATCGTGGAATCACCGAACCAGAAATAATTGCTCCCGTAACTGCACATGCTGGGTTTGACAAAGCTGCT
TATTACTTTGGCATGAAGCTACGCCACGTGGAGCTAGATCCAACGACATATCAAGTGGACCTGGGAAAAGTGAAA
AAATTCATCAATAAGAACACAATTTTACTGGTCGGTTCCGCTCCAAACTTTCCTCATGGTATTGCCGATGATATT
GAAGGATTGGGTAAAATAGCACAAAAATATAAACTTCCTTTACACGTCGACAGTTGTCTAGGTTCCTTTATTGTT
TCATTTATGGAAAAGGCTGGTTACAAAAATCTGCCATTACTTGACTTTAGAGTCCCGGGAGTCACCTCAATATCA
TGTGACACTCATAAATATGGATTTGCACCAAAAGGCTCGTCAGTTATAATGTATAGAAACAGCGACTTACGAATG
CATCAGTATTACGTAAATCCTGCTTGGACTGGCGGGTTATATGGCTCTCCTACATTAGCAGGGTCCAGGCCTGGT
GCTATTGTCGTAGGTTGTTGGGCCACTATGGTCAACATGGGTGAAAATGGGTACATTGAGTCGTGCCAAGAAATA
GTCGGTGCAGCAATGAAGTTTAAAAAATACATCCAGGAAAACATTCCAGACCTGAATATAATGGGCAACCCTAGA
TATTCAGTCATTTCATTTTCTTCAAAGACCTTGAACATACACGAACTATCTGACAGGTTGTCCAAGAAAGGCTGG
CATTTCAATGCCCTACAAAAGCCGGTTGCACTACACATGGCCTTCACGAGATTGAGCGCTCATGTTGTGGATGAG
ATCTGCGACATTTTACGTACTACCGTGCAAGAGTTGAAGAGCGAATCAAATTCTAAACCATCCCCAGACGGAACT
AGCGCTCTATATGGTGTCGCCGGGAGCGTTAAAACTGCTGGCGTTGCAGACAAATTGATTGTGGGATTCCTAGAC
GCATTATACAAGTTGGGTCCAGGAGAGGATACCGCCACCAAGTAG

>YEL069C 0.69   Cold
ATGTCTAGTGCGCAATCCTCTATTGATAGCGATGGAGATGTTCGAGATGCTGATATTCATGTCGCACCACCCGTG
GAAAAAGAGTGGTCAGATGGATTTGATGACAACGAAGTCATAAACGGGGATAACGTTGAGCCACCAAAAAGAGGG
CTCATAGGTTATCTTGTCATTTACTTACTGTGTTATCCAATATCCTTTGGGGGTTTCCTGCCTGGTTGGGATAGT
GGTATCACAGCAGGTTTCATTAACATGGACAACTTTAAAATGAACTTCGGTTCTTACAAGCATAGCACTGGTGAA
TATTATTTGAGCAACGTGCGTATGGGTCTTCTTGTGGCTATGTTCAGTATTGGATGTGCCATAGGTGGCCTTATT
TTTGCCCGTCTTGCTGATACTTTAGGTAGAAGGCTGGCAATTGTGATCGTGGTGTTGGTATATATGGTTGGTGCA
ATTATTCAGATCAGTTCAAATCACAAATGGTACCAGTACTTTGTCGGTAAGATCATCTACGGTCTTGGTGCTGGT
GGCTGTTCGGTGTTGTGTCCAATGCTTTTGTCTGAAATAGCCCCCACAGATTTGAGAGGTGGACTGGTCTCATTG
TACCAACTGAACATGACGTTCGGTATTTTCTTGGGTTATTGTAGCGTTTATGGTACGAGAAAATACGATAACACT

GCACAATGGAGAGTCCCCCTTGGGCTTTGCTTTTTATGGGCTTTGATTATCATCATTGGTATGTTATTGGTTCCA
GAGTCCCCAAGATATCTGATTGAATGTGAGAGACACGAAGAGGCCCGTGCTTCCATTGCCAAAATCAACAAGGTT
TCACCAGAGGATCCATGGGTACTCAAACAGGCTGATGAAATCAACGCCGGTGTCCTTGCCCAAAGGGAACTAGGA
GAAGCTTCATGGAAAGAACTTTTCTCTGTAAAAACTAAAGTCCTTCAACGTTTGATCACAGGTATTCTTGTGCAA
ACCTTTTTGCAACTTACTGGTGAAAACTACTTCTTCTTCTACGGAACTACCATTTTTAAATCAGTCGGTCTTACT
GATGGGTTTGAGACGTCGATCGTCCTAGGTACAGTGAACTTCTTCTCCACTATTATTGCTGTTATGGTCGTAGAC
AAAATTGGCCGTCGTAAATGTCTGTTATTTGGTGCAGCTGGGATGATGGCTTGTATGGTCATATTTGCAAGTATC
GGGGTGAAATGTCTTTACCCTCATGGCCAGGACGGTCCTTCTTCGAAAGGTGCAGGTAATGCCATGATTGTGTTC
ACTTGTTTCTATATATTCTGCTTTGCAACGACATGGGCTCCTGTTGCTTATATTGTGGTTGCCGAGTCGTTCCCT
TCGAAGGTCAAGTCTAGAGCCATGTCGATTTCAACTGCATGCAACTGGTTATGGCAATTTTTGATCGGTTTTTTC
ACACCATTCATTACTGGGTCTATCCACTTCTATTATGGTTATGTGTTCGTAGGTTGTTTGGTTGCTATGTTTTTG
TACGTTTTCTTCTTTTTACCAGAAACGATTGGTCTATCTTTGGAGGAAATCCAATTACTATACGAAGAAGGTATA
AAACCATGGAAATCTGCATCTTGGGTCCCACCTTCTAGGAGAGGTATTTCTTCCGAAGAAAGTAAGACCGAGAAG
AAGGATTGGAAGAAATTTTTGAAGTTCTCAAAGAATTCTGATTGA

>YBR171W 0.82 Cold
ATGTCCGAATTTAATGAAACAAAATTCTCCAACAACGGGACGTTTTTGAAACGGAAGAGCCAATTGTGGAGACG
AAATCAATCTCCGTTTATACCCCACTCATATATGTCTTTATTCTGGTGGTGTCCCTTGTGATGTTTGCTTCAAGC
TACAGAAAGAAGCAGGCCAAAAAAATTAGTGAGCAACCATCCATATTTGACGAAAACGATGCCCATGATCTGTAT
TTCCAAATAAAGGAAATGAGTGAAAATGAAAAAATTCACGAGAAGGTGTTGAAGGCCGCTTTATTGAACAGAGGA
GCAGAATCTGTTAGACGATCATTAAAGTTAAAAGAGTTGGCTCCTCAGATAAACCTTCTATATAAAAATGGCTCT
ATTGGGGAGGATTACTGGAAGAGATTTGAAACTGAAGTTAAATTAATTGAATTGGAATTTAAAGATACTTTACAA
GAAGCTGAAAGATTGCAACCGGGCTGGGTTCAATTGTTCGTTATGGTTTGTAAAGAAATTTGCTTTAATCAAGCT
CTCTCTAGACGTTATCAATCAATCTTGAAACGGAAAGAAGTGTGTATTAAAGAGTGGGAGCTGAAAATAAATAAT
GATGGAAGATTAGTCAATTAG

>YBR170C 0.82 Cold
ATGCTTATCAGATTTAGATCAAAAAACGGTACACACAGGGTTTCTTGTCAAGAAAACGACCTTTTCGGAACGGTC
ATTGAAAAGTTGGTGGGTAATCTGGACCCCAATGCCGATGTCGACACATTTACTGTTTGTGAGAAGCCCGGTCAA
GGTATTCATGCTGTTTCTGAACTAGCTGATCGAACAGTGATGGATCTAGGACTAAAGCACGGTGACATGCTGATC
CTTAACTATTCGGACAAGCCCGCTAATGAGAAAGATGGTGTCAATGTTGAAATCGGATCCGTAGGTATTGACAGC
AAGGGAATTCGTCAACACAGGTACGGTCCACTAAGGATCAAAGAACTCGCTGTAGATGAGGAACTAGAGAAAGAG
GACGGATTAATTCCTCGTCAAAAATCAAAACTATGCAAGCACGGTGATAGAGGTATGTGTGAATACTGCTCGCCT
TTACCTCCTTGGGACAAAGAATATCATGAGAAGAATAAGATCAAACATATATCGTTTCATTCATATCTTAAAAAG
CTAAATGAAAATGCTAATAAGAAGGAGAACGGCAGCTCTTATATTTCCCCTCTTTCAGAACCTGATTTTAGAATC
AATAAGCGTTGCCATAATGGCCATGAACCATGGCCTCGGGGGATATGTTCTAAATGTCAACCATCGGCAATTACA
TTACAACAGCAAGAATTTAGAATGGTTGACCACGTTGAATTTCAGAAGAGTGAAATAATTAATGAATTTATTCAG
GCGTGGAGGTACACGGGTATGCAAAGATTTGGCTATATGTACGGATCTTATTCTAAGTATGATAACACACCTTTA
GGTATAAAGGCCGTCGTTGAGGCGATATACGAGCCCCCTCAGCATGATGAGCAAGACGGTTTAACCATGGACGTA
GAACAGGTCAAGAATGAAATGCTACAGATTGATAGACAGGCTCAAGAAATGGGGCTTTCGCGAATTGGCCTAATA
TTTACAGATTTATCTGACGCGGGAGCTGGGGATGGGTCTGTTTTTTGCAAAAGACATAAGGATTCGTTTTTTCTT
TCATCATTAGAAGTTATTATGGCTGCTAGGCATCAAACAAGGCATCCTAATGTAAGCAAGTATAGCGAACAAGGT
TTTTTTTCTTCCAAGTTTGTAACTTGCGTTATATCAGGTAATTTGGAAGGTGAAATTGATATTTCGAGCTACCAA
GTATCCACAGAAGCTGAAGCATTGGTTACTGCAGATATGATAAGTGGGTCCACATTTCCTTCAATGGCATATATT
AATGACACTACAGATGAAAGATATGTACCTGAGATATTTTACATGAAGTCGAATGAATATGGTATAACAGTGAAG
GAAAATGCGAAGCCTGCATTTCCGGTAGACTATCTTTTAGTGACGCTGACTCATGGGTTCCCGAATACCGATACG
GAAACCAACTCGAAATTCGTTAGTTCCACCGGATTTCCATGGAGCAATCGACAAGCTATGGGGCAATCTCAAGAT
TATCAAGAATTAAAAAAGTATTTATTCAATGTGGCTTCAAGTGGAGATTTCAATCTTTTGCATGAAAAAAATCTCG
AACTTTCATTTACTATTATACATAAATTCTCTGCAGATACTCTCTCCAGACGAATGGAAGTTACTAATAGAATCT
GCTGTGAAAAAATGAATGGGAAGAATCTCTACTAAAACTTGTCTCATCGGCTGGTTGGCAAACGTTAGTCATGATC
CTTCAGGAAAGCGGCTAG

>YDR021W 0.82 Cold
ATGTCGTTTGACAGAGAAGAAGACCAAAAATTGAAGTTTAAAACATCGAAAAAATTAAAGGTGTCTTCTACTTTT
GAAAGCATGAATCTAAAAGATGACTTACTTCGAGGAATATACTCATACGGTTTTGAAGCACCATCCTCTATCCAA
TCAAGGGCTATTACACAGATTATTTCGGGCAAGGACGTTATAGCACAAGCACAATCAGGTACAGGTAAGACAGCA
ACTTTTACAATCGGCCTTTTACAAGCTATTGATTTAAGGAAGAAAGATTTACAAGCTCTAATACTATCTCCAACA
CGTGAATTAGCAAGCCAAATTGGGCAAGTAGTAAAAAATCTAGGGGATTATATGAATGTCAATGCATTTGCCATT
ACAGGTGGTAAAACTTTGAAAGACGACCTGAAGAAAATGCAAAAGCATGGTTGCCAGGCCGTTAGCGGAACACCA
GGAAGAGTATTAGACATGATTAAGAAACAAATGTTGCAAACAAGAAATGTTCAAATGTTAGTTTTAGATGAGGCT
GATGAATTATTGAGTGAGACACTAGGTTTCAAGCAACAAATATACGATATTTTTGCAAAATTGCCTAAAAATTGC
CAAGTTGTTGTTGTCAGCGCAACAATGAATAAGGACATTTTAGAAGTAACCAGAAAATTTATGAATGATCCAGTT
AAAATCCTAGTGAAGAGGGATGAAATATCACTTGAAGGTATCAAACAATATGTCGTTAATGTTGACAAAGAAGAG
TGGAAATTCGATACTCTGTGTGATATATACGATTCTTTGACCATTACACAATGTGTGATATTCTGTAACACCAAG
AAAAAAGTAGATTGGTTATCTCAGCGGCTAATCCAATCAAACTTTGCTGTAGTCTCCATGCATGGTGATATGAAA
CAAGAAGAAAGAGATAAAGTAATGAATGATTTTAGAACAGGTCATTCTCGTGTACTGATATCGACGGATGTTTGG
GCGCGCGGTATTGATGTCCAACAAGTTTCTTTAGTCATTAACTATGACCTACCAGAAATAATAGAAAATTACATC

CATCGTATCGGTAGAAGTGGTCGATTTGGTAGAAAGGGTGTGGCTATAAACTTCATTACCAAAGCTGATTTAGCA
AAACTTAGAGAAATTGAAAAATTCTATTCTATCAAAATAAATCCGATGCCAGCAAATTTTGCAGAATTATCATAA

>YNL332W 0.78   Cold
ATGTCTACAGACAAGATCACATTTTTGTTGAACTGGCAACCAACCCCATACCATATTCCAATTTTCTTGGCTCAA
ACCAAAGGTTACTTCAAGGAGCAAGGTCTAGACATGGCCATCCTAGAACCAACCAATCCTTCCGATGTCACTGAG
TTAATTGGATCTGGTAAGGTCGACATGGGTTTGAAAGCCATGATTCACACCTTGGCTGCCAAGGCCCGTGGTTTC
CCAGTGACCTCTGTTGCCTCTTTGTTGGACGAACCATTTACCGGTGTCTTGTACTTAAAGGGCAGTGGTATCACT
GAAGACTTCCAGTCCCTAAAGGGTAAGAAGATCGGTTACGTTGGTGAATTCGGTAAGATCCAAATCGATGAATTG
ACCAAGCACTACGGTATGAAGCCAGAAGACTACACCGCAGTCAGATGTGGTATGAATGTCGCCAAGTACATCATC
GAAGGTAAGATTGATGCTGGTATTGGTATCGAATGTATGCAACAAGTCGAATTGGAAGAGTACTTGGCTAAGCAA
GGCAGACCAGCTTCTGATGCTAAGATGTTGAGAATTGACAAGTTGGCTTGCTTGGGTTGCTGTTGCTTCTGTACC
GTTCTTTACATCTGCAACGATGAATTTTTGAAGAAGAACCCTGAAAAGGTCAGAAAGTTCTTGAAAGCCATCAAG
AAGGCAACCGACTACGTTCTAGCCGACCCTGTGAAGGCTTGGAAAGAATACATCGACTTCAAGCCTCGATTGAAC
AACGATCTATCTTACAAGCAATACCAAAGATGTTACGCTTACTTCTCTTCATCTTTGTACAATGTTCACCGTGAC
TGGAAGAAGGTTACCGGTTACGGTAAGAGATTAGCCATCTTGCCACCAGACTATGTCTCGAACTACACTAATGAA
TACTTGTCCTGGCCAGAACCAGAAGAGGTTTCTGATCCTTTGGAAGCTCAAAGATTGATGGCTATTCATCAAGAA
AAATGCAGACAGGAAGGTACTTTCAAGAGATTGGCTCTTCCAGCTTAA

>YNR011C 0.79   Cold
ATGTCAAGTATTACATCTGAAACCGGTAAGCGAAGAGTGAAACGAACGTATGAAGTTACAAGACAGAATGACAAT
GCAGTAAGAATAGAGCCTTCTTCCTTGGGTGAGGAAGAAGACAAAGAGGCCAAGGATAAGAACTCTGCCTTGCAG
CTAAAACGATCACGCTATGACCCCAATAAAGTTTTTCTAATACGAACCAAGGTCCAGAAAAGAACAATTTAAAA
GGAGAACAACTTGGATCACAAAAAAAGTCATCAAAGTATGATGAAAAAATAACGTCAAATAATGAACTTACTACA
AAGAAGGGTCTACTAGGTGACTCCGAGAATGAAACTAAGTACGCCTCTTCTAATTCTAAATTTAACGTAGAAGTT
ACTCATAAAATAAAGAATGCAAAAGAAATTGACAAAATAAACAGACAACGCATGTGGGAAGAACAACAATTGCGA
AATGCTATGGCGGGGCAAAGCGATCACCCTGATGATATAACATTGGAAGGCTCTGACAAGTACGATTATGTGTTC
GACACAGATGCAATGATTGATTATACTAATGAGGAGGACGATTTACTACCTGAAGAGAAACTTCAATATGAGGCA
CGTCTAGCACAAGCTTTAGAAACTGAGGAAAAGCGTATACTGACTATACAAGAGGCAAGAAAACTTTTGCCTGTA
CATCAATACAAGGATGAGCTACTACAAGAGATCAAAAAAAATCAAGTTTTGATTATAATGGGTGAAACCGGTTCT
GGTAAAACCACACAGTTACCCCAATATTTGGTAGAAGATGGATTCACTGATCAGGGAAAGTTACAGATTGCCATA
ACACAACCACGTCGTGTGGCGGCCACTTCTGTTGCTGCAAGAGTAGCAGATGAAATGAATGTTGTGCTTGGGAAG
GAAGTGGGTTATCAGATCCGTTTTGAAGACAAAACAACCCCAAATAAAACGGTTCTAAAGTATATGACGGATGGT
ATGTTACTAAGAGAATTTCTTACAGATTCCAAATTAAGCAAATATTCTTGTATTATGATTGATGAAGCTCATGAA
CGTACTCTAGCCACAGATATCCTGATAGGTTTGCTGAAAGATATTTTGCCGCAGAGGCCCACTTTAAAATTGCTT
ATATCATCAGCAACAATGAACGCAAAAAAATTTTCAGAGTTTTTTGATAATTGTCCCATTTTCAACGTTCCTGGG
AGAAGATATCCTGTAGATATCCACTACACATTACAGCCGGAGGCAAACTATATACATGCCGCTATAACAACTATT
TTTCAAATTCATACTACGCAAAGTTTACCGGGGGATATATTGGTGTTTTTAACGGGGCAAGAAGAAATTGAAAGA
ACAAAAACCAAACTGGAGGAGATCATGTCTAAATTAGGTTCTAGAACAAAACAAATGATTATAACTCCTATATAC
GCAAATTTACCCCAAGAGCAGCAATTGAAAATTTTCCAGCCGACTCCGGAAAATTGTAGGAAAGTTGTTTTGGCA
ACTAATATTGCGGAAACTTCATTGACCATTGATGGTATTAGATATGTCATTGATCCTGGATTTGTTAAGGAAAAT
TCGTACGTACCCTCCACTGGAATGACCCAACTTTTAACAGTTCCATGCTCAAGAGCTTCAGTTGATCAGCGTGCC
GGTAGGGCTGGTCGTGTTGGGCCGGGAAAATGTTTTAGAATTTTTACCAAATGGTCATATTTGCACGAGTTGGAA
CTTATGCCTAAGCCTGAGATCACAAGAACAAATCTTTCTAATACGGTGTTATTATTATTATCGCTTGGCGTTACT
GACCTAATAAAGTTTCCCCTGATGGATAAGCCCAGTATCCCCACATTACGGAAGTCTTTGGAAAATTTATACATT
TTAGGTGCCTTAAATAGCAAAGGGACAATCACTCGGTTAGGTAAAATGATGTGCGAGTTTCCTTGTGAACCTGAA
TTTGCAAAAGTGCTTTATACAGCTGCTACGCACGAACAATGTCAAGGTGTACTAGAAGAGTGTTTGACCATAGTT
TCGATGCTACATGAAACACCTTCATTATTTATTGGTCAAAAAAGAGATGCTGCGGCAAGTGTGTTAAGCGAAGTG
GAAAGTGATCACATATTGTATTTGGAAATCTTCAACCAATGGAGAAACTCTAAATTTTCCAGAAGTTGGTGTCAA
GACCACAAAATTCAATTCAAAACGATGTTGAGGGTAAGAAATATTAGAAACCAATTATTCAGGTGCAGTGAAAAG
GTTGGATTAGTTGAAAAGAACGACCAAGCCAGGATGAAGATAGGCAATATAGCGGGCTACATAAATGCTAGAATA
ACTCGATGCTTCATCAGCGGGTTTCCGATGAATATTGTACAATTAGGCCCCACCGGCTATCAAACCATGGGTAGG
TCATCAGGTGGGCTGAACGTTAGTGTTCATCCTACCAGTATACTTTTCGTTAATCATAAGGAAAAGGCGCAGAGA
CCAAGCAAATATGTGCTTTATCAACAATTGATGCTAACGTCGAAGGAATTTATCAGGGACTGTCTAGTTATCCCT
AAGGAAGAATGGTTGATAGACATGGTTCCACAAATCTTTAAAGATTTAATTGACGATAAAACAAATAGGGGGAGG
CGGTGA

>YDL240W 0.77   Cold
ATGATTCAAAATTCTGCTGGTTATCGATCGCTGAATACTGCATCTCCGATGACCGTGCAGGTGAAGAATCAGAAG
AAAATATGCGCCCGTTGTAACAAGTTAGTTATACCCGATAGCCAACGAACTAAAACAACTTTAAAGGCTCTCGGT
AAATATTATCATGAAAGTTGTTTTACCTGCCAAGACTGTCAGAAACCTTTAAAACCGAAGTACTTCCCATATCAA
GTAGACAAGACAAGTGAATCTATATTGTTATGCCAATACGATTATTTTAGAAGACATAATTTGTTATGTCATGTA
TGTGATACCCCATTGCGTGGATTATATTATACCGCATTCGGTTACAGATACGACGAAGAGCATTTTTCTTGTACA
ATATGTGCTACTCCTTGTGGGGTTAAAAAATGCTTCATGTACGGAAACCAATTGTATTGCAAGTACCACTTTCTC
AAGTATTTTTCGAAACGTTGCAAAGGGTGCGAGTTCCCAATATCGGATCAATATATCGAGTTTCCTAAAGGTGAA
GAAATACATTGTTGGCATCCGGAATGTTATGGAATTCATAAATATTGGCATGTAAATTTAGCAGCAGAAACTGTT

GGTTTGCAGTACCTTCCCAAGCTGGAATATAACCCAAATTCCGGAGACAAAGATATAAACCCAACTGCCTACGAG
TTAGATAAACAAATGCAGGCGTTTAACTTTATTCTATCTAAAACGTGGTCGGTTTTATATCGTTTTGAGGAAGAG
GCAGCTTCTTGCATTTCTGATATGTTTCAATATTTGACAAGCAATGATCAGTTAAAGGGCATAGAGTCTACGGGT
CTGTTGGTACTAAAAATCGACTGTCTTTTCCGGGGACTGGATACTTTAAACCTCTCGACGAACAAAAGTATGCCA
GTTAACAGTGATCAAGAATGCATTGAAAACAACGCAATGGCGGCCAGTAAATACAGCAAGTTTCCCAAAAATCTG
TCCACAAAAATTATGATATATTTACAACTCTTAAGAAAGTTAGGGACAGAAAACAAAAACGAAACTATCACAATT
TCATCTTTTATGTCTGTTATTACTGGATTAGCCCATTTTCTAAAACTGTTAACAAGATTTGGGCTATATACGGCT
CTAGAAAACAATAAGTTGACTCATTCTGTTAATCCATTGTTGAGGTTTTTAAGAGAAGTAGAGAAAAATGAGCTT
TTTGAAAATAACCCGTTTCAGTATATAAAGACTCCTGTTAACGCCACAGATAGTTGCGCTGGATGCAATAAATAC
ATACAGGAGGAATGCATTCAATTTTATGAGCACCGGTGGCATATTGCTTGTTTTACTTGTTCTTCATGCCATAAG
AATATAAATCCGAGGAGTTTGACGGATCCCACCTTTAATAAAGAGAAAAAAAAGATACTATGTTCTCACTGCTCA
ATTGATGATCCGGCCTCTGTGCCGGGTTTCAAATTTGTTACTAAGCTAGCACAACTAATTTTTCTTCTAAAAATT
GCCCTAGTCAAATCAAGAACTGTAATGTTGAAATCGAAAGCATCTAACAAGGTAGGCAGAAACTCTTTACAGAGT
ACTATGCTAAAGGAACACACTTACATTAGGACACTGAATGATATTAAAAGATTGAGATCTAGAAGAGAAAGTGTT
CGGGTCACTCATAACAAACAACAAGCTAGAAAATCAGTGATACTGGAAACTGCAGAGACAGATTTAAATGACCCG
ACAAAACAAGGAGATAGTAAAAATCTTGTGATTCAAACAGATGACCCATCATCGAGCCAACAAGTGAGCACACGT
GAAAATGTTTTCAGTAATACTAAGACGTTGACACTAGATGATATTTCACGTATTGTTGCCGCTGAACAAGCAAGG
GAGCTAAGACCTAACGCTTTTGCACACTTTAAAAAACTAAAAGAAACCGATGACGAAACTTCGAATGTTGTTCCC
AAGAAAAGCGGAGTTTATTACTCTGAATTAAGTACGATGGAACTCTCGATGATAAGGGCTATCAGTTTGTCACTG
CTAGCTGGTAAACAACTGATATCGAAGACTGATCCTAACTATACTAGCTTAGTCTCAATGGTCTTCTCGAATGAA
AAGCAAGTCACAGGTAGCTTTTGGAACAGAATGAAAATCATGATGAGCATGGAGCCTAAAAAGCCCATCACTAAG
ACTGTTTTTGGTGCTCCTTTAGATGTGTTGTGCGAGAAATGGGGTGTTGATTCCGATTTAGGTGTGGGGCCAGTG
AAGATTAGGATCCCCATAATTATTGACGAGCTAATATCATCTTTGAGGCAAATGGATATGTCAGTAGAAGGTATT
TTCAGGAAAAATGGAAATATCAGAAGACTGAGAGAACTAACTGCTAACATCGACAGTAATCCAACAGAAGCTCCA
GACTTCTCGAAAGAAAATGCAATCCAATTATCTGCTCTTTTGAAGAAATTTATCAGAGAGCTTCCCCAACCGATT
TTATCGACAGACTTATATGAACTATGGATTAAGGCCGCGAAAATTGATTTAGAGGATGAGAAACAGCGTGTTATA
TTGCTAATATATTCCTTATTACCCACTTACAATCGCAATTTACTGGAGGCACTTTTGTCATTTTTGCATTGGACG
TCCTCATTCTCTTACATTGAGAATGAAATGGGATCAAAAATGGACATTCACAATTTATCCACAGTCATAACACCT
AATATTTTATATTTGCGACATAAAGAAATTTCCAATGATAACGTCCCTGACGAACCAGAATCAGGTTTAGTTGAT
TCATTCGCTCAAAACAAGGGAGAAAATTACTTTTTGGCTATTGAGATAGTTGATTACCTGATTACTCACAATGAA
GAAATGGCAATGGTTCCGAAATTTTTAATGAATCTTTTAAAAGATGTGCAGTTACAAAAATTAGATAACTACGAA
TCAATCAACCATTTTATTTCCACCGTTATGCAAAGTAAAACAATCGATTATTCCGAATGTGACATAAAAACTCCT
GTCACAGTCAAAGATTCGACCACAACGGTCATACAAGGTGAAATAAACAAATAA

>YOL139C 0.77   Cold
ATGTCCGTTGAAGAAGTTAGCAAGAAGTTTGAAGAAAACGTTTCAGTCGATGATACCACAGCTACTCCAAAGACT
GTTTTAAGTGACAGTGCTCACTTCGATGTCAAGCACCCATTGAACACCAAATGGACTTTATGGTACACAAAGCCA
GCCGTCGATAAATCTGAGTCGTGGTCTGATCTATTACGTCCCGTCACTTCATTCCAAACTGTTGAAGAATTTTGG
GCTATCATTCAAAATATTCCTGAGCCACACGAACTACCATTGAAATCAGATTACCACGTCTTCCGTAATGACGTT
AGACCTGAATGGGAAGATGAAGCCAATGCTAAAGGTGGTAAATGGTCTTTCCAACTTAGAGGAAAAGGTGCTGAT
ATTGATGAATTATGGCTAAGAACTTTACTAGCAGTTATTGGTGAAACAATTGATGAAGACGACTCCCAAATTAAC
GGTGTCGTTTTAAGCATTAGAAAAGGTGGTAACAAGTTTGCCTTATGGACTAAATCTGAAGACAAAGAACCACTA
TTGAGAATTGGTGGTAAATTCAAGCAAGTTTTAAAATTAACCGATGACGGGCATTTGGAATTCTTTCCACATTCC
AGTGCCAATGGTAGACACCCTCAACCATCAATCACCTTGTAA

>YOR391C 0.78   Cold
ATGACTCCAAAAAGAGCGCTAATATCTCTTACTTCATACCACGGTCCCTTCTACAAAGATGGTGCGAAAACAGGC
GTTTTTGTAGTTGAGATTTTGCGATCGTTCGATACATTCGAAAAGCATGGTTTCGAAGTGGACTTCGTTTCTGAG
ACTGGTGGATTTGGCTGGGATGAACATTACTTGCCAAAGAGCTTTATTGGTGGCGAAGATAAGATGAACTTTGAA
ACGAAAAATTCCGCCTTCAATAAGGCGTTAGCGAGGATCAAGACCGCAAATGAAGTCAACGCCAGCGACTATAAA
GTATTCTTTGCATCTGCTGGACATGGTGCTCTATTTGACTATCCCAAAGCTAAAAATCTGCAAGATATTGCATCC
AAGATATATGCCAATGGGGGTGTGATCGCTGCCATCTGTCATGGACCGCTCCTTTTCGATGGATTAATAGATATC
AAAACAACAAGACCATTAATCGAAGGCAAAGCTATAACAGGTTTCCCACTCGAGGGTGAAATCGCCCTGGGAGTT
GACGACATCTTGAGGAGCAGAAAATTGACAACGGTTGAACGCGTTGCAAACAAGAATGGAGCCAAGTACTTGGCG
CCAATCCATCCCTGGGATGACTACTCTATTACAGATGGAAAGCTAGTTACGGGTGTTAACGCAAATTCTTCCTAT
TCGACCACAATTAGAGCTATAAACGCATTATATAGCTGA

>YLR224W 0.77   Cold
ATGAATCAGAGCGATAGCAGCTTGATGGATTTACCACTGGAGATACATTTATCATTACTAGAGTACGTGCCTAAT
GAACTTCGTGCTGTCAATAAATACTTCTACGTTTTGCATAACCACAGCTATAAGGAGAAGAGTTTGGCGTGGATA
GCTGAGGACAACTATATATGGGCCGTTGTCAAACATTCATTATGTTTATATGTCAAGAGTTTGGACCCACTTCGA
CAGCATGCCAGAGAAATCATTCAAGAAACGAAGGAACCAGGTTTTAATGTGCCACTGTGCATGACTAAATACATC
GCAGATTCTTGGTATATTGTATACAATGCGCTGCAATATCCTGGAAAGATAATTAATATGGGATGGGACAAATAT
ACCAAAAGTCAAGATTCAAATGGTTCTGATTCTACCAGTAACTTTAATAGTCGGCCCAAGGAAAGAACTCTTATG
CAGTCGTTGACAGCTTTACCTGTTAACTTTTGGTCCAGGAGGAAGGACGAGCCTACACCGGTAAACGTTTGGTTT
TATGTAAAAAATGCGCACGTTGCCAGATACATACCGAAAATTATTACGGAAATAGGCATATGCAACTATGGGCCG
AAGCAGATCGTGGCAAGTGCAGGATATATCAACGAATTGATAACATCTGAAGGAATATACTGTGTTAACCTAGGC

CACCTTCCCAGGCTATACGATGAGCAAATTTTTGAAGGCACTGGAACGACACATCTTCCCCTGGAACTGAAAGCT
ATCGACAGGACAGATTCAGATGTTTGTATCAATGGTGATTTAGTTCTACTCGGTTATGACTTTATTCCATATCAG
ATATCGAAGCCGTGGTTACTCTTTAGAATCGAACCCGTAAATAGCATTGAAGCAATCTTCAACTATAGTGAATGT
TCTTTTTCATACCAGTTTGCATGGAGTTTGGCCTGCTTACAATCTGAAGAGAAAATTTCATTTCCTAGAGACACG
ATAATTGGGCACGGTTTACCTTACAAGCCATCCAAATTGATAAGAATTTTCGTCTACAAGCATCCGGAACAAAAG
CAAGATCTTGGTCAAGAAATAGCATTGCCCAACTGGAATACCCCTTATCTTCGAAGATGA

>YPL099C 0.81   Cold
ATGTTAAAAAGACGCTCTAATGCTCTCATTACTTTGAGTAGAACTAAATTGTTTCCCATAACAACCGTTGCCTAT
TACCACAGGCGCCTCCTCAACCAACAACGAAGAGCTGTTTCAACGTCACCGAAGAAAGAAATCAAGAGCCTAGAA
GACTTGGCTAATTTGGATTCTTTGGATGGTGTTGATACTGAACTGATACGCGATTTGATTAATGAGCATACTACA
AAATTAAATATCAAAAAGGAGTTAGATATGCTCAAAAAATTCTCGCAAGAGGAAGAATCAGGACATGAGATCCCT
GTAAAAAGATTTATTAGGCCTCTTTGGATGTTCATTCTAATGGGATCTTCCGTTTATTTACTGCTTCACTTCTCT
TGGTGGAAACTAGAGCATGAGGAGAGAGAGAAAGTCAGCTAAAGAAAGAGGTAGAAATCCTGGAACACCAATTAAAT
GAGTTAATTGTACAGGATAAAACCCACAATACTAGTAGGGGAAAGGGCAGTAATGAATCAACACATATGAAGCCT
TGGTATAGGAGATGGTTTTGGTGA

>YOR005C 0.75   Cold
ATGATATCAGCACTAGATTCTATACCCGAGCCCCAAAACTTTGCGCCTAGTCCAGATTTCAAATGGCTTTGTGAA
GAGCTATTTGTGAAGATACATGAAGTTCAAATTAATGGAACGGCCGGCACTGGCAAATCAAGGTCTTTCAAGTAC
TATGAAATAATATCGAATTTCGTCGAAATGTGGAGAAAAACCGTGGGAAATAATATATATCCTGCACTGGTTCTT
GCTCTTCCCTACCGCGATAGACGAATCTATAATATTAAGGATTATGTATTAATAAGAACTATATGCTCTTACTTG
AAGTTGCCAAAAAAATTCTGCAACAGAGCAGCGGTTAAAAGATTGGAAACAGCGTGTCGGTAAAGGTGGGAATCTT
TCTTCTCTTCTTGTGGAAGAAATTGCTAAAAGAAGGGCTGAACCTAGCTCAAAAGCGATTACAATTGATAACGTC
AATCACTATCTGGATAGTTTGAGTGGAGACAGGTTCGCTTCCGGACGAGGATTTAAGAGTCTTGTCAAGTCCAAA
CCTTTCCTGCACTGTGTGGAGAATATGAGTTTCGTCGAATTAAAATACTTCTTTGATATCGTGCTTAAAAATAGA
GTAATAGGAGGTCAAGAGCACAAATTGCTAAACTGCTGGCATCCTGATGCTCAGGATTATCTTAGCGTGATATCT
GATTTAAAGGTGGTAACTTCAAAACTTTATGATCCAAAAGTTCGTCTAAAGGATGATGATTTGAGTATAAAAGTT
GGCTTTGCATTCGCCCCCCAATTAGCCAAAAAAGTGAATCTTTCTTATGAGAAAATATGCCGTACACTACATGAT
GATTTTTTGGTAGAAGAAAAAATGGATGGAGAACGAATTCAAGTTCATTATATGAATTATGGTGAATCCATAAAA
TTTTTTAGTAGACGGGGCATCGACTATACCTATTTGTACGGAGCGAGCTTATCATCAGGAACTATATCTCAACAT
TTGAGGTTTACAGATAGTGTTAAAGAATGTGTTTTAGATGGAGAAATGGTGACGTTTGATGCAAAAAGACGGGTG
ATTCTTCCATTCGGTCTTGTTAAAGGAAGTGCAAAGGAAGCGCTATCTTTTAATAGTATAAATAATGTTGACTTT
CACCCCTTATATATGGTGTTTGATCTGTTATACCTGAATGGGACTTCGTTGACACCATTACCCCTTCATCAAAGG
AAGCAATATCTGAACAGCATTTTAAGTCCCTTGAAAAATATTGTAGAAATAGTACGATCTTCTAGATGTTATGGT
GTGGAGTCAATCAAAAAGTCTTTAGAAGTTGCAATCTCACTGGGTTCAGAAGGAGTTGTTTTGAAATATTATAAT
TCAAGTTATAATGTCGCCAGTCGAAACAACAACTGGATCAAGGTAAAACCTGAATATTTGGAGGAATTTGGAGAG
AATTTAGACTTAATAGTAATAGGCAGAGATTCTGGGAAAAAAGATTCTTTTATGCTAGGGTTACTTGTGCTAGAT
GAAGAAGAGTATAAAAAGCACCAAGGAGACTCCTCTGAAATTGTAGACCACTCAAGCCAAGAAAAACACATACAA
AATTCAAGAAGAAGGGTGAAAAAAATACTTTCATTCTGTTCTATCGCAAACGGTATATCTCAAGAAGAATTCAAA
GAAATCGACCGCAAAACGAGAGGACATTGGAAAAGAACCTCCGAAGTTGCTCCCCCTGCTTCAATTTTAGAATTT
GGCTCAAAAATACCTGCCGAATGGATTGACCCCAGTGAATCAATTGTTCTAGAAATAAAATCACGGTCTTTGGAT
AACACAGAAACGAATATGCAGAAGTACGCTACCAATTGTACTTTGTACGGTGGCTATTGTAAAAGAATACGGTAC
GATAAAGAATGGACAGATTGTTACACACTTAACGACTTATACGAAAGTAGGACGGTTAAATCTAACCCCAGCTAT
CAAGCGGAAAGGTCACAGCTTGGATTGATACGGAAAAAGAGAAAGAGAGTACTTATTTCAGACAGCTTTCACCAA
AACAGGAAACAACTGCCAATTTCAAACATCTTTGCCGGATTACTTTTTTATGTTCTCTCTGACTATGTCACGGAG
GACACTGGAATACGGATTACACGGGCAGAACTTGAAAAAACTATTGTGGAACATGGTGGTAAACTGATATATAAT
GTAATTTTAAAACGTCATTCAATTGGGGACGTTCGGTTAATCAGCTGTAAAACTACCACGGAATGCAAGGCTTTA
ATAGATCGAGGATATGATATATTGCACCCAAATTGGGTACTCGATTGTATAGCATATAAGAGGCTCATCCTGATC
GAGCCCAATTATTGCTTTAACGTCTCTCAAAAAATGAGAGCCGTCGCTGAAAAAAGGGTAGATTGTTTGGGTGAT
AGTTTTGAAAATGACATTTCGGAAACCAAACTGTCATCATTGTATAAATCACAACTAAGTCTACCACCGATGGGG
GAACTCGAGATAGATTCTGAGGTTCGGCGGTTTCCATTATTTTTATTCTCCAACAGGATTGCATACGTACCACGT
CGCAAAATTAGCACAGAAGATGACATTATAGAAATGAAAATTAAGTTGTTTGGTGGAAAAATAACAGATCAACAG
TCACTTTGTAACTTAATAATTATACCATATACTGATCCTATTTTGAGGAAAGACTGCATGAATGAGGTACACGAA
AAAATAAAAGAACAAATAAAGGCTTCTGATACTATACCGAAAATAGCCAGGGTCGTTGCCCCTGAATGGGTGGAT
CATTCTATTAATGAAAACTGTCAAGTGCCTGAAGAAGACTTCCCCGTAGTCAACTACTGA

>YGL133W 0.8Cold
ATGGTGTTATATAAAAGGAAACCTATATTACTTCCTGATCCAAAACCTTTACCATTGGATTTGAATGTACAAGTA
TGGCATATTGAAGAGACTGGAGAATGGTTTTCTAGTTACGAAGAATTTTTAGAAAGGTTTGATTTCTACACACGC
CACCATTTTACATGCGAAATCACTGGTACTTCTTGCCTGACTTTTTTCCAAGCTCTGGATAGTGAGGAGACACAA
TTCAAATACGTCGAAGACAGATTCCCATTAAAGTTGAGAGAACCAGTTGCAAGATTTTTACATTTTAACGGAATA
AGAAGGTTAGATGCATTGGTGGAGAAAGTTTACGCAAGATTTAAAAATGACTTTTTCCCAGGAGAAGTTGTTTAT
CTACGTAAACAGAAAGACTCTTCTACCACAAGCTCTAATTCCCAACAAAGTACTCCTCAACCTGATGATATGGTC
GAAATCAACAGTGTTGGTAATCCTGGCTTACCGCAGTACCAATACCAAAGACGTTATGTTATCAAAGAAAAGGTC
CAATTTAACGCAACGATAAATCCAGAGAGCAGGGAAATTGTGATGCCCGCTCATACTAAATACATGCTGATAGAA
GAAGCAGCTTCCAGTAATAAATCCTTCATTGTTGATCAAGGACAAATTTACAGGGACCGTTCCACTTTCACTAAG

CACTTAATCAAATGTTTTTTCAAGATTACTTTACAGAGGGCATCTTCGAAAATGGGTGCACCTTGGTGTGTCAAA
CCGGAATATTTAGCGATGTATGGTTTAACGATGGAATGGCCAAAAGATATGTTAAAATATAAAGAAGATGAACCT
GTCGTAGCAAGACGTTCCAACAGTGCCAATGTTTCATCCCCAGAAAGTGAAAAGAATAAACGTCAAAGTAAATCA
TCGGGTAAAAGTAACACCTCAAACGATGCTTCTAACAAAAAAGAAACCAAGAAAAAAAGAAAGCCAACCGAAGTA
AATGATTCTGAGAATAATTCTTCGGAAGAAGACAAGAAGAAGGGTCAAAATGTCACATCAGAAACACATTCTAAA
AAAAGGAAAAAGGAAGCCAATGAGGAACCGAACACGGAAAACGTCGAAAGCGTTCCTACACCAGCTAATGCGGAG
CCTCAGGCTGTAACAATAACGAGCATAATGGATGACCTAGCTTTGCCATATCAACATCCACCGAACATCTTTCCA
AACTTAACGTATTATAATGAAAAATTAGAATGTATTTCATTAGGTTCTACAAAACTTTCCAGACCTTTTGATTCT
TTTGGAAAGCTATTACAAGCCTATCAGTTTCTGAATACGTTTGGTTCGAAAATTTGTCTCTCTCATTTTAGCCTT
GACCAATTCATTACTTCTTTGAAATGTACAGACCCATATGAGTTAAAGGGCGAAGTTGTTTTAGTGAACATAAGA
ACGCAAACGTCTAAAGAACAAGAAATTGAAAATAACGGCCTGCCGATGAAAAACAAAGCAGAGACTACTACTGAG
GAAGACTCTGAAAACCCAAGTGACTGGCAAAGAAATTCATTTATTCGTGATATGATAATGAAAAGAAATTCCGAC
AAGGTTGAGTACAAGATTGTTCACGATGACCCAGCATCCGATGATATACTTGATAATATCAATCATAATGGGTCT
GCTCTTCTCATTGAAGTTTTTACTGCGCTCTTACGTTTGTTCATCAACGAAGAAGGCGATTGGAGTTGCATCGTC
GTTGAAAACTGGATAATAGACGACAAGGGAGTCCTTATGGAGAGAAAAGATGAAAGAGGGGAGGGGGAGGCGAAG
CAAAAACGAAATGCTCACGGATACTTTTTACAAGATAAGGAAAAGATCGACAACCTAAAGGACACATTAAAGGAA
AACGCAACAGAAGTTCAAAAAGAATCAGACGCTAAAAACGAAACAAATTCAGAATCTGATTCTAAATCTGATTCT
GACAGTGAAGAACGAGATCCTAAATTAGAAAAGTGTTTGAACTACCGCAATGTTAACTGGATAGAACGCTTGACG
AAAAGGCAATTTAATAACAGTTACTGGCTAATAATTTTACTGGGAGTCTTAGAAGATTGTAGACATCTTCCCATG
TATACAGAGTTTATTGATTCTTTCATAGAAAAGATCATACCCAAAGATATATCAGCTACTCAATTGCCCAAACAG
CTTTGGAGAAATTTCTGTCGAAAGCTGTCGTTCAGTGACAAAGTCAATGCGCTGTGGATACTTGTAGATCTCGTA
TCGCATTTTTCTCCAGATATCAAAGCTGCTGTAGATGATTCCATGGAACTATGTGGTCAAATTCGTAGCGAAAGA
TTCAAAGTTGCGAGAGAGTTGAAAACGGAGGCAGCCGTCTTAAGTAATCTTCAAGGCGACCTGCAGGCAATTCAA
GAGAAGTTAAATAAAACAGATGAAAACACACCATCAGCCGATGGTGCCGATAAAAAAGATGATTCCGAATCAAAT
AGTGAACCAATTGATCTTATTATCATTGAGAAAAAGCAAAAGTTAATTGAAGAGCAGGATAAAAAGGTACAAGCT
TTACAATCAGATAAAAATTTCCTCGATAATTGTCTATTTGAAAACGATCTACAAAGATTAAAGCCGTTGGGATTG
GATCGATACGGGAACAGGTATTTTTGGCTGGATCACAATGGTGTTCCATTTCCTCAATATCCTGCTGGTATGAAT
GAGACGCCGAAAAGTAACAATAGTTTGAGTTATCACTCCGGTCGTTTATTAATTCAGGGACCTAAAGCATCATCG
GCGAAATTCTTTTTGAACGTCAGTGATGAGCAATTGAGCAATTGGCAAAAAATCAGAAATTCCGAAGGAATATCC
GAGGCAACTAGGGAAGTATTTGGTATCTCCAAAACTAGTTCCGGATCTTATAATTACGTAGAAAATGGAATAGAG
GTCGAACTATTGGACAGTAATGATCGCGTAAACCCACTTATAGAGCTGACACCTATTCAAAAGAAAATTATGGAT
GAAACACCTTCAAGGTTGCTACTATCACCAGATCAATGGTATTGCATTGACAAGTTAGAGGACTTGTCAAGAATA
ATGGACTGGTTAGATAATTGGGGTCGCAAAGAGCATGATCTCTTGAGGCAAATCAGACCCATAATGGAACGAATT
AAAAGTTCCCTTAGCTTGCGCGACCATGCGCTGTCACTGACAGCATTCACCAAAAATGAAGAAAAGTTGCTAAAG
GAGTTAGAAAATAATGAATTTACCGAAAACGAATTGAACGTTGATAGTATGGATGTTGATGATAAAAATAGCGGT
GTTAAAAGTGAGGTTGATGTACAGGTTGATGCCGAAGAGAAGAGAGAAGCTGTGATAGACGAAAAGTTGGAGGTG
ATTGCAGACGAACTCATGAAGCTAGATGATAGCTCGAAAACACGCAATGTTCTTAATAGAATACAAGAATTAGAA
GATCAGAGAGATGAACTCTTGGAACAGAAAAAATCCATAATAAATTCGCAGAGGCCAGGCGCGAGGATTTTAGCC
CGTTCAGAAAGGAAAAGAACCAAAATCTCCCGTGGTAATAAGGTGAATAAGCAGATTGAAATATTGACTGACTTA
GTCAATTATAGACATTTTAAAGCTATGGAAGATGTGATAGCTTGGAAAAACGTTTTAGCAAATTCGATATGGGGT
TCCTCACTTCGCAAAAATGCATCTGGTAATAAAAGGAGTGGTGTCATTGAAACAGTAGACGATAAATTAAAAGAT
ATAGTGGGCCAAACCTCAAGAACAGTAACACCTGCCCCAAATTAA

>YER162C 0.81   Cold
ATGAATGAAGACCTGCCCAAGGAATATTTTGAACTAATAAGGAAGGCTCTTAACGAGAAAGAGGCAGAAAAAGCG
CCCCTGAGTAGAAGAAGAAGAGTCAGAAGGAAGAACCAGCCCCTACCAGATGCCAAGAAGAAGTTTAAAACAGGG
TTGAATGAATTGCCACGAGAAAGCGTTGTTACTGTAAACTTAGATTCTTCAGATGATGGCGTCGTTACGGTACCG
ACGGATGATAGTGTAGAGGAAATCCAGTCATCTGAAGAAGATTATGATTCGGAGGAATTTGAAGACGTGACAGAT
GGAAATGAGGTGGCCGGTGTAGAAGATATATCTGTTGAAATTAAGCCCTCTAGTAAAAGGAATAGTGATGCTAGA
AGGACAAGCCGCAACGTTTGTTCTAATGAAGAACGGAAAAGGAGAAAATATTTCCACATGTTGTATCTAGTTTGC
TTGATGGTTCATGGATTTATACGAAATGAATGGATTAACAGCAAGCGCTTGTCTAGAAAACTGAGCAACTTAGTT
CCTGAGAAAGTGTTTGAGCTACTGCATCCTCAGAAAGATGAGGAACTCCCACTTAGGAGTACTAGGAAATTATTA
GATGGGTTGAAGAAATGCATGGAGTTATGGCAAAAACACTGGAAGATCACTAAAAAATATGATAATGAGGGACTT
TATATGAGAACCTGGAAAGAAATTGAAATGAGCGCCAATAACAAAAGAAAATTCAAGACGTTGAAAAGATCTGAC
TTTTTAAGGGCCGTCAGTAAAGGTCACGGAGATCCTGATATTTCAGTGCAAGGTTTCGTTGCGATGCTCAGGGCT
TGTAATGTTAACGCACGACTTATCATGTCATGTCAACCGCCAGATTTTACCAATATGAAGATTGACACTTCTTTG
AATGGAAACAATGCGTACAAAGATATGGTCAAGTATCCGATTTTTTGGTGTGAAGTGTGGGACAAATTCTCTAAG
AAATGGATAACTGTTGACCCAGTAAATCTCAAAACCATTGAACAGGTCAGACTTCATTCCAAGTTGGCACCGAAA
GGCGTGGCGTGCTGCGAAAGAAATATGTTGAGGTACGTGATTGCTTACGATCGGAAGTATGGCTGTAGAGATGTA
ACTCGAAGATATGCCCAATGGATGAACTCTAAAGTAAGAAAAAGAAGAATTACTAAAGATGACTTTGGTGAGAAA
TGGTTCAGAAAAGTGATAACAGCACTTCACCATAGAAAGAGAACCAAAATTGATGATTACGAAGATCAATATTTT
TTTCAGAGAGATGAAAGTGAAGGCATACCAGATTCGGTGCAGGATTTGAAAAACCACCCATATTATGTTTTAGAA
CAAGATATCAAACAGACTCAAATTGTAAAGCCAGGATGTAAAGAGTGTGGATATTTAAAAGTACATGGAAAAGTT
GGGAAGGTTTTGAAAGTTTATGCAAAAAGAGATATTGCGGATTTGAAAAGTGCTAGACAATGGTACATGAACGGC
CGAATTTTAAAAACTGGTAGCAGGTGTAAAAAGGTGATAAAGAGGACAGTTGGAAGGCCTAAGGGCGAAGCAGAA
GAAGAAGATGAAAGATTATATAGCTTTGAAGACACAGAATTATACATCCCTCCATTAGCCAGTGCGAGCGGTGAA
ATAACCAAGAATACATTTGGAAACATCGAAGTTTTTGCACCAACGATGATACCTGGTAACTGTTGTTTGGTTGAG

AATCCTGTGGCAATTAAAGCTGCTAGGTTTTTGGGGGTGGAATTTGCACCTGCTGTAACTTCTTTTAAGTTTGAA
CGCGGCAGTACAGTTAAGCCAGTTTTAAGTGGCATTGTTGTTGCAAAGTGGCTCAGAGAAGCTATTGAAACCGCT
ATTGATGGAATAGAGTTTATTCAAGAGGATGATAATAGGAAGGAACATTTGCTTGGTGCTTTGGAGAGTTGGAAT
ACTTTACTGCTAAAACTGCGTATTCGTAGCAAGCTGAACTCCACATATGGTAAAATTGCCGAGGAAGAACCTAAC
GTTACGAAGGAACAGAATATTGCGGACAATCACGATAATACGGAGACTTTTATGGGAGGTGGGTTCCTACCAGGT
ATAGCAAACCACGAAGCAAGGCCGTATAGTGAACCTTCAGAGCCAGAAGATAGTTTAGATTATGTTTCTGTTGAC
AAAGCGGAGGAAAGTGCTACAGACGACGATGTCGGGGAGGATTATTCGGATTTTATGAAAGAACTAGAGATGTCA
GAGGAATCAGACTGA

>YKL047W 0.76   Cold
ATGAATTCCGGAGGTGAAGAACCAACTATAAAGCCAAATGTATTTAATATTACTCAACTTTTAAACTCAAATGGT
GAAAAACCGGGAATTGCTTGCATCTTCTTAAGTAAATTTGACATGAAAAAGGGAAATATCATAATATGGTCTAAA
AGTATTAATGGTGCGGCAATTGACTTAAGCAATATTGAATTTAAATCGTTACCAGCCGGTATTCACGAACAAACT
GACGATGTTGTCAATTTTGTAGTTCCTAAAGAATTAGATGTCTGTCAAACTGCCAAGACGACAACTTATGATTAC
GGCATCGCCTATTTCAAACAAAATTCGTTCGATATTATTGAGAATGACAATCGTATTGATAGGAGTAAAGTTCAA
ATGTTTTCTCTTGGTGTAATTATCGATGTCCAAAATGCCTCATCAGATAGTAAAAAGCACTTTTATAAGGAAATA
TACCATGCGTATGCTGCCAACAGATACTCAAGCTATCTAGAAAGTTTACTCGGTCAATGGATAAGGCAGCGTGAT
CTTGATAAGTTTGATATTTTTGAAAAGTTTTTTGACGAGAATAACCAAGGGCACATGGCTGAAAATTCAGTAGAA
GTCTTTGAACATTCACCCAAAGAAAGACGGCATTTGGTTGAATATTTGCCATACTGGACAAGGAAGTTAGGGCCT
CTCATATTTCCACTTTGGAAAGCAAGTCTCCTTCAGTCAAGGATATTGATACTAGTTCCTCAAGGAGAATCGTTC
GAATTGTGCAATTCTTTGGCCTATTGTGTCTTCTTAATATCAATGCTTCCAAAAAACTTGATAGGAAACCATGTA
AGCGATGAATATATTAAGCCTATTTTCACGGTATCAACATCAGATATTCCTTTTCTAGAGTCGTTCAAAAAGGGG
AACGGTTATGTGGCTACGACCAGCGAGGAAATTTTGTTGTATAAGCCTGAAATTTACGACATAGTAGTCAAACTT
ACTTCGAGCTCAACTATAGAAGAATCACCAGAAAAAGAAGTGGAAATTCTTACCGCATCTGGTGAACAAAACAAA
GCAACTCCTCTTGACTTAGAGGTGTATGAAAAACTTATACTGGGGGAGTTACAAGAGGATGCCTCTACAAATGCG
ACATGTCGACATCATGAGGTGACAGAACCCATATCATGGCTACAGTTTTTAATTGATGGGTTTTTTCTTTTAACT
ACTGCAGGCTATCTGGTAGCGCCATATCACCTTGCAAATAATTTCAAGATTCCGAGGCACGTTTCTGGCCCAGAA
CCCAACAATTCAGAAATACAGATCGCTGAAAATTTAGTTCGCTATTTTCACCGCAGAACATCAAATCTGTACAAC
GATCTGAAGGATGTCATACAAAAAAGTGAAAATATTGATTCAGAACAACCGATTACTATCGCAGCTTCATTTTTA
ACCAAGTTAAATTTGGATTGCTTTAGCAAACAAGATCATCAATTCGTCAAGGATATCGCCCTTAAATGGTTCCAA
AGAAGCATTGATATTAGCAACCTACCAGAATGTTTGGGAAATCTCTGTTAA

>YBL009W 0.75   Cold
ATGAATTTTGATGCGGTAGCAGATCAACAGATGACTGACAGAAGGTATTTTGCTCTCGAAGTAGCAGAAAGCGAT
GATGCCGACAGCTCATTAAATTCTTCATCCATGGGAAGCCCCGCAGTGGATGTAGGAAGAAAAGTTTACAAAATC
ACTTCGCACAAGGGTTCAGCAGAAGATGAGAGTCAATCCTTTTTTACTTCTTCAGATTCTCCAACATCCAAAACA
AGGCCTGTAGGTAAAACCATCGAAAACGATGATTACTATGGTAAAAGATCTTCTACAGGTTCATCGCTCAAACAA
CTCTTCAATAAAATTAATATTAATGATACCGCTCACTCTTCAAACAAAGAAAATGTATCTCAGTCGGTGCTATCG
GAAAACAAGCTGCTCTCTCCATCTAAAAGGTTGTCGAAGCAAGGTCTTACAAAGGTGACTAACTCCAAGTTTCGT
ACACCCTTGAGACCTATTTCAAACCAATCGACTTTATCAAGGGATGAGCCTGTTAAAGATTTTAGATCACTTAAG
TTTCGGAGCGGTAGTGATTTCAAATGCTGGGGTGACGAGAAGACAAGTTCTCATGTTCATTCATCCAGTGTGAAC
TCAGTTAATTCCTTTACTTCTACCACCTCTTCTTCAAAGTGGAAGTTCTGGAAAAATGATAACCTATTGTCGAGG
TCGCTATCTTCCAGATCTGTGAATGACCAAGATCCGAACTTTGTTCAGCCAAAACCAACCAATTCGTTACAAAAG
AAGTCTTCAATTTCAAGTTTTCACAATTCTATTTTTGGTGGTGGCAAACACACAGAGAAGAAGAGGAATTCTGGA
TTTATTATGCCCGATCATCAGAGCACGAAGGAGCTAAACCACAAACATTCATCATCGAACCTTTCCTTTAGAAGT
TTGAAGCATAAAACATCACATTCTTCACTAAATAAGCTCAAAGTAAGGCGTAAAGGAAATACACAAGAACTGAAT
CATCCGATCAAAAAAACTTGCCAAATATCATTGCCTGTTCCAGACCAAGTCTCAAAGGACAAGATTCAACTGAAA
TTGAAAAATTCAACATCATTGGCATCCTTATCTTCAGAAGTCACTCCCATAAACACTTTGGACTACAATGATTCA
ATTTTGCAACAAATATTACAACTTTGTGATGTTAAGTATATATTACATGATCTACGTGAAGCTCAGTCATTAGGC
TTGTTTACGTTGAATACTAGATCGGTTCAGCTGTCTCATAACTTTTGGCAAACTTATCATAGCGATATGCAAACT
TCACTCATTTGCAAGAAAGTATGTCTAGGGGCTCTAAGTGATTTGACTACTTCAAACTTGATATCCTTACATGAA
TTGAAATCATTACGGTTAATACAAGGAACTAGCGGTGTCGCCAATTTACTGCAAGCTTATGTTGTGCCCTCAAAT
CAATGTGAAAATGACCAAAACTTGATACTGTACTTATTTTTCAAATACCAGGGAACTCCTCTATCAAGGTGCTCT
AACATTGATTACTCTCAAGCATTGTCTATTTTCTGGCAGTGCAGCAGTATTTTATATGTTGCTGAATCCAAATTT
CAGCTCGAACACAGGAATCTAACTTTGGACCATATTTTGATAGACTCTAAAGGGAATGTTACTTTAATTGATATG
AAGTGCTGTCGTTTCTTGAATATAGACAACAATAAGGCTTCCTATACAAGATTAGACCATCATTATTTCTTCCAA
GGCCGGGGGACTCTTCAATTCGAGATATATGAACTGATGAGAAGCATGCTGCCTCAGCCAATATCCTGGGCTACA
TTTGAACCAAGAACAAACCTATTATGGTTATATCACCTAAGTAGCAGCCTGCTAAAAATGGCCAAAAAAGCCGTA
GTCAGCGGCGCTTTGAACCGGGAAGAAAATATCTTAATCGAGTTGACGCACTTACTCGATCCTGCTCGAAAACAT
TCCAAAACAATTTTCAAAAAGGAACTTGTTATAAGAACTTGCGGTGATTTGTTATCTTTGAAGGGAGAAATAATG
CAGTAA

>YKR023W 0.82   Cold
ATGACAAGAAAGCAGGCTATTGACTATGCAATCAAACAAGTTCCGCAAATATTGCCTTTGGAAGAGTCTGATGTA
AAAGCGCTGTGCGAACAAGTACTGAGTACATCATCTGATGATCCAGAACAAATTGCTTCAAAATTTTTGGAGTTC
CTAGGTCATGAAGATCTATCGTTTGAATTTGTAATGAAGTTCAATGAATTATTGAATCAGAATGATAAAAAAGAG
GAAAAAAAAACGAAAAATGTGCATCTTGAACATACTGCCCCCACTTCATGGAAAAATGAATCAAAACAGCCCACT

AATAACTATATCAATAAGAAGGGAGATGAAAAACCAAAAAAATTAAAGGATGAGAAGAAAAGCTCGACAACGAGG
CCTACCGTTCAACCATCAAATCAATCTACGCAATCAAACCCCATAAAGGAGAAAAAAGAGCACAGATCAAAGGGG
AAATTACAATCGTTACAAGAAATAGACGAAGCAATCAAGATGCTAGAGCTTCGAGATAGCGGTTCTTCTAAAAAT
TGTAATTGTCAAGGTACAAGGCATCCAGTTTTCGACATCGCGCCAAACTGTCTTCACTGTGGTAAAGTGGTTTGT
GTTATAGAGGGATTGAATAAAGGTAAATGCGGTCATTGCCATGAACAGTTGATTTCTGATAACGAAAGAACCCAA
ATGGTAGAAATTTTAAATCAGGAAAAAAACGAACTAAACGGTTCATCATCGTCCTTATCCAATGCGTCAAATGGA
GCTAATGTCCCAAAGAAGAAGACTAAAACCTACAAAATAACTTCCGGTATGGGTAAAAATTTATTTGCTGAACAG
GACAAATTATTTGATTTCATCGAAAGGAAAAGAGAAAGAGAAAGAAAGCGTAATGAAGTGTTGAAATTGCAGGAG
GAAAAAGAAGAGAGCGAAGCAAAGGAAAGACAAGCGAGCGAGCATGACCATAAAGCTGAGGAAAACCCGGAGTTG
TTAGCAGCACAAGAACGATTAGATAGACTTTTATATTTCCAAGACACTTCGGCAGAAAGAACAAAAATTATTGAT
AATGCCAGCGATTTTGACATGAATCAGGAGGTGGGTTTATGGGGCAGCGCAAGAGAAAGAGCTCTTGCGTTGAAG
AAGCAACAACGTAATTTGAGAAAGTGGGAAAAAGTAGAAAAGGAACGGAATGGCAGGCGCGAGAAGTACGTTGTT
AGCATGAATATAGGTTCAAACGGTAAAGTTACTATGACAGAAGTCCCTAAGGATACAGAGAACGTAATTGCAGGT
TCAGATGATGACATCAGTGATATAAGTGACGAAGAAGATATCAGCGACCTTAAACATATACACGCTCTGAAAAGT
GAAATAAATACAACGAAATCCTTGGAGAATTTGCATTTACAGTCCAAAGCATGGGATTACGAACGTGATAAAAAA
CAATTCGATAGGCCTACGTATGTGAAAAAAAATTCAGATACTGCACAACAGAATAGAAAAACGGAAGAAAAGGCT
CACGACATGCAAGCTTATGATTTAAAGTCAAGAGTACAAGTGGACCAAAATGCAGACGCATCAGTAGAACAGAAT
ATTCTTGCTGTACTTTGA

>YOR262W 0.82   Cold
ATGCCCTTCGCTCAGATTGTTATTGGTCCACCAGGTTCAGGGAAGTCAACCTATTGCAACGGCTGCTCACAGTTC
TTCAATGCCATCGGAAGACATTCCCAGGTAGTGAATATGGATCCTGCTAATGATGCCTTACCTTACCCATGCGCT
GTGGATATTCGTGATTTTATAACATTAGAGGAGATCATGCAAGAGCAACAGTTAGGCCCTAATGGAGGTTTGATG
TATGCTGTTGAATCATTGGATAATTCTATTGATTTGTTCATTTTACAGATCAAGTCACTTGTAGAAGAAGAAAAA
GCATATCTTGTATTCGACTGTCCGGGCCAAGTGGAGCTATTTACTCATCACTCATCTTTGTTCAACATCTTTAAA
AAAATGGAAAAGGAATTGGACATTAGGTTTTGTGTTGTAAATTTGATTGACTGTTTTTACATGACATCCCCTTCA
CAATATATCTCGATTTTGTTACTTGCATTGAGGTCTATGTTAATGATGGATCTCCCTCACATCAACGTTTTTTCT
AAAATAGATATGCTGAAATCATACGGAGAATTACCCTTTAGATTAGACTATTATACAGAGGTCCAGGATCTGGAT
TATTTGGAGCCATATATTGAAAAGGAAGGCTCTAGTGTACTGGGAAAGAAATATAGCAAGTTAACTGAAACAATC
AAAGAGCTAGTCTCAGATTTCAACTTAGTATCATTTGAGGTTTTGTCCGTGGATGACAAAGAAAGTATGATAAAT
CTTCAAGGTGTTATAGATAAAGCAAATGGCTACATATTCGGCGCATCCGAAGTTGGTGGTGATACCGTGTGGGCT
GAGGCTTCGCGAGAAGGTGCATTAATAGCGAATTACGACATTCAAGACAGGTGGATAGACAATAAAGAGAAGTAT
GATAAAGAAGAAGAAGAAAAACGTACGGCGTTGTTAAAAGAACAAGAATTGCAAAATAAAGCTGTTGATGTGAAT
GAAGAAGATGAGTGGGAAAATGCGCTGAAGGAGTGGGAAGAGAAACAAGGAATGGATTTTGTTAGGTAG

>YDR017C 0.8Cold
ATGGATACCTCTCACGAAATTCATGATAAAATACCCGATACATTAAGAGAGCAGCAGCAGCATTTGCGCCAAAAA
GAGTCGGAAGGGTGCATAACAACATTGAAAGATCTTAATGTACCTGAGACGAAGAAACTTTCCTCCGTCTTACAT
GGCAGAAAGGCGAGTACATATTTAAGAATTTTTAGAGATGATGAATGTTTAGCCGATAACAACAATGGTGTAGAT
AGCAATAATGGCGGGTCCGTGACATGCGCGGACAAAATAACAAGGTCAGAAGCAACTCCCAAATCGGTACCAGAA
GGACTACAAGTTTCTGAAAAAAAGAATAACCCAGATACTTTGTCCTCTTCATTGTCAAGTTTCATTTTATCTAAC
CACGAGGAGCCGGCAATTAAGCCCAATAAACACGTGGCGCATAGAAATAATATTACTGAGACAGGTCAAGGCTCT
GGTGAGGATATCGCAAAACAACAATCACACCAACCACAGGTGTTACACCACCAGACTTCTTTAAAGCCAATTCAG
AATGTTGATGAGGGATGTATTTCGCCTAAGTCCACTTATCAGGAGAGCTTACATGGAATATCGGAGGACTTAACG
TTAAAGCCTGTTTCTTCTGCAACATACTATCCTCACAAAAGTAAAGCAGATTCTGGTTATGAAGAAAAGGATAAA
ATGGAAAATGATATTGATACTATTCAGCCTGCCACCATAAATTGTGCCTCTGGTATAGCGACACTTCCTAGTTCA
TATAACCGACATACTTTCAAAGTTAAGACATATTCAACTTTATCACAATCCCTAAGGCAAGAAAACGTCAACAAT
CGTAGTAACGAAAAAAAACCTCAACAGTTTGTGCCACATAGCGAATCAATTAAAGAAAAACCCAATACGTTTGAA
CAAGATAAAGAAGGCGAGCAAGCGGACGAAGAAGAGGATGAAGGTGATAATGAACATAGGGAATATCCCCTAGCA
GTTGAACTAAAACCATTTACGAATAGAGTTGGAGGTCACACTGCAATATTCAGATTTTCGAAGAGAGCGGTTTGT
AAGGCTCTAGTGAATAGAGAAAACAGGTGGTATGAGAATATAGAGCTGTGCCATAAAGAATTATTGCAATTCATG
CCACGTTATATTGGTGTCCTGAATGTTAGGCAACATTTTCAGTCAAAAGATGACTTTTTAAGCGATTTGGATCAA
GAAAACAACGGTAAAAATGATACCAGTAATGAAAACAAAGACATAGAAGTCAATCATAACAATAACGACGATATT
GCGCTTAATACAGAACCCACGGGGACCCCCTTAACACACATACATTCCTTCCCCTTGGAACATTCGTCGAGACAA
GTACTTGAAAAAGAGCATCCTGAAATTGAGTCTGTCCATCCACACGTGAAAAGATCACTGTCAAGTTCCAACCAA
CCTTCCTTGTTGCCCGAGGTTGTATTAAATGATAACAGGCATATTATACCGGAATCTCTGTGGTACAAATATTCC
GATTCTCCAAACTCGGCACCGAATGATTCGTACTTTTCGTCCTCCTCTTCTCATAACTCATGTTCGTTCGGTGAG
CGTGGCAATACAAATAAATTAAAAAGACGCGATTCTGGGTCCACCATGATCAATACTGAATTGAAGAACCTAGTC
ATAAGGGAAGTATTTGCACCGAAATGTTTTAGAAGGAAAAGAAACTCTAATACGACTACAATGGGAAACCATAAT
GCGCGTTTGGGTTCCAGTCCATCTTTTTTAACACAAAAATCAAGAGCCTCGTCCCATGATGCATCGAATACGTCT
ATGAAGACTCTTGGTGATTCATCGTCACAAGCGAGTTTACAGATGGATGACTCGAAAGTCAATCCGAATTTGCAG
GATCCTTTCTTGAAAAAATCGCTTCATGAAAAAATATCTAATGCTTTAGATGGCTCGCATTCAGTAATGGATTTG
AAGCAATTCCACAAAAACGAACAAATAAAACATAAAAACTCTTTCTGCAACTCTTTATCACCCATTCTCACTGCA
ACCAATTCTAGAGATGATGGCGAATTTGCCACCTCCCCGAATTATATCAGCAACGCACAAGATGGTGTTTTTGAT
ATGGATGAAGATACGGGAAACGAAACTATAAATATGGATAACCACGGCTGCCATCTTGACTCTGGTAAAAACATG
ATTATAAAGTCATTGGCGTATAATGTTTCAAATGACTATAGTCATCATGATATTGAATCAATTACGTTTGAGGAG
ACGTCCCACACCATTGTTTCAAAATTTATCTTATTGGAAGATTTGACAAGAAACATGAACAAGCCTTGTGCACTG

GATCTAAAGATGGGGACAAGACAATATGGCGTAGACGCCAAAAGAGCCAAGCAACTGTCACAGCGTGCAAAGTGT
TTAAAGACCACTTCTAGGCGTTTAGGCGTTAGAATCTGCGGATTAAAGGTTTGGAACAAAGATTATTATATCACA
AGAGATAAATACTTTGGTAGAAGGGTTAAGGTTGGTTGGCAATTTGCTAGGGTACTTGCACGTTTTTTGTATGAT
GGTAAGACCATTGAAAGTTTAATCAGGCAAATTCCAAGGTTGATCAAGCAACTAGATACTTTGTATTCTGAAATT
TTTAATTTGAAAGGTTACAGGCTATATGGCGCGTCTTTATTACTAATGTACGATGGAGATGCTAATAAATCAAAT
TCTAAAAGGAAAAAAGCTGCTAATGTTAAAGTCAACTTGATCGATTTTGCACGTTGTGTTACTAAAGAAGATGCA
ATGGAATGTATGGATAAATTTCGTATACCCCCAAAATCGCCAAATATAGAAGATAAAGGGTTCTTACGAGGTGTG
AAGAGCTTGAGGTTTTATCTGTTATTAATCTGGAATTATCTGACTTCTGATATGCCGTTAATTTTCGATGAAGTA
GAGATGAATGATATGATTAGCGAAGAAGCAGATTCGAATAGTTTCACCTCCGCCACCGGTTCGAAAATCAACTTC
AACAGTAAGTGGGATTGGTTAGATGAATTTGACAAAGAAGACGAAGAAATGTATAATGATCCAAATAGTAAACTA
AGACAAAAATGGAGAAAATACGAGCTTATTTTCGACGCTGAACCAAGATACAATGACGATGCTCAAGTTAGTGAT
TGA

>YMR189W 0.8 Cold
ATGCTTAGGACAAGAGTGACTGCTCTCCTTTGTAGGGCTACTGTCAGGTCAAGCACCAATTATGTTTCATTAGCG
AGGACTAGATCATTCCATTCTCAATCTATTTTGCTCAAAACAGCCGCTACAGACATAACGTCTACACAGTACAGC
AGGATTTTCAATCCTGACTTGAAAAACATTGATAGACCGCTAGATACTTTTGCTAGACGTCATTTAGGTCCTTCT
CCTAGCGACGTTAAGAAAATGTTAAAAACAATGGGTTATAGCGATTTAAACGCATTTATAGAAGAGCTCGTTCCT
CCCAATATTTTGAAGAGAAGACCCTTGAAACTAGAAGCTCCTAGTAAGGGATTCTGTGAACAAGAAATGCTTCAA
CATCTAGAAAAGATTGCCAATAAGAACCACTATAAAGTTAAAAATTTCATAGGTAAGGGTTACTACGGTACGATT
TTACCACCGGTTATACAAAGAAACCTGCTAGAAAGTCCAGAATGGTATACTTCTTATACGCCCTATCAACCCGAA
ATTTCTCAAGGTAGGCTAGAAGCGCTATTAAACTTTCAAACGGTTGTTTCAGATTTGACTGGTTTGCCTGTGGCG
AACGCCTCATTGTTGGATGAGGGTACAGCGGCTGGAGAAGCTATGCTCTTGTCATTCAATATTTCCAGAAAAAAG
AAACTAAAATACGTAATAGATAAAAAATTACACCAGCAAACAAAGAGTGTCCTTCACACCAGAGCCAAGCCGTTC
AATATTGAAATTATTGAAGTTGACTGTTCGGATATCAAGAAAGCTGTGGATGTTTTAAAGAACCCCGACGTATCT
GGTTGTTTGGTTCAATATCCAGCGACAGATGGTTCAATCTTACCGCCTGACTCGATGAAACAGTTATCTGATGCG
TTACACTCTCACAAGTCTTTGCTCTCTGTGGCCTCAGATTTAATGGCTTTGACACTTCTAAAACCACCTGCTCAT
TACGGTGCTGATATCGTCCTGGGTTCCTCTCAACGATTTGGTGTCCCAATGGGTTATGGTGGTCCTCATGCTGCT
TTTTTCGCTGTTATTGATAAATTAAACAGAAAAATTCCAGGTAGAATTGTCGGTATCTCTAAAGACCGCTTAGGC
AAGACGGCCTTGCGGTTGGCCCTCCAAACAAGAGAACAACATATCAAGCGTGATAAGGCAACTTCAAATATATGT
ACCGCTCAAGCTTTACTGGCTAATGTTGCTTCGAGCTACTGTGTTTATCACGGTCCTAAGGGCCTACAGAATATT
TCCAGGAGGATATTTAGCTTAACATCAATATTGGCAAATGCCATCGAAAATGACAGTTGCCCTCACGAACTAATT
AATAAAACATGGTTTGATACTTTGACTATAAAGTTAGGTAATGGCATATCCTCCGAGCAGTTATTGGACAAGGCC
TTGAAAGAATTTAATATCAATTTGTTTGCCGTGGACACCACCACTATTTCCTTGGCTCTTGATGAAACAACTACA
AAAGCTGATGTTGAAAATCTACTAAAAGTGTTTGACATTGAAAATTCTTCGCAGTTTCTTTCTGAGGACTATTCT
AACAGTTTCCCAAGGGAATTTCAGCGTACTGATGAAATATTGAGGAACGAAGTCTTTCACATGCACCATAGCGAA
ACAGCAATGTTGAGATATTTACATAGGTTGCAATCTCGTGATTTATCTCTTGCTAATTCTATGATTCCTTTAGGT
TCCTGTACTATGAAATTGAACAGTACTGTTGAAATGATGCCAATCACTTGGCCCCAATTTTCGAATATCCATCCG
TTCCAGCCATCAAACCAAGTCCAAGGATACAAGGAACTAATTACTTCGTTGGAGAAAGATTTATGCAGCATTACA
GGTTTCGATGGTATTTCTTTACAACCAAATTCAGGTGCTCAAGGTGAATATACTGGTCTGAGAGTAATCAGATCC
TACCTGGAAAGCAAAGGTGAAAATCATCGTAACGTGTGTTTAATCCCTGTATCCGCTCATGGTACAAATCCGGCT
TCTGCCGCTATGGCGGGTTTAAAAGTTGTTCCTGTCAACTGTTTGCAGGATGGCTCATTAGATCTGGTTGACTTA
AAGAATAAGGCTGAACAACATTCTAAAGAACTAGCCGCCGTAATGATCACCTATCCTTCCACTTACGGTTTATTT
GAACCAGGCATCCAACATGCTATTGATATCGTACATTCTTTTGGTGGACAAGTCTATTTGGATGGTGCTAATATG
AATGCGCAGGTTGGGCTAACTTCACCCGGAGATCTTGGTGCAGATGTTTGCCACTTGAATTTACATAAGACATTT
TCCATTCCTCATGGTGGTGGTGGTCCAGCTGGAGCTCCCATTTGCGTCAAATCTCATTTAATACCCCATTTACCT
AAACATGACGTTGTTGATATGATCACTGGAATCGGCGGTAGCAAATCCATCGATTCGGTCTCCTCTGCTCCATAT
GGTAATGCTTTAGTGTTACCAATTTCTTATGCCTATATCAAAATGATGGGTAATGAGGGATTACCATTTTCTAGT
GTGATAGCAATGCTAAATTCAAATTATATGATGACAAGATTAAAAGATCATTATAAAATTCTTTTCGTCAATGAA
ATGAGCACACTAAAACACTGCGCTCATGAATTTATAGTTGATCTAAGAGAATACAAAGCTAAAGGTGTTGAAGCT
ATCGATGTTGCCAAGAGATTGCAAGACTACGGATTCCATGCCCCAACGTTGGCCTTCCCTGTTCCCGGAACTTTG
ATGATAGAACCAACAGAATCGGAAAACTTGGAAGAATTGGATAGATTCTGTGATGCCATGATATCCATCAAAGAA
GAAATAAATGCCTTAGTAGCAGGTCAACCAAAAGGACAGATTTTGAAAAATGCCCCTCATTCATTGGAAGATCTT
ATTACTTCCTCCAATTGGGATACGAGAGGTTATACCCGTGAAGAAGCCGCTTACCCATTACCCTTTTTGAGATAC
AATAAATTCTGGCCTACTGTCGCTAGACTGGATGACACTTATGGTGACATGAATTTAATATGTACATGCCCTTCT
GTAGAAGAAATTGCGAACGAAACTGAATGA

>YGR133W 0.78 Cold
ATGCCAAACTTCTGGATTCTTGAGAATCGTAGGAGCTATACGTCTGATACATGTATGTCGAGGATCGTGAAGGAG
TACAAAGTCATACTGAAAACATTGGCCAGTGATGACCCCATAGCGAACCCCTACCGGGGGATAATCGAGTCACTT
AACCCGATTGACGAAACCGACTTGAGCAAGTGGGAAGCTATAATTTCTGGGCCGTCAGACACACCTTATGAAAAT
CATCAGTTTCGAATACTAATAGAAGTCCCTAGTTCCTACCCCATGAATCCACCAAAGATAAGTTTTATGCAGAAC
AATATTTTGCACTGCAATGTTAAATCAGCCACAGGAGAAATATGTCTGAACATCCTTAAACCAGAGGAGTGGACT
CCTGTATGGGATTTGTTACACTGTGTTCATGCTGTTTGGAGACTCCTAAGGGAACCCGTTTGTGATTCTCCACTT
GACGTAGACATTGGTAATATCATCCGCTGTGGCGATATGAGTGCCTATCAGGGGATTGTAAAGTACTTCCTAGCA
GAAAGAGAGCGGATCAACAACCATTGA

>YFL055W 0.79 Cold
ATGGCAGTCCTTAACTTGAAACGTGAAACTGTCGACATTGAAGAGACAGCGAAGAAAGATATCAAACCTTATTTT
GCTTCGAATGTTGAAGCGGTTGATATTGATGAAGATCCCGATGTTTCAAGATACGATCCCCAGACAGGAGTGAAA
AGGGCGCTCAAAAATAGGCATATCTCATTGCTAGCTTTGGGTGGTGTTATTGGCCCAGGTTGTCTTGTTGGTGCA
GGAAACGCACTCAACAAAGGTGGGCCACTTGCTTTACTTTTAGGCTTTAGTATTATTGGGATCATTGCTTTCTCA
GTGATGGAATCTATAGGTGAAATGATCACTTTATATCCCTCGGGCGGTGGATTTACCACTTTGGCTCGAAGATTT
CATAGCGATGCACTGCCTGCAGTTTGCGGTTATGCTTACGTTGTTGTGTTCTTCGCAGTTTTGGCAAATGAGTAC
AACACTCTCTCCTCCATACTACAGTTTTGGGGCCCACAAGTCCCTCTATATGGTTACATCTTGATATTCTGGTTT
GCATTTGAAATTTTTCAACTAGTTGGCGTTGGTCTTTTTGGTGAAACGGAGTACTGGCTTGCTTGGTTGAAAATA
GTAGGATTAGTAGCCTATTATATTTTCTCGATTGTTTACATATCTGGGGATATTAGGAATAGACCAGCTTTCGGC
TTTCATTATTGGAATAGTCCAGGTGCATTATCACATGGGTTTAAGGGAATTGCGATAGTGTTTGTGTTTTGTTCG
ACCTTCTATTCTGGAACGGAATCAGTTGCCTTGGCTGCAACGGAATCAAAAAACCCTGGGAAGGCTGTGCCACTT
GCTGTTCGACAAACTCTGTGGAGAATTTTAGTTGTTTATATTGGAATTGCTGTTTTCTATGGAGCAACTGTTCCG
TTTGACGACCCAAACCTCTCTGCTTCTACCAAAGTCCTAAAATCTCCCATTGCTATCGCCATATCTCGTGCTGGT
TGGGCCGGCGGAGCTCATCTGGTTAATGCCTTCATTTTGATAACTTGCATCTCCGCCATTAATGGGTCACTTTAT
ATAGGGAGCAGAACCTTGACGCATTTAGCACATGAAGGCCTAGCTCCAAAAATTCTGGCTTGGACCGATCGAAGA
GGCGTTCCCATCCCCGCCATCACTGTTTTCAACGCCTTGGGCCTAATATCATTGATGAATGTGAGCGTTGGAGCT
GCAAATGCGTACTCTTATATCGTTAATCTTTCTGGTGTTGGCGTCTTTATTGTCTGGGGTGTAATAAGTTATACG
CACCTGAGAATAAGGAAGGCGTGGGTTGCTCAAGGAAGATCCATAGAAGAGCTACCTTATGAAGCGCTATTTTAT
CCGTGGACGCCAGTACTTAGTCTGGCCGCTAACATTTTTCTAGCACTCATCCAAGGATGGAGCTATTTCGTACCT
TTTGATGCGGGCAATTTTGTTGATGCTTATATCCTTCTGCCTGTTGGAATTTTATTGTATATTGGCATATGTGTT
TTTAAGAGCAATCATTTTAGAACTGTTGATTTGCGGTCAATCAACCTAGACGAAGGACGAAGAAAAGACATGGAG
GCTGATCTTTCTGATCAAGAGAGTAGCTTAGCATCTTCGGAAACGATGAAGGATTATAAAAGTGCAACTTTTTTC
AGATACCTCAGCAACATTTTCACCTGA

>YOR353C 0.8 Cold
ATGGTAGCGACATCTTCAAAGAGGACGTTGGATCCGAAGGAGGAACATTTGCCTGCTGACAAAACATCCACTAAT
TCAAGCAACACTATAATATCTGAGTTGGCAACACAAGAGAAATCCAGCTCTAGTGGTACTACACTAAAATTAATA
GCTCTTAATATCAAGTCCATATCAGATGAAGATGTGGGATATATCCAAAATGTTGAAAGACTGTCTTTAAGGAAA
AATCACTTAACATCCTTACCAGCGAGTTTCAAGAGGTTATCAAGGCTGCAATATCTGGATTTGCATAATAACAAT
TTTAAAGAAATCCCATATATTTTGACACAATGCCCTCAATTAGAGATCTTGGATCTGTCCTCCAATGAGATTGAA
GCATTGCCAGATGAAATATCATCGTTTTGGCAAGATAATATTCGTGTATTATCATTGAAAGACAACAACGTTACT
AGCATACGTAATTTGAAATCGATCACAAAATTAAACAAGCTGAGCATCTTGGATTTAGAGGATAATAAAATACCC
AAAGAAGAGCTTGACCAAGTACAGAGTTATACTCCATTTCATACGGGCATCCCCAAAGAAGAATATTGGGCAATT
GCGATATCACGATATCTAAAAGATCATCCTAACCTACCTACTCCAGAACCTAAAATATCTAGAGCAGCGAAAAGA
ATGGGATTCATAAATACGAACTTGTCTAATGGAGCAATGAATGAAAACAATATAATTTCACTTGCCCCAAGTGCA
AATACAACTATAAGTGCATCTACTGCAATGGTCTCATCTAATCAAACCTCTGCAACATCTTTCAGTGGTACAGTC
AATGCAGAATCAGAACAAAGTGGGGCAGTGAATGGTACGGAACTATACAATCACACCAAATATAATGACTACTTC
AAGAGGTTGTCGATTTTACCAGAAGAGTCAATGAGTAACGGGCATCAAAAAATATCGCATGCAGAGTTGGTTGTT
TCCTGTCGGAAGCTCCTATTTAGTTTTACAGAATGCCAACAAGCTATCAGAAAAATCGCCTCTTTCTGTAAAGAG
AAGGCCGTAGCGGTTAATGTAGTATCATTATTATACTCTGTCAGATCACATACTGATAATCTGGTGGAGGTCTTG
CAGCAAACAGAGAACGAAGACGAATCGCATGATCAGGCGTTAATCAAGCTGTGCCTCACTATCATCACAAATTTT
AAACAAATTATAACATTGTTGAGAAAAAATTTTGAGATTTTTTTCAAAGAAGACGATCTATGCTTCATAAGGATG
TTTTATATGACATTAATGTGTGCTTATATGGAAATGTATAATGCATGGTCTTTTATCAAAGAAGATGATCAAGTG
TCCGGTTCTGCAAGTAAGGCACCCAAGAAACATTCTTTTTCGAGGCACGAGACATCGTCCAGTAGTATCACAAGC
GGTGGAGGACCCGCAGCAAGTACGACCAGTACACATTGTAGCGGAAACATAAAATTACTGCCGAAAACAAGGAGC
ACAAGAACACCATCTGCATCTGCATTGCTTTCAAATAGTAATATTCTGACGGGTGATACCACTGCTGTTCCTCTT
TTATCACCAAATCTAAATGGTGCACACACCCATGGTCCAATTTTGGGACACCAAAATGCAATAAGCAATGGAAGT
TCTCAAACGAACATGAATGAAGTAAAAACTACTAGCGATACTATTCCAAGGCAACAGCTGCTTCAGCACAATAAA
TCCATCAGCGATTCGAAAAAAGAGTCACAGGCGCATGAACCTAAACAGCATCCGGTTATGACTTCTTCAATAATT
AACGCATCAAATAGTAATAACGTCTCGAATGTTAATATAACCCCACCACCGATGAATGGTGGTGGTGCCGCGAAC
AGTAGTGCTAATGTAGTGGAGACAAATATTGACATTCAACTGTATCAAACGTTGTCCACAGTAGTTAAAATGGTG
AGCGTTGTATATAACCAGTTAACTTCAGAGATATCCAAGATAGCCATTGCTAGCACCATGGGAAAGCAAATTTTG
ACGGACTCGCTAGCACCTAAAATTCGTGATTTGACGGAAACATGTCGTCAAGCGATGGATTTATCCAAACAATTG
AATGAAAGGTTAAATGTATTAATACCGAACGATTCAAATTCAGAGAAGTATCTGACATCTTTGGAGAAGCTGAAA
ACGTGGGAGATAATGAATTCGTTCTTAAAAGTAATAATATCAATTCTGGCTAATACTAAAATCGTAATGAGCGAC
GTGCCTAATTTGAATGAGCTGAGACCTAACCTAGCCAACTTGGCGAAGATTACCAAGGATGTCACTGTGATATTG
GACTTGAGTTCATACAAGGCTGTATCAGTAAGCGCCAACTCTCCGGAGTAA

>YBR169C 0.82 Cold
ATGAGCACTCCATTTGGCTTAGATTTAGGTAACAATAACTCAGTACTAGCAGTTGCCAGAAATAGGGGTATTGAT
GTCGTTGTCAATGAAGTTTCTAATAGGTCTACACCATCCTTGGTCGGCTTTGGCCCCAGAAATAGGTACTTAGGT
GAATCTGGTAAAACTAAGCAAACATCGAATGTTAAAAACACTGTGGAAAACTTGAAAAGAATCATTGGACTAAAG
TTCAAAGACCCTGAATTTGATATCGAGAATAAGTTCTTCACTTCGAAATTGGTACAGCTAAAAAATGGTAAAGTT
GGTGTGGAAGTGGAGTTCGGCGGTAAAACACACGTATTTTCAGCTACTCAACTGACTGCTATGTTCATTGATAAG
GTGAAGCACACCGTTCAAGAGGAAACGAAGTCATCAATTACCGATGTCTGCCTCGCAGTTCCTGTATGGTATTCG
GAAGAACAACGTTATAACATAGCCGATGCTGCCAGAATTGCAGGATTAAATCCTGTAAGGATTGTCAACGATGTG

ACTGCAGCCGCCGTTTCGTACGGCGTCTTCAAGAATGATCTGCCAGGTCCTGAAGAAAAGCCAAGAATCATTGGC
TTAGTGGACATTGGGCATTCTACCTACACCTGTTCTATTATGGCTTTCCGCAAAGGCGAAATGAAAGTATTAGGT
ACTGCTTATGACAAGCACTTTGGTGGTAGAGATTTCGATCGCGCAATCACAGAACATTTTGCTGATCAGTTTAAG
GACAAGTACAAGATTGACATTAGGAAAAATCCGAAAGCTTATAACAGAATTTTAATCGCTGCTGAAAAATTAAAA
AAAGTGCTTTCTGCGAACACTACTGCCCCCTTCTCCGTTGAATCTGTTATGGATGATATCGACGTTTCCTCTCAA
TTGAGCCGTGAAGAGCTGGAAGAATTAGTAGAGCCCTTGTTGAAGCGTGTGACGTATCCAATCACCAATGCATTG
GCTCAAGCTAAATTAACTGTCAATGATATTGACTTCGTAGAAATAATTGGTGGTACAACCCGTATCCCAGTTTTA
AAGAAGTCAATTTCTGATGTTTTTGGAAAACCTTTGTCATCTACTTTAAATCAAGACGAAGCTGTGGCCAAGGGG
GCCGCTTTCATATGTGCCATTCACTCTCCAACTTTAAGGGTCAGGCCGTTTAAATTTGAAGATATTGATCCGTAT
TCAGTGTCATACACTTGGGATAAGCAGGTCGATGACGAAGACCGTTTGGAAGTATTCCCTGCTAATTCATCATAT
CCATCAACTAAACTAATTACTTTACATCGTACTGGAGATTTCAGCATGAAAGCGGTGTACACTCATCCTTCGAAA
CTGCCAAAAGGTACTTCCACCACTATTGCAAAATGGAGCTTCACTGGGGTCAAGGTTCCTAAAGATCAAGATTTT
ATTCCTGTAAAGGTCAAGTTAAGATGCGATCCTTCCGGCTTGCATATTATCGAGAACGCTTACACAACGGAAGAT
ATTACGGTTCAAGAGCCAGTGCCTTTACCGGAAGACGCACCAGAAGATGCCGAGCCCCAGTTTAAAGAAGTTACT
AAAACAATTAAGAAAGATGTGCTAGGTATGACTGCAAAAACATTCGCGCTAAACCCGGTTGAGTTGAACGATCTA
ATTGAAAAAGAGAATGAATTAAGAAACCAGGATAAGTTAGTTGCCGAAACCGAGGATCGCAAAAATGCCCTTGAA
GAGTATATTTATACCCTTCGTGCCAAACTCGATGATGAATACTCCGATTTTGCGTCTGACGCAGAAAAAGAAAAG
CTAAAAAACATGTTAGCCACTACTGAAAATTGGTTATATGGTGATGGTGACGATTCTACCAAGGCAAAATACATT
GCTAAATATGAGGAGCTGGCATCGTTGGGGAATATTATTAGAGGTAGATATTTAGCAAAGGAGGAAGAAAAAAGA
CAAGCACTCAGAGCGAATCAAGAAACTTCTAAAATGAATGATATTGCTGAAAAATTGGCTGAGCAAAGAAGGGCA
CGCGCTGCAAGTGATGATAGCGATGACAACAATGATGAAAACATGGACCTTGATTAA

>YAR060C 0.8Cold
ATGTCAAAAACCGATGTCAAAAAATCTCGTGAGGCCTCCGGAATTTTGACGCTGCAAGTCAATCTACGGGAAAGA
AGAAATTTTTTAAACTTAATGCAAAATAAGCTTTTTTCTTGGAAAATAAGATTTTCGGCAATAAAAGGTAAATGC
AGCCAAAAATCAAAATACTTCAGAAGAAGTCGTAGCGAGGACTGCTACGTGGAAGCGGATTTGAAGATCCTTTCC
AGAACAAGAAGGAGCCGAAAGCTGCCAGGAACTGTTCCTGATTTTTTAGGAAAACAATTAATAGGTATCTCGTCT
AGCGTAGTATCTCGAGTTTCCAGAAGTTGCAGATAA

>YIL075C 0.75 Cold
ATGTCTTTGACGACTGCTGCTCCTCTTTTAGCATTGTTGAGAGAGAATCAAGATTCTGTTAAAACATATGCCCTA
GAATCTATAAATAATGTTGTGGACCAATTATGGTCTGAGATCTCGAATGAACTGCCGGATATTGAGGCATTGTAT
GATGACGATACCTTTTCAGACCGTGAAATGGCTGCTTTGATTGCATCCAAAGTTTATTACAATCTTGGTGAATAC
GAATCGGCTGTGAAGTATGCACTTGCCGCAAAGGATCGTTTTGATATAGACGAAAAGTCACAATTTGTTGAAACT
ATAGTCTCAAAGAGTATTGAGATGTACGTCCAAGAGGCTTCCAAGCAATACACTAAGGACGAACAATTTTACACC
AAGGATATTATTGACCCAAAATTAACCTCTATTTTTGAGCGAATGATAGAAAAATGTTTGAAAGCTTCAGAATTA
AAATTAGCTTTAGGAATTGCCTTAGAAGGTTACAGATTGGATATCATTGAAAGTGCTTTAAAGAGCAAATTGGAT
CAGGATTCCACTTCTGAAAACGTTAAAATTATTAACTACTTATTGACCTTAGCTATAACTACTGTGACAAATTCC
AAGTTCAGATCCTCGATATTGAGAAAATCATTTGATTTCCTAATGAATATGCCTAACTGCGATTACTTAACGCTC
AATAAAGTAGTTGTAAATTTGAATGACGCCGGATTGGCACTTCAGCTATTTAAAAAATTAAAGGAAGAGAACGAT
GAAGGATTATCTGCTCAAATTGCATTTGATTTGGTTTCGTCTGCGTCTCAGCAGTTGCTTGAGATTTTAGTAACC
GAACTAACTGCCCAGGGTTACGATCCTGCCTTATTGAATATTCTATCTGGGCTACCGACTTGTGATTATTACAAT
ACCTTCCTTTTGAATAATAAAAACATTGACATCGGTCTTTTAAACAAGTCTAAATCATCTTTGGATGGGAAATTT
TCTTTGTTCCACACCGCAGTCAGCGTTGCCAACGGTTTTATGCACGCTGGTACTACCGACAATTCATTTATCAAG
GCGAACTTGCCATGGTTAGGTAAGGCTCAAAATTGGGCTAAATTTACAGCTACAGCCTCTTTAGGTGTAATTCAT
AAAGGCAATTTATTGGAGGGTAAAAAAGTCATGGCCCCTTACTTGCCAGGCAGTCGAGCTTCTTCTAGGTTTATC
AAAGGTGGTTCATTGTATGGTTTGGGTTTAATTTATGCTGGCTTTGGTCGTGATACAACTGACTATCTGAAAAAC
ATAATAGTTGAGAATAGCGGAACTTCAGGCGATGAAGACGTAGATGTATTGTTACATGGTGCTTCACTGGGTATC
GGTCTTGCTGCTATGGGTTCTGCTAATATTGAAGTTTATGAAGCCTTAAAGGAAGTTCTGTACAACGATTCTGCC
ACATCTGGAGAAGCAGCTGCATTAGGTATGGGTCTCTGTATGCTCGGAACAGGTAAGCCTGAAGCGATTCACGAT
ATGTTTACATATTCCCAAGAAACGCAGCATGGCAATATTACTCGTGGTCTAGCCGTTGGTTTAGCACTGATAAAT
TACGGCCGTCAAGAGTTAGCTGATGATTTAATTACTAAGATGTTAGCAAGTGATGAGTCCTTGTTACGCTATGGA
GGTGCATTCACAATCGCGTTAGCGTATGCGGGTACGGGAAATAATTCAGCTGTCAAGAGATTACTGCATGTGGCA
GTCTCGGATTCTAACGATGATGTCAGAAGGGCAGCAGTCATCGCATTAGGGTTTGTTCTCTTGCGTGATTATACC
ACTGTACCAAGGATTGTCCAACTTCTATCTAAATCGCATAACGCTCATGTCAGATGTGGTACCGCATTTGCTCTT
GGGATTGCTTGTGCCGGCAAGGGTTTACAATCAGCTATTGACGTTTTAGATCCGCTAACAAAGGACCCAGTAGAT
TTTGTTCGTCAGGCTGCCATGATAGCTTTATCCATGATTTTGATTCAACAAACAGAAAAACTAAACCCTCAAGTT
GCTGATATTAACAAAAATTTTTTGAGCGTGATTACAAACAAACACCAAGAAGGTTTAGCCAAATTCGGTGCATGT
GTGGCCCAAGGTATAATGAATGCCGGTGGGCGTAATGTTACAATTCAGCTGGAAAATGCGGATACAGGCACATTG
GATACCAAGTCCGTAGTTGGTTTGGTCATGTTCTCACAATTCTGGTACTGGTTCCCACTGGCTCACTTTTTATCC
CTTTCTTTTACACCAACAACTGTCATTGGTATTCGTGGTAGCGATCAAGCTATACCCAAATTTCAAATGAATTGT
TACGCTAAAGAAGATGCATTCAGTTATCCGAGGATGTATGAGGAAGCAAGTGGTAAGGAGGTAGAGAAGGTAGCA
ACAGCTGTTCTTTCCACGACAGCAAGAGCAAAAGCAAGGGCAAAGAAGACAAAGAAGGAAAAGGGTCCAAATGAG
GAAGAAAAGAAAAAAGAGCATGAGGAAAAAGAAAAGGAAAGAGAGACGAATAAAAAAGGCATAAAAGAAACAAAA
GAAAACGACGAAGAATTTTACAAAAATAAGTACTCTTCTAAACCTTATAAAGTCGACAATATGACCCGTATACTA
CCACAACAATCGAGATATATTTCCTTTATTAAGGACGACAGATTTGTTCCTGTACGTAAATTCAAAGGAAATAAC

GGTGTGGTGGTTTTAAGAGATAGGGAACCCAAGGAACCTGTAGCACTAATTGAAACTGTTAGGCAAATGAAAGAT
GTTAACGCTCCACTTCCAACCCCATTTAAGGTCGATGATAATGTCGACTTCCCTAGTGCTTAG

>YPL190C 0.81 Cold
ATGTCAGATGAAAACCATAACAGTGATGTTCAAGATATTCCTTCACCTGAACTATCCGTCGATAGTAACTCTAAC
GAGAATGAATTGATGAATAACTCAAGCGCAGACGATGGAATCGAATTTGACGCCCCAGAGGAAGAAAGAGAAGCC
GAAAGGGAGGAGGAAAATGAAGAACAACACGAACTGGAAGATGTGAACGATGAAGAGGAGGAAGATAAGGAGGAA
AAAGGAGAGGAAAACGGGGAAGTAATAAACACAGAAGAAGAAGAAGAAGAAGAACATCAACAAAAAGGCGGAAAT
GATGATGACGATGATGATAATGAAGAGGAAGAAGAGGAAGAAGAGGATGATGACGATGATGATGATGACGACGAT
GATGATGAAGAAGAAGAAGAAGAAGAAGAAGAAGAAGAAGGCAACGACAACAGTTCGGTAGGCTCAGATAGTGCCGCT
GAAGACGGTGAGGATGAGGAAGACAAAAAGGATAAAACCAAAGATAAAGAGGTCGAACTTCGCCGTGAAACATTG
GAAAAAGAACAAAAGGACGTAGATGAAGCTATAAAAAAAAATAACTCGTGAAGAAAATGATAATACTCACTTTCCA
ACTAATATGGAAAATGTTAATTACGATCTTTTACAAAAGCAAGTCAAGTACATTATGGACAGTAACATGCTAAAT
TTGCCTCAGTTTCAACATTTACCTCAAGAAGAAAAGATGTCTGCGATTTTAGCAATGTTAAATTCAAATTCTGAC
ACAGCTCTTTCCGTACCTCCTCATGATAGTACTATCTCAACAACAGCTAGCGCCTCAGCCACAAGCGGCGCAAGA
AGCAATGATCAAAGAAAACCTCCATTGTCAGATGCCCAAAGACGTATGAGATTTCCTAGGGCGGATTTATCTAAG
CCGATTACCGAAGAAGAACACGACCGTTATGCAGCCTATTTGCACGGTGAAAATAAAATCACCGAGATGCACAAT
ATTCCTCCGAAGTCAAGATTATTCATTGGTAATTTGCCGCTAAAGAACGTTTCTAAGGAGGATTTATTTAGGATT
TTCTCTCCATACGGTCATATCATGCAAATCAATATCAAAAATGCCTTTGGATTCATTCAGTTTGACAACCCTCAA
AGCGTTAGAGATGCAATTGAATGCGAGTCTCAAGAAATGAACTTTGGCAAAAAGTTGATCCTGGAAGTTTCTAGC
TCGAATGCTCGTCCTCAATTTGATCATGGTGATCACGGTACAAACAGTAGTTCTACTTTTATTTCTTCCGCAAAA
CGACCATTTCAAACTGAATCTGGTGACATGTACAATGACGACAATGGTGCTGGCTACAAGAAATCCAGAAGACAC
ACCGTTTCTTGCAACATTTTCGTTAAAAGAACCGCAGATCGTACGTATGCCATTGAGGTTTTCAACAGGTTTAGG
GACGGGACTGGTTTGGAAACTGATATGATTTTCTTGAAACCAAGAATGGAACTGGGAAAGCTTATCAATGATGCC
GCATATAATGGGGTGTGGGGCGTTGTTTTAGTTAATAAAACACACAATGTAGATGTTCAAACTTTCTACAAAGGC
TCACAAGGTGAAACGAAATTTGATGAATATATTAGCATATCCGCTGATGACGCAGTTGCCATTTTTAATAACATC
AAAAACAACAGAAATAATTCTCGTCCTACTGATTACCGTGCTATGAGCCATCAGCAAAACATATATGGCGCTCCT
CCTCTTCCTGTTCCAAACGGCCCAGCTGTCGGACCTCCTCCTCAAACAAACTATTACCAGGGTTACAGTATGCCT
CCTCCACAACAACAACAGCAACAGCCATATGGTAATTATGGGATGCCACCACCATCCCATGACCAAGGATATGGT
TCTCAACCTCCAATTCCAATGAATCAGAGCTACGGTCGCTACCAGACTTCTATTCCACCACCACCTCCACAACAA
CAAATTCCTCAAGGGTATGGTCGTTATCAGGCTGGTCCGCCTCCTCAACCACCTTCTCAAACTCCAATGGACCAG
CAACAACTATTATCTGCCATTCAAAACCTTCCACCTAACGTTGTATCGAATTTGCTTTCAATGGCCCAACAACAG
CAACAACAACCTCATGCTCAGCAGCAATTGGTTGGTTTAATACAATCAATGCAAGGCCAGGCTCCTCAACAACAG
CAACAACAGTTGGGTGGATATTCCTCTATGAACTCATCCTCTCCCCCTCCTATGAGTACCAATTACAATGGTCAA
AATATATCTGCAAAACCCTCTGCCCCACCAATGTCACACCAACCTCCGCCACCTCAACAACAACAACAACAACAA
CAACAGCAACAGCAACAGCAACAGCAACCTGCTGGCAATAATGTTCAAAGTCTATTAGATAGTTTAGCAAAACTA
CAAAAATAG

>YMR309C 0.79 Cold
ATGTCCCGTTTCTTTTCGTCTAATTACGAATACGATGTAGCCAGTTCTTCATCCGAAGAAGATCTTTTATCTTCG
TCTGAAGAAGATTTGTTAAGCTCTTCCTCCTCTGAGTCTGAATTGGACCAAGAATCTGACGACTCCTTTTTCAAT
GAAAGTGAAAGTGAAAGTGAAGCTGATGTAGACTCTGATGATTCTGATGCAAAGCCTTATGGTCCTGACTGGTTC
AAGAAATCTGAGTTCAGAAAACAAGGTGGAGGTTCAAATAAATTTTTGAAAAGCTCTAACTATGATTCCAGTGAT
GAAGAATCCGATGAAGAAGATGGCAAGAAGGTAGTCAAGTCTGCCAAAGAAAAACTATTGGATGAAATGCAAGAC
GTTTATAATAAGATCTCTCAAGCTGAGAACTCTGATGACTGGTTGACTATTTCTAATGAGTTTGATTTGATCTCG
CGTCTCTTAGTTAGGGCTCAACAACAAAACTGGGGGACTCCAAATATTTTCATCAAGGTTGTTGCCCAAGTGGAG
GACGCTGTGAATAATACACAACAAGCTGATTTGAAGAATAAAGCTGTTGCAAGAGCTTATAACACTACAAAGCAA
AGAGTCAAGAAAGTTTCTAGAGAAAATGAAGACTCAATGGCTAAATTCAGAAATGATCCTGAATCATTTGATAAG
GAACCAACCGCAGATTTGGATATTTCTGCTAATGGATTCACAATTTCTTCGTCTCAAGGCAATGACCAAGCGGTA
CAAGAAGATTTCTTCACTAGATTACAAACAATAATTGACTCAAGAGGTAAGAAGACTGTCAATCAACAATCCTTG
ATTTCTACTTTGGAGGAGTTATTAACTGTAGCTGAAAAACCATATGAATTCATAATGGCTTATTTGACTTTGATT
CCATCAAGATTCGATGCCTCAGCTAACCTATCTTACCAACCAATTGATCAATGGAAATCTTCATTCAACGATATT
AGTAAATTATTGTCTATTTTAGACCAGACAATTGACACCTACCAAGTTAATGAATTTGCTGATCCAATCGATTTC
ATTGAAGATGAACCTAAAGAAGATTCTGATGGTGTCAAGAGGATTCTGGGTTCCATTTTCTCATTTGTTGAAAGA
TTAGATGACGAATTCATGAAATCCCTGTTAAACATCGATCCTCATTCCAGTGATTATTTGATCCGTTTAAGGGAT
GAACAATCAATCTATAATTTGATCCTAAGAACTCAATTGTACTTTGAAGCGACTTTGAAAGATGAACACGACCTA
GAAAGAGCATTGACACGTCCATTCGTCAAGAGATTGGATCATATCTACTATAAATCCGAAAATTTGATAAAAATT
ATGGAAACTGCTGCTTGGAATATCATACCTGCTCAATTCAAATCTAAATTTACTTCAAAAGACCAGCTCGATTCT
GCTGATTATGTCGACAATTTAATAGACGGATTATCGACAATCTTATCCAAGCAAAACAACATTGCTGTTCAAAAA
CGTGCTATTTTATACAACATTTACTACACTGCATTAAACAAAGATTTCCAAACTGCTAAAGATATGTTACTAACT
TCCCAAGTTCAAACAAATATCAACCAATTCGATTCATCCCTACAAATTTTATTCAACAGGGTTGTTGTTCAATTG
GGTCTATCCGCCTTTAAATTATGTTTGATTGAAGAATGTCATCAAATTTTGAATGATCTTCTGTCAAGTTCTCAC
TTAAGAGAAATTTTGGGCCAACAATCCCTACACAGAATATCTCTCAATTCTAGTAACAATGCTTCAGCTGATGAG
CGTGCTAGACAATGTTTGCCATATCACCAACACATCAATCTCGATTTAATCGATGTCGTCTTCTTAACATGTTCC
TTATTGATCGAAATTCCAAGAATGACTGCCTTCTATTCCGGTATTAAGGTCAAGAGAATTCCTTACTCTCCAAAA
TCCATTCGTCGTTCCTTAGAACATTACGACAAGTTAAGTTTCCAAGGTCCACCAGAAACTTTAAGAGATTATGTC
TTGTTTGCTGCCAAATCAATGCAAAAAGGTAACTGGAGAGACTCTGTTAAATACTTAAGAGAAATAAAATCTTGG

GCTTTATTACCAAACATGGAAACGGTGTTGAATAGTTTAACGGAAAGAGTACAAGTTGAATCTTTGAAGACTTAT
TTCTTTTCTTTCAAGAGGTTCTATTCAAGTTTTTCTGTTGCTAAACTAGCCGAATTATTTGATCTTCCAGAAAAT
AAGGTGGTTGAAGTTTTGCAATCTGTTATCGCAGAATTGGAAATCCCAGCCAAATTAAACGACGAGAAGACCATC
TTTGTTGTCGAAAAGGGTGATGAAATTACTAAATTGGAAGAAGCAATGGTAAAATTGAACAAAGAATATAAAATC
GCTAAAGAACGTCTTAACCCACCATCAAATCGTCGTTGA

>YMR187C 0.75   Cold
ATGACCCCACCTCATTTTTTTTTATCTCTCATAAAAAAAAGATGCATATGCTGGATATGTTTGGAAGAATCCACA
TATGACTCTACATGGCTACAGCACACGTGCGGTTGCAACTTACAAATTCATAAACGCTGCTATATAAGATGGCTC
TATCAAATGCATGTTGAACTTTTTCTTCCGAACACAGTGGACCTACCAAAGGATGCTGATTTACCTATAATCACC
TGTTTAAAATGTCTCGTTGATGGGCACCATGATTTTATGACGACTTTCTCTCTTACAGAAATATGGGAAACTCGT
CCCATTTGGGGGCAAAAATCAGTACCGTTTCAAAATGACTACGTGTTCAATCTAATGTCTCTTTATACCAAACGG
GATAATCATCCACCTTATGTGTTAGTGAAATTTGGCGAATGTCCTCAATGCAAGAAAACAAATTTCATAAAAAGA
CCTACAGTAACCATCCAGTCTTCTGTACTATCCCTGTTTTATCAGTGGCAAAAAATTACACGATACGTGATACCA
TTAGGGATAACTAGTTTATTTCTTTTAAACCCTGAAAAGACAAGTTTTGACATTGGATTATGGCAACTACGCTGC
CTTTTCCCTGAGAATGTTCTACGAAATATGTTAAACATCAGCACTACAAAGGCATTGGACGTATATGCGCAAACT
GAACGTGGCTTATTAAGCATTCCTTTAACCTCCTCGATCATAATATATGGGTTCATACATTATCTTAGCAACATT
TCTAACGTCAGTGCTAATGCAATACTCTTTAAGTGGGTGTATCTCAGTATTGTAAAAACTGCTGGAAACAAATAT
TACAAGGGTATAGGTTTACCTAAAATAATTTTGTATTCTAACTTGGCTACATTTTGCTATAATTTTACCTTTAAA
AGATTGGTTGACTTAATTTACAGGAGGCTGATCAATAAAGGAGGTAAGTATTTGTATCACGGAAATTTTGAAAAC
TCTAGTAATAGCGTTCCAGCTGAGGAATTTTTTATCAGGCGGAATTGGTATGCTATTCTTGCAGAAAAGATACTA
TGGCCCTTTGTTGGTAAATGTACAGGAGGTCTTCTATTGAATGCTTTTCTATGGATACAGAGAAAGTTTAAAATT
GAATGGACGCCGAATTGCTCCCCTTCGGAATTTAGAATGATTTTCAATATTATAGGTTGTGGTACTGCTGCTATA
GGCTGGTCAAGTCTGAAGCTCTATGCCAGTTACAAAAGATGTCAGGAACTTGAAAAGATCAATGAATTTATTGAA
CAAAGTTGCAAAGGTGAGTAA

>YLR125W 0.78   Cold
ATGGGAGAAATTTTAGAACTAACTAATAAGAATTTCATGAGCCACTTGAAAAAGGACATAACTTCGCAAGAATCC
TTGAAAAGTAGAATTGAGGATAAAAATGGTGATGTCGCTAGCCCGAAAGAGGATAACTATCCTCTATTGAATGAA
ACAGCGGCATGGCCAGATGGGGTGATTACCTCTGAAGAAGGATGTTCATCTTCAGGTGAGAAAGAGAATTCAGGC
TTATGTTCAGAAGAAAGTTCAGAAGAAGATCCAGAAGAAGCTGAAGAGGAATCTGCAAGGGCTTTTGGAGAGTTA
GTTGCAGTCTTGCGGGACAAAGACATACCACTGAATGTATTGGACGAACCTCAGATGAAAGATTGGCTTGAAAAA
TACACCGGTGTTTATAGAAGCTCTTGGCATGGTTGA

>YPL003W 0.82   Cold
ATGGAAAGATATGATAGACAGCTGAGACTATGGGGAGCATTGGGCCAAGATAGTTTGAACCGGTCCCGTGTGTGC
GTGGTAGGACCTGCTACACCTTTGCTGCAGGAAGTTTTTAAGAATCTGGTGCTTGCCGGAATATCATCTCTTACT
TGGCTCAAAGTAGAGTGTGCAGTTCAATCGGGTTCTCTGTTTTTAGCAGAGCTTAAAAAAGATCTTGAGCCATTG
GCTTCTAAACAGCTAGAATATGAGGAAAATGACCTTAGAAAGACGTTACAGCAACCTCAGTATGATTGGACCCGC
TTCTCGGTGGTGATCTTGACTTGTATTGGTGAGCAAACTGCGATGCTCGATTTAAATGAGATTAGGCGGCAGAGA
GGAACAAAGTTTCCGCCTGTTCTAAACACTTTCGTCTCTGGATTTTATGGATATATATATCTGGTACTCTCAGAA
ACACATTTTGTTTTGCAAGCACACCCAGATAGTAAGAAGTATGATTTACGGTTACAGAACCCTTGGCCTGAACTA
ATAAATTACGTAGATACATTTGACCTAAGTAAAATGGATACAGCCACGTTTTCTGGTATTCCATATACTGTTTTG
CTCATGAAGTGTATTGCAAAACTTGAAAGGGATGGTAATAATGGGAGGATAACGATTGATCAGATGAAGAAAGTT
TTAGACCAAATTTGCCTTCCTTTAGGGAATGATGTTATCTACGAACCCAATTATGTGGAAGCTAAACGCTATGCT
TACTTGGCCTGCTCTCAAAATGATTGTTGTAAAGAACTGGAAGATCTGCTCCGAAATTTGGAAATATCAGATTAT
GGGAACGATTGGCATGATACTTATAACTACGAGATTCTCACACTATTATTGACTTTGAAAAATATTGCTAAAGAG
AATGGGGAGCTCAGCTTTCAACCTTTAACGGGCACTCTCCCAGATATGGAGTCAACAACGGAAAATTATATCAGA
CTCAAGAAACTGTATGAAGTAAAAGCAAAGCTTGACAAGTCTCGTGTGGAGGAAAGTTTGGCTCGCAGTAAAAAA
ATAGTTTCACAGGATGTCTTGGAGACATTTTGTTCTCATTACGGGGAGGTCAGGAAAATATTGCCGCCAAAAAGT
GACTTGTTAGGTATTTTTAGCACCTCGAATGCCCTTTTAGATGCATTAGTTATGGTCCAATTCTGGGAGCAACCG
GCAGTAACTGCAGAGGATAAAGATGAGTTTATTGGTTTACGAGTCGACGATAACTATTCCGTAATGGCCTTTTTT
GGAGGTGCAGTGGTTCAAGAGGCTATAAAGCTTATCACACACCATTACGTTCCTATTGATAACCTATTTTTATAT
AATGGCATTAATAATTCATCCGCAACATATAAAATATAA

>YDR028C 0.65   Cold
ATGTCAACAAATCTAGCAAATTACTTCGCCGGTAAGAAAGATATTGAAAACGAGCATGTAAATAGAAATGCGAGC
CATGAAAGTAATAGCAAAAGTGATGTCAAAATTAGTGGTAACGATAACGACAACGACGAAGATATGGGGCCTTCA
GTATCGATGGCTGTTCAAGCGAAAAATGATGATGATTTCCATAAATCAACTTTCAACCTTAAAAGAACAAGATCA
ATGGGCCTTCTTGATGAATATATTGACCCTACCAAGAAATTGCTAGGAAGGTCAGACGACTTATATGATAACGAC
AATGAATATTATGACAACTCATCTAATAATTCTTCAAGCAATTCTTCAGATGATGATTATGATGACGGCTATCAG
GAACACTCAACCTCCGTTTCTCCACCGCCGGCGGATAATGATAGCTACTTAATTCCACAAGATGATAATGACGTA
GTGGTAGAGCCAGAAAGACATGTTGACTATCTGTCACATGAATGGAAAGAATCAGAAATTTCTAATTCTTGGAAA
TATATCATCCTAAAGAAAAAGAAAAGAGATGTTGATCTAGTAAATGCTGCTAGGTTAGAAAATGCCTCATGGAGA
ACATGGGCAAAAGCAAGAAACAACTTGAAAACAGTGTCACCTGAAGTGGTTAATTGGTCAAAAGATTCTGATGTG
ACTTGGCTATATGGCCCTATTGTAAGAGATTCTGAAGGTAATGCCCAAAGTGAAGAAGAACATGATCTAGAAAGA
GGATATGGTTCGGATGATGAAAATTCCAAGAGAATTTCAATGCCCACTAAAAATTCAAAGTCGATCGCAGCTGCT

CCGAAACCAATTTTGAAGAAGAGAACCGTCACAGAAATTATAGAGGACAACGCTTTATGGAAACTGAATGAGGCA
AGAAAACACATGACAGAAATGAAACATGCTTCGGTAATAATGGATCCCAATGGCAACAAAAACGTCCATGACGAT
TTCGATGCTCTGGCCGCTCAAGTTAACGCCCAGTACTACCATTATCCCAAAGAATCGAACAGTAGCGTTAGTTTG
AAGAGTCAACACTCTGACAAAAAAGATAATTCTACCATACCGAATCCTGTAGGAGAAAATTCAAATGGTGGCGGT
GACAAGGGAGAAGAAGACCTGCATTTAAAGTCAGCGCTTCATGTTCAAAACAATAGGTCAACGGCACAATCAAAC
AAAAGCATACTAGAAAATAGTACCAATGATCGTAAGGCTAACTTGGATCAAAACCTAAATTCTCCTGATAATAAT
AGGTTCCCCTCCTCAACATCTTCCTCCAATAGAGATAATGAAAACAATTCCATGGGATTATCATCAATATTAACC
TCAAACCCAAGTGAAAAATCTAACAAACCTACTAAAAATAGACATATACATTTTAATGACAGGGTGGAACAATGT
ATGGCACTACGATATCCAGCTTCACAATCGGAGGATGATGAAAGCGATGATGAAAATAAACAATATGTTGATGTT
AATAACAATGCGAACGTTACAACAATAAACAACAATAGGACTCCATTGCTAGCCATTCAGCATAAATCTATTCCT
ATCAACTCCGCAACTGAACATTTGAACAAAAATACTAGCGACGATGATACATCCTCACAATCATCATCTTCATCA
CATTCTGATGATGAAGAACACGGCGGCCTTTACATAAATGCAAGATTTTCCAGGAGGTCTGACTCTGGAGTTCAT
TCACCAATAACAGATAATTCCTCTGTGGCTTCATCTACTACTTCCAGAGCTCATGTACGTCCCATAATAAAGTTG
CTTCCTGATACCACTTTGAATTATGGATCAGACGAAGAATCTGATAATGGCGAATTTAATGGGTATGGTAATGCT
GTGTCACATAACGTCAATACCTCCAGAGGCTATGATTACATATACGATTATAACTCGGTCTACACTGGTGATACT
TCCAGTTTTTTACCAGTAGATTCATGTGACATTGTAGATGTTCCTGAAGGAATGGACTTACAAACAGCCATTGCA
GATGATAATGCATCAAACTATGAATTTAACAATGCGGTAGAATCCAAGGAAAAACATGTTCCACAACTACACAAA
GCTTCAGCAAATAATACAACACGTCAACATGGATCGCATATGTTATTATATGACGACGACAATTACAGCTCTTCA
TCAGATAGCGAACAGCAATTTATTGAAGACTCACAATACAATAGTAGCGACGACGAAGAGGAAGAAGATGACGAT
GACCAAGAAGTAGACGATAACCACGATGAAGGACTTTCACTGAGGAGAACATTGTCACTTGGGAAATCAGGATCT
ACCAATTCTTTGTACGATCTAGCACAACCATCACTCTCTTCGGCAACTCCTCAACAAAAAAATCCTACTAATTTT
ACAGGGGGTAAAACTGATGTAGACAAAGATGCACAACTGGCTGTCAGGCCGTATCCGTTGAAGCGCAATTCCTCT
TCAGGGAACTTCATATTCAATTCAGATAGTGAAGAAGAGAGTAGCAGCGAGGAGGAACAAAGACCATTACCCGCT
AACAGTCAGCTGGTTAATCGAAGCGTATTGAAAGGCAGTGTGACACCAGCAAACATATCATCTCAGAAGAAAAAA
GCCCTTCCAAAGCAGCCAAAAGCGTCCGATAGTTCACAAAGCTTTAGAATTGTAAATAATACTCCTTCACCAGCC
GAAGTGGGGGCGAGTGATGTTGCCATAGAGGGTTATTTCTCTCCTAGAAATGAATCTATCAAGTCTGTTGTTTCC
GGGGGAAATATGATGGACCATCAAGATCACTCAGAAATGGACACTTTGGCAAAAGGATTTGAAAACTGCCATATA
AATAATGCTAGTAAGCTAAAGGACAAGAAAGTTGATAGCGTGCAAACCACTAGAAAAGAAGCGTCTTTGACGGAT
TCGAGTAATGAAAGCTTACACAAAGTGGTGCAGAATGCAAGAGGTATGGCAAGCAAGTACTTGCACTCTTGGAAA
AAGAGTGACGTCAAGCCACAAGAAAATGGAAATGACAGCAGTTAG

>YJL002C 0.82 Cold
ATGAGGCAGGTTTGGTTCTCTTGGATTGTGGGATTGTTCCTATGTTTTTTCAACGTGTCTTCTGCTGCCCAATAC
GAGCCACCTGCGACTTGGGAGAATGTTGATTATAAGAGGACAATAGACGTGTCAAACGCTTATATTTCAGAAACA
ATCGAAATAACTATCAAAAACATAGCAAGCGAACCTGCGACTGAATACTTCACAGCCTTTGAGAGTGGCATCTTC
AGTAAAGTTTCCTTTTTTTCAGCCTATTTTACCAACGAGGCAACTTTTTTAAATAGCCAATTACTTGCCAATTCG
ACTACAGCACCTGGTGACGATGGTGAAAGTGAAATTAGATACGGGATCATTCAATTTCCAAATGCAATTTCCCCT
CAGGAAGAAGTTTCTTTAGTGATTAAGAGCTTCTATAATACCGTAGGTATTCCTTATCCTGAGCACGTTGGAATG
TCAGAAGAACAACACCTATTGTGGGAAACGAACAGATTGCCGCTTTCTGCTTACGATACCAAGAAGGCCTCTTTT
ACGCTGATTGGTAGCTCATCATTTGAGGAGTACCACCCCCCAAATGACGAGAGTTTACTGGGAAAAGCTAATGGA
AACTCTTTTGAGTTTGGGCCTTGGGAAGATATTCCGAGATTTTCTTCAAACGAAACCTTAGCAATTGTTTATTCC
CACAATGCCCCATTGAATCAGGTAGTGAATTTGAGAAGAGATATTTGGCTTTCTCATTGGGCTTCCACAATACAA
TTTGAGGAATATTATGAATTAACAAACAAAGCCGCAAAACTGTCTAAAGGATTTTCAAGATTAGAATTAATGAAA
CAGATTCAAACTCAGAATATGAGACAAACTCACTTTGTTACTGTCTTAGACATGCTCCTGCCTGAGGGAGCTACT
GATCATTATTTCACTGATTTGGTTGGCCTTGTTTCCACGTCGCATGCAGAACGTGACCATTTCTTTATAAGACCA
AGATTCCCAATCTTTGGAGGTTGGAACTACAATTTTACTGTCGGTTGGACTAATAAATTGTCCGATTTCTTGCAT
GTATCCTCTGGCTCAGACGAGAAATTCGTTGCTTCTATCCCAATTCTAAACGGCCCACCGGACACTGTATATGAT
AATGTTGAATTATCGGTATTTCTTCCGGAAGGGGCCGAAATATTCGATATTGATTCTCCAGTCCCTTTTACAAAT
GTTTCTATAGAAACCCAGAAATCATACTTTGACCTAAATAAAGGTCACGTTAAATTAACTTTCAGTTACAGAAAT
TTGATTAGTCAAGTTGCCAATGGCCAAGTCTTGATAAAGTACGACTACCCGAAAAGCTCTTTTTTCAAGAAGCCT
CTGTCTATTGCTTGCTATATTTTCACCGCACTAATGGGAGTTTTTGTCTTAAAAACTTTGAACATGAACGTAACT
AACTGA

>YJL009W 0.77 Cold
ATGATACAGCCTAGCAAGTTTGAAAACTTGATTGCAATTTATGACATTATATTCTGCTGTTTGCAGGTATATCTA
CTTTGTAAGTATAAAGATGCAAACAAATCAGTCTTCTTGGTCCCAGTTACCTGGCCTTGGGCCTTGAGGTGCTCT
TGGACCACCCGCCTTCTTTGCCATGATAATTTGGTCAATGGAAAGCACAGTAGTTGCAGCTTCTGTAGCGACGTT
AATAGCAAACTTTTTCGTTGCAAGCATGTCATAAATATTTTCCTCTCTGATGTCCTTTACACCTTCATCAGATTC
GCCATCAATATCCACACCTTTATATAA

>YOL137W 0.78 Cold
ATGGATGCTTCCTCGGTTCCACCAAAGGTAGATGATTATGGAATGTATACAACTGAAATTTCACATCATAACCCA
ATAGAGTTGAAAAATCTGCTATCATCATCAGACTCCAGAAGAAATTCTCAAGATGAAGATAGTCTACCTAACAAT
ACAAACTTAATCAAAGAAATTGACTGGCAGGGCGAGAAAGTTAAAACATATCCACTAAACTATCAAACTGTACCA
CTAGTAAAGCTGCAGGTGATAGCATGCTTGATTATGTTCGTAGTCTTTGGTATGAACGATCAAACAGTAGGTGCA
CTACTTCCTACGCTAATTGAATACTATCATATATCGCGGGTAGATGTCTCAAACGTATTTATAGTCCAATTATGT
GGTTATGTGATGGCGTCTTTATCAAAAGAGAGATTGAATAAGCACTTTGGTATGAGAGGTGGTATGCTTCTAGCA

GCTGGTTTGTGTATAGTATTTCTTATCATTTTAGCAACTGCACCTTCTAGTTTCTATGTTTGTATGTTTTGTGGC
CTTCCTCTTGGTTTAGGGATCGGCATCTTAGATTCTACCGGTAATGTTTTAATGGGCAGTCTTTTAGTCCATAAG
AATGAACTCATGGGTATCATGCATGGTCTTTACGGGGCAGCAGCTATGGTTACTCCTCCCTTAGTTTCATATTTT
GTTGAATGGGGTCATTGGTCTCTTTTTTTCCTTATTCCTCTGTTCTTTTCTATAATAGGCATGATTGTAATCTTC
CCAGCTTTTAAATTTGAAACTGCAAGTAAATACGACTACCTCTGCTCTGTGGAAAACAAAGAAAGCAACAATGAT
GTGGAAGAAGCGGGTGATAACTCGCTGATGGAATCTACCAAGGCGAGCCCAGGATTTTTTGAACTTTTAAGAAAT
CCTGCTATTTTCTTGTACTCATTGTATTTGTTCCTTTACTTAGGTGCTGAAATTACAACTGGTTCATGGTTCTTT
AGTTATTTATTGGAAACTAAATCAAGTAATAAGGTTGCTATGTCATACATAGCGGCATCGTTTTGGACAGGTTTG
ACTGTAGGCAGGTTGTGTCTAGGATTTGTTACTGAAAGATTCTTCGAAAACGAGTATAAAGCAAGCAAAGCGTAC
GCTTTTTTAACTCTATCTTCATACACCTTATTTGTGCTTGTTGGGTTGATCAATTCAAGCTCAGTTTTCTATTTC
GTCGTGTTATTTTTTGTTGTTTTTTGTTGTGGTACGTTTATCGGACCATTATTCCCAAACGCAAGTATAGTTGCA
TTACAAGTATTGCCCAAAAGACTACATGTGAGCGGGGTTGGGGTTGCCGTCGCCGTTGGTGGTTGTGGTGGTGCA
GCCATTCCATATTTGGCTGGAGTTATTGCACACACAGTGGGAATCCAGTATATCCCACTATTGTGTTGGATTATG
GTTGCGTTATTTACATTGGAATGGACATTGTATCCTAAATTCATAAAAGGGCATGAAGAATATTTTTAG

>YBL022C 0.81 Cold
ATGCTAAGAACAAGAACCACAAAGACCCTTAGCACAGTGGCGAGGACCACAAGAGCTATCCAATATTACCGATCT
ATCGCTAAGACTGCTGCAGTTTCTCAAAGAAGGTTCGCATCGACCTTGACCGTGCGTGACGTAGAGAATATCAAA
CCTAGCCATATTATTAAGAGTCCAACATGGCAAGAGTTCCAGCATCAATTGAAGGATCCAAGGTACATGGAACAC
TTTGCACAACTTGATGCACAGTTTGCTCGGCATTTCATGGCTACAAACAGCGGCAAAAGTATTCTGGCGAAAGAT
GATAGTACATCTCAGAAAAAGGATGAAGATGTCAAGATAGTACCTGATGAAAAAGATACAGACAATGATGTTGAG
CCCACGCGAGATGATGAAATTGTTAATAAGGACCAAGAAGGTGAAGCTTCGAAAAACTCCAGAAGCTCCGCTTCT
GGAGGTGGACAGTCATCATCCAGTCGATCGGATTCTGGAGATGGAAGTTCGAAACAAAAGCCTCCAAAGGATGTA
CCAGAAGTATACCCGCAAATGCTAGCATTACCAATAGCCAGACGGCCCTTATTTCCAGGGTTCTACAAGGCGGTT
GTGATATCGGACGAAAGAGTTATGAAGGCAATCAAGGAAATGTTAGACCGCCAGCAACCCTACATTGGCGCTTTC
ATGTTGAAGAACTCGGAAGAAGATACTGATGTAATCACAGATAAAAATGATGTATACGATGTTGGTGTTCTGGCT
CAAATAACTAGCGCTTTCCCGAGTAAAGACGAAAAGACGGGCACTGAAACAATGACTGCTCTTTTATATCCTCAC
AGGAGAATAAAAATTGATGAATTATTTCCACCAAACGAGGAAAAGGAAAAATCAAAAGAACAAGCAAAGGATACG
GATACTGAAACGACCGTCGTTGAAGACGCTAACAACCCAGAAGACCAAGAAAGCACCAGCCCTGCTACGCCTAAG
TTGGAGGATATTGTGGTGGAGAGGATACCAGACTCAGAATTGCAACATCATAAGAGAGTAGAAGCCACTGAAGAA
GAGTCTGAAGAATTAGACGATATTCAAGAAGGTGAGGACATTAACCCCACAGAATTCTTAAAAAATTATAATGTT
TCCCTTGTCAATGTGTTGAATTTAGAAGATGAACCGTTTGATAGAAAGTCACCCGTTATTAATGCGCTAACATCA
GAAATATTGAAGGTATTTAAAGAAATTTCTCAATTGAACACGATGTTCAGAGAACAAATTGCAACTTTTTCTGCT
TCAATTCAATCTGCGACCACGAACATTTTTGAGGAACCAGCTAGATTGGCAGATTTTGCCGCTGCTGTTTCTGCC
GGCGAAGAAGACGAATTACAAGACATACTGTCATCTTTAAACATTGAGCACAGACTAGAAAAGTCGCTACTAGTC
CTTAAGAAAGAATTGATGAATGCCGAATTACAGAACAAAATCTCCAAAGATGTAGAGACAAAGATTCAAAAGAGA
CAAAGAGAATACTATTTGATGGAACAATTAAAGGGCATCAAAAGAGAATTAGGTATTGACGACGGCCGTGACAAA
TTGATTGACACTTATAAAGAAAGAATCAAGTCATTAAAATTGCCTGATAGCGTTCAAAAAATTTTCGATGATGAG
ATAACTAAATTAAGTACCTTGGAGACTTCTATGTCCGAATTTGGCGTTATCAGAAACTATTTGGATTGGTTAACA
TCGATCCCATGGGGAAAACATTCCAAAGAACAGTATTCCATTCCGAGAGCAAAGAAAATTTTAGATGAAGATCAT
TATGGTATGGTAGATGTCAAGGATCGTATCTTGGAGTTTATTGCCGTCGGTAAACTATTGGGTAAAGTCGATGGT
AAAATCATATGTTTCGTAGGACCCCCAGGTGTTGGTAAAACGTCCATAGGTAAATCTATCGCTAGAGCTCTTAAC
AGGAAATTCTTCCGATTTTCTGTAGGCGGTATGACGGACGTGGCAGAAATAAAAGGTCATAGGAGAACGTATATT
GGTGCCTTACCTGGCAGAGTAGTTCAAGCATTAAAGAAGTGCCAAACTCAAAATCCTTTGATTTTGATAGATGAA
ATCGATAAGATTGGACACGGCGGCATACACGGCGATCCGTCCGCGGCATTGTTAGAAGTATTGGACCCTGAACAA
AACAACAGCTTCTTGGATAACTACTTGGATATTCCTATTGACTTATCAAAAGTGCTTTTCGTTTGCACTGCTAAC
TCTTTGGAAACTATTCCAAGACCTTTACTGGATCGTATGGAAGTCATTGAGTTGACAGGATATGTTGCTGAAGAC
AAGGTTAAGATTGCCGAGCAGTATTTAGTTCCAAGTGCCAAGAAAAGCGCTGGTCTAGAAAACTCTCATGTTGAT
ATGACAGAAGATGCAATAACAGCGTTAATGAAGTACTACTGTAGAGAAAGTGGTGTAAGGAATTTGAAAAAACAC
ATCGAGAAAATATACCGTAAAGCAGCCCTACAAGTGGTAAAGAAGTTGAGTATTGAAGATTCCCCAACGTCATCA
GCCGACAGCAAGCCTAAAGAAAGCGTTTCTTCCGAAGAAAAGGCGGAAAATAACGCCAAAAGCTCATCGGAGAAG
ACTAAGGACAATAATTCGGAAAAGACAAGCGATGACATTGAAGCCTTAAAGACCTCTGAAAAGATCAATGTTTCC
ATATCCCAGAAAAACCTAAAAGATTACGTTGGGCCTCCAGTCTACACTACAGACCGGTTATACGAAACAACTCCA
CCAGGGGTAGTTATGGGGTTGGCGTGGACTAATATGGGAGGTTGCTCACTTTATGTAGAATCTGTCCTTGAACAA
CCTTTACACAACTGCAAGCATCCAACATTTGAAAGGACCGGGCAACTGGGAGATGTTATGAAGGAATCATCAAGG
CTTGCTTATTCATTCGCCAAGATGTATTTAGCACAAAAATTCCCTGAGAACAGATTTTTTGAAAAGGCGTCTATC
CACTTGCATTGTCCTGAAGGTGCTACCCCTAAAGATGGTCCATCTGCAGGTGTCACTATGGCCACTTCCTTCCTT
TCCCTAGCATTGAATAAGTCGATTGACCCAACGGTTGCGATGACCGGTGAATTGACACTTACCGGTAAAGTTTTA
CGTATCGGTGGGTTGAGAGAAAAAGCTGTGGCCGCTAAAAGATCTGGTGCCAAGACTATTATTTTCCCTAAAGAT
AACTTAAATGATTGGGAGGAACTTCCAGACAATGTAAAGGAAGGGTTGGAACCTTTAGCTGCTGATTGGTATAAT
GATATATTCCAAAAATTATTCAAGGATGTGAATACGAAAGAAGGTAATTCAGTATGGAAGGCTGAATTTGAGATT
TTGGATGCTAAAAAGGAAAAGGACTAA

>YPL151C 0.79 Cold
ATGGACGGAAATGATCACAAAGTCGAAAATTTAGGAGATGTAGACAAATTTTATTCCAGAATACGCTGGAATAAC
CAATTTTCATATATGGCCACTCTGCCGCCCCACCTACAAAGCGAAATGGAAGGTCAGAAATCATTGCTAATGCGA
TATGATACTTATAGGAAGGAAAGTTCTTCTTTTAGTGGTGAAGGCAAGAAAGTTACTCTGCAGCATGTTCCTACA

GATTTTTCAGAAGCATCACAAGCAGTGATTAGCAAGAAAGATCACGATACGCATGCATCTGCTTTTGTGAATAAA
ATTTTCCAACCAGAGGTTGCTGAAGAACTTATAGTTAATCGATACGAAAAACTTCTGTCACAAAGGCCGGAATGG
CATGCACCCTGGAAACTTTCACGCGTTATCAATGGCCATCTTGGATGGGTACGATGCGTTGCAATCGATCCTGTT
GACAACGAATGGTTCATCACCGGAAGTAATGATACGACAATGAAAGTTTGGGATCTTGCAACAGGAAAATTAAAA
ACTACCTTAGCAGGGCATGTAATGACAGTGAGAGACGTTGCTGTGTCAGATCGACATCCTTATTTATTTTCTGTT
AGTGAAGATAAGACGGTCAAATGCTGGGACCTAGAGAAAAACCAAATTATTAGAGATTACTATGGACATTTATCG
GGGGTTCGTACGGTGAGCATACATCCAACGCTGGATCTCATAGCTACCGCAGGCCGAGATAGCGTTATCAAACTC
TGGGATATGAGAACCAGAATACCTGTTATTACACTAGTTGGGCATAAGGGTCCAATCAATCAAGTACAGTGTACT
CCAGTAGACCCTCAAGTGGTGAGTTCATCGACTGATGCTACGGTAAGGTTATGGGATGTAGTTGCTGGGAAAACA
ATGAAAGTTCTAACACATCATAAGAGGTCTGTGAGAGCTACAGCGTTGCATCCTAAGGAGTTTTCGGTGGCTTCT
GCGTGTACTGATGACATCAGATCATGGGGATTAGCAGAGGGGTCTTTACTCACCAATTTTGAGTCTGAAAAGACA
GGCATAATCAATACTTTAAGCATTAATCAAGATGATGTATTATTCGCTGGCGGTGACAATGGTGTGCTTTCCTTT
TATGATTATAAGTCTGGTCACAAATACCAATCGTTGGCCACGAGAGAAATGGTAGGCTCTCTGGAAGGTGAACGG
AGTGTTCTTTGTAGCACTTTCGATAAAACAGGTTTAAGATTAATCACTGGAGAAGCAGACAAAAGCATAAAGATT
TGGAAACAGGATGAGACGGCTACAAAAGAGTCAGAACCGGGGCTAGCGTGGAACCCCAACTTAAGCGCCAAAAGA
TTTTAG

>YOR388C 0.78 Cold
ATGTCGAAGGGAAAGGTTTTGCTGGTTCTTTACGAAGGTGGTAAGCATGCTGAAGAGCAGGAAAAGTTATTGGGG
TGTATTGAAAATGAACTTGGTATCAGAAATTTCATTGAAGAACAGGGATACGAGTTGGTTACTACCATTGACAAG
GACCCTGAGCCAACCTCAACGGTAGACAGGGAGTTGAAAGACGCTGAAATTGTCATTACTACGCCCTTTTTCCCC
GCCTACATCTCGAGAAACAGGATTGCAGAAGCTCCTAACCTGAAGCTCTGTGTAACCGCTGGCGTCGGTTCAGAC
CATGTCGATTTAGAAGCTGCAAATGAACGGAAAATCACGGTCACCGAAGTTACTGGTTCTAACGTCGTTTCTGTC
GCAGAGCACGTTATGGCCACAATTTTGGTTTTGATAAGAAACTATAATGGTGGTCATCAACAAGCAATTAATGGT
GAGTGGGATATTGCCGGCGTGGCTAAAAATGAGTATGATCTGGAAGACAAAATAATTTCAACGGTAGGTGCCGGT
AGAATTGGATATAGGGTTCTGGAAAGATTGGTCGCATTTAATCCGAAGAAGTTACTGTACTACGACTACCAGGAA
CTACCTGCGGAAGCAATCAATAGATTGAACGAGGCCAGCAAGCTTTTCAATGGCAGAGGTGATATTGTTCAGAGA
GTAGAGAAATTGGAGGATATGGTTGCTCAGTCAGATGTTGTTACCATCAACTGTCCATTGCACAAGGACTCAAGG
GGTTTATTCAATAAAAAGCTTATTTCCCACATGAAAGATGGTGCATACTTGGTGAATACCGCTAGAGGTGCTATT
TGTGTCGCAGAAGATGTTGCCGAGGCAGTCAAGTCTGGTAAATTGGCTGGCTATGGTGGTGATGTCTGGGATAAG
CAACCAGCACCAAAAGACCATCCCTGGAGGACTATGGACAATAAGGACCACGTGGGAAACGCAATGACTGTTCAT
ATCAGTGGCACATCTCTGGATGCTCAAAAGAGGTACGCTCAGGGAGTAAAGAACATCCTAAATAGTTACTTTTCC
AAAAAGTTTGATTACCGTCCACAGGATATTATTGTGCAGAATGGTTCTTATGCCACCAGAGCTTATGGACAGAAG
AAATAA

>YGL252C 0.78 Cold
ATGTCAACACTTAGCGATAGTGATACCGAGACTGAGGTCGTGTCGAGAAACTTGTGTGGAATCGTCGACATAGGT
TCTAATGGTATTCGTTTTAGTATATCTTCCAAGGCTGCACATCATGCAAGAATTATGCCTTGTGTTTTTAAAGAT
AGGGTTGGTCTTTCTCTATACGAAGTTCAATATAATACACATACGAACGCAAAATGCCCTATTCCCAGAGATATT
ATAAAAGAGGTTTGTTCTGCCATGAAGAGATTCAAATTAATTTGCGATGATTTTGGTGTACCTGAAACTAGTGTC
AGAGTAATTGCAACAGAAGCCACGCGAGATGCTATTAACGCGGATGAATTTGTTAATGCTGTTTACGGTAGCACT
GGCTGGAAAGTAGAAATATTAGGCCAGGAAGATGAAACTAGGGTCGGCATATATGGTGTTGTTTCCTCATTTAAT
ACAGTAAGAGGTCTATATCTAGATGTGGCAGGTGGTAGTACTCAGTTATCATGGGTAATAAGCTCGCACGGAGAA
GTCAAGCAATCCAGCAAACCTGTATCTTTGCCATATGGAGCTGGAACTCTTTTGAGAAGAATGAGAACAGATGAT
AATAGGGCACTTTTTTATGAGATTAAAGAAGCGTACAAAGATGCGATTGAAAAAATTGGTATACCTCAAGAAATG
ATTGATGACGCCAAGAAAGAAGGTGGATTTGACCTTTGGACCCGTGGGGGTGGTTTAAGAGGTATGGGACATCTG
CTTCTTTACCAGTCGGAAGGTTATCCCATCCAAACAATAATTAACGGATATGCTTGCACTTATGAAGAATTCTCG
TCTATGTCAGATTATCTATTCCTAAAACAAAAAATACCAGGTTCTTCAAAAGAGCATAAAATATTTAAGGTTTCT
GATAGAAGGGCTTTACAACTTCCTGCCGTTGGTTTGTTCATGAGTGCTGTTTTTGAAGCGATTCCCCAGATCAAA
GCTGTACATTTTAGTGAGGGTGGTGTTCGAGAGGGTTCACTTTATTCTCTTCTTCCAAAAGAAATTCGTGCACAA
GATCCATTGCTAATTGCGTCCCGTCCTTATGCTCCATTACTTACTGAAAAATATCTATATCTATTGAGAACATCA
ATCCCACAAGAAGATATACCAGAAATAGTAAACGAAAGGATTGCTCCTGCTTTATGTAACTTAGCATTTGTTCAT
GCCTCTTATCCAAAGGAGTTACAACCAACAGCTGCATTACATGTTGCTACAAGAGGGATAATAGCCGGCTGTCAT
GGATTATCTCACAGAGCTAGAGCGCTGATAGGAATTGCTCTATGTAGTAGATGGGGCGGCAACATTCCGGAATCT
GAAGAAAAATACTCCCAAGAATTAGAACAAGTAGTTCTACGCGAAGGTGATAAAGCTGAAGCATTGAGAATTGTA
TGGTGGACGAAGTATATTGGTACGATTATGTATGTGATTTGCGGTGTTCATCCAGGTGGTAATATCAGAGATAAC
GTATTTGATTTCCATGTTTCTAAGCGTAGTGAGGTGGAGACCAGTTTAAAAGAATTAATCATTGATGATGCAAAC
ACTACAAAGGTAAAAGAAGAATCCACGCGTAAAAATCGCGGGTATGAAGTGGTTGTGAGAATTAGTAAGGACGAT
CTTAAAACAAGTGCTTCCGTTCGTTCCAGAATTATCACGCTACAAAAGAAAGTACGCAAGCTATCTAGAGGAAGT
GTAGAGAGGGTTAAAATTGGCGTGCAATTTTATGAAGAATAA

>YBR061C 0.82 Cold
ATGGGTAAGAGCAGCAAAGATAAAAGAGATTTGTACTATAGAAAAGCAAAAGAGCAGGGCTATAGGGCTAGATCT
GCTTTCAAACTACTTCAACTCAATGACCAATTTCATTTCCTGGATGATCCAAACTTAAAAAGAGTTGTAGATTTG
TGTGCAGCACCAGGTTCATGGTCACAAGTGCTCTCGAGAAAACTGTTTGATGAAAGTCCCAGTTCAGATAAGGAG
GACAGGAAAATTGTCTCTGTCGATTTACAACCGATGTCCCCCATACCTCATGTGACAACGTTGCAAGCCGATATC
ACTCATCCTAAAACATTGGCGAGGATTCTAAAACTGTTTGGCAACGAAAAGGCCGATTTTGTTTGTAGTGATGGT

GCACCTGATGTTACTGGGTTACACGATCTTGACGAATACGTGCAACAACAGTTAATTATGAGTGCGCTGCAACTT
ACTGCATGCATTCTGAAAAAAGGTGGAACTTTTGTGGCAAAGATCTTCAGAGGTCGTGATATAGATATGCTATAT
TCCCAATTGGGCTATCTATTCGATAAAATCGTTTGCGCAAAGCCAAGATCATCAAGAGGTACATCTCTGGAAGCT
TTTATTGTTTGTTTAGGCTATAACCCACCATCCAATTGGACACCAAAATTAGATGTAAATACATCCGTTGATGAA
TTTTTTCAAGGCTGTTTTTTGAATAAATTGTGTATATCAGACAAATTGTCTCATTGGAATGAAGAGGAGAGAAAC
ATAGCCGAATTTATGGCTTGTGGAAGTCTTCAAAGTTTCGACTCAGATGCCACTTATCATGACCTACCTTCTTCG
GTTGCAGGCACTTCATCGTCCTTAGATCCTGTTCAAAGCCCGACGAACCCTCCCTACAAGAAAGCTTTGGAATTA
AAAAGGAGCGGGAAACTCACTAGATCAGTTTGA

>YOL010W 0.75 Cold
ATGTCATCTTCCGCCCCCAAATACACCACTTTCCAAGGGTCACAAAATTTTAGGTTACGGATCGTCTTGGCAACA
TTATCAGGGAAACCAATAAAAATTGAAAAAATCCGTTCAGGCGACTTAAATCCCGGTCTGAAAGATTATGAAGTG
TCTTTTCTAAGGCTGATCGAGTCGGTCACCAACGGGAGTGTAATTGAAATTTCTTATACCGGTACTACTGTGATT
TATAGGCCCGGTATCATAGTTGGTGGTGCATCAACTCATATCTGCCCTAGTTCCAAACCGGTAGGTTATTTCGTC
GAACCAATGCTATATTTGGCTCCATTTTCAAAAAAGAAATTTTCTATATTATTCAAAGGCATAACTGCATCTCAC
AACGATGCCGGTATTGAAGCTATCAAATGGGGACTCATGCCCGTTATGGAAAAATTTGGCGTTAGAGAGTGCGCC
CTACATACTTTGAAAAGAGGTTCACCACCACTAGGAGGTGGCGAAGTGCATTTGGTTGTTGATTCTTTGATTGCG
CAACCTATAACTATGCATGAAATAGATAGGCCCATAATTTCATCGATTACCGGTGTAGCATACTCTACCAGAGTA
AGTCCGTCGCTTGTGAATAGAATGATCGATGGTGCTAAGAAGGTATTGAAAAATCTGCAATGCGAAGTTAACATA
ACGGCAGATGTCTGGAGAGGTGAAAATTCAGGGAAGAGTCCAGGCTGGGGTATTACTTTGGTTGCTCAGTCCAAG
CAGAAAGGTTGGAGTTATTTTGCAGAAGATATCGGTGATGCAGGTTCTATACCTGAAGAACTTGGTGAAAAAGTT
GCCTGTCAATTATTAGAAGAAATATCAAAGAGTGCAGCAGTTGGTAGAAACCAGCTTCCATTAGCAATTGTTTAC
ATGGTCATCGGGAAAGAAGATATCGGCAGATTGAGAATTAATAAGGAACAGATAGACGAAAGATTCATAATCCTC
TTGAGAGATATTAAGAAGATCTTTAATACTGAAGTCTTTTTAAAACCAGTTGACGAGGCGGATAATGAAGACATG
ATAGCTACTATCAAGGGTATTGGTTTCACAAACACAAGCAAAAAGATTGCATAG

>YMR269W 0.8 Cold
ATGGATAGCAAAGAATATCTGATATCATATGGTTGGAAAGAAGGAGAAGCGTTTAGAGAAGGTGGTTTGAAAAGA
CCCATACTGGTAAAGCACAAGAGAGATAAGAAGGGATTGGGTAATGCTCCTGGCGGGAATGATGGCGAAGCATGG
TGGGAAAGGCTATTTGATGGACATCTGAAGAACCTGGATGTAAGCACTGATTCGAATAATGGCAGTATTAAATTT
ACTCAAAATGAGGCAGTTGCTACTGCTGTATCGAAAAGTAGCTCACCTCTATACAGGTGGTTTGTAAAGGGAGAA
GGGCTGAAAGGAACCATTACTAATCTTGGTAAAAAGGAGGAAGCCAGCTTTGTTGTATCTAGTGCAAGTTCGAGT
AAAGGGAAGAAGAGAAGAAGGCGTGATGAAGATGATAACAAGGTCAAGAGAAAAAAATTGAAAAAAGATAAAAAG
ACTAGTAACGACAGTGAGAGTAAGAAGAAGAAGAAGAAGAAAAGCAAGAAGGAGAGTAAAAAAGGAAAGAAAAGT
AAGCATAGCAGCGATGAAGGTGATAAATCGAAGCATAAAAAATCCAAAAAGTCAAAAAAACATAAAAAGGAAGAA
AGTTCAGCAAGAAGAGATAGAAAGGAGCATATATGA

>YOR198C 0.79 Cold
ATGTCCTCCCAACAACACAAGTTCAAGCGCCCAGATGTCTCCGTTAGAGACAAGAAATTGGACACTCTTAACGTT
CAATTGAAAAAAATCGACACTGAAATCGGTTTAATTAGAAAGCAAATCGATCAACACCAGGTCAACGATACCACC
CAACAAGAACGTAAGAAGTTACAGGATAAGAACAAGGAGATCATCAAGATCCAAGCTGACTTGAAAACCCGTAGA
AGCAACATTCACGACTCTATTAAGCAATTGGATGCTCAAATCAAGAGAAAAAACAACCAAATTGAGGAGAAGTTA
GGCAAAAAGGCCAAGTTCTCGTCCACTGCAGAAGCCAAGCAAAGAATCAATGAGATTGAAGAGTCTATTGCCTCT
GGTGACCTTTCTTTGGTTCAAGAAAAACTACTAGTCAAAGAAATGCAATCTTTGAACAAATTGATTAAGGACTTA
GTTAACATCGAGCCAATCAGAAAGTCTGTTGATGCTGACAAGGCTAAAATCAATCAATTGAAGGAAGAATTGAAC
GGATTGAATCCAAAGGATGTCTCCAACCAATTCGAAGAGAACCAGCAAAAATTGAACGATATCCATTCAAAAACT
CAAGGTGTTTACGACAAAAGACAAACTTTATTCAACAAACGTGCTGCCTTGTACAAGAAGCGTGACGAATTATAC
AGTCAAATCAGACAGATCAGAGCTGACTTTGACAACGAATTCAAATCATTCAGAGCCAAATTGGACAAAGAACGT
TTGAAACGTGAAGAAGAACAAAGATTATCCAAACTTTTGGAACAGAAAGATGTTGATATGGGTAAATTGCAAGAA
AAGTTGACTCATGCCAAGATTCCAGCCTTCACTTATGAAATCGGTGCCATTGAGAATTCTTTGCTTGTCCTGGAC
CCAACTTATGTGAAACCAAAGAAAAACATCCTGCCTGACTTGAGTAGCAATGCTTTGGAGACCAAGCCTGCCAGA
AAAGTTGTTGCTGATGATTTGGTTTTGGTCACTCCAAAGAAGGATGATTTTGTCAATGTTGCTCCATCTAAATCT
AAGAAATACAAGAAGAAGAACCAACAGAAGAACACTGAAAATGAGCAACCTGCTTCTATTTTCAACAAAGTCGAT
GGCAAGTTTACTTTAGAACCAACACTAATTGCTACTTTGGCCGAATTAGACGTAACTGTCCCAATCAACAGTGAT
GATGTCAAGATCACCGTTGAGCAATTGAAGAAGAAGCATGAAGAGCTTTTGTCGAAACAAGAAGAGCAAACAAAA
CAAAACATTGAATCTGTCGAAAAGGAAATTGAGAAATTAAATTTGGACTACTCTAACAAAGAGCAACAAGTAAAA
AAGGAATTGGAAGAAAAAAGATTGAAAGAACAGGAAGAGTCTGAAAAAGATAAAGAAAATTAA

>YKL169C 0.69 Cold
ATGGGGGTACTTTTATTCTTATATGACCCTACTTGCCAAAGAGCATATCTTATTGTATCCCTTGTCTTTCAGTGT
TCTATCTACACAACCATCATTAGCCATAATTCTTGTCCCCAGAGGTTCACCGGTATTTTTATTAATCAAAACGCA
AGCAGTATCTCCGAATGCAACGGTGGAGCCATCCTTTCTGCACATGTTACGCTGTTTAGACCTTACGACAATTGC
GTGACAAATATCACCTTTTTTGACACGGTTGGTGTTGGCTGTCCCCGTAATGTTTTGAGTCAAGGGCTTTGCTTT
CTGTATAACACAGACTATTCTGTCTCCAACCATTGCAGGACTCTTGGGGGACCCTTTCCTTATTACTTTAATACA
TTCTGCTAA

>YHR054C 0.81 Cold

ATGGTACCCGCTGCTGAAAACCTATCTCCGATACCTGCCTCTATTGATACGAACGACATTCCTTTAATTGCTAAC
GATTTAAAATTACTGGAAACGCAAGCAAAATTGATAAATATTCTGCAAGGTGTTCCTTTCTACTTGCCAGTAAAT
TTAACCAAAATTGAAAGTCTGTTAGAAACCTTGACTATGGGCGTGAGTAATACAGTAGACTTATATTTTCATGAC
AACGAAGTCAGAAAAGAATGGAAAGACACTTTAAATTTTATCAATACCATTGTTTATACAAATTTTTTCCTTTTT
GTTCAAAACGAATCCTCTTTGTCCATGGCAGTTCAACATTCTTCTAACAACAATAAGACCTCGAACTCTGAAAGA
TGTGCAAAGGATCTGATGAAAATTATTTCTAATATGCACATTTTTTACTCAATAACATTTAATTTTATCTTCCCC
ATAAAGTCGATAAAGTCATTTTCAAGCGGCAATAATCGCTTTCATTCTAATGGTAAAGAATTTTTATTCGCAAAT
CATTTTATTGAAATCTTACAGAATTTTATAGCAATCACATTTGCTATTTTCCAACGTTGTGAAGTAATATTATAT
GACGAATTTTACAAAAATCTTTCAAATGAGGAGATTAATGTTCAATTGCTATTGATTCATGACAAGATTTTGGAA
ATTTTAAAAAAAATAGAAATTATCGTATCCTTTTTACGAGATGAAATGAATAGCAACGGAAGTTTCAAATCTATT
AAAGGTTTCAACAAGGTTTTGAATCTGATTAAATATATGCTGAGATTTAGCAAGAAAAAACAAAATTTTGCGAGA
AACTCTGATAACAATAATGTTACAGATTATAGTCAGTCGGCGAAGAACAAAAATGTTCTCTTGAAATTCCCCGTT
AGTGAACTGAACAGAATCTATTTAAAATTTAAGGAGATTTCAGATTTTTTAATGGAAAGAGAAGTTGTCCAAAGG
AGTATAATTATTGACAAGGATTTGGAATCTGATAATCTGGGTATTACTACGGCAAACTTCAACGATTTCTATGAT
GCATTTTATAATTAG

>YNR075W 0.76 Cold
ATGGGGGAAGAGATTAGACTTTTGTCCTCTAAAGGTCTTGAGACCCATAGTAGCTTTTGTTTGCCCGAGGATATT
TTCAGAAACCGTTTAACATGGTTGTGCAGTGAGATTCGCAACTCAGGATTTTGGATCTGGTCGCTATCATGGTTA
CCGCTTGCCGTTTGGTGGGGGATGGCCAGTACTTGGTTTTATCCCCTTCTGACTTCAGTAGTTCTAATCCTGGCG
CTAATTGCTATTTTGCCAGTAGTTCAAATACAGTACAGTAAATATACTTTATCAAATCAACTCACCCAGCTTTCT
AAAGAAATCATCAAAAGTGCACCAGGTGCATACTCTGCTGATTGGGACGCTGTTGCAATACATTTCAATTCCTAC
TTGTATGAAAACAAAGCATGGAAAACTGCACATTTTTTTTTTAATGGTACTGACTGCCAAGAAGCATTCAGGAAA
ACCATTCTAGAGCCAGCTGTTTTGAGGAGACAAAACGAAGATCCTAGGTTCAGTTCAATTGAGATTTTGGTCCCT
TACACCGAAGACGCGGTGCAGGTTTATTTTACAAAAGTAAACGCACAGTGGAGGTTGATCCATGGTAAGAAGGAA
TGCAAACTTACTATGTTGGAAAATGTCAAGCTTCCTAAGGAAACCTATCGCTGCAAGCTAGCATGGAGTTGCCAG
AGAATCAGGATTTCTTTCTCTCCACTAGACTTCCTTCCTGATATGTCTGACTTCTTTATTTGTGCAAATTTATCA
CCAGCATGCTTGGTATCTTATTGGCTAATAGACATTTTCTTTAGAATGATAGATGATTTTCAAAACATAAGGCCT
AAATCGATGAAAGTTGATGATAAAATGCAGTACCTATCAGATATTATAAACGAGCAGGGTGCTAGCCCTGAAAAA
TGGGATACAATTGCAAGGAAAACGAATACACGATTATTTGAAAAGAGGGTCTGGAAAAACGAAGAGTTTTTCTTT
GATGGGACTGATTGTCAGGCATTCTTTGAACGGAACTTTAGTAGCCTTTTGTTCTCAAAAAAATCTGCGTCGCCT
AGATCATTAAACGTTGAACTTTGGAAATACATTCAAGAAGCGCAATTGAGCTGTAACTATGAGCCGTTACCATAA

>YCR019W 0.75 Cold
ATGATGAATGAAGAGGATTCTACAGAAACGAAAAGCCTAGTCATAACTAATGGCATGTTTATCATAGACGACATC
GAGCGTAGTAAATATAATATTCACTATAAGAATGTCCCAGGAGGCGGAGGGACTTTTGCCATTTTGGGTGCATGC
ATAATATCTTCCGGCAATGTCACATCCAAAGGTTTGAAGTGGATAGTGGACAGAGGCTCTGACTTTCCAAAGGAA
GTTATAAGGGAAATAGACTCATGGGGTACTGATGTGAGGTTTCGAGATGACTTTAGCAGATTAACTACCAAAGGG
TTGAATTATTACGAGGGAAGTGATGATTTGAGAAAGTTCAAGTTTTTGACGCCGAAGAAGCAGATTAACGTCGAT
GACTGGATTTCCACATTTGGGCAGAAGATAATTGATGAAATGCATGCGTTTCATTTGCTATGTTCTGGGTCTAGA
TGCTTAGACATAATAAACGATCTGCTACGGGTGAAAAGTTCAAAGGGCACAAAACCAATCGTGATTTGGGAGCCA
TTCCCAGATCTTTGCGACTTTGATCATCAAAATGACATTAAAAGTGTAATGCAGAGGAACGATGTTACGGTAATA
TTATCTCCAAATGCCGAAGAATCAAGTCGCTTATTTGGTTTAAGTAGCAAGGAACCGACTAGTTTGGAAGAATGT
CTAGCATTAGCGCATCGTTTCGATGATTTCATGGATGAAAACAATATGTGTATTCTACGATGCGGTGCCCTCGGA
AGCATATCGGTAAGTGAGAAGTTTAAGAACGGACGAACCTATGACCATTTCCCCGCCTACCATTTCAAAACTCAG
TCTAAAGTACTAGATCCTACTGGCGGGGGAAACTCGTTCCTTGGCGGCTTTGCAGTTTCTTATGCCCTAACGAAA
AGCTTAGATATTGCTAGTATATGTGGGAACATCGCTGCAGGCGCAATAATTGAACAATTCGGAATACCGAGGTAC
GATCCAATTGCTAAAACCTGGAACGGAATCACATTCTTGGATAGACTGAAATTTTACCTTTCACAGTCCGGTCTT
CAATATAATATAAACGATCTTTACAAAAGTCTAACACGATGA

>YBR290W 0.8Cold
ATGCCAGAGCAAGAACTACTTATAGGGCAAGAAATGAATACACTTCATGCAGGTTCATCTACTGATGGCATAAAT
GTCGGAAACGCAGGACGAACTAGAGACACACAAACTGGCGTAGAAGGGGAAACGGAAATAGGGTCTGACGAGGAA
GATAGCATAGAGGACGAGGGAAGCAGCAGTGGAGGAAATAGTACGACAGAAAGACTAGTACCACACCAGCTGAGG
GAACAAGCAGCCAGACATATAGGAAAAATAGGAAGACATTTTAATATTCTTGATAGACTTTTTAAGAAACGTACA
CAACAGTCTTCGGATATACAACAAGGTGCCATGTTTGATGGTGTCTTTAGCAATTTAAGCGCAAAACCAGATACC
ACAGAGACTGAAGGTAATAACGAACAAGACATACCACCTACATACGACGAAGCTGCTGCTGATATGGCCCCCTCG
TATTATGGAATGGATTTGAACAATTCAGATATCTACTACGACGAAATATGCATTGAGGGGCTTCCTGTAGGAAAT
ATAGCAAATTTATTATGGAATATCATCGTAAGCACGAGTTTTCAGTTCATTGGATTTTTAATAACCTACATTTTA
CACACATCTCACGCAGCAAAGCAAGGTTCAAGGTTTGGATTAGGGTTAACGTTCATTGGATATGGTTATTCAATG
ATTCCCAATGATGTTACTTCAAAAGTCGGCAAAAATAAAAGCTTAAATAGAATGGAATTAGAGGATCCAAATGAA
TTCGATGATGTTCGTCTAAATTCACAATCAACGACACAAGATAAATTTGAATCACATTTGAATCACGGTCTGGAT
GAAGAAAAACAAAACATACCATGGTTGGCTGTTTTTGTGGCATTTCTTGGGCTATTTATCACCCTGAAGAGTATA
TACGACTACATTCAAGTCAAAAAAATTGGAAAAGAAATACCTCAACCAAAGTCAAAATCAAGCATAA

>YNL252C 0.81 Cold

ATGAAGGTAAATTTAATGTTGAAAAGAGGGCTTGCTACTGCAACTGCAACTGCCAGTTCCGCTCCCCCCAAGATT
AAAGTCGGAGTACTACTGTCAAGAATCCCTATAATTAAATCAGAATTAAATGAACTAGAGAAAAAATACTATGAG
TACCAATCAGAACTAGAAAAGAGACTAATGTGGACGTTTCCGGCATATTTTTATTTCAAAAAGGGTACTGTAGCA
GAACACAAATTTCTATCCCTGCAGAAAGGACCTATCTCCAAAAAAAAATGGCATTTGGTTTCCTAGAGGCATACCG
GACATTAAACATGGCAGAGAAAGAAGTACTAAGCAAGAAGTTAAACTTTCTGATGACAGTACAGTAGCATTTAGC
AACAATCAAAAAGAGCAAAGCAAAGACGATGTTAATAGGCCCGTGATTCCCAACGACAGGATAACGGAAGCAGAT
AGGTCAAATGATATGAAGAGCCTTGAAAGACAATTGAGCAGGACCTTATATCTTTTGGTTAAGGATAAAAGCGGT
ACTTGGAAATTCCCTAACTTCGATCTTTCTGATGAATCTAAGCCGTTACACGTACACGCAGAGAACGAATTGAAA
TTGTTGAGCGGTGATCAGATATACACTTGGTCTGTTTCTGCTACGCCCATAGGTGTTTTGCAGGACGAGAGAAAT
AGGACTGCTGAGTTTATTGTGAAGTCACACATTTTGGCTGGAAAATTTGATTTGGTGGCGTCGAAAAATGATGCA
TTCGAGGATTTTGCTTGGCTGACAAAAGGTGAGATCAGTGAATATGTACCAAAAGATTACTTTAACAAAACAGAA
TTTCTTTTAGCGGATAATTGA

>YOL147C 0.74 Cold
ATGGTCTGTGATACACTGGTATATCATCCCTCCGTGACGAGATTCGTCAAATTTCTAGATGGCTCAGCTGGCAGA
GAAAAGGTTCTCAGATTACTGCAGTATTTAGCAAGATTTTTAGCAGTACAGAACTCATCTCTCTTGGCCAGGCAA
TTACAAGCTCAATTTACCACAGTCAGAAAATTTCTGAGGTTTCTGAAGCCTTTAAACCACTTGCAGGCAGCTGCT
AAATTCTACGATAACAAGTTGGCCAGCGACAACGTCGTTAGAGTCTGCAATGTTTTGAAAAACATCTTCTTTGCT
GCATATTTGTCGTTAGATCAAGTCAATCTTTTGAGAATTTTGAAAGTGATTCCTGTAACCGTTCTTACCGGTAAG
AAAATACCTCGCTGGTCCAATTGGTGTTGGCTGTTCGGCCTCCTAAGTGGTCTGGCTATGGATCTTCGTAAGATC
CAAACATCTCATGCCCAGATTGCTGCGTTTGTCAAGGCAAAATCACAAAGCCAAGGCGATGAGCATGAGGATCAC
AAGAAGGTACTAGGGAAGGCATACCAAGACAGGTACACCGCGTTAAGAAGACTATTCTGGGATGCTGCAGATTCG
TTCATCGTCCTCAACAACCTAGGGTACTTGTCTAGCAACGAAGAGTATGTTGCGCTATCCGGTGTTGTCACATCT
ATCCTTGGTATGCAAGACATGTGGAAAGCTACATAG

>YMR186W 0.77 Cold
ATGGCTGGTGAAACTTTTGAATTTCAAGCTGAAATCACTCAGTTGATGAGTTTGATCATCAACACTGTCTATTCT
AACAAGGAAATTTTCTTGAGAGAACTGATCTCTAACGCCTCCGATGCTTTAGACAAAATTAGATACCAAGCTTTG
TCTGATCCAAAGCAATTGGAAACCGAACCAGATTTGTTCATTAGAATCACCCCAAAACCAGAAGAAAAAGTTTTG
GAAATCAGAGATTCTGGTATTGGTATGACCAAGGCTGAATTGATTAACAATTTGGGTACCATTGCTAAGTCTGGT
ACTAAAGCTTTCATGGAAGCTCTATCTGCTGGTGCCGATGTATCCATGATTGGTCAATTCGGTGTTGGTTTTTAC
TCTTTATTCTTAGTCGCCGACAGAGTTCAAGTTATTTCCAAGAACAATGAGGACGAACAATATATTTGGGAATCT
AATGCCGGTGGTTCTTTCACCGTTACTTTGGACGAAGTTAACGAAAGAATTGGTAGAGGTACCGTCTTGAGATTA
TTCTTGAAAGATGACCAATTGGAGTACTTGGAAGAAAAGAGAATTAAAGAAGTCATCAAGAGACATTCTGAATTC
GTTGCTTACCCTATCCAACTTCTAGTCACCAAGGAAGTCGAAAAGGAAGTTCCAATTCCAGAAGAAGAAAAGAAA
GACGAGGAAAAGAAGGATGAAGATGACAAGAAACCAAAATTGGAAGAAGTCGATGAAGAAGAAGAAGAAAAGAAG
CCAAAAACCAAAAAAGTTAAAGAAGAGGTTCAAGAATTAGAAGAGTTGAACAAGACTAAGCCATTATGGACTAGA
AACCCATCTGATATCACTCAAGAGGAATACAATGCTTTCTATAAGTCTATTTCTAACGACTGGGAAGACCCATTG
TACGTTAAGCATTTCTCTGTTGAAGGTCAATTGGAATTTAGAGCTATCTTGTTCATTCCAAAGAGAGCACCATTC
GACTTATTTGAGAGTAAGAAGAAGAAGAACAATATCAAGTTGTACGTTCGTCGTGTCTTCATCACTGATGAAGCT
GAAGACTTGATTCCAGAGTGGTTATCTTTCGTCAAGGGTGTTGTTGACTCTGAAGATTTACCATTGAATTTGTCC
AGAGAAATGTTACAACAAAATAAGATTATGAAGGTTATTAGAAAGAATATTGTCAAGAAATTGATTGAAGCCTTC
AACGAAATCGCTGAAGACTCCGAGCAATTTGACAAATTTTACTCTGCCTTCGCTAAGAACATTAAGCTGGGTGTA
CATGAGGACACTCAAAACAGAGCTGCTTTAGCTAAGTTGCTACGTTACAATTCTACTAAATCTGTCGATGAATTG
ACTTCCTTGACTGATTACGTTACTAGAATGCCAGAACACCAAAAGAACATCTATTACATCACCGGTGAATCTCTA
AAGGCAGTCGAAAAGTCTCCATTCTTGGACGCCTTGAAGGCTAAGAACTTTGAAGTTTTGTTCTTGACCGACCCA
ATTGATGAATACGCTTTCACTCAATTGAAGGAATTCGAGGGTAAAACTTTGGTTGACATTACTAAAGATTTCGAA
TTGGAAGAAACAGACGAAGAAAAAGCTGAAAGAGAGAAGGAGATCAAAGAATACGAACCATTGACCAAGGCCTTG
AAGGATATCTTGGGTGACCAAGTGGAGAAGGTTGTTGTTTCTTACAAATTGCTAGATGCTCCAGCTGCCATCAGA
ACTGGTCAATTCGGCTGGTCTGCTAACATGGAAAGAATCATGAAGGCTCAAGCCTTGAGAGACTCTTCCATGTCC
TCCTACATGTCTTCCAAGAAGACTTTCGAAATTTCTCCAAAATCTCCAATTATTAAGGAATTGAAAAAGAGAGTT
GATGAGGGTGGTGCACAAGATAAGACCGTCAAAGATTTGACTAACTTATTATTCGAGACCGCTTTGTTGACTTCT
GGTTTCAGTTTGGAAGAACCAACTTCTTTTGCATCAAGAATAAATAGATTGATTTCTTTAGGTTTGAACATTGAT
GAGGATGAAGAAACAGAAACCGCTCCAGAAGCTTCTACCGAAGCTCCAGTTGAAGAGGTTCCAGCTGACACCGAG
ATGGAAGAAGTTGATTAA

>YKL079W 0.74 Cold
ATGCACTGGAATATAATTTCGAAAGAGCAAAGTAGCTCTTCTGTATCGTTACCAACTCTAGACAGCAGTGAACCC
TGTCACATCGAAGTTATCCTCAGGGCGATACCCGAAAAAGGATTACAAAACAATGAGTCGACCTTCAAAATAGAC
CCATATGAAAATACTGTGCTATTTCGCACAAACAATCCGTTACATGAGACAACCAAGGAGACCCATTCAACATTT
CAATTCGATAAGGTATTCGATGCTAACGCCACTCAAGAAGATGTTCAGAAATTTCTGGTGCATCCAATAATAAAT
GATGTTTTGAATGGTTATAACGGTACTGTAATAACATATGGACCAAGTTTCAGCGGAAAGTCCTATTCCCTTATT
GGATCAAAAGAAAGCGAAGGAATTCTACCGAACATATGCAAGACCTTGTTTGATACGCTAGAAAAAAATGAAGAA
ACAAAGGGAGATAGTTTTAGCGTAAGTGTTTTGGCATTCGAAATATATATGGAGAAAACGTATGACTTATTGGTA
CCTTTACCTGAAAGAAAACCATTAAAGCTTCACCGTTCTTCAAGCAAAATGGACTTAGAAATCAAAGATATTTGT
CCGGCACATGTCGGATCGTATGAAGACTTAAGAAGCTACATTCAGGCAGTCCAAAACGTCGGCAATAGGATGGCA
TGTGGCGACAAGACAGAGAGATCAAGATCACACCTAGTTTTTCAATTACACGTAGAACAAAGGAATAGAAAAGAT

GATATATTAAAAAATAGTTCTTTATATCTGGTTGATTTACACGGGGCAGAGAAGTTCGATAAAAGAACTGAAAGT
ACGCTATCACAAGATGCGTTAAAAAAATTAAACCAATCTATTGAGGCGTTGAAAAACACTGTGCGGTCCTTGTCA
ATGAAAGAGCGTGATTCAGCCTACAGCGCAAAAGGATCACATAGTTCTGCTTACCGTGAATCGCAATTAACTGAA
GTGTTAAAAGATTCCCTTGGGGGAAATAGGAAAACAAAGGTGATATTGACATGTTTCTTAAGTAATGTTCCAACT
ACCCTATCAACACTAGAATTTGGTGACAGTATTAGACAGATCAATAACAAGGTTACAGATAACACCACAGGTTTA
AATCTGAAAAAAAAAAATGGATCTATTTATTCAGGACATGAAAATTAAGGATGATAATTATGTTGCCCAAATTAAT
ATACTAAAGGCTGAAATAGACTCTTTAAAAAGCCTTCACAATAAATCTCTTCCCGAAGACGACGAGAAAAAAATG
TTAGAAAATACAAAGAAAGAAAATATCAAACTAAAGCTTCAATTAGACAGTATTACCCAATTATTGAGTAGTTCT
ACCAATGAAGATCCTAACAATCGTATTGACGAAGAAGTTTCTGAAATATTAACGAAAAGATGCGAACAGATTGCT
CAGCTTGAGTTATCTTTTGATCGACAGATGAATTCCAATTCTAAGTTGCAGCAAGAGTTAGAATACAAAAAGTCA
AAAGAGGAAGCCTTAGAGTCTATGAACGTTAGGCTACTAGAACAAATTCAGCTTCAGGAAAGAGAGATTCAAGAG
CTTTTAACTACTAACGCCATCTTAAAGGGTGAACTAGAAACTCACACTAAACTTACTGAAACACGCAGTGAAAGG
ATAAAATCTTTGGAAAGTTCTGTCAAAGAGCTTTCTCTTAATAAATCTGCAATCCCATCTCCTAGAAGAGGGTCT
ATGAGCTCGTCATCAGGAAATACTATGTTGCATATCGAGGAGGGTTCTGAAATATCAAATAGCCCTTGGTCAGCC
AATACTTCCTCAAAACCTTTAGTATGGGGCGCGAGAAAGGTTTCTTCTAGCAGTATAGCAACCACTGGATCACAG
GAGTCTTTTGTGGCAAGACCGTTCAAGAAGGGATTAAACCTCCATTCGATAAAAGTCACTTCGAGTACTCCAAAA
AGTCCCTCTTCTGGAAGCTGA

>YNL237W 0.67 Cold
ATGACAGCAGCTAATAAGAATATTGTCTTCGGATTTTCCAGATCCATTAGCGCAATTCTACTAATATGCTTTTTC
TTTGAAAAAGTCTGCGGTGATATGGAGCATGATATGGGCATGGATGATACTTCGGGATACACGAGGCCAGAAATT
GTGCAGGCTGGGTCGAAATCTTTCCACTGGCTCTGCACTTTGGGATTCTTGTTGCTTTTACCATCCGTGGTGACG
TGCCTTTCGTTCGCTGGCAGGATATATTCAGCTACCCTCTTACAATGCACTTGTGCCGTTTACGCTTTCTTAGAA
GCTGCCGTATTAAGATTTCAAGACAATGATGGGGTAGAAAATAGAACTTCAAGGGGAACCGCATGGTTTTTGGTG
GGACTTACTTGGATAACCTTATTCTTTGGTGGATTAGCTGGAGGAACTGGTTTCTTAGTGAAAAGCAAGAGGTTG
CAAACGTTCATATCAAATGCAGGTGAGAAAAGGTTGTCATATATCCATCGTGGTTTATCCTTTCTAACTGTTCTA
ACAGGTTGGGTTAAAGTCTGTTTGGCACCTGTTGCGCTCTTTGGGTTTTGTAGAGAGGCACACACAGGGCAATGC
ATCGCTCATGGTATCATGGGATCCGCGTTTGTGTTGTACGGGTTCATTTATGTACTGGTTTTGGTTATACCATGG
ATTCGCAGTGCCCAAACCTCATATTCACAGGATTACGTCGACAGTTGGGTTATGTGCATATGGGGCGTTGTAAAT
ACTTTTACTGAACACAGGTGGGGACGTGAAGGATGGAGTGTTCACGATTATCAACACACGTTTATGGGAATTATC
TGGTGGACTGGTGGAATACTCGGGATTTTCCTTTCCAGAAATGGCAGAAGAACATTTGTACCAAGTTTGATTATT
ATTTTCACCGGATGGGCCATGTCTGAGCACGCCCAGCACTTGATAATTAGCACAAAAGTCCATAATATGTTTGGG
CTAGTTTTAATGTGTGGAGGCGCTTTAAGAATTATCGAAATATCTTTTTTACTACGTGATAAAAGAACGCTGGAC
AAAATACATTCATTCCAATACCTTGCTCCCTTTTGTTTAGTATGTTCAGGTCTCCTATTTATGGGCGCAAACGAA
GAACAACTCATTTTAGTGTTGCGGTTGGGAGGCGACCATAGTGCGTACGTTCTAATTATCGTATCAGGCGCATTT
TTGGTCTACTTCTGGATGATTGCATGTTTGGAATTCTACCTATATCTACTTGAAAAGGGAAAACAAGGATTCCTT
CCGAAATCATACGAACTTGAAGAGGAAAACAATAACGTGAGCTTTGAGCTAGATAACATATCGAACGAAGATGTG
GATGAAGATACCACTCCCTTTAACGTTTGA

>YNL286W 0.81 Cold
ATGGATGCTGATGAATTGGAATTGAAAGGTCATTTGAAAAAATTGAAAAAAGAAGAATTGCTTAGAAGGAAACAG
TTAAAAGAGAGTAATCTTCAGAAAAGAGAGCTTGAATATAATAATGCCTCAAAAAATACTTCAATATATATTTCT
GGTCTTCCCACAGACAAAACAACGAAAGAAGGACTCACTGAGCAGTTTTGCAAGTATGGAATGATAAGAACCAAT
CGAGATGGGGAACCCCTTTGCAAGCTATATGTGAATGATAAAGGCGCTTTCAAAGGGGATGCCTTGATCACTTAT
TCCAAGGAAGAAAGTGTTACATTAGCAATCGAAATGATGAACGAGTCGATCTTCCTGGGAAAACAGATCAGGGTC
GAAAGAGCCCAATTTCAAAACAAGGAAGGTGACAATATGCACGGGAAAGAAAATGATTTAAAGGAGTTCAACGGA
CCTGAGCCTCCAATAAAGAGGCTAAAAAAAGCGAAGTCTGAAGGGGAGGGGGAAGTAATAGATTACAATGACGAT
GAAAGCCTAGCAAAAGCTGATCGAACGGTGATATTTGCCAACGTATTCAATATTTACAAATCATACACAAATGAT
GATATAAATGATATTCAGGAAGATCTTTTGGAAGGCTGTGAAGAGATAGGTCAAGTAGACAGTATATCTGTATCT
CCCAATAAGGGGGAAGCGACGGTCGTCTTTAAGAACAATAAAGTTGCATTGCAATGTTGCAAAATCATGACCGGA
CGTTATTTTGATGGGCAAAAGTTGCTTGCTTTCATTTCTGGAGATGAGAACACTTCAAGCACAAGTGACAAGAAT
GAGGACAGCGAAGTGGAAGATGACCTTATATAG

>YLR008C 0.75 Cold
ATGAGTTCTCAAAGTAATACTGGTAATTCTATTGAGGCACCACAACTACCCATTCCTGGTCAAACTAATGGCTCT
GCGAACGTTACTGTTGATGGAGCTGGTGTTAATGTCGGTATCCAGAATGGTTCGCAGGGTCAAAAGACCGGAATG
GACCTTTATTTTGATCAAGCTTTGAACTACATGGGAGAACATCCTGTGATAACAGGTTTTGGGGCCTTTTTAACT
TTATATTTTACAGCCGGTGCATATAAATCAATATCGAAGGGACTTAACGGTGGAAAATCCACTACTGCCTTCTTG
AAAGGCGGATTTGACCCGAAAATGAATTCTAAAGAGGCTCTACAGATTTTGAATTTGACAGAAAATACATTGACT
AAAAAAAAGTTGAAAGAGGTTCATAGGAAAATTATGTTAGCTAATCATCCTGACAAAGGTGGTTCTCCATTTTTG
GCCACTAAGATAAACGAAGCTAAGGACTTTTTGGAAAAAAGGGGTATTAGCAAATAA

>YKL138C 0.73 Cold
ATGTTTGGCCCATTCAAATTAACAAGCCCCGTCGCTGGTGGCCTACTATGGAAAATTCCATGGAGAATGTCCACA
CATCAAAAAAACCCGTCAAAGGGAAAGATTAAGAAACGTTGATCAAGTCATAAAGCAATTAACTTTAGGTCTCCAC
GTCCAAAGATGTCAGGATAAAGGTCTTACTTATCAAGAAGCCATGGAGAGCAAAAAGAAATACAAGCCAAGAAGT
AAATCGTTAAGGTTGTTAAACAAACCATCAGTCTTTCCAAAGGAGAATCAAATGTCTTCTAAAGATAAATATTGG

ACTTTTGATAAAAAAGCTGTTGGTTATAGGAAGGGTATTCATAAGGTGCCCAAGTGGACGAAGATTTCCATTAGA
AAGGCCCCAAAATTCTTTTGA

>YPL167C 0.78   Cold
ATGTCGAGGGAGTCGAACGACACAATACAGAGCGATACGGTTAGATCATCCTCTAAATCAGACTATTTTAGAATC
CAGCTAAATAATCAAGACTACTATATGTCAAAACCCACCTTTTTGGACCCATCGCACGGTGAAAGCCTGCCCTTA
AACCAGTTTTCACAGGTCCCAAACATCAGGGTGTTTGGTGCTTTACCCACAGGGCATCAAGTATTGTGCCACGTC
CATGGCATTCTACCTTACATGTTCATCAAGTATGATGGACAAATAACTGACACGAGTACATTGAGACACCAAAGA
TGTGCCCAAGTTCATAAAACGCTGGAAGTAAAAATTAGGGCATCCTTTAAAAGGAAAAAAGATGATAAACACGAT
TTAGCCGGCGACAAACTTGGAAATCTCAATTTTGTCGCTGATGTCTCTGTTGTAAAGGGTATACCATTTTATGGT
TACCACGTTGGCTGGAATCTGTTTTACAAAATTTCTTTGCTTAATCCTTCTTGTCTAAGTAGGATTTCGGAATTG
ATTAGAGATGGCAAGATTTTCGGAAAGAAATTCGAAATTTATGAATCACATATTCCTTACTTGCTACAATGGACC
GCAGATTTTAACTTATTTGGATGTTCATGGATAAACGTGGATAGGTGTTATTTTCGCTCTCCTGTTTTAAATAGT
ATACTGGACATAGATAAGCTGACAATTAATGATGATCTTCAGTTGTTACTAGATCGATTTTGTGACTTCAAGTGT
AACGTACTCAGTAGAAGGGATTTTCCACGGGTTGGAAATGGATTGATTGAGATAGATATACTACCGCAATTCATC
AAAAATAGAGAAAAGTTACAGCATAGAGATATACATCACGACTTTTTGGAAAAACTGGGAGATATTTCTGACATA
CCTGTCAAACCATATGTATCCTCTGCTAGGGACATGATAAATGAACTTACCATGCAACGAGAGGAGTTGTCCTTG
AAGGAATATAAAGAACCACCAGAAACAAAGCGCCATGTGAGTGGTCATCAATGGCAATCTTCTGGAGAGTTCGAA
GCGTTTTATAAGAAAGCACAACATAAAACAAGTACTTTTGATGGTCAAATACCAAATTTTGAGAACTTTATAGAT
AAGAATCAAAAATTCTCAGCCATAAATACGCCCTATGAAGCATTACCGCAATTGTGGCCAAGGCTTCCACAGATT
GAAATAAACAATAATAGTATGCAAGATAAAAAGAACGACGACCAAGTTAATGCATCGTTCACCGAATACGAGATA
TGTGGTGTTGATAATGAGAACGAAGGTGTAAAAGGAAGCAATATAAAGTCTCGGTCTTATTCATGGTTACCGGAA
AGCATTGCGTCTCCGAAAGATTCTACTATACTATTAGATCATCAAACAAAATATCACAATACGATAAATTTTTCA
ATGGATTGTGCTATGACGCAAAATATGGCAAGTAAACGAAAACTTAGATCATCTGTTTCTGCTAATAAAACATCG
TTGCTGTCTCGGAAAAGAAAGAAGGTTATGGCTGCGGGATTACGCTATGGAAAAAGAGCCTTTGTTTATGGTGAG
CCTCCTTTCGGTTATCAAGATATTCTGAATAAATTGGAAGACGAGGGATTCCCAAAAATAGACTATAAGGACCCT
TTCTTTTCGAATCCAGTTGATCTAGAAAATAAACCTTACGCGTACGCAGGTAAGAGGTTTGAAATAAGCTCGACA
CATGTATCAACGAGAATACCCGTCCAATTTGGGGGAGAGACCGTATCAGTTTATAACAAGCCAACTTTCGACATG
TTTTCCTCCTGGAAATATGCGTTAAAACCACCTACGTATGATGCTGTTCAAAAATGGTACAATAAAGTACCCTCG
ATGGGAAACAAAAAAACTGAGTCTCAGATAAGCATGCACACCCCTCATAGTAAGTTTTTATACAAATTTGCTAGT
GACGTTTCTGGAAAACAAAAAAGAAAAAAAAGTAGCGTTCACGATTCTCTTACTCATCTGACACTTGAGATTCAT
GCAAACACGAGAAGTGATAAAATTCCAGACCCTGCAATAGATGAAGTTTCCATGATTATATGGTGTCTTGAAGAA
GAAACTTTCCCTTTAGATTTGGATATCGCTTACGAAGGGATTATGATAGTCCACAAAGCTTCTGAGGATAGTACA
TTTCCTACTAAAATTCAACATTGTATTAACGAAATTCCCGTGATGTTCTATGAAAGTGAATTTGAAATGTTTGAG
GCTCTAACTGATTTGGTATTACTTCTTGATCCTGATATTCTTTCCGGTTTCGAAATACATAATTTTTCGTGGGGT
TATATAATTGAAAGGTGTCAAAAAATACATCAATTTGACATTGTCAGGGAACTTGCCAGAGTCAAATGCCAAATT
AAGACGAAGTTGTCAGATACTTGGGGATATGCCCATTCCTCAGGAATTATGATTACCGGAAGACATATGATTAAT
ATATGGAGAGCGTTGAGGTCCGACGTAAATTTAACGCAATATACTATTGAAAGTGCTGCGTTCAATATCCTCCAT
AAACGATTACCTCACTTTTCATTTGAGTCATTAACTAATATGTGGAACGCTAAAAAGAGCACAACCGAATTAAAG
ACTGTGTTAAATTATTGGTTATCAAGAGCTCAGATAAATATACAACTATTAAGAAAGCAGGATTACATTGCTCGA
AATATCGAACAGGCAAGATTGATAGGAATAGATTTCCATTCTGTATATTACAGAGGATCACAATTTAAAGTTGAG
TCTTTTTTAATCCGAATATGCAAGTCCGAAAGTTTTATCCTTCTTTCTCCAGGCAAAAAGGATGTTCGTAAGCAA
AAAGCACTTGAATGTGTGCCTTTGGTTATGGAGCCAGAATCTGCTTTCTACAAAAGTCCTTTAATTGTGCTGGAT
TTCCAATCATTGTATCCATCCATTATGATTGGATACAACTATTGCTATTCGACTATGATAGGAAGAGTGCGAGAA
ATAAACTTAACGGAAAATAACCTTGGAGTATCTAAGTTTTCATTACCAAGAAACATTTTAGCTTTATTAAAAAAT
GATGTAACTATCGCTCCAAATGGTGTTGTTTATGCCAAGACCTCTGTTAGAAAATCAACGTTATCCAAAATGTTA
ACAGATATCCTTGATGTCAGAGTGATGATAAAGAAAACAATGAACGAAATAGGTGATGACAACACTACCCTAAAA
AGGCTTTTGAATAACAAACAGTTAGCACTAAAATTATTGGCGAATGTCACCTACGGTTATACATCAGCTTCATTT
TCTGGACGAATGCCATGCTCTGATTTAGCTGATAGCATTGTACAAACAGGCAGAGAAACATTGGAGAAAGCAATA
GATATTATTGAGAAAGATGAAACTTGGAACGCCAAAGTTGTCTATGGAGACACAGATAGTTTATTTGTATATCTA
CCTGGAAAAACTGCTATTGAGGCTTTTTCTATAGGACATGCTATGGCAGAAAGAGTTACTCAAAACAATCCAAAA
CCAATCTTTTTGAAGTTTGAAAAAGTATACCATCCCTCCATATTAATTAGCAAAAAAAGGTACGTAGGATTTTCC
TATGAAAGTCCTTCGCAGACCCTTCCTATTTTTGATGCTAAGGGTATTGAAACTGTTAGAAGGGATGGTATCCCA
GCCCAGCAGAAGATTATTGAAAAATGTATTCGATTACTTTTTCAAACCAAAGACCTGTCAAAAATAAAAAAGTAT
CTTCAAAATGAATTTTTTAAGATTCAAATAGGAAAGGTATCTGCCCAAGATTTTTGTTTTGCAAAAGAAGTTAAA
TTAGGAGCGTATAAAAGCGAAAAGACAGCCCCTGCAGGTGCCGTGGTTGTAAAAAGAAGAATAAATGAAGACCAT
AGAGCAGAACCCCAATACAAGGAGCGTATACCTTACCTTGTTGTTAAAGGAAAGCAAGGACAACTGCTTCGGGAA
AGATGCGTATCACCAGAAGAGTTCTTAGAAGGTGAGAATTTAGAGTTAGATTCGGAGTATTATATAAACAAAATT
CTGATACCTCCTCTTGATAGATTGTTCAATTTGATTGGTATAAATGTTGGCAACTGGGCTCAGGAAATAGTAAAA
TCCAAAAGGGCGAGCACAACTACTACAAAAGTGGAAAACATAACAAGAGTAGGAACTTCTGCAACATGTTGTAAT
TGTGGTGAAGAATTGACTAAAATATGTTCACTTCAGTTATGTGATGACTGTTTAGAGAAAAGAAGCACCACAACC
TTATCATTTCTCATAAAGAAATTAAAGAGACAAAAAGAATACCAAACACTAAAGACCGTGTGCAGGACGTGCAGT
TATCGTTACACTTCCGATGCAGGCATCGAAAATGACCATATAGCTAGTAAATGCAATTCATATGACTGTCCAGTA
TTTTACTCTCGTGTCAAAGCAGAAAGATACTTGAGAGATAATCAATCTGTTCAAAGGGAAGAAGCATTAATATCT
CTAAATGATTGGTAA

>YPL022W 0.74   Cold

ATGTCTCAGTTATTTTATCAGGGCGACTCTGATGATGAGCTCCAGGAGGAACTTACGAGGCAGACAACTCAAGCA
TCTCAAAGTTCTAAAATTAAAAATGAAGATGAACCCGACGACTCCAATCATCTTAATGAGGTGGAAAATGAAGAT
AGCAAAGTTTTAGATGACGATGCAGTGTTATACCCTCTTATACCTAATGAGCCAGATGACATAGAAACGTCTAAG
CCCAATATTAACGATATTAGGCCAGTTGATATTCAATTGACTTTACCATTGCCGTTTCAGCAAAAAGTGGTAGAG
AATTCATTAATTACTGAAGATGCATTAATCATAATGGGGAAAGGACTAGGATTGCTTGATATTGTGGCCAATTTA
TTGCATGTTTTAGCTACACCAACATCCATTAACGGACAACTAAAGCGAGCGCTCGTCCTAGTGTTGAATGCAAAA
CCTATAGATAATGTAAGAATCAAGGAGGCCTTAGAAGAGCTGTCGTGGTTCTCTAATACTGGGAAGGACGACGAC
GATACTGCTGTCGAGAGCGATGATGAACTTTTTGAAAGGCCTTTTAACGTAGTTACCGCGGACTCGCTGAGCATT
GAAAAGAGAAGAAAGCTATATATTTCTGGCGGAATCTTGAGCATTACTTCTAGAATTCTCATTGTGGATCTCTTA
TCCGGCATTGTTCACCCAAATAGGGTTACGGGTATGCTGGTATTGAATGCAGACTCACTTCGACATAATTCGAAT
GAATCGTTTATATTAGAGATTTACAGGTCTAAAAATACTTGGGGTTTTATTAAAGCCTTTTCTGAAGCACCAGAG
ACGTTTGTCATGGAATTTTCACCCCTCAGGACGAAAATGAAAGAATTACGGCTAAAGAACGTTTTGCTATGGCCG
AGGTTCAGGGTAGAGGTCTCTTCCTGTTTGAATGCCACTAATAAGACGTCACACAATAAAGTCATTGAAGTCAAG
GTCTCCTTAACAAATTCCATGTCTCAGATACAGTTTGGCTTGATGGAATGTTTGAAAAAATGTATTGCTGAGTTA
AGCAGAAAAAAATCCTGAACTAGCTCTGGACTGGTGGAATATGGAAAATGTCCTGGATATAAACTTTATCAGGTCA
ATTGACTCGGTGATGGTGCCGAACTGGCACCGAATTTCTTATGAATCAAAACAACTGGTTAAGGATATAAGATTC
CTACGCCACCTTTTAAAGATGCTCGTAACTTCAGACGCAGTTGACTTTTTTGGAGAGATTCAATTAAGTTTGGAT
GCCAATAAACCGTCAGTATCCCGAAAATACAGCGAATCACCGTGGCTATTGGTCGATGAGGCACAATTAGTCATA
TCGTATGCGAAGAAAAGAATATTTTACAAAAATGAATATACTTTAGAAGAAAATCCAAAATGGGAACAACTTATT
CATATATTACATGATATTTCACATGAGAGAATGACCAATCACCTTCAGGGGCCTACTTTAGTTGCCTGTTCCGAC
AACCTTACATGTTTAGAACTAGCAAAGGTCTTGAATGCCTCAAACAAAAAAAGAGGAGTACGTCAAGTGCTTCTG
AATAAATTGAAATGGTACAGAAAACAGAGGGAGGAAACGAAAAAAATTGGTCAAAGAAGTGCAAAGTCAGGACACT
TTTCCAGAGAATGCAACATTAAATGTAAGCTCGACATTTTCCAAAGAACAAGTGACCACGAAAAGAAGAAGGACA
AGAGGTGCTTCACAAGTTGCGGCCGTTGAAAAGCTAAGGAATGCAGGTACCAATGTAGATATGGAGGTGGTTTTT
GAGGATCATAAGTTATCTGAAGAAATTAAGAAGGGAAGCGGTGATGATTTGGATGACGGTCAGGAAGAAAATGCC
GCAAACGATTCAAAGATTTTTGAAATACAAGAACAGGAAAATGAAATCCTTATCGATGATGGGGATGCTGAATTT
GACAACGGAGAATTAGAGTATGTGGGCGACCTTCCGCAGCACATCACAACCCATTTCAATAAGGATTTATGGGCA
GAACATTGCAACGAGTATGAATATGTTGATCGTCAGGACGAAATTTTAATCTCTACGTTTAAAAGTCTCAATGAC
AATTGCTCATTGCAGGAGATGATGCCCTCTTACATTATAATGTTTGAACCAGATATATCGTTTATCAGGCAGATT
GAAGTTTATAAGGCCATAGTGAAGGATTTGCAACCAAAAGTATACTTCATGTACTACGGTGAAAGTATTGAAGAG
CAAAGTCATTTGACTGCTATCAAGAGAGAGAAAGATGCTTTCACAAAGTTGATTAGAGAGAATGCAAATCTGTCC
CATCACTTTGAAACGAATGAAGATCTTTCTCACTACAAAAATTTAGCTGAAAGGAAGTTGAAGCTTTCAAAATTA
CGAAAATCTAATACCAGAAATGCGGGTGGGCAGCAGGGATTCCATAATCTTACTCAGGATGTCGTCATTGTGGAT
ACACGTGAGTTTAATGCCTCATTACCAGGCTTACTCTACCGATATGGCATAAGGGTTATTCCTTGTATGTTGACA
GTCGGCGATTATGTGATAACTCCTGATATTTGTCTCGAAAGAAAATCGATTTCTGACTTAATTGGGTCATTACAG
AATAACAGATTAGCCAACCAATGTAAAAAAATGTTAAAATACTATGCATATCCGACACTATTGATTGAGTTTGAT
GAAGGACAGTCGTTTTCTTTAGAACCTTTTAGTGAACGTAGAAATTATAAGAATAAAGACATATCAACTGTTCAT
CCTATATCAAGCAAGTTATCCCAGGATGAAATTCAGCTAAAACTAGCCAAATTAGTATTGCGGTTTCCCACTTTA
AAGATTATATGGTCTTCCTCACCCCTGCAAACTGTAAATATAATCCTAGAGTTGAAATTAGGACGTGAGCAACCT
GACCCTAGTAATGCAGTTATATTGGGAACGAATAAAGTTAGATCGGATTTTAATAGCACTGCAAAGGGCCTGAAG
GATGGTGATAACGAGTCTAAATTCAAGAGACTGTTGAATGTTCCTGGAGTGTCAAAAAATTGATTATTTCAATCTC
CGCAAAAAGATCAAGAGCTTCAATAAGCTTCAAAAGCTTTCATGGAATGAGATTAATGAACTTATTAATGACGAA
GATTTGACGGATAGAATATACTACTTCTTGAGAACAGAAAAAGAAGAACAAGAACAAGAGTCAACAGATGAAAAT
CTTGAATCTCCTGGTAAGACCACTGATGATAACGCTTTACATGATCATCATAATGATGTTCCTGAAGCACCTGTG
TAA

>YIR023W 0.68 Cold
ATGGACCCTCACCAATCACCAGCTGATAATGCCGCATCGCCTACGAAGAGCGTGAAGGCAACCACTAAAAATTCG
TCCACGAATAATAATGTCAATAGCAACAACAGCAATAATAACAGTAACCATGATATACTGAATTTTAATGATAAC
TATACTACCATTCTGCAACATTTGGCAAACGACCATCCTAATATACTGAGGGAGAAAGGAGGATCACAACAACAA
CAGCATCAGCAGCAGCAACAACAGCAACAACAGCAGCAACAACAGCAGCAACAACAGAGCCTGGATACCCTTTTG
CACCATTATCAAAGTTTACTCTCCAAGAGTGATAATGCAATAGCCTTTGATGACAATGTTAGTAACAGCGCAGAT
CATAATGGCAGTAATAGCAACAATAATAACAACAATAATGACATATCTAGTCCCGGTAATCTGATGGGATCTTGC
AATCAATGTAGATTAAAGAAAACAAAGTGCAACTATTTTCCCGACCTAGGCAACTGTCTCGAATGTGAAACGTCA
AGAACTAAATGCACCTTCAGTATTGCTCCAAATTATTTGAAAAGAACGTCCTCTGGGGCAAACAATAACATGCCA
ACCTCTTCAAACTCCAAACGAATGAAAAATTTTGAAGACTATTCGAATCGCTTACCCAGTAGCATGCTCTATAGA
CATCAACAGCAGCAGCAACAGCAACAGCAGCAACAGCGTATACAATACCCCAGATCCTCTTTTTTCGTCGGTCCG
GCCTCAGTTTTCGATTTGAACTTGACCAAGCATGTAAGACTAGATAATGTTGACCAAATACAGCTATCAAAAACT
TTATCATTAAGAAAGGTGTCTCCTACCGCACAGTTTATTCTTCAGGACGATTTTGACACGACCCTTCATAGTAAG
CAAGAATACGAAGTTGATTTGGTCGAAAATCTGGTACATCCGCACGGTCACCTTTTAGTAGAGATTTTCTTCAAA
TTGATTCATCCTTTTCTTCCAATTTTACACGAACGTGTATTTTTGGAAAAGTATTCTAGATCTTATAGAGAACTC
ACTGCGCCTTTACTGGCATCCATTTACTCACTGGCATTACAATATTGGGATTTTCATCCTGCACTTTTGGGGTTC
CCTAAACCCGATGTAACTGCTCAACTAAACAATATCGCATTGGAAACATTTTATGCTCGAGTTGGAAGGCCAAAA
TTGAGCATTATACAAACAGGCCTTTTGATTTTGCAATGCCGTAGTGAATGTCATAACAACTGGGTCCTCTGCTCC
AGCGTAGTGGCCCTTGCAGAGGAATTGGGGCTCGGGGTTGAATGTAATGATTGGAAACTTCCCAAATGGGAAAAA
GATTTGAGAAAGAGATTAGCGTGGGCAGTTTGGTTGATGGATAAATGGTGTGCATTAAACGAAGGTAGACAATCC
CATTTAATTTTAGGTAGGAACTGGATGATCAAGCTATTAAACTTTGATGATTTTCCTCTGAATTCGCCCACTATT

TTAAATTCCTTGCAAAATGACCAGTCGGGGTCTTCACCCTCGTCATCAAATGATGTGAAAAACCATCAGATAGCA
TTTGGTAACTTGCCCATATTTAACATAAACCCCACTTTAGAAGATTTCAAGAATGGAACGCTTATGTTTCAACAA
ATGGTTTCCTTGAGTATAATTCTTGGGGAAATCATGGACACATTTTACACTCAGGGATCCATGACAATAAACAAA
AGCATTGAACAGGTATTGAAACTGGCAAAGCCGTTGCAACTGAAATTAAGAGAATGGTACCATTCCCTACCCAAG
AATTTGTCAATGAGTTATGCCACGCCACAAAAATTGAACTCAAATTCAACTTTAACTCTAGCTTATTTTGCCACG
GAAATTACGTTACATAGGAAAATTATCTGTGCCTTGAATCCTCAAACTCCAAAGGAACTGGTTCAAGTATGCAGG
ACTGCTGCAAGAACCAGGCTTGTAGCAGCAATTGAATTTATAAGAGATCTAAAAAACGAACATATTAATGCTTTT
TGGTATAACTGCTCAACTGGAAATCTGATGCTAATTGGAACATTTGCTGCCCTGCTGTATGTGACTTCAGCTACG
AAGGAAGAGGCTATGATTTTCAGAGATTATGTTAGAAATTATACCTGGGTATTGAAGATAGGTTCCAAATACTTT
GATAAACTATCGAATGCTTTGAATAATATGCATTTACTATTTGCACAAATTCCTGGTCTTCTGACCGATGAACCG
GTGGTCGTGAGCCCTAACTCTAACATCAATTCCGTTAACCCACAAAGGTCAGGTGTACAATCGCAAATCCCAATA
CAATTTAACGTGGGATCACCTGCCATGACCGAGCAAGGATCTCCTTTAAACCAGTGGAAGAATTTGCCCCAGGAA
ATCTTGCAACAACTAAACTCCTTTCCCAACGGTACGACCAGTACAACAACGCCAGTAAACCCCACTTCCAGACAA
ACACAACTAGAATCGCAGGGAAGTCCAGCCATAAATAGTGCTAATAATAATAGCAACAACACGCCTTTACCATTT
GCGCCAAATAAGTCTTCTAAAAAAACTTCACAATCTTCACCAAATGTTACGCCCTCTCATATGAGCAGACACCCC
CCCTCTAATACATCTTCACCAAGAGTCAATTCCAGCACAAATGTCAACTCTAATACACAAATGAATGCTTCTCCC
TTGACCTCAATTAACGAAACCAGACAAGAGTCAGGTGACGCTGCAGATGAAAAAACAGCAGGAAGAGAAAGAACC
GCCAACGAGGAATCATCAACGGAACTCAAGGACGATAATCCAAACTCGAACCAGGAAACAAGCGCAACTGGTAAC
CAAACTATAAAAATGAATGATGATAAAAATGTAACAATTAACACAAGGGAAACCCCTCTGTAA

>YNL026W 0.67 Cold
ATGACCTCATCATCTGGTGTTGATAATGAGATTTCCTTAGACAGCCCGATGCCAATTTTCAATGAGAGTTCTACT
TTAAAGCCAATTAGGGTTGCGGGTGTAGTAACAACAGGGACAGATCATATCGACCCTAGCGTACTACAGGCATAC
TTAGATGATACTATTATGAAATCCATTACTTTGGGGCAGCTAGTGAAAAATGCGGATGTGTTAAATAAGAGATTG
TGCCAACACCACATTGCCTTGAACGCCAAACAGTCGTTTCACTTCCAAGGCAATACATATATCTCCGATGAAAAA
GAAACACATGATGTGGTGCCTTTGATGGAAGTTGTTTCACAATTAGATATATTACCTCCTAAAACGTTTACAGCG
AAGACAGGGACAAATTTTGGGAACGATAATGATGCAGAGGCTTACTTACAATTTGAAAAACTAATAGATAAAAAA
TACTTAAAGTTACCTACTAGAGTCAATCTCGAAATTTTGAGAGGTACAAAAATACACTCCTCCTTTCTATTCAAT
TCTTACTCTTCTCTTTCTCCCCAATCAATTCTAAACTTGAAAGTTTTAGTCAATTTTATAATTGGAACACAAAT
AAAGGATTAGATATTGGGCAAAGAGGAGCAAGACTATCTTTGAGGTACGAGCCACTGTTCTTACATAAACTATTA
CACAATCCGCATTCTAATGAAAGTCCCACGTTATTTCATGAGTGGTTTTTAGAGACCTGCTGGAGATCCACTAAG
ATATGTTCACAAGGCACTAGTGCGCCCTATATGTATTCAGGTACAATGTTATCTCAAGCAGGCGATCAGTTAAGG
ACAATTTTGGGTCATACATTCGTGCTGGACAAGAGAGATCATATTATGTGCCCTACTAAGGGTTCGATGTTGAAG
TGGAGTAATGAACTTTCGCCTGGAAAGCATTTAAAGACCCAATTAGAATTGAACAGTGTAAAAAGTTGGATGAAT
GACGACTTTATTACTTTTTCTACTACCATTAAAACAGGATATCTAAAGAACTTATCCTCACAACAATCCTTGCCT
GTACATATATGTGATAAGTTTCAGAGTGGTGGGCCCAGCGACATTCGGGGGTTTCAAACATTTGGGTTAGGCCCC
AGAGATTTATATGATGCAGTAGGTGGTGATGCTTTTGTTTCCTATGGTCTTAGCGTATTTTCTCGATTGCCCTGG
AAAAAAGTTGAAAAGTCAAACTTTAGGTTACATTGGTTTTTTAATGGGGGTAAATTGGTTAATCATGACAATACG
TCGTTGGGTAATTGCATAGGACAGCTTTCAAAGGAACACTCCACATCCACAGGTATTGGTCTTGTACTAAGGCAC
CCAATGGCAAGATTCGAGCTAAACTTTACTTTGCCTATTACCGCTCACGAAAATGATTTGATAAGAAAAGGATTC
CAGTTTGGTCTTGGTCTGGCATTTTTATAA

>YHR197W 0.82 Cold
ATGTCAGAGGAATTTATCGCAGTTTCTACTTTGGCTAGAAACCTGGAAATTGCCAAGGGTAATGAGTTTCATACC
ATTTTGGCGACTTTAAGATCACCAGTTTATATCAATGAACAGCTGCTAAAATCCGAATTGAGCTTCCTTGTTACA
AAAATCCTAAAACTTATCAGATCAGGCAATGACTTTGATCTTTGGAAAGGATGTCACACCTCTGTTGTGACTTGC
GCCTATAATCCACTGGTCTTATCGACTCATGGAGGTCAATTGCTTGCTGCCATTTATTCCAGATTGGAACAGAAG
ACGGGTTTCTATTCTTCGGTTATTTCTTCATCACATGGAAAGCAGCTATTCAATACCCTTATTTCTTCTGTAGCG
ATTATCATTGATTTAATGAAGAATAAACCTACGTTATCGAGAGAAGCACTGGTTCCCAAATTAAAGGCTATCATT
CCAACGTTAATTACATTGTCTCAATACGAGCCCGAACTTGTTCTACCAGTACTACAGAGGATATTGAAGAGAAAC
ACTACAACATTTAAGCCATTTACCAACAAATTCCGCACTGTGTTAATCAATTTAATCATATCAGACTATGCTTCT
TTAGGCACTAAGACGCAGAGGCTTGTTTGCGAAAACTTTGCTTATTTGCATTTGCTGAAAATTCAGGTTAGTGAT
ACTAGCGATGACGAAACTCAAGCACATCACAAAATATATGCTGATTCTAATTGGAGAACAGGATTAATGTCCATC
TTATCACAATTCAAGCCCATAATTCAATTATGTGGAGAAATCTTGGATTTTGAACAAGATAATGAGCTGTATAAA
CTAATTAAAAGTTTACCGGTTATCGATGAATCCAATAACAAAGAAGAATTTTTACCCTCATTGAAATTAGACTTC
AACGCACCTTTAACTTTATGGGAAATTCCACAACGTTTGTCCTTATTGGCTGACATGCTTGTTGCTTTCATCTCT
TTACCAACTCCCTTTCCCATTCGAGTTCCGCTGGGTGGTATTAATTCCCTTTGTGAAGTTTTGTTGGGAGTTAGT
AACAAATATTTACCGTTAAAAAAAGAATTGCGTCATGATAATGAACTAAATGGGGTCATCAATACCATCTTACCT
CAAATACAATTTCAAGGCATTAGGTTATGGGAAATTATGGTTTCAAAATATGGCAAATGCGGTTTATCATTTTTC
GAAGGAATCCTTTCTTCCATTGAACTTTTCATTCCATTAAAAAAGAAAAGTAACAATGAAATTGATTTTAATGTT
GTTGGGTCTTTAAAATTTGAGTTTGCTACTGTTTTCAGATTGGTAAATATGATTTTGTCTCATTTAGGCCACCAA
CTGAACATAATCAGCGTCATATCCCAATTAATCGAAGTGGCGTTGTTCTTATCTCATGATAAAACATTGATTGAT
TCTTTATTCAAAAATCGTAAAAGCATCATGAAACAACAGACTAAAACAAAACAATCAAAGAGGAGTAAAAGTGCC
GAAGGTGCATTTTCTGATATTTATACGCACCCAGAGCTATTTGTGTGTAAAAATTCCATGAATTGGTTCAATGAA
ATCAATGATTTTTTTATTACTGCACTAAATAATTGGATTTTGCCTTCAACTCCACATATTCAAATTTTGAAATAT
AGTATCACGCAATCTTTAAGATTAAAGGAAAGATTTGGTTATATTCCAGAAAGCTTTGTTAATCTCCTGCGTTGC
GAGGTTCTTCACCCAGGTAGTGAACGTGTTTCAATTTTACCGATTGCAATATCACTATTAAAGAATATCAATGAT

GATATGTTTGAATTACTGTGTCATCCAAAAGTGCCAGTCGGTATGGTGTACCAGTTGCACAAACCTTTGGACTTA
GGCGAAGACGGGGAAGTAAGGGATGATATCAACAAGAAGGAGGTTGAAACGAACGAATCATCCTCAAACGCCAAT
ACTGGCCTGGAGACTTTAAAAGCGTTAGAAAATTTGGAAAATGTTACAATCCCTGAACCCAAACACGAAGTCCCA
AAGGTCGTCGATGATACTGCCATATTCAAGAAAAGGTCAGTAGAAGAAGTCATTGAAAGAGAGTCTACATCTTCA
CATAAGAAAGTGAAATTCGTTGAAGAAACAACCGTGGATAATGGCGAAGAATTGATTGTAAAAAAAGCTGTAAGC
CAAACTAAGGAAGAAGAGAAGCCAATGGAGGACAGTGAAGATGAAGAACAGGAGGAGTTTGAAATTCCCGCTATC
GAATTAAGTGATGACGAAGAGGAGGAGGAAGAAGGAGAATAA

>YOR284W 0.82 Cold
ATGCAACAAACAAAATTTGGCAAGATGTATTTGGACCACGATTCGGTAGTTGAGTACTCAGAAGATGAAATTGTC
GAAGCCGATCGTATTACATTGGGCTATAAAAAGAGATTGTCAATGATTGAAAATCAAATGCGACATCTTTTGGAG
GATTTTAGTTTGGACGTTCAGCAGATTGAACCCATTCTTGCCGACTTGCAGAAGTACTACGACGCGTTTCTACAA
CTTTTACAGAAAAGGAACAAATCATTACAATGTAAGAGGTCCACTCACCAGCCGGTCCCATCCCCAATGAATAGC
CAGACATCAACAAATGCAAAGGTTAATTTGAGCGGAAAGCTTATGAAGTTTCAGCTAAATAGTGTACAAAAGTTC
GACGAAGAAAATATACTAAGGATTCTGCAAAATAAAATAGAATTCGAACATTATTTCCAAATTGATAAAGGTAAG
AAGCAAAAGGTGCTATTACTTGCCGTATATCAATGCCTCAATGGGCCAACACGTTTGCATAAAGTGCTTAATATT
GAGGGAATTATACATAATAATAGTATCAGAACTATACTGGGCAAACAAGTGTCCAGTTCTAAGTGGACAGTGTTT
CTGTACGATGTAAAACTAGTTTTATTGGCGCATAGACAAGATGTTCCGAACTTAGAAACTAGCAAGATGATCGTC
AGATATGGTGATTTATTTCCCTGTGCATTGTACTTCAAAGATCATACAGCATACTAA

>YNL211C 0.8Cold
ATGCCTCCAAGAAGTATAGAAGAATGGTTTTACTACAAGTTACTGAGTAGTCCAGGTTTCCATAGATTTGTACGC
AAGGTATATAGGAAAGTAAATGGTATTAAAGAAGATCCTTTCACTGACCAATCAACTGCTTTTCAATATCTTTAT
AAGCCAACTCCACGACAGAAATTTAAGGCTTTAAGGCTTTTATTTTGGGATGAAATGAGATCTACTTTTGGGTTC
AGAAGGAGACTAGGAGACCGCTTTAAAAAAGATTAA

>YDR389W 0.79 Cold
ATGCCAAATAATACTCTTAAACAAGGCTCCAAAATTGAAAATGTTTCTCCCTCTAAAGGTCATGTCCCAAGTTTT
TGGAAGCAGTTCATAAACAACCCTAAGAGTATGTCATCCGAAAATATTACAGTCCCTAGATCACCAACTTCTCTT
TCAAGGAATGCTCAACCAACTACTTTAAAACGGCCTCCATTATCTTCAAGACCATATTCATATAATACCCCAACC
AAAGATAGGAAATCCTTTTCCAAATCCGCGAAACAGAACAATAACAATAACAACGCTAATTCCGGAACATCGCCT
CACGCAGAGTTCAAAAATTACAGAGATATGTTTTTATCTAATAGAAATGGTTTCACAGGTAGGGTTTTCGGTGTT
ACTTTAGCAGAATCGTTGAGCGTCGCCAGTGCAGAGGTCATTGTTCAAAGTGAGTTGGTGAGTTTTGGTCGGATA
CCCATCGTGGTAGCCAAGTGCGGCGCATACTTAAAAGCAAATGGATTGGAGACCTCGGGTATATTTCGTATAGCG
GGCAATGGTAAAAGAGTAAAAGCCCTTCAATACATATTCTCGTCGCCACCTGATTATGGTACCAAATTCAACGAT
TGGGAAACATATACAGTGCACGATGTTGCATCGCTCCTGAGGAGATACCTTAATAATTTGGCCGAACCACTAATA
CCTTTATCCCTATATGAACAATTCAGAAACCCGCTACGATCTAGACCAAGAATCCTAAGGCATATGTTGACCCAC
GAAGTTTCTCATCCGAATGCGAATAAAACAAATAATGTAACAGTAAAATCAAGTAGACAGAACTATAATGATGAT
GGTGCTAATGATGGTGACATCGAAAAGGAGGACGCTAAAGATGATGAAGAAAAAAGACGAAGAAAAATACGTCAT
AAGAGAAGGCTGACCCGCGATATAAGGGCAGCTATCAAGGAATATGAAGAGCTCTTTGTTACCTTATCAAATGAC
ACGAAACAGCTAACTATATACCTACTCGATTTACTGAGTCTTTTTGCAAGGCAATCACAGTTTAACCTAATGTCT
GGTAGAAACTTGGCGGCCATCTTCCAACCTTCAATATTATCGCATCCTCAACATGATATGGACCCCAAAGAATAC
GAGTTATCCCGACTAGTAGTGGAATTTTTGATTGAATACTCGTACAAGCTATTACCCCATCTTTTGAAGTTGGCT
AAAAGGGAACAACAGGAACGGTTGTCAACCGAAAATAAGAAAAATAATGGAGATAAACAGAAAACTGATCCTATA
GAAATACCAAAGATCACCTCATCGGATTCGCCACCAATAGTTTCTTCCAATAAAAACCCACCGGCGATTGACAAT
AACAATAAACTAGACCATACGACGTTATCGCCAATATCTACTTCGATACCGGAGAACTCATCAGACCTACAGACT
TCAAAAATGTTAAAGCCTCCGAAGCAAAGAAGACCACACTCAAAATCGTTTGGTTCTACTCCGGTTCCTCCGGAT
GTTATTGCTAGTAATAAAAGAAGAACGAGTTTATTCCCGTGGTTACATAAACCGGGAATTTTGAGTGACACAGGA
GACAATGGCGACTTAACTGCTACTGAAGCTGAAGGTGATGATTATGAAGAAGAAAATGTTGATCCATACGGTCAG
TCTCCATCTAGCGTACACTCAGGTTCTTTGCCCAAACAGCATTATCTGCCAATTCCTCGAATGAACAGATCGTTG
AGTGGAAACAGTACCAATTCGTCCTTTAACACTAGACCTATTTCAATGATCCTGACCAGTGGTAATGATAATTCC
GCTGATCAATTAGAATTACTAAGTAACACTCATAGCAATAATGAGCGCAGTAATGCTTTACCATTAACCGAAGAT
GATGGGGATGAAAGAAATTCAAGATCACGCAAGAGGGAATCATGGTTTCAAAGACTAACAAGCCGTTCAGGCTCT
GCTAACAGGGCTTGA

>YLL061W 0.78 Cold
ATGGATGAATTTGAATCTACCAAACTTTCGAAGGTTCAGTTTTCCACTAGCGTGTTAAGTACGCCTTCGAATGAA
GGAAATAATCTAATACACAGATTCAAGAACTCTTTCAAGCGAAATGATTCACCAGCGATCCAAGAAGGTTTGCTG
TATTCGGAGCTTTCAGAGGAAGAAAAAAATACAATGGGACTTAGCCAACCAGCCGTATAAAAAGGTCCTAGATCAA
AGACACCTGACCATGATAGCCATTGGAGGTACTCTAGGGACGGGTCTTTTCATTGGTTTAGGGGAATCACTAGCG
TCCGGACCTGCTTCTTTGCTAATTGGTTTCTTGTTGGTCGGCGCATCTATGCTCTGTGTTGTTCAGTGTGGGGCC
GAACTTTCATGTCAGTACCCTGTTTCCGGCTCCTATGCGCTGCACGCCAGTAGATTTATAGACCCATCAGTCGGC
TTCAGTATAGGAATTAACTACTTATTGATGTGGCTCATATCTTACCCTAGCGAGCTGGTAGGATGTTCGCTTACC
ATCTCTTATTGGGCACCATCGGTCAATCCTGCTGCCTGGGTTGCAATCGCGTTCGTTTTAAGCATGCTATTGAAT
CTTTTTGGGGCAAGAGGCTTTGCAGAATCAGAGTTTTATATGTCAATTTTCAAAATTGTGGCTCTATTCATTTTT
ATAATTATTGGTATTGTTCTCATTGCAGGTGGTGGGCCTGATTCTACTGGATACATCGGCACGAAGTATTGGCAC
GATCCAGGCTCCTTCGCTGTTCCTGTTTTCAAAAACCTCTGCAATACTTTTGTTTCGGCAGCATACTCCTTTAGT

GGTACAGAAATGGTTGTTTTAACCAGCACTGAAGCAAGGAGTGTTTCCTCAGTCTCTCGGGCAGCGAAAGGTACG
TTCTGGAGAATTATCATCTTTTATATTGTTACAGTGATTATAATTGGTTGCCTGGTCCCTTATAATGATCCTCGT
CTGATTAGCGGTTCATCCAGTGAGGATATTACTGCTTCCCCGTTTGTTATTGCCTTGAGCAATACTGGTGCTATG
GGAACAAGAGTGTCTCATTTTATGAATGCGGTCATCTTGATAGCCGTATTTTCTGTTTGCAATTCCTGCGTTTAC
GCATCCTCTAGATTGATTCAGGGTTTGGCGACAGCAGGCCAACTTCCAAAGATCTGCGCTTACATGGACAGGAAT
GGTCGGCCTTTAGTTGGCATGGCTATATGTGGCGCTTTTGGATTGTTAGGCTTTTTGGTTGTTTCCAAGAACCAG
GGTACAGTTTTCACATGGTTGTTTGCGTTGTGTTCCATTTCATTTTTCACAACTTGGTTCTGTATTTGCTTCTGT
CAAGTCAGATTTAGGATGGCAATGAAAGCTCAAGGAAGGTCAAAGGATGACATTATCTATAGATCGACATTAGGG
ATATACGGAGGAATTTTCGGCTGTATCTTAAATGTTCTATTGGTAATTGGAGAAATATATGTATCGGCTGCACCC
GTGGGTAGTCCCAGTTCTGCTGCTAATTTCTTTGAATATTGTATGAGTATTCCCATAATGATTGCTGTATATATT
GGCCATAGAATTTACCGCAGAGACTGGAGACACTGGTACATCAAGCGGATGGATATTGACCTCGATAGCGGACAT
TCGTTAGAGGACTTCGAAGCCACCAAGCTTGAGAGGGATGAGGATAAGAAATACGTTTCCTCTAAGCCACTTTAC
TATAGGATCTATCGGTTTTTCTGTTAA

>YNR054C 0.82   Cold
ATGAGTGAAAAAGTAAATAGTGACTTTGAAGACTTTTCCTCAGATGAGGAAACCGATCAACATAACGTCTTAATT
CAGACCAAGAAAAAAATTTCTAGTAAGGATGATATCTTCAGTAAGAAGGTAGAAGACATCGAAAGTGAAAACGAG
TCAGACATAGAAGAAGAGCAAAAACAAGAAGAAAAAGAGGATGTAGAACAACCAGATAAAGAGAATGGAGAAAAG
CTCGATCGAGAAGTAGAAGAACAAGCTTCGTCCACCACTTCACTGGATTTAAAAACAGAAAAACTAAGGCAACTG
GTAAAATCAAAGGCCGCCAAGAAGTCTAAACACAAGACTGGGGTGGTATACTTCTCCAGTATTCCACCTTACATG
AAACCTGCAAAAAATGAGACAAATATTAACTCGTTTTGGTGAGGTAGATAGACTATTTCTAAAGAAAGAGGACGAT
CAAAAGTACAAGCAAAGAGTTAAAGGTGGAGGAAACAAGAAAAACAAGTATGAAGAAGGTTGGGCCGAGTTTATT
AGGAAAAGAGACGCCAAATTGTGTGCGGAGACGTTGAACGGGAACATTATTGGTGGCAAGAAAGGTACTTTCTAC
CATGATGATATCCTGAACGTGAAGTACCTCCCAGGTTTCAAATGGGCAGATTTGACTGAACAGATTGCCCGCGAG
AACGATATCAGACAGGCCAAGTTGGAGATGGAAATCTCGCAGGCCAACAAACTGAACGCGGAATTCATTAGAAAC
GTGGAGCAAAGTAAGATGATACAAAACATTAAGAACTCTAGGAAGCGCGCCGGAAAAGAAGGAGAGTCTGCAGAT
TCTCACCCTCACAGGGAATTTAAACAACGTCGTGTGGAAACAAGCCGTGCCAATGCTCCCTCTGATATCAAGCAG
CAATCCTCTGGGTCCAAAGACTTGGGTAATGTTCTCACCAATTTACTATAA

>YGL250W 0.81   Cold
ATGAGTGAAGAAGAAGCGCATAAAACAGTGGAGGTGGATGACGTCGGGGTTCAATTGGATGAGGGCGATGAAGAA
GACCTTTTGGAGTATGATGATGAACTCGTGGAGGAACAACCATCGGACGCCCGTATACGCAACGTGGCAGAGACT
TTAATGAAAAGCGAATTGCCCAAAGTCACCGTGGAATATAAAGATACTACATTTCTGTTGTTCACGTCGGATGAT
AAGAACGAAAGTAACAACCCCATAATATGTGAAAACGCTGCTCTTTATCAGCGACCCATGGGGGAATTCATGGAA
TCCATTCGTAAATTTATGGGAAATCGTTTTGGCAGGTTGGCTTTTGCTACGAAGGAGTTGGTTTTACAACTGAAA
AGCCTCGATTTGACCTTGTTCGAAGATAATGTCTACAACAACCATATTTCATTCAGCGATGTTTATACTATATTT
AAAATCCTGAAGGAAAGATCGGAGTCTAATTTTGAAACTGATATACCGACGCACTTGGCTATTGAATTAAGTACA
AGGCCAAGATTTGTCTCTCGGTACAACGCCTTGGTTGAATTAACTGAAAGCTCAGCTACGTTGAAAAACATAAAG
CCATTTTCTAACGATGAAACGCATCCCTTAATTGTGGATGACAATGACCAATATACACACCAGAATACTTCAGAA
GTCATTGTAATGGACATAGATGATGACGTAGGTGAGGATTCTGAAGACTAA

>YIL172C 0.79   Cold
ATGACTATTTCTTCTGCACATCCAGAAACAGAACCCAAGTGGTGGAAAGAGGCCACAATTTATCAAATTTATCCC
GCAAGTTTCAAAGACTCCAACAACGATGGTTGGGGTGATATGAAGGGTATTGCCTCCAAGTTGGAGTACATCAAA
GAGCTTGGTACCGATGCCATTTGGATCTCGCCATTTTACGACTCGCCACAAGATGATATGGGTTACGATATTGCC
AACTACGAAAAGGTCTGGCCAACCTACGGTACGAACGAGGACTGCTTTGCCTTGATCGAAAAGACACATAAGCTT
GGTATGAAATTTATCACCGACTTAGTCATCAACCATTGCTCCAGCGAACATGAATGGTTCAAAGAGAGCAGATCC
TCAAAAACCAATCCAAAACGTGACTGGTTCTTCTGGAGACCTCCTAAGGGTTATGACGCCGAAGGCAAGCCAATT
CCTCCAAACAATTGGAGGTCTTACTTCGGTGGTTCTGCATGGACGTTCGATGAAAAGACACAAGAGTTTTACTTG
CGTTTGTTTTGCTCCACCCAACCTGATCTAAACTGGGAGAACGAAGACTGCAGAAAGGCAATCTACGAAAGTGCC
GTTGGATACTGGTTAGACCATGGTGTAGACGGCTTTAGAATTGATGTGGGAAGCTTGTACTCCAAGGTTGCTGGT
CTACCAGACGCTCCTGTGATTGACGAAAACTCAAAGTGGCAACTCAGTGATCCTTTCACAATGAACGGACCACGT
ATCCATGAGTTTCATCAAGAAATGAACAAGTTCATCAGAAACAGAGTGAAGGATGGCAGAGAAATTATGACAGTT
GGTGAAATGCGACATGCTACTGATGAGACCAAGAGGTTGTATACAAGTGCGTCAAGACACGAACTTAGTGAGTTA
TTCAACTTTTCCCACACTGATGTCGGGACTTCGCCCAAGTTCCGTCAAAATTTGATACCATATGAACTAAAGGAT
TGGAAGGTTGCCCTTGCTGAACTTTTCAGATACGTTAACGGAACTGATTGTTGGTCAACAATTTATCTGGAAAAT
CACGACCAACCTCGTTCAATTACCAGATTTGGTGACGATTCTCCCAAAAACCGTGTTATTTCTGGTAAGTTGTTG
TCTGTGTTGTTGGTGTCACTGAGCGGTACTCTATATGTGTACCAAGGACAGGAACTGGGTGAAATCAATTTCAAG
AACTGGCCTATCGAAAAATACGAGGATGTCGAAGTTAGAAATAACTATGATGCGATCAAGGAAGAGCATGGAGAA
AACTCGAAGGAGATGAAGAGGTTTTTGGAAGCAATTGCCCTTATTTCCAGAGACCATGCTAGAACACCTATGCAA
TGGTCTCGTGAGGAGCCAAATGCTGGTTTTTCTGGTCCTAATGCTAAACCATGGTTTTACTTGAACGAGTCTTTC
AGGGAAGGAATTAACGCTGAAGACGAATCCAAGGACCCCAACTCGGTTTTGAACTTCTGGAAGGAGGCCTTGAGA
TTTAGAAAGGCACACAAGGATATTACTGTGTATGGATATGATTTTGAGTTTATTGATTTGGACAATAAGAAACTG
TTCAGCTTCACAAAGAAATACGACAACAAAACATTGTTTGCTGCTTTGAACTTCAGCTCTGATTCGATCGACTTC
ACGATTCCAAACAATAGCTCATCGTTTAAGTTGGAATTCGGAAACTACCCAAGAAGTGAAGTTGATGCGTCTTCC
AGAACATTGAAGCCATGGGAGGGCAGAATTTACATATCTGAATGA

>YDR523C 0.69 Cold
ATGGAAAGCAAAGAAATATCAATTAGGTCAAGAACCCCTCCATCGAAGCTATATTCCATCCAATCATGTATTGGC
AGAGGTAACTTTGGTGATGTGTATAAAGCAGTGGATAGAGTTACGCAAGAAATTGTGGCAATTAAGGTGGTCAAC
CTGGAGCATTCCGACGAAGACATTGAACTGTTAGCACAGGAAATTTTTTTTCTGGCGGAATTGAAATCTCCTCTT
ATTACAAACTATATTGCAACAATGTTAGAAGATGTCTCTATGTGGATTGTCATGGAATATTGCGGCGGTGGATCA
TGTTCAGATTTACTGAAGCGAAGTTACGTCAACGGTTTGCCTGAAGAAAAAGTTTCCTTCATTATTCATGAAGTC
ACCTTGGGTTTGAAATACCTGCATGAGCAAAGGAAAATTCATCGTGATATCAAAGCAGCTAATATTTTACTAAAT
GAAGAAGGTATGGTTAAGTTGGGTGATTTCGGAGTAAGTGGCCACATTCGTTCCACTTTGAAAAGGGACACTTTT
GTAGGAACGCCGTATTGGATGGCTCCGGAAGTTGTTTGCTGCGAAGTTGATGGGTATAATGAGAAAGCAGATATA
TGGTCCTTGGGTATCACCACCTATGAGTTACTCAAGGGCTTACCCCCATTATCTAAATATGATCCTATGAAAGTT
ATGACTAACTTACCAAAAAGGAAGCCTCCAAAATTACAAGGTCCCTTTTCGGATGCAGCAAAGGATTTCGTAGCT
GGTTGTCTTGTTAAAACACCTGCTGATCGGCCATCGGCATATAACTTGTTATCATTTGAATTTGTTAAGAATATC
ACCATAACGAACTTGAAAAGTGATGTTGATCTAATTAAACAGAAAAAGGTTCAGGAGAGATATACAAAAGTTCCC
AAATACCCTCTTCAAAATCGCTTGTATAAAAACAGCAACACAGTGAGGGGAAAGGAATTTTGGAATTTTGAATCG
ACAAGACTAAGCACAACACAAATTTCCAAAGAGGAATTATCTCCCATTACACAAGACTCTCCAACATCATCTTTA
AATATGGAAAGTCCTTATTTACTTCATGGGCAAACTGTAACGCCGATAACCAACCCAAGTTCTTCATCATTTAGA
AAATGTACGCAACCAGTTTTCGAGCTTGATTCAGGAATGGATATAGATTCAGGCTGCCCAAATGCTCAAGCAGAG
ACCGAAATAGTTCCTCTCTCTAATCATAATAAGAAGCATAAAAAGAACGATATTCAGGCGTTGAAGATAGAAAAG
TTTGACTACTTGAAAAACATTGTATCTCATATTCTCAACAGAATGTATGATCGTGCGCGCGACGATGAAACAAGA
AAATACGTAAATGAAATGTTAAAGCAATTCATTAAAACTGAGGCAAACGTTCCTGGATTTAATGAGGTTTTTATA
GAAGAGATCTCACTAAGAATTGAAGCAATAAAGAAAGGATTCGTTTAA

>YIR004W 0.8Cold
ATGGTTGTTGATACTGAGTATTACGATTTGTTAGGTGTGTCTACCACTGCATCTTCCATTGAAATAAAAAAGGCC
TATAGAAAGAAATCTATTCAAGAGCATCCTGATAAGAATCCCAATGACCCCACGGCTACCGAAAGGTTTCAAGCA
ATATCCGAAGCTTATCAAGTTTTAGGTGACGATGATCTTCGCGCAAAGTATGATAAGTATGGAAGAAAAGAAGCT
ATTCCTCAGGGCGGCTTTGAAGATGCAGCTGAACAGTTCTCTGTCATCTTTGGTGGAGATGCGTTTGCCTCATAT
ATTGGCGAACTGATGCTATTAAAGAACCTACAGAAAACTGAGGAGCTAAATGCTGAAGACGAAGCTGAAAAGGAG
AAGGAGAATGTGGAAACAATGGAAGAATCACCTGCAGACGGTAAGACGAATGGCACCACTAACGCTGTTGATGCA
GCATTGGGCAATACTAACGAAAAAGATGACAAAAATAAGGCGAGGACAACTTCTGGTAATTTAACTGTACACGAT
GGAAACAAGAAAAATGAGCAGGTAGGAGCAGAAGCTAAGAAGAAGAAGACAAAATTAGAGCAGTTTGAGGAAGAA
CAAGAGGTAGAAAAGCAAAAAAGAGTAGACCAATTAAGCAAAACATTGATTGAAAGATTATCGATATTAACAGAA
AGTGTCTATGATGATGCATGTAAAGATTCCTTTAAAAAAAAGTTCGAAGAGGAAGCCAATCTTTTAAAGATGGAA
TCATTTGGTCTGGACATATTACACACAATAGGCGACGTTTACTACGAAAAAGCTGAAATTTTTCTTGCATCCCAG
AACCTGTTCGGAATGGGTGGTATATTTCATTCTATGAAGGCTAAAGGGGGAGTATTTATGGATACACTAAGAACT
GTTTCGGCAGCCATAGACGCTCAGAATACTATGAAGGAGCTTGAAAAAATGAAAGAAGCTAGCACGAATAATGAG
CCTTTGTTTGACAAAGACGGAAATGAGCAAATTAAGCCAACCACTGAGGAACTGGCGCAGCAAGAGCAGCTATTG
ATGGGCAAAGTATTGTCGGCTGCTTGGCATGGTTCTAAATATGAAATAACATCCACTTTACGTGGCGTTTGTAAA
AAAGTACTAGAAGATGACTCGGTAAGTAAGAAAACGCTTATCAGAAGAGCTGAAGCAATGAAACTATTGGGTGAA
GTCTTTAAGAAAACTTTCAGAACCAAAGTCGAACAAGAAGAGGCACAGATCTTTGAAGAACTTGTAGCAGAAGCT
ACAAAAAAGAAGAGACATACATGA

>YOR026W 0.8Cold
ATGCAGATAGTACAAATTGAGCAGGCCCCAAAAGACTACATAAGCGACATCAAAATAATCCCTTCCAAGTCACTG
CTTTTGATTACGTCTTGGGATGGCTCTTTAACAGTCTACAAATTCGACATTCAAGCAAAGAATGTTGACCTTTTA
CAATCGCTACGATATAAACATCCGTTATTGTGCTGCAATTTCATCGACAATACCGATCTGCAAATATACGTGGGA
ACTGTACAGGGTGAAATTCTAAAAGTTGATTTGATAGGTAGTCCCAGCTTCCAAGCTTTGACGAACAATGAAGCC
AATTTGGGTATTTGCCGAATATGCAAATATGGAGACGATAAACTCATTGCCGCGTCATGGGATGGCCTGATAGAG
GTTATCGACCCTCGCAATTATGGTGATGGAGTTATTGCTGTTAAAAATTTGAACTCTAATAACACAAAGGTGAAG
AATAAGATATTTACTATGGATACAAACTCCTCTCGATTGATCGTTGGTATGAACAATAGTCAGGTTCAATGGTTT
CGCCTGCCACTCTGTGAGGATGATAACGGAACAATTGAAGAATCAGGACTGAAGTACCAAATAAGAGATGTCGCT
CTTTTACCGAAAGAACAAGAAGGTTATGCATGTAGCAGCATTGACGGGCGAGTTGCTGTGGAGTTTTTCGATGAT
CAGGGCGATGATTACAACTCAAGCAAAAGATTTGCATTTAGATGCCACCGTTTGAATTTAAAAGATACAAACTTA
GCGTATCCAGTAAATTCTATTGAATTTTCCCCCCGTCATAAGTTCCTATACACGGCTGGCTCTGATGGCATAATT
TCATGCTGGAACCTACAAACCCGCAAGAAAATAAAAAATTTCGCCAAATTTAACGAAGACAGCGTGGTTAAAATT
GCTTGTTCGGACAATATTCTATGTCTGGCAACTTCTGATGATACTTTCAAGACAAACGCCGCAATTGACCAAACT
ATTGAACTAAACGCAAGTTCAATATACATAATATTTGACTATGAGAACTGA

>YBR195C 0.79 Cold
ATGAATCAGTGCGCGAAGGACATAACTCATGAAGCCTCCAGTATACCCATCGATTTGCAAGAAAGATACTCGCAC
TGGAAGAAAAACACTAAACTACTTTATGATTACCTAAACACGAATTCAACAAAGTGGCCGTCCTTAACGTGCCAG
TTCTTTCCTGATTTAGATACCACTTCGGATGAGCATCGCATCTTGTTATCCTCATTTACATCTTCCCAAAAACCT
GAAGATGAGACCATATATATTAGCAAAATATCCACGTTGGGTCATATAAAATGGTCATCTTTAAATAATTTCGAC
ATGGACGAAATGGAATTCAAACCGGAGAACTCGACAAGGTTTCCCTCCAAACACTTAGTAAATGACATCAGTATT
TTCTTCCCAAACGGGGAATGCAATAGGGCAAGATATTTGCCTCAAAATCCAGATATTATAGCCGGCGCCTCTTCA
GATGGTGCAATCTACATATTCGATAGAACAAAACACGGCTCTACTAGAATAAGACAGTCCAAAATTTCACATCCC
TTTGAGACAAAGCTGTTTGGTTCACATGGTGTTATTCAAGACGTGGAGGCAATGGATACTTCTTCGGCAGATATA

AATGAGGCGACTTCTTTAGCCTGGAACTTGCAGCAGGAGGCCCTTTTACTTTCTTCTCACTCCAACGGCCAAGTT
CAAGTTTGGGACATTAAACAATATTCGCATGAGAACCCTATAATAGATTTACCCTTAGTGTCAATAAACAGCGAC
GGAACAGCGGTGAATGATGTAACTTGGATGCCAACACACGATTCCCTCTTTGCTGCTTGTACTGAAGGAAATGCG
GTCTCCCTATTAGATCTGAGGACTAAGAAAGAGAAGCTCCAGAGTAACCGTGAAAAACACGATGGTGGAGTAAAC
TCCTGTAGATTTAACTATAAGAACTCTTTAATTCTAGCATCTGCAGATTCAAATGGGAGGCTAAATTTATGGGAT
ATTAGAAACATGAACAAAAGCCCAATCGCTACCATGGAGCACGGTACTTCCGTTTCAACTTTAGAATGGAGTCCA
AATTTCGATACTGTATTGGCAACGGCTGGCCAAGAAGATGGGTTAGTCAAGCTATGGGATACCTCCTGCGAAGAA
ACTATATTTACCCATGGTGGTCATATGCTCGGTGTGAACGACATTTCGTGGGACGCTCATGACCCTTGGTTAATG
TGCAGTGTGGCAAATGATAATTCAGTTCACATATGGAAACCTGCAGGAAACCTTGTTGGACATTCGTGA

>YPR001W 0.79   Cold
ATGGTACAAAGGCTTCTACCGGGCGCACATATATGCAGAAGGTCCTTCAATTCGTCTGCAATTATAAAGTCTTCT
GCATTGACTCTCAAGGAAGCATTAGAAAACGTGATACCTAAGAAAAGAGATGCTGTGAAGAAATTGAAGGCCTGT
TATGGCAGCACGTTTGTCGGACCGATTACCATTTCATCAGTTCTAGGTGGGATGAGAGGTAATCAGTCAATGTTT
TGGCAAGGAACATCATTAGATCCCGAACATGGCATTAAATTTCAAGGTTTAACGATTGAAGAATGTCAAAATAGA
TTACCTAATACAGGTATTGATGGCGATAATTTCCTGCCAGAATCAATGTTATGGCTGTTGATGACAGGTGGTGTG
CCAACTTTCCAGCAAGCTGCTTCTTTCAGGAAAGAATTGGCTATTCGTGGAAGGAAATTGCCACATTATACAGAG
AAAGTTTTGTCAAGTTTACCCAAAGATATGCACCCTATGACCCAACTTGCAATTGGTTTAGCATCAATGAACAAA
GGATCCCTCTTTGCTACAAACTACCAAAAGGGCCTCATAGGAAAGATGGAATTTTGGAAAGATACTCTTGAAGAT
TCGCTAAATTTAATTGCTTCACTCCCGCTGCTGACTGGGAGAATTTATTCGAACATTACAAATGAGGGACACCCA
TTAGGTCAATATAGTGAGGAGGTGGATTGGTGTACAAACATATGTTCCCTGCTCGGCATGACAAATGGAACGAAC
TCTTCTAATACATGCAACCTTACTTCCCAGCAATCCCTGGACTTCATAAACTTGATGCGGTTATATACCGGTATA
CATGTAGACCATGAAGGTGGTAATGTTTCTGCTCACACCACCCACTTGGTTGGAAGTGCGTTGAGTGACCCGTAT
CTCAGCTATTCATCTGGAATAATGGGATTAGCGGGTCCTTTGCATGGGTTAGCAGCACAAGAAGTAGTAAGGTTT
CTCATAGAGATGAACTCTAATATTTCCAGCATTGCACGAGAACAAGAAATCAAAGATTATCTTTGGAAAATTTTG
AATTCAAACCGTGTGATCCCCGGTTATGGGCACGCAGTTTTGCGTAAACCGGATCCTCGATTTACAGCAATGCTT
GAATTTGCGCAAAAGAGGCCTATTGAATTTGAGAACGACAAGAACGTTTTGTTGATGCAAAAATTGGCAGAAATA
GCGCCTAAGGTTTTGTTGGAACACGGAAAGAGTAAGAATCCATTTCCCAATGTTGACTCTGCATCGGGGATTTTG
TTTTATCATTATGGAATCAGGGAATTATTATTCTTTACCGTCATTTTTGGGTGTTCAAGGGCCATGGACCCTTG
ACACAACTTGTTTGGGATCGCATTCTAGGTTTACCAATTGAAAGGCCCAAGAGTTTGAACTTGGAGGGTCTGGAA
GCACTTACCAAAGCAAGCAATGTTAACAAGTTGTAA

>YOR113W 0.71   Cold
ATGCCTCCTCCAACTGCACAGTTCATGGGCCCTACGCAGGCGGACAAAATGAAAGCCAAAATCAATCATCAGGC
GAAGCTGGGGAGCAAAACCAGGAGCATGGGCAAGGCCCTACTCCTATTCTTAATCAAAGTCAACCGGCTTCTTCT
CAACCGCAACATCAACAACAAAGGAATGAGTCGATTTCATATTATACAAATTTCAACCAGCCACGATATTCCACG
GACGCCTCTATCAACTCATTCTTGAACATATCTGATAACGTACCAGTAACAAGTACAGGAGGACCTAGTTCTGGC
GGCGCCTATTCCAATCTTCCACGATTATCCACTTCAAGTACACATCAACCGCCAGACCTGTCGCAAATCGGACGC
GGCTTTTCCATTGTAAACAACCTCTTCCCACAACAACAGCAGCTTCAAAATCAGCATCGGCAACAGCAGCAACAA
CAACAACAGCAGTCGCACCAGCAGCCTCCCTTCAAGACCCCTTCATTTTCGACGGGATTAACGGGAAGTTCTTCT
CAATATCAATTTTTACCAAGAAATGATAATACTTCGCAGCCACCTTCAAAAAGAAACTCTGTTTATCTTGGACCT
AATGATGGGCCTGATTTTGAATTTTTCAGTATGCAGCAGTCACAGCAACCGCAGTTCCAGCCTAGCAGTAGAAGA
GAATCAAACTCTATGAGACCTCCACTGTTAATACCTGCAGCAACTACTAAAAGCCAGTCCAATGGCACCAATAAT
AGTGGGAATATGAACACAAATGCAGATTATGAATCATTTTTTAATACTGGTACAAACAACAGTAATTCTAATCAG
AACCCGTACTTTTTGAGTTCAAGAAATAATTCTTTGAAGTTTAATCCTGAAGATTTCGATTTTCAATTCAAAAGG
CGGAATTCTTTTGTTAGAGGTACTTTGGACCATAGCAGCCAAAATGCGTTTATACCTGAATCAAGATTAAACTCA
CTATCCGTTAACAACAAAGCTAATGGTGATCCTGTCGCGGATAATGTTACCAATAACATGAAAGGAAAAAGTAAT
GAAGTTGACAATGATGATGGCAATGACAGTAGCAATAACAATAACAACAACAACAACAACAACAACAATGAAAAC
AACAATGACAACAATAATGATAATAACGACAATAGTATTAATTCCGCCACCAGTACTAATATCCCAAACCAAGAG
GACCATAGCCTTGCTTCTACCGATACCACAAGCAATAGTAGGAAAGATCTAAAAGAAATAGAACAAAGACTTCGA
AAACATTTGAATGATGAGGATAATTACTCTAGTGCTATATCAAGACCATTGGATAAAAACGACGTAATTGAAGGC
AGTGAGGGATTGAACAAACATATAGACGAGTCTGGTATGCAACCTAATATTATCAAGAAAAGGAAAAAGGATGAT
TCTACTGTGTACGTCAAGAATGAGATGCCCCGTACTGATCCCCCGATGAGTAAAGACAATTCTACTTCTGCTGAA
GGAGCAGCAATGGCAAACTTTTCTGGTAAAGAACCTCCTATACCTGACATAAGTTCAGTAAGTGATGATGCTACT
AACCTGATAGGTGCAACAAAGGTCGACCAACTAATGTTGATTATTCAAGCAAGAAAGAAAGGTTTCACGGAGAAA
GTAAATACCACTCAAGATGGAGACTTACTGTTTAACCAAACGATGGACATTTTACCACCTAAAAGCGAACTAGTA
GGTGGTGTGGAGAAACCAAAAGGCACACAAAATACACGAGCAGTTAAAAAACATGAATGTCCCTATTGTCATCGG
CTTTTTTCGCAAGCGACTCATCTGGAGGTTCACGTTCGTTCTCATATAGGGTACAAACCATTCGTTTGTGATTAT
TGTGGCAAACGTTTTACTCAGGGTGGGAACTTAAGAACTCATGAACGACTACACACAGGTGAAAAACCGTATTCA
TGTGATATTTGTGATAAAAAATTTTCTAGGAAAGGGAACTTAGCTGCTCACTTGGTTACTCACCAAAAATTGAAA
CCATTTGTTTGCAAGCTTGAAAACTGCAACAAGACCTTCACTCAACTAGGAAATATGAAGGCCCATCAAAATAGA
TTTCATAAGGAAACATTAAATGCCTTAACGGCAAAATTAGCTGAGATGAATCCATCTGAGAATATTCCACTTGAA
GAGCGGCAACTTTTGGAGTACTTTGCGTCCATTTATAAAAATTCAAACAGGGGAATTAAAGGTAGAGGAAAAGGT
GTAGGAACCAAAAAAATCAACAATTTCCTCACCAGAAAACCATCCTGCGAGCACAATTTTGAATCCAAATACAAAT
GCAAATAATGCTATTGCTAATGATTCTGAAAATAACGGCAACCCTGAAGGTAACATTGACAGCAGTAGTAACAGC
AATCCGGGCTCACATAGTATGATTTCGCCGACGCAAAAAGACATGGGTACGTTGCAATCACAATTTATCCAAAAT
AATTTTAACAATTCTGTGAACAGTTCGAATCCGTCCAACCAACCAATCATAAACTACAATTACACCACACTTCCT

CATTCTAGATTAGGAAGTAGTAGTTCCAGCAATACCAACAATAATAACAGCAACTTTTCGGTAGGTGCAGCTCCG
GGTGTATTAATGGCGCCAACCACCAATAATGACTTCAGTTTCAACTTGGATCAATCTAATGATAATGAAAGATCT
CAACAAGAACAAGTGAGATTTAAGAACATTAATTACAAAAGCTAG

>YLR388W 0.79 Cold
ATGGCTCACGAAAACGTCTGGTTCTCTCACCCAAGAAGATACGGTAAAGGCTCCCGTCAATGTCGTGTTTGTTCT
TCCCACACCGGTTTGATCAGAAAGTACGGCTTAAACATCTGTCGTCAATGTTTCAGAGAAAAAGCTAACGACATT
GGTTTCAACAAATTCAGATAA

>YFL047W 0.81 Cold
ATGCTATCATTTTGCGACTATTTTTGGTCTGAAGACCTTGTCTCAGGTCTAGATGTGCTTTTTGATAGATTATAC
CATGGTTGTGAGCAATGCGACTTGTTCATTCAATTATTTGCGTCAAGAATGCAATTTGAAGTCAGTCACGGGAGG
CAGTTGTTTGGTATAGAAGCCGGTATGGACAATCTGAAGGCAGTGCAGGAAGATGAAGATGAAGGGGTGACAGTT
TCGAGGGCCCTGAGGGGAATTCTACAGGAAATGTCTCAGGAAGGGACGCATCACTTGACAATAGCGTCGAACATA
GAAAGTTTGGTGCTGCAGCCATTCAGTAAATGGTGCATAGAACATAGGGAGAGAATTCAATACTCCGAGAAGACT
TTGTTGACCAATGTAAATAATTTCAGGAAGTCTAAAAAGTACGTCGGTAAGCTGGAGAAGGAATATTTTAATAAA
TGTAGGCAGCTAGAAGAATTTAAAAGAACTCATTTCAACGAAGATGAGCTAGCAAATGCAATGAAATCATTGAAA
ATACAAAATAAATACGAAGAAGATGTGGCCAGGGAAAAGGATCACAGATTTTTCAACAGGATAGCGGGAATAGAT
TTTGATTATAAGACAATGAAGGAGACGCTTCAGCTGTTACTGACCAAGCTTCCTAAAACGGACTATAAGCTACCT
CTCATTAGTTATTCTTTGAGCAACACCAACAATGGTGGAGAAATAACGAAGTTTCTGCTTGACCACATGTCGTTA
AAGGATATTGATCAAGCTGAAACGTTTGGCCAAGATTTGTTGAATTTAGGGTTTTTGAAATATTGCAACGGTGTT
GGTAATACATTTGTCAACTCTAAAAAATTTCAATACCAATGGAAGAATACCGCGTACATGTTTGCCAATGTGCCA
ATGCCCGGTTCAGAGGAGCCTACCACTGGTGAATCCTTAATATCCCGGTTCAACAACTGGGATGGTTCATCTGCA
AAGGAAATTATTCAATCCAAGATTGGTAACGACCAGGGGGCAGCGAAGATCCAAGCTCCGCATATATCCGATAAT
GAAAGGACGCTTTTCAGAATGATGGATGCGCTTGCAGCGTCAGATAAGAAGTATTACCAGGAGTGCTTCAAGATG
GATGCCTTGAGATGCTCGGTTGAGGAGTTGTTGATTGACCATTTGTCGTTTATGGAAAAATGTGAGTCCGATAGA
CTGAATGCGATCAAGAAAGCGACACTAGATTTTTGTTCTACCTTGGGTAACAAGATTTCTTCGTTAAGACTGTGC
ATAGATAAAATGCTCACGCTAGAAAACGATATTGATCCCACTGCAGACCTTTTGCAACTTTTAGTTAAGTACAAG
ACGGGCAGTTTCAAGCCGCAAGCCATTGTTTATAATAATTACTACAATCCTGGTTCATTTCAAAACTTTGGTGTT
GATCTAGAAACTCGTTGTAGACTAGACAAGAAAGTCGTTCCGTTGATAATTTCTTCGATATTCTCATATATGGAC
AAAATATATCCTGACTTACCTAATGATAAAGTAAGAACTTCAATTTGGACTGATTCGGTGAAATTAAGTTTAACA
CATCAGCTTCGAAACCTATTGAACAAACAACAGTTCCACAACGAAGGGGAAATATTTGATATACTTTCTACGTCC
AAGTTGGAGCCAAGCACCATCGCCAGTGTTGTTAAAATTTATTTATTAGAATTACCAGACCCACTAATTCCCAAT
GATGTCTCTGATATTCTTCGGGTGCTTTACCTTGACTATCCACCTTTAGTTGAAACTGCGTTGCAAAATTCTACA
TCATCTCCCGAGAATCAGCAGGACGATGACAACGAAGAAGGCTTTGATACCAAGAGAATAAGAGGCCTTTATACT
ACTCTGTCCTCTCTAAGCAAACCACACATAGCGACACTAGACGCTATTACAACTCATTTTTACAGATTAATTAAA
ATTCTGAAAATGGGAGAGAACGGTAATGAAGTAGCAGATGAGTTTACCGTCTCAATTTCACAAGAATTTGCCAAT
TGTATTATCCAGTCAAAGATCACCGATGATAATGAAATTGGTTTTAAGATTTTCTATGATCTACTGACACATAAG
AAACAGATTTTTCACGAATTGAAAAGGCAAAATTCAAAAAAATTAG

>YLR131C 0.72 Cold
ATGGATAACGTTGTAGATCCGTGGTATATAAATCCCTCAGGCTTCGCGAAAGACACTCAAGATGAGGAGTATGTT
CAACATCATGATAATGTCAATCCTACCATACCCCCACCCGACAATTATATTTTGAATAATGAAAACGATGATGGC
CTCGATAACTTGTTAGGTATGGACTACTATAACATCGATGACCTGTTGACTCAAGAGTTAAGAGATCTGGATATT
CCTTTAGTGCCTTCTCCTAAGACGGGCGATGGTTCTTCTGATAAAAAGAATATTGATAGAACTTGGAACCTTGGT
GATGAAAACAACAAAGTCTCCCACTATAGCAAAAAATCAATGTCCTCACACAAGAGAGGTCTAAGTGGCACAGCG
ATATTTGGATTTCTCGGCCATAATAAGACATTGAGTATTTCCAGTTTACAGCAATCCATTCTAAATATGTCTAAA
GATCCGCAACCCATGGAACTCATAAATGAATTGGGTAATCATAATACGGTAAAAAATAACAATGATGACTTTGAC
CATATAAGGGAAAATGATGGTGAAAATAGCTATTTGAGCCAAGTTTTGTTGAAACAGCAGGAGGAGTTAAGAATT
GCTCTTGAAAAACAAAAGGAAGTGAACGAAAAATTGGAGAAGCAGTTGAGAGACAATCAAATACAGCAAGAAAAG
TTGCGTAAAGTATTAGAAGAGCAAGAAGAGGTGGCGCAGAAGTTGGTTTCTGGGGCTACAAATTCTAATTCCAAA
CCTGGATCTCCAGTAATACTAAAGACACCTGCCATGCAAAACGGTAGAATGAAAGATAATGCTATAATCGTCACA
ACGAACTCTGCAAATGGCGGATATCAATTTCCTCCTCCGACGTTAATATCGCCTCGGATGTCAAATACTTCAATA
AATGGTTCACCATCCAGGAAATACCATAGGCAACGATATCCAAATAAAAGCCCAGAAAGTAATGGATTGAACCTT
TTTTCCTCTAACAGTGGTTATTTGAGAGATTCTGAACTGCTTTCATTTTCTCCACAAAATTATAATTTAAACTTG
GACGGCTTGACTTATAATGACCATAATAACACCAGTGATAAAAACAATAATGATAAAAAAAAATAGTACTGGTGAT
AACATATTCCGTCTGTTCGAAAAGACTTCCCCGGGTGGGCTAAGTATCTCTCCAAGGATAAATGGAAATAGTTTG
AGATCGCCCTTCCTCGTCGGCACAGATAAAAGCAGGGATGATCGATATGCTGCTGGCACGTTCACGCCTAGAACA
CAGTTGTCACCTATCCACAAGAAAAGGGAATCCGTAGTTTCCACGGTCTCGACAATATCACAACTGCAGGATGAC
ACTGAACCCATCCACATGCGAAATACCCAGAACCCAACATTAAGAAATGCAAACGCTTTAGCGTCATCAAGTGTA
CTACCTCCTATTCCTGGTTCCAGCAATAACACTCCAATTAAGAATTCTTTGCCACAAAAACATGTATTTCAACAT
ACTCCCGTCAAAGCTCCACCAAAGAACGGAAGTAACCTAGCTCCGCTTCTAAATGCACCGGATTTAACAGATCAT
CAGTTAGAAATTAAGACACCCATACGAAATAACAGTCACTGTGAAGTGGAAAGCTATCCGCAAGTACCACCTGTC
ACACATGATATTCACAAAAGCCCCACTTTGCATAGTACGTCTCCTTTACCAGATGAAATAATACCTAGGACTACG
CCAATGAAAATAACCAAGAAACCAACTACTCTGCCTCCGGGTACCATTGACCAGTACGTCAAGGAACTACCCGAC
AAACTATTCGAGTGCTTATACCCTAACTGTAACAAAGTATTCAAGCGTAGATACAACATAAGGTCGCATATTCAG
ACACATTTGCAAGATAGACCGTATTCATGCGACTTTCCCGGTTGCACCAAGGCGTTTGTTCGCAATCATGATTTA

ATAAGACACAAAATCTCCCATAATGCCAAGAAATACATCTGCCCATGCGGAAAGAGATTTAATAGGGAGGATGCT
CTAATGGTGCATAGAAGTCGGATGATTTGCACCGGCGGTAAGAAATTAGAACATTCGATCAACAAGAAACTTACA
TCTCCCAAAAAAAGCCTGCTTGACAGCCCGCATGACACAAGTCCCGTAAAAGAAACTATCGCCCGGGATAAAGAT
GGGAGCGTCCTAATGAAAATGGAGGAACAGCTGCGAGATGATATGCGCAAACATGGATTACTGGATCCACCCCCA
TCCACAGCAGCGCACGAGCAAAACTCGAACCGCACCCTTTCAAACGAAACTGATGCTCTCTGA

>YDR359C 0.75   Cold
ATGTCCTCACGTCCAAGTTCAGCAGTGCCTAATTCAGCATCACTCAGTGAGGACCAGAGCAGTGATAGAAGTAAA
TTTCCTAAGGCAGATGACTTAATTGATGAAAGAGATAGAAAACTGACCGAACTATACTGCGTATCCCGCTTGAAC
CAGCTGCTAGAGTTAACAGATGAAAACAAACTTCGGAAGGAAATAGATGCATTTTTAAAGAAAAACGATATACGA
AGGGGTATAAGGTTTGACGAGGCGTCACTGCCGAAGCTCCTACATACAGCTGCGACTCCCATAACAAAAAAGAAA
TTGAAGGATGTAAATTTAATAAATGTACCTAACCAGAGGCTATCCGATTCTAAAATGAGTAGGGAACTGCCAGAA
AATAGCGAAAATGTATCAGTCAAATCAGAGAGTCATTTTGTTCCAAGCCACGATAATTCAATCCGTGAAAATATG
ATGGATTCTTTGAGGCCAGCTGAAAAGACAGGAGGTATGTGGAACAAAAGGCCACTTGAGTCAACTATGGGAGGT
GAAGAAGAGCGACATGAAAAAAGACAGAAGATGCAATCTCAGTCATTGGAATCTTCTAATAATTCGGAAATGGCT
TCATTACCTATTTCGCCGCGTCCCCCAGTGCCTAATGCATTAGCACACTACACTTATTATGAGAACATCGAGTAC
CCACCAGCAGATCCCACTGAGGTGCAACCTGCAGTAAAATTCAAGGATCCGTTGATAAAGAACATTATGGCGAAA
GAGATAGATACATCAGACCATTATAACGAGAACAATGTTGACGCATTAGAGACCGTATTTTTATTAATGAACGAT
TATATACCATCGAAAATACCCCAGGCTTTGCCCTTAGCAGAATTGAAGTACATGTCACAAACTCTGCCTCTTATT
AATCTAATACCGAGAGCTCACAAGGCTTTGACCACTAATATTATAAACAACGCCTTAAACGAGGCCAGAATTACT
GTTGTTGGCTCGCGAATAGAAGAACTAAGAAGACTTGGATTATGGTCTTTAAGACAACCTAAAAGATTCATCGAT
CCGTGGAAACAACACAATACGCATCAAAACATACTTTTAGAAGAAGCTAAATGGATGCAAGCAGATTTTAAAGAG
GGCCACAAATATAAAGTAGCTATCTGCACAGCAATGGCACAGGCAATCAAGGACTACTGGACCTATGGGGAAATA
TGCTGTGTAAAACGTAAAACACTTCTACCTGGTAAGGAAAATAAATTATCTGACGACGGGCGCATATCTGAAAAG
TCCGGTAGACCATCGGATACTTCAAGAAATGATTCCGATATTAGCATAGCCGGTAAGGATGACATTGGAATTATT
GCGAACGTTGACGATATTACTGAAAAAGAAAGTGCTGCTGCTAACGATAATGATGAGAATGGAAAGAATGAAGCA
GGAGCTAAGAGTGATTTTGATTTTGCAGATGGTTTGTTATCTCAAGAAGGTGCCCACGATCAAATTATATCTTCT
ATTGACACCAAGTTACTTCTAAAAAAGCCATCCTCCTCAAGCGAAGTTGTTTTAATACAACATGAAGTAGCTGCA
TCATCAGCGTTGATCGAAACCGAAGAATCAAAGAAAGAGCTAGCCCCCCCATTTAAACTTTCAATCTTTGTTGAC
GAACTCAATACTTTTGAAAAAACATTAATACAGGATTTACCGCTGTATAATGGTATTAATGAAGAACGGCCAAAA
AAAGATGATTCTTTACCATTTATTCCAATTTCCAAATCGGTAGTGTCACTGGATGATAATGGATTTTATAAGTTA
CTTGAGCGTCAATTGATTGACGAAGAACCATCAATATCACAATTGAGCAAAAGGCGGGGTATGTTCTATGGTAAT
AGAAGAAATCATTATTTAAGACCACCAGCAGTACCTTCATTACGCTATCTTCAAAATAGAACACCGACTATATGG
CTGTCTGAGGACGATCAGGAACTGGTCAAAAATATTAACACATATGGCTACAATTGGGAATTGATTAGTGCACAT
ATGACACATCGCCTCACATATTCTTATTTATCGAATATTGAACGTAGAACGCCATGGCAGTGTTTTGAGCGTTTT
GTACAATTAAATGAACGATTCAATTTTAGTGACCTCAAGGGTCCCAGAGCACATAGTGCTCAACAGTGGTTGATT
GAAGCCCATAAATTTCAGCAGAGACAGAATAGAAGAATATCGCCTTTAGGTGTAAATACAGAATCCATACAAAGA
GGTCATAGGAGACTCCGTTGGGCGAGCATGTTTGAGGCGATTAGAAAATGTATGAAAAAAAGAGAAAATACGCCG
CGTCCCAATCCAACCCAGCCAAGGAAACCGTTAGATTGTAAAAACATGAAGGTCCCTACCCCAGCAGAAATGTCG
CTTTTGAAAGCCCAAAGAGATGAGGCATTGAGAAGGGATATACAATTGAGAAGAACAGTGAAAAATAGATTACAA
CAGAGGCAACAGCAAAGTCAGCAGGCACATTCATCCCGAGCTCAAAGTCCAATACCTTCAAATGGCAAATCTTCT
TCAAATTTGGCTAGAAATGGGCAAGCGTCGGCTCCAAGACCAAACCAGAAACAGTATACTGAGCAAGATATCATT
GAAAGTTACTCGAGGAAGTTGTTGGAGCAAAAACCAGACATTGGTCCTGAGATGGCCCTTAAGGCGGCAAAAAAC
TATTATAGAACCTTGAGAGAACAACAGCAACAGCTAAAGCAGCACCAGATTCAACAGCAAAGGCAGCAGTTACAG
GAGGAGTCCAGTCATGTGCAGCAACTGCAGCAACTACAACCAGGGTCTCAAGCTCCGCCACCGAAATCATCTCCA
TCTCAATCTTCTCTTTCCAATATTTCTAACATTAATTCTGCTCCAAGGATAAAGTCACCAACTCCACAGGAGATT
TTACAAAGGTTCCAAAAACAATGA

>YBL102W 0.8Cold
ATGAGCGAGGAACCACCTTCTGACCAGGTCAATAGTCTCCGTGACTCATTGAATCGATGGAATCAAACAAGACAG
CAGAACTCGCAGGGTTTTAATGAATCTGCGAAGACATTGTTCTCAAGCTGGGCGGATTCTCTCAATACCAGGGCC
CAGGATATATATCAGACGTTGCCTGTATCTAGACAGGACTTGGTGCAAGACCAGGAGCCGTCGTGGTTCCAATTG
TCAAGAACGGAAAGAATGGTACTTTTTGTCTGTTTTCTTTTGGGTGCAACAGCCTGTTTCACTCTTTGTACTTTC
CTTTTCCCCGTTCTAGCCGCTAAACCAAGAAAGTTTGGTTTACTATGGACAATGGGGTCCCTACTATTTGTTCTT
GCGTTTGGGGTACTTATGGGACCACTCGCGTACTTAAAACATTTGACTGCAAGGGAAAGGCTGCCTTTTTCGATG
TTCTTTTTCGCCACATGCTTCATGACGATTTATTTCGCAGCCTTTTCCAAGAACACGGTGCTGACTATTACATGT
GCTCTTCTTGAATTAGTTGCCGTCATTTATTATGCTATTTCATATTTCCCATTCGGTGCAACAGGTTTGAGGATG
TTAAGCTCTGCTGGTGTCAATTCGGCAAGAGGTGTTCTGCGCATCTGA

>YCR016W 0.79   Cold
ATGTCCGAAAATCACGTTCCTGCCTGGAAAAGAATTGCTTTGAAGAGACAGACTATAAGCAGTGGTGACGAAAGT
AAAGAAAAAGGCCAATCCAATCTAATAGATGATGATCCACTAAATATCACTACGCATTTATCCACTGGTAATTTG
ACAAAGAAAGAGAAAAAAAGAATCATTAATGGTGAGAGCAAATCCTCTACCAAAAAAGGGAAGCGTGTGTCGAAA
CCAGGGACGAAGAAGAAGGAAAAATTGTCAAAGGATGAAAAGAATTCGAAAAAAAATAAAATTCTTAAAGATCAA
TTACGTTACCTGATAGAATTTTTCAGGACAAAGTCTGAGAGCAAGTTTCCCACCGGAATCCTAGAATTGGAAAGT
GTAAAAGAAAATTACGGCGACTCCCTGATCAAAGACGAGCCATCAGAGTCTGGTGTTGTTGAAGTTTGGAAATTT
TCCAAGCAAAAGCAAAATTGGCTTATTAAGCATTTTTTCAACTTGGACGAAATCCCCTCAGTATATAATGATCTT

TTGCTTTTATATTTCAGGGACTTACAAGGTAAATCAAAAGAAGAACTAATATCAAAATGCAAAGGAAAACTGAAG
CAATGGAACGACTATGTGGAAGATCAAGAAACAAAAATAAAAGCGTTAATTGCAGAGGATAAGGCTAGCGAACCA
ATTAATGGTGAAGAAAAAGAAGAAGGTGAAAAAGACGGTAATGCCGAACAAGGGAAGCAAAAAGAGGTACAAGAC
GAGCAAGAGGAAGTACAAATGCCGAATAAAGAGCTTGTTCAAAGAAGTCTGAAATTACTAGAAATTTGGAAGAAT
GATGACTCGGAGCAGATAGAACTTAAGAATTTCTTCGTTGATGTTTAA

>YLR086W 0.74   Cold
ATGTCTGATAGTCCATTGAGCAAAAGACAAAAGCGGAAGTCCGCCCAAGAACCAGAATTATCTCTTGATCAAGGT
GATGCCGAAGAAGATTCGCAGGTAGAAAACCGAGTTAACCTAAGCGAGAATACACCAGAGCCGGATCTTCCTGCG
TTGGAAGCATCTTATTCCAAATCTTATACTCCCAGAAAGCTTGTTTTAAGTTCTGGGGAAAATCGGTATGCCTTT
TCTCAACCTACAAACTCAACAACCACGTCATTACATGTACCGAACTTGCAACCACCAAAAACCTCTTCTAGGGGT
CGCGACCATAAGTCCTACTCTCAATCACCACCAAGGTCTCCAGGAAGATCGCCAACTAGAAGATTAGAATTGCTC
CAGCTTTCGCCAGTGAAAAATAGCAGGGTTGAACTACAAAAAATTTATGATAGGCACCAGTCGTCGAGCAAGCAA
CAGAGCAGACTATTTATTAACGAACTAGTCTTAGAGAACTTCAAGTCCTACGCTGGTAAACAAGTAGTAGGACCC
TTTCATACTAGCTTCTCGGCCGTGGTAGGCCCCAATGGTTCAGGTAAATCAAATGTCATCGATTCCATGTTATTT
GTATTTGGATTTAGAGCGAACAAGATGAGACAGGACAGATTGTCGGATTTAATTCATAAATCAGAAGCTTTCCCA
AGTTTGCAATCATGTTCCGTAGCTGTACATTTTCAGTACGTTATTGATGAATCTTCGGGTACTTCCCGAATCGAT
GAAGAAAAACCTGGATTGATCATTACAAGGAAAGCCTTTAAAAACAACTCATCGAAATATTACATAAACGAAAAA
GAAAGTAGCTACACAGAGGTGACAAAGCTTTTAAAGAATGAGGGTATTGATTTAGACCATAAACGATTTTTAATT
CTACAAGGTGAAGTAGAGAATATTGCTCAAATGAAGCCTAAAGCAGAAAAAGAGAGTGACGACGGACTACTGGAA
TATCTGGAGGACATAATTGGAACTGCAAACTATAAGCCGTTAATTGAAGAGCGAATGGGACAGATTGAGAATCTA
AATGAAGTTTGCCTGGAAAAGGAAAATAGATTTGAAATTGTTGATAGAGAAAAAAACTCTTTAGAGTCAGGGAAA
GAAACGGCGTTAGAGTTTTTAGAGAAGGAAAAGCAGCTGACGCTTTTAAGATCAAAATTATTTCAATTTAAATTG
TTGCAAAGCAACTCTAAACTTGCCAGCACCTTGGAAAAGATCTCCTCTTCGAATAAAGACCTCGAAGATGAGAAG
ATGAAATTTCAAGAATCTTTGAAAAAAGTGGATGAGATTAAAGCACAACGTAAGGAAATAAAAGATCGAATATCA
TCTTGTAGCTCGAAAGAAAAGACCCTGGTTTTAGAAAGAAGAGAATTAGAAGGCACCAGGGTGTCTCTAGAAGAG
AGAACAAAAAATTTGGTAAGTAAAATGGAAAAAGCAGAAAAGACTTTGAAATCCACCAAACATTCAATATCGGAG
GCCGAGAATATGCTTGAAGAGCTTCGTGGACAACAAACCGAACACGAGACGGAGATCAAAGACTTGACTCAATTG
CTGGAAAAGGAACGAAGTATACTTGATGATATTAAACTATCTTTGAAAGATAAAACGAAGAATATATCTGCAGAG
ATTATTCGGCATGAAAAGGAACTGGAGCCTTGGGACCTCCAACTTCAGGAAAAAGAATCACAGATACAATTGGCT
GAATCCGAACTATCTTTGTTGGAGGAAACTCAAGCTAAACTGAAAAAAAACGTTGAAACTTTAGAAGAAAAAATT
CTTGCCAAGAAAACACACAAGCAGGAGCTACAAGATCTTATCCTCGATCTGAAAAAGAAGCTGAACTCACTTAAA
GATGAAAGGTCGCAAGGTGAAAAGAATTTTACTTCTGCTCATCTAAAGTTAAAAGAAATGCAAAAGGTTTTGAAC
GCGCATCGCCAGCGTGCAATGGAAGCTAGATCCTCTTTATCAAAAGCTCAGAATAAAAGTAAGGTTTTAACAGCT
TTATCGAGACTGCAAAAGTCAGGACGTATAAATGGTTTCCATGGACGTTTGGGCGATTTGGGCGTTATCGACGAT
AGTTTTGACGTTGCTATTTCTACTGCTTGTCCGAGACTAGATGATGTCGTGGTTGATACTGTAGAATGTGCGCAA
CACTGCATCGACTACTTAAGAAAAAACAAACTTGGTTATGCAAGGTTTATTCTCTTGGATAGGTTACGCCAATTC
AATTTACAACCTATCAGTACACCAGAAAATGTACCGAGACTATTTGATTTAGTTAAACCTAAAAACCCAAAATTC
TCAAATGCATTTTACAGTGTTCTTAGAGACACTTTGGTTGCTCAGAATTTAAAACAAGCCAATAATGTAGCATAT
GGGAAGAAAAGATTTAGAGTTGTCACTGTGGATGGGAAATTAATTGATATTTCTGGTACAATGAGTGGTGGTGGC
AACCATGTAGCAAAAGGTCTAATGAAATTAGGTACGAATCAGTCAGACAAGGTTGATGATTACACCCCCGAGGAA
GTAGATAAAATTGAGCGTGAGCTATCTGAAAGAGAAAATAACTTCCGCGTGGCAAGCGATACGGTCCACGAGATG
GAGGAAGAACTGAAAAAATTGAGGGACCACGAACCAGACTTGGAATCACAAATATCAAAGGCAGAAATGGAAGCT
GATTCCTTGGCGAGTGAATTGACACTGGCAGAACAACAAGTGAAAGAGGCAGAAATGGCGTACGTCAAGGCAGTT
AGTGACAAAGCGCAACTAAACGTGGTGATGAAGAATTTGGAACGCTTGAGAGGCGAATACAATGATTTGCAATCC
GAAACAAAAACTAAAAAGGAAAAGATCAAAGGCTTGCAAGACGAAATCATGAAAATTGGTGGTATCAAATTGCAA
ATGCAAAATTCAAAAGTTGAATCAGTTTGTCAGAAATTGGATATTCTAGTGGCCAAACTTAAAAAAGTCAAATCT
GCCTCAAAGAAGTCAGGAGGAGATGTCGTAAAGTTTCAAAAACTGCTCCAAAACTCTGAAAGAGACGTAGAACTA
TCATCAGATGAGTTAAAAGTCATCGAAGAACAACTAAAACATACAAAACTGGCTTTGGCAGAAAATGACACAAAT
ATGAATGAGACGCTCAACTTGAAAGTTGAATTAAAAGAACAGAGCGAACAACTGAAGGAACAAATGGAGGACATG
GAGGAAAGCATCAATGAATTTAAATCTATAGAAATCGAAATGAAGAACAAGCTGGAAAAATTGAACTCACTGTTG
ACGTACATCAAAAGTGAGATAACGCAGCAAGAAAAAGGATTAAACGAACTATCAATTAGGGATGTAACCCACACC
CTGGGAATGTTAGACGATAATAAAATGGACTCAGTGAAAGAGGATGTTAAAAACAATCAAGAACTTGATCAGGAA
TACCGGTCCTGTGAAACTCAAGATGAGAGTGAAATAAAAGACGCTGAGACTTCTTGTGACAATTATCATCCTATG
AACATTGATGAGACTTCAGATGAGGTATCCAGAGGGATACCAAGACTTTCTGAGGACGAATTAAGGGAGTTGGAC
GTAGAACTGATTGAAAGTAAAATAAATGAATTGTCATATTATGTCGAAGAGACTAACGTGGATATTGGAGTTTTG
GAAGAGTACGCTAGGCGCTTAGCAGAGTTCAAAAGAAGGAAGCTGGATTTAAATAATGCTGTTCAAAAAAGAGAT
GAAGTTAAGGAACAATTAGGAATACTTAAAAAGAAAAGATTTGATGAATTTATGGCTGGCTTTAATATCATATCA
ATGACCCTAAAAGAAATGTACCAAATGATTACTATGGGTGGAAATGCTGAATTAGAACTTGTGGATAGTCTAGAT
CCTTTTTCTGAAGGAGTTACGTTTAGTGTTATGCCTCCTAAAAAGAGTTGGAGAAATATAACAAACCTTTCAGGT
GGTGAGAAAACACTAAGCTCCTTGGCTTTGGTTTTTGCTTTACATAAGTATAAACCAACCCCCCTTTATGTCATG
GATGAAATAGATGCCGCTCTGGATTTCAGAAATGTCTCAATCGTAGCTAACTATATCAAAGAAAGAACCAAGAAT
GCACAGTTTATTGTTATTTCGCTGAGAAATAACATGTTCGAATTAGCACAACAGTTAGTTGGTGTTTATAAAAGA
GATAATAGAACCAAAAGTACCACGATTAAAAACATAGATATCTTAAACAGAACTTAG

>YGR289C 0.74   Cold

ATGAAAAATATCATTTCATTGGTAAGCAAGAAGAAGGCTGCCTCAAAAAATGAGGATAAAAACATTTCTGAGTCT
TCAAGAGATATTGTAAACCAACAGGAGGTTTTCAATACTGAAGATTTTGAAGAAGGGAAAAAGGATAGTGCCTTT
GAGCTAGACCACTTAGAGTTCACCACCAATTCAGCCCAGTTAGGAGATTCTGACGAAGATAACGAGAATGTGATT
AATGAGATGAACGCTACTGATGATGCAAATGAAGCTAACAGCGAGGAAAAAAGCATGACTTTGAAGCAGGCGTTG
CTAAAATATCCAAAAGCAGCCCTGTGGTCCATATTAGTGTCTACTACCCTGGTTATGGAAGGTTATGATACCGCA
CTACTGAGCGCACTGTATGCCCTGCCAGTTTTTCAGAGAAAATTCGGTACTTTGAACGGGGAGGGTTCTTACGAA
ATTACTTCCCAATGGCAGATTGGTTTAAACATGTGTGTCCTTTGTGGTGAGATGATTGGTTTGCAAATCACGACT
TATATGGTTGAATTTATGGGGAATCGTTATACGATGATTACAGCACTTGGTTTGTTAACTGCTTATATCTTTATC
CTCTACTACTGTAAAAGTTTAGCTATGATTGCTGTGGGACAAATTCTCTCAGCTATACCATGGGGTTGTTTCCAA
AGTTTGGCTGTTACTTATGCTTCGGAAGTTTGCCCTTTAGCATTAAGATATTACATGACCAGTTACTCCAACATT
TGTTGGTTATTTGGTCAAATCTTCGCCTCTGGTATTATGAAAAACTCACAAGAGAATTTAGGGAACTCCGACTTG
GGCTATAAATTGCCATTTGCTTTACAATGGATTTGGCCTGCTCCTTTAATGATCGGTATCTTTTTCGCTCCTGAG
TCGCCCTGGTGGTTGGTGAGAAAGGATAGGGTCGCTGAGGCAAGAAAATCTTTAAGCAGAATTTTGAGTGGTAAA
GGCGCCGAGAAGGACATTCAAGTTGATCTTACTTTAAAGCAGATTGAATTGACTATTGAAAAAGAAAGACTTTTA
GCATCTAAATCAGGATCATTCTTTAATTGTTTCAAGGGAGTTAATGGAAGAAGAACGAGACTTGCATGTTTAACT
TGGGTAGCTCAAAATAGTAGCGGTGCCGTTTTACTTGGTTACTCGACATATTTTTTTGAAAGAGCAGGTATGGCC
ACCGACAAGGCGTTTACTTTTTCTCTAATTCAGTACTGTCTTGGGTTAGCGGGTACACTTTGCTCCTGGGTAATA
TCTGGCCGTGTTGGTAGATGGACAATACTGACCTATGGTCTTGCATTTCAAATGGTCTGCTTATTTATTATTGGT
GGAATGGGTTTTGGTTCTGGAAGCAGCGCTAGTAATGGTGCCGGTGGTTTATTGCTGGCTTTATCATTCTTTTAC
AATGCTGGTATCGGTGCAGTTGTTTACTGTATCGTTGCTGAAATTCCATCAGCGGAGTTGAGAACTAAGACTATA
GTGCTGGCCCGTATTTGCTACAATCTCATGGCCGTTATTAACGCTATATTAACGCCCTATATGCTAAACGTGAGC
GATTGGAACTGGGGTGCCAAAACTGGTCTATACTGGGGTGGTTTCACAGCAGTCACTTTAGCTTGGGTCATCATC
GATCTGCCTGAGACAACTGGTAGAACCTTCAGTGAAATTAATGAACTTTTCAACCAAGGGGTTCCTGCCAGAAAA
TTTGCATCTACTGTGGTTGATCCATTCGGAAAGGGAAAAACTCAACATGATTCGCTAGCTGATGAGAGTATCAGT
CAGTCCTCAAGCATAAAACAGCGAGAATTAAATGCAGCTGATAAATGTTAA

>YGL132W 0.6Cold
ATGGAAAAAAATAACACAACAAAGCCGCGCAAGATTGGACCATACTTTCTATCTAAGGTAACTATCACTTACTGG
CTTGATGGATGCGTATTTTTGTCGGTACCAGCTTCTGAAATATCGATATCAAACATAGCTGGCTCAATTCCTTCC
ATCAATTGCTGGGTAATTTCGACTGGTAAACCGTTATCGTTAGAGATATTAGCAGATGCGCTATCAAAATCGAGT
TCTTGGGCACCTGATCTCAAATTGCAAGAATGGCAAATAATATCTTCATACCAGTATAAACTTTTGTTTGTACCA
CAATGTGGGCAAGTCCTTTCGAAAGGAAGTACGTAA

>YOR020C 0.75 Cold
ATGTCCACCCTTTTGAAGTCTGCTAAATCTATCGTTCCATTGATGGACCGTGTCCTTGTCCAAAGAATCAAGGCA
CAAGCAAAGACAGCATCCGGGTTGTATTTACCTGAAAAGAACGTGGAGAAGTTAAACCAAGCTGAAGTTGTTGCC
GTAGGCCCGGGCTTTACTGATGCTAATGGTAATAAGGTTGTTCCTCAAGTTAAAGTTGGTGACCAAGTTTTGATT
CCACAGTTTGGTGGTTCTACCATTAAATTGGGTAACGACGATGAAGTTATTCTTTTCAGGGACGCTGAAATCCTG
GCTAAGATTGCCAAGGACTAA

>YPL207W 0.81 Cold
ATGGATGGTTTTCGTGTAGCTGGTGCGTTAGTAGTTGGTGCGTTAACCGCTGCATATTTATATTTTGGCGGAAGG
TTTTCGATAGCGCTGGTCATTATAGTAGGTTATGGTATTTATTGCAATGAAGCCAGCGGTGGTAGTCAAGATAGT
CAAGAGAAGCTCGATTTGAACAAACAACAGAAAAAGCCATGTTGCAGTGATAAGAAGATTGCGGATGGAGGAAAA
AAAACTGGCGGATGTTGTTCTGATAAGAAGAACGGTGGTGGTAAAGGTGGAGGATGCTGTTCCTCAAAAGGTGGA
AAGAAAGGGGGGTGTTGTTCATCTAAAGGTGGAAAGAAAGGAGGATGTTGTTCCTCTAAAAAGAATATTGGTGAC
AATGAAAATACTGCCACTGAAGTTGAGAAAGCCGTAAATTACCCTGTTACTGTGGATTTTACAGAAGTTTTTAGG
AAGCCCACTAAGAAAAGGTCGAGTACCCCCAAGGTTTTTTCGAAAAATAGCTCGTCTAACTCTAGAGTAGGTAAA
AAATTAAGTGTTTCAAAGAAGATAGGTCCAGATGGATTGATCAAGAGCGCGTTGACTATTTCGAATGAAACGCTT
TTGAGTTCACAGATCTATGTGTTATATAGTTCCCTGCAAGGTGCAGCTTCGAAAGCTGCAAAGAGCGTTTACGAC
AAACTAAAGGAATTGGATGAATTGACTAATGAGCCAAAACTTTTGAACCTTGACGATCTATCCGATTTCGATGAC
TATTTTATAAATGTTCCCGTTGAAAATGCATTGTATGTGCTTGTTTTACCGTCTTATGATATCGATTGCCCTCTA
GATTATTTCTTACAAACTCTTGAAGAGAATGCGAATGATTTTAGAGTAGATAGTTTCCCATTACGAAAATTGGTT
GGCTACACAGTTTTGGGGCTTGGTGATTCAGAATCATGGCCTGAAAAGTTTTGTTATCAAGCCAAAAGGGCCGAT
CACTGGATTTCTCGTTTAGGTGGCAGGAGAATTTTCCCCTTAGGCAAAGTTTGTATGAAAACAGGAGGTAGTGCC
AAAATCGATGAGTGGACATCATTGTTAGCAGAGACTTTGAAAGATGATGAACCAATTATTTATGAGTATGATGAA
AACGCAGACTCTGAGGAAGATGAAGAAGAAGGTAATGGTAGTGATGAATTAGGCGATGTAGAAGATATTGGTGGT
AAGGGTAGTAATGGAAAGTTTTCAGGCGCAGATGAAATCAAACAAATGGTGGCGAAAGACAGTCCAACGTACAAG
AACTTGACAAAGCAAGGTTACAAAGTTATTGGTTCCCATTCAGGCGTTAAGATCTGCCGATGGACTAAGAATGAA
CTACGTGGTAAAGGGTCTTGTTATAAAAAATCGCTCTTCAATATCGCGTCCAGTAGATGCATGGAATTGACTCCT
TCTTTGGCATGTTCATCCAAATGCGTTTTCTGTTGGAGGCATGGTACAAATCCCGTGTCAAAAAACTGGAGATGG
GAAGTAGATGAACCAGAATACATTCTAGAAAATGCTCTGAAGGGACACTATTCCATGATCAAACAGATGAGAGGT
GTGCCCGGTGTTATTGCAGAAAGATTTGCGAAAGCGTTTGAAGTTCGTCATTGTGCCTTGTCTCTTGTCGGCGAG
CCTATTCTTTATCCTCATATCAATAAATTTATCCAATTATTACACCAAAAGGGCATAACTAGTTTCCTTGTGTGC
AATGCCCAACATCCAGAGGCCTTGAGAAATATTGTTAAAGTAACACAATTATATGTTTCTATTGACGCACCCACC
AAGACAGAGCTGAAAAAAGTGGATAGACCTTTGTACAAAGATTTCTGGGAGAGAATGGTAGAATGCTTAGAGATT
TTGAAAACTGTACAAAACCATCAAAGGACAGTTTTCAGGTTAACTTTAGTGAAAGGTTTCAATATGGGTGATGTC

AGTGCATATGCAGATCTGGTCCAACGTGGTTTACCAGGTTTCATTGAAGTTAAAGGCGCCACGTTTAGTGGCTCA
TCTGATGGGAATGGTAATCCTTTGACAATGCAGAATATTCCATTTTACGAGGAATGTGTAAAATTTGTGAAGGCG
TTTACCACGGAATTACAAAGACGTGGATTACATTACGACTTGGCTGCCGAACATGCGCATTCCAACTGTTTATTA
ATTGCAGACACGAAATTCAAAATTAATGGAGAATGGCATACACATATCGATTTTGATAAATTTTTCGTATTGCTA
AACTCGGGCAAAGACTTCACGTACATGGACTATTTAGAAAAGACACCCGAATGGGCATTGTTTGGCAATGGTGGG
TTTGCACCAGGGAATACAAGAGTGTACAGAAAAGATAAGAAAAAGCAGAATAAGGAAAATCAAGAAACTACAACG
AGAGAGACGCCTCTCCCTCCTATTCCTGCTTAA

>YGR014W 0.75   Cold
ATGCAGTTTCCATTCGCTTGTCTCCTATCGACCCTTGTAATTAGTGGGTCATTGGCCCGGGCCAGCCCCTTCGAC
TTTATATTCGGCAATGGAACGCAACAAGCTCAGAGCCAAAGCGAGAGTCAAGGTCAAGTTTCTTTCACCAATGAA
GCTTCTCAGGATAGTTCCACCACCTCTTTGGTAACAGCCTATTCTCAAGGTGTTCATTCGCACCAGTCTGCAACA
ATAGTGAGTGCCACAATCTCTTCCCTCCCATCTACTTGGTATGATGCGAGCTCCACTTCCCAGACTTCTGTGTCA
TATGCCAGTCAAGAATCCGACTATGCCGTTAATCAAAACTCTTGGAGCGCGTCTACTAATCAACTGCCATCTACC
AGTACGACAAGCTACTATGCGCCAACCTTCAGTACATCGGCCGATTTTGCTGCTTCTAGTGTAAATGCAGCTTCT
GATGTCTCCACTGCCAGTGTTCCCATTGATACGAGTGCTAATTCTATCCCTTTCACAACTACAAGTAACATAGAG
ACTACAACGAGTGCACCTCTCACTTCGGACACTCCACTTATTTCCACTAGTACGATGTCCGCAGCTGATAACGTA
TTTTCGTCAGCAAACCCTATTTCTGCCTCCCTAACAACCACCGATAGTTCAGAAAGTTTTGACCAAACTTCGACT
GCTGGTGCCATTCCGGTGCAAAGTTCAGCAGATTTTAGTAGTTCTAGTGAAATTTTAGTACAAAGTTCGGCGGAT
TTCAGTAGCCCTAGTTCTCCAACTACTACCGATATATCGCTATCAGCTGCCCCACTGCAAACAAGTGAATCAAGC
AGTTTTACCACTGCATCAGCAGCTCTACCAGTAAGTTCAACAGACGTTGATGGCTCAAGCGCCTCACCTGTAGTG
AGCATGAGCGCCGCAGGACAAATAGCTAGCTCAAGCAGCACAGATAATCCAACTATGTCAGAAACCTTTTCGTTA
ACATCTACAGAAGTTGATGGTTCCGATGTTTCATCAACAGTGAGCGCATTATTATCGGCTCCTTTTTTACAAACA
AGTACTTCCAACAGTTTCAGCATTGTTAGCCCATCGGTATCTTTTGTTCCATCACAGAGTTCCTCAGACGTTGCT
AGCTCCAGTACTGCAAATGTAGTTAGTTCATCCTTTTCTGATATTCCACCGCAAACTAGTACCTCAGGGAGCGTA
GTTTCGGTAGCGCAATCCGCATCTGCCCTCGCATTTCAAAGTTCAACAGAGGTATATGGTGCCAGTGCCTCGAGC
ACAATGAGTTCATTATTATCAACTACTTCGCTACAGTCTACTACTTTGGATAGCTCAAGTTTAGCTAGCTCCTCT
GCGTCGAGTTCAGACCTTACAGATTATGGCGTCTCCAGTACAGCAAGCATACCGCTGTTGTCAGCCTCAGAACAA
GCAAGTACTTCCAGCAGTTTTAGCGTTGTTAGCCCTTCGGTATCTTTTGTTCCATCACAAAGTTCCTCAGATGTT
GCTAGCACCAGTGCTCCAAGTGTAGTTAGTTCATCCTTTTCTTATACTTCACTGCAAGCAGGTGGCTCTAGCATG
ACCAATCCCTCTTCATCAACTATAGTATATTCAAGTAGTACTGGCAGTTCTGAGGAATCCGCTGCATCTACAGCT
TCTGCAACACTGTCGGGCTCCTCGTCTACTTATATGGCAGGAAATTTGCAATCACAGCCTCCATCCACTTCAAGT
TTGCTTTCGGAGTCTCAAGCTACAAGCACTTCAGCTGTGCTAGCTAGCAGTTCTGTTTCTACAACTTCACCCTAT
ACCACTGCAGGTGGTGCATCTACAGAGGCCTCATCCCTCATATCATCTACATCTGCGGAAACTTCCCAGGTAAGT
TATTCACAAAGCACAACTGCATTGCAAACTTCCTCATTCGCATCGTCTTCAACAACAGAAGGAAGTGAAACATCT
AGTCAAGGTTTTTCTACCAGCTCTGTTTTAGTTCAAATGCCTTCTTCGATTTCCAGCGAATTCTCACCCTCTCAG
ACGACAACTCAAATGAATTCTGCAAGCTCATCATCTCAGTACACTATATCATCCACTGGTATACTTTCTCAGGTT
TCAGACACATCGGTGTCTTATACAACTTCAAGTTCGTCTGTTTCTCAAGTTTCAGACACACCAGTTTCTTATACA
ACTTCAAGTTCGTCTGTTTCTCAAGTTTCAGACACACCGGTTTCTTATACAACTTCAAGTTCGTCTGTTTCTCAA
GTTTCAGACACACCAGTTTCTTATACAACTTCAAGTTCATCTGTTTCTCAAGTTTCAGACACACCGGTTTCTTAT
ACAACTTCAAGTTCGTCCGTTTCTCAAGTTTCAGACACGTCAGTACCTTCTACAAGTTCCAGATCGTCCGTTTCT
CAAGTCTCAGACACTCCGGTGCCTTCTACAAGTTCAAGGTCGTCCGTTTCTCAAACATCTAGCTCACTACAGCCC
ACCACTACATCCTCCCAACGTTTCACCATTTCCACTCATGGAGCGCTTTCTGAAAGTAGTTCTGTTAGCCAACAA
GCTTCTGAGATTACTAGCTCAATCAATGCAACAGCTTCCGAATACCATAGCATCCAGACAACCGCGGCTACTCAA
TCCACAACTCTATCTTTTACCGACGCAAACAGCAGTTCTGCTTCCGCTCCATTGGAAGTGGCAACGTCTACGCCA
ACCCCATCTTCAAAGGCATCCTCTCTGTTGCTTACACCATCAACATCCTCTTTAAGTCAGGTTGCTACAAATACT
AATGTACAGACGAGTTTAACAACGGAATCGACGACCGTTTTAGAACCATCAACGACTAACAGTTCCAGTACGTTT
AGTCTGGTCACTTCAAGTGACAACAATTGGTGGATTCCAACTGAGTTAATCACGCAGGCACCAGAAGCTGCATCC
ACTGCATCTTCTACCGTTGGAGGAACACAAACTATGACTTTGCCCCATGCAATTGCAGCCGCGACACAAGTTCCC
GAGCCTGAGGGCTACACCCTAATCACAATAGGGTTCAAAAAAGCTTTGAACTACGAATTTGTTGTATCAGAACCA
AAATCATCGGCTCAAATCTTCGGATACTTGCCTGAAGCTCTGAACACACCTTTTAAGAACGTATTCACAAACATT
ACGGTACTACAAATAGTGCCATTACAGGATGACTCACTCAACTACTTAGTAAGTGTTGCTGAAGTATACTTTCCA
ACTGCAGAAATAGAGGAGCTGTCAAATCTAATTACCAACTCTTCAAGCGCTTTTTACACGGATGGAATGGGTACA
GCAAAATCTATGGCTGCAATGGTTGATTCCTCAATACCGCTAACGGGCCTCTTACACGATAGTAACAGCAACTCT
GGCGGATCTTCGGACGGATCCTCCTCCAGTAATTCGAACTCAGGATCTTCAGGTTCAGGATCTAATTCGAACTCC
GGTGTGTCTTCATCTTCCGGAAATTCCTATCAAGATGCCGGTACTTTGGAATATTCATCCAAATCTAACTCCAAC
GTATCCACTTCTAGCAAATCAAAGAAAAAAATCATTGGTTTAGTTATCGGCGTTGTTGTTGGTGGATGCTTATAT
ATTTTATTCATGATTTTTGCTTTCAAGTATATCATAAGAAGGCGGATTCAAAGTCAAGAAATTATCAAGAACCCA
GAAATTTCCAGTATCAGTTCAAGTGAATTTGGTGGAGAGAAAAATTACAATAATGAAAAGAGAATGAGCGTTCAA
GAATCCATAACACAATCTATGCGAATTCAAAATTGGATGGATGATAGTTACTATGGTCACGGGTTGACAAATAAT
GACTCAACTCCAACCAGGCACAATACATCGAGTTCCATACCAAAAATTTCAAGACCAATTGCTAGCCAAAACTCC
CTGGGTTGGAACGAAGTTTGA

>YBL103C 0.75   Cold
ATGATGAACAATAACGAAAGTGAGGCTGAGAACCAACGTCTACTGGACGAATTAATGAACCAGACAAAAGTCCTC
CAGGAAACTTTAGATTTTTCGTTAGTGACACCCACTCCACACCACAATGATGATTACAAGATACACGGAAGTGCC
TACCCAGGTGGTGAGACTCCTGCCCAACAGCATGAAAAACTCTCATACATCAATACGCACAACTCTAACGATAAT

AATAACTTAATGGGCAGTCAAGCGAGGTCCAATTCACAAACTCCTACAGCTTCGACCATATATGAGGAAGCAGAA
TCGCAATCGTCTTACCTGGATGATATGTTTAGAACAAGCCAAGGCGGTAGACCTGTCACTCAAAATTCCATATCT
TCCATAGGGCAGGGTCCCTTGAGATCATCTTATTCTATGGCTTACGACTCACCTGTGGATAGAGCAATGAATACT
CCATTACAGCAACAAGAAGGCTTAAAAGCTGAGTTACCACATGACTTTTTATTTCAGCATGGCACCGATGACACA
ATGTATAACTTAACTGATGATTTGAGCTCCTCTTTATCTTCTAGCATCAATTCTGATATGATGACACCAAACACA
TATTCATCATCATTTTCTTATAATCCACAAAGCTTGGGCCCAGCATCCGTATCTTCCACATATTCCCCAAAGGTA
AGATCACCATCATCGTCATTTCGTGCAGGAAGTTTCCTTTCATCTTCTTTTAGGCATGGTAGCATAAACACACCC
AGAACAAGACACACTTCAATAAGTAGCAATATGACTGAAAATATAGGACCTGGAAGTGTTCCAAAGATTTTAGGC
GGGTTAACTTCTGATGAGAAACTGAGGCGCAAAAGAGAGTTTCATAATGCCGTCGAAAGGAGAAGAAGAGAATTA
ATAAAGCAAAAGATAAAAGAACTCGGCCAGTTGGTTCCACCATCTTTATTGAATTACGATGACCTAGGTAAACAA
ATCAAACCAAATAAGGGTATCATTTTGGATAGAACGGTCGAATATTTACAATACCTGGCCGAAATTTTAGAAATA
CAAGCACGAAAAAAGAAGGCGTTATTGGCGAAGATAAAAGAATTGGAAGAGAAGAAAAGTTCTGTCGCAGCATTA
TCTCCTTTTACCAATAACCACCATGCCAGTTCGGGGCAAAATAATAGCGAGAATAGTGAGGAAAGAATTATAGAT
ATTAGATCTGTCCCAAATGCTTTGATGAATGAACAAAATAGTAAGGCTGAATTGCACAATTGGGAGCCACCGTTA
TATGATTCGGTCGGTAACCACAATCATGCTGGCACTATGGAGAGTCATCCACATACAAACATTCATGAAGAATTA
AAGGAGTTCTTATCAGGCGATTTGATTGAAGCCGAAGATAATGCAAAATTAATGTTTGGAGATGACAACTCTAAT
CCAGCTGACTATCTTTTAGAATTTGGTTCGGGGTAG

>YLR290C 0.81 Cold
ATGATACCAAAGCTTATAGTTTTTGGAGGCAATGGATTTTTAGGTAAAAGAATTTGTCAAGAAGCAGTGACTTCG
GGCTACCAAGTCGTTTCTGTGTCAAGGTCCGGTAAAGCTCCTCATAGCAATGAATTAAATGATAAGCAATGGATG
CAGGAGGTCCAATGGACCGCTGCTGACATTTTTAAGCCTGATTCTTACCATGAATTATTGAATAATGCTACTAAC
GTAGTTCATTCTTTGGGAATCCTCCTAGAAAATGAAAATTATAAGCAAACTCTATCCAAATCCCCTACATATGAT
TCAAAATCACGCCTTCTATCTTTTGGTGCAGGTCCAAATCCTTTAAAAAAAAGCAGTCCTTATTTTACTTATGAA
ATGATGAATAAGCAAAGTGCTATTATTTTGGCAGATACATTTAAACAAAAAATCCTAAAGAAAAGCAAAAAGGAG
CAAGAAAAAGCAAATCAAAGATCTTTTACGTATATTTCCGCCGATAAAGGTTTCCCATTGATTCCTAGTGGCTAT
ATTAATTCGAAAAGAGAGGCGGAGATTGAACTAGAAAAAATGCAGAGATATTTCAGGCCTATTATTGTGAGACCC
GGTTTTATGTTCGATGAGCACAGAAATGCAATTGGACCACGGTCTTTTATTCATACTGCACTTGAACTACTTTAC
TGCGGTAACAAATTTCTATTGAGAAATAAGCTGCAGCTTTTGAACGACTTGATTAGGCCAACAGTATCCACTCAG
CAGGTAAGCAAATCCGTCCTGAAAAATATCGAAAACCCAGATTTTAAAGGGGTAGTTACACTTGAGGAAATACTT
AAAGCATAA

>YJL059W 0.8Cold
ATGAGTGACAAATCTCATCAGATATATTGCTACTTTTGGCTCTTTGGTTTGATCAATAATGTACTTTATGTGGTA
ATACTTTCAGCAGCTGTTGATATCGTGGGTCCTACCTTGCCTAAATCTCTAGTATTGCTAGCAGATATATTCCCA
TCACTCGCCATTAAATTGTGTTCCCCATTCTTTATTGATAGAATCAAGTACAGCTATAGAATATGGTCTTTGATC
ACGATGAGTTGCTTAGGAATGTTCTTAGTTTCTTTTAAAAACTTGTTTGTTTGTCTTTTAGGAATATCTTTCGCA
TCTATATCTTCCGGATTTGGAGAAGTGACATTCCTACAGCTAACACACTATTACAAACAAATTTCTTTGAATGGA
TGGTCGTCAGGTACTGGTGGTGCAGGAATCATTGGCGGAGCATCTTATATGTTCTTAACTTCAATCTTTAAAGTT
CCAGTGAAGCTAACTTTACTAGTATTTAGTCTCCTTCCATTTGCATTTTTGTTTTATTTTAAATTAGAATCCAAT
GATACCAACCTGACTTACCAAAGTCTTCAACAAATTGACGAAGCAGAGGATGACCAGTTGGTGCCCTTTCCTGTC
GCCTTTACACATACCAACGCTTCGCAATCCCTGTATTCTACAAGACAGCACATTTTGCAAACGGTCAAGAGACTT
CGAAGATTGGTTTTTCCATACATGGTTCCATTGACCACCGTCTACTTATTCGAATATTTAATAAACCAAGCGGTA
GCTCCGACCCTATTATTTCCGATTAATGGTGATGAAAGAAGTAAATCGATGCCATTTTTCTTCCATAAATACAGA
GATATATACGTAACCTATGGAACGCTCTACCAATTAGGTGTCTTTATATCAAGATCCTTTGGACATCTTATGAGA
ATGAGGAGTTTATACATCTTAGCGTTTTTACAAGGTGTGAATTTGTGCATAACGGTACTGCAATCATGGTTTTAT
GTCACACATTCGCCCTGGGCGGTGATGATATTAATATTCTATGAGGGTTTTCTTGGTGGTGCGTCATATGTTAAC
ACATTCTTAAATATTCTCGAACAAGAGGATCCTGATGAAACAGAGTTCGCCATGGGTGCTGTGTCCATCGCTGAT
TCCTTTGGCGTTTTTTTGGCTGCGTTACTTGGTTTGGGGCTAGAACCCAAACTTTGTAGGCATCAAATTGCCGAT
GACAGACCTTGGTGTAGGATGGAATAA

>YPL007C 0.82 Cold
ATGAAGCTTCTAAAGGACTTATTGGTAGATAGAAAGGAATTTGAGGATTGGAAGAACAATTTAACATGGGCTCGG
GATGGTACTCTATATTTGACCACATTTCCTGATATTAGCATTGGGCAGCCAAAATATGCTAAAGACATTAATTGC
AACAGTAAGAATTTATTTCATGTGAAAGAATTCCCGCTAGAATTTGAAAATAAACTGGATTTCGAGCTGGCGCAA
CAGAATGGGTTATTAAATTCACAACCTGTTTGTTATCCCAGAGTCTGTAAGCCATCACCAATAGACGATTGGATG
GCCGTATTGAGCAATAATGGTAATGTTTCTGTGTTCAAAGATAACAAAATGCTCACAAATCTCGATTCAAAAGGA
AACTTAAGTAGCAGAACCTACCATTGTTTTGAATGGAATCCTATTGAATCATCAATCGTGGTTGGTAATGAAGAC
GGTGAGCTTCAATTCTTTAGCATTCGCAAAAATTCAGAAAATACTCCAGAATTTTATTTTGAGAGTAGTATTAGA
TTAAGTGACGCAGGCTCAAAAGACTGGGTCACTCATATAGTATGGTACGAAGACGTTCTTGTAGCGGCGCTTTCA
AACAATTCTGTATTTTCAATGACAGTTTCTGCTTCCTCTCATCAACCCGTTTCTAGAATGATACAAAATGCTTCG
AGAAGAAAAATTACTGATTTAAAGATAGTGGATTATAAGGTGGTTTTAACTTGTCCCGGATATGTGCATAAGATA
GATTTAAAAAATTATTCAATTTCTAGCTTGAAAACAGGATCTCTCGAAAATTTTCATATAATCCCATTGAATCAT
GAGAAAGAGAGCACGATATTATTGATGTCCAATAAGACTAGTTATAAAGTTTTACTAGAAGACGAACTTCATGTG
ACGGCTGATAATATTATCGCGCCTTACTTAGAGAAGAAATTCAAGAAATGGAGCACAATTTGGAATGAATTCAAT
AATTATGAAACAACTCTTGTCATACACGGAATATCTCTCTCTCCAGACGGCTATTCAATAGCCATAGTATACGAT
ATGGAACGGGTGGCTTTCAAATATAAAATAGCTTCAGAACAATCGTTTAACATCATGTTCGCCCCACTATATCAT

ACTTGGACAATTTCTGAACGTGCTGTTGGATTAGCGTGGTATCAGACGTACCAAATTTACAACCAATCGCTGCCC
AAACTTCCAGAAAATTTCAGTATGAACAAGAAATTACTGAATGGTAATTACCCTATTAGTCTGGATTTCCAATCA
TACCTCAATGCATTAATGAAAAGTGAAGAGATGAGGATTATAATGTTCTTGAATATGACTATCGATAAACCGTCA
ATTTTGTCATTTTTGGAAGCCTTATATGAATACGCTATAAACAAAAAATCCGAATTAACGAATAGTTTTGATTTA
GCTTGCGTATTATCTATAGCGGCAATACTTAAAAGAGAAGCGCCAATTTACAACGGCACGTTATTGATGAAGAAC
AGCTTTCTCGAGGAAACGTTCAATTTAGAAAGCTTCACGGCTGACCCTGAAACTGTAACATCCACCACGAATAAC
ACGTGGAAGAGGTGTGGTGTCACTTTGCTTCCAATACTTACAACGCATGTAAAGATATGTCCCGTGAGCAAACAA
CGGGTAATAGACATAAAACGAGATGATTTGAATGATTATGGTTGGTTTACAAGGGGTCTATTAGAAAGATTTAAT
GAAATAAGCGTATATTGTGGAACAACGCTGGAAGTTATGTAA

>YNL164C 0.74   Cold
ATGACACCTACAAACCAATCTAGTGGAACGACTAATGCATCTGTGGAGGTACTTTCAGAGGATGGACCTATGCCA
ATAAACGTTATGATGCAAGAAGGTGTGAAAGCGCTGACTAAAATTCTTTCCAATCAGCTTCAAGACAGACAAGCT
TTTCAAAACGCTCCTCATGCAATGCAATTCGTTATAAGAAATGGAGGAAAAGCTTTATCAAATGCACGGTTAGAA
GAGCTCAAAGATGCTCTTCCTAAGATGGACTCGTTGAGTTTGGAAGATGAACTTGCGAAAATAGATGGCCAAAGT
GCATACCACATTGATTCCGCAGAGGAAAAGGAAACCTTTGAAAGTAAAATAGGCCAAATAGCTTCTAGAAATAGT
GCAGACTTCATAATAGAGGAGGACTTGCAAAATATTTTAGATGATGATCTTAAAGACTCAGAACTTAACTTGGAT
GGCGAAGAAGCCGAAATAATTTTCGATTATGAGAGCCAAGAACTGGATACACCGGATGGTATAGGGGAAAAGATT
TCTCAAATGATAGAATCGGTGTTACCGGGAGGGTTTGGAAGTGAAGAACAGGGGGGGTTGCGAACTGTAACAAAT
GTAGAGGATTTAGATGTTGCAGAAGAAGTCACAGATATCGATCATGATACCGTTGACGCAGCTCGTTTGCATGGA
GACGGTCAACATTCCATATCTTCTAGAAAGCACAGCAGGTCAAAGAACTCGAAAAAGAATGGGCATGTAAGGCGA
CATGACTTTTATGACGAATCTAGAGACCATAAAAGTTGTTGCCCGCATCATCATTATGAAAACTTATCCAAACTA
CGAAACTACTATTATCATGATTTTGAATATATTTCCAGAACGGAAAACCGTGTTCCAGATTTCTCTGTCCTAGTT
AACGAATCAAGTCCCATGTGTTTATTTTGCGAGTACTACATGGTGTTTGGTGAACCACCACGTAACATGATAAAG
TGGTATAATCGTACGTTTGGTTACAATCGTATGCCGAATCCACCAAGAGATGAACAAGATAGTAGGAAAAGGAAT
AGATAG

>YGL003C 0.74   Cold
ATGTCCACAAACCTGAACCCATTCATGAATAATACGCCTTCCTCCTCCCCACTCAAGGGTTCTGAAAGTAAGAGG
GTATCGAAAAGGCCAATATCTAGTTCTTCGTCCGCCTCACTATTATCATCTCCCTCCAGGCGATCGAGGCCGTCT
ACAGTATATGGTGATAGGTATATTCCTTCCCGAACAGATATAGATTTCAATTCCATCGTTTCCATCAGTAGTATG
GCAAGCGTTCCAGCCCTTAATCCATCGAGTACGGAAGATCAAGTAGAATACCAAAAGGAAAGACAGGCACATGAA
ACTTATAATACTTTGCTGAAGAATGAGCTTTTTGGCGAGATGCTAAGCAAGGATACGGTAGGCTCAGAGAGCAGT
ATAGATCGCATTAAGAATACGAGACCATCTACTAGAGGCAATGTGCACGCAGAAAATACAACTAGACATGGTTAT
GAACTGGAACGAGTATCAACTCCACCTCCTGAAGCTGCTGGTTTAGAGGAATTTTCGCCACATTCCACGCCAGTC
ACTCCTCGACGTCTTTTTACATCACAGCAAGATGAAATCACGAGACCCTCAAGCAACTCAGTTAGGGGCGCCAGC
CTGTTGACTTATCAACAACGTAAAGGTAGAAGACTATCTGCGGCCTCTTTGTTACAATCTCAATTTTTTGATTCC
ATGTCGCCAGTTAGGCCGGATTCCAAACAGCTCCTATTATCACCGGGGAAGCAATTTAGACAAATAGCTAAAGTT
CCCTATAGAGTTTTAGATGCCCCATCATTAGCAGACGATTTTTACTACAGCTTGATAGATTGGTCAAGTACTGAT
GTTTTGGCAGTTGCTCTTGGAAAATCGATTTTTTTAACCGATAATAACACTGGCGACGTTGTTCACTTATGCGAC
ACGGAAAACGAATACACAAGCTTAAGCTGGATTGGAGCAGGCTCTCATCTGGCAGTAGGTCAAGCAAATGGACTT
GTAGAAATTTATGACGTAATGAAAAGAAAATGTATTAGGACGTTGTCGGGACATATTGACAGAGTAGCGTGTTTA
TCTTGGAATAATCATGTTTTGACGTCTGGGAGTAGAGATCATCGAATACTGCATAGGGATGTTAGGATGCCAGAT
CCCTTTTTTGAAACTATAGAATCGCACACTCAGGAAGTCTGTGGCCTAAAGTGGAATGTAGCGGACAACAAACTT
GCCTCAGGTGGTAACGATAACGTCGTCCATGTTTACGAAGGAACGTCGAAATCTCCAATCTTGACGTTTGACGAA
CATAAGGCTGCTGTTAAGGCAATGGCCTGGTCTCCTCATAAACGAGGTGTGCTAGCTACCGGTGGTGGTACAGCA
GATAGAAGACTAAAAATCTGGAACGTTAATACGTCAATAAAGATGAGTGATATAGATAGCGGCTCTCAAATATGT
AATATGGTATGGTCAAAGAATACTAACGAGCTTGTGACATCACATGGTTACTCAAAATATAACTTAACCTTGTGG
GACTGTAACTCTATGGATCCAATTGCAATTCTGAAAGGTCATAGTTTCAGGGTTCTGCATTTAACGTTATCCAAT
GATGGAACCACGGTGGTTTCTGGGGCTGGAGATGAAACATTGCGATATTGGAAACTTTTTGATAAACCAAAAGCG
AAGGTACAGCCAAATTCGTTAATATTTGACGCATTTAATCAAATACGTTAG

>YNR056C 0.77   Cold
ATGAACAGAGTAGGGGCAGTGTTCCTATTTGTATATGAAAGAAATTTTTTTTTGTCTATTGTTCCAGATCGTCAC
AGGACGGAAATAAGAATGTCTAGTTCAGAAAGGTCAGAAGTCAAGTTTGACAAGCACTTTAATTGGTGGTCCCTA
TTAGGTATCGCGTTCTCATTAAGTTGCTCATGGGTCGGTATCTCAGCGTCGATGGCCGTTGGTATTGCCAGTGGA
GGGCCACTGCTTATCATCTATGGGTTGATAATTGCTGCTTTTTTCAGTCTCATGTGTGGTATATCTCTGGGAGAT
TTTGCTGCTATCCTGCCAAACAGCAGCGGTGGTTCATTTTGGGTTCTTAAAATGTTGGAACAAGAATCAGTCACT
TTGAAAACCCCTGAGTACGAGGACCCTTCTGACGATGATGAAGAAGTGTTCCTCGAGAATTATTGTCAAACTTTC
AACGTGGAAGTTTCTTCTAAATTTCAAAAGGTTTCTTCCATGGTTGTAGGGTTGCTGAACTATTTCGGTGCCATT
TTTACTACCGCAAGTATCTGCTCATCTTTGTCAATGAGCTGTATCGGTATTCATAAGTTGTTGCATCCGGACTAC
GAGTTAAAGCACTGGCATGTTTTTGTGGGTTACGAGTGTATCAATGCCGTTTTGACACTTTTCAACATTTATTCA
ACTCCGTTGCCCTATATCTCTCAGTTTGGGCTCTATACGTCTCTATTGTCTTTCGCCATGACTTTCATTATTTGT
ATTGTTTCAAGATCTGACAACACCGTAGACCCATGGCCCAAAGCTTCTAATATATTTGGGAGCTTTGATAATCAA
ACAGGCTGGAATTCGTCTGGAATGGCGTTTGTTGTCGGTTTGGTCAATCCGATCTGGGCATTTGTTGGCATTGAC
TCTGCTACGCACATGATTGATGAAGTGGGTTATAGTAAGTCACGTTTCTTAGTTCCCAAAGTTATTATCACTACC
ATCATTGTGGGCTTTGTAACTAGTTTTATTTATTGCGTTGGTTTGTTTTTCTGCATCACTGACCAAACGGCAGTC

GTTGAGTCTATTTTACCTATCGTAGAAATATTTTACCAGGCCACTGGTAATAGGAATCTCAGTGTCTTCCTGCAA
TGTATGTGTATCACAACAGGTTTTGTTTCAGGCATTGCAAGTGGAACCTGGCAAAGTCGAATCCTCCAATCATTC
GGGAAAAGTTACGCACCATTTTATAAAGAGGGTTCCTTGGGAAACAAATCCTTGAAGAAGCTAGCAGTGTTGACA
CCTGGGTTTAAGTCTCCATTATATGCCCATTTTTTGTCACAAATATGTGTAACGATAATTGGCTGTATATTTATG
GGATCTAGTACCGCTTTCAACGCAATTATCACTGCATGCATCACCCTGTTACTAATGTCGTACGCAGTTCCTTCT
TTTATATTCCTGTTCGTTATTAAAAAGGAAAAATTCATTCATAGAATCGAGAGTGACGTAAATTGTGTCAGCAGG
CCTAACCGTCGTCGTATGTCTATGATTCCTCATATTATATGTATTCTATGGACCTTGTTCTGCCTGGTATTTTTA
TCTTTCCCATATACACTACCGGTTACTGCAGGAAACATGAATTACACCTCAGTAGTGTATGCAGTAGTTTTCTGC
ATTATCAGCATCGTTGTTTTCCCTACATGCATCTAA

>YDL094C 0.81 Cold
ATGGCAGTAAATATCAGCAAACAGCCAACTAGAAGCTTTTCCTTAGATGAAGTAACTGTTCGTAATGAACTCAAA
TTATCACAGGGTTTTGTTATGAGAAAAGACCTCAAAGGGCCGCGTTTAATAGTCACATCGGGGATGGGATCCACC
TTCAGCAAATGCTCTTTATTCATCTTTAAAGCAGTTATGATCTTACACACCTGCTTAATTGTTAAATCAATAAGA
ATATTTTCGAAAAAGAAGAAAAGAAACGGGTCTATGCATAATATGTACTACGCTTCTGTTCCTTTTCTATTGTTC
TCGAATGCCTATTCGATAGACTTTTCGCGGCACGTAAACGAGTTCTTGGAAAAAAAACGGTGCGAAATGATAATA
CCACTGAAACTACTTGCAGATCATACTTATTTGAGCGAAATAGAATACGTGTCTCTAAACGCAGATGGACAATAT
AGTCAGCTTCAAGACATTTTTTTTATTCATGATGTTTTTTTTGTCTATTTTCGTTTTTAA

>YLR017W 0.8Cold
ATGAACAGGATTAAGAATACATTTTCTGTTGCTAAGAGATTAAAACTAAGCAAAGTTATGACGAACTCAGAATTA
CCGAGCATATTCGAAGGAACTGTTGATTTAGGGATTATTGGTGGTACAGGTTTATATAACCTTGACTGTCTGGAG
CCCATCGCTTTGCTTCCACCCATGGTAACACCATGGGGTACCACATCGTCTCCTGTCACAATCTCTCAGTTCGTA
GGAACTAACAGCCACTTTCACGTTGCGTTCATAGCCAGACACGGTATTAACCACGAATACCCACCCACTAAAGTC
CCATTTAGAGCAAACATGGCGGCCTTAAAGAACTTAAATTGTAAAGCCGTTCTTTCTTTTAGTGCCGTGGGGTCT
TTACAACCCCATATAAAGCCTAGAGATTTTGTGTTACCACAGCAAATAATCGACAGAACTAAAGGCATAAGACAT
TCTTCATATTTCAACGATGAAGGCTTGGTAGGTCACGTTGGTTTCGGACAGCCGTTCTCTCAAAAATTCGCAGAG
TATATCTATCAATTCAAGAACGAGATAACAAATCCTGAATCCGAAGAACCGTGCCATTTGCATTACGACAAGGAT
ATGACCGTTGTGTGTATGGAAGGCCCACAATTCTCCACGCGCGCTGAATCCAAGATGTACAGAATGTTTGGTGGC
CATGTTATTAACATGAGTGTTATTCCAGAAGCCAAATTGGCGCGTGAGTGTGAGCTGCCTTACCAGATGATTTGT
ATGTCTACCGATTACGACGCATGGAGAGATGAGGCAGAACCTGTTACCGTAGAAACCGTTATTGGTAATTTGACG
AATAATGGGCGCAATGCAAATATTTTAGCTTCTAAGATCATCGTCTCAATGGCCAAGGAAATCCCAGAGTTCATG
CATACTGGCGATGGGCTGCGCGGTTCCATCAAGAAATCTATCTCTACCAAACCAGAGGCTATGTCCAAGGAAACC
TTAGAAAGACTAAGATACTTATTTCCAAACTATTGGTAA

>YIR010W 0.71 Cold
ATGAGTCTGGAACCCACACAAACGGTCTCCGGTACGCCGCCGATGCTGCATCAAAGAACACACAAACAAGTATAC
CCATTGCGGATGGAAACTATCCCAATATTGGAATCCGACTCCAAGGCTACTCTACAAAGCAACGAGCCCACCCAG
AAAGATGAGGAGGAGACCGAATATTTTGAGAATAAACAATCCGTGTCGAATTTGAGCCCCGATTTGAAATTCAAA
CGACACAAGAATAAACATATCCAGGGGTTCCCTACTCTGGGCGAAAGGCTGGATAACCTGCAAGATATAAAAAAG
GCCAAGAGAGTGGAGAATTTCAATTCTTCTGCGCCAATTGCTGACGACAATCATAGTGGAGATGCGACTGCAAAT
GCGACTGCAAATGCGACTGCAAATGCGACTGCGAATGTTAATGCAAGTGCAATGCCCGCTCCATATATGCCTTAT
TACTATTACTACCATCCGATGAATGCACCAACTCCTGCAATGATACCATATCCTGGATCACCAATGCATTCGATT
ATGCCCAACTCCTCATTACAGCCCTTTTATTCGCAACCCACTGCTGCAGGCGGTCCCGATATGACTACTCCCCAA
AATATATCTTCATCACAACAATTGCTGCCAGCTCCGCAATTGTTTCCGTACGGATCATTCCATCAACAACAACTT
CAGCAACCACACTACATTCAACGAACAAGGGAAAGGAAAAAATCAATCGGAAGTCAGAGAGGTAGAAGATTGTCT
ATGTTAGCATCTCAAGCTAATGGGGGAAGCACTATTATATCTCCGCACAAGGATATTCCTGAAGAGGATTTTTAC
ACCGTGGTTGGAAATGCGTCTTTTGGTAAAAATTTACAAATACGACAGCTTTTCAACTGGTGTTTAATGAGATCT
CTGCATAAATTGGAGTTGAAGGCTAAAAACCAAGAGGAAGAAGGGGAACTTGAACATCTGACCAAAAAATCCAAG
CTCGAATCAACAAAGGCAGAAACTGATTATGTGGATCCCAAACGTCTGGCTATGGTGATAATAAAAGAGTTTGTC
GATGATCTTAAAAAAGATCATATTGCGATAGATTGGGAAGATGAGGAAAAATATGAGGATGAAGATGAGGAGAAG
ATTCTGGATAATACTGAAAACTACGATGACACGGAACTGCGACAACTGTTTCAGGAAAACGACGACGACGACGAC
GACGACGATGAGGTGGACTACTCGGAAATACAGAGGTCTAGACGTAAATTTAGTGAAAGGAGAAAAGCCCTACCG
AAAGAACCAAAAAAACTATTACCTAATAGCAAAAACGTGGAAAACACAAAGAATCTTAGCATTTTAACGAGCAAG
GTGAACGCGATCAAAAATGAAGTGAAAGAATGGGCAGTCACTTTAGACACTTCGAGACCAGATCTTGAATGGCAA
GAATTGACTTCATTCTCATCACAACCACTGGAACCGTTGTCGGATACAGAAGAGCCAGACCTTGCGATTGCAGAT
GTTGAGACAAAGTTAGAAACAAAAGTCGATGAGCTAAGGTACCAGTCGCATATATTAAACTCGCACTCACTAGCC
TTAAACGAAATAACAAATTCAAAAGTGAATAAATTGAACATAGAAACAATGAGAAAGATCTCAAGCGAAACGGAC
GATGACCACTCACAAGTGATTAATCCTCAACAGCTGTTGAAGGGATTAAGTTTATCTTTCAGTAAAAAACTGGAT
TTATGA

>YJL033W 0.8Cold
ATGGCCAAAAAAAATAGATTGAACACAACTCAAAGAAAGACTTTACGTCAAAAGGAAGATGAGTACATCGAAAAC
TTGAAAACCAAAATTGATGAGTATGACCCTAAGATAACCAAGGCCAAGTTTTTCAAAGATCTCCCCATTAGCGAT
CCCACATTAAAAGGTTTAAGAGAATCATCCTTTATAAAACTTACTGAGATCCAAGCCGATTCTATTCCCGTGTCC
CTGCAAGGTCATGATGTATTGGCGGCCGCAAAGACAGGTTCCGGTAAGACATTAGCATTTTTAGTTCCAGTAATT
GAAAAGCTATACCGCGAAAAATGGACCGAATTCGATGGGTTAGGTGCCCTTATCATATCTCCAACAAGAGAGTTG

GCTATGCAAATATATGAGGTTTTGACGAAAATAGGTAGTCACACTTCATTTTCAGCTGGGTTAGTTATCGGTGGT
AAAGATGTTAAATTCGAACTAGAAAGGATATCAAGGATTAACATTCTTATCGGTACACCTGGTCGTATTTTACAA
CATTTAGATCAAGCCGTAGGTCTAAATACTTCCAATTTGCAGATGCTTGTTCTTGACGAAGCTGATAGATGTTTA
GACATGGGGTTTAAAAAAACTCTGGATGCCATCGTAAGCACATTGTCACCATCTAGACAAACACTTTTATTTTCA
GCGACTCAATCACAATCTGTTGCAGACTTGGCTAGGCTTTCCTTGACAGATTATAAAACCGTTGGTACACATGAT
GTGATGGATGGGTCGGTTAACAAAGAAGCATCTACTCCAGAAACTTTGCAACAATTCTACATTGAAGTTCCTTTG
GCAGATAAATTGGATATCCTTTTCAGTTTTATAAAATCACATTTAAAATGCAAAATGATCGTTTTCTTATCAAGT
TCCAAACAAGTGCATTTTGTCTATGAGACATTTAGGAAGATGCAGCCTGGTATTTCTTTAATGCATTTACACGGT
AGACAGAAACAACGTGCTAGGACAGAAACTTTGGACAAATTCAATCGTGCTCAACAGGTGTGTCTTTTTGCTACC
GATGTCGTTGCTAGAGGTATTGACTTTCCTGCAGTCGATTGGGTTGTTCAAGTGGATTGTCCAGAGGACGTTGAT
ACATATATCCACAGAGTCGGTAGATGTGCTCGTTACGGTAAAAAGGGTAAATCTTTAATCATGTTGACTCCACAA
GAGCAGGAAGCGTTCCTAAAAAGACTTAACGCTAGAAAGATCGAACCAGGCAAACTTAATATAAAACAGTCAAAA
AAGAAATCCATTAAACCACAATTGCAGTCATTATTATTCAAGGATCCAGAACTAAAATATCTAGGACAAAAGGCT
TTTATATCGTACGTGAGATCTATATATGTGCAAAAGGACAAACAAGTTTTCAAATTCGATGAGTTACCAACAGAA
GAATTCGCTTATTCTCTTGGTTTACCAGGGGCCCCAAAAATCAAAATGAAGGGTATGAAGACCATTGAGCAGGCC
AAAGAAAGAAAGAATGCTCCAAGACAGCTAGCTTTCTTATCAAAAGCAAATGAAGATGGTGAAGTAATTGAAGAT
AAGTCGAAACAACCAAGAACAAAATATGATAAAATGTTTGAACGTAAAAATCAAACCATCTTAAGTGAACACTAT
TTGAATATAACGAAGGCTCAAGCACAAGAAGACGAGGATGACGATTTCATTTCTGTCAAGCGTAAAGATCATGAA
ATAAATGAAGCTGAGTTACCAGCTCTAACTCTTCCAACTTCAAGGCGAGCACAGAAGAAAGCGCTTTCAAAGAAG
GCATCTCTGGCCTCCAAGGGTAACGCTAGCAAATTAATATTTGATGATGAAGGCGAAGCCCATCCAGTTTATGAA
CTTGAAGATGAAGAAGAATTTCATAAGAGAGGTGATGCGGAGGTACAAAAGACAGAATTTTTAACTAAAGAATCT
GCAGTAATGGCAGACATTGACAATATTGATAAGCAGGTGGCTAAAGAAAAAAAACAAGAGAAGAAGAGAAAAAGA
CTAGAAGCCATGAGAAGGGAAATGGAGGCTGCTATGGAAGAAGAAATTTCTGGAGATGAAGAAGAAGGGAAGACA
GTAGCATATTTAGGAACAGGAAACTTGAGCGATGATATGAGCGACGGTGATATGCCTGACTCGGAAGGCCACTTG
AAAAAGAAAGCTAGAACTGTTGACTATTCCCACGGCCACAATCCATCCAATTCAGTTGATGATGACATTATTGAA
GTGGAAGAACCACAAACTTTGGAGGATTTGGAATCTTTGACAGCTAAATTAATCCAGGGTTAA

>YFL042C 0.8 Cold
ATGTCGGATGTAGATAACTGGGAACCCGTATCAGATAATGAGGACAGTACTGATTCTGTGAAACAATTGGGTCCC
CCCTTTGAGCATGCAAGTAACAATGACAATGCCGGCGATACTGAAGCTGAGTCACTGCAAGAAGTGCCCCTAAAT
ACAGAGACCAACGACGTACGCAAGAATTTAGTAGTGATTACAAACCAAAGTGCCGCCGATGAACACCCTACAGAG
ATCAAGCACGATCAATCAAGGACTTCTTCGACGTCCTCGTTCTTCAGTGGAATGATCTCTTCGTTTAAGTCAAAC
GTGCCATCCCCAGTTTCTAGATCAACTACGCCAACTTCGCCAGTTTCGCAACCAAGCATTATATCACATAGAAGG
GAGCCTTCCATGGGGTCCAAAAGAAGATCAAGCCGCCGTATCAGTAATGCCACAATTGCTGAAATAGGCTCTCCC
TTGCAACAGGTGGAAAAGCCTGATGAGGTCAAGACAAGACTCACTCCTTCCCAAATGAAAGAAGACAATTACGAT
CATAGACGATTCGTGGAAGAACGTTACATGGACACACCTTATCACTATGCGTCTGAGCAAAGAAATAAAGACTTT
CATGAGACATTCAAGTCCGTTCCTAAAGATGACAGACTACTGGATGATTTTAATTGTGGGCTGAATAGAGAGCTG
CTTTACCAGGGGAAACTATACATAACAGAAACGCATCTCTGCTTCAACTCCAATGTTCTTGGTTGGATTGCTAAA
GTACTGATTGCGTTCGAAGATGTCACGTTTATGGAAAAAACTTCTGCTGCTGGGTTGTTCCCCAGCGCAATATCC
ATCGAAACAAAGATGGGCAAAACTCTCTTTAATGGTTTTATATCCAGGGATGCTGCATTTGGATTAATGAAAGAA
GTGTGGTCAAGAACCTTGTTGCAGAAGGACATGGCCAGCGAAAACATCAATACTAAAGCAGAAAAGTCAGGAAAT
GGCAAAGAGATTGATGATGCAATAAACTCCATCGATGAGGAAAACAATGATAAAGACGCTAATGATAATGACACT
AATGAAAATGACGATGAAAATATCTCTACAAATGAGACTACCCCCAATAGTACGAGTTCCTCGCCGGATAAAGAA
AAAGAGAAAGCGTATAAACTGCGTGCCGATTCCTCGTATCAGTATGATGGTCCAATATATCATCATTCAACAAGC
TTTCCGGCTGAACCTATGGCCAATAACGAGTTTGTTCTCAAGGAGTTACCATTCGATTGTGCACCTGGTATACTT
TTTGAGATCATGTTCAACTCAGAGCAAAACGAATTTCTTCTAGATTTTTTACGGGGTCAAGAAGGTTCACAAATT
ACCACTATTCCAAATTTCACGAGCATTGACGGATCTTCCATGACTTTGAAGCGTGAGTATTCATACGAGAAAGCA
TTGCACTTTCCTGCGGGGCCCAAGTCCACGACATGTTATGTTGCTGAGGTAATAAAGAGAAAAGATCCTGATACT
TACTATGAGGTTATCAGTAGCATACGCACTCCGAACGTGCCCAGTGGTGGTAGTTTCTCAACTAAGACAAGGTAT
CTAATCCGTTGGAATGACGAAATAACGTGTCTGTTACGGGTATCCTTTTGGGTGGAATGGACTGGTTCCAGTTGG
ATCAAAGGTATGGTTGAAAATGGATGCAAGAATGGTCAATTGGAGGCTGCGCAATTAATGGAGCGTATTCTTTCC
AAGTTCATCAAGAACAATGTCGAAGAGTGCCAAATTACTATCAGCAAGGAGGAAGAGGAGCAAGATGATAAAGAA
GTAAAAAATAAGTTGAAAGAGGTTGACCTTGAACAACCAAGAGAAGCGGTTGTTACGGCTCCCGCAATTGCAGAG
CAGCAAGGTCTCAAGGTCACCATGGAAACATGGTTGTTCTTATACTTGATTGTGGTCGTTTTGCTATTGTTTAAT
CTGTTCTACATACGTTCAATTGCTGTCTCATTACATCAACTGGTGAAGCTGCAATTGGTAGAACTGAAGTTGTGA

>YOL131W 0.8 Cold
ATGAATACTAATAAGATAGCACAAGACGAAGTCCAAGATAAGGTTTTGCAAAGAGCAGAACTAGCACATTCTGTA
TGGAACTTAAGGTTCAACCTCAGTAAAGTTGCCAAACGGATTCGCATGGAAACAAAGGTATTTCCAGAGATAAAG
ATAAATGACGCGCAATCACAGTTAGAGCGATCTAGGTGTAGAATATTTAGCCCTGACCTGGAGGAAGAACATGTG
CCCTTGATTCAAGGCTTTAAATGTTTGGATTCACCCCCTCCCGTTCCACCAAGCTCATCGCAGGGGGAGGATGAA
GAGAACACGGTAGATTCGCAATACTAG

>YHL043W 0.75 Cold
ATGGAGGGCCGCAAAAGTGAAGATGAAAAGAACGAAGCCGCTTTAGCCTGTGATGTCTTTGAATCCTCCAATGCC
AAACTACCAAAGAATGTATTCAGAAGTTCCTTTACTTGGTATTGCTATGAAGTTATCAATAGGTCAGCATTCCAT
ATTTGGTTATTGTTATGTTTAACGCTCATTGTTGGTTGGAAGGTTTTTTCAGGCATCGGTGGTAGAAGACCATCT

GACTCAAACATGGACGGCCCCCAAACCAAACATAAACGGAATCCAGGTTTTTTGAGGCGTCATTCGACCATCGTT
ATCTTAGTTATATCTTTAGCAGTGTCTTTCTCATGGGAAGCTTTCAAAATGTATCGTGAACGCACCTTCGGGAAA
CAAATCACTCAGTTTGCCAAAGAAATTATTAAAAGTGCGCCAAGCACAGATATGGAAAGCTGGGACCGTGTTGCA
GCGGATTTTAACTCTTATATGTACGAAAATAAACTTTGGAACACAGAATACTTTTTTTGTTGA

>YOL165C 0.81   Cold
ATGGCTAGGCATTTCGGTATGGCCCTCGCCCCATGGGATGTCATGGGAGGTGGAAGATTTCAGAGTAAAAAAGCA
ATGGAGGAACGGAGGAAGAATGGAGAGTGTATTCGTTCTTTCGTTGGCGCCTCCGAACAAACAGATGCAGAAATC
AAGATTAGTGAAGCATTAGCCAAGGTTGCTGAGGAACATGGCACTGAGTCTGTTACTGCTATTGCTATTGCCTAT
GTTCGCTCTAAGGCGAAAAATGTTTTTCCGTCGGTTGAAGGAGGAAAAATTGAGGATCTCAAAGAGAACATTAAG
GCTCTCAGTATCGATCTAACGCCGGACAATATAAAATACTTGGAAAATGTAGTTCCTTTTGACATCGGATTTCCT
AACACTTTTATCGTGTTAAATTCCTTGACTCAAAAATATGGTACGAATAATGTTTAG

>YLR362W 0.75   Cold
ATGGAACAGACACAAACAGCAGAGGGCACTGACTTACTAATTGGTGACGAAAAGACCAACGATTTACCTTTTGTG
CAGTTATTTCTGGAGGAAATAGGATGCACTCAATACCTGGATAGCTTTATTCAGTGCAACCTTGTCACAGAAGAA
GAAATTAAGTATCTCGACAAGGATATCCTCATTGCTTTGGGTGTAAACAAAATAGGAGACAGACTCAAAATTTTA
AGGAAGTCAAAATCGTTCCAGAGAGATAAACGGATTGAACAGGTGAATAGATTGAAAAACCTGATGGAAAAAGTA
AGCTCTCTATCCACTGCTACGCTATCGATGAATTCAGAATTGATTCCTGAAAAGCACTGTGTTATATTTATCTTA
AACGATGGTTCCGCTAAGAAAGTTAATGTAAATGGTTGCTTTAATGCAGATTCTATTAAGAAAAGGCTAATCAGA
AGATTGCCACATGAATTATTAGCCACAAACTCCAATGGAGAAGTAACTAAAATGGTCCAAGATTATGATGTGTTT
GTCTTAGATTATACCAAGAACGTACTGCATTTGCTATATGACGTGGAATTAGTCACTATTTGCCACGCAAATGAT
CGTGTTGAGAAAAATAGGCTAATTTTTGTTTCCAAAGACCAAACACCAAGTGATAAAGCTATATCCACATCCAAA
AAACTATATCTAAGAACGTTGAGTGCATTGAGCCAGGTTGGGCCATCCTCGTCAAATTTGTTGGCACAGAACAAG
GGGATTTCGCATAACAATGCTGAAGGGAAACTCCGGATCGACAACACAGAAAAGGACAGAATTAGACAGATTTTT
AATCAGAGGCCTCCTAGCGAATTTATTTCTACCAATTTGGCCGGATATTTTCCTCATACAGACATGAAGCGGTTG
CAAAAGACGATGAGAGAGTCATTTCGCCATTCAGCAAGGCTAAGCATTGCTCAAAGAAGACCTTTAAGTGCAGAA
TCAAATAATATCGGTGACATACTATTGAAACACTCAAACGCTGTTGATATGGCCCTATTACAAGGATTAGATCAG
ACAAGATTAAGCAGTAAACTTGACACAACTAAAATTCCGAAGCTTGCCCATAAAAGGCCAGAAGATAATGATGCC
ATATCTAACCAGTTAGAACTATTAAGTGTAGAGTCTGGTGAAGAAGAAGATCACGATTTCTTTGGGGAGGACAGT
GACATTGTTTCATTACCGACGAAAATTGCCACGCCCAAGAATTGGTTAAAAGGTGCTTGCATTGGATCAGGCAGT
TTTGGGAGTGTTTACTTGGGCATGAATGCTCACACTGGTGAACTAATGGCAGTAAAGCAAGTGGAGATAAAAAAT
AATAACATTGGTGTTCCCACAGACAACAATAAACAAGCCAATTCTGATGAGAATAATGAGCAGGAGGAACAACAA
GAGAAAATAGAAGATGTTGGGGCGGTAAGTCATCCAAAAACCAATCAAAATATTCACAGAAAGATGGTTGATGCT
TTACAGCATGAAATGAATTTATTGAAGGAGTTACATCATGAGAACATTGTTACTTATTATGGTGCTTCTCAAGAA
GGCGGAAATTTAAATATTTTTCTTGAATACGTTCCTGGGGGTTCGGTTTCCTCCATGCTGAATAATTACGGTCCA
TTTGAGGAATCACTGATTACTAATTTCACTAGGCAAATACTGATTGGGGTTGCGTATTTGCATAAGAAGAACATT
ATTCACAGAGATATCAAGGGTGCAAATATTTTGATTGATATCAAAGGTTGCGTAAAAATTACTGATTTTGGTATT
TCAAAAAAATTATCACCTTTGAATAAAAAACAAAATAAGAGAGCTTCTTTGCAAGGTTCCGTATTCTGGATGTCA
CCAGAGGTGGTCAAACAGACCGCTACTACTGCTAAAGCGGATATATGGTCTACAGGATGTGTTGTCATTGAAATG
TTTACCGGTAAGCATCCTTTCCCAGATTTTTCTCAAATGCAAGCGATCTTCAAAATAGGCACAAACACGACCCCC
GAGATACCTTCCTGGGCTACGTCAGAAGGAAAGAATTTCTTAAGAAAGGCATTTGAGTTGGATTATCAATACAGG
CCTAGTGCCCTTGAATTGCTGCAGCATCCATGGCTGGATGCACACATAATTTGA

>YOR257W 0.76   Cold
ATGAGTAAGAACAGGTCATCGCTACAGTCTGGTCCACTTAATAGCGAGTTATTGGAGGAGCAAAAGCAAGAAATA
TATGAGGCGTTTTCATTGTTCGATATGAACAATGATGGGTTCCTCGATTACCACGAGTTGAAAGTAGCAATGAAG
GCCCTGGGATTTGAGCTACCCAAGAGAGAAATACTTGACTTAATAGATGAATATGATAGTGAGGGGCGGCATTTG
ATGAAGTATGATGATTTTTATATTGTAATGGGGGAAAAAATATTGAAGAGAGATCCGTTAGATGAAATAAAAAGG
GCATTCCAATTATTTGATGACGACCATACTGGGAAAATAAGTATTAAAAACTTGAGGCGTGTAGCTAAAGAATTA
GGAGAAACTTTGACGGATGAAGAGCTGAGAGCGATGATTGAAGAGTTTGATTTGGATGGTGATGGCGAGATTAAT
GAAAACGAATTTATAGCTATTTGCACCGATAGTTAA

>YNR024W 0.78   Cold
ATGAGTGCTAACAATGGTGTCACTGGCAAACTCTCTAGTAGAGTCATGAATATGAAGTTTATGAAATTTGGTAAG
ACGGATGACGAAGAGAGTTCCAACTCCAATACGCCGTCTAATATCAACTCAGATGTGGAACCTATAGAGCAGAAA
GGTAAACTCTTTGGTCTGGATGACTCAGCGTGGGACCTCAATAGCTACAAAGATGATTTAAAAAAAATTTCAGGT
AAGGAGAAGAAAAAGGTGAAAAGAGTAGTATATAAGAAGCGACCCAATCTCATAATCTCCAACGTTGGTTACAGT
GAACTGCGAAAACCTGAGGGTGTGATAAGTGGGAGAAAAACCTTTGGCGATAATTCTGATGATAGTGGTTCCAGG
AAGAGAAAGTTTGATGAAGGCGAACAAAATGAAGACGAGAAAAGGGATGCTAAGGATAAGGAATTTACAGGGAGC
CAAGACGATGGAGAGGATGAATATGACCTAGATAAATTATTTAAGGATAGCATCAAAAAGAAAAAGACCAACCAT
AATGGTAAAAATAAAAATAGAAATTCAAAAAAGTAG

>YNL003C 0.73   Cold
ATGAATACTTTTTTTCTTTCCTTGCTAAGTGGCGCCGCTGCTGGTACCTCTACGGATTTGGTTTTTTTCCCTATA
GACACAATTAAAACCAGGCTTCAAGCTAAGGGTGGATTCTTTGCTAATGGAGGATACAAAGGCATATATCGTGGT
TTGGGAAGCGCTGTTGTAGCATCGGCGCCAGGTGCTTCACTTTTCTTTATTAGTTATGATTATATGAAAGTAAAA

TCAAGGCCATATATCAGCAAACTTTACTCACAGGGATCTGAGCAATTGATTGATACGACAACGCACATGCTTTCC
TCTTCCATTGGTGAAATTTGTGCTTGTCTTGTTCGAGTTCCCGCAGAAGTCGTTAAGCAAAGGACGCAAGTTCAC
TCTACCAATTCTTCCTGGCAGACTTTACAGTCAATTCTACGAAATGACAATAAGGAAGGTTTAAGGAAAAATCTT
TATAGAGGGTGGTCTACCACCATTATGAGAGAAATACCGTTTACTTGTATACAGTTTCCTTTATACGAGTATCTA
AAAAAGACTTGGGCAAAAGCCAATGGCCAAAGTCAAGTGGAGCCATGGAAAGGGGCTATCTGCGGTTCTATTGCG
GGTGGGATCGCAGCAGCTACAACGACACCATTAGATTTCTTGAAAACCAGATTAATGCTCAACAAAACAACCGCC
TCACTTGGAAGCGTTATTATTAGGATTTACAGAGAGGAAGGTCCTGCTGTATTTTTCAGTGGAGTGGGGCCCCGG
ACAATGTGGATCAGTGCAGGTGGTGCTATCTTCCTAGGCATGTATGAAACTGTTCATTCGTTATTATCGAAAAGT
TTCCCAACGGCTGGAGAAATGAGAGCGTAA

>YOR004W 0.79   Cold
ATGCGTCAAAAGAGGGCTAAGTCATATAGGAAACAACTTCTTGTTTACAGTCACACATTTAAGTTCCGTGAGCCA
TATCAAGTTTTGGTTGATAATCAGCTTGTTTTAGAATGTAATAATTCTAATTTTAATCTACCGAGTGGGTTGAAA
AGAACGTTGCAAGCAGACGTAAAGGTCATGATCACACAGTGTTGCATACAAGCGTTATATGAAACTAGAAATGAT
GGTGCAATTAATTTAGCGAAACAATTTGAGAGACGTCGTTGTAACCACTCTTTTAAAGATCCAAAGTCACCTGCG
GAGTGCATCGAAAGCGTCGTTAATATTAGCGGTGCAAATAAACACAGGTATGTGGTTGCTTCTCAGGACATAGAT
TTGAGGAGGAAGCTGAGGACGGTTCCGGGTGTTCCATTAATCCATTTAACCAGGTCTGTTATGGTTATGGAACCT
CTCAGTACGGCGAGTGCCAAGGCAAGCAAGATTACTGAAGAACAAAAATTGTATAAGGGTCTCAATGATCCTAAT
ATTGAAAAACTTCAAGAAAGTGGTGATGGATCAGGAAAGGAATCTATCACTAAGAAAAGAAAGCTTGGTCCAAAG
GCTCCTAATCCTTTGAGCGTAAAGAAGAAGAAGAAAGTCAACTCGCCCAGCGATGAGGTGAAAGACAAAGAAGAT
ACTTCTAAAGAAAAGAAAAAAAGAAGAAGAAGGAAACACAAAAGCAACACTAACGTCCCCGTTTCAAATGGGACC
ACAGCCGCGCAGTAA

>YFL001W 0.78   Cold
ATGAGTAATTTCATTAGAAGGCTAGTTGGGAAAATGAAAGCGATTTCAACAGGTACCAATGCTATTGTTAGTAAG
AAGGACTCCATTTACGCAAACTGGTCCAAAGAGCAGCTAATACGAAGGATCACTGAACTAGAAAACGCAAATAAG
CCACATTCTGAAAAATTCCAGCACATCGAGGATAACAAAAAGCGCAAAATTTCGCAAGAAGAGGTTACGAGAAGC
AAGGCGAAAAAGGCTCCGAAGAAGTTTGACTTTTCTAAACATAATACCAGGTTCATCGCCTTGAGATTTGCCTAT
TTGGGATGGAATTACAATGGCTTAGCTGTTCAGAAGGAATACACACCGTTGCCTACAGTAGAGGGTACCATTTTG
GAGGCCATGAATAAGTGTAAACTTGTTCCCTCGATGGTTTTACAAGACTATAAATTTAGCAGATGTGGCAGAACA
GACAAGGGAGTTAGCGCCATGAACCAAGTTATATCGTTAGAAGTCCGTTCCAATTTAACAGATGAGGAACAGCGG
GATCCGACCAACGACAGCAGGGAAATACCCTATGTTCACGTTTTAAATCAATTACTACCCGACGATATTCGTATA
TCAGCTGTCTGCCTCAGACCGCCACCTAACTTTGACGCAAGATTCAGCTGTGTTCACCGGCACTATAAGTACATC
TTTAATGGAAAAAACCTTAATATTGAAAAAATGTCTAAAGCCGCATCATATTTTGTTGGAGAGAGAGACTTCAGG
AATTTTTGTAAGCTTGATGGCTCAAAACAAATTACCAATTTTAAACGAACAATAATAAGCTCAAAAATTCTTCCC
CTTTCTGAAACTTTCTATTGTTTTGATCTCGTTGGTTCAGCATTCCTATGGCACCAAGTTCGTTGCATGATGGCC
ATTCTTTTCTTAGTTGGTCAATCACTTGAAGTGCCGGAAATTGTCTTGCGTCTGACTGATATTGAGAAAACCCCT
CAAAGGCCTGTTTACGAAATGGCAAATGATATACCATTATTGTTATATGATTGTAAATTTCCTGAAATGGATTGG
CAAGAACCTACTGTAGATGACTATAAGGCAATAAAATTCACGACAGCTACTGAAGCATTGACGTTACACTACGAA
CTTAAGGCCGCAGTATGCAATATTTTTAAAGATGTTTTGCCCACAGCAAATACAAACAACTTCTCAAAGACAATT
ATTAATTTGGGCGATGGAAGAGGTAAAGTAGTCGGTACCTATGTGAAACTAGAGGATAGAAGCGTTATGGAACCT
GTCGAAGTTGTTAATGCTAAATACTCCAAGAAAAAGAACAACAAAAATAAGTAA

>YLR031W 0.78   Cold
ATGCCGGTCTTAAATACGAGAACTTCTTATCCCAATATCGACTTTCATGGTACTAAGGTTTCTGATGTTCTAGAT
GCCTTTGAATTTGAAAAGCATGATGATCCCTTAAGGGATAAATGGAACACTTTACAGTTTTTGGAAAAGAGCTTT
GAATCTAAATTTGAATCTGCATCAGAGTTAATCCAAGGAGGCGAGCTAGCTGCTATTAAAGAAAGAAATTTTCAA
TTAGCCAAACTAAATAATCTTTGCTTTCGGGTACGAGAGTCCATCAAAAGAAGACAAGACCTGGAGAAAAAGTTA
AGAACTTTATCGCAGGACACTGACAACGAATTGCTTTTTCTGATGTTGGAAAATGAGAGAAGAAAGAAAAGTTCA
GTGATAATAGAGTTTCTTTCTGAGATAATCAGAGAGAAATCCAAAAGATTAACTGCAGAAGAGCAAGGCTTTGTA
AATCAAAATGAAGTAAAACCCCTCATTCTGGACCTTTCTGCACGGATAAATAGATTGAATTCTATTCTAGAAACA
AAAAATACTTGTATTAGAAGATTAAGTAATCAATAA

>YOL146W 0.72   Cold
ATGGGTTACTATGACATTGATGATGTCCTAGCAGATGGGACAGAGTTTCCTTGTAAATTTCAATATGATATTCCT
GGTCTTGGTTATTTGGAAAACAACCCAGGACGACCCATAACGAAAAACACTAAACTAAGCCTGCCACTATGGTTA
GCAAGAATCCTTGCAATTGTGGGTGGTGACGAAGCCTTGGTAGATGAAGAACCTGTACCTTTTGTGGAACTCCTG
CCACCAGATATGTTTTCTACGAAAGTTATGAACGCTATAAAGACCGATCCGGTGGCTCTGGACTTGCATTCCATA
AATTCGCATTTCTTCAGTCTAGCCATAAAATGGATAATGTTGTTTAGCGAAAAAGAACTGGCCAATGTCGTTAGT
GAGCTACTTTTGCAACGTGCCCAAGAACTTAATCATCATGCTAGTAGTTTATCGATTGATCTCAATGCAGACTCG
ACCGGAAAAAAATTCAGCGAACACTAATATAGCAACTAGCACATTTTTACTGAAACTAGAAGAAATGGAGAAGGAG
ATATATAAAAAGTCTCATGAATCTTATAAGGACACGAAAAGGTGGATGTTTAAAAAATAA

>YDR324C 0.71   Cold
ATGAGCTCATCGCTACTTTCAGTTTTAAAAGAAAAGTCAAGAAGCCTGAAAATACGAAACAAACCGGTGAAGATG
ACCAGTCAGGAAAGGATGATAGTGCATAGATGCAGATTTGTGGATTTCACTCCTGCCACAATCACATCTCTAGCA
TTTTCACATAAATCAAATATAAATAAGTTAACTCCGTCCGATTTGAGATTAGCTATTGGTAGATCTAATGGTAAC

ATAGAAATCTGGAATCCTAGAAACAATTGGTTTCAGGAAATGGTTATCGAAGGTGGCAAAGACAGATCAATTGAA
GGCCTTTGCTGGAGCAACGTTAACGGCGAGTCTCTAAGGCTATTTTCTATTGGCGGCTCGACTGTGGTAACAGAA
TGGGATTTAGCAACAGGTTTACCATTAAGAAACTATGATTGCAATTCAGGTGTGATATGGTCTATTTCCATCAAC
GATTCACAAGACAAGCTGTCTGTGGGTTGCGATAATGGGACGGTGGTCCTCATAGATATCTCTGGCGGGCCTGGT
GTCTTGGAACACGATACTATTTTGATGAGACAAGAAGCCAGAGTATTGACTTTGGCTTGGAAAAAGGATGACTTC
GTGATTGGTGGTTGTTCTGATGGTAGAATAAGGATTTGGTCTGCACAAAAAAATGACGAAAACATGGGTCGTCTA
TTACACACTATGAAGGTCGACAAGGCCAAAAAAGAATCAACTCTAGTTTGGTCAGTTATATATTTACCAAGAACT
GATCAGATTGCCTCTGGTGATTCTACAGGCTCCATTAAATTCTGGGATTTCCAGTTTGCCACGCTAAACCAGTCA
TTTAAGGCGCACGATGCAGACGTACTGTGTCTAACTACCGATACTGATAATAATTATGTTTTTAGTGCTGGTGTG
GACAGAAAAATCTTTCAATTTTCTCAAAACACTAACAAATCTCAAAAGAACAACAGATGGGTAAATTCTTCTAAT
AGGTTGCTTCATGGAAACGACATTAGAGCAATATGTGCATACCAATCTAAAGGTGCAGATTTTCTAGTTTCAGGA
GGTGTTGAAAAAACACTAGTCATCAACTCACTTACTTCTTTTTCTAATGGAAACTACAGGAAGATGCCAACTGTC
GAACCTTATTCAAAGAATGTTTTAGTTAACAAAGAGCAACGCCTTGTTGTTTCATGGAGCGAATCTACTGTTAAG
ATATGGACAATGGGAACCGATTCTAGTACAGAACAGAATTATAAGCTAGTTTGCAAGTTAACTTTAAAAGATGAC
CAGAATATCTCAACTTGTTCTTTATCACCTGATGGACAAGTTTTAGTCGTAGGGAGGCCCTCTACCACTAAAGTT
TTTCATTTACAGCCCGTAGGTAATAAATTGAAAGTGACTAAACTAGATAATGACCTATTATTGAGAACTTCCACA
AAATTAGTCAAGTTCATCGATAATTCCAAAATTGTCATATGCTCTTGCGAAGATGATGTATTTATTGTGGATCTA
GAATCTGAAGAAGATGAAAAACCGCAAGAAGTTGAACTTTTGGAGGTTACTTCAACTAAGAGCAGTATCAAAGTT
CCCTATATTAACAGGATCAACCATTTGGAAGTAGACCAGAACATTGCAGTAATTTCTCGTGGCTGTGGAGTCGTA
GACATATTAGACTTAAAAGCAAGGATTTCTAAACCACTTGCTCGTTTGAATAATTTCATCACCGCTGTTCATATC
AATACTTCTAGAAAATCTGTAGTAGTAATTACTGCAGACAATAAGATTTATGAATTCAATATGAATTTGAATTCG
GAAGCCGAGAATGAAGATAGCGAAAGCGTATTAACTCAATGGTCAAAAAACAATACGGATAACTTACCAAAAGAA
TGGAAGACATTAAAAGAGAACTGTGTAGGGATATTTTCAGACATAGAAAATAGTAGCAGATTATGGTTTTGGGGG
GCTACTTGGATATCAAGAATAGACTTTGATGTTGACTTCCCTATAAATAAGAGAAGAAAACAGAAAAAACGTACA
CACGAAGGGCTAACTATCACAGATGAAAGTAATTTCATGAACGACGAGGAAGATGACGAAGATGACGATATTGAT
ATGGAAATCAGCGAAAACTTGAATGTATTATTAAACCAAGGAAACAAAATAAAGTCCACAGATGTACAAAGAAAC
GAAGAAAGCTCCGGTCACTTCTTCTTTACTGATAAATATAAACCTTTACTGTTTGTTGACTTAATTTCTAGTAAT
GAATTGGCAATAATTGAAAGAAATCCATTAACTTTTCACTCCAAACAAAAGGCGTTTATTCAACCAAAGTTAGTG
TTTTGA

>YJL155C 0.75   Cold
ATGGGGTACAGTACTATTTCCAACGATAATGATATCAAAGTATGTGTGATAATGGTTGGCCTACCAGCTAGAGGA
AAGTCTTTTATTTCCCAAAAAATTATCAGGTACTTATCGTGGTTATCCATAAAAGCCAAGTGTTTTAATGTGGGA
AATTACAGAAGAGACGTGAGTGGAAATGTCCCAATGGATGCTGAGTTTTTTAACTTCGAAAATACAGATAATTTT
AAACTCAGAGAATTGGCTGCCCAAAATGCCATAAAAGATATTGTTAATTTTTTTACTAAAGAAGACGGATCTGTG
GCAGTTTTCGATGCTACTAATAGTACACGTAAAAGAAGGAAATGGCTTAAAGATATATGTGAAAAGAATAATATT
CAACCGATGTTTTTAGAGAGCTGGAGTAACGATCATGAACTGATTATAAATAACGCTAAGGATATCGGTAGCACA
TCTCCTGATTATGAAAACTCTGAACCTCATGTGGCGGAAGCTGATTTTTTGGAAAGAATTAGACAATATGAAAGA
TTTTATGAACCTTTGGACCCCCAAAAAGATAAGGATATGACGTTCATTAAGTTGGTCAATATTATTGAAGAAGTA
GTAATTAATAAGATCAGAACATATTTGGAAAGTAGGATTGTATTTTATGTTATGAATATTCGTCCTAAACCAAAA
TATATCTGGCTCTCCCGTCACGGCGAATCGATCTATAACGTAGAGAAAAAAATTGGCGGGGATTCATCACTGTCT
GAAAGAGGCTTTCAGTACGCTAAAAAATTGGAGCAGTTAGTGAAAGAGAGCGCAGGAGAAATAAATTTGACCGTG
TGGACTTCCACCTTAAAAAGAACACAACAAACGGCAAATTATCTTCCCTATAAGAAACTGCAATGGAAAGCACTT
GATGAATTAGACGCTGGCGTTTGTGACGGAATGACGTATGAGGAAATTGAAAAAGAATATCCTGAAGATTTTAAA
GCACGTGATAATGACAAATACGAGTACAGATATCGTGGTGGAGAATCATACAGAGATGTAGTGATTCGTTTAGAG
CCCGTCATTATGGAATTGGAGCGCCAAGAAAATGTTCTCATTATAACTCATCAAGCCGTACTTCGGTGTATATAT
GCATATTTTATGAACGTTCCACAGGAGGAATCCCCTTGGATGTCAATCCCACTACACACATTGATCAAGCTGGAG
CCTAGGGCCTATGGCACAAAGGTCACCAAAATTAAAGCAAACATCCCTGCAGTGAGTACATATAAAGAGAAGGGT
ACAAGCCAAGTAGGTGAGCTTTCTCAAAGCTCAACTAAACTTCATCAACTGCTCAATGACTCTCCTTTGGAAGAC
AAATTTTAA

>YKL175W 0.77   Cold
ATGGAAAAAATTCCCAGGTGGTTGTTGTTTTCTCTTATATCGTCGGTGCTGTGCATACTTGGGGCCCTGTGTGTG
CCGTTGTTATCGGTTGCCTTCGATAGCAAGCGCAATAGCCAATCTAAGTTGGTCAACTATGGTCTTTCTCTAAGT
GCCGGATCTATGATCACTACTTCACTATACATGCTATTGCCTCGGATCGAAAAATCAAACCGGTTCAAGGTTTTT
CCTGGATTGCTTTTGGGTATTTGCCTCAGTTTTTTCTTAAACTACCTAGTCCACGCTTTCGCCAGCGAATCGTTG
GTACACTGTGCTGATAGCGGTGACCATGCTACAGGTTCACATATACACTCCAAATCACATTCGCATTCGCACTCA
CACTCACATGCGGATTCGCATTCGAATTTTAGCAACGATCATGATTTAGAAAATGCTCCCTCTGAGCATGGTTAT
GCAACATCTTCTTCCAGTGTTTCTGAAAATGACCCGTTGATTACAAAGGATAGCGATAGACCCCAAATGAAAAAG
AAAATGTCCCTAATTGACTTATTAACCAGAAGGAAATCAGAAGGCGAGTGTTGTGACCTAAACAAGTGCACTCCG
CTCTTGCAATCAGAGCAGCCAGAATATATTGCATGTGTCCCACCAGTGATAAAGTCTTCTCAAAGTGAAAGAAAC
GTGCCACATGGATGCGAGGGATCAGAAGATAATGGACAATCCGATGACAAGGATCATCGTGGCCTTGTATGCGTG
GAAAATAATATCGGTTACGATCTTGAAAACCTATCGTTGTACCGTAAGAATTTTCTTTCGAGCCGCCATCATCAT
TCCTCTGAATCGCCCGAAAATTATGGTTCCAACCAGTTGTCGCACTCTTTTTCGTCACCTTTGGGAAATGATGTA
ACTGAGAATCCGGCCGCATTGGCAGACACACAATACCATCCCGAAAATGGTTCATTATACCCCCACCATCATCAC
TTAGAGACCCCTTTTTCCAAATTACTGTCTATTGGTATGCAAACTTGTCTAGTCCTTGCATTACACAAATTTCCC
GAAGGGTTTATTATTTTTTACACTAACAGGTCAGATTCGTCAAAGTCCTTGGGATTTTCCATTTTTTTAAGTTTG

ACGATCCATAATTTTGTCGAAGGATTTGCAATGACGTTACCATTCTACACTGTTTTCGAATCTAAATGGGTTGCC
ATTTTGATTACTGCCGTTCTCGGTGGTGGATCTCAACCATTAGGTGCATTGATAGGTTATTTCATCTTTAAAGGT
AGTACTCCAAGGGATCACGAACCCAACATGGATTTTTTACTGAGCGTTACTGCAGGGTTCCTATTAGTAATTGGT
TTACAAATGTTCCAAACTGGTATCGGATTTAGCGATGGACATCATCATCATCAAGGAGAAGGTGATGAAGAAATG
AAACAGTCCCATAGCTCAGGCACAACTTGCTTGAAATGGTGCTGCACTGGCGTTTTGCTAATTCTAGCGAGTGCC
CTTTTCACTTGA

>YHL010C 0.81 Cold
ATGGATCAATTTGAGTATATCATTACTTTAGAATTTGGAAACCAAAATCAGGTTGAATCCGCTTATCAAATTTTC
AAGAGTATTCCGAAAAAGTTGAAGGCAAAAAGTATCGGGGAAGAAAGTATTAAAAGTAATAATCAAGACTGGCAG
GATTGGAGAGTCTGTGACTTGGAAATAGACATGATAACTGATTTCAAAAATCAAACATCAAAAGAAGAGGAAAGT
GATTTGATTACTTCACAATACTTAGGCCATGGGATAATAAGGCTATTCAAGCTAAGCAATGCCAATAATACTCTT
AATGAGAAGGAGATTTTGACTATCCCGGGAGATGATACTATGATATGTATCCTGTTTGTTCCAACTTATTTTACC
GTTCACGATTTATTGCATTTTTATATTGGTGATGATATTGTTAATAAGCAAGTTTCAAACTTTCGTATATTACGA
AATCAGCAGAAGGGAATGGGTTTTAATTTCACGGTCTTAATTAAATTCAGGAATGCACTTGATGCGAAAAATTTC
AAGGAAGAGTTCAATGGCAAAAGTTTCAGCAGGATGGATCCCGAAACATGCCATGTGATCTCTGTAAAAGAAATA
GTTTTTCAAAAAAAGCTCTTCCAAAGACCAGCCGCTAACGAGGACTTCCCCTATCTGCTTACTGATCCATTCACT
GTTAAGAAAAAAAAAGGAACTGGTAAAAGTCGAACTCCCCACATGTCCCGTTTGCCTTGAAAGAATGGATTCAGAA
ACTACTGGATTAGTAACGATCCCGTGCCAGCACACTTTTCATTGCCAATGCTTAAATAAGTGGAAGAATTCAAGG
TGTCCAGTGTGTCGACATTCAAGTCTGCGGCTAAGCAGGGAATCATTATTGAAACAGGCAGGTGATTCCGCACAT
TGTGCAACATGTGGTTCCACTGATAATCTATGGATTTGCTTGATCTGCGGTAATGTTGGATGCGGTCGTTACAAT
TCCAAACACGCAATCAAGCATTATGAAGAAACTTTACATTGTTTTGCGATGGATATACGGACACAAAGAGTGTGG
GATTATGCGGGTGATAATTATGTCCATCGCCTAGTTCAAAATGAAGTTGATGGGAAGTTAGTAGAGGTCGGCGGC
TCCGGTGACGATGACAACAATGATATAGGCAACTCCGATGAACTACAAAATGTTGTTTACGGGAACAGGAGCAAA
AACGGTGAGAAATCGAACTCTAATAAAAAAGATGGCGAGCTGGCGGCAAATTTTTTAAGGCATAGGGAGTATCAT
CTAGAATATGTGCAAGTCCTAATATCTCAATTAGAGTCTCAAAGAGAATATTATGAACTGAAATTACAAGAAAAA
GATCAAACTGCGTCTGATTCTTCGAATGTTGAAAGTTTGAAGAAGTCTATGGAAGATTTGAAACTCCAATTTCAG
GTCACTCAAAAGGAGTGGCAGAAAAGGGAAATGGCCCAGAAATCTAAGCTTGAGGAAGATATGTTAGTAATCGAA
GGACTACAAGCGAACCTAGATCACTTATCAAAAAAACAAGAGCAGTTAGAGCGAGAAAACAAAGCGTTAGAAGAG
TCTAAACAAGATTTGGAAGAGCAAGTCAAGGACCTAATGTTTTATCTAGATTCTCAAGAAAAATTCAAAGACGCT
GATGAGAGTGTAAAAGAAGGTACCATCCTAATACAGCAACCACATGGGGCAGCACAGGCATCCAAAAGCAAGAAG
AAGCGCAACAAAAATAAAAAAGCAGGGAAATGA

>YPL098C 0.8Cold
ATGCCTCCTCTTCCACAAAATTATGCGCAACAGCAGCCTTCGAATTGGGACAAATTCAAAATGGGGTTGATGATG
GGTACTACCGTCGGTGTCTGCACAGGCATCCTATTTGGTGGATTTGCCATCGCAACTCAAGGCCCAGGTCCTGAT
GGTGTAGTTAGAACACTAGGGAAATACATTGCTGGTTCAGCGGGTACCTTTGGGCTATTTATGTCCATCGGGTCT
ATAATCAGAAGTGATAGTGAAAGTAGTCCAATGTCCCATCCTAACCTGAACCTACAGCAACAGGCAAGACTGGAA
ATGTGGAAGCTTCGTGCCAAATACGGTATACGTAAGGACTGA

>YKL173W 0.79 Cold
ATGGAAGGTGACGATTTATTCGATGAGTTTGGAAATTTGATCGGAGTTGATCCTTTTGACTCGGATGAAGAAGAA
AGTGTGCTGGATGAGCAAGAACAATATCAAACCAATACGTTTGAGGGGAGCGGCAACAACAATGAGATTGAGAGC
AGACAACTTACCTCGTTAGGAAGTAAAAAGGAACTTGGAATTTCCTTAGAGCACCCTTATGGTAAGGAAGTGGAA
GTATTAATGGAAACTAAGAACACACAGTCACCACAGACTCCACTGGTAGAGCCGGTTACAGAACGAACAAAGTTG
CAAGAGCATACAATTTTTACACAATTAAAAAAAAACATTCCGAAGACTAGATATAATCGAGACTATATGTTATCA
ATGGCTAACATTCCGGAGAGGATAATAAATGTTGGTGTCATTGGACCTCTTCACTCAGGTAAGACCTCTTTGATG
GATCTCCTAGTAATCGACTCACACAAACGCATACCAGATATGTCTAAGAATGTAGAACTTGGATGGAAACCGCTA
AGATACCTGGATAATTTGAAACAAGAGATTGACCGGGGTCTTTCCATTAAACTTAATGGTTCCACTCTACTCTGC
ACCGATTTAGAATCCAAGTCTAGAATGATAAACTTTTTGGATGCACCAGGGCATGTTAATTTTATGGATGAAACG
GCAGTCGCACTTGCAGCAAGTGATTTGGTTTTAATCGTGATAGATGTTGTAGAAGGTGTTACATTTGTGGTAGAG
CAATTGATAAAACAAAGTATTAAAAATAATGTAGCAATGTGTTTTGTTATAAATAAACTCGACAGATTGATTCTA
GATTTAAAGTTGCCACCAATGGACGCATACTTGAAGTTAAATCATATAATTGCAAATATCAATTCCTTTACGAAG
GGAAACGTGTTTTCACCAATAGACAATAACATAATATTTGCGTCTACAAAGCTGGGCTTCACATTCACTATCAAG
GAATTTGTTTCTTATTATTACGCCCACTCCATACCTTCTTCTAAGATAGACGACTTTACCACAAGGCTATGGGGT
AGCGTATATTACCATAAAGGTAATTTCAGGACAAAACCATTTGAAAATGTAGAGAAGTACCCGACTTTCGTTGAA
TTTATCCTCATCCCGCTTTATAAAATATTTTCCTACGCTTTATCTATGGAGAAAGATAAATTAAAGAATTTGTTA
AGGTCTAACTTTAGAGTTAATTTAAGTCAAGAGGCCCTCCAGTACGATCCGCAACCTTTTTTAAAACACGTTCTA
CAGTTAATATTTAGGCAACAAACCGGTCTTGTAGATGCGATAACCAGATGTTATCAACCATTTGAATTATTCGAC
AATAAGACAGCTCATCTTTCAATTCCAGGAAAATCTACACCAGAAGGCACTCTTTGGGCTCATGTACTGAAAACT
GTGGATTACGGTGGAGCAGAGTGGTCCCTTGTACGGATATACTCTGGACTTTTGAAGAGAGGGGATACAGTACGT
ATACTTGATACATCACAATCTGAATCTCGCCAAAAAAGACAGTTGCATGATATCTCAAAGACGGAAACCTCCAAC
GAAGATGAAGATGAAGATGACGAAACGCCTTCATGTGAGGTCGAAGAAATTGGCTTATTAGGTGGCAGATATGTC
TATCCGGTTCATGAAGCACACAAAGGACAGATAGTATTAATAAAAGGTATTTCAAGTGCATATATTAAATCCGCG
ACGTTGTACTCTGTAAAGAGTAAGGAGGACATGAAACAGCTAAAATTTTTCAAACCTTTAGATTATATTACCGAG
GCAGTTTTCAAAATTGTTCTTCAACCTTTACTGCCAAGGGAATTACCCAAGTTATTGGATGCCCTGAACAAAATT
TCGAAATACTACCCAGGCGTTATTATTAAAGTTGAGGAATCTGGAGAACATGTAATACTAGGAAATGGAGAGCTG

TATATGGACTGTTTGTTGTATGATCTAAGAGCGAGTTATGCAAAGATAGAAATTAAAATATCTGATCCTTTAACT
GTTTTTTCTGAAAGTTGCTCAAATGAATCGTTCGCTTCAATACCTGTAAGTAATTCGATATCTCGCCTTGGTGAG
GAAAATTTACCAGGTCTGTCGATTAGTGTAGCCGCTGAACCAATGGACTCTAAAATGATTCAGGATTTGAGCAGG
AACACATTGGGAAAGGGTCAAAACTGCTTGGATATTGACGGAATAATGGACAACCCAAGGAAACTATCCAAAATA
TTGAGAACCGAGTATGGGTGGGATTCGTTGGCATCAAGAAACGTTTGGTCTTTTTATAATGGCAACGTGTTGATT
AATGACACTTTGCCAGATGAAATCAGTCCTGAATTATTATCCAAATATAAGGAACAAATAATACAAGGATTTTAC
TGGGCTGTAAAAGAGGGGCCTTTGGCAGAAGAACCAATTTATGGTGTACAGTATAAATTGTTATCGATCTCAGTA
CCTTCCGACGTAAACATTGACGTTATGAAAAGTCAAATTATTCCGCTAATGAAAAAAGCCTGTTACGTTGGCTTA
CTGACAGCAATCCCAATTTTATTGGAACCCATCTATGAAGTTGACATCACGGTCCACGCCCCCTTGCTGCCAATA
GTAGAGGAACTTATGAAGAAGAGACGTGGAAGCAGGATATACAAAACAATAAAAGTGGCAGGGACACCATTGTTG
GAGGTTCGTGGACAAGTTCCGGTTATTGAATCTGCAGGATTCGAGACAGATTTGAGATTATCTACGAATGGTCTT
GGTATGTGTCAGCTGTACTTTTGGCACAAGATATGGAGGAAGGTTCCTGGTGATGTTTTGGATAAAGATGCGTTT
ATTCCAAAATTGAAACCCGCACCTATCAACAGTTTAAGTCGTGATTTCGTGATGAAAACAAGAAGGCGGAAGGGT
ATTTCTACAGGTGGATTTATGTCAAATGATGGTCCTACGCTTGAAAAGTATATAAGCGCTGAATTATACGCTCAA
TTAAGAGAAAATGGCTTAGTACCGTGA

>YNL192W 0.75 Cold
ATGAGTGATCAAAATAATCGATCGAGAAATGAATATCACTCAAACCGGAAGAATGAACCTTCCTATGAACTCCAA
AATGCACATAGCGGGCTATTTCACTCTTCTAATGAAGAATTAACAAACAGGAACCAAAGATATACCAATCAAAAT
GCCAGCATGGGTTCATTCACTCCAGTCCAATCTTTGCAATTTCCAGAACAATCTCAGCAAACAAATATGCTTTAT
AACGGTGACGATGGCAATAATAATACTATCAATGATAACGAACGAGACATATATGGAGGTTTTGTCAACCACCAT
CGCCAGCGTCCCCCACCAGCAACTGCAGAATACAATGACGTTTTTAATACGAATAGTCAACAGCTACCGTCGGAA
CATCAATACAATAACGTACCTTCATATCCACTTCCTTCGATAAATGTGATTCAAACCACTCCAGAACTCATACAT
AACGGCTCACAGACTATGGCCACCCCCATCGAAAGGCCCTTCTTTAACGAAAACGACTACTATTATAATAACAGG
AACTCTAGGACGTCACCGAGTATTGCTTCTAGTAGCGATGGTTATGCAGATCAGGAAGCTAGGCCCATTTTGGAG
CAACCCAACAATAACATGAATAGCGGTAATATTCCTCAATACCATGACCAACCTTTTGGATACAACAATGGTTAC
CATGGCCTACAGGCAAAAGATTACTATGACGATCCGGAGGGTGGTTATATTGATCAGAGAGGAGATGACTATCAG
ATTAATTCATATTTGGGTAGAAACGGTGAAATGGTTGATCCTTACGATTATGAAAACAGTTTAAGACATATGACT
CCTATGGAGCGTAGAGAATATCTTCATGATGATAGCAGACCCGTAAACGATGGAAAAGAAGAATTAGACAGTGTG
AAAAGCGGTTACTCTCATAGAGACTTGGGGGAATATGACAAGGATGATTTTTCAAGGGATGACGAGTACGATGAT
CTCAACACTATTGATAAATTACAGTTTCAAGCTAATGGTGTACCTGCATCATCCTCGGTGTCTTCTATCGGATCT
AAAGAATCCGACATAATAGTAAGCAATGATAACTTAACCGCAAATAGAGCACTAAAGAGAAGCGGTACTGAAATT
AGGAAATTCAAACTTTGGAATGGTAATTTTGTTTTCGATTCTCCAATCAGTAAGACGCTATTGGACCAATACGCT
ACTACAACAGAAAATGCAAACACTTTACCAAATGAGTTTAAGTTTATGAGATATCAAGCAGTTACTTGCGAACCT
AATCAACTTGCAGAGAAGAATTTCACGGTGAGGCAGTTGAAGTATTTAACTCCAAGGGAAACGGAATTGATGCTA
GTAGTCACAATGTATAATGAAGACCATATCCTGTTAGGAAGAACTTTGAAAGGTATTATGGACAATGTCAAATAT
ATGGTGAAAAAAAAAAATTCAAGCACTTGGGGGCCGGATGCATGGAAAAAGATTGTCGTTTGTATCATTTCAGAT
GGTAGATCCAAAATTAATGAACGCTCGCTAGCATTACTAAGTTCGTTAGGTTGTTACCAGGACGGGTTTGCTAAG
GATGAAATTAATGAAAAAAAAGTGGCAATGCATGTCTACGAACATACGACAATGATCAACATCACAAATATTTCG
GAATCAGAGGTTTCATTAGAATGCAATCAAGGTACCGTTCCAATACAACTTTTGTTTTGTTTGAAAGAGCAAAAT
CAGAAAAAAATTAACTCACATAGATGGGCATTTGAAGGCTTTGCAGAATTACTGCGTCCCAATATCGTTACATTG
TTAGATGCTGGTACCATGCCAGGTAAAGATTCTATTTACCAGTTATGGAGAGAGTTCAGGAATCCAAATGTTGGT
GGCGCATGTGGTGAAATAAGAACTGATTTGGGTAAGAGATTTGTAAAGCTTTTGAATCCTTTAGTTGCATCACAG
AATTTCGAATACAAAATGTCCAATATTTTAGACAAAACAACCGAGTCTAACTTTGGATTTATTACTGTTCTACCG
GGGGCATTCTCTGCGTATAGGTTTGAAGCTGTGAGAGGCCAACCATTACAGAAGTACTTTTATGGTGAAATTATG
GAAAATGAAGGTTTTCATTTTTTTTCTTCCAATATGTATCTTGCTGAAGATCGTATTTTATGCTTTGAAGTGGTC
ACAAAAAAAAATTGTAATTGGATTTTGAAATACTGCAGAAGTTCTTATGCTTCAACAGATGTACCGGAGAGGGTC
CCTGAATTTATTCTTCAGAGGAGGCGTTGGTTGAATGGTTCATTTTTTGCTAGTGTATATTCCTTTTGTCATTTT
TACAGAGTCTGGAGCAGTGGTCATAATATTGGTAGAAAACTCCTTTTGACGGTTGAATTTTTTTACCTTTTCTTC
AATACATTGATTTCATGGTTTTCATTGAGTTCATTTTTCCTATTCTTTAGAATTCTCACTGTTTCTATTGCACTG
GCATACCATTCAGCATTTAATGTGTTGTCCGTCATATTCCTGTGGCTTTATGGGATTTGTACCTTATCAACATTC
ATACTGTCATTGGGTAATAAACCTAAAAGTACTGAGAAATTTTATGTTCTAACTTGCGTCATTTTTGCGGTGATG
ATGATTTACATGATATTCTGCAGTATATTCATGAGTGTCAAATCCTTCCAAAATATATTGAAAAACGATACCATC
AGCTTTGAGGGTTTGATTACCACAGAAGCTTTCAGGGATATTGTTATCTCTCTGGGCTCCACTTATTGTTTGTAC
CTAATCAGTTCAATTATCTATTTGCAGCCATGGCATATGTTGACAAGTTTTATTCAGTATATTTTATTGAGTCCT
TCTTACATCAATGTTTTGAATATCTATGCATTTTGTAATGTCCACGACTTATCATGGGGTACAAAGGGTGCAATG
GCAAATCCGCTGGGTAAGATTAATACTACAGAAGATGGTACGTTCAAAATGGAAGTTCTGGTCTCTAGTTCAGAG
ATTCAAGCAAACTACGATAAATATTTGAAAGTTTTAAATGACTTCGATCCAAAATCAGAATCTCGGCCTACTGAG
CCATCTTATGATGAAAAAAAGACTGGCTATTATGCAAACGTTAGATCTCTCGTGATTATCTTTTGGGTCATCACA
AATTTCATCATCGTTGCTGTTGTCTTAGAAACCGGTGGGATTGCAGATTATATTGCTATGAAATCCATATCAACT
GATGACACTTTAGAAACTGCAAAGAAGGCGGAAATTCCCTTAATGACCAGTAAGGCCTCAATTTATTTTAATGTA
ATTTTATGGTTAGTTGCATTATCGGCATTAATAAGGTTCATTGGTTGCTCAATATACATGATAGTAAGGTTTTTT
AAAAAGGTTACATTTCGCTAA

>YLR133W 0.68 Cold
ATGGTACAAGAATCACGTCCAGGGAGTGTAAGAAGTTACTCGGTCGGTTACCAAGCAAGGTCCAGATCGAGTTCT
CAAAGAAGACATTCGTTAACACGCCAACGTTCCTCGCAAAGACTGATTAGAACCATCAGTATCGAGTCTGATGTG

TCTAATATTACTGACGATGACGATTTGAGAGCTGTCAATGAGGGAGTAGCGGGTGTGCAACTGGACGTCTCTGAA
ACCGCAAATAAGGGACCAAGAAGAGCATCAGCAACTGATGTCACAGATAGTTTGGGTTCGACTTCGTCGGAATAT
ATTGAGATTCCCTTTGTTAAGGAAACATTGGATGCAAGTTTACCTTCGGATTATCTGAAGCAGGACATATTAAAT
CTCATTCAGAGTTTGAAGATATCCAAATGGTATAACAACAAGAAAATCCAACCGGTAGCACAAGATATGAACTTA
GTCAAGATCTCTGGTGCGATGACAAACGCAATTTTCAAAGTTGAATACCCTAAGTTACCATCGTTGCTATTGAGA
ATATACGGACCGAATATTGATAATATCATTGACAGGGAATATGAATTGCAGATTTTGGCTAGGCTTTCATTGAAA
AATATAGGTCCTTCCCTTTACGGCTGTTTTGTAAACGGTAGATTTGAGCAGTTTCTGGAGAATTCTAAGACTTTA
ACAAAAGACGACATTAGAAACTGGAAGAACTCTCAAAGGATTGCAAGGAGAATGAAGGAGTTACATGTAGGTGTT
CCTCTCTTGAGTTCAGAAAGGAAGAACGGGTCGGCTTGTTGGCAAAAGATTAACCAGTGGTTGCGCACGATTGAG
AAAGTCGACCAATGGGTGGGGGATCCTAAAAACATTGAAAACTCTTTATTATGTGAGAATTGGTCCAAGTTTATG
GATATTGTCGATAGATATCACAAGTGGCTTATTTCTCAAGAACAGGGTATAGAGCAAGTCAACAAAAATCTTATA
TTCTGCCATAATGATGCCCAATACGGCAATTTACTTTTCACTGCTCCTGTGATGAACACACCGAGCCTATACACT
GCACCTTCGTCTACATCATTGACTTCCCAATCAAGTTCCTTATTTCCTTCGAGCTCCAATGTCATTGTAGATGAT
ATAATCAACCCGCCAAAGCAGGAGCAAAGCCAAGATTCCAAATTGGTCGTCATTGATTTTGAATATGCAGGTGCC
AATCCCGCCGCATATGATTTAGCGAATCATCTTTCCGAGTGGATGTATGATTACAACAATGCTAAGGCCCCACAT
CAGTGCCACGCTGATAGATATCCCGATAAAGAACAGGTTTTGAATTTCTTATACTCTTATGTTTCGCATCTAAGG
GGTGGTGCTAAGGAACCCATAGATGAAGAGGTTCAAAGACTCTATAAGTCAATCATTCAATGGAGACCCACTGTA
CAACTATTTTGGTCGCTCTGGGCCATCCTACAAAGTGGTAAATTAGAGAAAAAAGAAGCCTCCACTGCCATCACT
AGAGAAGAAATTGGACCCAATGGAAAAAAATATATCATCAAGACTGAACCCGAATCCCCTGAAGAAGACTTTGTT
GAAAATGACGACGAGCCTGAAGCTGGCGTCAGCATTGACACGTTCGATTATATGGCTTATGGTCGTGACAAGATT
GCGGTCTTTTGGGGCGACCTCATTGGCTTAGGCATAATCACCGAAGAAGAATGCAAAAATTTCAGCTCTTTCAAG
TTCCTCGATACTAGTTATTTGTAA

>YDR330W 0.82 Cold
ATGTCAGAGGGAAAAGTAGACGAATTTATGGCCATCACTGGCGCTGATGACGCTGCTATTGCCACTCAATTCATC
GAAATGGCAGACGGGAACTTGAACACAGCCATATCATTGTTTTTCGAAAATGGTGGGGCTGCTTTACTGAGCAGT
AACAACACACCTACTCCGTCTAATTCGACTCCTATGGCGCCCACCTCTGTAGATTCAGATGCCGATGCACAATTA
GCGGAGAGGTTGCAAAGGGAAGCTTACCAACAACAACAGCCTGACCAAGATTATGTCAGGCCACCTGATGAGGCA
AGACATGAAGTACTTACTGAAACTTCAGGATTCCCAATTTCTTACGGCGGCATTGGCGGAAGATTTGAACCATTG
CATAGGGTGAACGACATGTTTGACGAAGGAAGACCTGAAAGCATTTTTAATCAACGATTGGATGACACAAATACT
AATACATACATCAATGACAACTCTTCAGATTCCTTGGACTCCGAGGAAGAAAATGACGACGACGAATATGAGTAT
GTGGAAGAGCCGGTCATCGAACTTGATGAAGACGGCAATATCAAGGAGTACACCAAATTAGTGAGAAAGCCTAAA
ACCATTTCCAAGGAGCAGAAATTAGCTTTGTTATTTAGGCCTCCATTTTCAATCATGTCCAAACTCGACTTAGAC
GCTGCAAAGCAAAAGGCAAGGGCAAAGCAAAAATGGATAATGATTAACATTCAGGATTCAGGAATCTTCCAATGC
CAAGCCCTCAACAGGGATTTGTGGTCGTCCAGACCGGTAAAAACTATAATCAAAGAGAACTTCGTTTTCCTACAG
TATCAATATGAATCACGTAATGCCCAACCCTATCTTCAGTTCTATCACTTGAATAATAAGGATGATTTGCCCCAT
ATCGCGATACTGGATCCAATTACCGGTGAACGTGTCAAGCAATGGAATAGGGTTGTTCCCATTCCAGAGCAATTC
ATCTCAGAAATCAACGAGTTTTTGGCCTCATTTTCGCTAGATCCAAAGGTCCCTAATCCCACGGTTAATGAACCT
CTTCCTAAAGTGGATCCAACAACTTTGACCGAAGAGCAACAAATGGAATTAGCCATTAAAGAGTCATTAAATAAT
AACTCGAGCAAATCAAACCAAGAAGAAGTGCCGAGTACTGGCGAAGAACAGAAACGAGTACAAGAACCGGATCCA
TTCAGTACCATTGAAGCCAGGGTGCATCCAGAACCGCCAAATAAACCAGGGATCACTACAAGAATTCAAATTCGT
ACCGGTGATGGCTCCAGACTAGTTCGTAGATTCAATGCTCTGGAAGACACTGTGCGTACTATTTACGAGGTTATC
AAGACAGAAATGGATGGTTTTGCCGATTCAAGATTCACACTAAATGATCACCAAAGAGAGGACTTGATTGATAAA
TTAAACATGACCATAGCGGACGCCGGCTTAAAAAATAGTTCTTTACTACTTGAGAAGCTTGACCCTGAAATAGAA
TAA

>YKL168C 0.76 Cold
ATGGTAATGCAGGAGGAGAAGAAGCGGCAACAACCTGTGACAAGAAGAGTAAGGTCATTTTCTGAGTCCTTTAAA
AACCTTTTCAGGCCGCCTCGTTCAAGAGATTCAAGTCCTATTAATGTCACCCGAATTCCTTATCGATCGTCTTCT
ACCTCACCCAAGAGAAGCTCTGAGCCACCAAGACGATCAACCGTATCCGCACAAATTTTAGATCCAAAAAATTCT
CCAATACGCCAGCGTAGTTATACATTGAAGTGTTGCACCCCGGGCCTGTCACATCCATTTAGACAGACAGGAAGT
GGTGCTAGTAATAGTCCTACCAGACATCGCTCTATATCTGGGGAAGAACAGGAAATTGTTAACAGTTTACCAGAA
TACAAAAGATCTGCTTCTCATACTTTTCATGGCATACGGCGTCCACGTTCAAGAAGTTCATCTGTTTCGTCATGT
GACAGTTCAAATGGAACGACAAGTTCATCAGACAGTCAATGGGCTATGGATTCTTTACTAGATGATTCAGACAAT
GACCTCACACCTTATCGTGGCTCGAACAAAGATATACTGAAAAGTAAAGACCGGGCGCCGTATAATTACATAGAT
GACTACAATAAGAAGGCTTTACGCAGAGCAACATCATATCCAAATCCTCTCCCCTCCAAGCAATTCTATAATGAA
AGGTTATACACCAGGAGGTCACATCCAGATGAAGAAAGTTTGGAGAGCTTGCCTCGATTTGCAGGAGCAGATGTG
CAATGTATAATTGAGCAAAATGGCTTCAAAGTTTACGAAGACGGAAGTCATGAACATAATATAAAGTTATCAGGG
GTAATTGCGAAATTAGAGAAAGGTAACTCCTTACCGGCACATCGACAGGGATCATTATCTAGACCAAGATTAGGT
ATTACTCTTTCTGGTCTATTCAAACATCATAAAAATGAATGCGATATTGAAAACGCTTTATCTCTTCTGCCAAAC
GTAGAAAAAATCTCAGACAAACCATGAGAAAAGAACCGGTCAAAGTCCTAACGATAGCAACCGCAGCTCGCCCACG
CAAGGAAGGGAAGATTATCTAAAAATAGTGAATCCCGACGCATCATTGGGCAGTGATGAGTTGAAGTTGATTAAT
TCATTATCAAGCAGAATTCATAAAAGTTTACAAAATTATTTGCAAGAAAAAAATCTAAAACCGGCTGAGTGTATC
GGCGAGCAAGCACCTACTTTTCAAGATAATTATGGCCATCCTGTTGGTCTCGTTGGCGCTGGAGCTTACGGTGAA
GTTAAGCTGTGTGCCAGACTACGGAATGAAAAGGACTCCCCCCCATTTGAAACATATCATGATAGCAAATACATA
TACTATGCTGTGAAAGAATTGAAACCCAAACCAGACAGTGATTTAGAGAAGTTCTGTACAAAGATCACCTCTGAG
TTTATTATTGGGCATTCCTTGAGCCACTACCACAAAAATGGTAAAAAACCAGCCCCCAATATTCTAAACGTATTT

GATATTCTAGAAGACAGTTCTTCGTTTATTGAAGTAATGGAATTTTGCCCTGCTGGTGACCTTTATGGTATGCTG
GTCGGTAAGTCGAAATTAAAGGGCCGATTACATCCGTTAGAAGCCGATTGTTTCATGAAGCAACTGCTTCATGGT
GTTAAGTTCATGCATGATCATGGTATCGCTCATTGTGATTTAAAACCTGAAAATATTCTCTTTTATCCGCATGGT
CTATTGAAAATATGTGACTTCGGTACAAGTTCTGTATTTCAAACCGCTTGGGAAAGGAGGGTTCATGCTCAAAAG
GGTATAATTGGATCAGAGCCTTACGTAGCTCCAGAAGAGTTTGTTGATGGGGAGTATTACGATCCAAGACTGATT
GACTGTTGGAGTTGTGGCGTTGTTTATATAACAATGATATTGGGGCATTATCTTTGGAAAGTAGCTTCGAGAGAA
AAAGATATGTCTTATGATGAATTTTACAAGGAAATGCAGCGTAAAAACCAGTTTAGGGTATTCGAAGAATTAAAG
CACGTAAACTCAGAGTTGGCCACAAATAGAAAAATCGCATTATATCGCATCTTTCAATGGGAACCAAGAAAAAGG
ATTTCTGTTGGCAAACTTTTAGATATGCAGTGGATGAAAAGTACGAATTGCTGCCTAATATATGATTCCACATAA

>YLR004C 0.79   Cold
ATGAAAAACATGTCACAACGATCCATGGACGTGGAGAAGAAGGCAGCTAACGCTGATAGTTGTTCCGTAAGTACC
TCGAGCATTAATGTAGACGATGCGGACGTTGCTTTAAGATTCCTGAAACAGAACGGTCTCGACGAAAGCTCCACG
GCTAATGAAGACGATGTTGTAGCAGGCGAAGAAGCCAACTTCTATGGTTCCCATGAATTATCTCCTAAGGTCCTG
AGAAAAGTTGACCTCTTCATCCTGCCCTTTTTATGCTGTACATATCTTCTGATGTTTTTGGATAAGGCTCTGCTG
AACTACGCTGCATCTATGGGTATCAAAGATCATTTGAAAGGTAACGAATTTTCTAATTTGGGTACTATCTTTTCT
GCTGCTTACATTTTCATGGAACCTGTGGTCACTTACTTAATTCAAAAATTCCCCATTTCCAAAATCCTAGGTACT
TTTATTACCGTCTGGGGTATCGTGTTGGCTTGCCATGCTGCTTGTAAGACGTACGCCTCGTTGATGGTCGTTCGT
ACTCTCCTAGGTCTCTTTGAATCCTCGAGTGCAGTTGGTTGTATCGCTATTAGTGGTATGTACTATACTAAATCT
GAACAGAGTGCAAGAATTGGATTCTGGGCCACACAGGCTGGTACAGGTTATATTGTGGGCGGGTTAATTTCTTTT
GGCTTTTTACATTATCATGGTACCGCTTTCACCTCTTGGCAAATAATGTTTTTGGTCGTTGGTCTGGTTACAGTT
GCGTTTGGTGTTCTAACATTCTTATACCTTCCAGATAACGTCACCAATGCTTGGTTCCTAAACAAAGAGGAAAAA
ATTCAAGTTGTTGAGCATATCAGAGCTAACCAAACTGGCCTGGAAACAAAGAAGTTTAAGAAGCAGCAAGTTAAA
GAACTATTCCTTCATGATAAGTTTACTTGGCCGATGCTTTTATTAACTGCATGTTCTCAAATTTCTACTGGTGCC
ATTGGTACATTTTCAGTGACCATCACTGGTACTTTTGGTTTCGACAAGTACGAAACAGCATTACTGCAGTTGCCT
ATTGGTGCCATTACGGCTATGATTATTCTTATAACAACTCAAATGTTGTCCCGCTGGGGTCATATCACACTCATA
ACCACATCTATGTATATTCCAGCTATTATAGGTTGTATCGTTCTTATCAGTTTACCCTTATCCCATAAGATCGGT
AACTTATTTTCTCTGTACTTGCTTTACAGTGGTTCCTGTGTCATTACCAATATTTACATCTGGAATTCATGCAAC
ACCTCCGGTTACACCAAAAGAGTTTTTAGAAATGCTATTACAATGATTGTATACAATGTCTCCTGTATTATTGCT
CCACAGATGTTTAGGGCATACTCTGCTCCACGTTACATACCAGCAAAGATTGCCTTATTGGTCACTCAGTGTGTT
TGTGTTCCCCTACAATTATACATTGGCTACATTTGCAAGAAGGAGAACGAAAAGCGTGATAAGGAACAAGAAGGT
CAAGAAAGGAAGAAGTACCAATTTTTGGACTTGACTGATATCGAAAACAGAAATTTCAGGTACATTTACTAA

>YKR009C 0.79   Cold
ATGCCTGGAAATTTATCCTTCAAAGATAGAGTTGTTGTAATCACGGGCGCTGGAGGGGGCTTAGGTAAGGTGTAT
GCACTAGCTTACGCAAGCAGAGGTGCAAAAGTGGTCGTCAATGATCTAGGTGGCACTTTGGGTGGTTCAGGACAT
AACTCCAAAGCTGCAGACTTAGTGGTGGATGAGATAAAAAAAGCCGGAGGTATAGCTGTGGCAAATTACGACTCT
GTTAATGAAAATGGAGAGAAAATAATTGAAACGGCTATAAAAGAATTCGGCAGGGTTGATGTACTAATTAACAAC
GCTGGAATATTAAGGGATGTTTCATTTGCAAAGATGACAGAACGTGAGTTTGCATCTGTGGTAGATGTTCATTTG
ACAGGTGGCTATAAGCTATCGCGTGCTGCTTGGCCTTATATGCGCTCTCAGAAATTTGGTAGAATCATTAACACC
GCTTCCCCTGCCGGTCTATTTGGAAATTTTGGTCAAGCTAATTATTCAGCAGCTAAAATGGGCTTAGTTGGTTTG
GCGGAAACCCTCGCGAAGGAGGGTGCCAAATACAACATTAATGTTAATTCAATTGCGCCATTGGCTAGATCACGT
ATGACAGAAAACGTGTTACCACCACATATCTTGAAACAGTTAGGACCGGAAAAAATTGTTCCCTTAGTACTCTAT
TTGACACACGAAAGTACGAAAGTGTCAAACTCCATTTTTGAACTCGCTGCTGGATTCTTTGGACAGCTCAGATGG
GAGAGGTCTTCTGGACAAATTTTCAATCCAGACCCCAAGACATATACTCCTGAAGCAATTTTAAATAAGTGGAAG
GAAATCACAGACTATAGGGACAAGCCATTTAACAAAACTCAGCATCCATATCAACTCTCGGATTATAATGATTTA
ATCACCAAAGCAAAAAAATTACCTCCCAATGAACAAGGCTCAGTGAAAATCAAGTCGCTTTGCAACAAAGTCGTA
GTAGTTACGGGTGCAGGAGGTGGTCTTGGGAAGTCTCATGCAATCTGGTTTGCACGGTACGGTGCGAAGGTAGTT
GTAAATGACATCAAGGATCCTTTTTCAGTTGTTGAAGAAATAAATAAACTATATGGTGAAGGCACAGCCATTCCA
GATTCCCATGATGTGGTCACCGAAGCTCCTCTCATTATCCAAACTGCAATAAGTAAGTTTCAGAGAGTAGACATC
TTGGTCAATAACGCTGGTATTTTGCGTGACAAATCTTTTTTAAAAATGAAAGATGAGGAATGGTTTGCTGTCCTG
AAAGTCCACCTTTTTTCCACATTTTCATTGTCAAAAGCAGTATGGCCAATATTTACCAAACAAAAGTCTGGATTT
ATTATCAATACTACTTCTACCTCAGGAATTTATGGTAATTTTGGACAGGCCAATTATGCCGCTGCAAAAGCCGCC
ATTTTAGGATTCAGTAAAACTATTGCACTGGAAGGTGCCAAGAGAGGAATTATTGTTAATGTTATCGCTCCTCAT
GCAGAAACGGCTATGACAAAGACTATATTCTCGGAGAAGGAATTATCAAACCACTTTGATGCATCTCAAGTCTCC
CCACTTGTTGTTTTGTTGGCATCTGAAGAACTACAAAAGTATTCTGGAAGAAGGGTTATTGGCCAATTATTCGAA
GTTGGCGGTGGTTGGTGTGGGCAAACCAGATGGCAAAGAAGTTCCGGTTATGTTTCTATTAAAGAGACTATTGAA
CCGGAAGAAATTAAAGAAAATTGGAACCACATCACTGATTTCAGTCGCAACACTATCAACCCGAGCTCCACAGAG
GAGTCTTCTATGGCAACCTTGCAAGCCGTGCAAAAAGCGCACTCTTCAAAGGAGTTGGATGATGGATTATTCAAG
TACACTACCAAGGATTGTATCTTGTACAATTTAGGACTTGGATGCACAAGCAAAGAGCTTAAGTACACCTACGAG
AATGATCCAGACTTCCAAGTTTTGCCCACGTTCGCCGTCATTCCATTTATGCAAGCTACTGCCACACTAGCTATG
GACAATTTAGTCGATAACTTCAATTATGCAATGTTACTGCATGGAGAACAATATTTTAAGCTCTGCACGCCGACA
ATGCCAAGTAATGGAACTCTAAAGACACTTGCTAAACCTTTACAAGTACTTGACAAGAATGGTAAAGCCGCTTTA
GTTGTTGGTGGCTTCGAAACTTATGACATTAAAACTAAGAAACTCATAGCTTATAACGAAGGATCGTTCTTCATC
AGGGGCGCACATGTACCTCCAGAAAAGGAAGTGAGGGATGGGAAAAGAGCCAAGTTTGCTGTCCAAAATTTTGAA
GTGCCACATGGAAAGGTACCAGATTTTGAGGCGGAGATTTCTACGAATAAAGATCAAGCCGCATTGTACAGGTTA
TCTGGCGATTTCAATCCTTTACATATCGATCCCACGCTAGCCAAAGCAGTTAAATTTCCTACGCCAATTCTGCAT

GGGCTTTGTACATTAGGTATTAGTGCGAAAGCATTGTTTGAACATTATGGTCCATATGAGGAGTTGAAAGTGAGA
TTTACCAATGTTGTTTTCCCAGGTGATACTCTAAAGGTTAAAGCTTGGAAGCAAGGCTCGGTTGTCGTTTTTCAA
ACAATTGATACGACCAGAAACGTCATTGTATTGGATAACGCCGCTGTAAAACTATCGCAGGCAAAATCTAAACTA
TAA

>YER006W 0.79 Cold
ATGAGAGTCAGAAAGCGCCAATCTAGAAGAACATCTACCAAGTTGAAGGAAGGTATTAAGAAGAAGGCTTCTGCC
CATAGAAAAAAGGAAAAGAAGATGGCTAAGAAGGACGTTACTTGGAGATCAAGGTCTAAGAAAGACCCTGGTATT
CCTTCTAACTTTCCTTATAAGGCTAAGATCTTAGAAGAAATAGAAGCCAAAAAAATGAAGGACTTAGAGGAAAGA
GAGCTTGCCAAACAACAACGATTGGAGGCTAGGAAAGCTGCCAAGGAACAAGGCGTTGATGCAATGGATGAAGAC
ATGATAGAAGACGATGAAAATGGATTAGCAGCTTTGGTTGAGTCTGCTCAGCAAGCAGCTGCTGAATATGAGGGC
ACACCCTCTAATGATGCAGATGTTCGTGATGATGAATTGGACGTCATTGATTATAACATTGATTTCTACGGTGAA
GATGTAGAAGGCGAGTCAGAACTTGAAAAATCTAGGAAAGCTTATGACAAGATTTTCAAATCCGTTATTGATGCT
TCTGATGTCATCCTATATGTTTTGGATGCCAGAGATCCAGAGAGTACAAGATCAAGAAAGGTGGAAGAAGCCGTC
TTACAAAGTCAGGGTAAAAGGCTGATTTTAATACTGAACAAAGTTGATTTAATTCCCCCACATGTATTGGAACAA
TGGTTAAATTACTTAAAGTCTAGTTTTCCTACAATCCCATTAAGAGCTTCTTCTGGTGCAGTCAACGGAACTTCT
TTCAACAGAAAGCTAAGCCAAACCACTACTGCAAGTGCACTGCTAGAGTCGCTGAAAACTTACTCTAATAACAGC
AATTTGAAGAGATCTATTGTAGTGGGTGTTATCGGTTATCCAAATGTTGGTAAATCATCTGTTATCAACGCTCTT
TTGGCGCGCCGTGGAGGTCAATCAAAGGCTTGCCCAGTCGGTAATGAAGCAGGTGTTACTACCTCTCTGAGAGAG
ATCAAAATCGACAACAAGTTGAAGATTCTAGATTCTCCTGGTATATGTTTCCCAAGCGAAAATAAGAAAAGATCG
AAAGTTGAACATGAAGCTGAATTAGCTCTTCTAAATGCTCTACCGGCAAAACACATTGTTGATCCGTATCCAGCT
GTCTTAATGTTGGTAAAGAGACTAGCTAAATCAGATGAAATGACAGAGAGCTTTAAAAAGCTGTATGAAATTCCT
CCTATTCCTGCTAATGATGCAGATACGTTTACAAAACATTTCTTAATTCACGTCGCTCGTAAAAGAGGTAGATTA
GGGAAAGGCGGTATTCCAAATCTTGCCAGTGCTGGTCTTTCGGTACTTAATGACTGGAGGGATGGTAAAATTCTT
GGTTGGGTTTTACCAAATACATCGGCAGCTGCATCTCAACAAGATAAACAAAATCTAAGTACTATAAACACTGGC
ACTAAACAAGCTCCAATTGCAGCAAATGAATCCACCATTGTATCCGAGTGGTCCAAAGAATTTGATTTGGATGGA
TTATTTAGCTCTCTTGACAAGGCCATAGATGCTAGCAAAGATCAAGATACAATGATGGAATAA

>YIR003W 0.8 Cold
ATGCCATCGGAAGTTACGCCAAAAGTACCTGAGAGGCCCTCAAGGAGGAAGACATCTGAGCTGTTTCCGTTATCC
GGCTCTGAATCAGGAGATATTAAAGCTAATAGCGAGCCACCAACACCAGCAGGAACGCCCAATGTACCAACACGT
AGACCCATACTTAAAGCAAAGACGATGACTTCGTTTGAGAGTGGTATGGATCAAGAGAGTTTGCCCAAGGTACCA
TTGCAGAGGCCAGTTAGACGCTCCACTACTGAAGAACTAAATAATGTAATGAATAACACCAGTAAGGAGTTAGAG
GAGATTGAAAGTTTGATTTCAAAGCACAATATACACAATGTTTCCAGGAAAAAAAGTCCAACTTCTGTGGAAGAG
GGAAAGGTGGCCGCCATTCATCAAAACGGGCAAAGAAGTGCATCAGATAACAAAACTTCTACAAATCCCTCCCCA
CTCGAAAAAAATGAGCACGAGGGTGCTGAGGGCAATGAGTCTGCTATTTCACCTTCTAATTTAGTTAACAAGTCT
AATAATGAAGTGACAGAGCATAGTGACTCCGAAGACCTTACTGAAAAGCAAAAGGTACATGCTGCCCTAGATAAT
GAGGCAGGGGATAGAAGCCATTTTGAAGAAAAGCTCATTCCTGGAGATATGAAAGTACAGGTAGATGTTAGTAAG
GACGTAGAAGAAGGCAGCCTCAATGCTCTGCCTCCATCTGGAATCACAGAATCAGACGACAAAGCTGAAAAGTTC
ACTAAACATCCTGAATCTAGTTTAGAAGAGCTACAGAAGCATCAAGAACAACAGGAAGAAAAAATTTTCCAAAAT
CCAACCGATGAAGAAAGTACTACCTCTTTGAATGAGAAACAAGAAGGCAAGGACAACATGGAAGTGAATTCTCAG
CCGCAAGGACCTTCTGATACTGAAACAGTTATAGCAGCAACTAGCTCAAACGTTCCTTCACAAATTGCAAGTGAA
GAGGAAAATGACGTTCCAGTGATTCCAAGGAGTAGGCCTAAGAAGGATTTTGAAGCACACGTACAAAAAGAGGAG
TTGCCTAACACACAAGAGAAGCGAGTATCAGAAGAGTGTGATTCGACCCTAATTAGTACCGAAGAGGAAAGTAAA
ATCCCAAAAATTCCCTCTGAAAGACCAAAGAGACGTGCCCCACCTCCGGTTCCCAAAAAACCTTCTTCAAGAATT
GCGGCCTTTCAAGAAATGTTACAAAAACAACAGCAACAAGATCTGCATAATAATGGTAATTCTTCAGCTACTACC
GCTTCAGCAGATATTGCTAAAAAACACACAGATTCTTCTATTACTAGCGATACCCACGAAGGCAGATTTTACGAGC
AAATTGAATGGACTGTTTGCGCTACCTGGTATGGTGAACCCTGGACAGCTTCCACCTTCCTTGGAGAAGAAGTTG
TCCTCCCCGGATACTGAATCTAAGCTAGGACCCCAGGACCAATCACAGGCAAAAACGGGACCTCTGGGCGGAACT
AGAAGAGGTAGAGGACCTCGTGGGCGTAAATTACCTTCTAAAGTAGCTAGCGTAGAGAAGATTGAAGAAGATGAC
AACACTAATAAAATAGAAATATTTAACAATTGGAATGTTTCCTCTTCCTTCTCTAAGGAAAAAGTCTTAATAGAT
ACTACTCCTGGTGAGCAAGCAGAAAGGGCCTTGGATGAAAAGTCAAAGAGCATTCCCGAGGAACAACGAGAACAA
TCTCCAAATAAGATGGAGGCTGCTCTTTGTCCTTTTGAGCTGGACGAAAAGGAAAAGTTACCTGCTAATGCTGAG
TCGGATCCTCTTTCTCAGTTGCCACAGACGAACGCCGTAGGTAATAGAAAAGCCATAAGTGAGGAATCTCTGTCC
CCATCGGAGGCCATCGCCAACAGAGATCAAAACGATACGACAGAAATACAGGAACAGCAGATGGAGGACCAAATG
GAGGTTGATATGGAAAGAGAACTCTCAGGTGGCTACGAAGACGTTGATTCTGCCTTACATTCAGAAGAAGCGTCT
TTTCACTCCCTTTAG

>YKL033W 0.79 Cold
ATGAATTCAGACACGAATGCATTTAAGGACATAAGAATTTCATGTGTTGAACTTTCTAGGATCGCTTTTTTACCC
ACCGAATCATTTGATCCAAATTCATTAACGCTATTGGCATGTTTAAAAAAAGTTGAAGAGAAGTTATCAGCATAC
GAAGACGATTCACTTTCGCCAAAATTTGCAGATTATGTTTTTGTTCCTATCGCGAGTTTATTGAAACAGCCAGCT
CTCGGCGAGTCTCAAACAGAATACGTCCTACTAATTATATTTCATCTCTTACGGACATGTTGGTCATCTAATGGT
AAATTCTCCGAGCAATTGGGACAACAACTGTTCCCCTTAATCACATTTTTAGTCAGTTCTGATAAAGATAATCAA
AAATTAATTACTAGATCAGACGAGTTCAAGTACGCTGGTTGTTTAGTCTTGCATCAGTTTTTCAAATCAGTAAGA
TCGCAAAGGTACCATAAAGAGTTCTTTTCGAACTCAAAACCTAATTTACTTCCCGCTCTAGGACATTCTGTTACC
ATTCTGTTGAAAATTCTGGAGCAATCGCCCCAAAATAATGAGCTTCAATTCAAAGCTTTGGCATCCTTAGAAGTA

CTATTTCAGGACATAATATCTGATGGTGAAATGCTTTCCTTTATTCTTCCGGGGAACGTTTCTGTTTTCGCTAAG
ATACTTACTAAACCAGGCCGTCAAATACATTATAAGGTGTGTGTTCGCACGCTAGAGGTTCTGGCAAAATTATTG
GTTTTGGTTTACGATGATTTTAGTTTGGATATCAAAGTAAATAAGTTAACAGATATTCGAGAGTTGAGCGATACA
AAGTTAAAACACGAGATAAATCAATCATTTATGTTTAATGGACCTATTGTTTTGTTACGCACAGATGGGAAGACG
CATAGAGATACAAGTTGGTTGACGGCAACTTCTGGGCAAATCAATATTGCTTTGGAGGCCTTTATACCCAAACTA
TTAAAACGTAATAATGAATCAATTGATGAGGCATTAGCGACATTTGTATCAATCCTTTTAACTAGATGTGAAAAT
TCTTTAAATAATTGTGAGAAAGTGTTGGTATCCACTTTAGTACATTTGGAGCGTGATCCGATGTCCAAATTACCA
TCGCATTTGGTAAAATTAAAGGAAGTTGTCAATGAAGACCTGCACAAGCTAAGTGACATTATTCGGTTTGAAAAT
GCAGATAGGTTATCATCTTTATCATTTGCAATCACTATTCTAGAGAAAAATAACGAAAGAGATACAATGATCAAT
GAGGTAGTTAGATGTTTATTTGAATCGCTTAATGAATCTATTGAGCCACCAAGTTTGATCAATCATAAAGAGAGA
ATAATTGAACAAAGTAGTCAATTAACAACTACAGTAAATTTCGAAAACTTGGAAAGTACGAATGCTCTGATTGCT
TTACCAAGATTATCAGAAGACATGTCACTCAAACTGAAAAAATTTACATACCATATGGGTTCTTTGTTGTTAGAA
AGACATATTCTCAACGATGTGGTGACAGAGTTGATTTCAGAACAAGTTGATTCACCAAGAACGCAGAAGATAGTT
GCGCTGTGGTTAAGCACCAATTTTATAAAGGCTATGGAAAAGCAACCGAAGGAGGAAGAAGTCTATCTACAATTT
GAATCTGATGCCAACTATTCCTCTTCCATGGTTGAAGAAGTGTGTTTGATTGTATTGGAATTTTGCAATGAACTT
TCTCAAGACATTAGCATGGAGATTGAAGGTAAAGGCATAAAAAAAAGTGATGAGTTTGCCGTATGTACAGTTTTA
TTCTCTATTGAAACGATTTGTGCGGTAATGAGAGAAGAATTTCAACCAGAATTGATTGACTATATATACACAGTA
GTTGACGCCTTGGCATCACCGTCCGAAGCCATTAGGTATGTGAGCCAATCCTGTGCATTGAGGATAGCAGATACC
CTATATCATGGGTCTATCCCAAACATGATCTTAAGCAACGTAGATTATCTTGTAGAATCTATTTCCTCAAGATTG
AATTCTGGGATGACTGAACGAGTGAGCCAAATATTAATGGTGATTTGCCAATTGGCTGGCTATGAAACCATTGAA
AATTTTAAAGATGTTATTGAAACGATATTTAAACTACTGGATTATTATCATGGATATAGTGATCTCTGTCTTCAA
TTTTTCCAACTATTTAAAATCATCATTTTGGAGATGAAGAAAAAATATATCAATGATGATGAGATGATTTTGAAA
ATAGCTAATCAACACATTTCACAAAGTACTTTTTCGCCCTGGGGTATGACCGATTTTCAGCAAGTACTCAATATA
CTAGATAAAGAGACACAGGTTAAAGATGACATAACAGACGAGAACGACGTCGATTTTTTGAAGGATGATAATGAG
CCCAGTAATTTTCAAGAATATTTCGACTCCAAATTAAGAGAGCCAGATAGCGACGACGATGAAGAAGAAAGAGAA
GAAGAGGTGGAAGGAAGTTCTAAAGAGTATACTGACCAGTGGACCTCACCTATACCATCAGATTCATACAAAATA
CTATTGCAAATTTTGGGTTATGGGGAAAGATTATTAACCCATCCATCAAAACGATTAAGAGTGCAAATTCTTATT
GTTATGAGGCTTATATTTCCCTTGCTATCAACACAGCATAATCTGTTGATAAGGGAGGTTGCTAGTACATGGGAC
TCCATCATACAGTGCGTCCTATGCTCTGATTATTCGATCGTTCAGCCCGCATGTTCATGCGTGGAGCAGATGATA
AAATATTCTGGTGATTTTGTCGCCAAGAGATTCATTGAACTTTGGCAGAAACTGTGTCAGGATTCTTTTATATTG
AAAGAACTGAGGATTGATCCAACGGTGCACAACCATGAGAAAAAATCGATTAGTAAGCATGTCAAATTCCCACCA
GTAACTGAAAATGCGCTAGTATCGATGGTACATATGGTGCTAGAAGGGGTCAAAATTACAGAGTATTTAATTTCT
GAAGCGGTACTTGAACAAATAATATATTGTTGTATTCAGGTTGTTCCAGTGGAGAAAATATCATCTATGTCATTG
ATAGTGGGGGATATCGTGTGGAAAATAAGAAATATAAATTAA

>YKL134C 0.72   Cold
ATGCTTCGCACGATAATATTGAAAGCCGGGTCCAATGCCTCCATACCGTCACCTTCCCGCCAAAATAAGTTACTC
AGGTTCTTCGCCACAGCCGGCGCAGTCTCTAGGACCAGTCCAGGAAGCATTAAGAAGATTTTCGACGACAATTCA
TATTGGAGAAATATTAATGGTCAGGATGCTAATAATAGCAAGATCTCACAATATCTTTTTAAAAAAAATAAAACC
GGACTTTTCAAGAACCCTTATTTGACTTCTCCAGATGGTTTGCGTAAGTTTAGCCAGGTTTCTTTGCAGCAAGCA
CAAGAACTTCTCGACAAAATGAGGAATGATTTTAGCGAGAGTGGTAAATTAACCTATATTATGAACCTGGACAGA
TTAAGCGATACGCTATGTCGAGTTATTGATTTGTGCGAGTTTATTAGGTCAACACATCCAGATGATGCATTTGTT
AGGGCAGCACAAGATTGCCATGAACAAATGTTTGAATTCATGAATGTCTTGAACACTGATGTTTCCTTATGTAAC
ATACTAAAGTCGGTTTTAAACAATCCAGAAGTGTCTTCGAAGTTAAGCGCAGAAGAACTTAAAGTTGGTAAAATA
TTATTGGATGATTTTGAAAAGTCAGGAATCTATATGAATCCAGATGTTAGAGAAAAGTTTATCCAGTTATCTCAG
GAAATCAGTTTAGTAGGTCAAGAATTCATCAACCATACAGACTATCCTGGTTCAAATTCTGTGAAGATACCATGT
AAAGATCTGGATAATAGTAAAGTGAGTACATTTCTATTGAAGCAATTAAATAAAGATGTAAAAGGGCAAAATTAT
AAAGTACCTACATTTGGGTATGCAGCTTATGCATTATTAAAAAGTTGTGAAAATGAGATGGTAAGAAAAAAGTTG
TGGACCGCTCTTCACAGTTGTTCTGACAAACAGGTTAAAAGATTGAGTCACTTAATCAAACTAAGGGCAATCTTG
GCTAATTTAATGCACAAAACAAGTTACGCAGAGTATCAATTGGAAGGTAAATGGCAAGATCGAAGATGTCAAGAT
TTTATTTTGACGTTAATGAACAATACTATAGAGAAGACAGCAAATGAATTGAAATTTATAGCTGAACTCAAGGCC
AAAGATCTTAAGAAGCCGTTGACTACAAACACGGACGAAATATTGAAGCTCGTGAGACCATGGGATAGGGATTAC
TATACTGGCAAATATTTCCAGCTCAACCCGTCAAACTCTCCCAATGCCAAAGAAATAAGCTATTATTTTACATTA
GGAAATGTCATTCAGGGCTTGTCAGATTTGTTTCAGCAAATATATGGTATTAGATTAGAGCCAGCAATTACTGAT
GAGGGAGAAACATGGTCCCCAGACGTGAGAAGATTGAATGTGATATCTGAAGAGGAAGGAATCATCGGCATAATT
TATTGTGATTTATTCGAACGAAATGGCAAGACTTCAAATCCGGCTCATTTCACAGTTTGTTGCTCTAGGCAAATA
TATCCCAGTGAAACTGATTTCTCAACCATCCAAGTCGGTGAGAATCCAGACGGTACCTACTTTCAATTACCTGTT
ATTTCTTTGGTGTGTAATTTTTCTCCAATACTAATCGCTTCTAAAAAAAGTCTTTGTTTTTTGCAGCTTAGTGAA
GTTGAAACGCTCTTCCATGAAATGGGACATGCAATGCACTCAATGTTAGGGAGAACTCATATGCAAAACATAAGT
GGTACAAGATGTGCTACTGATTTTGTAGAGTTACCAAGTATCCTGATGGAGCACTTCGCTAAGGATATACGAATT
CTGACAAAGATTGGCAAACATTACGGGACTGGAGAAACAATTCAGGCTGATATGTTACAGCGCTTCATGAAAAGC
ACTAACTTCCTTCAAAATTGTGAAACATACTCTCAAGCAAAGATGGCTATGCTGGATCAATCATTTCATGATGAA
AAAATCATTTCTGATATTGATAACTTTGACGTTGTGGAAAACTATCAAGCACTAGAACGACGTTTAAAGGTCCTA
GTGGACGATCAGAGTAATTGGTGTGGAAGATTCGGCCATTTATTTGGATCGGGGCAACTTATTACAGCTACTTAT
TTTGATAGGACGATAGCTTCTAAAATCTGGTACGCCCTTTTCGAGGATGATCCGTACAGTCGAAAAAATGGTGAT
AAATTTAAAAAGCATTTACTAAAATGGGGAGGGCTAAAAGACCCTTGGAAATGTATCGCTGATGTTTTAGAATGC
CCCATGCTAGAGAAAGGCGGTAGCGATGCGATGGAATTTATAGCACAGTCTCACAAGTCTTAG

>YDR521W 0.73   Cold
ATGAAAGGTTCAAAATCGCACCTTGTCTTCACTCTCCTGCAAGTATCACAACTAAATGTTTTTCTTTTCTTTCTA
GGTTTTTTGTTGCCATTATTCTTAGGACTGTTCGTTTCACTGCGCTCTTTGGCTCTTGCACTATCCTCTGGGTGG
TTTATTATGGATCTGATACTATTTCGCACCTTTCCAGAAGCAGAATTATATCCAGCAGTCATCGGTAAACCATCT
GGTCTGGGGCTTACTGAGGCATTTGAGTTTATATCCATTTTTTTCCCTGATGTTCAGCAAACCGAAAGAAATATA
AAATACAACTGGGAAAGATGTTTTAATGGTGAGTAA

>YCL014W 0.76   Cold
ATGGAGAAAGACCTGTCGTCTCTTTACTCTGAAAAGAAAGACAAAGAGAACGATGAAACCTTATTTAACATCAAA
CTATCCAAATCTGTTGTCGAGACCACACCGCTAAATGGTCATTCATTGTTTGATGATGATAAATCACTTTCAGAC
TGGACGGATAATGTGTTCACTCAATCAGTATTCTATCACGGGTCAGATGACTTGATATGGGGGAAGTTCTTTGTC
TGCGTGTACAAGTCCCCCAACAGCAATAAGTTGAACGCTATAATATTCGACAAATTAGGAACATCATGCTTCGAA
TCCGTCGATATATCTTCCAACTCGCAATACTATCCGGCCATTGAGAATTTGAGTCCAAGTGATCAGGAAAGCAAT
GTTAAGAAATGCATTGCTGTCATTCTGTTACAGCGCTATCCATTACTTTCACCATCAGACTTATCACAAATATTG
TCCAATAAATCGGAAAATTGCGACTATGACCCCCCTTATGCTGGAGATTTGGCTAGTAGTTGCCAGTTGATAACA
GCAGTTCCTCCAGAAGATCTGGGGAAGCGCTTCTTTACATCAGGACTTCTGCAAAATAGATTTGTCAGCTCTACC
CTGTTAGATGTTATTTATGAAAACAATGAATCCACCATCGAACTAAATAATAGGTTGGTATTCCATCTGGGTGAA
CAACTTGAACAACTTTTTAACCCAGTCACAGAATACTCACCGGAACAGACAGAATATGGTTATAAGGCGCCAGAG
GACGAATTACCCACAGAATCGGATGATGATCTTGTCAAGGCCATTTGCAACGAGTTATTACAACTACAAACAAAT
TTTACTTTCAATTTGGTAGAATTTTTGCAAAAATTCCTGATCGCCTTGAGAGTCAGAGTACTCAATGAAGAAATT
AATGGGTTATCCACAACCAAATTAAATCGACTCTTCCCACCTACAATAGATGAAGTCACAAGAATCAATTGTATT
TTTCTAGACTCGCTAAAGACAGCAATCCCTTACGGTTCCCTCGAAGTACTGAAGGCATGCAGCATTACTATTCCT
TATTTCTACAAAGCATATACAAGACACGAGGCGGCCACAAAGAACTTCAGCAAAGATATTAAATTGTTTATTAGG
CATTTCAGCAATGTAATTCCAGAAAGAGAGGTCTACACGGAAATGAAAATCGAGAGTATAATTAAGGGACCTCAG
GAAAAACTACTGAAGCTAAAGTTAATTATAGAGAGATTGTGGAAGTCGAAAAAATGGAGACCGAAAAATCAAGAA
ATGGCAAAAAAAATGCTACAACAATATCATTGATGTCATTGATTCGTTTGGAAAATTAGATTCCCCACTTCATTCT
TATAGTACCAGAGTATTTACTCCATCGGGAAAAATCCTTACAGAATTAGCCAAATGCTGGCCCGTAGAACTGCAA
TACAAATGGCTGAAGAGAAGGGTAGTCGGTGTGTATGATGTAGTGGATTTGAATGATGAAAATAAGAGAAATTTA
TTAGTCATATTCAGTGATTATGTGGTTTTCATCAATATACTGGAGGCAGAAAGTTACTACACTTCAGATGGATCA
AACAGGCCCTTAATCTCAGATATTTTAATGAACTCATTGATCAACGAAGTTCCGTTGCCCTCCAAGATCCCTAAG
TTGAAAGTGGAGCGTCATTGCTATATAGATGAGGTTCTAGTTTCTATATTAGACAAAAGCACTCTACGTTTTGAT
CGATTGAAGGGAAAAGATTCTTTCTCAATGGTATGTAAATTATCCTCTGCATTTATCTCTTCTTCGTCAGTTGCT
GACTTGATTACGAAGGCTAGAATTTTGGAAAAAGACACTGCATTTCATTTATTTAAAGCTAGTAGAAGCCATTTT
ACATTATATTCTACTGCTCACGAGCTTTGCGCTTATGATTCCGAAAAAATAAAATCAAAATTTGCCTTATTCCTG
AACATACCACCATCCAAGGAGATATTGGAGGTCAACAACCTTCATTTGGCTTTTTTTGCAAGATTTTGCAGTAAC
GATGGTAGAGATAACATCGTAATCTTAGACGTCTTAACCAAACATGACGATAAACATATAGAAGTTACATCCGAT
AACATTGTTTTCACCATAATTAATCAATTGGCCATTGAAATACCGATATGCTTTTCTTCCTTAAACTCATCGATG
GCCAAAGATTTACTCTGTGTAAATGAGAATTTGATAAAAAACTTAGAACATCAATTGGAAGAGGTCAAGCACCCT
TCAACAGACGAACATAGGGCTGTTAATAGCAAACTTTCCGGTGCATCCGATTTCGATGCTACTCACGAGAAGAAA
AGATCATACGGTACCATAACAACATTTAGAAGCTATACAAGCGACTTGAAGGACAGTCCATCAGGCGATAATAGT
AATGTCACCAAGGAAACTAAGGAAATTTTACCAGTGAAACCTACGAAAAAGTCTTCAAAAAAACCAAGAGAAATT
CAAAAGAAGACCAAGACAAACGCCTCTAAAGCAGAGCACATAGAAAAGAAGAAGCCTAACAAAGGCAAAGGGTTT
TTTGGCGTGTTAAAAAATGTTTTTGGAAGTAAAAGCAAGAGCAAGCCTTCACCAGTTCAAAGAGTGCCTAAAAAA
ATATCGCAGAGGCATCCTAAGTCTCCAGTGAAGAAGCCAATGACCTCAGAAAAGAAATCCTCCCCTAAAAGGGCA
GTCGTTTCATCTCCCAAAATTAAAAAGAAAAGTACTTCTTTTTCCACAAAAGAATCACAAACTGCTAAATCTTCT
CTTCGAGCAGTTGAATTCAAATCTGATGACTTGATCGGAAAACCACCTGATGTTGGAAATGGCGCACATCCTCAA
GAAAATACCAGAATATCTTCAGTAGTAAGGGATACAAAATATGTCTCCTACAATCCCTCTCAGCCTGTGACAGAA
AATACCAGTAACGAAAAAAAATGTCGAACCAAAAGCGGATCAATCCACAAAGCAGGATAACATTTCCAATTTTGCA
GATGTAGAGGTATCTGCGTCTTCTTATCCTGAAAAACTTGATGCAGAAACAGATGATCAAATAATTGGGAAGGCG
ACGAATTCGTCATCAGTTCATGGAAATAAAGAGCTGCCAGACCTTGCTGAGGTGACTACAGCAAATAGGGTTTCT
ACAACATCGGCTGGGGACCAACGTATTGATACCCAAAGCGAATTTTTACGTGCAGCTGATGTTGAAAACTTAAGT
GATGACGATGAACACAGACAGAATGAAAGTAGAGTTTTTAACGATGACCTCTTTGGTGATTTTATTCCTAAGCAT
TACCGTAATAAACAGGAGAACATTAACAGCTCGAGTAATTTGTTTCCAGAGGGAAAGGTGCCCCAAGAAAAGGGC
GTATCAAATGAAAACACTAACATATCTCTCAAAACTAATGAAGATGCATCTACATTGACGCAGAAACTCTCTCCA
CAAGCGAGTAAAGTGCTGACAGAAAATTCTAATGAATTAAAAGATACCAACAATGAAGGGAAGGACGCAAAGGAC
ATAAAATTAGGAGATGATTACAGTGATAAAGAAACAGCGAAAGAAATAACTAAACCAAAAAATTTTGTTGAAGGA
ATAACTGAACGGAAAGAAATATTCCCCACTATTCCTAGGTTAGCGCCGCCAGCTTCAAAAATTAACTTTCAAAGG
TCACCATCCTATATTGAGCTCTTTCAAGGAATGAGGGTGGTTTTAGATAAGCATGATGCCCATTATAACTGGAAA
CGCTTGGCTAGTCAAGTCTCCTTAAGTGAGGGACTAAAAGTCAATACTGAGGAAGATGCGGCAATTATAAATAAA
AGTCAGGATGATGCCAAGGCGGAAAGAATGACTCAAATTTCTGAAGTGATTGAGTATGAAATGCAGCAACCTATC
CCAACTTATTTGCCTAAGGCGCATCTAGATGACTCGGGTATTGAAAAAAGTGATGACAAATTCTTCGAAATTGAA
GAAGAACTTAAGGAAGAATTGAAGGGCAGCAAAACCGGTAATGAAGATGTCGGTAATAATAATCCATCCAATTCT
ATTCCAAAAATCGAGAAGCCCCCAGCATTCAAAGTTATTAGAACATCGCCTGTGAGAATTATCGGAAGGACTTTT
GAAGACACTAGAAAATATGAAAATGGCTCTCCATCTGATATTTCGTTCACTTATGATACTCACAACAATGATGAA
CCTGACAAAAGGCTGATGGAATTAAAATTTCCATCCCAAGATGAAATTCCGGATGACAGATTCTATACTCCAGCA
GAGGAACCCACTGCTGAATTTCCGGTGGAAGAACTTCCAAATACTCCGCGAAGTATTAACGTTACAACTTCAAAT

AACAAGAGCACAGACGATAAGTTGAGTAGCGGTAATATTGATCAAAAACCTACCGAACTGTTAGATGATTTAGAA
TTCAGTTCATTTAATATAGCATTTGGAAATACCTCCATGAGTACTGACAATATGAAAATATCATCCGACTTAAGT
TCGAATAAAACCGTGTTAGGAAATGCTCAGAAAGTTCAAGAGTCTCCTAGTGGACCATTAATCTATGTTTTGCCT
CAGAGTAGCACAAAGCATGAGAAAGAGGGGTTCCTTCGAAAGAAACAAAAAGACGAGCCCATTTGGGTTTCCCCT
AGCAAAATTGACTTTGCTGATCTAAGTAGGAGAACTAAAGCATTGACGCCAGAGCGTAATACTGTTCCTTTGAAA
AACAACGACAGTAGAAAATACAAATATACTGGAGAGGGATCTATCGGTAATATGACAAATATGCTGTTAACTAAA
GATGCTTCGTACGCGTACTTAAAAGATTTTGTTGCGTTGAGTGACGATGAAGATGAAGATGGGAAACAGAACTGC
GCTGTTGGTGGCCCAGAGAAACTGAAATTTTATTGA

>YIR026C 0.81 Cold
ATGGCTGGAAATGCAAACTCAGTAGACGAGGAAGTTACCAGGATATTAGGAGGCATATATCTTGGCGGAATCCGT
CCAATTATTGACCACAGACCATTGGGTGCAGAATTTAACATTACTCATATTCTTTCTGTTATCAAATTCCAGGTC
ATTCCAGAGTATCTAATAAGGAAAGGTTACACGCTAAAAAACATACCCATCGATGATGATGATGTGACTGATGTG
CTGCAATACTTCGATGAAACGAACCGATTCATTGATCAATGCTTGTTCCCCAATGAAGTTGAGTATTCGCCCAGA
TTAGTAGATTTCAAGAAGAAACCACAACGTGGTGCTGTTTTTGCTCATTGTCAAGCAGGACTCTCGAGATCTGTA
ACCTTCATAGTAGCCTACCTAATGTATCGTTATGGATTGTCACTATCAATGGCTATGCACGCTGTCAAGAGGAAG
AAACCGAGTGTTGAGCCAAACGAGAATTTCATGGAACAATTACATCTCTTTGAGAAAATGGGTGGAGATTTTGTC
GATTTCGACAACCCAGCCTATAAGCAGTGGAAGCTGAAGCAATCTATCAAGTTAGATCCATCGGGCAGCGAATTG
GTCTCCAATTCTGGAATGTTTAAAGATTCGGAGTCGTCGCAAGACTTGGATAAATTAACTGAAGCTGAGAAGAGC
AAGGTCACCGCAGTGAGATGTAAGAAGTGCAGAACCAAATTGGCGTTATCTACATCTTTCATCGCGCATGACCCA
CCAAGTAAGGAATCATCAGAGGGACACTTCATCAAGAGGGCCGCCAACTCCCATCGTATTATTGATATTCAAGAG
TCACAAGCAAATTGCTCTCATTTTTTCATCGAACCTTTAAAATGGATGCAACCTGAACTACAAGGCAAGCAAGAG
TTAGAAGGGAAGTTTTCATGTCCAGGCTGTTCAAGTAAAGTTGGCGGTTACAACTGGAAAGGATCTAGGTGCAGT
TGTGGTAAGTGGGTCATCCCCGCTATCCACCTGCAAACTAGTAAAGTGGATCAATTTCCCTTACAATCCACTGCT
TTGCCCAACATGGTTAATTTTGAATCCGAGAAAGTAAATAGATAA

>YJL220W 0.79 Cold
ATGTGTCTTTTCGATCAAGGCAAAGCAGTCCTCGTTCGTACCGTAGGTTGGCCAGACCTTTTCGTAGTTGGCAAT
ATCGTAACCCATATCATCTTGTGGCGAGTCGTAAAATGGCGAGATCCAAATGGCATCGGTACCAAGCTCTTTGAT
GTACTCCAACTTGGAGGCAATACCCTTCATATCACCCCAACCATCGTTGTTGGAGTCTTTGAAACTTGCGGGATA
AATTTGATAAATTGTGGCCTCTTTCCACCACTTGGGTTCTGTTTCTGGATGTGCAGAAGAAATAGTCATTGTACG
TTGCTTTCTTATCTCTATGAAAAAACTTTCACTTTTGTTTGTGTGTTTGGATTGGTGAATGGAACATATATTCTA
GTACAGCTAATTCCTCGAGTGAAGAAGTCTTCCTTACTTATATATGTCTGTGGACGTCTTACGAATGATCGAAAA
TAA

>YLR361C 0.79 Cold
ATGATCCGGTTACCGAGGCTGTATCAAAGGTACCTATTATACTTGGTAGTTTTTGTAGTGATTGCCCTATTTTAC
TTCCTACAGGCGCCAAGAGTTGAAGAGCACATTGGTTTCGACCTTGCACTCCCGATATCACACGTTGATAATCTA
TGGTTCCAAAATAAAGGTCTAGAGGGATTCTCAAATGACGATAAGCTAGTTGTAAATATAGGCTATGACGAATGT
TTTCATATTGGTCGTTTTTATGAAGGATGTTTTAATCGGCATGAACTTAAATCAACTTTAACTGATGGGCACCAA
TACTTACAAAGGAAAAGAATTCATAAAGACTTGAGAGGTTCCTTTGGACGCAGGTGGTTTGGAAAGTCAGAATAC
CTTTACTATGATGTGTTATACCCTGCTTTGGTGGACTACTTTGGATCCAACCTAGAAAAGCTAAATGTTGAGGCA
GTAACTGGAATTTCCAAATATCCTAAAGACAAATCGCTACCCTTTATGGATGTATCCATCACATTCGAGCCTATT
AGCATAGAACTATTGCAGAAAAGGAGCTACATAAGCGATATTAACATTCTCTTTGGTGTTGATTGCATCCAGCCG
ATAGCAAATTGGACTCTTCAAAAAGAATTTCCCTTAGTAAAATACCGCTACAGCGAACCTGCTTATTTGACTTAC
AAATTCGTTGGTACTCGTCCTGTAGACACAGGAGCTCAACGGCTGCAAGAAACTGATGAAGGAAAGTTCAAAATT
GTTCAGTTGGCGGATCTCCACTTGGGTGTGGGGGAGTCTGAATGTATCGATGAGTATCCAAAGCATGAAGCTTGC
AAGGCAGATCCAAAAACGGAAACTTTCGTTCAACAAGTTCTTGACATTGAAAAACCGCAGTTGGTTGTTTTTACT
GGTGACCAAATCATGGGTGATAGATCTATTCAAGACTCTGAAACCGTATTATTAAAGGCGGTAGCACCCGTCATT
GCAAGAAAAAATTCCGTGGGCAATGGTATGGGGAAATCACGACGACGAGGGAAGCTTAACGCGCTGGCAGTTATCC
GAAATAGCTTCAGTACTACCATATTCCCTTTTCAAGTTTAGTCCCCATGATACACACGATAATACGTTTGGCGTT
GGTAATTATATTTATCAGATTTTTTCGAACAATGATACAGAAGTCCCAGTAGGTACACTTTATTTTCTGGACTCA
CATAAATATTCTACGGTTGGTAAAATTTATCCTGGATATGATTGGATTAAGGAATCACAGTGGAAATACATTGAA
GATTATCATGATGTGAACTTGAAGTTCAAAACAGGTCTTTCAATGGCATTTTTTCACATCCCACTGCCAGAATAC
TTGAATATTGAATCCAAGACACATCCCGGAGAGAAAAATCCCCTCATTGGAATGTACAAAGAAGGTGTTACTGCA
CCTAAGTATAATTCTGAGGGGATAACAACTTTGGATAGGTTAAGTGTGGATGTCGTCAGTTGCGGACATGACCAT
TGTAACGATTACTGTCTTCGAGATGACTCTACGCCGAACAAAATCTGGCTATGCTATGGCGGTGGAGGTGGAGAA
GGTGGTTATGCTGGCTATGGAGGTACAGAAAGACGTATCCGTATTTATGAAATTAATGTAAATGAGAACAATATA
CACACATGGAAAAGATTAAATGGCAGTCCAAAAGAGATTTTTGATTTTCAGTCTATGTTGGACGGTAATTCTCCA
GAATCTGTTTAA

>YKL016C 0.82 Cold
ATGTCTTTGGCCAAATCCGCTGCTAACAAACTTGACTGGGCAAAAGTCATCTCTTCTCTACGTATAACAGGGTCG
ACTGCCACTCAATTATCGAGTTTTAAAAAGAGAAACGATGAGGCACGTAGACAATTACTAGAGCTGCAAAGTCAA
CCCACCGAAGTAGATTTCAGTCATTATAGATCTGTGTTGAAAAACACTTCAGTCATTGACAAGATAGAATCATAC
GTTAAGCAATATAAGCCGGTCAAAATCGATGCCTCCAAACAATTACAAGTGATTGAGTCTTTTGAAAAGCACGCA
ATGACCAACGCCAAGGAGACAGAATCCTTAGTTTCCAAGGAGTTAAAGGATTTGCAAAGCACCTTAGATAATATT

CAATCTGCAAGACCTTTCGATGAATTAACTGTTGACGACCTGACAAAGATCAAACCTGAAATTGATGCTAAGGTA
GAAGAGATGGTCAAGAAGGGTAAATGGGACGTACCTGGTTACAAGGACAGATTCGGCAATTTGAATGTGATGTAG

>YKL050C 0.79   Cold
ATGTCACTAATATCTGCGTTGCAAACCACCGATGTGGAATCTGTCCAGACGTCCCCTGAGCAAATTACTGAAAGA
AAGGCGGTTAGAGTATCCACTTTGCAAGAATCGCTACATTCTTCTGAAATGCATCGCGCTGCTCCAGAAACACCT
CGCTCAATTTCAAATAGCGTTCACAAACTGAAAACTATATATTCTACTTACCAGCAGAGTGGACAACCATTGAGT
AAAGAGGCGATATTCCGTGCGAAACAGAAGTATGGCATTTTGAATACTCCGGCTAATTACAAAACACTTGGTTTA
GGAGACTCCAAGAGTGAGTCAGTTGATCTTGCGGCCCGTTTAGCTAGTAAGAGAACGAAAGTCTCGCCTGATGAC
TGTGTCGAAACAGCAATCGAGCAAAAGGCCAGAGGTGAGGCTTTCAAAGTAACCTTTTCCAAGATTCCATTGACC
CCGCCTGAAGACGTCCCAATAACGGTCAATTTAGGTCTGAAGGGAAGGCGTGATTTTTTAACAAGATTAGCTGCA
CAAAAAGCATTGGCTTTCAGTCCTTCTTTAGACAATTCCATGAAGGGCACCTCCGACAGTTCCAGTGTTAAAAAA
AAGAGGTTTTCTGGTGCCCCTATTGGTAATGAGTTTGATGCAAACTTAGTTAATCCACAGCACCCTGCAGGTTTC
AAATCCCTCGATTTATCTAAGGTATTGGATGGAGCGGAAAGAAGGGCGATTAGCAGAGTTAATGACAGGTTGTAT
CCCCAAAAGGTAAACTTTAAAAATGGATTACAAAGCAGCGATCAAAGTGGTGTATCAAAAGCTAACAAAGAAGTT
TTTAAGAAAGGAACTTTGGAAAAACTTGAACATTCCGCAGAGCAGTTCTTAGAGTCTCATGCTGGTAATGAAAGA
CAAAGGCTAAGTGATCAACAATATATGTGTGCAAAGGGTGCAGCTGATGCAGTTAAAGATCTTGACCCGAAGACA
TTGGAAGACCCAGATTTTGCTGCAAGAGAGGCACAAAAGAAACTGTACATAAAGCAGGTAGCATCTCCGGTAGTG
CTAAATGAAGCTCAAAAGCTTGCGAATAGGAAATTGCAGGATATTGACTCGCGCGATACTTACATGCTTTTATTT
GGTAACCAAGCTTACAATAAGCTTGCTGTTAACATAGCTCTACAGCACTATTCTGTTAAACAAGAAGAAAAAAAG
AAAATATATTTGGGTGGTGGTTTGTGGATGACTCCAGAAGAGGTTAACGCAGTCGCCAAAAAGTTGATATCACCA
GTGGTGAACGAAATTGATGAACGAGCCAGCAGACAACGTGATGTTGATAAAGATATTGAGAGAAGGTCAAGAGTT
CTTGATCAGGAATATGAGGATGGGAATTCTATGGAACGTGCAAAAGAGCAAAACGACGGGCAATTATTGTTAGCG
ATGGCTTCAAAACAACAACAGGAGAAAGAAGCTAAAAAGGCCGAGGAGGGACAACGGTATGATCAGTTCGTACAA
AAGATGAATATCAAATTACAGCAGAAGGAAAAAGAACTTGAAAATGCTAGGGAAAATCGTGAAAATCTAAGAAAC
GAACTACAGGAAAGGTTGTCCAAGAACCTGTCAGGAGAGAATGATGAATTGAATGATTGGAACGATGCATGTGAA
AGAGATCTCAAAAATTCGAGCATTGAACACTATTATGCCGTCAGATCACACTTTGACAATTTGGGAAATTCGGAA
AGAGGGTATGATGAGCTTTTGGAGGAGCGCAGCAAAATACAGGTAGAAATCGAAAGACTTGTCGCTTCCATTGCT
GAACATAAAACTGCAATCCATGGTTTTGGGGAAACAGCAGATGCTGGTGGAGCTATACCAGCTGTACAGAAACAG
AAAATTCCAACAAGGAAGGACCTTCTTGATGCTACCGTGAATGATCCTTTGGTTATTTCTGCAGAAATGGCAAAA
GAAGAAGCAGAAATGGCTACAGAAGAATGTATGTTGAAAGAATTACAGGTAGATGAAATGATTATAATCAGGAAC
ATTATGCTGCGGGAGTGTGAAAAGAAGCTCGAAGAGGAGAAGGAAACGGCTAAGAGGTCTAGAAGGGGCACTGAG
GAGTCAAAGAATAATTCTAATTTTTCTCGAGATGTTATCATGAGTACACCTGATAACAATGAAAAAGTGACTCCT
ATAGGGAAGAGTGCATCTCCAAAAGATGTTGTTAAAAGCCGTTTCCTTTCTACTTATAATACAGGAAAGGATATT
GATAGTTCTGCCAGTGCTAGGTCAATCACTGGTGTTAGCGGCGTGTTGGATGATGGGCCTAAAACACCAACCTCC
AATAAGGAGAATGAATTAATTGACGATGAAGTGAAGTCATATAAAGTGCACCAAGCAGTAGATGGCACCGGTGAA
GATTCCATCGCAAACAAGAGGGATAAATCTTCAAGACCAGCGGCGAACAGTGGAGGCTCCATAACGATAGAACAA
TTTTTATTTAATAAGAATGCCGATAAACAAGGACTATCCAAAACAGAGTCTGTAACTATGAAGAGGGAGCCTGTG
GTGGATCAAATGGACTCAAAAAAGGGGCACGATTTTACCCATTGTAACGACAACGGCCGCCGGTCTTTTAGTGGC
TTTTCTCAAGGTTCGATAGAAAATGATTATAGTAATGAAGTTACAGATGATCAAGACGATCAAGAGGGATCGGAG
ATAAGAGTTCGCGATTCCAACGATTCCAATACCTCTCCTAAGGAAAGTTTTTTCAAAGAGGTTATCTAA

>YDR016C 0.69   Cold
ATGATGGCTAGTACATCCAATGATGAAGAAAAGTTGATATCAACCACTGACAAATACTTCATCGAGCAGAGGAAT
ATAGTATTGCAGGAGATCAATGAGACTATGAATTCAATCTTGAATGGTCTGAATGGTCTCAATATATCATTGGAG
AGCTCGATAGCTGTGGGAAGAGAATTCCAGTCAGTTTCTGATTTGTGGAAAACACTTTATGATGGGTTGGAAAGT
TTATCAGACGAAGCGCCCATCGACGAGCAACCTACTTTATCTCAATCGAAAACGAAGTAA

>YDR261C 0.72   Cold
ATGCCTTTGAAGTCGTTTTTTTTTTCAGCATTTCTAGTTTTATGCCTGTCTAAATTCACGCAAGGCGTTGGCACC
ACAGAGAAGGAAGAATCGTTATCGCCTTTGGAACTAAATATTTTACAAAACAAATTCGCCTCCTACTATGCAAAC
GACACTATCACCGTGAAAGGTATTACTATTGGCGGCTGGCTAGTAACAGAACCTTATATCACGCCATCATTATAT
CGTAATGCTACGTCACTGGCAAAACAGCAAAACTCTTCCAGCAATATCTCCATTGTCGACGAATTTACTCTTTGT
AAAACCTTAGGATATAACACCTCTCTAACTTTATTGGATAATCACTTCAAAACTTGGATTACAGAGGATGATTTT
GAACAAATCAAAACCAACGGTTTCAATTTAGTTAGGATCCCCATCGGATATTGGGCGTGGAAACAAAATACTGAT
AAAAACTTGTACATCGATAACATAACTTTCAATGATCCATACGTAAGTGATGGATTACAACTGAAATATTTAAAT
AATGCTCTCGAATGGGCGCAAAAGTACGAACTAAATGTATGGTTAGATCTACATGGTGCTCCTGGATCCCAGAAT
GGATTCGATAATTCCGGTGAAAGAATACTCTATGGCGATTTAGGCTGGTTAAGGTTGAATAATACTAAAGAACTG
ACTCTGGCTATTTGGAGAGATATGTTCCAGACATTTTTAAATAAAGGTGACAAAAGTCCTGTGGTGGGTATTCAA
ATCGTCAACGAACCGCTTGGTGGCAAAATCGATGTTTCAGACATAACGGAGATGTATTACGAAGCATTTGACTTG
CTCAAGAAAAATCAGAATTCGAGTGACAACACTACGTTTGTTATTCATGACGGTTTTCAAGGAATCGGTCACTGG
AACTTGGAGCTAAACCCAACCTACCAGAATGTATCGCATCATTATTTCAATTTGACTGGTGCAAATTACAGCTCT
CAAGATATATTGGTCGACCATCATCATTATGAAGTGTTTACTGATGCGCAATTGGCCGAAACTCAGTTTGCACGT
ATTGAAAACATTATCAATTATGGGGACTCTATCCACAAAGAACTTTCTTTTCACCCAGCAGTAGTCGGAGAATGG
TCAGGCGCTATTACTGATTGTGCAACCTGGCTAAATGGTGTTGGGGTGGGTGCACGTTACGATGGATCATACTAC
AATACAACGTTGTTTACCACCAACGACAAGCCAGTTGGAACATGTATATCCCAAAATAGCTTAGCTGATTGGACG
CAAGATTACCGTGACCGTGTGAGACAATTCATTGAGGCACAGCTAGCCACTTATTCGTCAAAAACAACGGGATGG

ATTTTTTGGAATTGGAAGACCGAAGACGCCGTAGAATGGGATTATTTGAAGCTAAAAGAAGCTAACCTTTTCCCT
TCCCCTTTCGACAACTACACGTACTTCAAAGCAGATGGATCTATCGAAGAAAAATTCTCATCCTCTTTATCAGCA
CAGGCATTTCCAAGAACAACGTCATCGGTTTTGTCCTCCACTACGACTTCCAGGAAGAGTAAGAATGCTGCAATT
TCTAATAAACTAACAACTTCGCAGCTATTACCAATCAAAAATATGAGTTTGACCTGGAAAGCGAGCGTATGCGCA
CTCGCTATCACCATTGCCGCTCTTTGCGCTTCTCTTTAA

>YDR253C 0.8Cold
ATGGAGGATCAGGATGCTGCATTTATCAAACAGGCTACAGAAGCAATAGTGGATGTATCATTAAATATAGATAAC
ATAGATCCTATAATAAAAGAGTTATTAGAAAGGGTAAGGAATAGGCAAAACAGGTTACAAAATAAAAAACCAGCA
CTCATACCGGCAGAAAATGGTGTTGATATAAATAGTCAAGGCGGTAACATAAAGGTTAAAAAGGAAAACGCATTA
CCAAAACCACCGAAGTCCAGCAAAAGCAAACCCCAAGATCGTAGAAATAGTACTGGTGAAAAAAGATTTAAATGT
GCGAAATGTTCGTTGGAATTTTCAAGATCATCAGATTTGAGAAGGCACGAAAAGACACACTTCGCCATATTGCCT
AACATTTGTCCTCAATGTGGCAAAGGTTTTGCAAGGAAAGATGCATTGAAAAGACATTATGATACACTGACATGT
AGGAGAAACAGGACTAAATTACTAACTGCGGGTGGTGAGGGTATCAATGAATTACTGAAAAAAGTCAAGCAATCC
AACATCGTTCATCGTCAAGATAACAACCACAATGGTAGCAGTAATGGCTGA

>YOL142W 0.8Cold
ATGTCTACGTTCATATTCCCTGGTGATAGCTTTCCTGTAGACCCTACTACACCTGTTAAACTGGGCCCTGGCATA
TATTGTGACCCCAATACTCAAGAAATACGACCTGTTAATACAGGTGTTTTGCATGTTTCCGCTAAGGGTAAGAGT
GGTGTTCAGACCGCATATATAGACTATTCTAGTAAGAGATACATTCCATCTGTAAACGATTTTGTAATCGGTGTC
ATTATAGGGACATTTTCAGATAGCTATAAGGTTTCGTTGCAAAATTTCTCCTCCAGTGTTTCACTATCGTATATG
GCTTTTCCCAATGCTTCAAAGAAAAACAGGCCAACTTTGCAAGTGGGAGATCTAGTGTATGCGAGAGTTTGTACC
GCAGAAAAGGAACTAGAAGCCGAAATAGAATGTTTTGACTCAACTACAGGACGCGATGCTGGTTTCGGGATATTG
GAAGATGGTATGATCATTGACGTGAATTTCAATTTCGCACGCCAGTTGCTTTTCAATAATGACTTCCCGTTATTA
AAAGTGTTGGCTGCACATACCAAGTTTGAAGTCGCCATTGGTCTCAATGGGAAGATCTGGGTTAAGTGCGAGGAA
TTATCTAACACTTTAGCTTGTTATAGAACCATAATGGAGTGTTGTCAAAAAAACGACACGGCAGCGTTCAAGGAT
ATAGCAAAAAGACAGTTTAAAGAAATACTTACGGTCAAGGAGGAGTAG

>YOL150C 0.76 Cold
ATGATAAAAAATAATTGTAATAACGTTAATATATATAAATATTATCTATTTTCATTTAAAGTTTATATTCTGCCC
TCAAATTTTAAAATTTGGGAGGCAGTGTCGTCAATGGTCTCTTTCAAGTTCCTGAACTTGAAACCTAACAATTTC
TTACTCTTTTTATTATCAAGAGTAGCACCAAGGGTGTTATGGGTAGCACCAGAACCTGGTTTCCCCACTGGAATA
TTGCCTTTTAGAACAGGGAAGTCTTCGTTAAGGATATCGAGAACATCCTGCATAGTAAATCTGGCCTCCGATACG
ATTAGTCTTTGA

>YOL140W 0.78 Cold
ATGTTTAAAAGATATTTATCCAGTACGTCATCAAGAAGATTTACAAGCATTTTAGAGGAAAAGGCCTTTCAAGTG
ACCACTTACTCTAGACCTGAAGATCTATGTATAACTAGAGGTAAAAATGCAAAGCTGTATGATGACGTGAATGGT
AAAGAATATATCGATTTCACCGCAGGTATTGCGGTGACCGCATTAGGCCATGCAAATCCTAAAGTGGCAGAAATT
CTGCACCATCAGGCTAACAAACTGGTTCATTCCTCCAACCTTTACTTCACTAAGGAATGTTTGGATTTAAGTGAA
AAGATTGTTGAAAAGACCAAGCAATTCGGTGGTCAACACGACGCCTCAAGAGTATTTTTATGTAATTCTGGTACG
GAAGCAAATGAAGCTGCTTTGAAGTTTGCAAAGAAACATGGTATAATGAAAAATCCTAGCAAGCAAGGCATTGTT
GCATTTGAGAACTCTTTTCATGGCCGTACTATGGGCGCTTTATCTGTCACTTGGAATAGTAAATATAGAACTCCT
TTTGGGGATTTGGTTCCCCATGTCTCATTCTTAAATTTGAATGACGAAATGACCAAACTACAAAGTTATATCGAG
ACCAAAAAGGACGAGATTGCTGGTTTAATTGTCGAGCCCATACAAGGTGAAGGTGGGGTTTTTCCCGTAGAAGTT
GAAAAGCTAACCGGATTGAAGAAAATATGTCAAGATAATGATGTGATTGTCATTCATGATGAAATTCAATGCGGT
TTGGGCCGTTCAGGTAAACTATGGGCTCATGCTTATTTACCAAGTGAGGCTCATCCGGATATTTTTACATCTGCC
AAAGCATTGGGAAATGGCTTCCCCATCGCTGCCACCATCGTCAATGAAAAAGTTAATAATGCTTTGAGAGTTGGT
GACCACGGCACCACGTATGGTGGTAATCCGCTGGCCTGTTCTGTAAGCAACTATGTTTTGGATACCATAGCAGAC
GAAGCTTTTTTGAAACAAGTCTCTAAGAAGAGTGATATCTTACAAAAGCGCTTGCGCGAAATTCAAGCCAAATAT
CCAAATCAAATAAAGACTATCAGAGGAAAAGGTTTGATGCTTGGTGCTGAGTTCGTCGAACCACCCACCGAGGTC
ATCAAAAAGGCCAGAGAATTGGGACTTTTGATCATTACCGCTGGTAAGAGTACCGTTAGATTTGTTCCCGCATTA
ACGATTGAAGACGAACTAATCGAAGAAGGGATGGATGCTTTTGAAAAGGCTATTGAAGCGGTTTACGCTTAA

>YGR187C 0.75 Cold
ATGACTTCACAATTGAATGAATTAGTGGAATTTCTGCATTCACCACAACCTGCCGTAAGACAGATTGCCATTGAT
AATCTAGTTGGATTTAGTGCTGGTCCCACTTCAAAAGTTTTCAAAAATGATAGCTACAGACCCATTAAGGATATA
ATAAAAATGATCATGGATCCAGAACACGGCACCCGTGTTATTATTCAGCAAGGTGTCACTATATTGGTCAATTTA
TCTGAAGACAAGTTAGTAAGAAATATTATATTGAGCGATGACAAGAAGTTCTTAAAATTTTTGGTTTGGAAAATT
GTCGATTTAACCAATCCAAATGCCGATATAATGTGTATCTTATTAAGCAACTTAGCCAAGGATGACGGCATTCTT
GCTGTTCTGAATATCAAAAGGAATTCAAGCGGTGAGGAGGTCGATGATGGTTTGAAGTTAGCAGCTTTAAATAAA
GAAGTTTTCAAAAGTTTGAGAGCCATGGATTGTTTAATGGATTGCTTTGTTAAGGGTTATGATAAAAAATTAACA
AAATATGCCAGTTTCAATTATTTAGCCTTTTTTTTTGCGGATATCTCGAGGTTTAAATTGGGCAGGATGTATTTT
ATTGAAGAGCAGGAATACGATGGGGTTGTTCCTATTTCAAAATTGCTAGTATTTACCGAAAAATATGATGCAAAG
GTAAGAAGAGAAGGTGTCGCTTCCACAATTAAAAATTCCCTATTTGATTCTGAAACTCATGAAAGGTTACTCAAA
GATGAAAAAATAAACTTGTTGCCTTACATCCTGCTACCTATTGCTAGTGCTAAAGATTCTGAAATTGATGAGGAA
GATATGTTCAATTTACCAGATGAACTTCAATTATTACCAGAGGACAAGGAAAGAGACCCGATTCCTGCAATTATA

TGCTGTCATTTAGAAAGTATTCTTTTGCTATGTACAACGCATGCTGGAAGAGAATACTTAAGAGACAAGTCCGTT
TATCCACTAGTGAGGGAGCTGCATAAAAACGTTGAGAATGAAGATATTGGTGAATTATGTTACAGAATTGTTAAT
ATGTTAATGAGAGGCGAGCCAGGTGCGGGAGCAGTGGAAGAGATGCCATCAAAGAATGCGGAAGAGGAAGAAGAG
GAGGAGAGTGAAGAAGAAGAAGATGACGACGAAGAAGATGAAATTGTCGAAGTGGCCTAG

>YHR056C 0.69 Cold
ATGATGGACATGCAAGTGAGAAAAGTGAGGAAGCCGCCTGCTTGCACCCAATGCAGGAAGAGAAAGATCGGGTGC
GACAGGGCCAAACCGATATGTGGGAATTGCGTCAAGTATAACAAGCCGGACTGTTTTTATCCAGATGGACCTGGT
AAGATGGTCGCTGTGCCCTCTGCGTCCGGGATGTCCACGCACGGCAATGGCCAAGGTTCCAACCATTTTAGTCAG
GGAAACGGTGTAAATCAGAAAAACGTAATGATTCAAACGCAGTATCCGATTATGCAAACGTCGATAGAGGCATTC
AACTTCTCGTTCAACCCCTCTGTGGATACTGCGATGCAGTGGACCAAGGCCGCTAGCTACCAGAATAATAACACC
AATAATAATACTGCTCCTCGTCAGAATAGTAGTACCGTTAGTAGTAATGTTCATGGAAACACTATTGTGAGAAGC
GATAGTCCAGATGTGCCCTCCATGGATCAGATTAGAGAATATAACACACGATTACAACTGGTTAACGCTCAAAGT
TTTGACTATACAGATAACCCATACTCTTTTAATGTTGGTATCAATCAAGACTCGGCCGTTTTCGATCTAATGACT
TCTCCGTTTACTCAAGAGGAAGTATTAATCAAGGAGATAGACTTTTTAAAAAACAAATTGCTTGATTTACAAAGC
TTGCAACTGAAAAGTTTGAAAGAAAAATCGAATTTAAATGCCGACAATACCACGGCAAACAAAATTAACAAAACA
GGTGAGAATTCTAAGAAAGGCAAGGTTGACGGTAAAAGAGCCGGATTTGATCATCAGACTTCAAGGACTTCTCAG
TCCTCACAAAAATACTTTACAGCGCTCACAATAACAGATGTGCAAAGTTTAGTCCAAGTGAAACCGTTGAAGGAT
ACCCCCAACTACCTTTTCACTAAAAACTTCATCATTTTTAGAGATCATTATCTTTTCAAGTTCTATAATATTTTG
CACGATATCTGCCATATTAATCAGTTCAAAGTAAGTCCTCCTAACAATAAAAATCACCAACAATATATGGAAGTT
TGCAAAGTTAACTTCCCACCAAAAGCAATAATTATTGAGACACTAAACTCTGAATCCCTTAACAATCTGAATATT
GAAGAATTTTTGCCAATCTTTGACAAAACCCTCTTACTAGAATTTGTTCATAACTCTTTTCCAAATGGTGATACC
TGTCCTTCATTCTCAACGGTCGATCTTCCTTTATCTCAACTGACCAAACTAGGCGAATTAACTGTGCTTCTACTG
TTGTTAAACGATTCAATGACCCTATTCAATAAGCAGGCTATTAATAACCATGTTTCGGCATTAATGAATAATTTG
AGGTTGATTCGAAGCCAAATCACATTGATAAACCTGGAATATTATGACCAAGAGACAATCAAATTTATTGCCATC
ACAAAATTTTATGAATCTCTGTACATGCATGATGATCATAAATCAAGTTTAGACGAAGATTTGAGCTGTCTGTTA
AGCTTCCAGATAAAAGATTTCAAGTTATTCCATTTTTTGAAAAAAATGTATTACTCAAGACATTCGCTTCTAGGT
CAGTCTTCATTCATGGTACCCGCTGCTGAAAACCTATCTCCGATACCTGCCTCTATTGATACGAACGACATTCCT
TTAATTGCTAACGATTTAAAATTACTGGAAACGCAAGCAAAATTGATAAATATTCTGCAAGGTGTTCCTTTCTAC
TTGCCAGTAAATTTAACCAAAATTGAAAGTCTGTTAGAAACCTTGACTATGGGCGTGAGTAATACAGTAGACTTA
TATTTTCATGACAACGAAGTCAGAAAAGAATGGAAAGACACTTTAAATTTTATCAATACCATTGTTTATACAAAT
TTTTTCCTTTTTGTTCAAAACGAATCCTCTTTGTCCATGGCAGTTCAACATTCTTCTAACAACAATAAGACCTCG
AACTCTGAAAGATGTGCAAAGGATCTGATGAAAATTATTTCTAATATGCACATTTTTTACTCAATAACATTTAAT
TTTATCTTCCCCATAAAGTCGATAAAGTCATTTTCAAGCGGCAATAATCGCTTTCATTCTAATGGTAAAGAATTT
TTATTCGCAAATCATTTTATTGAAATCTTACAGAATTTTATAGCAATCACATTTGCTATTTTCCAACGTTGTGAA
GTAATATTATATGACGAATTTTACAAAAATCTTTCAAATGAGGAGATTAATGTTCAATTGCTATTGATTCATGAC
AAGATTTTGGAAATTTTAAAAAAAATAGAAATTATCGTATCCTTTTTACGAGATGAAATGAATAGCAACGGAAGT
TTCAAATCTATTAAAGGTTTCAACAAGGTTTTGAATCTGATTAAATATATGCTGAGATTTAGCAAGAAAAAACAA
AATTTTGCGAGAAACTCTGATAACAATAATGTTACAGATTATAGTCAGTCGGCGAAGAACAAAAATGTTCTCTTG
AAATTCCCCGTTAGTGAACTGAACAGAATCTATTTAAAATTTAAGGAGATTTCAGATTTTTTAATGGAAAGAGAA
GTTGTCCAAAGGAGTATAATTATTGACAAGGATTTGGAATCTGATAATCTGGGTATTACTACGGCAAACTTCAAC
GATTTCTATGATGCATTTTATAATTAG

>YKL136W 0.72 Cold
ATGTATGGTATAATACTTAAGTTCACTACAACGGACGTTTATAGTCAGAATAAATCGAGGAGATCCTGTGACACT
ATATTGCCGCCCGAAACATCGGGATTGTTTTTACCACCAGAAGATATACCGTTTGAGTCGCCACCTAGTTTCAGC
TCCTTCAACCCTTGTACAGCCTTGGTATCATTTGTATTTATTTGTGTGGCATCCTCAGATGGTCCGAAATCAAAT
AAATCATCCAAATCGCCTAAAGTTGTTAAAGTATTCAGCATTCCTGCATTTGTATTTGTTACATCATCTCTTTCA
TCAAAATCCAATAGCACATCATCGTTAGCCTTGCTAGAAATTTCATTTTTTGCCATGCTTTCTAATTCCTCTATA
TGTTTTCCTTTAACTATATTTTGA

>YJL003W 0.81 Cold
ATGTCGTTCAGCGGAAAAAAAATTTAGGTCTAGAAGACAGCAGCTAGTATATGAAGCATCTTTAGCGGGGCGATAC
AAAAAAGCATTGAGTAAGCACCCCTTTCTTTTTTTTGGTTTACCATTTTGTGCAACAATAGTATTGGGTTCGTTT
TGGTTGTCAAGCTTTACAGCGATCAAGTACGAGCAAGGCGATCGTAAAGTTCAGGAAATTAATGAAGAGGATATC
TTGAAAATAAGGAAGAATCAAAGGGAATTTGATATTAAAGAAGAATATTATCGTTTACAAGGTCTTTCTGAAGAG
GATTGGGAGCCTGTGCGCGTCGCTAGGTTAAAGGATGAATCTGAGAATGTCTGGTAA

>YGR029W 0.82 Cold
ATGAAAGCAATAGATAAAATGACGGATAATCCACCACAAGAAGGCTTAAGTGGGAGGAAAATAATATATGACGAA
GATGGCAAACCTTGCCGATCATGTAACACCCTACTTGACTTTCAGTACGTGACCGGGAAGATATCTAATGGCCTG
AAGAACCTCTCATCTAACGGTAAACTAGCAGGTACGGGGGCTCTCACTGGCGAAGCTTCAGAGTTGATGCCTGGC
TCAAGAACATACAGGAAGGTTGACCCTCCTGACGTAGAGCAACTAGGTAGATCTTCATGGACGCTGTTACACTCT
GTAGCTGCCAGCTATCCTGCTCAACCTACAGACCAACAGAAGGGTGAAATGAAACAGTTCTTGAATATCTTCTCA
CATATTTATCCTTGCAACTGGTGTGCTAAAGACTTTGAAAAATATATCAGAGAAAATGCACCACAAGTTGAGTCA
AGAGAAGAACTTGGGAGGTGGATGTGTGAAGCCCACAATAAAGTCAATAAGAAATTGAGGAAGCCCAAATTTGAC
TGTAATTTCTGGGAAAAAAGATGGAAGGACGGCTGGGACGAATAA

>YNR007C 0.73 Cold
ATGATTAGATCTACACTAAGTAGTTGGAGAGAATATCTTACCCCCATAACGCACAAATCTACCTTTTTAACCACA
GGTCAAATAACTCCTGAGGAGTTTGTACAAGCAGGTGATTATTTATGTCATATGTTTCCCACCTGGAAGTGGAAC
GAAGAGTCGTCAGATATTAGTTACAGAGATTTTTTACCGAAGAATAAGCAGTTTCTGATAATTAGAAAAGTTCCC
TGTGATAAACGTGCCGAGCAATGTGTCGAAGTTGAAGGGCCAGATGTAATCATGAAAGGTTTTGCAGAAGATGGA
GATGAAGATGATGTTCTGGAATACATAGGATCTGAAACTGAACATGTGCAAAGTACGCCTGCGGGGGGGACCAAG
GACTCATCTATCGATGATATTGATGAGCTAATACAAGACATGGAAATAAAAGAGGAGGATGAAAATGACGATACA
GAAGAATTTAATGCTAAAGGTGGCCTAGCCAAAGATATGGCGCAAGAAAGGTATTACGACCTTTATATTGCGTAC
TCGACATCTTATAGGGTCCCTAAAATGTATATAGTGGGGTTTAATTCTAATGGTTCACCACTAAGCCCTGAGCAG
ATGTTCGAAGATATATCAGCAGATTATAGAACAAAGACAGCCACCATTGAAAAGCTACCTTTTTACAAGAATTCA
GTGTTATCTGTTTCCATTCATCCATGTAAGCATGCTAATGTAATGAAAATATTGCTAGATAAGGTTCGTGTGGTT
AGACAACGAAGAAGGAAAGAGCTGCAGGAAGAACAAGAGCTGGACGGTGTCGGAGACTGGGAGGATTTACAAGAC
GATATTGATGATTCGTTACGGGTAGACCAATACTTGATTGTTTTCTTAAAGTTTATTACTAGTGTTACACCGAGT
ATACAACATGACTATACCATGGAAGGTTGGTAA

>YCR028C 0.78 Cold
ATGATGAAGGAATCGAAATCTATCACTCAACATGAGGTTGAGAGAGAATCTGTTTCTTCCAAGCGTGCCATTAAA
AAGAGATTACTTCTGTTTAAAATAGACTTGTTTGTGCTATCATTTGTTTGCTTGCAATACTGGATTAATTATGTC
GACCGTGTCGGTTTCACCAATGCATATATATCCGGTATGAAGGAAGATCTTAAGATGGTCGGAAACGATTTGACC
GTGTCTAACACAGTTTTCATGATTGGTTACATTGTAGGTATGGTCCCCAATAATTTAATGTTATTGTGTGTTCCG
CCCAGGATATGGCTAAGTTTTTGTACGTTTGCTTGGGGTTTATTGACCTTGGGAATGTACAAAGTTACATCGTTC
AAACATATTTGCGCAATTAGATTCTTTCAAGCCTTATTTGAGAGTTGCACATTTTCAGGAACACATTTTGTTTTG
GGTTCGTGGTATAAAGAAGACGAATTGCCCATTAGAAGTGCTATTTTTACAGGTAGCGGTTTGGTGGGATCTATG
TTCAGTGGATTTATGCAAACAAGTATCTTTACTCATTTGAATGGGCGGAATGGCTTGGCGGGTTGGAGATGGTTA
TTCATTATTGATTTTTGTATCACATTACCCATTGCAATTTATGGGTTTATTTTCTTCCCCGGCCTTCCTGATCAA
ACAAGTGCTGTTAGCAAATTTTCTATGACGAGATACATTTTTAATGAACAAGAGCTACATTATGCTAGGAGAAGG
CTCCCCGCTAGGGACGAAAGCACCCGGTTAGACTGGTCGACTATTCCTAGAGTCTTAAAAAGGTGGCACTGGTGG
ATGTTCTCTCTTGTTTGGGTTCTGGGAGGTGAGAACTTGGGTTTCGCATCTAATTCTACATTTGCATTATGGTTA
CAAAACCAAAAATATACGTTGGCGCAAAGAAATAATTATCCTTCGGGGATATTTGCCGTAGGTATAGTTTCTACG
CTTTGTTCTGCTGTATATATGAGTAAGATCCCAAGAGCTAGGCATTGGCATGTTTCTGTTTTCATATCATTGGTA
ATGGTTATTGTTGCGGTACTAATACGTGCAGACCCACTAAATCCAAAAGTCGTCTTTTCTGCACAGTATCTTGGA
GGCGTAGCATACGCTGGACAAGCGGTTTTTTTTTCGTGGGCAAACATTATTTGTCATGCAGATCTTCAAGAACGT
GCTATCGTTCTTGCTTCCATGAATATGTTTTCAGGGGCCGTTAACGCATGGTGGTCTATATTATTCTTTGCTTCA
GATATGGTGCCCAAGTTTGAGAGAGGTTGCTACGCCCTCTTGGCTACGGCAATATCAAGCGGAATTGTCTCGGTC
GTCATACGCTCACTACAGATAAAAGAGAATTTGTCTAAGAAACAGGTTCCTTATATAGATGCTAATGACATGCCC
GGAGAAGATGACGATGACGACAACCAGGATAATGAAAATGATGGCGACGACGAGAGTATGGAAGTTGAACTTCAT
AATGAGGAAATGGCCGAAATTTCAAATCCTTTCCGATAG

>YKL057C 0.8Cold
ATGGCATGCCTCTCAAGAATTGATGCAAATTTGCTCCAATATTATGAAAAACCTGAACCCAATAATACGGTTGAT
TTATACGTCAGTAATAACAGTAATAATAACGGGCTGAAAGAAGGAGATAAGTCCATCTCTACACCAGTTCCTCAG
CCTTACGGAAGTGAATATTCCAATTGTTTGTTATTGAGTAACTCTGAGTATATCTGTTACCATTTTTCCTCAAGA
TCAACATTGTTGACATTTTATCCTTTGAGTGACGCATACCATGGGAAGACAATTAATATACACTTACCAAATGCT
TCTATGAATCAGAGATACACTTTAACGATACAAGAAGTGGAACAGCAATTACTGGTTAATGTTATCTTAAAAGAT
GGTTCGTTTTTAACGTTACAACTACCCCTAAGTTTCCTATTTTCATCGGCCAACACTCTCAATGGAGAGTGGTTT
CATCTCCAAAATCCCTACGACTTTACAGTTAGAGTTCCTCATTTCCTTTTCTATGTATCACCCCAGTTCTCAGTT
GTTTTTTTGGAAGATGGCGGTTTGTTGGGTTTAAAAAAAGTTGACGGCGTACATTACGAGCCTTTACTATTCAAT
GATAATTCATACTTGAAAAGCCTAACGCGATTTTTTTCTAGGAGTTCTAAATCGGATTACGATAGTGTGATTAGT
TGTAAATTATTCCATGAAAGATACTTAATTGTGTTGACTCAGAATTGCCACTTGAAAATTTGGGATTTAACTTCA
TTTACTTTAATACAAGATTATGATATGGTTTCACAGTCAGATTCAGACCCAAGCCATTTTAGAAAAGTGGAAGCT
GTAGGGGAATACCTTTCCTTGTATAACAATACTCTAGTTACATTGTTGCCTTTGGAAAATGGGCTTTTTCAAATG
GGCACCTTGTTGGTTGATTCAAGCGGTATTTTGACTTATACTTTTCAAAATAACATCCCCACTAATCTTTCAGCT
TCTGCTATTTGGTCAATTGTAGATTTAGTTCTAACGAGACCATTGGAGTTGAATGTCGAGGCTAGTTATTTAAAT
TTGATCGTTTTATGGAAAAGTGGCACTGCTAGCAAGTTACAGATACTAAACGTAAATGATGAAAGTTTCAAAAAC
TATGAATGGATAGAATCAGTTAACAAATCCTTAGTAGATTTGCAATCCGAACATGATTTGGATATCGTTACAAAG
ACTGGAGATGTTGAGAGGGGTTTCTGTAATTTGAAATCAAGATATGGTACCCAAATTTTTGAACGTGCACAGCAA
ATATTGAGTGAAAATAAGATTATTATGGCTCATAACGAAGATGAAGAATATTTGGCTAATTTAGAAACAATATTG
AGAGACGTAAAAACCGCATTCAACGAGGCTTCTTCTATAACACTTTATGGCGATGAAATTATTCTCGTCAATTGT
TTCCAACCGTATAATCATTCCCTTTACAAGCTGAATACTACTGTGGAGAATTGGTTTTATAATATGCACTCTGAA
ACTGATGGTTCTGAATTGTTCAAGTATTTAAGGACATTGAATGGTTTTGCTTCTACTTTGTCAAATGATGTTCTT
AGATCCATTTCTAAGAAGTTTCTTGATATAATAACAGGAGAGCTGCCTGATTCTATGACTACCGTAGAAAAATTT
ACGGACATTTTCAAAAATTGCCTAGAAAATCAATTTGAAATCACAAATCTAAAGATTCTTTTCGACGAATTAAAC
TCTTTTGATATACCCGTTGTTTTAAATGATTTAATCAATAACCAAATGAAGCCTGGTATATTTTGGAAAAAGGAT
TTCATCTCTGCAATCAAATTCGATGGTTTCACAAGTATAATTTCCCTAGAATCTCTTCACCAGTTACTTTCCATA
CATTATCGTATTACTTTACAAGTATTGTTAACATTCGTTCTGTTCGATCTTGATACCGAAATTTTCGGACAGCAT
ATATCTACATTATTGGATTTGCATTATAAGCAATTTTTATTGTTAAACCTTTATAGACAAGATAAGTGTCTGTTG

GCGGAAGTTCTTCTCAAGGATTCTTCTGAATTCTCTTTCGGTGTCAAATTTTTTAACTACGGCCAACTCATTGCC
TATATCGATTCCTTAAACTCTAATGTTTACAATGCTTCCATCACTGAAAATTCTTTCTTTATGACGTTTTTCCGC
TCATATATCATTGAAAACACATCTCATAAGAATATAAGGTTCTTTCTCGAGAACGTTGAATGCCCCTTCTACTTA
CGCCACAACGAGGTGCAAGAATTTATGTTTGCCATGACGTTATTTAGTTGTGGTAATTTCGATCAATCCTATGAA
ATTTTTCAACTACACGATTATCCGGAGGCTATCAATGATAAATTGCCGACATTCTTAGAAGACCTTAAGAGTGAA
AACTATCACGGTGATAGTATATGGAAAGATCTTTTGTGCACCTTTACAGTTCCATATAGGCATTCTGCATTCTAT
TATCAATTATCTCTCTTATTTGATAGAAATAACAGCCAAGAATTTGCTTTGAAATGTATATCTAAGTCAGCAGAA
TATTCATTAAAAGAAATTCAGATTGAAGAACTGCAAGACTTCAAAGAAAAACAGCATATTCATTATTTAAATCTT
TTAATCCACTTCAGAATGTTTGAAGAAGTCTTGGATGTTTTGAGATTGGGTCACGAGTGTCTTTCCGACACAGTA
AGAACAAATTTTCTCCAATTATTATTACAGGAAGACATATATTCACGAGATTTTTTCAGCACACTTCTGCGCTTG
TGTAACGCACATTCAGACAATGGCGAGCTATATCTTCGTACAGTAGATATTAAAATAGTGGATAGTATTCTGTCA
CAAAATCTACGGAGTGGTGATTGGGAGTGTTTTAAGAAACTTTATTGTTTTAGAATGTTGAACAAAAGCGAGAGG
GCTGCTGCAGAAGTACTGTACCAGTACATATTAATGCAAGCTGATCTTGATGTTATTCGAAAGAGAAAATGTTAC
CTAATGGTCATCAACGTTTTATCTAGCTTTGATAGTGCATACGATCAATGGATTCTAAACGGAAGTAAAGTGGTT
ACTTTAACTGATTTAAGAGATGAGTTACGAGGTCTATAG

>YDL194W 0.75 Cold
ATGGATCCTAATAGTAACAGTTCTAGCGAAACATTACGCCAAGAGAAACAGGGTTTCCTAGACAAAGCTCTTCAG
AGGGTGAAGGGCATAGCACTGCGACGAAACAATAGTAACAAAGATCATACAACAGATGATACGACAGGTAGCATA
CGAACCCCTACGAGCTTGCAGCGGCAAAATTCTGACAGGCAATCTAATATGACATCCGTGTTTACGGATGACATT
TCTACCATAGACGACAACTCAATTTTATTTTCAGAGCCTCCTCAGAAACAATCTATGATGATGTCTATATGCGTA
GGTGTTTTTGTTGCAGTTGGCGGATTTTTATTTGGTTATGATACAGGTCTGATCAACAGTATTACATCTATGAAC
TATGTGAAGTCACACGTAGCACCTAATCACGATTCATTTACCGCCCAACAAATGTCCATTTTGGTGTCATTTTTG
TCATTGGGAACTTTTTTTGGGGCTTTAACTGCACCATTTATATCTGATTCGTATGGCAGGAAGCCTACTATCATT
TTCAGTACAATTTTCATCTTCTCTATCGGAAATTCTTTACAGGTAGGTGCTGGAGGAATCACATTATTGATTGTG
GGAAGGGTCATTTCAGGTATCGGTATAGGCGCAATTTCAGCGGTTGTTCCATTATACCAAGCAGAAGCTACACAT
AAATCATTAAGAGGTGCTATTATTTCTACTTACCAATGGGCCATTACCTGGGGCTTGCTCGTGTCAAGTGCAGTG
TCGCAAGGGACACACGCAAGAAACGACGCATCTTCGTATCGGATTCCCATAGGGTTGCAATATGTCTGGTCGTCA
TTTCTCGCTATCGGGATGTTCTTTCTCCCTGAGAGTCCACGCTATTACGTTTTGAAAGACAAGCTAGATGAAGCA
GCTAAATCTTTATCGTTTTTAAGAGGTGTACCAGTCCATGATTCTGGGTTACTGGAAGAACTAGTTGAAATAAAG
GCAACATATGATTACGAGGCATCTTTTGGTTCTTCGAACTTCATTGATTGTTTTATTTCAAGTAAAAGTAGACCA
AAGCAAACTCTAAGGATGTTTACGGGAATTGCCCTTCAAGCATTTCAACAATTTTCAGGTATCAACTTTATATTT
TACTACGGTGTCAATTTCTTCAATAAGACAGGAGTCAGTAATAGTTATCTGGTTTCATTTATAACCTATGCTGTT
AATGTTGTCTTTAATGTTCCTGGTTTGTTTTTTGTGGAATTTTTTGGTAGACGTAAGGTGCTGGTTGTTGGGGGT
GTTATCATGACTATAGCCAACTTTATTGTGGCCATTGTTGGGTGTTCCTTAAAGACTGTAGCGGCCGCAAAAGTT
ATGATAGCATTTATATGTCTATTCATAGCTGCCTTTTCTGCTACATGGGGTGGTGTTGTTTGGGTTATTTCAGCA
GAACTGTACCCATTGGGTGTGAGATCTAAATGTACGGCTATATGCGCTGCTGCTAACTGGCTTGTAAACTTTATT
TGTGCTTTAATTACCCCTTATATTGTAGATACTGGGTCGCATACATCATCATTAGGTGCAAAAATATTCTTCATT
TGGGGCTCCTTAAATGCGATGGGGGTGATAGTTGTTTACTTGACCGTTTATGAAACGAAGGGTTTGACATTAGAA
GAGATTGATGAATTATATATTAAGTCATCCACTGGTGTCGTGTCACCAAAATTTAATAAAGATATTAGGGAACGC
GCACTTAAATTCCAATACGATCCTTTGCAAAGATTAGAAGACGGAAAGAACACTTTTGTTGCTAAAAGAAATAAT
TTTGACGATGAAACACCAAGAAATGATTTTCGAAATACGATATCGGGCGAAATAGATCATAGTCCCAATCAAAAA
GAAGTTCATTCTATCCCAGAACGTGTTGATATTCCTACTAGTACAGAAATTCTTGAAAGCCCGAACAAAAGTAGT
GGTATGACAGTCCCTGTGTCACCTTCTCTGCAAGACGTTCCAATCCCGCAAACAACAGAGCCTGCTGAAATTCGA
ACCAAATATGTGGACCTAGGAAATGGGCTTGGTCTTAATACGTATAATAGAGGGCCTCCCTCACTCTCAAGCGAC
TCAAGCGAAGATTACACAGAAGATGAAATAGGCGGGCCCTCATCTCAAGGCGACCAAAGTAATAGAAGTACTATG
AATGATATTAATGATTATATGGCACGTCTCATTCACAGTACTTCTACTGCAAGTAACACGACAGATAAGTTCTCC
GGTAACCAAAGTACCCTTCGTTACCACACGGCTTCCTCACATTCGGATACAACTGAAGAGGACAGCAATTTGATG
GACCTGGGAAACGGGCTTGCCTTGAATGCTTATAACAGAGGTCCACCTTCAATTTTAATGAATTCCAGTGATGAA
GAGGCAAATGGTGGTGAGACGTCTGATAATTTGAACACAGCTCAAGACTTGGCTGGTATGAAGGAACGAATGGCG
CAGTTTGCGCAGAGCTATATTGACAAGAGAGGCGGTCTGGAACCTGAAACTCAATCTAATATTTTGAGCACTTCT
CTCTCCGTGATGGCTGACACTAATGAACATAATAATGAAATCCTCCACTCAAGCGAAGAAAACGCCACTAATCAA
CCTGTAAATGAAAATAATGATTTGAAATAA

>YNL229C 0.81 Cold
ATGATGAATAACAACGGCAACCAAGTGTCGAATCTCTCCAATGCGCTCCGTCAAGTAAACATAGGAAACAGGAAC
AGTAATACAACCACCGATCAAAGTAATATAAATTTTGAATTTTCAACAGGTGTAAATAATAATAATAATAACAAT
AGCAGTAGTAATAACAATAATGTTCAAAACAATAACAGCGGCCGCAATGGTAGCCAAAATAATGATAACGAGAAT
AATATCAAGAATACCTTAGAACAACATCGACAACAACAACAGGCATTTTCGGATATGAGTCACGTGGAGTATTCC
AGAATTACAAAATTTTTTCAAGAACAACCACTGGAGGGATATACCCTTTTCTCTCACAGGTCTGCGCCTAATGGA
TTCAAAGTTGCTATAGTACTAAGTGAACTTGGATTTCATTATAACACAATCTTCCTAGATTTCAATCTTGGCGAA
CATAGGGCCCCCGAATTTGTGTCTGTGAACCCTAATGCAAGAGTTCCAGCTTTAATCGATCATGGTATGGACAAC
TTGTCTATTTGGGAATCAGGGGCGATTTTATTACATTTGGTAAATAAATATTACAAAGAGACTGGTAATCCATTA
CTCTGGTCCGATGATTTAGCTGACCAATCACAAATCAACGCATGGTTGTTCTTCCAAACGTCAGGGCATGCGCCA
ATGATTGGACAAGCTTTACATTTCAGATACTTCCATTCACAAAAGATAGCAAGTGCTGTAGAAAGATATACGGAT
GAGGTTAGAAGAGTTTACGGTGTAGTGGAGATGGCCTTGGCTGAACGTAGAGAAGCGCTGGTGATGGAATTAGAC
ACGGAAAATGCGGCTGCATACTCAGCTGGTACAACACCAATGTCACAAAGTCGTTTCTTTGATTATCCCGTATGG

CTTGTAGGAGATAAATTAACTATAGCAGATTTGGCCTTTGTCCCATGGAATAATGTCGTGGATAGAATTGGCATT
AATATCAAAATTGAATTTCCAGAAGTTTACAAATGGACGAAGCATATGATGAGAAGACCCGCGGTCATCAAGGCA
TTGCGTGGTGAATGA

>YNR073C 0.73 Cold
ATGACAAAATCAGACGAAACAACAGCTACCAGCTTGAATGCCAAAACTCTAAAGAGTTTTGAATCCACTCTTCCA
ATACCAACTTATCCAAGAGAAGGTGTTAAACAAGGTATTGTTCATCTGGGAGTCGGTGCATTCCACCGTTCCCAT
TTAGCTGTTTTCATGCACCGTCTGATGCAGGAGCACCACTTAAAGGATTGGTCCATATGTGGTGTTGGTTTAATG
AAAGCAGATGCACTTATGCGCGATGCCATGAAGGCCCAAGATTGCCTATACACCCTTGTGGAGCGTGGTATCAAG
GACACTAACGCTTATATCGTCGGTTCTATTACTGCTTACATGTACGCTCCCGATGATCCAAGAGCTGTTATTGAA
AAGATGGCCAATCCAGACACACACATTGTTTCTTTGACGGTCACAGAAAACGGTTACTACCACAGTGAAGCAACA
AACTCCTTAATGACAGATGCTCCCGAGATTATCAATGATTTGAACCACCCAGAAAAGCCAGATACTCTGTATGGG
TATCTATATGAGGCCCTGTTGTTGCGTTACAAGAGAGGTCTTACCCCATTCACTATTATGTCATGTGACAACATG
CCCCAAAATGGTGTCACAGTAAAGACCATGCTTGTTGCATTTGCCAAGTTAAAGAAGGATGAGAAATTCGCCGCC
TGGATTGAAGACAAGGTTACTTCTCCTAACAGCATGGTGGACCGTGTGACCCCACGTTGTACCGATAAAGAGCGT
AAATACGTTGCTGACACCTGGGGAATCAAGGATCAATGTCCCGTTGTCGCAGAACCTTTCATCCAATGGGTTCTT
GAAGACAACTTCTCCGATGGCCGTCCTCCATGGGAACTTGTTGGTGTTCAGGTCGTCAAGGATGTCGATTCCTAC
GAATTGATGAAGTTGCGTCTACTTAACGGTGGACATTCTGCTATGGGATATTTGGGATACTTGGCAGGCTACACC
TATATACATGAGGTTGTCAACGACCCAACTATCAACAAGTATATCCGTGTTTTGATGCGTGAGGAAGTTATCCCA
TTATTGCCTAAAGTGCCAGGTGTTGATTTCGAAGAGTACACTGCATCAGTGTTGGAAAGATTCTCCAATCCAGCT
ATTCAGGACACTGTCGCACGTATTTGTTTGATGGGCTCTGGTAAGATGCCTAAGTATGTTTTGCCATCAATTTAC
GAGCAATTGCGTAAACCAGATGGTAAGTACAAGTTATTGGCAGTATGTGTTGCTGGCTGGTTCCGTTACCTGACT
GGTGTAGACATGAATGGGAAGCCATTCGAAATCGAGGATCCTATGGCACCAACCTTGAAGGCAGCCGCAGTTAAG
GGTGGTAAAGATCCTCACGAACTGCTCAACATTGAGGTGCTTTTCAGTCCTGAGATTCGTGACAACAAAGAATTC
GTTGCACAGTTGACCCACTCCCTAGAAACAGTTTATGATAAAGGGCCAATTGCCGCTATTAAGGAAATTTTAGAC
CAAGTGTGA

>YLR264W 0.79 Cold
ATGGATTCTAAGACCCCAGTCACCTTAGCTAAAGTCATCAAAGTTTTAGGCCGTACCGGTTCTCGTGGTGGTGTT
ACCCAAGTTCGTGTTGAGTTCTTGGAAGACACTTCCCGTACCATTGTCAGAAATGTCAAGGGTCCAGTTAGAGAA
AACGACATTTTGGTCTTGATGGAATCTGAACGTGAAGCTCGTCGTTTGCGTTAA

>YGR006W 0.77 Cold
ATGGACCTAGATCTAGCCAGTATCTTAAAAGGTGAAATTTCTAAGAAGAAGAAAGAGCTGGCCAACTCTAAAGGT
GTTCAGCCACCATGCACTGAGAAATTCCAGCCACATGAATCCGCAAACATAGACGAAACACCGCGACAGGTAGAA
CAAGAAAGTACAGATGAAGAAAACCTGTCAGACAATCAGAGTGACGATATTAGGACCACCATTAGCAAATTAGAA
AATCGGCCAGAAAGGATACAAGAAGCGATAGCTCAAGACAAAACCATCTCTGTAATCATTGATCCATCACAAATT
GGTTCTACTGAAGGAAAACCTCTTTTGTCAATGAAATGTAATTTATATATTCACGAAATATTATCTCGTTGGAAG
GCGTCTTTGGAAGCTTATCATCCGGAGCTGTTCTTAGACACTAAAAAAGCACTCTTTCCACTCCTACTGCAACTA
CGAAGGAACCAGCTTGCCCCGGATTTACTTATTTCCCTTGCGACCGTTCTCTATCATTTACAACAACCTAAAGAA
ATAAATTTAGCCGTTCAGTCCTATATGAAACTCAGTATCGGCAATGTGGCCTGGCCTATTGGTGTTACTAGCGTA
GGCATTCATGCTCGTAGTGCACATTCGAAAATTCAAGGAGGCCGGAATGCTGCTAACATAATGATTGATGAAAGA
ACGCGATTATGGATTACCAGTATTAAAAGATTAATAACTTTTGAAGAATGGTATACCAGCAACCACGATAGCTTA
GCCTAA

>YLR252W 0.8Cold
ATGTCGTTGCAATATTTTGGAACACTTACTTATCTTATAAAAACTCAAAGGTTATGGAGAAAGACAAGGTACCTG
TTCATTATCCACCCGTGGTCGAATAACTGGATAAGAAATCCTGATTTTGAAAATATTTGGAATTCAACTAGCATA
TTTATCAGACCTTGTTCCTTGGGGTACTGGAACAGCATATACCTAATAGACTTTCGGCAGAGAGCAGACCAACTT
CATAAATATTTGAGTCACCAAAGTTGGCTAGCAATCTGCTTTTCCTTTACAGATGTGCTTGGTCAGGAACAATTT
ATTTAA

>YDR468C 0.72 Cold
ATGAACAACAGTGAAGATCCGTTTCAACAAGTTGTTAAGGACACCAAGGAGCAATTGAACCGCATAAACAATTAC
ATAACTCGTCACAATACTGCTGGTGATGACGATCAAGAGGAGGAAATACAAGATATTTTAAAGGATGTTGAGGAA
ACAATAGTTGATTTGGACAGAAGCATAATCGTAATGAAAAGGGATGAAAACGAAGACGTGAGTGGTAGGGAAGCA
CAAGTTAAAAATATAAAACAGCAACTTGATGCTCTGAAGTTGCGTTTTGATCGAAGAATACAGGAATCTACTCAA
ACAACTATTCCTCTAGAAGAGACGGTGGAAAATTCAACACTTAACACCAGCATGGCTGAGAACAATGATGGTGGT
ATGTCCAATCCGTTTCAGGAACAAATGTTAAGAGAACAAGATGTTCATTTAGATGGTATTCACAAGACAATGCAA
AATTTGCATATTCAAGCTCAAACAATGGGGGATGAATTAGAGAACCAGGGACAATTGTTGGATAATATGGACGAG
GGTATGGACGGTGTTGTAAATAAGCTGGCTAGAGGTCGTAGGCAATTGGAATGGGTCTACGAAAAAAATAAAGAA
AAATACGACGATTGTTGTATAGGACTTCTTATTGTCGTCTTGATAGTTTTATTAGTTTTGGCATTCATTGCTTGA

>YOR023C 0.75 Cold
ATGATGAGTCCCGCCCAAGATAAACTGCAGCACCAGCATCATAATCCTAATTCTTCCTCATCGTCATCCTCTAAG
ATGACGAACGTTTATCAGGTTACCACGCCAAAGTCTCCTCAGGATTTGGAAAACAACATGGATGAGCCCTTCAAA
ATGGACACCGCCACCAGTAATCCGGACAAGGATAGCGAAAATACCCAGCGGTTGAAGTACGAATGTGCCAAAGGG

GAAATACAGAATGTTTTGAACCTACATATAATGCTAAACCACAAGCATGTTAGGCACTTACGAAGAAATGTTCAA
AAGGTTAACGCAAAACTAGCTCTCTTGGAAACCCTGCATAAGGATACTGGTCTATTGAACAAAATCGAACGGACG
TATCAACTCAAAATAAAACAACATCAGCAGCATAGCGTGCTCGGCGGCCACTTTCACGACAGTACGGCTACGGAA
AATACGAACGCATCAAATTATAACCTGTCATACCCGGTTCTATCGGATTATAACATCAACTGTCAGCCTTTGTCT
TCGAGCAGCAACAGAAATCTGTCAACAACACGCATACCACACCACCATTATCATACTAGAAGCAAGAGTAACGGG
CTTTTGCTAGAACCTTCTGCCCTCCGTCCGGCAAATTCGAATATAATTGATTACAGGCTAACAGGTTCTAAATCT
CTATCAGAAGCCATAACCAAGCCCACTCCCGTCTCGCTTCCTCACTCTAATTCGGATGGCATTTCTTCACCTCGT
TCCTCCTCAATCTCCCCCTTAGACGAGCAGCCGGGGTTCCAGATACTCCCTTTTAAACCAAGTCAGATGCATCTT
AACCACAGACGTAATTACAGTAGCACTTGTTTGACGAGCAATAGCGGTATCATCGGTAAGACTGAAAATAATGAG
CCCATTTTTAGAAGATATGACGGCATTTTGGTTATAATTACGTGCTCCAAATGTGACCGTTCCGGTTTCACTTCC
GCGCAAGGTATCGTGAACCATACTCGACTAAAACATTCTAAACTATATTCAAGCCAACCATTGGCAGTTTTAAAC
AACCAAAAACTACTTCCTAATGATAAGCAGGATCCGGAAATTTTATCTAAGTTCAAGAAACTAAATTTGGACCCA
AACAAAGACTACCTACCCTCTGACATTGCAATCCCAAAACCACAATCACCAATCAACCACTCCGAGAACCATACG
CGGGCTCCAAAGACTGTCAAGAACACTCCTCATTTAGAAAAACTATACCAAAATAAAGAAGACTTCAAAAAACTG
ATTGACATGGTTAATGAAACTCCGGATGATTTGAATGAATATTTGAAGCAACGTGAAATTCAATTAAGATATCAA
AAGGAGCAAGAAGAGGAATCTTCCAAATCTGATGACGAAGCTTCTTACGTCCCATCACCGTCTCTCTCAGCAACG
GCTACAACAACAACGACTACGGACCCACCCTCTCCGCCAGTGCTATCCTCCTCCTTACAAAGAAAATTGCTGCGT
AAGAGAAAACTGAGCTTGAATAGCTCTACTCCCATGGAAGATTTACCTTTAAGAGAAAGGTTGAGAGCAAATCCC
ACTGACAAGAAACCAAGAAAAGCGGCCCTTTTGACTAATGAACTTGAGGGTCCTGATCCTGCAGCAAAATCATCA
TCTTATTACAATCTAAGGTCGAAATCAAGACTGCGTGGTTCTCATACATAG

>YDR304C 0.8 Cold
ATGAAGCTTCAATTTTTTTCCTTTATTACCTTATTTGCTTGTCTCTTCACAACAGCCATTTTTGCGAAAGAGGAC
ACGGCAGAAGATCCTGAGATCACACACAAGGTCTACTTTGACATTAATCACGGTGATAAACAAATTGGTAGAATT
GTTATGGGATTGTATGGTCTCACCACACCCCAAACCGTTGAAAACTTTTACCAGTTGACCATTTCCAGGGACCCT
AAGATGGGTTATTTGAACTCTATCTTCCATCGCGTTATTCCTAACTTCATGATTCAAGGTGGCGATTTCACTCAC
AGATCAGGTATTGGGGGTAAGTCTATCTTCGGAAACACGTTCAAAGATGAGAATTTTGATGTCAAACATGACAAA
CCAGGCAGATTGTCTATGGCCAATCGTGGTAAAAACACCAACGGATCCCAATTTTTCATCACCACCGTCCCATGC
CCATGGTTGGACGGTAAGCACGTTGTCTTTGGAGAAGTCTTGGATGGTATGGACGTAGTTCACTACATTGAAAAC
GTTAAGACCGACAGTAGAAATATGCCTGTAAAGGAAGTTATTATTGTGGAAAGTGGTGAACTGGAAACTGTTCCT
TTGGACAATAAAGACGCCGCCAAGCTACAGGAAGAGATCAAAGCGGAAGCTAGCGAAGCAGCCCACGATGAACTC
TAA

>YER061C 0.73 Cold
ATGTCAAGAAGAGTGGTTATCACAGGATTGGGCTGTGTAACGCCGTTGGGAAGATCATTAAGTGAGTCATGGGGG
AATCTGCTCTCTTCCAAAAATGGACTCACACCAATCACATCTTTGCCCAACTATAATGAGGACTACAAACTCAGA
GAAAAAAGTATCCCATCAACGATAACAGTGGGGAAGATTCCAGAGAATTTTCAAAACGAAAATTCAGCCATCAAT
AAACTGCTGTTCACTAGCCAGGATGAGAGAAGAACCTCAAGCTTTATCAAGCTAGCACTACGTACAACTTATGAA
GCGCTTCACAATGCTGGTCTCTTGAACCCAAATGATATAACCATCAATACATCTCTGTGCAATCTGGATCACTTT
GGTTGCCTGATAGGTTCTGGTATAGGATCCATTCAAGACATATACCAAACTTCTCTACAATTCCATAACGACAAT
AAAAGAATAAATCCATATTTCGTCCCTAAAATCCTTACAAATATGGCAGCTGGTAATGTTTCCATCAAGTTTAAC
CTTAGAGGATTATCCCATAGTGTTTCCACAGCATGCGCAACAGGTAATAACTCCATTGGCGATGCATTCAATTTT
ATTCGGTTAGGCATGCAAGACATCTGTGTCGCCGGTGCAAGTGAAACGAGTTTGCATCCGTTAAGTTTAGCAGGT
TTCATCAGAGCAAAGTCGATTACTACAAACGGGATCTCTAGACCCTTTGATACACAACGTTCTGGATTCGTACTT
GGTGAAGGATGCGGAATGATTGTCATGGAATCGCTAGAACATGCTCAAAAGAGAAATGCAAACATAATTTCTGAG
CTCGTGGGCTATGGTTTAAGCAGTGATGCCTGCCATATTACCTCCCCTCCTGCTGATGGAAATGGTGCCAAAAGA
GCAATAGAGATGGCTCTAAAAATGGCTAGATTAGAACCAACTGATGTTGACTACGTCAATGCACATGCTACATCA
ACTTTACTAGGCGATAAAGCAGAGTGTCTGGCAGTAGCCTCAGCACTCTTACCAGGAAGATCCAAAAGCAAGCCA
CTGTACATATCCAGTAACAAAGGTGCAATTGGCCATCTTTTAGGTGCACGTGGCGCCGTAGAAAGTATATTTACA
ATTTGTTCCTTGAAGGATGATAAGATGCCGCATACCTTAAACCTGGACAATGTTCTGACTCTAGAAAATAACGAG
GCCGATAAGCTACATTTCATAAGAGACAAACCTATTGTGGGAGCTAATCCGAAGTACGCATTATGCAACAGCTTC
GGATTTGGAGGAGTTAACACATCTCTTCTCTTCAAGAAATGGGAAGGGAGTTAA

>YGL142C 0.73 Cold
ATGGCTCACGAGGTTCATAGAATAAAACCTAAACTAGGACGGACCCAAATATTTTGGGTTTTTCTAGCCTTTAGA
GTACTGAATGCAGTTCTGACGAGAACCTTCTTTCAGGCTGATGAGTTTTGGCAAGCATTGGAGCCGGCACATTGG
AAGGCTTTCAAGTATGGTGAACTGACTTGGGAATGGAAATTCGGAGTTCGTAGCTATTTATTTCCGATGATTTTT
GAGCTGACTTATAGATTGGTTTCTCTATCGTCAATCCTTCTTCACTATGCACTTCTTCTTCTCTCAACGATAGGG
TCTGATTTATTAATTTTACTACTGCCGAAATATGAGTTAAGTTGGCAAGTCGCTGAAGACTTGAAGAGGCTTCCT
TTTGATGTCACACGGTCGTTCGAATATTATGGTGTGATATATGCGCCAAAAATAGTTATGGCAGTACTAGCTTCC
ATAGGGGAATATTATATCGTAAGATTTGTTCAAAAGCTCTATTTACTTACACTTGATAAGAGAAACGAGAAGGAG
GAAGAGGAGAGACGCAGCGGTTTATCCGAGATTACGAAGTTTGCACTGCTTTTAAGTTTAACTAATTTTTTTAAT
TGCTTTTTTATTACAAGAACGTTCATCAACTCTTTTGAAATGATTTTAACTTCTATTGCCCTCTACTACTGGGAC
TGGACAGGCGGACAAATGATAAAAGAGTCATCTTTTACTAAGTCGCTTATTTTTGCATTTCTGGCATGTTTGCAA
AGACCCAGCAGTGGACTTATTTGGGTAATTCCCAGCATTTCCCTAATATTAAACTTGGTAGGCAAAAAGCAGTAT
CATTTACTTTTCATCACATTTTCCAAAGTTTTACGGTCTTTTTTTCTGGTATTTACTGCAAATGCAATTATCGAC
ATGTATTTTTATGAAAAAGTAACGTTTCCCTTTTTCCGATTTCTAAAATTCAATTTCACAACACCATTATCAAAG

TTTTATGGGGTAGCTCCTTGGCATTTTCATTTCTTTCAAAGCTTACCTATCGTATTAGGTGCAAGTATTCCCGCT
TTTGCATTTGGATTATTTTTTCCACTAAGCAAAAGGAGTTTCCCAAAGAAGTATTTAAACCCGTTTTTCCAAGTG
AAACTCACTATCTTGTTGAATCTGTTGGTGTACTCTACGTTACCTCATAAGGAATTTAGGTTCATCTTCCCATTA
CAGCCACTTTTCATATTAATATCATCATTTGGCCTTTTGAGACTTGATAGAGATTATTGGAAAAGACTATCTGGC
CTAAAATCTTTACTCTGGCTTGTTCCATTCGTGTCGGTGTTCATCGCATTACTTTTGGACACATTCCACGAATCT
GGATCTATCGAAGTTATGAAATTTTTACATGAAGAGCCAGAAATAGATAGTTTGGGATTCATAATGCCCTGTCAT
TCGACACCTGGTCAAAGCTATTTACACCGCAGTGACATTCAAGATCTGTGGTCGATTACTTGTAACCCTCCATTG
CATTTACTTGGAGACCCAGAGGCTTACTCCAAACTAGAGACCTACATGGATGAAAGTGACCATTTGTATGACGAT
ATATCGGCATTCATTTACAAAAATTTCCCACCGCCTTTTCGTAAGGATCTAAGGAGTCCAGGTAAAACATACAGC
CACGAATGGCCAACATATTTGGTTGTTTTCGAGCACATGGAAAACGCATTTCTAAAGGATTTCCTGAAAGATTCA
TCATACATTGAATACAATAGATTCTTCAACTCTCTTGCGCACTGGGATTCAAGAAGATCAGGAGACATAATAATT
TATTATAAATTACCATTCGATTATAGCGATATTCCAGCGGCAGATATTTAA

>YNR070W 0.66 Cold
ATGGAATGCGTTTCAGTAGAAGGTTTGGATTCTTCTTTTTTGGAGGGCCAAACCTTTGGCGATATTTTGTGTTTA
CCATGGACAATTATCAAGGGTATCCGTGAGCGGAAGAATCGCAATAAGATGAAGATCATTTTGAAGAATGTCAGT
TTGCTGGCTAAATCAGGAGAGATGGTCCTTGTCCTAGGAAGACCAGGCGCTGGCTGTACATCATTTTTAAAGAGC
GCTGCTGGTGAGACCAGTCAGTTTGCAGGTGGTGTAACAACAGGACATATATCGTACGATGGTATCCCTCAGAAA
GAAATGATGCAACATTACAAGCCAGATGTAATCTATAATGGTGAGCAAGATGTTCATTTCCCACATTTGACAGTA
AAACAAACTCTAGATTTTGCTATTTCCTGTAAGATGCCCGCAAAAAGAGTCAATAATGTAACGAAAGAAGAGTAT
ATTACTGCCAATAGAGAATTCTATGCTAAAATTTTTGGTTTGACGCATACTTTCGATACCAAAGTTGGTAACGAT
TTCATCAGCGGTGTATCTGGAGGTGAGCGTAAACGCGTTTCCATTGCTGAAGCATTAGCAGCGAAAGGTTCAATT
TACTGCTGGGATAATGCTACAAGAGGTCTTGACTCTTCTACCGCGCTAGAATTTGCACGAGCTATTCGTACTATG
ACAAATCTGTTAGGTACAACGGCCCTTGTTACGGTTTACCAAGCCAGTGAAAACATTTATGAAACTTTTGATAAA
GTCACTGTTCTATACGCTGGAAGACAAATATTTTGCGGCAAAACTACTGAAGCAAAAGATTATTTTGAAAACATG
GGTTACTTGTGTCCACCGAGACAATCGACTGCTGAATATTTGACCGCAATTACTGATCCTAATGGTCTGCACGAA
ATAAAGCCTGGCTTTGAGTATCAAGTACCTCATACCGCTGATGAATTCGAAAAATACTGGCTTGATTCCCCAGAA
TATGCCCGCCTAAAAGGTGAAATTCAGAAGTACAAACATGAAGTGAATACTGAATGGACCAAAAAAACATACAAT
GAGTCTATGGCACAAGAAAAGTCGAAAGGTACAAGAAAAAAATCTTATTATACGGTCTCTTATTGGGAGCAAATT
AGACTTTGCACTATTAGAGGTTTCCTAAGGATTTACGGTGATAAGTCATACACCGTTATCAACACATGCGCTGCT
ATAGCACAGGCTTTTATCACTGGGTCATTGTTCTACCAAGCACCTTCTTCAACTCTAGGGGCCTTTTCTAGAAGT
GGCGTTCTGTTTTTTTCCCTCTTATATTATTCTTTGATGGGTTTAGCTAATATTAGTTTCGAGCACAGGCCAATA
TTGCAAAAACACAAGGTCTATTCACTATATCATCCCTCAGCTGAAGCGTTAGCAAGTACGATTTCTTCTTTTCCA
TTCAGAATGATTGGTCTAACATTTTTCATAATCATCCTGTACTTCTTAGCCGGTTTGCATAGAAGCGCCGGTGCT
TTTTTTACTATGTATTTGTTATTGACAATGTGTTCAGAAGCTATTACAAGTTTGTTTCAGATGGTTTCATCTTTA
TGCGATACATTGTCCCAGGCCAACTCCATTGCCGGTGTTGTGATGTTATCTATTGCCATGTATTCGACGTACATG
ATACAATTACCTTCAATGCATCCATGGTTTAAGTGGATTTCGTACATTCTACCCATTAGATATGCATTTGAATCG
ATGTTAAATGCAGAATTTCATGGAAGACATATGGATTGTGGTGGCACTTTGGTTCCTTCTGGACCTGGGTTTGAA
AACATCTTGCCAGAAAATCAAGTGTGTGCTTTTGTTGGTTCAAGGCCTGGCCAATCTTGGGTCCTAGGTGATGAT
TATTTGAGGGCCCAATATCAATATGAGTACAAAAATACTTGGAGAAACTTCGGCATCATGTGGTGTTTCTTAATT
GGCTACATCGTCTTGAGGGCGGTTTTCACTGAGTACAAAAGTCCTGTCAAAAGTGGTGGTGATGCTCTGGTCGTC
AAGAAGGGCACAAAGAATGCTATACAAAGATCATGGAGCAGCAAAAATGACGAAGAGAACCTTAATGCCTCTATA
GCAACACAGGATATGAAAGAGATAGCTTCAAGTAACGACGATAGCACAAGTGCAGACTTTGAAGGTTTGGAATCT
ACCGGGGTGTTTATTTGGAAAAATGTTTCTTTCACAATTCCTCATTCTAGCGGACAACGTAAACTTCTGGACAGC
GTAAGCGGTTACTGTGTTCCTGGTACTTTGACAGCATTAATAGGTGAGTCCGGCGCTGGTAAGACCACATTATTA
AACACTTTGGCTCAAAGGAACGTGGGAACGATTACTGGTGATATGTTAGTTGATGGTCTCCCAATGGACGCTAGT
TTCAAAAGGCGTACCGGTTATGTTCAACAGCAAGATCTTCATGTTGCTGAACTTACTGTCAAAGAATCGCTACAA
TTTAGTGCTCGTATGCGTCGGCCACAGTCTATTCCTGACGCTGAAAAGATGGAATATGTTGAAAAAATTATATCT
ATTCTTGAAATGCAGGAGTTCTCAGAAGCTCTTGTCGGCGAAATTGGTTACGGCTTGAATGTTGAACAGAGAAAG
AAACTATCAATTGGCGTTGAGCTAGTTGGCAAGCCGGATCTGTTATTGTTTTTGGACGAACCGACCTCTGGCTTG
GATTCCCAATCCGCGTGGGCCGTCGTCAAAATGTTAAAAAGATTAGCTCTAGCAGGTCAATCAATTTTATGTACT
ATTCATCAACCATCAGCTACTCTTTTTGAACAGTTTGACAGATTATTGCTTTTGGGAAAAGGCGGTCAAACAATT
TATTTTGGTGAAATAGGTAAAAATTCAAGTTCTGTCATTAAGTATTTCGAAAAGAACGGAGCCAGGAAATGTCAA
CAAAATGAAAATCCGGCAGAGTATATTTTAGAAGCCATAGGAGCTGGGGCCACCGCTTCTGTTCAACAGAACTGG
CCTGATATATGGCAAAAATCTCACGAGTATGCAAACATTAACGAAAAAATAAATGACATGATTAAGGACTTATCT
TCTACAACTTTGCATAAAACAGCTACAAGGGCCTCTAAGTATGCAACATCGTATTCCTACCAATTCCATCATGTG
CTGAAAAGATCCAGTTTAACATTTTGGAGAAACTTGAACTACATCATGGCCAAAATGATGTTATTGATGATCAGT
GGCTTGTTCATTGGTTTTACCTTTTTCCATGTGGGTGTAAATGCTATTGGATTACAAAATAGCTTATTTGCCTGT
TTCATGGCTATCGTTATATCAGCTCCTGCAACAAACCAAATACAGGAGCGTGCTACCGTTGCTAAGGAGCTATAT
GAAGTTCGTGAGTCCAAATCTAATATGTTTCATTGGTCTTTACTTTTGATTACCCATTATTTAAATGAATTGCCT
TACCATTTATTGTTTTCAACAATTTTTTTCGTTTCATCATATTTCCCTCTGGGTGTCTTTACCGAAGCCTCTAGG
TCAAGTGTTTTTTATCTGAACTATGCCATACTTTTTCAACTTTACTATATTGGTCTTGCTTTAATGATTCTGTAC
ATGTCTCCAAATCTACAATCTGCCAATGTTATTGTAGGTTTTATACTTTCGTTTTTGCTCTCTTTCTGCGGTGCT
GTCCAACCTGCCTCTTTAATGCCTGGTTTCTGGACATTCATGTGGAAACTATCCCCTTACACGTATTTTTTGCAA
AATTTAGTTGGATTATTGATGCATGACAAACCCGTAAGATGTTCAAAGAAAGAGTTGTCTCTTTTCAACCCCCCG
GTAGGCCAAACATGTGGTGAATTTACCAAACCGTTTTTTGAATTTGGGACTGGGTATATTGCAAATCCAGATGCA

ACAGCAGATTGCGCCTATTGTCAGTACAAAGTAGGTGATGAATACTTGGCGCGCATAAATGCTAGCTTTAGTTAC
TTATGGAGAAACTTCGGTTTCATTTAG

>YHR168W 0.82 Cold
ATGTCGATAGCTTGGTCTAGCGTTTTCAAAAGAGAATTACGGCTTGAAAGATTTTTACCAAGAGTATATTCGACG
AAAGTTCCTGATAATGCACCAAGAGCGGCTGACAATGAACAATGGCTAGAAACCCTAAGACCTATAACTCACCCA
GAACAGAAAAAGTCCGACCATGATGTCAGTTACACAAGACACATTAATGTTCCTCTGGGCGAAGTCACTTCTGTC
AACTATCTACAACGGTATAATAAGCATAAGCATAGCCAGGGCAATTTCGTGGATGTTCGAATAGTGAAATGCAAA
AGTGGCGCTGGTGGAAGTGGAGCTGTCTCCTTCTTTAGGGATGCAGGGAGGTCTATAGGTCCTCCGGATGGTGGA
GATGGGGGAGCTGGTGGTAGTGTTTATATTCAAGCTGTTGCGGGGTTGGGATCTCTGGCGAAGATGAAGACCACA
TATACCGCTGAAGACGGAGAAGCAGGTGCTGCCAGGCAACTGGATGGCATGCGAGGAAGAGATGTTTTAATACAA
GTTCCTGTGGGTACTGTTGTAAAATGGTGCTTACCACCCCAAAAAGTTCGAGAGCTCGTGGAAAGAGAAATGCGC
AAAGACAATAATGCTACATTAAGGTCTATACTTGGCTCAACAGCCGTCAACTTAAGCGTTTCTTCTGGTTCTCAT
CGGAAAAAAATACAACTATACAGACATGAAATGGCGGAGAGTTGGCTCTTCAAGGACAAGGCCAAGGAATACCAT
GAGAATAAGGACTGGTTCAAAGATTTACATAAGAAAATGGAGGCATACGACCACTCGTTAGAACAATCTGAACTG
TTTAATGATCAGTTTCCACTGGCTGGTCTTGACTTAAATCAGCCAATGACGAAACCAGTATGTCTGCTGAAAGGT
GGCCAAGGTGGACTCGGTAACATGCACTTTTTAACAAATTTGATTAGAAATCCAAGATTTTCCAAACCCGGCAGG
AATGGGTTGGAACAGCATTTTTTATTCGAATTGAAAAGCATTGCTGATCTTGGACTCATTGGTTTGCCTAACGCT
GGAAAATCAACTATACTAAACAAAATCTCAAATGCAAAACCGAAAATAGGGCACTGGCAATTCACCACACTGAGC
CCTACGATTGGCACAGTTTCCTTGGGTTTTGGGCAAGACGTCTTCACTGTGGCCGATATACCTGGAATAATACAG
GGAGCATCATTAGACAAAGGGATGGGTCTTGAGTTTCTAAGGCATATAGAACGTTCCAATGGTTGGGTATTTGTC
CTTGACCTCTCTAATAAAAACCCTTTGAATGACTTACAATTACTGATAGAGGAAGTTGGTACTTTAGAGAAAGTC
AAGACCAAAAACATACTAATTGTATGTAATAAAGTCGATATTGATGCCGAGAAATCCGAATCATTTGCCAAGTAC
CTACAGGTTGAGAAATTTTCCAAAAGTCAGGAATGGGACTGTGTTCCGATAAGCGCCCTCAGAGAGAAAACATAG

>YOR124C 0.78 Cold
ATGCCGAACGAAGATAATGAACTTCAAAAAGCAATTGAGAACCATCATAATCAACTACTAAACCAGGATAAAGAA
AATGCTGACAGAAATGGGTCTGTTATAGAAGACCTCCCATTATACGGGACAAGTATAAACCAGCAGTCTACCCCT
GGAGATGTTGACGATGGAAAACACTTACTGTATCCAGATATTGCCACCAACCTACCACTGAAGACTTCTGACAGA
CTTTTGGACGATATACTTTGCGATACTATTTTTCTCAATTCTACAGACCCGAAGGTCATGCAAAAGGGCCTGCAA
TCGAGGGGTATTTTAAAAGAGTCTATGCTTTCTTACTCAACTTTCAGAAGTAGTATTCGCCCTAACTGCTTGGGT
TCATTAACTGATCAAGTGGTTTTTCAAACAAAATCCGAGTATGATTCCATTTCATGCCCAAAATATAATAAAATA
CATGTATTTCAGGCGGTCATCTTTAATCCATCACTGGCAGAACAGCAAATTTCAACTTTTGATGATATTGTTAAA
ATTCCTATTTATCATCTTAAGGTTAGCGTAAAAGTCCGCCAAGAACTGGAGCGGTTGAAGAAGCATGTCGGTGTT
ACTCAATTCCACTCACTAGATCATTTGCACGAATACGATCGAGTAGACCTTTCGACTTTTGATTCTTCCGATCCT
AATTTGTTGGATTACGGTATTTACGTTTCTGATGATACTAACAAACTGATCTTGATTGAAATTTTTAAACCCGAG
TTTAATTCACCTGAAGAGCATGAGAGTTTTACTGCCGACGCAATTAAGAAGAGATACAATGCTATGTGTGTAAAA
AATGAATCACTAGATAAAAGCGAGACGCCATCTCAAGTTGACTGTTTTTACACACTTTTTAAAATTTTTAAAGGG
CCTTTGACGAGGAAAAGTAAAGCGGAACCTACAAAGACAATTGATTCTGGAAATTTGGCCCTTAACACTCACCTG
AATCCTGAATGGTTAACGTCCAAGTATGGATTTCAAGCAAGCTCAGAAATCGATGAGGAAACTAATGAGATATTT
ACTGAATACGTCCCTCCAGATATGGTGGACTATGTAAACGATTTGGAGACAAGAAAAAATTCGAGAATCGTTTGTG
AGGAAGTGTTTACAACTGATATTTTGGGGTCAACTATCTACCTCATTACTGGCACCTAATTCTCCCTTGAAAAAT
ACGAAAAGCGTAAAGGGAATGTCTTCATTACAAACTTCTTTCTCAACACTACCTTGGTTCCATTTATTGGGAGAA
TCCAGAGCAAGGATTCTATTAAATTCCAATGAGCAAACTCATTCGCCTTTGGACGCAGAACCTCATTTTATTAAT
CTTTCCGTTTCGCATTATTATACCGATAGAGATATAATCAGAAACTACGAATCTTTGTCTTCTTTGGATCCTGAA
AATATTGGGCTGTATTTTGACGCACTGACATACATTGCAAATAGGAAGGGGGCATATCAATTGATTGCTTACTGT
GGAAAACAGGACATTATAGGCCAAGAAGCTCTAGAAAATGCTTTGTTAATGTTTAAAATTAACCCTAAAGAGTGT
AACATCTCCGAATTAAATGAGGCGACTTTGCTATCTATTTACAAATATGAAACATCAAATAAGAGCCAAGTAACC
TCTAATCACCTAACAAATTTGAAAAATGCTCTAAGATTGTTGGCCAAATATACCAAATCTGACAAACTAAAATTT
TACGTCGATCATGAGCCCTACAGAGCTTTATCCCAGGCATACGACACACTTTCAATTGACGAGTCTGTTGATGAA
GACATTATAAAAACTGCATATTCGGTCAAGATTAACGACTCTCCCGGATTAAAGTTGGATTGTGATAGAGCACTT
TACACCATTGCTATCAGTAAAAGAAGCCTTGATTTGTTCAATTTTTTAACAGAGGAATGCCCACAGTTTTCCAAC
TATTATGGTCCAGAGAAGCTTGATTACCAAGAGGCATTGAAGCTTCTTCAAGTGAATGAAAATGCCTCTGACGAA
ACCATTTTGAAAATCTTTAAACAAAAGTGGTTTGATGAAAACGTTTATGAGCCTGACCAATTTCTTATTTTGAGG
GCAGCATTGACCAAAATCAGTATAGAAAGAAATTCAACTTTAATCACCAACTTCTTACTAACTGGTACGATAGAT
CCAAATTCCTTGCCGCCAGAAAATTGGCCAACTGGCATTAATAATATCGGGAACACCTGTTACCTAAATTCTTTA
TTACAATATTACTTTTCCATTGCGCCACTAAGAAGATATGTATTGGAATATCAAAAAACGGTAGAAAATTTCAAT
GACCACCTCTCTAATAGTGGGCATATTAGAAGAATTGGTGGAAGAGAAATTAGTAGAGGCGAAGTGGAAAGATCT
ATTCAATTCATATACCAACTTCGCAACCTTTTCTATGCGATGGTTCATACAAGAGAAAGATGTGTAACACCCCTCA
AAAGAGCTAGCATATTTGGCATTTGCTCCAAGTAATGTTGAAGTAGAATTTGAAGTGGAAGGCAATAAAGTAGTT
GATCAAACAGGAGTTCTTTCGGATTCAAAGAAGGAAACAACGGATGACGCATTTACTACAAAAATAAAGGATACA
AGCCTGATTGATTTAGAAATGGAAGATGGCCTTAATGGCGATGTTGGTACAGATGCGAACAGAAAAAAAAAATGAA
TCGAATGATGCTGAAGTAAGTGAGAACGAAGATACAACAGGATTAACTTCACCTACGCGTGTGGCAAAAATCAGT
TCTGATCAATTAGAAAATGCTTTGGAAATGGGTAGGCAACAAGATGTTACTGAATGCATAGGAAACGTGTTATTT
CAGATAGAAAGCGGTTCAGAGCCTATCCGATATGATGAAGACAACGAGCAATATGACTTGGTTAAGCAACTATTT
TATGGTACTACTAAACAAAGTATTGTTCCTTTGTCCGCAACAAATAAAGTCCGTACGAAAGTTGAAAGATTCCTA
TCGTTACTGATAAATATTGGCGATCATCCTAAAGATATTTATGATGCGTTTGATTCTTATTTTAAAGACGAATAT

CTGACAATGGAAGAGTATGGTGATGTTATACGTACCGTTGCTGTTACAACTTTTCCTACTATTTTGCAGGTACAA
ATCCAAAGAGTTTATTACGATCGTGAAAGATTAATGCCGTTTAAATCCATTGAGCCCTTACCATTCAAAGAAGTT
ATTTACATGGACAGATACGCGGATACAGAGAACCCTTTATTGTTGGCAAAAAAGAAAGAAACAGAAGAAATGAAG
CAAAAGTTGAAGGTAATGAAAAATAGACAAAGAGAGCTTTTGAGTCGTGATGATTCAGGGCTTACAAGGAAGGAT
GCATTTTTGGAGAGTATCAAGCTATTGGAATCGGATACCATAAAGAAAACTCCTTTAAAAATTGAGGCTGCTAAT
GATGTGATAAAGACGCTGAGAAACAACGTTCAAAATATCGATAATGAATTGATGAAATTATACAATGATATCAAC
AGTTTGGAAGAGAAAATAAGCCATCAATTTGACGATTTCAAGGAATATGGTTACTCACTGTTTTCGGTTTTTATT
CATCGCGGCGAGGCCAGTTATGGTCACTATTGGATATATATCAAGGACAGAAATCGCAATGGAATTTGGAGGAAG
TACAATGATGAAACCATCAGTGAGGTCCAGGAAGAGGAGGTCTTCAATTTCAATGAGGGTAACACTGCAACTCCA
TATTTCCTAGTATATGTCAAACAAGGACAAGAAGGTGATATTGAGCCATTGAAAAGAATTCTAAAGTAG

>YLR156W 0.81 Cold
ATGAAGTTCCAATATGCGTTGGCCAAGGAACAGCTAGGCAGCAACTCGCGCAGTGGCGTCAAAAAACTAATAAGT
AAACACCACTGGCTTCCGGAATACTATTTCTCTGATCTCTCATTTTCTGTTGTACAGCAGTGGGACAGTAGAGCC
ATTGAAAAAACTACAATCATATCTTGTATGCGGCCCGCAAACCAAGAGATTTATCCTTTGAGACATTGTGAGACC
CTCCGTTCGCAACCGTGCTCTCTGTTTTCATCACTATATGCACGCTCTTTCCAAAGCTCCTGCACTTTGCACGTG
GCGGAGCCATCGCCCGGCTTCCATATGTACGGCTGCCACACCTAA

>YBL024W 0.81 Cold
ATGGCTAGAAGAAAGAATTTCAAAAAAGGGAACAAGAAGACTTTTGGTGCTCGTGATGACTCGAGAGCTCAAAAA
AACTGGTCTGAACTGGTAAAGGAAAATGAAAAATGGGAAAAAATACTATAAGACTTTAGCTCTTTTCCCAGAAGAT
CAATGGGAAGAATTTAAAAAGACATGTCAAGCTCCACTTCCTCTAACTTTTAGAATTACAGGTTCTAGAAAGCAT
GCCGGTGAGGTCCTGAATTTGTTTAAAGAAAGACATCTACCAAACTTGACTAATGTTGAGTTTGAAGGTGAGAAG
ATTAAGGCCCCTGTAGAATTACCTTGGTATCCAGACCATCTTGCTTGGCAATTGGACGTTCCTAAGACGGTTATT
AGAAAGAATGAACAATTCGCAAAAACTCAGAGATTTTTAGTTGTTGAAAATGCCGTTGGTAATATCTCAAGACAA
GAAGCCGTTTCAATGATTCCTCCAATCGTTCTAGAAGTAAAACCTCATCACACTGTTTTAGATATGTGTGCTGCT
CCTGGCTCCAAAACTGCTCAATTAATCGAAGCCTTGCACAAGGATACAGATGAACCATCTGGTTTCGTTGTAGCT
AATGATGCCGATGCCAGAAGATCTCATATGTTGGTTCACCAATTGAAGAGATTGAACAGTGCCAACTTGATGGTT
GTCAACCATGACGCCCAATTCTTCCCACGTATCAGATTACATGGCAACTCAAATAACAAGAATGATGTTTTAAAA
TTTGACAGAATCCTGTGTGACGTTCCATGTTCTGGTGATGGTACCATGAGGAAAAATGTTAATGTTTGGAAAGAC
TGGAACACACAAGCAGGTCTTGGTTTGCATGCTGTTCAGCTGAATATATTAAACAGGGGTTTGCATCTTCTAAAG
AACAACGGTAGATTGGTTTACTCAACCTGTTCTTTAAATCCTATTGAAAATGAAGCGGTTGTTGCCGAAGCGTTA
AGAAAGTGGGGTGACAAGATTAGATTAGTTAACTGTGATGATAAGCTTCCTGGCCTAATAAGATCCAAGGGTGTA
TCCAAATGGCCTGTCTATGACAGAAATTTGACTGAGAAAACCAAAGGAGACGAAGGTACACTAGATAGTTTCTTT
TCACCATCTGAAGAAGAGGCATCGAAATTCAATTTACAAAATTGTATGAGGGTTTATCCTCACCAACAAAACACA
GGCGGATTTTTCATTACTGTTTTCGAAAAAGTCGAAGATAGCACTGAGGCGGCTACAGAGAAACTATCTTCTGAA
ACCCCAGCTCTAGAGTCTGAAGGACCTCAAACAAAGAAAATAAAGGTAGAAGAAGTCCAAAAGAAAGAAAGACTA
CCACGTGACGCGAACGAAGAGCCTTTTGTTTTCGTTGATCCACAGCACGAAGCTTTAAAAGTTTGTTGGGATTTC
TACGGCATCGATAATATTTTCGACAGAAACACTTGTTTAGTGCGTAACGCCACTGGTGAACCAACAAGAGTGGTT
TACACTGTGTGTCCAGCATTGAAGGATGTTATTCAAGCGAATGACGATAGGTTGAAGATTATTTATTCTGGTGTA
AAATTGTTTGTCTCTCAAAGAAGTGATATCGAATGTTCATGGAGAATCCAAAGTGAATCATTGCCAATAATGAAA
CACCATATGAAATCTAATAGAATTGTTGAAGCTAATTTAGAGATGTTAAAACACTTGTTAATCGAATCTTTCCCT
AACTTTGACGACATTCGTTCGAAGAACATCGATAATGATTTTGTTGAAAAGATGACAAAATTAAGCTCTGGTTGC
GCCTTTATTGATGTGTCAAGAAATGACCCTGCCAAAGAAAACTTATTCTTGCCTGTGTGGAAAGGCAACAAGTGT
ATCAATTTGATGGTTTGTAAAGAAGATACTCATGAGCTATTATATAGGATCTTTGGTATTGATGCGAATGCCAAG
GCTACTCCAAGCGCTGAAGAAAAAGAAAAAGAAAAAGAAACGACTGAATCTCCCGCAGAAACTACTACCGGAACC
TCTACTGAAGCTCCTAGCGCTGCTAATTGA

>YHR185C 0.78 Cold
ATGAATCAAGGTTACACACAGCTTTCCGCACCGGAACTGAAGGAGACTAAAACATCGAAGCTAAATAAAATGAAC
AACTTTCGAAGCTCTCCGATTGCTGAGATAATTAATAAGATACCACCAGATTGCGGGAAGATACAGAATACAACA
TTTCCTGAGTTCAATCCAGCGTTAAGAAGACGACAACACGAACAGTGGCCTGCATACGAAAAACCAATAAGAGTT
ACTGACAGTATGTCACCACAGCTTTCTTCAATCAATTGTTTGCCAAATTTGTATCCCCACGGTACGCTACCATTA
CCAAACCCTTATTTATCCTATTTAAATCACATTGAAAAAGTTAATTGCCAAGATGTCAAGTTTAGTAACTGGAGT
GTTTTACATAATTCGAATAATGGATTTGAAATTCCGACATATTTCAGTCCACGGACAACACAAAACATGCCTTGT
TCCGAAAAAGTTGAAAGCTGGCTTGAAAGATTACCAATTTTTGTGGGGTTCGACGGTTATTTATTTACAAACTGC
TTTGATTATGAATATATGTTGGATTGGGAAGAGACAGAATTTACATTCGAGAAAACATCATGTATGGAGACGGAT
TATTCTAAAGCTTTAACCGATACTGATATTATATATATTCAAGAGAAAAAAATTGAAGCGTTGATAAGAAACCAA
TATCTGAAGGAGTATGAATTTTCTCAAAAAGATTTTTGA

>YJL091C 0.78 Cold
ATGTCGACTTTAAAACAGAGAAAAGAGGACTTTGTGACAGGGCTCAATGGCGGTTCTATAACAGAAATTAACGCA
GTGACATCAATTGCTTTGGTAACTTACATATCATGGAACTTATTGAAAAATTCCAACCTTATGCCTCCTGGCATT
TCCAGCGTGCAATACATAATTGATTTTGCATTGAACTGGGTTGCTTTGCTTCTATCTATTACTATTTATGCTAGT
GAACCATACCTTCTAAACACGCTAATACTGTTACCTTGTTTGCTCGCATTCATATATGGAAAATTTACTAGCTCG
AGTAAACCTTCTAATCCAATATACAATAAAAAAAAAATGATTACACAGCGGTTCCAACTAGAAAAAAAGCCGTAT
ATTACTGCGTATCGTGGTGGGATGCTTATTCTGACTGCTATTGCCATCTTGGCTGTAGATTTTCCAATTTTCCCA

AGGAGGTTTGCCAAGGTGGAAACTTGGGGGACATCCCTGATGGATCTTGGTGTAGGATCATTCGTTTTCAGTAAC
GGTATTGTTTCTTCTAGGGCACTGTTGAAAAACCTAAGCTTGAAGAGTAAACCCAGCTTCTTAAAAAATGCATTT
AATGCCTTAAAATCAGGAGGAACTCTATTGTTCCTAGGATTGCTGAGGTTGTTTTTTGTAAAAAATTTGGAATAT
CAAGAACATGTCACAGAATATGGGGTTCATTGGAATTTTTTTATCACCCTATCATTGTTGCCACTTGTATTGACC
TTTATTGATCCCGTCACAAGAATGGTTCCACGCTGCTCAATTGCAATATTCATTTCATGCATTTATGAATGGCTA
CTTTTAAAGGACGATCGCACTTTAAACTTTTTAATTTTGGCTGATAGAAATTGTTTCTTCAGTGCTAATAGAGAA
GGCATCTTCTCATTTCTAGGTTATTGCTCGATTTTTCTTTGGGGCCAAAACACGGGATTTTACTTGTTGGGAAAT
AAACCAACTTTAAACAATCTTTATAAGCCTTCTACGCAAGACGTAGTTGCAGCATCAAAGAAGTCTTCGACTTGG
GACTATTGGACTTCAGTAACCCCATTAAGTGGCCTCTGTATATGGAGTACAATTTTTCTTGTTATCAGCCAGTTG
GTTTTTCAATACCATCCTTATAGTGTTTCAAGAAGGTTTGCTAACTTACCATATACTTTGTGGGTCATTACTTAT
AATTTACTATTTTTGACTGGGTACTGCTTGACTGACAAAATTTTCGGTAATTCTTCGGAATATTATAAAGTTGCC
GAATGCTTGGAATCAATCAACTCCAATGGGTTGTTTTTATTTTTGTTGGCAAATGTCTCTACTGGTTTAGTCAAT
ATGTCTATGGTCACGATAGATTCTTCACCCTTAAAATCATTCCTGGTTTTGTTGGCATACTGCTCATTCATAGCT
GTCATATCGGTTTTCTTGTATAGAAAAAGAATATTCATTAAGCTATAA

>YPL189W 0.76 Cold
ATGTCGATGTTAAGAATCTGGAGTTGCATCGTACATTTCTTTTCCGTGCAGGCACTTGATTCACGAATAAAGCCA
GACATTGAATTCAAAAGGCGACAAAGGATATTTATCAATTCATCTAAAGAAGAGAATGGTTCATCTTCTAGTGCC
GTTACTGTTACCCGTAACCCTGTGCTCAGCTCTAATAGCCCATCTCCTCCGCTATGGAATACATGGGAATTTAGA
CTATATTATTTGGCGTTCACCGTAGTAGTTCCTTTCATGATAAAGGCGGCATTGGCTACGAGCTCCGAGTCTAAT
CCAAACTACTATAAATTCAGCGGACTGCTAGCCCATGGTTGGATTCTGGGCCGCAAAGTGGATAACAGCGATCCT
CAGTATAGGTTTTTTAGATCCAATTTTTTTTTGTTGGCCATCTTAATATTACTACAAATTATATTAAAAAAGGTT
TTTGTTAAATTTAGTAAAATTCCGAAAACAAAATTCGATTTTGCGTGTGGATTAGTTTTTGTCTGTTTCATGTAC
GGGATCAATTCTGTTAAGCTCTTTACGCATGCTTTTATTTTCTTCACTTTGGCACATTCGCTAAAGAGAAAGCGT
TTGATAGCCGCTTTTGCGATTTGGTCCTATGGTATCTTTACACTATTCATCAATCAAAAAATGAAAAATCTCCCC
TTTAATAATATTGCTATCATCCTAAGTCCCATGGATCAATGGTATAAGGGTATCGTTCCTCGATGGGATTTTTTT
TTCAATTTTACATTATTGCGTTTGTTAAGTTACTCCATGGATTTTTTGGAAAGATGGCATGAACAATTGAGCCGC
CAACCTTCGATAGATTACGATGATAGACGACCTGAATTCAGAAAAAGTTTATCTGGTTCTACTCTACAAACCATT
TATGAGTCAGGTAAGAATGTTCTGGAGGAAAAGGAACGACTGGTAGCAGAACATCACATCCAGGATTACAACTTT
ATCAATTTTATCGCTTATATTACTTACGCGCCATTGTTTTTAGTGGGCCCAATTATCACTTTTAATGACTACCTT
TATCAATCAGAAAATAAGCTTCCTTCGCTAACGAAAAAAAACATAGGCTTCTATGCCCTCAAAGTATTTTCGAGT
TTGCTTTTGATGGAAATTATCCTACATTATATCTATGTGGGTGCAATAGCAAGGACCAAGGCATGGAACAATGAT
ACACCCTTGCAACAGGCTATGATCGCGCTGTTCAACTTGAACATTATGTATTTAAAACTTTTGATCCCATGGAGG
CTCTTTCGGCTGTGGGCCATGGTCGATGGTATTGATGCACCTGAAAATATGCTACGATGTGTGGATAATAATTAT
AGTACAGTGGGATTCTGGAGAGCCTGGCATACAAGTTTTAACAAGTGGGTAATCCGTTACATCTATGTTCCATTT
GGCGGGTCCAATAACAAAATATTAACGAGCTTTGCCGTATTCTCATTTGTAGCAATATGGCATGACATCCAATTA
CGAGTGTTGTTTTGGGGGTGGTTAACAGTCCTTTTATTATTAGGCGAAACCTACATTACTAACTGTTTTAGTAGA
TATAGATTCAGAAGCTGGTACAGGTTTGTTTGCGGTATCGGTGCTGCAATAAATATTTGCATGATGATGATTATT
AATGTCTATGGATTTTGCTTGGGTGCAGAGGGAACGAAGCTTCTATTGAAGGGCATATTTAACAATTCACATAGT
CCGGAGTTTTTGACTGCGGTAATGGTAAGCCTATTTATTGCTGTTCAGGTAATGTTTGAGATTAGAGAAGAAGAA
AAAAGACATGGCATCAACTTGAAATGTTGA

>YFL051C 0.81 Cold
ATGTCTATACCCCATTCCGTATTTTCGGCACTCTTGGTCTTCGTGGCGCTAGCTACTACAACCTTAGCCAGTACA
GAAGCTTGCTTACCAACAAACAAAAGGGAAGATGGTATGAATATTAATTTTTATGAGTACACAATAGGCGACCAA
ACCACATACTTGGAGCCTGAATATATGGGCTATGAATACTCCAATACAAAGAAGTTAGGTTCCGTTAGCGGACAG
ACCAATCTCTCCATATACTATAGTCCGCCTTGTGAGAGCACTCCTACCTGTGTGACTTATGCAGTTTTGAAGCGT
GATGAGGATGGATATGATCCTTGCGGACCTCTTTATGAAACTAAAAAAACGTGACACTGAATACTGTGACCCAAAT
ACTGCCTATTGGAGTTCTGATCTTTTTGGTTTCTATACTACTCCAACTAATGTAACTGTGGAAATGACAGGGTAC
TTAATATGGAGTATGGGCAACCGACGCCGTTGA

>YKL075C 0.71 Cold
ATGGCTAAAGATTTATTGCCCAAGCAAGCGGCCAACGAGCCATCGTTAAAGGACTGTACCTGTAAAAGATGTCTT
AAATTGGGTGCTTCAAAAGAAAAAAAGATTAGAAGAAAGAAGAAAGGTGAGGAGAAAAGAGAAAGACATTATGGA
AATAGACGAAAGTTAACATTCAACTTTTTGAAGCATACGAACATGGAAAATACAAATTACGACGTAATCACTTCG
GTTGGATATCTGAATGAAAAATACGGATTGAAAAAATCACATTACATTGAAAAATTTATCAAATGCATCCATAGA
AAAATAAATATCGATGTTAGCAAAATTACGGACGCCTATGTAAACTCGCTAAACCCGTGGGTTAAAGTAAAACTC
TTCCTTTTATTGGTGACATTGTCTGAAAAGGGTGGTCCGGAATACTGGCTAGATAAGACAGATGGTGAAAAGAAC
TCAGAGGCGTCTTCAACAGATAATAGTTTGGAAAATTCTACTAAAGGAGCAGATAGCGCTGGATCTACTGCTTTG
AGAGATGAAATGGTCAAAAGCCATAAAAACCTTTTCCCAACTCTTACAGAGCAAATCATACAGCATAACATCAAC
CAAGATTTTACGGAATCGACTTACGACGAGGATTATGTATTTTCGTCAATTTGGGCAAACTTCATGGAAGGACTG
ATCAATCACTATTTGGAAAAAGTCATTGTACCTTACTCGGAAATGAAAGTCTGTCAGCAATTATACAAGCCTATG
ATGAAAATTATCTCCCTCTATAACGAATACAATGAGCTTATGGTCAAAAGCGAAAAGAACGGATTCTTGCCTTCT
CTACAAGACTCCGAAAATGTCCAAGGTGATAAAGGCGAAAAAGAGAGCAAGGATGATGCCGTTAGCCAAGAAAGA
CTGGAAAGAGCGCAAAAGCTGTTATGGCAAGCTCGAGAAGATATTCCAAAAACAATTAGCAAAGAATTAACGTTG
TTATCAGAAATGTATTCTACATTATCTGCTGATGAGCAAGATTACGAATTAGATGAATTTGTTTGTTGCGCAGAG
GAGTACATTGAATTGGAATACCTACCTGCCCTTGTAGATGTCTTGTTTGCTAATTGTGGCACGAACAACTTCTGG

AAAATTATGTTGGTGTTGGAACCCTTTTTCTACTACATTGAAGACGTCGGCGGAGATGATGATGAAGATGAAGAT
AATGTGGACAATAGCGAGGGCGACGAAGAAAGCTTACTTAGCAGGAATGTGGAAGGAGATGATAATGTGGTTGAG
CGTCATTTTAAGCCTGATCCAAGGGTCATTACACTGGAAAAAATATGTGAAGTTGCTGCAAGACAGAAATGGATA
TAA

>YPL153C 0.78 Cold
ATGGAAAATATTACACAACCCACACAGCAATCCACGCAGGCTACTCAAAGGTTTTTGATTGAGAAGTTTTCTCAA
GAACAGATCGGCGAAAACATTGTGTGCAGGGTCATTTGTACCACGGGTCAAATTCCCATCCGAGATTTGTCAGCT
GATATTTCACAAGTGCTTAAGGAAAAACGATCCATAAAGAAAGTTTGGACATTTGGTAGAAACCCAGCCTGTGAC
TATCATTTAGGAAACATTTCAAGACTGTCAAATAAGCATTTCCAAATACTACTAGGAGAAGACGGTAACCTTTTA
TTGAATGACATTTCCACTAATGGGACCTGGTTAAATGGGCAAAAAGTCGAGAAGAACAGCAATCAGTTACTGTCT
CAAGGTGATGAAATAACCGTTGGTGTAGGCGTGGAATCAGATATTTTATCTCTGGTCATTTTCATAAACGACAAA
TTTAAGCAGTGCCTCGAGCAGAACAAAGTTGATCGCATAAGATCTAACCTGAAAAATACCTCTAAAATAGCTTCT
CCTGGTCTTACATCATCTACTGCATCATCAATGGTGGCCAACAAGACTGGTATTTTTAAGGATTTTTCGATTATT
GACGAAGTGGTGGGCCAGGGTGCATTTGCCACAGTAAAGAAAGCCATTGAAAGAACTACTGGGAAAACATTCGCG
GTGAAGATTATAAGTAAACGCAAAGTAATAGGCAATATGGATGGTGTGACAAGAGAGTTAGAAGTATTGCAAAAG
CTCAATCATCCAAGGATAGTACGATTGAAAGGATTTTATGAAGATACTGAGAGTTATTATATGGTGATGGAGTTC
GTTTCTGGTGGTGACTTAATGGATTTTGTTGCTGCTCATGGTGCGGTTGGAGAAGATGCTGGGAGGGAGATATCC
AGGCAGATACTCACAGCAATAAAATACATTCACTCTATGGGCATCAGCCATCGTGACCTAAAGCCCGATAATATT
CTTATTGAACAAGACGATCCTGTATTGGTAAAGATAACCGACTTTGGTCTGGCAAAAGTACAAGGAAATGGGTCT
TTTATGAAAACCTTCTGTGGCACTTTGGCATATGTGGCACCTGAAGTCATCAGAGGTAAAGATACATCCGTATCT
CCTGATGAATACGAAGAAAGGAATGAGTACTCTTCGTTAGTGGATATGTGGTCAATGGGATGTCTTGTGTATGTT
ATCCTAACGGGCCACTTACCTTTTAGTGGTAGCACACAGGACCAATTATATAAACAGATTGGAAGAGGCTCATAT
CATGAAGGGCCCCTCAAAGATTTCCGGATATCTGAAGAAGCAAGAGATTTCATAGATTCATTGTTACAGGTGGAT
CCAAATAATAGGTCGACAGCTGCAAAAGCCTTGAATCATCCCTGGATCAAGATGAGTCCATTGGGCTCACAATCA
TATGGTGATTTTTCACAAATATCCTTATCACAATCGTTGTCGCAGCAGAAATTATTAGAAAATATGGACGATGCT
CAATACGAATTTGTCAAAGCGCAAAGGAAATTACAAATGGAGCAACAACTTCAAGAACAGGATCAGGAAGACCAA
GATGGAAAAATTCAAGGATTTAAAATACCCGCACACGCCCCTATTCGATATACACAGCCCAAAAGCATTGAAGCA
GAAACTAGAGAACAAAAACTTTTACATTCCAATAATACTGAGAATGTCAAGAGCTCAAAGAAAAAGGGTAATGGT
AGGTTTTTAACTTTAAAACCATTGCCTGACAGCATTATTCAAGAAAGCCTGGAGATTCAGCAAGGTGTGAATCCA
TTTTTCATTGGTAGATCCGAGGATTGCAATTGTAAAATTGAAGACAATAGGTTGTCTCGAGTTCATTGCTTCATT
TTCAAAAAGAGGCATGCTGTAGGCAAAAGCATGTATGAATCTCCGGCACAAGGTTTAGATGATATTTGGTATTGC
CACACCGGAACTAACGTGAGCTATTTAAATAATAACCGCATGATACAGGGTACGAAATTCCTTTTACAAGACGGA
GATGAAATCAAGATCATTTGGGATAAAAACAATAAATTTGTCATTGGCTTTAAAGTGGAAATTAACGATACTACA
GGTCTGTTTAACGAGGGATTAGGTATGTTACAAGAACAAAGAGTAGTACTTAAGCAAACAGCCGAAGAAAAAGAT
TTGGTGAAAAAGTTAACCCAGATGATGGCAGCTCAACGTGCAAATCAACCCTCGGCTTCTTCTTCATCAATGTCG
GCTAAGAAGCCGCCAGTTAGCGATACAAATAATAACGGCAATAATTCGGTACTAAACGACTTGGTAGAGTCACCG
ATTAATGCGAATACGGGGAACATTTTGAAGAGAATACATTCGGTAAGTTTATCGCAATCACAAATTGATCCTAGT
AAGAAGGTTAAAAGGGCAAAATTGGACCAAACCTCAAAAGGCCCCGAGAATTTGCAATTTTCGTAA

>YGR010W 0.79 Cold
ATGGATCCCACCAAAGCACCCGATTTTAAACCGCCACAGCCAAATGAAGAACTACAACCACCGCCAGATCCAACA
CATACGATACCAAAATCTGGACCCATAGTTCCATATGTTTTAGCTGATTATAATTCTTCGATCGATGCTCCTTTC
AATCTCGACATTTACAAAACCCTGTCGTCAAGGAAAAAAAACGCCAACTCAAGCAACCGAATGGACCATATTCCA
TTAAATACTAGTGACTTCCAGCCACTATCTCGGGATGTATCATCGGAGGAGGAAAGTGAAGGGCAATCGAATGGA
ATTGACGCTACTCTACAGGATGTTACGATGACTGGGAATTTGGGGGTACTGAAGAGCCAAATTGCTGATTTGGAA
GAAGTTCCTCACACAATTGTAAGACAAGCCAGAACTATTGAAGATTACGAATTTCCTGTACACAGATTGACGAAA
AAGTTACAAGATCCTGAAAAACTGCCTCTGATCATCGTTGCTTGTGGATCATTTTCTCCCATAACATACCTACAT
TTGAGAATGTTTGAAATGGCTTTAGATGATATCAATGAGCAAACGCGTTTTGAAGTGGTTGGTGGTTATTTTTCT
CCAGTAAGTGATAACTATCAAAAGCGAGGGTTAGCCCCAGCTTATCATCGTGTCCGCATGTGCGAATTAGCATGC
GAGCGGACATCATCTTGGTTAATGGTTGATGCCTGGGAATCTTTACAATCAAGTTATACAAGGACAGCAAAAGTC
TTGGACCATTTCAATCATGAAATAAATATCAAGAGAGGTGGAATCATGACTGTAGATGGTGAAAAAATGGGCGTA
AAAATCATGTTATTGGCAGGCGGTGATCTTATCGAATCCATGGGCGAGCCTCATGTGTGGGCTGATTCAGACCTG
CACCATATTTTGGGTAATTATGGATGTTTGATCGTGGAAAGGACTGGTTCTGATGTTAGGTCCTTCTTGCTTTCC
CATGATATCATGTATGAACACAGAAGAAATATCCTTATTATCAAACAACTTATTTACAATGATATTTCCTCTACG
AAAGTGCGGCTTTTCATCAGACGTGGAATGTCAGTTCAATATCTTCTTCCAAACTCTGTCATCCGTTACATCCAA
GAGTATAATCTATACATTAATCAAAGTGAACCGGTCAAGCAGGTCTTGGATAGCAAAGAGTGA

>YOR323C 0.75 Cold
ATGTCCAGTTCACAACAAATAGCCAAAAATGCCCGTAAAGCAGGGAATATTTTGAAAACCATCTCAAACGAGGGC
AGATCAGATATTTTATACAAAATTCACGATGCCCTGAAGGCTAATGCGCATGCCATTGAAGAAGCGAATAAAATC
GATTTAGCTGTTGCCAAAGAGACTGGCCTAGCGGATTCTTTATTGAAACGTCTCGACCTATTTAAAGGGGACAAA
TTTGAAGTTATGTTACAAGGTATTAAGGATGTAGCCGAACTAGAAGACCCTGTTGGCAAGGTTAAAATGGCCAGG
GAATTAGATGATGGCTTGACGTTGTACCAAGTAACCGCTCCAGTCGGCGTTTTGTTAGTTATCTTTGAATCCCGT
CCAGAAGTTATTGCCAATATTACCGCATTGAGTATCAAGTCTGGTAATGCTGCAATTTTGAAGGGTGGTAAAGAG
TCTGTGAACACGTTCAGAGAAATGGCAAAGATCGTTAACGACACCATTGCACAATTCCAAAGTGAGACTGGTGTT
CCTGTGGGCTCTGTGCAATTGATCGAAACCAGACAGGATGTTTCCGACTTGTTGGATCAAGATGAGTACATCGAC

TTAGTTGTTCCTCGTGGTTCCAATGCCTTAGTCAGAAAAATCAAGGACACTACAAAAATTCCCGTGTTGGGTCAT
GCGGATGGTATCTGCTCAATTTACTTGGATGAAGACGCAGATTTGATTAAGGCAAAAAGAATTAGTTTGGATGCA
AAGACTAACTACCCAGCTGGTTGCAACGCTATGGAAACGTTGTTGATTAACCCAAAATTCTCTAAGTGGTGGGAA
GTTCTGGAAAACTTAACTTTGGAAGGCGGAGTGACTATCCACGCTACAAAAGATTTGAAAACTGCGTATTTTGAT
AAACTGAATGAGTTAGGGAAATTGACAGAAGCAATTCAATGCAAAACCGTTGATGCTGATGAGGAGCAAGATTTT
GATAAGGAATTTTTATCCTTGGATTTGGCTGCCAAGTTTGTTACATCTACAGAATCGGCCATTCAACATATAAAT
ACACACTCTTCGAGACATACCGACGCTATTGTAACGGAAAATAAAGCAAACGCTGAAAAATTTATGAAGGGTGTC
GACTCCTCTGGTGTTTACTGGAATGCATCAACTAGATTTGCCGATGGTTTCAGGTACGGTTTTGGTGCTGAAGTG
GGTATTTCTACCTCTAAGATTCACGCCCGTGGTCCAGTTGGCTTGGACGGTCTGGTGAGTTATCAATACCAAATA
AGAGGTGACGGCCAGGTTGCTAGTGACTACCTTGGTGCTGGTGGTAACAAAGCTTTTGTTCACAAGGATTTAGAT
ATAAAGACTGTGACATTATAA

>YHR212C 0.8Cold
ATGTCAAAAACCGATGTCAAAAAATCTCGTGAGGCCTCCGGAATTTTGACGCTGCAAGTCAATCTACGGGAAAGA
AGAAATTTTTTAAACTTAATGCAAAATAAGCTTTTTTCTTGGAAAATAAGATTTTCGGCAATAAAAGGTAAATGC
AGCCAAAAATCAAAATACTTCAGAAGAAGTCGTAGCGAGGACTGCTACGTGGAAGCGGATTTGAAGATCCTTTCC
AGAACAAGAAGGAGCCGAAAGCTGCCAGGAACTGTTCCTGATTTTTTAGGAAAACAATTAATAGGTATCTCGTCT
AGCGTAGTATCTCGAGTTTCCAGAAGTTGCAGATAA

>YAR069C 0.74 Cold
ATGGAGGACCACACCCTTGTGGCCATCGTTGTTTTCTTTGGGAATGGTGAACCTTTCCATGTGTCGTTGTCTGTA
GAAATGGTGTTCGTGCTGCTTCTTTCATCCACTAGAATTCATGAGGTAGTAGTTCTTATATGTTATAAACTTCAA
CACGCCACCTGGTCTTGGGGTAATATGAGCAAGAATTTCTCTCTTAAACCGGATATAAGTCTGAGCTTTCTGTTG
GATATAATTTCTATAAATGATATATGCATCTACGGTTGCATTGCCCTTACAGTTGTGTTTATTTTATAA

>YBR002C 0.77 Cold
ATGGAAACGGATAGTGGTATACCTGGTCATTCATTTGTGTTAAAGTGGACAAAAAACATCTTTTCGCGCACATTG
CGTGCATCTAACTGTGTACCTAGACATGTTGGGTTCATCATGGATGGGAACAGGAGATTCGCTAGAAAGAAAGAG
ATGGACGTAAAGGAGGGCCACGAGGCAGGATTTGTTAGTATGAGTAGAATCTTAGAACTGTGTTATGAAGCAGGA
GTCGATACGGCTACCGTGTTTGCCTTTTCAATTGAAAATTTCAAGAGGAGCTCACGGGAAGTTGAATCACTGATG
ACTTTAGCGCGCGAAAGGATACGACAAATCACAGAACGTGGAGAGCTGGCCTGTAAGTATGGGGTACGCATTAAA
ATTATCGGCGATCTCTCTTTGTTGGATAAGTCTCTATTAGAAGATGTTCGGGTTGCTGTGGAAACTACAAAGAAC
AACAAAAGGGCCACGTTAAATATCTGCTTTCCATATACAGGCAGGGAAGAAATCTTGCATGCCATGAAAGAAACA
ATTGTTCAACATAAGAAGGGCGCCGCTATAGACGAAAGCACGTTAGAATCGCATCTCTACACGGCGGGGGTACCC
CCTTTAGATTTATTGATTAGGACAAGTGGCGTTTCCAGATTAAGTGACTTTTTGATATGGCAGGCATCGAGTAAG
GGCGTACGCATCGAATTGCTGGATTGTTTATGGCCAGAGTTTGGACCTATACGGATGGCATGGATTTTATTAAAA
TTTTCGTTTCACAAATCCTTTTTAAACAAAGAGTACAGATTAGAGGAAGGTGATTATGACGAGGAAACCAATGGG
GACCCCATCGATTTGAAAGAAAAAAAGTTGAATTAA

>YDR536W 0.81 Cold
ATGAAGGATTTAAAATTATCGAATTTCAAAGGCAAATTTATAAGCAGAACCAGTCACTGGGGACTTACGGGTAAG
AAGTTGCGGTATTTCATCACTATCGCATCTATGACGGGCTTCTCCCTGTTTGGATACGACCAAGGGTTGATGGCA
AGTCTAATTACTGGTAAACAGTTCAACTATGAATTTCCAGCAACCAAAGAAAATGGCGATCATGACAGACACGCA
ACTGTAGTGCAGGGCGCTACAACCTCCTGTTATGAATTAGGTTGTTTCGCAGGTTCTCTATTCGTTATGTTCTGC
GGTGAAAGAATTGGTAGAAAACCATTAATCCTGATGGGTTCCGTAATAACCATCATTGGTGCCGTTATTTCTACA
TGCGCATTTCGTGGTTACTGGGCATTAGGCCAGTTTATCATCGGAAGAGTCGTCACCGGTGTTGGAACAGGGTTG
AATACATCTACTATTCCCGTTTGGCAATCAGAAATGTCAAAAGCTGAAAATAGAGGGTTGCTGGTCAATTTAGAA
GGTTCCACAATTGCTTTTGGTACTATGATTGCTTATTGGATTGATTTTGGGTTGTCTTATACCAACAGTTCTGTT
CAGTGGAGATTCCCCGTGTCAATGCAAATCGTTTTTGCTCTCTTCCTGCTTGCTTTCATGATTAAACTACCTGAA
TCGCCACGTTGGCTGATTTCTCAAAGTCGAACAGAAGAAGCTCGCTACTTGGTAGGAACACTAGACGACGCGGAT
CCAAATGATGAGGAAGTTATAACAGAAGTTGCTATGCTTCACGATGCTGTTAACAGGACCAAACACGAGAAACAT
TCACTGTCAAGTTTGTTCTCCAGAGGCAGGTCCCAAAATCTTCAGAGGGCTTTGATTGCAGCTTCAACGCAATTT
TTCCAGCAATTTACTGGTTGTAACGCTGCCATATACTACTCTACTGTATTATTCAACAAAACAATTAAATTAGAC
TATAGATTATCAATGATCATAGGTGGGGTCTTCGCAACAATCTACGCCTTATCTACTATTGGTTCATTTTTTCTA
ATTGAAAAGCTAGGTAGACGTAAGCTGTTTTTATTAGGTGCCACAGGTCAAGCAGTTTCATTCACAATTACATTT
GCATGCTTGGTCAAAGAAAATAAAGAAAACGCAAGAGGTGCTGCCGTCGGCTTATTTTTGTTCATTACATTCTTT
GGTTTGTCTTTGCTATCATTACCATGGATATACCCACCAGAAATTGCATCAATGAAAGTTCGTGCATCAACAAAC
GCTTTCTCCACATGTACTAATTGGTTGTGTAACTTTGCGGTTGTCATGTTCACCCCAATATTTATTGGACAGTCC
GGTTGGGGTTGCTACTTATTTTTTGCTGTTATGAATTATTTATACATTCCAGTTATCTTCTTTTTCTACCCTGAA
ACCGCCGGAAGAAGTTTGGAGGAAATCGACATCATCTTTGCTAAAGCATACGAGGATGGCACTCAACCATGGAGA
GTTGCTAACCATTTGCCCAAGTTATCCCTACAAGAAGTCGAAGATCATGCCAATGCATTGGGCTCTTATGACGAC
GAAATGGAAAAAGAGGACTTTGGTGAAGATAGAGTAGAAGACACCTATAACCAAATTAACGGCGATAATTCGTCT
AGTTCTTCAAACATCAAAAATGAAGATACAGTGAACGATAAAGCAAATTTTGAGGGTTGA

>YJL167W 0.79 Cold
ATGGCTTCAGAAAAAAGAAATTAGGAGAGAGAGATTCTTGAACGTTTTCCCTAAATTAGTAGAGGAATTGAACGCA
TCGCTTTTGGCTTACGGTATGCCTAAGGAAGCATGTGACTGGTATGCCCACTCATTGAACTACAACACTCCAGGC

GGTAAGCTAAATAGAGGTTTGTCCGTTGTGGACACGTATGCTATTCTCTCCAACAAGACCGTTGAACAATTGGGG
CAAGAAGAATACGAAAAGGTTGCCATTCTAGGTTGGTGCATTGAGTTGTTGCAGGCTTACTTCTTGGTCGCCGAT
GATATGATGGACAAGTCCATTACCAGAAGAGGCCAACCATGTTGGTACAAGGTTCCTGAAGTTGGGGAAATTGCC
ATCAATGACGCATTCATGTTAGAGGCTGCTATCTACAAGCTTTTGAAATCTCACTTCAGAAACGAAAAATACTAC
ATAGATATCACCGAATTGTTCCATGAGGTCACCTTCCAAACCGAATTGGGCCAATTGATGGACTTAATCACTGCA
CCTGAAGACAAAGTCGACTTGAGTAAGTTCTCCCTAAAGAAGCACTCCTTCATAGTTACTTTCAAGACTGCTTAC
TATTCTTTCTACTTGCCTGTCGCATTGGCCATGTACGTTGCCGGTATCACGGATGAAAAGGATTTGAAACAAGCC
AGAGATGTCTTGATTCCATTGGGTGAATACTTCCAAATTCAAGATGACTACTTAGACTGCTTCGGTACCCCAGAA
CAGATCGGTAAGATCGGTACAGATATCCAAGATAACAAATGTTCTTGGGTAATCAACAAGGCATTGGAACTTGCT
TCCGCAGAACAAAGAAAGACTTTAGACGAAAATTACGGTAAGAAGGACTCAGTCGCAGAAGCCAAATGCAAAAAG
ATTTTCAATGACTTGAAAATTGAACAGCTATACCACGAATATGAAGAGTCTATTGCCAAGGATTTGAAGGCCAAA
ATTTCTCAGGTCGATGAGTCTCGTGGCTTCAAAGCTGATGTCTTAACTGCGTTCTTGAACAAAGTTTACAAGAGA
AGCAAATAG

>YGL196W 0.8Cold
ATGAGCGATGTTCTATCTCAATATAAAGGGTGCTCAGTCAGAGATTTACCCACACCCAATTTCGTTATTAATGAA
GAAAAATTTGATAAGAATTGTACGACAATGTTGAACAACGTTGAAAAGCTCAGCCAGGAATGTGGCGTACCAATC
AAATTTCGTGCACACGTAAAAACGCATAAGACAGCAAAGGGAACTTTGAAACAATTGGGCCACGGACTTCCATTG
GCTAAACGCACTACAAGAGCCATATTAGTATCAACTTTGAAAGAAGCAGAAGAACTTCTTAATTATCAAGATAGA
CAATGTTCGGATTATATTGACGATATAACATATAGTTTACCCTGTTGCGTTCCAGAGTTTATTCCTCTTTTGAGC
AATTTGTCAAGAAGGGTGAATAATTTTCAGGTTTTTGTTGATAACATTGAACACTTGGAGAATTTAAAGAATTTC
GGTAGGCCTGCTTCCGGCAAGAAATGGTCGGTTTTTATCAAGGTTGATATGGGGACTAAGAGGGCAGGTCTTGCT
TTCGACTCTCCAGAATTTTTGAGTCTTTTAAAAAAACTGACTTCCTCAGAAATTAAAGAAGTAATTGAGCCATAT
GGGTTTTATGCTCATGCCGGACACAGCTACTCTTCAACCTCGATCAACGACACTCAGAATCTTTTGATGGAAGAA
GTGAAAGCAGTCAATTCTGCCGCTAAAGTTTTGTGCTCTGTGGATCCTCAGTTTGATCCTTCTAAATTAACACTT
TCTGTGGGCGCTACTCCGACTTCCAATTCTTTGAAACTCGATAATAAAAGTACCCTTGTTAAATTCATTACTACT
CAATTAGTTAGTACGCTTGAAATCCACTGCGGTAATTACTGCATGTATGACCTGCAACAGGTGGCAACAGGCTGT
GTCCAAGATCACGAATTGTCTGGTTTTGTATTAGGAACAGTACTATCATCTTACCCTTCTAGAGGTGAATTGTTG
AGTAATACAGGTGTAATGTGTCTAACGCGAGAAGCATCCTCAATAAAAGGGTTTGGAATATGTGCTGATTTGGAA
CATGTGTTAAAATCCGAGAGTTTCAGTAGGGAATGGTATGTAGCAAGGGTCTCTCAAGAACACGGGATACTGAGG
CCAATAAGAAACTGGAACGAAACTACTCCATTAAAATTAGGCAGCAAAATTGCCGTCCTTCCTCAACACGCTTGT
ATCACAATGGGACAATTTCCATATTATTTCGTGGTAAACAGCGAAGGCATTGTCAACGATGTTTGGTTACCTTTT
CAGAAATGGTAA

>YHR076W 0.72 Cold
ATGTTTGCAAACGTTGGATTTAGAACTTTGAGGGTCTCGAGAGGTCCCCTTTATGGATCCTGCAGTCAAATCATC
AGCTTTTCCAAGAGGACTTTTTACTCAAGTGCCAAGAGTGGGTATCAATCGAACAACAGTCATGGTGATGCGTAT
AGTTCAGGTAGCCAATCAGGCCCGTTCACCTATAAAACCGCGGTAGCTTTCCAACCAAAAGACAGAGACGATCTG
ATATACCAAAAACTAAAGGATTCTATTAGATCACCTACAGGAGAAGACAACTATTTTGTTACATCAAACAACGTT
CACGATATTTTTGCCGGTGTCGCAGACGGTGTTGGAGGATGGGCTGAGCATGGGTATGACTCCAGCGCCATCTCA
AGAGAATTGTGTAAAAAAAATGGACGAAATAAGCACTGCTTTAGCAGAAAATTCATCCAAAGAAACGCTTTTAACG
CCAAAGAAAATTATTGGTGCTGCATACGCAAAAATTAGGGATGAGAAAGTAGTGAAGGTTGGTGGGACAACAGCC
ATTGTGGCTCACTTCCCATCTAATGGAAAATTGGAAGTTGCCAACTTGGGTGATTCTTGGTGTGGAGTCTTCAGA
GATTCTAAACTTGTATTCCAGACAAAGTTTCAAACTGTTGGATTTAATGCTCCTTACCAATTGTCAATCATTCCA
GAAGAAATGTTGAAAGAGGCCGAAAGAAGGGGTAGCAAGTATATTTTAAACACCCCCAGAGACGCTGATGAATAT
AGCTTCCAATTGAAAAAAAAGGATATTATTATATTGGCTACCGATGGTGTAACCGATAATATTGCCACAGATGAT
ATTGAGCTTTTTCTGAAGGATAACGCTGCGAGAACAAACGATGAGTTACAGCTATTATCCCAGAAGTTTGTGGAC
AATGTTGTCAGTTTAAGCAAGGACCCCAATTACCCCAGTGTTTTTGCGCAAGAGATTTCTAAGCTAACTGGCAAA
AACTACAGCGGTGGTAAAGAAGACGATATAACTGTTGTTGTCGTGAGAGTTGACTAA

>YNR025C 0.8Cold
ATGACAAATACGAAAAAAATACTACACAATGCTTTATATTATGTTCTCATTATTATATACGAATACGTACTTCTG
CTGGTGCATTGTCTGCGCTACTTTTTTGAATTTCTATTTTTATTTTTACCATTATGGTTGGTCTTTTTCTTTTTG
ATGCTATCCTTAAATAATTTATCTAGGTCATATTCATCCTCTCCATCGTCTTGGCTCCCTGTAAATTCCTTATCC
TTAGCATCCCTTTTCTCGTCTTCATTTTGTTCGCCTTCATCAAACTTTCTCTTCCTGGAACCACTATCATCAGAA
TTATCGCCAAAGGTTTTTCTCCCACTTATCACACCCTCAGGTTTTCGCAGTTCACTGTAA

>YNL188W 0.81 Cold
ATGAATGTAACTTCTCCAAAAGATGGGAATCACAGTTTCTCGAAGAAAAATAGATTTAATACAAATAAACCGCGA
TTCCACAAACTAAATGAGCAGGCGCAGAGTATAAATTTACCAGAAGACCGTGATTCAATTGTTTCAAGTAATACA
ACGTCAATTATGACAGATGATGCATTTGATTACAATGAGGGCATTGCATCGCGTACCAAAAATATTAATTCTGAT
AGTGATAGAAGCAATGATACCATAAAACAAAACAACTACAATAAAAGGGAGACCGGATATAACCCTTTCTACAAT
GGATCAGGGATCAATCAGCGATATACACAGTTTCGAAAAAGAGAGTTTGAACCAACACTTGCAGAAAACAAAGCC
GAAGAGTACATATCGGACGAAGATAATGTAAAAATTGATGAAGATAATATAGAGAACGAACTCCAGTTTACGCCG
AAAATTAAAGAGGCTAGTATACTCCGATCTAGTTTACTAGGACAAAGAAATGTTTTAAATACTCGGAATCCAAAA
TCAAAAGAATCACACATTAAAGTAAAACCCATCATCAATAACAAGAGCTCCTCACAAAGAAAATCTAGTGCAGCA
CTTCGGAAACAATTAGGAAAACCCCTACCGCTGCCGTATTTGAATAGCCCTAATAGTGATAGTACACCCACATTA

CAGAGAAAAGAAGAAGTATTCACAGACGAAGTGCTTCAAAAAAAGAGAGAATTGATTGAGTCTAAATGGCATAGA
CTTCTCTTTCATGACAAAAAAATGGTGGAAAAAAAGCTAGAAAGTTTAAGAGAATACGAAAGGAAAAGAATGCCT
CCACGAGGAACTGATGTTTCTAGCTCTGAGCAGGACAATTCTTTCAAAATATCGACGCCAACAAAATCGTATGTT
TCTTTGGAGCAAAAACCCTTACCAAATCTCTCTGCTATGAATAACTTTAATGATGTTACCGACAATAAGGAGAAA
GAAGAAACGAACAACAATATATTAAAGTTCCAAGCGCAACGAGATCCATTACAAATACTACAGTCTGAGATCGAA
ATGCATACTAAGAAACTTGACACGATAATAGAGTTACTAAAAGACGATACCGATTCAAAGGAAAAAAGGAAAGTA
GTGACTAATGACAACGCAGCGCCTGAACAAATGGTCAACAAAGGATGGCGGAAAAACGTGATGATGATCTACAAA
AAATCAGGAAATATTATGAAAAAGTATAGGGAATATTTCTTATGGACAATTTGTATTTTAATATTGTTATATTGC
AATATATATGTGTATTATAGGTTTTAA

>YOL002C 0.81   Cold
ATGTCAACTTTATTAGAAAGGACTAAGAGTGTGCAAGAGCTGAAGAAGAGAGCTGCAGGGAAAACATCCGCAAAT
CCCGCAGAGGTCGCTAAAGCTAAAAAAGTACTAAGAAGGCTATATAGTTGGGATGAAATTCCGGAATGGCAAAGA
GACAATGATTTTATTTTACATGGATACGTGAAAGAAACTAGTAGCTTCATTGAAACTTTTAAAAGTTTGTTTTAT
TTGCATAATGAAAGTGTCAATATTTATTCACATTTAATTCCTGCTCTCGGGTTCTTCACTGTACTGTTGCTAGAT
AAATCTACTATCAAAGTGTTTGCAACAACTACATGGCTAGATCATATGGTAATCGACCTCTTTTATTCGGGGGCG
TTTGCATGTTTAATATTGAGTAGCTCCTTTCATTGTCTAAAGAGTCACTCCTTAAGAATTGCTACCTTAGGAAAT
AAGTTGGACTACCTTGGTATTTGTATATTGATTGTTACGTCAATGGTCAGTATTTTGTACTACGGCTATTTTGAG
AAATTTTCCCTATTTTGCCTATTTGCGCTTATTACCGTTAGCTTTGGGATCGCGTGTAGTATTGTGTCACTAAAA
GATAAGTTTCGGAAAAGAGAGTGGAGACCTTACAGAGCTGGGCTATTTGTTTGTTTTGGTTTATCCTCAATTATT
CCAATATTCAGCGGCCTTTACTGCTATAGTTTTTCAGAAATTTGGACCCAAATTCAGCTCTTTTGGGTATTACTT
GGGGGTGTCCTATATATAATTGGCGCTGTTCTTTATGGAATGCGGTTTCCTGAAAAGATTTGCCCCGGTAAATTT
GATATTTGGGGTCATTCTCACCAACTTTTCCATTTTCTAGTTGTTATTGCGGCATTGTGCCACTTGAGAGGTTTA
TTAAATAGTTATGAGTTAGTCCATATAAAGATGGAGAACGGGATTGTCTCCTAG

>YEL070W 0.76   Cold
ATGACAAAATCAGACGAAACAACAGCTACCAGCTTGAATGCTAAAACTCTAAAGAGTTTTGAATCAACTCTTCCA
ATACCAACTTACCCAAGAGAAGGTGTTAAACAAGGTATTGTTCATCTGGGAGTCGGTGCATTCCACCGTTCCCAT
TTAGCTGTTTTCATGCACCGTCTGATGCAGGAGCACCACTTAAAGGACTGGTCCATATGTGGTGTTGGTTTAATG
AAGGCAGATGCACTTATGCGCGATGCCATGAAGGCCCAAGATTGCCTATACACCCTTGTGGAGCGTGGTATCAAG
GACACTAACGCTTATATCGTCGGTTCCATTACTGCTTACATGTACGCTCCCGATGATCCAAGAGCTGTTATTGAA
AAGATGGCCAATCCAGACACACACATTGTTTCTTTGACGGTCACAGAAAACGGTTACTACCACAGTGAAGCAACA
AACTCCTTAATGACAGATGCTCCCGAGATTATCAATGATTTGAACCACCCAGAAAAGCCAGATACTCTGTATGGG
TACCTATATGAAGCCCTGTTGTTGCGTTACAAGAGAGGTCTTACCCCATTCACTATTATGTCATGTGACAACATG
CCCCAAAATGGTGTCACAGTAAAGACCATGCTTGTTGCATTTGCCAAGTTAAAGAAGGATGAGAAATTCGCCGCC
TGGATTGAAGACAAGGTTACTTCTCCTAACAGCATGGTGGACCGTGTGACCCCACGTTGTACCGATAAAGAGCGT
AAATACGTTGCTGACACCTGGGGAATCAAAGATCAATGTCCCGTTGTCGCAGAACCTTTCATCCAATGGGTTCTT
GAAGACAACTTCTCCGATGGCCGTCCTCCATGGGAACTTGTTGGTGTTCAGGTCGTCAAGGATGTCGATTCCTAC
GAATTGATGAAGTTGCGTCTACTTAACGGTGGACATTCTGCTATGGGATATTTGGGATACTTGGCAGGCTACACC
TATATACATGAGGTTGTCAACGACCCAACTATCAACAAGTATATCCGTGTTTTGATGCGTGAGGAAGTTATCCCA
TTATTGCCTAAAGTGCCAGGTGTTGATTTCGAAGAGTACACTGCATCAGTGTTGGAAAGATTCTCCAATCCAGCA
ATTCAGGACACTGTCGCACGTATTTGTTTGATGGGCTCTGGTAAGATGCCTAAGTATGTTTTGCCATCAATTTAC
GAGCAGTTGCGTAAACCAGATGGTAAGTACAAGTTGTTGGCAGTATGTGTTGCTGGCTGGTTCCGTTACCTGACT
GGTGTAGACATGAATGGGAAGCCATTCGAAATCGAGGATCCTATGGCACCAACCTTGAAGGCAGCCGCAGTTAAG
GGCGGTAAAGATCCTCACGAACTGCTTAACATTGAGGTGCTTTTCAGTCCTGAGATTCGTGACAACAAAGAATTC
GTTGCACAATTGACCCACTCCCTAGAAACAGTTTACGATAAAGGGCCAATTGCCGCTATTAAGGAAATTTTAGAC
CAAGTGTGA

>YLR382C 0.75   Cold
ATGCTGTCTCGACCTTCAAGCCGATTCCTATCCACTAAAAGGGGCCCTGGACCTGCAGTGAAAAAATTAATTGCA
ATTGGGGAGAAATGGAAACAGAAGACAACCCGTGGCTTGCCTAAGCAGGATACTCTGAATAGCGGGTCGAAATAT
ATTCTGTGCCAGTTTCCATATCCTTCTGGGGCGCTTCACATAGGACATCTCCGAGTTTATGTCATTAGCGACTCT
TTGAATAGATTTTACAAACAAAAAGGGTACAACGTGATACATCCGATGGGATGGGATGCTTTTGGGTTACCTGCC
GAGAACGCTGCTATAGAAAGAAGCATTAATCCGGCCATATGGACTAGGGACAATATTGCAAAAATGAAACAACAA
ATGCAAAGTATGCTGGCAAATTTTGATTGGGACAGAGAAATAACTACATGTGATCCCGAATACTATAAATTCACC
CAATGGATTTTCTTAAAACTTTTTGAAAATGGCTTAGCTTATCGTAAAGAAGCAGAAATTAATTGGGATCCCGTT
GATATGACAGTTTTGGCTAATGAACAAGTGGATGCCCAGGGCCGTTCTTGGAGATCAGGGGCTATTGTGGAAAAG
AAGCAGCTAAAACAGTGGTTTTTGGGAATAACGAAATTCGCTCCTAAATTGAAAAAGCACTTGAACCAACTGAAG
GACTGGCCTTCCAACGTGAAGCAAATGCAAAAAAATTGGATAGGCGAATCTGTGGGTGCAGAATTAGTGTTCAAA
GTTGCGGACCCCAAATTTGAAAACTTGATTGTCTTTACAACAAGACCGGAAACTCTTTTTGCTGTACAGTATGTT
GCTCTCGCATTAGACCACCCAATTGTACAAAAATACTGCGAGGAAATGCCGGATTTAAAAGAGTTTATACAGAAA
AGTGATCAATTACCAAACGATACGAAGGAAGGATTTCAGTTACCTAACATAAAGGCCGTAAATCCCTTAACTAAA
GAGGAAGTCCCCATATTTGCAGCTCCATATGTGGTCAGCAGCTATGGTTCAGCACCTAGTGCAGTAATGGGTTGT
CCAGGACACGATAACCGAGATTTTGAGTTTTGGCAAACAAATTGTCCTGGTGAACATATCAAGACGTGCATAGCA
CCTTTTTTTGACGATGCTTCAAAAGTAACTGAACAGGAAAGACAAAGAATAATTGATACTGTCCCGTTCACATCT
ACTGACGGTGTCCTAACCAAAGAATGCGGAGAACACTCAGGAGTTCTCACTGTAGTGGCAAGGAAATCGATAATG
GGGATGTTGAATAGCGAAGGACTGTCTAAGAGCGTCGTTAGATACAAAATTAGAGATTGGCTGATAAGTAGACAA

AGATACTGGGGTACTCCAATACCCATCATTCACTGCGACAACTGTGGACCTGTCCCCGTTCCAGAAAGTGATCTA
CCTGTCAAGCTACCAGAACTTGAAGGCTTGGATACAAAAGGGAATCCGCTGTCCACAATCGATGAATTTGTAAAT
GTCGCCTGTCCTTCATGCGGGAGCCCTGCGAAAAGAGAAACTGACACCATGGATACTTTTATCGATAGTTCTTGG
TATTATTTCAGATTCTTGGATCCCAAAAACACTTCAAAACCATTTGATCGTGAAATTGCAAGTAAAAATATGCCG
GTTGATATTTATATTGGTGGAGTAGAACATGCTATCTTACACTTGTTATACTCAAGGTTTATTGCTAAATTTCTC
GGATCTATCAATGCATGGAGCGACCCTGCTGGCATCTTCGAACCATTCAAAAAACTGGTGACACAAGGAATGGTT
CAAGGGAAAACCTATGTTGACCCCGATTCTGGTAAATTTTTGAAGCCTGACGAGTTAACCTTTGTAAATGACTCT
CCAGATGGCAACACAGTGATTATTAAATCAAATGGCAAGGTTCCGGTAGTCTCTTATGAAAAAAATGTCGAAATCT
AAATACAATGGTGCAGATCCAAATGAATGTATTTTAAGACATGGACCTGATGCTACGAGAGCACATATCCTCTTC
CAAAGTCCAATTGCAGATGCCCTGAATTGGGATGAATCCAAGATTGTTGGTATAGAACGTTGGTTGCAGAAGGTT
CTTCATTTGACCAAAAATATTCTCAGTCTCGAGAAGGATTTGGCAATAAGTAAAGACTACAAGACCCCGACCGAC
TTAAATGATGCAGAAGTGAAATTTCACAACGATTTCCAACGCTTCTTGAAATCAATTACGGAATCATTTGAAGTT
AATCTATCGTTAAACACTGTAATATCTGATTATATGAAGCTAACCAATATTTTAGAAAGTGCATTGAAAAAAGGT
GAAGTAAGGAATGAAATGATAGTACAGAATTTACAAAAGTTGGTAACCGTTATATATCCTGCTGTCCCGTCAATT
TCAGAAGAAGCTGCAGAGATGATCAACTCCCAAATGGAATGGAACCAATACCGCTGGCCAGAAGTTGAGCGCACT
ACTGAGTCCAAATTCAAAAAGTTTCAAATTGTGGTAAATGGCAGAGTCAAATTCATGTACACGGCTGACAAAAAC
TTTTTGAAATTAGGTAGGGATGCTGTTATTGAAACTTTGATGAACTTACCGGAAGGGAGAATGTATTTGATGAAT
AAAAAAATCAAAAAATTTGTCATGAAATTCAATGTGATTAGTTTCTTATTCCACAAGTAA

>YDR105C 0.81 Cold
ATGGGTGCCGTAATTTCTTTGCCAGTTAGCATGGCGGGCTCATTCGTGGCGTCCTGTTTTGGAGGTTGCTGCTCA
AACTTGGTGACTAAGACTGCATCTTCCCTAGGATCTTCTTCTTTAGGGACAAGACTTCTGTACGCCGTTTGGCTT
TTACTTAACTCATTGATATCATGGGTGTCCTATTCTGCGAACAAATCAATCCTATGGCCAGGGAAGACATGTACT
GGGACTGGGGAATGTGGATTTTTTACAGTTCATAGGTTGAATTTTGCATTGGGATGCTTACATTTGATATTGGCG
CTTGTACTAACGGGCGTAAAGTCGACTAATGACGTAAGAGCAGCATTACAGAATTCGTGGTGGAGTTTAAAATTT
ATACTGTATTTATGTCTCATTGTTTTGTCATTTGTCATCCCCAATGACTTTTATATTTTCTTTTCCAAGTGGGTG
TCAGTCCCTAGTGGAGCAATTTTCATCCTGGTTGGGCTTATATTATTAGTAGACTTCGCTCATGAGTGGGCTGAA
ACGTGTATTAGTCACGTTGAGTCAGAAGATGAAGATTCCTCATTCTGGCAACGATTTTTGGTTTTAGGGACAACT
TCAATGTATACCGCATCGATTATCATGACTGTTGTTATGTATGTTATGTTTTGTCATCAACAATGTAACATGAAT
CAAACAGCGGTAACAGTCAATTTAATATTAACTGTTATAACGCTTGTCTTATCGGTGAATCCTAAGATCCAAGAG
GCCAACCCTAAAAGTGGGCTGGCCCAAAGTAGTATGGTTTCTGTTTACTGTACTTATTTGACAATGAGTGCCATG
TCTTCCGAACCGGATGACAAAATGTGTAATCCGTTGGTTAGATCTAGTGGAACCCGTAAGTTTAGTATCATTTTG
GGCTCTTTATTTACTTTTATTGCGATTGCTTACACCACAACTAGAGCCGCTGCTAATAGTGCTTTTCAAGGCACC
AATACTAATGGTGCTATATATCTCGGGAATGATATCGAGTACGAAGGACTAGGTGGACAGACAAGAAATCAGTTG
AGGTATGAAGCTATTAAACAAGCTGTAGAAGAAGGATCTTTACCGGAAAGTGCCTTATATGATACCGCTTGGTTA
GGGACATCATCTCCTACAGGCGCAATGGATAACCAAAATGATGATGAAAGGACTGGAACCAAGTACAATTACACT
CTGTTCCACGTTATATTCTTTCTGGCAACCCAGTGGATTGCAATTTTACTAACAATAAACGTTACCCAGGATGAT
GTAGGTGATTTTATACCCGTAGGAAGAACATATTTTTATTCTTGGGTAAAAATAGTTAGTGCATGGATATGTTAT
GCCCTTTACGGTTGGACAGTGGTAGCCCCAGCGATTATGCCCGATAGATTTGATTACGAGAATTACTATTAA

>YCR023C 0.77 Cold
ATGGCGCGTCAAAAGCTTACTTTCAAAGAACAAATGGATGGTTTCCCCTGGGTCCAACTTGTTGTTGTGTCCTTA
GTTAGGTTCAGCGAACCAATTGCGTTTTCGTCACTATTTCCTTATGTTTATTTCATGGTTAGAGATTTTAATATT
GCTCCCAATGATGCTCAAGTGTCCAAATATTCAGGTTATTTATCTTCATCATTTGCGTTATGCCAAGTCATATCT
GCGTACCACTGGGGTAGATTCTCTGAAAAGCATGGCAGAAAAATAACATTGACTTGCGGGCTTATAGGAACATCT
GTATCATTGTTAATACTGGGATTTTCACGCAATTTCTATCAGGCTTTGGTGGCAAGAAGTTTAATGGGATTGCTA
AATGGTAACGTCGGCGTTATTAGAACCATTATTGGTGAAATAGCAACTGAAAGAAAACATCAGGCTTTAGCTTTC
AGTACTATGCCTTTATTATTTCAATTTGGTGCCGTTGTTGGGCCTATGATCGGTGGGTTTCTTGTATTTAGAGAT
GGAACAATGAATGAAGTGCCACTATGGTTTCCACATTTTGCAAAAAGAATAATTAGGTCATATCCGTACGCCTTG
CCAAACGTGGTAGTGTGCATGTTTTTGATGTTTGGTTTAACTAATGCAACATTGTTTTTGGAAGAAACACATCCT
GCTTTTAAAGATAGAAGAGATTACGGTTTAGAGGTCGGTGATTTTATTAAGAAGAATATATTTGGTATACAGCCG
AAAAGAAGACCCTGGCAAAAGCGCATTCAGGATGATTCGGAAAACATTCACCACCGTAATGAGAATGTGAACAGC
ATTCGAGGACAAGATAGTGAAGAGGATGAAAATAGTCCCCTAGTGAATACTACCAATGACGATGATACTGAAAGC
ATACAATCGATTGATCCTATTTTAACAAGAAGACAGTCTGTAGGCCTGATTAGGACATATTCTCTGCATGAACCA
ACAGACGCTGTGCATGCCAATATAGATACAGCTCCAGACGGTTGTAAAGAAAGTAGTATATTTCATCACGTTTTT
CATACAAAAGTATTTTACCCTATATCGGTGAATTTTATTATGGCTTTACATTTGATTGTATACAACGAATTTTTG
CCTGTTTTTTTAGCTTATGATTTAGCCGTAGATCCAGAAAATCCAAAGAAGCTGGCTTCAAAATTTCCGTGGAAA
ATATCTGGCGGTATAGGTTATGAACCAGAACAAACCGGTACTCTTTTGTCGACAACAGGTATCTTTGGTTGTTTT
GTGGTTATTTTCATTTTTCCCATAGTTGATCGAAATTTCGATTGTTTAACAATTTTCAGAACTTTAGTCAAGCTG
TACCCTATTATGTACGTTATGGTTCCTTACGTTGTTTTTCTACAGAATGAACGGATTCCTAGCTGGTATACTGTC
GTCTACTTGTACATAATCACAGGGATAAAAACATTTTGTGGCGCTTTAACGTCACCACAAATTATGTTATTAATT
CATAATTCGAGTCCCTTGAGTTGTAGATCAGTCATCAATGGCGCCACCATTAGTATTTCTGCCTCTGCTCGTTTC
ATAGGTCCCTTAGTATGGGGCTATATTATGTCTTGGTCCCAGCAAAATGACGTCGCCTGGGTCAGTTGGTGGTCG
TTAAGTCTTTTTTGTATGGTAGCTCTTTATCAAAGTTATAAGATAGCACCAATTGATGATAACGAAAATGAGCTT
CATGGACAGGGTAGTGAAGATGCCTACAATTCGCAGTCACAGTCTTCTGATTTAAGAATGGCTCATCGATCTAGT
TTAAGCAGCTTAAGTAACCAACGCTGTACCACATGA

>YIR033W 0.81 Cold
ATGCAGCAGAACAGTGAGTTCTTAACGGAAACACCTGGAAGCGACCCTCATATATCTCAATTGCACGCGAATAGC
GTAATGGAATCACAGCTCTTGGACGATTTCCTCCTGAACGGGTCTCCCATGTACCAGGATGATAGCATGGCGCAT
ATTAATATTGATGAGGGTGCTAATTTCCAAAATTTTATCAAGACAGATGAGGGTGATTCGCCCAACCTGTTGTCT
TTCGAAGGTATCGGTAACAATACTCATGTCAACCAAAACGTGTCCACTCCACTGGAGGAGGAAATGGAAAGTAAC
AGAGCCTTGAAGGAGGAAGAAGAGGACGAGCATGAAAATAAGGTTTTTAATGAAAAAAATATAGGCAACCCTGCT
CATGACGAGATTGTATTTGGAAGAAAGGAGACGATTCAATCTGTTTACATAAATCCTTTAGATTACCTTAAAGTG
AACGCAGCGCAGCTACCTTTGGATGTAGAGGTCTCAGGTTTGCCACAAGTATCTAGAGTGGAAAATCAACTGAAA
CTGAAAGTGAAAATTACGTCTGAAACACCACTAAACCAAAGCATGCTTTACTTGCCTAGCGATTCCATTTCAAGA
GAAAAGTTTTATTTAAAAAAAAATATCGAGGATTTTTCAGAAGACTTCAAGAAAAATCTTCTGTACATCAATGCG
TTTGTTCTATGTGCGGTCAGCAACAGAACGACAAATGTTTGTACCAAGTGTGTTAAGCGAGAACAAAGAAGAGCC
GCTAGAAGGAAATCAGGTATTGCAGACAATTTACTCTGGTGTAATAATATTAATAGAAGGTTAGTCGTGTTCAAT
AACAAACAGGTTTTCCCCATAATGAAAACTTTCGATAATGTTAAGGAGTTTGAATTAACTACCAGGCTAGTTTGT
TATTGCAGGCACCATAAGGCAAATAATGGCTTTGTCATATTATTCACTATAACAGATTGGCAAAATAGACTGTTG
GGTAAGTTTACGACAACACCTATTATGATCACGGATAGAAAACCAGCAAATATGGATACCACCAAGTTTAATAAC
ACTACTACCTCGTCCAGAAGGCAGCTAACGGAAGAAGAATCTACCACAGAATATTATTCAACGGATAACAACCAA
TTGAGCAAAGACGAAAATATGCCATTTCAATATACTTATCAACACAACCCATATGATAATGACAGTCAAATGAAT
AATATTCCACTGAAAGACAAAAACGTACCATTCCCATATTCCATCTCTCAACAGACAGATTTGCTTCAGAACAAT
AACTTATCACTGAACCTTTCTCTGCCCAATCAGCATATTCCATCACCAACATCTATGAGCGAAGAAGGCTCAGAA
TCATTTAACTATCATCATCGCGATAATGACAATCCCGTCCGTACTATCTCTTTGACAAATATTGAACAACAGAGT
CAATTGAACCAACGGAAAAGAGCACGCAATAATTTGGAAAATGACATTGGTAAACCCCTATTCAAGCATTCCTTT
TCAAATTCAATCAGTGCAACAAATACGATGAATCCAGCTTTACATTCAATGCAAGATTTCTCAATGAAAAACAAC
AACAATAATTTGCCATCAATTAATCGCGTTATACCTTCACAAGGCCCAATCAATGGTGGTATCGAAGTTACATTA
CTGGGTTGTAACTTCAAAGATGGTCTTTCTGTAAAGTTCGGCTCTAATCTTGCCCTTTCTACGCAATGCTGGAGT
GAGACCACGATCGTCACTTATCTCCCTCCCGCTGCCTACGCGGGTCAAGTTTTCGTCTCTATTACTGATACGAAT
AATGAAAATAATAACGATGACCTTCCCCAAGAAATTGAGATCAATGACAATAAAAAGGCCATATTTACCTATGTT
GATGATACTGATAGGCAACTGATTGAATTGGCTTTGCAAATTGTGGGATTAAAAATGAATGGTAAGTTAGAAGAT
GCAAGAAATATCGCGAAGAGGATTGTTGGCAATGATTCTCCTGATAGCGGTACAAATGGCAACAGCTGTTCAAAA
AGCACAGGTCCCTCTCCAAACCAACACAGTATGAATCTGAACACAAGTGTTCTTTACTCCGATGAAGTCTTGATA
CAAAAAGTTATAAAATCATTGAACATAAATTCCAATATTTCCATATGTGATTCATTAGGGAGAACTTTATTACAT
CTTGCCTGTTTGAAAAATTACTCAAGCCTTGTGTATACATTGATTAAAAAGGGTGCTCGTGTTAACGATATTGAT
TCCTTTGGGCTAACTCCATTACATTTTGCTTGCATAAGTGGTGACCCTAAAATTATTAAGATGCTTTTAAATTGT
AAAGTAAATTATTCACTGAGGTCACACAACGGATTAACTGCAAGAGAAGTATTCATAGCAAACCACATTCATTCA
AAGGAAATAGACAAAAAACAAGATAACAGAGACAACCATAAGTTTGTTCATAATGACACTTATATCAGCGAAGTA
TTGTCATTGTTTGAAGAATTCCAAAACGGTACGAAGTTTACCGATAGTGTAGAAACAGACAGTAATTATTCTATT
AGCAGGAAATATTCACAATCCAGTTTCAATTCAAGCCTGCTAGACAATGAATCTTTGAATGAGAACTTATTCGAA
AGCCAAAGCATGATAAATCCCACTTCTATGGAGATTCAGCATCCAACCTTGCAACTATTTGAGAATTCAAGTTAC
TCTGAGTACGACCAAAGTGATTTCGAAGAAGACGGGGATGAAGATCTGTTCGTCACTGACGAAGTAGAAAAACCA
GGTGTTGCATGCAGGGAGGAACAAAGCGAACTCCTTGATATTGGATCTAGCGCCAACGAACCCGAAGAGGATAAT
GGTAGTACATCTCTCTGGAATAGAGTTTTACATCGAATTAATGATGACTTACCAAAATATGAGGATCTGTTCCCG
TTGTCTTGGGGTAAAGATGATAAATTGAAAACCACAAATCAAGACAGTATTGTGGAGCAGTCAGCATCTAATATT
GAAAACTCTGAAAATTCGGAGGAAGAGGATTATGAGGAAGAGGAAGAATTTTTGAAAAAACAGTTTAACAGATTC
TTCCAAAACAAACAAAACTTCCGAAATGATAAAATGTTAATATTTTTCTGGATACCCTTAACACTACTACTTTTG
ACATGGTTCATCATGTACAAATTTGGCAACCAAGATAGTTCCATCAATCATATAAGCGAATTAATCTCAGAGTAC
TTGAGAATTGCATTAGCAAAGTTCTTGCTGGGAAATGAAAGGATGAAAACTGCATTCAGGTCAAAATTATCAAAC
CTGCAAACAACAAGAATGTTGAACGATTTAATTGTCAGTTAG

>YGR288W 0.8Cold
ATGACTTTAACTAAGCAAACATGCGCCAAGCAGGCATGCGACTGCTGTCGTATTCGTCGAGTGAAATGCGATGGT
AAAAGGCCGTGTAGCAGTTGCCTACAGAATAGTTTGGATTGCACTTATCTGCAACCGTCGAGAAAAAGAGGTCCG
AAGTCCATTAGGTTGAGGAGCTTGAAAAGAATAGCAGAAGTGCAGAGGGAAAGCGGTCCTAACACCATTGCAACT
GCTCCTGTAATATATAAGAGGGTTCCCAAAAAGCTAATCGATCAGTGCTTGCGGCTCTATCACGATAATTTATAC
GTAATCTGGCCCCTTCTTTCGTACGATGACCTTCACAAACTTCTGGAGGAAAAATACAATGACAATTACGTATAT
TGGTTTCTGACCGCTTTATCAGCGGCCACCCTCAGTGATTTACAAACTGAAATAAAATCTGAAGAGGAAGTCACT
TTCACGGGAAAACAGTTATCTAATCTTTGCATCTCATCGTGTCAGCAATTTGACGATTTGGATAACAGCAATATA
TTCAATATTATGACGTACTACTGTTTGCATCGTAGCTTTGCACAAATATCGAACGCAAGAACTTCTTACAGACTC
TGTTGTGAAGCGGTCGGTCTGATTACGGTAGCAGGGTTACATCGGGAAGAAACTTACGGATCCCTTACATTTGAA
GAACAGCAACTTAGACGGAAACTTTATTACTTGCTTCTCATGACGGAGAGATACTATGCCATATATCTTCATTGT
GCGACGAGCCTGGATGCCACAATAGCACCACCGCAACTTGAACTTGTAACTGATCCTCAGCTTTCTATGGACAGT
TTCCTTGAAATGATTAGGGTATTTACTGTACCAGGAAAATGTTTCTTCGATGCTTTAGCCGCTGACTCTACAGAT
GCTTCTTGCACTGAAGAGTCATTGAAAAAGATATGGAACGAACTCCACACAACTTCCTCGGAAATAGAGCCATGG
TCTAACGGTTACATAGACATCTCATTTTCCCGGCATTGGATTAGGATACTAGCATGGAAGCTAGCTTATCAAATG
AGGGGTAGCAACTTTTCATTGAACGCTAACAATGGGCAAATACCAATAGAAATTGCGAGAGATATGTTAATAGAC
ACTTACTTAACCCCAGAGAATCTTTACGATGTCCATGGTCCCGGGGTACCAGTGAAAACATTAGAAATAGCTACT
GCTTTGGTGGACATTGTAGGCCAGTATGATCATAACATGAAATTAGAAGCATGGAATGTTTTGCATGATGTATGC
AAATTTGCTTTTTCTTTAAACCACTATAACAATGATATGCTGAAGAGATTTTCCACCAAATGCCAGAATGCCCTA
ATTACTCTGCCCATTTCTAAACCTTTACAATTGGATGGTTATCCCAAGGATAATGAAGACATAGACCCTTGA

>YGL247W 0.77 Cold
ATGGAACTCCGTAGTTTTTCTAGACAGCCTGATGGCATACTTGCTAACCCAAGATTGGGAAGGGAGGAAGTATTG
GAAGGTGAACATCCGCAAGATGCTAGATTAGCCCGACAAAGCATTTGGTTGAGCCCAAGTTTAATTGCGGAGTAT
ATACAGCTTTTTTTCAATTTTATTATAGGAACTATAGGACTGTCTCTTGCTATTAAATTCATTTTGATGATAAGA
AACGATGTTAACTTAAAACTGGAGCATAACGTGAGGGAAGAATTAGATAAAATTGCAACTTGCAAATCAAGGTAT
TTTGAAAACCAGTGCGAACCTCATATGAGGGTTCCAGCATTGGAGGTACGTTGCAATGAATGGTCAAAATGTATG
AACAAGGAAATAGTGTCTGGCTCCGATTACCAATGGGCTAAAGCATGGGCTCGTACTCTGGCGGAGGTAATAAAT
GCTTTTTTTGAAGCGTTCAGTATACGATCTTTCCTTTTCATTTTAATTAGCATAATAGGTATAATATTTGTTACC
AATACGAGTTTCGGATCATACAGGGTTTATCTCAATAATAAAGATACAAAATCGGTTCGCCATGCATAG

>YDR537C 0.74 Cold
ATGTTTTCAAGATGGATGGAAGATAAAGGGGTTTCCCGAGTAACCAGTAGTAACTTACGATTCTCTTTAATCGAA
ACATCGGCAGCTCTTGTAATTAAATCCTCTGTAAAACCGATTCTAATAGCAGCTAGTGATTTCATGGAACAGGGC
ACAACAATCATACCATCATGCTGGAAAGATCCGGACGAAATGCATGCAGAAACATCACGAACAGAGTATGTCTTG
GTTGCCAAGGCCGCCACGTCATGCGGTTCCCAATCTGTTTCATATTTCATTGTTGCTGCACCCCATTTTGAAATC
ACCAAATGGGTTTCTACGCTCAACTCTTTTAGCACTTGTAGAAGTCTGATTCCCAGTGCAACACCAGTCGCACCA
GTAATTGCGACAACAATTCTCTTTGGTCTTGGAGGCGAAGTTGATGCCCTGGTTACTTCCTTTGACAGTTTATGT
GTAAGGAAAGATGGTGAAGTTGTAATGGTTCTACCTAGCAAAGGAAAATTAGCAAAAATGCCTGTTGTTTTGAAA
AAGGCTATATTAGTTCTTCTTGGAAATAGGAGCATATCTACTCAATTGAAGCTGCTCAATGCGTTGGACTCCGTA
GATTGA

>YHR121W 0.73 Cold
ATGAGTGTCAGCCTTGAGCAAACGCTCGGATTCAGAATAAAAGTTACGAACGTGTTGGATGTAGTTACTGAAGGA
AGATTGTATTCGTTCAATTCATCCAACAACACTCTTACTATCCAAACAACAAAGAAGAATCAATCTCCACAAAAC
TTCAAGGTGATAAAATGTACATTCATCAAGCATTTGGAAGTCATTGGTGATAAGCCCTCGTTTAACTCATTCAAA
AAGCAACAAATCAAACCCTCATATGTCAACGTGGAAAGAGTTGAGAAGCTTTTGAAAGAAAGTGTAATAGCATCT
AAAAAGAAAGAACTCTTAAGGGGCAAGGGTGTGAGTGCAGAGGGTCAGTTCATTTTCGATCAAATCTTCAAGACC
ATAGGAGATACTAAGTGGGTGGCTAAAGACATCATTATTCTTGATGACGTTAAGGTGCAACCTCCATACAAGGTC
GAAGATATCAAAGTGCTACATGAGGGAAGTAACCAATCCATTACATTAATTCAAAGAATAGTGGAAAGAAGCTGG
GAGCAGCTAGAACAAGACGATGGTAGGAAAGGTGGATAG

>YNR067C 0.77 Cold
ATGCAATTATATCTGACACTTCTTTTTCTATTAAGTTTCGTCGAATGTTCATATATAAGTTTCATATCGAATAAT
GCAGACGAAATATTGGAAACTGATTTAATTGAAACTTTATCTTACGCTACTTTGACAGTAGGTGAGCCTTATGTT
GCACAATCGGTGGTTGTAACAAGAGTATCTGCAGCTTCTCATAGCCCTCTCTCAGTTTCGCCAAAAAAATAGAGTG
AGCGCATCTCCAATTAATTCACAAGACAGTGATTCTAATACAAGAACAGCAGTTCAACTGTCATTGTCGTTATCA
AACTACGCAAGCCAAGTATCTCAAAAAAATAAGCGCACAAACCAATAATGATCCTGTCACTGTTTCAAATATTTAC
GCAAATGATAATTCGAAAAGCAAAAGTTCAGTACACAATTTGAGTTCGGTTTCCGGCGTCGCCTCAGTCATGCCA
AGTGCATCAACAATGCGTAAAGTCACCACATTATTATCTCAAACTGCTTCGACTTCGACTTCGACTTTATTTTCT
TCATCTTTATCTATTTCGGGGACACAATTAAATGGAACTTTATTGACTTCTGTTTCAAAAGGTACAATCGATCCA
CTGGTAACTCAAATGCCTTCCTATTCTTCCCAAGAAACAAAAATTATTCCTTCTTCATTGACCTCGAATAAAACG
ATATATACTATTTCAGTAAGGACAAATGCAGCTACAGCTACCGGCGAAGATTCCTTCATTGCTTCAACTCCTGCT
TCCTCGACTTTGTTCTATCCATCCAACTCAACCCAGGATTTAGTCCAGACGCTCGCATCTACAACAGCAAGCCCT
GCTTACCCATCTAATAGGACACAAATCACTCTTTCTCCATCTGTATCACTATATTCGACAACTAGTCCAATTTAC
CCTTCAAACATTACAGAAAACGGTTCTTCACCATCACCATCACTATCATCGACTGTTAGTCCAGTTTATCCATCG
AGTTCAACAGGAAACATTCTTTTGTCATCACTATTTTCAACAGTAGATTCTTCTTCCTCTCCGGTTTCTTCGACA
TTAGATACCATTTATGTATCTTCTTCAATGCAAGCTACCATCTCTTCGTCCTCATCTTCGAGACAAACAAAAACT
TCCTCCTCTTCTCTGTCAACCTCGACCAGTAGCACCGCTACCACAACGGAGAACTCTTCAACAACGACCATTGTC
AATCTTTTCAATGCTGTTTCGACAGATGAACCACCAACTGTTTTTGACAGATCACCAAATCCTATGTCATTGGCT
GATGGGGTGTCAAACGATGGTCCCATTCAAACAAACAAGTTTTATACAAATTTGATTGTTGGTAGTCAGGAATCA
CCTGCCTTTGTGTATCCTTATTCATTGTGGAAGTACACCTCGAGTTCATATGGTTTTGCTGTTCAACATACAACA
GTAGATCAATATAGCTACGGTGGTTATGACAGTTCCGGTAATGCAGAATATTTGGTCAATCCTTTGGGAATTGCT
CATGTTGTTTTCTCAGCTTCGAATTTTGATTCAAGTATGACTATGCAAGTTGATGAAATGACCTTGTCTTCAACA
AGGGTAGTGCTATCAGAATCCAATGATTCTTCGAACTACCTGGAAATACCTTTGGTTCAAGGTATGGGATTTGCA
ACTGGTATATATCATGGATCTTTAAATGCCAAGATTGGTTCCAGCGTCGGATTCAATACCATTGTTTCTGAATCA
TCAAGCAATTTAGCTCAGGGTATTCTCAAATATCGTATCACCCTTTTGAATGGAGTCACTTGGTTATGTTACGTG
ATAGGACCAGACGATTTAACCTCAACGGACTTTTCATTGGAGGTGAGCTCAGAATATGAAATTAAAGCAAGCGCT
AGTGTTGATGGTCTTATCATTCAATTGGCCGTCGCACCTTCTGAAACTGATTACGAGGTCTTTTATGATCAAGCC
GCAGGTATGTACGTTACTAATTTCAAGCTGCAGGGTGTCTCTGACGGTTCTACAGCTACCTATGAATTTTCTTAT
ACCACCCAAGGTGAGTCGGCATCAGGTAGCACAATGATTTTTGCCTTGCCCCATCATGAATCTTCATTTAGTGAT
ATAATGCAAGACTACTATACCGGCATTCAACTCGCTTCTACAACTAAGGGTGTAATGAACGGTTATCTAACAACG
AGCCTACAATTTTCCACATCTCTAAACAGACAAATATCTTGGTTACCATGGTCTTCTCAACTTGGCTCAAACCTT
TTAGAGTATTCTAAAGAACAATTGCAATTATTAGCTGAAGTTGCCAATTCCGAATTACAGGTTAGTATTTCCGAA
AGTATAAGCGGGTTAAACACATATTATTTGGGTAAAGTTATCGATAAGTATTCTTACATTCTATTAACGGTCTCC
GAAATCATCCAAGATGAAGCCAGTACTAAGAGTACTTTGGAAAACATCAAATCGGCCTTTGATATTCTGCTACAA
AATGAACAGACGTATCCACTAATTTACGACACAAAGTTCAACGGTTTGGTCAGTTCAGGAGATTGGGGTTCCACC

AGCACGCAATACGATTTTGGTAACACTTATTACAATGACCATCATTTCCATTATGGTTACATTATTCATGCAGCT
GCTGTCATCGGCTATGTTGACTCTAAACTGAATGGTACTTGGGCTGCCGACAATAAAGATTGGGTCAATTCATTG
GTAAGAGATGTCGCAAATCCATCTGAAAAAGATGAATACTTTGCACAATCGAGAATGTTTGATTGGTTCAACGGT
CATTCATGGGCAGCTGGACTTTATGAAAACGGTAACGGTAAGAACGAAGAAAGTAGTAGTGAAGATTACAATTTT
GCCTATGCTATGAAGTTATGGGGGGCCACTATCGGTGACCAGTCAATGGAATTGAGGGGTGACTTGATGATTAGT
ATAATGAAGGATGCAATGAACGACTATTTCTATTATCAAAATGACAACACAGTTGAGCCTGAAGAAATCATAGGA
AATAAAGTGAGTGGTATTTTATTCGATAATATTATCGATTATACTACTTATTTTGGAACAAACACAGAATATATC
CACGGTATTCATATGCTACCTATCACACCAGTTTCTTCTAATATTCGTTCTGAGACTTTCGTCGAAGAAGAATGG
CAGACTAAAATCGAGCCAATTATTGAATCGATAGAAAGCGGCTGGACAGGCATATTGAAGCTGAATCAAGCACTC
TTCGACCCAGTAGATTCGTATGCATTTTTCAGTGATTCAACTTTTGATTCATCCACATATTTGGATAACGGAATG
AGTCGCACATGGGCATTAGCATTTTCAGGGGGACTGGCCAACTCAATTGCTTAG

>YJL201W 0.8Cold
ATGATAGATATCAACGTTAATAACATATTTTTAGATCTTATTCAGTTGATCCTAACTCTGGTCACGCAATTTAT
GTCTTTGATTCGACTTATTTGCCAGCATCTGATGAAATTGGCGATAAGCAAGTTTATGACTTGCTTATTAATGCC
CTCATGGATCGATTGGTAATGAAATTGCCACAGGCACCATATTCACTGGTGATTTTTTCATCTGGGTTTTCCCAG
AGAAAAATAAGCTGGGTTTACGGTATTAAAATGTTTGCAAAGTTACCCAAAGAGACCAAGTTCTATTTACAAAAG
ATTTTTATTGTTCATGAGTCATTCTTCGTTAGGTCGGTCTATCAAGTAATTTCAAACGCTATGAACTTTAACTTC
TTGGACTCAAAAGATAGTCAGCATGATTTTCCAAGTTTAGTTCATGTCTTGGACCTGACTTCTTTATCCGAGCTA
ATAGATATTACGCGACTTAGAATATCGTTGAACGTGTACTTATATGATTACCAGATACGCGAACATATAAATGTC
CCGGAGGAATATTATAACAGATTGACCCCTCTGGCAATAAGACAGTATAGGCAGTTAGTATTCGATAAAATTTTC
AAGAAATTACAGAATGACGCTCTGTTGTGTGAATTGATATTCCAGAAGCCGGGAAACTATAAAAAGGTAAACATT
TTTTTGGATATTATCAAAAGAAACAACTACATTGATTTATCTCAATGGGATATTTATTCTTTGGCATCCGTGTGG
TTGAATTACTTCATTAAGAATAAAGCCAAACCTCTAATCCCAATTGAATTAATTCCGTTACCAATTGTTGATGAT
CTAAAGTTCACAAGTGAAACATTCCGCAAAATTATTAAATTCAACCAATACCAGGACCTCTTTATGGTCATAATT
CCTTTTTTTAATAGGATCATAGCGCATGGTGAATCGACTAAACATGATTCAAGGACCCTAAGTAAAGCTTTAACT
CCGGCGTTATGTAAGGAAAAACTTTCCATGATGACTAATGATCGTTTAGCTATTGGGTCGAGATATATTAAAAAT
TTACTGGACTTTTTCCCCGAAATCGCGAAGGAAATATCATCACCTCCTTCATCCGTATCATCGTCTTCTACTATC
CCTGTTTTGCCTAAGCCAAGGAAGTCGTCGCCAACCAGATACAGTGAACTAGGCTGCTTAACCCTTCCTAGAAGT
CGAAGCCCTAGTCCACAAAGATCTGTTACGTCTCCAACGTACACACCTGTCGCATTACAAAATACACCAGTCTTG
AAACCTAAGTCTTCAAGCAGAAATGTTTCATCCCCATCATTCAATGCCAAACCTCCACTTCCTATTAAGGCAGTT
ACACGACCACAACTGTCTCTAACTTCCAATTCTAATACAGATTTGGCATTAGCTTCTTCTTCTACAGATACATTA
TCCTCACCGACGAAGACTCCATCAGCAGACTCGCTTCCATTGAGTAACAGCAGCACCGATTTAACGATATCAGAT
AATATCAAAGAAATGGTTAAGGACGAGCCAGCAAAGGATAAAAATTCTGTGGAAACAGATATATTCGTACAACAG
TTTGAAAGTTTAACCCTCGTCCAAAACGCGAAGATTAAGAAGTTCGACAAAGAACTACAAGAAAAGAAGAAGAAA
AATGAAACTACTTCAAAGACAGCAGATAAATTCTCTCAAAAGGGATATTCAGATATTAAAGCAAGCAACAAGGTT
AGCAGGCTAGCTGCATTGTACGAAGAACGTTTACAAGGTTTACAAGTAATGAATGAAATGAAACAAAGGTGGTAA

>YOR297C 0.76 Cold
ATGCTATTGTTTCCTGGCTTGAAGCCTGTTCTTAATGCTTCCACTGTCATTGTAAATCCTGTACGAGCTGTTTTT
CCAGGATTAGTACTTTCAACCAAAAGATCCTTTTATAGCATTAATCGTTTAAATGCTGAAAATAAAATAAACGAT
ATTGCAAATACGTCAAAAGAGGCATCCTCTTCAGTGCAGATGTTTAAGCCCCCAGAGTTCTCTCAATTTAAGGAC
TCTTATCAAAAAGACTATGAGAGAATAGCAAAATATACTTTGATTCCATTAACAATGGTACCTTTTTATGCCTCC
TTTACCGGCGGGGTTATAAATCCTTTACTCGATGCGTCTTTATCGTCCATTTTTTTGATATATTTACAGTATGGC
TTCACAAGTTGTATTATTGACTATATACCGAAGGGAAAGTATCCAAGATGGCATAAGCTCGCCCTTTATTGCCTC
TATGGAGGATCCATGTTGTCCCTGTACGGTATCTACGAATTGGAAACGAAAAATAATGGTTTTGTTGACCTGGTA
AAGAAACTTTGGAATGAAAATGATGACCATTTGTATATATTTGGAAGAAACTGA

>YLR410W 0.73 Cold
ATGAGTGGGATAAAGAAGGAACCGATTGAATCTGATGAGGTTCCTCAACAAGAGACAAAAAATAACTTACCTAGT
GCACCTTCTGAGATGTCACCCCTTTTCCTAAACAAAAACACACAGAAGGCTATGCAATCTATTGCACCAATATTG
GAGGGTTTCAGTCCTAAGACGTCAGCTAGCGAAAATATGTCTTTGAAATTGCCACCTCCCGGTATTCAAGATGAT
CACAGTGAAGAGAATCTCACCGTGCATGATACTCTGCAAAGAACCATAAGCACTGCACTAGGAAACGGAAACAAC
ACCAATACAGTTACAACTTCCGGTTTAAAAAAGGCAGACAGTGAATCAAAGTCAGAAGCTGATCCTGAGGGGCTC
AGCAATTCTAACATAGTCAATGATGCAGATAACATAAATTCCATCTCCAAGACCGGATCGCCCCATTTACCTCAA
GGAACAATGGACGCTGAGCAGACTAACATGGGAACTAACTCTGTACCTACTTCATCAGCTTCTTCACGCAAATCC
TCGACTAGTCATCCCAAACCTAGGCTACCCAAGGTAGGGAAGATTGGGGTATGTGCAATGGATGCAAAAGTCCTT
TCAAAGCCAATGAGACACATTTTGAATCGTTTAATTGAACATGGTGAATTTGAAACTGTCATTTTTGGAGATAAG
GTGATTCTAGATGAAAGAATAGAGAATTGGCCAACTTGCGACTTTTTGATATCTTTCTTTTCTTCAGGATTTCCC
CTAGATAAAGCAATTAAATACGTCAAGTTACGCAAGCCTTTCATTATCAATGATTTAATAATGCAAAAGATTTTG
TGGGACAGGAGATTATGTCTACAAGTATTAGAAGCCTACAACGTTCCAACACCTCCTAGACTGGAAATCAGTAGA
GATGGTGGTCCCCGTGCCAACGAAGAATTAAGGGCAAAATTACGTGAGCATGGCGTAGAAGTTAAACCCGTTGAA
GAACCGGAATGGAAAATGGTTGATGATGATACTTTAGAAGTTGATGGGAAAACCATGACTAAGCCCTTTGTTGAA
AAGCCCGTTGATGGTGAAGACCACAACATTTATATATACTATCATTCTAAGAACGGAGGAGGCGGTCGTCGTCTA
TTCCGTAAAGTTGGTAACAAGTCTTCTGAGTTTGATCCTACTTTGGTTCATCCTCGTACGGAGGGGTCTTACATT
TATGAGCAGTTTATGGATACCGATAATTTCGAAGATGTGAAGGCTTATACGATTGGTGAGAACTTTTGTCACGCT
GAAACTAGGAAATCACCTGTGGTTGATGGTATAGTTAGAAGAAATACTCATGGTAAAGAAGTTAGATATATTACT

GAGTTATCAGACGAAGAGAAAACCATTGCTGGGAAAGTTTCCAAAGCTTTTTCACAAATGATCTGTGGTTTCGAT
TTGCTTCGCGTTTCTGGTAAAAGTTACGTCATTGATGTTAACGGATTTTCTTTTGTAAAGGATAACAAAGCTTAC
TATGATTCATGCGCCAATATATTGAGAAGTACCTTCATTGAAGCCAAAAAGAAGATGGATATGGAAAAGAAGAAT
CTGCCCATTATTCGTGAAGAAAAGGAACAAAAGTGGGTATTCAAAGGACTGGCTATCATTATCCGTCACGCGGAC
AGAACACCAAAACAGAAATTCAAACATTCATTCACTTCGCCTATTTTTATATCGTTACTAAAAGGTCACAAAGAA
GAAGTTGTCATTCGGAATGTAAATGATTTAAAGATTGTTCTTCAGGCCTTAAGAATTGCGTTAGATGAAAAAGCA
GGAAATCCAGCTAAAATTAAAGTGTTAGCAAACGCTCTAGAAAAGAAACTGAATTTTCCCGGTACGAAAATACAA
TTGAAACCGGTTTTAAATAAAGAGAACGAAGTGGAAAAAGTTCAGTTTATTTTGAAATGGGGTGGTGAACCCACT
CATTCTGCCAAATATCAGGCTACGGAACTCGGAGAGCAAATGAGACAAGATTTTGACCTATTGAATAAGAGCATT
CTACAAAACATCAAGATATTTTCATCGTCTGAGAGACGTGTTCTTCATACGGCGCAGTACTGGACCAGAGCTCTT
TTTGGGGCCGATGAATTAGGTAGCGATGAAATCAGTATTAGAAAGGATCTTTTAGATGACAGTAATGCCGCCAAA
GATTTAATGGATAAGGTGAAAAAGAAGTTGAAGCCACTTTTAAGAGAGGGTAAGGAAGCTCCCCCACAATTTGCC
TGGCCTTCAAAGATGCCGGAGCCATATTTAGTCATAAAGCGAGTGGTTGAATTGATGAATTACCATAAAAAAATT
ATGGATAACAACTTTGCTAAAAAGGATGTCAACTCGATGCAAACAAGATGGTGTACTTCTGAAGATCCTAGTTTG
TTTAAGGAAAGGTGGGATAAACTTTTCAAAGAATTCAATAATGCTGAAAAAGTCGATCCTTCTAAAATTTCTGAA
TTGTATGATACTATGAAGTATGATGCTCTTCACAACAGGCAGTTCCTAGAGAACATATTTGACCCGGGTCTTCCA
AATGAAGCTATCGCCGATGAACTCGGCAGTCATTCTTTAGTTGACCGATACCCAATTAACGTTCTCGCCAAAAAT
AATTTTAAGATAATAGACAGTCATAGCATGAACAATTCTGGAAAGAACAGTAGTAATAGTGTTGGGTCGTTAGGA
TGGGTTCTGGAAAGCGGAAAAACATCTACAGCAAGAAACCCCAAATCTTCTTCTCAATTCGATGAGCCACGCTTT
ATGCAGTTGCGGGAGCTGTATAAGCTGGCCAAAGTATTGTTTGATTTTATCTGCCCCAAAGAATATGGTATTTCA
GATGCAGAGAAACTGGATATCGGTTTGTTAACTTCGTTGCCTTTGGCAAAGCAAATACTAAATGATATAGGAGAC
ATGAAAAATAGAGAAACTCCAGCTTGTGTTGCGTACTTCACCAAAGAGTCCCATATTTACACTCTATTGAATATC
ATATACGAATCTGGTATTCCAATGAGAATCGCTAGAAATGCTTTACCAGAACTAGACTACTTGTCGCAGATCACT
TTTGAACTTTATGAGAGTACGGATGCTTCTGGTCAAAAATCGCATTCCATTAGACTAAAAATGTCTCCTGGGTGT
CATACTCAAGATCCGTTAGATGTTCAATTAGATGACAGGCATTATATTAGTTGTATTCCAAAGATTTCCCTGACG
AAGCATTTGGATATGGACTATGTTCAACAGAAATTGAGAAACAAATTTACCAGGGTCATTATGCCTCCGAAATTT
ACACCAGTAAACATTACGAGCCCCAACTTGAGTTTCCAGAAACGCAAAACCAGAAGAAAGTCGGTATCTGTTGAG
AAGTTGAAACGTCCTGCCTCGTCCGGATCTTCATCATCTACCTCCGTTAACAAGACATTAGATTAG

>YPL278C 0.82 Cold
ATGACAGATAACACAACTTCCAGCGACTTAATAAAGAATGTCGAAACGGCTCGCTCCACTATCGATGGTTTAATT
GAATCTCTAGGGTGGATTGAACTAAATTACCGTTGTGAGAGGCAATGCAATTGGGATGAAGTTTGTTACACTCCT
TCCTGGGGCCCATCTCCAATGGGTATGACTGAACCAGGTTCCCACAATGAGGGATTTGGAACCCACTTTGATGAA
TCTCGACAAAGGTTGGTTATTAACAGTAAACTACAGTGTATCAATATAAATGATTTGATGGTTAATCGTAATCAT
TAG

>YIL076W 0.82 Cold
ATGGATTACTTTAATATCAAGCAGAATTACTACACGGGGAACTTCGTGCAATGTTTGCAGGAAATAGAGAAGTTT
AGCAAGGTCACAGATAACACCTTATTATTCTACAAGGCGAAAACCCTTTTAGCTCTAGGCCAATATCAATCACAA
GATCCTACCTCTAAGCTTGGCAAGGTACTAGACTTGTACGTCCAGTTCTTAGATACAAAAAACATTGAAGAATTA
GAAAATTTGTTAAAGGATAAACAGAATTCACCATATGAATTATATCTGTTAGCCACGGCTCAAGCTATCTTGGGT
GACTTAGATAAAAGTTTGGAGACATGTGTAGAAGGGATTGACAATGACGAAGCAGAAGGGACTACAGAATTATTG
CTGCTTGCCATCGAAGTCGCTTTGTTAAACAACAACGTTTCGACCGCGTCCACCATCTTTGATAACTACACTAAT
GCTATTGAGGATACTGTTTCCGGTGACAACGAAATGATTTTAAACCTGGCTGAATCGTACATTAAATTTGCCACA
AACAAAGAAACGGCAACATCCAACTTTTACTACTATGAAGAGCTATCTCAAACCTTTCCTACTTGGAAAACTCAA
TTGGGATTGCTCAATTTGCATTTACAGCAAAGAAACATAGCTGAAGCTCAGGGTATTGTCGAGCTATTACTATCT
GACTACTATAGCGTCGAACAAAAAGAAAACGCCGTATTGTACAAACCTACTTTCTTAGCAAACCAAATTACGCTT
GCTCTTATGCAAGGTCTTGATACTGAAGATTTAACAAATCAATTGGTTAAATTGGATCACGAACATGCATTCATC
AAGCATCACCAAGAAATTGACGCAAAATTCGATGAATTAGTGAGGAAATATGATACGTCCAACTGA

>YDR159W 0.81 Cold
ATGAACACATCATTTGGCTCAGTAGTGCCTTCCACTAATTTCAATTTTTTCAAAGGGCATGGAAATAATGATAAC
ACTAGTGCCAATAGTACTGTTAACAACAGCAATTTCTTTTTAAATAGCAATGAAACGAAACCTTCGAAAAATGTC
TTCATGGTGCACTCTACCTCTCAAAAGAAGTCGCAGCAACCGTTACAAAATCTCTCACACTCACCTTCCTACACG
GAAAATAAACCAGATAAAAAGAAAAAATATATGATAAACGATGCCAAGACCATTCAACTTGTAGGTCCACTGATT
TCCTCTCCTGATAATTTGGGATTTCAGAAGAGATCGCACAAAGCAAGAGAATTACCAAGGTTTTTGATTAATCAA
GAACCTCAATTGGAGAAAAGAGCTTTTGTACAAGATCCCTGGGACAAGGCTAATCAAGAAAAAATGATCTCATTG
GAAGAATCCATAGATGATCTCAATGAATTGTATGAAACACTAAAAAAAAATGAGAAATACTGAGCGATCGATAATG
GAAGAAAAAGGGCTAGTGGATAAAGCTGACTCAGCAAAGGATCTTTACGATGCTATAGTATTTCAAGGTACCTGT
TTAGATATGTGTCCCACATTCGAGAGATCAAGAAGAAACGTTGAATATACTGTGTATTCATATGAAAAAAATCAA
CCAAATGATAAAAAAGCTTCTCGAACTAAAGCATTGAAGGTCTTCGCGAGGCCAGCAGCAGCAGCAGCTCCTCCT
TTACCTTCTGATGTTAGGCCACCCCATATTTTAGTCAAGACATTGGACTATATTGTAGATAACTTATTAACAACA
TTGCCTGAAAGCGAAGGATTCTTATGGGATAGGATGAGATCCATAAGGCAGGACTTTACATATCAGAACTATTCA
GGACCTGAGGCGGTAGATTGTAATGAGCGTATTGTTAGGATACATCTTCTGATCTTGCATATCATGGTGAAATCA
AATGTGGAATTTTCGCTTCAACAAGAACTAGAACAATTGCATAAATCCCTTATCACGTTATCAGAAATATACGAT
GACGTTCGTTCCAGCGGCGGAACATGCCCAAATGAGGCTGAATTTCGAGCTTATGCTCTCCTGAGTAAAATAAGA
GACCCTCAATACGATGAGAATATCCAAAGATTACCAAAACATATTTTCCAAGATAAATTAGTTCAAATGGCTTTA

TGTTTCAGAAGGGTTATATCTAACTCAGCTTATACTGAACGCGGGTTTGTTAAAACTGAGAATTGTTTAAACTTC
TACGCAAGGTTTTTCCAGTTAATGCAATCACCCAGCTTGCCATTATTAATGGGATTTTTTTGCAAATGCATCTG
ACGGATATTAGATTTTATGCGCTAAGAGCTTTATCACATACTTTAAACAAAAAGCATAAACCAATACCATTTATT
TATTTGGAAAATATGTTACTCTTTAATAATCGGCAAGAAATAATTGAATTTTGTAATTATTACTCAATTGAAATC
ATAAATGGGGATGCAGCCGATTTGAAAACATTGCAACATTATTCTCACAAATTGTCAGAAACACAACCTTTAAAA
AAAACATACCTTACCTGCTTAGAAAGGAGATTACAGAAAACTACTTACAAGGGTTTAATTAATGGCGGTGAAGAT
AATTTGGCTTCTTCGGTTTATGTGAAGGACCCAAAAAAGGATAGAATACCTTCTATCGCAGACCAATCTTTTTTA
ATGGAAAATTTTCAAAACAACTATAACGAAAAGTTGAACCAAAACTCATCCGTTAAACCACAGATCAATACCTCT
CCAAAAAGAGTTGCAACGCGGCCCAACCACTTTCCTTTTTCACAGGAATCTAAGCAATTACCTCAAATATCTCAA
TCTCATACTTTATCAACAAATCCTTTATTAACGCCACAGGTTCATGGAGACCTATCGGAACAGAAACAACAACAA
ATTAAAACCGTCACAGACGGAGGCTCCCCATTTGTTTTTGATCAATCAGCACAGAATTCTACGGTGGAAGCTTCC
AAGGCTCATATGATATCCACGACCAGCAATGGTGCATATGATGAGAAGTTGAGTTCAGAGCAAGAAGAAATGAGG
AAAAAAGAAGAACAACGAATAGAAGAAGAGAAAACTCAATTGAAAAAGAAGCAAGAAAATGCGGACAAACAAGTA
ATCACTGAACAAATTGCAAATGACTTAGTAAAAGAAGTGGTAAACAGTAGCGTGATTAGTATTGTTAAGCGTGAA
TTTTCAGAGGCTAATTATAGGAAGGACTTCATTGACACAATGACCCGTGAACTTTATGACGCATTTCTTCATGAA
AGGTTGTATCTGATATATATGGATTCTCGTGCTGAATTGAAAAGGAATTCCACTCTAAAAAAAAAGTTCTTTGAG
AAATGGCAAGCATCTTATTCTCAAGCAAAGAAGAACCGTATATTAGAAGAAAAAAAAAAGGGAAGAAATTAAACTC
GTAAGCCACCAATTAGGAGTACCAGGTTTTAAAAAATCTACATGTTTGTTTAGAACCCCGTATAAGGGTAACGTA
AATTCATCATTTATGCTATCATCTTCAGATAAGAATCTAATATTTTCACCTGTAAATGACGAGTTTAATAAGTTT
GCGACTCATTTAACCAAGATTTCAAAACTTTGGCGGCCGTTAGAGATGCAATCAATCTATTATGATAACCTTACG
AAAAAGTTCCCCTCCAATTCTCTTACTCCGGCAAATTTATTCATTTATGCGAAAGATTGGACATCACTCTCAAAC
CGTTGGATCTTAAGTAAGTTCAATCTGCAAACAGCACAGGATTCAAAAAAGTTCAGCAATAATATTATCTCTAGC
AGAATAATTTGTATTGATGATGAATATGAACCATCTGATTTTAGTGATTTGCAATTATTGATATTTAATACAGGT
GTTACGAATCCAGATATATTCGATTTGGAGATGAAACTGAAAGATGATGGTGAAGAACTGATAAAGCTAATCACT
GGTATTTCTCTAAATACAAATATATGCTTTTCCCTTTTGATTATTTATTGGGAATCCGCAGAAAATACTTTATCG
GAAAGTACCATTAAACACTTACTAAAATTAAACCGAATATCTAAAAACTATAGTAGTGTTATTGAACGCATTGAC
TTAATGAATCTCACCGAAGAATCCCCTCATAAGTGTTTAGAGGACAAATTATCTGAAATATCACATTCTTATGTG
TACAAGTTGACAGAAAGGGGAAAATATGATAAAACACTTCGTCAGAAAAGGTCATTGGCCGGCATTCACTCACGT
AGCACTCAACTACAAACTACAAAGGACATTGATCAAAAGATGAAGAAAATGCTGGAAAAAGAGAAAAACAAGTAC
CAACAACAAATAGGCGAAAGGAACACCTATGCGCATCTAGAATCGCATATAGATGCATCACCAAGAAGCAAGAAA
AGGAAATTGCCAATACTCCTGTCAACTTCTCATTCGAGCCAATTTAAAACTCCTCTAGCTTCTAGATTAAACACC
TCTGGTTCGTCTACTTCACCACCTTTACCATCTCATTTGGCGATGAAATTCAGGAAAAACTCGAGAGTTACTAGT
TTACATACCGTATTACCCGTTAGCACACCAAGCCATAGTAATAACATACCAGCTGCAAGTTTTAGCGGGAATAAC
ACCACCGATATACAGTCCCAACAATTAATCGAAAACCAGAAGAGTACATCTGTATATTTGAATAACGTTTCTGAA
AGGATTCTGGGAAATCAAGAAATATGTCAAACACCAATTAATCCTGTTACTCCTGTGCTAGATGGGGCCGATCAA
GGTAAGGAGGACATTCCTGATAGTATATTAGAGCTGAAGATCTTGATCGATTCTGTCAAGAAGAAAGTAAATAAT
GATTAA

>YKR003W 0.73 Cold
ATGGGCTCCAAAAAACTGACCGTAGGATCTGATTCGCACCGGTTGAGCAAATCCAGTTTTTCAAGTAATAAGTCG
TCACATTCAGCAACAAAAGATCAGCCAATTGATACCGACGATATTGATGAAGACGATGAATCTGGTCATAATATT
ATCTTGAACATCATCTCACAATTGAGACCAGGCTGCGATTTGACCAGGATCACCTTGCCTACTTTTATTTTAGAA
AAAAAATCGATGCTTGAACGTGTCACAAATCAGTTACAATTCCCTGAGTTTTTGTTACAGGCGCATTCAGAAAAG
GACCCCTTGAAAAGATTTTTGTACGTAATGAAATGGTATTTGGCAGGCTGGCATATTGCTCCAAAGGCTGTAAAA
AAACCATTGAACCCAGTCCTTGGTGAATATTTTACAGCTTATTGGGATCTACCAAACAAGCAGCAGGCATATTAT
ATATCTGAACAGACAAGTCACCATCCTCCAGAATGTGCATATTTTTACATGATTCCTGAATCTTCGATTAGAGTG
GATGGGGTCGTTATTCCTAAATCTAGATTTTTAGGTAATTCAAGTGCGGCCATGATGGATGGATCAACAGTCTTG
CAATTTCTGGACATAAAGGATGGAAACGGAAAGCCCGAAAAGTATGTTCTTACACAACCAAATGTATATGTAAGA
GGAATTCTTTTTGGAAAAATGAGAATCGAACTTGGAGATCATATGATAATTAAATCTCCTAACTTCCAAGCTGAT
ATAGAGTTCAAGACAAAGGGATATGTTTTTGGAACTTACGATGCAATCGAAGGGACTGTAAAGGATTATGATGGC
AATGCGTATTACGAAATATCTGGTAAATGGAATGACGTTATGTATTTAAAGGACTTAAAGCAACCTCGTTCTTCA
CCAAAAGTTTTCCTTGATACTCATAAAGAGTCACCATTGAGACCAAAAGTTCGGCCATTGAGCGAGCAAGGTGAA
TATGAATCCAGAAAACTGTGGAAAAAGGTTACAGATGCGTTGGCCGTTCGCAATCATCCTGTTGCGACAGAAGAA
AAATTTCAGATTGAAGATCACCAAAGACAGTTAGCCAAAAAACGCATTGAAGATGGCGTAGAATTTCATCCAAAG
TTGTTTAGAAGATCAAAGCCTGGCGAAGATCTTGACTATTGTATTTATAAAAATATCCCTGTCGACGAGGACCCT
GAGAAGCAAATACGAAGCATATTGCAAATAGCACCTATTTTACCTGGTCAACAATTTACCGACAAATTTTTCATC
CCCGCATTTGAGAAAATAAAATCACAAAAGAAGATGATTGAAAACGAAAAGCAGAACCCAGCAAAACAATAG

>YDR279W 0.77 Cold
ATGACCGTTTCCAACATTGGGGGGGAAGAACGACTAATAATTTTACCAGACGATTATGAAACTTCGAAAACTATA
AACACTTTTTACACTGCCGCCACCTTCCAATATTACATCCAAACCTCGTATCGAGCTCTTCGAAAATATTAATGGA
AAACTTTACGAAATAAGATCTTTTCAATTCGGCAAAGGGCCCTCGTATTCACATGAGGAAGATTTGGCAAATGAT
AAATATCACTATACTAAGGAAAATCACCCGATCAAATCAACTTTCATTGTAAATACGTCTGATCCCACCGATGGT
TATGTTTTTAACTCAAGCAAAATACACTTTTGTTCTTTATACGATATTGCTTTTAGTTTGATTGGATTTTACTAT
AGAAACAGTGTTTCAGCAGATGAACAAGATTACTCCAATTCAAGTGATACTGGTGAAAACCAAAAAAGCAACAGC
AAAACCAATGAGAAATTTCTTACGGTGCGTGATTACCATGATTTCTTAACGGATAACCATGACAAGAATTGGGAA
AACATTTCTTTAAGTCGTCTCAAGAGTGGCTTAGCAAAAGTTAGTGAAACCATTGAGGAAGCTGGTGACGTCTAT

TACAAGATCACATCTGCAATGATAACACAATTTTTGTTAGGCAAGGTATCAAAAATTGTAGAAAACTTTCCTCCA
AGTATCCCTACACTTAAAAATGCCCCAACGGAAATAAAACAGTGCTACAAGGTAGTTATGGCTACAAATCTTTTA
GTTTCCCTGATTCCAAGGGCGGCCTACCATAATTTACTTACCTTTTCACCTACAATGGATAGTGGTTGCCTAAAT
CCGGATATAAAAGCTAGCTTCATAGAACTCGAAAACTACGAGACTACGAACGAATTACAGAATGCTGAAAGAGAA
TTACTGATGAAAAGTGCCATGAATGTAGGCCTAAATTCGAATGGCAGGGTTTCATTGCCAGTGAAAAAAGTTACC
AAAAAAATAGTTCAAAATAAAAAACCAAAAGTAGCCATAGGAAAAGGGGCCATTGATGGATTTTTTAAACGTAAG
TAG

>YHR215W 0.82 Cold
ATGTTGAAGTCAGCCGTTTATTCAATTTTAGCCGCTTCTTTGGTTAATGCAGGTACCATACCCCTCGGAAAGTTA
TCTGACATTGACAAAATCGGAACTCAAACGGAAATTTTCCCATTTTTGGGTGGTTCTGGGCCATACTACTCTTTC
CCTGGTGATTATGGTATTTCTCGTGATTTGCCGGAAAGTTGTGAAATGAAGCAAGTGCAAATGGTTGGTAGACAC
GGTGAAAGATACCCCACTGTCAGCAAAGCCAAAAGTATCATGACAACGTGGTACAAATTGAGTAACTATACCGGT
CAATTCAGCGGAGCATTGTCTTTCTTGAACGATGACTACGAATTTTTCATTCGTGACACCAAAAACCTAGAAATG
GAAACCACACTTGCCAATTCGGTCAATGTTTTGAACCCATATACCGGTGAGATGAATGCTAAGAGACACGCTCGT
GATTTCTTGGCGCAATATGGCTACATGGTCGAAAACCAAACCAGTTTTGCCGTTTTTACGTCTAACTCGAACAGA
TGTCATGATACTGCCCAGTATTTCATTGACGGTTTGGGTGATAAATTCAACATATCCTTGCAAACCATCAGTGAA
GCCGAGTCTGCTGGTGCCAATACTCTGAGTGCCCACCATTCGTGTCCTGCTTGGGACGATGATGTCAACGATGAC
ATTTTGAAAAAATATGATACCAAATATTTGAGTGGTATTGCCAAGAGATTAAACAAGGAAAACAAGGGTTTGAAT
CTGACTTCAAGTGATGCAAACACTTTTTTTGCATGGTGTGCATATGAAATAAACGCTAGAGGTTACAGTGACATC
TGTAACATCTTCACCAAAGATGAATTGGTCCGTTTCTCCTACGGCCAAGACTTGGAAACTTATTATCAAACGGGA
CCAGGCTATGACGTCGTCAGATCCGTCGGTGCCAACTTGTTCAACGCTTCAGTGAAACTACTAAAGGAAAGTGAG
GTCCAGGACCAAAAGGTTTGGTTGAGTTTCACCCACGATACCGATATTCTGAACTATTTGACCACTATCGGCATA
ATCGATGACCAAAATAACTTGACCGCCGAACATGTTCCATTCATGGAAAACACTTTCCACAGATCCTGGTACGTT
CCACAAGGTGCTCGTGTTTACACTGAAAAGTTCCAGTGTTCCAATGACACCTATGTTAGATACGTCATCAACGAT
GCTGTCGTTCCAATTGAAACCTGTTCTACTGGTCCAGGGTTCTCTTGTGAAATAAATGACTTCTACGGCTATGCT
GAAAAGAGAGTAGCCGGTACTGACTTCCTAAAGGTCTGTAACGTCAGCAGCGTCAGTAACTCTACTGAATTGACC
TTTTTCTGGGACTGGAATACCAAGCACTACAACGACACTTTATTAAAACAGTAA

>YDR541C 0.69 Cold
ATGTCTAATACAGTTCTAGTTTCTGGCGCTTCAGGTTTTATTGCCTTGCATATCCTGTCACAATTGTTAAAACAA
GATTATAAGGTTATTGGAACTGTGAGATCCCATGAAAAAGAAGCAAAATTGCTAAGACAATTTCAACATAACCCT
AATTTAACTTTAGAAATTGTTCCGGACATTTCTCATCCAAATGCTTTCGATAAGGTTCTGCAGAAACGTGGACGT
GAGATTAGGTATGTTCTACACACGGCCTCTCCTTTTCATTATGATACTACCGAATATGAAAAAGACTTATTGATT
CCCGCGTTAGAAGGTACAAAAAACATCCTAAATTCTATCAAGAAATATGCAGCAGACACTGTAGAGCGTGTTGTT
GTGACTTCTTCTTGTACTGCTATTATAACCCTTGCAAAGATGGACGATCCCAGTGTGGTTTTTACAGAAGAGAGT
TGGAACGAAGCAACCTGGGAAAGCTGTCAAATTGATGGGATAAATGCTTACTTTGCATCCAAGAAGTTTGCTGAA
AAGGCTGCCTGGGAGTTCACAAAAGAGAATGAAGATCACATCAAATTCAAACTAACAACAGTCAACCCTTCTCTT
CTTTTTGGTCCTCAACTTTTCGATGAAGATGTGCATGGCCATTTGAATACTTCTTGCGAAATGATCAATGGCCTA
ATTCATACCCCAGTAAATGCCAGTGTTCCTGATTTTCATTCCATTTTTATTGATGTAAGGGATGTGGCCCTAGCT
CATCTGTATGCTTTCCAGAAGGAAAATACCGCGGGTAAAAGATTAGTGGTAACTAACGGTAAATTTGGAAACCAA
GATATCCTGGATATTTTGAACGAAGATTTTCCACAATTAAGAGGTCTCATTCCTTTGGGTAAGCCTGGCACAGGT
GATCAAGTCATTGACCGCGGTTCAACTACAGATAATAGTGCAACGAGGAAAATACTTGGCTTTGAGTTCAGAAGT
TTACACGAAAGTGTCCATGATACTGCTGCCCAAATTTTGAAGAAGGAGAACAGATTATGA

>YHL047C 0.76 Cold
ATGATCGAAGTCCCAGAAGACAATCGTTCTAGCCAAACAAAGCGTAAGAATACTGAAAAAAATTGTAATGAACTG
ATGGTTGATGAAAAGATGGATGACGATTCGTCGCCCAGAGATGAAATGAAAGATAAATTAAAGGGAACAAAATCA
TTGATTATTCGTAAGTCTGAGCTAATGGCTAAAAAGTATGATACTTGGCAATTAAAAGCTATATTCCTGTTTTCT
GCATTCATTTGCACTTTCGCTTATGGATTAGATAGCAGCATAAGGGGTACATATATGACCTACGCAATGAACTCG
TACTCCGCACATTCTCTCATTTCTACAGTAAGCGTCATAGTTCTAATGATATCTGCTGTTTCACAGGTGATCTTT
GGAGGGTTATCAGATATCTTTGGAAGGCTAACGTTGTTTCTTGTATCCATTGTACTTTACATTGTGGGTACAATA
ATTCAATCTCAAGCATATGATGTCCAGAGGTATGCTGCTGGAGCTGTCTTTTATTATGTGGGACTTGTCGGTGTT
ATGCTACAGGTTGTTTTGATGTTGTCTGATAATTCTTCATTAAAATGGAGGCTATTTTACACATTGATACCTTCA
TGGCCCTCGATTATCACTACGTGGGTCTCAGGTAGTGTAGTGGAGGCAGCAAACCCGTTAGAGAACTGGTCGTGG
AATATTGCTATGTGGGCGTTCATTTTCCCTCTATGCTGTATCCCATTGATTTTGTGTATGCTCCATATGAGATGG
AAAGTAAGAAATGACGTGGAATGGAAAGAATTACAGGATGAAAAATCGTACTATCAAACGCACGGATTAGTACAA
ATGCTGGTACAGTTGTTCTGGAAGCTAGATGTTGTCGGTGTGTTGTTGTTTACTGCTGGTGTTGGTTGTATTCTT
GTACCACTCACTCTTGCTGGTGGCGTCTCTACAAATTGGAGAAATTCAAAGATTATCGGGCCCTTTGTGCTTGGC
TTTGTCCTGGTTCCAGGCTTCATATATTGGGAAAGTCGACTTGCTTTGGTTCCTTTCGCTCCATTCAAGCTGCTA
AAAGATCGTGGCGTGTGGGCCCCATTGGGAATTATGTTTTTTATCTGCTTTGTATATCAAATGGCTGCTGGGTAT
CTCTACACCATTCTAGTAGTTGCGGTAGATGAGAGCGCTTCCTCAGCTACAAGAATAATAAACTTATACTCTTTT
GTGACTGCTGTTGTTGCTCCATTTCTGGGGCTCATCGTTACGAGATCATCTAGATTGAAGTCGTATATTATTTTT
GGTGGCTCACTTTATTTCATTACTATGGGACTATTTTACCGCTACAGGTCAGGTCAAGACGCCGATGGAGGTATA
ATTGCTGGTATGGTTATTTGGGGTCTCAGTAGTTGTTTATTCGATTATCCTACAATTGTTTCTATTCAGTCGGTA
ACATCCCATGAGAATATGGCTACTGTCACAGCATTAAACTATACAGTTTTCAGAATAGGTGGAGCAGTTGCAGCT
GCCATTTCCGGTGCCATATGGACGCAGTCATTGTACCCTAAATTATTGCACTACATGGGAGACGCTGACCTGGCT

ACAGCAGCATATGGCTCACCACTAACTTTTATTCTTTCCAATCCCTGGGGTACTCCAGTTAGGTCTGCGATGGTT
GAAGCTTACAGACACGTTCAAAAATACGAAGTTATTGTTGCGCTTGTATTTTCAGCGCCTATGTTCCTTCTAACA
TTCTGCGTAAGAGATCCTCGGTTGACTGAAGATTTTGCCCAAAAGTTACCTGACAGAGAATATGTCCAAACCAAG
GAAGATGATCCTATCAATGACTGGATCGCGAAACGTTTTGCAAAGGCCTTAGGAGGTCATAAGAAAGATTTACAA
AACCCCAACAGGATATGTGTTCGAAAAAATGATCTCTGA

>YNL009W 0.69 Cold
ATGAGTAAAATTAAAGTTGTTCATCCCATCGTGGAAATGGACGGTGATGAGCAGACAAGAGTTATTTGGAAACTT
ATCAAAGAAAAATTGATATTGCCATATTTAGATGTGGATTTAAAATACTATGACCTTTCAATCCAAGAGCGTGAT
AGGACTAATGATCAAGTAACAAAGGATTCTTCTTATGCTACCCTAAAATATGGGGTTGCTGTCAAATGTGCCACT
ATAACACCCGATGAGGCAAGAATGAAAGAATTTAACCTTAAAGAAATGTGGAAATCTCCAAATGGAACAATCAGA
AACATCCTAGGTGGAACTGTATTTAGAGAACCCATCATTATTCCAAAAATACCTCGTCTAGTCCCTCACTGGGAG
AAACCTATAATTATAGGCCGTCATGCTTTTGGTGACCAATATAGGGCTACTGACATCAAGATTAAAAAAGCAGGC
AAACTAAGGTTACAGTTTAGCTCAGATGACGGTAAAGAAAACATCGATTTAAAGGTTTATGAATTTCCTAAAAGT
GGTGGGATCGCAATGGCAATGTTTAATACAAATGATTCCATTAAAGGGTTCGCAAAGGCATCCTTCGAATTAGCT
CTCAAAAGAAAACTACCGTTATTCTTTACAACCAAAAACACTATTCTGAAAAATTATGATAATCAGTTCAAACAA
ATTTTCGATAATTTGTTCGATAAAGAATATAAGGAAAAGTTTCAGGCTTTAAAAATAACGTACGAGCATCGTTTG
ATTGATGATATGGTAGCACAGATGCTAAAATCAAAGGGCGGGTTTATAATCGCCATGAAGAATTATGATGGCGAT
GTCCAGTCTGACATTGTGGCACAAGGATTTGGGTCTCTTGGTTTAATGACGTCCATATTGATTACACCTGATGGT
AAAACGTTTGAAAGCGAGGCTGCCCATGGTACGGTGACCAGACATTTTAGAAAACATCAAAGAGGCGAAGAAACA
TCAACAAATTCAATAGCCTCAATATTTGCCTGGACAAGGGCAATTATACAAAGAGGAAAATTAGACAATACAGAT
GATGTTATAAAATTTGGAAACTTACTAGAAAAGGCTACTTTGGACACAGTTCAAGTGGGCGGAAAAATGACCAAG
GATTTAGCATTGATGCTTGGAAAGACTAATAGATCATCATATGTAACCACAGAAGAGTTTATTGATGAAGTTGCC
AAGAGGCTTCAAAACATGATGCTCAGCTCCAATGAAGACAAGAAAGGTATGTGCAAACTATAA

>YCR015C 0.8Cold
ATGAAAACCATTATTATTTCAGATTTTGATGAAACAATCACAAGAGTTGACACAATCTGCACCATTGCTAAATTA
CCGTATCTACTGAACCCGCGGTTGAAACCTGAGTGGGGTCATTTTACCAAGACATATATGGACGGATACCATAAA
TACAAATACAATGGAACGAGATCACTGCCGTTGTTATCTTCAGGCGTACCTACGATAATTTCGCAGTCTAACTTC
AATAAGTTGTTTGCGGACGAATTAAAATATCAAAATCATAACAGGGTTGTTGAGTTAAATAGTGTAAATGAAATT
ACAAAACAACAGATTTTTAAGTCGATTTCTTTGGATCAAATGAAAACGTTCGCCAGAGATCAGAATCACGAAGAT
TGTTTACTAAGAGATGGTTTCAAAACGTTTTGCTCTTCAGTTGTCAAAAATTTTGAAAGTGATTTTTACGTTTTA
TCTATAAATTGGTCAAAAGAGTTTATTCATGAAGTCATTGGTGACAGAAGACTTAAAAATAGTCATATTTTTTGT
AACGATCTAAAAAAAGTTAGTGACAAGTGTTCTCAAAGCTACAATGGTGAATTTGATTGTCGGTTATTGACAGGC
TCTGATAAGGTCAAGATACTGGGTGAAATATTAGATAAGATCGACTCAGGTTGCAATAAAGAAGGAAATTCTTGC
AGCTATTGGTATATAGGGGATAGTGAGACTGACTTGTTGTCCATATTACATCCATCTACTAATGGTGTACTGCTA
ATAAACCCGCAAGAAAATCCTTCCAAATTCATAAAAATTACCGAAAAGATTATTGGTATCCCAAAGGATAAAATC
TCGAGTTTTGAAGCTGATAATGGTCCAGCGTGGTTACAGTTTTGTGAAAAGGAAGGCGGTAAAGGTGCCTACCTT
GTGAAATCCTGGGACTCTTTAAAAGATTTGATCATGCAGGTAACAAAAATGTAG

>YJR005W 0.69 Cold
ATGTCCGATCAAAAAGTTTTTGCCAGATATAAAGCAAATGAAATCGTCACAGATCTGCAACATTTTGGAGTAAAA
AAGTTTAAATCGAATATAACAAGAAGGAAAAATGCTCTGAGAAAAATTATCGCCAATTTGGTTCTAGGAAACTAT
GGCGAAATGTCCGTTCTGTTTTCTGAGCTCTTGAAATTTTGGCAAATTGAAGATGATCTAGAAGTGAAAAGAATT
TGCCATGAGTATATTAGGGTGATCGGCGCATTAAAACCTCAGCAGGCCAGAGAGGCTTTGCCATTTATAATGGAC
GATTTCAAAAGCAGAGATGAAAAGTTACAAATAATGGCACTAAGAACTCTGGTATTAGTCCCGGTGAAAGAGCTA
TCTGATCAGGCCTTCGACTGTATCATTTCATTGGTAAATCACAAGTCTCCACCTGAACAAGTGACGAGGACAGCA
ATTTACGCACTGTTGGATTTAGATGAAATTGATCATGAAAGGGTATTGGGGTTATCAAGCATTCTACATGACATT
GTAAAAGCACAGTCGAGCTCTCCAGAAGTCATTGTAGCTGCATTACATACTTTATATTCCATTCACGAAAAAAAC
GCTAACATGGAACCCTTCAGGATTCCCTTGGAACTCGCTTTTGACATGCTAGAGCTTTTACCCGAATTAAATGAG
TGGAATAAGGCTACTGTTTTGGAAGTTTTGACGACTTCGGTTGTGCCGCAGCATTATTTGGATACCCATGAAATG
ATCGAACTAGCTTTACCGTACCTGCAACAAGTAAACACATACGTAGTGTTGAACTCTTTAAAATTCATCATGTAC
TTGTTGAATTATGTTGACGTCATCAAAGAAACTCTAGCCGAAAAGCTATCTAACTCTGTAATAGCTTTGTTAGAT
AAGCCGCCTGAATTACAATTTTTAGTATTAAGAAACGTTATTCTTCTTTTATTGAGTAGGGAGTCATCTCTACTC
AGGCTAGACATTTCGTATTTTTTCATCGAGTACAATGACCCCATATACATCAAAGACACGAAATTAGAGTGTTTA
TATCTTTTAGCTAACAAGGAAACATTACCAAGAATTCTAGAAGAGTTGGAACAATATGCTACAGATATTGATATT
CAAATGTCAAGGAAATCAGTCAGAGCCATTGGTAATCTGGCTGTTAAGCTAGATGAAGACTCTGTACACGATTGT
GTCGCTGTTCTACTAGATTTGTTAGAATTCGGTGTTGATTATGTCGTCCAGGAGATTATTTCGGTTTTCAGAAAT
ATTTTGAGGAAATATCCCAATAATTTTAAAGCAAACGTAACAGAACTAGTCAAACACACTGAAGTTGTACAGGAA
CCTGAATCAAAAAACGCTATGATCTGGATAATAACGCAATATTCGGATGTAATTCCAAACTACCTGGAGTTATTT
AGAGTTTTTTCCTCAAACATGTTTAGTGAGACGCTAGAAGTGCAATTTTCCATTCTGAACTCAGCAATAAAATTC
TTCATCAGAAGTCCTACAAAGGAAACTGAAGAACTTTGTATGGATTTATTAAAAGGATGCATCGATCACGAAAAT
AATCCTGACTTAAGAGATAAAACACTGATGTATTGGAGATTGCTGTCATTAACGAAAACGTCTCGTATATCAAAC
GCGATAACTTTTGAATCTTTAAAATCAGTACTGGACGGAGAGTTGCCACTAATAGAAATGAACACGAAATTAGAT
CCCACCGTTTTGGAGGAATTGGAGCTAAACATTGGTACAATAGTATCGATTTACTTAAAACCTGTTTCTCATATC
TTTAGATTGAATAAAACCAAACTGCTACCACAAAGTCCTATTTTGAATCCAAACAAAGACCTCTTGCCAGTTGTA
GGTAACTCATTTCCTCCAACTGGAGCTAATAGGGACCGTCAAAATTCAGAAAGCCAGTCCTCGACGAAGTCCAGA

AAGACTGCTATGATGGATGATTATGATAAGCCCGCTGAAAAAATTAATCAGTTGAAAGGTAAGCGTAAATCTAGT
TCAAACAACCCCTCAAAACTATCACGGAAACCCTCAACTTTATTAAGAAAACTCTCTATGAAACGGCCCTTTTCG
TGA

>YKL034W 0.79 Cold
ATGGAAATCGATGGCAACACTCTGGTGTTCATAATAGTGATCCTTTTCCTATTTTTCTCCTCACCGGGTGGTGAT
GGTGTGTCTTCTCAATATGAGTTCAATCAACTCCAGAGACTCAAGCAGCAGTTCCGAACAGAGCACAATACGTTC
GTCAATATGACATATACAGATAGTTTTCGAAATATTACCGGGCTTAAACTCAGCTACCAAGACATGCTGAATAAT
CCTCTCCAAAATGCCACTTACCCACTACCAGGAAAAGATTATGATCGATGGTTTCCCAATCAAAACTACATGGTT
TTGCCCAACGATGTCATAGAGGCCATAAATACTGAAGTCTGGAATACTTCGAATGATGACGCTTCCAACCTTTTC
CCTCCCAATATTACAAGCACCCTGTTAGGTAAAATCGACCTTGTATCAAATAACAAGTACGAAAAGATAAGGATG
CCTGTACCCAGATTCTATGAGCCGGCAACTGATTTTTCAGAGGATATTCCTCCTGAAGGTGAGACGTACTGGTCT
GAGTGGCCTTCGTATGGCGAACTTCATAATGTGAGTTTTCAGCATGGCGAAATAGCTATTCAAATTTCTCATATG
AGCAATCTTCAGGACAATAATAATTACTTAAGGAGAAATTTCATAAATAAGAAAAATGACCGTTGGAAGTTGTTA
AATCTTCAGATTGATTTCTCAGATAAGGCTGAAAAGGAAAAGCATTCCATATACTCGAAGGCTGTATATGACATT
CAACGTGGTAGAATTCTTTCTATTTCTCAGAGTTCTAAATTTCATTCATTGTTCGCCCTTCCTCATTACATGTCC
TTCCAGAATGATTATAATGAGAAAATATTTAATGATGTCAAGGAACTCGTTGATGAATTTTGGAATTTCACGGAT
TACACAGATGTCATGACCATGAAAGACGTACAGGATGCTTATAATAATGCCAACTTTAAATGTGAGTATTTGATT
TTTCTTCAGTTGGAACCATGGAATCAGTACACAAGAGATCAGATCAAGTTAATAGATGATGAATTGAATTGGCCC
CTGGGGCGTCCGGCAAATCTGTCTAGTTTACCGCCCATAAATGTTGTCTCTGGATTACTGTATTCTCCAGACTGT
GGCGTACGATTAGGGCTTCATAACGTAAAGGGTACACGGTACGAATTAAAAATAATGTCGATCAGAAAACATCTA
TTGTTCGGTATTGCATTATTTGCAGCCCAGATATACTTATTGCTTACTCAGATGCATCATACAAATACCCCCTCC
ATGGTCAACAAAATCTCATTCTATTGTTTCTCAATGATAAACTTAGTCGATGGTTCCTTGGCCACTTTGTACTTT
GTCGCAGCTAGTGTCGTTCCCGAATTGTACTTACCCCTAGTTATAAGTGCTTTTTCGTGCTTTATCTTGGCATCT
ATTTTTGAAATACGCTACTTAATTTCAATTTACGCTTCACAGGTTAACGAACAGAATGTTGGGATTATCAACCTC
CTGCGTGGTAACACTGGCACATATGATGAGAATAGGCCAAGACCCGCATTCATTCCCGATGAAGGGTCTATCGGT
GGCTCGTTATATGGTAGGTTTTTCTTTATGCTAATCATTTTCACGTTTTTAATATTGAGTTCAACTTCATGGCCC
CGTCAGCTAAGGATGGTATTTGAGTATATCCTAATCTTCATATTAAACTCATATTGGATCCCTCAAATTTTCCGT
AACGCCGTTAAAGGTATTCCTTCAAGAAGAGAAAGAGCAAGATCTAGCATTGGAGGAAATAGAAGTCAAAACAAA
ATGCCATTATTGTGGAGTTTTGTAATTGGTACAACAATAATTAGAAGCTTGCCCGTTGTGTATGTTTTCACTTAC
TCCTCTAATGTGTTCAGGCACCATAAAGATGTTCATTTCGTCGTATTTTTATCATTGTGGCTACTGTTTCAAATT
AGTATACTGTATTCTCAAGATGTATTGGGATCGCGCTGGTTCTTGCCTAAGCACACAATACCTGATGGATATTCG
TATTTCAAGCCCCTTTCAAACGAGTATATATCGGAGCATGGTGGTGGAACTGCTGAACATACCGTTGATTGCGCG
ATATGTATGTCTGATGTTCCAATTTACATAGAGGAAATTCCAGAAACTCATAAGGTGGATCAGCACTCTTACATG
GTAACGCCTTGTAATCACGTATTTCATACTTCGTGTCTAGAAAACTGGATGAATTATAAGTTACAATGTCCTGTG
TGTAGGTCACCATTACCTCCGTTGTAG

>YGR283C 0.82 Cold
ATGGCAGTAAAACATAAATCCGAGAGCCTTAAGCACGAAGAGGGGGCTGCCAAAAAGGCAAAGACAGGCCTATTG
AAGCTAAAAAAAATCATGGATATAGAATCTAATGTGGTCAAATATTCCATATGCATACCGACAACTGTTATCGAC
AATTGTAATAATTTGGAGCAAGTTACATTTACTGCATATCAAATAGCTCGGACAGCTGTATTGTTCAATGTTCAA
GAAATCATAGTTCTAGACCAGTCAAAAGATAAAAAGCATGAAAAAAAATCAAGATCAAAGGAGACTATCTCTGAT
TGTTTATTACTTGCCACACTATTACAATATTTTGTAACGCCTCCAAACTTACTGGATACTACATTCAAAAAAAAA
AACAAGTTATATTTAAAGTGTGCATCTACCTTTCCGCCATTGAACCAGCTACCTTTTATGAACGCATCTGCCGAA
CAACACTACAAGGAAGGCTTATCAATCGCACGAGATAGCTCAAAAGGTAAATCTGATGATGCTCTAACAAATTTG
GTGTATATAGGAAAGAATCAAATTATTACGTTATCTAACCAAAATATTCCCAATACTGCGCGTGTAACAGTTGAT
ACAGAGCGCAAAGAAGTAGTGTCACCCATAGATGCTTACAAAGGAAAACCACTCGGCTATCATGTTCGAATGGCA
AGCACTTTGAATGAGGTGTCCGAAGGTTACACAAAGATCGTTTGGGTCAACAGTGGTGACTTCCACTACGACGAA
GAGTTGTCAAAGTACCACAAGGTAGAAACAAAGCTACCATACATCGCAAAACTGAAAAAAAGTTCAACATCAGAA
AAACCCTGTAATATCCTGCTTATCTTCGGGAAATGGGGCCATTTAAAACGTTGTTTCAGGAGATCTGACTTAGAA
TCCTCTTCTTTGCATCATTACTTTTCTGGTCAACTGCAATTCCCTGCTTCCGTACCCCAGGGAAATATACCCATT
CAAGATAGCCTACCCATAGCTCTTACCATGTTTCAGCGTTGGGCAAGTTGA

>YKL023W 0.81 Cold
ATGAATAAAGAAGAATTATTGGGTTTTCTACTAGATGACAGTATTGATAGCCAAAAAAGATGCGTCACGGATCAC
GAGGCCTACTCTAATTGGTTAAAGAATGATAATGATGAACGTACGGCCCATGAGGAAAGCTCTTCACAAAGTACG
ATAGCCGCATTGAATAAGAAGAAGCAGACAGAAGCTGCTCAAGAAGATATAGAAGAACTGTTAAACGGCTTAGAA
GGCATTATTGGAGGAGCCGACCCCCGTAATCTCAAGAGTAAGTCAAAGAGGAAAACCAAAAAGGGCGGATCGAAA
CCAAGGGAAGAAAACGTGAATACGGAGAAACATATTGTAATGCTCGAGGTAGAAGATTTTAGTGATATGAGCACC
CACGAAGATGTCAACGGAGCAAGTCCGTCGCCAAATCTGGACCGGTCGAAGAAGAATGAGAAGCGAAGGAAAAAC
GCCAAGGAGCTGTCATACGATGAACTAAAAGACAAACTAGAAGTTACCACTCGCAAATCACGTCTTGAGTGCAAA
GACTTGAAAAAGAAAGTTCATGGGCTCGAAAGAAGGAACCTTGAACTTGAACAGCGTTTAGAAGAGCTGAAGATT
GAGAACCAGACTTTGATAGAGATTAACAATAAACTGTTGAAAAACACTAATGAGGATGAGATTAACAAGAGCCAG
AGGAACAAGGAGAAGGACCGAAAGCGGAGGGAAAGAAGAACGGCAAGGAGAAAAGATGAAAGAAAGCAGGAAAAA
AAGCAGGAAAAAAAGCAGGATAATAAAACATCTCAATCTTTTCCTTCCTCAACTGACATGAATGGACAGCCTATA
GAATTTTGA

>YIR024C 0.79   Cold
ATGTTTATGGCAAGACAAGTGTTAAGAAATGGTCTATTTTTAAGGAGCCTTGCTCCCATAAAGATAACCGCTAGA
ACTGTTGCATCAGCAAATGCGGGCATTAAGAGAAAGTCAAGATTTGATAAAACAATGATCAAACCTTTACTTTTG
GTAATGATATTTGGGTCGATATTAAACGCAGTAATAGCCGAGAAAAGGAACATTATTGATATGGAAAGGAAATAT
AAGCTAAAACTTGATAAATTGAAAGAGCTGATACGGAGGGTTCATGATAATAATGGAAAGGTAGACTTCGACGCA
GATGATGAATTGAAACTTGTGAATTTGCGTTTAGGAATCGTCGGCAAGAACGCAACTGGGATGAAGGAAGATGAG
ACTGATATTGTGGTTCCTAAGGAAGAGTCATTGGAAGAGATCTGGCAAAGTATAATAGACGAGGCAAAAAAAGAA
GTAATCGAAAAAACTCCAGATGCTGGCGTTAAGAACAAAGAAGGGATTGTCACTGATTTGAATGTTCTAAAAGAT
TTAGAAAAGTCGAAAAAAGAAGATGAAAAGGTTTACCTAAGTGGTGATGTCCATATGATGATGAACCAACCAGGT
GATTTGAATGAAATCGCCAAAGAACATGATAAAATCCCAAAATTTCTATAG

>YIL004C 0.71   Cold
ATGAGTTCAAGATTTGCAGGGGGAAACGCTTATCAACGTGATACTGGTAGAACACAGTTATTCGGACCGGCTGAT
GGATCAAATAGTCTCGATGACAATGTATCATCAGCGCTAGGGAGCACAGATAAATTAGACTACTCCCAAAGTACT
TTGGCATCTCTTGAATCTCAAAGTGAGGAACAGATGGGAGCTATGGGTCAGAGAATAAAAGCACTCAAGTCATTA
TCGTTGAAGATGGGTGATGAGATTAGAGGCAGCAATCAAACTATTGACCAGCTTGGTGATACTTTCCATAACACT
TCTGTAAAACTCAAAAGGACTTTTGGAAACATGATGGAGATGGCCAGAAGATCTGGGATCAGTATAAAAACATGG
TTAATAATATTTTTTATGGTAGGCGTGCTATTTTTTTGGGTATGGATTACATAA

>YHR111W 0.72   Cold
ATGAATGACTACCATCTCGAGGATACCACGTCTGAACTTGAAGCATTAAGATTGGAGAATGCACAATTAAGAGAG
CAGCTTGCCAAGAGAGAAGACAGCAGCCGCGACTACCCATTATCTTTGGAAGAATACCAACGTTACGGAAGACAA
ATGATTGTTGAAGAAACAGGTGGTGTAGCAGGTCAAGTCAAGTTGAAAAATACAAAAGTTTTGGTAGTTGGTGCT
GGAGGTTTGGGATGTCCCGCCTTGCCCTACTTAGCGGGCGCTGGCGTGGGCCAAATAGGCATAGTAGATAACGAT
GTGGTGGAGACTTCCAATTTGCATAGGCAGGTTCTTCATGATTCTAGCAGAGTTGGAATGTTGAAATGTGAGTCG
GCCAGGCAATATATCACGAAACTGAACCCACACATTAACGTCGTTACCTATCCCGTTAGATTGAACTCCAGTAAT
GCTTTTGACATTTTCAAAGGTTACAATTATATATTAGACTGCACAGATTCTCCATTAACGAGATACCTGGTGTCT
GATGTTGCAGTTAATCTGGGAATAACAGTAGTGTCTGCATCCGGTTTGGGAACAGAGGGCCAGCTAACTATATTG
AATTTTAACAATATAGGGCCATGCTACAGATGCTTTTATCCAACACCTCCGCCACCAAATGCCGTGACCTCTTGC
CAAGAAGGCGGTGTGATAGGACCTTGCATTGGACTAGTTGGAACAATGATGGCTGTAGAAACTTTGAAGCTTATC
CTAGGAATCTACACCAATGAAAATTTTAGTCCCTTTTTGATGTTATATTCAGGTTTCCCACAGCAGAGCCTGCGC
ACTTTCAAAATGAGAGGCAGACAAGAAAAGTGTCTATGCTGTGGTAAGAATCGAACGATAACAAAAGAAGCCATC
GAAAAAGGTGAGATCAATTACGAACTGTTTTGTGGCGCACGAAACTATAATGTATGCGAGCCTGATGAGAGAATC
AGTGTGGACGCATTTCAGCGTATCTACAAGGATGATGAATTTCTAGCAAAACATATATTTCTTGATGTTAGGCCA
TCCCATCATTACGAGATATCTCATTTCCCTGAAGCAGTTAATATTCCAATCAAAAATCTAAGAGATATGAACGGT
GATCTTAAGAAACTGCAAGAAAAACTTCCCAGTGTAGAAAAAGACAGTAATATAGTGATTCTTTGCCGCTACGGT
AACGACTCTCAGCTAGCTACAAGATTATTGAAGGATAAATTTGGGTTTTCTAATGTACGAGACGTGAGAGGAGGA
TACTTCAAATACATAGACGATATTGATCAAACCATTCCTAAATATTAG

>YCL002C 0.75   Cold
ATGCTTGTTATTGTTCTGCAGGGCTTGGCAGGTTTTTTATCAATCATCGCCATTCTATGTCAAAAGCGCTATAAT
AAACTTCATCGATCTATTTACGGGCTTTCATATGATTTATTTCTGCTAGATTTTGTTGGTAATGGTCTGTACTTG
TATTGCGCGTTGCATTATTGTTATTCATCTCTGGTGAGGGAACAACTGTCTCAAAGATTCCCACTTTTTTATCCG
TTGAATGATGCCCGTAGTATACCTATATCCAGTTTCCTCATTCTAAAAGATTTTTGCGTTTCCTGTTGCTGCATG
ATGGTTTTAAGGCAGTTATACTATTATAGGTCAACTAAACATATTTATCAGGGCATTTCCATTACTTCCATAATA
ATTATAAGTGTGTTCCTTGTATTAGGCATATTTACATATGGCTGTTCCATCTCTAATTTACCGTTGAAAAATTCA
GGTAAATTTGGCGTTTTCTATTTGGAACACATAAATTATTTGTGGGTAATGGCAAATCTGCTTAAATGTTTCAAG
TATGTGCCTCAAATGAGTATAAACTGGATGGGATGCTCAACAGTAGGTTTATCATCAAAGTTTGCATTGATATCA
TTTTTGGCTGAATCTATCGATTTATTAGGTAGACTGGTAATACCGACCAATGCATTGTTTTACGAGATACCATTT
AATTCAACGCCCTTTTGGGTAAAATTGATACAATTTGTCACCTATTGGTCATACTTTGTCAAGTTCAGTATGTTT
ACGTAG

>YIL015W 0.81   Cold
ATGTCTGCAATTAATCATCTTTGTTTGAAACTTATTTTGGCGAGTTTCGCGATTATTAACACCATTACTGCTTTA
ACAAACGATGGCACTGGTCACTTAGAATTCCTTTTACAACACGAAGAGGAGATGTATTACGCAACAACCTTAGAT
ATAGGTACACCGTCCCAAAGTCTGACAGTGTTGTTTGATACCGGATCTGCCGATTTTTGGGTTATGGATTCTAGC
AATCCCTTCTGCTTACCAAATTCAAATACGTCATCCTATTCAAACGCAACTTATAATGGCGAAGAAGTTAAGCCT
TCAATTGATTGCAGGTCTATGAGTACTTATAATGAGCATAGATCTTCCACCTACCAATATCTGGAAAATGGTAGG
TTTTACATCACATATGCTGACGGAACATTTGCTGACGGTAGTTGGGGGACGGAAACTGTATCAATTAATGGAATT
GACATCCCCAATATCCAGTTCGGAGTTGCCAAGTATGCTACGACACCCGTTAGTGGTGTTCTTGGAATTGGGTTT
CCTAGAAGAGAGTCCGTTAAGGGCTATGAAGGTGCTCCTAATGAATATTATCCTAATTTTCCTCAGATTTTAAAA
AGTGAAAAAATAATCGATGTGGTCGCGTATTCGCTGTTCTTAAACTCACCTGATTCAGGTACTGGTTCGATTGTT
TTTGGTGCCATTGATGAATCAAAGTTTTCTGGTGATTTGTTCACTTTCCCTATGGTAAATGAATATCCCACAATA
GTCGACGCTCCTGCAACTTTAGCAATGACTATACAAGGATTAGGTGCCCAAAACAAAAGTAGTTGTGAACATGAA
ACGTTTACGACGACCAAGTATCCAGTTTTGTTGGACTCAGGAACCTCGCTATTGAATGCGCCCAAGGTCATAGCA
GATAAAATGGCTTCTTTTGTAAATGCGTCCTATAGTGAAGAGGAAGGTATATATATATATTAGACTGTCCAGTATCT
GTAGGTGACGTGGAATACAATTTTGATTTCGGCGATTTGCAAATAAGTGTTCCACTGTCTAGTTTGATTTTAAGT

CCCGAGACAGAAGGCAGCTATTGTGGGTTTGCGGTCCAGCCAACAAACGATTCGATGGTTCTGGGTGATGTGTTC
CTGTCCTCTGCATACGTCGTATTCGATCTCGATAATTATAAGATATCTTTAGCACAGGCAAATTGGAACGCAAGC
GAAGTTTCGAAAAAGCTAGTAAATATTCAAACAGATGGGTCTATTTCAGGTGCCAAAATTGCTACAGCTGAACCC
TGGTCCACCAATGAACCATTTACAGTCACCTCTGACATTTATTCATCTACAGGCTGCAAGAGTAGGCCTTTTCTT
CAATCATCGACAGCCTCTTCGCTTATTGCAGAAACCAACGTACAAAGTCGCAACTGCTCTACGAAGATGCCAGGC
ACTAGATCAACTACTGTCTTAAGTAAGCCTACTCAAAATAGTGCTATGCATCAAAGTACAGGCGCTGTCACACAA
ACCTCAAATGAAACTAAATTAGAATTATCCTCGACTATGGCAAATTCGGGCAGTGTCTCGCTTCCCACTTCGAAT
TCAATAGACAAAGAGTTCGAACATTCGAAATCTCAAACTACCAGCGATCCAAGTGTAGCAGAGCATTCTACGTTT
AACCAAACGTTTGTACATGAAACTAAATATCGGCCTACTCATAAGACAGTCATAACAGAAACTGTCACGAAGTAT
TCTACAGTCTTAATAAATGTCTGTAAACCAACATATTAA

>YLR106C 0.82 Cold
ATGTCCCAGGATAGAATTTTGTTAGATTTAGACGTAGTTAACCAAAGGTTAATTCTCTTCAATTCTGCTTTTCCC
TCTGATGCTATTGAAGCGCCCTTCCACTTTAGCAACAAGGAATCAACTTCTGAGAATTTAGACAATTTAGCAGGA
ACTATACTACATAGCCGTTCCATCACTGGTCATGTTTTCCTTTACAAACATATTTTTTTAGAGATAGTGGCACGT
TGGATAAAGGACTCCAAAAAAAAAGATTACGTTCTTGTAATTGAAAAGTTGGCAAGTATAATTACAATATTTCCC
GTTGCAATGCCATTAATTGAAGACTATTTAGATAAAGAGAATGATCATTTCATCACTATTCTTCAAAATCCATCC
ACACAAAAGGATTCCGACATGTTTAAAATACTATTAGCATACTATAGGTTACTATATCACAATAAAGAAGTATTT
GCACGCTTCATTCAACCCGACATTTTATACCAACTTGTGGATTTACTCACCAAAGAACAAGAAAATCAGGTTGTT
ATATTTCTAGCATTAAAGGTTTTATCTTTATACTTAGACATGGGAGAAAAAACATTAAACGATATGCTCGACACT
TATATAAAATCACGCGATTCATTGTTGGGCCATTTTGAGGGCGATTCTGGAATTGATTACAGCTTCCTCGAACTG
AACGAAGCTAAGAGATGTGCAAATTTCTCTAAACTACCCAGTGTTCCAGAATGTTTCACTATAGAAAAAAAATCT
AGCTACTTCATTATTGAGCCTCAGGATTTGAGTACTAAGGTCGCGTCAATCTGTGGCGTTATCGTCCCTAAGGTC
CACACTATCCACGACAAAGTATTCTACCCATTAACCTTTGTCCCTACTCATAAAACGGTTTCCTCTCTTCGTCAA
CTCGGGAGAAAGATTCAAAATTCAACTCCAATAATGCTCATTGGAAAAGCGGGATCCGGTAAAACCTTTTTAATC
AATGAGCTAAGTAAATATATGGGTTGCCATGACTCCATTGTGAAAATTCATTTAGGTGAACAAACTGATGCCAAG
TTACTGATTGGTACTTATACCTCTGGTGACAAGCCAGGCACGTTTGAATGGAGAGCTGGTGTTCTCGCTACCGCG
GTCAAAGAAGGGAGATGGGTACTTATAGAAGACATAGATAAAGCTCCTACAGATGTTTTATCAATACTTTTATCG
CTTTTAGAAAAGCGCGAACTAACCATTCCATCCAGAGGAGAGACTGTAAAGGCTGCAAATGGTTTCCAGTTAATA
TCAACTGTTAGAATAAACGAAGATCATCAGAAGGATAGTTCCAATAAGATCTATAACTTGAATATGATTGGTATG
AGAATCTGGAATGTTATTGAATTAGAGGAACCTAGCGAAGAGGATTTGACACACATTTTAGCCCAAAAGTTTCCC
ATTTTGACCAACCTTATCCCCAAACTAATCGATTCATACAAAAACGTCAAAAGTATTTATATGAACACCAAGTTC
ATTTCATTGAACAAGGGGGCACACACAAGAGTTGTATCTGTAAGAGATTTAATTAAGCTGTGCGAAAGGTTAGAC
ATCCTATTCAAAAACAATGGCATCAACAAACCTGACCAACTAATCCAGTCTTCTGTCTACGACAGTATTTTCTCC
GAAGCCGCCGACTGTTTCGCTGGCGCTATTGGAGAATTCAAAGCTTTGGAACCTATTATACAAGCAATCGGCGAG
TCATTAGACATTGCTTCCTCAAGGATTTCGTTATTTTTGACCCAACATGTGCCCACTTTGGAAAACCTTGATGAT
AGCATTAAAATTGGAAGAGCCGTATTACTTAAGGAAAAGCTAAATATACAAAAAAAATCAATGAACAGCACATTG
TTCGCATTTACGAATCATTCTTTAAGACTAATGGAACAGATTTCTGTTTGTATACAGATGACTGAACCTGTTTTA
CTAGTCGGTGAAACGGGTACCGGGAAAACTACAGTCGTTCAACAACTTGCTAAGATGTTAGCAAAAAAATTAACG
GTAATCAATGTTTCACAACAAACAGAAACTGGTGATCTTCTTGGTGGGTACAAACCCGTAAATTCTAAAACAGTT
GCAGTTCCTATACAAGAAAATTTTGAAACTCTTTTTAATGCTACATTTTCCTTAAAGAAGAACGAGAAGTTTCAC
AAGATGCTACATAGGTGCTTCAACAAGAATCAATGGAAGAACGTTGTTAAACTTTGGAACGAAGCGTACAAAATG
GCTCAAAGCATATTAAAAATTACAAACACTGAAAATGAAAACGAAAATGCAAAGAAGAAGAAAAGAAGACTCAAT
ACTCATGAAAAAAAATTGTTACTTGACAAATGGGCTGACTTCAATGACTCAGTGAAGAAGTTTGAGGCCCAGTCT
AGCTCTATTGAAAACTCGTTTGTCTTCAATTTTGTCGAGGGTTCTTTGGTAAAGACTATCAGGGCTGGGGAGTGG
CTATTGCTCGATGAAGTGAATTTAGCAACCGCAGATACCTTAGAAAGTATTTCTGACCTTCTCACCGAGCCTGAT
TCTCGTAGTATTCTTCTTTCAGAAAAGGGTGACGCTGAGCCTATAAAAGCTCACCCCGATTTTAGAATATTTGCT
TGTATGAACCCCGCTACTGATGTTGGTAAAAGAGACTTACCTATGGGTATCAGATCAAGATTTACCGAAATATAT
GTACATTCTCCGGAACGTGATATTACTGATTTGCTTTCCATAATTGATAAATATATTGGAAAGTACAGCGTCAGC
GATGAATGGGTAGGAAATGACATCGCTGAACTTTACCTTGAAGCCAAAAAACTCTCCGATAACAATACTATAGTT
GATGGCTCAAACCAAAAACCACACTTTAGTATCCGTACCCTGACGCGTACATTGCTTTATGTTACTGATATTATT
CATATCTACGGTCTACGCCGTTCCCTGTATGATGGATTCTGTATGAGTTTTTTGACTTTACTGGACCAAAAATCT
GAGGCCATTCTGAAGCCAGTAATAGAAAAATTCACATTGGGGAGGCTAAAAAATGTCAAGTCAATCATGTCACAG
ACACCTCCCTCTCCAGGACCAGATTATGTTCAGTTTAAGCATTATTGGATGAAAAAGGGCCCTAATACAATTCAA
GAACAAGCTCACTATATCATTACGCCTTTTGTTGAAAAGAATATGATGAATTTGGTAAGAGCTACATCAGGCAAA
AGATTTCCTGTGCTTATTCAAGGTCCAACTTCATCGGGAAAAACCAGTATGATCAAATATCTTGCGGATATAACG
GGCCATAAGTTTGTCCGTATAAATAACCATGAGCATACAGATTTACAGGAATATTTAGGTACATATGTTACCGAT
GATACCGGAAAGCTGTCGTTTAAGGAAGGTGTTTTAGTCGAGGCACTACGAAAAGGATATTGGATTGTTTTGGAT
GAATTAAATCTTGCCCCAACCGATGTTTTAGAAGCCTTAAATAGGTTACTTGACGATAACAGAGAACTTTTTATT
CCAGAAACACAAGAAGTTGTACATCCTCATCCTGATTTCCTTCTTTTTGCTACCCAGAATCCACCTGGGATATAC
GGTGGCCGTAAAATTCTTTCAAGAGCTTTTAGGAATCGTTTTCTGGAGTTGCACTTCGACGATATTCCGCAAGAT
GAATTAGAAATTATCCTACGTGAGCGCTGCCAAATTGCCCCATCATATGCAAAGAAAATCGTTGAAGTATACCGC
CAATTATCCATCGAACGTTCCGCTTCAAGGTTGTTCGAACAGAAAAATAGTTTTGCAACTCTACGTGATCTTTTC
CGCTGGGCTTTAAGGGACGCAGTGGGATATGAGCAGCTTGCTGCCAGTGGCTATATGCTTCTTGCAGAAAGGTGC
AGAACTCCACAAGAAAAAGTTACAGTCAAAAAGACTTTAGAAAAAGTAATGAAAGTTAAATTAGATATGGACCAA
TATTATGCAAGTCTGGAAGATAAATCCTTAGAAGCCATTGGCTCTGTTACTTGGACAAAAGGAATGCGCCGACTA
TCCGTCTTAGTTTCTAGTTGTTTAAAGAATAAGGAGCCAGTACTACTGGTAGGTGAAACTGGGTGCGGCAAAACA

ACAATTTGCCAATTATTGGCGCAATTCATGGGTCGAGAACTGATCACACTAAATGCCCATCAAAACACAGAAACT
GGAGACATATTAGGTGCTCAAAGACCTGTTCGTAACAGGTCTGAAATTCAATATAAGCTAATAAAATCCTTAAAA
ACAGCATTGAATATTGCTAATGACCAAGATGTAGACTTGAAAGAGTTATTACAGCTATATTCTAAGTCAGATAAT
AAAAATATAGCCGAAGATGTTCAACTGGAAATACAAAAGCTGAGGGATAGTTTGAATGTTTTATTTGAATGGAGT
GACGGGCCTCTGATTCAAGCTATGAGGACGGGAAATTTTTTTCTACTTGATGAAATATCGCTAGCAGATGATTCC
GTTCTTGAAAGACTAAATAGTGTTTTGGAGCCAGAAAGGAGTTTGTTGTTAGCAGAACAGGGTTCATCCGATAGC
CTTGTAACGGCTTCAGAAAACTTTCAATTCTTCGCAACCATGAACCCTGGTGGAGATTACGGTAAAAAAGAATTA
TCTCCAGCGTTAAGAAATAGATTTACTGAAATATGGGTACCATCCATGGAAGATTTCAACGATGTAAACATGATC
GTATCCTCAAGGCTTTTAGAGGACTTGAAAGATCTTGCTAATCCAATTGTGAAATTCTCTGAATGGTTTGGCAAG
AAGCTGGGAGGCGGAAATGCAACAAGTGGTGTCATTTCTTTACGTGACATACTTGCATGGGTTGAATTTATTAAT
AAAGTGTTTCCGAAAATACAGAACAAGTCAACTGCCTTAATTCAAGGTGCCTCAATGGTTTTTATTGACGCCCTT
GGTACAAATAACACTGCTTACTTAGCTGAAAACGAAAATGATTTAAAGTCACTTAGAACAGAATGTATTATACAA
CTATTGAAACTATGTGGTGACGATTTGGAACTACAACAAATTGAAACGAACGAAATTATTGTGACTCAGGATGAA
TTACAAGTCGGAATGTTCAAAATACCAAGATTTCCAGATGCTCAGTCTTCATCCTTTAACTTAACCGCCCCCACT
ACTGCCTCAAACTTAGTAAGGGTTGTACGGGCTATGCAAGTACACAAGCCAATTTTACTTGAAGGTAGTCCAGGT
GTTGGTAAAACAAGTTTAATCACCGCTCTTGCCAATATTACAGGAAATAAGCTTACAAGAATCAATTTGTCTGAA
CAAACGGATCTAGTAGATCTTTTCGGTGCAGACGCTCCTGGTGAGCGAAGTGGTGAATTTTTATGGCACGATGCT
CCGTTTTTAAGAGCAATGAAGAAAGGTGAGTGGGTTTTATTAGATGAAATGAATTTAGCCTCCCAATCCGTCCTA
GAAGGGTTGAATGCCTGTTTAGATCATCGAGGCGAAGCGTATATCCCCGAGTTGGACATATCGTTTTCCTGCCAC
CCCAATTTTCTGGTCTTTGCTGCTCAAAATCCTCAGTATCAAGGTGGTGGTAGGAAAGGTCTTCCTAAATCTTTC
GTTAATCGATTTAGCGTGGTATTTATTGACATGCTTACATCTGATGATCTCCTTTTGATTGCAAAACACTTATAT
CCAAGTATTGAGCCAGATATCATCGCGAAAATGATTAAGTTAATGTCTACTTTAGAGGATCAAGTCTGTAAAAGA
AAACTTTGGGGTAATTCTGGTTCACCATGGGAGTTCAATTTGAGAGATACATTGCGTTGGCTAAAGTTACTCAAC
CAATATTCGATTTGCGAAGATGTGGACGTGTTTGACTTTGTTGATATAATCGTTAAGCAGAGGTTCCGTACTATC
AGCGATAAGAATAAAGCACAGCTTCTCATTGAAGATATTTTTGGTAAATTCTCAACTAAAGAGAACTTTTTCAAA
CTTACAGAAGACTATGTTCAAATTAACAACGAGGTAGCTCTAAGAAATCCCCATTACCGCTATCCAATTACCCAG
AATCTTTTCCCATTAGAGTGCAATGTCGCCGTTTATGAATCTGTTTTGAAAGCAATCAACAATAATTGGCCGTTG
GTACTTGTTGGGCCCTCAAACTCCGGTAAAACGGAAACGATAAGATTTTTAGCTTCAATCTTGGGTCCCAGAGTA
GACGTGTTTTCCATGAATAGTGATATAGATAGTATGGATATCCTTGGTGGTTATGAACAAGTTGATTTGACTAGA
CAAATATCATATATCACTGAGGAGTTAACAAATATCGTAAGAGAAATAATCTCGATGAATATGAAGCTCTCACCA
AATGCTACCGCAATCATGGAAGGTTTGAACTTATTGAAATATTTGCTCAATAATATCGTCACACCTGAGAAATTC
CAAGATTTCAGAAATCGTTTTAATAGGTTCTTTTCGCACTTAGAAGGGCATCCCCTATTAAAAACTATGAGTATG
AATATTGAAAAAATGACTGAAATCATCACTAAGGAAGCTTCGGTTAAATTTGAATGGTTTGATGGCATGTTGGTC
AAAGCAGTTGAAAAGGGCCATTGGCTAATCTTAGATAATGCTAATCTTTGTTCTCCTTCTGTTCTTGATAGACTG
AACTCTCTATTGGAGATAGACGGGTCGCTGCTCATCAATGAATGTAGCCAAGAAGATGGCCAACCAAGGGTTCTC
AAACCCCACCCTAACTTCAGATTATTTTTGACCATGGATCCAAAATATGGTGAATTATCCCGAGCTATGAGAAAC
AGAGGTGTGGAGATTTATATCGACGAATTGCACAGTCGTTCTACAGCCTTCGATCGCCTCACCCTAGGCTTTGAA
CTTGGAGAGAATATAGACTTTGTTTCTATTGATGATGGCATCAAAAAAAATAAAACTAAATGAACCTGATATGTCG
ATACCATTAAAACACTATGTTCCATCCTATTTAAGCAGACCATGCATATTTGCACAAGTACATGATATTTTATTA
TTATCTGACGAAGAACCAATTGAAGAATCTCTTGCTGCTGTTATTCCGATTTCCCATTTAGGGGAAGTTGGTAAA
TGGGCCAATAATGTATTGAATTGCACGGAGTACTCAGAAAAAAAGATCGCAGAAAGGCTTTATGTTTTTATAACA
TTTTTGACTGATATGGGTGTCTTAGAAAAGATTAATAACCTATACAAACCTGCAAACCTAAAATTCCAGAAGGCT
TTGGGGCTGCATGATAAGCAATTGACAGAGGAAACTGTCTCGCTTACCTTGAACGAATATGTTCTTCCAACAGTC
AGCAAGTATTCAGATAAGATCAAATCTCCAGAGTCGTTATACTTATTATCAAGTTTACGTCTGCTACTCAACTCT
TTGAACGCTCTCAAATTAATCAATGAAAAATCAACTCATGGTAAAATTGACGAACTGACGTATATTGAATTATCT
GCGGCTGCCTTTAACGGTCGCCATTTGAAGAATATTCCTCGCATTCCAATATTCTGTATTCTCTACAATATTTTA
ACAGTAATGTCTGAAAACCTTAAGACGGAAAGTTTATTTTGCGGCTCCAACCAATATCAATATTATTGGGATTTA
CTGGTAATTGTAATTGCAGCATTAGAAACCGCCGTTACAAAGGATGAAGCACGTCTAAGAGTATATAAGGAGTTG
ATAGATAGCTGGATTGCGTCCGTAAAATCCAAATCAGACATTGAGATAACTCCCTTCCTTAACATCAATTTGGAA
TTTACCGATGTTTTACAGTTGTCGAGGGGCCACTCCATAACTTTACTATGGGATATATTCAGAAAAAATTATCCG
ACAACCTCCAACAGCTGGCTAGCTTTCGAAAAGCTCATTAATTTATCTGAAAAATTTGACAAAGTACGTCTGCTC
CAATTTTCTGAATCATACAATTCCATTAAGGATTTGATGGACGTTTTCCGGTTGCTAAATGATGATGTGTTGAAT
AATAAATTAAGTGAGTTTAATCTCTTGCTATCAAAGTTAGAAGATGGTATAAATGAATTGGAACTAATTTCAAAT
AAGTTTTTAAACAAAAGAAAACATTATTTTGCTGACGAATTTGACAATTTGATCAGATATACCTTCTCAGTTGAC
ACAGCCGAGCTAATAAAAGAACTTGCTCCCGCTTCTTCTTTAGCCACGCAAAAACTTACAAAACTTATTACCAAC
AAATACAATTATCCGCCTATCTTTGATGTTCTTTGGACAGAAAAAAAATGCTAAATTGACTTCCTTTACTTCTACC
ATATTTTCATCCCAGTTTCTCGAAGATGTCGTTCGTAAATCTAATAATTTGAAATCATTTTCGGGAAATCAGATC
AAACAATCTATTAGTGACGCAGAATTGCTACTTTCCTCCACTATCAAGTGTTCACCTAACCTACTTAAGAGCCAA
ATGGAGTACTATAAAAAATATGTTACTGTCATGGCTCAGAAAAGTTATTGATATTCACGTCGGCGGGGACTGCTTG
AAATTGACTTTGAAAGAACTTTGCTCTCTCATAGAAGAAAAAACTGCTTCAGAGACACGCGTGACTTTTGCAGAA
TACATCTTTCCCGCTCTAGATTTGGCTGAAAGTTCGAAATCATTAGAAGAACTGGGAGAAGCTTGGATCACATTT
GGTACAGGTTTGCTTCTGCTATTCGTCCCAGATTCTCCCTATGATCCGGCAATACACGATTACGTTTTATATGAT
TTATTTTTGAAGACAAAGACATTTTCTCAAAACCTAATGAAAAGTTGGAGAAATGTTCGTAAAGTAATCAGTGGC
GATGAGGAGATTTTCACAGAAAAACTTATCAATACCATAAGCGATGACGACGCACCTCAATCCCCTAGGGTATAT
CGTACAGGCATGTCTATTGACTCTTTATTTGATGAATGGATGGCATTTTTGTCGTCAACTATGAGTTCTCGTCAA
ATAAAAGAGCTAGTGAGTTCCTACAAGTGCAATTCAGATCAATCGGATCGTAGATTAGAAATGCTCCAACAAAAT
AGTGCACATTTTTTGAATCGTCTCGAAAGCGGTTATTCAAAATTTGCTGATTTAAATGATATCCTCGCGGGATAT

ATTTACAGTATCAACTTTGGCTTTGATTTATTGAAGCTTCAAAAATCTAAGGATAGAGCATCATTCCAAATTTCT
CCTTTATGGAGTATGGACCCTATCAATATTTCATGCGCAGAGAATGTTTTAAGCGCTTATCATGAGCTGTCAAGG
TTCTTCAAAAAGGGTGATATGGAAGATACTTCGATTGAAAAGGTTTTGATGTACTTCTTGACGCTGTTTAAGTTC
CATAAGAGGGATACAAATTTACTGGAAATTTTTGAGGCTGCTTTGTATACTCTGTATTCAAGATGGTCTGTGAGA
CGTTTCAGACAGGAGCAGGAGGAAAACGAAAAGTCAAATATGTTCAAATTCAATGATAATTCAGATGATTACGAG
GCAGACTTTAGAAAGCTGTTTCCAGATTATGAGGACACAGCTTTGGTAACTAATGAAAAAGATATTTCGAGTCCT
GAAAATCTTGATGACATCTATTTCAAGCTTGCCGACACATATATCTCGGTATTTGACAAAGATCATGACGCCAAC
TTTTCTTCTGAGTTGAAGAGCGGTGCGATAATAACAACAATTTTGAGTGAAGATTTAAAAAACACCAGGATAGAA
GAGTTGAAAAGCGGATCGTTGTCTGCTGTGATTAATACACTTGATGCAGAAACACAATCTTTCAAAAACACGGAA
GTTTTTGGTAATATCGATTTTTACCACGATTTCTCTATTCCGGAGTTTCAAAAAGCAGGTGATATCATTGAAACT
GTGCTTAAGTCAGTCTTGAAATTGTTGAAGCAGTGGCCAGAACATGCCACCCTCAAAGAATTATATCGAGTTTCC
CAAGAATTTTTAAACTACCCCATTAAAACACCCTTAGCAAGACAGCTTCAAAAGATCGAACAAATATATACATAC
CTAGCTGAATGGGAAAAATACGCTTCCTCGGAAGTATCCCTGAATAACACCGTTAAACTGATTACAGATTTGATA
GTTTCATGGAGAAAACTAGAATTACGCACCTGGAAAGGTCTTTTCAACTCGGAAGACGCAAAAACCAGAAAATCT
ATTGGAAAATGGTGGTTTTATTTGTACGAGTCAATTGTCATTTCAAATTTTGTTAGTGAAAAGAAGGAAACGGCC
CCTAACGCTACACTTTTGGTAAGCTCACTTAACTTATTTTTTAGTAAGAGTACATTGGGCGAATTCAACGCCAGG
CTAGATCTTGTAAAAGCATTCTACAAGCACATTCAACTAATTGGATTGAGGAGCTCGAAGATAGCGGGCTTGCTC
CATAATACTATAAAATTCTATTATCAGTTCAAACCATTAATTGATGAGCGGATTACGAATGGAAAAAAGAGTCTG
GAAAAAGAAATTGACGATATAATTCTTTTAGCCAGTTGGAAAGATGTTAACGTTGATGCCCTGAAACAAAGTTCT
CGTAAGTCTCATAACAACCTTTACAAAATTGTCAGAAAATACCGTGATTTGCTAAATGGGGATGCGAAAACCATT
ATTGAGGCCGGTCTTTTGTATTCCAACGAAAATAAACTAAAACTCCCTACTTTAAAACAACATTTTTACGAAGAC
CCCAACTTAGAGGCTTCGAAAAATTTGGTGAAAGAAATTTCTACTTGGAGTATGAGAGCTGCGCCATTAAGGAAT
ATTGACACGGTAGCAAGTAACATGGACTCCTACTTGGAAAAGATATCATCACAGGAATTCCCTAACTTCGCAGAT
TTAGCATCGGACTTTTATGCTGAAGCTGAAAGGTTACGTAAGGAAACCCCTAATGTATATACAAAGGAGAACAAA
AAGAGGTTGGCATACTTGAAGACTCAAAAAAGTAAACTTTTAGGTGATGCATTGAAAGAATTGAGAAGAATCGGT
TTAAAAGTCAATTTTAGGGAAGACATTCAAAAAGTTCAATCTTCTACAACAACTATTTTGGCAAACATTGCCCCT
TTCAATAATGAATATTTAAATTCGTCAGACGCATTCTTTTTCAAAATCCTTGACCTTTTACCCAAGCTGAGAAGC
GCTGCATCGAATCCAAGTGATGATATCCCTGTTGCTGCTATAGAAAGAGGTATGGCACTCGCACAGAGTTTGATG
TTTTCCCTGATTACAGTTCGTCACCCTCTATCAGAGTTCACGAATGATTACTGTAAGATTAATGGTATGATGTTA
GACCTAGAACACTTTACTTGCTTAAAGGGCGATATTGTTCATTCTTCATTAAAAGCGAATGTAGATAATGTCAGA
TTGTTTGAAAAGTGGCTGCCTTCTTTGTTAGACTATGCCGCACAGACCTTGTCTGTTATTTCGAAATACTCAGCA
ACTTCAGAACAACAAAAGATCTTGTTAGATGCAAAATCGACCTTATCTTCGTTTTTCGTACACTTCAATTCCAGT
CGAATTTTTGATTCTTCATTTATCGAGTCATATTCTAGATTTGAGTTATTCATTAATGAATTATTGAAGAAGCTT
GAAAATGCAAAGGAGACAGGGAACGCTTTCGTTTTTGATATCATTATAGAGTGGATCAAAGCAAATAAAGGGGGT
CCTATTAAAAAGGAACAGAAGAGAGGCCCTTCGGTGGAAGATGTTGAACAGGCTTTCCGCCGTACTTTCACATCT
ATTATATTATCTTTCCAGAAGGTAATTGGAGATGGAATTGAATCTATATCCGAAACTGATGATAACTGGTTATCA
GCTAGCTTCAAGAAAGTTATGGTAAACGTTAAACTATTACGATCCAGTGTTGTTTCTAAAAATATAGAGACAGCA
TTATCCTTGTTGAAGGACTTTGATTTTACCACTACTGAATCAATTTACGTTAAATCAGTTATATCCTTTACACTA
CCGGTAATCACACGTTATTACAACGCAATGACCGTGGTTCTTGAAAGATCTAGAATCTACTACACGAACACAAGT
CGTGGTATGTACATTCTATCGACGATATTGCATAGCTTAGCCAAAAATGGGTTCTGTTCTCCGCAGCCACCATCT
GAAGAAGTTGACGACAAAAACTTGCAAGAGGGTACAGGCTTGGGAGACGGTGAAGGTGCTCAAAATAACAATAAA
GACGTCGAACAAGACGAAGATTTGACTGAGGATGCACAAAACGAAAACAAAGAACAACAAGATAAAGATGAGAGG
GATGACGAGAATGAAGATGATGCAGTTGAGATGGAGGGCGATATGGCTGGTGAATTGGAAGATTTGTCTAATGGT
GAAGAAAATGATGATGAAGATACTGATAGCGAAGAAGAAGAATTAGATGAAGAAATTGATGACCTGAATGAAGAT
GACCCCAACGCTATTGATGATAAAATGTGGGATGATAAGGCAAGTGATAATTCAAAAGAAAAAGATACAGACCAG
AATTTAGATGGAAAAAATCAAGAGGAAGATGTACAAGCTGCTGAAAATGACGAACAACAGCGGGATAACAAGGAA
GGAGGGGATGAGGACCCAAATGCACCTGAAGATGGCGATGAGGAAATTGAAAACGATGAAAATGCAGAAGAGGAA
AACGACGTTGGTGAACAGGAGGACGAAGTTAAAGATGAAGAAGGCGAGGATTTGGAGGCTAATGTTCCTGAAATC
GAAACCCTAGACCTTCCTGAAGATATGAATTTAGATTCAGAGCATGAAGAATCAGACGAAGATGTTGATATGTCA
GATGGAATGCCTGATGACCTAAACAAGGAAGAGGTTGGAAACGAAGACGAAGAAGTCAAACAAGAATCCGGTATC
GAAAGTGATAATGAAAACGACGAACCTGGTCCTGAGGAAGATGCCGGCGAGACAGAAACAGCTCTTGACGAAGAA
GAAGGTGCTGAAGAGGATGTGGATATGACTAATGATGAGGGAAAGGAAGACGAAGAAAATGGCCCAGAAGAGCAA
GCCATGTCTGATGAAGAAGAGTTAAAGCAAGATGCTGCTATGGAAGAAAACAAAGAGAAAGGAGGTGAGCAGAAT
ACAGAAGGTCTTGATGGCGTTGAGGAAAAGGCTGATACTGAAGACATCGATCAAGAAGCTGCCGTTCAACAAGAT
TCTGGATCCAAAGGTGCTGGTGCTGATGCTACGGACACACAAGAACAGGACGATGTTGGAGGTTCAGGAACGACT
CAGAATACCTATGAAGAAGATCAAGAAGATGTGACAAAGAATAATGAAGAATCACGCGAAGAGGCTACGGCTGCT
TTAAAGCAACTAGGTGATAGCATGAAAGAATATCATAGACGTCGTCAAGATATAAAAGAAGCACAAACTAATGGC
GAAGAGGACGAAAATTTGGAAAAAAACAATGAACGTCCCGATGAATTTGAACATGTTGAGGGTGCAAATACTGAA
ACAGATACGCAAGCTTTAGGTTCTGCAACACAGGATCAATTACAAACCATTGATGAAGACATGGCCATTGATGAT
GATAGGGAGGAGCAAGAAGTGGACCAGAAAGAGTTAGTAGAAGACGCCGATGATGAAAAAATGGATATTGATGAA
GAAGAGATGCTATCTGATATCGACGCGCATGACGCTAACAATGACGTTGACAGCAAAAAGTCAGGATTCATCGGT
AAAAGAAAGAGTGAGGAAGATTTTGAGAACGAACTTTCAAATGAACACTTCTCGGCTGATCAAGAGGATGACAGT
GAAATACAATCTTTAATTGAAAACATTGAGGATAATCCACCTGATGCAAGTGCTAGTTTGACACCGGAAAGATCG
TTAGAAGAATCCCGTGAATTGTGGCATAAAAGTGAAATTTCGACCGCCGATTTAGTATCACGTCTTGGAGAACAA
TTAAGACTAATCTTGGAACCTACCTTGGCTACAAAGCTGAAGGGTGATTACAAAACAGGTAAGAGACTGAATATG
AAACGGATCATTCCATATATTGCCTCACAATTCCGTAAAGATAAAATCTGGTTGAGAAGAACTAAACCAAGCAAA
CGTCAGTATCAAATAATGATTGCGCTTGATGATTCTAAGTCTATGAGCGAATCTAAATGCGTTAAACTCGCTTTT

GATAGTTTATGCTTGGTCTCCAAGACGTTAACTCAGCTAGAGGCTGGAGGGTTATCTATTGTCAAATTCGGTGAA
AATATTAAAGAAGTTCACTCATTTGACCAACAATTTAGTAACGAATCTGGCGCAAGAGCTTTTCAATGGTTTGGT
TTCCAAGAAACTAAAACAGACGTTAAGAAACTTGTGGCTGAATCTACTAAAATTTTTGAACGTGCTCGCGCTATG
GTACATAATGACCAATGGCAACTAGAAATTGTAATCTCTGATGGTATTTGCGAAGACCATGAAACAATACAAAAA
CTGGTCCGCCGTGCTAGAGAAAACAAAATTATGCTGGTTTTTGTCATCATAGATGGTATTACTTCTAATGAATCC
ATCTTAGACATGAGCCAAGTGAACTACATTCCAGATCAGTATGGCAATCCGCAATTAAAGATTACCAAATACTTG
GACACATTTCCTTTTGAATTTTATGTTGTTGTACACGATATAAGCGAACTACCCGAAATGCTTTCACTGATTTTG
CGTCAATACTTTACAGACCTGGCATCCAGCTAA

>YOR067C 0.8 Cold
ATGAAAGGTGATCGTTCGAGGCAAAATATGGCTGTGACAAAGAAGGCAAAGTTAAAAAAAAATGACGAGCCAAAA
AAGGTTTTGAAGACTGCCGCGACCGAAAAAGGTGAGGGATCCAAGCGCTACTCCTTATGGAATTTCTGGATAAGC
ACATTGTTTTTAAAATTACTATTGATTCCTGACTACTTCAGTACAGATTTTGACGTTCATAGAAATTGGCTGGCA
ATCACCAATAAATTGCCCATCAGCGAATGGTATTATGAGCACACTAGTCAATGGACATTGGATTATCCGCCATTT
TTTGCTTATTTTGAATGGTTTTTATCGCAATTTGTTCCTAAATCTGTACGTGATGACGGTTGTTTGGATATAGTG
GAGATCGGAAAGTTCGGTTTGCCAACAATCGTTTTCCAACGACTCACTGTCATCTTCAGCGAAATTCTTCTATTT
GTCATCCTTCAAATATACATTAATACTACAAAGCTAAGTGAAAGATCTCAAAGTTTTGTGGTTGCTTCTAGCATT
GTACTATCACCCGGGTTTTTAATAATAGATCACATTCACTTCCAATATAATGGTTTTCTTTTTGCGATTCTAATA
GGCTCCATTGTCGCCGCCAAGAATAAGAGATACATCTTGTGCGCTGTTCTGTATACTACAGCCATATGTTTCAAG
CATATATTTTTATATTTGGCGCCATGTTATTTCGTGTTTTTATTGAGAGCATACGTTCTCAATGTCAACAATTTC
AAGTTTAAAAGTTACAAAGATTTTTTGTTCCTGATAAGATGGGCAAATCTTTTGAAGTTGGCTACCGTTGTTGTT
GGAATTTTCACTATTTGTTTCCTTCCATTTGCTCATCAAATGCCGCAAGTGTTGAGCAGGTTATTCCCATTCTCA
AGAGGACTGACGCATGCATATTGGGCACCAAATTTCTGGGCGTTGTATTCTTTTATGGATAAAATACTCACTACG
GTAATGTTGAAATTACCGTACGTCCACACTTTTGCTACAAAATTTATTAAACCTCCTCTGATCCCTCAGAATATC
AAGGAAATTAATGAAAGGCTCGCGGCAAACAACAATGGAAGTAAAGGTCTAGTTCAAGACGTATTTTTTGTTATT
CTCCCTCAAATTCCTCCAAAATTAACGTTTATTTTGACGATATTTTACCAGGTCTTAGCAGTACTTCCACTTTTG
TTTGATCCATCGTTCAAAAGATTTGTTGGCTCGTTGACTCTCTGTGGGTTGGCATCGTTTTTGTTCGGGTGGCAT
GTGCATGAGAAAGCTATAATGCTAGTCATCATTCCGTTCACATTTCTTGTTGGATTTGATCGTCGTCTTTTGGTA
CCGTTTATGTTAGTTGCCTCAGCTGGTTATGTTTCCTTGTATCCTCTTCTGTACAAAGGCCAAGATTTTTTCATT
AAAACATTATACACTTATGTTTGGTGTATAATTTATTTTGCCGCTTTTAGAAAAACTACGAAGATTTCTTCAAGT
GTAGAAAGAAGAATCTTTTTCTTGGATAGGTTGGCATTAACGTACATCTTTTCATTGTTACCAATCGTCACCGTA
TTACAAATATTGGATGAAGTCAAGTGGAGATATTCATTTTTGCAAAAATTTGAATTCTTAGGATTAATGATATAT
AGTGTCTATTGTTCATTAGGTATAATCAGCTCGTGGTTTGCCTTATCGTGGTTATATAATTTTGACGAATTACTA
TGGCAGTGA

>YKL004W 0.73 Cold
ATGGCAAACCCTTTTTCGAGATGGTTTCTATCAGAGAGACCTCCAAACTGCCATGTAGCCGATTTAGAAACAAGT
TTAGATCCCCATCAAACGTTGTTGAAGGTGCAAAAATACAAACCCGCTTTAAGCGACTGGGTGCATTACATCTTC
TTGGGATCCATCATGCTGTTTGTGTTCATTACTAATCCCGCACCTTGGATCTTCAAGATCCTTTTTTATTGTTTC
TTGGGCACTTTATTCATCATTCCAGCTACGTCACAGTTTTTCTTCAATGCCTTGCCCATCCTAACATGGGTGGCG
CTGTATTTCACTTCATCGTACTTTCCAGATGACCGCAGGCCTCCTATTACTGTCAAAGTGTTACCAGCGGTGGAA
ACAATTTTATACGGCGACAATTTAAGTGATATTCTTGCAACATCGACGAATTCCTTTTTGGACATTTTAGCATGG
TTACCGTACGGACTATTTCATTTTGGGGCCCCATTTGTCGTTGCTGCCATCTTATTCGTATTTGGTCCACCAACT
GTTTTGCAAGGTTATGCTTTTGCATTTGGTTATATGAACCTGTTTGGTGTTATCATGCAAAATGTCTTTCCAGCC
GCTCCCCCATGGTATAAAATTCTCTATGGATTGCAATCAGCCAACTATGATATGCATGGCTCGCCTGGTGGATTA
GCTAGAATTGATAAGCTACTCGGTATTAATATGTATACTACAGCTTTTTCAAATTCCTCCGTCATTTTCGGTGCT
TTTCCTTCACTGCATTCCGGGTGTGCTACTATGGAAGCCCTGTTTTTCTGTTATTGTTTTCCAAAATTGAAGCCC
TTGTTTATTGCTTATGTTTGCTGGTTATGGTGGTCAACTATGTATCTGACACACCATTATTTTGTAGACCTTATG
GCAGGTTCTGTGCTGTCATACGTTATTTTCCAGTACACAAAGTACACACATTTACCAATTGTAGATACATCTCTT
TTTTGCAGATGGTCATACACTTCAATTGAGAAATACGATATATCAAAGAGTGATCCATTGGCTGCAGATTCAAAC
GATATCGAAAGTGTCCCTTTGTCCAACTTGGAACTTGACTTTGATCTTAATATGACTGATGAACCCAGTGTAAGC
CCTTCGTTATTTGATGGATCTACTTCTGTTTCTCGTTCGTCCGCCACGTCTATAACGTCACTAGGTGTAAAGAGG
GCTTAA

>YNR063W 0.82 Cold
ATGGACAGATCTAAGGACGCTAGAAAGAGATCTATTAGTTTGGCCTGTACTGTGTGTAGGAAACGAAAGTTGAAG
TGCGATGGGAACAAGCCATGTGGAAGATGTATAAGGCTAAATACACCAAAAGAATGCATTTATAATATTGATAAA
AGGAAAGACAAGAGAAAAATCAAAAACGGGTCGAAGGTTTTTTTATTCAAGAATAATACTATCGATAACGGGAAT
AACTCTATACTAGAGAACAAAGGACTAAATGAAGACCTTTCTTCTCATATATATGAGAAAGAAGCGCCAAAATTC
GATTCAGATATTGATATATCAAGATTTGGCACAAATGATGCTGTGATTTTTAATAATGACGGGTGGGACACTTCT
CTTCCGATCGATTTTGATTTCGATGAGTTTAACACTGAGACAACAGATTTCGATGACTTTTTAAAACTATTAGGC
GATAATTCACCTTCAAAGGAACAAAAAAGTCTTTCCTATTCACCTACTGCTACAGGCTTAAGCGGTGTGGTTAAA
GAAACTGAGAGCGAAGATAACGCTCCTACGAGGTCTCGGCTAATCGATGTCTTGTTTGAGAACAAGCTTCACTCC
GTACCAGGAATATCAAAATGGCATCTATATGAGCTGGAATCCCAATACCCAAATTTGGAATGTACAGAAGGAAAT
AGTGATGAAAAGTTTTTACTTTCAACTGTATTGTGCCTGGGGTCGTTGACCATACGAAAAAGGGAACTATTGAAT
CATTCGAACATAGACAATCGTCCACTTTTGCCGGAGAATAGTATTTCAAAACTGACCACTGATGCTTTTAAGTAC
TATAATGCTGCGAAAACGCTTGTTCCCGACTTGTTATCTCATCCCACAATCGATGGATTTTGCGGCCTCGTTCTA

ATGGCAAATTTCATGACGATGATGATATCCTTAGAGCACCAATTATATTTGAGTATAAATGCTTTACAACTTGCT
GTGGCTTTAAACTTGAACAACAATACAAAATGCAAGGAATTACTTGAGTCGAACAGCGACGGAATTGGTGTGATC
TTGCTTTTTTGGAACATTTGGTGTTCTTCTTGCATGTTGGCAACAATTCATGGAAAAAATCCTTTTATCACTTTG
GAACAAATTACAACACCTCTGCCGTGTGAAATATCCCCCCGCAATAAAACTAATAAACTTTTGATAGATTTCATG
CAAATCAGAATCAAGCTAGCCACTCTACAAAGTAAGATTTTTCAACGGCTATATACTTCCAGCACCGCAAACGAG
GTACCATTCGTAAACTTAGAAAGAGAATTTGAGGAGGTTTCACTCCAGATTACCAGGTTAAAAGGCTTTCCGATA
TTCGAAGAACATCTTTTTTACAGGAGCAGAGTCTTAATGTTAGAGCTATCATGTTTAAGAGCTCAAGCTTCTTTT
CTATTATATCGTCCGTATCTGATCACTGGAGAATCCTTACAAGCAGTAACCATGGCAAAATCAATAATTCACGAA
ATATGGAGTCAATACACTAAACAGTTTCCCGATAACGAAAAGGAAAGGCATGAACGTTTGGATTGGAATTTTTGT
TATCCTTTAAGAACAGCGTCACTGACATTATGTATTTCATGTATTATACTCCTAAGGTATAAGCAGGTGGTGCAG
TTCCTTAAAGGTACTGAACTATTTGAATACATTCTAGCATTGGAAATATTGCAAGATTTAGTTCAGGTACTTCCT
ATTGAACAAAACCTTATTGATATAATCAAATATCCGATCAGTCCAGTACAGCTGAGTGGTGATAGCTTTGTCGAA
TTTTGGGGTCGCATACTTTACTAA

>YCL063W 0.79   Cold
ATGGCAACCCAAGCCCTAGAGGATATCACGGAGAGGCTTTTAATAAGGTCGCAAGAGGCTATCTTGCAATTAGAT
CTGTGGATACAACGTCAGCAGAGATCATCAATATGCCAGACAACAGATCAAGAGTCATTGGATAAGTTATCCCAA
CAGTACAACCAGTATATGTCTCAACTGAACTCTTTGTATGTTAGATCGGAATCTGTTCGAGACAAGTTGAGCAAG
GAACAACAACGCAGATTGATCACAGAGGATAATGAGCATCAACGCATAGAAGACTTGGTTCGTGAATTCCAGGAT
ATCACTTTGAGGTTGAACGAGCTGGCCACCGTCCCAAATGAAGCGCCTAATGATTCTCCACAATCGCAATCCACC
AGAAGTAGTTTAGGGTCATTTCAACCTCGACCATTGAAAATAATTGAGAGGCAACGTCTGTGTATGGTAACTCCA
TCGAAACCACCAAAAAAATCGGTAGGCTTTAACCCCATCAATGAAGTCGATTGTCCTTCGAAAACTAACTCTTTA
CCGTGCTCACCTAAAAAACAACCTGCAAGAAATCGCACTTTACGTGCAGCCAAATCACATGATACTGGCTTGAAC
AAAAGTAAGAAACCGTCTTCCTCGGATACGTATGAGTCTTTCTTCAAAAATAGACAAAGACTTTCGTTGACCTTC
TTTGATGAAATGGATGATGAAGATTTTGATTCTGATCAAGATACTATCATTCTACCAAACATAAGTACCCCTCCA
CATGTTGGTGTGACCGCAAAGGGTGCTGAATTCGAACCTTTAAGGAGATATAACTCTCACGAAAGTATACTATCT
AACAAACCAGCACCTTCTAAGTCGCTCAATCTGGGAAGTTTCTCCGCCTCATTCTTCAGGCCATCTAATCCGACG
TTTGGAACTTCGATATCAAATGTCCAAGTGAACTGTCATCCAACTGTTGCAGCGACAATGGCTCCTAGTCGTAAT
GGTCCTCGTATTTCAAGTTCTAAGGCGTTGTTATCATCATTCATTGCACGATCAGATACACATACGGTAAAAGAG
AACAACACAAATCTTAAGCATGCATCTTTTATGGATAAGTTTAATTCATCGTTAAGCACAATATCAGAATCTTTT
CAAAGTAAGAGGGGGAGAAAGAATAAGGGCATGAATGAAGAACGAATATCAAATCATAATGTAGCACAGGAACAA
AAAAATAATATGGATATAAGCGTCTCTATAGAAGAATTGCAAGATGCTTTGAATACAGAACTGCTGTTTTAA

>YHR060W 0.79   Cold
ATGAGTGAAACAAGGATGGCACAGAACATGGATACTACAGATGAACAGTACCTGAGACTTATTGAGCTACTCTCA
AACTATGATTCCACCTTAGAGCAGTTGCAGAAGGGGTTCCAGGATGGCTACATCCAATTGAGCAGATCGAACTAC
TATAATAAAGACTCATTACGAGGAAACTATGGTGAAGATTACTGGGATGAAACGTATATAGGTCAACTAATGGCA
ACAGTGGAAGAAAAAAACTCCAAGGTAGTTGTCGAAATCGTCAAAAGAAAAGCTCAAGATAAGCAAGAAAAGAAA
GAGGAAGAAGATAATAAATTGACTCAGAGAAAAAAAGGAACGAAGCCAGAAAAGCAAAAAACTCAGAGTCACAAA
CTTAAACAAGACTACGACCCAATTTTGATGTTTGGTGGAGTACTGTCCGTTCCTTCTTCATTGAGACAATCGCAG
ACAAGTTTCAAAGGTTGTATTCCCTTAATAGCCCAATTGATTAATTACAAGAACGAAATATTAACGTTGGTTGAA
ACGTTGTCTGAGCAGGAATAA

>YPL215W 0.78   Cold
ATGATGTCAGTCAATAGATTTACTTCAGGTCGACTCCCTGTTTTCCTTAGAAAGTCCCCTTTTTACTACTCGAGA
GCTTATTTGCATCAAACATGTGTTTTCAAGCAAAATAAAGAAACTGCACAAGATTCGCCAGAGCTTTTAGCAAAA
AGCTCACATCTGAATTCGAAACCACTAGATGTAAGCAATAAAGCACCAGTGAAAACTGCTCAGAATAAAATTCCT
CTAGCTCATAGTAAATATGAATCGTCAAAGTACGAACTTCCCAAATGGAAAGAAGCCTTGGGTGAACTAGTAATT
CGAGCATTTCATTTAGATATGGATAGAGTGAGGGCAGGGCCCGTTGCAGGATCTTACTATTATAAAATTTGTAAA
GAGCAAGGTTTACAATACGAGGACGAGCCATTATCAGAAACTGCTAAATACTTTTACGAGGATTTGAAGCTACCA
CGTACTTTTTCACAATGGTTTCAAATCACTGTATTGCACGAGTGGATACTTTTTGTACGTATGAGAGCCATGCCT
TTCAAATACGGCAGAAACTATCAGCAGAAATTGGTAGATAGAACATTTTCTGACATTGAGCTGAGATTATTCGAG
GAAATGAAAGTTAATTCTGGTAGAATCGCAGATCAATATTTGAAAGATTTCAATACGCAATTAAGAGGAGCGATA
TTTGCATATGATGAAGGATTTGCTACAGATGATGGTACACTTGCGACAGCTGTCTGGAGAAATCTTTTTGGTGGA
AGAAAGAACATTGATATGGTTCATTTAGAGTCTGTTGTGAGATACATTTATTCTCAACTATATGTTCTAAGCAGG
TTATCAGACAGAGAGTTTGCCACAGGTAAATTCAAGTTTGTTCCTCCTGGGGTGAAGGTCGAAAAACTGACACCA
AAGCAAGAGGAAGAGTTGAAGGCCAAGACCATTGCAAAATATGAAGCTCTAGACAAGGATCCTAAAACCTTACCA
AGCGAGAGAAGTAGGCTGTCATATACAAACTAA

>YCR037C 0.79   Cold
ATGAGATTCTCACACTTTCTCAAATACAACGCTGTCCCTGAATGGCAGAATCATTACCTAGATTATAACGAATTG
AAAAATTTGATCTACACATTACAGACAGATGAATTGAAACAAGAAACGCCAACCGGTGACTTAAACGATGACGCT
GACTCTCAGACTCCAGGTCCAATCGCTGATATAGAAAGCAACATAGCTGCAGGAGAACCATCTTCATCGAAAAGA
AGATTTACACATAAACTCAAGCGTAAGCTCTTTGGTTCTAAAACACCTTCAGGAAGCAAAAGGGGAGACTCCGAC
GAAAAGGCCATAGATGGGAACAATATTAACGAGGAAACAATTGAGTTAGACGAGTTATCTCCTCAAGGGAAAACC
ACCTCTTTCAATAAGAATTTTATACGTAAGAAATTCTTTGAATCACGCAGCTCATCTGTGAGTAGCGAGGGAAAG
ACGCTCTTCAGTTCTTATGATACATTCGTAACTAACCTGAGCGACGAGAAATTGAAAGTAGATGATTTCTACAAA

AGAATGGAAGCTAAGTTCTATGAAAGATTTGACCACTTGATTAATGATTTGGAGAAGGAAGGCATTGTAACAAGA
TTGAATGAAACTTTCAATCCTGAAATTCAAGCATTGCCTCCTTTAAGAGAAATTATTTCTGGTACATCAGAGACA
CATTCATCTAATAACCCATTTGAAATACACTCTTCAAACATCGACAGTGAATTGAGAAATAGGTTTGATTACAGC
GAAGAAGAAATGGATGAAGATGATGACGTTGACGTGTTTGCTGACACTACCGACAATACCGCCCTCTTGAATTAT
TCGCAATTTAACATTAAATCTCAGAAAAAGTCATTATTAAAACAGACAATAATAAATCTTTACATAGACCTTTGC
CAGTTGAAATCTTTTATCGAATTGAACAGAATGGGTTTCAGTAAAATTACTAAGAAGTCTGATAAAGTATTGCAC
ATGAACACTAGGCAAGAATTAATAGAAAGTGAAGAATTTTTCAAAGACACCTACATCTTCCAGCATGAAACTTTA
AGCAGTTTAAACAGTAAAATTGCACAACTTATTGAATTTTATGCTGTTCTCATGGGTCAGCCTGGGAACGTAGAT
TCATGCAAGCAAGAGTTAAAGTCGTACCTGCACGACCACATTGTTTGGGAAAGAAGCAACACATGGAAAGACATG
TTGGGCCTCTCTTCGCAAAATAACGATATAATAACTATTGAAGATGAAGCTGAGAAACTTATGCAAGAAAAGCTT
CAAATTGAATATTTCAAGTATCCATTGCCTAAGCCAATTAATTTGAAGTTTACTAAAATTGAAAATTTGGCAGTT
CCTAAGCTATTTTTTGGGAAAAGAGCAATGAAAATAGGCTTCATTATCATTGTCACAGGTGTTTTGTTGGGTGTT
AAAACTTTCAATGACCCTGTCGAACACCGGTGTATGGCATTGGTAGAATGCTGTGCTTTCTTATGGGCTAGTGAA
GCCATTCCATTACACATCACAGGTTTATTGGTTCCCCTTCTAACTGTCCTTTTTAGGGTACTAAAAGACGATGAC
GGTAAGGTAATGGGAGCAGCAGCTGCCTCTACAGAAATCTTAGGTACAATGTGGTCGTCAACAATTATGATTTTA
TTAGCAGGTTTCACATTGGGTGAAGCCTTGTCGCAATATAACGTTGCGAAAGTTTTGGCATCGTGGTTATTGGCC
CTTGCAGGTACCAAGCCAAGAAATGTCCTTTTAATGGCAATGAGTGTTGTATTCTTTCTTTCGATGTGGATTTCC
AACGTTGCCTCCCCAGTATTGACATATTCTCTATTAACACCCTTACTAGATCCGCTGGACTACACTTCACCGTTT
GCTAAGGCATTAGTCATGGGTGTTGCACTTTCGGCAGATATTGGTGGTATGGCTTCACCTATTTCTTCGCCACAG
AATATCATCTCCATGCAGTACTTAAAACCTTATGGAATCGGCTGGGGGCAATTTTTTGCTGTCGCTCTGCCTACA
GGTATTCTATCGATGCTGTGCTCCTGGGCCTTGATGATACTCACCTTTAAAATAGGCAAAACTAAACTGGAAAAA
TTTAAACCAATAAGGACCAGATTTACTATAAAGCAATATTTTATCATCATTGTAACTATTGCTACTATTCTTCTA
TGGTGTGTAGAGTCACAAATAGAAAGTGCTTTTGGATCGTCCGGTGAAATTGCAGTAATACCGATAGTCCTGTTT
TTTGGTACAGGTCTACTATCAACAAAGGATTTCAACACATTCCCTTGGTCAATTGTTGTTCTTGCTATGGGTGGT
ATAGCCCTTGGTAAGGCAGTTTCATCTTCAGGCTTGTTGGTAACTATTGCAAGAGCATTACAAAAGAAAATTCAG
AACGATGGTGTTTTTGCTATCTTATGTATTTTCGGTATTTTAATGTTAGTTGTGGGCACTTTTGTCTCACATACT
GTGTCAGCAATCATCATTATTCCCTTGGTGCAAGAAGTTGGTGACAAATTATCCGATCCAAAGGCAGCTCCAATT
CTTGTGTTCGGTTGCGCCTTGTTAGCCTCATGCGGTATGGGGTTGGCTTCATCTGGATTTCCAAACGTTACTGCT
ATTTCTATGACCGATAAAAAGGGTAATAGATGGCTAACTGTAGGCGCTTTTATCTCCAGAGGTGTTCCTGCTTCG
TTGTTAGCGTTTGTCTGCGTAATTACTCTCGGTTATGGTATTAGTTCTTCCGTCTTAAAAGGTAGCACTTAA

>YJR160C 0.73   Cold
ATGAAAAACTTATCTTTTCTCATAAACAGAAGAAAGGAAAATACAAGTGACTCGAATGTATACCCAGGAAAGGCT
AAGTCGCATGAACCCAGCTGGATAGAAATGGATGATCAAACTAAGAAGGACGGCTTAGATATTGTTCATGTTGAG
TTCAGTCCGGATACAAGAGCGCCAAGCGATAGCAATAAAGTAATAACAGAGATATTTGACGCTACTGAGGATGCC
AAGGAGGCAGACGAAAGTGAAAGAGGAATGCCACTTGCGACAGCATTGAATACATATCCCAAGGCAGCAGCTTGG
TCACTATTGGTCTCTACAACTTTAATCATGGAAGGGTACGACACAGCTATTCTTGGAGCTTTCTACGCCTTGCCT
ATTTTTCAGAGAAAGTTTGGCTCACAAAATGACAAAACTGGAGAATGGGAAATTTCAGCGTCATGGCAAATCGGG
CTGACCTTATGTTATATGGCAGGTGAAATTGTGGGGCTACAGCTAACGGGGCCCTCCGTGGATCTTGTTGGAAAT
CGTTACACATTGATTATTGCATTGTTTTTTTTAGCTGCCTTCACTTTTATACTGTACTTTTGCAATAGTTTAGGC
ATGATTGCTGTGGGACAAGCATTGTGCGGGATGCCATGGGGTTGTTTTCAGTGTTTGACGGTGTCTTACGCTTCT
GAAATATGCCCATTGGCTCTGAGATATTACCTTACAACCTATTCAAACTTGTGCTGGTTATTTGGACAACTTTTT
GCAGCTGGAATCATGAAAAATTCTCAAAAAAAATATGCGGATTCAGAATTAGGATATAAACTACCATTTGCCCTA
CAGTGGATTTTGCCAGTTCCTTTAGCACTGGGAATTTTTTTTGCACCTGAATCTCCATGGTGGCTAGTTAAAAAA
GGAAGGTTTGATGAGGCAAGGAGATCACTTAGAAGAACTTTAAGCGGCAAGGGACCTGAAAAAGAAATACTAGTA
ACTCTGGAGGTGGACAAAATAAAAGTGACTATAGATAAGGAAAAGAGACTCACAAGTAAAGAAGGCTCCTATAGT
GATTGTTTTGAAGACAAGATTAATCGAAGAAGAACGAGAATTACTTGTCTCTGCTGGGCTGGTCAAGCCACTTGT
GGTTCAATACTAATAGGTTATTCAACTTACTTTTACGAAAAGGCTGGCGTTAGTACTGAAATGTCCTTCACTTTT
AGTATTATTCAATACTGTCTCGGTATTTGTGCTACATTTCTATCTTGGTGGGCGTCAAAGTATTTTGGTAGATAT
GACCTTTACGCTTTTGGACTAGCCTTCCAAACCATTGTTTTTTTCATTATTGGCGGTTTGGGGTGCTCAAGTACG
CACGGTTCCAAAATGGGAAGTGGTTCTCTTTTAATGGCTGTAGCATTTTTTTACAATTTAGGAATTGCCCCGGTC
GTTTTTTGCCTAGTCTCTGAAATGCCATCTTCAAGACTAAGAACGAAAACAATCATTCTAGCACGTAACACTTAT
AATGTGGTCAGTATCATATGTAGCGTTCTCATCTTATACCAACTGAACTCTAAGAAGTGGAACTGGGGCGCTAAG
TCAGGCTTTTTCTGGGGTGTCTTATGCTTTTGTACTTTAATCTGGGCTGTGGTTGACCTACCAGAAACTGCCGGA
AAGACTTTCGTGGAAATAAATGAGTTATTTAAACTTGGAGTTTCTGCAAGAAAGTTCAAATCGACGAAAGTAGAT
CCGTTTGTTGTCAAAACCCCCCCTAAAGACGTCTCTCATAACGACCCCAAGGGAGATATCGAAGCTTCCATTGCA
GAGGAATAG

>YGL153W 0.81   Cold
ATGAGTGACGTGGTCAGTAAAGATCGTAAGGCATTGTTCGACTCAGCAGTATCCTTTTTAAAGGATGAGTCCATT
AAAGATGCTCCACTTTTAAAGAAAATCGAATTTTTAAAATCCAAAGGGTTAACAGAAAAGGAGATTGAAATAGCC
ATGAAAGAGCCCAAGAAAGACGGTATCGTAGGCGATGAAGTATCGAAAAAAATTGGTAGTACTGAGAATAGAGCC
TCACAGGATATGTATCTCTATGAAGCGATGCCACCAACGCTGCCCCACAGGGATTGGAAGGACTATTTTGTGATG
GCTACTGCCACAGCTGGGCTGTTGTATGGTGCATATGAAGTAACTAGAAGGTATGTGATACCAAATATTTTACCA
GAAGCAAAAAGCAAGTTGGAAGGGGACAAAAAAGAAATTGATGATCAGTTCTCCAAAATCGATACAGTCCTCAAT
GCCATCGAAGCGGAGCAAGCTGAGTTTAGGAAAAAGGAAAGCGAAACATTAAAGGAACTTAGTGACACGATTGCT
GAACTGAAACAGGCGCTTGTGCAGACAACAAGAAGCAGGGAAAAGATCGAAGACGAGTTTAGAATAGTTAAACTC

GAGGTGGTCAATATGCAAAATACGATCGACAAATTTGTTTCAGATAATGACGGCATGCAAGAGTTAAATAATATC
CAAAAAGAAATGGAATCTCTGAAAAGCTTAATGAATAACCGTATGGAATCCGGTAATGCGCAGGACAACAGATTA
TTTTCCATATCTCCTAATGGTATACCTGGCATAGATACGATTCCATCTGCGTCTGAGATTCTTGCCAAAATGGGC
ATGCAAGAAGAAAGTGATAAAGAAAAGGAAAACGGCAGCGATGCTAATAAAGATGACAATGCTGTTCCAGCGTGG
AAAAAAGCAAGAGAACAAACTATTGATAGCAACGCCTCCATTCCAGAATGGCAAAAAAATACCGCCGCCAATGAG
ATCAGTGTCCCTGACTGGCAAAATGGACAGGTCGAAGACTCCATCCCATAG

>YNL228W 0.78   Cold
ATGGTTTGGTGTCACTATATTCTTTTGGTCTTGACTTTCTTTCTTTTCACTACGTTTTTCACTGCCGCATGCCCT
GCTATCTTCACGTGGTTGAATAGCTTATTTCGACTATCAAATGATTCTCCGCATGTAGTGCACACCTCGATCGCG
GAGGTTGGAGACATAGAGGACGGTAGAGTCGACAAGGACGGAGTGCTTTTTGTGGATTTAGAATTCTTTTTAGGT
TGTTTGCCCTTTTTCTTTTTCGCTTTAGTAGACCAATCTTCATCGTCATCCGTTTGTAAGCCCTTGTCTCCTAGT
GATGCCAAAAGATCATCCAACTCATTACTCCGTTTGTCTTTAGTATCATCGAACGATTCAGATTCCTCTGTTTCT
GTGTCAACCTTTGCTTTTTTTTTCTTTTTTCTTTTCTTTTTATTCTTCGTATTCACATGTACATTTTCATCCGAA
CTGACGTCTTCTACCTCTATATCCATTTCTATGTTGAGATTGTCATCTTCACTTTCGTCTTCAGAAGACGATTCT
GCCAGTTTTCTTTCAATTTCAGCGAGTTCAGCTTGCAATGCTTGCAGATCAATATCTTCTTTTTCTTTAACACTT
TCATCTGCTGAATCAAATTTCTCGAGATCGGAGAGATTATCCAACCCAAGCGTTATGTTTTCCTCTTCCATTTCT
TTCCGTATCTCTTCCATATTTTTCTTATGCAGTTTAGTGTTTATGTGGTTTTTCAATTGCTTTTCCGACTTAAAT
GTCTTGTTGCAGATAAAACACTCGTAA

>YIL020C 0.81   Cold
ATGACGAAGTTTATTGGTTGTATAGACCTGCATAATGGAGAGGTTAAACAGATTGTAGGTGGAACGTTAACGAGC
AAAAAGGAGGACGTTCCAAAAACTAACTTTGTATCACAACATCCTTCTTCATATTACGCTAAACTTTACAAAGAC
AGAGATGTCCAAGGATGTCATGTTATTAAGTTGGGACCTAACAATGACGACGCTGCACGCGAGGCACTCCAGGAG
TCACCACAATTTCTACAAGTGGGCGGAGGAATTAATGATACGAACTGTTTGGAATGGTTAAAATGGGCCAGTAAA
GTAATTGTTACGAGTTGGCTATTTACAAAAGAGGGTCATTTTCAATTAAAAAGGTTAGAAAGACTGACAGAACTA
TGTGGGAAAGACCGCATTGTTGTAGACTTAAGCTGTAGAAAAACCCAGGACGGTCGTTGGATTGTGGCCATGAAC
AAATGGCAAACTCTAACTGATCTTGAGCTTAATGCTGACACTTTCAGAGAATTGAGGAAATATACAAATGAGTTT
CTAATTCACGCTGCAGACGTTGAAGGTTTGTGTGGTGGTATCGATGAATTATTGGTTTCTAAGCTTTTCGAATGG
ACCAAAGATTACGATGATTTGAAAATCGTTTATGCTGGTGGGGCCAAAAGTGTTGATGATTTGAAATTAGTAGAC
GAACTAAGTCACGGAAAAGTAGATTTGACATTCGGTAGTTCCTTAGATATATTTGGTGGTAACCTAGTTAAGTTT
GAAGACTGCTGTAGATGGAATGAAAAGCAAGGTTAG

>YLR104W 0.8Cold
ATGAGTCAAAGTCGCTGGAGTATCGTTCTCATCTTTGCATTGTTTATTTTTGGCAGCACTGGAGTGAATGCCTTC
TTCAATTTCGGTCATCATCAGCAACAGCAACAGCAACAGCAACAATCATATGAAGATCAAGTTTTAAATAACCCT
TGTGATGGGTATTTGTGTCCGGACACCCTAACGTGCGTTGCACAGCAGAAAGACTGTCCTTGTCCCTTCCCTAAA
TCTCAGTTGAAATGTGTCCTTCCTGATAATAAATTTGTTTGCATATCCAAACCAGCCACACACAATGAAAAGTTT
AGAGCAATATATGATGACCCTGTCAAGGGCCCCAAAGCAAAGAACAAGGGGTTTAGGGATTGTGGATGGGTATCA
GATGCTTACAAAAACCATTGA

>YDR291W 0.78   Cold
ATGGAGGAAGGACCTATCAAAAAGAAACTGAAGTCAGCAGGTCAAGGGAGTGGAAAGACCGATGCCTTTAGAAAT
TTTGAACAATTTTTTTTCCGCCTTAATACTCTTTACACCTTTTTGATTTGCAGAAAGCATGTTGTGCCTACATTT
AAAACACTTTGTGGACCTATAGAAACTGCTTTGAAAAGAACTGTGACGAAAGAAGACCTAGCCATGGTTATGGCG
CTAATGCCAAGAGAATGTGTTTTCAAGTATATTGATGAGAACCAAATTTATACTGAAACTAAGATTTTTGACTTC
AATAATGGCGGCTTTCAGCAGAAGGAAAATGACATATTTGAACTGAAGGACGTTGATGACCAAAATCAGACTCAA
AAATCTACTCAGTTACTTATATTTGAATTTATAGACGGTACAATGCAACGCTCATGGTCTGCAAGTGATAGATTT
TCTCAAATAAAGATACCAACATATACTACGGAGGAAATGAAAAAGATGATTTCTAAAAGGGAAGCTCTCTTTAAA
TCCAGACTACGGGAATTTATCCTGGAAAAGGAAAAGGCTAACCTTGACCCATTTTCTGAGTTAACAAATCTAGCG
CAGAAATATATTCCAAGGGAGAGAGATTATGAGGATCCTATTGAGGCAATGATGAAGGCGAAGCAAGAAAGTAAT
GAGATGAGCATTCCCAATTATTCCAATAATTCAGTGATAACAACAATTCCTCAAATGATTGAGAAATTAAAAAGT
ACTGAATTCTACGCTTCTCAGATAAAGCATTGTTTTACAATACCATCAAGAACAGCGAAGTATAAAGGTCTCTGT
TTTGAGCTTGCACCGGAAGTATATCAGGGTATGGAACACGAAAATTTCTACAGCCATCAAGCAGATGCGATAAAC
AGTCTTCATCAAGGCGAAAACGTCATTATTACAACATCGACCTCTTCAGGTAAATCCCTTATTTACCAACTAGCA
GCTATCGATCTTCTTTTAAAGGATCCTGAATCAACGTTTATGTATATATTTCCAACAAAGGCCCTGGCACAAGAT
CAAAAAAGAGCATTCAAGGTAATACTTTCAAAGATCCCCGAGCTAAAGAATGCCGTGGTTGATACGTATGACGGC
GATACGGAGCCAGAGGAAAGAGCTTATATTCGTAAAAATGCTAGAGTTATTTTTACCAATCCTGACATGATACAT
ACCAGCATTTTACCTAATCATGCAAATTGGAGACATTTTTTGTACCATCTAAAGTTAGTGGTGGTCGATGAATTG
CATATATATAAGGGTTTATTTGGATCGCACGTGGCATTAGTGATGAGACGTTTACTAAGACTGTGCCACTGTTTT
TACGAAAATAGTGGTTTACAATTTATTTCATGTTCTGCAACTTTAAAGTCTCCAGTACAACATATGAAGGACATG
TTTGGAATTAATGAGGTTACGCTAATACACGAGGATGGCTCACCTACAGGTGCTAAGCATCTAGTGGTGTGGAAT
CCTCCAATTTTACCTCAACACGAACGTAAACGAGAAAATTTCATTAGAGAGAGTGCAAAGATATTGGTACAATTG
ATATTGAACAACGTAAGAACCATAGCATTTTGCTACGTTCGTCGTGTTTGTGAATTGTTGATGAAAGAAGTTCGC
AATATTTTTATTGAAACTGGGCGTGAGGATCTGGTTACAGAAGTCATGTCTTACAGGGGTGGTTATTCTGCTTCC
GACAGACGTAAAATAGAACGTGAGATGTTTCACGGAAATTTAAAGGCTGTCATATCTACCAATGCTCTAGAACTT
GGTATTGATATCGGTGGACTAGACGCAGTCTTGATGTGCGGTTTCCCACTATCAATGGCGAATTTTCATCAACAA

AGTGGTAGAGCTGGAAGAAGAAATAATGATTCTTTAACCCTCGTGGTTGCAAGTGACTCGCCTGTTGATCAACAT
TATGTTGCTCATCCAGAATCCTTGCTGGAGGTTAATAATTTCGAATCTTATCAAGATTTGGTTCTTGATTTCAAT
AACATTTTGATACTGGAAGGACACATACAATGCGCTGCTTTCGAACTGCCAATAAATTTTGAACGTGACAAGCAA
TACTTTACTGAATCTCATCTTCGCAAAATCTGTGTTGAACGCTTACATCACAATCAAGATGGGTACCACGCCAGT
AATAGATTTTTACCATGGCCGTCCAAGTGTGTATCTTTGAGAGGCGGAGAAGAAGATCAATTTGCCGTAGTGGAT
ATAACAAATGGAAGAAATATAATAATTGAGGAAATAGAAGCATCCAGGACTAGTTTTACTTTATATGATGGCGGT
ATATTCATTCACCAAGGCTATCCCTACCTTGTGAAGGAGTTTAATCCTGATGAAAGATATGCTAAAGTGCAAAGA
GTAGATGTTGACTGGGTAACCAACCAAAGAGACTTTACAGATGTAGATCCACAGGAAATTGAACTTATACGCTCT
TTAAGGAATAGTGATGTTCCTGTTTACTTTGGTAAGATCAAGACTACAATTATTGTATTTGGCTTTTTTAAAGTA
GACAAGTACAAGAGGATCATCGATGCCATCGAAACCCATAACCCTCCTGTAATAATCAATTCTAAAGGATTATGG
ATAGATATGCCAAAATATGCCTTAGAAATTTGCCAAAAGAAGCAATTAAATGTAGCAGGAGCTATTCACGGGGCG
CAACATGCAATTATGGGTATGTTACCGCGATTTATAGTGGCGGGTGTGGATGAAATACAAACTGAATGCAAAGCT
CCGGAAAAGGAATTTGCAGAACGTCAAACTAAACGAAAGCGACCAGCTAGATTAATATTCTATGATTCCAAGGGC
GGTAAGTACGGGTCTGGCTTGTGTGTTAAAGCGTTTGAGCATATTGATGATATTATAGAGTCTAGTTTAAGAAGA
ATAGAAGAATGCCCATGCAGCGATGGATGTCCTGACTGTGTAGCGGCTTCTTTTTGTAAGGAAAACAGCCTGGTC
CTGTCGAAACCTGGCGCCCAAGTCGTTTTGCATTGTATTCTAGGTCACTCGGAAGATAGTTTTATAGATCTTATC
AAAGATGGTCCAGAGCCAAATATGCCAGAGATAAAAGTAGAGACTGTTATTCCTGTGTCAGAGCATGTCAACTTT
TCAGATGATTTTAAGATTATTGACGTTAGAAGAGCTACGAAAGACGATACTCATACAAATGAAATCATTAAAAAA
GAGATATGA

>YNR055C 0.81 Cold
ATGGACAAATATACCAACAGGGATCATCCGGACTATATTCCTGGCACATTTAACATCTATTCTTCCCAAAATTTG
GAAAATGGTATTATATACGAATCAAAATTGAAGAAAACCTCCTCAGGAGTCGTCCTAATCCCTCAACCATCATAC
TCGCCAAATGATCCACTGAACTGGTCTAGCTGGAGGAAATTGGCTCATTTTGGTCTAATGGCCTTTATAACTGCG
TTTACCGCCGCTACTAGTAACGACGCTGGTGCTGCTCAAGACTCATTGAATGAGATTTATGGGATATCCTACGAT
TCCATGAACACAGGGGCTGGTGTTCTTTTCCTAGGTATTGGTTGGTCCACTTTATTCTTGGCTCCATTTGCTAAC
TTGTATGGCAGGAAGATTACGTACATAGTTTGCACCACATTAGGTCTCTTTGGTGCGCTTTGGTTTGCTTTGGCC
AAAAGAACGAGCGACACAATATGGTCTCAGTTGTTTGTAGGTATTAGTGAGTCCTGTGCTGAAGCCCAGGTGCAA
CTGTCCCTGAGCGACATTTTTTTCCAGCACCAATTGGGCTCCGTATTGACCGTGTATATCATGTGCACTAGTATC
GGTACGTTCTTGGGCCCATTGATCGCTGGGTACATATCTGCATTTACCAACTTCCGTTGGGTCGGTTGGGTCGCA
GTGATCATATCTGGTGGCCTTTTAATAACTATTATATTTGGCTGCGAAGAAACGTACTTCGACAGAGGCCAGTAT
ATGACCCCTTTGACCAGCTGTCAATCGGGATACGAAGACGGTACCACTTTACAAAACTCTGACAATACGGCCGTG
TCGCGCAGGAAACGTCATCTTGACGCTAAATTATCAACTCCTGGAGCCATGGGTGAGAAAGGTGTAGACCTTTCA
GAGACGGCTGAATTCGAAGTTAACAATGAAGAGGAAGTTACCATACCTGAGACTCGCGAATTGATTGATGGTTCA
AAAGAGCATTTGAAACCATACCCAAAAAGAGTAGCAATATTAACCAAAGCTACTAATTTGAAAGGCTACGGTTTT
AAACAGTATTTCAAATATCTAAAGATCAACCTTAGAATGTTCTTATTCCCGCCAGTGTGGTTGTCTGGTATGTTT
TGGGGTATTCAAGACGTTTTCCTGACGTTTTATTTGACCACTCAAGAAAGCGCCTACTACGAACCTCCATGGAAC
TATAGTGATTTTGGTGTCGCAATTATGAATGTTCCCACACTTATTGGAGCAGTCATCGGTTGTATCTGTGCTGGC
ATTGTTAGTGACTACTTTGTTCTTTGGATGGCTCGTCACAATAGAGGAATTTTAGAGGCAGAATTTAGACTATAC
TTCTCTATCGCAACTGCAATTATTGGGCCAGCGGGTTTGCTGATGTTTGGTATCGGTACCGCTAGACAATGGCCT
TGGCAAGCTATATACGTCGGGTTGGGTTTTGTTGGGTTTGCATGGGGTTGTTCTGGTGATATTGCGATGGCGTAT
TTAATGGATTGTTACCCCGATATGGTTTTGGAAGGTATGGTTTGTACTGCTATTATTAACAACACGATATCTTGC
ATTTTCACCTTTACCTGTTCTGATTGGCTAGCTGCATCTGGTACTGAAAATACTTACATTGCTTTAGCTGTCATC
AACTTTGGGATTACTGCATTTGCTTTACCAATGTACTACTATGGTAAGAGGATAAGACTTTGGACTAAGAGATGG
TATTTGCAATCTGTCAATTTGAGAGACGGTGTGTAA

>YJR032W 0.68 Cold
ATGATTCAAGATCCCCTTGTATATTTAGACATCTCCATTGATAAAAAACCAATTGGTCGTATTGTCTGCAAACTT
TTTCGTGAGAAGGCTCCTAAAACAACAGAGAATTTTTATAAGTTATGCGCTGGTGATGTTAAGAGTCCTTTGAAG
GATCAACAGTACCTGAGCTATAAGGGAAATGGTTTTCATAGAGTCGTTAAGAATTTTATGATCCAGGCAGGTGAC
ATTGTCTTTGGGACACAAAAGGATTCCTCTTCATCGTCTGTTGGAAAAGGTGGATGTTCAATTTACGCTGATAAA
GAGGAAGTGAAAACCGATGATGAATCTTTCTGTTACGGGAATTTTGAAGACGAAAACTTGGGAGAGTTTGTAGAA
CCCTTTACCTTAGGTATGGCCAATTTAGGTTCTCCAAATACGAACAATTCCCAGTTTTTCATAACTACATACGCC
GCACCTCATTTGAACGGCAAGCATTCTATTTTTGGGCAGGTGGTTCATGGAAAATCAGTGGTTCGCACTATAGAA
AACTGCAGGGTAGATTCTGATGGAGTCCCTGAATCAGACGTTAGAATCAGCGACTGTGGAGTGTGGGAAAAAACT
ATGGGTGTCCCGCTTTACAACGCTTCTAATGACCAAATTGGCGGTGACGTATACGAAGAATATCCAGACGATGAT
ACACATTTCGGTGACGATGATTTTGGTAAGGCTCTTGAGGCTGCTAATATAATCAAAGAATCTGGGACGCTCTTA
TTTAAGAAAAAGGATTACTCTAATGCCTTTTTCAAATACAGAAAATCTTTAAATTATATCAATGAATACATGCCT
GAACCCGACGTAGACAAAGAAAGAAACATCCAATTTATTAACCTAAAGATGAAAATTTACCTAAATTTATCTTTG
GTGCTCTTTAATCTGGAAAGATACGATGATGCTATCATGTATGCTACGTATTTATTGGAGATGGATAACGTTCCT
AACAGAGATCAAGCAAAGGCTTATTATAGAAGAGGTAACAGTTACTTAAAAAAAAAGAGACTAGACGAAGCTTTA
CAAGATTATATTTTTTGTAAAGAGAAAAATCCCGATGACGAAGTTATAGAACAAAGAATAGAATATGTCAATAGG
CTCATAGAAGAGAATAAGGAAAAGACAAGAAAGAATATATCAAAGTTTTTCTCCTAA

>YDR531W 0.77 Cold
ATGCCGCGAATTACTCAAGAGATATCTTACAATTGCGATTATGGCGACAATACTTTCAACCTTGCTATTGATATA
GGAGGCACTCTGGCTAAAGTAGTCTTCTCGCCTATACACAGTAACAGGCTGATGTTCTACACCATTGAAACAGAG

AAAATTGACAAGTTCATGGAACTTCTGCATTCTATTATCAAAGAACATAACAATGGATGCTATAGAATGACTCAT
ATAATTGCCACTGGTGGTGGCGCCTTCAAGTTTTATGATTTGTTGTATGAAAATTTTCCTCAAATAAAAGGTATA
TCGAGGTTCGAAGAAATGGAAGGCTTAATTCACGGTTTAGACTTTTTCATTCATGAGATTCCCGATGAAGTTTTC
ACTTACAACGATCAAGATGGCGAAAGGATAATACCCACCAGTTCCGGCACCATGGACTCAAAGGCTATCTACCCA
TACCTTCTAGTCAATATAGGGTCGGGTGTCTCAATATTAAAAGTCACCGAACCAAACAATTTTAGTAGAGTAGGC
GGTTCTTCACTGGGAGGAGGAACTCTTTGGGGCCTATTATCACTAATTACTGGCGCTCAAACTTATGATCAGATG
CTCGATTGGGCACAAGAGGGTGACAATTCTAGCGTTGATATGCTAGTTGGAGATATTTATGGAACAGACTATAAT
AAAATTGGTCTAAAGTCGTCAGCTATTGCAAGTTCATTTGGTAAAGTTTTCCAAAATAGAATGACATCTAACAAA
TCTTTGGAAAACAACGAAAATAAATTATATTCCTCACATGAGTCTATTGAGAAAAACAATGGACAAATGTTTAAG
AATCCTGATATTTGTAAAAGTCTTCTATTCGCCATCTCCAACAATATTGGGCAAATAGCTTATTTGCAAGCTAAA
ATCAATAATATACAGAATATATACTTTGGCGGATCTTATACCAGAGGACATTTGACTACCATGAACACTTTGAGC
TACGCTATTAATTTTTGGTCACAAGGATCAAAGCAAGCGTTTTTCTCAAACATGAAGGCTATTTGGGTGCAATG
GGTGCTTTCCTAAGCGCGTCTCGTCATTCATCTACTAAGAAAACAAGTACGTAG

>YJR007W 0.81 Cold
ATGTCCACTTCTCATTGCAGATTTTATGAAAACAAATACCCAGAAATTGACGATATCGTCATGGTTAACGTCCAG
CAGATTGCTGAAATGGGTGCTTATGTTAAATTGTTAGAATATGACAACATTGAAGGTATGATTCTACTAAGTGAA
TTGTCCCGTAGACGTATTAGGTCAATCCAAAAATTAATTCGTGTTGGTAAAAATGATGTCGCCGTTGTTCTTCGT
GTCGACAAAGAAAAAGGTTATATTGATTTGTCCAAACGTCGTGTTTCTTCTGAAGATATCATTAAATGTGAAGAA
AAATACCAAAAATCTAAGACTGTTCATTCCATTTTAAGATACTGTGCCGAAAAATTCCAAATCCCTTTGGAAGAA
CTATATAAGACCATTGCTTGGCCATTAAGTCGAAAATTTGGTCACGCTTACGAAGCTTTCAAACTATCCATCATT
GACGAAACTGTTTGGGAAGGTATTGAACCGCCATCAAAAGATGTTTTAGATGAATTAAAGAACTATATCTCCAAG
AGATTAACACCACAAGCTGTAAAGATTAGAGCCGATGTTGAAGTGTCTTGTTTTAGTTACGAAGGTATCGATGCC
ATTAAAGACGCATTAAAATCAGCTGAAGACATGTCCACAGAACAAATGCAAGTTAAAGTTAAATTAGTCGCCGCC
CCATTATATGTTTTGACCACCCAAGCCTTGGATAAGCAAAAAGGTATTGAACAACTGGAAAGCGCTATTGAAAAA
ATTACAGAGGTTATTACAAAATACGGCGGTGTTTGCAACATTACCATGCCACCAAAGGCTGTCACTGCTACTGAA
GACGCTGAGTTACAAGCTCTATTAGAAAGCAAAGAATTAGATAATAGATCTGACTCTGAAGACGATGAGGATGAG
TCAGACGACGAGTAA

>YCR026C 0.74 Cold
ATGGAACTTCAGAATGATTTAGAGTCGCTCGATAACGAGCTGAATGATTTTAGTGAAGATCCATTTCGTGATGAT
TTCATAACGGATGAAGATGCTGTAAGATCGGGGTGGCGATCTGCGTGGACCAGGATGAAATATTGGTTTTATAAG
AATAGACTGAAGTGGACAAACAATCCCATAGTGATTGGCGACGCGAAAGATAGTAGGGATGGTTCTAACTTTAGA
AGGGGTATACCGCTATATGAATTAGACGCGAATGGTCAACCCATTGATACTGAACTTGTTGATGAGAATGAACTT
TCTTTTGGAACGGGATTTCATTCCAAAGTGCCTTTTAAAATAATATTTCGCACATTGTTTGGCTCGCTGGTGTTT
GCCATTTTTTTAATTCTGATGATTAACATAGCAAAACCCCATCACTCCACGAGAGTGCTATCGCACTTTGGCAGT
CCTGAATTTGACCCTTACGTGAAGTATTTTAACGGTACGCATGAATTTTTCCCCTTAACGATAGTAATTTCACTA
GACGGTTTCCATCCTTCACTCATATCTAAGAGGAACACACCGTTTTTACATGACTTATATGAATTGAAATATGAT
GGAGGTATGAATATCACGTCCACACCTTTTATGGTACCCAGCTTCCCTACGGAGACCTTTCCCAACCATTGGACG
TTGGTTACTGGACAATACCCAATACACCACGGTATAGTCTCTAACGTATTTTGGGATCCTGATCTTAATGAGGAA
TTCCATCCAGGTGTATTGGACCCTCGAATATGGAACAATAATGATACAGAACCAATATGGCAAACTGTTCAGTCT
GCATTTGACGGTGATATACCATTCAAAGCTGCTACCCATATGTGGCCAGGTAGCGATGTGAATTATACCAAGTAT
AATGAAGAGAAACTACAACCTGAACATAAAAATCCTATTGCTAGAGAGAGAACTCCATTTTACTTCGACGAATTC
AATGCTAAAGAACCACTTTCGCAAAAATTATCCAAGATTATTGAATATGTGGATATGAGTACACTGAACGAAAGA
CCACAGCTAATTCTCGGTTATGTACCGAACGTAGATGCCTTTGGACATAAGCATGGATATCCGTCAGAGTCGGAA
TACTATTATGAAGACTTCACTGAAACACTGGGGGAAGTAGATACATTTCTGAAGCAACTAGTGGAATCGCTGCAA
GAAAGAAATTTAACCAGCTTTACTAATTTGGTCATTGTTAGCGATCATGGTATGAGCGATATCGTAGTTCCCTCA
AATGTTATTATATGGGAAGACTTACTGGACGAAAAATTGAGGAAGGATTATGTATCGCACGCATATCTAGAGGGT
CCGATGATGGCTATATCGTTGAAAGATTCCGGAAACATCAATGAGGTTTACCACAATTTAAAGACTTCTATAGAT
GAAGACAAGTATACGGTTTACGTTAATGGAAATTTCCCCAAAGAATGGAACTTTAATGATGGAAAAAATCATCAC
ATGGCGTCAATCTGGATTGTGCCCGAGCCTGGGTATGCAGTGATGAAGAAAGAACAATTGAAGAAGGTGGCAAAA
GGTGATCATAAGGACAAAAACGAAGACAATGTGTTCACGATTGGATCACATGGATACGACAATAACGCGATCGAT
ATGAGATCTGTATTTATTGGTATGGGGCCATATTTTCCACAGGGATACATTGAGCCGTTCCAAAATACCGAAATT
TACAACCTTTTGTGTGATATTTGCGGTGTGGCAGAAAAGGACAGAAATTCCAATGATGGGACTGGGATGCTTATG
AACCAACTCCGCGAACCCCAGAGCAGCGAAGAAGTAGAGATTGAAGATGACTTTGATTATTTGGTCAGTAAGTTT
GGTGAATTCAGCACTTATAATATAATTTGGGGCGGGTACCCCGAAGAGACAGAACAAGACAATGTTGACAATGAT
AATGATGACAACGACGATGGAAACACTGATGAAATAGCCGCTATGCCATCTTCGTCATTAACGATAAAACTAGAA
ATGACAACTTCAATACCATCAGCAACTGAGACTCTACTGGGCGAAACATCACCATCATCAAGAAGCAGCAGCAGC
AGCAGCATACAAGCTAGCGCTACTGCTAGCACAGTGGGGGATTGGCTTCAAGACATAATCAACGACGCAAAAGAT
CTCATTGACGACATAATTGACAGCATCGACGATTTAGTCGATTCTGATACCTAA

>YNR026C 0.82 Cold
ATGAAGTTCGTGACAGCTAGTTATAACGTCGGGTATCCTGCGTACGGTGCAAAATTTTTGAATAACGACACATTA
CTTGTGGCAGGCGGTGGAGGAGAAGGAAACAATGGCATACCAAACAAGCTGACGGTCTTGCGCGTGGATCCTACC
AAAGATACTGAGAAGGAACAGTTTCATATATTGAGCGAGTTTGCATTGGAAGACAACGACGACTCTCCTACTGCA
ATTGACGCTTCCAAGGGTATCATTTTGGTTGGCTGCAATGAAAATAGCACTAAGATTACCCAAGGTAAAGGTAAT
AAGCACTTGAGAAAATTTAAATACGATAAAGTGAATGATCAATTGGAGTTCCTCACTAGTGTAGACTTTGACGCA

TCTACAAATGCGGATGACTACACGAAGCTGGTTTATATTTCACGAGAAGGTACCGTTGCAGCTATCGCATCATCT
AAAGTACCTGCTATAATGAGAATCATTGACCCGAGCGACTTGACAGAGAAGTTTGAGATCGAGACTAGGGGTGAA
GTAAAGGATTTACACTTTTCCACTGATGGTAAGGTTGTTGCTTATATCACCGGTTCTAGCTTGGAAGTGATTTCA
ACAGTGACTGGAAGTTGCATTGCTAGGAAAACAGATTTTGATAAGAATTGGAGTTTATCTAAAATAAACTTCATA
GCCGATGACACAGTATTGATAGCAGCCTCTTTAAAAAAAGGGAAAGGTATTGTGCTGACCAAAATAAGCATCAAA
TCAGGAAACACTTCCGTATTAAGATCCAAACAAGTGACAAACAGATTCAAAGGGATTACTTCTATGGATGTCGAC
ATGAAGGGTGAATTGGCGGTACTGGCAAGTAATGACAATTCCATAGCTCTTGTGAAACTAAAAGACCTGTCAATG
TCTAAAATATTCAAACAAGCTCATAGTTTTGCCATTACAGAGGTCACTATCTCTCCGGACTCTACATATGTGGCG
AGTGTTTCGGCAGCCAACACTATCCACATAATAAAATTACCGCTTAACTACGCCAACTACACCTCAATGAAACAA
AAAATCTCTAAATTTTTCACCAACTTCATCCTTATTGTGCTGCTTTCTTACATTTTACAGTTCTCCTATAAGCAC
AATTTGCATTCCATGCTTTTCAATTACGCGAAGGACAATTTTCTAACGAAAAGAGACACCATCTCTTCGCCCTAC
GTAGTTGATGAAGACTTACATCAAACAACTTTGTTTGGCAACCACGGTACAAAAACATCTGTACCTAGCGTAGAT
TCCATAAAAGTGCATGGCGTGCATGAGACGAGTTCTGTGAATGGAACTGAAGTCTTATGTACTGAAAGTAACATT
ATTAATACTGGAGGGGCAGAGTTTGAGATCACCAACGCAACTTTTCGAGAAATAGATGATGCTTGA

>YEL029C 0.77   Cold
ATGCCTCGTCTCTTGGCCACGCAGTCTCATGTTGTACATGGATATGTGGGAAATAAGGCTGCAACGTTTCCCTTA
CAGTGTCTAGGCTGGGATGTGGATTGTTGTAACAGTGTTCAGTTTTCCAACCATACCGGATATGGTTTAGATAAA
GTGTTTGGGACTATAACGAGGGAAACAGATTTGAAGGAACTCTTATCAGGTCTTTTTGATAATTTTTCCCAAGAC
TACCAAGCCTTACTTTCAGGCTACTTGCCCAATAAGAATTCCGTTCGATGTATGGGAACATACTACGCTAAATTT
AAAGAGGCGAATCCAGAAATGATATGGCTTATGGATCCTGTTATGGGTGACGAAGGACAATTATATGTTAGTGAG
GATGTCATACCAGAATATAGAAAGCTGGCTTTGTCTCCAAAACAGCTAGTCGATATAATAACTCCGAATCAATTT
GAGCTGGAGATACTTTATGGGGGGGAAATCAAGACTAAAGAACATTTGAAGAAGGCCTTAAAGAAATTACACCAA
ACTATCCCAGTGATCATTGTCACATCCTGTGACTGTAAGATGTTTGATGACAAAGATTTTATTTATTGCGTAGCT
TCAATGGAGGGTAAAACCCCCATTGTTTACAGAGTGCCGTTTATTGATTCGTATTTCACTGGGGTTGGTGATTTG
TTTTCTGCTTTGTTATTGGATCGAGTGTATAAGATCTTATCGAACCCTACAACAACATTAAAATTTGAAGACCAA
GTCAATAATGTTCTTAATGTTATTCAGAAGGTTTTGAAAATTACTAGGAGCTATGCTTCAGGAAAAAATGAAAGCA
AAAATGGGTTCCGCCTTGGAAATGAAAGAAATGGAATTGCGTCTTATTGAATCAAGAGATATATATGAAACAATC
AACATTCATCAAACAGATTATATTTACGCAAGGTTGTGA

>YNL238W 0.71   Cold
ATGAAAGTGAGGAAATATATTACTTTATGCTTTTGGTGGGCCTTTTCAACATCCGCTCTTGTATCATCACAACAA
ATTCCATTGAAGGACCATACGTCACGACAGTATTTTGCTGTAGAAAGCAATGAAACATTATCCCGCTTGGAGGAA
ATGCATCCAAATTGGAAATATGAACATGATGTTCGAGGGCTACCAAACCATTATGTTTTTTCAAAAGAGTTGCTA
AAATTGGGCAAAAGATCATCATTAGAAGAGTTACAGGGGGATAACAACGACCACATATTATCTGTCCATGATTTA
TTCCCGCGTAACGACCTATTTAAGAGACTACCGGTGCCTGCTCCACCAATGGACTCAAGCTTGTTACCGGTAAAA
GAAGCTGAGGATAAACTCAGCATAAATGATCCGCTTTTTGAGAGGCAGTGGCACTTGGTCAATCCAAGTTTTCCT
GGCAGTGATATAAATGTTCTTGATCTGTGGTACAATAATATTACAGGCGCAGGGGTCGTGGCTGCCATTGTTGAT
GATGGCCTTGACTACGAAAATGAAGACTTGAAGGATAATTTTTGCGCTGAAGGTTCTTGGGATTTCAACGACAAT
ACCAATTTACCTAAACCAAGATTATCTGATGACTACCATGGTACGAGATGTGCAGGTGAAATAGCTGCCAAAAAA
GGTAACAATTTTTGCGGTGTCGGGGTAGGTTACAACGCTAAAATCTCAGGCATAAGAATCTTATCCGGTGATATC
ACTACGGAAGATGAAGCTGCGTCCTTGATTTATGGTCTAGACGTAAACGATATATATTCATGCTCATGGGGTCCC
GCTGATGACGGAAGACATTTACAAGGCCCTAGTGACCTGGTGAAAAAGGCTTTAGTAAAAGGTGTTACTGAGGGA
AGAGATTCCAAAGGAGCGATTTACGTTTTTGCCAGTGGAAATGGTGGAACTCGTGGTGATAATTGCAATTACGAC
GGCTATACTAATTCCATATATTCTATTACTATTGGGGCTATTGATCACAAAGATCTACATCCTCCTTATTCCGAA
GGTTGTTCCGCCGTCATGGCAGTCACGTATTCTTCAGGTTCAGGCGAATATATTCATTCGAGTGATATCAACGGC
AGATGCAGTAATAGCCACGGTGGAACGTCTGCGGCTGCTCCATTAGCTGCCGGTGTTTACACTTTGTTACTAGAA
GCCAACCCAAACCTAACTTGGAGAGACGTACAGTATTTATCAATCTTGTCTGCGGTAGGGTTAGAAAAGAACGCT
GACGGAGATTGGAGAGATAGCGCCATGGGGAAGAAATACTCTCATCGCTATGGCTTTGGTAAAATCGATGCCCAT
AAGTTAATTGAAATGTCCAAGACCTGGGAGAATGTTAACGCACAAACCTGGTTTTACCTGCCAACATTGTATGTT
TCCCAGTCCACAAACTCCACGGAAGAGACATTAGAATCCGTCATAACCATATCAGAAAAAAGTCTTCAAGATGCT
AACTTCAAGAGAATTGAGCACGTCACGGTAACTGTAGATATTGATACAGAAATTAGGGGAACTACGACTGTCGAT
TTAATATCACCAGCGGGGATAATTTCAAACCTTGGCGTTGTAAGACCAAGAGATGTTTCATCAGAGGGATTCAAA
GACTGGACATTCATGTCTGTAGCACATTGGGGTGAGAACGGCGTAGGTGATTGGAAAATCAAGGTTAAGACAACA
GAAAATGGACACAGGATTGACTTCCACAGTTGGAGGCTGAAGCTCTTTGGGGAATCCATTGATTCATCTAAAACA
GAAACTTTCGTCTTTGGAAACGATAAAGAGGAGGTTGAACCAGCTGCTACAGAAAGTACCGTATCACAATATTCT
GCCAGTTCAACTTCTATTTCCATCAGCGCTACTTCTACATCTTCTATCTCAATTGGTGTGGAAACGTCGGCCATT
CCCCAAACGACTACTGCGAGTACCGATCCTGATTCTGATCCAAACACTCCTAAAAAACTTTCCTCTCCTAGGCAA
GCCATGCATTATTTTTTAACAATATTTTTGATTGGCGCCACATTTTTGGTGTTATACTTCATGTTTTTTATGAAA
TCAAGGAGAAGGATCAGAAGGTCAAGAGCGGAAACGTATGAATTCGATATCATTGATACAGACTCTGAGTACGAT
TCTACTTTGGACAATGGAACTTCCGGAATTACTGAGCCCGAAGAGGTTGAGGACTTCGATTTTGATTTGTCCGAT
GAAGACCATCTTGCAAGTTTGTCTTCATCAGAAAACGGTGATGCTGAACATACAATTGATAGTGTACTAACAAAC
GAAAATCCATTTAGTGACCCTATAAAGCAAAAGTTCCCAAATGACGCCAACGCAGAATCTGCTTCCAATAAATTA
CAAGAATTACAGCCTGATGTTCCTCCATCTTCCGGACGATCGTGA

>YER051W 0.81   Cold

ATGCAAGATCCCAATATTTGCCAGCATTGCCAGTTGAAGGATAATCCAGGCGCATTAATTTGGGTGAAGTGTGAT
AGTTGCCCGCAGTGGGTCCACGTGAAATGCGTGCCTTTGAAACGCATTCACTATTCAAATCTTACAAGTTCTGAA
GTTCTGTCTTATCCAAATTCTGCGAAGCAAATCAAGAGCTACCGTTGTCCTAATCATAAGGAAGGAGAATATCTT
ACCGCATACGCTCTCATCACACAAAAAGGAAAGCGGCAAAGGAATAAAGAAAACCCTGAAGATAGTCATATAAAT
AAGCGGTATAATTTCAGAAAGAAGAAATTACTTGACTATATCGCTTTGAATGAGGGTGAATCGAAAAGGATAAA
ATGAATCACCCTCATAAGGAGAGTTTCATGAAATCTTTTGAAAAATGGAAAAATGGCTCAAATATTATAAACGCC
GCTGACTTTGCTGAAAAGTTTGATAATATAGATGTGCCGTACAAGATCATCGATCCACTGAATAGCGGAGTATAT
GTACCGAATGTGGGCACAGACAATGGATGCCTCACAGTTAATTATATCACCGAAATGATAGGCGAGGATTATCAT
GTTGATGTAATGGACGTTCAATCACAAATGAATGAAAATTGGAACTTGGGATCTTGGAATGAATATTTTACAAAT
ACTGAACCAGACAGGAGGGATCGAATAAGGAATGTTATATCATTAGAAGTCTCTAATATTGAGGGATTAGAACTA
GAGAGGCCCACTGCAGTTAGGCAGAATGATCTTGTTGATAAAATTTGGAGTTTCAATGGACATTTAGAAAAAGTC
AATGGGGAGAAGGCGGAGGAGAATGACCCCAAGCCAAAAGTGACCAAATATATTTTGATGTCTGTAAAGGATGCT
TATACGGATTTTCATTTGGATTTTGCCGGTACCTCTGTTTATTATAACGTTATCTCAGGACAGAAGAAGTTTTTA
TTATTTCCACCTACCCAATCAAACATAGATAAGTATATTGAGTGGTCTTTAAAAGAAGACCAAAATAGTGTTTTC
CTCGGTGATATTCTTGAGGATGGTATTGCGATGGAATTAGATGCTGGTGATTTGTTTATGATTCCAGCTGGATAT
ATTCATGCAGTTTATACACCAGTAGACTCTTTGGTATTTGGAGGCAACTTTTTAACCATCCGTGATTTGGAGACA
CACCTTAAAATTGTGGAAATTGAAAAGTTAACAAAGGTTCCTAGAAGATTTACCTTCCCGAAGTTTGATCAAGTG
ATGGGTAAATTATGCGAGTATCTTGCGCTTGATAAAAATAAAATCACTAGTGATGTCAGTGATGGGGATTTGCTT
TCCAGGACCACTAATTGCGCAATTCAATCACTTCATGCATACGTTATAAAACCTGAAGTTAAGTACAAGCCGTTA
AATTTCACTTCAAAGAAGCATTTAGCGAAAGCTTTAGCCGATCTTATTTCGTAA

>YOR110W 0.7Cold
ATGGTGGTGAACACGATATATATCGCAAGGCATGGATACAGATCCAACTGGCTTCCGGAGGGTCCATATCCTGAC
CCACTTACAGGAATTGATAGCGATGTTCCTTTAGCAGAACACGGAGTCCAGCAAGCCAAAGAGTTGGCCCATTAC
CTTTTGTCCTTGGATAATCAACCAGAGGCCGCCTTCGCTTCTCCATTTTATAGATGTCTTGAGACTGTACAACCG
ATAGCTAAGCTGTTAGAAATACCTGTTTATCTAGAAAGGGGTATCGGGGAATGGTATAGACCTGATAGAAAACCT
GTTATTCCAGTACCTGCTGGATATGAGATACTAAGCAAATTTTTTCCAGGTGTCATTAGCCAAGAATGGGACTCT
ACATTAACACCAAATGAAAAGGGTGAGACAGAACAGGAGATGTATATGAGATTCAAAAAATTTTGGCCCTTATTT
ATTGAACGTGTAGAAAAGGAATATCCAAATGTTGAGTGCATACTGTTGGTCACACACGCTGCATCTAAGATTGCC
CTTGGAATGAGCTTGCTGGGATACGATAATCCTCGGATGTCTTTGAATGAAAATGGTGATAAAATAAGAAGTGGT
AGTTGCTCATTAGACAAATACGAAATTCTTAAAAAAAGCTACGATACTATAGATGAAACTGATGATCAAACATCC
TTTACCTATATACCATTTAGTGACAGAAAATGGGTTTTAACGATGAATGGGAATACCGAGTTTTTGAGTAGTGGT
GAAGAAATGAATTGGAATTTTGATTGTGTGGCAGAAGCTGGTTCAGATGCTGATATCAAAAAGAGACAAATGACA
AAAAAAACCAGCTCACCAATACCAGAAGCAGATGATCAAACGGAAGTAGAAACCGTTTATATCAGTGTTGATATT
CCCAGCGGCAATTACAAAGAGAGGACCGAGATAGCAAAGAGCGCAATTTTACAATATTCCGGTTTAGAAACAGAT
GCTCCGTTGTTTAGAATCGGAAATAGATTGTATGAGGGAAGCTGGGAGAGACTTGTTGGCACGGAGCTTGCTTTC
CCAAATGCTGCACATGTACATAAAAAGACAGCCGGTCTACTATCACCGACTGAAGAGAATGAAACAACAAATGCT
GGTCAAAGTAAAGGCTCATCGACTGCCAACGACCCAAACATACAAATACAAGAAGAAGACGTTGGACTACCGGAT
TCAACTAACACAAGTAGAGATCACACGGGAGATAAAGAAGAGGTTCAATCAGAAAAGATTTATAGAATAAAAGAG
AGGATAGTGCTAAGCAATGTCCGCCCTATGTAA

>YIL067C 0.76   Cold
ATGGGTGTGCATTTTGATGATAATGCTAACACAACGTGGGAAGCCACAGACCCTGGCGTTTCAAGTGATTGCGAT
GGACAGCACCGTGTGACTGAGTCTATACAACTGCAGAACTTTTCCAATACAGACATGGAAAGTATGCTTGATGAA
GAAGGGAGAGAAAACAGTAAGTCAAAATGGTTACTATTAAAAAGGAAACATCCTATACAAAAGTTTATTGAAAGA
GTTTGGAATGGCCCTGTTGAGCCAAGTGACGAACCGCCTAGCTTTCCTAAACGTTGGGGATGGCTGAAGAAGATA
GATGATTTTCCGCAGACTACATTCAAAACTAAAATACCAAGTAAGTTGATACGACTGTTGCTACTGATTGTGTAC
TGCTGCTTTTGGATGCGCATTTTTTATTCTCTCATATACCCCTATCTTATCAAGCCGCCATACTTCCATCCAAAT
GATGGCTCAGAAAAGATTCCTATATTGTCTTTAAGTTGCAATTCCTACTTGAACTGGGAGGGTACCAATAACGAA
TGTGGTTTAAATGCTAAAAACTGCGGTCCACTTGATAATAAAGAATACATGATCAGATGTCCAGCACTGTGTGAT
AGAGGTGGATGGACATACTCCGCCATTGCCGTAGGGAATAGGAGAGTCAAATATACAGGGTATGAAATTGGCGGA
GGTGCCCTGTTTAGCGAAGAAGACCCGATGGTCGTGTCCTACCCTTATAGAAGTGACTCGTTTCCATGCGCCTCT
GCAGTACATGCAGGTGTTATCTCACCATTTTACGGTGGCTGTACCAAAGTTTCTATGCAAGGGGCCCAAAATTCT
TTTCCATCAAAAAAAGGAATGTATAACACTGGGTTTTCAGTAGCATTCAATTCATTTTTTCCCGGTTCATATTCA
TTCAGAGATATACAAGGCGGTATCCTGTCGGGCTGTTATGATCCTCGAGCAGCTGTGGTCGCATTGAATATGCTT
TTTGGACTGCCAATATTTTATTTGTATGACAGTATTTACGGGTACTGGATAAACACGATAGTGGGCTATTGGACT
CTTGTATTATCTCTAGACCCTCCATTACTAACGGATGCACATGACCCAGCTAGCGTGTATGAGCTTTTCAGTGTG
GGATTTCAAAGACTTTTACCTTTATGCTTTGTTTTATACGTCGTTTGGAAGAGTGCGGTGAAAAGAACACTAGAA
AATGGATCGCCCATAGCAAAAGTAATACTTTGGTACCCCACATTTTGGTTGGGAATCTCAAATAATGTCACATTT
GACCGATTACCTGTTGATAGACTTACGACGACGGATTTGAAAGAGCAGGCTGGAGCACTGACTGCTGTGGGATCT
ATTGCAGCCACTATTCTTACATGCGCAGTTATACAGGCTTATTCACTCTGGAAATCAGGAAGATTCAAAAAGTAT
TTTAAAATATACATTTGTTTCATTGGCGGATTAATAGCTTTGGGATCTTTGCCGGGACTGAACTTGAGGATACAT
CACTACATCTTAGGTTCTATACTGGTTCCAGGCTGTGCCACGAGAGGATCATCTGCATATCTGTTCCAAGGAATT
TTAGTCGGGTTAATTCTGTCTGGTGTAGCAAGATGGGATTTTGCAAGCATAGTTGAGACAGATACGGCCTTGTTA
AGAGGAGAAGCAGGTGCTTCTTTGAAGCCACCTATACTGGATTTTAACGACGATCAAAACCATTCTCTGTCGTGG
CATCTAAATGCAACTGATCCAGTAATTGATCAAATAGGAAACATCGACGGTTTTTCATTACTTTTGAACGATGTG
GAAGTGTACGTAGGCAAGAATGAGACTGTGAGTATCGATGTTCTTCGGATGGAGAATCCAGCACTTGCTCAAATG

ATGGACGATGCTCTTGATGCCTCTAACGGGACAATAGACTTGTATTTAAGAGTGGCTCGTGCCTCTGTACGGTCT
CCAACGAATAGAGGTGACTATACGAATGCAGGAGTTTTACAGTGGCCGAACGGAATGTGGCAAAAGCCTGAACCA
GGCGTATCATAA

>YBR194W 0.76 Cold
ATGGATCAAAAGAAGGACCCAAGTAATAACTTAACAGAAAGAAGGGTTTCCAAGGTCCAACGCCCAAATAAGAAA
AAGGTACGAAATCAAGTTGAATCACTTTCTAGAAACCTAGAGAGAAATAAAGAGGGACAGCTTCTGCAGACTGTA
TCAAAAGGTCATTTAGAAGCTGATTCTGGACACTCACTGGGTCGTGAAAAAGAGAACGGTGAACTCGGTATAAGA
AGTATATTTTACGATAAAGACTGGAATCCAAGAGGCACTGCACCATCACATTACAGAAATATACCTTATAATCCT
GCCACTTTCAAAAGGCGAACTGAAGTTCAAGCCAGACTGGGAAACCTCGAGAATATCAAAATACCAAAGTGA

>YBR180W 0.81 Cold
ATGGGAAGCGAACCGTTTCAGAAAAAGAATTTGGGTCTGCAAATTAATTCGCAAGAAAGTGGAACAACCCGCTCA
ACATTTCATTCGCTAGAAGATCTAGGAGATGATGTAATTAATGAAAGCTGGGATCAGGTGAACCAAAAGAGAGCC
AATATAGATCATGATGTCTTTCATGAGCACCCTGACTCTTCCCCATCATTGTCAGCACAGAAAGCAAAAACAAAA
GAAGAGGAAGTTGCTGTAAAGTCATCGAACTCCCAGTCAAGAGACCCTTCTCCTGATACTCAAGCACATATTCCA
TATACTTATTTTTCCAAGGATCAAAGACTAATCATTTTTGGAATTATCATTTTTATAGGATTTTTGGGCCCAATG
TCTGGAAACATATATATACCGGCTTTACCATTGCTGCAAAGGGAATATGATGTAAGTGCAACAACAATAAACGCT
ACAGTTTCTGTATTTATGGCTGTTTTTTCCGTTGGTCCATTGTTTTGGGGCGCACTGGCGGATTTTGGTGGAAGG
AAATTCTTATATATGGTGTCGTTATCACTAATGTTAATTGTTAATATACTTTTGGCCGCTGTACCAGTCAATATT
GCTGCCCTTTTTGTTTTAAGAATTTTCCAAGCTTTCGCTTCCAGCTCTGTGATTTCTCTGGGAGCTGGCACTGTA
ACAGACGTTGTTCCTCCAAAACACAGGGGAAAGGCCATAGCGTATTTCATGATGGGTCCAAACATGGGTCCTATT
ATAGCACCCATTGTTGCTGGGCTTATTTTAATGAAAGGAAATTACTGGAGATGGCTTTTTGGTTTCACTTCTATC
ATGACAGGAATAGCATTGATCTTGGTTACTGCTTTACTTCCAGAAACGCTACGTTGTATAGTTGGTAATGGAGAC
CCTAAATGGGGTGACAAAAAAGATGAACGTGAAAATAACGAATCTCCATTCTTCGAAGGTAATAAAATATCACAT
CGGCGTCTGTTCCCAGACATTGGTATCCGTAAACCAGTCAATAATGATGCTTTCTTCCAAGAAAATTTTCCAAAG
CCGCCTAAAGCAGGTTTGACACTATATTGGAAAATGATTAAATGTCCTCCAATAATAATCACGTCCGTCAGTACT
GCACTCCTGTTCTCCAGTTACTATGCGTTCAGCGTCACGTTTTCGTATTACCTTGAACATGACTACCGTTTTACT
ATGTTAGAAATTGGTGCTGCTTATGTCTGCCCAGGTGTAGCTATGTTACTAGGATCTCAATCTGGTGGGCACCTC
TCAGATTATCTTCGTTCACGCTGGATCAAAAGTCATCCTAAAAAGAAATTCCCGGCAGAGTTTCGTTTATTACTG
AACCTAATTGGAATTTTACTAACAATATGTGGCACAATAGGATACGGATGGGCAATCTTCTTTCATTATCATTTT
GTGGTTCTTTTAGTTTTTTCCGCTCTCACTGCCTTTGGTATGACCTGGTGCAGCAACACATCAATGACATATTTA
ACTGAGTTATTCCCCAAAAGAGCTGCTGGAACTGTTGCTGTTAGTAGTTTCTTTCGAAATGTGGGCGCTGCCATT
AGTTCCGCTATCATTTTACAGCTCTGTAACGCAATGGGAATTGGATGGTGTTTTACAGGGCTCGGTCTCTGCAGT
TCAATTTCATTGATTGGTATATTATATCTTCTCATTTTTCAAAGAAAATATACTGCCAAAGAATTTTAA

>YIL036W 0.81 Cold
ATGTTTACTGGTCAGGAGTATCATTCCGTAGACTCTAATTCCAACAAGCAAAAAGACAACAATAAACGTGGTATT
GATGACACATCAAAGATCTTGAATAATAAGATACCGCACTCTGTTAGTGATACTTCTGCCGCCGCCACCACCACT
TCTACTATGAACAATTCTGCTTTAAGTAGATCCTTAGATCCTACTGACATAAACTATAGCACAAATATGGCTGGT
GTGGTTGACCAAATACATGATTATACTACTTCCAATAGAAATTCTTTAACCCCACAATATTCTATTGCAGCTGGA
AACGTCAATTCGCATGATCGGGTTGTTAAACCCAGCGCCAATTCAAACTATCAGCAGGCTGCATACCTTCGACAA
CAGCAACAGCAGGATCAGCGACAACAGTCACCCTCTATGAAAACTGAAGAGGAATCCCAACTCTACGGTGATATT
CTGATGAATTCTGGTGTCGTACAGGATATGCATCAGAATCTGGCCACTCATACAAATCTGAGCCAACTGTCGTCT
ACCCGTAAGTCCGCTCCGAATGATTCTACTACAGCCCCGACTAATGCGTCCAACATCGCCAATACGGCTTCTGTG
AACAAGCAGATGTATTTCATGAACATGAATATGAATAACAACCCACATGCCTTGAACGATCCATCCATCCTGGAA
ACATTGTCGCCATTTTTTCAACCTTTTGGTGTTGATGTAGCACATTTACCTATGACGAATCCACCAATTTTCCAA
AGTTCTTTGCCTGGATGCGATGAGCCAATTAGAAGAAGAAGAATATCAATCTCTAACGGTCAAATAAGCCAGCTA
GGCGAAGATATTGAAACTTTGGAAAACCTGCACAACACACAGCCGCCCCCGATGCCCAATTTTCACAATTATAAT
GGTCTGAGCCAAACTAGGAATGTATCAAACAAGCCGGTCTTCAACCAAGCAGTGCCGGTTAGTAGTATTCCACAA
TACAATGCAAAAAAAGTTATTAATCCCACGAAGGACTCCGCATTGGGTGATCAGAGCGTTATTTACTCGAAAAGT
CAGCAGCGAAATTTTGTAAACGCGCCATCAAAGAATACTCCAGCGGAGAGTATAAGTGATTTGGAAGGCATGACG
ACGTTTGCGCCAACTACTGGAGGTGAAAATAGGGGCAAATCTGCACTTAGGGAATCTCACTCTAATCCTAGCTTC
ACTCCAAAATCTCAAGGATCTCATTTAAATTTAGCGGCGAACACACAGGGAAATCCAATCCCTGGTACTACGGCA
TGGAAGAGAGCAAGATTGTTAGAAAGAAATCGAATTGCAGCTTCGAAATGTAGACAAAGGAAAAAGGTTGCGCAG
CTGCAGCTCCAAAAGGAATTTAACGAAATTAAAGACGAGAATAGAATTTTACTGAAAAAGTTAAATTACTATGAA
AAACTAATCTCTAAATTCAAGAAATTCTCCAAAATTCATTTACGTGAACATGAAAAACTAAATAAAGACTCAGAT
AATAATGTTAATGGCACTAATAGTAGCAACAAAAATGAAAGCATGACTGTGGATTCATTAAAGATCATTGAAGAA
CTTTTAATGATCGATTCAGACGTTACAGAAGTGGATAAAGATACTGGTAAGATCATAGCCATCAAGCACGAGCCA
TACTCTCAACGTTTCGGAAGCGATACTGACGATGACGATATAGATCTCAAGCCCGTAGAAGGTGGTAAGGATCCA
GACAACCAATCATTACCCAATTCTGAAAAGATAAAATAA

>YOR132W 0.81 Cold
ATGACTTCGGCTGTACCTTATGATCCATATGATGATCTGGATAACAATCCATTTGCTGAGCCCCAGGAGGAAGAT
TCTGAACCTGCTGCAACAACCACAGATGGATCATCTTCTATGTCAGAGGAGCGTGTAGGTACTGAGCAAACTGCA
GCTTCCGTTCAGGACAACGGAACTGCAAATAACATTCAAAATGGTCTTGGTGAAGAAGGAAACGCAACACGATCA
AAAACTTCAAACGAACACAATGAAAACCAACAACCATCTCAACCATCAGAACGTGTGTTATACTTCCTGAAAGAAGT

GATGAGAAAAAAAAATACACTCTACTTGCAAAAGTAACAGGATTGGAACGATTTGGATCTGCAACCGGTAAGAAA
GAGAATCCGACTATTATATTTGATTGCTCCACAAATTTGCCAACATTTCGCAAACAACAATACAAGAATGTCAAA
AAATCATACGAAGAATTCCACCAATTGTTCAAATATCTGAATGTTGCCATTCAGGAATCTTTTGTTCCTACTCTT
CCCTCTGCGTATACTACATTCGGAATTAATAGCGAAGAAGATAGGATGAAAGTAACGCGAAATTTCCAACTTTGG
TTTAACAGGCTATCGCAAGACCCCTTAATTATTCGGAATGAAGAAGTGGCCTTTTTCATTGAAAGTGACTTTAAT
ACGTACACACCCATCAACAAGTCCAAATCACTCGCATCTGGGTTGAAAAGAAAAACATTGAAACAATTAGCACCT
CCATATGATGAAATCACAGAATTAGCAGAATTCCGGCCATTAGTCAAGTCTATATATGTTGTTTCTCAGAGCTTG
CAAGAAAAACTGCTAAGAGTTTCCAGAAATCGCAAGATGATGGTTCAAGAAGAAAATGCCTTCGGCCAGGATTTC
GTTAATCTAGATGAGCACAATAAACTTTACAGAAGATACGGTAAAATATTGACTGCTGTGGGTGATATTGATAGC
ATTATAGCAACCATGGACATGGCGACATTGTACGATGGTTTAGAGTGGATTGTCAGAGATGCTTATGCCGTGAAG
GAAGCATTGACCAACAGGCATTTTATCATGCGAAATCTAGTACAGGCACAGCAGAATTCAAAGGCGAAACAGGAA
CAAGCACGCAGGTTCCGGTCAAGAAGAGATATTAACCCTATGAAAATCGATGAAGCACTACGTCAGTTGAAAGCT
GCAGCTAAAAACGAACAAGTTTTGACCCTTAAGCTTCAACGAATCACGTCCAACATGATTATCGAGAGAAAACAG
TGGATTAGCTGGTATGAAGAATGGATAAGAAGCTCGATCAAAGAATTTACACTAAGAAAGATAGAGTATGAAAGA
AAGAAGCTGACGTTACTAGAACGAGTACGTTCTGATATCAGAAAAGCTGATGAAAACGGAGGTTTGTCACGTCTA
GGACGCCATGCTGTCTCAAACAACAACTCCGATACTTCTCAAACCCTTAAAGGGGACAGTTGGACGGGAGAGAGC
AACCGCAAAAGTCAAATCCCCATCAACAAGATCGCTCATACCGAATTTGATGATGAACTATTCACTGAAGACGAT
GGGTACAACTCTCAGGACTCAGACACTACATCACTGAATGCGCGCCATGCTGCTTCACTTTTGGGCATGTCCACT
AAATAA

>YKR101W 0.62 Cold
ATGAGAACAATAATGTCAATAATCACACGTCGTTATAAGCAGCTGAAAAATTATAAGATAATATCAAAAAAAATG
CTACAGATCAACTCCAGGCTTGCAGTTATTGATGGATGGCTGGTAGACACTGTGAAGCGAAAACCAATAAATTTT
CGGAGTCCTGAAGTAAGATTACTGTTACCCAATGACGATGACTACAAAAAGCTATCACAACAAAACTTGGTGGAC
TGGACGCGATTAAAGAAGGATTCTAATTCGGTGCTCGTTGGAGTGAAATCTATGGAACTATTTAAACACATAAAG
CTAGTTTTGCGAGAGTTTTTCTTGTTAGAAGATGGAAGAATAATTCTGAAGAGGATCAGAAGCAAATTACGTTAC
AAAGTTGTCAAGAAATTAACTTGTAAATGCTGCAGGTTATATTTACCAAAATGGGGCACCGTGTACATACATCCG
ATGCTAAAAGATAAAGAAAAGCCCTTAGCGGGGGTATGTGAATTTTCATTGGATGTAAACCCTGATCGGGAGTAT
CCCCTTATTGAGATCAATGTTAGTCATCAATACATTATAATTGAGGGCTTCCTTCTATACTTAAATGAAAGGAGG
CTCTATAGGTGGAACGATAACAATTTGAGGAGTCAGGTTGGCTTAACAAAATGGGCCCATTTAAGAAAAACTTAC
AATCCGGTAAGCCTTGACATACTTTATAGTTTGAATTCAAATTTTTATTTTGTAAAGGATGATCTGCTATTTCAA
TTATTAGGAAAGAGGGTATTTGTTAAATTTTGTAAAGTAATGGAAAATGGAAAATGCGGTAAGGCTCCACTGTGG
TATCGTGTGAAGAGAACGACAACTGCCAAAGCAACACATATTGCATATGCCATATCAAATTCAACAGCCCCAGAT
TCATTCAAAAGTAAAAATAACGATTATAGGTTTATTGTCAGGGAAAAGCCAATTGTGGAGAATACTATCTCCAAC
CTGGATTATTCAGACATAAAAAAACAGCAGTTTACTGAAGCAGAGGTTGTAAAAAGAAAGATCTCAGCAGATATT
TCTCAAATAGAGAATGTGCATACGCAATTTAATAGTCAAAAGGAAAAAAATAATATCAGGGTGAATAAGGTTTCT
AGCGAGGTCCTAGATCAAATTTCGAAATTTCCTGTGTCCAGAGTCACCTTACTGTTGATGTCTGCTGGTCAAGAT
AAAAATTATATTGAACTTGTTGAAGAATTGGCAAGAAGGTTGGAAAAAAATATGCATAGAAAAAACCACACAATCT
TTAGAAGAGATAAGGGATACTTTTCAGGCGAATCCTGAGATGCAGGCTAGCTTTGATAAGGAATATTACCAGAGC
ATTGAAGAATATAAAATTACACTCGAACTTATTAAGGAAGACCTTTTGATTACTCTGATAAAACAAATGGAAAAT
ATGTGGGCAGCTGAAAAAAAGTTTAGTACAGAGGAGGAGTATGTTTCGCCGAGGTTTTTAGTAGCAGATGGATTT
CTAATCGACCTAGCAGAGGAAAAACCGATTAACCCAAAGGATCCGCGCTTACTGACACTGCTAAAAGATCATCAG
CGTGCCATGATTGACCAAATGAATTTAGTTAAGTGGAATGACTTCAAAAAATATCAAGATCCTATCCCGCTGAAA
GCCAAAACCTTATTTAAATTTTGTAAACAAATAAAGAAAAAATTCCTACGAGGTGCGGACTTCAAGTTACATACA
TTACCTACAGAAGCAAATTTAAAGTATGAGCCGGAGCGGATGACAGTTTTGTGTTCCTGTGTCCCTATTCTTTTG
GATGACCAAACTGTCCAATATCTGTATGATGACAGCATTATTCCTGAATTTGAAGCAACATCTTCATATGCAACA
AAGCAGTCAAAGTGTGGGCGGAAAATGTCTTTGCAAATGGAGCCTGACCTCCTTTTTCAAGAGGCCATTAGACGG
ATGCGACATTTAACTGCTTATGACGTTTTGAGAAGAAACTATATTGCGGCATTTGAGGAGCTATATATGGGAAAC
TGTAACGATTAA

>YBR017C 0.81 Cold
ATGGCATCGACATGGAAGCCCGCCGAAGACTATGTGTTGCAACTAGCAACTCTTTTACAGAACTGTATGTCACCA
AATCCAGAGATTCGTAATAACGCAATGGAAGCTATGGAGAACTTCCAGCTGCAACCTGAATTTCTCAATTATTTG
TGTTATATTTTAATTGAAGGCGAATCTGATGATGTATTGAAGCAACACTACTCCCTACAGGATCTTCAGAACAAT
AGAGCTACCGCCGGTATGCTGTTGAAAAATTCAATGCTAGGGGGAAACAATTTAATTAAGAGCAATAGCCACGAC
TTAGGATACGTCAAATCAAACATTATACATGGCCTTTATAATTCGAACAATAATCTCGTTTCGAACGTGACAGGT
ATCGTTATTACTACTTTATTTTCCACTTACTATAGGCAGCATAGAGATGATCCAACTGGTCTTCAAATGCTTTAC
CAGTTGCTAGAGCTAACCTCAAATGGAAATGAGCCAAGTATTAAGGCTTTATCTAAGATCATGGAAGACAGCGCT
CAATTTTTCCAATTGGAATGGTCGGGAAATACGAAGCCTATGGAAGCCTTATTGGATAGTTTTTTTAGGTTTATT
TCGAATCCAAATTTCTCACCTGTGATTCGCTCAGAATCGGTGAAATGTATAAATACAGTGATCCCGCTACAAACA
CAAAGTTTTATTGTGAGATTAGATAAATTCTTAGAAATTATTTTTCAGTTGGCACAAAACGACGAAAACGACCTA
GTTAGGGCACAGATTTGCATTAGTTTTAGTTTCTTATTGGAATTCAGACCAGATAAGCTGGTTTCCCATTTAGAT
GGTATTGTACAATTCATGTTGCATTTGATCACCACTGTAAATGAGGAAAAAGTGGCTATTGAAGCCTGCGAGTTT
TTGCACGCCTTTGCAACGAGCCCAAATATTCCTGAACATATCTTACAACCATATGTTAAGGATATCGTGCCAATA
TTATTATCGAAAATGGTCTATAACGAAGAATCCATCGTTCTCCTGGAAGCTTCTAATGATGATGATGCATTCTTG
GAGGATAAAGATGAGGACATCAAGCCCATTGCACCCCGTATTGTGAAAAAGAAAGAGGCAGGAAATGGAGAGGAT
GCAGATGACAACGAAGATGATGATGATGATGATGATGATGATGAAGATGGCGATGTTGATACGCAATGGAATTTGAGA

AAATGTTCCGCGGCAACGCTAGATGTAATGACGAATATTTTACCTCATCAAGTGATGGATATAGCGTTCCCATTT
TTAAGAGAACATTTGGGTTCTGATAGGTGGTTTATTAGAGAAGCTACTATATTAGCACTGGGGGCCATGGCAGAA
GGTGGAATGAAGTATTTTAATGATGGCTTACCAGCACTAATACCATTTTTAGTGGAACAATTGAACGATAAGTGG
GCACCAGTGAGGAAAATGACATGTTGGACATTAAGTAGGTTTTCACCATGGATATTACAAGACCATACTGAGTTT
TTAATTCCAGTCTTAGAACCTATAATAAACACATTAATGGACAAGAAAAAGGATGTTCAAGAGGCGGCTATTAGT
AGTGTAGCAGTATTTATTGAAAACGCCGACTCCGAATTGGTTGAAACTTTATTTTATAGTCAATTATTGACGAGT
TTTGATAAATGTTTGAAATATTACAAGAAAAAGAATTTAATTATATTATATGATGCCATCGGCAGGTTTGCTGAA
AAATGTGCATTAGACGAGACAGCGATGCAAATAATTTTGCCGCCCTTAATTGAAAAATGGGCTTTGCTGTCAGAC
AGTGACAAGGAGCTGTGGCCACTTTTAGAATGTCTTTCCTGCGTGGCATCATCACTGGGGGAAAGATTCATGCCT
ATGGCACCAGAAGTGTACAACAGAGCCTTTAGAATTCTATGTCATTGTGTCGAATTGGAAGCCAAATCACATCAA
GACCCGACAATAGTAGTGCCTGAGAAGGACTTCATCATCACCTCATTAGATTTGATTGATGGATTGGTACAAGGT
CTTGGCGCTCACTCGCAGGATCTATTGTTCCCTCAAGGGACGAAGGATTTAACGATATTGAAAATCATGCTAGAA
TGTTTGCAGGACCCTGTCCATGAAGTAAGACAAAGCTGCTTTGCCCTGTTGGGAGATATTGTATATTTTTTCAAT
TCGGAACTGGTAATTGGTAATTTGGAGGATTTCTTGAAGTTGATTGGTACGGAAATAATGCATAACGACGATAGT
GATGGTACTCCTGCTGTGATAAATGCGATATGGGCGCTTGGTTTGATAAGCGAACGTATCGATTTGAATACTTAT
ATCATTGATATGTCTAGAATCATTCTAGATTTATTTACCACCAACACACAAATCGTAGACAGCTCTGTGATGGAG
AACTTGTCTGTGACCATCGGAAAAATGGGGCTAACACACCCTGAAGTTTTCAGTTCTGGCGCATTTGCCAATGAT
TCCAACTGGAATAAATGGTGTTTGTCCGTTAACGCATTGGACGATGTAGAGGAAAAGAGTAGCGCGTACATGGGT
TTCCTGAAAATTATCAATTTGACCAGCACGGAGGTCACAATGAGTAATGATACCATTCATAAAATTGTTACGGGC
CTTTCAAGCAATGTAGAGGCGAATGTTTTTGCGCAAGAGATCTACACCTTTTTGATGAACCATTCTGCCCAAATT
TCTGCAATAAATTTCACGCCCGATGAAATCTCCTTCTTACAACAGTTCACCAGCTAA

>YBR226C 0.79 Cold
ATGAATGGTATAGAATATATAAATATAGATGTAATAAATGTAGTAAATGAACAAATAATCTTCCTACAAAGTACT
ATGCCATGGGAAGTAAAAAAAATTAATAAATATTGGCTAAAGTGTTTCAAATTGGTTCTACGATACTGGGAGACT
ACTCCTTATCATAATGCTTTTTGTCCAGTAGTAAAAATTGAAGTAATTCTTGAACAATTTAAAAAAAAAATTGGAA
CGGCGGCACAATTATTTGGTATATGGCAGCTTTGGCTTTTGCGCCACCACATACAAAGTTCTAAAGCAGCCAGCG
TTGTCATCGATTTTTCCTATGTGTTCTTCCACATATCTTTTGAAAAGAGTGGTGGGGTCGGTATCCTTTGGAGGA
CAACTACTATCTGGTAGTTGCCTCCTTAACATGTAG

>YLR223C 0.78 Cold
ATGGCAGGCAAAAAAAGTCCTCGAAAAAGTACGATCAATCATAGTACACATTCTGGTAAACTGCCAGCAAATATT
AAAAGGCTCATAAAAAAGGGCGAATCCGATACAAAGTCCAGGCAATCACCACCCACACTGAGTACAACGAGGCCA
AGAAGATTTAGCCTTATATACTCTTCTGAGTCATCCTTGAGTGACGTATCTGATTCTGATAAAAACAAAAGTACA
AACCCACATAAAATTAAAAGAAAAGCAAAGAACATTTCAAATAATTCCCAAGGAAAAAAAAGTAAACTTATACAA
AGGCAAATAGACAATGACGACGAGGGCACAGAGTCATCCGATTACCAAGCTGTAACAGATGGCGAAGAGAGTGAA
AATGAAGAAGAAGAGAGTGAAGAAGAAGAAGAAGATGATGACGAAGACGACGACGACGATGATGATGACGGAAGT
GATAGTGATAGTGATAGCGAAACAAGTTCTGATGACGAGAATATAGATTTTGTCAAACTGACGGCCCAAAGAAAG
AAAAGAGCCATGAAAGCTTTATCTGCCATGAATACAAATAGCAATACACTCTATTCCTCTCGTGAGAACAGTAAC
AAAAATAAATCAGTTAAACTATCCCCTAAGAAAGAAAATGAAGAAGAGCAGAAAGAAGAAAAAGAAAAAGAGAAA
GAAGAGCAACAAAAACAACAAGAATCAAACAAAAAAGAAGTAAACGGTTCAGGCACTACTACTACACAACAGGCG
CTATCGTTTAAATTCAAAAAAGAGGACGACGGCATTAGTTTTGGTAATGGTAATGAAGGCTATAACGAGGATATA
GGTGAAGAAGTCTTGGATTTAAAAAACAAAGAGAACAATGGTAATGAAGAAGATAAACTGGATTCTAAGGTGATG
TTAGGTAACAACGATGAGTTACGATTTCCCAATATTTCAGAGTCAGATGAATCTGAATATGATATTGACCAGGAT
GCGTACTTTGACGTGATTAACAATGAAGATTCTCATGGAGAAATTGGTACAGATCTTGAAACGGGGGAAGACGAT
CTTCCCATATTGGAAGAAGAAGAACAAAACATTGTTTCTGAGCTACAAAATGACGACGAACTCTCATTCGATGGT
AGTATACACGAAGAAGGGTCTGATCCTGTAGAAGATGCTGAAAATAAATTTTTGCAAAATGAATACAATCAAGAA
AACGGATATGATGAAGAAGATGACGAAGAAGATGAAATAATGTCTGATTTTGATATGCCGTTTTATGAAGATCCT
AAATTTGCAAATCTTTATTATTATGGCGATGGTTCAGAGCCAAAGCTATCCTTGAGTACATCTTTACCGTTAATG
CTAAATGATGAAAAACTATCTAAACTAAAAAAGAAAGAGGCCAAAAAACGGGAACAGGAAGAAAGGAAACAAAGA
CGAAAGCTCTATAAAAAGACGCAAAAACCTAGTACGAGAACAACCTCCAATGTGGACAATGATGAGTATATTTTC
AATGTTTTTTTCAATCAGATGATGAAAATAGTGGCCATAAGAGCAAGAAAGGCAGGCATAAATCGGGCAAAAGT
CATATTGAACATAAGAATAAAGGCTCGAATTTGATAAAATCCAATGATGATCTGGAACCATCCACTCATAGTACG
GTCCTGAATTCCGGGAAATATGATTCTTCTGACGATGAATATGATAACATTTTGTTGGATGTTGCCCATATGCCT
TCAGATGATGAATGCAGTGAATCTGAAACGTCCCACGATGCTGACACGGATGAAGAATTGAGGGCACTAGATTCA
GATAGCTTAGACATTGGCACAGAACTGGACGACGATTACGAAGACGACGACGATGATTCCAGCGTGACAAATGTG
TTCATAGACATCGATGATTTAGATCCAGACTCTTTTTACTTTCATTACGACAGCGATGGATCTTCCTCTTTGATA
AGTTCTAACTCAGACAAAGAAAATTCTGATGGATCCAAAGATTGCAAACATGATCTCTTAGAGACTGTTGTGTAC
GTTGATGACGAATCCACAGATGAAGATGATAACCTACCGCCCCCAAGTTCAAGGTCAAAAAACATTGGCTCAAAA
GCAAAGGAAATCGTAAGTTCAAATGTTGTTGGATTACGTCCACCAAAATTGGGTACTTGGGAGACGGACAACAAA
CCTTTTAGTATTATTGATGGTCTGTCTACTAAATCATTATACGCCTTAATCCAAGAACATCAACAGCTTCGCGAG
CAACATCAAAGGGCTCAAACCCCAGATGTTAAAAGAGAGGGAAGCTCTAATGGCAATAACGGTGACGAATTGACA
CTCAATGAGCTGCTAAACATGAGTGAATTGGAGGATGATTCACCATCCCACACAGACGATATGGAGAACAATTAC
AATGATGCAATTAATAGCAAAAGCACAAATGGCCATGCTGCAGATTGGTATGAAGTTCCTAAGGTTCCATTATCT
GCATTTAGAAATAAGGGTATTAATGCCTATGAAGAAGATGAGTACATGATACCAGCAAATTCTAACAGAAAAGTT
CCCATTGGCTATATTGGTAATGAAAGAACAAGAAAGAAGATTGATAAGATGAAAGAGCTACAACGGAAAAAAACT
GAAAAAAAAAGGCAGTTAAAGAAAAAAAAGAAGCTTCTTAAAATAAGAAAGCAAAGACAAAAGGCAATAAAGGAG

CAAGAAACTATGAATTTACAATTGGGAATCAATGGCCATGAGATCATCGGTAACAATAACAGCCATAGCGACATA
AATACCGGTACCGATTTTACAACCAATGAAAATACCCCTATGAATGAACTTCCCTCTCACGCACCTGAAGATGCG
TCATTAATACCTCATAATTCTGATCTTGCCGTGGACAGCAATACAAGGAAAAATTCAACAAAAAGTGTTGGTTTA
GATGAAATTCATGAGATTTTGGGCAAAGATGAAAATGACTTACTGTCTGTAGGTGATATTAACGGTTATGATGCA
CAAGAAGGTCATGTGATCGAAGATACTGACGCCGATATCCTAGCATCGTTAACCGCTCCTGTGCAATTCGACAAT
ACATTAAGCCATGAAAATAGTAATTCCATGTGGAGAAGAAGGCAAAGTATGGTGGAAGCAGCGGCTGAAAATCTT
CGTTTCACTAAAAATGGTTTATTTAGTGAGAGTGCATTGGCAGATATCGAAGGAATTATGGGCAATGATGTTAAC
CATTCATTCGAATTCAATGACGTCTTACAATGA

>YEL010W 0.79 Cold
ATGGGGAAATATTCATCATGTAGAATTTCAAATATTTTCATTGTAATTTCTAAAATTAATGAGGAAAATTCGATA
TATCTGGTATCACTTTATTATTCCTTCAGCACGAAATGTCGAGCGATCTCGATGCAGGAACCAGGTATAAGTAGC
GATAGTAAATTTTTTTCTCTCTTTTTAATAATCCGGAAAGTCTCAGTTGCGAGTGATTGCAGACAGTTGTATGAA
TGTAAAAAAAGTAATGAAAACATTTGGGAGTATTTCAAACGGAGGTTAGAGACGAGGCTTTCGAGCTTTTCTATT
ATTTTAAGTGCTGTGTTTCCGGACGTGCTCTTCACTTTCTTATTTTCTTGA

>YLR019W 0.82 Cold
ATGGGATTTATAGCAAATATACTGTGCTGTTCTTCAGATACTTCCAAAACACATCGTCAACGCCAACCACCAGAA
ACTAATCACAACCGCAACCGCAACCGTAAGCACAGTTCAAACAAAGCTCAAACTCAGGGTCGTAAGCAGAAGGCT
ACTCCAAACGGCGACAAGATGCAATATTCCACACCGGAGATCCTTTTGTCTAGTTCCGATTCAGGCAGCAATGCG
GGTTCCAAGACAATGCAGGAGAATGGGAATAGCGGCAATGGAAAACTAGCACCTTTATCAAGAGATCACTCCAAC
AACTCGTACGATGAGGAGAAAGAATATGAAGATTATAATGAAGGAGACGTAGAAATGACGGAGGTTAACAATGCC
GGAGAAGAAGAAGAAGAAGATGACGAAGCAAAAGAAAAGCAAGACCATGTCGTTCATGAATATAACGTTGACGCA
GATAGAAATTCCAGTATTAATGACGAGGCGCCACCACAACAGGGTCTGTATCAGGTGGGTCAAGAAGATATGAAT
CCACAATATGTCGCCAGCAGTCCTGATAATGATTTAAACTTGATACCTACAACGGAGGAGGACTTCTCTGACCTG
ACGCATTTGCAACCAGACCAGTACCATGCCCCCGGATACGACACTCTGCTACCACCCAAGTTGCAAGAATTTCAA
CAGAAAAAAATGTCTGATATTGGATCTAGACGAAACTTTGGTACATTCTTCCTTTAAATATATGCATTCAGCTGAT
TTCGTTTTGCCCGTCGAAATCGATGATCAAGTTCACAATGTCTATGTTATTAAGAGGCCAGGCGTCGATGAGTTC
TTGAACAGGGTGAGTCAACTATACGAAGTTGTTGTGTTCACGGCAAGTGTTTCGAGGTATGCAAACCCCCTTTTA
GATACGTTGGACCCTAATGGAACCATCCATCATCGATTATTCAGAGAAGCTTGTTATAACTACGAAGGTAACTAC
ATCAAGAACTTATCGCAGATCGGAAGACCATTGTCCGAAACAATCATCTTAGATAATTCGCCGGCCTCTTACATC
TTTCATCCGCAACATGCTGTTCCGATATCTTCTTGGTTCTCTGATACTCATGATAATGAGTTACTAGATATTATC
CCACTTTTGGAGGACCTTTCATCAGGAAACGTGTTGGATGTGGGGAGCGTGTTGGATGTGACGATATAG

>YGR005C 0.72 Cold
ATGAGCAGTGGTTCAGCAGGGGCACCAGCACTTTCTAATAATTCCACGAACTCTGTCGCGAAGGAGAAATCAGGT
AACATTTCCGGTGATGAGTATCTTTCGCAAGAGGAGGAAGTTTTTGACGGTAATGATATTGAGAATAATGAAACC
AAAGTTTATGAAGAATCCTTAGATTTGGACTTGGAACGTAGTAATAGACAAGTCTGGTTAGTTAGATTGCCCATG
TTTCTAGCAGAGAAATGGAGGGACAGAAACAACTTGCATGGCCAAGAACTGGGTAAAATTAGGATAAACAAGGAT
GGGAGTAAAATCACACTTCTATTGAATGAAAATGATAACGATTCTATACCGCACGAATATGATTTAGAACTTACA
AAGAAAGTAGTAGAAAATGAATATGTTTTCACAGAACAAAATTTAAAGAAATATCAACAACGTAAAAAGGAGTTG
GAAGCAGATCCTGAAAAGCAAAGGCAAGCTTACCTGAAGAAGCAAGAACGTGAAGAGGAACTTAAGAAGAAGCAG
CAGCAGCAAAAACGTAGAAATAATAGAAAAAAGTTTAATCACAGAGTTATGACAGATAGGGATGGTAGAGATAGA
TATATACCATATGTGAAGACGATTCCCAAAAAAACCGCCATTGTGGGTACAGTTTGCCACGAATGTCAGGTTATG
CCATCAATGAATGATCCTAATTATCACAAGATTGTTGAACAGAGAAGAAATATTGTCAAGCTTAATAATAAGGAA
AGGATCACAACTTTGGATGAAACCGTTGGTGTTACGATGAGCCACACAGGTATGTCCATGAGGTCAGACAACTCG
AATTTCTTGAAAGTGGGGCGTGAGAAGGCCAAGAGCAATATTAAATCTATTCGTATGCCAAAGAAGGAAATCTTG
GATTACTTGTTCAAGTTATTTGATGAGTATGACTACTGGTCCTTGAAGGGGTTGAAGGAACGTACTAGGCAACCT
GAAGCACATTTAAAGGAGTGTTTGGATAAAGTTGCCACTCTAGTGAAGAAGGGCCCATATGCATTCAAATACACT
TTAAGGCCAGAGTATAAAAAGTTGAAAGAGGAGGAGAGAAAGGCAACCTTAGGTGAACTAGCTGATGAGCAAACA
GGTTCCGCTGGAGACAATGCGCAAGGAGACGCGGAGGCTGACTTGGAAGATGAAATAGAAATGGAAGATGTCGTT
TAG

>YNL166C 0.81 Cold
ATGGGCTTGGACCAGGACAAGATAAAGAAGAGGCTTTCTCAAATAGAGATAGATATCAACCAGATGAACCAGATG
ATTGACGAAAATTTACAATTAGTTGAACCTGCGGAGGACGAAGCTGTCGAAGATAATGTCAAGGATACGGGTGTG
GTCGACGCTGTAAAGGTGGCAGAAACGGCTCTTTTTAGTGGCAATGATGGAGCGGATAGTAACCCAGGAGATTCA
GCTCAGGTTGAAGAGCACAAAACCGCACAGGTCCATATTCCCACTGAGAATGAAGCGAATAAGAGTACGGATGAC
CCATCCCAGCTATCGGTTACTCAACCTTTTATAGCGAAAGAGCAGATAACTCACACCGCCATTGCTATTGGTGAC
TCCTATAATTCATTTGTCGCAAATTCCGCTGGAAATGAAAAGGCAAAGGACTCCTGCACTGAGAACAAAGAAGAT
GGTACAGTCAATATAGATCAAAACAGGGGTGAAGCTGATGTTGAAATTATCGAAAATAATGATGATGAATGGGAG
GATGAAAAATCGGATGTTGAAGAGGGGCGAGTAGATAAGGGAACAGAGGAAAACAGTGAGATTGAATCTTTCAAA
TCACCCATGCCCCAGAATAACACCTTGGGCGGTGAGAACAAGTTAGACGCTGAACTTGTATTAGACAAATTTTCT
TCCGCTAATAAGGATCTAGATATACAACCGCAAACCATAGTTGTAGGAGGTGACAATGAATACAACCATGAGAGC
AGCCGTTTAGCTGATCAAACACCCCATGACGATAATTCAGAGAATTGTCCCAATCGCTCTGGAGGAAGCACTCCC
TTAGATTCTCAAACTAAAATTTTTATCCCTAAAAAGAATTCTAAAGAAGATGGTACCAATATAAATCACTTTAAT
TCAGATGGTGATGGACAGAAAAAAATGGCGAATTTCGAGACACGACGCCCTACAAATCCGTTCAGAGTTATCTCG

GTGAGCAGCAATTCCAATTCAAGAAACGGAAGTCGTAAATCTTCACTAAATAAGTATGATTCGCCAGTCTCCTCT
CCTATCACATCAGCGTCTGAGCTGGGCAGTATTGCCAAGCTGGAAAAAAGACATGATTATTTATCCATGAAATGC
ATTAAGTTACAAAAGGAAATTGACTATCTAAACAAGATGAATGCACAGGGTTCTTTATCTATGGAAGACGGAAAG
AGACTACATAGAGCCGTAGTGAAGCTTCAAGAATATCTTGACAAGAAAACGAAGGAGAAATATGAGGTCGGAGTG
TTATTAAGTAGACATCTGAGAAAGCAAATTGATCGTGGCGAAAATGGCCAATTTTGGATTGGAACTAAATGA

>YDL193W 0.78 Cold
ATGCCCACGATGATCAAAAAGGATGATAAAGCAATGGAGCCCCCTAATGAAAAACCGCATAGAAAGATCGAAAGA
GATGATGTTCCAGAATCTTCCAATCACATCCCACCTCCAGAATCTGGTGTTTTAAAGGGCGGTAAAGTTAATTCA
AAAACCAGAGCTTTAAAGGCCGTTACAAGTATCATTGCAGACGCCGATGAGAACCCTCAGAAGAAAGTGAACAAT
GAGACGAATGGAGTCCAAAAGCAAAAGACAGAAGATTTGAGTAAAAGAATAGGTAAATTTGAATACCTTTTTTAC
AAGTTTTTACTTGTGTTGTTATACATCTGCTTCGGGTTGTTTCGGTACGGTCAATACCAATATAATAAAATGAAA
CTAAGAATATTCAGTATCATCTACAACCATGCATATACACCACAGTTGATTAGACAGGACGTTATTCCTCTGAAA
AAAATTCCTAAAAGGTTGGCCGCTATCTTGGAAGTCAAGCCAGTTGGCGACGTTGGCGGCGGTGTGACAGGTTTA
TTAAATGACGCGAGTGAAATTGTTTGCTGGACTGTTTCAGCTGGTATAAAACATTTGATGTTGTACGATTACGAT
GGAATATTACAAAGAAATGTTCCAGAGCTGAGAATGGAAATTCATTCCAACCTGGCTAAATATTTTGGGCCAGCT
CATGTTCCAAACTACGCTGTTAAAATACCTCATTCTAACAAGATATTCTACAATCTAGACGGAATTGAAACCGAG
ACTGATGTAGGCAATGAGATAGAAGCTAACCAAGAAAAGGACAAAATTGCTATTGAAATTTCTTTATTGTCTAAC
AGAGATGGTAGAGAAACGATTGTCGATCTGACCAAAACTATGGCTGAGTTATGTGCGGTTAACGAATTGAGCGTT
TCTGACATCACAATGGATTTAGTTGATTCAGAACTGAAACAACTAGTTGGACCCGAACCAGATTTACTGTTATAC
TTCGGGCCTTCGTTGGATTTACAAGGGTTCCCACCTTGGCATATTAGATTAACCGAATTTTATTGGGAAAAAGAT
AACAACGAAGTCATATATTCGGTTTTCATCCGCGGCCTAAGACAGTACGCAGGATGTAAAGTGAATGTTGGTAAA
TGA

>YOR304C-A 0.78 Cold
ATGAGTACTGAAAAACTGGAAGCTTCGGAGGAACCACAGGCTCCCTTGGCCAATACATCTGAGACTAACTCAATA
AAGGGAGATACAGAAAATATTGTAACCGTGTTTGACCTGGCAAATGAAATAGAAAAATCACTAAAGGATGTTCAG
AGGCAAATGAAGGAAAATGATGATGAGTTTTCACGCAGTATACAAGCAATTGAAGACAAGCTTAACAAAATGAGT
CGGTGA

>YDR280W 0.8Cold
ATGGCCAAAGACATTGAGATATCCGCATCCGAGTCAAAATTTATCTTAGAAGCACTGAGACAGAATTATAGGTTG
GACGGCCGCTCCTTTGATCAATTTCGTGACGTGGAAATAACGTTTGGTAAAGAATTTGGTGATGTTAGCGTAAAA
ATGGGCAACACCAAAGTTCACTGCAGGATTAGTTGTCAAATAGCACAACCATACGAAGACAGGCCATTTGAAGGA
TTATTTGTAATATCTACAGAAATATCTCCTATGGCTGGTTCTCAATTTGAAAATGGAAATATCACGGGGGAAGAT
GAAGTTTTATGTTCAAGAATAATTGAAAAATCTGTTAGGCGGTCGGGCGCATTGGACGTGGAAGGACTATGTATC
GTTGCTGGCAGTAAATGCTGGGCTGTGAGAGCTGATGTACATTTCCTGGATTGTGATGGTGGATTTATTGACGCT
TCGTGTATTGCCGTCATGGCAGGATTGATGCATTTTAAGAAACCAGATATAACTGTCCATGGCGAGCAGATCATT
GTTCATCCTGTTAATGAAAGAGAGCCAGTTCCACTAGGTATACTGCATATTCCAATATGTGTAACTTTTTCTTTC
TTCAATCCTCAAGACACCGAGGAAAACATAAAAGGTGAGACTAACTCAGAAATTTCCATCATTGATGCAACATTG
AAAGAGGAACTACTGCGAGATGGTGTATTGACGGTTACGCTAAACAAAAACCGTGAAGTGGTACAAGTTTCCAAA
GCGGGTGGTTTACCAATGGATGCGTTGACGCTAATGAAATGTTGTCACGAGGCTTACAGCATTATTGAGAAAATA
ACTGATCAAATACTGCAGCTTTTAAAGGAGGATTCAGAAAAGAGAAACAAGTATGCAGCCATGCTTACATCTGAA
AATGCCCGCGAGATCTAA

>YNL335W 0.8Cold
ATGTCACAGTACGGATTTGTAAGAGTTCCTAGAGAGGTAGAAAAGGCCATTCCAGTGGTGAATGCACCTAGACCA
CGGGCCGTTGTTCCGCCTCCAAACAGTGAAACTGCTAGGCTTGTTCGGGAATATGCCGCTAAAGAATTGACTGCC
CCCGTTCTAAACCACTCTTTGCGTGTTTTTCAATATAGTGTAGCTATCATAAGAGACCAATTTCCAGCATGGGAC
TTGGATCAGGAAGTTTTGTACGTCACCTGCTTACTTCATGATATTGCAACAACAGATAAGAATATGAGAGCCACG
AAGATGTCATTTGAGTATTATGGTGGCATACTTTCAAGGGAGCTTGTATTTAATGCGACAGGTGGAAATCAGGAC
TACGCAGATGCAGTAACTGAGGCCATCATTCGTCACCAGGATTTGACTGGGACTGGCTACATTACCACCTTGGGG
CTCATTCTGCAGATTGCTACTACGCTTGACAATGTCGGATCCAATACCGATCTGATTCATATCGATACAGTTAGT
GCCATTAACGAGCAATTTCCAAGACTGCACTGGTTATCATGTTTTGCTACGGTGGTGGACACTGAAAACTCGAGA
AAACCGTGGGGCCACACCAGTTCTTTGGGTGATGATTTTTCAAAGAAAGTCATATGCAATACATTTGGGTATAAC
TAA

>YNR072W 0.67 Cold
ATGCAATCATCCACTGAAAGTGATAGAGATATTCAGGATGGTCCTGATGCTGATATTCACGTCGCACCACCCGTG
GAAAAAGAGTGGTCAGATGGATTTGATGACAACGAAGTCATAAACGGGGATAACGTTGAGCCACCAAAAAGAGGG
CTCATAGGTTATCTTGTCATTTACTTATTGTGTTATCCAATATCCTTCGGGGGTTTCCTGCCTGGTTGGGATAGT
GGTATCACAGCAGGTTTCATTAACATGGACAACTTTAAAATGAACTTCGGTTCTTACAAGCATAGCACTGGTGAA
TATTATTTGAGCAACGTGCGTATGGGTCTTCTTGTGGCTATGTTCAGTATTGGATGTGCCATAGGTGGTCTTATT
TTTGCCCGTCTTGCTGATACTTTAGGTAGAAGGCTGGCAATTGTAATCGTGGTGTTGGTATATATGGTTGGTGCA
ATTATTCAGATCAGTTCGAATCACAAATGGTACCAATACTTCGTTGGTAAGATTATCTATGGTCTTGGTGCTGGT
GGCTGTTCGGTGTTGTGTCCAATGCTTTTGTCTGAAATAGCTCCTACAGATTTGAGAGGTGGACTTGTCTCATTG
TACCAACTTAACATGACCTTCGGTATTTTCTTGGGTTATTGTAGCGTTTATGGTACGAGAAAATACGATAACACT

GCACAATGGAGAGTCCCACTTGGGCTTTGCTTTTTATGGACTTTGATTATCATCATTGGTATGTTATTGGTTCCA
GAGTCCCCAAGATATCTTATTGAATGTGAGAGACACGAAGAGGCCCGTGCTTCCATTGCCAAAATCAACAAGGTT
TCACCAGAGGATCCATGGGTACTCAAACAGGCTGATGAAATCAACGCCGGTGTCCTTGCCCAAAGAGAACTAGGA
GAAGCTTCATGGAAAGAACTTTTCTCCGTCAAAACTAAAGTCCTTCAACGTTTGATCACAGGTATTCTTGTGCAA
ACCTTTTTGCAACTTACTGGTGAAAACTACTTTTTCTTCTACGGAACTACCATTTTCAAATCAGTCGGTCTTACT
GATGGGTTTGAGACGTCGATCGTTCTAGGTACAGTGAACTTCTTTTCCACTATTATTGCTGTTATGGTCGTAGAC
AAAATTGGCCGTCGTAAATGTCTTTTATTTGGTGCAGCTGGGATGATGGCTTGTATGGTCATATTTGCAAGTATC
GGGGTGAAATGTCTTTACCCTCATGGCCAGGATGGTCCATCCTCAAAAGGTGCAGGTAATGCCATGATTGTGTTC
ACTTGCTTCTATATATTTTGCTTTGCAACGACATGGGCTCCTGTTGCTTATATTGTGGTTGCCGAGTCGTTCCCT
TCGAAGGTCAAATCTAGGGCCATGTCGATTTCGACTGCATGCAACTGGTTGTGGCAATTTTTGATCGGGTTTTTC
ACACCATTCATTACTGGGTCTATCCACTTCTATTATGGTTATGTGTTCGTAGGTTGTTTGGTTGCAATGTTTCTG
TACGTTTTCTTCTTTTTACCAGAAACGATTGGTCTATCTTTGGAGGAAATCCAATTACTATATGAAGAAGGTATA
AAACCATGGAAATCTGCATCTTGGGTCCCACCTTCTAGGAGAGGTATTCCTTCCGAAGAAAGTAAGACCGAGAAG
AAGGATTGGAAGAAATTTTTGAAGTTCTCAAAGGGTTCTGATTGA

>YJR126C 0.81   Cold
ATGAGAATGATACAGAGAGAGAGAAAGAGAGAGAAAGAGGAAGGTCAATTAAAGGAGAGAACTGTTGTTAATATG
GCAGACCCTGATGACAATGAGGCCGAAGCCACTGGATTACAACAATATAGTGGCGAGACCACTCGCGATGACAAT
GAAGAAAGCATGAATGATTCTTTCACTTTAACATCCAGGAATAGAGGCAGAAGTAATACAATATCTAGTATTGTT
AGTGGTTATGAAATAATGAAAGAACATATGGACAAGGAAAAGTTTATGTACTTGATTCTAGCGAGTCTCCTTTTG
TACATGGGATTTGTTGCCGCATTTGCTCCCAGGACGTCTTTATCAAGAGACTTTCGGCGGTTTCACTCTTCCAGA
TTGACGAATGCAGAGGTTTATAGGATATACTTGAACTCCTTGCAACAGGAAAATAGAGCGAAAGAACATGTATAC
AAGTATGCTGGGTACATGAGCAACGGAGCAAGTGATTCGTCAACGTTTAAATATACCTTGGACGAGTTTCTAGAT
ATGGGGTACAAACCCAAAGTTGAAAAATATTACCCATGGATAGGTGAACCAGTAGACACTAACGTAGCTCCTTTA
GAAAATGGTAAAGTGGTCTACGAAGCAAGCATGATCGAGGATAGAGTTAAAGGTGATCCTGCTTCTCACGCTAGG
AAAAGGCAAAAGGGTTTCCATCAATATTCAAAAAATGGAAGTGTAACTGCTCGATACGTGTTTTGCAATTATGGT
AGCATCAGTGATTACAAGCTACTTTTGAAGAAAAACATTGATATTGAAGATAAAATCCACATCGTACGATCGGGT
AAAATATTACCTGGATTAAAGGTAAAGAATGCAGAACTTTATGGCGCTTCCAGTGTCATTATATATACAGACCCA
TTTGACGATGGTAAAGTTACTGAGGAAAATGGGTTTTTACACTATCCTTATGGACCAGCAAGAAACCCAAGTTAT
ATTAGGAGAGATTCTGTAAACTATTTCAGTGACACTCCAGGAGATCCGACAACTCCAGGGTATCCCTCCAAGGAT
TCCGACACTGAACATATGTCACCGGTAGGGAGAGTGCCGAGGATACCATCGGTGCCGATGAGTGCTAGAGATGTC
CAACCAATTTTAGAAAGATTGAATGGCAGGGGTTTTCAAATTGGGCCCGGTAGTAATATAAAAGATTTTGGATCA
TTCACTGGACCTTCAAGCTCTATCGATAAAGTCCATTTGCATAATGAGCTAACTTACAACATCAAGGAAATGAGT
AGTGTAGAGGTTAGTATCCCTGGTATATTCACTGAGGGGGAGATTATTATCGGAGCTCATAGGGATTCGCTCGCC
TCGAGTAGCGCCGGTGATGCAAATAGTGGCAGCGCTATTCTTTTAGAAATTGCACGAGGAATGAGTAAATTACTT
AAGCATGGTTGGAAGCCACTGCGTCCTATCAAACTAATAAGTTGGGATGGTGAACGATCCGGCCTTCTGGGATCT
ACAGATTATGCAGAAGCTCATGCTGCGATTCTCAGGAGAAGGGCCTTGGTATACCTAAATCTAGATAATGCAATC
TCTGGGACAAATTTTCACTGTAAAGCCAACCCACTTTTACAAGACGTGATATACGAAGCTGCTAAGCTCACGGAA
TTTAATGGGCACGAAGACTGGTCATTGTTCGACCATTGGAAATACACTTCTAATGCCACTATTTCTCTACTTGAT
GGGTTGTCTAGTTACACTTCATTTCAGTACCATCTTGGAGTGCCCGCTGCACATTTTCAGTTTAATGCCAATGAT
ACTTCAGGCGCAGTCTATCATAGTAACTCCGTATTCGATAGCCCAACTTGGTTGGAAAAATTTACCAATTCTGAC
TACAAGTTACACAACACCATGGCCATGTTTGTAGGTTTGACAACGCTGATGCTGAGTGAAAACGAACTGGCAAGA
TTCAATACACATGTTTACCTGAAGAAAATATATAACTGGTATATCGCATGGCACTCTAATCTATCTTCAGCATTT
CCCCAGGACGATGAAGTGAACAGCTTAGCAAAAAGGGTTCTGGACTTATTAAAAGTTGCCACACAGGAAGATAGC
ATCCAATTTGACCAACAAAATGGTATTCTCTATAAAGAGTGTAGGGAAGCTTTACCTGTTTGGGCTTTTTACAAA
AAAATCAAGAGCTATATTAAACTGCAACGATCCAATAGCAAATCAAAGCAAATTGATCAATTATTTATTACACAC
AGAGGACTGAAAGACAGGGAATGGATGAAGTACTCTCTTTTAGCACCTAGTAAGTTTGAGGGATCTGTCGGGGAA
GTTTTGCCCGGCCTTCACGAAGGATTGGCTGATATTGATAGAAACGAGGTCATTCAGTGGTTAACCATTTTGCTA
AGCCAATTCAGCAACGTTCGCTATTTACTTCAATAA

>YOR021C 0.75   Cold
ATGAAGTACATTATTGAGCATATGGAGGAAGGGTTTAGTGAATGGGTCATTTTAGAGTATAGCCAAATCCTGAGA
GAAGTAGGAGCTGAAAACTTGATTTTATCATCGTTACCAGAGAGTACCACGGAAAAGGACATCCCTCAAAGGCTA
CTAAAACTTGGTTTAAGATGGACTACAAAGGATTTGAAGGGCATTAACGAAGATTTCAAGGATTTGGAACTGTTA
AAAGATGGCAGAGTCTGTCTATTGGACCCTAGGGCTACAATCGATTTACAGCCGGAGGATGCTACGAAATTCGAC
TATTTCGTATTTGGTGGCATATTGGGTGATCATCCTCCAAGAGATCGTACCAAAGAACTAAAGACTGCCTATCCG
AATCTTTTAATTAGCAGAAGACTAGGTGATAAACAAATGACTACGGATACTGCCATAAGAACAACACAGTTGATT
ATCAAAGACAGGATTGCATTTGAAGACATCAAGTTCATTGACTACCCTGAATTTAGGTTCAATAAAAATGAGGCC
ACGGAAATGCCATTTAGATACGTTTTAGATAAAGAAGGTAAACCTATTTTGCCTGAAGGGATGTTGGATTTGATC
AAGAAGGATTCTGCTCAAAGTTTGGACGATCTGTTGATGTAG

>YOR022C 0.64   Cold
ATGCTACGGTTTACTCATCGAGGCCTTCCCTCCAGCACTCGTTTTAGAAACATTTTCGTAAGGTTAAATCACATT
TATGTACCATGGTTCTATGCCATAGACGTACCCAACTCCAAACCCTATTTACCCACATATCAAACTTTACACTCA
CCCAAAAAGTTTAAGCCGTTCTCCGTTGATGATTCTAATCGTTTGGAAAAGGCCAGTAAGCGTCAAGAACGCAGG
CCTGTTTTAGTCAACGAAGATTACCTGTTTAAAGTCGACCTCTCTCACATGGAATTGTCCCCTACCTATTGGGAA
GGCCCTACTTATCAAGTACGAAGAGGTGTGTGGTTCGACTCTTCAAATCAACCTTTGTCCAGTGATCTTACCTCC

GAAATTGAAGGGTTGTATAAGCAGCTCAAATTCGACGACAGTAATGATGATCCGACCACGACACCTCCTGCAGAA
TCGCAAGATATATTTAGGCTCAAAGGTAAATACCCAGTCGATAAAGAAAACGAAGGAGAGCAAAAAAATGGATCC
AGCAATAAAGATGAGAACGAATCTACTTTCAAGTTTATTTTGTTTGCCAATAAACAAACTGCATTTTTACTATCG
GATTTAGATGGAGGAAAACTGCAATTAGCCTTTCTAAGGTCTAATCTGGCTCAATCCTTACCGATTAATGCTACA
ATGATTACAAGATCATACAAATATTCATCCTCCGCAACTACTAAACAGACATCCACATCTTTTAAGGCAGCAAAA
ACGCCTCAAACGGAAGTAGCAGATGGTAGTAACAGTTCCAAATCGAGGAGCATTGAAACAAAGTTAGAAAAGAAA
GTTTCAAACCTCTTCAATTTATCGGACTTTTTACAGTTGTTCAATGGTAATGCTAGTAAAGATCAAGATGATGCA
CAGAGCTTGGAAAAGCAAATGGAAACAGACTATAACAATGCAGATAACAGTCAAGGCGCTAATGCTAGTAGCAAA
ATAGAAGATGGCAAAAATTCGGGCGCAAGCGATAGACAAATTAGAAGTAACAGAAGAGATGTGGATAACTTGATA
CTATGCGTTCACGGTATAGGTCAGACTTTGGGTAAGAAGTACGAATACGTAAATTTCGCACACACGGTAAATCTA
CTTAGGTCTAATATGAAAAAAATCTACAATAATTCTGAGAAACTACAATCATTAAACACAGCACCTGATTATAAA
AGCAACTGTAACGTTCAAGTGCTACCCATCACCTGGAGGCACTCGATAAGTTTCCAAACGGATGCTAAGGAGGAA
AATATAGAAAATCCTGACCTACCGACTTTGTCACAAGTCACAGTAAATGGAGTGTTACCCTTGAGGAAGCTACTG
GCTGACGGTCTACTAGATATTTTGTTGTATGTCGAACCATACTACCAAGATATGATTCTACAACAAGTAACCTCT
CAATTGAACAAAACATATCGGATTTTTAAAGAATTTAATCCAGAGTTTGATGGGAAAGTCCATTTAGTGGGCCAT
TCGTTGGGTAGTATGATATTATTTGATATTCTATCCAAACAAAAAAAATATGAATTAGAATTTCAAGTAGACAAC
CTGTTCTTTATTGGCTCACCAATTGGATTGTTAAAGTTAATTCAAAGAACAAAAATTGGTGACCGTCCCGAATTT
CCCAATGACTTGGAGAGAAAATTAACCGTACAGAGACCGCAATGTAAGGACATTTATAATGTTTACCACGTCTGT
GATCCCATTTCTTATAGGATGGAACCTCTTGTTAGTAAAGAAATGGCTCATTACGAACAAACTTATTTACCACAT
TGTAGTGAAGCTTATGGACTAACTTCTAAAGTTTTGGAATTTGGCGAAAACATATGGAAAGATTTGCCAGGTACT
GATGAAAATAATTTGCAGTCCAAAAAAACTTCTCCAGAAAAAAAAGAGGTCAAGTTATCAGAAAATCTTACGAGA
ATGCTTACAGGTTTGAATTACACCGGACGTCTAGATTATGCTATGTCTCCAAGTCTGCTAGAAGTGGATTTTATA
TCAGCTATAAAATCACATGTTTCTTATTTCGAAGAACCGGATATTGCAGCGTTTATCCTAAAAGAAATTTTAAGT
AAACATGAAAATGCATCAGAAATATATGTAAAAAGAAAGACTGGTTGA

>YLR259C 0.78   Cold
ATGTTGAGATCATCCGTTGTTCGTAGTCGCGCTACTTTAAGGCCTTTATTGCGTCGTGCTTACTCCTCTCATAAA
GAATTGAAATTCGGTGTAGAAGGAAGAGCCTCCCTTCTTAAGGGTGTCGAAACTTTAGCTGAAGCGGTTGCTGCT
ACTTTGGGTCCAAAGGGTAGAAACGTTTTAATCGAACAGCCTTTCGGTCCTCCAAAGATTACTAAGGATGGTGTT
ACAGTTGCCAAATCTATTGTGTTGAAGGACAAGTTTGAAAATATGGGTGCCAAGTTACTACAAGAAGTTGCCTCC
AAAACCAATGAGGCTGCTGGTGACGGTACTACTTCTGCTACTGTCTTAGGTAGAGCCATCTTCACAGAATCCGTC
AAAAATGTCGCCGCTGGTTGTAACCCTATGGATTTGAGAAGGGGTTCTCAAGTTGCAGTTGAAAAAGTGATTGAA
TTTTTGAGCGCCAACAAGAAAGAAATTACCACATCTGAGGAAATTGCTCAAGTAGCAACCATTTCTGCCAATGGG
GACTCTCATGTTGGTAAGTTACTAGCTTCAGCTATGGAAAAGGTTGGAAAAGAAGGTGTCATCACTATCAGAGAA
GGTAGAACATTGGAAGATGAACTTGAGGTTACTGAAGGTATGAGGTTTGATCGTGGTTTTATTTCTCCATACTTC
ATCACTGATCCAAAGTCGAGCAAGGTGGAATTTGAAAAGCCATTGCTATTGTTGAGTGAAAAGAAAATTTCTTCC
ATTCAAGATATCTTGCCAGCTTTGGAAATTTCCAATCAAAGCAGAAGACCTTTGTTGATCATTGCTGAAGATGTT
GACGGTGAAGCTCTTGCGGCTTGTATTTTGAACAAGTTGAGGGGTCAAGTTAAGGTTTGTGCTGTGAAGGCGCCT
GGTTTCGGTGATAATAGAAAGAATACAATTGGTGATATTGCAGTCTTGACGGGCGGTACTGTTTTTACTGAGGAG
TTGGATTTGAAACCAGAACAATGTACCATAGAAAACTTGGGTTCTTGTGACTCTATTACCGTTACTAAGGAAGAC
ACCGTTATCCTGAACGGTAGTGGTCCAAAGGAAGCTATTCAAGAGAGAATTGAACAAATCAAAGGCTCCATCGAC
ATTACCACCACAAATTCATATGAGAAGGAGAAACTGCAAGAGCGTTTGGCCAAATTGTCCGGGGGTGTTGCTGTC
ATCAGGGTCGGTGGTGCATCTGAAGTTGAAGTTGGTGAAAAGAAGGACCGTTACGATGATGCTTTGAACGCTACC
AGAGCTGCAGTTGAGGAAGGTATCTTGCCAGGTGGTGGTACTGCCTTAGTGAAGGCATCTAGAGTTTTGGATGAA
GTTGTTGTCGACAATTTCGATCAAAAATTGGGTGTCGATATCATAAGAAAGGCCATTACAAGACCAGCCAAGCAG
ATCATTGAAAACGCTGGTGAAGAAGGTTCAGTTATCATCGGCAAATTGATTGATGAATATGGTGATGATTTTGCC
AAGGGTTACGATGCCTCTAAGTCAGAATACACCGACATGTTAGCCACTGGTATCATCGATCCATTTAAAGTGGTT
AGATCCGGTTTAGTTGATGCTTCTGGTGTTGCCTCACTATTAGCTACTACCGAAGTTGCTATTGTTGATGCCCCA
GAACCACCAGCAGCTGCTGGCGCTGGTGGTATGCCAGGTGGTATGCCAGGAATGCCAGGTATGATGTAA

>YFL034W 0.82   Cold
ATGTCCGATTCGGAGGAAGATTTAGGTGTACAGTTGAAAGGCTTGAAGATAGCGAGGCATTTGAAGGAGTCAGGG
GAGCATACTGATGAAGAATCCAACTCAAGCCCCGAACATGATTGCGGCTTGAGTAATCAAGATGATTTAACTGTT
ATGCATACACAGGCTAAAGAAGAAGTTTTTAAACGAAGAGAAGAAGACGGAACACGAACCGAAGATGCTTTACAT
GAGGGAGAAGCAGGAAAAGAGGGCACAGGTTTTCCCTCATCACAGTCAGTGTGCAGTCCTAATGAAGCAGATAGC
GGTATTGATCGGGCCGATAAACCTATTTTGCTGGACCCTTTTAAATCTGTGCACGATACGGATCCTGTTCCAGGT
ACAAAGTCCAGGAGCAATTCAGACTCCGACTCTGATTCAGATGATGGAGGCTGGCAAGAGATGCCCGCTGTCTCA
TCGTTCAATATATATAACCACAGAGGCGAACTAGAGTTGACTTCCAAGGTTAGAAACTCAGAACAAGCCTCTGAG
ACGTCTCCCACAGTTCCTCCAGGAAAAAATTGCAAAAGTGTCAATGATTCTAGATTTGACTATACGAAAATGGCC
GCTGAGCAGCAAGCCCAACGATCCTATCGAACAAATAAAAAAACAGATTTCCTTTTTGACCACAAAGTACTGAAA
AAGAAAATTAACAGTTCGCAAACTTCCGTAAATTTGACTTCTTCCCCTTCGACTACATCCTTGAACAATGAAAAA
AATAATGATGACGATGATGATGATTCTTATGACGAATATGAGGACGACGTGGAACCAGTGAACGACTTGAATCGG
GACTCGCAGTTAAACATAACGAAAAATCTGCTATCTGATATGGAGAAGTTTGCCTACGTAGGTGCAATAAACATT
TTGGCAAATCAAATGTGCACGAATTTGGCTACGCTGTGTCTTTGTATTGACATTAAATCCCACAAGAACCTAGCA
CATAGATTACAGTTTACCCAGAAGGATATGGCTGCTTGGAAAACTGTGGTTTTATCACGGCTATATGATCATTTG
GGATATCTCAAGAAGAAATTGTTATGATTGAGAAGCTTTCTCTGCACAAAATCCAATTGGAAGATTTGTGTAAA
TGTTTGAAAACCACTCAGAGCATTGATAATCCATGGGAGAATGATAGAGACCACGAAGAAGACGGTATAGAGGAA

ACTACGGAACGAATGAGTCCAAATGAACAAAATGGCTCAGTACAGGCTAGTACTCCCGACCCAGAACAATCCGCA
ACACCTGAAACACCAAAGGCTAAACAATCACCGTTATCTTCGGACGTTCCAGGAAAAGTACTCGATCCGGAAAAT
GTCAAAAGCCAAGACAAATTAAATATAGATGTGGCGTGGACCATTATATGTGATCTTTTCCTAATATGCTTGCAA
TCCTCCACCTATGATTCGAGGTCAAGGACTTTGTTGATAAATTTTGCCAAAGTCTTGAATATGACTAGCTTGGAA
ATTTGTGAATTTGAAAGACGAGTAACAGATTCCTTAGACATGGAACAATCTACAGAAGATCAAGTATGGGATGAA
CAAGATCACATGAGAAATAGACGGAGAAGCAAAAGAAGAAAGAAAATGGCATATGTCGCACTTGCCATGGTAGGT
GGTTCTTTGGTCCTTGGACTAAGTGGTGGTTTACTAGCCCCCGTCATTGGCGGGGGGATTGCTGCTGGTTTGTCA
ACAATAGGTATTACTGGTGCTACAAGTTTCTTGACCGGAGTGGGTGGTACCACTGTTGTTGCCGTTTCAAGTACT
GCTATTGGTGCCAACATCGGTGCTAGAGGTATGTCAAAAAGGATGGGAAGTGTGAGAACTTTTGAGTTCAGGCCA
CTGCATAATAATAGAAGGGTTAATCTAATCCTAACAGTGTCAGGCTGGATGGTTGGTAACGAAGATGATGTTAGA
TTGCCATTTTCTACGGTGGACCCTGTTGAAGGTGACCTGTACTCGTTATATTGGGAGCCCGAAATGCTAAAGTCT
ATTGGTCAAACGGTCAGTATTGTAGCTACCGAAATTTTTACCACCTCGCTTCAACAAATTTTGGGTGCCACTGTT
TTAACAGCCTTGATCAGTTCTATCCAATGGCCGATGGCTTTATCGAAATTGGGTTATATTTTAGATAATCCGTGG
AATGTTTCTTTGGACAGAGCTTGGTCTGCAGGCAAAATTCTTGCTGACACCCTAATTGCAAGAAATTTAGGTGCC
CGCCCCATTACACTGGTTGGCTTCTCAATAGGTGCGAGGGTCATTTTTTCTTGTTTAATCGAATTGTGCAAGAAA
AAGGCTCTAGGTTTGATTGAAAACGTTTATCTATTCGGTACACCAGCTGTCATGAAAAAGGAGCAACTAGTCATG
GCAAGATCTGTAGTTAGTGGGAGGTTTGTAAATGGTTATTCGGATAAAGATTGGTTTTTAGCATATTTATTTAGA
GCTGCTGCTGGTGGATTTAGTGCTGTTATGGGTATTTCTACGATAGAAAACGTTGAAGGTATCGAGAATATTAAT
TGCACTGAATTTGTTGATGGTCATTTGAATTATCGTAAAAGCATGCCTAAGTTATTAAAAAGAATTGGTATTGCT
GTTTTAAGTGAGGAGTTTGTCGAGATAGAGGAGATGATGAACCCAGAAGAAGTGAAAAGAAAAAGGAAATTAATA
AATGATGTCGATGCAGCCCAAAAAAAAACTAAGTGAAAGAAAAAAGCATAACAGTTGGGTGCCGAAGTGGTTGAAA
CCGAAGAAATCCAAATGGAAGGTCATGGTCGAAGAAGCTGTCGAAGAAGGAAGAGATATGCAAGACCTACCAGAA
AATGACGTCAATAACAATGAAAACGAAAATCCAGATGAACATGAAGGGATAGCAAGGCAAAAACGCAGAGATGCT
GCTCTTGTCGATCATGGGGCATTAATGCATGAGTTACAACTTATAAAACAAGCGATGCACGAAGACGAAATAAAG
AATAAAGCATGCCTTCCAGGAGAAGACAAGGAAGTGGAATCATCAAACGACTTCTTGGGGGAGTCGCATTACAAA
CCACCGTCAACACCAAAAATTAATCCACCACAAAGCCCTAATAACTTCCAATTGTTAAGTGCTGGGAGAACTATT
CTTCCAGAGGATGATGATTTCGACCCTCGAGGAAAAAAAAAGGTTGAATTCTCTTTCCCAGATGATATCTAG

>YLR152C 0.75   Cold
ATGTCCCTTTCTCTGGGTGCCGCTATTTACATTGCATTAAAACCTATATTTAAAATTTACACCATTATGCTTGTT
GGCTATTTGGTAGCTAAGTTCGATATTGTTTCCATGGAAAATGCGAAAGGTATATCAAATATGGTTGTTAACGCT
ATTTTGCCTTGTTTGACCTTCAACAAAATTGTGTCCAATATTTCATGGAGGGATATAAAGGAGATTGGAGTGATA
ATACTTTCAGCTTTTATCTTGTTTGTTTTAGGTGCCACGGGCGCCTTATTTACTACATTTGCTACGACTGTGCCC
AAAAAATTCTTCTGGGGGCTCATATTTGCGGGTTTCTTTCCAAATATATCAGATTTACCTATTGCCTATATTCAG
AGCATGGGTAACGGCTCCATTTTTACTGCTGAAGAAGCTGATAAAGGTGTTGCTTATTCCTGCATTTTTTTATTT
ATTCAAAGTTTCCTAATGATGAATTTTGGAATGTGGAGAGTGGTCGGACTTGATTTTAGAGATACAAAAGAACCA
GATAGTGAGAATATTACTCCGTCAGTAAGTCCCGCTATCGACGATCGCAAACTTACTGAAATAACAAAGTTACCT
AATATCACACGTCCTACAAATGCCTATCAATCGGAAGATGCGAGAAGTAATAGCGATTTGTCTTGTAACTCTATT
ACTACAAATGAAATGACACCGCAGGCATTCTACGAAGGTTTTACTGGCTACATCAAACCCTATAAAGAATCAAAT
GGTGCATCCCATAAATTTGAAAGTGACCTTCCGCATGCTGAAATATATAGGGTTTCCTCGACTTATTCAAGCCCA
GGCGCATTGGAGTTCTCGCGAATAGATGGCAGTTCCCTCTCATATAGTCGTATATCAAAAAATAGCGATGGCAGA
AGTTATAGAAGAAAAAGGAAAGCGGATATGAATGAGTTGATTTCCAAATACAGTGCCGCCGAAAAAATAAGGCAA
GGTGAATTAGATTTATCAAGACCACTATCCTTGACTGAAGAAGTTGGAAGCAGGAACGCAAGTATTGGTAATGTG
CACACTGGTTATACCGATGAAAGTTCCATCGAAGAAGAAAATTGCACTAATATGGCCACCGACGGGAGGGGTAGC
CTCTCCTTCTTTATAGAGAGACACAATCTCAAATGGTTGCAGTACTTTATTATAAATTGCCTCAGGCCAGCATCT
CTGGGGGCAATACTAGGAATAATATGTGCATTAATTCCCTGGGTTAAAGCATGCTTCGTAACGACATATGTTCAC
GTTCATAAGGCGCCTGATGGAGAACCTGTTCTGAATTTTTTAATGGATTTTACAGAGTATATTGGAAATGCATGT
GTTCCATTGGGGCTTCTATTACTTGGCGGAACGTTGGCAAGATTAGAAATTAAGTCATTGCCACCTGGATTTATT
AAATCCGCCTTATTGATGACATGCTTTCGATTAATTGTCATTCCTATTATTGGCGTCCTATGGGTAAATAAACTA
TATTCGATCGATTGGCTGGATACGGGCATAGGAAAATTTGACATGATATTAACGTGGTCCATGCCGAGTGCTACT
GCTCAAGTATATTTCACAGCATTTTATACGCCAGCATGTGGTGATCACATTCAAATGAACTGTTTGTCCGTCTTA
TTTGTCATGCAGTATGCGATTCTTTTCATCACTGTGGCGTTTGTTGTTACATATACCTTAAAGGTCGATTTAAAA
GTTTGA

>YKL161C 0.76   Cold
ATGGCGACTGACACCGAGAGGTGTATTTTCCGTGCATTCGGCCAAGATTTTATCCTAAATAAACATTTTCATTTG
ACAGGTAAGATTGGTCGGGGCTCACACAGCCTTATTTGTTCTTCAACTTACACAGAATCGAACGAGGAAACTCAC
GTGGCTATCAGAAAAATACCAAACGCGTTTGGCAATAAACTATCTTGCAAGAGAACTCTTCGTGAATTGAAACTA
CTAAGACATTTAAGAGGGCACCCAAATATAGTGTGGCTCTTCGATACTGATATAGTATTTTACCCAAATGGGGCA
CTAAATGGCGTTTATTTATATGAAGAACTAATGGAATGTGACCTTTCTCAAATTATAAGGTCCGAACAACGCCTG
GAAGACGCACACTTTCAAAGCTTCATATATCAGATACTGTGTGCTCTGAAATACATACATTCTGCTAATGTTTTA
CATTGTGACCTGAAACCAAAAAACTTACTTGTTAATAGTGATTGCCAACTAAAAATTTGTAATTTTGGGCTATCG
TGTAGTTATTCAGAAAACCACAAGGTTAACGACGGCTTCATTAAGGGTTATATAACCTCGATATGGTATAAAGCA
CCAGAAATTTTGCTGAATTATCAAGAATGCACAAAAGCTGTCGATATTTGGTCAACAGGCTGTATCTTGGCCGAA
CTACTTGGTAGGAAACCAATGTTTGAAGGGAAGGATTATGTAGATCATTTGAATCATATTCTACAAATACTTGGA
ACACCACCTGAGGAAACATTGCAGGAAATTGCCTCTCAAAAGGTGTATAATTATATCTTTCAGTTCGGTAATATC
CCGGGAAGATCGTTTGAAAGCATACTACCTGGTGCTAATCCAGAAGCGCTTGAATTGCTAAAGAAAATGCTAGAA

TTTGATCCTAAAAAAAGGATTACTGTAGAGGATGCACTAGAGCATCCATATTTGTCAATGTGGCATGATATAGAT
GAGGAATTCTCATGTCAAAAGACCTTTAGATTCGAATTCGAGCATATCGAAAGTATGGCGGAATTAGGAAACGAA
GTTATAAAGGAAGTATTTGATTTCAGGAAAGTTGTTAGAAAACATCCTATTAGCGGTGATTCCCCATCATCATCA
CTATCTTTAGAGGATGCCATTCCTCAAGAAGTTGTACAGGTCCATCCTTCTAGGAAAGTTTTACCCAGTTATAGT
CCTGAATTTTCCTATGTAAGCCAACTTCCATCACTAACTACAACCCAGCCATATCAAAACCTTATGGGAATAAGC
TCTAATTCATTTCAGGGTGTTAACTAA

>YMR210W 0.8 Cold
ATGCGTCTAAAAGAATTGTTACCTAATTTTTTGATTGTTCATCAAGAGGTCCCTGAGGATCCTATTGCATTCAAG
TCCACCGATAAACGAGAGAATGAAAATAAAGAGATCACCATCCCTGAGCTAATAGATACTAAAGTTCCTGAATTA
GCTGACGGTGCTACTGACACTTTATATGGTTTACTGGTCAATGGCCATTTACAAACTGCATATGGTTCCTTTAGA
CACTTTGACAATATATACAAAGTTCAATATAAAAGAATGATAATCAAATACCCACATGGGGGAGAAGGGACTGTG
GATTTTGCTGTAAATGGTAGAAGTACCAAAAGAAGAAAAGTGGAAAAAGAATACGTGCCGACAAGTCAACCGGTA
TTTAACGGAAACTTGAAACGAAGATATTCGTACTATTCCCCTGATGATCCTAAATTGAACTCAGACGATGCTAAG
CCTATGCTTATTATTCTCCATGGATTAACAGGGGGTTCGAGGGAAAGCTACGTGAGGGCAATTGTTCATGAAATC
ACCACGAAGTATGACTTTGAAGCATGCGTGTTTAATGCTAGAGGATGTTGTTATTCTGCAATTACAACGCCGCTA
TTATACAACGGTGGTTGGACCAATGATATAAGATATTGTGTTAATGACTTGAGGAAAAGATTTCCGAATAGAAAG
TTTTATATGATGGGATTTTCGTTAGGCGCATCTATAATGACAAATTACTTGGGAGAAGAGTCAGATCGTACTAAA
ATCGAATGTGCTATTTCCGTGAGTAATCCATTTGACCTGTACAACTCTGCATATTTTATTAACAGTACACCAATG
GGGTCACGATTTTATTCACCTGCTTTGGGTCACAACTTACTACGCATGGTTCGAAACCATCTCTCTACTCTGGAA
GAAAACCCTGATTTCAAAGATGTTATCGAGAAGCATTTAAAAAAGATTCGCACTGTGAGGCAATTTGATAACTTG
TTGACAGGCCCAATGTTTGGATATAAAAATGCAGAGGAGTATTACAAGAATGCTTCATCATATAAAGAATACCC
GGGATCAGAACTCCTTTTATAGCTTTGCATGCTCAGGATGACCCAATTGTCGGAGGTGATCTTCCTATAGACCAA
ATAAAATCCAATCCATACACTTTGCTTCTAGAAACTTCGACGGGGGGGCATGTTGGATGGTTCAAAGACAGATCC
GGCAGAAGGTGGTACGCAGAACCCTTATGCAGATTTTTGAAAATATTTCACGACGAAATTACTGTAAAGGGCTTA
AAACCTGACTTGGAAAATGTTCAACTCCCAGATCCTAATTGCGAACCCATAGCCACAACCTTTCGCGCGAATTAG

>YGR129W 0.8 Cold
ATGGATTTTTACAAATTAGACGAGAAGCTGAAGGAGTTGAAAAGGAAAAGAGTAGATGTATCTATAAAGAGTAGG
AAGTTGGCTGACAGAGAGATTCAGGAAGTAAGCGCAAATCGAAAACCAAGAGTATACAGTATGGAAGACGTAAAT
GATGCAGATGAATCAGTAGGAGATACAGAAAGTCCTGAAAAAGAGAAAGCATTTCATTACACTGTCCAAGAATAT
GATGCGTGGGAACGTAGGCATCCTCAAGGGAAGACTGGGCAAAGCCAAAGAGGTGGAATTTCCTATGATCAACTT
GCAAAATTGAGTTATGAGAAGACCTTGCGGAATCTCGCTACGCAAACACAGAATTCGAGCAAACAGGATAGTTCC
GCCGATGAAGAAGACAATAAAAACGTACCCAAGAAGGGTAGAATCGGCAAGGTACAGAAGGACACTAAGACGGGT
AAGATAACAATTGCAGATGACGACAAGCTAGTTAATAAGTTGGCCGTTTCTTTGCAATCTGAGTCGAAAAAAAGA
TACGAAGCAAGAAAAAGACAAATGCAAAATGCAAAAACACTGTATGGGGTTGAAAGCTTCATTAATGACAAAAAT
AAACAATTCAATGAAAAATTGAGCAGGGAATCAAAAGGATCAGAATAA

>YOR311C 0.81 Cold
ATGGGGACCGAAGATGCCATTGCCCTTCCAAATAGCACGCTAGAGCCGCGTACCGAAGCTAAGCAAAGACTATCA
TCTAAGAGTCATCAAGTCTCGGCGAAAGTAACGATTCCAGCAAAAGAAGAAATTAGTAGTAGCGATGACGATGCA
CACGTTCCAGTGACAGAAATACATTTGAAATCTCATGAATGGTTCGGCGATTTTATAACTAAACATGAAATTCCC
CGTAAGGTGTTCCATTCTTCCATTGGCTTTATTACTTTGTACCTGTATACGCAGGGTATTAATTATAAAAATGTT
TTATGGCCTTTGATATACGCCTTCATCATATTGTTTATTTTGGATCTGATAAGACTAAACTGGCCCTTTTTCAAT
ATGCTTTACTGTAGAACTGTGGGTGCGCTAATGAGAAAAAAGGAGATTCATACATACAATGGGGTATTGTGGTAC
ATTCTTGGGTTAATCTTTTCTTTTAACTTTTTCTCTAAAGATGTTACCTTAATATCGTTATTTTTGCTAAGTTGG
TCCGATACAGCCGCCGCAACTATTGGAAGAAAGTATGGTCATTTAACACCCAAAGTGGCAAGAAATAAATCCCTT
GCAGGTTCGATAGCTGCGTTTACAGTTGGTGTTATCACCTGCTGGGTATTTTATGGCTATTTTGTTCCTGCCTAC
AGCTACGTCAACAAACCTGGCGAGATCCAATGGAGCCCAGAAACAAGCAGATTAAGTTTGAATATGCTATCCTTG
TTAGGTGGTGTGGTAGCTGCTTTGAGTGAAGGTATAGATTTGTTCAACTGGGATGATAATTTCACTATTCCTGTC
CTGTCATCACTTTTTATGAACGCAGTAATCAAAACATTCAAGAAATAA

>YJL203W 0.69 Cold
ATGGAACCAGAAGATACCCAGCTGAAAGAGGACATTAAAACCACAGTAAATTACATAAAGCAGCATGGTGTTGAG
TTTGAGAACAAACTGCTAGAAGATGAGAGATTCTCATTCATCAAGAAAGATGACCCACTCCATGAATACTACACC
AAACTCATGAATGAGCCAACAGACACTGTCAGCGGTGAAGATAATGACAGAAAGAGTGAGCGAGAGATAGCAAGG
CCGCCGGATTTTCTGTTCTCTCAATATGATACTGGAATATCCCGAAGAGACATGGAAGTAATCAAATTGACAGCA
CGCTACTATGCAAAGGATAAAAGTATTGTTGAACAGATGATTTCAAAGGATGGTGAAGCCAGATTAAACTTTATG
AACAGCTCACATCCGCTACATAAGACATTCACTGATTTTGTCGCCCAGTACAAGCGAGTATATTCTTTTACTGGT
CAAGAAATCAAGAAAAGTAAGAGAACCATTTTAGATAATTGTTTTGAGAGAACACAGTATTGGGAATTTGAGAAA
GATAAAGACCGGGAACATGATAAGCTCGTAGAGTTGTGCAAAATACAATTTGCAGCCATTCCATGGGATAAATTT
ACTCAAGTGGCAAAATTTTCTATCCCTGAAGACACAGAAATCTTCGAAGGCTCTCTAGATTTAGAACAGATGAGG
CTGCGAAGAGTACAAACTGGTATTAAGTTATTCGATAGTATCAAGCCGACAAATGAAGAAGAAAAGATAGTATCC
GACCAAGGTAAGCAAAAAGGAGGAGATAGTAAGGGCAAAAAGCGTAAAATTAGAGCTGTGGGAGAGACAAGGTTA
AAGAAAAGTAAAAAATAA

>YGR011W 0.79 Cold

```
ATGTTAGGTCCTTCTTGCTTTCCCATGATATCATGTATGAACACAGAAGAAATATCCTTATTATCAAACAACTTA
TTTACAATGATATTTCCTCTACGAAAGTGCGGCTTTTCATCAGACGTGGAATGTCAGTTCAATATCTTCTTCCAA
ACTCTGTCATCCGTTACATCCAAGAGTATAATCTATACATTAATCAAAGTGAACCGGTCAAGCAGGTCTTGGATA
GCAAAGAGTGAGTTTATTACAACTCTGATACTGCAGCAGTTCAAATTTACCACTTTCCTCTTCAAGGTGCATAGA
AAAAAAGTTCCTGGATGCACGATTTAA

>YHR112C  0.74   Cold
ATGGTGGACTTGTCAACAGCTTTGATTCATGGCGATGATAAAGATAACAGGGTTACTGACGTGGCGCCCCCAATT
AATGTCTCTACAACCTTCCGTTACGACGATGACGACCTAATTCCATGGACAGAGCGTGAAAACTTGGACTTTATG
GAGAAGAAGCCCGTATATTCACGGCTAGCGCATCCAAATAGCACCAGATTAGAGAGTATCTTCTCAGAGATCCTC
GATGGTTATGCTGTCATTTATTCTTCAGGGTTAGCTGCGTTCTATGCGGCAATGGTACATTACAACCCTAAGAAG
ATCTTTATCGGACAGAGCTACCATGGTGTTCGAGCAATTGCTAACATATTGACCCGTAATTACGGCATTGAGCAA
CACCCCTTGGAAGATATTGAAAAATGTGCTTCTGAAGGTGACATTGTCCATCTGGAATCTCCTGTAAACCCATAT
GGTACCTCTTCTGATATCGAGAGTTTGGCCCGCAGAGCTCATGCAAAAGGCGCCCTGCTTATAGTGGATTCCACA
TTCGCATCTCCTCCATTGCAATACGCATGGAATTTCGGAGCCGATATTGTCCTATACTCTGCTACTAAGTACTTT
GGTGGTCATTCTGACTTGTTAAGTGGCGTCCTTGTAGTCAAAGAGGAAGCAACTTCCCGACAACTAAAAGACGAC
AGAATCTACTTGGGTACAAACGTGGCAAATTTGGAAAGTTTCATGTTGTTGCGATCCCTGAGAACCTACGAAATG
AGAATAACCAAACAATCAGAAAATGCTACGAAATTAGTTAGATTTTTGTCAGATCACCAATCTGAATTTGACAAA
GTATTGAAGACGATTTACCACTCTTCTTTACAAACAGAAGAGTTCGTTAAAAAGCAACTGGTAGGAGGATATGGT
CCCGTTTTTGCTATCACTTTGTACACCAAAGAGCAGTGTAAACAATTACCCCTCAAGCTGAAATATTTCCATCAT
GCGACCTCACTGGGTGGAATTGAATCCCTTGTGGAATGGAGAGCAATGACTGACCCATATATCGACCAAACTTTA
ATAAGAGTATCTGTGGGCTGTGAATCCGCTAATGATTTGATTAAGGATTTGGCTTCGGCCTTGAAAGAATTGCAG
GATGCAGCCTGA

>YGL099W  0.81   Cold
ATGCCACCAAAAGAAGCTCCCAAGAAATGGAAGGCGCCAAAAGGGCCAAAACCTACCCACCGTAAAAATAAAAAT
AAGCTTGAATTAGGCAGAGCTATTAAATATGCACGTCAAAAAGAAAATGCCATCGAGTATTTACCTGATGGTGAA
ATGAGGTTCACTACCGATAAGCATGAGGCCAACTGGGTTAAATTAAGATCTGTAACTCAAGAATCAGCTTTAGAT
GAATTCTTGAGTACAGCTGCACTGGCAGACAAAGATTTCACGGCCGATAGACATTCAAATGTTAAAATTATTAGA
ATGGATAGCGGTAATGATTCTGCGACATCTCAAGGGTTTTCTATGACTAATGAGCAGCGTGGAAATCTTAATGCG
AAGCAAAGAGCGCTTGCTAAGGATTTGATTGTTCCAAGGAGGCCTGAATGGAACGAGGGCATGTCCAAGTTTCAG
CTTGATAGGCAAGAAAAGGAAGCGTTTTTAGAATGGAGAAGAAAATTGGCACATTTACAAGAAAGCAATGAAGAC
TTGTTGTTAACACCGTTTGAAAGAAATATCGAAGTTTGGAAACAGTTATGGAGAGTTGTTGAAAGATCAGATTTA
GTTGTTCAAATTGTAGATGCGAGGAATCCGTTGCTGTTTAGATCTGTCGATTTAGAAAGATATGTAAAAGAGTCA
GATGACAGAAAAGCAAACTTACTGCTAGTTAATAAAGCAGATTTATTGACCAAAAAGCAACGTATCGCTTGGGCA
AAGTACTTTATCTCCAAGAATATTTCGTTCACGTTTTACTCTGCATTGAGAGCTAATCAATTATTGGAGAAACAA
AAGGAAATGGGGGAAGATTATAGAGAACAAGATTTCGAGGAAGCTGATAAAGAAGGGTTCGATGCTGATGAAAAA
GTTATGGAAAAAGTTAAAATTCTGTCCATTGACCAACTGGAAGAATTGTTTTTATCAAAAGCTCCAAACGAGCCT
TTATTGCCACCTCTGCCCGGTCAACCTCCACTGATTAATATTGGTTTGGTTGGTTATCCAAATGTAGGTAAATCC
TCCACTATTAATTCGCTCGTGGGTGCCAAGAAAGTTTCTGTTTCATCCACGCCTGGTAAAACAAAACACTTCCAA
ACTATTAAGTTATCTGATTCTGTCATGCTTTGTGACTGTCCCGGTCTTGTCTTCCCAAACTTTGCATATAACAAG
GGTGAGCTCGTGTGTAATGGTGTTTTACCTATTGATCAATTACGTGATTATATTGGTCCAGCAGGTTTAGTAGCA
GAAAGAATACCAAAGTATTACATTGAAGCGATTTATGGTATCCATATTCAAACCAAATCAAGGGATGAAGGTGGG
AATGGGGATATACCAACTGCTCAAGAATTGTTAGTCGCATACGCCAGAGCTCGTGGTTATATGACTCAAGGTTAC
GGTTCTGCTGATGAACCAAGAGCAAGTCGTTATATATTGAAAGATTACGTCAATGGGAAATTACTGTATGTCAAC
CCTCCGCCCCATCTAGAGGATGATACACCTTACACTAGAGAAGAGTGTGAAGAATTTAACAAAGATTTATATGTG
TTCGACAGATTACCGGACACCAGAAAGGAGCAAGTGCAAAATGCTGCTAAGGCTAAAGGCATTGATATCGTGGAT
TTAGCTCGTGATTTGAATCAGCTAACGTTTTCAGCTCACACTGGTGGTGACACACAAAAAGAAGCCAAATCTGTT
ACGCACGGTGGTAAACAAGCTGCATTGTACAATGCCGCTGAGGACTTGGATAGAGACTTCTTCAAGATGAACAAC
GTCGAAGGTAGATTAAGTACACCATTCCACAAAGTTCAAAATAGTTCAGCTGGTAAGAGACATAACAAAAAAAAC
AAAAGTAAAAATGCGAAAAGCAAAGTTTTTAGCATTGAAAATAATTAG

>YNL227C  0.75   Cold
ATGAAGACCTGCTACTATGAGCTTTTAGGGGTCGAAACGCATGCTTCTGATCTTGAGTTAAAAAAAGCTTACCGT
AAAAAGGCCCTACAATATCACCCAGATAAAAACCCAGATAATGTTGAAGAAGCCACACAAAAATTTGCTGTGATT
CGAGCCGCTTATGAAGTACTGTCTGACCCCCAGGAAAGAGCATGGTATGACTCACATAAGGAACAAATTTTAAAT
GATACTCCACCAAGCACTGATGATTACTATGATTATGAGGTAGACGCTACAGTCACAGGTGTCACAACTGATGAA
TTACTCTTATTTTTTAACTCTGCTCTTTATACTAAAATAGACAACTCAGCTGCTGGGATATATCAAATTGCAGGA
AAAATATTTGCCAAGTTAGCTAAAGATGAGATTTTAAGTGGTAAGCGACTGGGGAAATTTTCCGAGTATCAAGAT
GATGTATTCGAACAGGATATTAATAGTATTGGCTATTTGAAAGCCTGCGATAACTTTATTAACAAGACGGATAAA
CTTTTATATCCTTTATTTGGATATTCGCCAACGGATTATGAATATTTGAAACATTTCTATAAGACTTGGTCAGCG
TTCAATACCTTGAAAAGTTTTAGCTGGAAAGACGAGTACATGTACTCTAAAAACTATGACAGAAGAACCAAGAGG
GAAGTTAATAGAAGAAATGAGAAGGCTAGGCAACAAGCTCGAAATGAATACAACAAAACCGTGAAAAGGTTTGTA
GTTTTCATAAAAAAGCTCGATAAAAGAATGAAAGAAGGTGCAAAAATTGCAGAAGAACAGCGTAAACTAAAAGAA
CAACAGAGGAAAAATGAGTTAAATAACAGAAGAAAGTTTGGGAACGACAACAATGACGAAGAAAAATTTCATTTA
CAAAGCTGGCAAACGGTAAAAGAAGAAAACTGGGATGAACTGGAAAAGGTATATGATAATTTTGGAGAATTCGAA
AATTCTAAGAATGATAAGGAAGGTGAAGTATTGATTTACGAGTGTTTTATCTGCAACAAGACATTTAAGTCGGAA
```

AAGCAATTGAAAAACCACATAAACACTAAACTGCATAAGAAAAATATGGAAGAGATACGGAAAGAAATGGAAGAG
GAAAACATAACGCTTGGGTTGGATAATCTCTCCGATCTCGAGAAATTTGATTCAGCAGATGAAAGTGTTAAAGAA
AAAGAAGATATTGATCTGCAAGCATTGCAAGCTGAACTCGCTGAAATTGAAAGAAAACTGGCAGAATCGTCTTCT
GAAGACGAAAGTGAAGATGACAATCTCAACATAGAAATGGATATAGAGGTAGAAGACGTCAGTTCGGATGAAAAT
GTACATGTGAATACGAAGAATAAAAAGAAAAGAAAAAAGAAAAAAAAAGCAAAGGTTGACACAGAAACAGAGGAA
TCTGAATCGTTCGATGATACTAAAGACAAACGGAGTAATGAGTTGGATGATCTTTTGGCATCACTAGGAGACAAG
GGCTTACAAACGGATGACGATGAAGATTGGTCTACTAAAGCGAAAAAGAAAAAGGGCAAACAACCTAAAAAGAAT
TCTAAATCCACAAAAAGCACTCCGTCCTTGTCGACTCTACCGTCCTCTATGTCTCCAACCTCCGCGATCGAGGTG
TGCACTACATGCGGAGAATCATTTGATAGTCGAAATAAGCTATTCAACCACGTGAAGATAGCAGGGCATGCGGCA
GTGAAAAACGTAGTGAAAAGAAAGAAAGTCAAGACCAAAAGAATATAG

>YAR018C 0.79 Cold
ATGCATAGACGACAGTTTTTCCAAGAATACCGTAGTCCTCAGCAGCAGCAGGGACACCCACCAAGGTCAGAATAC
CAAGTTCTCGAAGAAATTGGGAGAGGTTCATTTGGGTCTGTACGAAAAGTCATCCATATACCTACCAAGAAACTT
TTGGTTAGAAAGGATATCAAATATGGCCATATGAATAGCAAAGAGAGACAACAGCTGATCGCTGAATGTAGCATT
CTATCGCAGTTGAAGCATGAAAATATTGTAGAATTTTATAACTGGGACTTCGATGAACAAAAAGAAGTGTTATAC
CTTTATATGGAATATTGTTCCAGGGGTGATTTATCCCAGATGATTAAGCACTACAAACAGGAGCATAAATATATA
CCAGAAAAAATTGTGTGGGGTATCCTGGCCCAATTATTGACTGCGCTCTATAAATGTCATTATGGTGTTGAATTG
CCAACTTTGACCACAATATATGACCGGATGAAACCACCGGTAAAAGGCAAAAACATCGTTATCCATCGTGATCTG
AAACCAGGAAATATATTCTTAAGCTATGATGATAGCGATTACAATATTAATGAACAAGTAGACGGTCACGAGGAA
GTGAATAGTAATTATTACAGAGACCATAGAGTGAATTCAGGGAAAAGGGGGAGCCCTATGGACTATAGTCAAGTT
GTGGTAAAGTTAGGTGATTTTGGGTTAGCCAAATCTCTGGAAACTAGTATTCAATTTGCCACAACATACGTCGGT
ACACCATATTACATGTCGCCTGAAGTGTTGATGGACCAACCATACTCCCCACTATCCGACATCTGGTCACTAGGT
TGTGTTATTTTTGAGATGTGTTCGTTGCACCCCCCATTTCAGGCAAAAAATTATCTCGAGCTACAAACTAAGATT
AAAAACGGGAAATGTGACACCGTCCCTGAGTATTACTCTAGAGGGCTTAATGCCATAATACATTCAATGATAGAT
GTGAACTTAAGAACCAGGCCTTCCACTTTTGAATTACTGCAAGATATTCAGATACGAACTGCAAGAAAGTCGTTG
CAATTAGAGAGATTTGAAAGGAAGTTACTGGACTATGAAAATGAGCTGACAAACATTGAAAAAATCCTCGAGAAG
CAAGCTATTGAATACGAAAGAGAACTGAGTCAGTTGAAGGAACAATTTACCCAGGCAGTGGAGGAGCGAGCCAGG
GAAGTAATTAGCGGTAAGAAAGTTGGTAAGGTTCCAGAATCTATAAACGGATATTATGGTAAAAAATTTGCCAAA
CCTGCATACCACTGGCAAACAAGATATCGATAA

>YKR084C 0.79 Cold
ATGGCTTACAGTGACTACAGCGATGGAGCAGACGACATGCCGGACTTTCACGACGAAGGTGAATTTGATGATTAT
TTGAATGACGATGAATATGATCTAATGAACGAGGTATTTCCTACTCTAAAGGCGCAGTTACAGGATTACCAAGGC
TGGGATAATCTTTCACTTAAGCTAGCTTTGTTTGACAACAATTTCGATTTGGAAAGCACGCTGGCGGAATTGAAG
AAAACTTTGAAGAAGAAGAAGACACCAAAGAAGCCAATTGCTGCTGCTAATGGGAGTGCGAATGTTACTCAAAAA
CTGGCAAACATCTCAATTTCGCAACAAAGGCCAAATGATCGGCTTCCAGACTGGCTTGACGAGGAAGAAAGTGAG
GGTGAACGCAATGGTGAGGAAGCCAATGATGAAAAGACAGTTCAAAGATACTATAAGACTACGGTGCCAACGAAA
CCAAAGAAACCTCATGATATTTCTGCATTTGTTAAATCTGCCTTACCTCACTTAAGTTTTGTTGTTCTTGGTCAT
GTTGATGCGGGAAAATCAACTCTAATGGGCAGACTACTTTATGATCTGAACATTGTCAACCAATCCCAACTGAGA
AAGCTACAAAGAGAGAGTGAAACTATGGGTAAGTCATCCTTCAAATTTGCATGGATTATGGATCAGACAAATGAA
GAGCGCGAACGTGGTGTAACAGTGTCAATTTGTACATCGCATTTCTCTACCCATAGGGCAAATTTTACTATTGTG
GATGCGCCAGGCCATAGAGATTTTGTTCCAAACGCTATAATGGGGATATCACAAGCAGATATGGCTATACTTTGT
GTAGACTGTAGCACCAATGCTTTCGAATCAGGATTTGACTTGGATGGGCAAACAAAGGAGCATATGCTACTGGCA
TCTAGTCTTGGAATTCACAATCTGATTATTGCTATGAATAAGATGGATAATGTTGACTGGTCCCAACAAAGGTTT
GAAGAGATTAAATCGAAATTGTTGCCATATCTGGTTGATATTGGGTTTTTTGAGGATAATATTAATTGGGTACCT
ATCAGTGGCTTTTCCGGAGAAGGTGTTTATAAAATTGAATATACAGATGAAGTGAGACAATGGTACAATGGTCCT
AACTTAATGTCTACTTTGGAAAATGCGGCGTTCAAGATTTCAAAGGAGAATGAAGGAATTAACAAAGATGATCCG
TTTTTGTTTTCTGTGTTGGAGATCATCCCATCGAAAAAAACAAGTAACGATTTAGCACTAGTTTCAGGAAAATTG
GAATCAGGTTCTATTCAACCTGGTGAATCTTTGACAATATATCCGTCGGAACAGAGTTGTATTGTGGATAAAATT
CAGGTTGGTTCTCAACAGGGCCAATCAACGAATCACGAAGAGACAGATGTTGCCATTAAGGGTGATTTTGTCACT
TTGAAATTGCGCAAAGCCTATCCAGAAGATATTCAAAATGGTGATTTGGCAGCATCAGTTGATTATTCGTCCATT
CATTCAGCACAGTGTTTTGTTCTGGAATTGACCACCTTTGACATGAACCGACCTTTGCTGCCAGGAACACCGTTT
ATTCTTTTCATCGGAGTCAAGGAGCAACCTGCTCGAATCAAAAGATTGATTTCATTTATAGACAAGGGCAATACT
GCAAGCAAGAAAAAGATTAGACATCTAGGCTCTAAACAACGAGCATTTGTAGAAATAGAACTAATTGAAGTAAAG
AGGTGGATTCCTTTATTAACTGCCCATGAAAATGACCGTTTGGGAAGAGTTGTTCTTAGAAAGGACGGTAGAACC
ATTGCGGCAGGTAAAATATCCGAAATAACTCAGTAG

>YOR126C 0.8Cold
ATGGATTACGAGAAGTTTCTGTTATTTGGGGATTCCATTACTGAATTTGCTTTTAATACTAGGCCCATTGAAGAT
GGCAAAGATCAGTATGCTCTTGGAGCCGCATTAGTCAACGAATATACGAGAAAAATGGATATTCTTCAAAGAGGG
TTCAAAGGGTACACTTCTAGATGGGCGTTGAAAATACTTCCTGAGATTTTAAAGCATGAATCCAATATTGTCATG
GCCACAATATTTTTGGGTGCCAACGATGCATGCTCAGCAGGTCCCCAAAGTGTCCCCCTCCCCGAATTTATCGAT
AATATTCGTCAAATGGTATCTTTGATGAAGTCTTACCATATCCGTCCTATTATAATAGGACCGGGGCTAGTAGAT
AGAGAGAAGTGGGAAAAAGAAAAATCTGAAGAAATAGCTCTCGGATACTTCCGTACCAACGAGAACTTTGCCATT
TATTCCGATGCCTTAGCAAAACTAGCCAATGAGGAAAAAGTTCCCTTCGTGGCTTTGAATAAGGCGTTTCAACAG
GAAGGTGGTGATGCTTGGCAACAACTGCTAACAGATGGACTGCACTTTTCCGGAAAAGGGTACAAAATTTTTCAT

GACGAATTATTGAAGGTCATTGAGACATTCTACCCCCAATATCATCCCAAAAACATGCAGTACAAACTGAAAGAT
TGGAGAGATGTGCTAGATGATGGATCTAACATAATGTCTTGA

>YKR085C 0.73 Cold
ATGATTGGCAGAGGTGTGTGCTGCAGATCGTTCCACACTGCTGGATCTGCCTGGAAGCAATTTGGATTTCCCAAA
ACACAAGTGACAACGATTTACAACAAGACTAAGAGCGCATCTAACTATAAAGGGTATTTAAAGCACAGAGATGCT
CCAGGAATGTACTATCAACCATCAGAATCCATCGCAACCGGATCTGTTAACAGTGAGACCATTCCACGTAGCTTT
ATGGCAGCCAGTGACCCTCGTAGAGGGCTTGACATGCCTGTTCAAAGCACTAAAGCGAAGCAGTGTCCAAATGTT
CTCGTAGGTAAGAGCACAGTGAACGGCAAAACCTATCATCTGGGACCTCAAGAAATTGATGAGATCCGGAAGTTA
CGTCTTGACAATCCTCAAAAGTATACACGCAAATTTTTGGCTGCAAAATATGGCATTTCGCCATTATTTGTATCC
ATGGTCTCGAAACCTAGTGAACAACATGTACAAATTATGGAAAGTAGATTGCAAGAAATCCAATCACGCTGGAAG
GAGAAGAGGCGTATAGCCAGAGAGGACCGTAAGCGTAGAAAACTCCTGTGGTACCAGGCGTGA

>YLR130C 0.73 Cold
ATGGTTGATCTTATAGCGAGGGATGACTCCGTAGATACTTGCCAAGCTTCTAACGGCTACAATGGGCACGCAGGT
CTTAGAATTCTGGCAGTATTCATTATACTGATATCGTCAGGATTGGGAGTTTATTTCCCAATTTTGTCATCACGG
TATTCGTTTATAAGGCTACCAAATTGGTGCTTTTTCATAGCGAAGTTCTTCGGTTCTGGTGTCATTGTTGCCACA
GCGTTCGTTCATCTTCTACAGCCCGCAGCCGAAGCTCTGGGAGATGAATGTCTTGGTGGCACATTTGCCGAATAT
CCATGGGCTTTTGGGATCTGTTTAATGTCGCTTTTCTTACTTTTCTTCACTGAAATCATCACGCATTATTTTGTA
GCGAAAACGCTGGGACACGATCATGGGGACCATGGGGAAGTTACCAGTATTGATGTTGATGCTCCCAGTTCGGGA
TTTGTCATCAGAAATATGGACTCGGATCCTGTATCTTTCAATAACGAAGCTGCCTACTCCATCCATAATGACAAA
ACTCCGTACACTACTAGAAATGAAGAGATTGTCGCTACTCCTATAAAGGAAAAAGAACCCGGCTCAAATGTTACT
AATTATGATCTGGAACCGGGAAAAACAGAGTCACTAGCTAATGAACTAGTTCCAACCAGTTCCCATGCGACAAAT
CTCGCTTCTGTACCTGGAAAAGATCATTATTCTCACGAAAATGACCATCAAGATGTCTCCCAGTTGGCCACACGT
ATCGAGGAGGAAGATAAAGAGCAGTATCTCAATCAGATACTAGCTGTTTTTATTCTAGAATTTGGCATCATCTTT
CACTCTGTATTTGTGGGTCTTTCGCTATCTGTCGCGGGTGAAGAATTCGAAACCTTATTTATCGTTTTAACTTTC
CACCAAATGTTCGAAGGTTTGGGTCTAGGCACAAGAGTTGCCGAAACGAATTGGCCAGAAAGTAAGAAGTACATG
CCTTGGTTAATGGGATTAGCCTTCACTTTAACGTCACCCATAGCAGTCGCGGTAGGTATTGGTGTCAGACACTCT
TGGATACCTGGCTCTAGAAGAGCATTAATTGCTAATGGTGTTTTTGACTCGATATCATCAGGAATTCTTATTTAT
ACTGGACTAGTCGAATTAATGGCTCATGAATTCTTATACTCTAATCAATTCAAAGGACCTGATGGCCTCAAAAAA
ATGCTTAGTGCATATCTCATCATGTGTTGTGGAGCTGCTTTAATGGCTCTTCTAGGGAAATGGGCATAG

>YHR196W 0.79 Cold
ATGGGCTCCTCTTTGGATTTGGTGGCGAGCTTCTCGCATGATTCCACTCGTTTTGCATTCCAAGCAAGCGTCGCA
CAAAAGAACAATGTGGATATTTATCCATTAAATGAAACTAAGGATTATGTGGTCAATAGTTCATTAGTGAGTCAT
ATTGACTATGAGACAAACGATATGAAGGTTTCAGATGTAATCTTCTTTGGGTGGTGTAGTGATTTGATAGATACG
CAGTCATCCAATATCAAAAGAAAATTAGATGAAGATGAAGGAACTGGTGAATCAAGTGAGCAAAGATGTGAAAAC
TTTTTCGTTAACGGATTTCCTGACGGAAGAATTGTTGTTTACTCTTCTAATGGTAAGGATATAGTTAATATCATC
AAGAATAAAAAAGAGATTTTGGGAGCAGACACAGACGAGTCTGATATATGGATTTTGGATAGTGATAAAGTCGTT
AAGAAATTACAGTATAACAATTCGAAACCTCTAAAGACTTTTACGCTAGTTGATGGTAAAGATGACGAAATAGTT
CATTTCCAAATTTTGCACCAAAATGGCACTTTATTGGTATGCATTATTACTAAACAGATGGTTTATATTGTTGAT
CCATCAAAGAGAAGACCCTCTACTAAGTATAGTTTTGAAATATCAGACGCTGTAGCATGTGAATTTTCTTCAGAC
GGTAAGTATCTGTTAATTGCCAACAACGAAGAATTAATAGCTTACGACTTGAAGGAAGATTCAAAGCTGATCCAA
TCATGGCCTGTCCAAGTGAAGACATTGAAAACTTTAGATGATTTGATAATGGCTTTGACTACTGATGGTAAAATT
AATAATTATAAGATTGGCGAAGCCGACAAAGTTTGTTCTATAGTTGTAAATGAAGATTTGGAAATTATAGATTTC
ACGCCAATAAACAGCAAGCAACAGGTTCTAATTTCATGGCTAAATGTCAATGAACCCAACTTTGAATCTATTTCT
TTAAAAGAAATTGAAACTCAGGGGTATATTACAATAAACAAAAATGAAAAAAATAATGCTGATGAAGCGGACCAA
AAGAAATTAGAAGAGAAAGAAGAAGAAGCTCAGCCTGAAGTTCAACATGAGAAGAAGGAGACTGAAACTAAGATA
AACAAGAAAGTTAGTAAATCAGATCAAGTTGAAATTGCTAATATTCTCTCATCGCATTTAGAAGCTAATTCAACA
GAAATATTGGATGATCTGATGTCTGGAAGTTGGACAGAACCAGAGATTAAAAAATTCATCTTAACCAAGATCAAT
ACAGTAGATCATCTAAGTAAGATATTTTTGACTATATCAAAGTCAATAACACAAAATCCATGGAATGAAGAAAAT
TTACTTCCGTTGTGGCTTAAGTGGTTGCTGACTTTGAAAAGTGGGGAGTTAAATTCTATAAAGGATAAACATACA
AAGAAAAATTGCAAGCATTTAAAATCTGCATTAAGATCTTCAGAGGAGATCTTGCCTGTTCTACTCGGTATACAA
GGTAGATTAGAAATGTTGAGGAGGCAAGCAAAATTGAGAGAGGATCTTGCACAACTATCTATGCAAGAGGGTGAA
GATGATGAAATAGAAGTTATAGAGCATTCAAATGTAATTAGTAATCCTTTGCAGGATCAAGCATCGCCTGTTGAA
AAGCTTGAACCAGATTCAATTGTTTATGCAAATGGAGAAAGTGACGAATTTGTCGACGCATCGGAATACAAAGAT
TGA

>YNL226W 0.71 Cold
ATGCTCTTTCCTGGGGGTCAGACAGTACTTCATAAGCGGCTCGAATCACAGCAAATTTTTGTGTGGCTTCTTCAA
CATTATCTGGGTTTTTATCTGGGTGATATTGTAGGGCCTTTTTACGGTAAGCTTTTTTTAACTCAAGATCAGAAG
CATGCGTTTCGACCCCTAAAAGCTCATAGTAGCAGGTCTTCATCCGTTATTTTTGAAGTAGTAGGATATCACTAT
ACTGATATTAACTCTTGGCTGGTCAGACTTTTACTATGTAAAAAGAGCTACTTTGCGATGACTTCAAATTTTTTT
CACGTATATTTTTTTTCTATTCTTAAAACAAGAAAAAGTGTAAACATAAAATGTTGGCCGCTTAAGCCCTCCAAT
TTTACAACAAATGAATATTTTATACATACTTCCTAG

>YPL183C 0.8Cold

ATGAAGGACTTGTCTCATTATGGTCCTGCGCTATGTGTTAAATTTTACAATGATTATGTTCTAGCTGGTTATGGG
CCTTTCATCCATGTCTATGACTATCATTCGGCTACGTTGATTAACAAATGTAGGCTATTTCACTACAATAAAGTT
CATGGACTTAGCCTTTCTAGTGAAGGGAAAATTTTGGCCTATGGTGCAAGATCAGTAACAATAGTGGAACTTGAA
GACGTTTTAAAGAAAGAGTCATTGGTGGATTTCGAAAGGATTAACTCAGATTGGATTACCGGTGCTACATTCAGC
TTTGACAACTTGCAAATATATTTGTTAACATGTTATAATAAAGTGCTAATTTGTGATTTAAATTGTGAAGTTCTT
TTTAGGAAGTCTCTTGGGGGAGAAAGATCTATTCTATATTCCGGTATAATTAAAGTTTTCGGTCCGGATAAAGTA
TATGTTAACGCTGGTACTGTAATGGGCGGTGTTATCATTTGGGACCTGTTCTCGGAGACAAAAAATTCATAACTTA
CTGGGTCATGAAGGTTCTATCTTTTATGTTAATTTAAGCAACAACGGAAGATATGTTGCCAGTTGCTCTGACGAC
AGATCAATTAGACTGTGGGATTTAGAAACTGGCAAGCAGCTGTCTGTTGGCTGGAGCCATACGGCAAGAATATGG
AATTTAATGTTTTTTGATAATGATTCAAAACTAATAAGTGTTTCTGAAGATTGCACATGCCGTGTATGGAACATT
ATCGAATCACGAGAAAACGTTGCCGAATTATCCATATCTAACGTTTACGAAGTACATTTAATCAAGAGTATATGG
GGCGTTGATGTGAAAGACGACGAAATGATAGCTGTAACCTCCGGGAATGATGGTAGGTTAAAGCTGATTGATCTT
CTTCAACTGAAGAGACACGGCGATGAAGAAACATCGTTCAGTCTAGATGATATCGCAAAGCAATGTGGCGATATT
TTCGAAAAGAATGAAAGTATCAAGGGATTTCAATGGTTTTCATTCGGTGTAATTGCTATCACTTCATTGGGTAAA
ATACTTAAATACAGTGATGTCACGAAACAATGGAAGTTGTTACTAACAAATGAAAAATTCAACTCGTATCCAATT
ACAAATGGGATTCAAACGCAAAATATCGCTGTCTTTTCCAATAATAAAAGTGACATCCTTCTTATCAAATTTAGC
AAAGATAGCGCTGACATAATTGAGACCGAAGAATTCCATTTAGATGAGCTTTCTAAGACAAATAACTGTCTCGTT
ACAGAATACGATGATGACTCCTTCCTATTGACTTTACAATCCCCAAATCCTCGAGAGAAGTTTGTATGTTTGGAA
ATAAGCCTTCAGAACTTAAAAATTAAAAGCAAGCACTGTTTCAATAAGCCGGAAAATTTTTCTTCCTCATGTTTG
ACCTCCTTCCGAAATCATATTTTAGTCGGCTCACGTTTTAGTACATTAGTAATCTACAACTTACTTGATGAGAGC
GAGGAGCCCTTCATAATTAGGAGATTAAGTCCCGGAGACACTACAACCTCAATCGAATTTGTAGAAGATAAAGAC
AACTCTGCGGTCTTCTCAGTAACAAATAGGGATGGCTATTATGTATTTATTGAGTTGACGAAAAACAGTTTGGAA
GAGGGTCCCTACCGACTAAGTTATAAGGTCTTGCATTCTAATAAGATGATGAAAGGTTTCTTGGAGGGCGCTTTC
TTTAATTCAAAAGGTGAATACATTACTTATGGGTTCAAGTCCAGCTTGTTTTATCTCTACAATGAAACCAACTGT
TATGAACTAGCTAGTGAAGTTTGTGGTGGTTCCCATCGTCTGTGGAATCTCGCAAAAATTACTGATGGCCACGTA
TTAATGTATATTAAGGCATCCCGATTTCATTTAAGGAAAATTTACAACTCCATTGTGCCTGAAACTTTAGAAAAT
GGTGTACACGGAAGGGAAATCAGAGATATTTCAATCTGTCCTGTTTCAAATACCAACACGAATGACAATTTTAAG
GATGGGCATATATTCTGTACTGCATCTGAAGATACTACAATTAAGCTGGGATATTTTAACAATAGAACAGGGAAG
GTGCAGAATTTTTGGACACAAAGAAAGCATGTGTCTGGATTACAACGTTGTCAGTTTATAAATCACAAACTAATG
ATATCATCTTCAGCTAGAGAGGAGTTATTCTTATGGGAATTGAACGATAAGTATAATAAACGACCATATATGACC
ATACGACAAGCTTTACCTGTATCCACAAATAATTCTGATCTAAGGATCATGGATTTTGATGTCAAATTTATATCA
CAGTCAGGTGACTTCTTATTAGTTACTGTTTATTCAGATTCAACAATAAAGATTTGGCACTATAGGGAAAATCAG
AATAAGTTTGACCTGATCATGCAAGGTCGTTATAAGACGTGCTGTTTATTCAACGTCGTATTTATTGCACTGAAA
GAAGAGCTCCTTGTTGTCATTTCACCAACTGATGGACATTTAGTGGTTTATAATATAACCGAGTACGTACCATTT
TCCGTTGATCCGATTTCTGGCGATTTGGTTGACCACAAACTGGACGCTACTATCTCAAATCTTCCCGCACCTGTA
GCGCAGTTGCCGGTACATCAATCTGGTGTCAAGTCCCTAGACTATGTTGCTAATGCTACAAGAACTTCCGCCACA
ATTTTGACAGGGGGTGATGATAATGGTTTAGGGTTAAGCAACCTGAAATTAGATGACTCGAATAAAGTAACGTTA
AAAACAAGTGACTTTATCGCTGCAGCAGCTTCTTCGACAATAACGTCCGGTATGTTGATTAATGGTGGCAAAGAA
GTCATTACCCACATCGGTTGATCAAGTAATACGCGCTTGGGAAATTACCGCAGGCAAGCTTTCACTGGTAGATAAA
AAGCGTACCACTGTGGCGGATACAGGATCATTAGAAATCATTTCCAATGATGAAGATGCTGATTCTGAGAAAACG
TTACTGATCGGAGGTGTTGGTTTATCAATTTGGAAAAAATGA

>YDL176W 0.78 Cold
ATGGCTACTGGCAGGATTCAATTTGCTGTTTCTACTCCCTGCAATACTAAGGGTAAACCGTCTGGTTATAGGTTA
TTTGAATTTAAAAACGATCGATTAGCACTGGTCCCATCAGAAAGAGGTTGTACAAAAGTAGATGTGAATGCAAAT
ATACAAGCGTTTTGTTATTTAAGGCCTAATGGTAGAGACACGTCAATATCTCCAGACGCAACGCATATTTTGGAC
TCATGTGATTACATGGTGCTGGCTAAATCCAACGGATTCATTGAAATCATAAGCAACTATCAATACAAGATAAAA
AATGGCCTGCGATTAGCGCCCTCTTATATCCTTAGATGTACTCCGGAAGATTTCGAATCCAATTTTTTTAGTGAC
TATATGATCGCTGGTCTTGAATATAGTCAAGGTTTATTATATTGTTGTATGTGCTCCGGTCGAATATATGTCTTT
GTAATGAACCTTCCCACTGATTACATTCAGTATAAGAACATGTACAACCCCATGTTTCCAGATTGCTTTTTCAAA
GTACATCACGATAATAATACAACACATTCTTCAGAAGAAGAAAAATTATTTGAAGGTAGTACGCGGTACACTGGA
AGGTCATGCTCTAAGCATATCTGCTACTTCCTTTTGCCTATAGAACCATCCCATTTAAGATCGTCACCCGTGGTA
TCTTCGTTTTGCAATATGTATCAAGGCCTGCCCATATATAGGCCTTCGATGTATTTACATATAGAACGAGGTATT
TCCACATTCCATATAAATCCATTAGACCGGTTTTGTTTCATGACAGTATCGCCACGATCACCACTATTTATAAGG
AAAATAATTTTACCATTGACTTATGTTACATTTTTAAGCACCTTTATCAGCCTAAAGAACAGTATACAGGGAGAT
ACATGCGGTGAGATACTTTCGTGGGACAACGTAGCGCAACAAAATGGATTTGGTTCTTTGTTCAGCTGGATTAGT
AACAAGTTTACATTCGATACGGATATTATAAATTCAACCATTTGGGATGATATTGTAAAATATTCTGGAACAGGA
ATGCTGGATTCAGGAATCGTCTGGAAACAGCGTCAAGGTCACGCTAAAGATGACATATATGAGTTATTCCACACT
CAAGATATGCTGGGAAGTAGTCGCCGAAATTCTTCATTCAGTACTGCTAGCAGCGAACCGAGACCGCTGAGTCGA
AGAAGGAGAGAATCTTTTCAAGCCTTAACTAGAGACGCTTTTAGAGAAAGAATGGATGTACCCTGTTCAACGAAG
TGGGAATTAGATTCTTTTATCCGAGGATTAAGAAGGAATACATTTATGGTAGATTTTGAAATTGTTGAAAAGATT
TCTCATAGAAATGGAAATGATGGGGTGAATGAAGATGATAACACTACAGATGAAAGTGATGAAACAATGACTTCT
TTTCTAACAGATAATTACAAAAAGATGGACATTGTGTGCATCGATCATTTCGTCACGTTGAGTGCATTCCGACCT
CGGTACTATGATGAACCAATAATTAAAATCGATTCATTATCGAACAAAAATGGCTCTGAAAATGGAACTAATGAA
GAAGAGTGGGCAGAAAGTCAAATGAAGGTGGATGGCCAAGTGATAGATGATGAGACAGCTCAATTCAAGCAGGCA
TTGGGAAATTTATGTTCATTCAAGAAATTATTTATGCTGGATGATTCTTTATGCTTTATATTGGACACACATGGA
GTTTTGTTGATAAATAGATTTGAAATAAAAAATACCAAAAATTTGTTAAGGAATTCCAAGGATACTATTCGGATA

ATACCCCATGATTTCGGATTGATTAATGATACAATTGTTATAATTAATGATATAGACGTAGGCACAGACAATGTT
TGCGCCCTTACGTTTCACTTGGTGGTAACATCAATGGCAGGAGAGATAACTGTTCTTAAAGGCGAATTTTTCAAA
AACTGCAGGTTAGGAAGAATAAAACTGTGCGATTCACTAAAGCTAAATAGAAAAGATCGGTTTGTCGACAAACTT
GCTCTAATCGATTATGACGGTTTAAATGCACAAAAAAGAAGGTTAGATTACGATGAAAAGGACCTTTATACATTC
ATTGTGAAAAAGGTAAAAAGAGACTAA

>YNR074C 0.79 Cold
ATGACAATTAACACAAAGAACATAGTTGTTGTTGGTGCTGGTGTGTTTGGTGTGTCTGTGGCAAATCACTTGTAC
AGGGAACTGGGTGGGACATACGCAATAAAGCTCGTCACAGCATCTAATTATGTGTATTTTCTACCCTCAGCTGTC
CGTTTGACGGTTTCAAAGGATTATACCAAGTCGATCCTGCCGTTAAAAAATGTTCTTGATAGTGGTATTGAAGTT
ATCAAAGATACGGCTGCCAGCTTTGATGATAAGGAGGTAGTTTTGGGGTCAGACAGGGCCATAAAGTTTGATATC
TTGGTTCTTGCAACTGGCTCAAAGTGGGCTGATCCAATTGGTTCAACTTATACCTTCGGGGATAACTATAAAGAG
TATTTTGAAAGAGAGGCTTCTCGAATCTCAGATGCAGATCACATACTTTTTCTTGGCGGTGGCTTTGTTAATTGT
GAACTAGCTGGTGAATTGTTATTCAAGTATTTGGAGGAGATCAGGTCTGGCAAAAAGCGTATTTCCATTATTCAT
AATTCCGATAAGCTACTGCCGGACTCTGGGTTATACAACGATACCCTAAGAAAAAATGTGACAGACTATCTCTCA
AAAAATGGTATTACGTTGTACTTGAACACAGTAGGGGCTTCTTTAGACACCTCGCCGAAACGTATTTTTCTAGGT
GAGGGCTCATCTAAATATATAGATGCTGATTTGATTTACAGAGGCGTTGGTATTTCTCCAAATGTGCCGGTCAAC
AGTATTTCAGACCTTTGTGACAAGAAAGGGTTCATTCAAGTTGAAAAAAACTTCAGGGTAAAAGCTGTTGAGGCA
GGAAACGTTTTTGCCATTGGTGATGTAACGAATTTTAGATATCACGGATTGGTTAAAAGAGATAATTGGGTTGAT
GTTTTGACCCGAAATGTTATAAGTTCTTTACAAGAAGGAACAGAGGCTAGTCTCGTTGATGCAGATTGTCTTGAG
ACAGGACATGCCCCAAGTGGTGTCTCCCTTGGGCCAAATGCAGGGTTTGGCCAATTTCCACTACCGTTACTTGGG
ACGATTAATATTCCATCGTTTTTAATTTCTAGAGCAAAGTCGAAGAATCTTTTCTCCGACAAAATGGAACCTTTA
TTCAAAAAATAG

>YGR185C 0.81 Cold
ATGTCCTCTGCTGCCACGGTTGACCCTAATGAGGCGTTCGGTCTCATTACCAAGAACTTGCAAGAAGTCTTGAAC
CCACAAATCATCAAAGATGTTCTCGAAGTACAAAAGAGACATTTGAAATTATACTGGGGTACCGCGCCCACTGGA
AGACCTCATTGTGGTTATTTTGTTCCTATGACCAAGCTTGCTGATTTCTTGAAAGCAGGTTGTGAAGTGACTGTT
CTTTTAGCAGACTTGCACGCCTTTTTGGACAACATGAAGGCGCCATTGGAAGTGGTGAACTATAGGGCCAAATAC
TATGAATTGACCATCAAAGCTATCTTGAGAAGCATCAATGTTCCAATTGAAAAGCTAAAATTTGTTGTTGGCTCT
TCTTACCAGCTAACTCCAGACTATACCATGGATATCTTTAGATTATCCAACATTGTTTCACAAAACGATGCTAAA
AGGGCAGGCGCTGATGTTGTCAAGCAAGTCGCCAATCCATTATTGAGTGGGCTTATCTATCCTTTAATGCAAGCG
TTGGACGAACAATTCTTAGATGTTGATTGCCAATTTGGTGGTGTCGACCAGAGAAAAATTTTTGTCTTAGCGGAA
GAAAATTTACCAAGTTTGGGCTACAAGAAAAGAGCACATTTAATGAACCCTATGGTTCCAGGTTTGGCTCAAGGT
GGTAAAATGTCTGCCTCTGATCCGAATTCAAAAATCGACCTTTTGGAAGAACCAAAGCAAGTTAAGAAAAAGATC
AATAGTGCATTTTGTAGCCCCGGTAACGTCGAAGAGAATGGTTTACTGTCATTTGTCCAATATGTCATTGCACCA
ATCCAAGAATTGAAGTTTGGTACAAATCACTTCGAATTCTTCATTGATAGACCAGAAAAGTTCGGTGGTCCAATT
ACCTACAAATCCTTCGAAGAAATGAAATTGGCTTTTAAAGAAGAAAAATTGTCCCCACCTGATCTAAAAATTGGT
GTTGCTGACGCTATCAACGAGCTATTGGAGCCAATCAGACAAGAATTTGCCAACAACAAGGAATTTCAAGAGGCC
TCCGAAAAGGGTTATCCAGTTGCCACACCACAAAAGTCGAAAAAGGCTAAGAAACCAAAGAATAAGGGTACTAAG
TACCCAGGCGCTACTAAGACCAACGAGATTGCCACGAAACTAGAGGAAACCAAATTGTAA

>YLR002C 0.79 Cold
ATGGCTAAGAGAAATAGATCTCAATTTCGCATTCAGGAAAGGACTGCAAAGAAAAGAAAGCACGAAGATTCCTTG
TTAGAAGGCAATGTTTTCCAAAATGCACCGGAAGATATGGATGAAAATACCATATATAGCGCAAAAGGCTCATCC
TGGGATGAAGAAGAACAGGATTACGAAATGGTTCCTCGGAAGAATCGGTCTGATACATCAAATCTTGTTGAAGGC
CTGCCTATAAAAGTTAACGGTAAAGTTGAAAGAAAATTGCATAAAGCCCAAGAAAAACCAAAGGACGATGATGAG
GAAGACGAAGACTCTAATGACTCTTCAGAAGATGATGAGGGACCTAATGAAGAACAAGAAGCAGAGGCAAAGGAG
GATGAGCCTGATACGGAGGAAAAAAATTCTGCAACTAAAAGAAGACATTGCCGATTTAGTCACTAAAGTTATGGAG
GAGCCTGAAGAAAATACTGCTGCGTTGGGGCGTCTATGTAAAATGGTGGAATCTAAAAATCCTAATACTTGTAAG
TTTTCCATGTTGGCTTTGGTCCCCGTGTTTAAGAGTATCATACCAGGCTATAGAATTCGACCACTAACTGAAACT
GAAAAGAAGGAAAAAGTTTCTAAAGAAGTCTCCAAGCTTAGAAACTTTGAGCAAGCCTTAGTTTATAACTACAAA
AATTACGTCGGAAGATTACAGAGTCTTTCAAAAACTCCCAGTAATGCTGCTCCTATACAGGTTTCATTAGGGATT
TTGGCCACTCAAGCCGCAAAAGAATTGATTTCAACGGCATCCCATTTCAACTTTAGAACAGACATTTTCACTTTA
CTGTTGCGTAGAATTTGTAAACCAAGAATTTCAACAGATCCAACTTCCATTCAAATAATCCAGACATTTGAAACT
CTATTAAATGAGGACGAAGAGGGCTCAATATCATTCGAGATACTAAGAATTTTCAACAAAATACTAAAGACGAGG
AACTTCAACATCGAAGAATCTGTTTTAAATATGTTATTATCGTTAGATGTCTTACATGACTATGACCCTAATACT
AAATTGAAAGGTAACGTTAGCGCTCCTAAGCTCAAAAAGAAAGATAGGGTCCATCTTTCTAAGAAGCAAAGAAAA
GCTCGAAAAGAGATGCAACAAATTGAGGAAGAAATGCGTAACGCTGAGCAAGCTGTTTCTGCGGAGGAAAGAGAA
AGAAACCAATCCGAAATTCTAAAAATCGTTTTCACTATATATTTGAATATCTTGAAAAACAATGCAAAAACACTC
ATTGGATCGGTTTTGGAAGGTCTAACGAAGTTTGGTAATATGGCCAACTTTGATCTATTAGGGGACTTCCTCGAA
GTAATGAAAGAACTTATTAGTGACACTGAATTCGATAACCTTTCCTCTGCTGAGGTCCGTAAGGCTTTGCTTTGT
ATCGTTAGTGCATTCTCACTTATTTCAAATACACAATATATGAAAGTTAATGTGGATTTGTCCAAATTCGTCGAT
GGGCTTTACGCGCTATTACCTTATATCTGTCTTGATGCAGACATCGAATTATCTTATAGATCGCTAAGGTTGGCT
GACCCACTCAATAACGAAATAATAAAACCATCAGTTAACGTTTCTACCAAAGCAGAACTTCTGCTAAAGGCTCTG
GACCATGTATTTTTTCCGCTCTAAATCAGGGACTAAAGAAAGGGCTACGGCGTTTACCAAAAGATTGTACATGTGC
ATCAGCCATACCCCAGAAAAAACAAGTATTGCCATTCTTAAATTTATTGATAAATTAATGAATAGATATCCAGAA

ATTTCAGGCCTTTACTCTTCCGAGGATAGAATAGGCAACGGTCATTTCATAATGGAAGCCGATAATCCTTCAAGG
AGTAACCCAGAGGCAGCAACCTTATGGGATAATGCTCTTCTTGAAAAGCATTATTGTCCTGTAGTTACAAAGGGG
CTACGCTCTCTATCATCTAGATCTAAAGAGTGTTCTAAATAG

>YKR088C 0.79 Cold
ATGAGTCAGTCTTACGAAGCAGGAAATGCCAATATGGGCCAAGGGGAGGATGATGAATTTGATGGCTATTTCGAA
GACTTTGACAACGATATCATGCCAAATAGTAATAATGGACAAAGGGTTGGTACTAATGCTGGATTGTCATTCAAT
GACGAAGTAAATGTTAATGATGATGATTTTTTAGACATTTATAATATGTCCCCTAGAGAGAGATTGATGCATAAT
ATCAGGAAGAATGTTCAAAAATTACAGTTTTATTTTTATTCACTACGCTTATGGCAACAGATTATCATTGTCCTC
CTCGGCATAATGCTAATGATAATGGGTATATTGTTGCTCGTCTTCCATAATGCGATTTTACATAAAGTTGTTGTT
ACTTCGAACGATTTGAGAGAAAAGATGTCAACACATTTTATATTAATGGTTCTGATATTCTTTGTCGCATTTCCT
CCTATGATTGGCTACTCCTTATTATCCACCACTACTGGGTTGATCTATGGTGTCAGTTTTGAGGGATGGGTTACG
CTAGCTTTGGGATCTGTAACAGGTTCCATTGCCTCTTTCGTTGTGTTCAAGACGATCTTGCATTCAAGAGCAGAA
AAGCTTGTGCATTTGAATAGAAGATTTGAAGCATTGGCTTCAATTTTACAAGAAAACAATAGCTATTGGATTCTA
GCTCTACTGAGATTATGTCCCTTCCCATATTCTTTGACTAATGGTGCCATAGCTGGTGTTTACGGTATCTCGGTA
CGCAACTTTTCCATTGCCAACATAATAACCACACCAAAGCTGTTCATCTATCTATTTATAGGTTCTCGTGTAAAG
AGTCTCGCAGAATCTGAATCTACAGGATCTAGGGTATTTGACTTGGTAAGCATTATTATCACATTATTAATCCTA
AGTCTTACAGCATGGCTCTTATACTTCAAGACTAAGAAGAGATATTTAGAACTACAAAACCGCGACCGTCAAGTG
TCCACTGATCAACTACCTGAATTGTCTTTTGAGGTCTAA

>YLR032W 0.76 Cold
ATGAGTCATATTGAACAGGAAGAAAGGAAGAGGTTTTTTAACGATGACCTTGACACTTCAGAAACATCGTTAAAC
TTCAAATCTGAGAATAAAGAGTCGTTTTTATTTGCAAATAGTCATAATGATGATGATGATGATGTTGTTGTATCA
GTGAGTGATACAACAGAAGGAGAGGGAGACCGATCTATTGTGCCAGTCAGGCGAGAAATAGAAGAAGAAGGCCAG
AATCAGTTTATTACAGAGCTTTTAAGGATTATTCCAGAAATGCCAAAGGATCTTGTGATGGAACTTAATGAAAAA
TTCGGCAGTCAGGAGGAAGGGCTCTCTTTAGCATTATCACATTACTTTGATCACAATAGCGGGACGTCTATCAGC
AAAATACCGTCTTCCCCGAATCAACTAAATACACTCTCAGATACCTCAAATTCAACCTTGTCCCCATCTTCATTC
CATCCTAAAAGAAGAAGAATATACGGGTTCAGAAACCAAACACGATTAGAAGATAAAGTTACTTGGAAAAGATTT
ATAGGTGCTTTGCAAGTCACTGGTATGGCTACCAGACCCACCGTCAGGCCCTTGAAGTACGGCTCTCAGATGAAG
CTAAAGAGATCAAGTGAAGAGATTTCTGCTACTAAAGTATATGACTCACGTGGTAGAAAGAAAGCGTCCATGGCT
AGTTTGGTAAGAATTTTTGATATCCAATATGATAGAGAAATTGGCAGAGTTTCGGAAGACATTGCTCAAATACTA
TACCCTCTTTTAAGTTCACACGAAATAAGTTTTGAAGTTACATTGATTTTTTGTGATAATAAACGGTTGAGTATA
GGTGATAGCTTTATTCTACAATTGGATTGCTTTTTAACATCTCTCATTTTTGAGGAACGTAATGATGGAGAATCC
TTGATGAAAAGAAGACGTACAGAGGGAGGAAATAAAAGAGAGAAAGACAATGGAAATTTTGGAAGAACATTGACT
GAAACTGATGAAGAGCTAGAAAGCCGCTCGAAAAGACTGGCTCTACTAAAGTTATTTGATAAATTGAGGCTAAAG
CCTATTTTGGACGAGCAGAAGGCATTAGAAAAGCATAAAATAGAGCTTAATAGTGACCCCGAAATCATTGATTTA
GATAACGACGAGATTTGCTCTAATCAAGTGACTGAAGTCCATAACAATCTCCGAGATACTCAGCACGAAGAAGAA
ACAATGAACTTGAATCAATTGAAAACATTTTATAAGGCCGCACAATCATCAGAATCTTTAAAAAGTTTGCCTGAA
ACAGAACCTTCTCGCGATGTCTTCAAGCTAGAACTAAGAAATTATCAAAAGCAAGGTCTTACTTGGATGCTAAGG
AGGGAGCAAGAGTTTGCCAAAGCAGCCTCTGATGGTGAGGCTTCAGAAACGGGTGCTAATATGATAAACCCATTA
TGGAAGCAGTTCAAATGGCCAAATGATATGTCGTGGGCAGCTCAAAATTTGCAGCAGGACCATGTAAACGTTGAA
GATGGCATATTCTTTTATGCGAACTTACATTCTGGTGAATTTTCGCTAGCAAAACCTATATTAAAAACTATGATA
AAGGGTGGCATATTATCAGATGAAATGGGGTTGGGTAAAACAGTGGCAGCGTATTCTTTAGTTTTATCTTGTCCT
CACGATAGTGATGTTGTTGACAAGAAACTGTTTGATATTGAGAACACAGCAGTCTCAGATAATCTTCCAAGCACT
TGGCAAGATAATAAGAAACCATATGCTTCAAAAACAACGCTAATCGTGGTCCCAATGTCTTTGTTAACGCAGTGG
AGTAACGAGTTTACAAAAGCTAATAATTCCCCCGATATGTATCATGAGGTGTATTATGGTGGGAATGTTTCCAGT
TTGAAAACCCTATTAACCAAGACAAAAACCCCTCCAACTGTAGTCCTTACTACATATGGTATTGTTCAAAATGAA
TGGACTAAACATTCCAAGGGAAGGATGACAGATGAGGACGTCAATATATCTTCAGGCTTATTTTCTGTCAATTTT
TATCGCATAATAATCGACGAGGGTCATAACATTAGAAACAGAACGACAGTTACATCTAAAGCAGTCATGGCTTTA
CAAGGCAAATGTAAATGGGTTTTAACAGGAACACCAATTATTAACAGGCTTGACGATTTATACAGTCTGGTTAAG
TTTTTAGAGTTAGATCCCTGGCGGCAAATTAATTACTGGAAGACCTTTGTATCAACGCCTTTTGAGAGTAAAAAT
TACAAACAAGCATTTGATGTGGTGAATGCAATTCTGGAACCCGTATTGTTAAGAAGGACAAAACAAATGAAAGAT
AAAGATGGTAAGCCATTAGTAGAGTTGCCACCAAAGGAGGTCGTTATTAAAAGACTTCCCTTCAGTAAATCTCAA
GATCTTCTATACAAGTTTCTGTTGGATAAGGCTGAAGTTTCTGTTAAATCGGGTATTGCACGCGGTGATTTATTG
AAAAAGTACTCCACTATCCTTGTCCATATTTTAAGATTGAGGCAAGTCTGTTGCCATCCCGGTCTTATTGGGAGT
CAAGATGAGAACGATGAGGATTTATCTAAAAATAATAAATTGGTTACGGAACAAACGGTGGAGCTTGACTCTTTA
ATGCGTGTTGTTTCCGAGAGATTCGATAACTCATTTTCTAAGGAGGAATTAGATGCAATGATACAAAGATTAAAA
GTTAAATATCCAGACAATAAATCGTTTCAGTCCTTAGAGTGCTCCATCTGCACAACGGAACCTATGGATTTGGAC
AAGGCTTTATTTACAGAATGCGGCCACAGTTTTTGTGAGAAATGTTTATTTGAATATATTGAGTTTCAGAACAGT
AAGAATTTGGGTTTAAAGTGCCCCAATTGCCGTAACCAAATAGACGCTTGTCGGTTGTTGGCATTGGTACAAACG
AATAGCAACTCGAAAAATTTGGAATTCAAACCATATTCACCAGCCTCCAAATCAAGCAAAATCACTGCTTTATTG
AAGGAGCTTCAATTGCTACAGGATAGTTCGGCAGGCGAACAAGTTGTCATTTTTTCCCAATTTTCTACATACTTG
GATATCCTGGAGAAAGAGCTAACTCATACTTTCTCAAAAGATGTTGCAAAAATTTATAAATTCGATGGACGTCTC
TCATTAAAAGAAAGAACTAGTGTATTAGCAGATTTTGCCGTTAAAGACTATAGCAGGCAAAAAATCCTATTACTC
TCGCTGAAGGCTGGTGGCGTGGGTTTGAATCTAACGTGTGCTTCCCACGCTTATATGATGGACCCATGGTGGTCG
CCCAGTATGGAAGATCAGGCAATCGATAGACTGCATAGAATTGGCCAGACAAACAGCGTCAAAGTTATGAGATTT

ATCATACAAGATAGCATAGAAGAAAAAAATGCTACGCATTCAAGAAAAGAAGAGAACCATCGGTGAGGCCATGGAC
ACAGACGAAGACGAGAGAAGAAAAAGGAGAATTGAAGAAATCCAGATGCTGTTTGAATAG

>YDR030C 0.72 Cold
ATGGATCCATTTTTAGAGTTCAGAGTAGGCAATATCTCATTAAATGAATTTTATAGAAGAACTATTCAAAGCGAA
TTTGAGAGGATCCTTGAAGATCCCCTATCAAATATGAAAAACTACAGATTTAGTAAACAATCAAACTATAGTACA
AAAGAAAAAAACACCGTTAAGTATAGGTGTTAACTGTCTTGATATTGATGATACTGGTCAAGTGTTATTAGGAGGT
GGAGATGATGGGTCACTTTCCATTTGGGGTTTAGATGAATCATTGCACCGCAATGATGAAGGTGAACAGGAATTG
ATCAATAAAAGGCTAAACTATATCAAGCGACAGCCTCACCAATCAGATGATGAGCCTGCTCAAATAATGGGTTAT
AAAAATAAAAGAACACGAATAAACGACAACAACACTATGAGACTGGTGCACAGTTTTCAAACACAGAGAAACAAA
TATCGAATGTATAGACAGTCTAGTGCTGCAGTTCCAGTTCAAAGATCACACATATCAAATAAAACGGATTCTCCT
ATCGGGTTTAGTGAGACACTATCTGAAACAGATTCAGAAGCTTCCATATCTCACCATAAATATGGTATAACAACT
TTGAAGTGGTATAAGGCTGATAATGGGATGTTTTTTACTGGCTCAAATGATAAAACTGTAAAAATATGGGACACA
AACAGGTTTGAAGCGGTTCAAGATATAAACCTGGGGTATAAAATAAATCAAATTGATAATAACGTTGTCGACGAC
AGTTCTTTGCTTGTAGTGGCAAGTGAAGACTACTATCCAAGATTAATTGACCTGAGAACCATGAATTCAGGGGTA
ACTGCGCTTGGAATGGGAAATCAAACACGAATGCAATCAGAAATTTTATGCTGCAAATTTAACCCCGTCAGGGAA
CAGATTATTGCTTGTGGTGACATGGAGGGTGGGGTAAAATTATGGGATCTAAGAATGAGGAACAGACTGTATTCA
GAATTGAAAAGAAATAAAAATAGGTTCAAGACTATAAATAACGATGATAATGACGATCAAAGCGATGTCTATTTC
AGCTCTAACCAATCAAAAGCCCATCTGAGATGTTGCAGTGACATTGTTTGGAATAGTGAGGGTTCAGAGCTATGT
TCAGTCGGCATGGATGGAAAACTGAATGTTTGGAGGCCATTTACGGAAATTCTGCAACCAGAAGGTCTAGCTAGT
TATTCCCAGTTGGGAACACAAGACCTGAGCCGTATCAAATACAAAAAAAGAGTATCTCGAAGGTTGCTTTGGTTT
GACAAGTTTTTACTGTGTATTACTGACAATGGTGAAGTAGAAATATACAATACGGAGGAAAAAAAAACTTTGGAAC
AAGCTCGAATACCCAATGGTTAATCAGGTAAAAAAAAATCAGGCTTCGCATTGTCAGTTTAGTTCCATGATTGTG
CAAACAAATATAATGAATTCAGTTGGCCTCAAATTGTTCTTTGGAACTAACAACAACACCGTTAGCGACGGCGGA
TCCATCTTTGAATGTTCATAA

>YKR100C 0.79 Cold
ATGACTGCCAGCACATCAGTTGCGGTGGGTTGTGCGGTGGGTATACCCGTCGGTGTAGGTATTATAATAGCAGTA
TGTTTCTGGTTTAACCTACAAAAAAGATACAAGAGGGAGGAACAAGACGATAGAGAATTGGAAAGGGCTATTTAT
GATGAAAGTGGTTTCGTGTCCTTTGACAATTTTGGACCCTTAAGAGACAGTAAAGATGAAGCAGCACTAGCCAGC
TCCGAGCTAAAAAATCCTGATCACACTTCTGGTTCATCGGAAGGATCTGCTCATCCAGAAGAAAAAGATGGAAAA
TCGCGAGATCAAGAGAAACCGTTAGGGAAAAAGAATAGTAAATACTATGTGCCAGCATACCGTAGGAAAATAAAT
TTACTTCAAGTACGAAATAATAACTACGGCAATAATGCCAGGCAGAAGTCCGTTGTGGATTTACCAAGTATAAAT
AACAGCTCCAATGTTTCCTTATCATCTTCCCAACGGCATATAACAAAGAGACAGATCAGCGTTTATGATCAGATG
GTCCCTGTTATTTCTGATGAAGGACCAAACTTTTTCGCAGATCCTTCCAGTGATACTAATACTAGCAATGACCAA
AACAAAGCTTCGATGATAGAACTTAAACATAATACTCGCCAATCTAGTAATGAAAATCTGATAAGAAATTTGCAG
AATCAGGACTTTGGTTCGTATTACCCAAGGAGAGCTTCATCCTCGTTTTTAAATGGTAACATTTCAAACGCGTCC
TTCCATACAAGGAATTCCTCTATAACATCGGTAAATAAGCGCGATGCCCTAGAGGATGTGTTTGCAACACCCAAA
AGTGCGGCTCAATCACAGTTACCAAACACTTTTGATAAAGATAACGAAGGAATGGATGCTGACCACTCGGTAAAG
GATAGCAGGTCTGCAATAACAGATAAAGATAAGGATTTATACAAATTACAGAATAACTATGATGTGGGCAATATC
GGGGAAATTGCTGAGGAAGATCAATACGAAAACGAATTTACCAACTACTCACAGAGTAAGAGAGAGTTCATAGAA
AGTCTAAGGCCTAAATAG

>YKR001C 0.69 Cold
ATGGATGAGCATTTAATTTCTACTATTAACAAGCTTCAGGACGCTTTGGCGCCCTTAGGAGGAGGATCTCAATCT
CCTATTGATTTACCACAGATCACTGTTGTCGGTTCCCAGTCGTCAGGAAAGTCGTCCGTTTTGGAGAACATTGTT
GGTAGGGATTTCTTGCCAAGAGGTACTGGTATTGTCACCAGGAGACCTTTAGTGTTACAATTGATTAATAGGAGA
CCAAAAAAAGTCAGAACATGCTAAAGTAAACCAAACTGCTAATGAATTGATTGACTTGAACATCAACGATGATGAC
AAGAAAAAAGGATGAATCAGGAAAGCACCAGAACGAGGGACAATCTGAAGACAATAAAGAGGAATGGGGTGAATTT
TTGCATTTACCTGGTAAGAAGTTTTATAATTTTGACGAAATTAGAAAGGAAATCGTCAAAGAAACTGACAAAGTG
ACAGGTGCCAATTCAGGTATTTCTTCTGTGCCCATTAACTTGAGAATTTATTCTCCGCATGTTCTTACTTTGACG
TTAGTGGATTTGCCTGGGTTGACGAAGGTTCCCGTAGGTGACCAACCTCCTGATATTGAAAGACAAATTAAGGAC
ATGTTGTTAAAGTATATTTCGAAACCAAACGCTATCATATTATCTGTTAATGCCGCTAACACCGATTTAGCCAAC
AGCGATGGTTTGAAGCTGGCTAGAGAGGTCGATCCAGAAGGAACGAGAACTATTGGTGTCTTGACAAAAGTCGAT
TTGATGGATCAAGGTACAGATGTCATAGATATTTTGGCTGGAAGAGTCATTCCTTTGAGATATGGTTATATCCCA
GTTATCAATAGAGGTCAAAAGGATATTGAACACAAAAAAACAATCAGAGAAGCCCTTGAAAACGAAAGAAAATTT
TTTGAGAACCATCCCTCTTACAGTTCTAAAGCTCATTACTGTGGTACACCATATTTGGCTAAAAAGTTAAACTCA
ATCTTATTACACCACATTAGGCAAACTCTGCCAGAAATCAAAGCGAAAATCGAAGCCACATTGAAAAAATATCAA
AACGAACTTATAAACTTGGGCCCAGAAACTATGGATTCAGCTAGTTCGGTTGTTTTGAGCATGATTACTGATTTT
TCCAATGAATATGCCGGTATCTTGGACGGTGAGGCGAAGGAGCTTTCCAGTCAGGAACTTTCTGGTGGTGCTAGA
ATTTCTTACGTATTCCATGAAACTTTCAAAAATGGTGTAGACTCTTTGGATCCATTCGACCAGATCAAAGATTCT
GATATCAGAACCATTATGTACAATAGTTCAGGTTCTGCCCCATCTTTGTTTGTCGGTACCGAAGCTTTTGAAGTT
TTAGTTAAACAGCAAATTAGAAGATTTGAAGAACCATCTCTACGTTTAGTTACTCTGGTGTTTGATGAACTTGTT
CGTATGCTAAAACAGATTATTTCACAACCAAAGTACTCAAGGTATCCTGCTCTAAGAGAAGCGATTTCTAATCAG
TTCATTCAGTTCTTAAAGGATGCTACTATTCCTACGAATGAGTTTGTTGTCGATATCATCAAAGCTGAACAAACT
TACATCAATACAGCCCATCCCGACCTTTTGAAGGGTTCTCAAGCAATGGTTATGGTGGAAGAAAAATTACATCCT
CGCCAAGTCGCTGTTGACCCAAAGACGGGTAAACCATTACCAACCCAACCATCGTCTAGTAAGGCGCCAGTTATG

GAAGAGAAATCAGGATTTTTTGGTGGGTTCTTCTCCACTAAAAACAAGAAGAAATTGGCAGCTTTGGAATCCCCA
CCTCCTGTTTTAAAAGCTACTGGCCAAATGACAGAGAGGGAAACAATGGAAACAGAAGTAATCAAGTTGTTGATT
AGTAGTTATTTCTCTATTGTCAAAAGAACCATTGCCGATATTATACCAAAGGCTTTGATGCTTAAATTGATTGTG
AAAAGTAAAACTGATATTCAGAAAGTTTTACTCGAAAAACTTTACGGAAAGCAAGATATTGAAGAATTAACGAAA
GAAAACGACATAACCATTCAAAGAAGAAAAGAATGTAAGAAGATGGTCGAGATATTGAGAAACGCTAGTCAAATC
GTCTCCTCTGTTTAG

>YLR397C 0.75 Cold
ATGGCTCCTAAATCTAGTTCTTCCGGTTCCAAAAAGAAATCATCGGCAAGTTCTAATAGTGCTGATGCAAAAGCA
TCCAAATTTAAATTGCCTGCTGAATTTATTACCAGACCACATCCTTCTAAAGATCATGGCAAGGAAACATGCACA
GCATATATTCATCCTAACGTATTATCCTCGCTTGAGATAAATCCGGGATCATTTTGTACTGTCGGTAAGATAGGC
GAAAATGGTATTTTAGTAATAGCTAGAGCGGGTGATGAAGAAGTACATCCTGTTAATGTTATCACCCTTTCCACA
ACTATACGATCTGTTGGGAACCTTATCCTTGGTGATCGTCTAGAATTAAAGAAAGCCCAGGTGCAACCACCTTAT
GCCACTAAGGTTACCGTGGGGTCCTTACAAGGATATAATATTTTGGAATGTATGGAGGAAAAAGTAATTCAAAAG
CTACTGGATGATAGTGGCGTTATAATGCCTGGAATGATTTTTCAAAACTTAAAAACAAAAGCAGGTGATGAAAGC
ATTGATGTCGTAATTACAGATGCGAGCGATGATTCGCTTCCCGACGTCAGCCAACTAGATCTTAACATGGACGAT
ATGTACGGTGGATTAGATAACCTGTTTTATCTATCTCCACCTTTTATATTCAGAAAAGGCTCCACACATATAACT
TTTTCGAAAGAAACCCAGGCAAATCGTAAATACAATCTTCCGGAGCCCTTATCCTATGCAGCAGTGGGCGGCTTA
GACAAGGAGATTGAATCACTGAAAAGTGCTATTGAAATACCTCTTCATCAACCGACGCTATTTAGTAGCTTTGGT
GTTTCTCCCCCTCGAGGTATACTTCTTCACGGACCCCCAGGTACTGGTAAAACTATGCTTTTGAGAGTTGTAGCA
AATACGTCCAACGCACACGTCCTAACCATTAATGGCCCCTCAATCGTCTCCAAATATCTTGGTGAAACGGAAGCG
GCATTAAGAGATATTTTTAATGAAGCAAGGAAGTACCAGCCTTCCATTATTTTCATTGACGAAATTGATTCAATA
GCACCAAATAGAGCAAACGATGACTCCGGTGAAGTTGAGAGCAGAGTCGTGGCTACATTGCTTACCCTAATGGAT
GGCATGGGCGCTGCAGGTAAAGTGGTGGTAATTGCTGCTACAAACAGGCCTAATTCTGTCGACCCTGCTCTCAGG
AGACCTGGCAGGTTTGACCAAGAAGTAGAAATTGGTATACCAGACGTTGATGCCAGATTTGACATTTTAACTAAG
CAATTCTCAAGAATGTCCTCGGATCGTCACGTATTAGATTCTGAAGCGATCAAGTACATTGCTTCTAAAACGCAT
GGCTATGTTGGTGCTGATTTAACTGCTCTCTGCAGAGAATCAGTTATGAAGACGATACAACGAGGACTAGGAACA
GACGCCAATATTGACAAGTTTTCCCTAAAAGTTACATTGAAAGATGTGGAGAGCGCCATGGTTGATATCAGACCC
AGCGCAATGAGAGAAATCTTCTTAGAAATGCCAAAAGTTTATTGGTCTGACATTGGCGGCCAAGAAGAGCTTAAA
ACAAAGATGAAAGAAATGATACAGTTGCCTTTGGAGGCTTCGGAGACTTTTGCCAGGCTGGGAATTTCTGCACCA
AAAGGTGTATTACTTTACGGGCCGCCAGGTTGCTCCAAGACATTAACCGCAAAAGCTCTCGCTACAGAATCGGGT
ATCAACTTCTTAGCTGTGAAAGGGCCTGAAATTTTTAACAAGTATGTAGGGGAATCCGAAAGAGCTATAAGAGAA
ATTTTCCGCAAAGCACGCTCTGCAGCTCCAAGTATTATCTTCTTTGATGAAATCGATGCATTATCTCCTGATAGA
GACGGGAGTTCCACCTCTGCAGCTAATCACGTGCTCACATCTTTACTCAATGAGATTGATGGTGTTGAAGAGTTA
AAGGGTGTAGTTATTGTAGCGGCGACGAATAGACCTGATGAAATAGATGCTGCTCTTCTAAGGCCTGGTAGGTTA
GATAGACACATTTACGTTGGCCCTCCAGACGTAAACGCCCGCTTGGAAATCTTAAAGAAGTGCACAAAGAAATTT
AATACAGAAGAGTCTGGAGTCGATCTTCATGAATTGGCAGACCGTACAGAAGGTTATTCCGGAGCTGAAGTTGTG
CTGCTTTGTCAAGAAGCGGGCTTGGCTGCCATAATGGAAGATTTAGATGTCGCAAAAGTGGAATTACGTCATTTT
GAGAAAGCTTTTAAAGGAATTGCTAGGGGCATTACTCCAGAAATGCTCTCTTATTATGAAGAGTTTGCTCTAAGA
AGCGGTTCATCTTCGTAA

>YAR061W 0.78 Cold
ATGCCTTATCACTATTTATTTTTGGCACTCTTCACCTACCTGGCCACGTCCAATGTTGTTTCAGGAAGTACACAA
GCATGCCTGCCAGTGGGCCCGAGGAAAAATGGGATGAATGTCAACTTTTATAAATACTCATTACTGGATTCAACA
ACGTATTCCTACCCGCAATATATGACTTCTGGATATGCCTCGAATTGGAATTAG

>YNL222W 0.76 Cold
ATGCCTAGTCATCGCAATTCAAACTTGAAGTTTTGCACAGTTTGTGCATCAAACAACAATCGTTCAATGGAATCG
CATAAAGTCCTGCAAGAAGCAGGCTATAATGTTAGCTCTTACGGAACAGGTTCAGCTGTGAGACTGCCTGGTCTA
TCGATAGATAAGCCTAATGTGTACTCATTTGGTACACCCTATAATGATATATATAATGATCTTTTATCACAATCA
GCAGACCGTTACAAGTCGAACGGTTTATTGCAAATGCTGGATCGTAATAGAAGACTCAAAAAAGCACCTGAAAAA
TGGCAAGAAAGTACAAAAGTCTTCGACTTCGTTTTCACTTGTGAAGAGAGATGTTTTGATGCCGTTTGTGAAGAT
TTGATGAATAGAGGTGGGAAATTAAACAAAATAGTGCATGTAATTAATGTTGACATTAAAGATGATGATGAAAAT
GCTAAAATTGGTAGCAAAGCTATATTGGAATTAGCTGATATGCTCAATGATAAAATAGAACAATGTGAAAAAGAT
GACATTCCCTTTGAAGATTGTATAATGGACATTTTAACTGAGTGGCAAAGCTCACATTCTCAACTACCGTCATTA
TACGCTCCTTCATATTACTAA

>YGR008C 0.73 Cold
ATGACGAGAACAAACAAGTGGACCGAACGTGAAGGAAAGGCTGATCCAAAGTACTTTTCGCACACTGGTAACTAC
GGTGAATCTCCAAATCACATCAAGAAGCAAGGTTCCGGCAAGGGTAATTGGGGTAAGCCAGGCGATGAGATTGAT
GACTTAATTGATAATGGTGAAATACCCCCAGTGTTCAAGAAAGATAGAAGAGGCTCAAATTTGCAATCGCATGAA
CAAAAGTTTGAAAACGTCCAAAAGGAATGA

>YOR196C 0.73 Cold
ATGTATAGACGATCTGTTGGAGTACTATTTGTTGGGAGAAATACAAGATGGATTTCGTCCACTATTAGGTGTGGA
ACGAGCGCAACTCGGCCAATAAGATCCAATGCATTGAATACTGATTCAGATAACGCTAGCGTTAGGGTACCAGTT

GGAAATTCAACTGAGGTTGAAAATGCAACCAGTCAGTTAACAGGGACTTCGGGAAAAAGACGCAAGGGGAACAGA
AAAAGAATTACAGAGTTCAAAGATGCTCTTAATTTGGGTCCATCTTTTGCAGATTTTGTTTCAGGTAAAGCTTCG
AAAATGATCTTGGACCCTCTGGAGAAGGCAAGGCAAAATACAGAGGAAGCTAAAAAACTACCTCGTTGGCTTAAG
GTTCCTATTCCTAAGGGTACCAATTACCATAAATTGAAAGGCGACGTAAAAGAATTAGGCCTGAGTACTGTTTGT
GAGGAGGCAAGATGTCCCAATATTGGTGAATGTTGGGGAGGCAAAGATAAATCTAAGGCAACGGCAACAATTATG
CTGCTTGGTGATACTTGCACTCGTGGATGTAGGTTTTGTTCTGTGAAGACCAATAGAACGCCTAGTAAGCCGGAC
CCAATGGAGCCCGAAAATACTGCCGAAGCTATCAAAAGATGGGGGTTGGGTTATGTTGTTTTAACTACCGTTGAT
AGGGACGATTTAGTCGATGGTGGTGCTAATCACCTGGCCGAGACGGTTCGCAAAATCAAACAGAAGGCACCAAAT
ACTCTTGTAGAGACTCTTTCTGGTGATTTCAGAGGTGATTTGAAGATGGTGGACATTATGGCACAATGTGGGCTT
GATGTTTACGCACATAATTTGGAAACAGTTGAATCACTAACACCACATGTCAGAGACAGAAGAGCTACTTATAGA
CAGTCTTTGAGTGTTTTAGAAAGGGCAAAAGCTACGGTTCCGTCACTGATTACTAAAACATCAATAATGCTGGGT
CTAGGAGAAACTGATGAGCAAATTACTCAAACTTTGAAGGATTTGCGCAATATTCAATGTGATGTTGTTACATTT
GGTCAATATATGAGGCCAACCAAGAGACATATGAAAGTCGTAGAATATGTGAAACCCGAAAAGTTTGACTACTGG
AAAGAGAGAGCTTTAGAGATGGGATTCTTGTATTGTGCATCTGGACCCCTAGTAAGATCGTCATATAAGGCTGGT
GAAGCATTTATTGAGAACGTTTTGAAGAAAAGAAACATGAAATAA

>YLR047C 0.81   Cold
ATGAATTTAAAGTCCATCGTTAGCTGGTTCAAGGAACATCTTCCCAGTTTTGATGTCGACGTGGACAAGCATTTT
AGAACTTTAAGAGTCCGCAAATATTCTCAGATATGTCTTTTAATTTCTTTCATTATAATATGTGTTATTATACCC
TTAATGAATTATTTGTTGCTAACTGACAAATTCTTCAAAATATGTCACCATTTAAAGCATCATGTTTTCAACAGA
AGATCATGGGTTCACAAAACACATATGTATCACAAACAGTCACTACAACTATGTTTAATATGCTTTGTTTTCACC
TCGTTTTTTGTTATACAAGGCGCTAACGGAGATCTGTTAGAAATTACTAAGCGAATGGGGAGAATTTCCGTTGCT
TTGATGCCACCTTTACTATTTCTAACGTTGAGGCCATCACCGTTACCTCACACGTTATACTTAGCATTGTTACCT
CTCCACAAATGGATTTCCAGAATTGTTGTGCTTGAATCTATTCTACACACGTGGTTCTATCTCTATTACATGTAC
ATTAATGATACGTTATACGTGAAAATGAGAAAACTACCCAACATTTATGGGGTTATTGCCCTCGGCCTTTTCCTA
CTGATTGCCATCACCTCTGTGAGATATGCCAGACGATGGAGCTACAGAGTTTTTTACTATGTTCACTATGTAAGC
ACATGGCTTATTTTGGTGTTTCTACATTATCACGCTCGCCCAGGAATTTCATATTATACCACTTTAAATGTGCTC
ATATTAACGGGACAAATCGTCTATAGGCTCCATATCACCAATGTTACGAGAGTTACGATAGTACCCATTTCTTCA
TCATTGTCTCTTTTGGAGTTCCCATTAACCGATTTACCCAAAAAGCCCATCCTACCAGGAGGGCATCTAAGGATC
AACATTTACCATAGAAATTTTTTAAGGAGGTTTTTCTCCCATTTGATACCCTTCCAACATCCTTTTACTATTGCT
AGTATTCCAAGTGATAATTTGGTCAGGTTAATTATTAGAAACGGGCATTTTCCATTGCGTACAAATGAAAAGTAT
TACATTACAGGGGCTTTTGAACCTGAGTTGAGTTTCATTTCTAAACCTACTGTTCCTTTTAATATTACCACGAAA
TCTTCCAAGAATCCATTTCGAAACAATTCTTCTGCACTAATAAATTCGCCTTTAAATTTTCTAATTAAAGCGCAA
AGAGTGTTTATGTGTGTTGGAGGTTCAGGAATTTCCTTTGGCTTACCACTACTTCGTATTCTAAACTTCAACGGT
GTAAACGTTAGGCTCCTTTGGGTTTCTAGAGATTATAAGGATCTGGAAGTATTGAACCACTTCAAAAACAATTTT
GAAGGTATGGAGATATATATTAGTGGCACCGAAGGCAACGAACAGGACATAGAAATTGATTATATTGACTATCAT
GACTGCGCTGCTGATATTAATGATGAAGTCAGGAGTATTTCTTCGAGCGGCCGAGTTTCAGAACTGGGAGATAAT
TCCATGTTATCAGATGGAAATCCCCAACCTACGGAGCCCAATGAGAACACAGCCCTTCTAAGTAAGAAATCTACC
TTGAGAAACCATCACCCACCAAAAACAAGTGATATACCTGACATCAATGCTGATGACGAGATAGATTTTACATAT
GCTTTTAGCAGATCCAAATCAAGGAAAAATACTGCACAAGGAACTCTAACTACACATTCTTCGTTTAACGGATCG
AGCGTTTTCAGACAACCAAAGATCATTGAGCCACCTGCCCAAGATCCTTGTTTAGAGGCGGCGCCTAAAAAGATC
AGAATTCCCGCAGGTGTTAAAGTGTTCTTTGGCAGGCCTACACTTGGAGATAAGGATTATGAATGGTGTCTGCAA
ACCGAATGTGATGCAGAGACGGATTCTATTCAATGTTGCAGGTGGGCGAATCAAGGCAGAGACCATGCTGAATAT
TTGTCACAAGTATGGGTTCTCGCTGCAGGCCCTAGAGGCTTAATTGAAAGCACCAAAAGATGGGCGACAGATGGT
GGTTTACACTTTCACGGAGAAAGTTTTGCATTATAA

>YKL011C 0.78   Cold
ATGTCGACAGCACAGAAAGCTAAGATATTGCAACTCATCGATTCCTGCTGCCAAAATGCAAAAAGCACACAACTG
AAATCTTTATCATTTGTTATTGGAGCAGTAAATGGCACGACGAAAGAAGCTAAAAGAACCTACATTCAAGAACAG
TGTGAATTTTTGGAGAAGTTACGACAACAAAAGATAAGAGAGGGAAGAATTAACATATTGTCTATGGATGCTGGT
GTTTCTAACTTTGCTTTCTCTAAGATGCAATTGCTCAATAATGATCCGCTCCCTAAAGTACTAGACTGGCAAAAG
ATAAATCTAGAGGAGAAATTTTTTCAAAACCTCAAAAAGTTAAGCTTGAATCCTGCTGAAACTTCTGAGCTTGTA
TTTAACCTTACGGAGTATTTATTTGAATCTATGCCGATACCAGATATGTTTACAATTGAAAGGCAACGTACCAGA
ACTATGTCTTCGAGGCATATTTTAGACCCAATTTTAAAAGTGAATATTCTCGAACAGATTCTTTTCTCTAACTTG
GAAAATAAAATGAAGTATACGAATAAAATACCGAATACGTCCAAGTTGAGGTATATGGTATGTTCGTCCGATCCA
CATCGGATGACTTCATATTGGTGCATTCCAAGAGAAGAGACACCGACCAGTTCAAAAAAGTTAAAATCTAACAAA
CATAGCAAAGATTCTCGAATAAAGCTAGTGAAAAAAATACTTTCAACCTCAATACTAGAAGGTAATTCAACTAGT
TCTACAAAACTGGTCGAGTTCATAGGAGTTTGGAATAATAGGATAAGAAATGCCCTTACCAAAAAAAAAAGTTTC
AAGCTATGTGATATACTAGAGATCCAAGATAATTCGGGGGTGAGAAAAGATGACGATTTGGCAGATTCATTCCTC
CATTGTTTGTCTTGGATGGAGTGGTTAAAAAATTATGAAAGTATTACTGAACTCTTGAATTCAAAAACACTGGTT
AAAACACAGTTCGGACAGGTGTTTGAATTTTGTGAAAATAAGGTACAAAAGCTGAAATTTTTGCAGAACACTTAC
AACAATGACTAA

>YCL056C 0.81   Cold
ATGGTTTCGAAGAAAAATACGGCTGAAATCAGTGCAAAAGATATATGGGAGAATATATGGAGTGGTGTAAGTTCG
CTGCTTGATTTCTTCGCAGTATTGGAAAACCTAGGCGTGGTCAACGATAAGTTATATGTAAGTGGGCTATTACGG
AAAGTTTGGCTGTGTTACTCGTGCATTTCAGTGATAAAATGTGTATGGAAATTAATAAAATTATGCAAGGTGAAA

TTTAAAATCGATCAACGATTGGATGGGGAGGGAAATGGTCTAGTAAAGGATAAGTTGATAAACTTCAAGAAAAAA
TATAACGAACATATCAGACATATTACTGCTGCTTTACTCCAAGATTTGAGCTATTTAATGGTTTTAATCTATCCA
GGGACGAGGTTATTTAAAAGATTATCTAACATAATAACACTTTGTAGAATAATTGTATAA

>YDL028C 0.73   Cold
ATGTCAACAAACTCATTCCATGATTATGTGGATTTAAAATCGAGAACTAATACACGACAGTTTTCAGATGACGAA
GAGTTCACTACGCCTCCAAAACTAAGCAATTTCGGATCAGCTTTACTTTCCCACACAGAAAAAACTTCAGCATCA
GAGATATTATCAAGTCATAATAATGACAAGATCGCAAATCGATTAGAAGAAATGGACAGGAGTTCATCAAGGAGT
CACCCCCCACCGTCAATGGGCAATTTGACATCCGGTCATACTAGTACCTCATCGCATTCAACCTTGTTCGGACGA
TATCTGAGAAATAATCACCAGACTAGCATGACGACGATGAACACTAGTGACATAGAGATAAATGTTGGAAATAGT
CTTGATAAGAGTTTTGAAAGGATAAGGAATTTGCGACAAAATATGAAAGAAGATATTACGGCAAAGTATGCTGAA
AGGAGAAGTAAGAGATTTTTAATATCCAATAGGACAACGAAGCTGGGTCCTGCAAAGAGAGCGATGACTTTGACA
AATATCTTTGATGAGGATGTGCCTAACTCTCCAAACCAGCCAATAAATGCAAGGGAGACAGTGGAATTACCACTT
GAGGATTCTCACCAAACAAACTTTAAAGAACGAAGAGAGAATACGGATTATGATTCAATTGATTTTGGAGATTTG
AATCCTATCCAGTATATTAAAAAACATAATCTTCCCACAAGTGACCTTCCACTAATATCTCAAATCTACTTTGAT
AAACAAAGAGAAGAAAATAGACAAGCAGCACTCCGAAAACATAGTTCCAGAGAATTGCTTTATAAAGTAGGTCT
TCTTCCTCTTCACTTTCTAGTAACAACTTATTGGCAAACAAGGACAATTCTATAACATCCAATAATGGTTCTCAA
CCCAGGCGAAAAGTTTCTACTGGATCATCTTCATCTAAGTCATCGATCGAAATAAGAAGAGCTCTCAAGGAGAAT
ATTGATACTAGCAATAACAGCAATTTCAACAGCCCAATTCATAAAATTTATAAAGGAATTTCCAGAAATAAAGAT
TCCGACTCCGAAAAAAGAGAAGTACTGCGAAACATAAGCATAAATGCAAATCACGCTGATAATCTCCTTCAACAA
GAGAATAAGAGACTAAAACGATCATTGGATGATGCAATAACGAATGAGAATATAAACAGTAAAAATCTAGAAGTA
TTTTACCATCGACCTGCTCCCAAACCTCCAGTCACCAAGAAAGTTGAAATTGTTGAACCTGCAAAGTCCGCTTCT
TTATCGAATAATAGAAATATAATTACAGTAAATGACTCCCAGTACGAAAAAATAGAACTTTTGGGTAGAGGTGGA
TCCTCCAGAGTTTACAAGGTGAAAGGATCTGGCAATAGGGTATACGCGCTCAAAAGGGTGTCTTTTGACGCTTTT
GACGATTCAAGTATTGATGGATTCAAAGGAGAAATAGAACTTCTGGAAAAATTGAAAGACCAAAAGCGTGTAATC
CAACTACTAGATTATGAAATGGGGGATGGTTTATTGTATTTGATAATGGAATGTGGTGATCATGATTTGTCACAA
ATCCTTAACCAAAGAAGCGGCATGCCACTGGATTTTAATTTTGTTAGATTCTATACAAAGGAAATGTTGCTGTGC
ATTAAAGTAGTTCATGATGCGGGCATAGTTCATTCGGATTTAAAACCTGCAAATTTTGTTTTAGTGAAAGGTATC
TTAAAAATCATTGATTTTGGTATAGCAAACGCGGTACCGGAACATACGGTGAATATATATCGTGAAACTCAAATT
GGGACTCCAAATTATATGGCACCAGAAGCACTAGTTGCTATGAATTACACACAAAATAGTGAGAACCAACATGAG
GGAAACAAGTGGAAAGTGGGGAGACCATCTGATATGTGGTCATGCGGTTGTATTATATATCAGATGATTTACGGG
AAACCCCCATATGGCAGTTTCCAAGGCCAAAATAGGCTGTTGGCTATTATGAATCCTGATGTGAAAATCCCATTT
CCTGAACATACTAGCAATAATGAAAAGATTCCAAAGTCTGCCATTGAATTAATGAAAGCATGTCTGTACAGGAAC
CCAGACAAAAGATGGACTGTGGATAAAGTCCTGAGTAGCACTTTCCTTCAACCTTTTATGATATCCGGATCGATT
ATGGAAGACCTTATTAGGAATGCCGTTAGATATGGCTCTGAGAAGCCTCATATATCACAAGATGATCTCAATGAT
GTGGTAGACACTGTTTTAAGGAAATTTGCAGATTACAAAATTTAG

>YDL178W 0.8Cold
ATGCTAAGAAACATTTTGGTGAGAAGTACTGGCAGCAATTTCAAATTTGCTGGCAGATATATGAAATCATCAGCT
CTTTTAGGATACTATAGAAGAGTTAACTATTATTCGACCAAGATACAAACCAGACTGACTAGCGAAAACTATCCA
GACGTGCATCGAGACCCTAGATTCAAAAAATTAACGTCCGATGATCTAAATTATTTTAAATCTATTTTATCGGAA
CAAGAAATATTAAGAGCCAGCGAATCAGAGGATCTTTCATTTTATAATGAAGATTGGATGAGAAAGTACAAAGGA
CAGTCCAAGTTAGTATTGAGACCTAAGTCAGTGGAAAAAGTTTCTTTAATCTTAAATTATTGTAATGATGAAAAA
ATTGCCGTTGTCCCACAAGGCGGTAACACGGGGTTGGTAGGTGGTTCTGTGCCCATTTTTGATGAATTAATTCTA
TCTTTAGCAAATTTAAACAAAATAAGAGATTTTGACCCTGTATCAGGTATCTTGAAGTGTGATGCTGGTGTAATC
TTGGAAAATGCTAACAATTACGTAATGGAACAAAATTATATGTTTCCGTTGGATCTGGGAGCTAAAGGTTCCTGC
CATGTTGGTGGCGTGGTTGCAACCAACGCTGGAGGACTAAGGTTATTGCGTTATGGCTCACTACATGGAAGCGTT
TTAGGCTTAGAGGTGGTAATGCCCAATGGTCAAATTGTTAATAGCATGCATTCCATGAGAAAAGACAACACCGGT
TATGATCTGAAACAGCTGTTCATTGGCTCAGAAGGTACTATCGGTATCATCACTGGTGTTTCAATCTTGACTGTT
CCTAAGCCAAAAGCGTTTAATGTATCTTACTTGTCTGTTGAAAGTTTTGAAGACGTTCAAAAAGTCTTCGTCAGA
GCCAGGCAAGAATTATCTGAGATTTTATCCGCTTTTGAATTTATGGATGCTAAATCTCAAGTATTGGCCAAGAGC
CAATTGAAGGATGCTGCCTTCCCTTTGGAGGATGAGCATCCATTTTATATTCTTATCGAAACGTCAGGGTCAAAC
AAAGATCACGATGATTCCAAACTTGAAACATTTTTAGAAAACGTCATGGAAGAGGGCATAGTAACGGATGGTGTA
GTGGCACAAGATGAAACCGAACTCCAAAACTTGTGGAAGTGGAGAGAAATGATTCCAGAGGCAAGTCAAGCTAAT
GGTGGTGTTTACAAATACGATGTTTCTTTACCACTAAAGGACCTATATTCTTTGGTTGAAGCCACTAATGCAAGA
CTTTCTGAAGCTGAATTAGTGGGTGATTCGCCCAAACCAGTGGTGGGCGCCATTGGATACGGTCACGTGGGTGAT
GGTAATCTACACTTAAATGTTGCAGTTAGAGAGTACAACAAGAACATTGAAAAGACTTTGGAACCATTTGTCTAC
GAGTTTGTTTCCTCAAAACATGGTTCCGTTAGTGCCGAACATGGGCTAGGTTTCCAAAAGAAAAATTACATTGGC
TATTCTAAGAGCCCGGAAGAGGTCAAGATGATGAAGGATTTGAAGGTTCATTATGATCCTAATGGAATTTTAAAC
CCTTACAAATACATTTGA

>YBR274W 0.82   Cold
ATGAGTCTCTCGCAGGTGTCACCTTTACCCCATATCAAGGATGTTGTTTTAGGAGATACAGTAGGCCAAGGCGCA
TTCGCCTGCGTTAAAAATGCTCATCTTCAAATGGATCCCTCCATTATTCTAGCTGTTAAATTCATTCATGTTCCC
ACTTGCAAAAAAATGGGACTCAGTGACAAGGATATCACAAAAGAGGTTGTTTTGCAATCGAAGTGTTCTAAGCAT
CCTAATGTTTTGAGACTTATCGATTGTAATGTCTCTAAAGAATATATGTGGATAATTCTGGAGATGGCAGATGGT
GGTGATCTATTTGATAAGATTGAGCCTGACGTTGGAGTTGATTCCGACGTGGCCCAATTTTACTTTCAACAGCTC

GTTAGTGCTATTAATTATCTGCATGTAGAATGTGGAGTTGCTCACAGAGACATCAAGCCTGAAAACATCTTACTC
GATAAGAACGGAAATCTGAAGCTAGCTGATTTTGGGCTCGCCTCTCAATTTAGGAGGAAAGATGGTACATTACGT
GTATCCATGGATCAAAGGGGTTCTCCACCCTACATGGCTCCTGAAGTATTATATTCTGAAGAGGGTTATTACGCA
GATCGAACAGATATATGGTCTATTGGCATCCTCTTGTTCGTATTGTTGACTGGTCAAACGCCTTGGGAATTACCT
TCATTAGAGAACGAAGATTTCGTCTTTTTTATTGAAAATGATGGAAATTTAAACTGGGGACCCTGGTCAAAGATA
GAATTTACTCACTTGAACCTACTCCGAAAAATTTTACAACCTGACCCAAATAAGAGGGTGACATTGAAGGCTTTA
AAGTTACATCCTTGGGTATTACGTCGAGCTTCATTTTCTGGGGATGATGGTCTCTGTAATGACCCTGAACTCTTG
GCTAAGAAACTGTTTTCTCACTTAAAAGTCTCACTGAGTAACGAAAATTATTTGAAATTCACTCAAGATACAAAC
TCTAATAATAGGTACATTTCTACTCAGCCAATTGGTAACGAATTGGCTGAGCTTGAACACGACTCAATGCATTTC
CAGACAGTTTCGAATACACAACGTGCATTTACCTCGTATGATTCAAATACGAACTATAATAGCGGAACAGGTATG
ACACAAGAGGCTAAGTGGACGCAATTCATAAGCTATGATATCGCTGCCTTACAGTTTCATTCTGATGAAAATGAT
TGTAATGAATTAGTAAAACGACATTTACAATTTAATCCGAATAAACTTACCAAGTTCTACACGTTGCAACCTATG
GATGTTTTGTTACCGATTTTGGAGAAAGCCTTGAATTTATCACAAATTAGAGTAAAACCCGACCTTTTTGCGAAT
TTTGAAAGATTGTGCGAATTATTGGGTTATGATAACGTTTTCCCACTTATTATAAATATTAAAACCAAAAGTAAT
GGGGGTTATCAATTATGCGGTAGCATTTCCATCATAAAGATTGAAGAAGAGTTGAAAAGTGTCGGATTTGAAAGA
AAAACTGGTGATCCTTTAGAATGGAGAAGATTGTTCAAGAAAATTTCAACTATCTGTAGGGATATTATCCTAATT
CCCAACTGA

>YNL258C 0.8Cold
ATGGAGTCTCTTTTTCCTAACAAAGGTGAAATAATACGCGAGTTACTGAAGGATCCTTTAATATTAAAAAATGAC
AGCAAAAGGTCCAATGGCAGTGAGTTAGAACTTGATTCTAGTGACCTACTACAACGCGAGGCAATTCTAGCCAAC
GAACTGAACATCCTAGATAATTTGAAGACGTTTTTAAATCTCATAAAAGAAGTTAAAACTAATTTGAACATACTA
GAACTAGAAAACTGCTACTATTCGTTGCAGTCATTGCGAAAAAAGATGCGTAATAATGCTGCTTATCTTAAGCAA
AGCTTTAATTTCCAGCAGTCCATTTCTACCTACGTCGACACACTGCATTTAGAACTTGTCAGCACGTTGTATAAA
ATTCTTACTAATGGATTTTGGAAGATTACCGAGAATAGCATCCAATTTACGCCCACAGTGGAATGGGGTAAAGAC
AAGGTTCATATTGAGTATGATACATTTATGGATTTTGTGGCCCAACAGTATTTTCCTAAGGGTAGTTTGGACAAT
CAAGCTTGGTTCATTTTAGATATGACCAGTGCCGACTCTCAAGAGCAGGTAAGAGCAAAATTGAATACAATCATG
AAGGAGTATATGAACCTTTCCAGGATTGTAAGCATGATCAAAAATTCTATTTTTATATCTGGTAAAGAAATCTCA
TATGAAAACGAAAAAAACATACTTGTCTTTTCTAAATCCTCTTCGCATGGCCAACATTGTGTGTCTACTGTATTA
ACAAGTTTTGAAGCAGTATGCGATTTCATGTTAGACGGATTAGCCTTTAGAGACAGAAAGACCTTAAGTTATGAG
CTAGGTCCCTTGTTCAATACGGAGTTTACCAAATTTGTGAAAAATAATGCATCGATAATTTTAGAATCACTTGAT
TCACCGCTAAAAAATCTAGTTTCTGTCATAAATAACAAACTAACTAGACTAGTTGCCAAATCAGAAGTTACCAAT
TGGACACATTCCGGTAAAGAGATTCAGGATTTGTTAATGAACAAACAATTATACTACAATTTATTACTAGATAAA
GTTCTTGAATCGCATATTAGTGAGATAAGAAGTATATTTGAAGATCCAAAAAAGAGCTGGCAAAATTTGGAAGTA
GTTGAGCTCACTACTAGCAATACTAATACGATGAGCGAAAAAATTGGAAAAAATGATTCAGATGTACAGAACGAA
AAAGAACTCCATAATGCCGTATCCAAAGATGATGATTGGAATTGGGAAGTAGAGGATGATGATGCCGATGCATGG
GGTGATGAAATTGATGTAAATATTGACGACGAGGAAGAAAAAACAAATCAAGAGAAGGAAAAAGAACCAGAAGAA
GAAGAAAATGCCTGGGATGAAGCCTGGGCAATTGACGAAAATATAGATGATGCTTCCCTAGAAAATGGAAAGGAA
CATTTAAAAGCTCATGATGTAGGATCACTTGATAAAGACCACATTGAAGTTACACAGTTACCAAAATTGTTCTTG
GCAATTTCACAGAATTTCAAGAGTAGCTTTGCAGATAGCCATGTTGATGAGCAATACTTTGCGTACAAATACAAC
CTTTTGCAGACATCGTATATGGCCATGTGCACAGCTAATTTTTCTCACAATTGGTGCCAATTATATGTGGATATG
AGATATCTAATAGAACGAGATGAAAAGCTGTATAGAATAAAAGAATTAACAAGGAACCTGCTGGAAACTAAACTG
AACATGAAATATCGTATTGTCTGTCAACTAATCAGGCATCAATTGACCGAATTTCGTGAGAACGAGAGAAATCCA
TCGTGGGACGCCACCATTGAGAAACTGCTACCTTATATCTTGAAGGAAATTGTTCGTCCACTGCAGAAAATAAGA
GGTGAAGAAGGTTCCCGTTACTTGTTGTCTTTCTTAAACTTTTTATATAACGATTGCGTGACAAAGGAAATATTA
AAATGGCAGATTATCTCTGAGGTCAATTCCGAAAATTTGGGTGAGCTCGTATCTTTATTAGTGAACAACACCGAT
ATACAATTATTAGCGAAGGAACCAAGTTACAAAAAGATGAGAGAAAAATTCGCCACTATGGGCAAGTTTCTACCA
TTACATTTAAAGGAGATTATGGAGATGTTTTACAATGGGGATTTTTATCTTTTTGCGACAGACGAACTAATCCAA
TGGATAGAGTTATTGTTTGCCGACACTCCCCTGCGAAGGAATGCCATTGATGATATTTACGAAATTAGAGGCACT
GCTCTAGATGATTAA

>YJR158W 0.72 Cold
ATGGCAAGCGAACAGTCCTCACCAGAAATTAATGCAGATAATCTAAACAGTAGTGCAGCTGACGTTCATGTACAG
CCACCCGGAGAGAAAGAATGGTCAGACGGGTTTTATGACAAAGAAGTCATTAATGGAAATACGCCAGACGCACCG
AAGAGAGGCTTTTTAGGTTACCTTATTATCTACTTACTATGCTATCCTGTATCCTTTGGCGGTTTTTTACCTGGT
TGGGATAGTGGTATTACTGCAGGCTTCATCAATATGGATAACTTTAAAATGAATTTTGGTTCTTACAAGCACAGT
ACTGGTGAGTATTATTTGAGCAACGTGCGTATGGGTCTTCTCGTGGCCATGTTCAGTGTAGGATGTTCCATTGGC
GGTGTTGCTTTTGCGAGACTTGCTGATACTTTAGGTAGAAGGCTAGCAATTGTAATCGTGGTTTTGGTATATATG
GTTGGTGCAATTATTCAGATCAGTTCGAATCACAAATGGTACCAATACTTTGTCGGTAAGATCATCTACGGTCTT
GGTGCTGGTGGCTGTTCGGTGTTGTGTCCAATGCTTTTATCTGAAATAGCCCCCACAGATTTGAGAGGTGGACTT
GTCTCATTGTACCAACTTAACATGACCTTCGGTATTTTCTTGGGTTATTGTAGCGTTTATGGAACAAGGAAGTAT
AGTAATACTGCGCAATGGAGGATTCCTGTGGGACTATGCTTTCTGTGGGCTCTAATTATCATCGTTGGCATGTTA
TTAGTTCCAGAGTCCCCAAGATATCTGATTGAATGTGAGAGACATGAAGAGGCCTGTGTCTCCATCGCCAAGATC
GACAAGGTTTCACCAGAGGATCCATGGGTACTCAAACAGGCTGATGAAATCAACGCCGGTGTCCTTGCCCAAAGA
GAACTAGGGGAAGCCTCATGGAAAGAACTTTTCTCCGTCAAAACAAAAGTCCTTCAACGTTTGATCACAGGTATT
CTTGTGCAAACCTTTTTGCAACTTACTGGTGAAAACTACTTCTTCTTCTACGGAACTACCATTTTCAAATCAGTT
GGGCTTACTGATGGGTTTGAGACTTCGATCGTCCTAGGTACAGTGAATTTCTTCTCCACTATTATTGCTGTTATG

GTCGTAGACAAAATAGGCCGTCGTAAATGTCTGTTATTCGGAGCGGCTTCAATGATGGCTTGTATGGTCATATTT
GCAAGTATCGGGGTAAAATGTCTTTACCCTCATGGCCAGGATGGTCCATCCTCGAAAGGTGCAGGTAATGCCATG
ATTGTGTTCACATGCTTCTATATATTCTGCTTTGCAACGACATGGGCCCCTGTTGCTTATATTGTGGTTGCCGAG
TCATTCCCTTCGAAGGTCAAATCTAAAGCAATGTCAATTTCGACTGCATTCAACTGGTTATGGCAATTCTTGATT
GGTTTTTTCACACCATTCATTACTGGGTCTATCCACTTCTATTATGGTTATGTGTTCGTAGGTTGTTTGGTTGCT
ATGTTTTTGTACGTTTTCTTCTTTTTACCAGAAACAATTGGTCTATCTTTGGAGGAAACCCAGTTACTATATGAA
GAAGGTATAAAACCATGGAAATCTGCATCTTGGGTACCACCCTCAAGGAGAGGAGCTTCTTCCAGGGAAACTGAG
GCTAAGAAGAAAAGCTGGAAAGAAGTTTTGAAGTTCCCAAAGAGTTTTAATTGA

>YOR094W 0.78   Cold
ATGGGCAATTCAATTTCGAAGGTTCTGGGAAAACTATTTGGCTCGAAAGAAATGAAAATTTTGATGCTAGGCCTG
GATAAGGCTGGTAAGACAACAATATTGTACAAACTAAAATTAAATAAAATAAAGACGTCTACTCCCACTGTCGGT
TTTAACGTGGAAACCGTTACTTATAAAAACGTAAAATTCAACATGTGGGATGTAGGAGGACAACAAAGATTGAGA
CCTCTCTGGAGACATTATTTTCCAGCCACCACCGCACTTATCTTTGTGATAGATTCTAGTGCTAGAAACCGTATG
GAAGAGGCCAAAGAAGAGTTATATAGTATCATAGGTGAGAAAGAGATGGAGAATGTAGTACTGCTGGTATGGGCA
AACAAACAAGATTTGAAAGATGCAATGAAACCTCAAGAGGTTTCCGATTTTTTAGAACTGGAGAAAAATTTGAAA
AACCAACCTTGGTGTGTCATCGGTAGTAACGCCTTATCCGGACAGGGTCTTGTTGAAGGATTATCCTGGATTTCG
AACAACACAAACGTTCCAAAGAAATAA

>YER025W 0.76   Cold
ATGAGTGACTTACAAGACCAAGAACCTAGCATTATTATCAACGGTAATTTGGAACCAGTTGGTGAACCAGATATC
GTTGAAGAAACGGAAGTTGTAGCTCAAGAAACACAAGAAACACAAGATGCTGATAAGCCAAAGAAGAAAGTCGCC
TTCACTGGCTTGGAAGAAGACGGGGAAACTGAAGAAGAAAAAAGGAAAAGGGAATTTGAAGAAGGTGGTGGTTTG
CCCGAACAACCATTAAACCCTGATTTTTCAAAATTAAACCCACTATCCGCCGAAATTATCAACAGGCAAGCTACA
ATAAACATCGGTACCATCGGTCATGTCGCCCACGGTAAATCCACAGTAGTTAGGGCTATTTCAGGTGTCCAAACA
GTCCGTTTTAAGGATGAATTAGAACGTAACATTACTATTAAGTTAGGTTACGCTAATGCTAAAATTTATAAATGT
CAAGAGCCTACATGTCCCGAACCTGATTGTTATAGATCATTCAAATCTGATAAAGAAATCAGTCCTAAATGTCAA
AGACCAGGTTGTCCGGGGCGTTACAAATTGGTCCGTCACGTTTCTTTTGTTGACTGTCCCGGTCACGATATCTTA
ATGAGTACTATGTTATCAGGTGCTGCTGTTATGGATGCTGCGTTACTGTTGATTGCAGGTAATGAATCTTGTCCA
CAACCTCAAACCTCAGAGCATTTAGCTGCCATTGAAATCATGAAATTAAAGCACGTCATCATCTTACAGAATAAG
GTCGATTTAATGCGTGAAGAAAGTGCCTTAGAACACCAAAAATCCATCTTGAAGTTCATCAGAGGTACCATTGCT
GACGGTGCTCCTATTGTACCAATATCCGCTCAGTTGAAGTATAACATCGACGCAGTTAATGAATTTATTGTCAAG
ACTATTCCCGTACCACCAAGAGATTTTATGATTTCTCCAAGGTTAATTGTTATTCGTTCATTTGATGTTAATAAG
CCAGGTGCTGAAATCGAGGACCTGAAAGGTGGTGTTGCCGGTGGTTCTATTTTGAATGGTGTTTTCAAATTAGGT
GATGAAATTGAGATTAGACCAGGTATTGTCACCAAGGATGATAAGGGTAAAATTCAATGTAAACCAATCTTTTCT
AACATTGTCTCCTTATTTGCCGAACAAAATGACTTGAAGTTTGCTGTTCCCGGTGGTCTGATTGGTGTTGGTACT
AAAGTTGATCCTACCTTGTGTAGAGCTGATCGTCTTGTCGGTCAAGTCGTCGGTGCTAAGGGTCATTTGCCAAAC
ATTTATACTGATATCGAAATCAACTACTTTTTGCTACGTCGTCTATTAGGTGTCAAAACAGATGGTCAAAAGCAA
GCCAAAGTCAGAAAATTAGAGCCAAATGAAGTTCTTATGGTCAACATTGGTTCTACCGCTACGGGGGCTCGTGTG
GTTGCCGTTAAAGCTGATATGGCAAGATTACAGTTAACGTCGCCCGCTTGTACTGAAATTAACGAGAAGATTGCT
TTGTCGAGACGTATCGAAAAGCATTGGCGTTTGATTGGTTGGGCAACCATTAAAAAGGGTACTACATTGGAACCC
ATCGCTTAA

>YJL180C 0.75   Cold
ATGCTGCCATCATTAAGGAAGGGATGCTTCATTGTGAATTCCATAAGATTGAAACTGCCCCGATTCTACTCATTA
AATGCCCAGCCACTGGGGACAGACAACACGATTGAGAATAACACTCCTACGGAAACAAATAGATTGAGCAAAACT
TCACAGAAGTTTTGGGAAAAGGTGTCACTAAATAGGGATGTTGAGAAAGGAAAGATTGCTCTACAATTAGATGGC
AGGACTATAAAAACTCCTCTAGGAAATGGAATTATAGTTGATAATGCAAAGTCTCTCTTAGCATACCTATTAAAA
CTGGAGTGGTCGTCCCTATCCAGTCTTTCCATCAAAACTCACTCTTTGCCACTAACTTCATTAGTGGCAAGATGC
ATAGATTTACAAATGACAAATGAGCCTGGCTGTGACCCTCAATTAGTTGCAAAGATTGGAGGCAACAGTGATGTT
ATAAAAAATCAGTTGTTAAGATATTTAGATACCGATACTTTATTGGTCTTTTCCCCTATGAATGAGTTTGAAGGA
AGATTACGCAATGCGCAAAATGAGTTATATATACCCATCATCAAAGGAATGGAAGAGTTTTTACGCAACTTTTCA
TCCGAGTCTAATATTCGACTACAAATTTTAGATGCCGACATCCATGGGTTACGAGGCAATCAGCAGTCGGATATC
GTTAAGAATGCAGCAAAAAAATATATGAGCAGCTTATCACCATGGGATCTTGCAATTCTTGAAAAAACTGTATTA
ACCACAAAGTCCTTCATTTGCGGCGTGCTATTATTAGAAAATAAAAAAGATACTGCGAACTTAATTCCCGCCTTG
AAAACTGATATGGATAATATTGTACGTGCCGCCACCTTAGAAACAATCTTCCAAGTTGAAAAGTGGGGAGAGGTT
GAAGATACTCATGACGTTGACAAAAGAGACATCAGAAGAAAAATTCATACTGCTGCGATTGCTGCTTTTAAGCAA
TAA

>YLR154C 0.78   Cold
ATGACCAAAGATGCCGTGAATCTAGATGCTTACACCGTGAGCTTCATGCCTTTCTATACCGAGTATCAAGGACCA
ACCGAAGAGTTTAAGGATTACAAATTCGAAGATACTATTTACTTTCGTGGCAAGGAACTGAAGAGGGAAAAGTCT
GCGACGCCTTCCAGTAGCGATAACACAACTAGTAATACCTTCAGTAATGGCGCCATCCTCTCGGGAAACACAATA
ACTGGCAAGATAGTTTCAGTGAATAATTACGAAAGAGAGGGCACTGATCGCAACGAATTGGCGCGATTGCAAGAA
TTGATCTCCCTCATCGATGTCATAAATCAGTAA

>YJL215C 0.78   Cold

ATGGTTAGCGATGATATTCCGTCGTCCAAAAGATTACGACCATGCCAGTTCATATTATTTACTTAAGAGTTCAT
GTCTTTCATGCGAAGAAGCGTTCGGAAATTCCAAAACTTCTTAGAGTTCAGGAACAGGCCCGGTCGCGGAATTAT
TCTCGGGATAATTCCAGCCGCTTGGCAGGTAGGCTAAATTACACATTTCAAGAACTTTCTCCGGCCTTTTCTGTG
AGATACGTTTATAAGTTGCTGCAGCAGGATGCTCTAGGCTGCTTTCTCTGGAATTTCTCTCCTTCTCCACATACC
TCGTTGTTGAATCCCCGCGGAGGTGATATAATAAAGTTGCGTGAAAGCGTAAATGCCTAG

>YDL117W 0.79 Cold
ATGGCCACTAACTTAACATCTTTGAAGCCACCATTTAAGGTGAAGGCCAGGTATGGTTGGTCGGGCCAAACTAAA
GGCGACTTAGGCTTTCTGGAGGGAGATATTATGGAAGTCACAAGGATTGCCGGTTCTTGGTTCTACGGCAAGTTA
TTGAGAAACAAAAAAATGTTCTGGGTATTTTCCTCATAACTTCGTCATACTATTAGAGGAGAGATTAAATTCGAGC
ACTGAAAATGGTAGGCAACCTTCAAAAATAGTAGAAAGTTTTGAAAAATCAAATAAAGTTGTTATTCCACCAGTA
CCATCAAGGTATTCTGACGAGAGGCCAAGGCCTAAAAAGAAGCTATCTTCATCAATGCCCAATTCACCCAAGAAG
CCGGTAGATTCTTTGACAAAGGCTAGAAAGGCAAAATCAAAAGAAATGGTAAATGAAAAAAATATCTATAATACC
CAATCCTCTCGACATCATAATAACTCAGCTCCAAATTTACCTTTGGCTAGCCATAGTAAGCCTCAAGTACGAAAT
TTCGAGGAGTCCATGAACAATCCGTTACCACCTCTACCGCCACTACCAGATTTGGACAATATGAGAAAGACAGAT
AAAAGGGCACCAAAGAAATCATATTCTGCAAACGATTTACATATGGCACGTTCTTCAAGAGAATATAACTATTAC
AAAGACAACCAGAAATTCTATGATGGGTTTATCCCAGAAAAGAGATATTCTTTAGAAGAGGACTCTATTTCATCA
GGCCTGTTTTCAAACTCTCAGTACTTAAATGATTCTGCTTGTAGTAGTGAGAACAGTTTTGCCTTAATGAGTGAT
TTTAGTGCCACGAGCGCGGGAAGTTTCGCCAGACATAAATATGCACAATCGTTTTCCGATTCATTGCAAAGATCA
CAGAATGCAAATGGCTGCTCTACAAAGATAAATGATTCACAGGAGTTTGGTGATTCCAATGCTAGTTCGAGAAAT
GGAAAAATGGGTGATATCCTTAGGAAAATAATTATTCCAAAGAGGAATACAAATATTTACTCCAGTTCAGTATCC
TCACCAAAGTCACCAAAAGCATATCCTAAACTACCAGATATTCAGAATTTAAACTTGTCTGCAACACCCGATGAA
GCTCGCGATTGGATTGCGGTAAAATGTCACTTGAACAGAGCAAGGACTCTTACCAAATATGACAAACATCCGAGG
TATATGAGAGCTCTCGAAGAAAATCGCGACTTGATCTTACATCCTCAAGATTCGATTTATAACGGGCTAAATACC
AATGAAGTCAAAGGTAATACAAAGCCCGGTTTGGTCGATGTGGAACTGGCCGAATTAAACATAGAATACATTGAT
AAGATGACTTGGAAACGGTGTATTAGAGACGGAACTATGACATTAGACAGTTGGGCTCAAACAACTTTTTCAGCA
AGATACTCAACTGTTCTGGAAAAGTTAAGAGGCATATACATTTTTTGCACAGAGATGTTTGCTCTGACTGATGAT
AATGGAACGTCAGATTTTTCTGCGGAACCCCAAAATTTAGAGAAAATACTATACAGAAAGCATTGTACGCCATAT
GAATTGACATGGCTTTTCAAGAAATTAGCAAACTCATTGGGAATCACTTGTGAAATTGTTATAGGTTTCCTGAAA
ACACCGAGTGCTATAAATTGGGAGTTTAAGTATAACCACTGCTGGTTGAGAATACTGGTTAACAAAGAATGGAGA
TTTATTGACGTTATTTTAGGAAATGTAACCAACCCAATTCATGAATTTGTAAATAACAGAAAGATCAAGAAAGCA
GAAAATAGCTACTTTTTGATGGCACCATTAGAAATGATATACACACATATACCGCCTAGGGAATTTGAGCAGCAT
ATTGTTCCTAGTATCGATCAATTATCTGCATTGTATCTGCCATTAGTTTTTCCATCTTTTTTCAAAAATGAGTTA
AAGTTATACAAATTTAGTACTGCACTATCATTTTTGGAAGATTCTGAGATCTACGAATGCTCCTTAGAAATTCCC
AATGACGTTGAAGTCTTTGCATCCGTGGTTATTCCAACGGATAATGAAGAAGCTTCATCTGCGTACAGGAATATG
GAACTCGCACTGACCCAAATCAAAAAGCAGAAAGCCGAGTCTGGTAGAAGAATTGCTCTAATCAAAGCAGTCCTA
CCACCCAATGTGAACAAAGGTTCACTGTACATACATTCAGGTGTAAGAGGTACGCAAACGAGTATTGCCAATATC
CATCCACTGTCCATGATGGTCCCATTAACACATAAGGGAAGTAACATGAAATACGAATTTGTGATCAAGATACCC
TCAGAAAGCATTCAGAAGATTGAGCTATACATTGTCGAGCCACAGAGTAGATATCTATTTGTTGGTAATGAGTAC
TCATTCGAAGTTATTCAAAGTCCCTCCGATGGTATTGTATACAGCAGTGATGAAGGGCCGAACCAAAATAGGAAA
CAACCAATGGCAATAAAATCACCCTCAGGAAGAGTTCATGAGCTAGTCAAGAGCGATCCCCACTTTCCTTATGGT
ACCTGGAAAGGAAGCATTAAAATCAAAGAGCCCGGCGTCTGGAGCGCTTTAGTGATCGCTGACTCTGGTATTGGG
TGGTCCGTTTTTGCTGAATGGTTGTGCGTATAA

>YOL138C 0.78 Cold
ATGAGCTTATCACCACACGTAGAAAATGCTTCCATTCCCAAGGGGAGTACCCCGATACCAAAAAACAGAAACGTT
AGTTCTATAGGCAAGGGCGAGTTTCTTGGATCTTCGTCAAGCAACAATTCTTCTTTTCGAATGAATCATTACTCA
AATAGTGGACAACCTTCCGTGTTAGATTCTATACGGCGACCTAATCTAACTCCAACTTTTTCTTACAGTAATGGG
GTTTATATGCCTGAAAGTCACAGAACTAGTTCATTCAACGATAGTTATTTGCCATATGATAAGAATCCTTACGCA
AAAACAACCGGAAGCATGAGCAATAAATCGAACATGAAAATCAAAACAAAGAAAAATGCAATCAACACAAATACA
AGAAAGTCGTCTGGACTCATTTACACCACCAAAGTGGATAAAGAATTATCTAGTATAGACAAAGTGAATGATCCA
AACATTAACGGTCTTGTTTGTGCAGGTAAAACTCATTTGGGGCTCTATAAATTCTCGCCCTCAGATAGATCTATC
AAATGCGTTCATGATTTCATAACACCTAATAGCAACACCTCAACAAGGGGAACGACATCCCTTTTGCCCAAGCTG
AGCAAAAGAACAAGACAGAACAAATTTAGTACCATTGCGGATGTTAAGACTGGATTTAATAATTACAAGAACTGT
ATTGCAGTGTGCAACAATTCTACGGCGATATCGATATATGATCTCAATAAGAGTTCCTCGATAGACAATCCCTTG
ATAACATCCTTATGCGAACACACTAGATCTATCAATAGTTTTGACTTCAACATGGTCGAATCAAACCTAATAATA
AGTGGTGGACAAGATAGTTGTGTAAAAAATATGGGATTTACGTTCCAATAAATCTAAAAGCTCAAATAGATCTGAC
ATTAGTATAAATACCGCATCTGACTCAATCAGAGATGTAAAATGGATGCCCGGTTACAATTTTGCATCTAAGAAC
GATCAAGGTTCATCAACGTATGGTAATTTAAAAAGCGGTTACAAATTTGCTTCTATTCACGATTCGGGATACTTG
CTAAAGTTTGATCTTCGACAACCTGCCCAATACGAAAAAAAATTAAATGCTCACACAGGACCCGGTCTTTGTTTA
AATTGGCATCCCAATCAAGAATACATTGCTACAGGTGGTAGAGACGGTAAATCCTGCCTCTGGTTTGTTGGCGAC
AATGCCAATGCCGCCGAAAATACAGTCCTCAATTACGGGAACTCTCCCTCGTTACATGCCCCCAATACGTCCTTG
AACAATAGCGGCTCGCTGGCATTTCCCAAACTAACAATTAATACGGGCTATCCAGTCACTAAATTGAAGTTCAAG
CCTGCTTATAGTAGTAATATATATAATTCATTACTAGGAATATCGTCAATGGGTGACGAAGCTGAAGTTCGCATC
TATTCCTTGGCAAGAAAGTACATTCCAAAACATGTTCTCCTATCAGAAACGCCTTCTTTAGGATTAGTATGGTGG
GACGAAAACTTGATCTTTAATATCGATAAGGGGACCCGTATAAATGGCTGGGACATTAACAAAGAACCAACAGTG
CTCGAAAATTTGAGCAAAAATACAACAACTTGGAGAGATTTGGATGGTAACGGTTTACTTTCAGTTGGCCAAGAG

ATAGGCTCATATGAGGTGGTAGAACCGGAGCTTCAACCTACCTCAAGTACCACATGCAAGAAGCATCCAGGTACA
ATAAAAAAACCCGAAGAATGGCAATCCAGAGAACCAGGGAATAATTGGAGGGATAAAAAAAGGATTTAGCCATACT
GGATTAACAAGTTTTACGCCAGAGAGGCCGCCTACTTTAAAAGCAGGCCCAACGTTTAGCACAAAAAGCTTAACA
TTAGCATCTGGAGCATCTTCTTTTAACAGCTCTTCCGCATCATTGACATCCCTGACGCCACAAACTGAAAATAGA
GAAGAAATCGCCATTGAACCGCCTTGTATCATCACTTTGGATATACCGCAGATCTTCAATAACATAAGATTAACC
AAAATCGCACATTCAAGAAAAAAGAACGTCATTTCCGAAAGTTCTTCTATGAAAAATTCTCCAGTAGAAAAGTTC
AAATATTTGGCGAGACAGCTCAAGTTTTCGTATATTCGAGAGCATAATGTTTCAGACAGTGCAGACACTGCATAT
AAAAATGATATAGAGAACATTGACGTCGTTAAAAACGCAACCGAAACACATGGTGATAATACTACTACTACCAAT
AATAATGATGATGGTGATGATGATGATGATGATGATGATGATGATGACAAAATAATCGAAAGTCACCTTTTGAAAAAA
TACAATTTTCCAGAAAATAATACATGGGCTACCTTAATGAATGAAAAAGTAAATAACAAAAAATCCAAAAGAAAT
TCCAGTAGTTCGAGGGAGTTTGACGAGAAGGATGTCAGATCAAGTATATCTTCCATTTCTGCAAGTAGGCAAAGT
CATGACAGAGCTAGGAAGATCGACAAGAACGTGGAAGCAGAACTGCAAGAAAAAATACAAACACTGGTAGATTTG
ATATCTATTGCAACGCATAACGCATCTGTTTATCTATCCATAGATGATCTAACCAACTTCAAAATCTGGATCTTG
ATAAGAGATTCATTGTTATGGGACTTGAAGTGGATGACCTCCTCCCAAATTTCATCAGATAACGCATCTAATATG
GATGCCAATGAAAGCTCCGATTTCGAAGCTGGAGAAAATCTGAAAACAGGAAAAGAGTTCCCAGAGGAGGATGGT
GCAGGGACAAGTGGGGCTGAGTCGTTAGTTGAAGAAAGGCCGCAGGCATTTCGTGCTAACTCTGATGAACCAAGT
GATGCCGAAAAGAAACCTGTTTCTAAGCTCAAAGAGCAGCTCAAAAACACGGAAATAATCCCCTATGCGCAGCCA
AATGAGGATTCAGATGAAGTTTTAACTAAGCTAAAAGAGCTACAGAACCAACGCTTGGAGTCAAGGACTAAAATG
GGAGAAACAGTGAGTGACGATGTGATTATAGAGGAAGACGAACATGAACATCAAGAAGAAGAACAGCCTCATGAC
TCACCGACTAAGTCAGCACAATTTCACGCGTCTCCTATCGCGAAAAGTATACCAATATTGCAGAAACGTGAGCAC
CGCAAGTCATTCATAGATACTTTCATGTTGCATTCTCCCAACGGTTACAATGGAGATACGGACATAGGTAACGAA
GATGACAATATATCTCCTAGGTTTACTTACAACAGCGTAAGCCCACGCAGTAAGGTTTCGTCGCTACAAAGCTAT
GCCACAACAACCTCGCAACTAGAAACTTTTAAAAAGCTTTCTTCCCATACAGCACCAATAATCGGTTCGCCCAGA
CATGCGCCATCACGGCCAGATAGCATTGGTAGGGAACAACTGTCTTCTTCATTGACGAAAAAGCTTGCCAAATGC
AAGAAAATTATCGCTGACCCACCCTGGGACACGAAAAAACTCATCAAACAACTCTACAATCAAGCTACTGAGACT
GGGAACGTCGTATTGACGGTGAATATTTTATTTCTTTTCCAAACGATATACCAAATAACAGAAATCGACATTGCA
AAGGACGCAATCGCTCACTTCTTGTTATTATTACATCGATACGAACTCTTCGGCATTGCTGCAGACGTCTTAAAA
TACTGCCCATTCGAAGATATCATGGGTTCTGAGGGTGACCAATCTTCCATTCGGTTGTTTTGCGAACGCTGTGGT
GAGTTAATCACTAACGAAAGTTCTAAAGAAAAGCTCAGAGCGGAAGCTCAGCAGACGGGCAATAAGAAGATCATG
GACAAGTTTGGATACTGGTATTGTGACTCTTGTAAGAAGAAGAATACATCTTGTGTTCTATGTGAAAGACCATTA
AAGAAACTGACCATGGTCATCCTCCCCTGTGGACACGAAGGTCACTTCCAGTGCATACAAGAATGGTTTCTCGAT
GAGAATGAACAAGAATGTCCCGGCGGTTGCCCCGGTGTTGCATTCATCTAG

>YBL001C 0.76 Cold
ATGCCCAAGATCTTTTGTTTAGCGGACGTGTGTATGGTCCCTATTGGCACCGACTCTGCTAGTATTTCTGATTTT
GTTGCACTCATTGAAAAAAAAAATCAGAGAAAGCCCATTAAAGAGCACTTTACACAGTGCAGGAACAACAATTGAA
GGGCCTTGGGATGATGTGATGGGTTTGATTGGCGAAATCCATGAATACGGTCATGAGAAAGGATATGTTAGAGTA
CACACTGATATTCGTGTTGGGACTAGAACTGATAAGCACCAAACTGCTCAAGATAAGATCGATGTTGTTTTAAAA
AAAATTTCTCAATGA

>YDR538W 0.76 Cold
ATGCTCCTATTTCCAAGAAGAACTAATATAGCCTTTTTCAAAACAACAGGCATTTTTGCTAATTTTCCTTTGCTA
GGTAGAACCATTACAACTTCACCATCTTTCCTTACACATAAACTGTCAAAGGAAGTAACCAGGGCATCAACTTCG
CCTCCAAGACCAAAGAGAATTGTTGTCGCAATTACTGGTGCGACTGGTGTTGCACTGGGAATCAGACTTCTACAA
GTGCTAAAAGAGTTGAGCGTAGAAACCCATTTGGTGATTTCAAAATGGGGTGCAGCAACAATGAAATATGAAACA
GATTGGGAACCGCATGACGTGGCGGCCTTGGCAACCAAGACATACTCTGTTCGTGATGTTTCTGCATGCATTTCG
TCCGGATCTTTCCAGCATGATGGTATGATTGTTGTGCCCTGTTCCATGAAATCACTAGCTGCTATTAGAATCGGT
TTTACAGAGGATTTAATTACAAGAGCTGCCGATGTTTCGATTAAAGAGAATCGTAAGTTACTACTGGTTACTCGG
GAAACCCCTTTATCTTCCATCCATCTTGAAAACATGTTGTCTTTATGCAGGGCAGGTGTTATAATTTTTCCTCCG
GTACCTGCGTTTTATACAAGACCCAAGAGCCTTCATGACCTATTAGAACAAAGTGTTGGCAGGATCCTAGACTGC
TTTGGCATCCACGCTGACACTTTTCCTCGTTGGGAAGGAATAAAAAGCAAGTAA

>YKR102W 0.71 Cold
ATGCCTGTGGCTGCTCGATATATATTTTTGACCGGCCTATTTTTGCTATCTGTAGCTAATGTTGCTCTAGGTACT
ACAGAGGCTTGTTTGCCAGCTGGAGAGAAGAAAAATGGTATGACTATAAACTTTTACCAATATTCCTTAAAAGAT
TCATCTACATACTCAAATCCGTCATATATGGCCTATGGTTATGCTGATGCAGAAAAACTGGGTTCTGTAAGTGGG
CAAACAAAGCTTTCCATCGATTATTCCATCCCATGTAATGGCGCATCAGATACTTGTGCTTGTTCCGATGATGAT
GCTACTGAATATAGCGCTTCCCAGGTTGTACCAGTGAAGCGTGGCGTTAAACTTTGTTCTGATAATACAACTCTT
TCTTCTAAAACTGAGAAACGTGAAAATGACGATTGCGATCAAGGCGCTGCCTACTGGAGTTCAGATCTGTTCGGA
TTCTACACAACACCCACCAACGTAACCGTGGAAATGACAGGTTACTTTTTACCACCAAAAACTGGTACCTACACA
TTTGGCTTCGCTACTGTGGATGATTCAGCAATTTTATCGGTTGGAGGTAATGTTGCCTTTGAATGTTGTAAACAG
GAACAGCCTCCTATCACATCAACGGATTTCACTATTAACGGTATTAAACCATGGAACGCAGATGCACCTACCGAC
ATAAAGGGGTCAACGTACATGTACGCCGGTTACTATTACCCGATCAAAATTGTTTATTCAAATGCTGTATCCTGG
GGTACGCTTCCTGTTAGTGTGGTATTGCCAGATGGTACTGAGGTTAATGATGATTTTGAAGGATATGTTTTTTCT
TTTGACGATAATGCTACTCAAGCTCACTGTTCCGTTCCAAACCCTGCTGAGCATGCAAGAACTTGTGTATCATCT
GCAACATCTTCCTGGTCGTCCAGTGAAGTCTGTACAGAGTGTACCGAGACCGAGTCTACCAGTTATGTGACACCA
TATGTCACTAGCTCTTCCTGGTCGTCCAGTGAAGTCTGTACAGAGTGCACCGAGACCGAGTCTACTAGCACCTCT

ACTCCATATGTCACCAGCTCTTCCTCGTCGTCCAGTGAAGTCTGTACGGAGTGCACCGAAACTGAGTCTACCAGT
TATGTGACACCATATGTCAGCTCGTCTACTGCTGCCGCAAACTACACTAGCTCATTCTCATCGTCCAGTGAAGTC
TGTACAGAGTGCACCGAAACTGAGTCTACTAGCACCTCTACTCCATATGTCACTAGCTCTTCCTGGTCGTCCAGT
GAAGTCTGTACAGAGTGCACCGAGACCGAGTCTACCAGTTATGTGACACCATATGTCAGCTCGTCTACTGCTGCC
GCAAACTACACTAGCTCATTCTCATCGTCCAGTGAAGTCTGTACAGAGTGCACCGAAACTGAGTCTACTAGCACC
TCTACTCCATATGTCACTAGCTCTTCCTCGTCGTCCAGTGAAGTCTGTACAGAGTGCACCGAGACCGAGTCTACC
AGTTATGTGACACCATATGTCAGCTCGTCTACTGCTGCCGCAAACTACACTAGCTCTTTCTCATCGTCCAGTGAA
GTCTGTACAGAGTGCACCGAAACCGAGTCTACTAGCACCTCTACTCCATATGTCACCAGCTCTTCCTGGTCGTCC
AGTGAAGTCTGTACAGAGTGCACCGAGACCGAGTCTACCAGTTATGTGACACCATATGTCAGCTCGTCTACTGCT
GCCGCAAACTACACTAGCTCATTCTCATCGTCCAGTGAAGTCTGTACAGAGTGCACCGAAACCGAGTCTACAAGC
ACATCCACTCCATATGCAACCTCATCTACTGGCACAGCTACTTCATTTACCGCTTCAACTTCCAATACCATGACG
TCTTTGGTCCAAACAGACACAACCGTTTCTTTCAGCCTATCTTCAACTGTAAGCGAGCATACCAACGCTCCAACT
TCATCTGTAGAGTCAAATGCTAGTACTTTCATATCGTCAAATAAAGGCAGCGTTAAAAGTTATGTTACGTCATCC
ATACATAGCATTACGCCCATGTATCCTAGTAACCAAACCGTAACATCTAGCTCTGTTGTCTCCACACCAATTACT
TCCGAATCTTCTGAATCCTCGGCCTCAGTTACCATTCTACCCTCAACTATCACTTCTGAATTCAAACCATCTACA
ATGAAAACGAAGGTCGTTAGTATCTCCTCATCACCAACTAATTTGATTACCAGCTATGACACTACATCTAAGGAT
TCAACTGTTGGTTCATCCACATCGTCTGTAAGCCTGATCTCTAGTATTTCTCTACCAAGTAGTTATTCAGCTTCT
AGCGAACAAATATTTCACAGCTCCATCGTTAGTTCAAACGGTCAAGCATTAACAAGTTTTTCTTCGACCAAAGTC
AGTTCCTCAGAATCTTCTGAATCACATAGAACATCGCCCACTACATCCAGTGAATCAGGCATCAAATCTTCAGGC
GTTGAAATCGAATCTACAAGTACCTCTTCTTTCAGCTTTCACGAAACTTCTACAGCCTCCACCTCCGTTCAAATA
TCTTCTCAGTTTGTGACTCCATCCTCCCCTATTTCCACAGTTGCCCCTCGTTCTACAGGGCTCAATAGTCAAACT
GAAAGTACAAATTCTTCCAAGGAAACCATGTCGTCTGAAAATAGCGCCAGCGTTATGCCTTCTTCATCAGCTACA
TCTCCCAAAACAGGCAAAGTTACCAGTGATGAAACTTCTTCCGGATTTTCTCGTGATCGCACCACTGTGTATAGG
ATGACTTCAGAAACACCCTCCACAAATGAACAAACAACTTTGATTACTGTAAGTTCTTGTGAATCAAATAGCTGC
TCAAACACAGTCTCAAGTGCTGTAGTTTCCACGGCCACCACTACCATCAATGGGATTACCACTGAATATACTACA
TGGTGCCCTCTTTCTGCTACGGAATTAACAACGGTAAGTAAATTAGAGTCAGAAGAAAAAACCACCCTAATTACG
GTTACTTCTTGTGAGTCTGGTGTCTGTTCCGAAACTGCTTCACCTGCTATCGTTTCGACAGCCACTGCTACCGTC
AATGATGTCGTTACAGTTTATTCCACATGGAGCCCACAGGCTACAAATAAACTAGCGGTTAGTTCTGACATCGAA
AATAGTGCCAGTAAGGCTTCATTCGTTTCAGAGGCTGCTGAGACAAAATCCATAAGCAGAAACAACAATTTTGTT
CCAACTTCTGGGACTACTTCTATTGAAACACATACAACCACTACAAGCAACGCGTCTGAAAATAGCGACAATGTT
TCTGCTTCTGAGGCTGTCAGTAGCAAAAGTGTCACAAATCCCGTGTTGATTAGTGTATCTCAACAGCCTCGTGGC
ACACCAGCAAGTAGTATGATAGGATCTAGTACAGCCTCTTTAGAGATGTCAAGCTACCTCGGCATTGCAAATCAT
CTACTAACCAATAGTGGTATTAGTATTTTCATTGCCTCCCTATTACTGGCAATCGTTTAA

>YOR019W 0.81 Cold
ATGATTTCTGTTTGCCCACAAAATGACTTGCAAAAATGCTACAGAAGCCTCACATTCGATGTTCCAGGACAACAA
TTCGAAGAGAGAAATGAACAAAACCTTAAAAAACGGGCCAAAAAGAAAGGCAGTTTCCAACCATCTGTTGCCTTT
GACACAGTGCCTTCCACCGCTGGTTATTCTTCTATAGACGACAGCAGGGAAGGATTCAAAGGTGTACCTGTTCCC
AACTATTACACGATGGAAGAGTGCTATGACGATGAAACAGACTCTTTTTCGCCAAATTTGCAATATTATTTGAGA
GATACATTCCAATCATCACCTTTTCTGAATACTAGAAAAGAGAACAAATCTGAATCCAGTAGTTTTCCAATGAGA
TCCTCAAAGTTGTTGGAAAAGAATTCTGACATCAAAAAATATTTCTTGGTATCCAAGAATGGAAAAATAGTGAGG
AGAGACTATCCAAGCACGCCAGTAATTGTCAACGAAACGTTGATGATAAACAGGTTTGAAAAGAACTGGATAAAG
TTATGGCGCCAAAGAAAACTACAAATAAATGAAAGGCTGAATGACAAAAAAAAATGGTTTACTTACCCAGAACTT
ATCTTCTCTGAAGAGCGTATTAAACCGTTATATAGAGGAGATGATAGTGCACCATGTACAAAAGAACAAAAAAGA
AAGCATAAAATACTTCAACAAAAGGTCGGATATCCCAATAACCCTAAGACAATAGTTTGTCACATTAACGGAAAA
AAACATACGTGGGTTGCCCTAGACTGGACAGTCTACAAGTTTGCACGAAATCTTGATCACATTGTTGTCATAACT
ACACTGCCAAAAATGATTTCTAACAGGAAAAAAACTGCAAAAGATGATACAGAATGGGCACCGGGATATCAAAAA
GAAGTAATAGATCAAAAATTAAACGACATTTTTGATTATATTTTACAGCTAGTAAAAGTGGTCAAAATATCCGTC
AAAATTACTTTAGAAATAATTGTAGGCAAAATTAAAAAAAGTCTGGTAGATGTCATTAATGTCCATACTCCAGAT
TTCTTAGTTCTTGCTACTTTAAAGCACGAGCGAAATGAGAATCTTATTACATATAAATCCAAAAAGCTGACAGAT
GTCTTTCCTGTTAGTTATCCGATTCCCACATTTGTTGTTCCCTCGAAACGAATGTATTCGTTCGAACTGAATCTA
CAAAGAGAAGTAAATGAACATTATGTCTCAAAAAATCATATGAAGCACGAACACACTGACGTTGAGAGCATGAGC
AGTTCAATGTTCAAAAAAAATACAATATCAGATATTTCTTCACATATTTCCGTAGATTCGTACGCCGAAGATTTC
AAAAGGCAAGGCTACATCAAAAAGCAGTTCAACACCTCTAATGATTCCATTCCAAGAAAATTGACCGGTCTCGCC
CAGCATTCAAGAAGGAAGATCACGGGTGATATAGAAAAATTACAAGACGATGAGAAAGATAGAGAATGTACTAAG
GAAAAACTTTTGTTGAAGAAAATTGATATCATAATTAGAGAGTCATTGAAGTCTTCTTTAGCGATAGAGACGTTG
CCTGGTAAAAATGTATCGCAGTCCAGTCACGGTGACCAAATTTCCAGCTTTAAGAATGCTTTGATAGGCAATGGG
TCGAAAAACACAAAGTTTAGAAAATCTTTAATACCATATTCTTCCTCAGAGGAACAAAATACCACAACAACTATT
AAACTCAGTAGCTCGCCTACGTCCCAAATCAAGTTTGCAACCTCTGTAAAACACAAAGATGGAAGAGCCGCCCTT
GGCAAAGCCAGAAATCTGCCTGATATAAGGCACAGTATTTCCTTCGACAAAGAAAATTCCTTTGATCCATCTGAT
AAAAGCAGTAGTGTTGATAATAGCATTCCTTTGAGGAAAGTTAAAAGTGCCGGTGCGTTAAGAAAAGTCAAAACT
AATGACTCCTCAAGTAGTGCAGGGTCAAAGAAAAGCTCGTCTAGTTTTAGTACTGTGAACACCTTCACTGGGGGT
GGAGTTGGGATTTTTAAGGTGTTTAAAAGTGGAAGTTCCTCTGGAAATAAATCATCCAGTAGAAGGAATAGTAGC
AGTGGCGATGTTTTTGAAAGTGATGATCGTAACGACAAGAAAAAGAAGAAAAAAAAAGAAGAAATCATTGTTC
TTATTCGGCAAAATATGA

>YCL059C 0.8 Cold

ATGGTGTCTACACATAACAGAGATAAACCTTGGGATACGGATGATATTGATAAATGGAAGATAGAGGAGTTTAAG
GAAGAGGATAACGCATCCGGTCAACCTTTTGCTGAAGAGTCCAGTTTTATGACTTTGTTTCCTAAATACAGAGAA
AGTTACTTGAAGACGATTTGGAATGATGTAACAAGGGCTCTAGACAAACACAACATAGCGTGTGTTCTAGATTTA
GTCGAAGGTTCTATGACAGTAAAAACAACTAGAAAAACATACGATCCCGCTATCATTTTGAAAGCCAGAGATTTG
ATCAAATTATTGGCGAGATCCGTTCCTTTCCCGCAAGCCGTTAAGATCCTACAAGATGACATGGCATGCGACGTT
ATTAAAATTGGTAATTTCGTTACTAACAAAGAAAGGTTTGTCAAGAGAAGACAACGTCTTGTAGGCCCTAACGGT
AATACTTTAAAGGCTTTGGAACTTCTAACTAAATGTTACATTCTAGTACAAGGTAACACAGTAAGTGCCATGGGT
CCCTTCAAGGGCTTGAAGGAGGTCCGTCGAGTAGTAGAAGATTGTATGAAAAATATTCACCCTATCTATCATATC
AAGGAATTAATGATAAAAAGAGAATTGGCAAAAAGGCCAGAGTTAGCCAATGAAGATTGGTCAAGATTCTTGCCC
ATGTTTAAGAAGAGGAATGTGGCCAGAAAGAAACCCAAGAAGATCAGAAACGTCGAAAAGAAGGTCTATACTCCA
TTTCCTCCTGCCCAATTGCCTAGAAAGGTTGATTTGGAAATTGAAAGTGGTGAGTATTTCTTAAGCAAGAGAGAA
AAGCAAATGAAGAAATTAAATGAGCAAAAGGAAAAGCAAATGGAAAGAGAAATCGAAAGGCAGGAAGAGAGAGCA
AAAGATTTCATAGCTCCGGAAGAAGAAGCATACAAGCCAAACCAAAATTAG

>YPL236C 0.82   Cold
ATGATTTCTATTGTATTGGAATTGTTCCAGAACTTGTGCTGCTGTCGCGGATTTTCCGATGCTACTATTAGGGTA
AATGATAAACGATATAGGATTCAACGACTACTTGGAGAAGGTGGAATGTCCTTTGTGTATTTGGTACAACTGTCA
AAGAATTCTCTGATTATAGACAACGGCATCGCAACACCAGAATTATACGCACTAAAGAAGATTATTTGTCCTAGT
GTGGAAAGTATATCCAATGGTATGCGAGAAATTGAAAATTACAAACGGTTTCAAAGTCCTTATGTTATAAAAAGT
ATCGACTCACAAGTAATGCAGGAAAAAGATGGGTCAAAAACAATTTACATAGTACTACCCTATTATTCATTAGGG
AGTTTACAAGACTCTATTAATCGGAGGTTACTCGAGGGCACCTTTGTATCAGAGGCAGAATGCGTAAGGATAATG
CTTGGAGTTACAAGAGGTTTACTTTGTTTACATGATCCTGCGTCAAGACAAGATAACGCTACTTCCAGAGTAAAT
GTCGATGCGGTTTCTATGACTTATAGCGATGAAACGGCGATGCTCCTAGAAGACACCCCCTTAGAGATGGACATG
CTTTCCTCCAACTCAGCAGGTTCAATAGCATATGCACATCGCGATATTACACCTTCAAACATTTTGTTCTCTTCA
GACGGTTTACCAGTAATTGGTGACCTCGGCTCCTGCTCTCAAGCTGATATAACAATTGAAAACAGACATCAACTC
AGCGAACTACAAGAATGGGTCAATGACAATTGTACGTTGCCATATACTCCACCAGAACTTCTGAACTTGAAATTA
AATCAGGTTTTGAGCTCTAAGGTTGATATTTGGTCTTTGGGTTGTACGTTCTATACTTTAATGTTCGGTATCTCC
CCCTTTGAGCGAGAAGAGCAGATACATGGAGCTTCTTTAACCTACGCTATAAACACTGGTAAGTACAGTTTCCCG
AGAAATTCCAGATTTTCTGAGGGGCTTTTGAGTGTAATCAAGAAATGCATTCAAGTGGATCCTATACAAAGGCCT
ACTACCAGCCAATTATTAAATCTTTTACAAGATTTAGACACTTGA

>YKL001C 0.82   Cold
ATGGCTACTAATATTACTTGGCATCCAAATCTTACTTACGACGAACGCAAGGCATTGAGAAAACAGGACGGTTGT
ACTATTTGGTTAACAGGTCTAAGTGCGTCAGGTAAAAGTACAATCGCCTGTGCGCTAGAACAGTTACTGCTCCAA
AAAAACTTGTCTGCATATAGATTGGATGGTGACAACATTCGTTTTGGATTGAACAAGGATTTGGGTTTCTCAGAA
AAGGACAGAAATGAAAACATTCGTAGAATTAGCGAAGTTTCTAAGCTATTTGCTGATTCATGTGCTATTTCAATC
ACCTCATTTATCTCTCCATACAGAGTTGACAGAGATAGAGCTCGTGAACTACATAAGGAGGCTGGTTTGAAGTTC
ATTGAAATATTTGTTGATGTTCCATTAGAAGTCGCTGAGCAAAGGGACCCTAAGGGTTTATACAAGAAAGCTAGG
GAGGGTGTAATCAAGGAGTTTACAGGTATTTCTGCCCCATATGAAGCGCCAAAAGCTCCAGAGCTACATTTGAGA
ACCGACCAGAAGACGGTTGAAGAATGTGCTACCATTATTTATGAGTACTTAATCAGTGAAAAAATCATCCGTAAG
CATTTGTAA

>YNL213C 0.75   Cold
ATGAACATTCTGCGAATAGCATGTCGTTCTTTTCATTGCCTGCGCTGCGGTCCATTACTCAATGAGAACAGGGGA
TGGTCATCCAAAAAGATTATTAAATTAGTCAATAAATCGAGTTTGTCCAATAAAGAGTTTACAGAAAAAGTACGA
GATGGTACGAAAGACATTCCTGAGTGGAAGAAGCAGAAAATGGCAGTTAGAAAAAAATTGCAAGGCCAGAGGTGG
AACCCACCAAAAAAGATATCACAGGAACAAATGGAGGCTCTAAGACTATTGAAATTCAACTTTCCTGAACTGACA
GCATCTGATCTTGCAGACCGGTTCAAAATTTCTCCCGAGGCTGTTCGAAGAATTTTGAAGTCCAACTGGAAGCGT
ACTGATGAGGAAAACAATAACACCTACGAGAGATGGAAAAGAAGAGGAGAACGGATAAAAGAAATGTATCAAAGA
AAAGAGGATGCCGATTTTGTATCCAATCAAATTGTTACTAGCAGAAAGATTATACTTGGGTCCAACTCAAATTCA
CCTGAATTAATAGCAAGGAATGTTCGCACTTTTAAACCTTTCAAGCCTAACAACAGTACACCTGAAAAGAAAAAC
ACCAACAAACTATACATTTTGAAGCATTTGGGCTCGAAACAATAA

>YLR088W 0.72   Cold
ATGGCCTTATTGGAGAAGTTGCATCGAAGGATTGTTGATATGGGGCTTGTCCCGCGTATAATCGCCTTATTACCA
GTTATTTCCATGCTATGCGCTCTATTTGGGTTTATTTCTATAGCTATTCTGCCTATGGATGGACAGTACAGAAGA
ACATACATTTCTGAGAATGCATTGATGCCTTCACAAGCGTATAGTTACTTTAGAGAATCTGAATGGAACATTTTG
AGGGGCTATCGATCTCAAATTAAAGAAATGGTAAACATGACTTCTATGGAAAGAAACAATTTGATGGGTTCTTGG
TTACAAGAATTTGGTACTAAGACTGCTATTTACGAAAATGAACAATATGGAGAAACATTGTACGGTGTAATGCAC
GCTCCTAGGGGTGATGGAACAGAAGCGATGGTGCTTGCCGTTCCATGGTTTAATTCAGATGATGAATTCAATATT
GGCGGCGCAGCTTTGGGTGTATCTTTAGCAAGATTTTTCTCACGTTGGCCAGTATGGTCCAAGAATATAATTGTT
GTCTTCAGCGAAAATCCTCGTGCAGCATTAAGATCATGGGTTGAGGCATACCATACTTCCTTAGATTTGACTGGT
GGTTCCATTGAAGCTGCTGTTGTGTTGGATTATTCGAGTACGGAAGATTTCTTCGAGTATGTAGAAATCTCATAC
GACGGTCTGAATGGTGAGCTGCCCAATTTGGATCTTGTCAACATCGCTATATCCATTACGGAACATGAAGGTATG
AAAGTTTCTTTGCACGGTCTACCCAGTGATCAGTTAACTAATAATAATTTCTGGTCAAGATTAAAAATATTATGC
CTGGGAATAAGGGATTGGGCGTTGTCCGGTGTTAAAAAGCCCCATGGTAACGAGGCATTTAGCGGCTGGAGGATT
CAATCTGTAACATTGAAAGCACATGGAAACAGTGGTCATGATATTACTACATTTGGACGTATACCCGAAGCAATG

TTTCGCTCTATTAATAACCTTTTGGAAAAATTTCACCAATCGTTCTTCTTTTATTTGTTATTAGCACCACGTCAG
TTCGTATCCATTAGTAGTTATTTGCCAAGCGCTGTGGCTTTATCTATAGCATTCGCCATAAGTTCATTAAATGCA
TTTATAAACAATGCTTATGCAAATATATCCTTATTTTCCGAGTATAATTTGGTAGCGTTGTTGGTTTGGTTCGTG
TCATTGGTGATATCATTTGTTGTTTCACAAGCGTTTCTTCTAATACCTTCATCGGGATTATTGATGACAATTAGC
ATGGCATCTTGTTTTTTACCTTTGATACTTTCCAGAAAAATTCACATCTCAGAACCACTATCATACAGGTTGAAA
AATGTTGCTTTTTTATATTTCAGTTTGGTTTCAACATCTTTGCTAATGATAAACTTTGCAATGGCTTTACTGATC
GGCACATTGGCATTTCCTATGACATTTGTGAAGACCATTGTTGAAAGTTCTAGCGAACATGAGGTGACAACTCAA
TCCTCTAACCCAATAAAAACTGAGCCGAAAGATGAGATAGAGCTCGTCGAGAATCACATGGATACAACGCCAGCA
ACCCCCCAACAACAGAAACAAAAACTAAAAAATTTAGTACTATTAATTTTGACAAATCCATTTATTTCAATAACC
TTATTCGGACTATTTTTTGATGATGAATTTCATGGATTTGATATAATAAACAAACTGGTTTCAGCATGGTTGGAT
TTGAAATGTTGGAGTTGGTTTGTACTTTGTATAGGTTGGCTTCCATGTTGGCTATTGATATTAGCGTCATCGTTT
GAATCTAAATCTGTCGTAGTAAGGTCGAAAGAAAAGCAAAGTTAG

>YKL214C 0.81   Cold
ATGGACAAAGCATTTGACGAAATTATTGGAAATAGTCACACAGACAGCTCATCTAATCATAAGGTAACGAGATAC
CGCAGAAGGGATTTAAGAAATGAATTAGGACCTAGACTGGGATTTGCGCCTTCGGATGCTGCATCAAGGTCGAAA
GATCGCCTTTATAGAGAGAGGGAAGAGCCTCCTTTGCCTAAGAGAATTAGAATATCCAAAATCCCACTAGACGTT
TCTGACTACACCCTCGATGATATGATTAAAGAGTTTGGCTCGCCAATATTTTCGAAAATTTTTGACAATAAAGAG
GATCGTACATGTATTTACGAATTTGAAGACCCAGAAGTTTTGGAAAAGATTGTCGAGCGTTATAATGGACATGAG
TTACATAATGCAAAAATTGAAGTGGAGATTTACCAACCACAGAGAAAGCATTCTAGAATGAATGCCCACAACCGT
CGCAAACAGACGGCTCAAGAACACGGAAGAGGCAGGCCAGGGAGCCATTATCGCCAAAAGCCTAACAGAGTCTCC
AAAAAGAACAAGGGCCGTGAGAAGAATAACACTCCTACTTCTGTCGAAGCTCTTGACGCTGAATTAGATGCTTAC
ATGAAAGGTTAA

>YNL328C 0.73   Cold
ATGGTTTTGCCTATAATAATTGGTTTGGGCGTGACAATGGTTGCTCTAAGTGTCAAGTCTGGTCTCAATGCATGG
ACCGTCTACAAGACCCTGTCCCCTTTAACTATTGCAAAACTAAATAACATTCGCATAGAAAACCCGACGGCGGGC
TACCGCGATGCACTTAAGTTCAAAAGCTCACTGATAGACGAAGAACTGAAAAATAGATTAAACCAGTACCAGGGA
GGCTTTGCACCGCGAATGACAGAGCCCGAAGCCTTGCTCATCTTGGATATCTCCGCCAGAGAGATTAATCACTTG
GATGAAAAATTACTGAAAAAAAAGCACAGGAAGGCTATGGTTCGTAACCACCCAGACAGAGGAGGGAGTCCCTAC
ATGGCGGCCAAGATAAATGAGGCGAAAGAAGTTCTCGAAAGAAGTGTTTTACTAAGAAAGAGATAA

>YHR055C 0.75   Cold
ATGTTCAGCGAATTAATTAACTTCCAAAATGAAGGTCATGAGTGCCAATGCCAATGTGGTAGCTGCAAAAATAAT
GAACAATGCCAAAAATCATGTAGCTGCCCAACGGGGTGTAACAGCGACGACAAATGCCCCTGCGGTAACAAGTCT
GAAGAAACCAAGAAGTCATGCTGCTCTGGGAAATGA

>YCL074W 0.76   Cold
ATGGACGTTGACACTGCGTTTCTAAACTCAACCATGGATGAGCCGATCTACGTAAAACAACCACCCGGGTTTGTT
AACGAGAGGAATCCCGACTATGTATGGGAACTATACGGCGGTATGTATGGACTCAAACAAGCCCCATTACTATGG
AACGAACATATCAACAATACTCTTAAAAAGATTGGTTTCTGTCGACATGAAGGCGAACATGGCTTATATTTTCGT
TCCACATCTGATGGTCCCATCTACATTGCCGTATACGTAGACGACTTACTTGTTGCTGCTCCCTCTCCTAAAATA
TATGACAGGGTTAAGCAAGAATTAACGAAATTATACTCAATGAAGGATCTCGGTAAAGTTGACAAATTCCTCGGT
CTTAACATTCATCAATCGTCAAACGGAGACATCACTCTCTCCCTTCAAGACTATATTGCTAAAGCTGCATCTGAA
AGCGAAATAAACACATTTAAACTTACACAGACTCCGCTCTGCAACTCAAAGCCTCTTTTCGAAACAACTTCCCCG
CATCTAAAAGACATCACTCCTTATCAGAGCATAGTTGGTCAGCTTCTCTTTTGTGCAAATACTGGTCGTCCGGAC
ATATCGTATCCAGTCTCATTACTCTCCAGGTTCCTTCGAGAACCTCGCGCAATCCATTTGGAGTCTGCTCGGCGG
GTTCTACGGTACCTATATACCACCAGAAGTATGTGTCTCAAGTATCGTTCTGGATCTCAGTTGGCATTAACTGTA
TATTGTGATGCATCTCATGGAGCTATTCACGATCTACCACACTCTACTGGGGGGTACGTGACTCTACTTGCTGGT
GCTCCCGTTACGTGGTCATCGAAGAAGCTCAAGGGTGTGATTCCTGTACCATCTACTGAGGCAGAATACATTACT
GCAAGTGAAACTGTCATGGAGATATAA

>YHR073W 0.82   Cold
ATGGAAACAATTGATATACAAAATCGATCATTTGTTGTTCGTTGGGTAAAATGTGGCCGTGGCGATGTAATCAAT
TATCAGATCAAGCCATTGAAGAAATCTATTGAAGTCGGCATATACAAAAAGTTGAAATCCAGTGTAGATGACCAT
GCTTCCGCAGTTCACATTGCACCTGACACTAAAACATTGCTAGACTATACTACGAAATCTCTATTACATAAGGGA
AGTTCGAGTAATATTGAGGAGCATCACAGGCGTTCCTCACAGCACTCTCATAGTTCGAGCAATGGATCGGATAAT
AAGAGGAAAGAGAGATCATATTCTTCACTGTCCATTAGCGGCATACAGCAGCAATCCCAGGAAATACCCCTACGT
GAAAAACTCTCTGCGTCAGGATTCACTTTGGTCAAGAGGGTCGGCAACGTTTCCGGTAACACTATGGTCCAAGGT
GATCTTGAAGTGAAAGATACAGACTACTATTATGCATTTATACTGGACAACTCGTCTTCCAAGAATGCAAAAAAG
AAAATTCTTTTTAACGCAAGTGTGATAAACGGCGATAATCAATCGATGATAAGCACAAGATCCACCCCTCCCGCA
AGGCCTACAGCTTTAAGCAGAACATCTACTCAACAGGATATGTTATTTAGAGTAGGACAAGGTCGTTACTTGCAA
GGCTACCTGCTGAAAAAAAGGAGAAAGAGACTACAAGGTTTTAAGAAAAGGTTTTTCACCTTGGACTTTCGATAT
GGAACCCTATCGTACTATTTAAACGACCATAATCAAACTTGTAGGGGTGAAATTGTCATAAGCTTGTCATCTGTT
AGTGCCAATAAGAAGGACAAAATAATCATAATTGATTCTGGTATGGAAGTTTGGGTCTTGAAGGCTACAACTAAG
GAAAATTGGCAGTCGTGGGTCGATGCGTTACAAACTTGTTTTGACGATCAGTTCGAAGATAAGGACACGTCCACT
TTGGAAGAAAATCCCGACATTCTTGACGATGACAAGGAAGTAATTAATAAAAGCTCACCTCAAGACCACGACCAC

CTCACGCCCACGGCTACGACCAAAAGTGCACTATCACATAGACAGCATACTCAGAAAGACATGGATGACATATAT
GTCCCATTGCCCAGCGAATCTTACGCTACTTTCTCCATGAATCTACGTTTGATTCAACAACGACTGGAGCAGTGT
AAAAAAGACTCGTTATCTTATAAACCAACTACTTTACATCAAAGATCGGAGGGGCTAAATGGAACACATTCGTCA
TCTTCCGTCTTTACCAATAATAGAGTGTCTTCATTCAATCATTCTTCTTCTGGTATGACGTCATCTGATTCTTTA
GCCTCTGAAGAGGTTCCTTCCAACAAAACATATATTGAGCATGCTTTATATAACCAATTGGCAGATCTTGAAGTA
TTTGTTAGCCGGTTCGTTACACAGGGAGAGGTTTTATTCAAGGACCACCAGATCCTGTGCAAGAAAGCAAAAGAC
ACGAGAGTTTCTTTAACTTCATATCTTAGCGAGAATGATGAATTTTTTGATGCAGAAGAGGAAATCAGTCGGGGA
GTCATTATATTACCTGACACAGAAGATGATATTAATAATATAGTCGAGGAAACTCCCCTTCTTGGTAAAAGTGAC
CAAAATGAGTTCACAAAAGAGGTCCAATTGTCGGGATCCGAACAGATAGCTTCATCAAGTGTGGAGAGCTATACA
ACTAACGATGAAAATCATAGCCGTAAACACCTCAAAAATCGTCACAAAAATCGTCGCCGTGGCCACCCTCATCAT
CAAAAAACCAAAAGCGCCCAATCCTCAACAGAAACATTTACAAGTAAGGATTTGTTTGCTCTTTCCTATCCAAAG
AGTGTTACACGCCGTAATGACATACCTGAAGCTGCAGCTTCTCCGCCAAGCCTATTATCTTTTTTGAGAAAGAAT
GTAGGCAAAGATCTGAGCTCTATTGCCATGCCAGTAACCTCAAATGAGCCTATTTCTATTTTGCAGTTGATATCA
GAAACATTTGAGTATGCTCCACTTTTAACGAAGGCTACCCAACGTCCTGATCCTATAACCTTTGTTTCTGCATTT
GCTATTTCCTTTCTTTCCATATATAGGGATAAAACAAGAACGCTAAGAAAGCCTTTTAATCCTTTACTGGCTGAA
ACATTTGAACTTATACGAGAAGATATGGGGTTTAGGCTTATATCGGAAAAGGTCTCACATCGTCCTCCAGTATTT
GCCTTCTTTGCAGAGCATCTCGACTGGGAGTGTAGTTACACTGTAACGCCATCGCAAAAGTTTTGGGGTAAGTCT
ATTGAATTGAATAACGAAGGTATACTGAGATTGAAGTTTAAGACAACTGGAGAACTATTTGAATGGACGCAACCA
ACAACTATTTTAAAAAATTTGATAGCGGGTGAGAGATATATGGAGCCTGTCAACGAATTCGAAGTACATTCTTCG
AAGGGAGACAAATCACACATCCTGTTCGATAAGGCAGGTATGTTTAGTGGAAGATCTGAAGGATTTAAGGTTTCG
ATAATCCCACCACCTTCGAGCAATCGCAAGAAGGAAACTCTAGCTGGTAAATGGACACAGAGTTTAGCTAATGAA
ACTACACATGAAACTATATGGGAAGTAGGTGATTTAGTTAGCAATCCGAAGAAGAAATATGGCTTTACTAAATTC
ACCGCAAATTTGAATGAGATAACTGAAATTGAAAAGGGCAATTTACCACCTACGGACTCAAGATTAAGACCAGAT
ATTAGAGCTTACGAGGAGGGAAATGTTGATAAGGCGGAAGAGTGGAAGCTGAAATTGGAACAACTTCAACGTGAA
AGACGTAATAAAGGGCAAGATGTGGAGCCTAAATATTTCGAAAAAGTATCTAAGAATGAATGGAAATACATAACT
GGACCGAAGAGTTATTGGGAAAGAAGAAAGAAGCATGATTGGTCTGATATTTCTCAACTCTGGTGA

>YLL021W 0.8Cold
ATGGGTACGTCAAGCGAGGTTTCTCTCGCACATCATAGAGATATCTTCCATTACTACGTCTCACTGAAGACTTTT
TTCGAGGTGACTGGCGAAAATCGTGACAGGTCAAATTCGACACGAGCTCAAAAGGCCAGAGCCAAGCTGTTGAAG
CTATCTTCTTCGCAATTTTACGAGCTGAGTACAGACGTGTCCGATGAGCTGCAGAGGAGAATCGGTGAAGATGCT
AACCAACCAGATTACCTTTTGCCGAAGGCAAATTTCCACATGAAAAGGAACCAGGCTAGACAGAAACTGGCCAAT
CTATCACAAACTCGATTTAATGATTTGTTGGACGATATCCTTTTTGAGATCAAGAGAAGAGGGTTCGACAAGGAT
TTGGATGCTCCACGGCCCCCATTACCGCAGCCGATGAAACAAGAGGTCAGCAAAGACAGCGATGATACTGCAAGA
ACATCCACAAATTCTTCCTCTGTGACTCAAGTAGCTCCAAACGTCTCCGTACAACCTTCTTTGGTCATTCCTAAG
ATGGCATCTATCGATTGGTCTTCTGAGGAAGAAGAAGAGGAGCAAGTAAAGGAGAAGCCAAATGAACCGGAGGGA
AAACAAACAAGCATGGATGAAAAGAAAGAGGCTAAACCTGCTCTAAACCCCATAGTTACAGATTCTGATCTGCCT
GACTCCCAAGTGCTCGCTCGTGATATCACATCAATGGCAAGGACTCCAACAACGACGCATAAAAATTACTGGGAC
GTTAATGATTCTCCAATTATCAAGGTAGATAAAGATATCGATAACGAAAAGGGTCCCGAACAGTTGAAGAGCCCT
GAAGTACAACGGGCTGAGAACAATAACCCTAACTCAGAGATGGAAGACAAGGTTAAAGAACTGACTGATTTAAAC
AGCGACTTACATTTGCAAATTGAAGATTTGAATGCTAAGTTAGCATCTTTAACCAGCGAGAAGGAAAAGGAGAAG
AAGGAAGAGAAGGAGGAAAAAGAAAAGGAAAAGAACTTAAAGATTAACTACACCATTGATGAAAGTTTTCAGAAA
GAATTGCTGTCATTAAACTCTCAAATCGGTGAATTATCAATTGAGAATGAAAATTTGAAGCAGAAAATTTCAGAA
TTCGAACTGCATCAAAAAAAGAATGACAACCATAATGATTTGAAAATCACTGACGGTTTTATTAGCAAGTACTCT
TCTGCCGATGGGCTCATTCCAGCTCAATACATCTTAAACGCTAACAACTTGATAATTCAATTTACTACTAGGCTT
TCCGCAGTACCCATAGGCGACTCCACGGCAATTTCCCATCAAATTGGCGAAGAGTTATTTCAAATATTATCCCAG
TTATCGAACCTAATCTCCCAGCTATTACTATCGGCCGACCTATTACAGTACAAAGATCAGGTCATTTTACTGAAG
GCATCATTATCGCATGCGATCACATCGATAAGATATTTCTCTGTTTACGGTCCCGTATTAATTCCGAAAATAACT
GTGCAAGCTGCTGTTTCAGAGGTTTGTTTTGCCATGTGTAATCTAATTGATTCAGCGAAAATAAAATCCGATTCA
AATGGTGAGAGCACCACCTCTAATGAAGGTAACCGACAGGTATTAGAATATTCTTCACCAACTGCTACCACCCCA
ATGACGCCAACTTTCCCCTCGACTTCTGGAATAAATATGAAGAAGGGGTTTATAAACCCAAGAAAACCAGCATCT
TTCTTGAATGATGTGGAGGAAGAAGAATCTCCAGTCAAGCCATTGAAAATTACACAAAAGGCAATTAACAGTCCG
ATCATAAGACCGTCATCGTCTAATGGAGTTCCAACAACCTCAAGAAAACCTTCAGGAACGGGGCTATTTAGTTTA
ATGATTGATTCATCAATTGCTAAGAATAGCTCCCATAAAGAGGATAATGATAAATATGTCTCGCCCATAAAGGCA
GTAACATCGGCCTCCAATTCTGCAAGTAGCAATATTTCCGAAATTCCTAAACTAACACTACCTCCACAAGCCAAA
ATCGGTACTGTTATTCCACCGTCAGAGAATCAAGTTCCCAATATTAAAATCGAGAATACAGAAGAGGATAATAAA
AGGAGTGACATAACAAATGAAATCTCTGTTAAACCAACTTCTAGCATTGCTGATAAACTGAAACAATTTGAGCAA
AGTTCCGAAAAGAAATCATCACCAAAGGAAAATCCTATAGCAAAAGAAGAAATGGATTCAAAACCAAAACTATCC
AATAAATTTATCACTTCAATGAATGATGTGTCCACAGATGATTCAAGCTCTGATGGTAACGAAAATGACGATGCA
GACGATGATGATGATTTTACCTATATGGCATTGAAACAAACAATGAAGAGAGAAGGTTCAAAAATTGAAAAAAAT
AATGACAGCAAACTACCTGCAAATATAGTGGAACTTGATTTACATGAGTCACCGGAGTCCGTGAAGATTGAATCT
CCTGAATCGATAAAGGAAATCACGTCATCTGAAATGTCTTCAGAAATGCCAAGTAGTTCGCTGCCTAAGAGATTA
GTAGAGGATGTTGAGCCTTCAGAAATGCCAGAGAAGGGCGCATCTGTAGAATCAGTCAGGAAGAAAAATTTTCAA
GAACCACTTGGTAATGTCGAATCTCCGGATATGACGCAGAAGGTCAAGTCTTTGGGTATGACAGGAAAGGCTGTA
GGCCCAGAATCAGATAGTAGGGTCGAATCTCCGGGCATGACAGGACAGATTAAATCTTTGAATATGGCAGGAAAA
GTTGTAGGCCCAGAAGCAGATAGTAGGGTCGAATCTCCGGGCATGAAAGAGCAGATTAAGTCTTTGGGTATGACA
GGAAAAATTACAGCTCAAGAATCAATCAAGTCCCCGGAAGCGGCTAGGAAGTTGGCGTCATCAGGAGAAGTTGAC

AAAATTGAATCTCCAAGAATGGTAAGGGAAAGCGAGTCCTTGGAGGCAGTAGGCAATACTATCCCCTCAAACATG
ACAGTGAAAATGGAATCCCCAAATTTAAAGGGAAATACTGTGTCTGAACCTCAAGAAATAAGGAGAGACATTGCC
TCCTCAGAGCCGATAGAGAATGTTGACCCCCCAAAAGTACTAAAAAAGATTGTCTTTCCAAAGGCTGTTAATAGA
ACTGGATCACCAAAATCAGTCGAAAAGACTCCATCTTCAGCTACACTGAAAAAGAGCGGGCTCCCAGAACCGAAT
AGCCAAATTGTTTCTCCTGAATTGGCAAAAAATTCACCTCTAGCACCGATAAAGAAAAATGTCGAGTTACGAGAA
ACTAATAAACCACATACTGAGACTATCACTTCTGTGGAACCAACAAACAAGGATGCCAATACTTCTTGGAGAGAC
GCCGACTTAAACCGTACGATCAAACGAGAGGAGGAGGACGAAGATTTTGATAGAGTGAACCACAATATCCAGATC
ACTGGTGCATATACGAAAACTGGAAAAATTGATTATCATAAAATACCTGTTGATCGTAAAGCAAAAAGCGAAGCA
GAAGTGCATACTTCCGAGGAAGATATTGATGAATCAAATAATGTTAATGGAAAAAGAGCTGATGCCCAAATACAC
ATCACTGAAAGAAAGCATGCTTTCGTAAATCCAACTGAAAATTCACAGGTAAAAAAGACGAGCCACTCACCATTT
TTAAACAGTAAACCGGTTCAATACGAGAACTCAGAGTCGAACGGCGGCATTAACAACCACATAAAGATAAAAAAT
ACTGGAGAAACTACGGCACATGACGAGAAACATTATAGTGATGATGATGATTCTAGCTATCAATTTGTTCCCATG
AAACATGAAGAACAAGAACAAGAACAAAACAGAAGTGAGGAAGAGGAAAGTGAAGATGACGATGAAGAGGAAGAA
GACAGTGATTTTGATGTGGATACATTTGACATTGAAAATCCGGATAATACACTATCAGAGTTACTATTGTATTTA
GAACATCAAACAATGGACGTCATATCCACGATTCAATCGCTTTTGACATCGATCAAGAAACCACAGGTGACGAAG
GGTAATTTGAGGGGAGAATCGAATGCAATCAACCAAGTCATAGGTCAAATGGTGGACGCTACTAGCATATCAATG
GAGCAAAGCAGAAATGCCAATTTGAAGAAACACGGTGATTGGGTGGTGCAAAGTCTAAGAGACTGTTCGCGTAGA
ATGACAATTTTGTGCCAATTAACTGGCGATGGAATACTAGCGAAGGAAAAGAGCGATCAAGATTATGCTGACAAA
AACTTCAAACAGCGGTTGGCAGGGATTGCGTTTGATGTTGCCAAATGTACAAAGGAGCTGGTAAAAACTGTAGAA
GAGGCAAGTTTGAAGGACGAAATAAATTATTTGAATTCGAAGTTGAAGTAA

>YOR218C 0.75   Cold
ATGAATAAATCATGCTTTCGTTTTCCTTTTTTTGCTACCACCAGGTTTACTGGTGGCAGTCTTCCTCTTCGTAGG
TTTGGCTTTCTGCTTGATAAGTTTATCCTTCTTCAAGTCTGTGCTACTATCTTGTGTTTCTTCATCATCTGCGGG
AACTGGATTGTCATCTGCGTCAACGACATCTTCGAAATCGGGGGTGCTAGTACTGGTGCCAACACCACCACCACC
AATAGTACTACCTGTTCTGTAAATTGCGACTGGATGTGTCATACTGTTGTATTTCCGCGTGAATCCACTTTTAAC
CGTAGCTGGTATCTTTTTGATAATGGCTGTGGTCACATCAGGACCTACGAAAAACTCCATAATAGAATCCCAGTC
TTCTTTGGTCAAATAGTAATCGTCCATTACCTCTATGACAGATGA

>YLL002W 0.69   Cold
ATGTCACTGAATGACTTCCTAAGTTCCGTGCTACCTGTCAGTGAACAATTTGAATACTTATCGTTGCAATCTATT
CCGTTAGAAACCCATGCTGTCGTAACCCCAAATAAGGACGACAAAAGGGTCCCAAAAAGCACGATCAAGACTCAA
CACTTCTTTAGTCTATTTCACCAAGGAAAAGTTTTTTTTTCATTAGAAGTGTATGTGTATGTCACGCTTTGGGAT
GAAGCAGATGCCGAACGGTTAATATTTGTATCAAAGGCAGACACTAATGGTTATTGTAATACGAGGGTAAGCGTT
AGAGATATTACAAAAATAATATTAGAATTTATATTATCAATCGACCCGAATTACTATCTTCAAAAAGTAAAACCG
GCAATAAGATCATATAAGAAGATATCCCCCGAGCTGATTAGCGCAGCCAGTACGCCAGCAAGAACTTTAAGGATT
TTGGCTAGAAGGCTTAAACAGTCAGGCAGCACCGTTTTGAAAGAAATAGAATCTCCACGTTTTCAACAAGATCTT
TATCTCTCATTCACCTGTCCTCGTGAGATTTTGACCAAAATTTGTTTATTTACTAGACCTGCATCCCAGTACCTC
TTCCCAGATTCTTCAAAAAACAGCAAAAAGCATATACTAAATGGCGAGGAACTAATGAAATGGTGGGGCTTTATT
TTGGATAGATTACTAATTGAATGCTTTCAAAATGATACACAAGCAAAATTAAGGATACCGGGCGAAGATCCTGCT
CGAGTAAGATCATACCTAAGAGGGATGAAATATCCACTATGGCAAGTGGGTGATATATTTACCTCTAAAGAAAAT
TCTCTTGCGGTATATAATATTCCATTATTCCCAGACGATCCTAAGGCTAGATTTATACACCAATTGGCAGAGGAA
GATCGCCTCCTCAAAGTAAGCTTATCATCCTTCTGGATTGAACTACAAGAGCGTCAAGAGTTCAAATTAAGTGTC
ACATCATCTGTAATGGGTATTTCGGGATACTCTCTTGCCACTCCATCTTTATTTCCATCTAGTGCCGATGTTATT
GTACCGAAGTCAAGGAAGCAGTTTAGGGCAATCAAGAAGTACATTACTGGAGAGGAATACGATACAGAGGAAGGC
GCAATAGAAGCTTTCACCAATATTCGTGATTTTCTATTGCTCAGAATGGCAACAAATCTTCAATCTTTAACAGGG
AAGAGGGAGCATCGGGAGAGAAATCAGCCGGTTCCTGCAAGCAACATCAACACGTTGGCGATAACAATGCTAAAA
CCGCGTAAAAAAGCTAAAGCCTTGCCTAAAACTTGA

>YNL234W 0.69   Cold
ATGACAGGAGAAAAAATACTTCATTCGCAGTTGTTAACCAACAGCGACATGTCTAGCGGGAATGTCCACCATACG
AAACCGATGATGTACAACGTAACCCTGCCTTCCTACAACAGCAGTAGTATAGGTCCAGTCGATAATTTGAAAATA
AACGAGAGACCTGGCTCTCATGATCATTCCATGAGAAGTGAAATGTCATCCAAAAACTCCGGAAGCGATTTTATG
CCACAATCTATTTCACGTTCCGAAGGTAGTGTCTACCAGGTCAAGATAGATCGTGGTGACTCCCCGAATACAGAA
GGATTCGACTTTAAAGTTAATGCAAGAGATCTGCTGTTACTTCGAATGTCCTGGGATATCCTCCTCAGGGAGTAT
TTAACACCTAAAGAATTAAAAGTTTTTCAAGCACTTCTGTATTCCAATAAACATATTACTTCAACAGAAAGACCC
TATTTGAATACTGCTCCTGACGGTATGATTTCCAAAACTATTGACCCTACTGCCAGGCCACGTAAAACAAAACAA
AGGGACAATGATAACAAGGTTGATACTGCACTATTTTGTTCACAGTTTTATGACAATTTGATTGCAATGGATCCC
TTGTTAGAAGAATATTTTCCATCATTAAAACATCAAGCAGTTTCGTTCTGTAAGGTCCTTGATTCTGCTATTGAC
AACCTTGAAAACGTTCATGTCCTAGATGATTATATTGTGAAATTAGGAAAACGCCATTCCAGAATTCTCGGCATC
AAAACCGTTGGTTTCGAAGTAATGGGAAAAGCATTTATGACTACATTGCAGGACAGGTTTGGATCTTTTCTCACA
CTGGAACTCAAAAATCTATGGGGACAACTTTACTCATATTTGGCAAATTGTATGATTACTGCAGGGAAGGACCCA
ATGGAAAAGATTCAACCAGATTTTTCGTATAATGGTGACTCTGTGGTTTTAAATTTTTCCATTCCCAAACTTGCG
ATGCATGATATAAGTACAGTTAACAAGCTACAAATGGTGAAAACTAAAAACGCTACTATACCTCATAATATAACA
CAAGTACCGACAAATAAAATTCCTACAGAAATACTCTTAGACAATTCTTCTACTCCAATAAAAAGCGACCGTGAA
TCGACGCCTCCAATCTCACCAAAGGGCTCTGGAAGTACAAAACCAAGCATCGGTAGCAGTACCGTCGTGGAAAGT

AATACCAAGAAAAATAATTATGATGAAAAAATCCATTTATTGCAAAAAACTGCTCAGCAGAAGAACTGCTCGATT
ATGTAA

>YNL189W 0.71 Cold
ATGGATAATGGTACAGATTCTTCCACGAGCAAGTTCGTTCCCGAATATAGACGAACAAACTTTAAGAATAAAGGC
AGATTCTCTGCAGATGAACTTCGTCGTCGTAGAGATACACAACAGGTCGAATTAAGAAAAGCAAAAAGAGATGAA
GCTTTGGCCAAAAGAAGAAACTTTATTCCCCCAACTGATGGCGCTGATTCTGATGAAGAAGATGAGAGCTCCGTT
TCTGCGGACCAACAATTTTACAGCCAGTTGCAGCAAGAACTACCACAAATGACTCAGCAACTTAACTCTGATGAT
ATGCAAGAGCAATTGAGTGCTACTGTTAAGTTTAGACAAATTTTGTCTAGAGAACACCGCCCTCCAATTGATGTC
GTCATTCAAGCCGGTGTTGTTCCAAGATTAGTAGAATTTATGCGTGAAAACCAACCTGAAATGTTACAATTGGAG
GCTGCTTGGGCTTTGACTAACATTGCATCAGGTACATCTGCTCAAACAAAAGTGGTTGTTGATGCTGACGCTGTA
CCTCTTTTCATTCAACTATTATATACCGGCTCCGTTGAAGTTAAAGAACAAGCCATTTGGGCCTTAGGTAACGTT
GCAGGTGATTCAACTGACTACAGAGACTACGTTTTACAATGTAATGCCATGGAGCCAATTTTGGGTCTTTTTAAC
TCCAATAAACCATCTTTGATCAGGACCGCTACGTGGACTTTATCCAATTTATGCAGGGGTAAAAAACCACAACCA
GATTGGTCAGTGGTCTCACAAGCGTTGCCAACCTTAGCGAAATTAATCTATTCGATGGACACTGAAACTTTAGTT
GATGCTTGTTGGGCTATCTCTTATCTATCTGACGGACCACAAGAAGCTATTCAAGCAGTGATCGATGTTAGAATT
CCTAAAAGACTTGTTGAATTACTGAGCCATGAATCGACTTTAGTCCAGACTCCTGCTTTAAGAGCTGTAGGTAAT
ATAGTCACTGGTAATGACTTACAGACTCAGGTCGTTATAAATGCTGGTGTCTTACCTGCGTTAAGACTTCTGCTA
AGCTCTCCAAAAGAAAATATCAAGAAAGAAGCGTGTTGGACCATTTCCAATATTACGGCTGGTAATACTGAACAA
ATTCAAGCGGTAATTGACGCGAACTTGATTCCTCCATTAGTTAAACTTTTGGAAGTTGCAGAATATAAAACTAAA
AAAGAAGCTTGTTGGGCTATTTCCAATGCCTCTTCAGGTGGTTTACAAAGACCAGATATCATAAGATATTTAGTA
TCTCAAGGGTGTATAAAACCATTGTGTGATTTGCTAGAAATTGCTGACAACAGAATAATTGAAGTTACCTTAGAT
GCTCTTGAAAATATTTTAAAGATGGGTGAAGCTGACAAAGAAGCTCGTGGTTTGAATATCAATGAAAATGCCGAT
TTTATCGAAAAGGCTGGTGGTATGGAAAAGATTTTCAACTGTCAACAAAATGAAAATGACAAGATTTATGAAAAA
GCATACAAAATCATTGAAACCTACTTTGGTGAAGAAGAAGACGCCGTAGACGAAACTATGGCTCCACAAAATGCC
GGTAATACTTTCGGCTTTGGTTCTAATGTCAACCAACAATTCAATTTTAACTAA

>YKL170W 0.64 Cold
ATGATATTTCTAAAATCTGTCATCAAGGTAATCGACAATTCAGGTGCACAATTAGCAGAATGTATTAAAGTAATA
AGGAAAGGGTCCCCCAAGAGTCCTGCAATGGTTGGAGACAGAATAGTCTGTGTTATACAGAAAGCAAAGCCCTTG
ACTCAAAACATTACGGGGACAGCCAACACCAACCGTGTCAAAAAAGGTGATATTTGTCACGCAATTGTCGTAAGG
TCTAAACAGCGTAACATGTGCAGAAAGGATGGCTCCACCGTTGCATTCGGAGATACTGCTTGCGTTTTGATTAAT
AAAAATACCGGTGAACCTCTGGGGACAAGAATTATGGCTAATGATGGTTGTGTAGATAGAACACTGAAAGACAAG
GGATACAATAAGATATGCTCTTTGGCAAGTAGGGTCATATAA

>YER007W 0.81 Cold
ATGACTTATGAAATTGGGGACAGACTCAAGATTGGTGGATATTTTTGCACCATCAAATTCATAGGTGTTATTAAG
CCGTGGCCTTCGGTAAAAGCTTATGGGGTGGAATGGGACGATCACAGCCGGGGAAAACATTCTGGAACAATAGAT
GACATACACTATTTTGACGTTCAAATTCCTAATTCAGGATCATTTTTGAAGGAGTCTAAGATTAAATCTCCAGGT
GTACGCAGAATTACGTTTTACGAAGCATTGTCAGAAAAGTATGGAGGCTCGAGTAATAACATTAATGACCTATCT
ATAGGTAACAAAAGGGTTGAGGGTTTGGGATTCGATGAACTAAATGCGAGAAATAAGAATTATAAGAAGTTAAGA
AAAATAGCTTTGCGAGATTCCGATGTCGCTATATTGTTTCAAAATCAAGATGAGTTAAACCGTGTAATACAAAAT
TGTGTGAACGTCAAGGACCTTGATTTATCGCTTAATCTATTCACGAATATCAACTCTTTATGTGAGTTCATAGAA
CCTTTGAAGAACTTGGAGAGCCTTAATATATCTCAAAATAAACTTTTAAGCGGTTGGGACAACTTGAAAGAATAT
GACCTATCACACATCAAAACTTTACGTCTGTCTTCCTGTGGTTTAAGTTATAAACATATTGGTAAGCTGCTCAAA
AGCTTCAGGACACTGAAAATGTTGGATTTAAGCTATAACAACTTGACCAGTGCGGGAATCCAAAATTTTGAGAAT
GAAATACCGTGTACTTTGGAGGAACTGAATATTAGCGGAAATAATCTTATATCGTTTCCATTGTTTCCAAAAAAT
TTGACATTGAAAGGTTTAAATGTCTCCAATAATCAAATTTCTAGAGCACCAAGTATTGCTATTTATTCTGTTGAA
TCCTTGGATATAACAGATAACAAATTTAAGGAAAGAAGTTTGATAGATGATTTGAATAAGACATTTCCCTCTTTG
AAAAATATCCATCTGAGTGGCAACGAATTCAACTACAACGGAAATTATATTAACGTCGAAGAACAGGCTACTTTT
TATGAAGTACTGGCTAGGTTTGATCGTGTGATGGTATTGAATGGATCAATATGTGATGTAAAAACAAGAAGAGAG
GCTGAGATGTTTTTTGTTTCCAAGGTAATGAATAACGAATTGAGCTATGACACCAATCTGCCGCGTTGGTCAAGC
CTCATAAAGTCGTATGAAATCGATATGAGTAAGCTAAGTTTCAACAATGAAAGAGAAACACGGCAATCTCTTGTC
TTAAAAATAAAGATAAGAGCTGGCAAGAAGCCGAGTAGTGATTTAGATTATTGGGTCCTTCCCAGTTTTACAGTA
AGGTACGTTAAGAGTGTAATATGTCGAAAGCTAAACTTTGACATACTGAATGTTAAGTTATTTCATGAAAATTCT
GAAGGGATGATAAACGAAATAAAGTATAATTTTCGCCCCATTTCAGATTTTAATGTTGTGAACGGAGATATTATT
CATGTTTCGTCTCCAGTAAATAACAAGAGCATTCAGAAGGTTAACAGCCCATCGTAA

>YOL007C 0.79 Cold
ATGAGACTGCCAGAAATCTCCATTTGGAAAGTGATACTACTACTTCATTTGTTTGCTCTACAAGAATTTCAACTT
GTCTCCGCTGCAAATTTGCCCTCTTTATCAAGTAGTACAAAGGCAGCAGATAGTTCTAGTAAAGGCAGCTCTTCA
GCCAAGACAACCACGTCCTTAGGCAAAAGCTCAGTAACCAGTAAGGATGTTTCGTCAAGTCATAATGTCACTTCA
AGCACTAAAATGCCCAAAATCACCACGAGCGCTAGTACAAGCTTATACACCAACTCTAGTTTATGGAGCAACAAC
AGTGTAATATCAACTTCATCTATTACACCTTCCAGCGTTTATATCCCGGTTACGGACGGAAATAAATTCTTATAT
CAGGCTCATCATCCTAACGGTACTGTGTTCATTGCATTTGCTGGCTGTTTAGGTGCAATTCTACTATCACTGACG
GGTGCATGGATTGCATTGAATATAAAGTCATGGCGAAGTGCTAGAAAGGAAAATAAGCTGAGAAATCTAGAAAAT
CAATACCAACACGATCCTTTCTACTTCCAAACAAACATTAATGACGACGAAAGTGAAACATCTTCCCATTCTGAC

GACAGTGACATATCAGAAAAAGTTCTGAAAAATAACTCATCTCGTATGAGTTTGTATACTCTAGGATCCACTTCT
GTTTTGAATCTTTTGAACAATAAAACAGATGCCAACGATAACTTCAGATCATCTATGTTTATTTCACCCACCGAA
ATTCTGCAATCTGATGCCAACAACTCTAATACATGGTCTCAAAGCAATGAGAGTGCTATATATGATTCCTTGTCC
AGTACTCCAAGGGAACCTGGTGCTACCCAAATACTGGGAAAGTTTACAGACAGCACTAATCCATTCAATTATACA
TCATACAATTTAAGTCCCGACTCTGAAGACCGCTCTACTCCAAAATCAAATGTTAGTCAGGGTAAAGTAAAGAAG
TACCGTCCTCCCAGTGTTCATCTGGATCAACTGCTCGACGGTAAGGAATAA

>YNL210W 0.76   Cold
ATGAGTAACCAACACAGCCCTCAGCCATTTTGTTTGGACACCAAATTGGTGAAACTATTAGAAGAGCTCCAGGAG
GGAAAGCAATTCAACAATAAAAACATATTCCCGGAAAAAGCATTATATTTGAAGCTCGCTCTTGATTATTCTTTC
TTCAGAAAGAATTTACTAGAGTTTTGCGTCCACCTTGACAAGATAAAAGGAGTCATTAGACCAAACTATGACACT
ATATATATTTTGTGCCTGTTGGAGGTGGATCTCCTCAATCTGGTATTTACCGACAATATATTGGAAATATGTTTG
CCCAGGTTTGTTTCAAGGGAGGACTTGAGGGTTTTTAATAATACTTTTTACACATATCACGATAACCGCCTACGT
ATTCTCCAAGAAGACTTTTCTCAATTGTTCAAAAAAATCAAAACTAAGGCTTCTGTACTATGTTTTACAGTTGAG
GAAATTTTTCTGACAAACCAAGAAATTTTACCTCAAAACTCAACAGTGGCAGAACTGCAAAAGAGCACTAATAAA
GTACAGACAAATGGGCCGCAACGGCACGATTTCATAGTCACTCTAGAAATAAAACTGAACAAAACACAAATCACT
TTCCTCATTGGAGCTAAAGGAACGAGAATTGAAAGCTTGAGGGAAAAATCAGGCGCCAGCATAAAAATAATACCT
ATTAGTGATAAAATGACTGCACATGAAAGGAACCACCCTGAATCTGTTCAACAAACAATACTAATTTCGGGTGAC
TTATACTCAATTGCATTAGCCGTCACCAGTATAGAGTCTGCATTAATTACTTTGGATTTATAG

>YAL009W 0.82   Cold
ATGGAGCCAGAGAGCATAGGCGATGTGGGGAACCATGCCCAGGATGATAGTGCCAGTATAGTGTCCGGGCCTCGC
AGGCGTTCTACTAGCAAGACATCCAGTGCGAAGAATATACGGAACTCCAGTAATATCTCTCCAGCATCGATGATT
TTCAGGAATTTGTTGATACTGGAGGATGATTTAAGACGCCAAGCTCACGAACAAAAGATACTGAAGTGGCAATTC
ACTTTGTTCTTAGCGTCTATGGCCGGTGTAGGCGCATTTACCTTCTACGAACTTTATTTCACTTCAGATTATGTC
AAGGGCCTCCATAGGGTTATTTTGCAATTCACTCTTTCTTTCATTTCCATTACTGTAGTTCTTTTTCATATCAGT
GGACAATATAGAAGAACTATCGTCATTCCAAGAAGATTTTTTACCTCTACTAATAAAGGGATTAGGCAGTTTAAT
GTGAAGCTAGTTAAAGTACAGTCTACGTGGGACGAGAAATACACAGATTCAGTAAGATTTGTGAGTCGAACAATT
GCTTATTGTAATATTTATTGTTTGAAAAAATTTCTGTGGCTTAAAGACGATAATGCCATTGTGAAATTTTGGAAA
AGTGTCACGATACAATCCCAACCGAGGATCGGAGCTGTGGATGTGAAATTAGTCCTCAACCCCAGAGCATTTAGT
GCAGAGATTAGAGAAGGATGGGAGATTTATAGAGACGAGTTTTGGGCCAGGGAAGGTGCTAGAAGACGCAAACAA
GCGCACGAACTCCGACCTAAATCAGAATGA

>YKR087C 0.75   Cold
ATGTTACGCAACATCATCAGGTTCAAAGGATTTGGAAAAGGGACCAGTGGAGGATTTTTAAAACCTGTATCATTT
CGTGTTCAGTTAACGCGATGTTACCGCTATGACAATGGTCCTTCATATCGTCGATTTAATAATGGAGAGTATTCC
CAAAAATCATCGTTTAAAAGCATTCTTCTCGATAAGTCATCAAGAAAATACTTAGCTTTATTATTTGGTGGATGC
TCTCTCTTTTATTATACACATTTGGATAAAGCACCGGTAAGCGATAGGTCCAGGTTCATCTGGGTATCGCGTCCA
TTAGAGTTAACTATCGGAAATTACACATACAAATCAATTTGGAGACAAACGCAGCAGGAAATATTACCTCCACAG
CATCCGCTGTCCATCAAAATTGAGAATATTTTCATGAAAATTGTCGAAGCTGCTTATAAGGACCCTAGTGTAGAC
AACTCACTACTGGACGGAATCAAGTGGGAAATTCATGTGGTCAATGATCCAACTGCTTCACCAAATGCATTCGTT
TTACCAGGGGGTAAGGTCTTTATATTCAGCTCTATTTTACCTATTTGTGCTAATGATGATGGGATTGCCACTGTT
TTAGCACACGAATTTGCTCATCAGCTAGCAAGACACACAGCCGAAAATTTGTCGAAGGCTCCTATATATTCTCTT
TTGGGTTTGGTGTTATATACTGTTACTGGAGCTCATGCTATCAACAATATACTATTGGATGGGTTTTTGCGAATG
CCAGCATCAAGACAGATGGAAACCGAAGCTGATTACATTGGCCTGATGATAATGTCAAGGGCATGTTTTCAACCA
CAGGAGTCAATAAAAGTGTGGGAAAGAATGGCAAATTTTGAAAAGCAAATGAATAGAGGGGGTGTTGTGAATATG
GAGTTTCTAAGTACACATCCAGCAAGCACTCGTAGAATAGAGAATATGTCGAAATGGTTGCCAAAAGCTAATGAA
ATTTATGAACAATCTGATTGTAGCAGTATGGGTAACTATTATAAAAGTTTTTTCTCAATGTAA

>YKL013C 0.82   Cold
ATGTCACAATCATTACGTCCGTATCTAACGGCTGTTCGTTATTCCCTGGAAGCTGCGTTGACTTTGAGCAATTTC
TCTTCGCAGGAAGTGGAGAGGCACAACAGACCGGAAGTTGAAGTCCCTAACACTAGCGCCGAATTGTTGCTCCAA
CCGATGCATATATCTCGTAATGAAAATGAACAAGTATTGATTGAGCCCAGTGTAAACTCGGTGCGTATGAGTTTA
ATGGTGAAACAGGCAGATGAAATTGAGCAGATTTTAGTTCACAAGTTTACTAGATTTTTGGAACAGCGTGCTGAA
GCATTCTACATTCTGAGACGTGTACCAATACCTGGCTACAGTATTTCTTTTCTAATAACTAATAAGCATACTGAA
TCAATGAAAACTGGGAAATTGGTTGATTTTATTATTGAATTCATGGAGGACGTGGATAAGGAAATCAGTGAAATT
AAACTGTTTTTGAATGCCAGAGCAAGATTTGTTGCGGAAGCATATCTTGATGAATTTGTCTATTAG

>YGL131C 0.72   Cold
ATGCCTAAAGAAGAAGATTTCCAATTACCTAGAAGAAGAGAGGCTGCTAAAAACGTGAATTATAATGAAATGGAA
ATAGATACTAAATTAGTTCAACAAATACAAATAGCGGAAAAAAGTGGTGCTAAGACCAAGGGTAGCAATAGCCAA
ACTCCCAGGAATTGTAAAAGGACAAGCAATCCAGCTAGTAGAAACGAGAAATTCAAGTACCAAAAATTCCTTCAT
GATAAGAATACTTGCTGGAACTTTATCCCTACATTACCCCCTTCGTTCAGAAAGAATAGCAGATTCTCTAACATA
CTTGATCTAGATGATGCCATGATTGACTTGAAAAAGATGTCTCTTTTCAATACAGAATCTGTCCTTTTATCTGCG
AACGACACTATTTATATGATATCAGAGCCTGCTGGCGAGCCTTATTATGTTGGCAGAGTTGTAAATTTTGTTAGT
AAACCAGAATTTTCTAACACTATTCATGAAGCTATAAAGACAACATCGGTCTTCCCTGCTAAATTTTTTCAAGTT
AGAATGAATTGGTTTTATCGTCCTAGGGATATTCAAGAGCACGTAAATACTTTTAACCCAAGGTTGGTGTATGCA

TCATTACACCAGGACATATGTCCTATTTCATCATACAGGGGAAAATGTAGTATATTTCATAAAGACGAAGTTTTT
GATGTTCTTCCTAATGAGAAGGAATGCATTATACGACCCAATATCTTTTACTTTGATGAACTTTTTGACAGATAT
ACTTTGAAATATTACAAAGTTTACAGCACGGATAAAATATTAAATAAATGGAACAGTAAGTCGCCATTTCTTTAT
GTTTTGAATAGGAGATTTCGTTATATCTATACAGAACCGAAGTATCCATTGGAGAACGTTTTGAAGAAATACGTT
TTCCATGAACTAGAGGTAAACGAATTAAGTCCTGCAGACTACCAATGGGATAAAAGATGTCAGTTCTGTAAAGAA
TGGTGTATCCAAAAGGAGAGCCTTTCATGTGATGAATGCGGCGTTTGTGCACATCTATACTGTATGGATCCGCCA
CTTGACAGGAAGCCGAACAAAGACGTCGTTTGGACCTGCTTCAGTTGCTTACAGAAACAACAAGGTACTAAGGAC
TCGCATGTTAGGTTTCTAGAAGAACAAGCACTGGAACTGGATTTTATTCGCTCTGTAAGACAAAAAATTGAAGAA
ATTTCTAGCAAAGCTATCAAAGAGAATGTTGGTTATAATACCGAAAATTGCTGGTTTCAATACTTAGGAATATAT
TCCATTTCTCATATTGGTGACGCATTAAATGATTCTATGTTTTTTCCATATCCTTTCAAACCCTCTAGGGTAGGA
GTGAAGTATCAATGGAACGGATGTAATCATAATGTGCCCTGGAGGCGGAACTCGTATATAAGTGCAAATTCAGAA
GAAGAGCGAGGCTCAACGAAAACTTCGGAACTAGCATGGGTACTTGATGCATCAAAAATTACTACTCGAAAATTG
AGTGAATATATTGAACAATGCAAAAGCGAAATATGCCCTATTCTCAATGTGAGAGGTGAGACTTGTAATTTCATT
GATGTAGTCCTAAAGAATTTATTATTTACAAATTATGATACAGCAGAGGCATTTAAAAAATGTAAAAGGGAGCTT
TCCAGAAAATTTTTGAAAGAGCCAAGTTTTACCGCCGTGGAGATCCGAAAGTTTGAGGAAGCTGTCGAGAAATTT
GGGAGTGAATTACGCCCAGTGTGTGAGTATGTGGGCACTCAACCTATGTCGATGATTGTTAGATTTTATTACAAT
TGGAAAAAGACTGAAAGAGGCCTTACCGTGAGAGGGAAATTGAGTAAGTTATCAAAGAATAAAAGAAAAAAAGAA
ATCGCTAATCATGAAAATGACGTAGAGACAAAGTATATAGATGATTCTTCTTTTGATACTGAGAAACTCTCTTTA
GCAGAATCCTCCTTCCAATGCATGTTTTGTAAAACAGATTACTCCCCGATGTGGTATAGAGTTACTGGAGGTTCA
GATGATGAAAAGATCAAGATAAGAATGCAGACAGGAGTCAACGAAAAAACAGAAATTTCAGAGAAATCGCCTGCC
CATAGCAAAAAGAATGAAAAACTTGGCGCTCTTTGCATTCGTTGTGCTCGTATGTGGCGAAGATACGCAATTAAG
TGGGTACCACCGCTTGAAACATTGAGAAAAATAACGGGAACCTGTCAGAATAGTTTTTATTCAGCGATAGAAGGG
ATTATAGAGGAGAATAACACAAACAAGTTTACGTTATCTCCCTTTCAAGCTCATAATAAACTACTAGAATGGGAA
CTTGTTCAGGATTCAGAGCTAATTATCAGGCAAAGAATGAAAGTTTACAAGAACCCAAATTCATTTGTGAAAATG
AAGCGGTATTCCATGACATTCCACACGCAACTCTACAAAATGGCTGTGAGATCATATCGAAAGAATGAGTTTCAC
CCGGAGACGATGCAACGAGATCTTGAGCTATTTATAGAGGACAACAAAGAGGTAAGAAAAGCCATCCCTGAACAA
AAGCCAGAAAGAGCGAAAAACACAAAGGACGAATTTCCCGTGAACATAATTCGACAATCACCTGGTACTATCAAA
ACAAGTGATACAAGCCGAAATCGCAAGTGCAACGATGTATTTATCGAGAAGGCTAGTAATAACAATATTCCTAAA
ATCACTAATGCTTCGAATGATTTGATAGAAATTTCAATTAAAACAGGCGGCAGTTCTTCTGGTTCAGTGTCTGTA
GATAAAGGTTTTAAATTTGTCAAATTTGATAACAAAACATTTCAGCGATTACGGAATAGCTTAAAGTTAGTGAAT
AATAAGCTGCCAAAATATAATGAACCTTCAACGAAGAAGATCAAAATGATCAATGACATAGCGTTAAGTAATCCG
TTAAACGAACCAAACGGGGCATCGTACAATTATACAGTTATTTCACATTCTAAGGAAACTTCTGTCGCCCTTGAA
AAATACCATGATGGTAATAAACCGAGCAAAATGTTAGAGAAAGACATGATTTTGAAACACACTAAGAATAAACCA
AAAAATCCTGATACAGCATGGGCAAATAATAGCGCAAGGACTTTTTGTAGTGTCTGCAAAGAAAAATTTAACGAC
AATGACAACTATGAGGTAGTATGTGGTAATTGTGGACTTACAGTACACTACTTCTGTTATGCTATTAAACTGCCG
AAAGATATGAAGAAAAACACTAACCTTAAGACATTTAAATGGTTATGTGACCCTTGTTCAAATGACCTCAATCCT
ATAATATCAACGACCTACCAATGTAGTATGTGTCCTACGAAGGACTATGACTATGATAGATATAGGAGCCAATCA
TTTAAAATATGTCCAGACGCATTGAAATGCACGAGCTTAGGGACATGGGTGCATTTGGTTTGCTCACTTTTCAAT
GAAGATATCAAATATGGAAACGGACAATCAATGCAACCTGCCCTTAATACCACTGCTGTGCTTATTAAACATAGT
AGATTTACATGTGGTGTGTGCAGGATAAATGGAGGTGGGTTAGTGAAGTGTAACAAATGCCAATATAGGTATCAT
ATTACATGTGCACAGAACAGCTCCAATTTTAAGCTTATGTTTGAAAAGAAAAATATGTCTGTAGACACCACTCTA
CCGTGCATAAAGGATGTCAAGCTTAACGATACATACACTTTGAGACCTATACTAATATGCGATAGACACGATATC
AGCTTAGAAGGAAACGAACTTTATCCATTATCGTACAAGCCTCAACATACTCTGTCATACATAGAACAATACTGC
AGATATTATAAGTGCGAATCTGACCATAGTCTAGTGGAGTTGAGATATTTTGAACAGCTTAGACTGCGACACGGC
GAAATGCCAGGAAACTCACACGACTCTGCTATTAAACCCAAAATTTACGTACTTCCTTTCGAAAGGACTTGCCCA
CATTGTGGTACAAACAAAAGTTTATACTGGTATGAAGATATTATTTGCCATTCTTGCAATTTGAGATCAGGTGCC
CAAGAACTCGATTTTGATAGCGCATCTGCTAATATCTCTAACGATAACGGTTTACCAGTCGAAATTACCCAGCAA
TTGATGGAAGGAATTGAGCCAGCTATGTTTGATATCGATATTTCAGAAGCTGGTACCGACAAAAATACGCATCCA
TCAAGCCAGTAA

>YGL094C 0.79 Cold
ATGAATAATTGGCAACATTTCTTCAACAATCCAGTTGATCTTTCGGAACATTTGAAGAAGCCATACTTTCGCTTC
GATAATAGGGATAAGGAAATTACAGCGATTAGCTTCGATGAGAAGGCAAACTTAATTTGGAGTGGAGACAGCTAT
GGTTGCATTTCGTCATATGATCCAACTTTTCAACTTTATACAAGATATAGGGGCCACATAGGTGGAAATTCCGTG
AAGGATATTCTCAGTCATCGGGATGGTATTTTATCTATTAGTGAAGATTCCTTACACTTTGCTAATAGAAGAGGT
GTTACTAAATTGAACCTCACTAGCATTGATATTGCTGCATTTAGCGAATTGAACACTATGTGCTATTCTCCTCAT
TCACTGAAAAACAATATCTACTGTGGTGGTGACAACACAAATTGGGGAATTGCGTCCATTGACTTGAACAGAGGT
TGCTTAGATTCCCTCTTGAATTACTCATCTAAAGTGAAGTTAATGTGCTCTAATAATAAAGTTTTGTCTATCGGA
AGACAAACAGGGACTGTGGATTTGCTAGATCCAACATCGAATCGTACTATCAAATCATTTAATGCACACTCTGCA
TCCATATCCGCTATGGATTTACGGGATAACACCTTGGTTACAGTAGGGAAGTCCAAAAGATTTTATAACTTATAC
GCTGACCCATTTGTGAATGTTTACGACTTGAGAACAATGCGTCAACTCCCTCCTGTTTCCTTTTCTAAAGGAACA
ACTATGGGATCTGGAGGCGCAGATTTTGTTCAATTACATCCTTTGCTTCCTACTGTTATGATCGTCGCCTCAAGT
TCTGGTTCGTTTGATTTCATCGACCTTTCCAATCCAACTTTAAGAACACAATATGTTCATCCTTGCCAGTCGATT
AAAAAGTTATGTTTGTCCCCCAATGGTGACGTATTGGGTATACTAGAAGCTGATAATCACCTAGATACATGGAGA
AGATCATCAAACAACATGGGAATGTTTACCAATACCCCTGAAATGCTAGCATATCCTGATTATTTTAATGACATT
ACCTCTGACGGCCCAATATCTGTCGACGATGAAACATATCCATTGAGTTCTGTGGGGATGCCGTACTATCTTGAT
AAACTTTTGTCTGCATGGCCCCCTGTAGTGTTTAAAAGTGAAGGTACCATACCGCAATTAACAGGTAAGTCACCC

TTACCATCGAGCGGCAAATTAAAAAGTAACCTTGCTGTGATCTCGAGCCAAAATGAGAAGTTGAGCACACAAGAA
TTTCCTTTGTTAAGATATGATCGCACCAAATACGGTATGAGAAATGCTATACCAGATTACGTTTGTCTAAGAGAT
ATAAGGAAACAGATAACAAGCGGTTTAGAAACCAGCGATATACAGACATATACCTCAATCAACAAGTACGAAGTA
CCCCCTGCATACAGTAGACTTCCACTGACATCAGGTAGATTTGGTACTGATAATTTTGATTTTACGCCCTTTAAT
AACACTGAGTATTCAGGATTGGATCCAGATGTTGATAATCACTACACAAATGCTATCATACAATTGTATCGCTTT
ATTCCAGAAATGTTTAATTTCGTTGTTGGGTGTTTGAAAGACGAGAATTTTGAAACAACGTTGCTAACTGATCTA
GGCTACCTCTTTGACATGATGGAAAGATCACATGGAAAAATATGTAGTTCTTCCAATTTTCAGGCGTCATTGAAA
TCCTTAACTGATAAAAGACAATTAGAAAACGGTGAACCACAAGAACATTTAGAAGAGTATTTAGAATCGCTGTGC
ATAAGGGAAAGTATCGAGGATTTTAATTCTTCTGAAAGTATTAAACGCAATATGCCTCAAAAATTTAACAGATTC
CTGCTCTCGCAACTTATTAAAGAGGAAGCGCAGACAGTCAACCATAATATCACCCTAAATCAATGCTTTGGTTTG
GAAACGGAAATACGAACAGAGTGTAGCTGTGATCACTACGACACTACCGTCAAACTTCTACCCTCCTTATCAATA
TCAGGAATCAACAAAACCGTAATCAAACAATTGAACAAGAAAAGCAATGGACAGAATATTTTGCCTTATATTGAA
TATGCCATGAAAAATGTAACCCAAAAGAACAGTATTTGCCCAACCTGCGGCAAAACCGAAACTATCACCCAGGAG
TGTACTGTCAAGAATTTACCTTCAGTGTTGTCATTAGAATTATCACTATTAGATACCGAATTTTCCAATATAAGG
TCGTCGAAAAACTGGTTAACTAGTGAATTTTATGGAAGCATCATTAAAAACAAGGCAGTTCTAAGATCGACGGCG
TCCGAATTGAAGGGCACAAGCCACATATTTAAATACGAATTGAATGGTTACGTGGCTAAAATCACTGATAACAAT
AACGAGACGCGTCTAGTAACATATGTCAAAAAATATAATCCAAAAGAGAATTGCTTCAAGTGGCTCATGTTTAAT
GATTATTTGGTTGTTGAGATAACAGAGGAAGAGGCGCTTAAAATGACATACCCTTGGAAAACACCAGAAATTATC
ATATATTGTGATGCGGAAGAATTACGAAAACCTTTCTTTTCTGTTGATACGTATTCCATCAACTATGACATACTT
TTCCGTGATTATTTCGCAAACGGAATAAGAGATACTGCAAGACGTGAATATAAGTTATTAACACATGATGAGGCA
CCTAAATCTGGAACCTTGGTTGCCATTGATGCCGAATTTGTCTCATTACAAAGTGAACTATGTGAAATCGATCAT
CAAGGAATCAGAAGTATTATTCGACCTAAAAGAACTGCTTTGGCCAGAATATCCATTATTAGAGGCGAAGAAGGA
GAACTGTATGGAGTACCATTTGTCGATGATTATGTGGTAAACACGAACCACATAGAAGACTATTTGACAAGATAT
AGTGGGATTCTTCCTGGTGACTTGGACCCTGAAAAGAGTACCAAAAGGCTTGTGAGAAGAAACGTTGTATATCGA
AAAGTCTGGCTTTTAATGCAGCTCGGATGCGTATTTGTTGGTCATGGTTTGAATAATGACTTCAAACACATTAAT
ATTAATGTCCCAAGAAACCAAATTCGCGACACTGCCATATATTTTCTACAAGGAAAGAGATATCTTTCATTGCGT
TATCTGGCATATGTGTTGTTAGGAATGAATATCCAAGAGGGAAATCACGATTCAATTGAAGATGCCCATACTGCC
TTGATTCTTTACAAAAAATATCTCCACCTGAAAGAAAAAGCTATCTTTGAGAAAGTACTGAACAGCGTGTACGAA
GAAGGAAGAGCCCATAATTTCAAAGTTCCAGAAACTTCAAAGGGATAA

>YPL154C 0.82 Cold
ATGTTCAGCTTGAAAGCATTATTGCCATTGGCCTTGTTGTTGGTCAGCGCCAACCAAGTTGCTGCAAAAGTCCAC
AAGGCTAAAATTTATAAACACGAGTTGTCCGATGAGATGAAAGAAGTCACTTTCGAGCAACATTTAGCTCATTTA
GGCCAAAAGTACTTGACTCAATTTGAGAAAGCTAACCCCGAAGTTGTTTTTTCTAGGGAGCATCCTTTCTTCACT
GAAGGTGGTCACGATGTTCCATTGACAAATTACTTGAACGCACAATATTACACTGACATTACTTTGGGTACTCCA
CCTCAAAACTTCAAGGTTATTTTGGATACTGGTTCTTCAAACCTTTGGGTTCCAAGTAACGAATGTGGTTCCTTG
GCTTGTTTCCTACATTCTAAATACGATCATGAAGCTTCATCAAGCTACAAAGCTAATGGTACTGAATTTGCCATT
CAATATGGTACTGGTTCTTTGGAAGGTTACATTTCTCAAGACACTTTGTCCATCGGGGATTTGACCATTCCAAAA
CAAGACTTCGCTGAGGCTACCAGCGAGCCGGGCTTAACATTTGCATTTGGCAAGTTCGATGGTATTTTGGGTTTG
GGTTACGATACCATTTCTGTTGATAAGGTGGTCCCTCCATTTTACAACGCCATTCAACAAGATTTGTTGGACGAA
AAGAGATTTGCCTTTTATTTGGGAGACACTTCAAAGGATACTGAAAATGGCGGTGAAGCCACCTTTGGTGGTATT
GACGAGTCTAAGTTCAAGGGCGATATCACTTGGTTACCTGTTCGTCGTAAGGCTTACTGGGAAGTCAAGTTTGAA
GGTATCGGTTTAGGCGACGAGTACGCCGAATTGGAGAGCCATGGTGCCGCCATCGATACTGGTACTTCTTTGATT
ACCTTGCCATCAGGATTAGCTGAAATGATTAATGCTGAAATTGGGGCCAAGAAGGGTTGGACCGGTCAATATACT
CTAGACTGTAACACCAGAGACAATCTACCTGATCTAATTTTCAACTTCAATGGCTACAACTTCACTATTGGGCCA
TACGATTACACGCTTGAAGTTTCAGGCTCCTGTATCTCTGCAATTACACCAATGGATTTCCCAGAACCTGTTGGC
CCACTGGCCATCGTTGGTGATGCCTTCTTGCGTAAATACTATTCTATTTACGATTTGGGCAACAATGCGGTTGGT
TTGGCCAAAGCAATTTGA

>YDL044C 0.79 Cold
ATGATCAGAACATCATCTATATTAAAAAACTGTAACTATAGATACATACATTGCATCCATAGATGCTTGCTGAAT
GAAGCCAATTTGAAAGATAGAAAAACTCACAACGTAGAAAGAGTTTCAAATGAAAAGACATTTGAACAAGCTCTC
GAAGAGGAACGTAAGGTATTTGGAGAACTATTCGAAGCTGGAGCAAGGGTTGAAAACATGAGGCACACTAATGCT
AGCAAAATAATTGACAAGTATTACAATGGGCTACAGGACAACTCCGAGGGAACTAGTGTAAAAAAAGAAAAAATT
GTTTTTAACCACAGTCAAAGAGCTCAAAGGAAGCTTCCAAATAAGGACCATGAATTCCTGAAGGAAACAGCAGGC
AATGACTATGTCTACGAACGGGCAGAACCTTCTGCTATTAGCACTAAAACGATAAGTGAGCAGACAAGAACTTTG
CTGGAAAAGATATTTGACGAAGATAATAGCATTAATAAAAGCAACAGAGAATTGCTGAATCTTAATCTGCGCAAG
GGCTCGGGAATGGAAGCTTTGAGGCAGCCAGTTGCACATTCCAACGTCAAATTTAGCGAGGAAGTGATGCAAGAA
ATTGGTAATAAGATTCGGTACCAAACCACACTGGATCAGGTTTTAGAACCTCACATTGACTACTTGAGGGAAGCT
GTTAAAAGTGATTACGATCTTTTAAGATACCTAAAGCAATCACTAGACATTTACAAGAAAAGGAACAAAGACCTA
GAGTTAAAGATGAATGCAGAATCTTCAAACATTTTCGAAGACATTAGAAGTGCGTGTATAAATAAACCGGCAGAA
CTACCGAAACCGCTTGCAATGACTTTACCATATATTATAGTCAAATCGCTTAGATTAGGTGACTTTGATTTCCCG
GCAGACAGAAAGTACACATTGATCTCCTATGTTTATAATGAATGTAAAAACAACATGGACGCTTCACTGTACCTA
ACAATTTGTAATGTTGATTTCTACAATTTGCTGGTTCAGCTATTGTGGGAAAACTTCCAGGAGATTCGCTACTTG
AGACGTGTGGTTACCGAGATGAGCGTTAATGGTGTCATCGGAAATATTGAAACCGTGGATATATTGGATAAGATT
GTCAAAGAAATGAGAAGTTTAAATGAGGATGTCTTTCTTGAAGCAGGAGAACAGCTCAGTGCAGATGAGGAAGTT

TCGTCCAGCGCAAATAAAATAGTGAATGTAGGTGTTTTATGGAATAAAGACACGAATAATGACTTACTAATTGTT
GAAAATTATCTCAAAAGCCTCAAAAAAAATTTAACTAGAGACAGGTAG

>YKL221W 0.81 Cold
ATGTCCGAAGAACGGCATGAAGATCATCATAGGGATGTTGAAAATAAATTGAATTTAAATGGTAAAGACGATATT
AACGGGAATACCTCAATCTCGATCGAGGTGCCTGATGGAGGATATGGGTGGTTTATTCTTCTTGCTTTTATTCTG
TACAACTTTTCTACTTGGGGGGCAAATTCTGGTTATGCTATTTATTTAGCGCATTATTTAGAGAATAATACTTTT
GCTGGTGGGAGTAAATTAGACTATGCTTCTATAGGTGGGTTAGCATTCAGTTGTGGACTTTTTTTTGCCCCAGTT
ATAACATGGCTTTATCATATATTTTCAATTCAATTCATTATAGGCTTAGGGATACTGTTTCAAGGGGCAGCGCTA
CTGCTTGCAGCTTTTTCTGTCACACTCTGGGAAATTTATCTCACGCAAGGCGTTTTAATTGGATTCGGTTTAGCA
TTTATTTTCATACCCAGTGTCACACTCATCCCACTATGGTTCAGAAATAAAAGATCTTTAGCCTCTGGTATAGGA
ACTGCTGGAAGCGGGTTAGGTGGTATTGTCTTTAACTTGGGAATGCAAAGTATTCTACAAAAGAGGGGCGTTAAA
TGGGCGCTCATTGCTCAGTGCATAATATGCACATCACTTAGCACCATTGCGCTTATGTTGACCAGAACAACACAT
CAAGGCCTACGTCAACATAAGAGATCTTACAAATTTGAATTGCTAGATTATGATGTGCTTTCAAATTTCGCGGTC
TGGTTACTTTTTGGATTTGTATCATTTGCTATGTTAGGATATGTTGTCCTTTTGTATTCCTTGTCTGATTTTACC
GTTAGTTTAGGTTATACTAGTAAGCAAGGCTCATACGTATCGTGCATGGTGAGTGTCGGCTCTCTGCTGGGACGA
CCAATTGTGGGTCACATTGCTGATAAATATGGATCACTAACAGTTGGCATGATATTGCACCTTGTCATGGCCATC
CTTTGTTGGGCCATGTGGATACCTTGTAAAAATTTGGCCACTGCGATACGTTTTGGATTATTGGTTGGTTCTATT
ATGGGAACAATTTGGCCAACAATTGCTTCAATTGTTACACGCATTGTTGGTCTTCAAAAGCTTCCTGGTACCTTT
GGTAGTACCTGGATTTTTATGGCGGCTTTTGCCTTAGTTGCCCCCATAATCGGTCTGGAACTTCGTTCAACTGAT
ACGAATGGAAACGATTATTATCGTACAGCAATATTCGTGGGTTTTGCGTACTTTGGTGTTAGTTTATGCCAATGG
CTATTGAGAGGGTTTATAATAGCTCGAGATGAGATTGCTGTGCGTGAAGCCTATTCAGCTGACCAAAATGAATTG
CATTTAAACGTTAAGTTATCACATATGAGTAAATGTCTTTTTCGTTATAAACAATTACCTAGGAGAGTCTAA

>YBR045C 0.79 Cold
ATGGAAACTATTTTGCAGCCAAAGGCTAGACCATTTGAGTCTTTGAAAAGAAAACGTTTTAGAGAATGGTTGAGG
CCGTCGACTGCGCATGGATCCCTGTTGCATTCTGATACATTAGATTTGCGTGACTTTGCAAAACCTAATCCCGCT
GACACATTTTCTAATCTTGATTCTGGTCATTGTCCTTTGGTCACAACTCCAATAAAATATGAGTGCCCAGATGGA
AAGAGTTCTTTTTTCCGAGGAGACACTAAATTTGAAACCCTGTTCAGTAATAGAAAATTCTATGAGTTCAAAGAT
AATTTGAAAAGGGGATTGAAGAAAATACGTCATGGGAGAAACGGACATCAAAGCGAAAAGAGATGTCCAGTTGTT
GAAGAAACAAAAAAGTCTGTGTCAGATAATCTGGACAAACCAGACAATAATACGCCCTGTTTCGACAGATTCCAC
ACAAATTCGAAAGAATTTGAAACGCAATTTGATCATTCAAATAGGAGCCAAAATTCCGAGAAGGCTTATCTAGAC
AATGAATCCTGTTGGAACCTAAGTGAGAAATTTATTCCTTTTAATAATTTAAAATATGAAGATTTGAAACATTTT
GAAGAGAATTTGCAAAGCTTAGCGCCTGCAACTTTTACTCCAATTGAATCAAATGAATCGCTTGATAGGTCAGAT
TCGACACGTGGCACAAAACGAAGCATTCGCAATGATTCCAGTGATACAACATCTGAAAAGAGGCTATGCTTAAAA
CAATACTCAGATGAACCTGAATCGGATCATTCGATGGAAAGTACACCATCCATTTACATTACCAAAGAAGTTCAA
GAAAGAATTGAAGCATTAAGCTCCACGGATTCGTTTTTAATTGAAAAAGTAGATTTTCCCTCTAACAAAATTGGT
TCCAGTGCCTCCGATTATGAAAGTGATAACGAATACAGAAATATGGATGAGGATTCAATAAACGATGTTACCACT
GAGAAAGAGGGAAATGTGGTCATACCAGACTCTAATACTAGCACGGTGGACGCAATGGAAAAACCAATTGAAGTG
AGCTCGGCCTTAAAAGATGATACATTGGATAAAGACATAGATGATGCGAGCAGTAGCTATTCGGATGATGTAGAG
ACCACATTCGAGCCAGTTGAATCTGAGGAACTTTCCGATTTATCTGATACAAGCTCAAGTGGAAGTAGTAAAATT
TATACTATCCCCACATTTCGCGGCCTTACTAATAGGACCAATATATCACAAATTCTTTCAAAAGTTGGTAAAGCT
GATTTAAGCCAAGATAACTTAACGCATTTGATCAAAAGTCATCAAAAAAAGAAAAGATGCGTGAATTTTAGAAAT
AAAAGATTCTATGATGCTTTCAATCCATATGTTGATAACGAAGAAGATGCAGAGTTATCTGACAGCGAAAACATT
TCAGAAATGGATACAGATCTTTGTATAAAGGATCGGAGTACCTCAAGTGTTAGGTTTGATGAAAACTCACGTCTT
TTAATCTACAAAAAATCTAAAAAGTTAAACAAAGATGAGACTCAAAGTGGTTATTCGACTACTGAAATGAGGTCA
ATTCTAAAGACAAAGATGAATTCACAGCATGATGAGGAGTCTCAGAGAGCTTCGAAGTGTGACACAGTAGGTGTA
GCCCAATTTTTACATTATTTTCAATATACAGAGTATAAAAGGCAGAGAAATGAAGCAGAAATTATAGACTGA

>YPL216W 0.76 Cold
ATGGTTCTGCTTAACAGAAGGAAAATACAACCTAAGGAAATAGGACAGTCTGCTGATTCCTTCTCTGAAACTCCA
TGGGTGATTAAAGAAAGTAGCGAGCGTATAAATGATTATGACAGTGATTTAAAAAAGCTAGATTTTTACAAAAGG
GATATATTCACCTGTGAGATTAGTGGAAAAGATGGCCTTTCGTATTTCAAGGCGTTAAAAAGCGAAGAACAGCAC
CGTGAAAAGGTCAGATATCTTTTACCAAAAGAACTAAGGAAGGCTATAGCAAATTTCGCAAACTTCAGTCCGATA
AGGAAGGTAGGACACCTTGTAGAAAGCGCATTTCAGCGTTTTAGTAACCGTTTCTTTATAGGCGATACGGTGTGC
TTGAAAACTATCCAAAAAAATGCATTGATTACCTATAAAGAAGGAGAGCCTAACTTGGTAGAATCACCAACTATT
GAAAACAATGTCACGTTGTTCCTTGTCAAGGATGTGTTTCAATCTAACGGGATGATGGAAAGTGAAAAGGGAGAA
ATTTCTGCCCCAAAATTGTCACTGTATTTAATCACAGAATGCCTGAATCGAGAATCTAAAGGTGCGGCCCTAATT
GTAGGCCAGAATGAAATTAAAAGGCCAGAATCACACTTTTCTAAATTCATAATTGCGTGTTTTCTTAATGAAATA
CTCATAAAAGTATCGAATAAGGAGCACGCACCATGGAGGGTGAAACAAGAATATATAGAAAGATATGATGTAAAC
CCTAAATGCTCTCCTAATATGATTGATTATTTGCCGGATAGAATGAACTCATCTTCTTCCGAGCTTTACACTCCT
CTTACTATACCACCAGAGAGCGATGTTGAACCTGCTGACTGGAAGGAAACGTCGGAAACGTCGGAAACGTCGGAA
ACGTCACTATCTAAAATAAAAGCAATTGACGACGAGATTTCAGTCTCATTTGACCACATTTACGATAATGTGAAC
TCCCTTGCCTACAATGATTTGAAGGGAACCGTCGACGACAAGGAGCTTCCATTCACTGGGCCTTCGATACCATTT
GAAAATATCAGCTACTTAGATAGCTCCTTAGAATATAAGAATATCGATCAAAAATGGTTCAAAGAGTGCAGTCAA
TTTCCAACGGAACGACTATTGGTTGTTTATCAATTCCTGAGCTTTTTTGGACGTTTTATTGGTCTATCACATTTT
AATTTTGATCAGTTCCTTACTACTATCAAGTGTACTAGTCCGGAGGCTTTAGTAGATGAATATGTCAAAATAAAC

TTTCTTAAAACTTACAACAGCAAAGGGAGTTTCACAAATGAGAAGCCAAGAAACGAAATATATAATCAAGTGACC
AGTAGTAACGTATCCCAGCGTGAGAAGGCTAACGTCTTTAATGCAGATGAGAGTCAACGAATACCTAGCAATTTC
ACCAGAAACCAGAAAATGAGGAAATTTATAACGGATAAAAGTACTGAATTTGTAATGTATTCCATTTTTAAAGGG
AAGCCTCTAAAAAATGACGATATGGAGTTTCAATCGTACGAAAAAGTGAATATACTTTACATTGATATAGTTTGC
TCGTTAATGTGTCTGATGACGGATAATGAACCTGATTGGAATTGTAATCTGATGGACAATTGGACTGAAGAAAAG
CGAAAGGAGGAAGGAAATAAAACAGAAATTGATATAGCTATAGAAAAGTGTTTGAACTATGGAGATACTAGCTGG
GTTAAACTTTTGCACAATAAAAACTTTAGCAACGGCAATTGGTTGATCTGTCTCCTTGGAATCTTGCAACAGAAT
ACACATATGATAGCATATAGTGATGTCGCAAAATGTATCACAAAAAAAATACTACCTTTATCCATGAATTTTGTA
AATTTAGGTGATGAGCTCTGGGATAACTTTCGCAAGAGATTATCTATAAAAGATAAAATTGATGTTTTATGGGTT
CTCGTAGATTTTGCGTCCAATTTCTCGAGTTATATTAAGGAGTTGGTAGATAAAGTACCAAAACTGTGCAATGGA
ATCCGCTTAAAGTTGGATAGTGCAAAAAAAGAGTATATCAAATTGAAACGGCAACTAAAAACGTTAACCAAAAAT
CGCGTGAAGCTTCACAGCAACGTTTCAATGAACCGATATGGATCAGATGAATGTAAAGGTAAGGTCAATGCCCTT
AAAGTGAAAATTGCATACTTAATGGAAGATATCGCCTTCCTAGAGGCAAAATTGATTCAGAGCGATATCAAGAGA
CTAGAGATTTTGGGTAAAGATAGAAATGGAAATCGATATTATTGGATGGACTCAAATGGATCGTCATCAGCCATC
AGTGAAAAAAATGAGGAACTTTACAACTGCTGTTTTTTGTGGGTTCAAGGACCCTCTGAAGCGGATATAAACTTT
TGCTTAGATGTCGACGTCGAATCTTTGAAAAAATGGGAGTTGTTAGCTAAAGCAAAAGGTACTGCCTACGCGACA
AAAGAAGTCTTTTCAATATTTAGGTCGACAGACGGGTCTTACTATCAAATTGCTCAAGGTGAGAATTTCATGATA
ATTAATTCAAACGGTATCTTAATGAGGCCAACTATTCCGGCATTCATAGACAAAAAAATTATATCAGAAACTCCA
GAAAAGCTCCTACTCTCTCATCATCAATGGGCTTTCTTCAACGATATAGAAGATATACATATGTTAGTAGATAGG
TTGGATGATTTAAGAGAAAATGAGGGACAATTGAAGAAAGCTTTGACATCCAAAATGGACCGTATTGAAGTATCA
TACAAGCAACAGTTCAAAATTAAAAGGCGTATTGAATGTGATGAAACCTTCAAAAAAAAATCACAAACTTTTGAAA
AATAACGAGTTCACGTTTCCTGAACTGAAAAGAATTGAGACAACTTGTACATCCAACGGCCAACATTTTTCCAAC
ATGGAGAAAATTTCCAAAAAACTATCAAGGACAAAGAATGATCTCGTTTTGGAAGCGATTTTAAAAGACGTAGCT
CACCTTGGAGAATGTGAAAGAGCACTTCTCAAAAAGCAACAAAATTTGATTTATCCTTTGAATTTTCATTTCGAA
CAATTGAGGACGATAGACCTCGAATTTATAGTTGAAACGAAAAGAAAAAGGCAAGAGGATATTCTTACGAAGCTT
TTAAATCATCAGAGGTATAAGCATATTAGTCATGTTTCAGGCTATGGGATAAGTTCTCAACGAGTAGATAAAGCT
GCACATCTTGATGTGCAGGGTATATTAGAGGAGATTGAATGTCAACTTATTTCAAGACGACGAGAGGATGAAGAA
CGCAACTAG

>YPR136C 0.75   Cold
ATGCAAGTAAGGTTATTTTCCCCTACCCTGGTAAAGCTCTGTTTTGCTTCAGATATTAGAAGTTTATCGCCAAAA
TATCTGAAGACTCGGCCTTGCTGTTCAGCAACGTCTTCTTTTAACCTGGACGCAATGATATCCTTATCCAAATCT
CCAGCATCAAAATTGTTGTACTCGTCGATTGTTCTTTCCTTGAGATTGTTTAAATCTTTTTCTTCAGCGTTCCTA
TTATCAGTTAAAATGTCATTAGCTTCGCTTTTCAGATTCTCCAGATATTGCTTTGCAAGCCTCCTTCTTTTATCG
GCTGGATTTTCACCCTCGAATTCTTCTTCAGATTCCAATGCGTCTTCTTCATCCGAAACTTCTAGTTGTTCATCT
TCGTTTGAGGATGGATCTGTGATTTCTTCGTCTACTGTGGGTTTTGATGGATTAACTTCTCCTTTGGATCTTTTC
CTCTTTTTCTGTTGGGTAACATCTGACATCTTTTCTAGATTTTCTATAATTTTGAAATGCTAA

>YDR267C 0.82   Cold
ATGGCGTCTATCAATCTGATTAAGTCTTTGAAACTTTACAAGGAAAAGATATGGTCTTTTGACTTTTCTCAAGGT
ATATTAGCAACAGGTTCAACTGATCGAAAAATTAAGCTAGTTAGCGTGAAATATGACGATTTTACGTTAATTGAT
GTGCTAGATGAGACTGCTCATAAAAAGGCGATTCGATCTGTGGCGTGGAGACCTCACACTTCGCTGTTAGCGGCT
GGCTCTTTTGATTCCACTGTATCTATCTGGGCTAAGGAAGAATCTGCTGACAGAACTTTCGAGATGGACCTGCTA
GCTATTATCGAAGGTCACGAAAATGAAGTAAAAGGTGTAGCGTGGTCGAATGATGGATATTACCTAGCAACCTGT
TCCAGAGATAAAAGCGTGTGGATATGGGAAACAGATGAAAGTGGCGAGGAATATGAGTGTATTAGTGTTTTGCAA
GAACATTCACAAGACGTTAAACATGTTATATGGCATCCATCTGAAGCATTACTGGCTTCAAGTTCTTATGATGAC
ACCGTCAGGATATGGAAAGACTATGACGATGACTGGGAATGTGTTGCTGTCTTAAATGGCCATGAAGGAACTGTT
TGGTCTTCTGATTTTGACAAGACTGAGGGCGTGTTTAGACTGTGCAGTGGAAGCGACGATTCCACTGTACGGGTA
TGGAAATACATGGGTGACGACGAGGATGATCAACAAGAATGGGTTTGTGAAGCTATTCTCCCGGATGTACACAAA
AGACAGGTATACAATGTTGCATGGGGCTTTAATGGCCTCATTGCTAGTGTAGGAGCGGATGGAGTACTAGCGGTC
TATGAAGAAGTAGACGGAGAATGGAAAGTTTTTGCTAAACGTGCATTATGTCATGGAGTTTATGAAATCAACGTT
GTGAAGTGGTTGGAGTTAAATGGTAAAACTATACTTGCAACAGGCGGTGACGACGGGATCGTAAATTTCTGGTCT
CTAGAAAAAGCAGCGTAG

>YOL132W 0.79   Cold
ATGATGGTTTTTTCGAGCACCTTTATTTTTCTAATTTTGGAGTTAGTTGTACTGTGTGAAGCTTCAGTCCACACC
ATTCAAATTAAGGACAAGCATTTCGTTGACACAGTGACTGGAAAACCATTCTTCATCAAAGGTGTGGATTATCAG
CCAGGAGGCTCTTCCGACGTTAGTGAAAAGCAGGATCCGTTATCCAATCCAGATGCATGCGCTCGTGATATTTTA
TTATTTCAAGAATTGGGAATAAACACAGTAAGAATTTATTCTATAAACCCGGACCTGAACCATGATGCGTGCATG
ACTATGCTCGCAATGGCAGGTATCTATCTTATATTGGATGTCAATTCCCCATTGCAAAACCAGCACTTAAACAGA
TATGAACCTTGGACTACCTATAATGAAGTTTATTTAGAACACGTTTTCAAGGTTGTCGAACAATTTTCTCATTAC
AATAATACTTTAGGATTTTTTGCTGGGAATGAAATTGTAAACGACAAAAGATCAGCACAATATTCTCCTGCCTAT
GTCAAGGAGTTGATTGGAACTATGAAGAATTACATAAGCGCACACTCGCCCAGAACAATTCCGGTAGGTTACTCT
GCTGCAGATGACCTGAACTATAGGGTATCACTGTCCGAATATTTGGAGTGTAAAGATGATGATAAGCCAGAGAAT
AGCGTTGATTTTTACGGAGTTAACTCCTACCAATGGTGTGGCCAACAAACTATGCAAACTTCAGGGTACGATACA
TTGGTGGATGCTTACAGGAGCTATTCCAAGCCAGTTTTTTTTTCAGAATTTGGATGCAATAAAGTATTGCCAAGG
CAATTCCAGGAAATAGGTTATTTGTTTTCTGAGGAGATGTATTCAGTATTCTGTGGAGGTCTGGTTTACGAATTT

TCACAGGAGGATAATAATTATGGGTTAGTTGAATATCAAGAAGATGATTCAGTACAGCTCTTAGCAGATTTTGAG
AAGCTTAAATCGCACTACCAAAACATTGAGTTTCCTTCTATGAAGACTTTAAAGGAAACTGTTCAGATGGAGGAG
ACGCCATCATGTGCGGAAGATTATGAAAATTTGAAGATTGAATCTAAAATTGCTAAAAATTTAGGAAGTTCTTTG
ATAAAAAAAGGCGTGAAAGTTGAAAAAGGCAAATACATAGATATTCACGAGGATCAGCTGTCCACCAATGTCACG
ATTTTAGACAAGCATGGTGACAGATGGAATGGGCCCAAAAAGATTGAAATTAGGCAAAGCCTGACATTGGCAGAC
CTGGAAGGAGAAGAACAAGAAGACGCAGATGAAGACAAAGATGATTTAAAACGAAAACACCGGAATTCTGCCTCT
ATTTCCGGCCCATTGTTACCTCTTGGGTTGTGTTTGCTTTTTTTTACTTTTAGTTTATTTTTTTAG

>YIR030C 0.73 Cold
ATGGAAACAAGAATACTTGTTGTGAATCCTAATAGTTCGAAGTCAATGACGGTGTCCTTGCGGGAAACCATTGAA
AAGACTTTTTCAATGGAATCATGTAAAATCAGTTATTTCACAGGGCCAGACACGTCGCCACCACAAATTGATGGG
CAGGAAACAAGTATTAAAAGTATGGAGGCCTGTCTTCCCCTCTTAATTGATGACCAGGAGTCGGTATATTACTTT
CAGAAATTCAACGGGATATTAATTGCCTGTTTTTCAGATCATCCTTTGGTAGCAAAGATAAAGGATAGAGCTGCT
AAAGAAAAAGCAGATGTTTCTATTGTTGGGTTATTAGATAGTAGTATTAATTATTGCAATTTGGTTGGAAAGAAG
TTCTCTATTATTACTTCAAATAAAGAATGGATTCCAATATTGAATAACTCTGTGGAATCGAAATTCTTGACAGGG
AATACAGTTAACAAAAATTTATGGAAAGGTACGGTATCTACTGATTTACAGGTCCTCGACCTACATAGTCCAGAA
AATTTCCAGCAGATAGCAGAAATTATATATCGCGAAAATATTAAAAAGCTTGATTCAGACATTGTAATATTAGGT
TGTGCAGGATTCTCGGGTTTGCAAAATAAACTGGCGAAAACATTCCAAAGGGATGGCACGCTGTTTCTGGACACG
ATTGAGATCGGTTTACAGATTCTAATCACTATGATAAGATTTGTCAACTCACAAAAATAA

>YMR220W 0.81 Cold
ATGTCAGAGTTGAGAGCCTTCAGTGCCCCAGGGAAAGCGTTACTAGCTGGTGGATATTTAGTTTTAGATACAAAA
TATGAAGCATTTGTAGTCGGATTATCGGCAAGAATGCATGCTGTAGCCCATCCTTACGGTTCATTGCAAGGGTCT
GATAAGTTTGAAGTGCGTGTGAAAAGTAAACAATTTAAAGATGGGGAGTGGCTGTACCATATAAGTCCTAAAAGT
GGCTTCATTCCTGTTTCGATAGGCGGATCTAAGAACCCTTTCATTGAAAAAGTTATCGCTAACGTATTTAGCTAC
TTTAAACCTAACATGGACGACTACTGCAATAGAAACTTGTTCGTTATTGATATTTTCTCTGATGATGCCTACCAT
TCTCAGGAGGATAGCGTTACCGAACATCGTGGCAACAGAAGATTGAGTTTTCATTCGCACAGAATTGAAGAAGTT
CCCAAAACAGGGCTGGGCTCCTCGGCAGGTTTAGTCACAGTTTTAACTACAGCTTTGGCCTCCTTTTTTGTATCG
GACCTGGAAAATAATGTAGACAAATATAGAGAAGTTATTCATAATTTAGCACAAGTTGCTCATTGTCAAGCTCAG
GGTAAAATTGGAAGCGGGTTTGATGTAGCGGCGGCAGCATATGGATCTATCAGATATAGAAGATTCCCACCCGCA
TTAATCTCTAATTTGCCAGATATTGGAAGTGCTACTTACGGCAGTAAACTGGCGCATTTGGTTGATGAAGAAGAC
TGGAATATTACGATTAAAAGTAACCATTTACCTTCGGGATTAACTTTATGGATGGGCGATATTAAGAATGGTTCA
GAAACAGTAAAACTGGTCCAGAAGGTAAAAAATTGGTATGATTCGCATATGCCAGAAAGCTTGAAAATATATACA
GAACTCGATCATGCAAATTCTAGATTTATGGATGGACTATCTAAACTAGATCGCTTACACGAGACTCATGACGAT
TACAGCGATCAGATATTTGAGTCTCTTGAGAGGAATGACTGTACCTGTCAAAAGTATCCTGAAATCACAGAAGTT
AGAGATGCAGTTGCCACAATTAGACGTTCCTTTAGAAAAATAACTAAAGAATCTGGTGCCGATATCGAACCTCCC
GTACAAACTAGCTTATTGGATGATTGCCAGACCTTAAAAGGAGTTCTTACTTGCTTAATACCTGGTGCTGGTGGT
TATGACGCCATTGCAGTGATTACTAAGCAAGATGTTGATCTTAGGGCTCAAACCGCTAATGACAAAAGATTTTCT
AAGGTTCAATGGCTGGATGTAACTCAGGCTGACTGGGGTGTTAGGAAAGAAAAAGATCCGGAAACTTATCTTGAT
AAATAA

>YOR046C 0.75 Cold
ATGAGTGATACAAAGAGAGATCCGGCCGATTTACTAGCTTCTTTAAAGATTGATAATGAGAAAGAGGATACAAGT
GAAGTTTCAACAAAAGAGACTGTTAAGTCCCAACCAGAAAAGACTGCTGATTCGATCAAGCCTGCAGAAAAATTA
GTTCCAAAAGTTGAGGAAAAAAAAACAAAACAGGAGGATAGTAATTTAATCAGCTCAGAATATGAAGTTAAGGTC
AAACTTGCTGATATCCAGGCTGACCCAAATTCTCCATTGTACAGTGCTAAATCTTTTGATGAATTAGGATTAGCA
CCAGAACTACTAAAGGGGATATATGCTATGAAATTTCAAAAACCATCCAAAATTCAAGAAAGGGCGCTGCCATTA
TTATTACATAATCCGCCTAGGAACATGATTGCTCAGTCTCAATCAGGTACAGGTAAAACTGCCGCCTTTTCTTTA
ACGATGCTAACAAGAGTAAACCCAGAAGACGCATCTCCACAGGCAATTTGCTTAGCCCCCTCCAGAGAACTGGCC
AGACAAACACTAGAAGTCGTTCAGGAAATGGGTAAGTTCACAAAAATAACCAGTCAGTTAATTGTTCCCGATTCT
TTTGAGAAAAACAAGCAAATTAATGCTCAAGTGATTGTTGGTACGCCGGGCACTGTTCTTGACCTAATGCGTAGA
AAATTGATGCAGCTACAGAAAATTAAAATTTTTGTTTTGGATGAAGCCGATAACATGTTGGATCAGCAGGGTCTA
GGTGACCAGTGTATTCGTGTTAAGAGATTTTTACCCAAGGATACTCAACTTGTTTTGTTTAGTGCTACTTTTGCC
GATGCAGTTAGGCAGTACGCAAAGAAGATCGTTCCAAATGCTAATACTTTAGAATTACAAACAAATGAAGTTAAT
GTTGATGCCATCAAACAACTATACATGGACTGCAAAAACGAAGCAGATAAGTTTGATGTTTTAACTGAGCTATAT
GGTTTAATGACAATTGGATCTTCCATTATTTTTGTTGCAACCAAAAAAACCGCAAACGTTTTATATGGGAAATTG
AAAAGTGAAGGTCATGAAGTTTCTATCTTGCATGGTGATTTACAGACACAAGAAAGAGACAGATTAATAGACGAC
TTCAGAGAGGGTAGATCCAAAGTTTTGATTACTACTAATGTCCTGGCCCGTGGTATTGATATTCCTACTGTCTCA
ATGGTTGTCAACTATGATCTTCCAACGCTTGCTAACGGACAAGCAGATCCAGCTACTTACATTCACAGAATCGGT
AGAACCGGTAGATTCGGCAGAAAAGGTGTTGCCATTTCCTTTGTACATGACAAAAATTCTTTCAATATTTTATCT
GCAATTCAGAAATACTTCGGTGATATAGAAATGACTCGTGTTCCAACGGATGATTGGGATGAAGTCGAAAAAATA
GTTAAGAAAGTGTTAAAGGATTAG

>YBR258C 0.82 Cold
ATGGCGTATAATCAAGAAGATAGTAAAAGACTATCAGACAAGTATAAGAAGGAGGGACATTTTGACAAGTTGAAA
AGAGAAATATTGTCTAACCCATGGAATAATACAGAAGAGAATAGTGAATCTTTTGAACAAGCGCTTCGGAAAAGA
GTTGCCAGTACTGTTAAAGAAATGGTTAACGAAGATGAAGAATTAATATTTAAAAACAGAGGGCTAACCAGTGCA

TTGATTGAATCACAATTGGTCAAGGACAACTACCTAAAGCTGGGTAGTAAAATGGAGGGGGATAATGGTGATGGT
GAGAAGAAATTTGACTTGGATGTCTATGTACGGTCTAAGTTACAGGATCCCAAACTATTGGAAATGATAAAGGGA
CAACTTCAGGAAACACTGAACTCTTATGAAGAGGAAGCAAATGGAAGTACGTAA

>YPR155C 0.8 Cold
ATGATCATCAATAGGCGTATTTTGAAATCTTTTGAAGAGATAAGTCATTCATTAGAGGAATCACTACGAGAGGTT
GCATTTGATTCACAGCAACAATTGATTCAAGACGTACGTGAGGAAAATGAAGAACTAAGTAGGTTACAAGATCAA
TTACAATTGATCAGGTCAATTGTTGAAAAAATCTGCATTTCAATCAAAACTGACAATATTGACTCTTATTGTTCG
GTGCCTTTTGATTTGCTATACAATATTTGCAAGGACATTGCTGATCCCTCATCCTTTGAAGACGGAGATTTGCAA
TATCTTGTTAGCCAAGCCATATTTGAGTACATTATATTATTGTGCTACTATTCTGTGACCAACGAATGCGTCCAA
GGTTTACCTGCCGTGTATGAAGCTGAACAGTACTACAAAACAGTAAGTGATTCAATCCTAAAGTCTTTTCTTTAC
TGTTTACAAAATTCAGTATCGACAATACGTCTTTTATCTCAAACTGTTTTGAAGGATGTAAATAAGAAAAAACTG
TCGCATCAAAAATGGTCCCTAAAGGCCTTGTCAGTTGATTTACTAGAAAAAATACGCCCAAGAATAAACAAATTT
ATGGTGATTCGGAACTTCAGGTTTGTCGGGCTACCGAAGAAACCAATTGAAATAGCTTCATTGGTTTCTGATATA
CCTCGCGGTATAGTGCACGAAAGACTTGATATGGTCACTCAATCATCAAAGTATTATACTATTAAACTGGGGCAG
TTGATCACTGAATTCGATCAACAACCAGAAGAAAATGGTATGTTCACCGAAGTACATTTACCGAACTACGAAAGA
CGTCTAAAATCTTTACAGGACTTCTTTGGATTAGCCATGTCTGATTCGAATTTACTAGATGTGATCCGATGCTCA
GCGAAATTTCATAAAGATCATCCTTTAAGAAGGTTCACTAAACCAAGTATATTGACTAGATACTGGCCTTCAATT
TTGTTGTGTCTTTTATATGGACCATCTTCTGTTATGTCGTTGTGGAATTCCCGGTATTTTATTCAAGATTTCATT
AAGACCAATGTCGTTGACTTTGCGAAAGGGCTGATCTTGAACTGGTTATGGGCACCCCTGAAACAGGTTTGGTCT
ACTGTTAAGCATGACGAGGGAAGCGCCATTTCAGTGACATCACAGGAGACATTAAACTCTGACATGGATTCTCTT
ACCAGAATGATTGTTAGTTTTGTTGTGGATAATAGTGATTCGACGTCTAACAGCCCCATAGACCCTATTCTATTG
AGTACTAAAGTCGAACATGGTGATTTGACCGAATTTATGGAAATATATGAAACACAACTACATCATCCGATAAAG
AATATAGCTACGGGTGGATTAGTCAGGTCTTTATTGATTCAACTGCAAAAAAACAAAAGTCGATGGATCTATGGCA
TTGAATGGCATTGATAAAATGCTGAAATCGCAACAGTTGGTGTTTGGAGTTGTAGCATTATCTCCGGCTTTAGTG
ATACTATATTCTTCTATAGTGGCGTTAAAAAGATTTGTCAAACTAGGCAATGTCTGGTCTAATGAAAAACGGTAT
AGGGAGCAAATAAGTATAAGTTTGAATAATGTGGAAAGAGTTTTGAATTATTCTAAACAAGGTGCGGACGCCGAT
GAGGAACACTTAAACCAGGGTCTACTAGTGATTGAGGTTTCTAACTTATACAAATTGGGAAGTTTTTTGATCCCG
CGTTCAAGAAAGAAAGAATGGTTTAGGGACGTTGAGGAACTGGTGGATACCAATCTTGACTCCGGAGCTCATATA
AATGTCGTCAACAGGATATACCACGTATATGGCAGGTTTTTAATCCATTAG

>YKL145W 0.69 Cold
ATGCCACCAAAAGAAGACTGGGAAAAATACAAGGCACCTTTGGAAGACGATGATAAAAAACCTGATGATGACAAG
ATCGTACCCTTAACAGAAGGTGACATTCAAGTCTTAAAATCATACGGTGCAGCTCCATACGCAGCCAAATTAAAG
CAAACTGAGAATGACTTAAAAGACATTGAAGCGAGAATCAAAGAAAAGGCCGGTGTGAAAGAAAGCGATACAGGG
TTAGCACCCTCCCATCTATGGGATATTATGGGTGATAGGCAGAGACTGGGCGAAGAACATCCCTTACAAGTCGCT
CGTTGCACGAAGATCATTAAGGGGAATGGTGAAAGTGACGAAACTACGACAGATAATAATAACAGCGGCAATAGC
AATAGTAATAGCAATCAACAGTCTACTGATGCAGACGAAGACGATGAAGATGCCAAGTACGTGATAAATCTTAAG
CAAATTGCCAAATTTGTCGTCGGATTAGGTGAACGTGTTTCTCCAACAGATATAGAAGAAGGTATGCGTGTAGGT
GTCGATAGGTCGAAATATAATATTGAACTTCCATTGCCTCCAAGGATTGACCCATCAGTTACTATGATGACCGTT
GAAGAAAAGCCTGACGTTACTTATAGCGATGTCGGTGGCTGTAAAGACCAAATTGAAAAGCTGAGAGAAGTTGTC
GAGCTGCCCTTATTATCTCCAGAAAGATTTGCTACTCTTGGTATTGATCCACCAAAGGGTATCTTATTATATGGG
CCACCTGGTACTGGTAAGACATTATGTGCTCGTGCTGTTGCTAATAGAACTGATGCAACTTTTATTAGGGTCATT
GGGTCTGAGCTAGTGCAAAAATATGTCGGTGAAGGTGCTCGTATGGTTAGAGAATTATTTGAAATGGCTCGTACC
AAAAAGGCTTGCATTATATTCTTCGATGAAATCGACGCTGTTGGTGGTGCTCGTTTTGATGATGGTGCTGGTGGT
GATAATGAAGTTCAAAGAACTATGTTAGAACTGATTACACAGTTAGACGGGTTTGACCCTCGTGGTAACATTAAA
GTGATGTTTGCCACCAATAGGCCCAACACTTTAGATCCAGCTTTATTGAGACCCGGTAGAATTGATCGTAAGGTA
GAGTTTTCATTACCTGACTTGGAAGGTCGTGCAAATATCTTCCGTATTCACTCTAAATCAATGAGTGTGGAACGT
GGCATCAGATGGGAACTTATCTCCAGACTGTGTCCAAACTCTACCGGTGCAGAATTAAGATCTGTTTGCACAGAA
GCCGGTATGTTTGCCATTAGAGCAAGAAGAAAGGTGGCTACTGAAAAGGATTTCCTGAAGGCTGTTGATAAAGTT
ATTAGCGGATACAAGAAGTTTAGTTCCACATCGCGTTATATGCAATATAATTGA

>YJL075C 0.81 Cold
ATGCTTGATTATTTTTTTTTACTAGCTTTCTGTGACGTGTATTCTACTGAGACTTTCTGGTATCATTTTTTTCTTG
AAATCTTTTATTAATGATGCAAATCCACCACTTGGCTTCTTCTTCTTACCTAAAGCAGCACTAGCGGATTTCGCA
CTAATAAAACTTTTTCCATCATCAGATGAATCCCCTGAGTCATCAGAATCTGACTCAGATTTAGAATCTGAACTG
GAATCCGATACGGAGTCCGAACTGGAGTTAGAGTCAGAATCAGAGCTAGATTCATCATCACTACTTGAAGGGGCT
TTTGTTTGTGACTTCTCATTCGATTTAGAAGTTTTTTCCTTTACATCTGGGATGCCTCTTGAAACAAGATCAGAT
AATGAACTCAAAGAAGGACGAACTTTTCTTGGAAGGTCATGA

>YJL004C 0.79 Cold
ATGGTTTCGATAAGAAGGTATTTGCGAGTGCCGAATGAGTTGAAACCTTCCCAGATCTTCAAACAAGATTCTCTC
TCTCCAAGTAAGATTGGACTTCAAATTGTTCTTTTACAGATTTTTTATTACACCACAGCGATCGTTCTTTTTTAT
TGTTGGGCGAAGCTAGCGGGATATGATCTCAATATCAAGGAATGGCTGTTTTCTTGGGAGAATATCGACTTTACG
AACGCATACGGTTTATCAATCTCACTGTTATGGCTTCTGGATTCATTAATATGTGTTTTTTTCTTAACTGTTATC
GTTGGACGGAGTAAACTCGCATGGGATTTTGCTATCACTATACATGCCATCAATTTTATTGTGGTGTTCCTCTAT
ACTCGGAAATTTCCTTCTTTTTCTTGGTTTTTTCTTCAAATTTTATCATCTTTAATACTAATATTTTGGGGACA

TGGACAACAAGATGGAGAGAGCTTAGAGACACCTTTTTTGAGGGCTTGGTTGATCCTAATGAAGGAGAAGTTGGA
TTGGTTACGCCTAGTCAGCAGCATAGTAATCATTCAGAATTGGAGCAATCACCAATACAACTAAAAGACTTAGAA
AGCCAAATATGA

>YDL029W 0.66 Cold
ATGGACCCACATAATCCAATTGTCCTTGATCAGGGTACTGGTTTCGTCAAAATTGGTCGTGCTGGCGAGAATTTC
CCAGATTACACGTTTCCTTCTATTGTTGGTAGACCCATCTTGAGGGCGGAAGAACGTGCCAGCGTTGCTACACCA
TTAAAGGACATTATGATTGGTGATGAGGCAAGTGAAGTTCGCTCTTATCTGCAAATATCTTATCCTATGGAAAAC
GGTATTATTAAGAATTGGACAGATATGGAACTTCTTTGGGATTACGCCTTTTTCGAGCAAATGAAACTACCATCC
ACCTCCAACGGTAAGATTTTACTAACGGAACCTCCAATGAATCCGCTGAAAAATAGGGAAAAAATGTGTGAGGTA
ATGTTCGAAAAATACGATTTTGGCGGAGTTTATGTTGCCATCCAAGCTGTTCTAGCATTGTACGCACAAGGTTTG
TCTTCAGGAGTCGTCGTCGATTCCGGTGACGGTGTTACTCATATAGTCCCAGTTTACGAATCTGTCGTTTTGAGC
CACTTAACAAGAAGATTAGATGTTGCGGGTAGAGACGTTACTAGGCATTTGATTGATCTGCTTTCTCGTCGTGGT
TATGCATTTAACAGAACTGCAGATTTCGAAACTGTGCGTCAGATAAAGGAAAAATTATGTTATGTTTCATATGAT
TTAGACCTAGATACAAAATTGGCTAGAGAAACAACCGCCCTTGTGGAATCGTATGAGTTACCAGATGGCAGGACA
ATCAAAGTGGGACAAGAGAGATTTGAAGCACCAGAATGTTTGTTCCAACCTGGTTTGGTTGACGTTGAACAACCT
GGCGTGGGCGAGCTGTTATTTAATACTGTGCAATCGGCTGATGTTGATATCAGAAGTTCCCTGTATAAGGCCATT
GTTCTTTCAGGTGGTTCAAGTATGTACCCAGGGCTGCCTTCGAGATTAGAGAAAGAATTGAAACAATTATGGTTT
AGTAGAGTTTTACACAATGACCCTTCAAGACTTGATAAATTCAAAGTTAGAATTGAAGATCCTCCAAGGAGAAAG
CATATGGTTTTCATTGGTGGTGCCGTTTTAGCTAGTATCATGGCTGATAAAGACCACATGTGGTTGTCTAAGCAA
GAATGGCAAGAAAGCGGGCCATCTGCAATGACTAAATTTGGTCCAAGATAG

>YKL073W 0.78 Cold
ATGCGAAACGTTTTAAGGCTTTTATTTTTAACAGCTTTTGTTGCTATAGGGTCTTTAGCAGCCGTTTTAGGTGTT
GATTACGGTCAGCAAAATATCAAGGCCATTGTGGTTTCTCCGCAAGCCCCATTAGAACTTGTGCTCACACCAGAG
GCAAAACGGAAGGAGATATCTGGTCTTTCGATAAAAAGATTACCAGGTTATGGAAAGGATGATCCGAATGGGATT
GAAAGAATCTACGGTTCCGCTGTTGGCAGTTTAGCAACAAGGTTTCCCCAAAACACATTGTTGCATTTGAAACCG
CTACTTGGGAAATCACTAGAAGATGAAACCACTGTAACTTTGTATTCAAAACAACACCCCGGTTTAGAAATGGTA
TCAACAAATAGAAGTACCATAGCCTTTTTAGTTGATAATGTGGAATATCCATTGGAAGAGTTAGTGGCAATGAAT
GTCCAAGAGATTGCCAATAGAGCCAATTCACTGTTGAAGGATAGAGATGCAAGAACTGAGGACTTTGTAAACAAG
ATGAGTTTTACAATTCCTGACTTTTTTGACCAACATCAAAGGAAAGCACTTTTAGATGCCAGTTCAATAACCACA
GGAATCGAAGAGACATATCTGGTTAGTGAAGGGATGTCTGTTGCAGTTAACTTTGTATTAAAGCAGCGCCAATTT
CCACCAGGTGAACAGCAGCATTATATCGTATATGACATGGGGAGCGGTTCTATTAAGGCCTCAATGTTCTCTATA
TTGCAGCCGGAGGACACTACTCAGCCCGTTACAATAGAATTTGAAGGATATGGGTATAATCCACATCTAGGTGGT
GCAAAGTTTACAATGGATATTGGCAGTTTGATAGAGAATAAGTTTTTGGAAACACACCCAGCCATAAGAACTGAT
GAATTGCACGCTAATCCCAAGGCCTTAGCAAAAATCAACCAAGCAGCAGAGAAGGCAAAGTTAATTTTAAGCGCC
AATTCTGAGGCAAGTATTAACATAGAATCACTGATCAACGATATTGATTTCCGTACTTCTATAACTAGACAGGAA
TTCGAAGAATTTATTGCAGACTCGTTATTGGACATTGTCAAACCCATAAATGACGCTGTTACAAAACAATTCGGT
GGCTATGGAACAAATTTACCTGAGATAAATGGGGTCATTTTGGCGGGAGGCTCTTCCCGAATTCCCATTGTGCAG
GATCAATTAATCAAACTCGTATCCGAAGAAAAAGTGTTGAGAAATGTCAATGCTGATGAATCAGCTGTGAATGGT
GTTGTTATGAGAGGGATCAAGTTATCTAATTCGTTTAAGACCAAGCCGTTAAATGTTGTTGACCGTTCTGTAAAT
ACTTATTCATTCAAATTATCAAACGAATCTGAACTGTATGATGTGTTCACGCGCGGAAGTGCTTATCCAAACAAA
ACATCTATTTTGACAAACACGACTGATTCGATTCCTAATAATTTTACCATTGACTTATTTGAGAATGGTAAATTG
TTCGAAACTATCACAGTTAATTCAGGAGCTATAAAGAATTCATATTCCTCTGATAAGTGCTCGTCAGGAGTTGCG
TATAACATTACTTTCGACTTGTCCAGTGATAGATTATTCTCTATTCAAGAGGTTAACTGCATTTGTCAGAGCGAA
AATGACATAGGTAACTCCAAGCAAATTAAGAACAAAGGCAGCCGTTTGGCTTTTACTTCTGAGGATGTTGAGATC
AAAAGGCTTTCTCCTTCAGAACGTTCGCGTTTGCATGAGCATATCAAGTTGCTCGATAAACAGGATAAGGAAAGA
TTTCAATTCCAAGAAAATTTAAACGTTCTTGAAAGTAACTTGTATGATGCTAGAAACCTGCTAATGGATGATGAA
GTTATGCAAAATGGACCAAAATCCCAAGTAGAAGAGTTATCGGAGATGGTTAAAGTATATTTGGATTGGCTCGAA
GATGCATCCTTTGATACTGACCCTGAGGATATAGTTAGCAGAATTAGAGAAATTGGAATATTAAAAAAGAAAATA
GAACTTTACATGGATTCTGCAAAGGAACCTTTGAACTCTCAACAATTTAAAGGAATGCTTGAAGAAGGCCATAAG
TTACTTCAGGCTATAGAAACCCATAAGAATACCGTTGAAGAATTTTTGAGTCAATTTGAAACCGAGTTTGCGGAT
ACCATAGATAATGTTAGAGAAGAATTTAAAAAGATTAAGCAACCAGCGTATGTGTCGAAGGCGTTATCTACATGG
GAGGAAACCTTAACCTCTTTTAAAAATTCCATTAGCGAAATAGAGAAGTTCCTGGCAAAAAACCTATTTGGCGAA
GACCTTCGTGAACATTTATTTGAAATCAAATTACAATTTGATATGTATCGTACGAAACTAGAGGAAAAACTGCGT
TTAATAAAAAGCGGTGATGAAAGTCGCTTAAATGAAATAAAGAAGTTACATTTAAGAAACTTCCGCCTACAAAAG
AGAAAGGAGGAAAAGTTGAAAAGAAAGCTTGAACAGGAAAAAAGCAGAAACAACAATGAAACAGAATCGACAGTA
ATCAACTCGGCTGACGATAAAACTACTATTGTCAATGACAAGACCACCGAGTCGAATCCAAGTTCTGAGGAAGAC
ATTTTGCATGATGAATTATAG

>YJL156C 0.81 Cold
ATGGTCAGATTTTTTGGTTTAAACAAGAAAAAGAACGAAGAAAAGGAAAATACAGACTTGCCTGCAGACAATGAA
CAAAACGCAGCAGAAACGTCGTCTAGCAACGTATCTGGAAATGAAGAAAGAATAGACCCAAACAGTCATGATACG
AACCCTGAAAATGCAAACAATGATGATGCGTCTACGACTTTTGGTTCGTCCATACAATCGTCATCCATATTTTCT
AGAGGAAGAATGACTTACGGAACGGGAGCCTCTTCTAGTATGGCCACATCAGAAATGCGTAGTCACAGTAGCGGA
CATAGTGGATCTAAGAATAGTAAAAATTTACAGGGTTTCAAAGATGTTGGGAAACCATTAAGAGCGGTGAGCTTC
TTGAGTCCTGTTAAGGAGGAGGAATCTCAGGATACGCAGAATACTTTAGACGTCAGCTCTTCAACGTCCTCAACA

TTAGCCACTTCAGAAAATGCGAGAGAAAACAGTTTCACTTCCAGAAGAAGTATCACTTTGGAGTACATTCACAAA
AGTTTGTCTGAGCTAGAGGAAAATTTGGTTGATATAATGGATGATATTCATCAGGATGTCATCAGTATTTCAAAA
GCGGTTATTGAAGCCATCGAATATTTTAAAGAATTCTTACCGACAACACGAGACAGGATACCGTACAGGATAAGT
TTAGAAAAATCGTCTTCATTACGAAAAATTAATAAAATTGTATTACATTTTCTGGATAATTTATTGGTTTCCGAC
GCATTTTCGAATTCTAGGTCGATACTGTTACGAAGATTTTACTTCTTCCTGAAGAAACTAAACCTTATTACGGAT
GACGATTTGATATCAGAATCGGCGTTTTACCATGCTTATCAGTCTTTTGTATTGGCAGCCATTGCAACTTACCA
AGTATGGATAAATTAGGCATGATTCTAGATGAGTTGACCAAAATGGATTCTTCAATAATCTCTGACCAGGAAGGT
GCTTTTATAGCACCTATACTTAGAGGTATAACCCCAAAGAGTTCAATTCTTACAATAATGTTTGGCTTACCCAAC
TTGCAACATGAACACTATGAAATGATAAAGGTTCTTTATTCGTTGTTCCCAGATGTACACATGTATTGTGTGAAA
GATTACATTAAAAAAGCTGCATCAGCAGTAGGCAGTATTCCGTCTCATACCGCAGCAACTATCGATACAATAGCC
CCAACTAAATTTCAGTTTTCCCCCCCATATGCAGTTTCAGAGAATCCACTTGAATTGCCAATTTCTATGTCTTTA
TCCACTGAGACAAGCGCTAAAATTACAGGTACTTTAGGGGGTTATCTATTCCCTCAGACTGGTAGTGATAAAAAA
TTTTCTCAATTTGCAAGCTGCTCATTTGCCATTACTTGCGCCCACGTTGTATTATCTGAGAAGCAAGATTATCCA
AACGTCATGGTACCTTCTAATGTTTTACAGACTTCTTACAAGAAAGTTTTAACGAAAGAATCAGATAGGTATCCT
GATGGTTCGGTGGAGAAAACAGCATTTTTGGAGGAAGTGCAAAGAATAGATCAAAACTTGAACTGGCAGAAATCA
AACAAATTTGGTCAAGTAGTTTGGGGCGAGAGAGCAATCGTAGATCACAGATTATCAGATTTTGCTATTATCAAA
GTTAATTCATCATTCAAATGTCAGAATACCCTAGGTAATGGCTTAAAATCATTCCCAGATCCAACATTAAGATTT
CAAAATTTACATGTGAAACGAAAAATTTTTAAAATGAAGCCTGGGATGAAAGTATTTAAAATAGGGGCTTCTACG
GGATACACTTCAGGCGAGTTAAATTCTACAAAATTAGTTTATTGGGCCGACGGAAAGTTACAAAGTAGCGAGTTT
GTCGTTGCATCTCCAACTCCTTTATTTGCTAGTGCGGGGGATTCAGGCGCATGGATCTTGACCAAGTTAGAAGAT
CGTCTAGGTTTGGGGCTTGTTGGTATGTTGCATTCATACGATGGTGAACAGAGGCAGTTTGGTTTGTTCACACCT
ATAGGTGATATCCTGGAGAGACTACATGACTGTAACTAA

>YDL093W 0.79 Cold
ATGAATAAAGAGCATTTGCTGAAGGTGGATCCCATCCCCGATGTGACTATTAAACGCGGCCCTTTGAGGTCTTTT
CTCATAACAAAACCCTGTGATAATTTGAGTTCATTACGAACAGTTACTTCATCTAAGGAAAAGCTTCTAGTTGGC
TGTTTGCTGATATTTACTGCCATCGTAAGGCTACACAATATCTCCCTGCCAAATAGTGTTGTTTTTGGTGAAAAT
GAAGTTGGTACATTTGTTTCTCAATACGTGAACAACATATTCTTCACCGATGTTCATCCTCCTTTAGTGGCAATG
CTATATGCAACCGTGTCCTCTGTTTTTGGTTATAAAGGGCTTTTCAATTATGGGAACATTGGTACTGAATACACG
GCAAACGTTCCATACGTCGCGATGAGGTTCTTTTCTGCTACTTTGGGCATCGTGTCCGTTTTGGTATTATACTTA
ACGTTACGAGTTTCTGGCGTGAAAATTGCGGTAGCTGCCATTTGTGCAGTATGTTTTGCAATTGAAAACTCCTTT
GTAACGTTGTCTCGTTTCACCTTGATAGAGGGACCCTTTGTTTTTTTCATGGCATGTGCAGTTTATTTCTTTAGA
AGATCGGAACTTTATTTGCCAAATTCATGTAAAGCAAACAAGTCATTACTTGCTGCGAGCATTGCATTGGGATTT
GCAGTTTCTTCAAAATGGGCTGGCCTCTTCACCATCGCATGGGCTGGTATTATTGTCCTTTGGAGAGTATGGTTC
ATGATTGGTGATTTGTCAAGACCCATTGGTTCTTCTATCAAATATATGGCGTTCCAGTTTACCTGTCTATTAGCT
ATTCCTGCCTTCATCTATTTTCTCATTTTCAGCGTGCATATTAAGACATTAAACGTGAATGGTATTAGTAGTAGC
TTTTTTCCCGCTGAGTTTAGAAAAACTTTAAAATACAACAACGTTATTAAAGAGACTGTGGCAGAAGTTGCAGTG
GGTTCCGCTGTTTCACTGAATCATGTTGGAACGGCAGGTGGTTACTTGCATTCTCATCTTCACAATTATCCGGCT
GGTTCCATGCAACAACAAGTTACTTTGTATCCTCACATCGACCAAAATAACAAATGGATTATTGAGCTGGCTGAA
CATCCAAATGAAAATGTCACAAGTTTCCAAAATTTAACCGACGGTACCATAATTAAATTAAGACAGCTTAAGAAC
GGCTGCAGGTTACACTCACATGACCACAAACCTCCAGTTTCTCAAAATGCGGATTGGCAAAAAGAGGTGTCATGT
TATGGATACGAGGGCTTCGAAGGTGATATAAATGATGATTGGATAATTGAAATCGATAAAAAGAGATCAGAGCCG
GGACCTGCCCAGGAACATATTAGGGCTATTGAAACCAAATTTAGGCTGAAGCATTATCTAACTGGTTGTTACTTA
TTTTCACATCCTGAAAAGCTACCTGAGTGGGGATTCGGGCAGCAGGAAGTTACGTGTGCATACTTTGCAAGGGAA
GACTTAACTTCATGGTATATTGAAGAAAACGAAAACGAAATTTCTTTGCCAAATCCAGAGAAGGTTTCTTATAAG
AAAATGAGCTTTTGGCAGAAGTTTGTTGCTATCCACAAGTTCATGTTCTACCTTAACAATTATATGGATACTAGT
CATGCCTACTCATCTGAACCAAAGACTTGGCCTCTTATGTTGCGTGGTATTGATTTTTGGAATGAAAATGGCAGA
GAGGTGTACTTTTTAGGTAACGCTGTTTTGTGGTGGTCTGTCACAGCATTTATTTGCACGTTCATCATTGGAGTG
GCTGTTGAGCTTCTTGCTTGGAAATTAGGTGTGAATATTCTACGGGATAAGCACATCATAAATTTCCACTATCAG
GTCTTTCAGTACTTATTAGGCTTTGCCGCCCACTATTTCCCTTACTTCTTTGTGGGGCAAAAGTTATTTTTGTAC
GATTATTTGCCGGCCTATTATTTTGGTATCCTAGCATTTGGTCATGCTTTAGACCTGATTTCAACCTATATTTCT
AACAAAAGAAACAATACTGGATATATAGTGGTGGCTATTTTCATGGTTGTATGTTTCTATTTCTTCAGCGAGCAT
TCTCCACTTATTTATGCTACTGGATGGTCGAGTAACCTGTGTAAGAGGTCCAAGTGGTTAGGAAGCTGGGACTTT
TATTGCAATTCACTATTACTATCCGATAGCCACTATGAATTAAACGCTGAATCATGA

>YOL151W 0.82 Cold
ATGTCAGTTTTCGTTTCAGGTGCTAACGGGTTCATTGCCCAACACATTGTCGATCTCCTGTTGAAGGAAGACTAT
AAGGTCATCGGTTCTGCCAGAAGTCAAGAAAAGGCCGAGAATTTAACGGAGGCCTTTGGTAACAACCCAAAATTC
TCCATGGAAGTTGTCCCAGACATATCTAAGCTGGACGCATTTGACCATGTTTTCCAAAAGCACGGCAAGGATATC
AAGATAGTTCTACATACGGCCTCTCCATTCTGCTTTGATATCACTGACAGTGAACGCGATTTATTAATTCCTGCT
GTGAACGGTGTTAAGGGAATTCTCCACTCAATTAAAAAATACGCCGCTGATTCTGTAGAACGTGTAGTTCTCACC
TCTTCTTATGCAGCTGTGTTCGATATGGCAAAAGAAAACGATAAGTCTTTAACATTTAACGAAGAATCCTGGAAC
CCAGCTACCTGGGAGAGTTGCCAAAGTGACCCAGTTAACGCCTACTGTGGTTCTAAGAAGTTTGCTGAAAAAGCA
GCTTGGGAATTTCTAGAGGAGAATAGAGACTCTGTAAAATTCGAATTAACTGCCGTTAACCCAGTTTACGTTTTT
GGTCCGCAAATGTTTGACAAAGATGTGAAAAAACACTTGAACACATCTTGCGAACTCGTCAACAGCTTGATGCAT
TTATCACCAGAGGACAAGATACCGGAACTATTTGGTGGATACATTGATGTTCGTGATGTTGCAAAGGCTCATTTA
GTTGCCTTCCAAAAGAGGGAAACAATTGGTCAAAGACTAATCGTATCGGAGGCCAGATTTACTATGCAGGATGTT

CTCGATATCCTTAACGAAGACTTCCCTGTTCTAAAAGGCAATATTCCAGTGGGGAAACCAGGTTCTGGTGCTACC
CATAACACCCTTGGTGCTACTCTTGATAATAAAAAGAGTAAGAAATTGTTAGGTTTCAAGTTCAGGAACTTGAAA
GAGACCATTGACGACACTGCCTCCCAAATTTTAAAATTTGAGGGCAGAATATAA

>YNL235C 0.71 Cold
ATGCGTACCCTTGGTATTCTAGAAGAAAGAAATTCCAGACACTGTCACTCTCATTTCTTTTTATTAAGTCGAGAA
GTGAAATGTTTAAAACAATTCTATACAAAACTATGCTATAGTACTAATAATCAGCCGTCCATCTCAAAAAGCATA
CCTGAACATATGCACAGTCTTGTATACATGGTTGGCCATCTTTTGGTTTGGATGCTAGTGGGTACAATTGTCTTA
TCACTCGATATTATATTTCCTGCTCTTGTGACGGAACCGCACCTACTGCATCTCTTAAGTTTCCCATCGGATATT
TCTGATACACTCAGTTTGTCACAGGTTACATCGTCGTACAGTAATTTGCTAAAAGATTTGGAGGTTCTATTTTTT
ATGGGTAGTTTATCTGTATCAATAATTAATCCCTCTTCTAATGGTTGCAATAAATGA

>YAL001C 0.78 Cold
ATGGTACTGACGATTTATCCTGACGAACTCGTACAAATAGTGTCTGATAAAATTGCTTCAAATAAGGGAAAAATC
ACTTTGAATCAGCTGTGGGATATATCTGGTAAATATTTTGATTTGTCTGATAAAAAAGTTAAACAGTTCGTGCTT
TCATGCGTGATATTGAAAAAGGACATTGAGGTGTATTGTGATGGTGCTATAACAACTAAAAATGTGACTGATATT
ATAGGCGACGCTAATCATTCATACTCGGTTGGGATTACTGAGGACAGCCTATGGACATTATTAACGGGATACACA
AAAAAGGAGTCAACTATTGGAAATTCTGCATTTGAACTACTTCTCGAAGTTGCCAAATCAGGAGAAAAAGGGATC
AATACTATGGATTTGGCGCAGGTAACTGGGCAAGATCCTAGAAGTGTGACTGGACGTATCAAGAAAATAAACCAC
CTGTTAACAAGTTCACAACTGATTTATAAGGGACACGTCGTGAAGCAATTGAAGCTAAAAAAATTCAGCCATGAC
GGGGTGGATAGTAATCCCTATATTAATATTAGGGATCATTTAGCAACAATAGTTGAGGTGGTAAAACGATCAAAA
AATGGTATTCGCCAGATAATTGATTTAAAGCGTGAATTGAAATTTGACAAAGAGAAAAGACTTTCTAAAGCTTTT
ATTGCAGCTATTGCATGGTTAGATGAAAAGGAGTACTTAAAGAAAGTGCTTGTAGTATCACCCAAGAATCCTGCC
ATTAAAATCAGATGTGTAAAATACGTGAAAGATATTCCAGACTCTAAAGGCTCGCCTTCATTTGAGTATGATAGC
AATAGCGCGGATGAAGATTCTGTATCAGATAGCAAGGCAGCTTTCGAAGATGAAGACTTAGTCGAAGGTTTAGAT
AATTTCAATGCGACTGATTTATTACAAAATCAAGGCCTTGTTATGGAAGAGAAAGAGGATGCTGTAAAGAATGAA
GTTCTTCTTAATCGATTTTATCCACTTCAAAATCAGACTTATGACATTGCAGATAAGTCTGGCCTTAAAGGAATT
TCAACTATGGATGTTGTAAATCGAATTACCGGAAAAGAATTTCAGCGAGCTTTTACCAAATCAAGCGAATATTAT
TTAGAAAGTGTGGATAAGCAAAAAGAAAATACAGGGGGGTATAGGCTTTTTCGCATATACGATTTTGAGGGAAAG
AAGAAGTTTTTTAGGCTGTTCACAGCTCAGAACTTTCAAAAGTTAACAAATGCGGAAGACGAAATATCCGTTCCA
AAAGGGTTTGATGAGCTAGGCAAATCTCGTACCGATTTGAAAACTCTCAACGAGGATAATTTCGTCGCACTCAAC
AACACTGTTAGATTTACAACGGACAGCGATGGACAGGATATATTCTTCTGGCACGGTGAATTAAAAATTCCCCCA
AACTCAAAAAAAACTCCGAATAAAAACAAACGGAAGAGGCAGGTTAAAAACAGTACTAATGCTTCTGTTGCAGGA
AACATTTCGAATCCCAAAAGGATTAAGCTAGAGCAGCATGTCAGCACTGCACAGGAGCCGAAATCTGCTGAAGAT
AGTCCAAGTTCAAACGGAGGCACTGTTGTCAAAGGCAAGGTGGTTAACTTCGGCGGCTTTTCTGCCCGCTCTTTG
CGTTCACTACAGAGACAGAGAGCCATTTTGAAAGTTATGAATACGATTGGTGGGGTAGCATACCTGAGAGAACAA
TTTTACGAAAGCGTTTCTAAATATATGGGCTCCACAACGACATTAGATAAAAAGACTGTCCGTGGTGATGTTGAT
TTGATGGTAGAAAGCGAAAAATTAGGAGCCAGAACAGAGCCTGTATCAGGAAGAAAAATTATTTTTTTGCCCACT
GTTGGAGAGGACGCTATCCAAAGGTACATCCTGAAAGAAAAAGATAGTAAAAAAGCAACCTTTACTGATGTTATA
CATGATACGGAAATATACTTCTTTGACCAAACGGAAAAAAATAGGTTTCACAGAGGAAAGAAATCAGTTGAAAGA
ATTCGTAAGTTTCAGAACCGCCAAAAGAATGCTAAGATCAAAGCTTCAGATGACGCTATCTCTAAGAAGAGTACG
TCGGTCAACGTATCAGATGGAAAGATCAAAAGGAGAGACAAAAAAGTGTCTGCTGGTAGGACAACGGTGGTCGTG
GAAAATACTAAAGAAGACAAAACTGTCTATCATGCAGGCACTAAAGATGGTGTTCAGGCTTTAATCAGAGCTGTT
GTAGTTACTAAAAGTATTAAAAATGAAATAATGTGGGACAAAATAACAAAATTATTTCCTAATAATTCTTTAGAT
AACCTAAAAAAGAAATGGACGGCACGGCGAGTAAGAATGGGTCATAGTGGTTGGAGGGCATATGTCGATAAGTGG
AAAAAAATGCTCGTTCTAGCCATTAAAAGTGAAAAGATTTCACTGAGGGATGTTGAAGAACTAGATCTTATCAAA
TTGCTTGATATTTGGACCTCTTTTGATGAAAAGGAAATAAAAAGGCCGCTCTTTCTTTATAAGAACTACGAAGAG
AATAGAAAAAAATTTACTCTGGTACGTGATGACACACTTACACATTCTGGCAACGATCTGGCCATGTCTTCTATG
ATTCAAAGAGAGATCTCTTCTTTAAAAAAAACTTACACTAGAAAGATTTCCGCTTCTACTAAGGACTTATCGAAG
AGTCAAAGCGACGATTATATTCGCACAGTGATCCGGTCCATATTAATAGAAAGTCCTTCGACCACTAGAAATGAA
ATAGAGGCGTTGAAGAACGTTGGAAACGAATCAATAGATAACGTCATCATGGATATGGCTAAGGAAAAGCAAATT
TATCTCCATGGCTCAAAACTTGAATGTACTGATACTTTACCAGACATTTTGGAAAATAGAGGAAATTATAAAGAT
TTTGGTGTAGCTTTTCAGTATAGATGTAAGGTTAATGAATTATTGGAGGCCGGAAACGCTATTGTTATCAATCAA
GAGCCGTCCGATATATCCTCTTGGGTTTTAATTGATTTGATTTCGGGAGAGCTATTGAATATGGATGTAATTCCA
ATGGTGAGAAATGTTCGACCTTTAACGTATACTTCAAGGAGATTTGAAATACGAACATTAACTCCCCCTCTGATT
ATATATGCCAATTCTCAGACAAAATTGAATACAGCAAGGAAGTCTGCTGTCAAAGTTCCACTGGGCAAACCATTT
TCTCGTTTATGGGTGAATGGATCTGGTTCCATTAGGCCAAACATATGGAAGCAGGTAGTTACTATGGTCGTTAAC
GAAATAATATTTCATCCAGGGATAACATTGAGTAGATTGCAATCTAGGTGTCGTGAAGTACTTTCGCTTCATGAA
ATATCAGAAATATGCAAATGGCTCCTAGAAAGACAAGTATTAATAACTACTGATTTTGATGGCTATTGGGTCAAT
CATAATTGGTATTCTATATATGAATCTACATAA

>YDL118W 0.81 Cold
ATGTTTGAATCGTTTGTTAATGAAGGCACAGCATTCCTCCTCTTTGCCAAAGATGAGCGCATAAGGTTCAAACAT
GATAAATATAAGGCGCTGCCTATTGATGTTCGTAATGCTGAAGGTAATGTACCTCTCCATAGCTGTAGAGGGCTG
TCTATCTCTTTCAAATTTTTCCATAATGTAGCTTTCTTGTCTTGCTGGATTCTCGTCTTCAAAAGGTCTAAAGGT
TGCAACGCAACAGCAGATGTTAGCCCACCGAAAAAGCCCCCGATCAAATGCGATGAATTTCTTGGCTTAGTTGCT

TGCTCAGTCATGTATGCTGCTTCACAAATTGTGTACCTTCTAGTTGTTCCCGCTGTGCTATTTGCTTTTCTTTTG
GTATAA

>YPL211W 0.7Cold
ATGAGACAGCTAACAGAAGAAGAGACCAAGGTTGTTTTCGAAAAACTCGCCGGCTATATTGGTAGAAATATTTCT
TTTTTAGTGGATAATAAAGAACTTCCTCATGTCTTCAGATTACAAAAGGATAGAGTATATTACGTACCCGATCAT
GTTGCAAAATTGGCTACTAGTGTAGCAAGACCTAACTTGATGTCTCTGGGTATTTGTTTAGGTAAATTCACGAAA
ACTGGAAAATTCAGGTTACATATTACTTCTTTGACAGTATTGGCTAAGCATGCCAAATATAAGATATGGATTAAA
CCTAATGGGGAGATGCCATTCTTATACGGTAATCATGTGTTAAAGGCACATGTGGGTAAAATGTCTGATGATATA
CCAGAACACGCCGGTGTTATTGTGTTTGCAATGAATGATGTGCCATTAGGGTTCGGTGTCAGTGCGAAAAGTACT
TCTGAGTCAAGAAACATGCAACCCACTGGTATAGTTGCTTTCAGACAAGCAGATATTGGTGAGTATTTGAGAGAT
GAAGACACCTTGTTTACTTAG

>YGR002C 0.79 Cold
ATGTCATCATCAGACATCTTTGATGTACTAAACATTAAGCAAAAATCCAGAAGTCCAACTAATGGCCAGGTATCA
GTGCCTTCGTCCTCGGCAGCCAACCGGCCCAAGCCTCAAGTGACGGGCATGCAAAGAGAACTATTTAACCTGCTA
GGAGAGAACCAACCCCCCGTTGTTATTAAGTCTGGAAATAACTTTAAAGAAAAAATGCTTTCGACGTCCAAGCCA
TCTCCTTGGTCATTTGTAGAGTTCAAAGCTAATAACTCTGTGACTCTGCGTCATTGGGTCAAGGGTTCCAAAGAA
TTAATAGGAGACACACCAAAAGAATCGCCATATTCTAAGTTCAATCAGCATTTATCTATTCCATCATTCACCAAG
GAGGAGTACGAGGCTTTTATGAATGAAAACGAAGGAACTCAAAAAAGCGTAGAAAGTGAAAAAAACCATAACGAA
AACTTTACTAATGAAAAGAAGGATGAAAGTAAAAATAGCTGGTCTTTTGAAGAAATAGAATACCTGTTCAATCTC
TGCAAAAAATACGATTTACGTTGGTTCCTGATCTTTGACAGATACAGTTATAATAATTCAAGAACACTTGAAGAT
CTCAAAGAAAAATTTTATTACACCTGTAGGAATTATTTTAAAGCAAGTGATCCTAGCAATCCGCTTTTATCTTCT
TTGAATTTTTCAGCTGAAAAGGAGATAGAGAGAAAAAAGTATTTACAGCGTCTTCTTTCTCGTTCTGCAGCAGAA
ATTGCTGAAGAAGAGGCACTCGTAGTAGAGTCCAAAAAATTTGAAATGGCTGCCAAGAGAACACTTGCAGAAAGA
GAATCATTACTCCGACTGTTGGACTCGCCACATTCAGATCAGACAATTACACAATACCTTACCTCTCAGGGAATG
TCTCAGTTATATAACGCGCTTCTAGCAGATAAAACTAGAAAGCGGAAGCACGATTTGAACATCCCTGAAAATCCT
TGGATGAAGCAGCAACAGCAGTTTGCCCAACATAGGCAACTTCAACAACTGAATGTAAAAAAGTCAGAAGTCAAG
GAAAATCTCTCCCCCAAGAAAACCAAAAGACAAAGACAGGAAATGCAAACGGCGCTCAAAAGAAAGTCAGAAAGC
GCATATGCAGAACAATTGCTGAAAGATTTCAATTCAGATGAAAGGAAGGCTTTAGGTGTGATTACGCATGGTGAG
AAGCTATCGCCGGGTGTATACTTAAGATCTACAAAATTATCGACCTTCAAACCTGCGCTGCAGAACAAAATTCTC
GCCATTCTTCAAGAATTATCGTTACCTTCTCGCCCTGTGATGCCCTCATTTGATGTAATGGAAAGACAAGAGGAG
CTTTTGAAAAAAATCAATACGCTTATCGATTTAAAAAAACACGTTGACAAATACGAAGCTGGTATGTCAATCACC
AAGTGA

>YOR130C 0.65 Cold
ATGGAGGACAGTAAAAAGAAAGGATTAATAGAAGGCGCTATACTCGATATAATAAACGGTTCCATTGCAGGCGCC
TGTGGTAAGGTGATCGAGTTTCCTTTCGATACTGTGAAAGTCAGGTTGCAAACACAAGCATCCAACGTGTTCCCA
ACAACATGGTCTTGTATAAAATTTACTTACCAAAATGAAGGAATAGCACGAGGGTTTTTTCAAGGCATTGCTTCA
CCTTTAGTTGGAGCATGTCTGGAGAACGCGACATTATTTGTGTCTTATAACCAATGTTCTAAATTTTTAGAAAAA
CATACAAACGTTTTCCCGTTGGGGCAAATCCTGATCTCTGGTGGAGTAGCGGGTTCATGTGCTAGTTTAGTTTTG
ACACCCGTGGAGCTGGTGAAGTGTAAGTTGCAGGTTGCGAACTTACAAGTTGCATCAGCTAAAACGAAACATACA
AAGGTGTTGCCTACAATAAAAGCAATTATAACTGAGAGAGGATTGGCAGGATTGTGGCAAGGGCAATCGGGCACT
TTTATTCGAGAAAGCTTCGGTGGTGTTGCCTGGTTTGCAACCTACGAAATAGTTAAGAAGTCGTTGAAAGATAGG
CACTCCCTTGATGACCCAAAAAGAGATGAAAGTAAGATATGGGAACTACTTATTAGTGGAGGGAGCGCTGGATTG
GCATTCAACGCCAGTATTTTTCCTGCGGATACTGTGAAATCAGTAATGCAAACTGAACATATAAGCCTCACCAAT
GCGGTGAAGAAGATATTTGGCAAATTTGGACTAAAGGGTTTTTATCGAGGACTGGGTATAACCCTTTTTAGGGCA
GTACCAGCAAACGCTGCAGTTTTTTACATCTTTGAGACTCTTTCTGCACTTTAA

>YLR195C 0.78 Cold
ATGTCAGAAGAGGATAAAGCGAAAAAATTAGAGAATTTATTGAAGTTATTACAGTTGAATAATGACGATACTTCA
AAATTCACTCAAGAACAGAAAAAAGCTATGAAAGACCACAAATTCTGGAGAACGCAACCGGTCAAAGATTTCGAT
GAAAAGGTGGTGGAAGAAGGCCCCATTGATAAGCCAAAGACACCGGAAGATATATCTGACAAGCCACTACCTTTA
TTGTCTAGCTTCGAATGGTGTAGTATTGATGTGGACAACAAAAAACAGCTTGAAGATGTTTTCGTTCTACTAAAT
GAAAACTACGTGGAAGACCGCGATGCAGGCTTCAGATTTAACTATACCAAAGAATTCTTCAATTGGGCTTTAAAG
AGTCCAGGTTGGAAGAAGGATTGGCATATTGGTGTTCGCGTTAAAGAAACACAGAAATTAGTTGCCTTTATCTCA
GCCATACCAGTAACACTTGGTGTTAGAGGTAAACAAGTGCCTAGTGTAGAAATCAATTTCTTGTGCGTTCACAAA
CAGCTAAGATCGAAGAGATTAACACCTGTTCTAATTAAAGAAATTACGAGACGAGTGAACAAATGTGACATCTGG
CATGCATTGTACACGGCAGGTATTGTTTTGCCAGCACCTGTGAGTACGTGTCGTTATACTCATCGTCCCTTGAAT
TGGAAGAAACTTTATGAAGTAGATTTCACAGGGTTACCAGATGGGCACACAGAGGAGGATATGATTGCTGAGAAT
GCGTTACCGGCCAAAACAAAGACAGCGGGATTGAGAAAATTAAAGAAGGAAGATATTGACCAAGTTTTTGAGTTG
TTCAAAAGATATCAATCCAGGTTCGAACTAATTCAAATTTTCACAAAAGAAGAATTCGAACATAATTTCATTGGT
GAAGAATCGTTACCATTGGATAAACAAGTAATTTTCTCATATGTAGTCGAACAGCCCGATGGAAAAATTACAGAC
TTCTTCTCATTTTACTCATTGCCATTCACAATCCTAAATAACACAAAATATAAGGACCTAGGCATCGGGTACTTG
TATTATTATGCCACCGATGCAGATTTCCAATTCAAAGACAGGTTTGATCCAAAAGCTACTAAGGCTTTGAAAACA
AGATTGTGTGAATTGATTTATGACGCTTGTATTTTGGCCAAAAACGCTAATATGGATGTTTTTAACGCGTTGACT
TCGCAAGATAATACATTGTTCTTGGATGATTTGAAGTTCGGGCCCGGTGACGGGTTCTTGAACTTCTATTTATTT

AATTATAGAGCAAAGCCGATTACCGGTGGCTTGAATCCCGACAATAGTAACGACATTAAAAGGCGTAGCAATGTC
GGTGTTGTTATGTTGTAG

>YDR238C 0.82 Cold
ATGACTTCACTTTCTTCACAGCCAGCGTACACGTTGGTTTTCGATCCTTCTCCGAGTATGGAGACTTACTCGAGT
ACCGATTTTCAGAAAGCTCTTGAAAAGGGATCTGATGAACAAAAAATTGACACGATGAAATCAATTTTAGTTACA
ATGCTGGAAGGAAATCCAATGCCTGAATTGTTGATGCACATAATAAGATTTGTCATGCCTTCTAAAAATAAGGAA
TTAAAAAAGCTTTTGTACTTCTACTGGGAAATTGTTCCCAAACTAGCTGAAGATGGAAAATTGAGACATGAAATG
ATTCTTGTCTGTAATGCCATTCAACACGATTTGCAACATCCTAATGAATATATTAGAGGTAACACATTAAGGTTT
TTAACGAAATTGAGAGAGGCCGAACTCTTAGAACAGATGGTTCCCTCTGTCTTAGCGTGCTTGGAATACCGTCAT
GCATATGTTCGTAAGTATGCAATCCTAGCAGTTTTCTCCATTTTCAAGGTCAGCGAACATTTACTTCCCGATGCT
AAAGAAATCATCAATTCGTTCATAGTAGCTGAAACTGATCCAATATGTAAAAGAAATGCATTTATTGGGTTAGCT
GAATTAGATCGTGAAAATGCCTTACACTATTTAGAGAACAATATTGCTGATATAGAAAACCTAGACCCTTTATTA
CAAGCTGTCTTTGTTCAATTTATCAGACAAGATGCAAACAGGACCCCTGCTTTGAAAGCCCAATATATCGAATTA
TTGATGGAACTGCTTTCGACCACGACTTCCGATGAAGTCATCTTCGAGACCGCATTAGCCCTAACTGTGTTGTCT
GCCAATCCAAATGTCTTGGTTCCTGCGGTTAACAAATTGATTGACTTGGCCGTCAAGGTTTCTGATAATAACATT
AAGTTAATTGTTCTAGACCGTATTCAAGACATCAATGCTAATAACGTAGGTGCTTTGGAAGAGTTAACCCTGGAT
ATTTTGAGAGTCTTGAATGCAGAAGATTTAGACGTTCGTTCAAAGGCGCTTGATATTTCAATGGACTTGGCCACA
TCCAGAAATGCTGAAGATGTTGTTCAGCTTTTGAAGAAAGAGCTGCAAACAACCGTAAATAACCCAGATCAAGAC
AAGGCAATGCAGTACAGACAATTGTTAATAAAAACTATTCGTACCGTGGCTGTAAACTTTGTAGAAATGGCAGCA
AGTGTTGTTTCGCTATTATTAGATTTCATCGGTGATTTAAACTCGGTTGCCGCCAGTGGTATCATTGCCTTTATC
AAAGAAGTGATCGAAAAATACCCACAACTTAGAGCCAATATCCTTGAAAACATGGTTCAAACGCTAGACAAAGTG
AGATCTGCTAAAGCTTACCGCGGTGCATTATGGATTATGGGTGAGTATGCTGAAGGAGAAAGTGAGATACAACAT
TGTTGGAAGCACATTCGTAACAGCGTAGGTGAAGTTCCTATCCTTCAATCAGAAATCAAAAAGTTAACACAAAAC
CAAGAACACACCGAAGAAAATGAGGTTGACGCTACCGCCAAGCCAACTGGTCCAGTTATTCTACCAGACGGTACG
TATGCCACTGAAAGCGCTTTCGATGTGAAGACTTCTCAAAAGTCAGTTACCGATGAAGAACGTGATTCTAGACCT
CCAATTCGCCGGTTTGTTTTAAGTGGTGATTTCTACACAGCTGCCATTCTGGCCAACACCATCATTAAACTTGTT
TTAAAATTCGAAAACGTTTCCAAGAACAAAACTGTCATCAATGCTCTAAAGGCGGAAGCTTTACTAATTTTAGTT
AGTATTGTAAGAGTGGGTCAAAGCTCTTTGGTGGAGAAAAAAATTGATGAAGATTCTTTAGAGAGAGTTATGACA
TCTATTTCTATTTTATTGGATGAAGTTAATCCTGAGGAAAAGAAGGAAGAAGTTAAACTTCTGGAGGTTGCATTC
CTGGACACCACCAAATCCTCATTCAAGAGACAAATTGAAATTGCAAAGAAGAACAAGCATAAGAGAGCATTAAAA
GACAGTTGCAAAAACATCGAACCAATTGATACGCCGATTTCTTTCAGGCAATTTGCTGGTGTGGATTCTACTAAT
GTGCAAAAAGATAGTATTGAAGAAGATTTACAACTGGCAATGAAAGGAGATGCAATCCACGCTACTAGCAGCTCT
AGTATTTCGAAGCTGAAGAAGATAGTACCTTTATGTGGCTTTTCTGATCCAGTTTACGCCGAGGCTTGTATTACA
AACAATCAATTTGACGTCGTATTAGATGTTCTTCTTGTTAATCAAACGAAAGAAACATTGAAAAACCTACATGTG
CAATTTGCAACTCTTGGTGATTTGAAGATTATTGACACACCACAGAAGACCAACGTGATTCCTCATGGCTTCCAC
AAATTCACTGTTACTGTCAAAGTTTCCTCTGCTGACACAGGTGTCATTTTCGGTAATATTATTTATGATGGTGCG
CATGGTGAAGATGCTCGTTATGTTATTTTAAACGACGTTCATGTTGACATTATGGATTATATCAAACCAGCCACT
GCTGACGATGAACATTTCCGTACCATGTGGAATGCATTTGAGTGGGAGAACAAAATATCGGTCAAATCACAACTA
CCAACATTGCATGCTTATTTGAGAGAACTGGTCAAGGGAACTAATATGGGTATTCTAACACCATCAGAGTCGTTG
GGAGAAGATGATTGTAGGTTCTTAAGTTGTAATCTGTATGCGAAGTCGTCCTTTGGTGAAGATGCCCTAGCCAAC
TTGTGTATCGAAAAGGATTCCAAAACCAATGATGTCATAGGTTATGTTCGTATCCGATCAAAGGGACAAGGTTTG
GCTCTGTCCCTAGGTGACAGAGTGGCATTGATTGCTAAGAAGACCAATAAACTTGCTCTCACTCATGTTTGA

>YDL223C 0.73 Cold
ATGAATATGAACGAGTCCATTTCCAAAGATGGTCAAGGCGAAGAAGAACAAAATAATTTTTCGTTTGGTGGTAAG
CCCGGTTCCTATGATTCCAATTCAGATTCTGCTCAACGCAAAAAGTCTTTCAGTACTACAAAGCCGACCGAATAT
AACTTACCAAAAGAACAGCCCGAATCAACGTCCAAGAATTTGGAAACCAAGGCTAAAAACATTCTGTTACCTTGG
AGGAAGAAGCATAATAAAGACTCGGAAACTCCACACGAAGACACTGAGGCGGACGCTAACCGCCGTGCCAACGTA
ACTTCTGATGTGAACCCTGTTTCTGCCGACACCAAGTCAAGTTCTGGCCCTAATGCTACTATTACTACCCATGGA
TACTCTTATGTCAAAACAACGACACCCGCTGCTACAAGTGAGCAATCCAAAGTGAAAACGTCACCTCCCACTAGC
CATGAACATTCAAATATAAAGGCGTCCCCCACTGCGCATAGACATTCCAAAGGTGATGCAGGGCATCCAAGTATT
GCAACCACTCATAACCACAGCACTTCCAAAGCTGCCACTTCTCCTGTTACTCACACGCATGGTCATTCCAGTGCT
ACCACTTCCCCTGTCACTCACACACATGGTCATGCCAGTGTGAAAACCCACATCTCCTACCAATACTCATGAGCAT
TCTAAAGCGAATACTGGTCCATCAGCCACTGCAACTACACATGGACATATCAACGTCAAAACTACCCACCCTGTT
AGCCATGGTCACTCTGGTTCAAGCACTGGTCCTAAATCCACCGCTGCTGCACAGGATCATTCCAGTACTAAAACT
AACCCTTCAGTTACTCATGGACATACAAGTGTTAAAGATAACTCCTCAGCTACGAAGGGTTATTCGAACACTGAT
TCCAACTCAGATCGAGATGTCATACCTGGTAGTTTCCGGGGCATGACTGGTACTGATGTGAACCCTGTTGATCCT
AGTGTTTACACAAGTACAGGCCCAAAGAGTAATGTTAGTTCCGGTATGAACGCTGTCGACCCTAGTGTCTACACG
GACACAAGTTCGAAGTCTGCAGATCGTAGAAAGTACTCTGGCAACACTGCGACTGGTCCTCCTCAAGATACAATT
AAAGAAATTGCACAGAACGTGAAGATGGATGAGAGCGAACAAACTGGATTGAAAAATGACCAAGTTTCTGGTTCT
GATGCAATCCAACAGCAAACTATGGAACCTGAACCCAAAGCAGCAGTCGGGACAAGTGGTTTTGTAAGCCAGCAA
CCTTCTTATCATGATAGCAATAAAAACATTCAACACCCAGAAAAGAATAAGGTTGATAATAAAAATATATCAGAA
AGAGCGGCTGAAAAATTTAACATTGAAAGAGATGACATTCTGGAATCCGCCGATGATTATCAACAGAAAAACATT
AAATCCAAGACGGATTCTAATTGGGGTCCAATTGAATATAGTTCCAGCGCTGGTAAAAATAAGAATCTGCAGGAT
GTTGTTATACCTTCTAGTATGAAGGAGAAATTCGACAGCGGTACATCTGGATCTCAGAATATGCCTAAAGCGGGA
ACCGAATTAGGACATATGAAGTATAATGATAATGGTAGGGATAACTTGCAATATGTTGCCGGTTCACAAGCTGGT

TCTCAAAATACGAACAACAATATTGACATGTCACCCCGTCATGAAGCAGAATGGTCCGGGCTTTCTAATGATGCC
ACCACCCGAAACAATGTTGTATCACCTGCTATGAAGGACGAAGATATGAATGAAGATTCTACTAAACCTCATCAA
TATGGGTTGGATTATTTGGATGATGTTGAAGACTACCACGAGAATGATATTGACGACTACTCAAATGCAAAGAAA
AACGACTTGTATTCTAAGAAAGCGTACCAGGGAAAACCATCGGATTACAATTATGAGCAGAGAGAGAAAATCCCA
GGTACTTTTGAACCTGATACGTTGTCAAAATCAGTGCAAAAACAAGATGAAGACCCGTTGTCTCCAAGGCAAACC
ACTAACCGTGCTGGTATGGAGACTGCAAGAGATGAGAGTCTTGGCAATTACGAATACTCGAATACTTCTGGTAAC
AAAAAATTATCTGATTTAAGCAAAAATAAATCAGGCCCAACTCCAACGCGTTCTAATTTTATTGACCAAATTGAG
CCAAGAAGAGCAAAAACAACACAGGATATTGCATCCGATGCCAAGGATTTCACCAATAATCCGGAAACAGGTACT
ACTGGAAACGTAGATACAACCGGAAGGATGGGTGCAAAATCCAAGACCTTCAGCTCGAACCCTTTTGATGATAGT
AAAAATACAGACACTCATCTCGAAAACGCTAATGTTGCTGCCTTTGATAATTCCAGATCTGGAGACACTACATAC
TCCAAATCAGGGGATGCTGAAACAGCTGCTTACGATAATATTAAAAATGCGGACCCTACATATGCCAAATCACAA
GATATTACTGGAATGACTCACGATCAGGAACCATCATCAGAACAAAAGGCCAGTTACGGTTCTGGCGGCAATTCC
CAAAATCAAGAATATTCTAGCGACGATAATATTGATGTCAATAAGAATGCGAAAGTTTTAGAGGAAGACGCACCG
GGCTACAAGCGTGAAGTAGATTTGAAGAACAAACGTAGAACCGACCTTGGTGGTGCCGACGCCTCAAACGCTTAT
GCTGCCGAAGTTGGTAATTTCCCTAGTTTGATTGACCCACATGTCCCAACATACGGATTTAAAGACACCAACACC
TCTAGTTCTCAAAAGCCATCTGAGGGCACCTACCCAGAAACCACTTCCTATTCAATTCATAATGAAACAACTTCT
CAGGGAAGGAAAGTGTCGGTGGGTTCAATGGGTTCCGGAAAATCAAAACATCATCACAATCATCACCGTCACAGC
AGACAGAATTCTTCAAAAGGGTCTGACTACGACTATAATAATTCTACTCATTCAGCCGAGCACACTCCAAGACAT
CATCAGTACGGTTCCGATGAGGGTGAGCAAGATTACCATGACGACGAGCAAGGTGAGGAACAGGCTGGTAAACAA
AGTTTCATGGGTAGAGTGAGAAAAAGTATTTCTGGCGGTACTTTTGGATTTAGGAGTGAAATTTAA

>YGR291C 0.82 Cold
ATGGAACTTTTTATACCTTGCCCAGAGCGCCTCAAGAAAATGATGCTGCAAGAAGAATTGAGGAAGGAACTATTC
ATCTTACGTTGTTTGTATCATCCCACGATCCAAATCATGTTACCTACGTTAGGTACGCTAGGAACTAAAAAAAGA
AAAGAAAAGTATGCGTTATCACTCTTCGAGCCAATTCTTAATTGTGTGGGGTCCGCGAAAATTTCCGGATAA

>YDR141C 0.82 Cold
ATGTCCTTACCACTAAAGCCCCTTACAATTGACTCAAATAATAAACAACTAGACTCCAAACAGAAGAAGTTTCGT
GCTAATGTCGAGCGAGCATTAGAAAGATTTGACTCTGTAACAGAATGGGCGGACTATATTGCTAGTTTGGGAACA
CTATTAAAGGCGTTGCAAAGCTGGTCACCTAAATTTCAGAATGTAAGGTACTATGTTCCTTCTCCATATCAAGTA
AGTCGAAGATTGACATCCTCATTATCGCCGGCGTTACCAGCAGGTGTTCATCAGAAAACTTTAGAAGTATATACG
TATATCTTTGAACATATTGGCCTTGAAACTCTGGCTACAGAATGTAACATTTGGATCCCGGGAATTTTACCTTTG
ATGACTTATGCCTCTATGTCTGTTAGGTCACATTTGATAGAGCTTTACGATAACTATATCCTTCTGTTGCCTCAA
ACAACGTTAAGACTGCTCATCAGACCTTTGATTTCTAGTTTATTGCCAGGAATTGATGATGAAAGCAACGATTTT
TTACCTTTAACTTTAAAACTCATTGAGACTCTGCAGGAGAACTTGGATGATGATTCCTTATTTTGGCAAACGTTG
TTTCTAGTCATGACTGCAAATAAAGGCAGAAGACTGGGCGGACTCACGTGGTTGACTAGAAAGTTTCCGTCGTTG
AATGCTGTACCTCATCTAGTAAATAAAATAAAAATGGAAGCGGAAGAGAACCCAAGTGAAACTGAAACCAACGAT
TCTCATCTAGACAGGAAAAAAAGAAAAGAAGAAGCTTTCAAGGTCTTATTGCCTGCTGCCAAAGATTTAGTAACC
CCTGAACCAGGTCTACTTATCCGATGCCTTGTCGGTTGTTTAGAAGATGAAAATGATATTCTTATTAAAAGGAGC
GTTTTGGACCTTTTATTACAGAGGTTGAGGCTAGACTCTCCCGTTTTGAATGTTCTTATTACTTCTGAGGATAAA
AAGTTATTGATAATGAGTTGTTGTAGAACTACTTTGAGCAAGGATATGTCTTTGAACAGAAGAATATGGAACTGG
CTTCTCGGTCCTACTGCTGGGGGCATGCTAAACAATAACGGCGGGAACTCCATGGAATATACTACCTCGGTTAAG
TCAGCAAACGAGGAAAGTAATGTATATTTTACAAAATATGGATTAAGCGCCCTTTTAGAAGGTTTAAGCGACCTT
CTTTCAGAAGAAGAATCCGTGTTAACTGCATTCAGGATAAGTATGGCAGTAATGGATAGATGGGAAATTGGCTCA
CTTGTAATTCCTGAATTGTTCATCCCACTTCTCTATTCCTCGGAAAAATTTAAACAAAACGAACAAATAATGAAA
ACGGCACGTACTTTCTTTGACAATACTGAAACAAATATTATATGGGGAAAGCTATTTCAAGAACTTGAAGACATC
AAAAACCTAAAAATTTTGGATTTCGTATTAACAAATTTTAATATTGGAAACGACGAAGAGATTATCGTACGCCAC
CTTCCTTTGATATTATTAACTTTACTGGCCCTTCCATCTAATGATAAAGATTTCGACAATATTTATAAGCTCCAA
AAATTTTCTTTGTACAACAAATTGTTAAACTATATCCCCGAGAGAGCCCTTCTCCCTCTCAGTCACTCAAAACTA
AAGCACGATGATGAAGTAAGCTGCGAAGAACTTTTGGCCAAAATACGTGGGTTTTATACCAATGTTTCTAATCCA
TCTAGCATTTTAGAGAAAGAAAATATAGCTGAGCGTTTGCCACCCTTTACAACAGAAGATCTAACTTTTTTAATA
GCAGACCTGATTCAGAAGAAGCTTCTTTCAAGTTTATGGGACTTGGAAAATATCAATGAAAGCTCCAAATTATTT
ATAGCTATTTTCGAAAAGATACCTGAGTCTGAAGAACTTAAAGGACGATCTCACATAAGCTGGTCGGATAAAAAA
ATAACTCAGAGCATATTTGAGGCTATTCCCAGGCTTTGTGAATCTAATAATGATGCAAAATCAGAAGAAATCGTT
GGAATTGTGGAAATTTTTGGTAACTACTTATATTCACGCATGGAATTCATTGAATCGATGAAATTATTGAAAGTA
GTCATGATGGCCGTATGGAAATCTTTAAAAGATCCACGCCATCAAATACTAGGTGTCAAGAACTTAAAGACTTTA
AACAGATTTATTCCATCCAAATTTATTGAAAGTGCGTTAGTGTATACTTTTGTGGAAGAGGAAGATATATCCGAG
AGATTAAGCGTGTTAGATCTGTTATGGACACAATTAGACTCAGATTCAAACTTGATTAGGCGCCCTCTTGAATTA
ATTTTGGGCGAACTTTTTGATGACCAGAATCCTTTTTATTTAACCGTTTCAAAGTGGATTTTATCGATATTAAAC
TCGGGATCTGCTTCAAGATTATTTTACATTTTGACTGATAATATTTTAAAGGTTAATCGTCTCGAAAAAGAAAGA
TTAGACGAAAGGGATGATCTTGATATGCTCACATATGAGTTCCAAATGCTTGCTTATGTTTTGAAAACAAACAAT
GGACGCACTAGGAAAGTTTTTTCCACTGAGCTTACCTCAATAAAATCTTCGACCATATGGAAGAATGAAGACGTT
TCCACATATAAAAGTTTGCTGTTGGTTACATTGATGAGATTTCTAAATATAAAGAGCAATACACATGCGAAAAGT
ATCAGGAGTGCTCTGATTCTTTTGGATATCTTACTCGATGGAACTGAGCAAAATTTCAAGGACATTGTCATATTT
TTGCTGCAAATGTCGTCTAAATATATTGCAGAAGAAGGAATTGAGCCCGAGTTAATAGCAGTTTCCTTGTTAGAT
ATTGTATCGAAGGTTCTCAGACTATCACACGATAATGGTATTAAACTAGACATTTTTGATGACAATGCTGCCCAT
TTAAAATATATCGATTTCCTTGTTACCAGCGTTTCAAATATGAAAAGCCCTCTTATTGTAACGGCCTATGTGAAG

CTTCTTTCCGAAAGCATTGTTTATTTTGAGAATTCTATATTTCGAATGATTTTACCATTGTCTGCATCTCTTGTA
CAGTGTGTTCAGAGATTGTTTTTGCTAGAAAAGAGAGAAGGTGGTTATTACCAACCAATAGCTTTGCTTCTGGGT
GGTCTGGAAGAGCTATTAGAGATTTCACATGGTTACCTTGTCACCGAGGAAAGGGAAGGATACTTTTCTGGGTCT
AATCTAAAGGGTGATTTTATTCAATCCGTTGTTTCAAACGTTTTTTCGTCAGATTCTTCCAATGAAGAAAGTAAG
ATTCAGGGGGAAAGAGACGTAATACTACAATCTTTCAGACAGGTGATTTCATGCTGTTTAGATATCTGGTATTGG
GCCCATAACATTTCGTGTAAATCTAACGATGATTCTAGCCTGGACGCCACTAATCATAACTCATACAAGTTCAAA
TTTAGGTCGAAGAAACTGTTGGAAACCTTATTTCTACTAGAACCTTTGGAACTTCTGGAAAATTTGATCAGCATT
AGATCAGACAATACTACAGTCACACTAGTACATGTGCTCGACGGCAATAAACCCGCCATTACAATACCACATTTA
TTGTATGGTGTAATTATCAGATACAACAGAACGGCATCTGTCAAGTTTTCTAATCGTGACGGAAGTAGGTCAAGC
ACAACTAAATTAACTAAAGGGGAGCCTTCCATGTTAAAAAGATTAAGCGGGGAATCGATTATTGCATTTTTGTTT
AACTACGTGGATTCTGTAGAAAACTCTGCAATGGAGGAGTTTTATGGGGATTTCCTGCTATTTTTCAGAGAAGTA
GCAACCAATTATAACCTTTATTCTGATGTTTCGTTGTCTATATTAAAACTTGTTGCCCTTATTTCTGGAAAAGTA
AGTAAAACGCAGTTTGGAGAACAAAAACGAGTTAGGAGGGAGATATCTGATGTGTTTTTCAAATACCTACCTAAT
GCATTTATAAACTTTACGAACTTATATCGTGGCCACCCTGATTCATTTAAAGATTTAGAATTTGTAGTATGGCGT
GTTCAATATATCGTCAACGATCAAATTGGAGGAGACAAGTTTAATACAACGTTAGCGACAATTGTAAATCAATGC
CTAACCCCTTATATCAAACCCAAAAGTGAAAAAACTATTCCAGGTTATGTCTTAGAATTGGCCGCGGTCGTATCC
CATTTAGGTTCAAAAGTTAAAAGTTGGAGGCTTTTAATTGCGGAATTGTTCCAAAATGACAAAAAACTTTCGGTA
ATTGGCAGCGATCAAACTTGGGAAAAGATTATTTACGAATGGTCCATTTATCCAGAAAATAAGTCAAAAATCTTG
AACGATTTACTATTAGAAATTGGCTCCAAGCGTTCAAGTGTGACTCCGACTTTAATCACGTTTAACTTAGGAAGC
GATTCTGAAGTCGAGTACAAGTGCCAAAACCTTTTGAAAATATCGTACTTGTTGATGGTATCGCCAAATGACGCA
TATTTGTTGCACTTTTCCTCTTTAATAAGTTGCATTTTCCACTATTTGGTGTCCAAAGATATCAAGCTCAAGGGA
AGCTGCTGGATCTTACTAAGGGTTTTACTTTTAAGATTTTCAGAGTCCCATTTCAATGACTATTGGTCTATGATC
AGTTACTGTTTACAAACTAATTTGCAAGAATTTTATGAATCACTTCAAATACAGTCAGAAGTCGATCCACAAACA
ATATTGCAAGTATGTAAAACTTTGGATTTGCTACTCTTACTCAACATGGAAGGCTTCACCTCTACGAATGAGTGG
ATCTTTGTTATTGATACAATAAATTGCGTATATAAAACGAACTCATTCGTCGCGCTGGTAGATGAAATCGCAGAA
TTCAAAGATTACGAAATAACCAAAACTGATGATCTTGAATTACCGACAACTTTAAAAGATGGTCTCCCATTATTA
CGAGGCATTCACAAAATCGAGAGACACACGCAACTAAGAAGCTTCTTCCAGAATTTGAGTTATCTACATTATGAG
AAAGTTTACGGACTAGGGTCAGTTGATTTATATGGTTGTGGTGAAGATCTCAAAAAAGATATTCTGTCATGA

>YLR161W 0.78 Cold
ATGAAGTTCCAATATGCGTTGGCCAAGGAACAGCTAGGCAGCAACTCGCGCAGTGGCGTCAAAAAACTAATAAGT
AAACACCACTGGCTTCCGGAATACTATTTCTCTGATCTCTCATTTTCTGTTGTACAGCAGTGGGACAGTAGAGCC
ATTGAAAAAACTACAATCATATCTTGTATGCGGCCCGCAAACCAAGAGATTTATCCTTTGAGACATTGTGAGACC
CTCCGTTCGCAACCGTGCTCTCTGTTTTCATCACTATATGCACGCTCTTTCCAAAGCTCCTGCACTTTGCACGTG
GCGGAGCCATCGCCCGGCTTCCATATGTACGGCTGCCACACCTAA

>YER163C 0.82 Cold
ATGACTAATGACAACAGTGGTATCTGGGTCCTAGGCTACGGATCTCTGATTTACAAGCCTCCGTCTCATTACACG
CATAGAATACCGGCAATAATTCACGGTTTTGCTCGTCGGTTCTGGCAAAGTTCCACTGATCATAGGGGTACACCT
GCAAACCCAGGAAGAGTGGCCACACTAATCCCGTATGAGGATATTATAAGGCAAACTGCCTTCTTGAAGAATGTG
AATTTGTATAGTGAAAGTGCACCTATCCAGGATCCCGACGACTTAGTCACCATAGGGGTAGTGTATTATATACCA
CCAGAACATGCTCAAGAGGTTAGAGAGTATTTGAACGTAAGAGAACAAAACGGGTACACTCTACATGAGGTTGAG
GTGCATCTAGAAACTAATCGGGAGCACGAGGCAGAATTGGGTGAAGCATTAGAACAGTTGCCTCGGCACAACAAA
TCGGGTAAACGGGTACTACTTACTTCGGTGTACATTGGTACAATAGATAATGAGGCCTTCGTCGGACCAGAGACC
GTGGATGAAACTGCGAAAGTAATTGCCGTGTCGCATGGACCTAGTGGATCTAACTATGAATACCTTGCAAAACTG
GAGCAAGCGCTAGCGCAAATGCCCATCATGAAAGAGCGAGGGCGTATTACCGATCATTATCTGACTGCTCTATTG
GAGACTGTAAATAAATACAGGTGA

>YLR368W 0.78 Cold
ATGACAAAGAGGAGAAACCTCTTTATGGTTGGTTCTTCCTTCACAATAGATCATTTACCTCCAGAAATATGGCTT
TGTATTTCTAAGCTGGTCGGGACGTCTGACCTGCATAACTTGTGTTTAATTAACAGAAGATTGTATTTGACAATT
ACAAGTGATGAAATTTGGAAAAGAAGATGCTATGACCGATGGATAAATCGAGAAAGCTTAGATATTTTAACTGGT
AATGATTACGATTCTATACCCGTCTCTCAATGGTATTCTTACTATTTAAGAAGAGCAAAATGGGAGAATAAAATT
TTTTGTCTATTATGGGGGTTAACTGAAGAAACAAATCCCCAACATTTCAGAGAGAAATATTTGCATATCCTCCAA
TTTAGACATTACAAACTGGCTACATTTCTGCATAGGATTATAAAACAAGGCTACATACCTGATAAAAGACCACTT
GATTTAATCACGTATGCAAACTATCTGTTGAAAAATGTGCGACATAAGTACGTATTTCCTTTATTTTATCCCACA
AATGCCGCTGAATTGAAAAATCTTAATAATATGGCCTCAAGGGATGCTGAAATGATATATTTAAGATTATCTGCC
ATTGATACGTCTTTTGATGACTTACTAGATGCTAGAGAATTCATATTAAATGGAATATGTTCCGATCTACTACAA
AAGTATAAAAAAATTGAAGAGTTTTTGAAATTACGACCGGTGACTAGGGTTTCCAAGTTAATCTCAATCAGTACT
GACTATCTGGACTGCTTCACACAACCTCATGATTCAGTGGGTCAAACAAATGATAGGGCCACAGGAAGGGAGCTA
CATAGAGAGGATTTCATGTTACTAAGAGTATATTCCCGAGAAGGACGAGGTTATAAAACTATCATTCTAGCAATA
ATACAGGCTATCACTAAGAGATATAACGTTGATTCATATCTTGCTCGAGATCATTTGGTGGTGTCTGAACCAGAC
TTTCCAGATGGACGGGCATTTGTTACCGTGAATGAGGATTTCCAGCCATACATTTTTGATAAAGAGGACCTTTTG
AGCGTTTGGTCGAACAACTTCCATAACGCAGAAAATTTTGAAAGTACAGTTTTACCAGCATTATTAGAACCAATA
TCGATACAACATCTTCTGACGGAGTTCTTTCGAGAACTGCTGCGATGCAAGCCCAGGCCATTTGAGGGATATCCA
AACAGAGCACACGGATTACGCGATATGTTTCCATACGGAAAAGTGGAAGTTCCCAGAGATGTGACGATGTATTTT
GCATTTATATACGATCTTTTCGATGGCATGTTTGAATCTGGAATGACAAGTTTACGGGGCCAGATGTTGAGAGAT

TTACTGAACTACGTTAACGCTAATAATTTTGGTGATTTGAATATCATCATTGGACAAAATGCTCTTAAGGAACCA
AATGACTGCTGGTCCAACAAGAGGGATTACGTTCTTCTTGATGACAATAATAAGATTGGATATTTCTATACGGAT
ATTGAAACTGAGGATACTTTATGTGCCTTAAACCAATATGAAGTGGACGGCAAAGTTTTTATAACAACAATAGAT
ATCTTGGGTGATATAAGAGTACGCCTGGCTGAAGGACTAACGCCTTTTCAGGGAGATAATGATAAGTTGTGGGAG
AGCTTTTCTTCCGTTGTTCCACGAACTGACTGGGGACTTTTTTTCAAAGGCTATGACAAAGAAAGGCGTAGAATG
CAATTAAACCCTTACATAGAGGAAAAGCTTTCCAATCTAGCGAATGATGAACAGCCTTTACATAATTTATAG

>YNL313C 0.82   Cold
ATGGAGACGTTATTGCATGCAAAATTATTGCTGTCTGCAGAAGTAGAATCTCTGAAAAGTGGTTCTTTCGACCAG
ACATATGTGAAAAAGGCTGAACATATCATCAGTGGTGAATCATACCAGCTAGTACAGCAATTTGTGGACAAATTT
AAGGGAAAAATTAGTATTTCGGGAGAAATATCCACATCTAGTGTTATTGCTGCTCTAAATGACTTTCTTAACGTT
GAGGTTTTCAAAATGGGCCAAGAAAATGAGATGCTATTTTTGGCTATTGCCCTCTTACAGACCTTTATTCAAAAT
AACTACACCGGCCCAGCAGCTAGGTTGAAAGCAATCTCAGGTTTGTTTGGCAAAACTGGAATTGAAATAGGAGCA
GTTAATACAGCCCTTTCCCGCTCATTAGCCATTATGGGCCAGCCTGCCTACGAATTTATGGATGATCCATTGTAT
TTGGTTTTATCACTATTATTACTTGAAAGGATAACCGGCCAAAAAAGCTTATTTGATGTTACCCCGGATCAAGAA
ATTCCGCTACCTATTATCTCTGCTGAGTCTACTCCTGGATTATTAGCTGTAGCTTATTGGTGGTGGGCAAGAGCT
CTTTTGACGCAATTGTCACTGATTCCAGAACCTTCTGGGTTCCAAGCTAGTGTTGCATCTGCAATATATCAGTCT
GCTGACCTTGCATATGCGATAACTAAGGAATTACCGGAGAGTATTCACGAAGATTTCAAAAGAGAACTTTGTGCA
ATGTACTATCTAGAGAATGTCAAATGTTCATTGGCCATCAACACAGAACATTTGTGCCTGCCATCCTTGACCAGA
GCGAAAAAGATAACAAATTTTGAATTTGTAATGACGGGTGCACGCGCTACTAGAACTAAATATCAGCAAAAGGCT
CATGCCGGTTTGATCATTCTTGCCAAATCCTTCACATTTCAAAATTTTGCTTTGAGAACGACATCTGCTACACCA
GAAACATTTGCTTTAGAGTCCGATCTATTATTGGAGAAGCCTCATTTTGAGTCCATTGCTGATGAACCTCTAGAC
GAGCAAATTTATAGTAAAAGACAAAAGGTAGACCTAAATGAAGGTTATGAAGAAGATAAACTATTGCCATTGGCA
CTACGTCAAGAAAATATTCCAAAGTTGTTGCTGGATTTGAACCCTAACGATCAACCTACCCTATCTGATTACGAC
AACATCCAGTTGCTGTTACGTCTTTACACCATAAAGAACACAACTCCGGCCAAGGATCCTCTTGTGGAAGAAGAA
CTTACTGCTTTGCTTTCTAGAATATTATATCAAAACGGTGACAAAAATTGGTCTATTTTCGCCCGATCATTATGG
GAAAGGTCAATTATTGAGACAACAAAGGCTAAAACTATTGAAAGAGGACTTTTACAAATGCAATCCCTCGTAGAA
GAATTGGATTTGAAGATTAAGAGTAAATTGGTTCCAAGTTCAAGCGAAATCAATGTTGCTAGCAGGCTGTCATAC
ATCCACCAGCTGCCGTTCATCCCTAGGTGGCAATTGGATGCCACCTTAGCTGAAAAATATATGTCGCTGGGTATT
TTGAAGTCCGCTGTGGAAATTTATGAAAGACTTGGTATGGCATGCGAGACTGCATTATGTTATGCCGCTGTTGGA
GATGAAAAAAAGGCAGAAGAAATCCTTCTTCAAAGAATAAATGAAAATGATTCGGATGCCAGGGCTTACTCCATT
TTGGGTGATATAAAACAGGATCCATCCCTATGGGAAAAAAGTTGGGAGATTGGAAAATACGTCAACGCAAAAAAT
TCCCTTGCAAAGTATTACTTTAATCCTCCTCCAAAGTCTGGAGCACAACCAAATTACTCTGCTACCTTGAAACAT
TTAAATGACTCGTTAAGGCAATACCCATTAAGCTTTGAAACGTGGTATTTTTATGGTTGCGTTGGATTACAATGT
GGTAAAATGCAGATAGCTGCGGAGGCATTTACCAGATGTGTTTCATTAGACCCTTATCATGCTCTTTCATGGTCA
AATTTGAGCGCTGCATATACGAAAATGGATAAATTGAAAGAAGCTTATAGTTGTTTGAAAAGAGCCATTTCATGT
GACGCTCAAAAGAACTGGAAAATTTGGGAGAATTACATGCTTGTTGCTGTTAAGTTGAATGAGTGGGAGGATGTC
TTAACTGCATGTAAACAATTAGTCAGTATTAGGAGGGACAAATCTGGCGAAGGTTCCATCGACTTACCAATTATT
GAAAAACTAGTAGAACTATTAGTGACTAGTGAATATCCAGAGGAGCCGCAGCAATTATCTTATTTTCAAAAGAGC
TGTACTGAGTTTATCTGTAACACTTTACCTCAGGTAATTACAACCAGTGCAAGATGTTGGCGACTGGTCGCAAGA
GTAGAACTTTGGAGGAAGAGACCATGGGCCGCCCTAGAATGCCACGAGAAGGCTTATAGGGCCATTTCTCACAAT
CCTGATTTAGAAGTTGAAGAAAAGGTTTGGAATGATACAGTGGATGCTTGTGAAGATTTGGTGGCTGCCTATGAA
TCACTTGGTGAAATGGAAGGTAAATATGGGCCAGGCAGTCTGGTTTGCAAAGACTGGAAATACAAATGTAGATCT
ACTATCAAAGCTCTAATGAGTAAAGGCAAAGGCAGATGGGATGACTCTCCTGGTTGGGATAGGCTAGTGGAAGCA
AGAAGCCAAATATGA

>YLR420W 0.81   Cold
ATGGTACAAGAAATCGATTTAGGTTTGACATGTGATATGCATGTTCATGTAAGAGAGGGTGCGATGTGTGAACTA
GTCACCCCCAAGATTAGAGATGGTGGGGTTTCAATTGCTTACATCATGCCAAATTTACAACCTCCAATTACCACA
CTAGATAGAGTGATTGAATACAAAAAGACACTGCAGAAACTAGCTCCTAAAACTACCTTCTTAATGAGTTTTTAT
CTTTCAAAAGACTTAACTCCAGATTTAATTCATGAAGCTGCCCAACAACATGCCATTCGTGGAGTAAAGTGTTAT
CCAGCGGGAGTAACAACAAATTCGGCTGCTGGGGTGGATCCAAATGACTTCAGCGCATTTTACCCAATTTTCAAG
GCTATGCAAGAAGAGAACCTGGTATTAAATTTGCATGGGGAAAAACCTTCTGTCCATGATGGAGACAAAGAACCT
ATTCATGTATTGAATGCAGAGGAAGCCTTTTTGCCAGCCTTGAAAAAATTGCATAATGATTTCCCAAACCTGAAA
ATAATTCTGGAACACTGCACTAGCGAGTCGGCAATAAAGACAATCGAGGATATAAATAAGAACGTGAAGAAAGCC
ACTGACGTAAAGGTTGCTGCCACATTAACGGCTCATCACTTATTTTTAACAATTGATGATTGGGCCGGAAATCCG
GTAAATTTTTGCAAACCTGTTGCGAAACTTCCAAATGACAAAAAGGCTCTAGTCAAAGCTGCTGTATCAGGGAAA
CCATATTTTTTCTTTGGATCTGATTCAGCACCTCATCCTGTACAAAATAAGGCCAATTACGAGGGTGTTTGCGCA
GGAGTTTACTCACAATCTTTTGCGATCCCTTATATCGCCCAAGTTTTTGAAGAGCAAAATGCCTTGGAGAACTTA
AAGGGCTTTGTTTCCGACTTCGGAATTTCCTTTTATGAGGTTAAAGATAGCGAAGTGGCTTCTTCAGATAAGGCA
ATATTATTCAAAAAAGAACAAGTTATCCCTCAGGTTATCAGCGATGGCAAAGACATAAGCATCATCCCATTTAAA
GCAGGTGATAAACTAAGTTGGTCGGTGAGATGGGAACCTCGTTAA

>YJR132W 0.71   Cold
ATGGATATTACAGAATTGTTACAGTGCTTTGCCTGTACTTTGGACCATAACGCTGCCGTAAGAACTAATGCAGAA
ACACATCTTAAAAATGCAAGTAAAGTACCGGGATTCTTAGGCGCATGCCTGGATATCATTGCTGCTGATGAAGTA
CCAGAAAACATCAAATTATCAGCTTCCTTATATTTCAAGAATAAGATTACATACGGATGGTCTGCTGGTGCAAGA

CAGGGTTCAAATGAATTATTAGATTCACATGTTGATCCGGATGAAAAACCAGTAGTAAAAGATATGTTAATTAAA
ACAATGGTCAGTGTGTCCAAGACCTCCCCGCGTTGTATCAGAGTGCTAAAATCTGCCCTTACTGTAATTATTTCA
GAAGATTACCCTAGCAAAAAATGGGGCAATTTGTTACCAAATTCCTTAGAATTACTCGCAAATGAAGACATCACA
GTAACATATGTCGGACTTCTGTGTCTTGCTGAGATTTTTAGGACCTATAGGTGGAAGAATAATGATGAAAGACAG
GACTTAGAAGAGTTGATTTTAAATTATTTCCCTGCTTTATTAAACTATGGCGCAAATGTCCTTTTCCAAGATGGT
AAATATATGAATAACGAGCAAATTGGCGAATTGGTAAAATTAATCATTAAAATTTATAAGTTCGTTTCATACCAT
GACCTACCATTTACATTACAACGCTCAGAATCGTTTACCCCATGGGCATGTTTTTTTGTCAGCATCATTCAGCAG
CCATTGCCTCAGGAAGTTTTGTCTATATCAGATATTGAGGTCAGAAGTAAAAATCCATGGGTCAAATGTAAGAAA
TGGGCTCTTGCAAACCTTTATAGACTTTTCCAGAGATACGCATCAACGTCATTAACAAGGAAGTTTCAGTACGAT
GAGTTTAAACAGATGTACTGTGAAGAGTTTTTGACTCAGTTTTTGCAAGTCGTCTTCGATCAAATCGAGAAATGG
GGAACTGGACAGTTGTGGTTAAGTGACGAATGCTTATACTACATATTGAACTTTGTTGAGCAGTGTGTTGTGCAG
AAAACAACTTGGAAACTTGTTGGGCCCCACTACAATGTAATACTTCAACATGTCATTTTCCCACTACTAAAGCCC
ACTGCAGAGACTTTGGAGGCCTTTGACAATGACCCTCAAGAATATATCAACCGGAATATGGACTTTTGGGATGTC
GGCTATTCCCCAGATCTTGCCGCACTGGCTTTGCTAACGACATGCGTGACGAAACGCGGTAAAACGACCTTACAA
CCGACCTTAGAATTTATGGTCTCAACCTTACAAAGTGCCGTTGGTGATTACAACAATATTATGTTGGATAATGCC
TTGCAAATTGAGTCCTGTTTGAGAATATTCTCCAGCATTATTGACCGTTTGATAACTAAAGATTCTCCATTTGCC
AGTGAGATGGAAAAGTTTATATTGACATACGTTTTACCTTTTTTTAAGTCTCAGTATGGGTTTCTGCAAAGTCGT
GTTTGCGATATTTGTTCGAAGTTGGGTTCTATGGATTTCAAAGACCCGGTAATAACCTCTACTATCTACGAAGGT
GTTATGAATTGTCTGAATAACTCTTCTAATTCTTTACCTGTAGAATTGACAGCTGCATTGGCATTGCAAACTTTT
ATCAGTGATGACCAGTTTAATATGAAGTTATCCGAACACGTTGTTCCTACTATGCAGAAATTACTAAGTTTGTCT
AATGATTTTGAATCTGATGTCATTTCAGGCGTTATGCAAGATTTTGTGGAGCAGTTTGCTGAGCAATTACAACCT
TTTGGTGTTGAATTAATGAACACTTTGGTCCAACAGTTTTTGAAATTAGCTATTGATCTGCATGAAACATCCAAT
TTGGATCCAGATTCCTTCACGAACGTTGATAGCATACCCGATGAATCTGACAAGCAAATGGCGGCGCTAGGTATT
TTGTCGACTACGATATCTATTCTGTTGTCTTTTGAGAATTCACCTGAAATTTTAAAAAACTTGGAACAGTCATTT
TATCCAGCTGCAGAGTTTATTTTGAAAAACGACATCGAAGATTTCTATCGCGAATGTTGCGAATTTGTAGAGAAC
TCTACATTCCTACTAAGAGATATCACACCCATCAGCTGGAAAATTTTGGAGCTGATTGGAGAATGTAATAGAAAA
CCAGATAGTATGGTATCTTATTATTTAAGTGATTTCATGCTAGCCTTAAATAATATCCTCATTTATGGAAGGAAC
GAATTAAAGAAGAACGAGTTTTACACAAAAATCATATTCGAAATATATCAAAAGGCTGTTACTGCAGAAGATAAT
TCGTTAGACGATCTTAGAGTGGTTTTTGATTTATCCCAAGAGTTGGTTCTTGCATTAGATGACAGTTTACCTCAA
CAATACAGAGAGCGCCTACTGGCGGATGTTGTCGGCTCTATTCTAACGCAAAAGAATGAATTGAAAACTAATGTT
GTATTTAGTGTGACTGCTTTTAATGTCGTAATCTCAAATATGATAACAGAGCCTTTGATCACGCTTCAGTACCTG
AAACAGCAAGGTTGTCTTGAAATCTTCTTTCAAACGTGGATTACAGACTATATTCCGAATTATAAAGGTGCTAT
GATATCAAATTATCAGTTCTTGCGTTATTAAAAATTATACTGAAGTTAGAAAGCAACGATTATTCAGTGTTGAAC
TTAGAAAATTTGGTTCCGCAATTAGGGAGTATTGTTACGCAGTTAGCTTCGAGGCTACCAACGGCGTTAAGGCAA
TTAGCTAACCAACGCAAGGAATTCTCATCATCCGGTTTTGAAGAGGATACTAAGTGGGATGAAAATTTTCTTGAC
GTTGGAGACGATGATGAAAATGATGACGAGGGAGACCTTACTGAGAAATATCTGGAACTGATAAAAAATAGAGCC
GATTCCTTGGACTTCGTAGATGGTTATGACGCAAAGGAAACTTTCGATGACCTAGAAGAGGACCCATTAACGGGG
TCGATCCTGGACACAGTTGACGTATACAAGGTTTTCAAGGAGTCCATTGCGAACTTACAACATGTTGACAGCAAT
AGATATCAGGGAATCTTGAGACATCTGACCCCGGCTGATCAAGAACTATTCATGGGAATTATGAATGCCTAA

>YNL158W 0.75   Cold
ATGGTCCGGCCTCAGAATGTTCACTGGTTTATCGCTACAATTGTATTTTTCATCGGATTTGTACACGCTAATACA
GAATCAATCTTATATAAGGTTCCTCACAACTTTCCCCTCAAAAAACCAAGAGACAGCTCTACTTATGCAAGAGAT
GTAAACTTGATATCTTCCATATCACTATCGGGAGAAGCAATGAGTCAGATCACTATCGAGGCCAATACCACTGAC
TTGGAGTTGCATAATACTACGTATATTGAACTGGCGGACTTACAGCGAGACGAAACATATCAGATTAAAGTGTGT
TGGTCAGCAATACATCCAATTTCCATTAATAATCTCCAAACTATCACAATACCACGTTTCACTGAATTTCAGGGA
ACTAAGTCAGACTACGCGAGAATACTAGTGACTTTCCAGGTCTTGTCTGACTCGTATCCTAGTGAGCATGCCATG
GTTCCAATACAAGTCTCATTAATCACCACTCGGCTAGGTATCCCCGTGGATATTTACCCAACATTAATCGTTATG
GTGCTATTAGTAGCAGGGCTTGTTGTTACCCGGGCACCACACGTATTGAACGATCTTTTGCTGAAATTTTAG

>YGR110W 0.81   Cold
ATGTTCAAGTCAACTTTAAACTCCATAATAAGAAGACCCTTGAAAGGTTTTCAACTTCTTAGAGGGGCTGACTCA
TCGAATACACGGCCACAGTCCCCTAGAGCCTCCGCAAGAGATGTTACAGAGAAACAGATATTAAGAACTCCGTCA
GCACCAACTGCAATACCATTAAGGGAGATTATCTATAGAGTCCCAAGCTTGTTTCCTCGCCCTTTAGAAGATTCT
GTTAAGGACTTCCGAGATTTTATCAAGAACGAGGATGCATTCCAGACTGAACTTTTGAAAACGCTGCCATTCTAC
CCCACTCCTTCGGAATCAAAAACAGCAAGACTTATAAGAACTGTTGTTGATGATGAGGGCAATTACATCAATGAA
TTTTGCATACGCCCTCGAAAAACCTCAGTGCCTGAGGCTGACTTAAAACATCTGGTTTTCATTCATGGGTATGGC
GCCGGATTGGGTTTTTTCATCAAGAATTTTGAGGATATTCCACTATTGGATAACGAGTGGTGCATACATGCCATC
GATTTGCCTGGGTATGGTTTTTCTTCAAGACCTAAGTTTCCGTTCGAGTATCCGAGAGATAATATTCACAGCGTT
CAAGATTGGTTTCACGAAAGAATACACACATGGTTTAGTAAAAGAAATCTTTTGAACCGACCTGAGAAAAACATT
GTTATGGCACATTCTTTAGGGTCTTACTTGATGGCTTTATATTTGCAAAAATATAAGGAATCTCCATCTTTTAAA
AAGCTGATCCTTTGTTCCCCAGCAGGCGTGTCTTATAGAGATTTCAACAATACTGCTTCAGAGGTTGAAAAATGG
AAACCGCCTCCTTGGTGGTATGTCAAGCTTTGGGACAGAAATATCTCGCCTTTTACACTAGTAAGAAATTTTCGC
CAGTTAGGTTCGAAGATTACAAGTGGATGGTCATATCGGCGCTTTAAGCACATTTTGAATGGTGATCCAGAACAG
TCAAAACGGTTTGAAGCCTTGCATAGATACGCATATGCTATTTTCAATAAACGTGGTTCAGGTGAATACTTATTA
AGCTTTGCTTTGAAATGTGGTGGTGAACCAAGACTATCATTGGAGCAGCAGCTATTCGATGGCAAAAAGTCTGAT
ATTTAAAGAATAGTAATTGTGACTGGCTTTGGCTTTATGGCGATGACGATTGGATGGATGTGAATGGTGGACTT

AGAGTATCAAGATTCTTGAAAGAGAAGTTGAAACAAAAAAGTAACGTCATCATTGTTCCTCATTCAGGGCATCAC
TTGTACCTGGATAACTATAAGTTTTTCAATAATATTCTTACGAAAGAAATGCAAAAAATATAA

>YGL078C 0.82 Cold
ATGACAAAGGAAGAAATCGCAGACAAGAAGAGAAAGGTTGTAGATGAGGAGGTAATTGAAAAAAAGAAGAGTAAG
AAACACAAAAAGGATAAGAAAGACAAGAAGGAAAAGAAGGATAAAAAGCATAAAAAGCATAAGAAGGAGAAGAAG
GGTGAGAAGGAAGTGGAAGTACCAGAGAAAGAATCTGAAAAGAAACCTGAACCTACTTCTGCAGTGGCTAGTGAG
TTTTATGTTCAGAGTGAAGCACTAACCAGTCTTCCACAATCTGATATTGACGAATATTTCAAGGAAAATGAAATT
GCTGTTGAGGATTCTCTGGATCTAGCCCTACGTCCGCTATTATCATTTGATTATCTTTCTTTAGACTCTTCCATT
CAAGCAGAAATTTCCAAATTTCCAAAACCAACGCCTATTCAAGCCGTAGCCTGGCCATATTTGTTATCTGGTAAA
GATGTTGTTGGTGTTGCTGAGACCGGTTCTGGTAAAACATTTGCTTTCGGTGTTCCAGCTATCAGCCATTTAATG
AACGATCAAAAGAAAAGGGGCATACAGGTTCTAGTCATTTCCCCAACTAGAGAACTTGCTTCCCAAATTTATGAT
AATCTGATTGTATTAACTGACAAGGTTGGTATGCAGTGTTGTTGTGTTTATGGTGGTGTTCCAAAGGATGAGCAA
AGAATTCAGTTAAAAAAGTCACAAGTGGTGGTTGCTACACCTGGTAGATTATTAGACCTATTGCAGGAAGGATCT
GTTGACTTATCTCAAGTTAATTACTTAGTATTAGACGAGGCAGACAGAATGTTGGAAAAAGGTTTTGAAGAAGAC
ATCAAGAATATTATCAGAGAAACTGATGCGTCCAAAAGACAAACTCTAATGTTTACCGCTACCTGGCCAAAGGAA
GTCCGTGAATTAGCTTCCACGTTCATGAACAACCCAATCAAAGTATCTATTGGTAACACGGACCAACTAACTGCT
AACAAAAGAATTACACAGATTGTTGAAGTGGTGGACCCACGTGGAAAAGAAAGAAAGTTATTGGAACTGTTGAAA
AAGTACCACTCTGGTCCAAAAAAGAATGAAAAGGTCTTGATTTTTGCTCTTTATAAGAAGGAAGCTGCTCGTGTT
GAAAGGAATTTGAAATATAACGGGTATAACGTCGCAGCTATCCATGGTGATTTGTCCCAGCAACAAAGAACGCAA
GCATTGAATGAGTTCAAAAGCGGGAAGTCTAACTTGTTACTGGCCACCGACGTTGCAGCAAGAGGTTTAGATATA
CCAAATGTTAAGACAGTTATTAATTTAACTTTCCCATTGACTGTGGAGGATTATGTCCATAGAATCGGTAGAACT
GGTAGAGCTGGTCAAACAGGCACAGCACACACTCTGTTCACTGAACAAGAAAAGCATTTGGCTGGTGGACTGGTT
AACGTTTTGAATGGTGCCAATCAACCTGTTCCTGAAGATTTGATAAAGTTCGGTACTCACACTAAGAAGAAGGAA
CATAGCGCTTATGGTTCATTCTTTAAGGACGTCGATTTGACTAAGAAACCAAAAAAAAATTACTTTCGATTAG

>YJR001W 0.75 Cold
ATGCCTGAGCAAGAACCATTGAGTCCCAATGGCCGTAAGCGCTCTGAGGTTCACTATATCTCAATCCCATTGAAC
AGAGGTTCCGCATTCTCACCAGATGATTCTGTATCACAGTTTCAGTCTGACGGGTTCATGACACGTAGGCAATCC
ATATTGGACCATCCTGTGGGCTCATTTAAGGGAGTTAATTCTTTAAGTCGATTTGCGACTTCGTTGAGGAGAGCC
AATTCGTTTCGTAATATCGAGCTAAATGCGGATAATGAAAGATCCTTTTTCAAAGAGAGTAACGATGAAACCTAC
GATCCGGATACTTTAGCACCAGCTTTGGACGGTAGAAGATTATCAGTAACTTTAAATAATGCTGGTCGCCCGCGC
ATTACTAATTTGGCTAACAACGATAGAGTTAGTACAGCCAGCATGGCTATTCACGATGATGATTATGGGTCCATC
CAAAATTCAACAATTGGGGATTCTGGGTCAATATTACGCCCTACTGCCTCCTTAACAGAAATGATGAGTGGTGGA
GCGGGGAGAAGGTTTACAAATAATGACATGGATTCAATTGTAGTGAAGAGAGTGGAGGGTGTAGATGGTAAGGTA
GTAACTCTTCTTGCTGGTCAGTCGACCGCTCCACAGACCATATTCAACTCAATCAATGTACTGATTGGTATTGGG
CTCCTGGCGTTACCTCTTGGTTTGAAATACGCCGGCTGGGTTATAGGACTTACTATGCTTGCAATCTTCGCCTTA
GCGACATTTTGTACTGCTGAACTTTTGTCTAGGTGCCTAGATACAGATCCAACTTTGATATCTTACGCGGACTTG
GGATATGCAGCATTTGGTACAAAAGGGCGCGCACTAATTTCTGCTCTTTTCACACTTGACCTACTGGGTAGTGGG
GTGTCCTTGGTGATTCTCTTCGGAGATTCTTTAAATGCTCTATTTCCTCAATACTCCACCACTTTTTTCAAAATA
GTTTCATTTTTTATCGTTACACCCCCGGTTTTTATACCATTGAGTGTTCTATCTAATATCTCGCTTCTGGGAATC
CTTTCAACAACCGGAACTGTGCTAGTCATTTGCTGCTGCGGACTATACAAGTCGTCTTCTCCAGGTTCATTAGTT
AATCCGATGGAAACGAGCATGTGGCCGATAGATCTAAAGCATCTCTGTTTATCTATAGGTTTATTGAGTGCCTGC
TGGGGAGGACATGCGGTTTTTCCCAACTTAAAGACAGATATGAGGCATCCAGATAAATTTAAAGACTGTTTGAAA
ACTACCTATAAGATAACATCGGTTACGGACATAGGTACTGCAGTTATTGGCTTTTTGATGTTTGGAAATCTTGTT
AAAGATGAAATTACTAAGAACGTTTTGCTGACGGAGGGATATCCAAAATTCGTTTATGGTCTGATTTCAGCGCTT
ATGACTATCATTCCTATCGCAAAGACCCCATTGAACGCAAGACCTATCGTTTCTGTATTGGACGTTCTGATGAAT
GTGCAACACATAGATGAAGCGGCCAGCGCCATAAAAAGAAGAGCTGCGAAAGGCCTTCAAGTATTCAATAGGATC
TTTATTAATGTTGTCTTCGTTTTGATAGCTATTAATTTCCCTGAATTTGATAAGATTATCGCATTTCTGGGGGCC
GGTTTGTGTTTTACAATTTGCCTAATTTTGCCGTGTTGGTTCTATTTAAGATTGTGCAAGACAACAATCAAACCA
TGGGAGAGAGTGGCTTGTCACGTAACCATATGTATTAGTGTCGTGTTGTCCACATTAGGTGTTGGTGCTGCAATT
ATTTCATAA

>YKL219W 0.79 Cold
ATGCAAATAGTCAGAAGTTGTAACTCCAATAACAAACCCCTCATACCGTCCTCAAATTGGCATATCGCTATCAGA
ATGAGAGGCGATGGGGTTAAAGATAGGAGTATAGATGTGTTATCCCTCAAACATTTCGAATCCCAGAAAGTTGTT
CTGCCTCAAGACCTTTTCATGGATAACTTCACCTGGATGTTTTATGAGTTCTTCAAGTGCTTCACGTTTCGTACC
TGGTTGCTATTATTACTGCTAATGTGGTTGCCAGGTTTTCTTTCGCAGATAAAGTCCATCAATCGGATTTTTCCG
TTTAAACTTTGTATTTTAGTCTCATGCCTAGTGGGGATATTTTTACCCAATATTTATTCATTCTCTCATAAAAGT
GTGTTAACGAATCAACTTACTCAGTTCTCCAAAGAAATTGTTGAACATGCACCAGGTACTGATACTCATGATTGG
GAAACAGTTGCGGCAAATCTAAATTCTTACTTCTATGAAAATAAAGCTTGGAATACTGAGTACTTCTTTTTCAAT
GCTGCAGAGTGTCAAAAAGCATTCAGAAAAGTTCTTCTCGAACCATTCTCTGTGAAGAAAGATGAATCTTCGAAA
ATAAAATCATTTGGGGATTCTGTCCCCTACATCGAAGAGGCCTTGCAAGTCTATTCCACAGAATTTGACAAAAAG
TGGAAGTTGTTCAATACTGAGAAAGTGTGGAGCCCTGATAACTTGGAACATGTTCAGCTCCCCAAGAAAACTTAT
CGTTATAAGTTTACCTGGGTTCTCAAGAGGATTTTCAATCTCTGGCTTTTTCCAGCATTTATTCTGTTTCTGGCT
TGTATCTATGTGTCATGGGATAAGGGCCATCTATTTCGTATCTTGTGTTGTGGGGGAGGTTTTCTCCTTATGGTA
AGAGTTTTTCAAAATATGAGGCCCTTTTCCATGCACATGGAAGACAAAATGCAGTTCTTGTCAACGATTATAAAT

GAGCAAGAAAGTGGTGCGAATGGATGGGACGAAATTGCAAAGAAAATGAACAGGTACTTGTTTGAGAAAAAAGTC
TGGACTAGTGAAGAGTTTTTCTTTGATGGGATTGACTGTGAATGGTTTTTTAACCACTTCTTTTACCGCCTTCTA
TCTACAAAGAAACCTATGTTTGATAGACCTCTAAACGTGGAACTATGGCCATACATTAAAGAAGCGCAATTGACC
CGCAAACAGGCGCCTCCCGTGTAG

>YIR021W 0.82 Cold
ATGTCTCCGAAGAATATAACAAGGTCGGTGATCCCGGCTATAGATTTGTATTGCCGCAAAGCAAATTTTAAAACT
CTAAAATCATTGTCGATGATCCTAGGTAGTAAAAAAGAATGGTATGACACTAAAAAGGCGCCATTGAGAACCTTT
TTGGTCTCTCGATGTGGCATTTTCGAGCAGTTGAGGGGTCGTCTCGTTGAAGATGGCAAAGTCAACTTGTTTAGT
GTTTTCCTGACAAACGACTCATTTTCTTTTTGCAAGATGACCGTTGATGATAAGTTCAACACTAGCTTGGTCGAT
TGGCAAAAAATACCCTTTGACAGTACATTTGCAACTGACAGAAGACAGAATATCAGTTTACTACCTGTTGATACA
CTGTTTGCAACTGAAAAGATCATATCGATTCTTGGTGTATCTCCTAATATGACGAATCTTGTTTCTATAGAAAGA
GAGCGATCAGACCTGGTGGATTTTAATTGTAAACTGCAATCAAACATCCTAGAACACCTACTATACGCGAAATGC
CAAGGAGTGTACGTAACTTCTACCAATGAAAAAGCTCGTTTGCTTGCAGCTGTCTGCAATCCTGAATTCATTGAC
ACCTTCTGGTGCGAATTGACTCCCATAAGAGTCTCATTAAAGGAAAACCCTTCCATTTCTGTACCTCGAGAATAT
CAGATGTACGATCCGGTGGTACGTGCCACTATAAAGGAGGTTGTCACCAAGCGATTACTGCGATCCGCCTTCGAT
AATGACATCGACCCGCTAATGTGTCTTCATTTGGATAAAGGCTGGAAACTTAAATTCCCCATACTATCCTCGACA
ACGGGCCTAAACTTCTCCCTGAAGGATTGTCTTTCCCTGGACACAGGAAAAGATGCATCTGATATGACAGAGGTG
TTTCTCGCTACTATGGAGTCGAGTAAAGTTCTTCGTACGTATAGCAACCTCGTTGACATTGTGATGAAGGACAAT
GGTAGGTTGGACTCAGGCGTCCTAAAGCAATTCAACGACTACGTTAAGCAAGAAAAGCTCAATCTACAACATTTT
CAGGCCGGTTCCTCAAAGTTTCTCAAAGGCGCAAAGATATAA

>YFL057C 0.76 Cold
ATGGCTAGGCATTTCGGTATGGCCCTCGCCCCATGGGATGTCATGGGAGGTGGAAGATTTCAGAGTAAAAAAGCA
ATGGAGGAACGGAGGAAGAATGGAGAGGGTATTCGTTCTTTCGTTGGCGCCTCTGAACAAACAGATGCAGAAATC
AAGATTAGTGAAGCATTGGCCAAGGTTGCTGAGGAACATGGCACTGAATCTGTTACTGCTATTGCTATTGCCTAT
GTTCGCTCTAAGGCGAAAAATGTTTTTCCATTGGTTGGAGGAAGGAAAATTGAACACCTCAAACAGAACATTGAG
GCTTTGAGCATTAAATTAACACCAGAACAAATAAAGTACTTAGAAAGTATTATTCCTTTTGATGTTGGTTTTCCT
ACTAATTTTATCGGTGATGATCCGGCTGTTACCAAGAAGGCTTCACTTCTCACGGCAATGTCTGCGCAGATTTCC
TTCGATTAA

>YGR017W 0.78 Cold
ATGTCGCATCAAATGGCGCCATGGATACCCATGTTTATTCAATCGTGCAAAAATAACACTGAACCGTTTGTATCA
TTTCAATTTGCTACTGTTGACGAACTAACGAACAAACCACGTTGTAGAACGGTAGTGTTCAGAGATTTTTTATTT
CATGACAAAAGAACTAATGTGTTGACTTTTAACACTGATATGAGGAGCTCAAAGATAACTGAATCATTTATAACT
CCAAATTCAAATAACTCTAGCGACAGCAAAAGATGTGAGACTCCATTTTTCGAAGCATGTTTCTATTTCCCGGAA
ACTTGGGAGCAGTATCGCTTCAGTGGCCAATGCTTTACAATATCTAAACAATTTAAGAAAATACCGGCTGAGATT
GTAACCAAGTATGACATATTTTCACCGCGGTTTTCAGAGACAAATGATGACAGCACTGACGAAGAAATAGATACT
CCAATTAACGACGACGACGACGACGATAAAAATAATGACGCCGACAATAATGACATTAACGAAGATAACAAACTT
ATAGAATCCATAGAGAACGATGAACACCATGAGGATGAAGACGACTACTACCCTCAACCACAAGAATGGGAGGCT
GAATTACTAAGACAATGGTCCTCTCTATCAAGGCATACCAAGTCATTGTATAGGAAGCCAGCCCCGGGCCAGAAA
CTTACCTCTGAAACTAGCAAACAACTGGACAAACTCCATAGAGGAGTTGACGGTGCTAAAGAGGACGCTGGACTG
GAAAACTTTGGTATTGTCTGTCTTTGTGTTGATTCTGTTGATTTTTTGAACTTGAAAGAGGGAAGAGGTGGTGAA
AGATGGATTTTCCAGAAAACCGACGGCAAAGACGAAGATCTGTGGGAGGAACAAGAAGTGTGTCCGTGA

>YPL214C 0.8Cold
ATGGTATTTACTAAGGAAGAAGTTGATTACTCATTATATCTGGTTACAGATTCTACCATGCTTCCACCGGGAACT
ACTTTATGTTCTCAGGTTGAAGCTGGGTTGAAAAATGGTGTAACGCTAGTTCAAATCCGTGAAAAGGATATTGAA
ACAAAAAATTTCGTTGCAGAGGCTTTAGAAGTTCAAAAAATATGTAAGAAGTACAATGTTCCACTTATTATCAAT
GACCGTATAGACGTCGCTATGGCAATTGACGCCGATGGGGTTCATGTGGGCCAGGACGATATGCCAATCCCAATG
GTAAGAAAACTTTTGGGCCCTTCTAAAATTCTTGGATGGAGTGTTGGTAAACCTTCGGAGGTAGAGACATTGGCT
AAATGGGGGCCAGATATGGTAGATTATATTGGTGTTGGTACTCTTTTCCCTACATCAACAAAGAAAAATCCTAAG
AAATCACCCATGGGTCCTCAAGGCGCCATTGCCATTTTGGATGCTTTGGAGGAATTTAAAGCTACATGGTGTAGA
ACCGTTGGTATTGGTGGTCTTCATCCTGACAATATTCAACGTGTGCTTTGTCAATGTGTTGCTTCAAATGGAAAG
AGATCGTTGGATGGTATCTCTCTAGTCAGCGATATTATGGCCGCTCCAGATGCATGTGCAGCAACCAAGAGGTTG
AGAGGCTTGTTAGATGCTACCAGATACCAATTTGTTGAGTGCGAGTTAAATAATACATTTCCGACCACAACTTCA
ATTCAAAACGTTATATCTCAAGTATCAAACAATCGTCCATTAGTGCAACATATCACCAATAAGGTTCATCAAAAT
TTTGGTGCCAATGTCACTCTAGCTTTAGGCTCATCTCCTATCATGTCTGAAATTGAAAGTGAAGTATCTGAACTA
GCAAGAATCCCAAATGCTTCTTTACTGTTAAATACCGGATCAGTGGCACCTATTGAAATGCTAAAAGCCGCAATT
AATGCTTATAATGAAGTAAATAGACCCATCACCTTTGACCCGGTCGGGTACAGCGCCACTGAAACAAGGCTCTGT
CTAAACAACACTTTGCTCACTTACGGCCAATTTGCTTGTATAAAGGGCAATTGCAGTGAAATACTGTCTCTAGCC
AAGTTAAATAACCATAAAATGAAGGGCGTCGACTCTAGCAGCGGCAAAACGAACATCGACACACTTGTGCGCGCT
ACACAAATTGTGGCATTCCAATACAGGACTGTTGCTGTTTGCACGGGTGAGTTTGATTGCGTTGCTGATGGTACT
TTTGGAGGCGAATACAAACTTTCATCAGGAACAGAAGGAATAACCGCTGAAGATCTTCCATGTGTAATAATTGAA
GATGGCCCAATTCCTATCATGGGTGACATCACTGCAAGTGGATGTTCACTTGGTTCTACAATTGCTTCCTTTATC
GGTGGGTTAGATTCCACTGGAAAACTGTTTGATGCTGTGGTTGGTGCTGTCCTATTATACAAATCAGCTGGTAAG

TTGGCTTCCACCCGTTGTCAAGGAAGTGGTTCATTCCATGTCGAATTGATTGACGCATTATATCAATTATTCCAT
GAAAATAAACCAGAAAAGTGGTCCGCTTCTTTGAAGAAATTCAAATAA

>YNR006W 0.75   Cold
ATGTCCGTTAGCACGCCAAGTGAGTTAGACGCATTAATAGAACAAGCCACTAGTGAGAGCATTCCCAACGGTGAC
CTTGATCTCCCCATAGCGTTGGAAATTTCAGATGTGTTAAGATCTAGAAGGGTAAATCCCAAAGATTCTATGCGT
TGTATAAAGAAAAGGATTCTCAATACGGCCGATAATCCAAATACCCAATTGTCTTCCTGGAAGCTTACCAATATA
TGTGTAAAAAATGGTGGAACGCCTTTTATTAAGGAAATTTGTTCCAGAGAGTTCATGGATACCATGGAACATGTC
ATTTTAAGAGAAGATAGCAACGAAGAACTTTCTGAGCTGGTAAAGACCATATTATATGAACTATACGTAGCCTTC
AAAAATGATTCGCAACTTAACTATGTGGCAAAAGTTTATGATAAATTAATCTCTCGTGGCATTAAATTCCCTGAA
AAGCTAACACTTTCCAATTCTCCGACTGCAATGTTTGACTCGAAGACGCCCGCAGATTGGATTGATTCTGATGCT
TGTATGATTTGCTCCAAAAAGTTTTCTCTACTAAATAGGAAACATCACTGTCGTTCCTGTGGTGGAGTTTTCTGC
CAAGAACACTCATCAAATAGTATACCCTTACCTGATTTGGGTATATATGAGCCAGTCAGAGTATGTGATAGCTGC
TTTGAAGATTACGATTTGAAGAGACATGACGACAGTAAAAAATCTAAAAAACATCGCCATAAAAGAAAGAAGGAC
AGGGACTACTCAACTCCTGAGGATGAAGAAGAGCTGATAAGGAAAGCAATAGAACTCTCCTTGAAAGAATCTAGA
AATAGCGCTAGTAGCGAGCCTATTGTCCCCGTTGTAGAATCAAAGAATGAGGTGAAGAGACAAGAAATAGAAGAA
GAAGAAGATCCTGATTTAAAAGCTGCTATTCAGGAAAGCTTGAGAGAAGCTGAAGAAGCGAAGCTACGCAGTGAG
CGTCAAAAAGCCTCTAGACAGATGCAACCCCAACAGCCATCACCTCAACCTCAACCTATCCATTCCGTTGATTTA
TCTGATGAGGAGAAAGACAGCATATACATGTTTGCTTCACTTGTCGAAAAAATGAAATCGAGGCCCTTGAACGAA
ATTTTGGAAGACTCTAAATTACAAAACTTGGCTCAAAGAGTATTTGCTTCCAAAGCAAGGTTGAATTATGCTTTA
AATGATAAAGCTCAGAAATACAATACTTTGATAGAAATGAACGGCAAGATTTCGGAAATTATGAACATATACGAT
AGATTATTGGAGCAACAGCTACAAAGCATCAATTTGTCTCAGCAATACACACTTCCACAAGTCCCTTCTGATCCG
TACAATTACCTAACAGAAAATGTGCAAAATCCAGCAGAAAGCTATCAAACACCTCCCCTCCAGCAACTATCATCT
CATCAATACAAACCACAACAGGATGTCTCACGCCAACAATCGGTAAAAGCAAATTCATCACCTACCACTAATATT
GATCACTTGAAAACCATCGATGTGACACCACACGCACAGCAAAAGCCTCAATCGCACGTTGAACTTGCACCTTCG
GACCCACCTTATCCAAAAGAAGAGGCCGAAGATGAAGGAACACAGGCAGTGCAAGATGAGGAATCCTCCACACAA
GAATCCCGCGAAAGGCCGTATCCAGTAGAAACAGAGAATGGTGAAACTTCAATAAATAAACGGCCTCAAGGCATT
ACACGTTACGATTTTCCAACTGTTCCTGCACGCAAATTCGTCCAACCGGAGTCAACTGTACCACTTCCTGCAAGT
TCCTCTGAAATTCCGATCAAAGAGGAAAGGCCGCCTAGTCCTCAAGAGGAACTGCTAATAGAGCTTTAA

>YOR322C 0.78   Cold
ATGGCATTTTCACGTCTTACATCTACTCATCAGTCCAATCATAACGGCTACAGTAATAGCAACAAAAAAGGACAA
AGTCTTCCACTAACCCTTTCTATTGATGTAGAATCACCGCCATGTGTTCTTTATGGGTCTGCTATGGAATCCTCA
GGTGCTGTGCTAAGTGGACTTTTTACTGTGACGGTTGTCGATCCCTACAGTAGTGCTGAAGATAAATCTTTAAAG
AATACAGAATCTAATGTGTCGACTACTTCTAAAAGCCTAAAAAGAAAGAGTACATTTGGTTCTGCACTTTCATCT
AGATTGTCCAGCTTGTCAGCGTCGACGTCAAATATCTCTCCTTCAACATCTTCTACATCCATATCGCATTCTCCC
ACGCCTGCTAATTTAAGAATAATGGCTGGTTATACCAAGATCACCATTACGTCGGTAACACTAAGTTTGGTACAG
AAGATCCATTTCCATAAACCCTTTGTGCCTAATATTTCCTCAATGCAAACCTGTATGAACTGTAAAACAAAGATT
ACAAACATGAAAAGCTGGGAAATTCAGAGTAATACCCAGGATCTTTCTGTTGGAAGCCACTCTTACCCATTTTCC
TATCTTATACCGGGGTCTGTCCCATGTTCTTCATCGCTAGGTGCTACGGCTGAAACCCAGGTCAAATACGAACTT
ATCGCTGTTGTGACGTATATAGATCCTCATAGAAATTCCTTTTCTTCCGGTCATTCTACTCCCAGGAAAGAAGGA
AGCTCTTCTAAGAAGCGGTTGTTGCAACTAGCGATGCCCATCGCCGTGACTCGAAGTATACCGCGCGGGCCTGAT
AAGAATTCTCTAAGAGTATTTCCTCCTACAGAGTTAACTGCTGCTGCTGTTCTTCCTAATGTTGTATATCCTAAA
TCTACTTTTCCTTTGGAGATGAAATTAGACGGTGTTTCTTCGGGGGATAGGAGATGGCGAATGCGTAAATTGAGT
TGGAGAATTGAAGAAACTACAAGAGTTAAAGCACATGCTTGTCCAGTGCACAAGCATGAATTGAGGCAGTTAGAA
GAGCAGGTTAAAATAAAAGAGTCAGAAAAAAGTAAAAAGCCACGCAGTCATATTAAGCGTTATGGTGAACTAGGC
CCACAAATCCGGGTTGCAGTAAACTCTTTAGAAAATATGCCGTCGCAAAGGCTTCCAGGCGAGCCCGGTCGTGAA
CAAGCACCTAATTCTTCGGGTCCAGCGTCAACTGGTAATGTTGGACTTGATGATGAAAATCCTGTTAATGAAGAT
GAGGAAGACCAACCCGGTAGCGAATTCATCCATCCCAGTGACGACGCTTTGCGTCAGGAGTTACTAATGCAGCAA
CAACGCGCAAGACAACAACAACTTCAACAAGAGCTAAAAAACAACAGTAGTTTATTTACGGAAGAGGTTCGGATA
ATATCTAAGGGAGAAATGAAGAGTGGATGGAAAACCGATTTTGACAATAATGGAAAGATTGAGCTAGTTACAGAA
ATAGATTGTATGGGACTTAATTCAGGCGTCTCTAATCCGGTAATGCATGCATCGACACTACAAACTCCTTCCACG
GGCAATAAGAAGCCCAGCATCAATGTGGCTTGCGATATACAGGATCCAAATTTAGGTTTGTATGTAAGCCACATC
TTGGCAGTGGAAATAGTTGTTGCAGAAGAAACATTGCAATATGCCAATGGGCAACCTATACGGAAACCAAACTCC
AAAAACAAGAAAGAAACTAATAACAATACGATGAATGTTCATAATCCTGATCAACGCTTAGCTGAATTATCTCCA
ATTTTTGCAAATAGAAATACACCGAAAGTACGGCGCATGGGGCCTGAAGATATAACACCGGTGAATAGTAATAAG
TCCAACCATAGTACTAATAAGGAGAAGGCATCTAACGGTGCTAGTAACTCTAATATAGTGAGTGTTCCCACTGGT
GCAGCACGTGTTTTAAGAATGCAATTCAGATTAACAGTCACTGAAAGATCTGGACTTGGAATCTCCTGGGACGAA
GAAGTTCCTCCCATTTACCAAGATGTCGAGTTGCTCTCGCCACCATGTTACGAGCTCTCTATAAATAATGGAATC
AAAAATAAACTTTATTCAACAATGAGTACTCCTGTTAGATCAGAGGATGATTTTGTGGGCGGCAGCGATGAAGAT
ATTGGGAACTATGAGAGCCAAGGGCTCGAACCTGGTCCTAACGTACAGGAAGTAACGATCACACAAAATAAATTA
ACGATACCACCAACCGCACATCACTACCAGCCTGCTTCCTCTTCGCAAAGATCCCTTACCACAGTACAGTCACCA
CCACTGGAAAGTGTTGTTAGTGTCCAGGGTAGTGTACCTTTTCGTGGACATGTGTTGACACCACATAGCACAAGA
GATATCAGAATACAAAACTTTTCCGATTTTCTAGATTCCAATAGAATAACCCAGTAG

>YLL008W 0.73   Cold

ATGGTGGTAGGAACTAAAAAAATACTCTAATTTGGACTTTGTCCCTACAATCAGTGACAGTGAAGACGATGTTCCA
ATTCTAGATTCTTCTGATGACGAAAAAGTCGAGGCTAAGAAGACTACGAAGAAGCGGAAGGGTAAGAATAACAAG
AAAAAGGTTAGTGAGGGGGATAACCTCGATGAGGATGTTCATGAGGACTTGGACGCGGGGTTTAAGTTTGATTTG
GACGCCGATGATACCACTTCGAACTTCCAAGGCTGGAACTTTCTAGCAGAGGGCGAGTCCAATAAGGACGATGCC
GAAGCTTTTGTGAAGAAGGACGTTGACTTGGATAAGATTATTAGAAGAAAAGGTGGGCTGGTGAAAATGGCCCAT
ATTGATAGTAAACAAGAAGAAGAAACCGAGAAAGAAAAAGTAGAAAAAGAAAACGATAGCGACGATGAGGAATTA
GCAATGGACGGGTTCGGTATGGGAGCTCCCATGAACAATGGAGACGAAAATCAGTCAGAAGAAGAAGAAGAAGAG
GAGGAAAAGGAAGAGGAAGAGGAGGAAGAGGAGGAACAAGAAGAGATGACGTTAGAAAAAGGCGGCAAAGATGAC
GAAATAGATGAAGAAGACGATTCTGAAGAGGCAAAAGCCGATTTCTATGCGCCTGAAACTGAGGGAGATGAAGCT
AAAAAGCAAATGTACGAAAATTTCAACAGTTTGTCTTTATCTCGTCCGGTTCTTAAGGGCCTTGCAAGTTTGGGT
TACGTCAAGCCTTCCCCTATTCAAAGCGCCACAATCCCCATTGCCTTATTGGGTAAAGACATCATTGCCGGTGCT
GTGACTGGTTCCGGTAAGACTGCTGCGTTTATGATTCCCATAATCGAGCGTTTGTTGTATAAACCAGCCAAAATC
GCTTCCACCAGAGTTATTGTTCTATTGCCCACTCGTGAGTTAGCTATCCAAGTCGCTGACGTTGGTAAACAAATT
GCACGTTTCGTCTCCGGTATAACCTTTGGTCTGGCCGTTGGTGGTTTGAACTTGAGACAACAAGAACAAATGTTG
AAATCTCGTCCGGACATCGTCATTGCTACCCCAGGTAGATTCATTGATCATATCAGGAACTCAGCAAGTTTTAAT
GTGGACTCAGTAGAGATTCTGGTTATGGATGAAGCCGATAGAATGTTAGAAGAAGGTTTTCAAGATGAACTGAAC
GAAATTATGGGCCTATTACCAAGCAATAGACAGAACCTATTGTTTTCTGCTACAATGAACTCCAAAATTAAAAGT
TTAGTTAGTCTTTCTCTAAAAAAACCAGTAAGGATTATGATTGATCCTCCAAAGAAAGCTGCTACTAAGTTGACA
CAAGAATTCGTTCGTATTCGTAAAAGAGACCATTTGAAGCCTGCCTTGTTATTTAATTTGATTAGGAAATTGGAT
CCAACGGGTCAAAAGAGGATTGTCGTTTTTGTGGCTAGAAAAGAAACTGCTCATAGGTTAAGGATTATCATGGGT
CTTTTAGGTATGAGTGTGGGTGAATTACACGGTTCTTTAACCCAAGAACAGCGTTTAGATTCCGTTAATAAATTC
AAAAATTTGGAAGTTCCTGTACTTATCTGTACGGATTTGGCCTCCAGAGGTCTTGATATCCCCAAGATTGAGGTT
GTTATCAACTACGATATGCCCAAGAGTTATGAGATCTACCTGCATAGAGTTGGTCGTACCGCCAGAGCTGGTAGG
GAAGGTCGTTCCGTCACCTTCGTCGGTGAATCATCTCAAGATAGAAGTATTGTACGTGCTGCTATAAAGAGTGTA
GAAGAAAATAAGTCCCTAACTCAAGGTAAAGCACTTGGTAGAAACGTAGACTGGGTTCAAATCGAAGAAACAAAC
AAACTTGTTGAATCCATGAACGATACGATTGAAGATATTCTGGTGGAAGAAAAGGAGGAGAAGGAAATATTAAGG
GCTGAAATGCAATTAAGAAAGGGTGAAAATATGTTGAAGCATAAAAAGGAAATCCAGGCAAGACCAAGAAGGACA
TGGTTCCAAAGCGAATCAGATAAGAAAAATTCCAAAGTATTAGGTGCTTTATCAAGGAACAAGAAAGTCACTAAC
AGCAAAAAGAGAAAGCGTGAAGAAGCTAAGGCAGATGGCAATGGTGCACGTTCTTATAGAAAAACCAAAACCGAC
CGTATTGCAGATCAAGAAAGAACTTTTAAAAAGCAGAAGAGTACAAATTCAAATAAGAAGAAGGGCTTCAAAAGC
CGTAGGTAA

>YJR131W 0.79 Cold
ATGAAGAACTCTGTCGGTATTTCAATTGCAACCATTGTTGCTATCATAGCAGCTATATACTATGTGCCATGGTAC
GAACACTTTGAGAGAAAGTCACCGGGGGCCGGAGAAATGAGAGATCGGATTGAAAGCATGTTCTTGGAATCGTGG
AGAGACTATTCCAAGCATGGCTGGGGATACGATGTGTATGGACCTATTGAGCACACTTCCCATAATATGCCTCGT
GGCAACCAGCCGTTAGGCTGGATTATCGTAGATTCAGTGGATACCTTGATGTTAATGTATAACTCCTCCACACTA
TACAAAAGTGAGTTCGAGGCAGAAATTCAGAGATCGGAGCATTGGATAAACGATGTTTTGGATTTTGATATTGAT
GCCGAAGTGAATGTTTTTGAAACTACTATTAGAATGCTAGGTGGTTTATTATCCGCATATCATCTATCTGATGTT
TTAGAAGTAGGTAATAAGACTGTCTACTTGAACAAAGCAATAGATTTGGGGGATAGGCTTGCTTTGGCGTTCTTA
TCCACTCAGACCGGAATTCCATACTCAAGTATAAACCTTCATAGTGGCCAAGCGGTTAAGAACCATGCAGATGGG
GGGGCATCTTCTACCGCAGAATTCACTACGCTACAAATGGAATTCAAATATCTGGCGTATTTGACAGGAAATCGT
ACTTATTGGGAGCTGGTGGAGCGTGTTTACGAGCCATTATACAAAAATAACGATCTTCTAAATACCTACGATGGA
TTGGTTCCAATTTATACATTTCCAGATACTGGGAAGTTTGGTGCTTCGACTATCCGGTTCGGATCAAGAGGTGAT
TCTTTTTATGAGTATTTACTAAAACAATATTTATTGACGCACGAAACACTTTATTATGATCTGTACAGAAAATCC
ATGGAAGGTATGAAAAAGCATTTATTAGCACAATCCAAACCCTCTTCTCTGTGGTACATTGGGGAAAGAGAACAA
GGTCTACATGGACAACTTTCTCCTAAGATGGACCACCTCGTGTGCTTTATGGGGGGATTGTTAGCATCAGGCTCT
ACTGAGGGCCTTTCTATTCATGAAGCCCGAAGACGTCCGTTTTTCTCTCTTTCCCTTGAAAGAAAAAGTGACTGG
GATTTGGCTAAAGGGATAACTGACACATGTTATCAAATGTACAAGCAGTCTTCCTCGGGGCTTGCGCCTGAAATC
GTTGTCTTCAATGATGGAAACATAAAACAGGATGGTTGGTGGCGGTCGTCTGTGGGTGATTTTTTTGTTAAACCA
CTCGATAGGCACAACCTACAAAGACCAGAAACGGTGGAATCGATTATGTTCATGTATCATTTATCTCATGATCAC
AAATATCGTGAATGGGGGGCGGAAATCGCAACTAGCTTCTTTGAAAATACCTGTGTTGATTGTAATGACCCAAAA
TTAAGGCGGTTCACCAGTTTAAGTGATTGTATCACGTTACCTACAAAGAAATCTAACAATATGGAAAGTTTCTGG
TTGGCAGAGACTTTAAAGTATTTATATATATTGTTTTTAGACGAGTTTGATTTGACCAAAGTTGTTTTCAACACA
GAAGCTCATCCTTTTCCAGTATTAGACGAAGAAATATTAAAATCGCAGTCTCTGACCACAGGTTGGTCGTTGTAG

>YNL157W 0.82 Cold
ATGTCGAATGAAAACTTATCTCCCAATAGTAGTAACCCAGATTTAACTAAACTGAATAACGGCGAAAGCGGAACT
ATTGATACCAGCAAATTCTCGCCCAACGAAATGAAGCTATACAAAATGTACGGAAAGCTGCCATCAAAGAAAGAT
ATTTTTAAGCATACCATGCAGAAAAGAAAATATTTCGACAGTGGTGATTACGCTTTGCAGAAAGCTGGCATTCAA
AACAATGATCCAATAAACTATGGAAAGAATAACCTGCCATTAACAAATCCCAGCAAACTACGAGAAGATATAATA
AAAAGAAGGATAAGCACTTGTCCGTCAACTGCCTCAACCGCTGGTGTAGTAGATAATGCGACGTTGATACAAAAG
GAGGGAAGTATATCGAGTGGACCACCATCGTCTAATAACGGTACTATTGGAGGTGGTAGTACAAGTTCAACGCCA
GTGGGGAACCACAGCTCTTCGTCGTCCAGTTTGTATACTGAATCACCCATTAGATAA

>YOL159C 0.79 Cold

ATGGTTCCCTTATTTGGTTTATTTTGCATTTTTTCTCAATTGTACTCATTATGCTCCGCATATGTTGATATCACC
AGCGGTTACCAAGTATTTTTCAACTTGCCAACTAATATGACAAACAATCAAATATGCTGGCTATTCCAAGCATCT
TACTATGATATTTATTCTGATAAAAGTGGTAGAACACTTAGAACGGGACGGTTTGAACCCGGCGATCAGCAATCT
TTGATTTATAGGGATACCTTAGTTGAGTTGGAGGCTATTACAGATTCTTATGAGTACAGTAACCTAGATCTATCG
ACTTATAACGGACCTGAGCCATATAATTCTGAGACTGATTATTGTACAGATATAATGGATTTGGTAATGCGTGTA
TATGATGAAGAAGGACATTATGTTCATCCAGTTGCGAATAACTCAACAAATGCTTGCGCTCATCCAACTCCTCCT
ACGTTAAACAATCTATTGATAAGTAATTATTCTGACGGAAGAAATTATAAAGAGTCTTCTATATAA

>YGL256W 0.79 Cold
ATGTCTTCCGTTACTGGGTTTTACATTCCACCAATCTCTTTCTTTGGTGAAGGTGCTTTAGAAGAAACCGCTGAT
TACATCAAAAACAAGGATTACAAAAAGGCTTTGATCGTTACTGATCCTGGTATTGCAGCTATTGGTCTCTCCGGT
AGAGTCCAAAAGATGTTGGAAGAACGTGACTTAAACGTTGCTATCTATGACAAAACTCAACCAAACCCAAATATT
GCCAATGTCACAGCTGGTTTGAAGGTTTTGAAGGAACAAAACTCTGAAATTGTTGTTTCCATTGGTGGTGGTTCT
GCTCACGACAATGCTAAGGCCATTGCTTTATTGGCTACTAACGGTGGGGAAATCGGAGACTATGAAGGTGTCAAT
CAATCTAAGAAGGCTGCTTTACCACTATTTGCCATCAACACTACTGCTGGTACTGCTTCCGAAATGACCAGATTC
ACTATTATCTCTAATGAAGAAAAGAAAATCAAGATGGCTATCATTGACAACAACGTCACTCCAGCTGTTGCTGTC
AACGATCCATCTACCATGTTTGGTTTGCCACCTGCTTTGACTGCTGCTACTGGTCTAGATGCTTTGACTCACTGT
ATCGAAGCTTATGTTTCCACCGCCTCTAACCCAATCACCGATGCCTGTGCTTTGAAGGGTATTGATTTGATCAAT
GAAAGCTTAGTCGCTGCATACAAAGACGGTAAAGACAAGAAGGCCAGAACTGACATGTGTTACGCTGAATACTTG
GCAGGTATGGCTTTCAACAATGCTTCTCTAGGTTATGTTCATGCCCTTGCTCATCAACTTGGTGGTTTCTACCAC
TTGCCTCATGGTGTTTGTAACGCTGTCTTGTTGCCTCATGTTCAAGAGGCCAACATGCAATGTCCAAAGGCCAAG
AAGAGATTAGGTGAAATTGCTTTGCATTTCGGTGCTTCTCAAGAAGATCCAGAAGAAACCATCAAGGCTTTGCAC
GTTTTAAACAGAACCATGAACATTCCAAGAAACTTGAAAGAATTAGGTGTTAAAACCGAAGATTTTGAAATTTTG
GCTGAACACGCCATGCATGATGCCTGCCATTTGACTAACCCAGTTCAATTCACCAAAGAACAAGTGGTTGCCATT
ATCAAGAAAGCCTATGAATATTAA

>YLL006W 0.76 Cold
ATGACTGATAGTGAGAATGAATCCACCGAAACGGATTCGTTAATGACGTTTGACGATTATATAAGCAAAGAGCTA
CCTGAACATTTACAGAGACTAATCATGGAGAATTTGAAGGGTTCTACTACTAATGACTTAAAGCAAACTTCAAAC
AACTCAGAGTTTAATGTCAGTAAAAACGGGAGCTTCAAAGGTCTCGATGATGCAATTCAAGCTTTGCAAATGCAA
AGCGTGTTGCATCCTTCTTCGTTAGGATCGTTAGCAACGTCCTCCAAATTTTCTGGATGGTCGTTTGCTCAAGGG
TTTTTTGTAGGACAGCTAAGCATAGTGTTGTTGTTCATCTTTTTCCTAAAGTTCTTTATATTCAGTGATGAGCCA
TCTAAAAGTAAGAATCCGAAACCTGCAGCCTCCCGTCACAGATCAAAATTTAAAGAATATCCCTTTATATCTCGC
GAATTCCTGACTTCTCTTGTTAGGAAGGGTGCTAAACAACACTACGAGCTCAATGAAGAGGCAGAAAATGAACAT
CTTCAAGAACTAGCTCTTATTTTAGAGAAAACCTATTATAATGTCGACGTGCACCCTGCAGAGTCATTGGACTGG
TTCAACGTTTTAGTTGCCCAAATAATACAGCAATTCCGCAGTGAGGCTTGGCACAGGGACAATATCCTTCATTCC
TTGAATGATTTTATTGGAAGAAAATCACCCGATCTGCCTGAATATTTGGATACCATAAAAATAACTGAACTGGAT
ACAGGTGATGATTTCCCCATTTTCTCGAATTGCAGAATACAATATTCGCCAAATTCAGGAAATAAAAAGCTAGAG
GCTAAAATTGATATAGATTTAAATGACCACTTAACTTTAGGAGTAGAAACAAAACTATTACTTAACTATCCAAAG
CCTGGTATTGCCGCACTCCCCATAAATCTAGTAGTGTCAATTGTGAGGTTTCAGGCGTGTTTGACCGTATCTTTA
ACTAATGCAGAGGAGTTTGCTTCTACTTCGAACGGTAGCAGTAGTGAAAACGGTATGGAGGGCAATTCAGGATAC
TTTTTGATGTTTTCTTTTTCTCCTGAATATAGAATGGAATTTGAAATCAAGTCGCTAATTGGCTCACGGTCTAAA
CTTGAAAATATTCCCAAGATCGGCAGTGTCATTGAATACCAAATAAAAAAATGGTTCGTTGAACGATGCGTTGAA
CCAAGATTCCAATTTGTCAGGTTACCAAGTATGTGGCCACGTAGTAAAAATACGAGAGAAGAAAAGCCTACAGAG
TTATAA

>YKR056W 0.78 Cold
ATGTACGAACAGTTTGAATTTTCTTTTTTTTTCTTCGAAAATTCAGACAATAAGGTTAAATATAAAGCTCATCTC
ATCTCATCGATAAAACGCTGGAGTATTATCACATGCATGCGTTGCTTTTGGACCGTACAGAAGTCTATATTTAAA
GCTAGGTTTTTCGCTTGCAGAAACTTTGTCAAGAAGCATAATTATAAACTAATCAGCACCATGACTGGAAGTACT
GAAATGGTACCACCAACAATGAAACATACCGTTGACAACAAAAGGCTTTCGTCTCCTTTGACAGATTCTGGTAAC
CGCCGGACTAAGAAGCCAAAGTTGAGAAAGTACAAGGCCAAAAAGGTTGAAACAACTTCTCCGATGGGTGTCCTA
GAATTTGAAGTGAACGATTTGTTAAAATCTCAAAATTTGTCCAGGGAGCAGGTTCTGAACGATGTTACTTCAATT
CTAAATGATAAGTCCTCAACAGATGGACCTATCGTCTTACAATATCACCGAGAGGTAAAAAATGTCAAGGTCTTA
GAAATTACTTCCAATGGCAACGGGTTGGCTTTGATCGATAATCCTGTTGAAACAGAAAAGAAGCAAGTTGTTATC
ATACCGTTTGGCCTGCCCGGTGATGTAGTTAATATCAAAGTCTTTAAGACCCACCCTTACTATGTCGAGAGTGAT
TTATTAGACGTAGTGGAAAAATCTCCAATGAGAAGAGATGATTTAATTAGGGATAAATATTTCGGGAAATCTTCA
GGAAGTCAATTAGAGTTCTTAACTTACGATGACCAACTAGAATTGAAAAGAAAAACAATTATGAATGCCTACAAG
TTCTTCGCACCAAGGTTAGTTGCTGAAAAACTTTTACCCCCATTTGACACCACCGTAGCTTCCCCTTTACAATTT
GGCTACAGGACCAAAATTACGCCTCATTTCGATATGCCAAAAAGAAAACAAAAGGAACTATCAGTAAGGCCTCCT
TTAGGATTTGGTCAAAAGGGTAGACCTCAATGGAGAAAAGATACTTTGGATATCGGCGGACATGGTTCGATATTA
GATATCGATGAATGTGTGCTTGCAACTGAAGTTCTCAACAAAGGATTGACTAATGAAAGAAGAAAGTTTGAGCAA
GAGTTTAAAAACTACAAAAAAGGCGCGACTATTTTACTGAGAGAGAATACCACTATTTTAGACCCTTCCAAACCA
ACTTTAGAACAGTTAACCGAAGAAGCCTCTAGGGATGAAAATGGTGATATAAGTTATGTCGAAGTCGAAGACAAA
AAGAACAATGTCAGGCTGGCTAAAACTTGCGTTACCAATCCTAGACAAATTGTCACTGAATATGTTGATGGATAT
ACTTTTAATTTTAGTGCGGGTGAGTTTTTTCAAAATAATAACTCTATCCTGCCAATAGTGACCAAGTATGTCCGT
GATAACTTGCAAGCTCCCGCTAAAGGTGATGATAATAAAACAAAGTTTCTAGTAGATGCTTATTGTGGATCAGGT

CTTTTCAGTATATGCAGCTCCAAGGGCGTAGATAAAGTGATTGGTGTAGAAATTTCCGCTGACAGTGTCTCTTTT
GCAGAAAAAAATGCAAAGGCAAATGGTGTTGAAAACTGTAGATTCATCGTTGGAAAGGCTGAGAAACTCTTTGAG
TCTATTGATACTCCAAGTGAAAACACTTCCGTTATCTTGGATCCACCACGCAAGGGCTGTGACGAATTATTCCTA
AAGCAATTAGCCGCATATAATCCAGCCAAGATTATTTACATATCGTGTAATGTCCATTCCCAGGCACGTGATGTC
GAGTACTTCCTCAAAGAAACAGAAAACGGTTCCGCCCACCAGATTGAAAGCATAAGAGGATTTGATTTCTTTCCA
CAAACGCACCACGTTGAGAGTGTGTGTATAATGAAGAGAATCTAA

>YBL019W 0.8 Cold
ATGTCATCAAGCGAAAACACGTTACTGGATGGAAAGTCAGAGAACACAATACGATTTTTAACTTTCAATGTCAAT
GGTATAAGAACCTTTTTTCATTATCAACCATTTTCTCAAATGAATCAATCCCTTAGATCTGTTTTCGACTTTTTT
CGAGCAGACATAATAACATTCCAAGAGCTCAAGACGGAAAAATTGTCTATCTCTAAGTGGGGGAGAGTTGATGGT
TTTTATTCTTTTATTTCTATCCCTCAAACCAGAAAGGGATATTCTGGCGTTGGCTGCTGGATTAGAATTCCGGAA
AAGAACCACCCACTATACCATGCATTACAAGTCGTTAAGGCAGAAGAAGGTATAACGGGTTACTTGACAATAAAA
AATGGTAAGCATTCAGCAATCTCCTATAGAAACGACGTAAATCAAGGAATTGGTGGTTACGATTCTTTAGATCCC
GATTTAGATGAGAAAAGTGCACTGGAACTAGATTCAGAAGGCAGATGTGTTATGGTTGAACTGGCATGTGGAATA
GTTATTATCAGTGTATATTGTCCCGCAAATTCGAACTCATCGGAGGAGGGTGAGATGTTTAGATTAAGGTTCTTG
AAAGTTTTATTAAGAAGAGTTCGGAATTTGGACAAAATTGGGAAGAAGATTGTGCTAATGGGCGACGTAAATGTT
TGCCGGGATCTTATAGACAGTGCCGATACATTAGAACAATTCTCAATTCCAATAACAGATCCCATGGGTGGAACA
AAGTTAGAAGCACAATATAGGGATAAAGCAATCCAATTTATTATCAATCCGGACACGCCACATCGGAGGATATTT
AATCAAATATTGGCTGATTCACTTTTACCAGACGCGAGTAAAAGGGGGATACTGATAGACACTACGAGGCTAATT
CAAACAAGAAATCGACTTAAAATGTATACAGTCTGGAATATGTTAAAAAATTTAAGACCTTCGAATTATGGCTCA
CGGATAGATTTTATCCTAGTGTCCTTAAAGCTTGAACGATGCATAAAAGCAGCTGACATTCTTCCGGATATATTG
GGCTCTGACCATTGTCCTGTGTATTCTGATTTAGATATACTGGACGACAGAATTGAACCTGGTACGACACAAGTT
CCCATACCAAAATTCGAAGCAAGGTACAAATATAATTTAAGAAACCATAATGTTTTAGAGATGTTTGCCAAAAAG
GATACGAATAAAGAATCTAATAAACAAAAATATTGTGTATCAAAAGTCATGAATACCAAAAAAAACAGCAACATC
AAAAACAAATCGCTCGACTCATTTTTCCAGAAGGTAAATGGAGAAAAAGATGACAGGATTAAAGAATCCTCTGAA
ATTCCACAGCAAGCTAAAAAAAGAATCTCCACGCCAAAGTTGAATTTCAAGGATGTCTTTGGAAAGCCTCCCCTG
TGCAGGCATGGGGAGGAATCCATGCTGAAAACATCGAAAACTTCGGCCAATCCAGGTAGAAAGTTCTGGATTTGC
AAGAGATCTCGGGGTGATTCAAATAATACAGAATCATCTTGTGGGTTTTTTCAGTGGGTTTAA

>YDR358W 0.81 Cold
ATGCCACAAAGAATTGAGCTTACCTCCGAACCAGTCAGAAAACCGCGGTCTACTGAGAGCTCACTACTCAGAAAG
ATACAAAGAGCGTGTAGATCCACATTGCCTGAACCAGATTTGGGCTTGAACTTAGATGTAGCTGACTATATCAAC
TCAAAGCAAGGAGCAACCCCTAGAGAGGCTGTGTTAGCAATTGAAAAATTGGTCAATAATGGGGACACGCAAGCA
GCTGTTTTTGCACTTTCACTACTAGACGTGCTGGTAAAAAATTGTGGTTACTCTATACATTTGCAAATCTCCAGG
AAGGAATTCTTAAATGATCTAGTGAAAAGATTTCCAGAGCAACCACCATTGAGATATTCCAAGGTGCAACAAATG
ATTCTTGAAGCCATCGAGGAATGGTACCAAACCATCTGCAAACATGCCAGCTACAAAGATGATCTTCAATATATC
AACGACATGCATAAATTACTGAAATACAAGGGTTATACTTTCCCAAAAGTTGGGAGTGAAAACTTGGCGGTGTTG
AGACCGAATGACCAACTGAGGACACCTAGTGAGCTACAGGAAGAACAGGAAAGAGCTCAAGCTGCAAAACTAGAA
GAGCTGCTAAGAAGCGGCAAGCCCGATGATTTGAAAGAAGCTAATAAACTGATGAAAATTATGGCGGGATTTAAG
GATGACACTAAAGTAGCAGTCAAACAAGCAATTAACAATGAACTAAATAAACTCAAAAGAAAAGCTGATTTATTC
AACGAAATGTTGACTTCAGCCGATGAGCCTGATCTTGAAAATGAAGCTATTCAAGAATTGTATGGCGACTTAAAA
TCAGCGCAGCCGAAATTCAAAAAGCTTATTGAGGAAGAACGCGATGACGATGCGCTTGTTAGTAATCTATCAAAA
TTTAACGATCTAGTGATCCAATTGTTAAAAAGGTACAAGTCAATAAAAGGTATGAAGGAAGAAGAACTAAATGTC
CCCGATACGAATGAACCGGCAAAAGAGCTTAATTTGATAGATTTTGATGACGATACAACAGCAAACACTCCATCT
GTGACATCTCCAAGCAAATCTTTGCAACCTTTTGACGATCTTTTAGGGGATTTTAATAAGGTAAGTTTATCATCC
CCAAAGTCTCCTCAAGAAAACGACACTGTTGTTGATATACTTGGTGACGCTCACTCAAAGTCATCTGGTATAGAT
TTATTAGATTTTGATTCGCAACCTGGAGAAAGTAAAACCGCTCTGTCCGCATATTCTAACTCGATTGTTCTACCT
AATGGTTTACTAAATAGTTCAAGTAACTCCAAGGAAATCACAGCTCAATCGCAAAGACATATACTTAACCAATCG
GATCACTTACGCATTGATTACGAATTAACTCGTGAGTCGATGACAAAACTACGATTAGTTATCTTCTATTCAAAC
ATAAGCAGTGATCCAATAACTAATTTTGCACTTTTAGTGGCATCACCTAAAGGCACGACATTGTCTCTGCAACCG
CAATCGGGCAATATGCTCCAAAGTAACTCCAGAGATGGTATCAAACAGATTGCTTCCGTAGAGGGTATATCTGTT
AATTTAGGTAAGCCCATAAAATTGAAATGGAAGGCTAATTACTGCACCAAGGGTGATTCGAAGGAAGAATCGGGC
ACGACCAGTTTGCCCACAATATAA

>YBR299W 0.74 Cold
ATGACTATTTCTGATCATCCAGAAACAGAACCAAAGTGGTGGAAAGAGGCCACAATCTATCAAATTTACCCAGCA
AGTTTTAAAGACTCCAATAACGATGGCTGGGGTGATTTAAAAGGTATCACTTCCAAGTTGCAGTATATTAAAGAT
CTTGGCGTTGATGCTATTTGGGTTTGTCCGTTTTATGACTCTCCTCAACAAGATATGGGGTATGATATATCTAAC
TACGAAAAGGTCTGGCCCACATACGGTACCAACGAGGACTGTTTTGAGCTAATTGACAAGACTCATAAGCTGGGT
ATGAAATTCATCACCGATTTGGTTATCAACCACTGTTCTACAGAACACGAATGGTTCAAAGAGAGCAGATCCTCG
AAGACCAATCCGAAGCGTGACTGGTTCTTCTGGAGACCTCCTAAAGGTTATGACGCCGAAGGCAAGCCAATTCCT
CCAAACAATTGGAAATCTTTCTTTGGTGGTTCAGCTTGGACTTTTGATGAAACTACAAATGAATTTTACCTCCGT
TTGTTTGCGAGTCGTCAAGTTGACTTGAATTGGGAGAATGAAGACTGCAGAAGGGCAATCTTTGAAAGTGCTGTT
GGATTTTGGCTGGACCATGGTGTAGATGGTTTTAGAATCGATACCGCTGGTTTGTATTCGAAACGTCCTGGTTTA
CCAGATTCCCCAATTTTTGACAAAACCTCGAAATTACAACATCCAAATTGGGGGTCTCACAATGGTCCTAGGATT
CATGAATATCATCAAGAACTACACAGATTTATGAAAAACAGGGTGAAAGATGGTAGAGAAATAATGACAGTCGGT

GAAGTTGCCCATGGAAGTGATAATGCTTTATACACCAGTGCAGCTAGATACGAAGTCAGCGAAGTTTTCTCCTTC
ACGCACGTTGAACTTGGTACCTCGCCATTTTTCCGTTATAACATAGTGCCCTTCACCTTGAAACAATGGAAAGAA
GCCATTGCATCGAACTTTTTGTTCATTAACGGTACTGATAGTTGGGCTACCACCTACATCGAGAATCACGATCAA
GCCCGGTCAATTACGAGATTTGCTGACGATTCGCCAAAGTACCGTAAAATATCTGGTAAGCTGTTAACATTGCTA
GAATGTTCATTGACAGGTACGTTGTATGTCTATCAAGGTCAGGAGATAGGCCAGATCAATTTCAAGGAATGGCCT
ATTGAAAAGTATGAGGACGTTGATGTGAAAAACAACTACGAGATTATCAAAAAAAGTTTTGGTAAAAACTCGAAG
GAAATGAAGGATTTTTTAAAGGAATCGCCCTACTTTCTAGAGATCATTCGAGAACTCCCATGCCATGGACGAAA
GATAAGCCCAATGCTGGATTTACTGGCCCAGATGTTAAACCTTGGTTTTTCTTGAATGAATCTTTTGAGCAAGGA
ATCAATGTTGAGCAGGAATCCAGAGATGATGACTCAGTTCTCAATTTTTGGAAAAGGGCCTTGCAAGCCAGAAAG
AAATATAAGGAACTTATGATTTATGGTTACGATTTCCAATTCATTGATTTAGACAGTGACCAGATCTTTAGCTTC
ACTAAAGAGTACGAAGACAAGACGCTGTTTGCTGCTTTGAATTTCAGTGGCGAAGAAATTGAATTCAGCCTCCCA
AGAGAAGGTGCTTCTTTATCTTTTATTCTTGGAAATTATGATGATACTGACGTTTCCTCCAGAGTTTTGAAACCA
TGGGAAGGTAGAATCTACCTCGTCAAATAA

>YJL095W 0.75 Cold
ATGCCCTTTTTGAGGAAAATAGCGGGGACAGCACATACACATTCTAGGTCTGATTCGAACTCATCTGTGAAATTC
GGCCATCAGCCGACTAGTTCGGTAGCATCAACCAAAAGTTCAAGCAAAAGCCCTCGTGCAACATCTCGCAAAAGC
ATTTATGATGATATTAGAAGCCAATTTCCCAACCTAACCCCCAACTCTACCTCTTCTCAGTTTTACGAAAGCACG
CCAGTTATCGAACAATCCTTTAATTGGACGACAGATGACCACATCTCAGCTGGAACGCTTGAAAACCCAACGAGC
TTTACAAACAGTTCTTATAAAAATGACAATGGACCTAGTAGCCTCTCTGATTCGAGGAAATCCTCCGGTGGCAAT
AGCGTAAATAGTTTGTCCTTTGACAAGCTAATTCTATCGTGGGATCCTACAGACCCTGATGAATGGACAATGCAT
CGCGTCACCTCATGGTTTAAATTTCATGATTTTCCAGAATCCTGGATATTGTTTTTCAAAAAGCATCAATTGTTT
GGTCACAGATTTATAAAGTTGCTTGCATATGATAATTTCGCTGTTTATGAAAAGTATTTGCCGCAGACTAAAACT
GCTTCATATACCAGGTTTCAGCAGTTATTGAAAAAAACAATGACCAAGAACGTAACAAATAGCCATATTCGTCAG
AAGAGCGCTAGCAAACTTAAAAGTTCCAGGTCTTCCAGCGAATCGATCAAATCAAAATTAAAAAATAGTAAATCG
CAAGAGGATATTTCAAATTCTAGATCAACGTCAGAATCTGCATTGAGCCCAACAAAATCGGGCCCTTCCAAGACC
GATGAAAAGAATTTTTTACATTCTACTTCAACACACCAAAAAACCAAAAGCGCAAGTTCACTATACAGAAGAAGT
TTTATATCCCTAAGAGGCTCATCATCGAGCAATGCTTCCTCAGCAAAATCACCTTCAAACATCAAGTTAAGTATA
CCGGCTCGGCCGCACTCAATTATTGAATCTAACAGTACACTTACCAAATCGGCGAGCCCACCTGCATCTCCTTCG
TATCCTAGCATATTTAGAAGACATCACAAAAGTAGTTCATCTGAGTCGTCATTATTAAATTCCCTTTTTGGTAGT
GGAATAGGCGAGGAAGCTCCAACAAAGCCTAATCCACAAGGTCATAGTCTGTCTAGTGAAAATTTAGCTAAAGGA
AAATCTAAACACTATGAAACTAATGTGTCTTCACCTTTAAAACAATCTTCACTACCCACTTCGGATGATAAAGGT
AATTTATGGAATAAATTCAAAAGAAAGAGCCAAATAGGGGTTCCTAGCCCAAATACGGTAGCTTATGTAACGTCT
CAAGAAACTCCATCCTTAAAATCGAATTCGAGTACTGCTACCTTAACCGTACAAACGGCAGATGTAAATATACCA
TCTCCATCTTCATCACCACCGCCAATACCCAAAACTGCAAACAGAAGTTTGGAGGTCATCAGCACAGAAGATACA
CCTAAAATTTCTTCAACCACGGCGTCTTTTAAAGAAACGTATCCTGATTGTATTAATCCAGACAAGACAGTTCCA
GTGCCGGTAAATAATCAAAAGTATAGTGTAAAGAACTTTTTACTGGACCAAAAATTTTATCCTCTGAAGAAAACA
GGGTTAAATGATAGTGAGAATAAATATATTCTGGTTACCAAAGATAATGTTAGTTTTGTTCCGCTAAACTTAAAA
AGTGTAGCAAAATTATCCAGTTTCAAAGAATCTGCTCTCACAAAATTGGGAATCAATCACAAAAATGTCACTTTC
CATATGACAGACTTTGATTGCGATATTGGTGCTGCAATTCCAGATGATACTTTGGAATTTTTGAAAAAAAGCTTG
TTTTTGAACACTTCTGGAAAAATTTATATCAAAGACCAAATGAAGCTTCAACAAAAACCGAAACCTGCTCCTCTC
ACCTCAGAAAACAATGTTCCTTTAAAATCGGTGAAAAGTAAGAGTTCAATGAGGTCCGGAACAAGCAGTCTGATA
GCATCGACAGATGATGTTTCCATTGTCACTTCGTCTTCTGACATAACATCATTTGATGAACATGCATCAGGAAGT
GGGCGCAGGTACCCCCAAACCCCGAGTTATTACTATGACAGAGTTTCCAATACTAATCCAACTGAAGAATTGAAT
TATTGGAATATTAAAGAAGTTCTTTCTCATGAGGAAAATGCACCAAAAATGGTTTTTAAAACAAGTCCAAAATTA
GAACTCAACCTACCAGATAAAGGAAGTAAATTAAATATTCCTACCCCCATAACAGAAAATGAAAGCAAGAGTAGT
TTTCAAGTGCTAAGAAAAGATGAGGGGACTGAAATTGATTTCAATCATCGTAGGGAATCGCCTTATACAAAACCA
GAACTGGCACCAAAAAGAGAAGCTCCCAAGCCTCCCGCAAATACTTCTCCTCAGAGGACCTTATCAACTTCTAAA
CAGAATAAACCGATCCGCCTAGTGAGGGCAAGTACAAAAATTTCGAGAAGCAAAAGATCGAAACCATTGCCGCCA
CAATTATTATCATCTCCTATAGAAGCTAGCAGCTCGTCTCCTGATTCGCTTACTTCCTCATATACTCCTGCTTCG
ACTCATGTTTTGATACCGCAACCTTATAAGGGTGCAAACGATGTTATGCGTAGGTTGAAAACAGACCAGGACTCG
ACGAGTACTTCCCCATCTTTGAAAATGAAACAGAAAGTGAATCGCTCAAATTCAACTGTATCGACTTCAAATTCA
ATTTTCTATTCTCCTTCACCATTGTTAAAAAGAGGTAACTCAAAAAGAGTTGTTTCGTCGACATCTGCGGCCGAT
ATATTTGAAGAGAATGACATAACATTCGCGGATGCTCCGCCGATGTTTGACAGCGATGATAGTGATGACGATTCT
AGTTCATCCGATGACATTATCTGGTCCAAGAAAAAAACAGCTCCTGAGACTAATAATGAAAACAAAAAGGATGAG
AAAAGCGATAACAGTTCTACGCATTCTGACGAAATATTCTATGATTCTCAAACGCAGGACAAAATGGAGAGAAAG
ATGACCTTTAGACCATCTCCGGAGGTCGTTTATCAAAATTTAGAGAAATTCTTCCCAAGGGCTAACTTAGATAAG
CCAATCACTGAAGGAATAGCTTCACCAACATCTCCGAAATCCTTAGACAGCCTACTTTCACCAAAGAATGTGGCT
TCATCGAGAACTGAGCCAAGCACTCCTTCCCGTCCCGTCCCTCCTGATAGCTCATACGAGTTCATACAGGATGGA
CTTAACGGTAAAAATAAACCATTGAATCAAGCTAAGACACCTAAAAGAACAAAAACCATAAGAACCATTGCACAT
GAAGCTAGTTTAGCAAGAAAAAACTCTGTAAAACTAAAAAGACAGAACACCAAAATGTGGGGTACAAGAATGGTC
GAAGTGACCGAAAACCATATGGTGTCAATTAATAAAGCCAAAAATTCGAAAGGTGAGTATAAGGAATTCGCCTGG
ATGAAGGGTGAAATGATAGGGAAGGGATCTTTCGGTGCTGTTTATTTATGTTTAAACGTTACTACAGGTGAGATG
ATGGCCGTTAAGCAGGTTGAGGTCCCCAAGTATAGCTCACAAAATGAAGCCATTCTAAGTACCGTGGAAGCATTA
AGATCTGAAGTGTCCACGTTAAAAGATTTAGATCATCTTAATATTGTTCAATACTTAGGTTTTGAGAATAAAAAC
AATATTTACAGTTTGTTTTTAGAATATGTTGCTGGTGGCTCCGTGGGATCCTTGATTAGAATGTATGGAAGATTC
GATGAACCGTTGATCAAACATTTAACAACACAAGTATTAAAAGGATTGGCATACCTACACTCGAAAGGTATTCTC

CACAGGGATATGAAGGCAGACAACTTACTTTTGGATCAAGATGGTATCTGCAAAATCAGTGACTTCGGAATTTCA
AGAAAATCAAAGGACATATACTCTAATTCGGATATGACCATGCGAGGAACAGTCTTCTGGATGGCTCCTGAAATG
GTTGATACAAAGCAAGGCTACAGTGCAAAAGTTGATATATGGTCTCTGGGATGCATCGTTCTGGAAATGTTTGCT
GGTAAGCGCCCGTGGTCCAACTTAGAAGTCGTCGCAGCCATGTTCAAAATTGGAAAGTCAAAATCGGCACCACCA
ATTCCTGAGGACACTTTACCATTGATATCGCAAATCGGACGAAATTTTCTGGACGCATGCTTCGAGATAAATCCA
GAGAAAAGGCCAACCGCTAACGAGCTTCTTTCTCATCCTTTTAGTGAAGTAAATGAAACATTCAATTTCAAATCT
ACCAGACTCGCGAAGTTTATAAAGTCAAATGATAAGTTAAACTCTTCAAAATTAAGGATAACCTCTCAGGAGAAT
AAAACTGAATAG

>YOR003W 0.69 Cold
ATGAAATTCTCTACCATCTTACCTATACTTTGGGCAAATTGCTGTCTTTGTATGATCATCCCTGATTTCGACGGG
ATAGTTCGTTTTATTGAGAATATTGATGGTACTCGCAGTGTAAGAGCAGGAGAAGGTTTGGGACAGCATGATCCT
GGAAATTTTCACACTGAACATCAACATGTCGCTCATAAAACAGAGTTTCTTCCTTATCGGTATGTCATAGTCTTT
AATGAAGATATTTCCCTCCAGCAGATTCAATCGCATATGCAAGTGGTACAGAAGGATCATAGTACCTCAGTAGGT
AAGCTTACAGAAAATGACGCTTTTTGGAGGGTAATTTCTTCTTCAGTATCATCTAAATCTCAATTTGGAGGTATT
GATAATTTTTTTGACATAAATGGTCTATTTCGAGGTTATACTGGCTATTTCACTGATGAAATCATTAAAATAATC
TCCCAGGACCCAATCATAAAGTTCGTAGAGCAAGAAACTACAGTAAAAATATCTAATTCGTCATTGCAGGAAGAA
GCGCCTTGGGGTTTACATAGAGTTTCGCACAGGGAGAAACCAAAATATGGACAAGATTTGGAATATTTATATGAA
GATGCCGCCGGAAAAGGTGTCACATCATATGTACTCGACACAGGAATTGATACCGAGCACGAGGACTTTGAAGGG
CGTGCTGAGTGGGGAGCCGTTATACCAGCAAACGATGAAGCTTCTGATTTGAATGGTCATGGAACTCACTGTGCG
GGGATTATCGGATCGAAGCACTTTGGTGTAGCCAAAAATACAAAAATAGTAGCTGTCAAAGTTCTTCGTTCTAAT
GGGGAAGGGACGGTTTCAGATGTTATTAAAGGTATAGAGTATGTTACTAAGGAGCATATAGAATCGTCGAAGAAA
AAGAATAAAGAATTCAAAGGATCGACTGCCAATCTTTCTTTGGGAAGTAGCAAATCGCTAGCTATGGAAATGGCT
GTAAATGCAGCTGTAGATAGCGGTGTCCATTTTGCTATTGCGGCAGGAAATGAGGATGAAGATGCTTGTCTCAGT
TCACCAGCAGGAGCTGAAAAAAGTATTACGGTCGGTGCTTCGACATTTAGTGATGATAGAGCATTCTTTTCGAAC
TGGGGCACATGTGTTGATGTGTTTGCTCCCGGTATAAATATTATGTCCACCTATATTGGTTCAAGGAATGCAACT
TTAAGTTTATCAGGTACTTCCATGGCGTCCCCGCACGTTGCTGGTATTTTAAGTTACTTTTTATCATTACAGCCT
GCACCAGACAGTGAATTTTTCAACGACGCTCCCTCACCTCAAGAATTGAAAGAAAAAGTCCTAAAATTTAGTACA
CAGGGAGTACTGGGTGATATCGGTGATGATACTCCTAACAAATTAATATATAATGGGGGAGGTAAGAAATTGGAT
GGTTTTTGGTAG

>YNR005C 0.78 Cold
ATGGTCTTTACCAGCTCAGAAAGTTCTTCGTTGCTATCTTCTCTTAAAATGACATGTTCCATGGTATCCATGAAC
TCTCTGGAACAAATTTCCTTAATAAAAGGCGTTCCACCATTTTTTACACATATATTGGTAAGCTTCCAGGAAGAC
AATTGGGTATTTGGATTATCGGCCGTATTGAGAATCCTTTTCTTTATACAACGCATAGAATCTTTGGGATTTACC
CTTCTAGATCTTAACACATCTGAAATTTCCAACGCTATGGGGAGATCAAGGTCACCGTTGGGAATGCTCTCACTA
GTGGCTTGTTCTATTAATGCGTCTAACTCACTTGGCGTGCTAACGGACATACTGTTCTTAGTTCTTTACTCACTA
CTCATTCACCTTAGCAAAAAAAAAATCTTGA

>YKL216W 0.81 Cold
ATGACAGCCAGTTTAACTACCAAGTTCTTGAACAATACCTATGAAAACCCATTTATGAATGCATCCGGTGTTCAT
TGCATGACTACACAAGAATTAGATGAATTAGCAAACTCTAAAGCTGGCGCATTCATTACAAAGAGTGCTACAACC
TTAGAAAGAGAAGGTAACCCTGAACCACGTTACATTTCTGTCCCTCTAGGCAGTATCAACTCCATGGGTTTACCA
AACGAAGGTATCGACTACTATTTGTCCTATGTATTAAACCGTCAAAAGAATTATCCTGATGCACCTGCTATTTTC
TTCTCAGTTGCTGGTATGAGCATTGATGAAAATTTAAATTTGTTGAGGAAAATCCAAGATAGCGAATTCAACGGT
ATTACCGAGTTAAACTTGTCTTGTCCTAATGTGCCTGGGAAACCACAAGTTGCTTATGACTTTGACTTGACAAAG
GAAACCTTGGAAAAGGTTTTTGCCTTTTTCAAAAAACCTCTTGGTGTCAAGTTGCCTCCTTATTTTGATTTTGCC
CATTTTGATATCATGGCAAAAATATTGAACGAGTTCCCATTAGCTTATGTCAACTCTATCAATAGTATAGGAAAT
GGTCTTTTCATTGATGTGGAGAAGGAGAGTGTAGTAGTGAAGCCAAAGAATGGTTTCGGGGGTATTGGAGGTGAA
TATGTTAAGCCAACCGCGCTCGCCAATGTTCGTGCATTTTACACTCGTTTGAGACCTGAAATCAAAGTTATCGGT
ACAGGTGGAATTAAGTCCGGTAAGGATGCATTTGAACATCTTCTATGTGGTGCCTCTATGCTACAGATTGGTACA
GAATTACAAAAAGAGGGCGTCAAGATTTTTGAACGTATCGAAAAAGAATTAAAAGACATAATGGAAGCTAAGGGT
TATACATCCATAGATCAGTTCCGTGGGAAGTTGAACAGCATTTAA

>YGR131W 0.73 Cold
ATGCTATCAGCTGCAGATAATTTAGTGCGCATCATAAATGCTGTTTTTCTTATTATATCCATAGGTCTAATCAGC
GGCCTGATAGGTACACAGACAAAGCATAGTTCTCGAGTGAACTTTTGTATGTTTGCCGCCGTTTATGGTCTGGTT
ACGGATTCATTATATGGGTTTTTGGCTAATTTCTGGACATCATTAACATACCCAGCAATTTTGCTTGTTTTGGAT
TTTTTAAATTTCATATTTACGTTTGTAGCAGCCACCGCTTTGGCTGTAGGTATAAGATGCCATTCGTGTAAAAAC
AAAACATATCTGGAACAGAATAAGATCATACAAGGCTCAAGCTCCAGATGTCATCAATCTCAGGCTGCTGTTGCG
TTTTTTTACTTTTCCTGTTTTCTATTCCTCATCAAAGTGACTGTGGCCACGATGGGTATGATGCAAAATGGTGGA
TTTGGCTCTAATACCGGATTCAGCAGAAGGAGGGCAAGAAGACAAATGGGCATACCTACAATTTCCCAGGTTTAA

>YIR031C 0.79 Cold
ATGGTGAAGATAAGCTTGGACAACACTGCTCTATACGCAGACATCGACACGACTCCTCAATTTGAACCTTCCAAA
ACTACTGTAGCTGATATTTTAACAAAAGATGCCTTAGAGTTCATTGTTTTGCTGCATAGAACTTTCAATTCAACA
CGGAAACAGCTTTTAGCCAACAGAAGCAATTTACAATCAAAGTTAGACTCTGGTGAATACCGATTTGATTTCTTG

CCGGAAACCGAACAAATCAGGAACGATCCTACATGGCAAGGTGCTATCCCAGCCCCTGGTTTGATCAACAGATCC
AGCGAGATTACTGGGCCACCATTGAGAAATATGTTAGTCAACGCTTTGAATGCTGAAGTAACGACATATATGACT
GATTTCGAGGACTCTTCATCTCCAACTTGGGAGAACATGATTTACGGACAAGTTAATCTTTATGATGCCATTAGA
AATCAAATCGATTTCAAGACACCAAGAAAGGAGTACAGGTTGAAGGATGACATTTCAAGACTGCCCACTTTAATT
GTCAGACCTCGTGGCTGGCACATGGTGGAGAAGCACCTTTACATAGATGATGAACCGATTAGTGCTTCCATCTTC
GATTTTGGTTTATATTTTTACCATAACGCTAAAGAGTTAGTTAAAATTGGTAAAGGACCTTATTTTTACTTACCA
AAGATGGAGCACCATATGGAGGTAAAACTATGGAATGACATATTCTGTGTTGCACAAGATTTTATTGGAATGCCC
CGCGGTACCATTAGGGCCACTGTTCTGATTGAAACTTTGCCAGCGGCCTTCCAAATGGAGGAGATTATCTATCAA
ATAAGAGAACATTCAAGCGGTTTGAACTGTGGTCGTTGGGACTACATATTTTCGACCATTAAAAAACTGAGAAAC
TTGCCTGAACACGTTTTGCCAAATAGGGATCTAGTGACTATGACTTCACCTTTTATGGATGCTTATGTGAAAAGA
TTGATCAATACATGTCACCGTAGAGGGGTCCATGCGATGGGTGGTATGGCTGCCCAAATCCCCATAAAAGATGAT
CCAAAGGCTAATGAAGCTGCAATGAACAAAGTTCGTAATGACAAAATTAGAGAAATGAAGAATGGGCATGATGGG
TCATGGGTAGCACACCCAGCATTGGCACCGATTTGTAATGAAGTTTTCAGTAACATGGGTACAGCAAATCAAATA
TATTTTGTCCCGGATGTACATGTTACATCATCTGATTTATTGAATACGAAGATTCAAGATGCTCAAGTCACTACT
GAGGGAATCAGAGTAAACTTGGATATTGGCCTACAATATATGGAGGCTTGGTTAAGGGGATCTGGTTGTGTCCCA
ATTAATCATTTGATGGAAGATGCCGCTACTGCGGAAGTATCACGTTGTCAATTGTACCAGTGGGTTAAACATGGT
GTTGTCTTAAGTGATACCGGTGACAAAGTAACTCCAGAATTGACCGCTAAGATATTAAATGAAGAGACTGCAAAA
TTGGCTTCAGCAAGTCCGCTGGGTGAAAAGAACAAGTTTGCCTTGGCAGCCAAGTATTTTTTGCCTGAAGTCACT
GGTAAAATCTTTAGCGACTTCTTGACCACTTTATTGTATGATGAAATTATTAAGCCAAGTGCCAAACCAGTTGAC
TTAAGTAAATTATAG

>YGR112W 0.82   Cold
ATGTCTCTACTAGGCGCCAGGTCCACTTACCGTTGGTTTTCAATCGCTGCATCAATTCCGACTAAGAATGCTATT
GGCAAATCCACGTACCTCTTAGCTTCAAGAAATCAACAGTACCGAGGCATCATAACGTCAACTGTAGACTGGAAA
CCAATCAAGACAGGTAAGAGCCCAAATGATGATTCTCGAAGAGAAAGATCCTTTGGCAAGAAAATTGTTCTGGGT
CTGATGTTCGCGATGCCAATAATATCCTTCTATTTGGGAACTTGGCAAGTAAGGAGATTGAAGTGGAAAACCAAG
CTGATTGCGGCATGCGAAACTAAACTTACTTATGAACCAATACCACTTCCTAAGTCATTTACACCTGACATGTGC
GAGGATTGGGAATACCGTAAAGTTATACTTACCGGACACTTCCTTCACAATGAAGAGATGTTTGTTGGTCCAAGA
AAGAAAAATGGAGAAAAGGGTTATTTTCTGTTTACACCGTTCATTAGAGATGACACCGGTGAGAAAGTTCTGATA
GAAAGAGGGTGGATAAGTGAAGAAAAAGTTGCTCCTGACTCAAGAAATTTACACCATTTGTCGTTGCCTCAAGAA
GAACACTTGAAAGTGGTTTGCTTAGTAAGACCTCCAAAGAAAAGAGGCTCGCTACAATGGGCGAAAAAGGATCCA
AATTCTAGATTGTGGCAGGTGCCAGACATATATGACATGGCAAGATCATCAGGGTGCACGCCCATTCAATTTCAA
GCCTTGTATGACATGAAGGACCATCCAATAATCGAGGAACACACAAGAAACGAGGCTTCACAAAACAATTCCACC
TCTAGCCTGTGGAAGTTCTGGAAACGAGAACCTACGACTGCTGTAAACGGAACGCAAGCTGTTGATAATAATACA
TCGAAGCCTCGTTCTAGGCAGGAAATGCCGACAGACCAAACAATTGAATTCGATGAACGACAGTTTATCAAAGCA
GGTGTTCCCATAGGCCGGAAGCCCACTATTGATTTAAAGAATAACCATCTACAATATTTGGTAACGTGGTATGGC
CTTTCGTTTTTGAGCACAATCTTCCTCATTGTGGCTCTTAGGAAGGCGAAAAGAGGGGGCGTTGTGTCCCAAGAC
CAACTAATGAAAGAGAAACTGAAGCATTCAAGGAAATATATGTAA

>YDR169C 0.8Cold
ATGTCAGAAAACCAAAAGGAGGTATCGCCTCCTCAAGCCATATCGGTCAAATCCGAGGCAAGCTCTAGTATATTT
TCCAAACCTATCTCCACATCATCACCCGCTGGGTTAGCAGCCGCTCAAAGAGTTACGCCCGGGAAACTGTCCACT
TTATTACTAGAAATGGGACCATTGGCCATTAGACACATTACACAGACCTTATGCCTAGATATTCCCTGTTTTAAA
GATTTATCATCTTCCAAGCAAAGACGACTTATAATGAGTGCTATGGAAAGTGGTGATAAAGAAAAATCAGTGGTA
TTTGAAAAAATTGGATGGGGTCAATGGTCCGCGAAAAGAGTAGACCCTGCCAACTTTGATAAGGAGCTGGAAGCG
ACCAATTTTGCAAATGCAAAGGTGAAAGACTTGATTTCTCAAGAGAGTCAAAGAAGGAAAAGCAACAATTCAAAT
TCTAATTCAGGCGGCAAAGTTGAAATGCCTATGAAAGTTGAGCATAATATCACGAATATCGATGGAGCCACCACC
CCGCCTACCGCTGTTGCTTCCACCACCATTCCTGTTAATATCAAACGCAGCAAATCTCCACTTGCAGCAGCAAAC
GTAGTATATATATAGATGAAAATGCGCTAGCATCGGAAGATGAGGATGAGGAGTTTGATGAAGATGACCATCATTTA
CATTACCAAAACAAATCGAGGAACAGCAGTAATAATTTTGGAAAATCTTCAAATGGTGATCCCTATAGTTTCGGC
AGGAGAAGGTCTCAAGTTGTGTTCGCCGATTCAACTCCCGAGAATATTGAGCATGAAATAATAGCACAAAAGATT
AGACCGCTATTAAGAAATAGACGCCGTTCGAGCATAAAACCACATACACCTTTCATTTCCAAACTAAACACACAC
CAAGATTCATCCTACCTTTCCCCCAATACAACCTCAACGACAACCCCATCAAATAACAACAGTAACTCAAACCAG
GCGAAGATAGATTTAGAGAAACTTACTGCGACAAGTGAGCCAACTTCAAGAAGAGCTTCTCGTCTGTCTGTATCG
AAAGAATCAAGTATTAGATCGACACTTTTCCCAAACAAAAACTATTTGATCGTCACTACAAACCCTAACTCTAAA
GCTACGTCGGTTTCCACCTCCCCTAAGCTAGAAGAACAGATGAATGTAAGTTCAAATCCGATAGTATTATCGGAC
AAGGAGAAACATAGAGTAGCTTCACAACACCTGAATGGGGAATCGTCGCCACAACTTGTTCCCCATTCTCATCAT
CAACCTCACTCAGATACAGACGAAGAAGACTGGGAATCAATTGGTGCAGCTTCTCTGAGAAATAACAGTTTAGCA
CCTAACATAGACAGCGTAGCAAGTTCTACTAATGGCGTAGTTAGCCCTAAACCTACAAATCCAAGTTTTACTAAC
TCTCAGAATGGAGATATTGAGCCTCCCCTACAACACGACCAACAGAAACATGAACAGCAGCCAAGAAATGGGGAA
GACAATAGTGCTGCGTTTTTATTAATGAGCTTAAAATCTTAA

>YNL327W 0.73   Cold
ATGAATAAACTATTGTTACATCTAGTACGAGTCATTTCGATACTCGGCCTAGCAAACGCTCTCACACAAACGCAA
CCGATTTTAAAAGATATTCAAATAACAGATAGTTATACCAAAACAAAAGAATGTACGGATCCAGATCATTGGTTC
ATAATAGAAGGGCAACTGTCTATTCCAAAGGGAAGTCAGCAAAATATAACATTCCAAGTACCAGATGCTTTCAGC
TCATTCCCTCAAGAACCCTTCAGCATAAAACATAATTCTAACAGTGTTGCCACGATATCACGCCCAGATAAATCG

ACGAACAATTTCACCATTTCTATTCCAGAAAAGAGTTCAGAAGATATAACTACTACATTTAACTTCCTTGCACAG
CTTACATCAGATGCTAAATCCAAAGTTACAGAACCAAAAAGCATCGTTTACAGTTTCTATTCAGAAAATACTATG
TTTAATGACGTTATTGATTACGTCGCTAAAAATACTTCTGCTATAACAACAGATGGTGGTATATACAAAACTAAT
AATACCGCCTGGTTTACCGTTGACTTACCAATGAGAACGTTCAGAAATCCCGTTTATTTGACTTCTCAGACATCT
TCCTCCTCAGATTACGTATTCGATACTTCGTTAACTAAGTTTGAGGTTGTCACTGCCGTTGATTCTTTTAACGAA
CCTATTAATGCTATCCCATATACAACTGTTCATGACTACTCCACAGAAGATGAAATTAGATGTTTATTTAATTCT
ACAATCAGCGGTGGGCTATACTTCCGTGTGACTTATTTCACAAAGAAGCTATCTACCTCGTCCATATCAAATACT
GTCGAATTAACATATCCCGATGAAGGTACATCTGTCAGACTTTTAGGAAAGAGAGATACTTCAACCACCCTAGCC
TCAGAATTATATTCTGAGTCAGCTGCTAACATTGATTCCACCACCAGTGATGACACAACTAGCTCTGATGCTGCT
ATAACACCAACATACTCAAATTCAACGCTTTCTTCTTATACTTCGCAATCATCCGCTATCCCTGAAGTTGCGGTT
ACTGCATCATTGAGTAGCGGGATCCTTTCTTCTACAGTTGACGGTGCTAGCACCTCGGCGGACGCTTCCATGTCC
GCTGTCTCTACGGTTTCTTCTAGCAGTGAACAAGCTTCTTCTTCAAGTATTTCTTTATCGGCTCCAAGTTCTTCA
AACTCAACCTTTACTACTCCTTCATCTTCTCTGTCTGCTACTGAAACATATAGTATTATCAGTTCAGCTAGTATA
TCTGTCACACAGGCTTCCTACATTGATAATTCTACAACTACCGCTGTTACCCAATCTACTTCTACTATAGCAGTT
TCATCTGCTGAAAAATTGTCAAGCACTCTATCATACACCAGTAACGTCACAATATCAGTATCATCAGCTACCCAA
CATACTACTACTCCTTCATATGTTAGTAACTCCACTACACTCTCATCCTCATCTGTTCTTGAATCTGTAATTAGC
TCTCCATACCTGGCAAACACTACTGTTTCAGGAGCTTCATCTGCAAGTCAATCTACTAATCCGCCATATGTCTCC
AACTCCACAACATCATCCGCAACTCAATTAGCCACCATTGCTCCGTTCGCGATTAACATAACAGGAACAAGTATT
TCTTCTAGTATCACTAATACTTCTTCAGTTAGTTCTACTACGTCCAGTTTATCATCCGGCCCATTTGTTTCAAAC
ACAGCGGTTGCCTCTGGGTCTTATATTCTAACCACTACTACAGAGTCTGCACAACTTACTGAAATAGGGAGTTTG
ATACCAATCTCGACTATTACTACTAGTACCACCACTTCTGGTACCGATAAAACTGGTAGTAATAAGGTGGCCTCT
TCAACTGAAATAGCGCAATCTATCGTAAACAACAGTTCACTTTCGGTGTCAACAATCAACACTAACGCTGCGACA
GCAGCTGCTAATGCCAGAAATGCTACTTTCACTCATGCAACCCATTCTGGTTCGCTTCAACCCTCATATCATAGC
AGCTCACTATTATCGTCCACAATTGACACCAAGGTAACTACTGCTACTACAAGCACCAGTAGAGACGGTAGTTCG
AGTTTGGCTTTCACCACTGGGCTCAATCAATCCGTAGTTACCGGTACAGATAAAAGTGATACCTATAGTGTAATT
TCATCCACAGAATCAGCCCAGGTTACGGAGTACGACAGCTTACTTCCAATTTCCACCCTGAAGCCTACAGTCGTT
ACAGGTACAAGTAGAAACAGTACCTTTAGTATGGTGTCCTCCACCAAATTGACCGAAGCTACTGCAACGGATAAA
GGTGATGCGTATAGTGTAATTTCATCTACACAGTCGGCTCAAGTTACTGAATATGGAAGTATGCTTCCAATTTCT
ACGCTGGAAACACCAACAGTCATTATGAGTACCGATGAAAGCGGCTATTTTACTTTAACTACATGTACTGAGTCT
GGGCAAGCTACAGAATATGGTAGTCTGATTCCAATTTCCACACTAGATGGTTCAGTTATATATACATTTACTGGC
GAAAGTGTAGTGGTGGGTTACAGTACCACAGTGGGTGCCGCCCAATATGCACAACATACAAGTTTAGTCCCAGTA
TCTACCATCAAGGGTTCCAAAACTTCTCTTTCAACCGAGGAAAGCGTAGTGGCTGGCTACAGTACTACAGTGGGT
GCCGCCCAATATGCACAACATACAAGTTTAGTCCCGGTATCTACCATTAAGGGTTCCAAAACTTCTCTTTCAACC
GAGGAAAGCGTAGTGGCTGGCTACAGTACCACAGTAGATTCTGCCCAATACGCCGAGCACACAAATTTAGTTGCC
ATAGATACATTGAAAACTAGCACATTCCAAAAAGCAACAGCAACTGAGGTTTGTGTCACATGTACCGCCCTTTCT
TCCCCTCATTCTGCAACTTTAGATGCAGGTACTACTATTTCATTGCCAACAAGTTCCAGCACATCTCTATCTACA
ATAATTACCTGGTATTCCTCATCTACTATTAAGCCTCCTTCAATTTCTACATACTCGGGCGCCGCTGGTCAATTA
ACCATCCGTATTGGCAGTTTGTTGCTAGGGTTAATCTCATTTCTGCTGTAA

>YGR009C 0.72 Cold
ATGGGATTAAAGAAATTTTTTAAGATTAAGCCTCCAGAGGAAGCGACACCAGAACAGAACAAGGATACTTTGATG
GAACTGGGTATTAGTGTCAAAAACCCTAGCAAGAAAAGAAAAGAGAAATTTGCCGCCTACGGTAAATTTGCAAAT
GATAAAGCTGAAGATAAGGTATACGCGCCACCAGGTTATGAGCAGTATGCTAGGCCACAGGATGAACTTGAGGAT
TTGAATGCTTCTCCCTTAGATGCCAATGCTAACGAGGCAACTGCTGGTTCTAATAGAGGTTCAAGTGGCACGCAA
GATCTCGGAAATGGAGCAGAATCCAATTCTATGCAAGATCCGTACGCTATCGAAAATGATGATTACCGATATGAT
GACGATCCTTATGCAAGGTTTCAAGCTAATAAAAGTAATGGGAGAGGTAGCGTCAATGCTGCGCCTTATGGTGAT
TATGGAGGGGGGTACAATGGCACATCTCTGAATTCATATAATAACGATGGCCCGTATAGTAACCAAAATACTTCA
AACAGCTGGGTGAATGCGAATGGCCGTAATAGTTTAAATCATTCAAACAGCACTTTAAACGTAGGGCCTTCGAGG
CAAACAAGGCAACCGCCGGTTTCAACGTCGACCAACAGCTTATCGCTTGACCAAAGAAGTCCCTTAGCAAACCCG
ATGCAAGAAAAGAGAAATCCTTACGCTGATATGAATAGTTATGGCGGGGCGTATGACTCCAATACTAATAGATCT
AGCGGTACTCGACAAGGAAGCTCCAAGAATGCAAATCCATACGCCTCTATGGCGAACGACTCATATAGTAATGGA
AATTTGAACAGGTCTGCAAATCCATATTCTAGTAGAAGTGTGCGTCAACCGCAATCGCAACAAGCTCCAATGACT
TATACGCCCTCTTTTATTGCTTCTGATGAAGCAGCACGCAATAGTGAAGTTGATTTAAATGAAGAACCTAGAACA
GGTGAATTTGATTTTGAAGAAGTTTATGCTGACAAGTCTGCAGAAAATAGAGCGGCATTAGATGAGCCTGATTTG
AATGCAGTAATGACGAATGAAGATTCAATAGATTTAAATGCGTCCGAAGTTGATCATAGTTCAAGACAGCAGCAG
CAGCAACAGTGGTTCATGGATGAGCAGCAACAGCAACAGCAACACTTTAACGCAACAAATAACCAATATGGAGAT
CAAAGGGGTTACAAAACATTTGAAGAAATACAAAAAGAAGAGGAGGCTCGCCAGCAGCAGGAAGAAGATGAAGCA
GTAGATGAAATCAAGCAGGAGATCAAATTTACGAAACAGAGTTCCGTAGCCTCTACTAGAAATACACTAAAAATG
GCTCAAGACGCCGAAAGAGCAGGTATGAACACATTGGGTATGCTGGGTCATCAAAGTGAACAATTGAATAACGTA
GAAGGAAACTTAGATTTAATGAAGGTGCAAAATAAAGTTGCAGATGAAAAAGTTGCAGAACTAAAAAAATTGAAC
CGTAGTATATTGGCTGTCCATGTTTCTAATCCGTTTAATTCCAAGAGAAGGAGAAGGGAGAGGGAAGAGCAGCTG
AAGAATAGGAAAATTGAAGAAAAATTAATGAGAGAGCAAACAAGTCAACAATTGTCTCAGTCCACTCAAAGAATA
GAGGGTGCTATGAACGCAAATAATAACATAAGTGAGGTGCGGGAAAGATATCAGAGGAAGAATGTTCTAGAAAAG
GCAAAGAGATATCAGTTTGAGAATGATGAAGAAGATGACGAAATGGAATTGGAAATTGATAGGAATTTGGACCAG
ATTCAGCAGGTTAGCAACAGATTGAAGAAAATGGCCTTGACCACTGGTAAAGAATTAGACTCTCAGCAAAAACGT
CTCAACAACATTGAGGAAAGCACTGATGATCTAGATATCAATCTCCATATGAATACCAACAGGTTGGCAGGTATC
AGATAG

>YDR292C 0.71 Cold
ATGTTCGACCAATTAGCAGTCTTTACCCCTCAAGGTCAAGTACTTTACCAATATAACTGTTTAGGAAAAAAGTTT
TCTGAAATACAAATTAACAGCTTTATATCCCAGCTGATTACTTCCCCAGTAACTAGAAAAGAAAGTGTTGCAAAC
GCAAATACAGACGGATTTGATTTCAATCTTTTAACAATCAACAGCGAACACAAAAATTCTCCTTCATTTAATGCA
CTATTTTATTTGAATAAGCAACCAGAATTGTATTTCGTAGTGACTTTTGCCGAGCAGACTTTAGAGCTTAATCAA
GAAACTCAACAAACACTTGCACTGGTGTTAAAACTCTGGAACTCATTGCATTTAAGTGAATCCATTCTAAAAAAT
CGTCAGGGCCAAAACGAAAAGAACAAGCATAACTACGTCGATATTCTTCAGGGAATTGAAGACGACCTGAAGAAA
TTTGAGCAATATTTTAGGATAAAATATGAAGAGTCAATAAAACAAGACCATATCAATCCAGATAATTTTACCAAA
AATGGATCAGTACCCCAATCGCATAATAAAAATACCAAGAAAAAATTGAGGGATACAAAAGGTAAGAAGCAATCT
ACAGGAAATGTTGGTAGTGGGAGAAAGTGGGGCCGTGATGGTGGCATGCTTGATGAAATGAATCATGAAGATGCA
GCCAAATTAGATTTCTCATCATCCAACAGCCACAATAGTAGCCAAGTAGCTCTAGACTCCACTATTAATAAAGAT
TCTTTTGGGGATAGAACAGAAGGAGGCGATTTCTTAATCAAAGAAATTGATGATCTTTTGTCTTCTCATAAAGAC
GAGATAACTAGTGGGAACGAAGCTAAAAATTCTGGATACGTCAGTACAGCTTTTGGATTCCTTCAAAAACACGTT
CTAGGTAACAAAACGATCAATGAAAGTGATTTGAAATCTGTATTAGAGAAGTTAACACAACAATTGATAACCAAA
AATGTAGCACCAGAGGCAGCAGACTATTTAACACAGCAAGTCTCACATGATCTTGTAGGCTCAAAAACTGCAAAT
TGGACCAGTGTTGAGAATACTGCTCGTGAATCTTTAACAAAAGCATTAACTCAAATATTAACGCCTGGTGTATCC
GTTGATCTCCTCCGTGAAATTCAGAGCAAAAGAAGCAAAAAGGATGAAGAAGGTAAATGTGATCCCTATGTGTTC
TCTATAGTTGGTGTTAATGGTGTTGGTAAGTCAACAAATCTTTCAAAGCTAGCGTTTTGGTTACTGCAAAATAAT
TTCAAGGTCTTAATTGTTGCTTGTGATACGTTTAGGTCTGGTGCAGTTGAGCAACTAAGGGTCCATGTTGAAAAT
TTGGCACAGCTAATGGATGATTCACACGTTCGTGGCTCCAAGAACAAAAGGGGTAAAACTGGTAATGACTACGTT
GAACTATTTGAAGCTGGTTATGGTGGATCTGACTTGGTAACCAAAATTGCAAAGCAAGCCATCAAATATTCTCGT
GATCAAAACTTCGATATAGTGTTAATGGATACTGCCGGGAGAAGGCATAATGATCCTACTTTAATGTCGCCATTG
AAGTCTTTCGCTGACCAAGCCAAGCCAGATAAAATCATTATGGTTGGAGAGGCTTTAGTAGGTACTGATTCTGTC
CAGCAAGCCAAAAATTTTAATGATGCCTTCGGAAAGGGAAGAAATCTTGACTTCTTTATTATCTCCAAGTGTGAC
ACAGTTGGTGAAATGCTGGGTACTATGGTAAATATGGTTTATGCTACGGGGATTCCTATCTTATTCGTTGGCGTG
GGACAAACCTATACCGATTTGAGGACATTAAGCGTAAAATGGGCTGTTAATACATTAATGTCTTAA

>YGR018C 0.81 Cold
ATGATTGACATTTCTCACGGACACACTTCTTGTTCCTCCCACAGATCTTCGTCTTTGCCGTCGGTTTTCTGGAAA
ATCCATCTTTCACCACCTCTTCCCTCTTTCAAGTTCAAAAAATCAACAGAATCAACACAAAGACAGACAATACCA
AAGTTTTCCAGTCCAGCGTCCTCTTTAGCACCGTCAACTCCTCTATGGAGTTTGTCCAGTTGTTTGCTAGTTTCA
GAGGTAAGTTTCTGGCCCGGGGCTGGCTTCCTATACAATGACTTGGTATGCCTTGATAGAGAGGACCATTGTCTT
AGTAATTCAGCCTCCCATTCTTGTGGTTGA

>YDR375C 0.81 Cold
ATGTCGGATAAGCCGATTGACATACAATATGATAAACAGGCTACTCCAAATCTGAGTGGTGTAATTACTCCGCCA
ACAAATGAAACTGGTAATGACTCAGTCCGGGAAAAACTTTCAAAACTAGTTGGCGATGCTATGTCAAACAATCCC
TATTTTGCCGCCGGCGGTGGTCTTATGATCCTTGGTACAGGTTTAGCTGTAGCTAGATCCGGTATAATAAAAGCC
AGCAGGGTCCTATACCGACAAATGATTGTCGACTTAGAGATTCAGTCGAAGGACAAATCATACGCCTGGTTTCTA
ACATGGATGGCCAAACATCCTCAAAGGGTATCTAGACACTTATCCGTTAGGACAAACTACATACAACATGACAAT
GGATCAGTTAGTACAAAATTTTCGCTGGTTCCGGGTCCGGGAAATCATTGGATTCGTTATAAGGGTGCTTTTATC
CTGATTAAAAGAGAAAGGTCCGCAAAAAATGATAGATATCGCAAACGGGTCACCATTTGAGACCGTAACCTTAACT
ACACTGTACCGTGACAAACATTTGTTCGATGACATTTTGAACGAGGCTAAAGACATAGCCCTGAAGACTACTGAG
GGCAAGACTGTTATTTACACTTCTTTCGGCCCTGAATGGAGAAAGTTTGGTCAACCAAAAGCTAAAAGGATGCTG
CCCTCCGTCATTTTGGACAGTGGCATCAAAGAGGGTATTCTCGATGACGTTTATGATTTTATGAAGAACGGCAAA
TGGTACTCTGACAGGGGTATCCCATATCGCAGGGGATATTTACTCTATGGCCCTCCCGGATCTGGTAAGACTAGC
TTTATCCAGGCTTTAGCGGGCGAATTAGACTACAACATTTGTATTTTAAACCTCTCCGAAAATAATTTAACAGAT
GACAGATTAAACCACTTAATGAATAACATGCCAGAAAGGAGCATACTATTACTGGAAGATATTGACGCGGCGTTC
AACAAAAGAAGTCAAACTGGTGAACAAGGTTTTCATTCTAGCGTAACATTTAGCGGTTTACTGAATGCCCTGGAT
GGCGTCACATCTTCTGAAGAAACTATTACATTCATGACTACCAATCATCCTGAAAAGCTAGACGCGGCTATAATG
AGACCAGGTCGTATTGACTACAAAGTCTTCGTTGGTAACGCAACTCCATACCAGGTAGAAAAAATGTTTATGAAA
TTTTACCCAGGTGAGACTGATATATGCAAAAAGTTTGTAAATAGTGTTAAAGAATTGGATATCACCGTAAGTACC
GCCCAGTTGCAAGGACTTTTCGTTATGAATAAAGATGCACCACACGATGCGTTGAAAATGGTCTCCAGCTTACGG
AACGCTAATCATATTTTCTAG

>YDR421W 0.79 Cold
ATGTCTGCTAAGAAAAGGCCTTCGGGAAACGCAGCATTTGAACTTCCAAAACGGAGAAGAACCTACCAAGCTTGC
ATCAGCTGCAGATCAAGGAAGGTGAAATGTGATCTTGGTCCGGTTGATAACCCACACGACCCACCGTGTGCACGT
TGCAAAAGGGAGCTAAAAAAATGTATTTTTAGCTCTAATAAGGGAACTTCGAACGACTTGCCTCCTAATTCGATT
AATGCAATAAGTCTGCCATCTCTAGGTAAGAGTAAGCAAGAGATACAAAATGACTCAACGAGCCCAATTTTATCG
GATGTACCTTTGTCAAGAAAAGGGATTAGCAGTGAAAAATCATTTAAATCAGAAGGGATGAAGTGGAAGCTTGAA
CTTTCCTCCATGCAGAACGCCCTAGAGTTTCTGGCCCAAGCTGCTGGAACTGTAGCAAAAGAAGGCGCAAAAGAA
ATCATAAAAGAGAAGTCCACAACACCGAAACCACTGAAAAGTTCTTTGGATGCGACAAATAAATCTGCTACGGAC
GAGGGGTTGAAACGACTTTCAAAAAGTGACAGTACTAACACACTTTACGAAAATACAGCAGATATGCTTAACCAC
ACGCTGAATACGAATAGGAAAACATCACAATTAATGGAAGAAATCGGGAAAGTGAGACCTCCACCTACCCGGAAA
ATTGACGACTTCGATTACATCGGTCCAGATAGCTTGCTTACTAAAGAGGAGGCGATTGAACTCATAGAGGCATTT

TTTCTCACGATGCACCCATTCTTTCCCAATATCCCACTTCAATTGCATGATCCGAAAGAATTAGCAGAATATCCA
ATTTTGTTTTGTGCAATTTTGACTGTTTCTGCTCGCTATCATCCTTTCGATACTTTAGGATTAGACAATGGAGAA
GATGGGATGAGACACATTGAAGTTCACGATAAATTATGGGTGTATTGCCAGAAGCTTATATCTCAAACAATTTGG
GCAGAGGCTAGTACTAGGTCAATTGGCACCGTGCTTGCTTTCATAATTTTCACGGAATGGAACCCAAGGAGTATC
CACTATAAATGGTCTGACTATGCAAATGATCCTGAGTTAAATAATGTCAACGCTAGAGGAAGTAAGAATATTAGT
ACGAGGAAGGACGAAGAAGGATTGACCGGTGTCGGCGCAATCCGTAGAAGCGACCGAATGTCATGGATGCTGACA
GGGTCTGCAGTAAGGCTGGCGCAAGATATGGGATTCATTGAAAACAGCTCAAAAGTATTCATTGTCACTCATATA
TCTGAGACTACCAGTGCTATGAATATGAATCAAAGATCCTTATTAGCAGAATCATTCAGCGTTTTAAACTTGAAT
TTAGGAAAAATTGAAAACGATGGAAATGAAAGCAATGAGGATTACCTTGGGAATGAAAAATTTTATCTGAATGAG
ATTTTGCCTGATGAAGAAAGTAAACTAAGGTGGAAGAGAGTTTTTGAAAACTCAGAAAATGACCACGACAACGAG
AAAAATTTTTTGACTGATTGGGAGAGGGAGTTTTTAAACGATGAGTATGTTCTATATTATTCTAATAAAAAAGAT
GATACTAATCTAGCACAGAATCACATACCACCATTTCCACTAAGATTCTCATTCGCCCAAAGAGCAAAAATAGAG
ATCATTCGAATCCTATCCATAGCATATGAGACGATTTACTGTGAGAAGAATAAGAGGAAATTGGCAACGACAGAT
CAGAGACACAACCTCTCTGTCCTAAGTGTTTTTTCTCCCTTGATAGAGGGCTGGCTCAGTAACTACAGAGAGCTT
CTTGTACCCCTTTCTGATGTCCCTTTCTCACTAGCAGATAGGAAGAACAAAAAACAAATATTTGATAACATCGAT
AGAATAAATGGTGAAAGTATTATCACAGATTTCAATTATTGTCAACTTTATATCTTTTCCCTCGCACTGCAAGTG
GATGGGAAAACAAGCAGATTGAATATGAATGAAATTGTGACAAGCGCGAGGTATGTGGAATTAGCATACAGATCT
GCAAAAGAAATATTGAGTTCTGCAAAGCGAGTTTCAAGGCAAGGAATGCTAAAATACATGCCCGTAAGATGGGTA
ATACGAATAATTAGATCCATTGCGTTCATAGTAAAATGCTACCTAACACTTACTGGTAGTGAACTGGCAACAAAT
CCAGATGCCAGAAACATTTTAAAATTAAGTGCAATATCGGTTGACGAAACATTCGATATCATCCGTGACACTGCG
GTTACTTTGAAAGAAGCTACACCAGATGAATTACATCTATGTCAAAGATACGCAGCCATATTAATGTACTTATGT
ACAGAGATGAAACTTCGAAAAAAATCTTACTTAGAACGACCACCCTTATTAAGAGATGGCACCACTCCTCTAGAG
AGCAATCGCGAATCCTCACTGGAGGGACAAGACTTAACTAAGAAGCCAATATTCTCTAAACGTATTGGATATAAT
AAAACCGAGACTACTTTTGAACCCTCGGAAAGGCCTTTGACGGAGGAAATTAATAGCAATTCTCAGAATTCAAAT
GATACATCTTCTAAAGGTATCGTTGATCCTTTTGTTGAACAAAACAACGATATCACAACAGCGTTACTGAACAAT
GAACTCTTCCAAGGTCCATCTCTATCTGACGAGGTTACAGATTGGTTCGGTGCTAGTGAAGATATCGGGCTTGAA
TTTGTAGAACCATGGACAGAACTTATTGAGCAACGATATATGCAATGTGGAGATGGTGATAATAATAATTTCGAA
AATTTATACAACTTGTTCGTGAATAGTAATAACATCAATAATGATATTAATAACTCAAGGCCAATAACGCGTAAA
TAA

>YDR104C 0.79 Cold
ATGGATTCTATCGTTAATGTTGTTGAAGACGATGTTAAGTACGCGCAGAGAGTGACTAGCTTCAGCTCACCACAA
AATGCAAATGTCAAGGTCTTCACCATTCCCCGGCATTCCTTCACAGCGTTTAGACTGTCGTATGTTTCACCTACA
GAGCTTTCAGCATGTTCACAGGTGACTTTGCTAGGTGGTATTCCAAAACAATGGTACGCTGATCAAAATAATCAA
GTCTGGAAACTTCTAACTAAAATTTCACTTCGGAAGGTTAGGAAGCAATCTGACATGCTAAGAAGATATGGCTAT
GGAACAATTTACAAAAAGCGTGTAGGGAAGATCCCAACTGCATTGTACTTAAGAAAGCACTTTACATGGTCCTAT
GAAGATAATACATCAATTCACAACGGTCATCGTCTTAAAGAAGCGGAAATGGAGATGAAGAGAACTAGGTCAAGT
CCTGTGCAGAAAAGTGAATATAAATTAAGTCTACCCAGAAGGTGTAGGAGTTCTTCCGATCAAAACTTCATGCGA
CAAGAGCTTTTGAAAGAAAAAAAATCAGAGCTTTCTAGAAACAATAGTTTGCCTCTCATCGATACGGCGCAAGCC
GTGGATATCCACCCAGTTTTACATGAAGAAGATCAAGAAAATACAAATAAAGGAATAAATCACTCCTTTCGAAT
TTGAAACGAAAAGATCTAGGGGAATCAAAAAGCATATCCAGAAAAGATTATTCGCATTTTGATAGAATACCGAGC
CCCTCTTCAGCCAGAAGTGTTGGAGAAACAGACTTTAATTATAATAGAGAACCTTCTGAAGATACTTTGAGATAT
CCCGACAGTATAATTGAAGTAACAAATCGGACAAGCCCCGCCCCAAATAGTATTCTAAGTAGATCAGGACAATTT
GTTAACTCGAATGACCTAAGTGATGGCTTTTCAACATCTAATACTATCAATAACGTGGGATTAAATGCAAATGAA
AAGATTTTTTTAATGCTCTACAAAGTATGGAAAAGGAAAACTTGATGCTGTGGAAAACATCCCAGAAATATGGG
ACTTATCTCGATGAAAGACGTAAAAGTGCGGATGCCTTTCAAAAAAGAGAAAGAGGTTCATGCGTGGACATTGGT
AAGTTGCATTCGAGTCATCTTCCGTTTATTAATATCCTACCTCCATGGCCTACCGAACTGACGGAGGAAGAGAGA
ATTATACACGATAGGCTAGCCTCTAAACATTCTCATCATATAAGAAAACATGTTCACAATGCTCGAAACAAAACT
AGCTGCAAAATCAAAGATAGTGTTGGAACTTTCTTAGGTATGACAAATTCACTAACAAATAAAGCAACTGTCAAG
AAACGCACCGGTCAGATCCTTAAAAAAGAAAAAAATGCTAGTGATGGTAAAAGAAGCTATACAAAATAAAGTACCA
CTTCCCAACTTCTCTGAAAACGAGTGCTTTGATACAAGGGTCAGTGAACGGTGGAAAGAATATATTGTTATTGCA
AGATCCACGGGAAGATTCGATCCCCCGATACTTTTACAGTTCTATCGTCATCGTCATATCCCAGAAATCGAAGAT
ATCAGTTCCATAGCTACAAAGTATCACCGCAACCCGTTGGATTTTTTTCTTTCAAGAAATTGTATTGTAAAATTC
TACAGCTCTCTAGACAAAACTATATCCATACAAAAGCCTGATAAGCGGTTAGGTGGGTTTATAGACGAGAGTATT
GAAAAGAAGGACGAATTAAAACACTATTCCCCCATTAAAATTTTTATCTTAAGATGCAGCAGTATTCGTTCGTCT
GGTAGATGGTACAAATTTCTACTAGAATCTTTGGACCGACAACTATTTACACCTGCCATCAACTTGAAAATTCCA
TTAACTGAGATCTCGATAAAAATAAACTTGAATGAAATTATATTCCAAAAGCTAATAGATTTAGGGAAGCAGGAA
AAAGATAGATTAAAAATATGTTTTCTTCAAAGAGGTTATAAAATTTTCCAGCATCCCATTTTACGATATTTCACA
GTTGCTATTTTGGAGAAATTAAAATTGGCGCATTATGACTATCTGATTAGAAAATGGGATACAGAAAATCCTGTA
CTAGGATGTGCCCTGAAAAGATATGATAGATTGGAGTGGATTCCATGTGATGAAGATTCTCTGGTAACTGGAATT
TTTGCATTTTGTCAATCTCACCTAATCCAGTACAGGCCAATTGCGAATCGTTTAAGGGAAACAAAATCTTTAGAA
GGAAAATGTCTGAAAGAACCAACACCTATTGAAGGTTTTTTAATAAGATTGACTGACAAGTATGGCTCTGCGAGG
ACAAATTTTGGTAAATATAGTATTTCAACAGCTTACTTCTTCACGTGTGAGAATTTGCTTTTTTCGATGAAAGCA
TATAGAGCAAACCCACCATTGCCTATAGATTCGATGATTGACGACACTAGCACAGAAATAGAAAAAGAAGAAATT
TGGAAACAGTGGAAGAAAATTCCAGAGGTTTACGAGCAACAACCTTATCCCTTAGACACAAATGATCACATCGAG
TGGATGAACTGTCAAACGACCCAAAGTGAATATGATTCCAGAGATTTCTATGCTTTCCATTGTTTTCATAGAAGG
ATTGATCAAATACTAAAAACGGACAATGTGATTGACCTAACTGAGGTGAAAGATATATATCAAGGAACAAGGACT

GATTACGAAGCAGACAAAATAAAATATGGGGTTTACAAGGAAGCTAGTGAAATCTTTTGGCATAGAAACTACGAG
ATTGATGATGTTTCACGGTCCGTAATAAATATAGAAACCTCAAATGGACTCCTCTTGAAGCTGCTTGCTACATCA
GCAACAGTTGCTGAAGAGTGGGTCATAAAGCTGAAACAAATGATATCCTATTGGAAAAACAAGCAAAGGGAAGAT
ACGGAGAGGCTTTTAAAAATAAGGCGTTCAAATGCAGGTCTTTTGATGCTAAATGGAGAAGAGGAAACAAAAATT
GGTGAGAATACATTGCGCTGGATCGTAGAACATGGGCGTGCAGATGAGCAAACCTTTAATGCAAATGGAATATCA
TTAAGTAGGCCGTTAATTCAAAAGGGACCTTTATATCAAAAGCCGCACAAGCATTCTGTTTTCTCGAAGTACTAC
GTCGTTTTGATATCAGGTTTTATTGTACTGTTCCACTGCTTTCATAGATCAACCACAGGTTTTGCTAAGGAAGTT
TTGGAGTATGCTCATTATGTAACCATTCCGATTGACGATTGCTACCTATACTCTGGGACCACAACAGAACTAGAC
CTTCTGCAGAGAGATCGTACTTTTGACGAAATAAACTATGGATCCCACGCTCTCCCACGAGTTTATGGGGACGGA
TGGCGATCTGTAGAAGACGAGAGTTCGCGATGCTTTACGCTTTGGTTTGGAACAAGGCGTGCCCTTTCTAGCAAT
CGCTTACAGAAGAAAGGGAATGAAAAGCAATATACGCAAGATTATGGTAGACAAGATAATAATATTGACCCACCT
TCTGCTCCGGAAGCTGACTTGAATAACTCTAATGTTCCAAGTAACACAGATAAGATCCATTTCACTAAAAAGTTA
GGTGTGAGCGGCAAGTCAATGGTTTTCATGGCAAGATCAAGACAGGAAAGAGATCTTTGGGTAATGTCTATTTAC
TATGAACTTGAAAGACTAAGAAGAACTGCGTCCACTTCAAATTCACGGAATCAAACTATGTAA

>YKL120W 0.71   Cold
ATGTCATCTGACAACTCTAAACAAGATAAACAAATTGAAAAAACAGCCGCCCAGAAGATATCGAAGTTTGGTTCG
TTTGTGGCTGGTGGGCTAGCAGCATGTATAGCTGTTACAGTTACTAATCCGATCGAATTGATTAAAATCAGAATG
CAGCTTCAAGGTGAAATGTCAGCATCAGCTGCAAAAGTTTATAAAAATCCAATCCAAGGTATGGCGGTAATTTTC
AAAAACGAAGGTATAAAAGGTCTGCAAAAAGGGTTAAATGCTGCTTATATCTATCAAATTGGGCTAAATGGTTCC
AGATTAGGGTTTTATGAGCCAATCAGATCATCATTAAATCAGCTTTTCTTCCCAGATCAAGAGCCACATAAGGTA
CAGAGCGTCGGAGTTAACGTCTTTTCTGGTGCCGCATCTGGTATAATTGGTGCAGTCATTGGCTCTCCATTATTC
TTGGTGAAAACAAGACTTCAATCATATTCCGAGTTTATAAAAATTGGTGAACAAACGCACTACACCGGTGTTTGG
AACGGGTTAGTAACCATTTTTAAAACCGAAGGTGTTAAGGGTCTATTCAGAGGTATTGATGCGGCAATTTTAAGG
ACAGGTGCTGGTTCCTCTGTTCAACTACCTATCTACAACACAGCAAAGAACATTTTGGTCAAAAATGATCTGATG
AAAGATGGCCCAGCATTACATTTAACTGCTAGTACTATCTCTGGGTTAGGTGTTGCCGTCGTTATGAACCCATGG
GATGTCATTTTGACAAGAATCTATAATCAAAAAGGTGACTTGTACAAGGGACCTATAGATTGTTTGGTCAAAACT
GTTAGAATCGAAGGTGTAACCGCTTTGTATAAGGGTTTTGCAGCTCAAGTGTTCAGAATCGCACCTCATACAATC
ATGTGTTTGACCTTCATGGAACAGACAATGAAACTAGTTTATTCGATAGAGTCGAGAGTTTTAGGCCATAATTAA

>YNR060W 0.79   Cold
ATGTTGTTGGTACATATTATATCTTTCCTTCTGTTCTTCCAGCTTTCGGCCGCAAAGGCCCCACCCAGTAAAACG
TCTCTAATAAATACTCATGAGAGAAGGTCGATATATTCATGCTACGTTGGTTTACGTAAAGAAACATGGGGGTTC
AATGGGTCCGCTATATGTCGGTATGAACCAGCAATCCAATCGATGCTTTACTGTCTTTACGAAGACACGCATGAG
AAAGGGTATTCAAATAAAACTTTGGAGAAAGGTTTTGAAGAAATGAGACAATTTTGCTATACACCAAAGTTTTTG
AACATGACTGATGCCGAGTTTTACACCTCATTGGATAATGGAACATACTATATACAAGATCAACCTAAAGCTGGC
ATCAATATCACTTATCCTATCAGACTGAACACTACACTAAGAAAAGCATACTATGATGCATACTATGGTTACTAC
TATAACCATGACATTCCGTATTATTTCGGGGGCATTATCTGTGCATACTTTGTGGGTGTCATGTTGCTTGCAGGT
TTAATTCGTTTTTTGAATTATACCCCAATAAAAAAGATTATGTTTCAGCAAAAGCTAGTCAATTACGTGAGAGGT
TATACTACTCTACCCACTCTTTATGAAAAGCATGCAGAGCCCTTCTCGTACTTAAAAGTGATAACAGGCTATCTT
CCTACTAGGTTTGAAACGTTGGTTATTTTAGGCTACCTCATACTTCATACCATTTTCATGGCCTACAAATATCAA
TATGATCCATACCACATCATATTTGCCGCTCATAGAGCAGAAGTGGCACATTTTGTTGCGTACAGAAGCGGTATA
CTTTCTTTTGCACACCTGCCACTCATTGTTTTATTTGCGGGAAGAAATAACTTTCTCCAACTTATTTCTGGCTTG
AAGCATACCTCATTCATTGTGTTCCATAAGTGGCTGGGAAGAATGATGTTTCTTGATGCAATAATTCATGCTGCC
GGCTTTACGAACTATTATTTGTATTATAAAAAATGGAATACGGTTAGATTAAGAGTCTACTGGAAATTCGGTATT
GCTACCACCTGTTTAGCGGGAATGTTAATTTTCTTTTCCATCGCAGCATTTAGAAGACACTACTATGAAACGTTT
ATGGCCCTCCATATAGTATTCGCAGCACTATTTCTCTATACTTGTTGGGAGCATGTTACTAACTTCAGCGGTATC
GAATGGATTTACGCGGCAATAGCAATTTGGGGAGTTGATAGAATTGTACGTATCACCAGAATTGCACTCTTAGGA
TTTCCTAAAGCAGATCTACAACTGGTTGGATCTGATTTGGTCCGTGTAACGGTTAAAAAACCAAAGAAGTTTTGG
AAGGCAAAACCAGGCCAATACGTTTTCGTTTCCTTCTTGCGTCCATTGTGCTTCTGGCAGTCGCATCCATTTACA
GTGATGGATTCTTGCGTAAACGATAGAGAATTGGTCATCGTTCTGAAAGCAAAGAAAGGTGTGACAAAACTGGTA
AGAAACTTTGTTGAACGTAAAGGCGGCAAGGCATCCATGAGATTAGCTATCGAAGGCCCTTATGGCTCCAAGTCC
ACCGCCCATCGCTTTGATAATGTATTATTGTTGGCAGGTGGCTCAGGGCTTCCCGGTCCAATTTCTCATGCCCTT
GAATTAGGAAAGACAACAGCTGCAAGCGGTAAAAACTTTGTACAGTTAGTCATAGCAGTAAGAGGACTAGACATG
CTCAACGCGTGTAAGAAAGAACTAATGGCATTAAAGGGCTTGAATGTTCAAGTTCACATTTATAATTCTAAGCAA
GAGCTAGCTTCGGCTGAAAAAATTTCCTCAAATGAAGTCAAAAACGGTGAAACGACAGCAGAGAAGGCCCCATCT
AGTCTAAGCAATTCAGAAAAAGCTCCTTCTGAAAGTGAAAATACAGAACTACCTCTTTCCCTGAATGACACGTCT
ATCTCCGATTTAGAATTTGCCACTTTCCATGTTGGAAGGCCAAATGTTGAAGAAATACTAAATGAATCTGTTAAC
CATTCTGGTTCACTTGCCGTCGTATGTTGCGGACCACCTATTTTCGTCGACACCGCTAGAAATCAAACTGCCAAA
GCTGTTATCAGAAACCCATCAAGAATGATTGAATACTTGGAGGAATACCAAGCCTGGTGA

>YKR105C 0.75   Cold
ATGGAGGAAACTAAGTACTCTTCGCAGCAGGAGATAGAAGGAGCATGTGGTTCAGACGCTTCATTGAATGCTAGA
GGTAGCAATGATTCTCCAATGGGACTTTCCTTGTACCTCTGCCTGGCTTCGTTAACTCTTGTACTATTCATAACT
GCACTGGATATTTTGATAGTGGGAACTATTATTGACGTGGTCGCAGAACAGTTCGGAAACTACTCCAAAACAGGT
TGGCTCGTTACAGGCTACAGTTTACCAAATGCTATTCTGAGTCTCATTTGGGGAAGATTCGCATCTATCATAGGT
TTCCAGCATAGTCTCATTTTAGCAATACTTATTTTTGAAGCCGGATCCCTAATTGCTGCCCTTGCCTCTTCAATG

AATATGCTCATTTTCGGTAGAGTTGTTGCTGGTGTTGGGGGAAGCGGACTCCAAACGCTTTGCTTTGTTATTGGT
TGTACGATGGTTGGTGAAAGGTCACGTCCATTGGTGATTTCCATCCTAAGTTGTGCATTTGCTGTAGCTGCTATC
GTTGGTCCTATAATCGGAGGTGCCTTTACAACCCATGTTACCTGGAGGTGGTGCTTCTATATCAATCTTCCTATC
GGTGGTCTTGCCATTATTATGTTTTTACTCACCTATAAGGCCGAGAATAAGGGTATACTTCAACAAATTAAAGAT
GCTATAGGAACAATCTCGAGCTTTACTTTTAGTAAGTTCAGACACCAAGTTAATTTTAAAAGACTTATGAATGGC
ATAATCTTCAAGTTTGACTTCTTTGGTTTTGCCCTCTGCTCTGCAGGGCTGGTCCTTTTCCTACTGGGGCTAACC
TTTGGTGGTAATAAATATAGTTGGAACTCTGGCCAAGTCATCACATATTTGGTTTTGGGTGTCTTACTTTTTATT
TTTTCATTGGTGTACGATTTCTTCCTATTCGATAAATTCAACCCGGAACCTGATAATATATCCTACAGGCCTCTC
CTTCTAAGAAGATTGGTAGCAAAACCAGCCATAATAATAGTAAACATGGTAACATTTCTATTATGTACCGGTTAC
AATGGGCAAATGATATACTCTGTCCAGTTTTTCCAACTTATATTTGCGTCGAGTGCATGGAAAGCCGGTCTTCAC
TTGATACCAATCGTTATTACCAACGTTATTGCGGCCATTGCAAGTGGTGTGATTACCAAAAAGCTCGGTTTAGTT
AAACCACTCTTAATATTTGGAGGCGTTCTTGGGGTAATTGGAGCAGGGCTTATGACACTTATGACAAATACGTCC
ACGAAGTCAACTCAAATTGGTGTTTTGCTATTACCGGGGTTTTCCCTTGGATTTGCTCTACAAGCATCGCTCATG
AGTGCACAGCTTCAAATTACCAAAGATCGTCCAGAAGCTGCTATGGACTTTATTGAAGTAACAGCTTTCAATACA
TTCATGAAGTCATTAGGTACAACTCTTGGTGGTGTGCTTTCAACCACTGTTTTTTCCGCCTCCTTTCACAATAAA
GTATCACGAGCTCATCTAGAGCCTTACGAAGGAAAAACGGTTGATGACATGATTTTGTATCGTCTTCAAAACTAC
GACGGTTCTCATTCGACTATTGGAAACATTTTAAGCGACTCCATTAAGAACGTATTTTGGATGGATCTAGGGTTT
TATGCCTTAGGATTTTTGTTTTGTAGTTTTTCATCCAATAAGAAATTAATCATACCAAAAAAGGACGATACACCA
GAAGATAATTTAGAAGACAAGTAG

>YOL141W 0.71   Cold
ATGAAGAATCTGACCACTATAAAGCAAACGAACAAGAATGTCAAACAAGAAAGACGTAAAAAGTACGCTGATTTG
GCGATTCAAGGCACAAATAACTCCTCAATTGCTTCTAAAAGATCGGTAGAGTTATTGTATTTGCCAAAACTGAGT
TCGGCTAATAATTTCCAAATGGATAAGAATAATAAACTGTTGGAATATTTTAAATTCTTTGTTCCTAAGAAAATT
AAAAGATCTCCCTGTATTAACAGAGGTTATTGGTTGAGGTTATTTGCCATCAGGTCAAGATTGAACTCCATCATT
GAACAAACGCCACAGGATAAAAAGATAGTTGTTGTTAACCTTGGTTGTGGGTACGATCCATTACCATTTCAACTA
TTAGACACCAATAACATACAAAGTCAACAATATCATGACCGAGTTTCCTTTATTGATATTGATTACTCTGATTTA
TTGAAAATTAAAATTGAGTTGATCAAAACTATACCCGAGCTTTCGAAAATTATTGGTCTTTCTGAAGATAAAGAT
TATGTTGATGATAGTAATGTTGATTTTTTGACTACTCCAAAGTATCTCGCCCGACCATGCGACTTGAACGATTCT
AAGATGTTCAGCACATTGCTAAATGAGTGTCAATTATACGATCCAAACGTTGTCAAAGTATTTGTTGCTGAAGTA
TCACTGGCGTATATGAAGCCGGAGCGTTCTGATAGTATAATCGAGGCAACTTCTAAGATGGAGAATAGTCATTTT
ATTATTCTTGAGCAGTTGATTCCAAAGGGACCTTTTGAACCCTTTTCTAAGCAGATGCTAGCTCATTTCAAGAGA
AATGATTCTCCGTTACAGTCTGTATTAAAGTATAATACTATCGAGTCACAGGTTCAACGATTCAATAAGTTAGGT
TTCGCTTACGTTAACGTGGGTGATATGTTTCAACTGTGGGAAAGCGCAGATGAGGCAACCAAGAAGGAACTTTTA
AAGGTAGAGCCTTTTGATGAACTAGAGGAGTTCCATTTATTTTGTCATCATTATGTTTTATGCCACGCTACGAAC
TATAAAGAATTCGCATTTACTCAAGGGTTTTTGTTTGACAGATCAATATCTGAGATAAACCTAACTGTGGATGAA
GACTATCAGCTTCTAGAATGCGAGTGCCCCATCAATAGAAAATTTGGCGATGTAGATGTCGCTGGAAATGATGTG
TTTTACATGGGAGGTAGTAACCCATACAGAGTAAACGAAATATTACAGATGAGTATACACTACGACAAAATAGAC
ATGAAGAATATTGAAGTAAGCAGCAGCGAGGTTCCTGTAGCAAGAATGTGTCACACTTTTACAACTATTTCTAGA
AATAACCAGCTACTGCTTATTGGAGGTAGAAAGGCACCACACCAAGGTCTCTCTGATAATTGGATATTTGATATG
AAAACAAGAGAGTGGTCGATGATCAAGAGTTTATCACACACGAGATTCAGACATAGTGCATGTAGCTTACCGGAT
GGAAACGTCCTGATCCTTGGTGGGGTTACAGAAGGACCTGCCATGCTGCTATACAATGTCACCGAAGAAATCTTC
AAAGACGTAACTCCAAAAGATGAATTCTTCCAAAATTCGCTAGTATCAGCGGGTCTAGAATTTGATCCGGTTTCC
AAACAAGGCATAATATTGGGTGGTGGATTTATGGATCAAACAACTGTCTCGGACAAGGCAATTATTTTCAAATAT
GATGCGGAAAATGCTACTGAACCAATTACGGTGATTAAGAAGCTCCAGCACCCCTTATTTCAACGTTATGGATCC
CAGATAAAATACATTACTCCAAGAAAGCTATTAATAGTCGGTGGAACAAGTCCTTCCGGGCTATTTGACCGGACC
AACTCTATAATCAGCCTTGATCCGCTGAGTGAGACGCTAACATCAATTCCCATATCAAGACGCATATGGGAAGAT
CACTCACTAATGCTTGCTGGATTTAGTTTGGTGTCCACTTCTATGGGCACCATACATATCATTGGAGGCGGTGCC
ACTTGCTACGGCTTTGGATCAGTGACTAATGTAGGTCTTAAGCTCATAGCAATCGCGAAATAA

>YNL330C 0.77   Cold
ATGGTATATGAAGCAACACCTTTTGATCCGATCACGGTCAAGCCAAGCGATAAAAGACGCGTTGCATATTTTTAC
GATGCAGACGTTGGGAACTATGCATATGGAGCAGGTCACCCGATGAAGCCGCATAGAATAAGAATGGCACATTCC
CTTATTATGAATTATGGCTTGTACAAGAAGATGGAAATTTACAGAGCTAAGCCGGCAACGAAACAAGAAATGTGT
CAGTTCCATACTGATGAATACATTGATTTTTTATCGAGGGTTACTCCAGATAATTTAGAAATGTTTAAAAGAGAA
AGTGTCAAGTTTAATGTCGGAGATGATTGTCCTGTCTTTGATGGGCTCTATGAGTACTGTAGCATATCTGGTGGT
GGCTCTATGGAAGGAGCTGCTCGTCTGAATAGAGGCAAATGTGATGTTGCTGTCAACTATGCGGGTGGTTTGCAT
CATGCAAAAAAATCGGAAGCTTCTGGGTTTTGTTATTTAAATGACATAGTACTGGGCATTATTGAGCTACTACGA
TACCACCCCAGAGTTCTGTATATTGATATTGATGTGCACCATGGTGATGGTGTAGAGGAAGCGTTTTATACAACG
GATCGTGTCATGACATGTTCTTTCCACAAATATGGTGAGTTTTTCCCTGGCACAGGTGAACTGAGAGATATAGGG
GTGGGTGCAGGAAAAAACTACGCGGTCAATGTGCCATTAAGAGACGGTATTGACGATGCTACGTATAGATCTGTG
TTTGAACCTGTGATAAAAAAAATTATGGAATGGTATCAACCTTCTGCTGTCGTGTTACAGTGTGGTGGGGACTCC
TTGTCCGGCGATCGTCTTGGTTGCTTTAATCTTTCCATGGAAGGCCATGCTAATTGTGTAAACTATGTGAAATCC
TTTGGGATCCCAATGATGGTTGTTGGTGGAGGAGGCTATACTATGAGAAATGTTGCAAGGACATGGTGCTTTGAA
ACAGGTCTACTAAATAACGTTGTCTTGGATAAAGATTTACCGTACAATGAATATTACGAATATTACGGTCCAGAT
TATAAGTTAAGTGTTAGACCTTCGAATATGTTCAATGTAAATACTCCCGAATATCTTGACAAGGTAATGACCAAT
ATATTTGCTAATTTGGAAAACACAAAGTATGCCCCTAGTGTTCAGTTGAATCACACACCTAGGGATGCCGAAGAT

TTGGGTGATGTTGAAGAAGATTCTGCCGAGGCTAAAGATACGAAGGGTGGTTCGCAATATGCGAGGGACCTACAT
GTTGAGCATGACAATGAATTCTATTGA

>YOR372C 0.79 Cold
ATGGACAGAGATATAAGCTACCAGCAAAATTATACCTCAACTGGGGCAACTGCAACTTCCTCAAGACAGCCCTCT
ACGGACAATAATGCAGATACAAATTTTTTGAAGGTAATGTCAGAATTCAAATATAATTTTAACAGTCCGTTACCT
ACAACGACTCAATTCCCCACGCCCTATTCTTCTAATCAGTATCAACAGACTCAAGATCATTTTGCCAATACAGAC
GCTCACAACAGTTCGAGCAACGAATCGTCGTTGGTAGAGAACAGTATATTACCGCATCATCAGCAGATACAACAG
CAACAACAACAACAACAACAACAACAACAACAACAGCAAGCTCTAGGTTCACTTGTACCTCCTGCTGTCACAAGG
ACAGATACAAGTGAGACTTTGGACGATATCAACGTTCAACCTTCTTCTGTTTTGCAGTTCGGCAACTCTTTACCC
AGCGAATTTTTGGTTGCATCCCCAGAGCAATTCAAAGAATTTTTGTTGGACTCTCCGTCCACCAATTTCAATTTC
TTTCACAAAACTCCGGCAAAGACACCACTTCGATTTGTAACAGATTCTAACGGTGCTCAGCAAAGCACCACAGAG
AACCCAGGTCAACAACAGAATGTTTTTAGCAATGTCGATTTGAACAATCTTTTGAAGAGTAATGGAAAAACACCC
TCATCTTCATGCACCGGCGCATTTTCACGCACTCCTCTGAGTAAGATTGACATGAATCTCATGTTCAATCAACCG
CTGCCGACATCTCCATCAAAAAGGTTCTCCTCCCTGTCGTTGACACCATATGGAAGAAAAATTCTGAATGACGTC
GGTACACCTTATGCAAAAGCATTGATATCGTCTAACAGCGCGTTAGTGGATTTTCAGAAGGCAAGAAAGGATATT
ACCACTAATGCAACATCCATAGGGCTGGAAAATGCCAACAACATCTTACAGAGAACGCCGCTAAGATCTAACAAT
AAAAAATTATTTATTAAAACCCCCCAGGATACCATCAATAGCACTAGCACACTAACTAAGGACAACGAAAATAAA
CAGGACATATACGGCTCTTCACCGACTACCATCCAATTAAATTCATCAATAACTAAATCTATCTCCAAATTGGAT
AACTCTAGAATTCCCTTGTTAGCTTCGAGATCAGATAACATTCTGGATTCCAATGTGGATGACCAATTGTTTGAT
TTGGGGTTGACAAGATTACCTTTATCACCAACACCAAATTGTAATTCTTTGCATAGTACAACCACAGGTACATCT
GCCTTACAAATTCCTGAGCTACCCAAGATGGGGTCTTTTAGAAGTGATACGGGAATCAATCCAATTTCAAGTTCA
AACACAGTTTCTTTTAAGAGCAAATCAGGCAATAATAATTCAAAGGGTCGAATCAAAAAAAAATGGGAAGAAACCT
TCCAAATTTCAAATTATTGTGGCAAATATTGATCAATTTAACCAGGATACATCATCGTCATCTTTATCATCATCA
TTGAATGCAAGTTCGAGTGCAGGGAATTCAAATTCAAACGTAACAAAGAAAAGAGCAAGTAAACTCAAAAGATCA
CAGTCTTTACTTTCTGATTCCGGATCGAAATCACAAGCAAGGAAAAGCTGTAATTCTAAATCTAATGGAAATTTA
TTCAATTCACAGTAA

>YPL214C 0.8Cold
ATGGTATTTACTAAGGAAGAAGTTGATTACTCATTATATCTGGTTACAGATTCTACCATGCTTCCACCGGGAACT
ACTTTATGTTCTCAGGTTGAAGCTGGGTTGAAAAATGGTGTAACGCTAGTTCAAATCCGTGAAAAGGATATTGAA
ACAAAAAATTTCGTTGCAGAGGCTTTAGAAGTTCAAAAAATATGTAAGAAGTACAATGTTCCACTTATTATCAAT
GACCGTATAGACGTCGCTATGGCAATTGACGCCGATGGGGTTCATGTGGGCCAGGACGATATGCCAATCCCAATG
GTAAGAAAACTTTTGGGCCCTTCTAAAATTCTTGGATGGAGTGTTGGTAAACCTTCGGAGGTAGAGACATTGGCT
AAATGGGGGCCAGATATGGTAGATTATATTGGTGTTGGTACTCTTTTCCCTACATCAACAAAGAAAAATCCTAAG
AAATCACCCATGGGTCCTCAAGGCGCCATTGCCATTTTGGATGCTTTGGAGGAATTTAAAGCTACATGGTGTAGA
ACCGTTGGTATTGGTGGTCTTCATCCTGACAATATTCAACGTGTGCTTTGTCAATGTGTTGCTTCAAATGGAAAG
AGATCGTTGGATGGTATCTCTCTAGTCAGCGATATTATGGCCGCTCCAGATGCATGTGCAGCAACCAAGAGGTTG
AGAGGCTTGTTAGATGCTACCAGATACCAATTTGTTGAGTGCGAGTTAAATAATACATTTCCGACCACAACTTCA
ATTCAAAACGTTATATCTCAAGTATCAAACAATCGTCCATTAGTGCAACATATCACCAATAAGGTTCATCAAAAT
TTTGGTGCCAATGTCACTCTAGCTTTAGGCTCATCTCCTATCATGTCTGAAATTGAAAGTGAAGTATCTGAACTA
GCAAGAATCCCAAATGCTTCTTTACTGTTAAATACCGGATCAGTGGCACCTATTGAAATGCTAAAAGCCGCAATT
AATGCTTATAATGAAGTAAATAGACCCATCACCTTTGACCCGGTCGGGTACAGCGCCACTGAAACAAGGCTCTGT
CTAAACAACACTTTGCTCACTTACGGCCAATTTGCTTGTATAAAGGGCAATTGCAGTGAAATACTGTCTCTAGCC
AAGTTAAATAACCATAAAATGAAGGGCGTCGACTCTAGCAGCGGCAAAACGAACATCGACACACTTGTGCGCGCT
ACACAAATTGTGGCATTCCAATACAGGACTGTTGCTGTTTGCACGGGTGAGTTTGATTGCGTTGCTGATGGTACT
TTTGGAGGCGAATACAAACTTTCATCAGGAACAGAAGGAATAACCGCTGAAGATCTTCCATGTGTAATAATTGAA
GATGGCCCAATTCCTATCATGGGTGACATCACTGCAAGTGGATGTTCACTTGGTTCTACAATTGCTTCCTTTATC
GGTGGGTTAGATTCCACTGGAAAACTGTTTGATGCTGTGGTTGGTGCTGTCCTATTATACAAATCAGCTGGTAAG
TTGGCTTCCACCCGTTGTCAAGGAAGTGGTTCATTCCATGTCGAATTGATTGACGCATTATATCAATTATTCCAT
GAAAATAAACCAGAAAAGTGGTCCGCTTCTTTGAAGAAATTCAAATAA

>YGL228W 0.81 Cold
ATGGGAAAGCTGATAAAGTTAATCACAACTCTGACGGTGTTGGTATCGTTGCTTCAGTACTGTTGCGAATTCAAT
AGTGGGAGCATATCGTGTGAACGCACGCAAACGCTTTGCCATTATACCAATCCAAGAGTCTGGAACACCTACTTT
TCAAGAAATTGTGAATTATACAAAAATAAAGTTAGTCCCGGTTTCGATATAGTGGCGCGTAAATATGACACGGCT
GTCAAACCTGTCATCGATGACGCTACAGTAAAAGTGAACAAGGTCGCTATACAGCCTGCATTCAAGGTCATCCAT
TCACAATGTAAGAAATGGAACTGTGGAAAGTATTACCAATTGGTCCGCTCCCCAATGGTCAAGACTAGACGATTT
TTCTTCGCAAAATACAATGCTTTTGTGAAACCTAACTTAGACAAATTCTTTACTGCGGAGTTTCGCTCTCATTTG
AAGGAAAGAATTTTAAAGTATAAAAATATAGGCCATTATTACTTTACTATCACTTCAAGATGCATCAAATCCAAA
TACGACTTTATTGTTGGTAATACAGAGGAAAAATTAATGGGAAAGTTCAAGAATAAGGACACGCATGGCATTCAT
GGTTCTGTCACACGTGAGCCTTCCTCGGAGGACATGGTTTTAACCGTAAGTACTATGGAGTCTGACGAAGAGGAG
CTAACTACAACAAGTACTCAAACAGTGGTTGAAACAATTACTTTGGACCAGGAAGAGGCTAGCGCTGTTGCTAAT
CATGCGCACGACGATGAGGCTTCCACAGACGTTGAAGGCTCCACAGACGTCAATGTCAATGAACAGGCTCTGTTG
CAAGAGGATTTTGACATGTGGAGTGAGACTATATTGCAGAAAACACAAGACGTCATACAGCTATTTGAAAAGGAT
GTTTCCAAATACATCAATGGTAAATTGGTTGAGGAGGCCAACCATTTTAAAGCCAAGTTTCAATCTTTAGATGAT
AAATCTAAGAAGTTCTTCTCCAAGATTTCGCTAGCCATTAACGATATTGAATGTGTGGAAGGCATTGATAGTGAA

ACTGGCAAGAAAATTTTCTTCGATAAATCTGGAAGTACTGAGATTTCTCAGTACATAACGAGAGAGTTAGTTCGT
GAGTACTTCAACGAGACGCGCTCGACGTTAGACGAACTGACAAATGCTATGGAGAAAGATTTGAGCGAAATCACT
GATGAAATTGAAAAAAAAGTTAATGCAATTAGAGAGGAAAACGTCGAGGTATTTGAAGAATGGGGTGATATCATT
GTAAATGAATGGTCCAAAAGAATGGCTTATGTGGATGTTATCAACGCTCACATGGGTGCAGATGATGACACTACG
CTTGATGAAGAGAAAGCAAAGTCTTCTGTTAACTGGAAGAAGTTTTTGAAGGGCAAGAAACAGATCATAGAATCT
AGAGACAAGCTGGCTCACCATTCCGCTGACTTATCCCGTGTTAACGCGTTTAGGCAAAAAGTGCAAAAGAAGATA
TTATCGTTCACTCAAGAGAGTGGTGAGTTTTTGTATATTCTAAGATCGAAAGCCAACCTGCAATTTCAGGAACGT
GAAAGAAAGGAACGTGAAAGAAAAGAGCGTGAAAAAGCTGCAGCAGAAGAGTTCCAAAGGCAGCAGGAGCTATTA
CGACAACAGGAAGAAGAAGACGAAGAAGACGTATCCTACACCTCTACTTCGACCATCACCACAACCATTACCATG
ACATTGTGA

>YGL111W 0.79   Cold
ATGAGGTTACTAGTCAGCTGTGTGGATAGTGGATCCATAAAGGAAGTTTTGTGTAACATCGGGACTGACACTTCC
GTACAATCAGCACTGCAGCCTTTTCATGTGGCACCTCATCTAGCCGAAGGATTGAAAGCTTACGTTGACAGGATG
TGGGTGATATCCGAAGATGAAGCCATCTTAGCAAGGAATTCAGGGGTCGTCGAACTTGTAAAGATTTCGAAACAC
CTTAAAGAAAATGAAGCTCTCCAGGTCGATCCCAAAGGAGAAAGCAAAAATGAAAAGAGCCTTTCCGATGATCTA
CCCAAATTTGACATATCCGAGTTTGAGATAACCAGCTCAGTTTCAGACCTTTTTGATGATGCCAAATTGGAATCA
CTTTCAAGTAAATCTGTTAAAAGAACCAAGTTGGTGGATGGTTTTGTCACATTATGTCCCATAAAAAAGGATTCG
TCGAATAACACATTCGTAGCTGCTACCAAATCGGGATTATTACATATTATAAAAAAAGGAGAAGATAAAAAGTTA
ATAAAACTAGCATCCCTTGGACTGAAAGCACCAGTAGAATTTCTTCAGTTATATGATCTAGAAGATACCGATACT
GATAAATATATATTTGCATACGGAGGGGAGGAGAATTTAATAAAATTAGTGGAAATAGATTCGAGTTTCCAATCT
TTAAAGCAAATTTGGGAAGCCAAGAATGTTAAAAACGACAGGTTGGATATGAGAGTCCCAGTATGGCCCATGGCC
CTAAGATTTTTGGAGCCCTCCCCTGGTAAAACTGAAAAGGGTAAGCTGAACTACCAGTTTGCAGCCATAACCCGT
TGGTCCCACCTCACCAAATACAGCACACAACATGGCAGAAAACCGTTCGCTCAGATAGATTTATTACCTAACCGT
GAACCATTATCTCAGATGGAAGTTTTTGATGCCAAGGGAGAAAATGTTGTCTCTTCGTTGGGGAATTTTCAATCT
GAAACCTTTAATGAACTGAATGTGATTACTACTGACTACAAAAAGAATGTTTTTAAATTCGATGGTAATGGAAGA
ATGTTGGGCAAGGTGGGTAGAGATGATATCACTGGTTCGTCAACCTATATTCATGTACATGACGGCAAATATCTT
TTACAAGGTGGTTTAGATAGGTATGTTCGGATCTTCGATATAAAAACTAACAAAATGCTGGTGAAGGTTTATGTT
GGGTCCCGTATAAATTTTATTGTTATGCTGGATGACGTTGAAATCGAGATGCCACTAAGTCCAAGTGCGAAAGCT
GCCAAAGGGAAGCAAAAAAGGAAGGTTACAGAACTTGAAGAAGACGCAGATGAGCTTTGGAATAAATTGGAGGGA
AAGGTAGCTGCTTCTAAAGCCAGTAAGAAAAGCAAAATTTAA

>YKL078W 0.74   Cold
ATGGCAGCAAATAGCAATAGTAGAGTTGCATCAAACCATACTTCTAAAAAACAAAAGGTTAGGAGGAATATACAT
CCATTCACGAATAACACTCGGATTAAAAGAGCTAGTAAAATCGTAAAGTTTAATGATTCAGGCGAGGGCGACCAT
GTTTCAGACCAACGTTCGAACAAGGAAAATGTCCTTACTTATAAATCTTTGAAAAGTCGGGCAAGCGATTTACTC
AAAATGAGAGAAACACTTCCCGTTTACCAACACAAGCGAGAAATAATGTCATATATTGAAAGCAATCCCGTTACC
GTCCTTATTGGTGAAACAGGTTCTGGTAAATCAACACAAATTCCGCAATTCGTATTAGAAAAATTATATGATACG
AAGAAGCATGGGTCGATTGCTGTAACTCAACCCCGTCGTGTTGCGGCCATCAATTTGGCTACACGTGTTGCTCAA
GAACATGGTTGCAAACTGGGTGAACAAGTAGGTTATTCTGTCAGATTTGACAATACGACTACTACAAGAACAAGA
CTTAAGTATCTGACTGACGGTATGTTACTTAGAGAACTTATGATGAATAGTGACCTTAGAGAATACAGCGTCATC
GTTATCGATGAAGCGCATGAAAGAACTGTGCTTACAGATTTGATATTGGGGTTCTTAAAATCCTTGATACAAGGG
CCAAGACCTGATTTGAGAATAATTGTTATGTCTGCAACATTGCAAGCCGAAAAATTTAGTGAGTTCTTCAATAAT
GCCCCAATCTTATTTGTAGAAGGAAGAAAATTTGATGTCAAACAATACTACTTGAAGGCGCCAACAGATGATATA
GTAGACGCTGTCATCAGGTGTTGTATACAAATAAACCAAGGTGAAGAACTGGGAGATATCTTATGTTTTTTACCG
GGCCAAGAAGAGATTGACAAAGCAGTGACTATAATGGAGAAAATTGCAAAGTATGTTTCAGATGAGGCCCCAGTA
CCATTAATAGTTCCTTATCCTTTATATGCAGCTCTTCCCGCGGTCCAACAGTCCTTGGTCTTCGCTCCGATAAAG
GGTTTCAAGAGAAAAGTCGTCTTTAGTACCAATATTGCAGAAACATCTGTTACCATATCCGGTGTTAAATTTGTC
GTTGATTCTGGTCTTCGAAAAGTCAAAGTTTGGAGACATCAGCTGGGATTAGCCACTCTACTTACCGTACCCATT
TCTCAGGCAAGTGCGATGCAGAGAAGTGGTCGTGCTGGTAGAGAAAGTGAAGGAAAGAGTTTCAGGCTTTATTGT
GAGTCTGATTATGTGAAATTACCTAAACAAAGTGAACCTGAAATAGCCAGAAGTGACGTCACATCTCCTGTGTTA
ATGTTGAAGAGATATGGTGTTGATGACCTGCTAAACTGGACCTGGTTTGAGAACCCTGGAAAGGAAGCTATAGTA
ATGGGGCTTCAGGAACTTTATGAATTGGGTGCTCTTGACACTCGTGGAAAGATAACTAAACGGGGTCAACAAATG
GCTCTGTTACCGCTACAACCGCATTTAAGTAGTGTCTTAATTAAAGCCAGTGAAGTCGGATGTTTGAGTCAGGTC
ATTGATATCGTCTCTTGCCTTAGTGTGGAAAATTTACTGTTGAATCCGTCACCAGAAGAAAGAGATGAGGTGAAC
GAGCGTCGTTTGTCCTTATGCAACGCTGGTAAAAGGTATGGTGACCTTATCATGCTGAAAGAGCTTTTTGATATC
TATTTCTACGAACTAGGGAAAAGTCAAGATGCAAGCTCTGAAAGAAATGATTGGTGTAAAGGATTGTGTATTTCG
ATACGTGGGTTTAAAAATGTAATTCGTGTCAGAGACCAGTTAAGAGTTTATTGTAAGCGTTTGTTTTCTTCAATC
AGTGAAGAGGATGAAGAATCCAAAAAGATTGGTGAAGATGGCGAGCTAATTTCGAAAATTTTAAAGTGTTTCTTA
ACTGGGTTTATCAAGAATACAGCTATAGGGATGCCAGACAGGTCTTATAGAACTGTTTCCACTGGAGAGCCGATA
AGCATTCATCCATCATCTATGCTATTTATGAATAAAAGCTGCCCCGGTATAATGTACACGGAGTATGTCTTTACT
ACGAAGGGATATGCCAGAAATGTTAGTAGGATTGAACTTTCATGGTTACAAGAAGTTGTCACTAATGCAGCCGCT
GTAGCAAAGCAAAAAGTTTCTGATTCAAAATAA

>YKL207W 0.74   Cold
ATGTTATTAGATGACCAGCTGAAGTATTGGGTCCTGTTACCTATTTCGATTGTCATGGTTTTGACGGGTGTACTC
AAACAGTACATCATGACACTTATAACGGGAAGCAGTGCAAATGAAGCACAACCAAGGGTAAAGTTAACTGAATGG

CAATATCTACAATGGGCACAATTATTGATTGGAAACGGTGGGAACCTGTCTTCCGATGCATTTGCTGCGAAGAAG
GAATTTCTAGTTAAGGATCTCACTGAGGAAAGGCACCTAGCCAAGGCCAAGCAGCAGGATGGTTCGCAAGCTGGA
GAGGTTCCTAATCCCTTCAACGATCCAAGTATGTCTAATGCTATGATGAACATGGCTAAGGGTAATATGGCCAGT
TTCATCCCTCAAACTATTATTATGTGGTGGGTGAACCATTTTTTTGCTGGATTTATTCTTATGCAACTACCTTTC
CCTTTGACTGCCAAATTCAAAGAGATGTTACAGACCGGTATTATCTGTCAGGACCTGGATGTGAGATGGGTTAGC
TCCATTTCATGGTATTTTATTTCTGTGCTCGGGCTAAATCCAGTCTACAACCTGATTGGGTTAAACGATCAGGAC
ATGGGTATTCAGGCCGGGATAGGTGGCCCTCAGGGCCCCCAAGGCCCTCCACAATCACAGGTTGACAAAGCAATG
CATGCGATGGCTAACGATTTGACTATCATTCAGCATGAGACTTGTCTTGATAACGTTGAGCAAAGGGTTTTGAAA
CAGTATATGTAA

>YIL028W 0.79   Cold
ATGGACGATGCAAGGTTATTCAGACGCGTTGTTTGTATGCCATTTCCAATGATAATGTTGGTTGCGTTATATAGA
AATGTGTTAGGAAGAGATTCTACAGTTGATTGCAGCTTTGACGTTATACTTACACCTTGGATAATGGCCAGTATG
TTTTCCAAACGTATGGTGAATTGGTCTAATCCACGTCAAGCTGTTTTAAAAGTATGCAGCGCAGCCGTGACCTCC
CGGATAGTTGCCAATTGGTTGTTTACGATGAACATATTATATACCAGTGGTGAAAAGGGTTTTTACTCGCTAGCA
ACAGCTGTGGTAATTAAAACGGTCACTGATGGGTTTACGGTGATACGGGTTTTCTCTATTCACCTGAACGAGTCA
GTATATAGCAAGCCATTACCATAA

>YBR294W 0.71   Cold
ATGTCACGTAAGAGCTCGACTGAATATGTGCATAATCAGGAGGATGCTGATATCGAAGTATTTGAATCAGAATAC
CGCACATATAGGGAATCTGAGGCGGCAGAAAACAGAGACGGACTTCACAATGGTGATGAGGAAAATTGGAAGGTT
AATAGTAGTAAGCAGAAATTTGGGGTAACGAAAAATGAGCTATCAGATGTCCTGTACGATTCCATTCCAGCGTAT
GAAGAGAGCACAGTCACTTTGAAGGAGTACTATGATCATTCTATCAAAAACAATCTAACTGCGAAATCGGCAGGA
AGTTACCTCGTATCTCTTTTTCCTATTATAAAATGGTTTCCTCATTATAACTTTACGTGGGGCTATGCTGATTTA
GTGGCAGGAATTACAGTTGGCTGCGTACTCGTGCCCCAATCTATGTCATACGCACAAATCGCTAGTTTATCTCCT
GAATATGGTTTGTATTCCTCCTTTATTGGTGCGTTTATATATTCTTTGTTTGCCACATCGAAAGATGTTTGTATT
GGTCCGGTCGCTGTAATGTCACTACAAACTGCCAAAGTCATTGCTGAAGTTCTAAAAAAATATCCCGAAGACCAG
ACAGAAGTTACAGCTCCTATCATTGCAACTACCCTTTGTTTGCTTTGTGGGATTGTCGCCACTGGGTTGGGTATA
CTGCGTTTAGGCTTTTTAGTGGAACTTATTTCTCTAAATGCTGTTGCTGGCTTCATGACCGGTTCCGCATTTAAC
ATCATCTGGGGTCAAATTCCGGCTCTCATGGGATACAACTCATTAGTGAATACCAGAGAAGCAACGTATAAGGTT
GTAATTAACACTCTGAAACATTTACCAAACACAAAGTTAGACGCCGTTTTTGGCTTGATTCCGTTGGTAATCCTC
TATGTATGGAAATGGTGGTGTGGTACATTTGGTATAACTTTGGCAGATAGATATTATCGAAATCAACCAAAGGTA
GCAAATAGACTGAAATCCTTCTATTTCTATGCACAAGCTATGAGAAATGCCGTCGTCATAGTAGTTTTTACTGCC
ATATCGTGGAGCATAACAAGAAACAAATCTTCAAAAGACCGTCCAATCAGTATTCTGGGTACAGTTCCCTCGGGC
TTAAATGAGGTGGGAGTTATGAAAATCCCAGACGGTCTGCTATCTAATATGAGTTCAGAAATACCTGCTTCAATT
ATCGTTCTGGTGTTAGAACACATCGCTATTTCAAAATCCTTTGGTAGAATTAACGACTACAAGGTTGTCCCTGAC
CAAGAACTTATTGCGATTGGTGTGACAAATTTGATAGGGACATTTTTTCACTCATATCCAGCAACTGGGTCATTT
TCCAGATCTGCTTTGAAAGCAAAATGTAACGTGCGCACTCCGTTTTCTGGGGTATTCACTGGCGGTTGCGTTCTA
TTAGCACTTTATTGTTTAACTGACGCCTTCTTTTTCATTCCTAAAGCGACACTATCGGCGGTTATTATTCATGCT
GTTTCTGATTTGCTGACTTCTTACAAAACCACCTGGACCTTCTGGAAGACCAACCCGTTAGATTGTATCTCATTT
ATCGTTACAGTGTTCATCACAGTATTTTCATCCATTGAAAATGGTATATATTTTGCAATGTGTTGGTCATGTGCA
ATGTTACTATTGAAACAGGCTTTCCCTGCTGGTAAATTCCTTGGTCGTGTTGAGGTGGCAGAAGTATTGAACCCA
ACAGTACAAGAGGATATTGATGCTGTGATATCATCTAATGAATTACCTAATGAACTGAATAAACAGGTTAAGTCT
ACTGTTGAGGTTTTACCAGCCCCAGAGTATAAGTTTAGCGTAAAGTGGGTTCCGTTCGATCATGGATACTCAAGA
GAATTGAATATCAATACCACAGTTCGGCCTCCTCCACCAGGTGTCATAGTCTATCGTTTGGGTGATAGCTTTACT
TACGTGAACTGCTCAAGGCATTATGACATTATATTTGATCGTATTAAGGAAGAAACAAGGCGAGGCCAACTTATA
ACCTTAAGGAAAAAGTCAGACCGTCCATGGAATGATCCTGGTGAATGGAAAATGCCAGATTCTTTGAAATCACTA
TTTAAATTTAAACGTCATTCAGCAACAACGAATAGTGACCTACCGATATCGAATGGAAGCAGTAACGGAGAAACA
TATGAAAAGCCGCTACTGAAAGTCGTCTGCCTGGATTTTTCCCAAGTTGCTCAAGTGGATTCAACCGCTGTTCAA
AGCCTGGTTGATCTGAGAAAAGCTGTGAATAGGTATGCGGATAGACAAGTCGAATTCCATTTTGCCGGAATTATA
TCTCCATGGATCAAAAGAAGTCTTTTGAGTGTTAAATTCGGAACTACAAATGAGGAATATAGTGACGACTCTATT
ATCGCTGGCCATTCTAGTTTTCACGTTGCAAAAGTTTTGAAGGATGATGTGGATTATACTGATGAAGACAGCCGT
ATAAGCACATCTTACAGTAACTATGAAACATTATGTGCTGCAACTGGGACAAATTTACCGTTTTTTCATATCGAT
ATACCCGATTTTTCTAAATGGGACGTTTAG

>YER060W 0.79   Cold
ATGCCTCAAACGCACGAAATGTCCCTGAACGGCACTCAATACTTAAAATATGAACTTAAAGACCTTGAGTCTCGA
GCTCACGATGCAAAGACTCCTTCAACAAATGAATTCTATGATGATGTAGAATCGCACGGAACAGAAGAATTAGTT
GAAGCTAAACTGTCGTTTTTGAACAGAATTGCTGCTGGTTTAAGTGCCGAGACAAAGGGTATTGAACCGATTACA
GAAGATGAAAAAACTGATGATTCTATACTGAACGCTGCATCTATGTGGTTTTCAGCAAATATGGTTCTGCCTGCT
TATGCCATTGGCGCCCTAGGGCCTATGGTGTTTGATCTAAATTTCGGCCAAAGCGTTTTCGTTATTATTTTCTTT
AACCTCTTAGGCTTGGTATCAGTTGCTTTCTTTTCTGTTTTCGGTGCAGAGCTGGGCCTAAGACAGATGATTTTG
TCTAGATATTTAGTTGGCAATATCGCAGCCAGGATCTTCTCTTTCATCAATTTTATTGCCTGTATCGGTTGGGGT
ATCGTTAATACCGTAGCAAGCTCGCAAGTCCTGAATATGGTTAACCCCGGTCATCAATGTCCTCTCTGGGCTGGT
TGTATAGTTATTATCGGTGCTACTGTAATTGTAACTTTTTTCGGCTATGGAGTCATTCATGCTTACGAGAAATGG
GCGTGGGTACCAAATTTTGCCGTTTTTTTGGTAATAATTGCTCGTTTGGCAAGGTCTAAAAAATTTGTCCTTGGG
GAGTGGACATCTGGTCCTACTACAGCAGGTAACGTGCTTTCATTTGGATCAACAGTTTATGGGTTTGCTGCAGGC

TGGACAACATATGCGGCTGACTATACCGTTTATATGCCAAGGAAAACGAACAAGTATAAAATTTTTTTCTCGCTT
GTAGTTGGGTTAGCAACACCATTATATTTACTATGATACTTGGTGCAGCTGTAGCAATGGCAGCTATCGGCGAT
CCAGCCTGGAAGACGTATTATGACGAAAATTCCATAGGTGGTTTAACTTTTGCCGTTCTTGTCCCTAATTCTGTT
CACGGATTCGGTCAGTTCTGTTGCGTGTTATTGTCTCTGTCCACTATCGCTAATAATGTTCCCAACATGTATACT
ATTGCTTTATCGGTGCAAGCCACGTGGGAACCTCTTGCGAAAGTCCCAAGAGTTATTTGGACTTTATTAGGCAAT
GCGGCCGCACTGGGTATTGCCATTCCTGCCTGCTACTATTTTTCTACCTTCATGAATTACTTCATGGATTCCATA
GGTTATTATTTGGCTATTTATATTGCCATTGCATGTTCAGAGCATTTTATTTATAGGCGTTCCTTCAGTGCTTAC
AATGTTGATGATTGGGATAGTTGGGAACGTCTACCTATCGGCATTGCAGGTACTGCTGCCTTAATTGTTGGTGCC
TTTGGCGTAGCGTTGGGTATGTGCCAAACTTATTGGGTTGGCGAGATCAGCCGTTTGATCGGAGACTACGGTGGT
GATATTGGATTCGAGTTAGGACTAAGTTGGGCATTTATAGTTTACAACATTGCTAGACCCTTTGAGCTCAAGTAC
TTTGGTCGTTAA

>YGR134W 0.79 Cold
ATGACGAAAAAGAAAGCAGCTACTAATTATGCTGAAAGGCAAAATTTAGCCAGTGAAGATTCTTCAGGTGATTCG
GTTCATTTTAAGGACTTTATTCCCTTACAAGAGTTACTGAAAGATAAAAATTATGTTCCATCGGTGGAAAATTTG
GAGAAGATCTTATATAATGAAACCATGTTTAATGATCAGAAAATATGCTCTAACCTATTGCTTGAGGCTCTCATA
ATTACGTTGTTCACAACAATTTCAGGGAAGTCAGCTTTACGATTAATTCAGACTTCTTCTTTGAAGGAAAGAAAA
TCCTGGGCCCAGTCCTTTGAAAATAACAGTTCTAGCTATGCTTCCATAGTACTGAGTTGGAAAGATAATGATATA
TTACTCCTGAAATTCTTAAGGTTTTTATTAGCAAATAAAACGGCCCCTCTTCAAATTAATCGGTACAATCTTCCA
GAATACAAACTCCCGTTGAGCTTTTTAATTGTTTCAAAAATTACTATTCCGTCCATATTACTCAATGAAACGTAT
AATTTGTTAAAGGACTATTTGTATTCGATAACTGGCCGTATAGAGAGTTTGATAAGCTGCAGCTCCACATTTGAT
AAGCCAGCACTTGTCGTTAGAAAAATTTTAAAAGATTACAATAGAATGATAGAATGCCGAAATTTTTATTTCTGG
TATTCCTTTAATGCTGAGAACAGAGTAAATCTTACTTTTAGTGATAACATTAGCCTGTTAATGGAAAATGATGAA
GGCAATGCTGGAAGTGGCCTTGACGATAGCCGTTTTGATCATCAAAAACAACCGAGAGAGGCGATAATGGGCCGA
ACAATAAATGACCAGGAACAAATATATTCATTCGAACTGAACCAAGATGGAACGCTTGAAATACCCAACGTTATG
GAGCATTCTTTACTAAGACACGAGCTTCTTTTTAAAATATTAAATCTCACTACGGTATTGACGCCATTATTGGAA
CTACAATTTTCTACGCTATGTGGCTTAGTAGATCCATTAATGCAGCCTACTCCCAATGATAAGCATATTATATCG
ATAGACTTTCTTTTTCAGCTTTTTTTGGGATTAATGTCTCAGTCGATCAAGACTTCTCAAGAACACAACGACCAT
TATGACTGGAAATTTTATATGTGTTTTAACATGCAAAAAATTATTGATGCTACCATGTTGAGGCTCAACTGCTTT
GATTTTGATATATTAAACTCGGTAAACAATACAGATAATGCAGTTCATTGGAAGACGCAACTTCACAGATGGCTA
CCGCATGGCCTGAATACACAAGACTTGGAATTACTCTATATGATTGATATACTGGCGGTATATACCATCTACAAG
TTGTATGAAAAAAATACCTATACAACTAAACCCATTTTTATTTTCATTAATATCCTTATGGAAAAACCTCTCCTGT
GTTATACTTTTAGCCTTAGAAATTGATAGAATTGAAGAGGAGAATGGCACTTACGAAACGCCGCTTATGGTTCGC
GCCACAATTCGTGGAGCCGCAGCATTGAGGTCTGTAATAGCTACCGTTCTCAATGGATTAGTGAAAAATAATGAT
CATGATTTCAAGCACGAGTCTTTAAACACATTCATGTCGCCCTATGGAAGAAAATTGTGTCATGGTGCTTTATAT
GCCGATTTAAGGTCACATACTGCATCTCTACTTGCGCTTGGAGCAAGCATCGAAGATGTAACAGATTTATTTGCC
GATTTACAGTCAGGTGATAGATTTGATGAGGATATTAGATATATGTTTGATTATGAATGTGAAGATTATGATGAA
TCTTTCTCTGAAAGCGATCATGGAGGATTGGATGAAAGCGTTGTTAACCCAACAGAAAAAATAGCATCTGGTAGT
AATAATGTTTTCTTTCGGAGACGGTGCAATTGCATCTTCAATGATGATAAGTTGGTGGCAGAGGATGGTGCAAAT
GAAGCCTTTGGGTCAACAAATAGCGAAAACGTTGAAGGCGCAATGCATAATAATAGGAATGCGGTACATAACGCG
ACTACAGCCACTTCTGATCACGTCGTTACGTCACCTAACCCACTTTCCGTTAGATCAAGAAGTACTTTTGAATTT
GACTATAGTGGGGAAGATTGGAGGGACGTTCCCAGAGATTTCAATATGTACTATTCGCCATCTTATTCATTCATC
CATGAGCCTAAGTTAGATGTCATATTTAGTTTGACTCTACGTGGCGCAACAGAAAAGTTGAATAAGGAGGAATCC
ATATTGTTAGTGCGTTCAGTTGCATCGTGTGTAAGGAACGAGCAGGACCAAATGATTTTAGCAGACCTGGAGTCT
AATTTTTCCGCAAGTATAAACGGCGATGTAGAAGGTGAAGGAAATACCAAAATGTCCAAGATAGATAATGAAGAT
CTCAGAAGAACCACGCCGGATGATATTTATGAAATATGGTCTGAAGAATCAGCATTTGAAAGGATGCTAAACGTG
AATCACGACGTTGCGTGGAGATTAATGGATGAAATGTTAATGTGCACCGGTTATAGGAGAATATTGATATGGTTC
CTTACGCATTTGGAGTTAAAACATTCGTTGATATATTACGTTTTTGAACTAATAATGGGGTTACGTGGAAAACCG
TTTTCCGGAGAAGCCAGTGATCAAGATAAAAAGGATGATATGATATATGAGATCTTGAAGAAAAAGCAGAAGAAT
GAAGATGCTTCAGGACTTCCCTTTTCAAGGCAAGGACCTATCGTATTATCTGATATTGAGACTAAAATGTTACTG
CAGGAGTTTTTTATGAATGCTGCTATTTTTTTTGTCATCAAAAAATAACGAAGAAGAGAATGAAGACGGCGAGAAA
ATCTCCTTGTACTCCCTTGGTTTGGTTAGGTTGATCTGTTACATGGTGCAGACACTCATCGCGAACGACAAGTTT
TTTTTTACCAAATCAGAATGCACTTTTGAGCTTCAAACTTTACTGATGACATGGATTGGCATCCTTCCTGAAGCC
AAAGATTTATTCTTTAAGATCAAGACAAGGCTCGCCATGGAAGAAGAGGATAGTGCCGATACTATGCAGCATGAG
GGCAGGAAGAACTCTGACATAGAGAAGAAGCTAAACGCTAAGCCAGCTTCTGAACTGAATCTGAAATTGTTAAAC
CTGTTTCCCTCAAAGCCTGCAAACAAAGACGATAGTTCCCCTATTAACACGTTGCGTAGTTTTATCGCTGATTAC
TCCTTCGACACCCAGGTGAACCCTCCAGGAAGAAGGGTGGTGTTCTACGATGGTAAGATTTTGCCATTGCCCAAA
GCCGATAAGCCTATCCCACTTCATGAATATATAACACTCGCAGAGCTCGATGTTGGAGACAGTGAGTGA

>YGL156W 0.82 Cold
ATGTCATCTGAGGATATCATTTATGATCCTCAGTTCAAACCTGTTCAGGGAATCTACGAGAATAGATTAAGACAA
TTTATTGATACTGGTGGGGATTACCATGACTTAAATTTACCCAAGTTTTATGATAAGAAGCGCATTTCATTAGAT
CATGATCACGTTAAGGTTTGGTGGTACCAGGTTTCTTTTGAACGTGGATCTTCCCCTGTTTCCCCTGATAAGAGG
CCATCTTGGAAATCTATTATTGAACGTGACAAGAAGGGTGAATTGGAGTTTAGAGAAGCGAACATAAATCAGCCC
TTTGGACCCAGTTGGTCTACCACTTGGTTCAAGGTGAAAATCTCTTTGCCTGAAGATTGGGTTAAATCAAACGAG
CAACTACTTTTCCAATGGGATTGTTCTAACGAAGGAATTGTCATAGACCCAAAGACTCTAATCCCTGTGACTGCT
TTTTCAGGTGGTGAAAGGACTGAATATGTTTTACCGAAAACATCGGATGGGAAACATTTCTTTTACATCGAAGCT

GGTAATAATGGTATGTTTGGCTGCGGTGCAGGATCCACTATAAATCCACCAGATGATAATAGATTTTTTCATTTG
AGAAAAGCTGATATCGTTTGGCCCGATCTAGATGCTCGTGCATTGTATATCGATTTTTGGATGCTAGGGGATGCT
GCAAGAGAGCTACCTGGAGACTCCTGGCAAAAACACCAGGCAAGACAACTAGGCAATGCAGTTATGAATCTTTTT
GACCCGAATGATCGTTCAAGTGTTCGTAAATGTCGTGAATTACTACAAAGGGAATACTTTGACTCCTTTTTAGAA
AGCAGTAAGGTTTACGAACAAGGTGAATCTCAAGTTTTAACAAATGTTTATGGTATCGGCAACTGTCATATAGAT
ACAGCTTGGCTATGGCCATTCGCAGAAACAAGAAGGAAAATTGTGAGATCTTGGTCTTCCCAATGTACTTTAATG
GATCGCTTCCCAGAGTATAAATTTGTTGCTTCACAAGCCCAACAATTTAAATGGTTATTAGAAGATCATCCCGAA
TTTTTCAATAAAGTGTTAATTCCAAAAATTCAGCAATCTCAGTTTTTTGCTGTTGGTGGTACATGGGTTGAGAAT
GATACCAATATTCCCTCAGGGGAATCGCTGGCAAGGCAATTCTTTTTTGGTCAAAGGTTTTTCTTGAAGCATTTT
GGTCTCAAGTCAAAGATATTTTGGTTACCTGACACCTTTGGTTATTCTTCGCAAATGCCACAACTTTGCCGCTTA
TCTGGTATTGATAAATTTTTGACTCAAAAGCTTTCCTGGAATAACATCAACAGTTTTCCGCATAGCACGTTTAAC
TGGGCAGGGATTGACGGTTCTCAATTATTGACTCACATGCCTCCTGGTAATACGTATACAGCAGATTCACACTTC
GGTGATGTTTTGCGTACTGCCAAGCAGAATAAGACACCAGAATATTACGGCTCCGGTTTAATGTTGTACGGAAAG
GGTGATGGTGGTGGAGGACCAACTGAGGAAATGCTGCAAAAAATGAGGCGTATTAGATCCATGAATAACAGAAAT
GGTAATGTTATTCCAAAGTTACAAGTTGGTATAACGGTTGATGAATTTTATGATGACATTTTGAAAAGGACTAAC
CAAGGCCATGATTTACCCACATGGAGTGGTGAATTGTATTTCGAATTTCATAGGGGGACATACACCAGTCAAGCT
CAGACCAAAAAATTAATGAGGTTGTCCGAAATTAAACTACATGATTTGGAATGGATAGCTGCCAAAACATCAGTT
TTATATCCCGATTCGTATAAATACCCTTCCAAACAGATTAACGAACTTTGGGAGAATGTTTTGTTATGTCAATTT
CACGATGTTCTACCAGGATCATGTATTGAAATGGTATATAAGTATGAGGCTGTCCCTATGTTGCACAACGTTGTG
AAAGAATGTACTTCCTTAATAGACAAAACCGTTCAATTTCTTCAGAGTCAGAGTAAGGCAGATTTGGTTGAGATG
AGAACATTAACTTGGTCAAAACCAGAAAAGGTTTCTGAGGAGTGCTCGTTGAACGGTAGTTACACGTCCTCTGTA
ACCGGGTACGATGACTATATAGTCCTTGCCAATGGGAAGTTGAAAGTTATCATTTGCAAAAAGACTGGGGTGATC
ACAAGCATTACGGATGAAACTTTGGGGGTAGAATACCTGGACACGGAACATGGCAGAAACAAATTGGGCGCCAAC
CAGTTTGTTATTTATGATGATAAACCTTTGGGCTGGCAGGCTTGGGATACTGAACTTTATTCCGTCAACCAATAC
AAGTATGTAACAAAGCCGAAGAAAGTTCAAGTTTCCTGCAACACAAAAGAGAAATGTGCTGTTGAGGTCATTTTT
CAAATTTCCGAAAAATGCAAAATCAAATCAGTGATATCACTCAATGCAACTGCGGTCACTGATGCTAAATTAAGT
AAAGTAGACATTTCTACGACGGTAGAAAACTGGGATGCTAGAAACAAATTTTTAAAAGTTGAATTTCCCGTTAAT
ATTCGGAACGATTTTGCTTCGTATGAAACTCAATTCGGGATTACCAAAAGACCAACACATTATAATACCTCATGG
GACGTCGCTAAATTTGAGGTTTGCCATCACAAATTTGCTGATTATTCTGAATACAGCAAGGGTGTTTCAATTTTG
AACGATTGCAAATATGGGTTTTCCACACACGGTAACTTGATGAGATTATCATTATTAAGGTCACCAAAGGCTCCG
GATGCGCATGCTGATATGGGTACTCATGAGATAAAATATGCTATCTATCCACATAGAGGAGCACTATCAAGCGAC
ACTGTTAAGCTCGCTCACGAATTCAATTATTGCTTCAAATACAAACTCCCCAAAGATATTGGCATGAATTTTGAT
GACATCATAAGCATTTCCGGAGATGAGAATGTCATTTTGTCTAACATAAAAAGAGGCGAAGATGATAGTGCTGTC
AAGTCCAACTATTCATTAAATCCAAGGGATGAACAAAGTATTGTGGTAAGGGTTTACGAATCTCTTGGCGGGGAA
TCTTTTGCTTCACTAAATACCACACTAAATTTGAAGCGTATTGAGAAGGTCGATAACTTGGAGATGAAAGTTTAT
AAGAGTTTGACAGCAACGCGAGACGAATCAAATCATGCAATAAACAGAATTCCCATTAAATTGAGACCTTTTGAG
ATTGCCTCATTCAGGTTGTATTTCTGA

>YNR062C 0.81 Cold
ATGTCAATTGCACAAGATAGGGGTATCGTTTTCAAGCTTCTATCAATTTATCGAGCAGCGGCTGGTATCTTCATG
GCGTTGGCACAATTAATTGTCATATTTTTTGGATATTGCGATTTTAAAATTAAAGGGTATCGCATTGCCTCTTAC
AATGCACCGACATTTGCCTCCAGTTTCATAATTCTTGCAGTATGCCTTTTATTAGTTGTCGTTCTGGAAAATCCT
GAGGTTAAAGTTACAAATTCGGAAAATAGCCTTTTCAGTGCTCTGAAACAATTTTTTAGAGTCGAAAGAAAAAAA
CTCATCTCCTGCTTGATTTTACTATGGAGCATGTTCCTATCGTCATTTATTATGAGCGAAGTAGTTTATTTTATG
CCACTATTCCTGACGCTCCACGTTAACTGGGACACCAAGTTTCAAGGCATAGCGTTTATGGTCGCATCTATACTT
GGTGTGACTGGAAGCTATTTCGCTCCAAAGTTAATAAACGTCGGGTGCTCCTGCGGCAGAGCCAAAGATGGCGGA
TTAGAAGAGTCAGATACAACTGGAAGTGAGACCGTTGAGGTAAAGAAAAAAGATTCGCTGTACAGCGGTCAAGTT
TTTCTGTCCATCTTTGCATTGTTTGTTTCATTGCTGGGACAAGCTTTTATGATTGGAGCATCCGAAGCCTTGAAG
CATAAATCAATGCCACCCACAAATTCTGGTATATTTTTTTCAGCTGGGATGTCGATTACATTGTTAGGGTACAAC
TTTTTGGCTTCGAGCATTCCAGCCCTTTTTTCCATGTACATCGACCCCAAACTCAAGGTCCAGTTGATGCCTTCA
ATTGGAGCAATCTCTGGGATTGGTAAACTGGTGGCACCGATTGTATTAGCAGCTCTTTATGGCACAAGATTGGGG
CTTTCAATTGCGGTTGGATTTGGTATGATTTTGGTAGCTGTTTCAATTCCACCGCTTATTTGGCTCAGGAAAAAA
AGGTGTTAG

>YDL006W 0.75 Cold
ATGAGTAATCATTCTGAAATCTTAGAAAGGCCAGAAACACCATATGACATAACTTATAGAGTAGGTGTGGCGGAA
AATAAAAACTCGAAATTTCGGAGGACAATGGAAGATGTTCATACGTATGTTAAAAACTTTGCTTCAAGATTAGAT
TGGGGATATTTCGCGGTGTTTGATGGACATGCTGGGATTCAGGCCTCCAAATGGTGTGGTAAACATCTTCATACA
ATTATAGAGCAAAACATTTTGGCAGATGAAACACGAGATGTTAGAGATGTATTGAACGATTCATTCCTAGCCATT
GACGAAGAAATTAATACAAAACTTGTAGGAAATAGTGGATGTACTGCTGCTGTTTGCGTATTACGTTGGGAGCTT
CCGGATTCAGTTTCTGATGATTCAATGGATTTAGCCCAACACCAAAGAAAGTTATATACAGCAAATGTTGGTGAT
TCTCGAATAGTATTGTTTAGAAACGGGAACAGCATAAGACTGACTTATGATCATAAGGCATCTGACACTTTGGAG
ATGCAGAGAGTTGAACAAGCAGGTGGCCTGATAATGAAAAGTCGTGTAAATGGTATGCTGGCAGTGACGAGATCG
TTAGGGGATAAATTTTTTGATAGTTTAGTAGTGGGCAGCCCATTTACCACGAGCGTAGAAATAACTTCTGAGGAC
AAATTTTTAATCCTAGCGTGTGATGGATTATGGGATGTTATTGATGATCAAGATGCATGCGAATTAATCAAGGAT
ATTACTGAACCTAATGAAGCTGCAAAAGTCTTGGTTAGATATGCTTTGGAAAATGGCACAACAGATAATGTAACG
GTCATGGTTGTCTTCCTCTAA

>YOL119C 0.78   Cold
ATGTTGAACATTCCCATAATTGCTAACTCCAAGAGGTTCCTGTTCTCAAAGGATCACGAAGCGCAATCTACGAGA
GATCACGATGTGGAGCTGGAGACAAGGGAGGGCCCAAGTTCAGGATATAATCCAAACTTCAATGCAGCGGACGCG
ATTTTGAAAAAGAACAGCGATCAAGTAGATCTTGACGTTAATAAGTTAACGAACGTCACCTCAAGAGTTTTAAAC
ACCCCAGAGGCCTCTTTGATTTATGATGATGACAGAGAGTTCCCAGATGGTGGGTTAAAAGCATGGCTGGTAGTA
TTTGGTGCGTTTATGGGACTGGTCCCGGTTTTTGGCTTAATCAACTCCTTAGGGGCTATTGAATCATATATCTCT
AAACATCAATTGGCAAATATATCTTCTTCAACAATATCTTGGATTTTTTCCCTATACTTAGCTATCAGCTTTTTA
AGCTGTATATTATCCGGTGGCTATTTTGATAGAAATGGTAGTATTGGTTTGATGTGCACAGGTACAGTCATATAT
GCTGGAGGACTATTCGCATTGGCAAATTGTAAGTCCGTATGGCAGTTCATTTTGGCATTTTCTGTGTGCTCTGGA
CTTGGGACGGGAATTTTAATGACTCCTCTGATAGGAACTGTTGCAACCTGGTTTTTAAAAAGAAGGGGTATAGCA
ACATCTATAAGTACCATGGGGGGTTCAATAGGGGGTATAGTTTTCCCTATTATGCTGAGAAAGTTATATAAAGAA
GTGGGCTTTCAATGGGCCATTAGAATCCTATCATTCATTTGTTTAACATGCCTCATTTGTGCTTCAGTTTTGGCT
AGAGAAAGAACTAAACCGGTCGTTCAACCATTCAAATCCAAGGCGGAAGTAGCAAAGTGGTACATTTCCTCTGTG
TTCAATTGGAGATATTTTTTGGAAGGAAAATTCTTGTTCGTCGCAATAGGCGCTTCTTTTGCAGAAAGTTCCCTA
ACTTCATGCGCTACATATTTAGCATCTTATTCTATGACAAGAGGAAACACAGAAAATGTTGCTTATACCATGATC
ACTGCTTCGAATGCTGTTGGTATACTTGGAAGGTATATACCAGGCTATTTTGCGGATAAGTTCATCGGGAGATTC
AATGTAGAAATTATCACCATTTCAATGGCTGCCTTATTTAATTTTGTCATGTGGCTGCCATTTGGCGGTAATACA
AAGGTACTTTGGGCGTACGTTTGTTTGTGGGGGTTTTCGACGGGTTCTATCCTATCACTGACACCCGTTTGCATC
GGGCAGATATCTAAAACTACTGATTTTGGTAAACGTTATGCAACGGTATATCTATTGCAAGCGTTGGTAACGATT
CCGGTTTTACCTATCGGCGGTACCTTAATCGGTAAAGGTACAGTTGCAAATTATAACCATTTTATAATATTCAAT
TCAGCGTTAATGGCGGCAGGTGCGGCATGTTATATAATATCAAGGCATATTTGCGTTGGTGCGAAGCTTTGTAAG
TTTTAA

>YBR287W 0.82   Cold
ATGGTTGAAACATTTAGTTTTGCTCATTTGGCTTATTTGGTCTTTGAGTCCGTTTTACAGGTAGTCATTATCGCA
CTCGCTGGTTTTTGGAGCGCTAGTTCTGGTCTGCTGCCCAAACAAAGTCAAAAAATCATTTCTCTGTTGAACGTG
GACCTTTTTACACCATGTCTTATTTTTAGTAAGTTGGCCAAATCCCTGTCCATGGCCAAAATCTTTGAAATAGCC
ATCATTCCTATTTTTTTTGGTTTGACTACTGGTATTTCATTTATTTCCGGAAAGATAATGAGCCGTATCCTAGAC
TTGGATAAAGATGAAACTAATTTTGTGGTGGCAAACTCTGTCTTTGGTAACAGTAACTCTCTACCTGTGTCCTTG
ACATTATCTTTGGCTTACACTCTGCCCAACTTAACTTGGGACCAAATTCCTAATGATAACAGAGATAACGTTGCA
TCAAGGGGTATTTTATACTTGCTGATCTTTCAACAAATCGGACAAATGCTGAGGTGGAGCTGGGGTTACAATAAG
CTAATGAAGTGGTCTGGAGAGAACACTCAGCATATGCCACCTTCTCAAGTGCAATCTTTGTTGGAAAGGACGCCC
AACATTGATAACGAGGAACTTGTCAATGAGGAACAAGAGGAACAAGAATTGCTTGAAGAAGAAAATAACAGAATG
AACTCATCTTTTTTGAGTTCGAGTTCCATCGGTGATAAGATTTGGCAAAAATCATGCACTGTATTTGAGAGAATC
AGAGCGAACCTTAATCCTCCGTTATATTCCATGATTTTTGCTGTTGTTGTGGCAGCAATTGGTCCGTTGCAGAGA
GAGCTATTTATGGAAGATGGGTTTATCAATAACACATTTGCCGAGGCTGTTACGCAGCTGGGTTCAGTTTCTATC
CCACTGATCCTGGTTGTCCTCGGTTCTAACTTATATCCTTCTGCGGAAGTCTTCCCTAAGACGGTTCACCATAGC
AAGTTATTGATCGGCTCCATCATCGGAAGAATGATTTTACCTTCTTGCTTCCTTTTGCCTATTATTGCTATTGCT
GTTAAGTACATAAACGTGAGTATTTTGGACGATCCAATTTTCCTTGTTGTCGGTTTTCTTTTGACTGTTTCCCCT
CCTGCCATTCAATTAACTCAAATCACTCAACTGAATGAATTTTTCGAGGCAGAAATGGCCGATATTCTATTTTGG
GGGTACGCTGTCTTAAGTTTGCCTGTAAGTATTATTGTTGTCTCAGGTGCAATTTACGTTTTACAGTGGGCTAAC
CCAACTTAA

>YDL189W 0.73   Cold
ATGACAGCACTAGATTCTCGAAACTGGGGATTGACACCCGCTATGGAAACGGGCTTGTTCCAGAAACCACAGGAC
CGTATCTTTATAATAGAGCTGGAAAATTCCATAGTGTCATTCATAAACTCAAACACAGAATCATTTCAGCTAAGA
CCGATGAATTCATATTATAGGCTTCTATCGCACCAAATAGCTGAGTACCACAACTTGAACCATGTTTTGGCAAGA
ACTCAAGATAGTTGTGTGATTCTTTTCAAAGGTGAGAATTTCCAAAAGATTGAAGGAAAGCCACTCTTACAAGAG
TTGCAGCTGAACAAAAAACCGGAAGAGTGTGCTTCCTCAAGTGAGAGTATTGAAAAATCGAACAATAACAGAATA
TTTCGAATCTTAAAGCGGAAAGAGGTAGGTAACGAGTGCGATTATAAAATCGATGGCAATACACGTACACCAAAT
AGCAATTTGACAGCCAATTCAAATAAGGACCAAAAAATCGAAATCGATGATAAATCTAGTACTGATTTGGAACAA
GAAAGAATTGAGAAGGAAAGACTTTATGAACAACGTAAACAAGAAATTTTTGATAAGCTCAACAAGAGTGAAGAC
GATGTAAAGTCAACCAACAGTAGCGGTAGTAGTGATAGTGACAATGAATGGAGTGATTGGTTGAACGGAGATGAC
TCTAACACACAAACTAGTAACGGTTCAGTTAGCTCTTCATCACCATTCAATTCATCTGTGACGACGATACAAGTA
AACAAACCTCAACAACAATTTTATGATAGTCGAAGAGGAAGAGGAGGAAGAAGAAGAGGAACAAACAATTACAAA
GATGCCTATAGAGGCCAAAGTCGTCGGAATAAAGAAAACGGCGGTTACCAATCGGGATATTCTTCGCCATATCTT
GTGTATCCTCCTCCCCAAATGGGTGGCAATAGTCTGCCAACCTACCCTCTAATGTATAACCCCGCTGGTCCTGCC
CCCGGTCCTGCACCTTCACCCATGGTAATGGGCAATAACACAGTCTTCATGAACCCTTACATGTACAATATGAAT
CCCCAAGGGTCCTGTTCGTTTGGCACCCCAATCCCTATGTATCCCCCATACCAATATCAATATCAATACCAATAT
AACACTCAGTATCACAGCGGGCCATATAGCAATACACCAAGCTATAATTCTAACAATTACACAAGATCCTCAGCA
AATAAGTACCACCATTTTCAAGGAAAAAATTCATATTCTGGTGCGATTCCGAAACGTAGTGATGACAGTAACAGT
AATAAGAATGAGGGAATTCGTAGGGCTAGTGTTGAGGGTTCGCCGTCATCTAGGGATACTGATTCGGTAGAGATG
AAATTTGATAAATTAAACATTTAG

>YPR097W 0.79   Cold

ATGATTACTCAAGACACTCCTGCTTTAAATCCAACGGAAGAACACTACCTGAAGAGGGAACTGCTTCGATGTCAA
TTGGATTATGAAATTGGAAAGCTTAACGATCAGTTTGCTTTGAGAAAGTTCGGTTATCCTTTCTCACCAAACGAT
CCGACAGCTCCACAGCCTATCTCCAATAACGATTCATCACCAGTGCTCGGTGGCAAAGGCCATTTTAGCGTTAAT
TATCCCATGCTGAGTTATGTTTTGCAAGAATTTATATCCACATTTCCTTTACTTTCAACAAATTTGCTAGTTGAT
GAAAAATTCTGGCAGAGTAAGGTTCAAGTGTTCTTTGAACATTTCATGAGTCTTGGGTTTAGTGAAAGTTATGAC
AGGGAGGAAGCTTCAAAAAGGAAGAAAGTTTCCAAAAAGCTATCAAAGGTTATCTTGTTGCTCTTTAACTCCGGT
GTTGGCTCTTTTCAAGAGCAAGCGTATTATAATGAGGATAAATTCGTTTTACAGTCAGGCCAAGCGAGAAAACGC
TCTAATATTGAGAAATTTGCAATGCCAACTAGAGAAAATTTAGAGAATTTGCTCACTAACGAATCCGTTTTTATT
AACGGTTGGGACGTCAATATTATATCCGTGTTCAATAAGAATTCACGGAAATGTACTGAAAGTGTGGATAATGAC
AAGTCTAGTAAATCTACTCCAACTTCGAGTCCCAAAAGCCATGCTATAAAATCATTTGCTTCCACTTCTAAATGG
ATGAAGAATGCCTTCAACAACACTATTAACAGTACCATAAATAGTATGCCCGAATCCTCAGCATCTTTATTTTCG
AAATTATCCCTTGGCGTGCCTTCAACTAAGAGCAAGCAATCTAGAAAACATCATTATTTCTTGATTAAGATTAAG
AAGCAAGATGACGACGACCAAGATAACAGCAATGAAGAGAATAGTAATCTTGATCACCATGCTGGTTATTTCTAT
GTGACAAGAACATACAGCGACTTCAAAAAACTATCTCATGATTTAAAATCAGAATTTCCGGGGAAGAAATGTCCG
CGCTTACCTCATAGAAATAAAAAAGTGACGTCTATGATAACTAAAACCGAAGTGTTACACAATGGGCAAACTAAA
TCAGCAGCCAGGGAAAAAATCGTCAACACTTTTGACACTGATTTACAATCTGCATCAGAGTCTGATAATTCTTCA
TTTTTACAGACAACGAATGAATTATCTGCTACAGAAACAGTACTCACAGAAAAAGAAACTGAAACGTTGAGAAAA
AATATTCTAAATGAGATCAAGGAAGAAGATAATATTGATGAGGACGAGTATGAGGAAGAAGGTGAAGGTGAAGAA
AGTGATTTTGATGAATATAAAGATGCATCCGATTCAAAAATCAATACTTTGGTGGGTGAAAAAATGAGAACTTCA
TTAAGACAATATTTAAGGACTTTGTGTAAAGATGCAGAGGTTTCTCAAAGCTCATCTATTCGTCGCTTCTTCCTA
AGTGGGCCTAATTTAGATATTAAGGACATAAATCCGAAAATAGCAGATGACATTAGAAACAGGGCTCTGATAGAT
GTAAGTAATTTGGAGAACCAAATACGTTTCCAACAGATGGCGTTAGAAAAGTCTTTAAAGCTGCAAGACTCAATG
AAAGATTTCAAAACATCCCTGTTAAAAGATGAAAAATACTTGATGAGTCTACTAGTAGAAATCAAGGACAATACG
AAAGTTGAAGACCTGTCACCACTTTTACAAGACTTTGTGGAATGGTGTAAGATTTATATTTCCTCAATGATTTAT
CAAATGTTTCTGGGTAACGATAACAGTTATGAACTCTATACACAAATAAGACGGTTACATAAGCTTATGCCGTAC
ACAGTAATGGGACAGATCATGAAATTTACTAATCCAATAGCGATCATGAGGGGTATGATTGAATTATTTATGGCA
CAACCCTTCGGTGGTCATTCGCTCCTTCAAACAATGTTTTCTACGATATTAACAGATGATTTGAAAACTCAAAAG
GTAGCTATAAAGGAATTAGAAAGGAAAATTGCTGAGATGGACCCGGGCGCTTCAGTTGTGACTAAATGCTTGAAG
GATTTTGTCTTTAATAATGATACTAAGGATGAGCATGATACTAAACTCTTTACTATGGACGCAGTAAATGCAGAA
TCCGAATCCATGAACATGCCTGTACCATTGATAGTATTAATGAAAAGTGCAGCTGCAAATCTAATTCCAGACGAG
GTTGTCGCGGGACTTATTGAATCCTATTCATCATGGAAACTGCAAAAAGAAGACACGGATGCCTTGAATGTAACC
AGCGAAGATCAGTCAGGTATTTATTTCACACACGTCAAAGATTTATGGCAATTGTATATTAAAGAACATGATAAA
CAGCTAATGAGGCAGCTATGGCAAGATCCTGAGTTAACCCAAATGCTAAAGGCAATTGTCACTATGATTTACGAA
CCTATGGTCAAGATCTTCAAAGTTGCAAGGATGGATGTTGCTTTGAAAAATTTTGAGAAGTTCATGGGTGATTTG
ATCAGGCTTGTTGATGATGTTATCAATGGTCAGTTGGGCGTTTCCACACAGTTCGATGTCGTGGAAGAAATCCAT
AACTTAGTAACCAAACATCAAGATGCCTTTTTTGAGTTTATTCATGATGTATATTTGAACGATTCAGAGGGCATT
TTTGAAGGTTTTATTACTTGGATTACTACGATAGTGAAATTTTTGCAAAAGAGTAAATTCGGAGGACCGAGTGAA
AGGATCGACTTCAATAAGTTAATTTGTAGAGATGATATCGATATTGATGTCAAACTTTTGAAAGTTCAAGTTAAT
AATGTTCTTAATAAAAAGATTGGTGCGAGAAAAATCTATAAGAAGTTGTTAGATTTGAAGGTTAAACAAGGTACT
AAACAAAATAACAAACACGCTGCTGGCATACTTCAAAAGAATTGGAGTGACATAAACTCCTTGGTTATGCCATCC
AGCTCTGGATCTTTTGGACTTGGAGATGGTGATTTGGTGGATTTGGATCTCGATACAGGTGATTACGATTTTTTA
CATAAAGAGAATGAAGTTGAATTAGAAAAGCAATACAAAGATTTATTGAATCTTGTTGTTGATGAAAGTGAAATT
GATAAATTGAGATCTCAAGTTTTCGCACAGGAATTAAAAAATTATTTAGAAGCTCAAATTGCTAAAAAGTAG

>YDR022C 0.8 Cold
ATGAATGTTACAGTTACTGTTTATGATAAAAATGTCAAGTACAGATTAGAAGAGAATATCAAAAATAACAAGGGT
CCCAGCAACGATGATCAACCAGCATACAATAATGAATCAAAGAGCACGGACGGAAGTGATTATGCAATGTTTCCC
ACTAACATTAAGTACATATTTGAGGACAACAATGATGAACTAGTAGATTCAAGTGATGCTGCTTTGACAGCAGGA
ATAGATAAGGTCGGAGATGAACTTGAAAATGTCATAATTGTCCAATTGGATGAATCGGGATCTCTTGAGGATATC
ACACTAATCAGCGACCAATATGAGCTTTTGTCACATAGGACAAATAGTCTCAGCTTGGAGGAAAACCAAATGAGA
ACATTATCATCACATGGTGATGATAAATCTAATGATGAAGAGGAAGAACTTAGCGTTGACAGTGATAGATTCAGA
GTGGACTCGGACATTGAGCTCGATGTAATTTCACAATTTTGCGATCTGTCTCCTTTTCTTCGCGATTTGAGTTTG
AATGACTTAATTAAACTCTACGTCACACAAAATGAACAATTACAAATGCTCTCCAATTCCGTATGA

>YER007C-A 0.78 Cold
ATGTTTAAGAAATTCACAAGAGAAGATGTCCATTCACGTTCAAAGGTCAAATCTTCAATTCAAAGAACGTTAAAG
GCCAAATTGGTCAAGCAGTACCCCAAAATAGAAGATGTTATTGATGAATTAATTCCTAAGAAGAGTCAAATCGAG
TTGATTAAATGTGAAGATAAGATCCAATTGTATTCCGTCGATGGTGAAGTTCTTTTTTTTCAAAAATTCGATGAA
CTGATTCCAAGCCTGAAATTAGTACACAAATTTCCAGAGGCTTATCCCACTGTTCAGGTTGATAGAGGTGCTATT
AAATTCGTTCTCTCAGGTGCTAATATCATGTGCCCTGGCCTAACTTCTGCGGGTGCCGACTTACCACCTGCTCCT
GGTTACGAGAAAGGTACGATTGTAGTCATCAATGCCGAAAATAAAGAGAATGCATTGGCTATTGGTGAATTGATG
ATGGGTACTGAAGAAATTAAATCAGTGAACAAAGGTCATTCCATAGAATTGATTCACCATTTGGGCGACCCCTTA
TGGAACTTCAGCGTTGAGTAA

>YOR197W 0.72 Cold
ATGTATCCAGGTAGTGGACGTTACACCTACAACAACGCTGGTGGTAATAATGGCTACCAACGGCCCATGGCTCCT
CCACCTAACCAGCAGTATGGACAGCAATATGGTCAGCAATATGAACAGCAGTATGGACAGCAATATGGGCAACAA

AATGATCAGCAATTCAGTCAGCAATATGCTCCACCACCAGGTCCTCCCCCTATGGCTTATAACAGGCCTGTGTAT
CCCCCCCCTCAATTCCAGCAGGAACAGGCAAAGGCACAATTAAGCAACGGCTACAACAATCCTAATGTAAACGCA
TCCAATATGTACGGTCCACCCCAGAATATGTCATTACCTCCACCTCAAACACAAACTATTCAAGGTACAGACCAA
CCTTATCAGTATTCTCAATGTACTGGGCGTAGAAAGGCTTTGATTATCGGTATAAACTACATAGGTTCAAAAAAT
CAACTGCGTGGTTGTATCAATGATGCTCATAACATCTTCAACTTTTTGACTAATGGGTACGGTTACAGTTCAGAT
GACATTGTCATATTAACTGATGATCAGAACGATTTGGTCAGGGTTCCCACTAGGGCTAATATGATTAGGGCCATG
CAATGGTTGGTCAAGGATGCGCAACCCAATGATTCTTTGTTCCTTCATTATTCTGGACATGGTGGCCAAACTGAA
GATTTGGATGGGGACGAAGAAGATGGGATGGATGATGTTATATATCCGGTCGATTTCGAAACTCAAGGGCCAATT
ATCGACGATGAAATGCACGATATAATGGTGAAGCCCTTACAACAAGGTGTTAGACTAACAGCATTGTTTGACTCT
TGTCATTCGGGTACAGTGTTGGATCTTCCATATACCTATTCTACTAAGGGTATTATTAAGGAGCCCAATATTTGG
AAGGATGTTGGCCAAGATGGCCTGCAAGCAGCTATTTCATATGCCACAGGAAACAGGGCTGCTTTGATTGGTTCT
TTAGGTTCTATATTCAAGACCGTTAAGGGAGGTATGGGCAATAATGTGGATAGAGAACGCGTGAGACAGATCAAA
TTCTCAGCAGCAGATGTTGTTATGTTATCAGGTTCGAAGGATAATCAAACTTCTGCAGATGCTGTCGAAGATGGG
CAAAATACAGGTGCAATGTCCCACGCCTTCATCAAGGTTATGACTTTACAACCACAGCAATCATATTTATCTCTT
TTACAGAACATGAGGAAAGAATTGGCTGGTAAGTATTCTCAAAAACCACAATTATCATCGTCACACCCTATTGAC
GTAAATCTGCAATTTATTATGTAG

>YCL064C 0.76   Cold
ATGTCGATAGTCTACAATAAAACACCATTATTACGTCAATTCTTCCCCGGAAAGGCTTCTGCACAATTTTTCTTG
AAATATGAATGCCTTCAACCAAGTGGCTCCTTCAAAAGTAGAGGAATCGGTAATCTCATCATGAAAAGTGCCATT
CGAATTCAAAAGGACGGTAAAAGATCTCCTCAGGTTTTCGCTAGTTCTGGCGGTAATGCCGGTTTTGCTGCTGCA
ACAGCATGTCAAAGACTGTCTCTACCATGTACAGTCGTGGTTCCTACAGCGACAAAGAAGAGAATGGTAGATAAA
ATCAGGAACACCGGTGCCCAGGTTATCGTGAGTGGTGCCTACTGGAAAGAAGCAGATACTTTTTTAAAAACAAAT
GTCATGAATAAAATAGACTCTCAGGTCATTGAGCCCATTTATGTTCATCCCTTCGATAATCCGGATATTTGGGAA
GGACATTCATCTATGATAGATGAAATAGTACAAGATTTGAAATCGCAACATATTTCCGTGAATAAGGTTAAAGGC
ATAGTATGCAGCGTTGGTGGAGGTGGTTTATACAATGGTATTATTCAAGGTTTGGAAAGGTATGGTTTAGCTGAT
AGGATCCCTATTGTGGGGGTGGAAACGAATGGATGTCATGTTTTCAATACTTCTTTGAAAATAGGCCAACCAGTT
CAATTCAAGAAGATAACAAGTATTGCTACTTCTCTAGGAACGGCCGTGATCTCTAATCAAACTTTCGAATACGCT
CGCAAATACAACACCAGATCCGTTGTAATAGAGGACAAAGATGTTATTGAAACCTGTCTTAAATATACACATCAA
TTCAATATGGTGATTGAACCGGCATGTGGCGCCGCATTGCATTTGGGTTACAACACTAAGATCCTAGAAAATGCA
CTGGGCTCAAAATTAGCTGCGGATGACATTGTGATAATTATTGCTTGTGGCGGCTCCTCTAATACTATAAAGGAC
TTGGAAGAAGCGTTGGATAGCATGAGAAAAAAAGACACTCCTGTAATAGAAGTCGCTGACAATTTCATATTTCCA
GAAAAAAATATTGTGAATTTAAAAAGTGCTTGA

>YNL331C 0.81   Cold
ATGACTGACTTGTTTAAACCTCTACCTGAACCACCTACCGAATTGGGACGTCTCAGGGTTCTTTCTAAAACTGCC
GGCATAAGGGTTTCACCGCTAATTCTGGGAGGAGCTTCAATCGGCGACGCATGGTCAGGCTTTATGGGCTCTATG
AATAAGGAACAGGCCTTTGAACTTCTTGATGCTTTTTATGAAGCTGGAGGTAATTGTATTGATACTGCAAACAGT
TACCAAAATGAAGAGTCAGAGATTTGGATAGGTGAATGGATGGCATCAAGAAAACTGCGTGACCAGATTGTAATT
GCCACCAAGTTTACCGGAGATTATAAGAAGTATGAAGTAGGTGGTGGTAAAAGTGCCAACTACTGTGGTAATCAC
AAGCGTAGTTTACATGTGAGTGTGAGGGATTCTCTCCGCAAATTGCAAACTGATTGGATTGATATACTTTACATT
CACTGGTGGGATTATATGAGTTCAATCGAAGAAGTTATGGATAGTTTGCATATTTTAGTTCAGCAGGGCAAGGTC
CTATATTTAGGAGTATCTGATACACCTGCTTGGGTTGTTTCTGCGGCAAATTACTACGCTACATCTCATGGTAAA
ACTCCTTTTAGCGTCTATCAAGGTAAATGGAATGTATTGAACAGGGACTTTGAGCGTGATATTATTCCAATGGCT
AGGCATTTTGGTATGGCTCTAGCCCCATGGGATGTCATGGGAGGTGGAAGATTTCAGAGTAAAAAAGCAATGGAA
GAACGGAAGAAGAATGGAGAGGGTCTGCGTACTTTTGTGGGTGGCCCCGAACAAACAGAATTGGAGGTTAAAATC
AGCGAAGCATTGACTAAAATTGCTGAGGAACATGGAACAGAGTCTGTTACTGCTATCGCTATTGCCTATGTTCGC
TCTAAAGCGAAAAATGTTTTCCCATTGATTGGAGGAAGGAAAATTGAACATCTCAAGCAGAACATTGAGGCTTTG
AGTATTAAATTAACACCGGAACAAATAGAATACCTGGAAAGTATTGTTCCTTTTGATGTTGGCTTTCCCAAAAGT
TTAATAGGAGATGACCCAGCGGTAACCAAGAAGCTTTCACCCCTCACATCGATGTCTGCCAGGATAGCTTTTGAC
AATTAG

>YJR004C 0.65   Cold
ATGTTCACTTTTCTCAAAATTATTCTGTGGCTTTTTTCCTTGGCATTGGCCTCTGCTATAAATATCAACGATATC
ACATTTTCCAATTTAGAAATTACTCCACTGACTGCAAATAAACAACCTGATCAAGGTTGGACTGCCACTTTTGAT
TTTAGTATTGCAGATGCGTCTTCCATTAGGGAGGGCGATGAATTCACATTATCAATGCCACATGTTTATAGGATT
AAGCTATTAAACTCATCGCAAACAGCTACTATTTCCTTAGCGGATGGTACTGAGGCTTTCAAATGCTATGTTTCG
CAACAGGCTGCATACTTGTATGAAAATACTACTTTCACATGTACTGCTCAAAATGACCTGTCCTCCTATAATACG
ATTGATGGATCCATAACATTTTCGCTAAATTTTAGTGATGGTGGTTCCAGCTATGAATATGAGTTAGAAAACGCT
AAGTTTTTCAAATCTGGGCCAATGCTTGTTAAACTTGGTAATCAAATGTCAGATGTGGTGAATTTCGATCCTGCT
GCTTTTACAGAGAATGTTTTTCACTCTGGGCGTTCAACTGGTTACGGTTCTTTTGAAAGTTATCATTTGGGTATG
TATTGTCCAAACGGATATTTCCTGGGTGGTACTGAGAAGATTGATTACGACAGTTCCAATAACAATGTCGATTTG
GATTGTTCTTCAGTTCAGGTTTATTCATCCAATGATTTTAATGATTGGTGGTTCCCGCAAAGTTACAATGATACC
AATGCTGACGTCACTTGTTTTGGTAGTAATCTGTGGATTACACTTGACGAAAAACTATATGATGGGGAAATGTTA
TGGGTTAATGCATTACAATCTCTACCCGCTAATGTAAACACAATAGATCATGCGTTAGAATTTCAATACACATGC
CTTGATACCATAGCAAATACTACGTACGCTACGCAATTCTCGACTACTAGGGAATTTATTGTTTATCAGGGTCGG
AACCTCGGTACAGCTAGCGCCAAAAGCTCTTTTATCTCAACCACTACTACTGATTTAACAAGTATAAACACTAGT

GCGTATTCCACTGGATCCATTTCCACAGTAGAAACAGGCAATCGAACTACATCAGAAGTGATCAGCCATGTGGTG
ACTACCAGCACAAAACTGTCTCCAACTGCTACTACCAGCCTGACAATTGCACAAACCAGTATCTATTCTACTGAC
TCAAATATCACAGTAGGAACAGATATTCACACCACATCAGAAGTGATTAGTGATGTGGAAACCATTAGCAGAGAA
ACAGCTTCGACCGTTGTAGCCGCTCCAACCTCAACAACTGGATGGACAGGCGCTATGAATACTTACATCTCGCAA
TTTACATCCTCTTCTTTCGCAACAATCAACAGCACACCAATAATCTCTTCATCAGCAGTATTTGAAACCTCAGAT
GCTTCAATTGTCAATGTGCACACTGAAAATATCACGAATACTGCTGCTGTTCCATCTGAAGAGCCCACTTTTGTA
AATGCCACGAGAAACTCCTTAAATTCCTTCTGCAGCAGCAAACAGCCATCCAGTCCCTCATCTTATACGTCTTCC
CCACTCGTATCGTCCCTCTCCGTAAGCAAAACATTACTAAGCACCAGTTTTACGCCTTCTGTGCCAACATCTAAT
ACATATATCAAAACGAAAAATACGGGTTACTTTGAGCACACGGCTTTGACAACATCTTCAGTTGGCCTTAATTCT
TTTAGTGAAACAGCAGTCTCATCTCAGGGAACGAAAATTGACACCTTTTTAGTGTCATCCTTGATCGCATATCCT
TCTTCTGCATCAGGAAGCCAATTGTCCGGTATCCAACAGAATTTCACATCAACTTCTCTCATGATTTCAACCTAT
GAAGGTAAAGCGTCTATATTTTTCTCAGCTGAGCTCGGTTCGATCATTTTTCTGCTTTTGTCGTACCTGCTATTC
TAA

>YLL003W 0.79 Cold
ATGGGCAAATTCGGCACGACAAATAAATCAACGGAGAATCTTCTGCGTGATAAATTCGTACCCGAGACATCTCCA
ACTAATATTCCCACTGATGTACTCATCAAGCAAGGGCAAATAACGGATTCCACCGAATCACTAATTCATGGAGGC
GCAGAAAGGTATATTGTTAACGCTTTAAAGCCTATAGAATTAAATAAAACTGAAGGCTTTTTCGAAGACCCGCCG
TTCCATCTTCCTTCTCCACCGGTTGATTCGACAAATCTGGAGTATGAAGACGTTACCGATCTTCCTAAGAATGGT
TTACGATATGATTTGAATGATATATCCGTTGAGGTAATCGAAGATTTATACCGCCAGATTGAAGCTTTTTTGGTT
CATTTCAAACTATCCAGAAGTTTTTTACAAATTTTCAAAAACTATGTCAATATTCTTATTCAAGAAGGCATCAAT
CCTTTACGCGATGAGTACTTCACAATATTGGAAGATGAACTGAAAGGTTTTTTCACTTTCAATTCTGTTATAGAA
GAGATTTTAGAAATATTTTTAATCCACCCTCGCAACAAATTCATTGCATTGTCCCTTGCAGAATATACCTACGCT
AAGAACAAAATCAGAAGACATTTTAATCACTGGAAGACTGTATGTGAATTGAATGAAGAGGCAAACAGGTTTGCA
AATCAAGCAAAGCTGAGGGTACAGGAAGCCGTCTTCTATATTTGGAGTGATAAAACATTAAAATACTCACAGATG
GCCAACGATGAAGCTGAAAGTTTTAGGAATACTTGGCTACTATTTCGCTCGTTCCAACAATGGATAACTTTAACA
CAAACTCTTAAGGAGCAGTCAAGGTTAGCAGATCAGGCCTTTTTGAATAAGATGTTTAGGAAAATTTTAAAGGCA
CAAGAGCATTGGAAACACTTAGAAACTGTTAACACTGACAACATTAAGAAGATATTTTTACGAACAACATTTCAT
ATATGGAAGCTAAGACATAAAGAAATAAACTACCACGGGTTGGAAAGAAGGATTTTCGAAAGAATAAAACAGAAA
GTTATAAACTATGAATACAATAAGAGCATTGCAGAAAAAGTGAGGTCGTTTTCTCTACAAAGAAAATATCTGAAT
AAATGGGAAAAGAAAAACATTGAAAACGAAGATAAACTTGGGGCACTTTATGAACTGGAGAATAAATTCATCAAA
CAAAAGTTTTTTCGCAAATTAAACCGGTCATTTCAACATAGTCAACAAGAGGCAATTGCAAAGAGTAAACTAAAT
CAGACACTTTTGAGGTGCGTTTTTGAGAAGATGTGGCTGAAAAGATTCGAAGACCATCTGCATTTGTATTCAATT
GTAAGTCTAAAAGAGGCTAACCTCGTGAAGCGTATTTTTCATTCATGGAAAAAACTTCTATATATTGACCTCAAA
GCAAGCGATTATTCGAGGACTAATTTGCTCAAGTCATCATTGCGAAGTTGGAAACTTGAAGTAAAGTTAAAAATA
TTTGAGCAGAAATGTAAAAAGAGTATTCAAGCAAGCGCGTATCGTACATGGAGGAAAAGAATACAGTATGGGAAA
ATATCGAGCGAACATGTTAAAACGGCATTTTGTGCAAAATATCTTGGTGTGTGGAAAAGGAGGATGCTACAAATG
AATTCTATGAATGACGAAGCATCCAAATTTTACGAAGAGGGTCTCGTAAATGAGTGTCTAGCTATATGGAAAGAA
CGCCTGATTAAAACTAAGGAATTGGAGGATAGATACAATTTCTTATGTAAGACACATGCAATTTTGACTGTAAAA
CGGACGCTAATGCATATTGATAATGTTCATTTGCTATATACGAAACTGGCGCCCTCTATGGATAGAGTAAAGCTT
TCTAAGGCCTTTTTAAAGTGGCGGAAAGCCACAAGGTTCAAAGTCAGGCATAAGTTAAACGATATTTTACACGTT
TATGAAAAGAGTAAAGAGCGCGAACTTCAAAGCCAACTGTTCAACGCTTGGCGAAATAGATTTTGCTTCTACACA
GAAGAATGTAACATTCAGGCTATTTCAAAGAGAAACTACCAGCTTGAAAAAATGGTGCTGAAGAAATTTAGAGAA
AGACTTTTAGAGATAGTAAAATCAGAAGAATTAGCAGACGAAGTTCGCGAAGAATTTGTGTTAGTCAAGACGTTT
TATATTTGGAAAACTCATCTAGACGAAATATTTTATATGAGTACATTATTGGAACAATCGGAAGCTAATAAACAA
TTCATAATTACATCCAAATTCTTGAAAATGTGGAGTCTTCGATTCCTAAAAATTAAGCGTAATGATGAGACAGTC
GAGGTGTTTCGTCATCGGTGGGACAGGGCCACTGTAAGGGGATTGTTATTATTATGGAAAAATCGTTCAGACAGT
TCTCCAAAGAGAAGGAAGGACTTCAATCTTAAACATGAACTAAAAACTCCCATAAGATCAGACTCTCAAAACGCC
TCAACCATACCAGGCTCAGAAAGAATAAAGCAGCACAGAATGGAAGCGATGAAGTCGCATTATAGCAGGGCAAGA
AGAGCCATACCAAGTCCGGTGAAATCTTCCAGTGTTCTTGATTCTACAGCTAAAAAACAGATCAACCTTGAAAGT
ACGACAGGCTTAAACGGATCTCCGACGCGAGGAAAACCTCTAAGGTATTCTCCTAGGCGTACCACTAGAAACATG
CCATCCAAAGTTGACCATATTGATTTTGGCAGAATACCCGCTGTACCTTTTAGCCTAAGCGCCAATTCTCCTAAA
ATCGATCAAGATATGGATTATATAAGAGAGCATGATAAATCCCCGTTAAGTCGTAAACGTCAATAG

>YDL061C 0.76 Cold
ATGGCTCACGAAAACGTTTGGTTCTCCCACCCAAGAAGATTCGGTAAAGGTTCCCGTCAATGTCGTGTCTGCTCC
TCCCACACTGGTTTGGTCAGAAAGTACGACTTAAACATCTGTCGTCAATGTTTCAGAGAAAAGGCTAACGACATT
GGTTTCCACAAGTACAGATAA

>YKL049C 0.8Cold
ATGTCAAGTAAACAACAATGGGTTAGTTCTGCTATTCAAAGTGATTCGAGTGGAAGATCACTCAGTAACGTCAAC
AGGCTTGCAGGAGACCAACAATCTATTAACGATCGTGCGTTATCGTTATTGCAGAGAACAAGAGCGACAAAGAAC
CTGTTTCCAAGAAGAGAGGAAAGAAGACGTTATGAAAGCTCAAAAAGTGACCTAGATATCGAAACAGACTACGAA
GACCAAGCAGGTAATCTAGAAATCGAGACAGAAAATGAAGAAGAAGCTGAAATGGAAACTGAAGTACCTGCACCA
GTGCGAACTCATTCATATGCCTTAGACAGATATGTTAGACAGAAAAGGAGGGAAAAACAAAGAAAGCAGAGCTTA
AAGCGCGTCGAAAAGAAATATACTCCTAGTGAATTAGCTCTGTACGAAATTCGAAAATACCAACGTTCCACGGAT
TTATTAATCTCCAAAATTCCATTTGCAAGGCTAGTGAAAGAAGTTACAGACGAGTTTACAACTAAAGATCAGGAT

TTACGTTGGCAGTCAATGGCGATTATGGCGTTACAGGAAGCAAGCGAAGCGTATCTGGTAGGATTATTGGAACAT
ACAAACCTCTTGGCGCTGCATGCAAAAAGAATTACTATAATGAAGAAAGACATGCAACTAGCAAGAAGAATCAGG
GGACAGTTTATTTAG

>YJR006W 0.74   Cold
ATGGACGCATTGTTAACAAAGTTCAACGAGGATAGAAGTCTCCAAGATGAAAACTTGTCCCAACCACGAACGAGA
GTAAGAATTGTTGACGACAATCTTTACAACAAATCGAATCCATTCCAACTATGCTATAAAAAACGTGACTACGGT
TCTCAGTACTACCACATTTATCAATACCGTCTAAAGACTTTCAGAGAACGCGTCTTAAAAGAATGTGACAAGAGA
TGGGATGCGGGCTTTACCTTGAATGGCCAATTAGTTCTAAAAAAGGACAAAGTGTTAGATATTCAGGGAAACCAA
CCATGTTGGTGCGTAGGATCCATATATTGCGAGATGAAATATAAACCAAACGTACTGGACGAAGTGATCAATGAT
ACATATGGTGCACCAGATTTAACTAAAAGTTATACTGATAAAGAGGGTGGTTCTGATGAAATTATGCTCGAAGAT
GAAAGTGGGAGAGTGCTTCTAGTGGGAGATTTTATTCGGTCTACACCCTTCATCACGGGTGTTGTTGTAGGTATA
CTGGGTATGGAAGCTGAAGCCGGGACATTTCAAGTTTTAGACATATGCTACCCCACTCCTTTACCACAAAATCCT
TTCCCTGCACCAATTGCTACCTGCCCCACCAGGGGAAAAATTGCTCTTGTTTCTGGGTTAAACCTCAATAATACA
TCACCTGATAGGCTATTGAGATTGGAGATTTTGAGAGAATTTTTGATGGGGAGAATCAATAACAAAATTGACGAC
ATTTCTCTAATTGGTAGATTACTGATATGCGGCAACTCTGTTGACTTCGACATTAAAAGCGTGAACAAAGACGAA
TTGATGATTTCTCTAACTGAGTTCAGTAAATTTCTACACAACATTTTGCCATCTATATCTGTTGATATTATGCCC
GGAACCAATGATCCAAGTGACAAATCTCTACCGCAACAGCCTTTCCACAAATCATTGTTTGATAAGTCCCTAGAA
TCATACTTCAACGGTTCTAACAAAGAAATTTTGAATTTAGTGACCAACCCATATGAGTTTAGCTATAATGGCGTA
GACGTTCTAGCCGTTTCAGGCAAAAATATCAATGATATTTGTAAATACGTAATACCATCAAATGATAACGGAGAA
AGTGAAAACAAGGTCGAGGAGGGCGAGAGTAATGATTTCAAAGACGATATAGAGCATCGCCTGGACCTTATGGAA
TGTACAATGAAATGGCAAAACATCGCGCCCACCGCACCTGATACCTTATGGTGTTATCCGTACACTGATAAGGAT
CCATTTGTATTGGATAAGTGGCCCCACGTTTACATTGTGGCAAACCAGCCATATTTTGGAACAAGAGTAGTGGAA
ATAGGTGGTAAGAACATAAAGATAATATCTGTCCCTGAATTCAGTTCTACTGGGATGATAATATTACTGGATTTG
GAAACGTTAGAAGCAGAGACGGTCAAGATTGACATTTAA

>YOR115C 0.77   Cold
ATGTCCTCTACACATAGTAATAATGTAGGACATCCCCAATCTTCTCCACAAGGTCCACTAACGGAACAACAAAGA
GCACAACAGCAATATCAAATTTTTGAGAATTCATTACCAAAAGTAAGTCAATCTGTGTATCAGATGCTGTTGAAC
GAGATGGTTCCTCTAGCTATGGGCATTGAGAGGCAAATATCAGGCGATGTTATCAGTTCTGATAGCAATGTAACT
TCCGAAAATGGTAACATCAACAATATGATAAAGAGACTGAAGATAGAGGAACATCATACAGTAGATATCATACGT
TCTCATAACTTGATACATGAGCTGTATAAGGCGGATGAAGAGGAAAAAGAAAAGGTTTTAGCAAGACTGAGGAAT
ATTGGGTTTCAAATCGGATTGAAGCTGTCAGAGTTATTGATATTTTCTAACAACCCAAATTTAAAATTCAAGGAA
ATGGACCTTCTATTAATAATGAAGTTCATTTGTAGGGATGTCTGGAAACAAATATTTGGTAAACAGATCGATAAT
TTAAAAACAAACCACAGAGGAACCTTTTACCTTCTTGATTATGACTATCGACCTATTCAATCATTTTCTTTGGAG
GAAGATGCAAAAAACGAGGAGTTAAAAATGATAGAACCGTTTTTGGAGATACCTGTGGGAATTATTAGAGGCGTG
TTGTCTTCCTTAGGTTATTCATCTGAAGAGGTCATTTGCTTGGCGTCTTTTATCGACAGGCCAACAGATAGGCCT
AAAACAGCTTTTCCCAAGGGAGTTAGCTTCCATGTTCAAGTCACAATGCCGCAGTAA

>YDR015C 0.81   Cold
ATGTCTTGTTCGTTTCATATATCAAGTCCTTTAGGCTCTTCTTCTTCCTTGGAGGGCTTTTCTTCATTGAAGGTG
GTGGTTGTAGCCTGTCAAGCTCTTTCGAGGGACCTCCTTGGTATTGATCTTGTTCTCTTACTCCTTTTTTGGCGT
CCAGATAAGCACCCCCATTGGGGCTTCCGCCACCTGTGTTCTCAGACAGATTTTTGGCACGAAGTTGTTGTTTTT
CCAATTCCAAATTAA

>YNR071C 0.76   Cold
ATGTCAAATAGTAACGGTGATAATAAGTATGGTGTCATTACAATTGGTGATGAGAAAAAGTTCCAAGCTACCATT
GCGCCACTTGGTGCAACTTTGGTAGACCTGAAGGTAAACGGCCAATCAGTCGTTCAAGGTTATTCAAACGTACAA
GACTATTTAACAGATGGTAACATGATGGGCGCTACAGTTGGTCGTTATGCCAACCGTATCGCCAAGGGTGTTTTC
AGCCTGGATGATGGTCCTCACAAGTTAACTGTTAACAATTGTGGTAACACAAATCACAGCAGTATCAGTTCCTTA
AATCTTAAGCAGTACAAGGCGTCCCCTGTTGAGAATCCTTCCAAGGGCGTTTACGTTGTTGAATTTAAATTGTTG
GATGATCACACACAGCCCAACCCTAACGAGTTTCCAGGTGACTTGGAAGTCACAGTGAAGTACACTTTGAATGTT
GCAGAAATGACTTTAGACATGGAATACCAAGCACAGTTAGTTCGTGGTGATGCTACTCCAATTAATATGACCAAC
CACTCGTATTTTAACTTGAACAAAGTCAAGAGTGAGAAGTCCATTCGTGGTACAGAAGTTAAGGTCTGCTCGAAC
AAATCGCTTGAAGTTACAGAGGGGGCATTGCTTCCAACTGGTAAGATCATTGAAAGGAACATTGCTACTTTTGAT
TCAACTAAGCCCACCGTTTTGCATGAAGATACTCCGGTCTTTGATTGCACTTTCATTATTGACGCTAACAAGGAT
TTGAAGACTACAGATTCTGTGAGTGTCAACAAATTGGTTCCTGTTTTTAAGGCCTATCATCCCGAATCTCACATC
AAATTTGAGGTTTCGACGACAGAACCAACCGTTCATTTGTACACCGGTGATAATTTATGTGGCAAATTTGTGCCA
AGGTCTGGATTTGCCGTTCAGCAAGGTAGATACGTTGATGCTATCAACCGTGATGAGTGGAGAGGTTGTGTTCTT
CTGAAGCGCGGCGAGGTATACACTTCAAAGACTCAGTACAAATTCGATATTTAG

>YHR169W 0.74   Cold
ATGGCAGACTTTAAATCTTTAGGTCTTTCAAAATGGCTTACGGAATCCTTAAGAGCTATGAAAATTACTCAGCCA
ACCGCTATCCAAAAGGCTTGTATTCCCAAAATCTTAGAAGGAAGAGATTGTATTGGTGGTGCCAAGACTGGTTCT
GGTAAAACTATTGCATTTGCAGGGCCTATGCTGACTAAATGGTCCGAAGATCCGTCAGGGATGTTTGGAGTGGTC
TTGACCCCAACCAGAGAGTTAGCCATGCAAATTGCCGAACAATTTACTGCACTTGGTAGCAGTATGAACATTCGC
GTGTCAGTTATAGTTGGTGGTGAAAGCATCGTTCAGCAGGCATTGGATTTACAAAGGAAGCCGCACTTTATTATT

GCCACGCCTGGACGTCTGGCACATCATATCATGAGCAGCGGAGATGATACCGTTGGAGGGCTAATGAGAGCTAAA
TATTTAGTTCTAGATGAAGCCGATATTTTGCTAACTAGCACGTTTGCCGATCATTTAGCAACATGTATAAGTGCT
CTACCTCCCAAGGATAAGAGACAAACACTTCTATTCACTGCAACTATAACGGACCAAGTAAAGTCGTTACAAAAT
GCACCAGTGCAGAAAGGGAAACCCCCATTGTTCGCTTACCAAGTAGAAAGTGTAGATAATGTGGCTATTCCATCG
ACGTTGAAAATAGAGTATATCTTGGTTCCAGAACACGTCAAAGAAGCATATTTGTACCAATTATTAACATGCGAG
GAATATGAGAATAAAACCGCTATAATATTTGTAAATAGAACAATGACCGCGGAGATCCTTAGAAGAACCCTGAAG
CAGTTGGAAGTGAGGGTAGCGTCGTTGCACTCTCAAATGCCCCAACAGGAAAGAACAAATTCCTTACATAGATTT
CGAGCTAACGCTGCAAGAATCCTCATCGCTACAGATGTAGCATCCAGAGGTTTGGATATCCCAACTGTTGAGCTT
GTAGTGAATTACGATATACCTTCAGACCCAGATGTATTCATCCATAGATCTGGTCGTACGGCCCGTGCTGGCAGA
ATTGGTGACGCCATATCATTCGTTACCCAGCGAGATGTTTCTAGGATACAGGCCATTGAGGACCGTATCAACAAG
AAGATGACAGAGACAAACAAGGTACACGATACCGCTGTAATAAGAAAAGCCTTGACCAAGGTAACCAAGGCGAAG
AGGGAATCGCTGATGGCAATGCAAAAGGAAAACTTCGGTGAGAGAAAAAGACAGCAAAAGAAGAAGCAAAATGAC
GGGAAAAGTTTGCGCTCTTGA

>YDL236W 0.75   Cold
ATGACTGCTCAACAAGGTGTACCAATAAAGATAACCAATAAGGAGATTGCTCAAGAATTCTTGGACAAATATGAC
ACGTTTCTGTTCGATTGTGATGGTGTATTATGGTTAGGTTCTCAAGCATTACCATACACCCTGGAAATTCTAAAC
CTTTTGAAGCAATTGGGCAAACAACTGATCTTCGTTACGAATAACTCTACCAAGTCCCGTTTAGCATACACGAAA
AAGTTTGCTTCGTTTGGTATTGATGTCAAAGAAGAACAGATTTTCACCTCTGGTTATGCGTCAGCTGTTTATATT
CGTGACTTTCTGAAATTGCAGCCTGGCAAAGATAAGGTATGGGTATTTGGAGAAAGCGGTATTGGTGAAGAATTG
AAACTAATGGGGTACGAATCTCTAGGAGGTGCCGATTCCAGATTGGATACGCCGTTCGATGCAGCTAAATCACCA
TTTTTGGTGAACGGCCTTGATAAGGATGTTAGTTGTGTTATTGCTGGGTTAGACACGAAGGTAAATTACCACCGT
TTGGCTGTTACACTGCAGTATTTGCAGAAGGATTCTGTTCACTTTGTTGGTACAAATGTTGATTCTACTTTCCCG
CAAAAGGGTTATACATTTCCCGGTGCAGGCTCCATGATTGAATCATTGGCATTCTCATCTAATAGGAGGCCATCG
TACTGTGGTAAGCCAAATCAAAATATGCTAAACAGCATTATATCGGCATTCAACCTGGATAGATCAAAGTGCTGT
ATGGTTGGTGACAGATTAAACACCGATATGAAATTCGGTGTTGAAGGTGGGTTAGGTGGCACACTACTCGTTTTG
AGTGGTATTGAAACCGAAGAGAGAGCCTTGAAGATTTCGCACGATTATCCAAGACCTAAATTTTACATTGATAAA
CTTGGTGACATCTACACCTTAACCAATAATGAGTTATAG

>YOR329C 0.75   Cold
ATGTCGTTTGATTGGCTTAATGTTCCGGGATTGGACTTAAGCAGCGGGGACCAAGCAGAAAAGAGGCCATCGAAT
GGATTAGGACCACCCAGTGTGTCTTTTGATTTTGGTATAAATACTGCTGCTCCACATGATTCCAGCTTTTGGGAT
CAGGGATCAAGAAGTCACAGTGATACAACTTTGTCTTACAGAAATAATCACTCAAATACAGCAGCTGATAATGCC
ACTAACGTGAGCTCTCCTCAAAAGGATAATCCACCTAATGGGGAGGTACGAACACTTTCTGGAGGCGATGTGTAC
GCGGAGTCACCAGAAGACATGCAAGTCCCATTGTCTTTATCACAAAACCAACTTACCCATGAAGAAATAAGGACA
TATCTAAGATGGTACCACTATATATGTTTGAGAACCCATGGAAAACTAGTAAGATTGAACGACGTGTTTAGGTTC
TTGACCAATTTTAATTTATCACAAAAAGTAAAAGATAGAATTGTGGAAATTTTCCGTAGCTGTAAAAATGCGTTA
AATATTGGTCAATTTTTCGCTGTTTTAAGATTAGTTTCTAGAGCTATTATATATGGCATCTTACCTCTAAGACGC
ATGATCTTGGAAAAGGCACCTGTTCCCAAACCACGTCCTATCCTAAGTAGTGAGAATCACGAAGAGGTGTACGAG
GAGGTGGAAGACGATGATAGTTCTGCCAAAACTGGTGATCAAAAGGTCGATTTTGACTCATTTGCTTCATTGCTG
TTGACTGGTAAGACAACGAGAAAAAGAGTCAGGAGAAGAATCAAAAATTTGAATTTTAAGAGTAAGAAGGTTAGG
TTTTCAGAGCATATAACATTTCAAGACCCTCCAAACCTCAATCAAGAATCATCTAATAATAGTGAAGCAAGAAAG
CAAGATCCTGATGCAGAAGATGAAGATCAGGATTCTAATAATGACAGTCCTTTGGATTTCACATTGCCCATGGAT
CAATTATTAAAGAGGCTATATAAGCGGAGAAAAAACAGCGGATTAGTTTCGAGTTTGCCCAGCGAACAACAAGAA
ACTGAGGAGGAGAAGAAAGTTTTGGAGGATATGAAGGATTCCTTGTCCCATTTCAAACAAATACAGACTGTTGAT
TCTGCCTCATTGCCAATTTCATCGGTATTTTTACAGAATGGCAATACTCTACCAACTAGCAATGTGAATAACACC
ACTGTTCCACAGCAACTTCCGTTAGAACCTTTGAAGCCAACCGCCACAGGTTCAGCTAATCACCTGGTGCGAGAA
GAATATAATCAGGGGTTGCATCCAAGCAACGGTGCCATCCAAACAGGTTTACAACCTTTAAAACCAACAGCAACG
GGCTCTGCAAATTATTTGATGCGAAGTCATATGGAACAACCTCAGTCTATCAAACCTTCGAGTACACCAGAAACA
GTTACAAACTCTGGGGGACTTCAACCTTTGAAACCTACCGCCACAGGTTCTGCAAATTACTTGATGAAACAGCAT
ATCTCACCTTCAGTTAATAACCCAGTATCTTCGATGTTTCAGGCGCAATTTACGAACCAATCTTCATCTCCACAA
AGTACAGGACCAGCATTTCTGAATTCTCCGAACATTACTCTTCCTCAAAGCAATCAACAGCAACCTTACCAAGAG
GTTAATCCGACTCAAGCGAAAATTGAACCATCTAACATATCGCCTCAACACACCTACTCTAACAATGTTCGGATT
AATAATGGTAACATCGTATCCATGCCGAAAGTAGAAATAACTGGTGCATTCCCCCCTCAAAACACTCTTCCTCAA
CATCAGCAATCGCATTTGCTTTCTCCTCAAAATACGATCCCGCAGCACCAACGGTCGCAATTGATATCTCCACAA
AATACCTTCACTCAAAATCAGCCCATTCTTTCTCCGCAACATACTTATTCCAATAATCAAGCAACAATGATATCC
CCCCAGAATACGTATACTAATAATCAACAGCAGCCCCAACATCTTCCACCTCCTCCACCTCCACGCGCTCAGCAG
CAGCAACAAGGGGCCATTGTGCCACCTCAACATATGTACTCTAACGTGCAAAAGCAGAATAATTTGGTGCCGACC
CAACCCTCCTATACAAATTCACCAAGCATACAATCTCCAAATTTTTTGTCACCTCAGAATGCTGCTAATAGTTAC
TTTCAATCTTTACTTTCTTCTTCACCATCTCCAAATCCTACGCCATCAAATGCGTCTACTGTTAATGGCAATAAC
GCCAGTAATGGCATAAGTTCATTCCAAAATACTAGTGCGGCAATGAACAATACTCAATCTCATCAGACGTATATT
CAGCAGCAACAACAACAACAAACTCAACAACGAATATATGGTGGCCAACTTTCTCAAATGCAACAACATCCGGGG
CAACTTCACTTAAACAACAGTGATATACATAGCCAACCTAACAAGCCTAATTACGGAATGCTAGGGCAGCAAGTT
CATCAACAGCAACAGCAACAGCAGCAGCAATTTCCATTCACTGCAGATGTTAATAGATCCAATTCAAGCGATATT
TTGGGTAACTTGCAGTCATTGCAGCAGCAAGTCGATGCTTTACAAATCCAATATAATAGGAGACCATAG

>YKR103W 0.79   Cold

ATGATAAAAAATGGTACATGCCCCTATTGGGAACGTGATGATCTTTCGGAATGTGCTAGGCGAGAATATATCGAG
TTTAAATTTCCTCTATTTATATTGTTGACTGGTATGATATACGCGTTTTGCAAAGTCTTTCGAGCCTTTTATCTA
AGGGGGAAAAATCATACAAATGAAGCGCCAGAATTTGAAGAACAAGGTAATGGAAACCACGAATATGCAAGGTTC
TCAGTTTTAAGACTAAAATCTGCATGGGAAAGCCGTAGCTTTTGTAATGTTAATAATAGATCTACTTTCGACAAA
TTCAAAAAATTTATAGAGGGTGCCTTCATTGTTTTGCAGCTTACTATCCACCTGTATATTTTATCTAGTATGCCA
ATGGATAACAAAAAGTTCTTTCACCAAGGTTTTCTGGTTCAAATGTTTCTCTGGATTTTACTGCTTGTTGTTATT
ACACTTCGTTTAATTTCAGCAAGCCAGTCATTTCGGTGGGTTTTAGCATGTAAGCGTGATTTATGGGCTGTTTCG
TTTTACTCATATGCATCCCTGTTTACTCTTTCGATTTTACCTCTTCGTTCCGTCTTCATTGGAAAGATAAAAGAT
AAAATTATGGTCAAATATATCATTTCCGAGACTTTCATTGATTTAGCGCTTTTATTATTATTATCAACGTCAAGT
ATAGAAGGAACTAGGTACTCATTTTTGGTTGAAAATGAGAACAAAAAGTTGCCACCGGCTCCTACCGTCTTTGGC
CTTCTTACTTTTTCTAGGATTGATCGGCTTATTTGGAAAGCATACAAACACTGCCTTGGAAACGCTGATATTTGG
GATTTGGATATTAATAATAAGTCTATAGCAATTTTGGCGAACTTTGAAATGTCTTCTAAAAAAGGAAGGCTCCTT
CCAAACATTATTTGTTATTTTAAGGCTGTTTTCATCTCCCAATTATTTCTGGCTTTTGTATCTAGTTTTTTGAAC
TTTGTACCCTCATTGTTGATGCCAAGAATACTATCATACGTTAATGACCCAAAGTCAAAATCATGGAACTTGGTG
TCTTTATACGTCTCATCTATGCTCGTCAGTAAAATCATTGCCACGACTTGTAGAGGTCAAGGATTGTTTTTAGGT
GAAAAGGGTACTATGCAACTAAGAACAGTTTTGATATCCAATATCTATTCCAAAACCTTAAGAAGAACAATTCTA
AAAGACTCAACAACGTCACTTCAAAAAAATGCGTCAACATCCTTTGAAGAAAATCCTGACTCTTCTGAAGCGGAA
CCTAGAAAAAAGTCTAGTAGGAAAGACAACTCTGTGAACAATGTTATGTCAATTGACGCTTTCAAGGTTTCTGAA
GCTATGAACACTTTTTATCTGGCATGTGAAGCAGTTTTCATGACAGTTACGGCCCTAATGATACTATATTCCCTA
CTGGGATGGTCCGCCTTTGCTGGTACCTTTGCTCTTCTTGCCATGATTCCTTTGAATTTTTGGTGTGCAACTTTT
TACGGGAACTATCAGGCCGATCAATTAATCCTAACTGACAAGCGTACCTCTGGGATTAGTGAGGCTCTGAACTCA
ATACGTGTAATAAAGCTACTGGCATGGGAAAATCTGTTTTATCAAAAGATTATAAACGTAAGAGATGGGGAAATA
AGACTCCTTAAAAAGAAGGCAACAATTTTCTTTTTGAACCATCTCATTTGGTTCTTTGGGCCCACTTTGGTCTCT
GCAATAACATTCTCAGTGTTTATTAAATTCCAAAATCAGACACTTACTCCTACAATAGCATTTACAGCTCTTTCT
TTATTTGCAATATTGAGGACACCCATGGACCAAATTGCTTCGACTGTCAGCCTTTTGATACAGTCTTTTATTTCT
CTTGAAAGAATCCAGGATTATCTTAATGAATCAGAAACAAGAAAATATGAGATTTTGGAACAAAGCAATACTAAA
TTTGGCTTTGAAGATGCAAGCATGGAATGGGAAGCTGCTGAAACAAGTTTTAAACTTAAGAACATTTCTATTGAT
TTTAAGCTGAATAGTCTCAACGCAATTATAGGTCCGACTGGGTCAGGAAAGTCTTCGCTATTACTTGGACTATTG
GGAGAATTGAACCTTCTTTCTGGAAAAATATACGTACCTACAGTTGAATCCCGCGACGACTTAGAGATTGGTAAA
GACGGAATGACGAATTCAATGGCATATTGTTCTCAAACTCCGTGGTTGATTAGTGGAACAATTAAAGATAACGTT
GTTTTTGGAGAAATCTTCAACAAACAAAAAATTTGATGATGTAATGAAATCCTGTTGTCTTGACAAAGATATCAAA
GCAATGACAGCTGGCATAAGAACAGACGTGGGTGATGGAGGATTTTCCTTATCTGGCGGACAGCAGCAAAGGATT
GCTTTAGCCAGAGCAATTTACTCCTCTTCCAGGTATTTGATCCTTGATGATTGCTTGAGTGCAGTAGATCCTGAA
ACTGCACTTTATATATATGAAGAGTGTTTATGCGGCCCCATGATGAAAGGAAGGACCTGCATCATTACGAGTCAT
AATATTTCTTTAGTTACGAAACGGGCTGACTGGCTTGTGATTTTAGATCGTGGCGAGGTGAAATCACAGGGTAAA
CCATCGGACCTCATTAAATCTAATGAGTTTTTGAGGGAAAGCATAAACAATGATTCAAAAAATACAACTCACAAT
CAAATTGACTTGAAAAGATCAACAACATCAAAGAAAACTAAGAATGGAGATCCCGAGGGAGGGAACTCGCAAGAC
GAAGTGTGTGCTGAAGTTGAAAATTTTGAGGAAACAAAAAATGGAAGGATCAGTTAAATTTTCAGCTTACAAATGG
TTAGCAGACTATTTCGGGGGACTGGGAGTTGTTTTTGTTTTCACCTCGTCCTCCATCCTTATTCATGGAATCACA
CTGTCTCAGGGCTTTTGGCTCAGATACTGGCTGGACACTGGATCTTCAGGGAGTAAGTCTACCTGGCTTTATAGA
ATAGTTGAGGGTCACTCTAACATCTATTTCTTACTGACTTATATTATCATAGGTCTTGTTTCTTCATTTTTAACT
TCTGGTAAAGTTTGGATAGCAATAATTTCAGGTACCAACGTCACCAAGAAAATATTTGCGAAGCTTCTATCCAGT
ATCTTATATGCCAAGTTACGTTTTCATAATGTCACGCCGACTGGAAGAATAATGAACAGATTTAGCAAGGATATG
GATATTATTGATCAACAATTGATCCCTAATTTCGAAGGACTCTCTTATAGCGTCGTTGTTTGTCTGTGGATTATA
CTTTTAATTGGATATGTTACTCCTCAATTTCTGTTATTTGCTATTCCTTTATGCGCTCTTTATTATACCGTATGT
ACGTTATATCTTCGTGCATCTAGAGAGTTGAAGAGAATAGATAACATCAATATCTCTCCAATACACCAGTTATTC
GCTGAAGCCATCAAAGGAGTAACTACAATTAGAGCATTAGCAGATGAGCGTAGGTTTATCACTCAATCTTTGGTT
GCAATTGACAGAAGTAATGCTCCATTTTTTTATCTCAATATGGCCACCGAGTGGATCACATATAGAGTGGATATA
ATTGGGACACTTGTTCTTTTTAGTTCTTCTGTAATGATCATAATGAAGGCCTCATAG

>YMR005W 0.82 Cold
ATGGCAAATTCGCCGAAAAAGCCATCTGATGGCACTGGAGTATCAGCGTCAGACACGCCTAAATATCAACATACC
GTCCCAGAAACGAAACCAGCATTTAATTTGTCACCAGGTAAAGCTAGTGAGCTATCACATAGCCTTCCGTCGCCT
AGCCAGATAAAATCAACCGCACATGTATCTTCAACTCACAATGATGCGGCAGGTAATACGGATGATTCTGTTCTT
CCTAAAAATGTATCACCCACAACTAATTTGAGAGTTGAAAGTAATGGAGATACAAACAATATGTTCTCTAGCCCT
GCTGGACTAGCTCTACCAAAAAAGGATGATAAAAAAAAAAACAAGGGTACGAGTAAAGCAGATTCTAAAGATGGC
AAAGCATCCAACTCCTCAGGACAGAATGCACAACAACAATCAGACCCAAATAAAATGCAAGATGTCCTTTTTTCC
GCAGGTATCGATGTTAGGGAGGAGGAGGCTCTTCTAAATTCATCTATTAATGCCTCAAAATCCCAAGTTCAAACA
AATAACGTTAAGATCCCCAACCATTTACCATTCCTTCACCCGGAACAAGTTTCCAATTATATGAGGAAAGTCGGA
AAAGAGCAAAACTTCAACCTGACCCCTACAAAGAATCCTGAAATTTTGGACATGATGTCAAGTGCCTGCGAAAAC
TATATGAGAGATATCCTAACAAATGCCATTGTCATCTCCCGACATAGAAGAAAAGCAGTCAAGATAAATTCTGGT
AGAAGAAGTGAAGTTTCTGCGGCTTTAAGAGCCATTGCACTAATTCAAAAAAAAGAAGAAGAAAGGCGTGTGAAA
AAAAGAATTGCGTTGGGACTCGAGAAGGAAGATTATGAAAATAAGATTGATTCCGAAGAGACGTTACACAGAGCA
TCGAACGTTACGGCTGGCCTTAGAGCAGGTAGTAAAAAACAGTATGGTTGGCTAACTTCATCAGTAAATAAGCCG
ACGTCCTTGGGAGCAAAATCTTCAGGCAAAGTCGCCTCCGACATCACGGCTAGAGGAGAAAGTGGGCTAAAGTTT
AGAGAAGCTAGAGAGGAGCCTGGTATAGTAATGAGGGATTTACTCTTTGCTCTCGAAAATAGGCGCAACAGCGTT
CAGACTATTATTTCAAAGGGTTATGCCAAGATCAGAGATTAA

>YJR093C 0.79   Cold
ATGAGCTCCAGTGAAGACGAAGACGACAAGTTCTTGTATGGTTCCGACTCCGAATTAGCACTACCTTCATCTAAA
CGATCAAGAGATGATGAAGCAGACGCAGGTGCGTCCAGTAATCCTGATATAGTTAAAAGGCAAAAATTCGACTCT
CCCGTGGAAGAAACTCCAGCTACTGCCAGAGATGATCGTTCTGATGAAGATATCTACTCTGACTCCTCAGATGAC
GATAGTGATTCTGACCTAGAGGTTATCATAAGTCTGGGTCCTGACCCTACTAGGTTAGATGCAAAACTACTCGAT
TCTTATTCTACCGCAGCGACATCTTCAAGCAAAGACGTAATTAGCGTAGCTACAGATGTATCCAATACCATCACA
AAGACATCAGATGAAAGACTAATAACAGAAGGAGAAGCAAATCAAGGTGTAACGGCAACGACCGTAAAAGCTACA
GAGAGCGATGGAAATGTACCGAAAGCAATGACTGGTTCTATAGACCTGGATAAAGAGGGAATCTTTGATAGTGTT
GGCATAACGACAATAGATCCTGAAGTATTAAAGGAGAAACCCTGGAGGCAACCGGGGGCCAACTTAAGTGATTAT
TTCAATTACGGTTTTAACGAATTTACCTGGATGGAGTATTTACATAGACAGGAAAAACTACAACAAGATTATAAT
CCTAGGAGGATCCTAATGGGCCTATTATCCCTCCAACAGCAAGGGAAGTTGAATTCCGCGAATGATACAGACTCA
AACCTCGGTAATATAATTGATAACAACAACAACGTAAACAATGCAAATATGTCTAATCTGAACAGTAATATGGGT
AATAGTATGTCTGGAACACCAAACCCTCCCGCTCCACCAATGCATCCAAGCTTCCCACCCTTACCTATGTTTGGT
AGCTTTCCACCATTCCCCATGCCAGGTATGATGCCACCCATGAACCAACAGCCTAATCAAAATCAAAATCAAAAT
TCGAAATGA

>YKL012W 0.82   Cold
ATGTCTATTTGGAAGGAAGCTAAAGATGCAAGTGGAAGGATATATTATTACAATACTTTGACAAAGAAATCTACG
TGGGAGAAGCCCAAGGAACTAATTTCTCAGGAGGAGCTACTTCTTCGAGAAAATGGCTGGAAGGCGGCCAAGACG
GCAGACGGCAAAGTATACTATTATAATCCAACAACAAGAGAAACCAGCTGGACTATTCCGGCCTTCGAGAAGAAA
GTAGAACCCATCGCAGAACAAAAACATGATACAGTATCCCATGCACAGGTTAATGGAAATAGAATAGCCCTTACG
GCTGGAGAAAAACAAGAGCCGGGACGAACTATAAACGAGGAGGAAAGCCAATATGCTAATAACTCTAAACTGCTT
AATGTCAGGAGAAGGACTAAAGAAGAAGCAGAGAAGGAATTTATTACCATGCTGAAGGAAAATCAAGTAGACTCT
ACTTGGTCATTCAGTAGAATTATTTCAGAACTGGGGACCAGAGATCCAAGGTATTGGATGGTCGATGATGACCCC
TTATGGAAGAAAGAAATGTTTGAGAAATATCTTTCCAATAGATCAGCCGATCAACTTCTTAAGGAACACAATGAA
ACAAGCAAATTCAAAGAAGCCTTTCAGAAAATGTTGCAAAACAATTCTCATATAAAATATTACACCCGTTGGCCT
ACCGCAAAGAGACTAATTGCCGACGAACCAATATACAAACACTCCGTGGTCAATGAAAAGACAAAGAGACAGACC
TTTCAAGATTATATAGATACCCTCATCGACACTCAGAAAGAATCAAAAAAAAAATTGAAAACACAGGCCCTAAAA
GAACTAAGAGAGTATTTAAACGGTATTATAACAACATCATCCTCTGAAACTTTCATAACCTGGCAGCAGCTTTTA
AATCACTATGTTTTTGATAAGAGTAAGAGATATATGGCGAACCGGCACTTCAAAGTCTTAACCCACGAAGATGTT
TTAAACGAGTATCTGAAAATAGTAAATACGATTGAAAACGATCTTCAAAACAAACTAAATGAGCTCCGACTGCGC
AATTATACCAGAGACCGTATTGCTAGAGATAACTTTAAAAGCTTATTAAGAGAAGTGCCAATCAAAATCAAAGCA
AATACTAGATGGTCAGATATTTATCCTCATATAAAGTCTGATCCGCGCTTTTTACATATGCTTGGAAGGAATGGC
TCGTCCTGCCTTGATTTATTTTTAGATTTTGTTGATGAACAAAGGATGTACATCTTTGCACAAAGATCAATAGCC
CAACAGACGTTGATAGATCAAAATTTTGAATGGAATGATGCCGATAGCGACGAGATCACCAAGCAAAACATAGAA
AAAGTTCTGGAAAATGACCGGAAATTTGACAAGGTGGATAAAGAAGACATCAGTTTGATTGTTGATGGTTTGATA
AAGCAAAGAAACGAAAAGATACAACAGAAACTCCAAAATGAGCGTAGGATATTGGAGCAAAAGAAGCACTATTTT
TGGTTACTTTTGCAAAGGACATATACAAAAACCGGTAAGCCCAAGCCTAGTACGTGGGATTTAGCTTCCAAAGAG
CTTGGCGAATCTCTTGAATACAAGGCACTAGGCGATGAAGATAACATAAGAAGACAAATTTTCGAGGATTTTAAG
CCTGAAAGCTCTGCACCGACTGCCGAAAGCGCTACTGCAAACTTAACGTTGACCGCGTCAAAAAAGAGGCATTTA
ACTCCGGCTGTGGAATTGGACTATTGA

>YLR196W 0.8Cold
ATGATTTCTGCTACTAATTGGGTTCCAAGAGGGTTTTCCTCGGAATTTCCTGAAAAGTATGTTCTGGATGATGAG
GAAGTGGAAAGAATTAACCAATTAGCACAGTTGAATTTGGATGATGCTAAAGCTACTTTGGAAGAAGCAGAAGGC
GAATCAGGTGTTGAAGATGACGCCGCAACAGGTAGTTCTAATAAGTTGAAGGATCAGTTGGATATAGATGATGAT
TTGAAAGAATATAACCTGGAAGAATATGACGATGAGGAAATTGCAGATAATGAGGGTGGTAAAGACGTTTCCATG
TTCCCGGGTTTAAGTAATGATAGCGATGTTAAATTCCATGAGGGTGAAAAAGGTGAAGATCCATATATTTCATTG
CCTAATCAAGAGGATAGTCAAGAGGAGAAGCAGGAGTTACAAGTTTACCCATCCGATAATTTAGTTCTTGCTGCG
AGAACAGAAGATGACGTTTCGTATTTGGATATCTACGTTTACGATGACGGTGCTGGGTTCCACAGTAGCGATATT
CCCGTTGAAGAAGGTGATGAAGCTGACCCAGATGTTGCTCGTGGATTGGTTCGTGACCCTGCACTTTACGTTCAC
CATGATTTAATGTTGCCTGCTTTTCCACTATGTGTTGAGTGGCTTGACTACAAGGTTGGATCCAATTCCGAAGAA
GCAGCCAATTATGCTGCCATTGGTACGTTTGATCCACAAATTGAAATTTGGAACTTGGACTGTGTTGACAAGGCC
TTTCCCGATATGATTCTGGGTGAACCTCTTGATAATTCAATGGTGTCTTTGAAAAGCAAAAAGAAGAAGAAGAAG
TCTAAGACTGGACACATTACTACTCATCATACCGATGCTGTTTTATCCATGGCACACAACAAATATTTCCGTTCC
GTTTTAGCATCCACCTCTGCAGATCATACCGTAAAACTATGGGATTTGAACTCAGGTAACGCAGCCCGCTCCTTA
GCTTCCATTCATTCAAACAAAAATGTTTCATCATCTGAATGGCACATGTTGAATGGTTCCATTCTTTTGACCGGT
GGTTATGATTCTCGAGTAGCTTTAACAGATGTTAGAATTTCTGACGAAAGTCAAATGAGTAAATACTGGTCTGCC
ATGGCAGGTGAGGAAATAGAAACTGTTACATTTGCCAGTGAAAATATAATATTATGTGGTACTGATTCTGGTAAT
GTGTATTCTTTCGATATTAGAAACAATGAAAACCGTAAACCAGTTTGGACATTGAAGGCACACGATGCTGGTATC
TCCACATTATGTTCAAACAAATTCATCCCTGGTATGATGAGTACAGGGGCCATGGGTGAAAAGACTGTCAAATTA
TGGAAATTCCCCTTGGATGATGCTACGAACACTAAGGGCCCAAGCATGGTTCTGTCTCGTGATTTCGATGTCGGA
AATGTATTGACATCGTCATTCGCTCCAGACATCGAGGTAGCGGGTACCATGGTCATTGGTGGTGTCAATAAAGTT
TTGAAACTATGGGACGTTTTTACCAATAGATCTGTACGTAAGAGCTTTAAAAGTGAACTAGAGAATGTTCAAGCA
AGAGCAAAAGAAGAAGCCCAAAAAATAGGAAAAAGTTCAAGAATCGCCAGAAAATACACCAGTAACGATAACCCA

GATACTGTTATAACCATTGATGATCAAGGTGAAGATGAAGAAGAAAGAGAAGGTGGAGATGAGCATGATGACATG
GCATAA

>YLR108C 0.77 Cold
ATGTCTGGCCAAAAGGGCGAGATCGTGGTTTATACAAAAGAGTTAGAAACTACCCCCGAGCTTTTACCAAACCAT
GAAGTTTTCAAGATTAGGATTGGCCAAAAGCTATTTGAGATCTCAGGTGCTACATTGAATTCGGATGCGCCCAAT
TTTTTCACCCAATTCTTCAATACGCACGATAAAAATACGATTCTATTTGTTGATAGATCGGAAGACGTGTTCATC
ATAATATACAGACATTTACAAGGTTATTTTCCCGATATTAAGAACGAAGTACAATTTACCTGTCTTTTCGCGGAC
GCATTATATTTTCAGTTACCCAAGTTAGTTAAACTCATAAAAGAATACGACTACCACTTCACTAATATTGGAGGT
GTTCCCTTTAAGGTACCAAAATCATTATTCCATGAAGAAGGGAACCGGTTGAACTATTTTGAAACGATTTCTCGC
ATATCTTATGAGGAAATTGAGAAGTGGGAGTCAAATAAGAAGCCGGGCTTTCCACCTTTATTACCACCCTCATAT
ATTGCAAGGTCCCCAGAATTTTTCCGAGATATATTATCTTTACTGGGCGGTTCCAAATTAGAACTGTCTGAGGAA
AGAACCGCATCTCTGATAAAAGAGTGTCGTTATTACCGTCTCAACAGGTTGGAGCAGGAATTAGTGCGGGCAAAA
ATTATATATAATCCCTTAACTAATTGTCAAGAAGTTTGCATAGCTCTTGATAGCGTCTCCAAGAAGGGTGTAACC
ATTGAAAGGTTAACATCACTTCATACCGGAAACCAATCTTTAGCGGTAAGCAGTTGTCTCAATGGAACTGAGGGT
GAAAAAGCCGCTACTGGATTTCATAAAACCGAGACTGATTCAGGCAACAATGATGAATATGAACCTCCAACAAAA
AAGGTAAAACATTGTATTGAAAGACACTGGAGCATGTTAAAATACCAAAGGCCTTATATCGATACAGTTTCGCAC
GATCTTATTTTCCAATTACACTCTAACCAGTGCAAAATAATATTCAACAAAAAGAATAAGACTGTTCATGTAGAC
CTATCCAGAGAGGCTGCAGTGTTGTTTGAAAACAAGTTCTCGGATGTTTTATTGGAAAACCCAGATTTTAAAATT
GATCTGTCCGAATATAAGGTCAAACTTCGTGACAGTCAAATGCAAGTTGAATCCCACTTAATTATTCCTGCATGT
GTCTCCATTTGTGACTTGACGGTAAATGGCGCAAAATGCTGTAATATTTTCTCATTAGTTAACGACTCCAAATGT
AAAGGGAGGGTCTTGGATTGCACCAATTTGAAAGTTTTGAATTGCGTTCATGGTTTGAAACTACACTTATCGAAA
TCCATGTGGAAGCTAGGTACAAATAATGGAAGAATCATTCTCGTTGCTGTAAAAGCCGAAACCTTCTCTGGGACG
AAGGAATATTGCAAAATGATTGATTTTCTGTGA

>YMR288W 0.77 Cold
ATGAGTCATCCGATTCAATTTGTAAACGCTAATAATAGCGATAAGAGCCACCAATTAGGCGGTCAATATTCTATT
CCTCAGGATTTGAGGGAAAACCTGCAGAAAGAAGCTGCACGTATTGGTGAGAACGAAAAAGATGTGCTACAGGAA
AAAATGGAAACTAGGACAGTGCAAAATCGAGAGGATTCATACCACAAAAGAAGGTTTGATATGAAGTTTGAGCCG
GATTCCGATACACAAACTGTTACGTCTTCAGAAAATACCCAAGACGCTGTCGTGCCAAGGAAAAGAAAATCTCGT
TGGGATGTAAAAGGGTACGAGCCACCTGATGAATCTTCAACAGCGGTCAAAGAAAACTCTGATAGTGCTTTGGTT
AATGTAGAAGGAATCCATGATTTGATGTTTTTTAAACCTTCTGACCATAAATATTTCGCCGACGTTATATCCAAA
AAACCCATAGATGAGTTAAATAAGGATGAAAAGAAAGAAAGAACCCTGTCGATGTTACTTCTGAAAATAAAAAAT
GGCAATACAGCGAGTAGGAGGACTTCTATGCGGATTTTAACCGATAAAGCTGTTACATTTGGTCCAGAAATGATA
TTTAACCGCTTATTGCCCATATTACTAGACAGAAGTTTGGAAGACCAGGAAAGACATCTAATGATCAAGACCATA
GATCGCGTCCTTTACCAGCTAGGAGATTTAACAAAACCTTACGTTCACAAAATACTTGTAGTAGCTGCCCCATTG
CTAATAGATGAAGATCCAATGGTTCGTTCAACAGGCCAGGAGATAATCACCAATCTGTCTACAGTCGCTGGCCTA
AAAACCATTCTAACAGTAATGAGACCTGATATTGAGAATGAGGATGAGTATGTTAGAAATGTAACTTCAAGAGCG
GCGGCTGTGGTGGCAAAAGCCCTTGGTGTTAATCAATTGCTTCCTTTCATCAATGCAGCATGCCACTCACGGAAG
TCTTGGAAAGCAAGGCATACTGGCATAAAAAATTGTGCAGCAAATAGGTATACTTTTGGGAATAGGTGTCTTGAAT
CATCTTACTGGGCTGATGAGTTGTATCAAAGACTGCCTAATGGATGATCACGTTCCGGTGCGAATTGTTACAGCA
CATACTTTATCAACACTAGCAGAAAATTCTTATCCATATGGTATCGAAGTTTTCAACGTTGTACTGGAGCCATTA
TGGAAGGGTATAAGAAGTCATCGTGGTAAAGTGCTATCTTCTTTTTTAAAAGCTGTTGGATCTATGATCCCATTG
ATGGACCCAGAGTATGCCGGTTATTATACCACAGAAGCAATGAGAATAATTAGACGTGAATTCGACTCTCCCGAT
GATGAAATGAAAAAGACCATTCTTTTAGTATTACAAAAATGTAGCGCTGTAGAATCCATAACGCCAAAATTTCTG
AGAGAAGAAATCGCGCCAGAATTCTTTCAAAAATTTTGGGTCAGACGAGTGGCATTGGATAGACCATTAAACAAA
GTTGTTACATATACTACTGTTACATTAGCAAAGAAATTAGGTTGTTCTTATACCATTGATAAATTATTAACACCA
TTGAGAGATGAAGCTGAGCCGTTCAGAACAATGGCTGTCCATGCTGTTACTAGAACAGTAAACTTATTAGGGACA
GCAGATTTAGATGAGAGATTAGAAACGAGGCTTATTGATGCGCTCTTGATAGCATTTCAAGAACAAACAAATAGT
GATTCTATAATCTTTAAAGGGTTTGGGGCAGTGACGGTGTCACTTGACATCCGTATGAAACCGTTTTTAGCACCC
ATTGTGAGTACGATTTTAAATCATTTAAAGCATAAAACTCCCTTGGTTCGTCAACATGCAGCAGATCTATGTGCC
ATACTAATACCAGTCATCAAGAACTGTCACGAATTCGAAATGTTGAATAAATTGAACATTATATTGTATGAATCC
TTAGGCGAAGTATATCCAGAGGTATTAGGTTCTATAATCAACGCTATGTACTGTATTACTAGCGTAATGGATTTG
GATAAACTACAACCTCCTATTAATCAAATTTTACCAACCTTAACTCCAATTTTAAGAAACAAGCATAGAAAAGTC
GAAGTAAACACTATCAAATTTGTTGGACTTATTGGTAAATTAGCACCTACCTATGCACCTCCTAAAGAATGGATG
AGAATATGTTTTGAACTATTAGAACTTTTGAAAAGTACAAATAAAGAAATAAGAAGATCAGCAAATGCCACGTTT
GGATTCATTGCTGAAGCAATTGGACCTCACGATGTTCTTGTTGCGTTATTGAACAATTTAAAGGTTCAAGAACGT
CAGTTACGTGTATGCACGGCTGTTGCTATCGGTATCGTTGCTAAAGTGTGTGGTCCTTACAACGTGCTGCCAGTA
ATCATGAATGAATACACAACACCAGAGACTAATGTTCAAAACGGTGTTCTCAAGGCCATGTCATTCATGTTTGAG
TACATTGGTAACATGTCTAAGGATTACATATACTTCATAACACCATTATTAGAAGATGCACTCACCGATAGGGAT
TTAGTTCATCGTCAGACAGCGTCGAACGTAATAACCCACTTAGCTTTAAACTGTTCAGGTACAGGTCACGAAGAT
GCCTTCATTCATTTAATGAATCTTCTAATACCCAATATTTTTGAAACTTCACCACATGCTATTATGCGTATTTTA
GAAGGTTTGGAGGCACTGAGTCAAGCTCTTGGCCCTGGACTATTCATGAACTACATATGGGCAGGATTGTTCCAT
CCAGCGAAGAATGTAAGGAAAGCATTTTGGAGGGTGTATAATAACATGTACGTAATGTACCAGGACGCCATGGTA
CCTTTTTACCCCGTTACACCAGACAACAATGAAGAATATATAGAAGAACTGGATTTAGTTCTGTGA

>YJL108C 0.78 Cold

ATGATTGTTTCATTTGGTGATGCAACAACAAGAACTTCCGAAGTTCAATTGGTTAGGTGTACTCAAGGTCTAAAC
CTTTGGAAGCTGCATCAAGTTCATGCCGTTTACAAAAGAGTTGTCCATGATACCTTAGGTGCCGATGAAGGTAAT
GCTTTACTCGATCAAATTTTGGCTGACACGAATCTATATCCACCTTGGATGTGTGTTCTTTTATATGCATTTTGT
TCCGCTATGGTCACTCCGTATGCCTTTGGTGGCGATTGGGTAAACCTGGCAATCTCATTTTTTATGGGGCTTTGT
GTGGGGTCATTACAATTTATTCTTTCACAAAAGTCATACATGTATTCAAACGTCTTCGAAATTTCTGCATCTATT
GTCGTTAGCTTCTGCGGTAGAGCTTTTGGCTCTATCCCTCGTTCTCACATCTGCTTTGGTGCAGTTACACAAGGC
TCATTGGCATTAATCCTGCCAGGTTATATTATTCTGTGTGGTGCACTAGAACTGCAAAGTCGAAGCCTAGTTGCT
GGCGCGGTCCGTATGTTCTACGCCATTATTTATTCTTTGTTTTTGGGTTTTGGCATAACATTGGGCTCAGCTTTG
TTTGGATGGATGTATCACAATGCCACTAACGAAATTTCATGTCCTCAATTAATTTCTCCGTGGTTCAGGTTCCTC
TTCGTTCCAGCGTTTACCATTAGTATCTCGTTATTAAATCAAGCACACATCTCACAACTGCCAGTTATGGTCTTC
ATTTCTTGCACCGGTTATGTCGTAACATATTGGGCCGGTAAGCATTTTGCGAATTCGACAGAGTTTACTGCCGCC
TTAGCTGCATTTGTCATAGGTGTTTTAGGTAATCTATATTCCAGAATTTGGAAAGGTTTAGCGGTATCAGCTATG
CTTCCCGCAATCTTCGTTCAAGTGCCATCTGGTATTGCTTCACAAAATTCGTTGTTGTCTGGGTTGCAAAGTGCA
AATACAATTGTTAATGCTAATGAAACCATTACGACAAGCACCTCCGATCCATCGTCCTCAATGTCATTTGGTATG
ACAATGATTCAAGTTTGTGTCGGTATTTCTGTGGGACTATTTGCTTCTTCACTCTTTGTCTACCCCTTTGGTAAA
AAGAAAACTGGTCTTTTCAGTTTATAA

>YDR259C 0.75 Cold
ATGCAAAACCCTCCGTTGATTCGTCCCGATATGTATAATCAGGGAAGCAGCTCAATGGCTACTTATAATGCCTCT
GAGAAGAATCTAAATGAGCATCCTTCTCCGCAAATTGCACAGCCCAGCACGTCCCAAAAGTTACCTTATAGAATA
AATCCTACAACCACTAATGGGGACACCGACATATCTGTTAACAGCAATCCTATCCAGCCTCCTTTGCCAAACTTG
ATGCATCTATCTGGTCCGTCTGACTATAGATCGATGCATCAAAGTCCTATACATCCATCTTATATCATCCCTCCG
CATTCAAATGAAAGAAAACAATCAGCTTCTTACAACAGACCTCAAAATGCTCATGTTAGTATTCAACCTTCCGTG
GTATTCCCCCCTAAAAGTTATTCCATATCTTATGCACCTTATCAAATAAATCCCCCTTTACCAAATGGACTTCCG
AACCAGAGCATATCTTTGAATAAGGAGTATATTGCAGAGGAGCAACTATCAACCCTCCCATCTCGCAATACCAGT
GTTACTACTGCACCTCCTTCTTTTCAAAACAGTGCTGATACCGCTAAAAATTCAGCTGATAATAATGATAATAAT
GATAATGTAACCAAACCTGTTCCTGATAAAGACACCCAACTCATAAGTAGTTCAGGCAAAACTTTAAGAAATACT
AGAAGAGCTGCTCAAAATAGAACCGCTCAAAAGGCATTTAGACAAAGGAAAGAAAAATACATCAAGAATCTCGAA
CAAAAATCAAAGATATTTGACGATTTACTAGCAGAAAATAATAACTTCAAATCATTAAACGATTCATTAAGAAAT
GACAACAACATTTTAATAGCTCAGCATGAAGCTATAAGGAATGCAATTACTATGTTAAGAAGTGAGTATGATGTC
TTATGTAACGAAAACAACATGTTGAAGAATGAGAATAGTATAATAAAAAATGAACACAACATGTCAAGAAATGAA
AATGAAAACCTAAAACTTGAGAATAAACGCTTCCACGCTGAATATATACGAATGATCGAGGATATTGAAAATACT
AAAAGAAAGGAACAAGAACAACGAGATGAAATAGAGCAACTAAAAAAAAAAATAAGATCCCTGGAGGAAATAGTA
GGGAGACACTCGGATAGTGCCACGTAA

>YOR310C 0.79 Cold
ATGGCTTACGTTTTAACTGAAACTTCAGCTGGTTATGCTCTTTTAAAAGCTTCCGATAAGAAAATCTACAAATCT
TCAAGCTTAATTCAAGACTTGGATAGCTCTGACAAAGTCTTGAAGGAATTCAAGATCGCCGCCTTTTCCAAGTTC
AACTCTGCAGCTAACGCTTTAGAGGAAGCCAACTCCATTATTGAAGGTAAAGTTTCTTCCCAATTAGAAAAACTT
CTGGAAGAAATTAAAAAGGACAAGAAGTCGACCTTGATTGTTAGTGAAACTAAACTGGCAAACGCTATTAATAAG
TTGGGATTGAACTTCAATGTTGTTTCCGATGCTGTAACCTTAGATATCTATAGAGCTATCAAAGAATATTTGCCA
GAATTGCTGCCAGGTATGTCTGATAATGATCTAAGCAAGATGTCTTTGGGTTTAGCTCATTCCATCGGTCGTCAC
AAATTAAAATTTTCTGCTGACAAAGTTGATGTTATGATTATTCAAGCAATTGCTTTATTGGATGATTTAGATAAA
GAATTAAACACATATGCCATGAGATGTAAAGAATGGTACGGGTGGCACTTTCCTGAGTTAGCTAAGATTGTTACT
GATTCCGTCGCTTATGCGAGAATTATTCTAACAATGGGTATCAGATCCAAGGCTTCTGAAACAGATTTGAGTGAA
ATCTTACCAGAAGAGATTGAAGAACGTGTCAAAACCGCTGCTGAAGTGTCTATGGGTACTGAAATCACTCAAACC
GATCTAGACAACATTAATGCTTTAGCTGAACAAATAGTCGAATTTGCCGCTTACAGAGAACAGTTGTCCAATTAC
TTGTCTGCAAGAATGAAGGCTATTGCTCCAAACTTGACTCAGTTGGTTGGTGAATTAGTTGGTGCTAGACTTATC
GCTCATTCAGGTTCCTTAATTTCCTTGGCCAAATCACCAGCTTCTACCATCCAAATATTAGGTGCCGAAAAGGCT
TTATTCAGAGCTTTAAAGACCAAGCATGATACACCAAAGTATGGTTTGCTATATCATGCCTCTCTTGTTGGTCAA
GCTACTGGTAAGAACAAAGGTAAGATTGCAAGAGTTCTGGCTGCTAAGGCCGCTGTCTCATTGCGTTATGATGCT
TTAGCTGAAGATAGAGACGATTCAGGTGACATTGGTCTAGAATCAAGAGCCAAAGTAGAAAATAGATTATCCCAA
TTAGAAGGTAGAGATTTAAGAACTACTCCAAAGGTTGTTCGTGAGGCTAAGAAGGTGGAAATGACTGAAGCTAGG
GCCTACAATGCAGATGCTGATACCGCAAAGGCTGCTTCTGATTCTGAATCCGATTCTGATGATGAAGAGGAAGAA
AAGAAAGAAAAGAAAGAAAAGAAGAGAAAGAGAGACGATGATGAAGATTCCAAGGATTCTAAAAAAAGCTAAGAAG
GAAAAGAAGGACAAGAAGGAAAAGAAGGAAAAGAAGGAAAAGAAGGAGAAGAAAGAGAAGAAAGAGAAGAAGGAA
AAGAAGTCCAAGAAAGAGAAGAAAGAGAAGAAATAA

>YNL011C 0.79 Cold
ATGAACGTCGTTGTGTGCTCCGGTGGGACTGCCACAAATTCATTGACTCCTTGCTTTTCTAATATTTCTATCTTA
AAAGGACATGAGCTGACGTATATATTGCCAATATCAGATAATGGCGGGTCCACAAGTGAAATTCTACGAATAGTT
GGTGGCCCAGCAATTGGTGATATCAGATCTAGAATAGTGAGACTACTTCAAGATGAACAGCTTGTCGAGTTGTTT
GGCCATAGGCTACCTAATGATAAGTTATTAGCTAAGAAAGAATGGAATGAAATAGTGGAAGGTTCGCATCCCATA
TGGAAAAATATTTCGATAGAAGTTAAGGAAATGTGTCGTTCTTTTATTATCCATATGCAAGCAGAACTCTTGAAA
AAAATCAAACACTCGAATCCATTTCAGTTTGAGAGTGCATCCATTGGAAATTTTTTCTTAACTGGTGCTAGATTG
TTTTTAGGTTCTCTAGACGCATCTATTGAACTAATGATGAGGATAGGAAGGTGCAGTCCTCTAGTTCATGTTATT
CCATGTATAAATACCAATCACACACATCATATCTCGGCTTTGTTGACGAATGGTGAGATGATAACTGGCCAATCA

CAAATATCGCATCCCTCGAAATCTGTTCCTAAAGATAATAGCATAGCACATTCAGCTAAATTTATTCATTTACTT
GGCTCTTATGACGACCATTTGAAAATTCTCCTCGACGATGAGGAAGAAGAAGCGGAAGAGGAGTATGCCAATCCT
ATCTATATTCTTCCAGAACTGAAAAACTCCCAGTTGCATTTTGACAAGCTTGATGAATCACAGAATCTACCTGCT
CCGGTACATAGGATATTGTATATAAACCCATATGGCGAAGAGATAAAACCAATGGGAAATCCAAGAGCTATTTCC
AAGGTGAAAAAAGCTGACATGGTCGTTTATTCTATCGGTTCTTTAATGACAAGTTTACTCCCAATCCTTATTCTT
GGTAATCTTGCTGAAGTAATTTTGGAATCTAATAATACGAAAAAAGTTTTGCTCATAAATAATAAATATGATAGA
GAGGTTTTTGGTTTAGACGGTTTACACTACGTTCAAATGATCATAGATTCTATGAGTAGGGCAATAGCAGGCTAT
AGGCAATCTAAGGGTGTCCATTCAGAGAATGATGACTTTGAATGGCAAGATTTTATTACTGATATTGTTTACCTA
AAAAATGGTGAAATTGAAATTGATGAAACGATATTCGAGAAGCATAGTATTAGATGCCACCAGATCGCGTCTTCG
GATAAGATGGAAAGTGAAGAACTTGAAAAGGTTCTGAATCAAATTGGCCTTAAAAACTGA

>YKL025C 0.77   Cold
ATGGACAAAATCAATCCTGATTGGGCGAAGGATATTCCGTGCAGAAATATCACTATTTATGGCTACTGCAAAAAG
GAGAAAGAAGGTTGCCCTTTCAAACACAGCGATAACACTACCGCTACTACCATAAATGACGTTCCTCCTCCAATA
GATGTGGGTGAGGCTACAACTCCGACCATGACATCAGTTCCTAAGTTCAATGCTAAAGTATCCGCAAGTTTCACT
CCGATGACAGTCGGTAGTGACTCCTTAACCACTGTGACGAATACCACCTCCGCTGCTACAAATGCTACTGGCAAT
ATCGCCATGGCAGCTACCTCTGCTACTGCTTCTACAGTTAATCCGATGATTAATCCGATAGTTAATAGCTCGTTA
GTGAATAACAATAACAATAATAGTAATATAAGCATATCAATACCAACTACCGCTTCGAGTTCCAATTACGACCCC
TTCAATGCCCCCATTTTCACTCCGTCTTCAACCTCCTCAATTCACACTAATGCAAATGCACATTCTTTTCCATTT
CCCTCCATTGCAAATTCTGGTGGCATAAATATAAACGCCACTGATGATAATAGTAACAATATGAGTATGGCTAAT
AATGTGCCACCTCCTATGCAACCGCCACCCATAGAGAGTAGTAATCTTAAGTACCCACGTATTTATCCGCCTCCT
CACAGTCTTCTACAGTATCACCTATATGCACCTGAACAGCCATCATCATTGAAATCATTATTAAAGCCTAATGAA
AGGTCTGCAGATCAGCTTTTCATTCCAAACAATATTAGAGAAGATTTAACCAAGAAAAACTTATCGATTTTGCAG
GTTTTCCCCTCTTCAGGTAAAGTTATACCAAGTATTGTACAAGATTATTTTAATTTGGTTCCATTGAACTTCAAT
AATAACGATTTTTTAAATAAAACTACGCTCTTCAAAGTTTTTTCCAATTATGACGGTAAAGCCTACGTTTTGAAG
AGGCTTCCTAACATCGATAAGTCAATGAATCCAAACAAAATATCCAAAATATATCAGATATGGTCAAAAATTAAT
TGTACAAATTTGATAAAGTTTAGGGACATTTTTCAAACTACTAAATTTGGTGATCTGTCTATTTGTTTGGTCTTT
GACTACTACCCAAACTCGCTATCTTTGTATGATTACCACTTTGTTAATTTCCCTAAGTTTCCAATAACGAATAAT
TATTTATGGATATATTTAGTTCAACTCACCAATGTAATAAACTCTATCCATTCACAAAACTTGAGTATTGGCAAT
ACATTAAACTGGAGAAAAGTTTTGATTACTGGGGACCCAGGGAGAATCAAGTTATCACACTGCAATTTTATGGAC
CTTTTGTTCAATGATGATACTGATACCGTAGTATCTTCCGGCGGAAGTACCATAGAGGGACAACAACAGCTAGAC
TACAAATATTTAGGAGAGCTATTATTTAACCTATCCATTAATATTGAAAACTCTAATAACAACACTGCCCCTAAA
GAATATCGATTGGAGGAAATAACCCCTCAATCAATTGATGACATGAGACAGATCGATGATAAGTTCAAGGATGTA
CTCAAGTATCTGATATCAGACAACGGCGATTCCAAAAAGAGCATTCATGATCTTACTAGTCACTTTTATGATAAG
ATGTTCATGGTCCTGGAATCGTCACAAACCTATACAGAATACATGGAGTCTGTCTTATCAAGAGAACTAGAAAAT
GGCAGATTATTTAGGCTGGTCAACAAGCTAAATTGCATTTTTGGTAGAATCGAATCAAGAATAGACATAAATTGG
TCCGAATCTGGGACTAAATTCCCCATTATACTATTTTATGACTACGTATTCCATCAAGTGGATTCGAATGGGAAA
CCAATAATGGATTTAACTCATGTCCTAAGATGTTTGAACAAATTAGACGCTGGTATTCAAGAAAAGTTAATGTTG
GTAACGCCCGATGAGTTAAACTGTATTATTATATCCTATAAGGAGTTGAAGGACTTGATAGAATCCACCTTTCGA
TCCATCACCCAATAA

>YDR002W 0.82   Cold
ATGTCTAGCGAAGATAAGAAACCTGTCGTCGACAAGAAGGAAGAGGCTGCTCCAAAGCCACCATCCTCTGCTGTC
TTCTCCATGTTTGGTGGTAAGAAGGCCGAAAAGCCAGAAACCAAGAAAGACGAAGAAGATACCAAGGAGGAAACC
AAGAAGGAAGGTGATGATGCTCCAGAATCACCAGATATCCATTTTGAACCAGTGGTTCACCTGGAAAAGGTAGAT
GTTAAGACAATGGAAGAAGACGAAGAAGTTCTTTACAAGGTCAGAGCCAAGCTTTTCAGATTCGATGCCGATGCC
AAGGAATGGAAAGAAAGAGGTACTGGTGACTGTAAGTTCTTGAAGAACAAAAAGACTAACAAGGTTAGAATATTG
ATGAGAAGAGACAAGACCTTAAAGATTTGTGCTAACCACATCATTGCTCCAGAATACACTTTGAAGCCTAACGTT
GGTTCTGATAGATCTTGGGTGTATGCTTGTACAGCAGATATTGCAGAAGGTGAAGCAGAAGCCTTCACTTTTGCT
ATCAGATTTGGCAGTAAGGAAAATGCTGATAAATTTAAAGAAGAATTTGAAAAAGCTCAAGAAATCAACAAAAAG
GCTTAG

>YDR164C 0.81   Cold
ATGTCTGATTTAATTGAATTACAGAGGAACTATTTGATAGGGGTTTTGAATCAGATTGAGACGAAAAATAACTTG
AAGTTTCTTATTATCGATAAAACTGTGGAGACAATATTGAGTTATCTTTTCCTCACACCCCAAGAACTTTTAAAT
AATGTCACATCTGTTGATTTGATTGATTCCCCAACAAGAAAAGGACAATCCTCCATCGAGGCAATTTACATATTG
GAACCAACGAAATATAACATCAATTGTATCGATGCTGACTTCATGGTAAGACCACCTAAGTACAGAAGATGTCAC
ATCAGATTTTTACCAGGATTAACAAACCCTATATTTCAGTTTTTTCAATCAAAACGTTACATTGCTCAAAATTTG
GAGTCATTTAAGCCGATCGAACTAGGGTTTTTCGTCAAAGAATCTCAATTCTTCGAAACTTTACAAATGGAACAC
TCATTACAGGTTTTTTTCAACAACAACTGTAAGGCTTTGATTCCTACTAACGTGAGGAAGATTGTAGGCTCACTA
GTTAGTTTGTGTGTTATTACGGGGGAATATCCGATTGTTAGATATTCTGTATCTAATCCTGTGGAAGAGGAAGAT
GCCCGCAATGGAAACGCCGTAGTCAACGCAAATTCTTTAACAAGGTCTATTGCAAACGCTTTCCAAATAGCGATA
GATACATACGCTCGCAATAATCCAGATTTCCCTCCTCAAAACACTGAAAGACCGCGTTCCATTCTAATAATCACC
GATAGAACACTGGACCCTTTTGCGCCAATATTACATGATTTTAGCTATCAAGCAATGGCTTATGACCTGGTAGCT
AATGTGGACACACAAAAAGATATATATCATTATTCTGCCGAGAATGAAGCTGGTGAGCAAGAGGAAAAGGTTTCA
AAATTGGTAGATTTGTATGATCCTGACTGGATTGATTTGAAACATCAGCATATTATGGATGCCAACGAATATATT
CAAGGAAGAATCAAGGAACTGATTGCTAAAAACCCTCTGCTGGTTGATAGATCGAACGTAAAGAATACTACCGAT

CTGCTGAGTGTCGTAGCGCACCTGAAAGATTTTGATGAAGAAAGAAGAAGGCTGATTTTGCATAAGACACTGGTA
GATGAATGCCTAGGAGAGAACGCGGAAAGAAAATTAGCGGATATTTCTGCTATTGAACAAAACCTATCCGGATTT
GGAATGGATTTTAGTGGTGAGAAGATAAAGCATATTATCGATGATCTCTTACCAGCGTTAGCAATGAAGGAACCG
ACAATCTTAGATAAATTGCGTTACATTATTGCGTATGCTCTTTTCAGAGGTGGAATTATCGAGTTAGACTTCATT
AAATTATTGAACTTTATAGGAGTTACTCATGAACATGAAAATTTCCAGCAATATTTAAAAATATTCAGAAATTAC
GATTTAATTGATTTCAAATTGATCAAAGACAAACCGAAGGATAAACCATTTCAAAAGGAATGGTTTCATGATACT
TTAGTGAATGATCCAAATATCTATCACACTTCAAGGTTCGTTCCCGCTGTAGGGAATATTCTTTCAAAGGTTATA
GCGAATCCATTGTTATTGAGCGAACAATACTTCCCATATTTAAAGGACAAGCCAATTGAGTTATTGAATGAAGAA
GAATTCCAAGCAGGCTTAGCGAATACCTCTGCTAACTCCTCCTCATCCTTAAGGAATCCCCGTCACAAGGCAGCT
TGGACCACGAAAAGCTCTAATATAAAAAAAAACATACCTAGACAAAGATTCTTCTACTATGTTATTGGTGGTATA
TCAATTCCCGAAATTAAAGCTGCTTATGATCAATCAAACTTGAAGAACAGAGATATCTTTATTGGCAGCGACGAG
ATATTAACACCAACAAAATTCTTAGATGAAGTGGAACGTTTACAAAATCCTAGAGAATTTTTCAAATTCAAGGAA
GATCAGCGCCAGCAAGTAAACCCGCCTGATTTCCTTCTAAGGGAAATGAAACCGGTGGCACAACCAGTCTCTCAT
GTTCATTTAAAAAGCCAAGACAATAGTCCTAAGTCTGGTACTTCTAGTCCCAAAGCAGCAGGTTCTCTCAAGTCA
GAACCACCCGAAAAAGAGAAGAAACGTAGCAAATTCTCGAGGTTCTTGAAAAGAAAATCTCACCATGATAAATGA

>YNL084C 0.79   Cold
ATGCCCAAGTTGGAACAATTTGAAATAAAAAAATACTGGCAAATCTTCTCGGGTTTGAAACCAATAGAGAATAAG
GTAAACCATGACCAGGTTTTACCAATTCTTTATAATTCCAAATTAGATTCATCGGTTCTAAACAAGATTTGGTTT
TTAGCTGATATTGATGACGACGACAATTTAGACTTTGAGGAATTTGTAATTTGCATGAGATTAATATTTGATATG
GTTAATAAAAACATTAGCTCTGTTCCAGATGAATTGCCTGATTGGTTGATTCCAGGGAGTAAGGTGAATCTAATC
AAAGAAAGAAAGAAGCGCAAGCAAATAGAAAACGCAGACTTGCCTCCAAAGAAGGAAATCAAAGTAGATTGGTAC
ATGTCTCCAGATGATTTGAATCAATATGAAAAAATTTATAATAGCTGTGCAAAACTAACCGATGGTACTATTACA
TTCAACGAACTGTCAACAAAACTTTCCACAAAATTTTTTAACATCAGTAAGACAGATTTAAATAAGGTTTGGAGT
TTAATTAACCCACAAAATTTGCCATCAATTGATAGAGATCCTACTTTTTATTTTATTCACTGTTTAAGACAAAGA
AATGATTTGGGTGCTGAAATTCCAGCAAGTTTACCAAATTCTCTGGCGGAGGTGTGCAATAAAAAACAACTGAGC
TATGATTTACGGTCCTCTCAACCTCCTACAAAGAGAAAAGAAGAAGCTAACGAAGTGGACAATCTGCGTGACAAT
GGGCAGAACTCGAGTTCCGATAGCAGTGGCAGTAACGTGCTTTCAAATGAAGACAGTATAAAGCAAAAATATGCT
TCTCTGACAGATGATCAAGTTGCGAACATGAGAGAACAATTAGAAGGCCTTTTGAACTACAAAAAGAGTGAAAAA
ACTCAAGGAGGCTCAAAACTTTCTAAGCGGATTAACATCAGGTCGATAACTGATGACTTGGATAACATTGAACAG
CAGGTTGAAGTATTGGAGAATTACTTGAACAATAAGAGACACGAATTGCAAGCATTACAAGCAGAAATCAATTGA

>YGL259W 0.79   Cold
ATGCAATTGTTTTCCATCCTCTCTTTGTTGTCATCATTGATGTGTTCATTGACCGTTTTAGGGTCTTCCGCAAGT
TCCTATGTTAAGTTTCCTGTTCAAAAGTTTGCTGATATTATAAATATAGGCACACAGGATGTTTCTACCGTCTTC
AAAAGAAATGAGGTTCTTAACACCACAGTGATAAATGGAATAGGAGTGTATGTTGTCAAGATGGAAATCGGAACG
CCTCCACAAACGGTGTACCTACAGTTAGATACTGGCTCTTCAGATATGATTGTGAATAATGCTGACATTGCCTAT
TGCAAATCGATGTCTGACGGATCTGACTACGCTTCAACTGATAATTACGAACTCACGGCAACATTCACTGGGCCT
CGTTCTACTACCACCTCACCAGAGCTTATAACACTGTCCGCTCTTATAGGGGTAAATTCAATGCAAGAAACTCAT
CTACTTTTAAGAATAACGCGACTTTCTTCAATGACATATACGTATTAG

>YPL208W 0.81   Cold
ATGTCATCAGATGCGTTAAAAGCACTACTTCAGTGGGGTGCGTCATTTGGTGTAATAGTGCCAGAGGAACTCAAG
TTTTTATATACCGATCTTAAGGGCATTATTTGCGTATGTGAAAAAGATATAGACAATCCCAGCATCAAGATTCCT
CCCGAGATTGTCATTTCAAGGAATCTGCCCATGAAGTTTTTTGGGCTAAGTGAATCCACTAAGAATATCAACGGA
TGGCTGAAGTTGTTCTTCGCAAAAATAAAATTTGATAGGGATAATGATACTATCGTGGACAATGTTCGTGTGAAT
GATAAATTTAAACCTTACTTGGATGCATTGCCTTCCCGCCTAAATTCGCCCTTGGTCTGGAACCCAAGCGAGTTG
AAGCGTTTATCATCTACAAACATAGGGAATTCGATTCATGAAAAGTTCGAAGGTATATTTAAAGAGTGGTTTGAA
CTGGTCAGTTCTTCAGATATGTTTGACTTGGAAAGAGTGGCAGATGATGTGCAGACCTTCCATAATCTCGATGAG
TTGACATATGAGGCTTTGTATGAGAAGATTTTGAAGATAACAGAACTTCAAAGACCAACTATCTGGTATTCTTTT
CCTGCATTTTTATGGTCGCACCTTATATTCATTTCAAGAGCATTCCCTGAATATGTGCTAAACAGAAACTGTCCT
GACAACTCTATTGTACTGCTTCCCATCGTCGATCTTCTAAATCATGACTACCGTTCCAAAGTCAAATGGTATCCT
GAAAATGGGTGGTTCTGTTATGAAAAAATCGGTACCGCCTCCCAATCACGAGAACTATCAAATAATTATGGCGGT
AAGGGAAATGAGGAGTTACTCTCTGGATATGGATTTGTTTTAGAAGACAACATATTTGACTCAGTGGCTTTGAAA
GTTAAATTGCCATTAGATGTGGTATCTACAATTCTTGAAACAGAACCTAGTTTGAAGCTGCCCTTACTTTCGGAT
TACACCACTTATGCTTTTGAAAACAAAGACTGTGTCCAGCAAGAAAAGAAGGCTACTCGTAGTGCTACAGACTAC
ATCAATGGGGTGACTTACTTCATTAACATACAAAATGAACAATGTTTAGAACCATTACTGGATCTTTTTACCTAC
CTTTCTAAGGCCGAAGAGGAGGATCTACACGATTTGAGAGCCCGTTTGCAGGGTATACAAATGCTACGAAATGCA
TTGCAGAGCAAACTCAACAGCATTACTGGACCACCTGCAACTGATGACTCTTATGCAATTGATCCTTACAGAGTC
TATTGTGCTGACGTTTATACTAAAGGTCAAAAACAAATTTTAAAAGAGGCTTTAACGAGGTTGAAAAAACTAGAA
AAAACAATGCTGTCAGAGAACAAGCACCAATTGCTAACCATGAGCAAAATTCTTAAGAATGACCCTGCTTTTGCA
GAAACTGAATTACCTTCGCTGTTCAGCAACGAAGATGGTGAAGAGGTCATCTTTGAATCTACTTATGATTTATTG
ATACTTTGGATTCTACTGAAAACGAAAAAGAATTCTTACCCCACCAAATATGAATGGGTTGGACAGCAGTATACT
AATTTCAAGCAAACTGCATATATTTCAGATGATGCCAAGGCTTTCCATACTGCATACTTCGAGAAGCAAGACGAT
GTGGATTTGGCCGAAGTAGATCATGCGATTCAGTTTGTAGTAGACAATTCCTTTACCAGAACTTCATCTACAACC
GAAGAGACTATTTTAGTACGTAAGTAG

>YBR280C 0.73 Cold
ATGAGTGAAGTTGAGAGCAGAGAAAAGGAGCCTGACGCAGGATTGTCCCCAGATATCGTTCAAGCTACGCTACCA
TTTTTAAGCAGTGATGATATTAAGAACCTCTCGCAGACAAATAAGTATTATAATACATTACTAGATTTTGACCAC
TCTAAAATTTTGTGGCATGAACTATTTCATAAGGCATTTGGGACATTAAAGACAAATGACGAGCCTTTCCAGGGT
CGAAATTCGGCGGAATTTAAGACCTGCACAGAGACGATTTTAAGAGAAGCATTCCCCACCTTGTCATGGCAGGAA
GTATACCAATTACGGGCATATGATGCAAAGTTTTATAGTTGGGGTTATTTAAAGCATGGAAGGTTGGGCTATACC
GCGAGTTCCAATAATGAACTTGTCGCCACTTCACTAAACGGGCCAAGTCCGCGGTTCAAATATGGTGTCAATACC
CCCACTGAAGTTCCTTGGTTTAACAGTAGGACAACATCTAGAACTAGTAACTTCACTCCTTCAGAGGACCCGTTA
AGCGCCATCAAGAAGGATGGAGACGAAATCATAGCTCAAGTATCTAGTGGTGGTTTTTCTTTCCAAATATTGACT
GAGTCAGGTAACCTTTACAGCAGCGGATCCACTTTTTCTGGAGGGTTAAAAGGACCAGGTCCCAGTGGTTCACAG
CATGATTATAATCCCTTCAGAGAAATGATTCACAATATGGAAAGATCGTATCCGCGGATCACTAGCCGCAGTAAT
GGGAGTACTGTCAATACCACGGGTACATTTTCTGGAAGGAGAATGAGCGGTAGCCATCCATCTACTGCTTATGAA
CCAGGAAATGCAACTACAGCTCAGCATATTACCATAGATAGCGGAGGTGCGCCTGCAGCCTCTCCCGGAGGGAGC
CATAGTGGTGTCCCTAGAACTACAATGCCTTCAATGGGGCCGCATGAGAATATTTACAGCCAGATAGAAATGCTG
GAACGAAGTGCTAACAAGGCTGTACCAGGAAATAATCATATCAGGAGAATGTTCGCAAGAAATTCTTTCCCATTA
TATAGTGGTAGGGATGAAAATTTAGGATCATTTAATGATATTCAATTTGTCGCGGTAAGCTCTGGGAGAAGTCAT
TTCCTAGCAATGGATACAGATAACAATATTTATTCTTGGGACTCAACGGAATCTGACCAAGGTGTCAAAATTGAG
TTTGCTAACCTACCTAGTCGTGCTACGAATCCTATTTTGAAAATTGCTAGTGGTTGGAATTTCAATTGTTGCTAT
ATATATAAGGTCGGGTTGGTTGCATGGAAAGAAAGAGATGCTATTCAAAAGGGCGAGAGCTTTGCTTTTGCAAAA
TACGAGATTGTCCCGAATACGAATGATGTTAATGGAGACTCCAGGATTGTCGACTTTGCATGTTTACAAGATAAC
TGCGTTTTCTTCATTAATAACAACGGCGATAAACTATGGAAGTACCACAATGGACTGAACCAGATAGTAGATTTA
AATATTGTCGGAAAACTGTGCAAAATTAACGCCTGTTTTGCTTCATTAGTCCTCTTCACCGATACTCACTGTTAT
ACTTTGAAAGTAACAAATGGAGATGTCGATAAAGATAGTTTAACGGAGCTAGACATCAATGAAAATGTTATCTCC
GTTGCAAGTGGTGACTATCACACGGTGGCACTAACCGAACGCGGTCATTTGTATTCATGGGGTATTGAAAGTCAG
GATTGTGGGTGCTTAGGATTGGGCCCTTCTGAAAAAATAGTTAACGAGCTACATATTGGCAATTGGGAAGGGCAA
AGAAACATTAGGGTCGTTAAGCCCACGAAAATAGAACTACCTGAAGATTATATTTGTGTCAGTGTAACTGCTGGT
GGCTGGCAAACCGGTGCATTAATCATCAAGAAGCATTGA

>YLR144C 0.76 Cold
ATGTGTTACAGTAGGCAAGCCATTCCTCCACCTGTTCCTAATAGACCTGGCGGTACTACCAATAGAGGACCTCCT
CCCTTACCACCTCGCGCAAATGTTCAGCCACCAGTTTGTTCTTCTGAGAACTCCAGTAAACCTCGAGAAAATAGG
GTGGCAGGTGAATCACTGCGCACACCAAGCAGTTCTAACCCTTTAGCTGACAGCCAGGTGAATAGTGACAATATT
TTTCAATCACCTGTCCTTTCTAACTTAAAAGCTCCTCCTTCCGTCTTCAACAAAGTTCAACACCCGGTACCAAAG
CCGAATATTGATGATCAATCTGTTGATCCATTGGAGACAAACAAGTTCTACACAAACATGTTATTAGACGATAAT
ACACAACCTATATGGACACATCCTTATTCAATTTGGTTTTCTCGTGATCCAGAATTGTTTGGTTTGGCCGCCAAT
CATACTTTAGCGTCTCAAAGGGTTTTTGATACAACTACAAATCCTCCCAGATTCTACTTCAATCCCACAAATATA
AAATCATTTGTATTCAAGGCCAGGGAATTTGTCTCTTCAAATGATATCAAGCTTGAATTCCGGGATATGAAACAT
ATGTCCATGTGCTTACTAATGTCTTTGAGTAGTTCCCAGTTTATAGAATTCCCGCTGGTTCAGGGGATGGGCTTT
GTCACGGCTATTTACCACGATTTGGGTTTTGAATTAAGAAGCGCCGTTGGTTTCAGGAGCTTAGAGCGCATAAGC
GTGAATGAAAGATATGGTAAATATAATATACAACTTGAGAATAATAGGAATTGGATACTTTATTTGACCTCACCT
GATTACTCTTTTCCACAAGATTTTCAAATTTCATTACTCGACAGCAATACAATAATATCATCTCATAAAATCAAT
GGTCTTATCTGTCAATTATCTGCAGACTCCGTCCCCAGTATTGACATGGCAGCGGGCTGTTATCCAGTATACTGT
GACCTATCAGGGCAAACTGTTGATGAACATTTCACAAATTACAGATTTAACTATACGGTTGCAGGCTACTCGCAG
TCTGGAACTACTCTAATGTATGCTTTACCACATCATAAAGCGGCATTCACACCAGAAATGCAGGAACGTGAAATT
GCCTCAAGCCTGGATTCCACAGTTAAAGGCTTAATGACTGGTTATCTCACAAACAGTTTTGATATGCAGGTTCAA
GTACCTCAAGAATTGGGCTTTGAACCTGTCGCGTTATCTTTGAATAAGAAGGCAGATTACAGTCAAGAAAAGCTA
TCTAAAATTCGGGAAGCTGCTGTTCAAGAAGTTCAATTGAGTGACCCTCAACAAGAATCAAACATAGATTCTATG
TATTTCTCTGGTAAGATACTTGCAAAGTATGCGTGGATTCTTTATGTGACACATTACATCCTTCATGATGAAAAT
TTAACAAAAGAATTATTATCTAAACTGACTATTGCAATGGAGAGATTCATCAGCAATCAGCAAGTCTTACCTTTA
AATTACGACGTTAGTTGGAAAGGCATAATTTCATCAGGCTCTTCTAGCCAAGACTTTGGTAACTCTTATTATAAT
GATCATCATTTTCATTACTCTTATCATGTCATAACGGCTGCCATTATCTCTTTAGTCGATTCTGATTTAAGTGGA
GTTACTAACAACTCCTGGTTAGAAAATAATAGAGATTGGGTTGAGTGCTTAATTAGAGATTACTCTGGTGTTGAC
AACGATGATCCGTACTTCCCTCAATTTAGGTCATTTGATTGGTTTAATGGCCATTCATGGGCCAAAGGACTATTT
CCAAGTGGTGACGGTAAAGATGAAGAATCTACTTCAGAAGATGTCAATTCTTGCTATGCGATAAAATTGTGGGGG
TTGGTAACTGGAAATTCTAAGTTAACTGACATAGCGAACTTACAACTCGGCATCATGAGAAATGTTTTCCAGAGC
TACTTCCTATACGAAAGCAATAATACTGTCCAACCGAAAGAATTTATTGGTAATAAAGTTAGTGGTATTTTATTC
GAAAATAAAATTGATCACGCCACGTATTTTGGCATGGAGCCTCAATACATTCATATGATTCACGCCATTCCTATA
ACATCAGCATCTTCATGGGTCAGAACACCAAATTTTGTCAAAGAGGAGTGGGAAGAAAAAATGCAGCCGATAATT
GATCAAGTGAATGACGGTTGGAAAGGAATAATCATGTTAAATATGGCTCTGCTTGATCCAAAATTTTCGTACGAC
TTTTTCAGCCAACCTGATTTCAACAGAAATTTCCTAGACAATGGGCAAAGCTTAACTTGGTCTTTAGCTTATTCA
GGTGCTTTTTCTTAG

>YDL140C 0.75 Cold
ATGGTAGGACAACAGTATTCTAGTGCTCCACTCCGTACAGTAAAAGAGGTCCAATTCGGTCTTTTCTCACCTGAA
GAAGTTAGAGCAATCAGTGTGGCCAAAATTAGATTTCCAGAGACAATGGATGAAACCCAGACGAGAGCGAAAATT
GGTGGTCTAAACGACCCTAGGTTAGGCTCTATTGATCGTAATCTGAAGTGTCAAACTTGTCAAGAGGGTATGAAC
GAATGTCCTGGTCATTTTGGTCACATAGATTTAGCAAAACCTGTATTTCATGTTGGTTTTATTGCCAAAATTAAG

AAAGTATGTGAGTGTGTCTGTATGCACTGTGGTAAGCTATTACTGGATGAACATAATGAATTAATGAGACAAGCT
CTAGCAATCAAAGACAGTAAAAAAAGGTTTGCTGCAATTTGGACTTTATGTAAAACAAAAATGGTCTGCGAAACA
GATGTCCCTTCTGAAGATGATCCTACTCAGCTCGTATCAAGGGGAGGTTGTGGTAATACACAGCCTACAATTCGT
AAGGATGGGTTGAAATTAGTTGGTAGTTGGAAAAAAGATAGAGCCACGGGGGATGCGGATGAACCAGAACTAAGA
GTTTTAAGTACGGAGGAAATCTTGAATATTTTTAAGCATATCTCAGTAAAAGACTTCACTAGTTTGGGTTTCAAC
GAAGTTTTTTCTCGTCCAGAATGGATGATTTTAACATGCCTTCCTGTCCCACCACCACCGGTGCGTCCATCCATT
TCCTTCAATGAATCTCAAAGAGGTGAGGATGATTTAACCTTTAAACTTGCTGATATTTTAAAAGCTAATATTAGT
TTGGAAACACTAGAGCATAACGGTGCTCCACATCATGCTATTGAAGAAGCAGAGAGTTTATTACAATTTCATGTT
GCCACTTATATGGATAATGATATTGCTGGTCAACCACAAGCTCTTCAAAAGTCCGGCCGTCCCGTTAAATCTATT
CGTGCTCGTTTGAAGGGTAAAGAGGGTCGTATCAGAGGTAATTTAATGGGTAAGCGTGTGGATTTTTCGGCAAGA
ACTGTTATTTCTGGTGATCCTAATTTGGAATTAGACCAAGTCGGTGTTCCAAAATCTATTGCCAAGACTTTAACA
TACCCAGAAGTGGTCACACCATATAACATAGATCGTCTGACGCAACTTGTTAGGAATGGACCAAATGAGCACCCC
GGTGCCAAATACGTCATTCGTGATAGCGGAGACCGTATAGATTTAAGATACAGTAAAAGGGCAGGTGATATTCAA
TTACAGTATGGGTGGAAAGTTGAACGTCATATTATGGACAATGATCCAGTTTTATTCAACCGTCAACCTTCGTTG
CACAAAATGTCCATGATGGCCCACAGAGTAAAAGTTATTCCATATTCTACATTTAGATTGAATTTGTCCGTTACA
TCTCCATACAATGCCGATTTCGACGGTGACGAAATGAATCTTCACGTTCCTCAGTCTGAGGAAACAAGGGCGGAA
CTTTCTCAATTATGTGCTGTTCCTCTGCAAATTGTTTCACCACAATCTAACAAACCTTGTATGGGTATTGTTCAA
GATACTTTGTGTGGTATTCGTAAACTGACATTAAGAGATACATTTATAGAACTTGATCAAGTTTTGAATATGCTT
TATTGGGTTCCAGATTGGGATGGTGTTATTCCGACACCTGCAATTATCAAGCCCAAACCTTTGTGGTCCGGTAAA
CAAATCTTGTCTGTGGCTATCCCAAACGGTATTCATTTACAACGTTTTGATGAGGGCACTACTCTGCTTTCTCCA
AAGGATAATGGTATGCTTATTATTGACGGTCAAATCATTTTTGGTGTAGTAGAGAAAAAAACCGTTGGTTCCTCC
AATGGTGGTTTAATTCATGTTGTTACGAGAGAAAAGGGACCTCAAGTTTGTGCTAAGTTGTTTGGTAACATACAG
AAAGTTGTTAACTTTTGGTTACTACATAATGGGTTTTCAACAGGTATTGGTGATACCATTGCGGACGGCCCAACA
ATGAGGGAAATTACAGAGACAATTGCAGAGGCTAAAAAGAAAGTTTTGGATGTTACGAAAGAAGCCCAGGCAAAC
TTATTGACTGCTAAACATGGTATGACTCTCCGTGAGTCTTTTGAGGATAACGTTGTTCGGTTCCTAAATGAAGCA
AGAGATAAGGCAGGTCGTTTAGCTGAAGTCAATTTGAAAGATTTGAACAATGTGAAACAAATGGTTATGGCAGGT
TCCAAGGGTTCATTTATTAATATCGCGCAAATGTCAGCTTGTGTAGGACAGCAATCTGTTGAAGGTAAACGTATT
GCTTTTGGGTTCGTTGATCGTACCTTACCTCATTTCTCTAAAGATGATTACTCCCCAGAGTCTAAAGGTTTTGTT
GAGAACTCATATTTGAGAGGTTTGACCCCACAAGAATTTTTTTTCCATGCAATGGGTGGTCGTGAAGGTCTTATC
GATACCGCCGTCAAAACAGCCGAAACAGGTTATATTCAACGTCGTTTAGTGAAAGCTCTAGAAGATATCATGGTT
CATTACGATAACACCACAAGAAACTCATTGGGTAACGTTATTCAGTTTATTTATGGTGAAGATGGTATGGATGCT
GCGCATATTGAAAAGCAATCGCTAGATACTATTGGTGGCTCCGATGCAGCTTTTGAAAAGAGATACAGAGTTGAT
TTATTGAATACAGACCATACCCTTGATCCCTCACTATTGGAATCCGGATCTGAGATACTTGGCGATTTGAAACTT
CAAGTTCTCCTGGATGAAGAATACAAACAATTAGTGAAAGATCGTAAATTTTTGAGGGAAGTTTTTGTTGATGGT
GAAGCAAACTGGCCATTACCAGTCAACATAAGACGTATTATTCAAAATGCTCAACAAACTTTCCACATAGATCAT
ACGAAACCATCTGATTTAACAATCAAAGACATCGTTCTTGGTGTAAAGGATTTGCAAGAAAACTTATTAGTGTTG
CGTGGTAAGAATGAAATTATACAAAATGCCCAGCGAGATGCAGTTACATTGTTCTGCTGTTTATTACGTTCCCGT
TTGGCCACACGTAGAGTTCTACAAGAGTACAGACTAACAAAACAGGCATTCGATTGGGTATTAAGTAATATCGAG
GCACAATTCCTCCGTTCTGTTGTTCACCCTGGTGAAATGGTTGGTGTTCTAGCAGCCCAATCCATTGGTGAACCA
GCCACACAAATGACCCTTAACACCTTCCATTTTGCTGGTGTTGCTTCCAAAAAAGTTACTTCTGGTGTCCCCCGT
TTAAAGGAAATTTTGAATGTGGCCAAAAACATGAAAACCCCTTCCTTGACTGTATACTTAGAGCCTGGTCATGCT
GCCGATCAAGAACAAGCGAAGTTGATCAGATCTGCTATCGAGCATACCACTTTAAAGAGTGTCACTATTGCTTCA
GAAATTTACTATGATCCTGATCCACGTTCCACAGTTATTCCAGAAGATGAAGAAATTATCCAACTTCATTTCTCA
TTATTGGATGAAGAGGCTGAACAATCTTTTGACCAACAATCACCTTGGTTATTACGTCTGGAACTGGATCGTGCA
GCAATGAATGATAAAGACTTAACAATGGGTCAGGTTGGTGAAAGAATCAAGCAAACATTCAAAAATGATTTGTTT
GTTATCTGGTCTGAAGACAACGATGAGAAGTTGATCATCCGTTGTCGTGTTGTTCGTCCAAAGTCACTAGATGCT
GAGACTGAAGCAGAAGAAGATCATATGTTGAAGAAAATTGAGAACACAATGTTAGAGAATATTACATTACGTGGT
GTAGAGAACATCGAGCGTGTTGTCATGATGAAATATGACCGTAAAGTACCAAGTCCAACTGGTGAATACGTTAAG
GAACCTGAATGGGTGTTGGAAACAGATGGTGTTAACTTATCTGAAGTTATGACTGTTCCTGGTATCGACCCAACC
AGAATCTATACCAACTCCTTCATTGATATAATGGAAGTTCTAGGTATTGAAGCTGGTCGTGCAGCCTTGTATAAA
GAAGTTTACAATGTTATTGCTTCTGATGGTTCGTATGTTAACTACCGTCATATGGCTTTGTTAGTCGATGTTATG
ACAACCCAAGGTGGCTTAACTTCTGTTACTCGTCATGGTTTCAACAGATCAAATACAGGTGCCTTAATGAGATGT
TCATTTGAAGAAACTGTCGAAATTTTGTTTGAAGCTGGTGCTTCAGCCGAATTAGATGATTGTCGTGGTGTTTCG
GAAAATGTCATTCTTGGTCAAATGGCTCCAATCGGTACCGGTGCATTTGATGTGATGATCGATGAGGAGTCACTG
GTAAAATACATGCCAGAACAAAAAATAACTGAGATTGAAGACGGACAAGATGGTGGCGTCACACCATACAGTAAC
GAAAGTGGTTTGGTCAATGCAGATCTTGACGTTAAAGATGAGCTAATGTTTTCACCTCTGGTTGATTCGGGTTCA
AATGACGCTATGGCTGGAGGATTTACAGCGTACGGTGGTGCTGATTATGGTGAAGCCACGTCTCCATTTGGTGCT
TATGGTGAAGCACCTACATCTCCCGGATTTGGAGTCTCCTCACCAGGCTTTTCTCCAACTTCCCCAACATACTCT
CCTACCTCTCCAGCGTACTCACCAACATCACCATCGTACTCACCAACATCACCATCGTACTCGCCAACATCACCA
TCGTACTCACCTACATCACCATCGTATTCACCAACGTCACCATCATATTCGCCAACGTCACCATCATATTCGCCA
ACGTCGCCATCGTATTCTCCAACGTCACCATCGTATTCGCCAACGTCGCCTTCCTACTCTCCCACGTCGCCAAGC
TACAGCCCTACGTCTCCTTCTTATTCTCCTACATCTCCATCATACTCTCCTACGTCACCAAGTTACAGCCCAACG
TCACCAAGTTACAGCCCAACGTCTCCAGCCTATTCCCCAACATCACCAAGTTATAGTCCTACATCGCCTTCATAC
TCTCCAACATCACCATCCTATTCCCCAACATCACCTTCTTACTCTCCCACCTCTCCAAACTATAGCCCTACTTCA
CCTTCTTACTCCCCAACATCTCCAGGCTACAGCCCAGGATCTCCTGCATATTCTCCAAAGCAAGACGAACAAAAG
CATAATGAAAATGAAAATTCCAGATGA

>YIR039C 0.81   Cold
ATGCAATTGATTTCCATCCTCTCTTTGTTGTCATCATTGATGTGTTCATTGACCGTTTTAGGGTCTTCCGCAAGT
TCCTATGTTAAGTTTCCTGTTCAAAAGTTGGCTGATATTATAAATATATGCACACAGGATGTTTCTACCGTCTTC
AAAAGAAATGAGGTTCTTAACACCACAGTGATAAATGGAATAGGAGTGTATGTTGTCAAGATGGAAATCGGAACG
CCTCCACAAACGTTGTACCTACAGTTAGATACTGGCTCTTCAGATATGATTGTGAATAATGCTGACATTGCCTAT
TGCAAATCGATGTCTGACGGATCTGACTACGCTTCAACTGATAATTACGAGCTCACAGCAACGTTCAATGGGCTT
CCTTCGACTACCATCTCATCAGAAGCTTATAACACTCTCTGCTCTTATTGGGGTACATTCGATGCAAGCAACTCA
TCTACTTTTGAGAATAATGCGACGTTTTTCAATAACACATACGGAGATGGTACGTATTATGCAGGGACTTATGGT
ACGGATGTTGTCTCTTTCGAAAATATTACTCTAAATGATTTCACTTTTGGTGTATCAAATGATACAATAGGCAAT
CCAAGTGGCATTTTAGGCATATCGCTTCCCATTGCAGAATTTACAGATGGTATCGAATACGCATTGGCATTGAAC
AGAACACCTTTCATATATGATAACTTTCCAATGGAATTAAAAAATCAGGGAAAGATTAATAAAATAGCGTATTCT
TTATTTTTGAATGGACCTGATGCACACTTCGGGAGCATCTTGTTTGGTGCAGTGGACAAAAGTAAGTACACAGGA
CAGCTTTACACCCTGCCTATGCTGCAGGCATTCAATACCCTTGGCTCAAATCCTGGGATGATTATTACTGCACAA
AGTGTGGCAATTTTGGATAGTGAATCTGGCAATAAAACTGTTTCGGATATTCAATTTCCAGTGATGTTGGATTCT
GGCACAACTTTCTCCTATTTACCCACCGAAATTGCAGAAGCAATCGGCAAAAGTTTTGACGGAGAGTACAGCTCT
GACGACCAAGGCTACATCTTTGATTGTTCAAAAGTGAACGATACTTTACTGTCCGTCGACTTTGGAGGATTTAAT
ATATCGGCAAACATATCCAATTTCGTAACATCAGCTAAGGATCGTTGCGTTTTAAATGTTAAGCAATCCGAGTCC
ACTTATATGCTGGGAGATGCTTTCCTTGTCGACGCATATGTAGTCTACGATTTAGAAAACTACGAAATTTCTATT
GCTCAGGCTAGCTTTAATAATCAAGAAGAAGATATTGAGGTTATCTCCGATACTGTCCCAGGTGCCACCCCTGCT
CCCGGATACTTTAGTACATGGGTATATAAGCCCGGTAGTCCCATCGGAACAGGAGATTTTATTAATGTCAGCTGG
ACCTCGTATTCGGAATTTTCACAGTACAAATCATTGCTTGCCACCGCAGCCCAAAGCGATGATGCTAGTTCTTTC
TCAAGTAGTGGCGGCTCTAGCGAAAGCACCACTAAAAAACAAAATGCAGGTTACAAATACCGTTCTTCTTTCTCC
TTTTCATTATTGTCCTTCATATCTTATTTTCTTTTATAA

>YDR467C 0.65   Cold
ATGATTTATATGTTGGTTTTTCTGGATCGTCAACAACTCGTGCATATTTTTTTGTTTCGAAGTAGAGGAACCACA
AATATTATTAAAGCGTGTTATTTTTTTTTCTTACTCTTCTGTAAACTTCTAAATGCTGCTGAGGCACCCCTACTA
GCAATTTCCCTATCAAAGTTTGTTTGGCTGTTATTAAGGGTATGTAAGACCTCGTATCTGCTGCTACTGATCACC
ATGTTAGAAGGAGCAGAATACTTTTCTCTCGTAGTTGGGAATTCCATTTGCGGAAGTGGAGGTGAAGGTGTTGGC
TGTCGATATCCTGTTGTTTTGATTTGA

>YER070W 0.82   Cold
ATGTACGTTTATAAAAGAGACGGTCGTAAAGAACCTGTCCAATTCGATAAGATTACCGCTCGTATATCACGCTTA
TGCTATGGTTTAGATCCAAAACATATCGACGCCGTTAAGGTCACCCAACGTATCATTTCTGGTGTCTATGAAGGT
GTCACAACAATCGAACTAGACAACTTAGCCGCTGAAACATGCGCTTATATGACTACTGTTCATCCAGATTACGCC
ACCCTAGCGGCCAGAATTGCCATTTCTAATTTACATAAACAAACCACAAAACAATTTTCTAAGGTTGTCGAAGAT
CTTTATAGATACGTCAATGCTGCTACTGGTAAGCCCGCTCCCATGATCTCTGATGATGTCTACAATATTGTCATG
GAAAACAAGGATAAATTGAACTCCGCAATTGTCTATGACAGAGATTTTCAGTACAGTTATTTTGGTTTTAAAACT
TTGGAACGTTCTTATTTACTAAGAATCAACGGTCAAGTGGCCGAACGTCCACAACATTTAATTATGAGAGTCGCA
CTAGGCATCCACGGTAGAGATATCGAGGCTGCTTTAGAAACGTATAACTTGATGTCTCTAAAATATTTTACTCAC
GCCTCTCCAACGTTGTTCAATGCCGGTACTCCAAAACCTCAAATGTCCTCTTGTTTCTTGGTTGCCATGAAGGAG
GACTCTATCGAGGGGATTTACGACACCTTGAAGGAATGTGCTTTGATTTCCAAAACTGCTGGTGGTATTGGTCTA
CATATCCATAACATTCGTTCAACTGGTTCTTACATTGCTGGTACAAACGGTACTTCTAACGGTTTAATTCCTATG
ATTCGTGTTTTCAATAACACTGCCCGTTATGTTGACCAGGGTGGTAATAAAAGACCTGGTGCGTTTGCCCTTTAC
CTGGAACCATGGCATGCTGATATATTTGATTTTATTGATATTAGGAAGAACCACGGTAAAGAGGAAATTCGTGCA
AGAGATTTGTTCCCTGCTCTATGGATTCCTGATCTTTTCATGAAGCGTGTCGAAGAAAATGGGACCTGGACATTA
TTCTCTCCAACATCAGCTCCTGGTTTAAGCGATTGTTACGGTGACGAGTTTGAGGCTCTATATACCCGCTACGAG
AAAGAAGGTCGTGGTAAGACTATCAAAGCCCAAAAATTATGGTATTCCATTTTGGAAGCTCAAACTGAAACTGGT
ACACCTTTCGTTGTTTACAAGGATGCTTGTAACAGAAAATCTAATCAAAAAAACTTAGGTGTCATCAAGTCATCA
AACTTATGCTGTGAAATTGTTGAATACTCAGCTCCAGATGAAACTGCTGTTTGTAACTTGGCTTCCGTTGCCTTA
CCAGCATTCATTGAAACTTCTGAGGATGGTAAGACTTCCACATACAACTTCAAAAAATTACATGAAATTGCTAAA
GTTGTTACTCGTAATTTAAACAGAGTCATTGATCGTAATTACTACCCTGTTGAAGAAGCGAGAAAATCCAATATG
AGACATAGACCAATTGCTTTGGGTGTTCAAGGTCTCGCTGACACTTTCATGCTGTTACGTTTGCCATTTGATTCT
GAGGAAGCCCGTTTGCTAAATATCCAAATCTTTGAAACTATTTATCATGCCTCCATGGAAGCTTCTTGTGAACTA
GCTCAGAAGGACGGTCCATACGAAACTTTCCAAGGATCTCCTGCTTCTCAAGGTATACTACAGTTTGATATGTGG
GACCAAAAACCCTACGGCATGTGGGATTGGGACACCTTAAGAAAAGATATCATGAAGCATGGTGTTAGAAATTCC
TTGACCATGGCACCAATGCCTACTGCATCCACATCCCAAATATTGGGTTATAATGAATGTTTCGAACCAGTCACT
TCCAATATGTACTCCCGTCGTGTCTTATCCGGTGAATTCCAGGTTGTGAACCCTTACTTACTGCGTGACTTGGTT
GATTTAGGTATTTGGGATGAGGGTATGAAACAGTATCTGATTACACAAAATGGCTCCATTCAAGGCTTACCAAAC
GTTCCACAAGAATTGAAGGACTTATACAAGACTGTTTGGGAAATTTCACAAAAGACTATCATTAACATGGCAGCC
GATCGTTCTGTCTATATTGATCAATCTCATTCTTTGAATTTGTTCTTACGTGCCCCAACTATGGGTAAACTAACA
AGTATGCATTTTTACGGATGGAAGAAGGGATTGAAGACCGGTATGTACTATTTGAGAACCCAAGCTGCATCTGCT
GCAATTCAATTTACTATTGATCAGAAGATTGCGGATCAAGCTACAGAAAACGTTGCTGATATTTCCAACTTGAAG
CGTCCATCATATATGCCTTCCAGTGCAAGCTACGCTGCCAGCGATTTCGTGCCCGCAGCTGTGACTGCAAACGCA
ACTATTCCATCTCTAGATAGCTCCTCGGAAGCTTCAAGAGAGGCATCTCCAGCTCCAACAGGTAGCCACTCATTA
ACTAAAGGAATGGCAGAATTAAACGTTCAAGAGTCTAAGGTAGAAGTTCCTGAAGTACCTGCCCCAACTAAGAAT

GAAGAAAAAGCTGCCCCCATCGTTGATGATGAGGAAACCGAGTTCGACATTTACAACTCTAAGGTTATAGCATGT
GCTATTGATAACCCAGAAGCTTGTGAAATGTGTTCGGGTTAA

>YDR529C 0.75 Cold
ATGCCACAGTCTTTTACGTCTATTGCGAGAATTGGTGACTATATTTTGAAGTCACCCGTCCTCTCCAAGTTATGT
GTTCCAGTTGCCAATCAGTTCATTAACCTCGCAGGTTACAAGAAGTTAGGGCTCAAATTTGACGACTTAATTGCA
GAGGAAAATCCCATCATGCAGACCGCTTTAAGAAGACTCCCTGAAGATGAATCTTATGCCAGAGCATATAGAATA
ATCAGGGCTCATCAAACCGAGTTGACTCATCATTTACTGCCAAGAAACGAATGGATCAAAGCCCAAGAGGATGTT
CCTTACCTGTTGCCATACATATTAGAAGCTGAAGCTGCAGCTAAGGAGAAGGACGAGTTAGACAACATAGAGGTC
TCCAAATGA

>YCL004W 0.75 Cold
ATGACGACTCGTTTGCTCCAACTCACTCGTCCTCATTACAGATTATTATCCCTACCTCTCCAGAAACCCTTCAAT
ATAAAAAGGCAGATGTCCGCTGCGAACCCTTCTCCATTTGGCAATTATTTGAACACGATCACTAAGTCCCTACAA
CAGAATTTACAAACATGCTTTCATTTCCAAGCAAAAGAAATCGATATAATCGAATCTCCATCTCAGTTTTACGAT
CTCTTGAAGACAAAAATACTTAATTCACAAAATAGAATATTCATTGCGTCTCTGTATTTAGGCAAAAGCGAGACT
GAGTTGGTGGACTGCATATCCCAGGCATTGACCAAGAACCCCAAGTTGAAAGTTTCTTTTCTACTTGATGGCCTT
CGAGGAACAAGAGAATTGCCTTCCGCCTGTTCCGCCACTTTATTATCGTCTTTAGTAGCCAAATATGGGTCAGAG
AGAGTGGATTGCCGATTGTACAAGACGCCTGCTTATCATGGTTGGAAAAAAGTCTTGGTTCCCAAGAGATTTAAT
GAAGGTTTAGGCTTACAACATATGAAAATATATGGGTTTGATAACGAGGTCATTCTTTCGGGAGCCAACCTTTCG
AACGACTATTTCACCAACAGACAAGATAGATACTATCTCTTTAAATCTCGAAACTTCTCCAACTATTATTTTAAA
TTACATCAACTCATAAGTTCCTTCAGTTATCAGATTATAAAGCCAATGGTGGATGGTAGCATCAACATCATTTGG
CCAGATTCGAATCCTACTGTTGAACCGACGAAAAATAAAAGGCTGTTTTTAAGGGAAGCATCTCAATTACTAGAT
GGCTTTTTAAAGAGTTCTAAACAAAGCCTCCCGATTACTGCCGTGGGTCAATTCTCCACATTAGTTTACCCAATT
TCTCAATTCACTCCACTTTTTCCCAAATATAATGACAAATCGACCGAAAAAAGAACAATATTGTCATTGCTTTCC
ACTATAACAAGCAATGCCATTTCTTGGACGTTCACTGCAGGATACTTCAATATTTTGCCAGACATCAAAGCAAAA
CTGCTGGCAACGCCGGTTGCTGAGGCAAATGTAATAACAGCTTCCCCCTTTGCAAACGGCTTTTACCAATCAAAG
GGCGTCTCATCAAATTTACCTGGTGCTTACTTGTACCTGTCAAAAAAATTTCTACAAGATGTATGTAGGTACAGA
CAAGATCATGCTATTACATTAAGAGAATGGCAAAGAGGCGTAGTAAATAAGCCGAATGGTTGGTCATATCACGCA
AAAGGTATTTGGCTTTCCGCTCGTGATAAAAATGATGCTAACAATTGGAAACCCTTTATCACGGTTATAGGATCT
TCAAACTATACGAGAAGGGCGTATTCATTAGATTTGGAATCGAATGCTCTCATTATTACAAGAGATGAAGAGCTA
AGAAAAAAAATGAAAGCAGAGTTAGATAATTTATTACAATATACAAAACCTGTAACTCTAGAAGACTTTCAATCA
GACCCAGAAAGACATGTTGGCACTGGTGTAAAGATAGCTACCTCCATTTTGGGTAAAAAACTTTAG

>YNR061C 0.77 Cold
ATGTTAAAGTTAACAACAACATCCGTTACTTTCCACGTACTAAGATATTTCCAACTTGGCTTATCTGTAACTAAC
TTGTTGTTAGCTAGTTTTGCCATAATCACAAACTATAAAGTGGATCGAATCCTTAGATTGAGCTTAGCTGTTTCA
ATCATATCTTCAGTTTATTTTGGGATTGTGAGATTTTTGCCTGTTCTGTTAATATTCGTGATGGAAATTGTTCAG
ACCGTCCTGTGGTTTACCGCTTTCGTAACTCTAGCTTCAAAATTTGGTTCTATGTCCTGCTCAAGTATGCCACGG
GGTATAAACTTTGATTATTCAGGTTCTTGTAAGATTGCTAAAATAGATATTTTACCAGAAGCCGTTTTATTCATA
CTATTTCTCGCTACCACATATGCTTCTTATATCACAGTCCTATCTCAAGCTAAGGAAAATGGGTCAAGCACAAGA
TCTGTTTTGAAGGCTTGTGTGAAGGCATTACGTGATACCGTGGACCGCTTGGAGACCAGTCTGGAAGAGAGTGAG
CCTCTTTTAGATCTTGAAGTTCAGGAAGATGCGAGGACTGAAACCGAAAGCATTGAGGATTCAACTGATAGTGAG
GATAATGCTAACATCGAACAAGAGAAAGTGATTGATGGTTCAATTGAGCATTCTTCATGA

>YOR102W 0.82 Cold
ATGCAAGCACGCCGGCAAACCCTTCCTCAATTTATAAAATGTAAACAAACGAAATGTAAAATCAAACTAGCAACA
ATAAACTCGGCAAAAGCTCTGTTTTTAGAAATGCCTGGAAAACTATTACACAATTGTAACCGCAAACTCATCAAT
AGGACGAATTGTCCCACACAAATAATGAATCCCGCTAAAAACGCATTGAATGGAAAATTATCACGGATCAAGATA
ATGAATGTACACTGGATCACCCCTAATAATACCAAAAAGAAGCAGAATGTATCAATTAGTTTCAGTTTAGGATAT
TTTTCAATTTGGGCAAAATATGCCCTCTTAGAAGTCTTGAAAGTTTCTTGA

>YMR318C 0.8 Cold
ATGTCTTATCCTGAGAAATTTGAAGGTATCGCTATTCAATCACACGAAGATTGGAAAAACCCAAAGAAGACAAAG
TATGACCCAAAACCATTTTACGATCATGACATTGACATTAAGATCGAAGCATGTGGTGTCTGCGGTAGTGATATT
CATTGTGCAGCTGGTCATTGGGGCAATATGAAGATGCCGCTAGTCGTTGGTCATGAAATCGTTGGTAAAGTTGTC
AAGCTAGGGCCCAAGTCAAACAGTGGGTTGAAAGTCGGTCAACGTGTTGGTGTAGGTGCTCAAGTCTTTTCATGC
TTGGAATGTGACCGTTGTAAGAATGATAATGAACCATACTGCACCAAGTTTGTTACCCACATACAGTCAGCCTTAT
GAAGACGGCTATGTGTCGCAGGGTGGCTATGCAAACTACGTCAGAGTTCATGAACATTTTGTGGTGCCTATCCCA
GAGAATATTCCATCACATTTGGCTGCTCCACTATTATGTGGTGGTTTGACTGTGTACTCTCCATTGGTTCGTAAC
GGTTGCGGTCCAGGTAAAAAAGTTGGTATAGTTGGTCTTGGTGGTATCGGCAGTATGGGTACATTGATTTCCAAA
GCCATGGGGGCAGAGACGTATGTTATTTCTCGTTCTTCGAGAAAAAGAGAAGATGCAATGAAGATGGGCGCCGAT
CACTACATTGCTACATTAGAAGAAGGTGATTGGGGTGAAAAGTACTTTGACACCTTCGACCTGATTGTAGTCTGT
GCTTCCTCCCTTACCGACATTGACTTCAACATTATGCCAAAGGCTATGAAGGTTGGTGGTAGAATTGTCTCAATC
TCTATACCAGAACAACACGAAATGTTATCGCTAAAGCCATATGGCTTAAAGGCTGTCTCCATTTCTTACAGTGCT
TTAGGTTCCATCAAAGAATTGAACCAACTCTTGAAATTAGTCTCTGAAAAAGATATCAAAATTTGGGTGGAAACA

TTACCTGTTGGTGAAGCCGGCGTCCATGAAGCCTTCGAAAGGATGGAAAAGGGTGACGTTAGATATAGATTTACC
TTAGTCGGCTACGACAAAGAATTTTCAGACTAG

>YLR421C 0.79   Cold
ATGAGTATGAGTTCAACTGTAATTAAATTCAGGGCTGGTGTTTGTGAATACAACGAAGATTCACGTCTATGTACG
CCAATTCCAGTTCAGGGTGAAATCGAGATCAAACCAAATGAAGAGGAAGAATTGGGATTTTGGGATTTCGAGTGG
CGTCCAACGGAAAAGCCGGTCGGGAGGGAACTTGATCCTATTTCTTTGATTTTAATTCCTGGAGAGACCATGTGG
GTTCCAATAAAATCCAGTAAAAGTGGCAGAATATTTGCTTTAGTTTTTTCATCCAACGAAAGGTACTTCTTTTGG
CTGCAAGAGAAGAACTCAGGTAATTTGCCCTTAAATGAATTGAGTGCGAAAGATAAAGAAATTTACAATAAGATG
ATTGGAGTGCTGAATAATAGTAGTGAAAGTGATGAGGAAGAAAGCAATGACGAAAAGCAAAAAGCTCAAGATGTG
GATGTTAGTATGCAAGATTAA

>YAR008W 0.71   Cold
ATGCCACCGCTAGTATTTGACATAGATCACATCAAACTTCTAAGGAAATGGGGTATTTGTGGTGTGTTATCTGGA
ACTTTGCCTACTGCAGCACAGCAAAATGTATTTTTGTCGGTACCTTTGAGGCTTATGTTAGAAGATGTGCTGTGG
CTGCATTTGAACAATCTTGCCGATGTGAAATTAATAAGACAAGAGGGAGATGAGATTATGGAGGGAATAACATTA
GAGCGGGGCGCCAAACTATCTAAAATTGTCAACGATCGTTTGAACAAGTCATTTGAATATCAGAGAAAGTTCAAA
AAGGATGAACACATTGCAAAATTAAAGAAAATCGGTAGAATCAATGATAAAACCACAGCTGAAGAATTGCAACGG
CTTGATAAATCTAGCAATAATGACCAGCTAATTGAATCTTCTTTGTTCATTGACATTGCTAATACCTCTATGATT
TTAAGAGACATACGGAGTGATTCAGACAGCTTATCCCGCGATGATATCAGTGATTTGTTATTTAAGCAGTACAGA
CAGGCAGGAAAAATGCAGACCTATTTCTTATACAAGGCATTGAGAGATCAAGGGTACGTTTTGTCCCCAGGTGGA
CGTTTTGGTGGGAAGTTTATAGCATACCCTGGTGATCCTCTTCGTTTCCATTCACATCTGACGATACAAGATGCG
ATTGATTATCATAATGAGCCGATTGACCTAATATCCATGATAAGTGGTGCAAGACTAGGAACGACTGTGAAAAAA
CTTTGGGTCATAGGCGGTGTTGCGGAAGAGACAAAGGAAACTCATTTCTTCTCAATAGAATGGGCTGGATTTGGT
TAA

>YHR110W 0.82   Cold
ATGAAATATAATATAGTGCATGGAATTTGCCTATTATTTGCTATTACCCAAGCTGTAGGGGCTGTCCATTTTTAT
GCGAAGTCCGGGGAAACCAAATGCTTCTATGAACACTTATCCCGGGGAAACCTACTGATTGGGGATTTAGACCTA
TATGTAGAAAAGGATGGTCTGTTTGAAGAGGACCCTGAATCCAGTCTGACAATAACTGTCGATGAAACATTCGAT
AACGACCATCGTGTCCTAAATCAAAAAAACTCACACACAGGTGATGTTACTTTTACAGCTTTAGACACAGGTGAA
CATAGATTTTGCTTCACTCCATTCTACAGCAAGAAATCAGCCACACTAAGAGTATTCATCGAACTAGAAATTGGC
AATGTTGAAGCGCTTGACAGCAAGAAAAAAGAAGATATGAATTCACTTAAGGGGAGGGTAGGCCAGTTGACTCAA
AGGCTATCTTCTATTCGTAAGGAGCAAGACGCTATCAGAGAAAAAGAGGCAGAATTCAGAAATCAAAGTGAATCA
GCTAACAGCAAGATAATGACGTGGTCTGTGTTCCAACTTCTCATATTGCTGGGCACCTGTGCCTTCCAGCTACGC
TATCTCAAGAATTTCTTTGTCAAACAGAAGGTAGTATAA

>YDL224C 0.8Cold
ATGTCATTAGTGCATAATCAGACGAATTTAAACGAATCCAAATTTCTTATAGAAAGGGCATTTAGTTCTAGTTCA
GAAACCGTTCCATTATCAAAGGAGGCAACATATCCCATGCCAACTGCATATAGTTTTTCCGCAGTTAGGAGCAAC
AGCGAGACTAACATTAAAAGAGAGAACCCGCAAGGTTTTGCAAAAGAACCTATTATGACTTCTATGCTTCATAAT
TTGACCATGTCCACGGGGAAAGGGAACGGGAATGACGTCAATTCTTTAGCCCCACATGATGTGGATGTTGGCCCC
TACTGTCTTTTGCTGAGGAATTTGCCAAAAGACATTACACTAAGAGAGTGTTATTGTATATTTTCACTAGCAACT
GGGGTGTCAAGTATAGAACTAAAAAGGGATGATAGAGAACCCTTTAATGACAATGAAAAAGTCGTTGTGGTCAAA
TTTGGATCCCTTTCTTTAGTAACCCATTATGCTAATATATTAAACTCGAAGTCTGAGATTTTTGGGCCTAGTTTC
CCCTTTAGGTCTCACATTGACGTAGTAAATGAACAAACGCAGCTTCCAGTGAGTTTTCAAGAACATGTTTCATCT
GGCACTACAAATAGTTCTCCAAAGAATTACCAATTGTCGTCCAGTGCGCAAAATGAAATTCAAAATCAAAGTTTT
AATACTATATCATATGGAAAAACTAGTTCCTCTCCATTAGGGCCTTCAGCGGCCAAGCCAAGGCCTTCATTACTT
TCAGAGAGATCCCTCCGTTTCTCATTCAATGACCCATTCGGATTGGAAACAATCTCTCAAAGAAAAGAATCCGTA
CCCTTTCTACGAAATAGCATCTCACAACATGATTTATCAAACGTAACCACCACACCAGTTCCAGCAGGAATGCCA
CCTCAGAAGGACGCTGGCAAGTCACTTTTGCTACTAGAAAAGGATGAAATAAATGAAAGTATTTGGAATGGTGAT
GAATTAGTTAATGATGTTGGTAATTCTTCATTTGGTGCATCTTTACAAGAGCCTCCGATGTCATCTACGCCTGTG
ATGGAGTGGAATGCTTCCTCAACTGCTAATATTCCTCTTTTTCAATTATCTAGCCAAGAAAATCATCAGTCTAAT
CTGTTGCCTCCATCCCACCATTCAATTAGCCAGGATGTTCCACATATTCAATCACAACCTAATCTGAACAATTCA
GGAGTAATACATTCCGCGACTTCTCTACCGCATTATCACTTATTGAACCAAATTAATGCTTCAACTAAGACACAA
AGTATTCAGCAGAGCGTTTCGAATGTTCCTTCCAATTTGGACCTGAATCTCCAAACGGAAAATGGCCACCCACAA
TCTTCTGCTCCTAACGGGTCTTCAATATTCAACAACCAAAAGGTGAATCAAGGCTTTTTAGTGAGTGAACAAGAC
ACCAGTACAATATCACGACAGAAAGAATGTTCTTCTACAGCTTCTGCCTCTGCATTTTCGAAAAATAACGAAACA
AATGTGGCAGGTTCCACAACTATTTCACAGGCTGACCTATCACTACTAGCCAAGGTGCCACCACCAGCCAATCCC
GCGGACCAAAATCCTCCTTGTAATACTCTTTATGTGGGTAATCTACCTCCGGATGCCACGGAACAAGAATTAAGG
CAATTGTTTTCTAATCAGCAAGGTTTTCGTAGATTATCCTTCAGGAATAAAATGAACTCTCACGGTCATGGCAAC
GGTCATGGCCATGGTCCAATTTGTTTTGTAGAATTTGAGGATGTTAGCTTTGCCACTAGAGCATTGGCAGAATTA
TATGGTAGTCAACTACCTCACCCCCGCCCTTCCCTCAACAACAAAGGAGGTATCAGGTTGAGCTTCTCTAAAAAT
CCATTAGGCGTTAGAGGCTCAAACAGTAGGAGTAAATCTGGTTACAGCTTTAATGGAAGTTACGGAAATCATGA

>YPL141C 0.74   Cold

ATGTCGTACACCAATAAACGTCATACATACTACGGAGGGTTTACCAACGATTTGTCTGACACATTTCAGTACCCT
CAAAGAACAGATGAGCAAAGGCGGAAACATGTAACGTTTGGTCCTTATATCCTAGGGTCGACGTTAGGAGAAGGA
GAGTTCGGAAAAGTGAAGCTAGGCTGGCCAAAGAATTTTTCAAACAGCTCTAATTCAACTTTTGACTTCCCAAAA
CAAGTAGCAATTAAACTTATCAAACGTGATAGCATATCGAATGATTACAGGAAAGAAGTTAAAATATACAGAGAA
ATCAACGCCTTAAAACACTTATCACATCCGAATATCGTAAAATTAGAAGAAGTGTTGCAAAATTCTAGGTACATT
GGTATTGTCCTAGAGTACGCATGCGGGGGAGAATTCTATAAATATATTCAAAAAAAAAGGAGGCTTAAGGAAATG
AATGCATGCAGGTTATTTTCTCAGCTAATAAGTGGAGTACATTACATACATTCTAAGGGACTTGTTCACAGAGAT
CTCAAATTGGAAAATCTATTACTAGACAAGAATGAGAACCTAGTTATTACAGATTTTGGGTTTGTCAATGAGTTT
TGCTCACGAAACGAATTAATGAAGACGTCATGTGGCTCTCCATGCTACGCAGCGCCTGAACTAGTGATAAGCGCC
GAACCATACGAAGCTAGAAAAGCAGATATATGGTCATGTGGAGTTATACTTTACGCTATACTAGCTGGATATTTA
CCGTGGGATGATGATCCAAATAATCCTGAAGGCAGCGATATAGGAAGACTTTATAATTATATTAACTCCACACCA
TTAAAGTTCCCTGATTATATTCTTCCTATACCTCGGGACTTATTAAGACGCATGCTGGTCTCAGACCCAAAGAAA
AGAATAAACCTGAAACAGATCAAGAAACATGAATGGCTAAAGCCTCATTCTTCATTCTTATCCATAACACCTGAT
GAGTGGGACAAACTAAACAATACTCAATCCGTGTTCAGATTAGCAAAACCAAGAAGAAGATATGGATCTAGACCA
CAATCCAGTTGTTCTACCTCATCTCTTGGCTCAAGGAGTGATAAAAGGGATTCACTGGTCATTGATTCAACTTTA
ATTACTTTCCCGGCTCCGCCTCAAGAATCACAAAATCATATTATAACCAGACCGGCTTCTATTGCATCTGACCAG
CGGCTGTCACCTATAAGAAGATCTAATAGGCACAATAGAAGTAACTCTGCTGCATCCGTTGCCTTGCAGGCAGTG
GTGAACGCAGATAGGGAATATGTGTTAAGTCATGAACAATCATTATCCCCAGTACAAAACATTAGACAAACTACG
GGAAACATGACTGCCAGTCTTTCACCTCCACCAGCCATAAGTCCAGGCGATATTATAATCGAAACCACACCTATT
AAAAGAAATACAATTTCTGGGAGTAGTATTGTACCAAGTTTAGAAGAAGAGTCGTCTACCACAATGCAAACAAGC
AAGATTCAACCAAATAACATGGCTTCATCACAAAATCATCAATACAATAAAAATAAGACCCAAAATAGTCTCCAA
TCTGCTAAAAATTTCTACCGTACGTCTTCCTCATCCCATACAAAGCCACGGCCAACTTCATACCATCCTGGCTCA
TACACAACACCTCCCTACAATTCAAATACGCTCTCCATATATGAGATCAACGAAAAAGCTAAAAGTAGCGCTTCA
TCACAAACGCTGAATCAAAGAGACACATCCCCATTTGATTCCACACCATACCTTGCCCTAGATACATGCATAACC
TCATCCTCTTCCATCGAAAGCTCTCCAAAGTTAATTACGCACGGCCAATTTTCAGTAGCAAAACCCTCTGTTGAT
TTGCAAAGCGTATCTGGTGACTTGATCAAGTATAAGAGGGACGCTGATGTAGTAACGAGAATCTATGATGAAAAA
TACAAACAGAAGCGCAAAAGTTTGAGATATAGCGGCATATTCAGTGACATCTCATGCGATACGGTCACCGAAGAG
TCTGATGAATTGAGGCCACCAGAATCTCCTCTTCAACAACATGAAGGACAAGAATCAATTGATAAGGCGAAAACG
GAGGACACAAGTGAAAAGGGTAGTAAAAGTAGCAACATCGCGAAAGCTACAGCACAAAAACACGTCAATAACCAT
CTAGAAAGATCACTTAACGAAGCAGAGTCAACAAAGAAAAGATTTAGCTTTCTTTCCTTGTACTCTTATGATACT
TCCAAATCTAGTTTATACTCATCAATGGATTCCAAACGGAAACCTTCTCCACCTTCCCAAAGACGCCCAAAAAAG
GATGACAGTTATCAAACAAATTCAAAGAATCATTATATCACTGCTTCAAATATGCAAACATCCCATCAAGTCTCA
AAAGACCTGCCTGCACCAACGATGGTGCAAAATAAATGTACTTTAGAAACTAAAAAAGCGGTCCGAAGCAACAGG
TCCTCCATAATGGTATCTGAGGTCAACAAGGCAAGCGTAGACAATAAAGCAGCCCAAAGTCCAGAACATTCAACC
GCAAAAAGAGTATTAGGGTTTTTCAAAAGAAGAAGTATGAAAATCTAG

>YGR292W 0.76   Cold
ATGACTATTTCTGATCATCCAGAAACAGAACCAAAGTGGTGGAAAGAGGCCACAATCTATCAAATTTACCCAGCA
AGTTTTAAAGACTCCAATAACGATGGCTGGGGTGATTTAAAAGGTATCACTTCCAAGTTGCAGTATATTAAAGAT
CTTGGCGTTGATGCTATTTGGGTTTGTCCGTTTTATGACTCTCCTCAACAAGATATGGGGTATGATATATCCAAC
TACGAAAAGGTCTGGCCCACATATGGTACCAATGAGGACTGTTTTGAGCTAATTGACAAGACTCATAAGCTGGGT
ATGAAATTCATCACCGATTTGGTTATCAACCACTGTTCTACAGAACACGAATGGTTCAAAGAGAGCAGATCCTCG
AAGACCAATCCGAAGCGTGACTGGTTCTTCTGGAGACCTCCTAAGGGTTATGACGCCGAAGGCAAGCCAATTCCT
CCAAACAATTGGAAATCTTTCTTTGGTGGTTCAGCTTGGACTTTTGATGAAACTACAAATGAATTTTACCTCCGT
TTGTTTGCGAGTCGTCAAGTTGACTTGAATTGGGAGAATGAAGACTGCAGAAGGGCAATCTTTGAAAGTGCTGTT
GGATTTTGGCTGGACCATGGTGTAGATGGTTTTAGAATCGATACCGCTGGTTTGTATTCGAAACGTCCTGGTTTA
CCAGATTCCCCAATTTTTGACAAAACCTCGAAATTACAACATCCAAATTGGGGGTCTCACAATGGTCCTAGGATT
CATGAATATCATCAAGAACTACACAGATTTATGAAAAACAGGGTGAAAGATGGTAGAGAAATAATGACAGTCGGT
GAAGTTGCCCATGGAAGTGATAATGCTTTATACACCAGTGCAGCTAGATACGAAGTCAGCGAAGTTTTCTCCTTC
ACGCACGTTGAAGTTGGTACCTCGCCATTTTTCCGTTATAACATAGTGCCCTTCACCTTGAAACAATGGAAAGAA
GCCATTGCATCGAACTTTTTGTTCATTAACGGTACTGATAGTTGGGCTACCACCTACATCGAGAATCACGATCAA
GCCCGGTCAATTACGAGATTTGCTGACGATTCGCCAAAGTACCGTAAAATATCTGGTAAGCTGTTAACATTGCTA
GAATGTTCATTGACAGGTACGTTGTATGTCTATCAAGGTCAGGAGATAGGCCAGATCAATTTCAAGGAATGGCCT
ATTGAAAAGTATGAGGACGTTGATGTGAAAAACAACTACGAGATTATCAAAAAAAGTTTTGGTAAAAACTCGAAG
GAAATGAAGGATTTTTTTAAAGGAATCGCCCTACTTTCTAGAGATCATTCGAGAACTCCCATGCCATGGACGAAA
GATAAGCCCAATGCTGGATTTACTGGCCCAGATGTTAAACCTTGGTTTCTCTTGAATGAATCTTTCGAGCAAGGA
ATCAATGTTGAGCAGGAATCCAGAGATGATGACTCAGTTCTCAATTTTTGGAAAAGGGCCTTGCAAGCCAGAAAG
AAATATAAGGAACTTATGATTTATGGTTACGATTTCCAATTCATTGATTTAGACAGTGACCAGATCTTTAGCTTC
ACTAAAGAGTACGAAGACAAGACGCTGTTTGCTGCTTTAAATTTCAGTGGCGAAGAAATTGAATTCAGCCTCCCA
AGAGAAGGTGCTTCTTTATCTTTTATTCTTGGAAATTATGATGATACTGACGTTTCCTCCAGAGTTTTGAAACCA
TGGGAAGGTAGAATCTACCTCGTCAAATAA

>YKL022C 0.79   Cold
ATGAAGTTTTGTCTTTATTGCTGTCATTGTTATATCGTTATTTGTGGAAAGGCTACACATTATTACAAGTCATCA
AAAGCCACATCAAACTTGAAATCGTCAAACAGGGTACTTATGAGAAACCCCATGTCGCCTTCGGAGCAACATTCA
CAACATAATTCTACATTGGCCGCCTCGCCATTTGTTTCTAACGTATCTGCAGCAAGAACACAACAGAGTTTACCA
ACCGATGCTCAGAATGATCGTTTGCAGCAACCCTGGAACAGAACCAATACGGCTACGAGTCCCTACCAGTCGTTA

```
GCAAATAGCCCTTTAATACAGAAGTTGCAAGCGAATATTATGACTCCGCACCAGCCATCTGCTAATTCTAATTCT
AATTCCAATTCCATTACGGGCAATGTTGTGAACGACAATAATTTGTTAGCTTCTATGTCTAAGAATAGTATGTTC
GGTTCTACCATACCGTCCACATTAAGGAAGGTGAGCTTACAGCGTGAATATAAGGATTCAGTTGATGGTGTGGTT
CGTGATGAAGATAATGATGAGGATGTTCATAACAATGGCGATGCAGCTGCGAATGCTAATAATGATCGGGAGAGT
AAACTAGGGCATAATGGGCCATTGACGACAACAACATTAACGACAACAACTACAGCAACTCAACTAGATGTTTCT
GAATTGTCAGCTATAGAAAGATTGAGACTTTGGAGGTTCGACGCATTGATGCAGCATATGTATAGGACCGCAGAA
TATATTGCTGATAAAGTGTATAACATATCCAATGATCCTGATGATGCCTTCTGGCTCGGCCAAGTATATTACAAT
AATAATCAGTACGTAAGGGCTGTAGAACTTATTACCAGGAACAACTTGGATGGCGTTAATATCCTGTGTCGATAT
CTGTTGGGACTCTCCTTTGTTAAATTACAGAGATTTGATGACGCTCTAGATGTTATAGGCGAATACAATCCATTC
AGCGAGGACCCATCTACGACGGCAGCAAACACCATGAGCAATAATGGCAATAACAGCAATACGTCACAGCCAGTT
ACTGACGGCGGTATAAAAATGGAGTCATCATTATGTTTTCTGAGAGGGAAAATATATTTTGCACAAAATAATTTT
AACAAGGCAAGGGATGCATTTCGTGAAGCGATTTTGGTAGATATAAAAAATTTTGAAGCTTTCGAAATGCTCCTG
TCCAAGAACCTGTTAACTCCACAAGAGGAATGGGACCTGTTTGACTCTTTGGATTTCAAAGAATTTGGGGAAGAT
AAAGAGATTATGAAGAATCTTTATAAGATCAACCTATCTAAATACATCAACACGGAAGATATAACGAAGTCCAAT
GAGATTTTAGCGAAAGATTATAAATTAGCTGACAATGTAGATGTCGTAAGAAGTAAGGTGGATATCTGCTATACG
CAATGCAAATTCAACGAATGCTTAGAGTTGTGCGAGACCGTTTTGGAAAACGACGAATTTAATACGAATATCTTG
CCAGCATACATTGGATGTCTATATGAACTATCAAATAAAAATAAGCTTTTCCTTCTGTCGCATCGATTAGCGGAA
ACTTTCCCGAAGTCTGCGATAACATGGTTTAGCGTTGCGACCTATTATATGAGCTTGGACAGAATTAGTGAAGCA
CAGAAATACTATTCCAAATCCTCAATACTGGATCCAAGCTTTGCTGCCGCATGGCTGGGATTTGCACACACGTAT
GCCCTAGAAGGTGAACAAGACCAAGCATTAACAGCATACTCTACAGCCTCCAGATTCTTTCCTGGAATGCACTTA
CCAAAACTGTTTCTCGGGATGCAGTTTATGGCGATGAATTCATTAAATTTAGCAGAATCGTATTTTGTTCTGGCA
TATGACATTTGTCCAAACGATCCATTAGTACTCAATGAAATGGGTGTAATGTATTTTAAGAAGAACGAATTTGTC
AAAGCCAAGAAATACCTGAAGAAGGCGTTGGAAGTGGTGAAAGATCTTGATCCAAGTTCAAGAACGACAATATCA
ATTCAATTAAATCTAGGACACACTTACAGAAAGTTAAATGAGAACGAAATTGCCATTAAATGTTTTAGATGCGTT
TTGGAGAAAAATGATAAAAACTCTGAAATTCATTGTTCCTTAGGTTACTTATATTTGAAGACGAAGAAATTACAA
AAGGCCATTGATCATTTGCACAAATCATTGTACCTAAAGCCTAATAATTCATCTGCAACAGCGCTTTTGAAAAAT
GCCCTAGAGCTAAACGTGACGTTATCATTGGATGCCAGCCACCCACTTATTGACAAGTCGAATTTAATGAGTCAG
GCAAGTAAGGACAAGGCTTCGCTCAATAAAAAAAGATCTTCATTGACTTATGACCCTGTCAACATGGCTAAAAGG
TTGAGAACACAAAAGGAGATCTTTGATCAGAATAACAAAGCTCTAAGAAAGGGAGGTCATGACAGCAAAACTGGA
AGTAATAATGCCGACGATGATTTTGACGCAGATATGGAACTGGAATAA

>YBR001C  0.68   Cold
ATGGTAGATTTTTTACCAAAAGTAACGGAAATAAATCCTCCATCCGAAGGTAATGATGGTGAAGATAACATAAAG
CCACTTTCAAGTGGTTCAGAGCAGCGGCCGTTGAAAGAAGAGGGGCAACAAGGTGGTAGAAGACATCACCGGCGG
TTGTCCTCTATGCATGAGTATTTTGACCCGTTTTCCAACGCAGAGGTCTATTATGGACCAATAACAGATCCAAGA
AAACAGTCAAAAATTCATAGACTAAATAGAACCAGAACTATGAGTGTTTTCAATAAAGTTTCTGACTTCAAAAAC
GGAATGAAGGATTATACCCTGAAAAGGAGGGGTTCTGAAGACGACAGCTTCCTCAGCAGCCAAGGTAACCGTAGA
TTTTATATTGATAATGTGGATCTCGCTTTAGATGAGCTGCTCGCTAGTGAGGACACAGACAAGAATCACCAAATT
ACCATAGAAGACACTGGGCCCAAAGTTATTAAAGTCGGGACGGCCAATTCCAACGGTTTCAAACATGTAAATGTT
AGGGGAACATATATGCTTTCTAACTTGTTGCAGGAACTAACCATTGCAAAAAGTTTCGGACGACACCAAATATTT
TTGGATGAGGCGCGTATAAATGAAAACCCTGTTGATAGATTATCAAGATTGATAACGACTCAATTTTGGACGAGT
TTAACGAGAAGAGTTGATCTTTATAATATTGCAGAAATTGCTAGAGACTCAAAAATAGATACGCCAGGCGCTAAA
AATCCAAGAATTTATGTTCCATATAACTGCCCAGAGCAGTACGAATTTTACATTCAGGCATCTCAAATGAACCCG
TCCTTAAAACTTGAAGTGGAGTACTTACCAAAAGATATTACAGCAGAGTATGTTAAATCATTAAATGATACACCG
GGTTTGTTAGCCCTGGCTATGGAAGAACACGTTAATCCATCTACTGGTGAAAGGTCCCTAGTTGGTTATCCTTAT
GCGGTACCTGGCGGTAGATTCAATGAATTGTATGGCTGGGATTCATATTTAATGGCACTGGGACTCATAGAAAGT
AATAAGGTTGATGTTGCAAGAGGTATGGTGGAGCATTTTATATTCGAAATTGATCACTATAGTAAAATTTTGAAT
GCTAATAGGAGTTATTATCTTTGCAGATCTCAGCCACCATTCCTAACCGATATGGCTCTGCTCGTATTTGAAAAG
ATAGGAGGTAAGAATAATCCAAACGCTATTCAGTTGTTAAAACGCGCGTTCAGAGCTGCTATTAAAGAATATAAA
GAGGTGTGGATGTCGAGCCCTAGGTTAGATTCTCTGACAGGGCTGTCTTGTTATCATTCTGATGGTATTGGTATC
CCTCCGGAAACTGAACCAGATCATTTTGATACTATATTATTACCGTATGCTGAAAAGTACAATGTTACTCTGGAA
AAGCTTAGGTACCTTTATAACGAAGGCATGATTAAAGAACCCAAACTGGATGCATTTTTTTTACATGATCGTGCC
GTGAGAGAGTCAGGACATGACACGACTTATAGGTTTGAAGGTGTATGTGCATATTTAGCGACAATTGATCTAAAC
TCCTTGCTCTATAAATATGAGAAAGATATTGCTTTCGTCATTAAGGAGTATTTTGGTAATGAATATAAAGATGAG
AATGATGGAACGGTAACCGATTCTGAACACTGGGAGGAGCTAGCTGAACTGAGGAAAACAAGGATTAATAAGTAT
ATGTGGGACGAAGATTCAGGGTTTTTCTTCTATTATAACACGAAACTGAAATGCAGAACGTCTTATGAGTCTGCA
ACGACTTTTTGGAGTTTGTGGGCTGGTCTTGCAACTGAAGAGCAAGCAAAAATCACAGTAGAAAAAGCTCTTCCT
CAGTTAGAAATGCTCGGCGGACTAGTTGCATGCACAGAAAAATCAAGAGGTCCTATTTCTATTGATAGACCGATC
AGGCAATGGGATTATCCTTTTGGTTGGGCGCCGCACCAAATATTAGCCTGGAAAGGTTTATCTGCATATGGTTAT
CAACAAGTAGCTACAAGATTGGCTTATAGGTGGCTGTACATGATTACAAAATCATTTGTTGATTATAACGGAATG
GTGGTAGAAAAATATGATGTGACAAGAGGGACTGATCCGCATCGCGTTGATGCGGAATATGGTAATCAAGGTGCT
GATTTTAAGGGCGTCGCTACAGAAGGCTTTGGTTGGGTGAACACAAGTTATTTGCTCGGATTGAAATATATGAAT
AATCATGCAAGAAGGGCTCTCGCTGCTTGTAGTCCACCATTGCCATTTTTCAATAGTTTGAAACCCTCTGAGAAA
AAATTGTATTACCTATAA

>YPL004C  0.81   Cold
```

ATGCACAGAACTTACTCTTTAAGAAATCAAAGAGCTCCAACGGCTGCTGAGCTACAAGCTCCTCCACCACCACCA
TCCTCAACGAAGTCCAAGTTCTTCGGAAAGGCCTCTATTGCGTCTAGTTTTAGAAAAAATGCGGCTGGTAACTTT
GGGCCAGAGTTGGCTCGTAAATTATCTCAATTGGTTAAGACTGAAAAAGGTGTCCTTAGAGCTATGGAAGTTGTT
GCCAGCGAACGCCGTGAAGCTGCAAAGCAACTGTCTCTATGGGGTGCTGACAATGATGATGATGTTTCCGACGTA
ACCGACAAATTAGGCGTCTTAATATATGAGTTGGGTGAACTACAGGACCAGTTCATCGATAAATATGATCAATAC
AGAGTCACATTAAAATCAATTAGGAACATTGAAGCCTCTGTTCAACCTTCGAGAGATCGTAAGGAAAAGATCACA
GATGAAATTGCCCATTTGAAATATAAAGATCCTCAATCAACAAAAATCCCAGTTTTAGAGCAAGAACTAGTTCGT
GCCGAAGCTGAATCTTTGGTTGCGGAGGCTCAATTATCGAATATCACACGTGAAAAATTAAAGGCTGCTTATAGT
TATATGTTTGATTCATTAAGAGAGCTTTCTGAAAAATTTGCCCTGATTGCTGGATACGGAAAGGCCTTATTGGAG
CTGCTAGATGACTCTCCTGTCACTCCAGGTGAAGCTAGACCAGCGTACGATGGCTATGAAGCTTCTCGCCAAATT
ATTATGGATGCTGAATCGGCCTTGGAATCTTGGACCCTAGACATGGCTGCAGTCAAACCAACTCTTTCATTTCAT
CAGACCGTTGATGACGTCTATGAAGACGAAGATGGTGAAGAAGAAGAAGAACCTGAAATCCAAAATGGAGATATC
CCCGGTCAAGTAGTCGAAGAAGAGGAAGTTGAATGGACTACTGAAGTTCCTGTAGATGATGAAGCCCATGAGGCC
GATCATCATGTTAGTCAAAACGGTCATACCTCCGGTTCTGAAAACATCTGA

>YLR003C 0.81 Cold
ATGTCTAATCCAGATGATTTAGATGATGGACTCGCCTATGATTTTGATGCCGAACATGAAGTAATTTTTGACGCC
AAGGATGGTAGTCCCCCAACCAAAAAGGTACAAAAGAGGTCTATAGAACAAGATGACGATGACGTTGATGATATA
GATGGGAAAAAAGAGGAGAGAAATTCAGAAGATGATTCGAATAGACCTATTTCGAAAAGACAGAAAAAGCTACAG
AAAAAATCAAAGCTAATTGAGAAAAAGAAAGAGGAAAGCCAGTATATCGTCTCACAACGAAAAGCACTTCCAGCA
AGTTCACCAGAAAAAATTATTGAGTACCTAACAACTTTGATACGAGAAAAAAATCCTGACTTGAGTGTTTTAGAA
TTAGAGGAACTTTACTTCAAAAGAAATGATTTTCTTTCGACAGAAAAATTTGACGCTGAACGCAGATTAAGCAAT
TTCCCAGCCTTTATTCAAAAATTTTCCGTAGCTCCAAAAAAAATTGTTTTTTCTATGTCTAATATTAGAGTAGCA
GACGTTTATCGTAGTCTAAACGGTGGCAAAAATTGCGTCAAATTATTCTCTAAAAGTAAACTAAAGGATGACATA
GCCACCGTGGAACGTTTACTTACTGACAGCTCTAAAAAATCAAACAAAAATAAAGATTCACTATATTTTATTGCC
ACTCCAACAAGAATGCAGAAAATTATAGAAGCTACTGACTTACTTTTCCAAGGTAAGGAAAAGTTAGATATCATT
CTTGATGCAAGTTATTTAGATCCTAAGGATAATACTATATTATCATTCGAGAATGCAGCCGTCCTCTGCCAAGTC
TTGAAAACCTTTTTGAATAAAAAAAGCTCCGTGAAAATACTGTTATATTAA

>YBL010C 0.8 Cold
ATGAGCGATAGAGATCAGATAGAACCTGTCACAAATGCATTAGATGCAGAAAGTGATTCTAGTGATGATTTTGGG
AATTTTTCAGATGCCTCAGTTGAAAATGACCTCTATAACCAGAATTCTACTTTAACGACGTCTTCCGAATCTGTA
GTAGATAACTGCTTGAATAAGATACTACCAAAGGGAGAATTTGACCTGGAAGAAGAAACCATAAAGAATGACTGT
TTCAAGCTGAGCAAACTTATTGAAGATGAACGACCCCATGTCATTTATGAACAACTCGTACAATTAGATCCTGTG
TTACAGCCATTCATATGGAATAAATCTCATATACGCAGAAACTTGCTCCACATTTTGAGATTATCGGATAATAAT
GGCTCTGAGGGTGTTGGTACGAAGAGGGAGGAAGAACCGTTGAATGATGAGTTGTTCAAAAGGATATGCGATGCA
GTGGAGAAAAATGAGCAAACGGCTACTGGTCTCTTCCTGAGGGATAATTTCAAAATTGACTATACACCACCAATG
ACTTTAAAATCTCTTCAAAAGGAAGAGGAGCGTGAGCAAGAGCAGCATATACCGCAATTGCTTATGGCAGATTTT
ACTAGTATGGATGAAGAATCTTTACGCCAATACCACGATACATTGTGTCAATCCATTGACTTTTTGGTTAGTAAA
TCAAGATCATTGAAGAAACAACAGCGAGACCTCCTAAAGGATAAGACAACTTTTGAAAATGTAGTGACGAATTTG
ACAGGACATACCCAAAGACTACAGCGAGACGAAATTGCATTGTATAACAAAAAGCGAAACAAAAAGAAAAGGTTC
AGTTGGGTAGGATACTAA

>YLR126C 0.77 Cold
ATGACAGTAAAAAAAAATAGCTATTCTTTATACAGATGAAGATAACGAATGGAGTAAACCATGGGGCAATTTTGTT
GATATGGCTATCAAGCTATTAGAACAGACCAGGAAATTGGAATGTATCGCAGAAGATGTCGAATATGAAGTGTTC
CACGTACAAAAAAACGTGTTCCCTCAATTGTCAGATCTTCAGAAAGATGAGTATTTAGGCATATACATCACTGGT
TCAAAATATGATTCATTTGATAATGAAATTGAATGGATTATGAAATTAAGAAGTTTCTTAAATGAGATGCTAACT
AGCAAGACTGAATATCCGCCCGTAGCTGGAATATGTTTTGGTCATCAAGTTATCGCGGCTGCTCTAGGAAGTTCA
GTTGGACGGAATCCGAAAGGGTTTGAAGGTGGGGTTGTGTCACTAAAACTGAACTCTGTGGGTCAAAAGTTATTT
GGTGCTCAAGAATTAAATCTTTCTGAAGTGCACAGTGACTGCGTGTTCGATGTCCCAGAGGGATACCAAAATTGG
GCGTCAAGTGAAAAATGTCAAAACCAAGGATTTTACAGGCAAAACAGAGTGCTGACGTTTCAAGGACACCCTGAA
TTCAATAGTGATGTCGCTCAGAAGGGGCTTTTGAAGTCTCAAGACAAGCTTACCCTTGAAGAGTTTAATAGATAC
GAAAGACAGTGCCAGGAGCTGGATAACAATGGCATACAAGCTGCCAGGAATATTTGGAGGTTATTCTTGCAAAAA
ATATGA

>YGR132C 0.74 Cold
ATGTCTAATTCTGCCAAACTTATCGATGTCATCACCAAGGTGGCGTTGCCCATTGGTATAATTGCTAGCGGGATT
CAGTACTCCATGTATGATGTGAAGGGTGGTTCTCGTGGTGTTATTTTCGACAGAATCAATGGTGTAAAGCAACAG
GTTGTGGGTGAAGGCACTCATTTCTTGGTGCCTTGGCTACAGAAGGCGATCATATACGATGTGAGGACGAAACCA
AAGAGCATTGCTACCAATACTGGTACGAAGGATTTGCAAATGGTGTCATTGACCTTGAGAGTCTTACATAGACCA
GAGGTCTTACAGCTACCCGCAATATACCAAAATTTGGGTCTCGATTACGACGAAAGAGTGTTACCATCTATCGGC
AATGAGGTTTTAAAGTCTATAGTAGCTCAATTTGATGCTGCTGAGTTAATTACTCAGAGAGAAATTATTTCTCAA
AAAATCAGAAAAGAGCTTTCTACGAGGGCCAACGAATTCGGTATTAAGTTGGAAGATGTCTCTATCACTCATATG
ACGTTTGGTCCCGAATTCACGAAAGCAGTTGAGCAGAAGCAGATTGCACAGCAAGATGCCGAAAGAGCCAAATTC
CTTGTCGAAAAGGCAGAGCAAGAGAGACAAGCTTCTGTTATCAGAGCTGAAGGTGAAGCAGAAAGTGCTGAATTC
ATTTCAAAAGCCTTAGCTAAAGTTGGTGATGGTCTGTTATTGATTAGAAGATTAGAAGCTTCTAAGGACATCGCT

CAAACATTAGCAAACTCATCTAACGTTGTCTATTTACCAAGTCAACATTCTGGTGGTGGTAACAGCGAGTCTTCG
GGATCACCAAATTCCTTGCTTTTGAACATTGGCCGTTAA

>YJL110C 0.81 Cold
ATGGCATCGCAGGCTACAACTCTTCGAGGCTATAACATTAGAAAACGAGATAATGTATTTGAACCAAAATCAAGT
GAAAACCTCAACAGCTTAAATCAAAGCGAAGAAGAAGGGCATATTGGGAGATGGCCACCTTTAGGTTATGAAGCA
GTATCTGCCGAGCAAAAATCGGCAGTTCAATTGCGTGAATCGCAAGCAGGAGCGTCAATAAGCAACAATATGAAT
TTTAAGGCGAATGACAAGTCTTTTTCCACATCTACTGCTGGAAGAATGAGTCCGGATACGAATTCATTACACCAT
ATATTACCTAAAAATCAAGTTAAGAATAATGGACAAACAATGGATGCCAATTGCAATAATAACGTATCCAATGAT
GCTAATGTTCCTGTTTGTAAGAACTGTTTAACCTCTACAACACCATTATGGAGAAGAGATGAGCATGGAGCTATG
CTTTGTAATGCGTGTGGTCTCTTTTTAAAGCTTCATGGGAAACCCAGGCCAATTAGTTTGAAAACTGATGTAATA
AAGTCTCGAAATAGGAAAAGTAATACAAATCATGCACATAATCTGGACAACTTTCGGAATCAGACGCTGATTGCA
GAGCTTAAGGGTGATTGTAATATAGAATCAAGCGGTCGCAAAGCTAACAGAGTAACATCTGAAGATAAAAAGAAA
AAAAGTTCGCAACTTTTAATGGGAACATCATCTACTGCGAAGATATCCAAGAAGCCAAAAACGGAGTCTAAGGAA
AGAAGCGATTCTCACCTATCAGCAACAAAATTAGAGGTACTGATGTCGGGAGATTGTTCGAGACCAAACTTAAAG
CCTAAACTGCCCAAACAAGATACTGCTATATACCAAGAGAAGTTACTTACGTTCCCAAGTTATACGGACGTTAAA
GAGTATTCAAATTCTGCACACCAATCTGCTTTTATCAAAGAACGGTCGCAATTCAACGCAGCCTCTTTCCCCCTC
AATGCTTCACATTCAGTAACATCAAAAACAGGCGCAGATTCTCCTCAATTACCTCACTTATCAATGCTGCTTGGA
AGCTTGAGCAGTACTTCAATATCAAATAACGGAAGTGAAATAGTGTCCAATTGCAATAATGGTATTGCCTCTACC
GCCGCAACTCTGGCACCCACTTCTTCACGGACGACTGACTCTAATCCATCCGAGGTACCGAATCAAATTAGATCG
ACGATGTCTTCCCCAGATATAATATCTGCTAAGCGTAACGACCCAGCCCCTTTATCTTTCCACATGGCTTCTATT
AACGACATGCTTGAGACGAGAGATCGTGCGATTAGCAACGTGAAAACCGAGACGACACCGCCTCATTTCATACCG
TTTCTACAATCTTCTAAAGCTCCCTGTATATCCAAAGCAAATTCACAATCCATCTCAAATAGTGTTTCTAGTTCT
GATGTTTCTGGACGAAAATTTGAAAATCACCCAGCTAAAGATTTAGGTGATCAGTTATCCACTAAATTGCACAAA
GAAGAAGAAATTATAAAGCTCAAAACTAGAATAAATGAGTTAGAACTTGTTACAGATTTATATAGGAGACATATC
AATGAATTAGACGGGAAATGTCGAGCTCTTGAGGAACGTTTGCAAAGGACAGTAAAACAAGAAGGGAATAAAGGA
GGATAG

>YAR071W 0.77 Cold
ATGTTGAAGTCAGCCGTTTATTCAATTTTAGCCGCTTCTTTGGTTAATGCAGGTACCATACCCCTCGGAAAGTTA
TCTGACATTGACAAAATCGGAACTCAAACGGAAATTTTCCCATTTTTGGGTGGTTCTGGGCCATACTACTCTTTC
CCTGGTGATTATGGTATTTCTCGTGATTTGCCGGAAAGTTGTGAAATGAAGCAAGTGCAAATGGTTGGTAGACAC
GGTGAAAGATACCCCACTGTCAGCAAAGCCAAAAGTATCATGACAACATGGTACAAATTGAGTAACTATACCGGT
CAATTCAGCGGAGCATTGTCTTTCTTGAACGATGACTACGAATTTTTCATTCGTGACACCAAAAACCTAGAAATG
GAAACCACACTTGCCAATTCGGTCAATGTTTTGAACCCATATACCGGTGAGATGAATGCTAAGAGACACGCTCGT
GATTTCTTGGCGCAATATGGCTACATGGTCGAAAACCAAACCAGTTTTGCCGTTTTTACGTCTAACTCGAACAGA
TGTCATGATACTGCCCAGTATTTCATTGACGGTTTGGGTGATAAATTCAACATATCCTTGCAAACCATCAGTGAA
GCCGAGTCTGCTGGTGCCAATACTCTGAGTGCCCACCATTCGTGTCCTGCTTGGGACGATGATGTCAACGATGAC
ATTTTGAAAAAATATGATACCAAATATTTGAGTGGTATTGCCAAGAGATTAAACAAGGAAAACAAGGGTTTGAAT
CTGACTTCAAGTGATGCAAACACTTTTTTTGCATGGTGTGCATATGAAATAAACGCTAGAGGTTACAGTGACATC
TGTAACATCTTCACCAAAGATGAATTGGTCCGTTTCTCCTACGGCCAAGACTTGGAAACTTATTATCAAACGGGA
CCAGGCTATGACGTCGTCAGATCCGTCGGTGCCAACTTGTTCAACGCTTCAGTGAAACTACTAAAGGAAAGTGAG
GTCCAGGACCAAAAGGTTTGGTTGAGTTTCACCCACGATACCGATATTCTGAACTATTTGACCACTATCGGCATA
ATCGATGACAAAAATAACTTGACCGCCGAACATGTTCCATTCATGGAAAACACTTTCCACAGATCCTGGTACGTT
CCACAAGGTGCTCGTGTTTACACTGAAAAGTTCCAGTGTTCCAATGACACCTATGTTAGATACGTCATCAACGAT
GCTGTCGTTCCAATTGAAACCTGTTCTACTGGTCCAGGGTTCTCCTGTGAAATAAATGACTTCTACGACTATGCT
GAAAAGAGAGTAGCCGGTACTGACTTCCTAAAGGTCTGTAACGTCAGCAGCGTCAGTAACTCTACTGAATTGACC
TTTTTCTGGGACTGGAATACCAAGCACTACAACGACACTTTATTAAAACAGTAA

>YGR007W 0.75 Cold
ATGACGGTAAACTTAGATCCGGATAAAGTTTGGATAGACGGCTGCTTTGATTTCACACATCATGGGCATGCGGGA
GCCATTTTGCAGGCTCGTCGAACAGTTTCAAAAGAAAATGGTAAACTATTCTGTGGTGTGCATACCGATGAAGAT
ATTCAGCATAACAAAGGAACACCAGTAATGAACTCTTCAGAAAGATATGAGCATACTAGATCAAATAGGTGGTGT
TCGGAAGTGGTTGAAGCGGCGCCATATGTTACAGATCCCAATTGGATGGATAAATATCAGTGCCAATATGTTGTT
CATGGTGATGATATCACCATAGATGCTAATGGAGAAGACTGCTATAAGTTGGTGAAAGAGATGGGGCGCTTCAAG
GTAGTTAAAAGAACATATGGCGTAAGTACTACCGAGATTATACATAGAATATTGACGAAAAAATCTTTGCCACCG
ACTCATCCTGATTATTATCCCACCACCCAGGAATTGAGTTTCTATTCTGTCGCCCAAGATGCCGTTTCAAAACAT
TGTTATGTCTTTCAGAGGGATTTGGACAATGTCTTGGTCAATGGTGGATACAAATTCGATGCAGAAGATTGTGTT
TATGTTGACGGGGATTTTGACTTATTCCATATGGGGGATATTGATCAGTTAAGAAAGCTGAAAATGGATCTTCAC
CCCGATAAGAAATTGATTGTTGGTATTACCACAAGCGATTACTCGAGTACCATTATGACGATGAAGGAACGCGTC
TTGAGCGTTTTAAGTTGTAAATATGTTGATGCCGTTATTATCGATGCTGACGCCACCTCAATGTCTCAGTATAAC
TGCGAGAAGTATCATATTGGCACGGCTGTCCTCACTGCTGCCGGAAAATTCAGTGAATACTTGACTAAAGAGCTT
ATTGTGAAGAGGGTGGAATCGCAAAGGGAGGTTTACATTGCGAGGAACCAAAAAAAGGGAATGTCCATATAA

>YNL214W 0.78 Cold
ATGACATCGATTAACAGTTTTCCCAGGAATATTGACTGGCCTTCCAACATAGGCATTAAAAAAATAGAGGGAACT
AATCCAACGGTGAATGCCATCAAGGGCTTGTTATATAATGGTGGATCAATTTATGCATTTCTATATTTTGTTATT

GCTATGTTTGTTGAACCAACGCTACAAAAGCAGTACCAGCAGAGAAATGATTTTTCCTTGTTTGTTTTGTTGCGT
TTGAGAAGAATCATAGCGCAATTGCAAAAACGACTGGTGATGACCCCAGTATCGTCGTTGGGGTTCAACGAACAG
AATAACTTTGTGGAGAGGTCCACTCAAACTTCAGACGACAATATAATACGAGAAGATAATAGCCATTGGGCTGAA
ATGATTTATCAACTGCAAAATATGAAACAAGAATTACAGTATTTTAACAGATCCTCAGGCCAACCATCTGAAAGT
ATAGACGATTTTGTCTTTCAAATTAAGATGGTGACTGACCAGGTTGAGTTAACAGATAGGTCCCGAGCTTTCTCA
AATAAATCAAGAAATATTATACAGGGAATCCGAGAAATCAAAGGTTGGTTTGTGAATGGCCAAGTGCCAAGGTAA

>YOR011W 0.8Cold
ATGTCAATTTCAAAGTACTTCACTCCCGTTGCTGACGGGTCACTCACTTTCAATGGCGCGAACATTCAATTTGGC
GCCGATGCTCAAGGCGAGTCAAAAAAGAGTTATGACGCTGAGGACAGCATGCCGAATCCGGCAAATCAACTAAAT
GACATAACCTTCCAAGCAGAGGCTGGTGAAATGGTTTTGGTTTTGGGTTATCCCACATCCACTCTATTTAAGACT
TTGTTTCATGGTAAAACTAGTTTGTCATACTCTCCTCCAGGCTCGATTAAATTTAAAAATAATGAGTTTAAGAGC
TTTTCCGAAAAATGTCCCCACCAAATCATTTACAATAATGAACAAGATGTGCATTTCCCATTTCTAACGGTAGAA
CAAACAATTGATTTTGCCTTGAGTTGTAAGTTCGATATTCCAAAAGGTGAGCGCGATCAAATAAGAAATGAACTT
CTAAGAGAATTCGGCCTGTCTCATGTATTGAAAACTATTGTAGGAAATGATTTTTTCCGTGGTGTTTCTGGTGGT
GAGCGTAAACGTATTTCTATTATTGAAACGTTTATTGCTAATGGTTCCGTTTATCTATGGGATAATTCTACTAAA
GGTTTAGATTCCGCCACAGCTCTCGATTTTTTGGAAATTCTTAGAAAAATGGCAAAAGCTACTCGTTCTGTAAAC
TTAGTCAGAATTTCCCAGGCAAGTGATAAAATTGTTGATAAGTTTGACAAGATTCTTATGCTATCCGATTCCTAC
CAGCTTTTCTATGGTACGGTCGATGAGTGTTTGACTTATTTCGTGACACTTTAGGTATTGAGAAAGATCCTAAC
GATTGTATTATTGAATATCTGACCTCTATCTTAAATTTTCAGTTCAAAAATAAAAATTTGGGGAATTTATCAAAT
TCATCATCTGCTAGCGTTCTCAAAACCGCAACAGGGGAAGTCACTAAGTATACTTATAATTCTGATTTCGATTTA
TATGATCAATGGAAACATTCTTCGTACTATAGAAATATAAAGCAGCAAATCCAGGGGTCTTCAATTGATGACTCC
ATTAAGGAAGTGGATCCCTCTGATGTCTCACCTATTTTTAATATTCCGTTGAAGAAACAATTATTATTTTGCACA
AAAAGAGCTTTTCAACGAAGTTTGGGTGATAAAGCTTATATGACGGCACAATTTATTTCTGTCGTTATTCAATCT
TTGGTCATTGGTTCACTGTTTTACGAAATTCCGTTGACTACCATTGGTTCATACTCAAGAGGTTCTTTAACCTTT
TTCTCCATTCTTTTCTTCACTTTTCTTTCTCTTGCAGATATGCCTATTGCCTTCCAAAGACAACCCGTCGTTAAA
AAGCAATCCCAACTTCACTTCTATACTAACTGGGTTGAAACCCTTTCAACAACAGTGTTCGACTACTGCTTTAAA
CTTTGTTTGGTAATTGTATTCAGTATCATCCTATACTTCCTTGCTCACCTGCAATACAAGGCTGCAAGATTTTTC
ATTTTCCTCCTGTTCCTTTCCTTTTACAATTTCTGTATGGTGTCCTTATTCGCTTTGACGACACTAGTTGCTCCA
ACCATTTCAGTTGCAAATTTATTTGCAGGTATTCTGCTACTAGCTATAGCAATGTATGCTTCTTACGTCATCTAC
CTGAAAAATATGCATCCTTGGTTTGTATGGATTGCTTATCTAAATCCTGCAATGTACGCTATGGAGGCAATACTG
TCTAATGAGCTTTACAATTTGAAGCTGGATTGTAGTGAGACAATTGTTCCAAGAGGTCCTACTTACAACGATGTT
CCATTTTCCCATAAGGCCTGTGCTTGGCAAGGTGCTACTCTGGGTAATGATTACGTTAGAGGCCGTGATTACTTG
AAGCAAGGTTTATCCTACACGTATCATCATGTGTGGAGAAACTTTGGTATTATCATTGGTTTCCTAGTATTCTTT
ATTGCCTGTACTCTGTTTGCATCTCAATATATTAAGCCTTATTTCAATAAGGATGAAATAGAGCGCAACAATAGT
CGTTTGACAAGATGGCTACCATTTTTGAATAAAAAGAGAGGTACCAGGTCCTCTGCAAGAAATGACAGTAAGTAT
GTTGGTATCCCAAAGTCGCACTCCGTTTCGTCTTCCTCGTCTAGCCTGTCGGCTGTTCCATATCAGATATCACCC
TCAAATAAGGAAATGGCTCTAAACGATTATAACGAGCAACCTATCACGGAAACAGTAGAGACTCAAAAGCATATC
ATCTCTTGGAAAAATATCAACTACACAGTTGGTACAAAGAAACTGATTAATAATGCATCCGGTTTCATCAGTTCT
GGTTTGACCGCCTTAATGGGTGAGTCCGGTGCAGGTAAAACTACTTTGCTGAATGTCTTATCACAAAGAGTTGAG
ACAGGTGTTGTTAGCGGCGAAATTTTGATCGATGGCCACCCATTGACAGATGAGGACGCGTTTAAGAGAAGTATT
GGTTTTGTTCAACAACAGGATTTGCATCTAGATTTGCTGTCTGTGAAAGAATCTCTGGAAATATCATGTCTTTTG
AGAGGTGATGGTGACAGGGCATACTTAGACACTGTTTCAAATTTACTGAAATTGCCATCTGACATTTTAGTCGCT
GATTTAAACCCGACTCAAAGGAAGCTTTTATCTATCGGTGTTGAACTTGTTACTAAGCCTTCACTTTTATTATTC
CTAGACGAACCAACCTCTGGGCTGGACGCTGAGGCTGCACTAACAATCGTCAAATTTTTAAAACAACTTTCTTTA
CAGGGTCAGGCTATTTTTTGTACCATTCATCAGCCTAGTAAAAGTGTCATCAGCCATTTTGACAATATTTTCTTA
TTGAAAAGGGGTGGTGAATGTGTCTTCTTTGGACCAATGGACGATGCCTGCGGCTATTTCATGTCTCATGACAAC
ACGCTCGTTTACGATAAAGAACATGATAACCCTGCAGATTTCGTCATTGATGCAGTAGGTAACAGTAACTCCTCG
GCAGGGAAAGATACAGCGGAGGAAGCTCTTACTTTGAACAAAGAAGCCATAGATTGGAGTGCATTATGGGAATCT
TCTGTAGAAAAGAAACTAGTTAAAAAAGAAACAGCTAGGTTAGAAGATGATGCTCGTGCGTCTGGTGTTGATTAC
ACAACCTCATTGTGGAAGCAGCCTTCCTACTTACAGCAATTAGCTCTTATTACAAGAAGACAATATATTTGTACG
AAAAGAGACATGACTTATGTCATGGCCAAGTACTGTTTGAATGGTGGCGCTGGTTTGTTTATCGGTTTCTCATTT
TGGCACATTAAGCATAATATTATCGGTTTGCAGGATAGTATCTTCTTCTGTTTCATGGCTCTTTGTGTTTCATCT
CCATTGATCAATCAAATTCAGGACAAAGCCTTGAAAACAAAGGAAGTTTATGTTGCTAGGGAGGCGAGATCTAAC
ACTTATCACTGGACTGTTCTTCTTTTGTCACAGTCAATTATTGAACTACCACTGGCACTTACAAGTTCCACACTA
TTTTTCGTCTGTGCCTTTTTCTCTTGTGGATTTAACAACGCTGGCTGGAGTGCCGGTGTTTTCTTTTTAAACTAT
ATGCTTTTCGCTGCTTATTACTCGACTCTTGGTTTATGGCTGATCTATACTGCTCCTAATCTACAAACTGCTGCT
GTTTTTGTTGCGTTTATTTACAGTTTTACAGCATCATTCTGTGGTGTTATGCAACCATACTCTTTGTTTCCAACT
TTTTGGAAATTCATGTACAGGGTTTCGCCATACACATATTTTGTGGAAACTTTTGTTAGTATTCTATTGCATAAC
TGGGAAATCAAGTGTGACATGTCGGAGATGGTTCCTGGTCAACCTCTGACGGGGCAATCTTGTGGCCAATTTATG
GAAGCTTTCATAGAAGAATACGGTGGCTATCTACATAATAAGAACACTTTCACAGTCTGCGCTTATTGTACGTAC
ACTGTTGGCGATGACTTCCTGAAAAATGAGAATATGAGTTACGACCACGTTTGGAGGAACTTTGGTATTGAATGG
GCTTTTGTTGGTTTCAATTTCTTTGCGATGTTTGCTGGTTACTACTTAACTTACGTGGCAAGAATATGGCCAAAG
GTCTTTAAAATTATCACCAAAGTAATTCCACACAGAGGGAAGAAGCCTGTACAGAACTAA

>YGR191W 0.74 Cold

ATGCCTAGAAACCCATTGAAAAAGGAATATTGGGCAGATGTAGTTGACGGATTCAAGCCGGCTACTTCTCCAGCC
TTCGAGAATGAAAAAGAATCTACTACATTTGTTACCGAACTAACTTCCAAAACCGATTCTGCATTTCCATTAAGT
AGCAAGGATTCACCTGGCATAAACCAAACCACAAACGATATTACCTCTTCAGATCGCTTCCGTCGTAATGAAGAC
ACAGAGCAGGAAGACATCAACAACACCAACCTGAGTAAAGATCTATCCGTGAGACATCTTTTAACTCTAGCTGTC
GGGGGTGCAATAGGTACTGGTTTATATGTGAATACGGGTGCTGCTTTATCTACAGGTGGTCCGGCCAGTTTAGTT
ATTGATTGGGTTATTATCAGTACATGTCTTTTTACTGTGATTAACTCTCTTGGTGAGCTGTCCGCTGCTTTTCCC
GTTGTTGGTGGGTTCAATGTTTACAGTATGCGTTTTATTGAGCCTTCATTTGCATTCGCAGTGAACTTAAACTAT
TTAGCACAATGGCTAGTTCTTCTACCCTTGGAATTAGTGGCCGCATCTATTACTATAAAATACTGGAATGATAAA
ATTAATTCCGACGCCTGGGTTGCTATCTTTTATGCCACCATTGCACTGGCTAATATGTTGGATGTTAAGTCATTT
GGTGAGACCGAATTTGTATTGTCCATGATTAAAATCCTCTCCATCATTGGCTTTACTATCTTAGGTATTGTTTTG
TCCTGTGGTGGTGGGCCTCACGGCGGTTACATTGGTGGTAAATACTGGCATGACCCAGGCGCTTTTGTAGGGCAC
AGCTCGGGAACTCAGTTTAAAGGTTTATGTTCAGTTTTTGTTACCGCTGCCTTTTCTTATTCCGGTATTGAAATG
ACTGCTGTCTCCGCTGCTGAAAGTAAAAATCCAAGAGAAACCATTCCCAAGGCAGCAAAGAGAACTTTTTGGCTG
ATTACCGCCTCTTATGTGACTATATTGACTTTGATTGGTTGCTTGGTTCCATCCAATGACCCTAGGTTACTAAAC
GGTTCAAGTTCAGTGGACGCTGCCTCATCTCCTCTGGTTATCGCAATTGAAAACGGGGGTATTAAAGGTCTACCA
TCATTAATGAACGCCATTATTTTGATTGCTGTTGTTTCCGTGGCTAACAGTGCTGTTTATGCATGTTCAAGGTGT
ATGGTCGCCATGGCTCATATTGGTAATTTACCAAAATTTTTGAACCGTGTTGACAAAAGGGGTAGACCAATGAAT
GCTATCTTGTTAACTTTGTTTTTTGGTTTGCTTTCCTTTGTGGCAGCAAGTGATAAGCAAGCTGAAGTCTTTACA
TGGTTGAGTGCCTTATCTGGTTTATCGACAATTTTCTGCTGGATGGCCATTAATCTTTCCCATATTAGATTTCGC
CAAGCCATGAAAGTTCAAGAAAGGTCTTTAGACGAATTACCCTTCATTTCTCAAACTGGCGTCAAGGGATCCTGG
TATGGTTTTATCGTTTTATTTCTGGTTCTTATAGCATCGTTTTGGACTTCTCTGTTCCCATTAGGCGGTTCAGGA
GCCAGCGCAGAATCATTCTTTGAAGGATACTTATCCTTTCCAATTTTGATTGTCTGTTACGTTGGACATAAACTG
TATACTAGAAATTGGACTTTGATGGTGAAACTAGAAGATATGGATCTTGATACCGGCAGAAAACAAGTAGATTTG
ACTCTTCGTAGGGAAGAAATGAGGATTGAGCGAGAAACATTAGCAAAAAGATCCTTCGTAACAAGATTTTTACAT
TTCTGGTGTTGA

>YIR028W 0.68 Cold
ATGGCTAACGACGCTCTAAGTGCTATTTTCAGTAATCCTTCGAGGAAAGGTGTCCAACCCTCCACATCTATTGTG
TCATATACAAACAATGAAGATGATATTATAGATGTGGAAAATGGGAAGTTCAACAAGAACAAGAATATCAACACT
AATGTGTATGTGGACAACTCCTCAATAGAGGAGAGCGAAGTCGTGCCCTTGCCCGAAACAAAGTCCATCTGGAGT
AAAATATACTACGATTTCATTGTGTTAGACAAGACAACTTTGAATGTTTCGTTGAAAGAGTCGTTCTTGTATAAC
AGAGACTTGAAACCGGTTGAAGAAGAAAGAAGGTGTTGGTCCTGGTTCAATTACTTATATTTCTGGCTGGCAGAC
TGTTTCAATATTAACACATGGCAAATAGCTGGTACAGGTCTACAACTAGGTCTGAATTGGTGGCAATGTTGGCTT
ACAGTTTGGATCGGCTACACTTTTGCAGGTATCTTCGTAGTATTGAACTCGAGATTTGGTTCCGCATATCACTTA
TCTTTCCCTATTACTGTTAGGGCCTCATTTGGTATATTCTTTTCTATGTGGCCGATTATAAATCGTGTCGTGATG
GCTATAGTATGGTATGCCGTGCAAGCCTGGTTAGGTGCAACGCCCGTGGCACTGATGCTAAAATCTATTTTTGGC
AAGAATCTGGAAGATAGAATCCCAAACCATTTTGGTTCTCCAAATAGCACTACTTTTGAATTCATGTGTTTCTTT
ATATTTTGGGTGGTCAGTATACCATTTGTCCTAGTGGCTCCTCATAAAATCAGGCATTTATTCACAGTAAAAGCA
GCTTTGATCCCCTTCGCAGCCTTTGGATTTTTAATCTGGGCTTTGAAGAAATCGCACGGTAAAATTGAGTTGGGG
ACGCTGAATGATTATTCACCTCATGGTTCCGAATTTTCATGGATATTCGTTAGATCCCTAATGGCCTGTGTTGCT
AACTTTGCCGCTTTGATTATCAACGCCCCTGACTTCGGTAGATTTGCCAAAAATCCTCAAGCGTCTTTGTGGCCA
CAATTGGTTGCCATCCCATTGTTCTTCGCCATAACATGTTTGATCGGTATCATTGTTACTGCGGCCGGTTATCAC
TTATATGGGGTTAACTATTGGTCACCACTGGATGTACTTGGTCAATTTTTGGAGACCACTTACACCAGAGGTACT
AGGGCTGGTGTGTTCTTGATTTCTTTTGTATTTGCCTTAGCTCAACTGGGTACAAACATTTCTGCCAACTCTCTG
GCATGTGGTGCTGATATGACGGCTTTGTTTCCAAGATATATTAATATTAGAAGAGGTTCTTTATTCTGTGTGGCA
ATGGCTCTATGTATCTGTCCATGGAACTTAATGGCCAGTTCAAGTAAGTTCACCAGCGCTTTGGGTGCTTATGCA
ATTTTCCTTTCCAGTATTGCTGGTGTCATTTGCGCGGATTATTTCGTAGTAAGAAGAGGATATGTGAAATTAACA
CATTTATTCCTGGCACAGAAGGGTTCCTTTTACATGTTTGGAAACAAATTTGGTGCCAATTGGAGGGCCTTTGTT
GCGTATATTTGCGGTATCGCTCCAAATTTACCAGGTTTTATAGGTGATGTTGGAGCTCCAAAAATTACGGTTTCA
GAGGGTGCAATGAGGTTATACTATTTAGGTTATCCGGTAGGTTTCTTTATTAGTGCGGTGATATACCTCATATTA
TGTTACTTTTTTCCTGTCCCTGGTACTCCCGTAACCAATTTTCTGACAGAGAAAGGATGGTTCCAAAGATGGGCT
TATGTTGAGGACTTCGAGCAAGATTGGAAGAATGAGTTACGTAGGGATGACCTCTGCGATGACACAGTCAGTATC
TATGATGGCACCGAGGAAAAGATAGTTTACTAA

>YLR248W 0.82 Cold
ATGCTTAAAATAAAGGCCCTTTTCTCGAAAAAGAAACCGGATCAGGCAGATTTGTCTCAGGAATCTAAAAAACCA
TTCAAGGGTAAGACCAGGTCAAGCGGTACAAATAACAAAGATGTTTCCCAGATTACTTCTTCCCCTAAGAAAAGC
TTTCAGGACAAAAATATAGTTCAGTACCCGAGTGTTGTCGCAGATGACCATCATATGAAGTCTTTAACCGATGAA
TTAGTAACCACGATAGACTCGGACTCTTCACCGAGTGATAATATTACCACGGAAAATGTGGAAACAGTTACTTCC
GTGCCAGCTATCGATGTCCATGAAAGTAGTGAAGGTCAATTAAGTTCCGACCCCTTAATATCTGACGAATCTCTT
TCGGAACAAAGCGAGATTATCAGTGATATCCAGGATGACAGTACTGATGATGACAATATGGAAGATGAAATTCCG
GAAAAATCCTTCCTCGAACAAAAGGAATTGATAGGTTACAAGCTGATCAATAAAATCGGTGAAGGTGCTTTTTCA
AAAGTCTTTAGAGCCATACCTGCTAAAAATAGTTCTAATGAATTTTTAACTAAAAACTATAAAGCTGTTGCCATT
AAAGTTATCAAAAAGGCAGATTTATCCTCGATTAATGGTGATCATCGTAAGAAGGACAAAGGGAAGGACAGCACT
AAAACTTCTTCCAGAGATCAAGTCTTGAAGGAAGTTGCACTACATAAGACGGTTTCCGCTGGTTGTTCACAAATT
GTCGCGTTCATAGACTTCCAAGAAACAGATAGCTATTATTATATTATTCAAGAGTTACTAACCGGTGGGGAAATC
TTCGGCGAAATTGTTAGGTTGACCTATTTCAGTGAAGATTTATCAAGGCATGTAATCAAACAATTAGCACTGGCT

GTTAAACATATGCATTCACTAGGTGTAGTGCATCGTGATATAAAACCTGAGAATCTTCTTTTTGAACCGATTGAA
TTCACACGCTCTATAAAACCAAAATTGAGGAAATCGGATGATCCGCAAACAAAGGCAGACGAGGGAATTTTCACA
CCAGGAGTTGGTGGTGGTGGAATTGGTATAGTAAAACTAGCTGATTTTGGTTTGTCTAAACAAATATTTTCCAAG
AACACCAAGACTCCTTGTGGTACAGTCGGTTACACTGCCCCTGAAGTTGTCAAAGATGAGCATTATTCTATGAAA
GTGGATATGTGGGGGATTGGTTGCGTTTTGTACACAATGTTATGTGGGTTCCCGCCATTCTATGATGAGAAAATT
GACACTTTAACTGAAAAAATATCAAGGGGTGAGTATACCTTTCTGAAACCTTGGTGGGATGAAATCAGCGCCGGT
GCCAAGAATGCCGTGGCTAAGCTATTAGAACTAGAGCCGTCTAAAAGATACGACATTGACCAGTTTTTGGACGAC
CCATGGTTAAATACATTCGATTGTTTACCAAAGGAGGGCGAATCTTCACAAAAGAAAGCAGGTACTTCCGAAAGA
CGCCATCCGCATAAGAAACAATTCCAACTATTTCAAAGAGACTCCTCGCTACTGTTTTCACCAGCTGCTGTTGCT
ATGCGTGACGCCTTTGATATTGGTAATGCTGTGAAACGTACCGAAGAAGACCGTATGGGAACACGTGGAGGATTA
GGCTCGCTTGCTGAGGACGAAGAATTGGAAGATAGTTACAGTGGCGCCCAAGGCGATGAACAGCTGGAACAAAAT
ATGTTCCAATTAACGCTGGATACGTCCACGATTCTGCAAAGAAGAAAAAAAGTTCAAGAAAATGACGTAGGGCCT
ACAATTCCAATAAGCGCCACTATCAGGGAATAG

>YOL152W 0.81   Cold
ATGATTGAAGAAAGAGATTTGGTTTTAAGCAATGGTATCCATTGTATTGCTGACATCCACTCCGAACTATACGCC
AGGTTAAAAAAAGAATCGCAGGCAGCGACACCATGGGTGTACCAAAAACAATACGGAAAATTCGTCACTTACTTT
GTCGCTGTGATAATTTTTTTGTCTTTGATAAAAAAGCTGGCATTTATGTATTATGATTCCAGTGAGGAATTTCTT
CCAGAAAAGAAGAACTCGCCGACTACCCCTTCTGTATTCCTTGCTCGAATAATGACGAAACTTGTCGCATTCAAC
AGATACATTTGCTACAGGAAATTTCCCACGTTGATATTTTCTTATTTAGGTATTCCGACATCTGTGGGTACTTTT
TTAGTAGTAATGGCTACCACTTTATACACACTTCTATACTGCTTTGTTCCTCATCCATTCTACAGACCTTGTGCA
GGATTTGGTTCGCCGCCTTTGTCTGTTCGTGCAGGCATAATGGCAATATCTTTGGTTCCGTTTGTATTCTCACTT
TCCGGGAAGATCAACGTTATAGGTTGGTTGGTTGGGCTTTCGTATGAAAAAATCAACATATACCACCAATGGGCA
TCCATTCTTTGTTTATTCTTTAGCTGGGTTCATGTCATTCCCTTCCTACGTCAAGCACGACATGAGGGAGGATAT
GAAAGAATGCATCAACGGTGGAAGGCATCCGACATGTGGAGGAGTGGTGTCCCACCCATCTTATTTTTGAATCTG
CTATGGTTATCTTCGCTGCCTATTGCTAGAAGACATTTTTATGAGATTTTTTTGCAACTTCATTGGATTTTAGCT
GTTGGATTTTACATTAGTTTGTTCTATCATGTATATCCCGAATTGAATTCCCATATGTATCTGGTTGCTACAATT
GTGGTTTGGTTTGCACAACTGTTTTACAGACTAGCTGTGAAGGGTTATTTAAGACCTGGTAGAAGTTTCATGGCC
TCGACCATTGCAAATGTCAGCATAGTCGGCGAAGGATGCGTAGAATTGATCGTCAAAGATGTGGAAATGGCCTAT
TCCCCAGGTCAACACATATTCGTGAGAACTATTGATAAGGGCATCATTTCCAACCATCCATTTTCTATCTTTCCG
AGTGCAAAGTATCCCGGAGGAATAAAAATGCTGATTAGAGCCCAGAAAGGGTTTTCTAAAAGGCTATACGAAAGT
AATGACGACATGAAGAAAATTCTTATTGATGGGCCTTATGGTGGAATCGAGAGAGATATTAGAAGTTTTACCAAT
GTCTACTTGATTTGCTCTGGTTCAGGTATATCTACATGCTTACCTTTCCTGCAAAAATATGGCCCCATACTCCAT
AAGACAAACTTAGAAGTTATTACATTGGACTGGGTGGTAAGACATAGGGAGGATATATCATGGATTAGAGATGAA
ATGTGTACCCTCTCAAATAATTTGCGCCAGTTATTTTTAGATGGGAAAATTGTGGTTAGAATTTACGTCTGCTCG
GACAGTACCGTCCCTGGTATTATTAAAACTTTCCCTCAAACAATAGACACCGCCAGTGACCAATCTGATTTAGCT
AAAAGAGAAAAAGATACCGAATTCGGCCAGGATGATACTGAGTCAAATTCAACTTTCGACAAATCCAATAACGAA
TATAAAGGTCTCATCACCATTATTCCTTCCAAACCTGATTTGAATCAGGTCATTAATGATTACCAAATTGGGTTC
AGGAACTGCTTCATTTGTTCAGGTTCTGACAGCCTAAGGTATACCGTCGGAAATTCCGTGGCAGGTTTACAGGCC
AAGGTTTTTTCTAACAAAAATGTCGAAGAGTGCTATTTACACAGCGAGAGTTTTGGCTACTAG

>YCR098C 0.73   Cold
ATGGAAGACAAAGATATCACATCGGTAAATGAGAAGGAAGTGAACGAGAACACTAATCCTAGAATAATAAAATAT
GATGCCGAGAGGCGTGCAACCCGTACTGAAACCTCAAAGAAAGATAAATGGAAAAACATAGTTACAATCATTGCG
TCCGGTTTTGCTCTGATAAGTGATGGTTACGTAAATGGTTCAATGAGTATGCTAAACAAGGTTTTTGTTATGGAG
TACGGTAAGAAAAACTATAGCTCAAAAGTGTCGACTAGAGTTTCCAACGCAGCCCTAGTTGGTATTATTTTTGGC
CAATTCTTTATGGGTATCGCTGCTGATTATTATAGTAGAAAATCTTGTATCCTTGTGGCCACTGCTATCTTGGTT
ATTGGTAGTGCTCTGTGTGCTGCCTCTCACGGTACTACTGTACCTGGCATGTTTTGGATGTTAACAGTTATGAGA
GGTTTGGTAGGTATTGGTGTTGGTGCAGAATATCCTACCAGTACATTAAGTGCTAATGAGTCTGCTAATGAATAT
ACCACTACCAAAAGAGGTGGTATCCTGGTTATGGTGACAAATTTGCCACTAGCCTTCGGTGGTCCATTTGCTACG
ATCATCTTTTTAATCGTCTACAAAATCTGTTCAGGAACAAAACATTTAGAGGCGATCTGGAGGACTGTTTTTGCA
ATAGGGTGCTTCTGGCCATTGAGTGTGTTCTATTTTAGATGGAAGACTGCTACTACAGAAGTCTATGAAAAAGGT
AGAATCAAGAGAAATATACCATATTTCCTAGCATTGAAATTTTATTGGAAAAGGTTACTTGGTACATGTGGTACA
TGGTTTATGTATGATTTTGTTACCTTCCCAAATGGTATTTTCAGTTCAACAATTATCAGTTCCGTTATCAAGGAC
CAAAATGATTTAGTAAAAGTGGCAGAGTGGAACTTACTGTTGGGAGTTTTAGCTGTACTGGGTGTACCAATTGGT
GCTTATCTGTCCGATCGTATTGGTCGTAAATATACGTTGATGTTTGGTTTCTCTGGGTACATCATCTTTGGTCTA
ATCATTGGATGTGCGTACGACCAATTGAAAAAAATCACCCCCTTGTTTATTATCTTCTACGCATTCATGAATATG
TTAGGTAATGCTGGACCAGGTGATATGCTTGGTGTTATTAGTAGTGAAGCGTCAGCAACCGCTGTTAGAGGTGTT
TTCTATGGTTTATCTGCTGTGACTGGTAAAATCGGTTCTGTAGTAGGCGTCGAATGTTTCCAACCCATTAGGGAT
AATTTGGGTGCAAGATGGACTTTTATTATTGCTGCAATTTGTGGTCTTATTGGTATCATTATTACATATTTCTTT
GTTCCACATTCTCTTGAAAGCGATTTAATGAAGCAAGACGTTGAATTTCACAACTATTTGGTATCCAATGGCTGG
ACTGGTAAGATGGGATTTGATGAGACAGATGAAGAATCAATGGTTAGAACTATTGAAGTTGAAGAGAATGGTACT
AATTGTAGTAAGAAAAACGCAGAAATAATTTCAGTCAGACAGGTCGATCAAAGTTGA

>YDR161W 0.81   Cold
ATGAGTGAACTTGAAGCCACAATTAGGCAAGCCAAAGAAGCCCTGGCTGAAAATAATGCCAAAAAGGCGCTCAAA
ATATTGAAGCCGTTCAAAAGTTCACTGAAAAAAGAAAACGCAAACAATGTAATACTGAATGAAGTGTTTGCCGAT

GCATACCTGGATAATGGACAGGTAGAGAAAGCCTACCCAATTTTAGCGCGTGCTTGCGAATTAGATCCTGAGGGA
CAAGTTGGCGGACCGGACAAATTTTTTACCATGGGTCAAATCATGGGTGGACAGGATGGTGTATCGATAATAACT
CGCGGAATAATGAACATCTCTAATACTGGAGGTGAAATGTTAACCAACGTTCAGGTTGAAAAGATTGTAGGTGGA
CTGCTTTCTGTGATTGAAATTTGGATGACGGATTTATGTATGGAGCCAAATGCAGAAGAGCAGTGTGAGGAGTTA
ATACAAAAGGCGATGGAACTCACAGAGGGAAAATCTCCTGAAACATGGTCTACCCTGGGTTCTATCAAAATCTCG
CAACAAAAATTTGGAGAAGCTTATGAAGCATTCTCACAAGCCTGGAATTTCTTTGAGTTGAAAAAACAAGAAATT
GGTAGCGGTATAAATGAAAATGGTGACACAACTCAGAAAGCTGGTTTACAGTCAGAGTATGTAGATCTTTTACAA
CCTCTTTTATCTTTAACAAAGATGTGCCTCGAAGTGGGAGCTTACGAAGTCGCATTGAAAGTTATTGCCGCAGTA
AGAGATATAGATGAGGATAACATAGAGGGTTATTATCTAGAGGGTTTTACTTATTATTTAATGAGTAAATTAGAA
ATTTTCAAGTTGAACAATCCCGAAGTAAGCCTGCGTCCGGAAAATATATACGAGTTTAACCAACTCATTCAAGAA
GTTCCTTTGGATTTATCCCACGAACCAATCTCCCAGTTAATCTATGACTCAAGATTAGCGCTAAGTTTTGCATTG
CAAGCAGGTGTCAATGCTGACAGCAAAGATGAAATTGTGCAAGAATTATTGGGCGGTGCTAATGCTTTACTCCAA
GAAATTGGTGGTCCTCTAGATCCAAGTGAGTTAACACAAATCAAGAAGGGTGATTTAGTGAATGAGAATGAAGAC
TTGGAAGAGTTGGACATAGAGGAAGAATATTCTGACTAA

>YLR085C 0.75   Cold
ATGGAAACACCACCCATTGTGATTGATAATGGCTCATACGAAATCAAGTTTGGTCCTTCCACGAATAAGAAACCG
TTCCGAGCTTTAAATGCATTGGCCAAAGATAAATTTGGGACATCGTATTTATCAAATCATATCAAAAACATCAAA
GATATTTCATCTATCACCTTCAGGAGGCCACATGAACTAGGACAGCTCACATTATGGGAATTAGAGAGTTGTATA
TGGGATTATTGCCTTTTCAATCCTTCAGAGTTTGATGGGTTTGATCTGAAAGAGGGAAAGGGTCATCATTTGGTT
GCTAGCGAGAGCTGTATGACTTTACCAGAATTAAGTAAGCATGCCGACCAGGTGATATTTGAAGAATATGAATTC
GACAGTCTTTTCAAGTCTCCTGTAGCAGTCTTTGTACCATTTACCAAGTCATATAAGGGTGAAATGAGAACAATT
TCAGGTAAGGACGAAGATATCGATATTGTCCGTGGCAACTCAGACAGTACAAATTCCACATCAAGCGAGTCCAAG
AATGCGCAGGATTCAGGTAGCGATTATCATGATTTCCAATTAGTTATTGATTCCGGGTTTAATTGTACTTGGATA
ATTCCTGTCCTGAAGGGAATACCGTACTATAAAGCGGTAAAAAAATTGGACATTGGAGGCCGTTTCCTAACTGGG
CTACTAAAGGAAACTCTATCATTCAGACACTACAATATGATGGATGAAACCATACTTGTTAACAATATCAAGGAA
CAATGCTTGTTCGTTAGCCCGGTGTCTTATTTTGATAGTTTCAAAACGAAGGATAAGCATGCACTAGAATATGTA
CTTCCTGACTTCCAAACAAGCTTTCTTGGTTACGTAAGAAACCCCAGAAAAGAAAATGTACCGTTACCTGAAGAT
GCGCAGATCATAACACTGACAGATGAGCTTTTCACAATACCAGAAACTTTTTTCCATCCAGAAATTTCGCAAATT
ACTAAACCAGGCATTGTGGAGGCCATCCTAGAGAGCCTTTCCATGTTGCCCGAAATAGTGCGACCTCTTATGGTA
GGAAACATTGTATGTACAGGAGGAAACTTTAATCTGCCCAATTTCGCCCAACGGCTTGCGGCAGAACTACAAAGG
CAATTACCCACAGATTGGACTTGTCATGTTTCGGTGCCCGAAGGTGACTGTGCTCTGTTTGGGTGGGAAGTGATG
TCACAGTTTGCAAAGACAGATTCCTACCGAAAAGCGAGGGTCACAAGAGAAGAATACTATGAGCATGGTCCCGAT
TGGTGTACGAAGCACAGGTTTGGTTACCAGAATTGGATATAA

>YDR365C 0.75   Cold
ATGGCTGGAGAGAACCCAAAGAAGGAAGGTGTAGATGCTAGGTTTGCCGGGATCTACAGCGATCCTAAATTTAAG
AACACGAAAACCAAAGATCATAAGATCAAGTTAGATTCTAGGTTCAGTAAGAAGGATCTGGAGGTCCAGCATAAA
TCTAAGGTTGACAAGTATGGTAGAAAGATCAAAAATGCGCAGAATAACAGGGAATTGGAAGATTTCGACAAATAT
TTCGAAAAGGAAGCAGAAAATGATGAAGATTCAGAAGTTAATGCGAAAACAGTAGTGGACCGTGCCCGTGGTGAA
GTTCCTGATGATTATGTTAGCTCTTCTGATGAATTCACTTCTTCTGATTCTGAATCGTCCGGAGAAAGTGAAGTT
GAAAGCGAAGAAGAAAATGAAGTAGAAATTGAAAATGCCAAACCAGAATCTGGTGACATCTCTAAGAATTTGGCC
GTCGTAAATTTAGATTGGGACCATGTTAAATCTGAGGATTTGATGATTACCTTTTCCAGTTTCGTTCCTAAAGGT
GGTAAAATTGAAAGAGTTGCTATTTATCCAAGTGAATTTGGTAAAGAAAGAATGCAACGCGAGGAAGTTGAAGGA
CCTCCAAAGGAACTTTTCCAAAAGAAAAATAAGAACAAAACCTCCAAAAAGAAGAAAACTGATGACTCTGATTCT
GATATGGACATCGGTATTAAGGATCTTTATGAAGAGGGAGATGCTGACAAGGACGTGGATTCCAGAGCTTTACGT
CAATACCAGTTGGACAGATTAAGATATTATTACGCAATAGTTTATTGTAGTGATACAACCACCTCCAAAGCTATC
TATGATAACTGTGATGGTACCGAATATGAGTCAACCGCTAATATGTTCGATTTAAGGTATGTTCCTGATGGTATG
ACTTTTGATGATGATGTAAGAGATGAATGCAGTATATTGCCTAAGAACTATAGGCCACACCAGTTTAGCACAGAT
GCTCTACAACATTCAAGCGTCAAATTGACCTGGGATGAAACGCCAGCTGATAGGGTCGAAGTTGCTAAGAGGGCC
TTTACCCAAAAGGAAATTGATGATATGGACTTTAAGGCTTACTTGGCGTCAGATAGTGATGAATCGGATGGGCAG
GTCGATGAAGAGGCGAAAAATAAACTAAAGTCGTTGGTTGGTGACTTTGGGTTTAATTCCAAGAAAGAAACCCCT
AACGATGAAGATGAAGAAGTTGATATGGAAATTACATTTACTCCAGCCTTGGAAGGAGGAAACGAGAAATCGTCG
GAAGACAAAGAAGAAACGACTATAGAGAAAATAAGAAGAAAAGAGAAGGAACGTCGTAAAGCAAGAAAGCAGAAA
GTAAAGGAATTGAAACAGCAGTCGGAGAAGGATAAGAAAAGCAAGTTGAAATCTGTTAATAAAAAGCATACTAAC
GATGAAGAGGAGATCGAAAAGAATGCCAAATCAAAGGCTGAATTGGAGCTTTTAATGGATGATGATGATGATACC
GAAACTCAGGGCACAATAAATAACAAAGCACATTTCAACATGAACGAAATCTTAAGATCAGAAAAAGAAAAACAC
AAGAAAGGCCGGTATCAAAAGAAAGAGAGAATTGTTGAAGACACCTTTACACCTGATTTAGAAGATCCTAGATTT
AAAGAAGTCTTCGAAGATCACGATTTTGCTATCGACCCGACCCAGCCTGAATTCAAAGGTACACAAGCTATGAGT
AAAATCCTAAAAGAACGTAGTAAACGTGTTAAGAATAAGAAAAGAAAACTTGGCGGTAGTGAAAACAATATGACC
AATAACGCAGATGATAATGAAGATATTGGTAACTTGGTAAACAAGTTGAAAAAGAAAAGCAAATCATCCAAGAAG
GTAAAGGTTTAA

>YGR113W 0.76   Cold
ATGAGCGAAGATAAAGCTAAATTAGGGACCACAAGGTCTGCCACGGAATATCGTTTATCCATTGGTAGCGCTCCG
ACTTCAAGAAGGTCGTCTATGGGTGAATCCTCATCCCTGATGAAATTTGCTGATCAAGAGGGATTAACGAGTTCC
GTTGGCGAATATAACGAGAATACCATACAACAGCTTCTGTTGCCTAAAATAAGAGAACTGAGCGATTCAATCATT

ACTTTAGATTCCAATTTCACGCGCTTAAATTTTATCCACGAGAGTTTAGCTGATCTTAATGAATCCCTAGGTTCG
TTGTTGTACGGTATTATGAGCAATTCATGGTGCGTAGAATTCTCACAGGCGCCTCATGATATTCAAGACGATTTG
ATCGCTATTAAACAATTGAAATCGTTGGAAGATGAGAAAAATAACTTAGTAATGGAACTCTCCAATATGGAACGC
GGGATTAAAAGAAAAAAAGACGAACAAGGTGAAAATGATTTGGCCAAGGCATCTCAGAACAAACAGTTTAACCAG
CCGCTATTTCCCTCCTCACAAGTAAGAAAATACAGGTCATATGACAACAGAGACAAGAGGAAGCCCTCCAAGATA
GGCAACAACTTACAAGTAGAGAATGAGGAGGACTATGAAGACGATACTAGTAGTGAAGCTTCTTTTGTTCTAAAT
CCAACAAATATCGGTATGTCCAAATCATCTCAAGGTCACGTGACTAAAACCACGCGTTTGAATAATAATACCAAC
AGTAAACTGAGAAGAAAGTCCATCTTACATACAATCAGAAATAGCATTGCTTCCGGCGCTGATTTACCCATCGAA
AACGACAATGTTGTTAATTTGGGAGATCTGCATCCAAATAATCGAATATCACTCGGAAGTGGTGCTGCAAGAGTG
GTCAATGGGCCCGTTACGAAGAACAGAAATTCAATGTTCTCAGGACGTGCTGAAAGGAAACCCACAGAAAGCAGA
CATTCTGTTGCAAAGAAAACTGAAAAAAAAATAAATACAAGGCCCCCCTTCAGATGA

>YAR002W 0.71 Cold
ATGCATCGTAAATCATTGAGGAGGGCTAGCGCTACTGTGCCTTCCGCTCCCTATCGAAAGCAGATTATTAGCAAT
GCACACAATAAACCAAGCCTTTTCTCTAAAATTAAAACTTTCTTTACCCAAAAAGATTCAGCCAGAGTGAGTCCA
AGGAATAATGTTGCTAATAAACAACCACGCAATGAGTCTTTTAACAGAAGAATCTCAAGTATGCCTGGAGGTTAT
TTCCATTCTGAGATATCCCCAGATTCTACTGTAAACCGTTCCGTAGTTGTTTCTGCAGTGGGTGAAGCCAGAAAC
GACATTGAGAATAAAGAAGAGGAGTATGATGAAACACATGAAACTAACATCTCCAATGCAAAGCTTGCAAACTTT
TTTAGTAAAAAAGGTAATGAGCCTTTATCAGAAATTGAAATAGAGGGTGTGATGTCATTGTTACAAAAATCAAGC
AAATCCATGATAACTTCGGAAGGAGAACAAAAATCAGCCGAAGGTAATAATATCGACCAGTCGCTTATCTTGAAG
GAGTCAGGAAGTACACCAATCAGCATATCTAATGCGCCGACCTTCAACCCAAAATATGATACTTCAAATGCGTCA
ATGAATACGACTTTGGGAAGCATTGGTTCAAGAAAATACAGTTTCAATTATTCTAGCCTGCCCTCACCATACAAA
ACAACCGTTTATAGATATAGTGCAGCGAAAAAGATCCCCGATACATACACAGCCAACACATCTGCTCAAAGTATA
GCATCTGCTAAATCGGTAAGAAGTGGTGTTTCAAAGTCAGCTCCTAGTAAGAAAATAAGTAATACAGCTGCGGCA
TTGGTCTCACTATTAGATGAAAATGACAGTAAGAAGAATAATGCAGCTTCAGAACTTGCTAATCCATACTCCTCA
TATGTAAGCCAAATACGCAAACATAAGAGAGTTTCTCCAAATGCTGCACCAAGGCAAGAGATCAGTGAAGAAGAA
ACTACTGTTAAGCCATTATTTCAAAACGTTCCTGAACAAGGCGAAGAACCAATGAAACAACTGAACGCCACCAAA
ATTTCACCATCTGCGCCAAGCAAAGATTCTTTTACTAAATACAAACCTGCAAGGTCCTCATCCTTACGCTCAAAT
GTCGTCGTAGCTGAAACCTCACCTGAAAAGAAGGATGGTGGAGATAAACCTCCATCCTCTGCTTTTAACTTCTCG
TTTAATACTTCAAGAAACGTTGAACCTACTGAGAATGCTTATAAGAGCGAGAACGCACCATCTGCATCATCAAAG
GAATTCAATTTTACCAACCTACAGGCGAAGCCGTTAGTTGGAAAGCCAAAAACCGAACTTACAAAGGGCGATTCT
ACTCCCGTCCAACCAGATCTTTCGGTTACTCCTCAAAAAAGTTCATCGAAAGGCTTTGTTTTTAATAGTGTTCAA
AAGAAATCACGGTCCAATCTTTCACAAGAAAACGATAATGAAGGTAAACATATCAGCGCCTCAATTGATAACGAC
TTTTCAGAGGAAAAGGCGGAAGAGTTTGATTTCAATGTTCCCGTGGTGTCTAAGCAGCTAGGAAATGGCTTGGTT
GATGAAAATAAAGTTGAGGCTTTCAAGTCCCTATATACCTTTTGA

>YJR108W 0.76 Cold
ATGTCTTGGAGGTACTCTATCTTGACCGTAGATGGAAGCTTTAAAATTTTCATCCCTTGGGAAATATTCCTCACG
TGGAATTTTTTGAGCGCTGCATGGCTAAATTCTACCGAATCCAATACGTATATCCACTACTCAACATGTTGGGGC
ACAAGTGATTACACACTTAATATCTCTGTCATAGAAGCTACTACAGAGAAATTGGTTGACACTAGATTATTAACA
ACACTCGAGAATGCAACCGCTTGGATTAACTCAAACTCTATTGATGAAGACGAAGATGATATGCCTCATGCCACT
AACGTAGCAGACCGGCTTGATGGGTTATCCCTTAGCAAGCGCGTATATAGCATCTGTCACTACGAATTTTAG

>YOR115C 0.77 Cold
ATGTCCTCTACACATAGTAATAATGTAGGACATCCCCAATCTTCTCCACAAGGTCCACTAACGGAACAACAAAGA
GCACAACAGCAATATCAAATTTTTGAGAATTCATTACCAAAAGTAAGTCAATCTGTGTATCAGATGCTGTTGAAC
GAGATGGTTCCTCTAGCTATGGGCATTGAGAGGCAAATATCAGGCGATGTTATCAGTTCTGATAGCAATGTAACT
TCCGAAAATGGTAACATCAACAATATGATAAAGAGACTGAAGATAGAGGAACATCATACAGTAGATATCATACGT
TCTCATAACTTGATACATGAGCTGTATAAGGCGGATGAAGAGGAAAAAGAAAAGGTTTTAGCAAGACTGAGGAAT
ATTGGGTTTCAAATCGGATTGAAGCTGTCAGAGTTATTGATATTTTCTAACAACCCAAATTTAAAATTCAAGGAA
ATGGACCTTCTATTAATAATGAAGTTCATTTGTAGGGATGTCTGGAAACAAATATTTGGTAAACAGATCGATAAT
TTAAAAACAAACCACAGAGGAACCTTTTACCTTCTTGATTATGACTATCGACCTATTCAATCATTTTCTTTGGAG
GAAGATGCAAAAAAACGAGGAGTTAAAAATGATAGAACCGTTTTTGGAGATACCTGTGGGAATTATTAGAGGCGTG
TTGTCTTCCTTAGGTTATTCATCTGAAGAGGTCATTTGCTTGGCGTCTTTTATCGACAGGCCAACAGATAGGCCT
AAAACAGCTTTTCCCAAGGGAGTTAGCTTCCATGTTCAAGTCACAATGCCGCAGTAA

>YBR295W 0.82 Cold
ATGAAGCCGGAAAAACTCTTCTCCGGTTTAGGTACCAGCGATGGCGAGTATGGAGTGGTAAATAGCGAGAACATA
TCAATAGATGCTATGCAAGATAATAGAGGCGAGTGTCATCGTAGATCCATAGAAATGCATGCTAATGACAATCTG
GGATTGGTCTCCCAGCGAGACTGTACCAATCGGCCCAAAATTACTCCTCAGGAATGTTTGAGTGAGACTGAGCAG
ATATGCCATCATGGCGAGAATAGGACTAAGGCTGGGTTAGATGTAGACGATGCTGAAACCGGTGGTGATCACACC
AATGAATCCCGTGTAGATGAATGTTGTGCTGAGAAAGTGAACGACACTGAGACTGGCTTGGATGTGGACAGCTGT
TGCGGCGATGCTCAAACAGGTGGTGACCACACCAATGAATCCTGTGTTGATGGATGCTGCGTTAGAGATTCTTCA
GTGATGGTCGAGGAAGTTACAGGCAGCTGCGAAGCTGTATCTTCTAAAGAACAATTGCTGACCAGCTTTGAAGTT
GTTCCAAGCAAATCTGAAGGTCTGCAATCTATTCATGATATTAGAGAGACAACTCGCTGCAATACCAATTCCAAC
CAACACACAGGGAAGGGTAGGCTTTGCATCGAATCAAGTGACTCTACACTCAAGAAAAGAAGTTGTAAAGTTAGC
AGACAGAAAATTGAGGTTTCCAGTAAGCCTGAGTGCTGCAACATTTCATGTGTTGAGCGGATTGCATCTCGTAGC

TGTGAAAAGAGGACTTTTAAAGGTAGCACCAATGTTGGAATTTCTGGGAGTAGCTCTACCGACAGTTTAAGCGAG
AAGTTCTTCAGCGAACAGTACTCTAGAATGTACAATCGTTACTCCTCAATTTTGAAAAATTTAGGTTGTATCTGT
AACTATTTGCGTACTTTGGGAAAAGAATCTTGTTGTCTCCCAAAGGTGCGTTTTTGTAGCGGGGAGGGTGCTTCT
AAAAAGACAAAATACTCCTACCGCAACAGTTCTGGATGTTTAACAAAGAAAAAGACCCATGGAGACAAAGAAAGG
TTGAGCAATGACAATGGCCATGCTGATTTTGTTTGTTCTAAAAGTTGTTGCACTAAAATGAAGGATTGTGCTGTT
ACCTCAACTATTTCTGGAACCTCTTCGAGTGAAATTTCAAGAATCGTATCAATGGAACCAATTGAAAATCATCTT
AATCTTGAGGCAGGATCTACCGGTACCGAGCACATTGTTCTTAGTGTTTCAGGAATGTCCTGTACTGGTTGTGAA
TCAAAACTTAAGAAATCATTTGGCGCGCTCAAATGTGTTCACGGTTTGAAGACCAGTTTAATATTATCGCAAGCT
GAATTTAATCTGGATCTTGCTCAGGGATCTGTCAAGGATGTTATCAAGCACTTGAGCAAAACTACTGAATTCAAG
TACGAACAGATTTCAAATCATGGTTCAACTATAGATGTTGTTGTTCCTTATGCAGCAAAAGATTTTATTAATGAG
GAATGGCCACAAGGTGTCACAGAGCTGAAAATTGTTGAGAGAAATATCATTCGTATTTACTTTGACCCAAAGGTT
ATAGGTGCCAGAGATCTTGTCAATGAAGGATGGAGCGTGCCTGTTAGTATTGCTCCATTCAGTTGTCATCCAACC
ATTGAAGTGGGAAGGAAGCATTTAGTTCGTGTAGGCTGCACGACCGCTTTATCCATAATATTGACGATCCCGATT
CTCGTGATGGCTTGGGCTCCACAGCTTCGTGAAAAAATTTCCACTATCTCCGCGTCAATGGTGCTAGCAACTATT
ATTCAATTTGTTATTGCAGGACCGTTTTACTTAAATGCCTTGAAGAGTTTGATATTTTCCAGGCTCATTGAAATG
GATCTACTAATAGTTTTAAGCACTAGCGCTGCCTACATATTTTCGATTGTATCATTTGGATATTTCGTTGTTGGA
CGCCCGTTATCTACGGAGCAGTTTTTTGAAACCAGCTCTTTGCTTGTAACTCTCATCATGGTTGGTCGCTTTGTG
AGTGAACTAGCTAGACATAGGGCTGTAAAGTCAATATCAGTACGTTCCTTACAGGCCTCTTCTGCTATTCTGGTG
GATAAAACTGGTAAAGAGACGGAAATTAATATTAGACTTCTCCAATATGGCGATATTTTTAAGGTTTTACCTGAT
TCAAGAATCCCAACTGATGGAACAGTCATTTCTGGGTCTTCTGAAGTCGACGAAGCGTTAATCACAGGTGAATCT
ATGCCAGTCCCGAAAAAGTGTCAATCAATTGTTGTTGCAGGATCGGTGAATGGCACTGGTACTTTGTTTGTGAAG
CTGAGCAAGCTTCCAGGGAACAATACTATTAGTACCATAGCCACGATGGTTGATGAAGCAAAATTAACTAAGCCA
AAGATCCAAAATATCGCTGATAAAATTGCAAGTTATTTCGTGCCAACTATCATTGGAATCACTGTCGTCACCTTT
TGCGTTTGGATAGCGGTTGGAATCCGTGTGGAAAAGCAATCCCGCTCCGATGCCGTAATTCAGGCCATTATATAT
GCCATTACGGTACTTATCGTTTCGTGTCCCTGCGTGATTGGACTTGCCGTTCCTATCGTATTTGTTATTGCAAGT
GGCGTCGCTGCGAAAAGAGGGGTAATCTTCAAATCAGCAGAGAGTATAGAAGTTGCTCACAACACTTCGCATGTT
GTCTTCGATAAAACGGGTACCCTAACTGAAGGAAAACTTACTGTAGTACATGAAACTGTTAGGGGTGATCGTCAT
AATTCTCAATCTTTGTTGCTTGGATTAACTGAAGGAATAAAACACCCAGTTTCCATGGCAATAGCATCCTATCTC
AAAGAAAAAGGTGTTTCTGCTCAAAATGTTTCTAATACAAAAGCTGTGACTGGTAAGCGGGTAGAAGGAACATCA
TACTCTGGTTTGAAGTTACAAGGAGGGAACTGTCGTTGGCTTGGTCATAACAATGATCCTGACGTTCGAAAAGCC
CTTGAACAAGGATATTCTGTATTCTGTTTTAGTGTTAATGGTTCAGTCACTGCAGTTTATGCATTAGAGGACTCT
TTACGGGCAGATGCTGTCTCCACTATTAACTTGTTACGCCAAAGAGGGATTTCACTACACATTTTATCAGGGGAT
GACGATGGAGCCGTTCGTTCTATGGCAGCCCGTCTTGGAATTGAAAGCTCCAATATTCGTTCTCACGCAACGCCT
GCAGAAAAGAGTGAATATATTAAGGATATTGTCGAAGGAAGAAATTGCGATAGTTCTTCGCAGTCAAAAAGACCG
GTTGTTGTTTTTTGCGGTGACGGAACAAACGACGCAATTGGTTTGACTCAAGCAACGATTGGAGTTCATATCAAT
GAGGGAAGTGAGGTTGCCAAGCTAGCTGCTGATGTAGTTATGTTAAAGCCAAAGCTCAACAATATTTTGACTATG
ATAACTGTAAGTCAAAAGGCCATGTTTAGGGTCAAATTGAATTTCTTATGGAGTTTTACTTACAACTTATTTGCT
ATCCTTTTGGCGGCTGGTGCCTTTGTTGACTTTCATATTCCACCAGAGTATGCGGGTTTAGGGGAACTAGTTAGT
ATCCTCCCTGTAATTTTTGTAGCTATACTTCTGCGTTATGCAAAGATTTAG

>YMR226C 0.79   Cold
ATGTCCCAAGGTAGAAAAGCTGCAGAAAGATTGGCTAAGAAGACTGTCCTCATTACAGGTGCATCTGCTGGTATT
GGTAAGGCGACCGCATTAGAGTACTTGGAGGCATCCAATGGTGATATGAAACTGATCTTGGCTGCTAGAAGATTA
GAAAAGCTCGAGGAATTGAAGAAGACCATTGATCAAGAGTTTCCAAACGCAAAAGTTCATGTGGCCCAGCTGGAT
ATCACTCAAGCAGAAAAAATCAAGCCCTTCATTGAAAACTTGCCACAAGAGTTCAAGGATATTGACATTCTGGTG
AACAATGCCGGAAAGGCTCTTGGCAGTGACCGTGTGGGCCAGATCGCAACGGAGGATATCCAGGACGTGTTTGAC
ACCAACGTCACGGCTTTAATCAATATCACACAAGCTGTACTGCCCATATTCCAAGCCAAGAATTCAGGAGATATT
GTAAATTTGGGTTCAATCGCTGGCAGAGACGCATACCCAACAGGTTCTATCTATTGTGCCTCTAAGTTTGCCGTG
GGGGCGTTCACTGATAGTTTGAGAAAGGAGCTCATCAACACTAAAATTAGAGTCATTCTAATTGCACCAGGGCTA
GTCGAGACTGAATTTTCACTAGTTAGATACAGAGGTAACGAGGAACAAGCCAAGAATGTTTACAAGGATACTACC
CCATTGATGGCTGATGACGTGGCTGATCTGATCGTCTATGCAACTTCCAGAAAACAAAATACTGTAATTGCAGAC
ACTTTAATCTTTCCAACAAACCAAGCGTCACCTCATCATATCTTCCGTGGATAA

>YJL221C 0.79   Cold
ATGACTATTTCTTCTGCACATCCAGAAACAGAACCCAAGTGGTGGAAAGAGGCCACAATTTATCAAATTTATCCC
GCAAGTTTCAAAGACTCCAACAACGATGGTTGGGGTGATATGAAGGGTATTGCCTCCAAGTTGGAGTACATCAAA
GAGCTTGGTACCGATGCCATTTGGATCTCGCCATTTTACGACTCGCCACAAGATGATATGGGTTACGATATTGCC
AACTACGAAAAGGTCTGGCCAACCTACGGTACGAACGAGGACTGCTTTGCCTTGATCGAAAAGACACATAAGCTT
GGTATGAAATTTATCACCGACTTAGTCATCAACCATTGCTCCAGCGAACATGAATGGTTCAAAGAGAGCAGATCC
TCAAAAACCAATCCAAAACGTGACTGGTTCTTCTGGAGACCTCCTAAGGGTTATGACGCCGAAGGCAAGCCAATT
CCTCCAAACAATTGGAGGTCTTACTTCGGTGGTTCTGCATGGACGTTCGATGAAAAGACACAAGAGTTTTACTTG
CGTTTGTTTTGCTCCACCCAACCTGATCTAAACTGGGAGAACGAAGACTGCAGAAAGGCAATCTACGAAAGTGCC
GTTGGATACTGGTTAGACCATGGTGTAGACGGCTTTAGAATTGATGTGGGAAGCTTGTACTCCAAGGTTGCTGGT
CTACCAGACGCTCCTGTGATTGACGAAAACTCAAAGTGGCAACTCAGTGATCCTTTCACAATGAACGGACCACGT
ATCCATGAGTTTCATCAAGAAATGAACAAGTTCATCAGAAACAGAGTGAAGGATGGCAGAGAAATTATGACAGTT
GGTGAAATGCGACATGCTACTGATGAGACCAAGAGGTTGTATACAAGTGCGTCAAGACACGAACTTAGTGAGTTA
TTCAACTTTTCCCACACTGATGTCGGGACTTCGCCCAAGTTCCGTCAAAATTTGATACCATATGAACTAAAGGAT

TGGAAGGTTGCCCTTGCTGAACTTTTCAGATACGTTAACGGAACTGATTGTTGGTCAACAATTTATCTGGAAAAT
CACGACCAACCTCGTTCAATTACCAGATTTGGTGACGATTCTCCCAAAAACCGTGTTATTTCTGGTAAGTTGTTG
TCTGTGTTGTTGGTGTCACTGAGCGGTACTCTATATGTGTACCAAGGACAGGAACTGGGTGAAATCAATTTCAAG
AACTGGCCTATCGAAAAATACGAGGATGTCGAAGTTAGAAATAACTATGATGCGATCAAGGAAGAGCATGGAGAA
AACTCGAAGGAGATGAAGAGGTTTTTGGAAGCAATTGCCCTTATTTCCAGAGACCATGCTAGAACACCTATGCAA
TGGTCTCGTGAGGAGCCAAATGCTGGTTTTTCTGGTCCTAATGCTAAACCATGGTTTTACTTGAACGAGTCTTTC
AGGGAAGGAATTAACGCTGAAGACGAATCCAAGGACCCCAACTCGGTTTTGAACTTCTGGAAGGAGGCCTTGAGA
TTTAGAAAGGCACACAAGGATATTACTGTGTATGGATATGATTTTGAGTTTATTGATTTGGACAATAAGAAACTG
TTCAGCTTCACAAAGAAATACGACAACAAAACATTGTTTGCTGCTTTGAACTTCAGCTCTGATTCGATCGACTTC
ACGATTCCAAACAATAGCTCATCGTTTAAGTTGGAATTCGGAAACTACCCAAGAAGTGAAGTTGATGCGTCTTCC
AGAACATTGAAGCCATGGGAGGGCAGAATTTACATATCTGAATGA

>YBL053W 0.8 Cold
ATGTTACATGTATATGCTTATATCCAATATATACCCATCGCCGCTTATTCTTCGTCATCCTCTATTAGAGTTATT
TTTCTTCTTTTTCTGACTCTCTCCTTCCTTATGTGGCTTTCTATATCGTTTTCATCCTGCTCTTGTCCTTTTCTC
TTCCCATCCTCATCATCATCACTGTCTTCTTCATATGTGTCTTCCTCCTCATCATTTTCTTCTGATATTTGCTCA
TCCTCAATGTCATCATCAAGGGTAAAGTCTTCGTCTTCGTCTTCATCTTCTTTGGCGTTTTCACCTACATATAAT
TCAGTATCTACATCGTTTTCTACTTCATCCTGCTCTAAACTTGATATTCCAGTATCGTTTCTCCTAGCTGCATGA

>YOR264W 0.71 Cold
ATGCCAAGAAAGTTTTTGGGCAATAAAATTGAAAAGAATGTTGATGCAGTCAGACCATCTTCGCTAACATTAACA
GCTGATGACTTGAAGTATATTCCACCGATACCACAAGATTTTGAAGATGAAGATGATAAGGTTTTACGTACCAGC
AACGGTGGTAACAGGTTGTCTAAAAGATTTGGGGGTACACTGAAACTGAAGAAACGGTTAGAGTCTGTTCCAGAA
TTGTTCTTACATGATTTCAAGAAGAGGCCACGCTCCCAACTTGAGGTGATTAGAGAGAAAAAATTTACTGACATG
CAAGTTCCCAAGGGACCAGTTTGTCCTCAAAGCACGATTCTACCATTACGAGAAAGAAAGAAGGTAAAATCACTA
CCAATTCAAAGAAAAAGTTTACGTAGGCCGACGCTGTCAAAGCCTGCAGTAGTACAATCTTTAGGGCATAAGACA
CATTCGGATCATATTATTGATAAAGTGTTTGTCTCTCGCCCGGCCCCAATTGTAATGCCTGTAAAAGCCCTTACT
CCTATTAATCCGGTCTCCCTAATGCAAACACAGACACAAGATTGCTGTAGAAAAAATAAGTATGGCAAAAGCGGT
AGTGAAATATTGTTTGACGAAATTCTATCAGCCTATGAAAACGTTTCTACAAGCGATTCTACTGCCCTAAATTCA
GAAATTGACAGAATTATCGATATATGCGCTTCAAAACAAATAGCTAAAAAAAAATGAGGCATTTCAGGTACCATAT
GTCGTCTGCCCTGACGACACAGAGACTTTGTTTTCGTCAACAACACCAAAGTTGAAACCTGTCAATTCTAACACA
TTAAACGATGTCATTTCTAGCCCAGAATACACCACGTCTGGATGCAGTACATACTCTGACCAATCGAATTCAGAT
GAGGAATTGTCCGAAGTAGAAAGTATAGTATGGAACACGAATAAGAGGACAATGAGATCATCCATTGTGTCTGAA
AGCACTAGTGAGGAAGGCTATTGCACTGCTGCGGAGACTTTGCCCTCAACAGTATCAGTGGAAGATCTGGATATA
CATAACAAGCTTCCAAAAGTTGCACAAACATCATCGTGCAATACGCTTTTGAATAAACTCTCTATAAGAAAATTA
AAAAAAGTAATTCTCGATCCGCCTAAAATAATGCATGTCATGACTTTTGACGATGACAGTGATGATGGAGATGAC
AACGATGACGAGGATAGGGCTTTGAATATTCTTCAAAAAAAAAATTGACTGCATTGAAATAGCAAGTTGCAGCAGT
AGCATTTACAGCGAATAA

>YIL008W 0.8 Cold
ATGGTAAACGTGAAAGTGGAGTTTCTAGGTGGACTTGATGCTATTTTTGGAAAACAAAGAGTACATAAAATTAAG
ATGGACAAAGAAGATCCTGTCACAGTGGGCGATTTGATTGACCACATTGTATCTACTATGATCAATAACCCTAAT
GACGTTAGTATCTTCATCGAAGATGATTCTATAAGACCCGGTATCATCACATTAATCAACGACACCGATTGGGAG
CTCGAAGGCGAAAAAGACTACATATTGGAAGACGGTGACATCATCTCTTTTACTTCAACATTACATGGTGGTTAA

>YJL001W 0.72 Cold
ATGAATGGAATTCAAGTGGACATCAATCGTTTGAAAAAGGGCGAAGTCAGTTTAGGTACCTCAATTATGGCCGTG
ACATTTAAGGATGGTGTGATACTAGGTGCTGATTCACGTACCACCACTGGTGCGTACATAGCTAACCGTGTGACA
GATAAATTAACGAGAGTACATGACAAAATTTGGTGTTGTAGGTCCGGTTCTGCAGCAGACACGCAGGCGATTGCC
GACATTGTTCAGTACCATTTGGAATTATATACTTCTCAATATGGTACCCCCTCCACAGAGACTGCTGCCTCGGTG
TTCAAAGAATTATGTTACGAAAATAAAGATAACCTTACTGCTGGTATAATTGTGGCTGGTTATGATGACAAAAAC
AAAGGGGAAGTATATACTATTCCATTGGGTGGCTCCGTCCATAAGCTGCCTTATGCGATAGCAGGATCTGGCTCT
ACTTTCATATATGGGTATTGTGATAAAAACTTTAGAGAAAATATGTCAAAGGAAGAAACCGTAGATTTCATAAAG
CATTCGCTATCGCAAGCCATTAAATGGGACGGATCTTCCGGTGGTGTTATAAGAATGGTTGTTTTGACAGCTGCT
GGTGTGGAACGTTTGATATTCTACCCTGATGAATATGAACAACTATAA

>YKL174C 0.76 Cold
ATGCCAGAGTATACGCTACTGGCTGATAATATAAGGGAGAATATCGTTCATTTCGATCCGAATGGTTTGTTTGAT
AACTTGCACACCATTGTTCATGAAGATGACAGTCAAGAGAACGAGGAGGCCGAGCATTTCAATTATGATCAGGTG
TTGGATAAATCGTTATTGTCAAGAGGTTCTATTGTCGGTCTCGGTTTAGGACTAATGAGTCCCGTTTTAGGAATG
TGCACTAGTATGGCCATTGGGCTAATTAATGGTGGTCCGTTAACTATAATGCTAGGTTTTTTAATCAGTGGAGTG
TGTATATGGTTTTCGTCGCTTTCTCTTGGTGAGATTGTTTCAAAATTTCCGATGGAACTGCATGTTGGGAGTGCC
ATGTTGGCCCCGGAGAAATTGAAATTAGTATGTTCGTGGTACACTGGCTGGTTAATGCTCATAGGGAATTGGACT
ATGAGTACCAGTATTACTTTTGCAGGCGCTCAACTTACCATTTCTTTGATTCTGATGACGAACTCCAACCTAATA
TCCGAGGCACACTTGATTTTTTACACAGTCATTGTATTTTACTTAGTTGTGACTGTTGTAGGCCTCGTGAATTTG
AAATTTGCAAGATTTATTGAAACAATAAACAAAGTCTGTGTTTATTGGATCATATATGCCATTATATTTATTGAT
ATTCTTCTACTAGTATTCCACAAAGGTAAATTTCGATCTTTGAAGTACGCGCTATTTCACTTTGATAATAATCTA

TCAGGGTATAAAAGCGCATTTCTTTCCTTCATCATTGGATTCCAACAGTCTAATTTCACGTTACAAGGTTTCAGT
ATGTTACCTGCTTTAGCTGACGAAGTCAAAGTTCCTGAGAAGGATATTCCACGTGGTATGTCGAATGCGGTATTG
TTATCCGCGTTCTCTGGAGTCATTTTTCTTATACCAATAATGTTAATCCTGCCAGATAATGATTTGCTTTTTACC
AATCATAAGGTTCTACCAATAGTGAACATTTTTACAAAATCGACTGATTCGGTGGTCTTGTCTTTTTTTTTAGTG
CTCCTAATTTTAGGAAACTTACTGTTTTCCGGAATTGGCTCGATTACTACATCTTCTCGTGCGGTATATAGTTTT
AGTCGTGACCAGGCTATACCATACTACGATAAATGGACCTACGTCGAACCGGATTCTCAGTCAAAAGTCCCCAAG
AATTCTGTTGTATTGAGTATGATAATATCATACTTTTTAGGTCTGCTAGCTTTGATTTCAACGGCCGCATTTAAT
GCTTTTATAGGCGCTGCAGTGCTCTGTCTTTGTTCTGCGACTTTCATTCCGTTAGTCTTGGTGCTGTTTACGAGA
AGAAGAGCTATCCGAAGCGCGCCAGTAAAAATCAGGTATAAGTTTGGTTGGTTCATCAACATTGTTTCTATTGTG
TGGCTCTTGTTATCTATGGTTTCTGTTTGCCTACCAACGCAAGTGCCTGTAACTTTCAAAACAATGAATTATGCT
TTAATGGTGTACGTATTCTGCATTTTAGTTATCACTGGTCTTTATTTCAAATGGGGGAAGTATAATTTTAGATTA
CCCTTGGCAGATGACATCAAGGCTCCAATTCCCAGTGATGCGGAAGAAACTGTTTTTGAACTAGAGGATAGCAAT
GTTGAACATACTCTAAACTCGGGAACCACAGTGAAAGAGTCTGTAGAAAATAATTCTGAAGAAGGTTTCATCAAG
GTGCATCCTAAAAGTAGTACAGAAAATCCCTTTGAGGAAAATGAGGAAAACGTGATAACCGATTATGGTGATGAG
CACCATACAGCAGAACAAGAATTTGATCTTGCCGATGATCGTAGATATGATATATGA

>YPL269W 0.81   Cold
ATGGATAATGATGGACCCAGATCTATGACCATTGGGGATGACTTCCAAGAGAACTTTTGTGAACGTTTAGAAAGA
ATTCACAATACATTACATTCAATAAACGATTGCAACTCATTGAATGAGAGTACCACAAGTATATCAGAGACATTG
TTAGTTCAATTTTATGATGATTTGGAGAACGTTGCCTCGGTAATTCCCGACTTAGTCAACAAGAAAAGACTGGGA
AAAGACGATATTTTGTTGTTTATGGACTGGTTATTATTGAAAAAATATATGCTATATCAATTTATTAGTGACGTT
CATAATATTGAGGAAGGTTTTGCTCATTTGTTGGATTTACTAGAGGATGAATTCTCGAAGGACGATCAGGATAGC
GACAAATACAATCGATTTAGCCCGATGTTTGACGTCATAGAAGAATCTACACAAATTAAGACTCAGTTGGAGCCA
TGGCTTACTAACTTGAAGGAATTATTGGATACGTCATTGGAATTTAACGAAATTTCAAAGGATCATATGGATACA
TTACATAAAATCATTAATAGCAATATATCGTATTGTCTGGAAATTCAGGAAGAAAGGTTCGCGTCCCCGATACGG
CATACTCCATCATTTACCCTAGAACAACTAGTCAAGTTATTAGGAACGCATACGGAGACAACTGAGCCAAAGGTA
CCAAAATTTTCTCCGGCAGAAGATATTTTATCAAGAAAGTTCTTGAACCTAAAAAAAAATATCCCTCCAATTGAA
AAAAGTTTGACTGATATCCTACCTCAAAGAATTGTGCAATTTGGGCACCGGAATATAACAAACATAACTACTTTG
CAAACAATCTTGCAAAAAAAATACGAGCTGATAATGAAAGATTATAGATTTATGAATTCAGAGTTTAGGGAATTG
AAAGTCGAACTAATCGACAAACGTTGGAATATACTCTTTATTAATTTAAATCATGAACTATTATACATTCTTGAT
GAGATAGAAAGGTTGCAGTCGAAATTACTGACAACAAAGTATACCAAAGATATCACTATAAGGCTCGAGAGGCAA
TTGGAGAGAAAATCAAAAACTGTTTCTAAGACATTCAATATTATTTACAGAGCATTGGAATTCTCACTTTTGGAC
GCTGGCGTCGCATCGAAGACAAATGAATTAGCTCAAAGATGGCTGAATATCAAGCCTACAGCGGATAAAATTCTA
ATCAAATCCTCCGCTTCAAACAAAATAGCTACTAGCAAGAAGAAGATTCCAAAACCGAAATCATTAGGCTTTGGG
CGACCGAATAGTGTTATTGGAACTATAACCCAGGATTTCCAGGAGAGAGTCGCCATTAATGAAGGTGACAGTAAT
AAAACACCGGAAAATTCAACTACAGTGGCTTTGAAAGGTAAAAAGCTTGGTAAAGCGTTACTGCAGAAGATGAAT
ATTAAACCTGCAACAAGCCCAAATTCATCAAATGCGATTAATCCATTTTTTGATCCGGAGTCGCCAAACAAAGGA
AAACTGATACTAAGTAGTGTTCCTCCCTTACCTTATGACGAAACCGATGAAACCACCCTCCGTGTTTCTCGTGGG
GAAAATGAAAAGTCACCAGACTCCTTCATTACATCTCGGCATGAAAATAAAGTACAGATTACAGAAACTCCTTTG
ATGGCAAAGAATAAGTCTGTGCTTGACATCGAAAAAGATAAATGGAATCATTACCGGTCCTTGCCCTCTAGGATC
CCTATATACAAGGATAAAGTGGTGAAAGTCACCGTAGAAAACACACCGATAGCAAAGGTTTTCCAGACTCCGCCA
ACAAAAATCACGACACCTAATAGTCAAGTTTGGGTCCCTTCGACAAGAAGAAGAACCCGATTGAGGCCTCCCACC
CCCTTATCACAGTTACTTTCACCAAGAGAAGGGCGTTTAGATAAAACCCCAACTTATTGA

>YOR017W 0.75   Cold
ATGGGGTTCTACAACTGTAGATTCCTGTCCAGACGTCTATCTGTTGAGCCTTGCAGAATAGGTAGCGCCGCTAAA
AGCTATCAACAGCGATCGATTTATCACTTTGGCGCTGCTCTCACAAATGCCCCTAGTGGAAGAGAAACACCAGAC
AAGCTACGTTCTGACCTGCATAGTGCTTTAGAGATGGTCGATGAAATATATGACACAAATTCGACTGTAGAGGAC
ATTGGAAACAAGGAAAAGGGAGGTCGACAAAAATATACGGAAGAAATGGACAAGGCGATTAATCTACTGAAAACA
AATATAAAGAAGGAGTATAGACATGATAAGTATTTGGAGCGGACAAAGGTAGGAACGTATCCTGGTAGAAGAACA
TATCCTGGTAGGAGAACATATCCCGCTAGGAGAACATATCCCGCTAGCAGAACATATTCAGATAGCAATTCATAC
ACTTTTCGGATAAATGTCCAGAAGATTAGGCACGCATTAGTACGTTACAATCAAGATGGTGTTCAAAAACACAAC
CAAAAGCCTCCTAGGATTGGTCATGGATTAACGAGAGTGTTATACCAACCGTTGTCGCTACAAAAATTGAGAGAT
AATAGGAGCAGAATGTATAATTTTGACCCTGCAGTGGAAAATATTAATCCAGAGTATTTGGAAAAGAAAAGTGAA
AAAGATGTGAACACAGACTCCTCTGGAGAAGGACAAAGCAAGCCAATATTCATCACACCCCACAAAGATGAATCT
CTGTTAAAGGTTGCCAAAGAACATAGAAAAAAGTACATATCTTCATCAAGTTCCATGACATCAGTACTTTCTCAG
TTGCACTATTTATTATCCAATTTCAGAAGATTGAATATCATTGATTCCTCGATATCGAAAAATTTTCCTCAAAAA
AACTGCAATTATTCAGAAAGTGCATATTTTCCGTCAGCAGTCATTCTGAGAAAGAAGAGAAATGGCATTTGTTCT
ATTGATTCAGATAGAAGTCTGGATAGGGAGATTGTGCTTTCTGTACTAGGTCATTATCTTGAAGACTTTTTAACG
GAAAAGTCCCTGAAGAATAGTTCAAAAAGTGAAAATTATCACTATTCTAGTATAGATGAATTCATTGTGAGATCT
CAATTGGACGCGTACGACCCAAATCTACCTGGAACGGGTGTATTTGATCTGAAGACAAGAGCAGTTTCTGCCATA
AGATACGATTTATCACATGTAGAGAGTAATAACAACCAAACTGGATATGAGATAGATAAAGTTTATGGGGAATTT
GAGTCTTTGGAAAGAGAATATTTTGAACTAATCAGGTCTGCCCTTCTAAAGTATTCTTTGCAAGCTAGAATTGGC
AAAATGGACGGTATTTTCGTTGCATACCACAATATTTCTAAGATGTTTGGATTCCAATATCTACCTTTGGACGAA
TTAGATTATATAATCCATTCTTCGTATAACAGCAAATTCGACAGTTTGTTAAAGGAGAAGAATGATATCACAAAA
GGGATATATGGTGAGGAGGACTACATTCTACACTATGACAGGGACGATAGGAAAATTGCTTGCTTGGTGGCTAAT
AGGGAATTTAAGATGTCTATGAATCTGTTCAGTAATATTTTGAAACACGTTGAGCAGTTACTTAACTCAAGTAAT

ACGAAATGGGAAAAAATGCAAAATAATGCTTAAAACCGAAGTTGAAGAAAAACGATCCAAAAGTGGCCGCTTCTTT
AACGAACCGGTGCTAAATATTGTAGCCCTGCCGTTGTCACCTGAGTACGAGGATAAATCTTTATTGGTAAAGGAT
ACTTCGAACGAACAATTAACAGAGGAGCTACTAAACCTCCGCTCATATAACGAAAATCTTTTGGAGGAACACCTG
AATTCCTTAGTTGGATTTAAAGTAAATGTCAAGCATTTCTATCACCATCATCCTAATACTACACATCTACCTGAT
TTTGCTTTAAAAAAGAACGATATTCTCGACACCGAATCCCGTAAATATATTTCTGACATGATGAAACGAGACTGG
TACAAAGATATACCATCTACACAGACTCCAAACTTTTTCCACGCTTCCGATGTTTCCACTTGGGAGGTGAACTCT
ACTTTTACAGATATCAATGACAAACAAATTTTACGAAAACTATACTTTAAATATTTGGATGTTAAATTAAACGCA
TTGAAAAATCAAGTAATCACACGTCAGGAACCTGATATGTCTAAAAAGGATGAAATTATGAATCGAATCAAATCA
CTACAAGCCCGCAATGATCACCGCGATAATGGAAGTAACAAAAGATATTCTAATTTTGGCCCTACCCGACTGCAA
ACTAAACTGCGCGCATATGCTAAAAAAGGTGCCCTTCGAAGAAAACTATTAGAAAGGAGTAACAAGTTCCATATC
TAG

>YJL050W 0.8 Cold
ATGGATTCTACTGATCTGTTCGATGTTTTCGAGGAAACACCTGTTGAGCTTCCTACAGATAGTAATGGAGAAAAA
AATGCTGATACAAACGTAGGAGATACTCCAGATCATACCCAGGATAAGAAGCATGGCCTTGAAGAAGAAAAAGAA
GAGCATGAGGAAAACAACAGTGAAAATAAAAAAATCAAATCCAATAAGAGCAAAACGGAAGATAAAAACAAAAAA
GTTGTAGTACCAGTGTTGGCAGATTCGTTCGAACAAGAAGCCTCTAGAGAAGTGGATGCATCGAAAGGACTGACA
AATTCAGAAACCTTACAAGTTGAGCAAGACGGTAAAGTCAGATTATCGCATCAGGTCCGCCACCAGGTTGCACTA
CCACCGAACTATGATTATACGCCTATCGCTGAGCATAAGAGGGTAAATGAGGCTCGTACATATCCATTCACATTA
GACCCTTTCCAGGACACTGCAATCTCATGTATAGATAGAGGCGAATCTGTGTTGGTTTCTGCGCACACATCGGCT
GGTAAAACAGTTGTGGCTGAATATGCCATCGCACAATCTTTAAAAAATAAGCAAAGAGTCATTTATACCTCTCCA
ATTAAGGCTCTATCAAACCAAAAATACAGAGAACTTTTAGCAGAATTTGGAGATGTCGGTTTGATGACTGGTGAT
ATCACGATCAATCCGGATGCAGGCTGTTTGGTCATGACCACTGAGATTTTGAGAAGTATGTTATATAGAGGCAGT
GAAGTTATGAGAGAAGTTGCCTGGGTCATCTTTGATGAAGTGCATTACATGAGGGATAAAGAAAGAGGTGTTGTG
TGGGAAGAAACGATTATTTTACTGCCAGATAAGGTCCGTTACGTGTTTTTATCGGCCACCATTCCAAATGCAATG
GAGTTTGCTGAATGGATATGCAAAATTCATTCTCAGCCATGTCATATTGTCTACACAAATTTCCGTCCAACTCCT
TTACAACATTACCTGTTTCCAGCCCATGGAGATGGTATTTATCTGGTGGTTGACGAAAAAAGTACCTTCAGAGAG
GAAAATTTCCAAAAAGCAATGGCGTCCATAAGTAACCAGATAGGTGATGATCCAAATTCCACTGATTCAAGAGGT
AAAAAGGGTCAAACCTATAAAGGTGGCTCCGCTAAGGGTGACGCAAAAGGTGACATTTATAAGATAGTGAAAATG
ATTTGGAAGAAAAAGTACAATCCAGTGATTGTATTTTCGTTCAGTAAACGTGATTGTGAAGAATTAGCATTGAAG
ATGTCTAAATTAGATTTTAATTCTGATGATGAAAAGGAGGCTTTGACGAAGATTTTTAACAATGCCATTGCACTA
TTACCAGAGACAGACAGAGAGCTACCCCAAATTAAACACATTTTACCATTATTAAGAAGAGGTATCGGTATTCAT
CATTCCGGTTTACTGCCCATTTTGAAGGAAGTCATTGAAATTCTGTTTCAAGAGGGATTTTTGAAGGTGTTGTTT
GCAACAGAAACATTTTCGATTGGATTAAACATGCCTGCTAAAACTGTTGTCTTTACATCGGTCAGAAAGTGGGAT
GGTCAACAATTCCGCTGGGTTTCAGGTGGAGAATATATACAAATGTCTGGTCGTGCAGGTCGTCGTGGTTTAGAT
GATCGTGGTATTGTTATTATGATGATTGATGAAAAAATGGAACCTCAAGTTGCTAAAGGAATGGTTAAGGGCCAA
GCAGATAGGCTAGACTCGGCTTTTCACTTAGGATATAATATGATTTTGAACTTAATGAGAGTTGAAGGTATCTCT
CCGGAGTTTATGTTGGAGCATTCTTTCTTCCAATTTCAAAACGTTATTTCAGTACCAGTTATGGAAAAGAAACTT
GCTGAACTGAAAAAAGACTTTGATGGCATCGAAGTCGAAGATGAAGAAAATGTTAAAGAATACCATGAGATTGAG
CAGGCTATCAAAGGTTACCGTGAAGATGTTCGTCAAGTTGTCACCCATCCGGCAAACGCCTTAAGTTTCCTACAA
CCAGGCAGATTAGTTGAAATCTCTGTCAATGGTAAGGATAACTATGGCTGGGGTGCTGTTGTCGATTTTGCTAAA
AGAATTAACAAACGCAACCCAAGTGCTGTCTATACAGACCATGAATCCTATATTGTCAATGTGGTCGTCAATACC
ATGTATATAGACTCTCCAGTCAACTTGTTGAAACCTTTCAACCCTACCTTACCAGAAGGGATTCGCCCAGCGGAA
GAAGGTGAAAAAAGCATATGTGCTGTTATTCCCATAACTTTGGATTCAATCAAGTCGATTGGTAATTTGAGACTA
TATATGCCCAAAGATATTAGAGCCAGCGGTCAAAAAGAAACTGTTGGGAAGTCTTTAAGGGAGGTTAATCGGAGG
TTCCCGGATGGTATTCCCGTGCTGGATCCTGTTAAAAACATGAAGATCGAAGATGAGGACTTTTTAAAGCTAATG
AAGAAGATTGACGTTTTAAACACAAAGTTATCCTCCAATCCCTTAACCAATTCCATGAGACTAGAAGAACTATAT
GGTAAATATAGTAGAAAACACGATTTACATGAAGATATGAAACAATTGAAACGCAAAATTTCAGAATCACAAGCC
GTAATCCAACTGGACGATCTTCGTCGCCGTAAAAGAGTTTTGCGCCGTTTAGGATTTTGTACTCCTAATGACATT
ATTGAACTGAAAGGTAGAGTTGCATGTGAAATATCTAGTGGTGATGAACTGTTACTAACAGAATTGATCTTCAAT
GGTAATTTCAATGAGTTGAAACCGGAACAAGCAGCAGCATTATTATCATGCTTTGCATTCCAAGAACGCTGTAAA
GAAGCGCCTAGATTGAAACCAGAGCTTGCCGAACCTTTGAAGGCTATGAGAGAAATTGCAGCAAAGATCGCTAAG
ATAATGAAGGATTCTAAAATTGAAGTTGTAGAAAAGGACTACGTTGAAAGCTTCAGACATGAACTAATGGAAGTT
GTTTACGAATGGTGTAGAGGAGCTACTTTTACGCAAATCTGTAAAATGACCGACGTTTACGAAGGTTCGTTGATC
AGAATGTTCAAGAGATTAGAGGAATTGGTGAAGGAGCTGGTAGACGTCGCCAATACCATTGGTAACTCTTCACTT
AAGGAGAAGATGGAAGCTGTCTTGAAATTAATTCATAGAGATATCGTATCTGCTGGTTCTTTGTATTTATAG

>YDR351W 0.78 Cold
ATGACAGCACGTCGTTTGATTAATATAGTTCCTAATACGTCTAAGCTAGACCCTTTAAAGGAGGAGGACTCAACG
CATCTGAAACAAAATCAACCTAAGAAGTTTTCCACAAAGGAGTTGATGCTTTCAGAATATACCGAACGCAAAAGT
TGCAGCCTTCCGTTGTCTAAATCCCGATCAGGCAGTAGTGCATCCAGTAGCACTACAGGTTCGAACGGAAAGAAT
ATTGGAACAAGACGCCCTAGCTCCAACCTAGATTTCAATTTCGCTAGCCAAGATGTTGTTAAAAATGTATTAGGT
AATAATAATCCACACGTACCCACAGCGAAGTGCATTAGGCCAATATCAGATGATTCTATAGGTACATCGAGCACA
GAGATATTTTCTTCATCGCACTCAAATACGACATCTGATTCTTTATGTACATCAGATATAAGCTCAGAAGAGGGT
GAAATAGCAAACAGTAAGATGGAGGATAACTGCTTCTTCAAAAGTATGAGAGAAGCTGACCATAGAAGCAATATT
ACGCCCTTAAAGAAAAGCCGACCGGGATCAATACTACAGAAAACACGTACAGCAAGTAGCGCTGATAAAACAATC
TGCTCAATGAGTACCATAACTACATGCATTCCATCTCGTCAAAATTCGGTGTCAACTCCTAAACTCTCCAGGACA

GTAGGGCTGCCTGGATCGAGCAATACAACCAACAGTATAGCCGCATCACAAACTTCATTCATAAGCGAGAATGAC
TCTCCTCTTAAACATCATTGCATGAGTACCGCAACTATCCAAGAACCAAAACTCATGCCGATCACAAAAACGCCG
TATGTGCATTCCAATAGTACTTCTGTAATATTACCATATAAAACAACCCAACTTACTCCTTCTCAACGTTACCGT
TTAAGGAAAGAGCAAAATGACCAATCTTTGCGTAAAGCCATAAAAATGAAAGAAAAATTTTATGAAGATCAAGAC
GTTAACTTGGAACTTCAAGAGGGCGATGTCGACGGCTCGTTAATTTGGAACATTCCAATGGCATCTTTATCGACC
AGTTCTTTTTTAACATTATCTAAGTTTAATAGAAAAGAAATGAGTTTAGATTCAGCGCGGGGGGATGAAGAGATA
TTAATACAGGAGAACAATTGCGAAGGCAAACAACACTCATCTTCGGCTCTCTGTGTGGACAAAACTTTCCATCAG
GTTCATAGTACTAGAAAACATACATCAAATTCAAGTAACACTTTAAAAGAATCATGTCTGGACTATAAGGAGCTT
CCGCCAACATGCATTCCTGGTATATCTCCTGTGTCTGATTCACAGTACATCCAGGATACAATGAAAAATCTCTCC
CAAATATACCTGCATAGTTCCGAAAAAATCTCCAAAAGTATTCTTTCTGGTCGTTCAAGATCTGTACAGAGTTTA
CCACTGGAATTTAAAGAAGCCAGTAGTCAGGGAATGGAAGACCTAATGCTAGTTTCAGAGGACAAACTAAAGGCC
GTAAGTCATTTCCGTCCGAGTTGGCTCCCGCCCAAGGATTTCAAAGAGAGGAAATTGCAAGATAAACAAATCTAC
AAAAATATCGACTTAGCATCAATGGAAGAGTTGCAAAAAAAATAAAGAACGTGACGAAAAAGCCAAAAAGAATGAG
CAGAATAAGGTCAAATTCCAGCATTTACTGGACCGAGGCATTACTCGTAACTCGTCATTAAGTGAATTAAAGAAA
ATTATTTGGGAAACACCTTTGATATCTAAAGTACGGCTTCAAATATATAGTCAACTTCTACAAAGTGACAATTGC
TTGATAACAAAATGTTTTATTGAATCATTTGAGGAAGTAATGCAGCTACTCAACAAAATGGATTTTCCCAAAGAT
AAAGAATTTGAAATAAGACAGCTTATTGAACACGATGTACAGGAAAAGGTTTTTTATAAAAATGGAACAGATAAG
CAAGTGGTTTCTGACTTGATGCTATTACTGCAACTGAAATCTATATCTCAACAAGGTTTGGTTACTGGTGATGAG
ATGCTATTCTACCATTTTTTAACTGATCAATCTTTTGGGACTTTGAAGGAAACATGGGAAATGGTCAACTTGATA
CAGATGACGTGCTTCAGTGAAATTTGCAAGGAAAAATACGATTCAAGAATATTAAATCCCAGAGGAATTGTTGCC
CATCTTTTACGAAAAGATGAGTTCAAAAATGAATTCAATGGCGGATGTTTGAATAGCAATACATGGTGGAACATA
TTGCAAAGAATGGACCATAAACTTTTTATGTGGGTAATGGATGTAATTATCGTTCATAATGGCCAAAACTTTGCA
AACTACCCCGTAAAAATGGAAATATTCAAAGACAAAGTATGGGAATACTATAGATCTAAGAAGGTTATTGTAAAT
TATAAGATTTTGGTTTCACTAACAGTTAACGTATTGTTAAACTACCACTTCGGATATGATAATCTGAAACATTTA
TCAGATTTGGATGATAAGCATTTTTGTATTCCACTGTATACGGAGGATAGTATCGAAGAGGAAAACCTCAATAAT
ATTTTCACAAAGTGGTGGTTGCACTACTACCGAAAATTACGTTAG

>YBL105C 0.82 Cold
ATGAGTTTTTCACAATTGGAGCAGAACATTAAAAAAAAGATAGCCGTCGAAGAAAATATTATTCGGGGAGCTTCT
GCCCTCAAGAAAAAGACTAGCAATGTTATGGTCATTCAGAAATGTAATACGAATATTAGAGAGGCACGTCAAAAT
CTTGAGTACTTGGAGGATAGCTTAAAAAAATTACGGTTGAAGACCGCTCAACAAAGTCAGGGTGAAAATGGCAGC
GAAGATAATGAGCGATTCAATTCGAAGGAATACGGGTTTCTTTCTACCAAATCGCCAAATGAACACATATTTTCT
CGTTTGGATTTAGTAAAATATGATTGCCCTTCTTTGGCGCAAAGAATCCAATACATGTTGCAACAATTAGAATTC
AAATTACAGGTGGAAAAGCAATACCAAGAAGCCAATACTAAATTGACTAAATTGTATCAAATTGACGGTGATCAA
CGTAGTAGTTCTGCGGCAGAAGGGGGTGCTATGGAATCCAAATACAGGATTCAAATGCTTAACAAAGCTTTGAAG
AAATACCAAGCTATTAACGTTGACTTTGATCAATTTAAACACCAACCCAATGACATAATGGATAATCAACAACCG
AAATTCCGAAGAAAACAACTGACTGGCGTTCTAACAATTGGAATAACTGCTGCTAGAGATGTTGACCATATACAA
TCACCGATGTTTGCCAGGAAGCCAGAGAGCTACGTTACTATAAAAATCGATGATACGATCAAAGCCAGAACGAAG
CCTTCTAGAAATGACAGGTGGAGTGAAGATTTTCAAATTCCAGTTGAAAAAGGGAATGAAATCGAGATTACTGTA
TATGATAAGGTGAACGACTCGCTCATTCCAGTGGCTATAATGTGGTTACTGCTTTCCGATATCGCTGAAGAAATT
CGTAAGAAGAAAGCCGGACAAACAAATGAACAACAAGGATGGGTCAACGCTTCAAACATTAATGGTGGTTCTTCT
CTCGCTAGCGAAGAGGGAAGCACATTGACTTCTACATACTCAAACTCAGCCATTCAGTCTACTTCAGCAAAGAAT
GTGCAAGGCGAAAATACATCAACTAGTCAAATCAGTACGAATTCATGGTTTGTCTTAGAACCATCCGGGCAGATT
TTGTTAACACTAGGATTCCACAAGTCTTCTCAAATAGAAAGGAAGCAGTTGATGGGTGGACTACATCGTCATGGT
GCTATTATCAATAGGAAAGAAGAGATATTCGAACAGCATGGCCACCACTTTGTACAAAAATCATTTTACAACATT
ATGTGCTGTGCATATTGTGGTGATTTCCTTCGGTATACTGGGTTCCAATGTCAAGATTGTAAATTTTTATGTCAC
AAAAAATGTTACACCAATGTTGTTACTAAGTGTATTGCTAAAACTTCTACTGATACGGATCCGGACGAGGCAAAG
TTGAACCACCGTATTCCTCATAGATTCCTGCCTACTTCAAACCGTGGTACTAAATGGTGCTGTCACTGTGGTTAT
ATTTTACCATGGGGTAGACATAAAGTACGCAAATGTTCTGAATGTGGTATAATGTGTCATGCTCAATGTGCTCAT
TTGGTTCCCGATTTCTGTGGCATGTCGATGGAAATGGCTAATAAAATTCTGAAAACGATTCAAGACACAAAACGT
AATCAAGAGAAAAAGAAGAGGACAGTTCCGTCGGCACAGTTGGGGTCATCTATTGGTACTGCCAATGGTAGTGAT
CTTTCCCCATCCAAATTAGCTGAAAGGGCAAACGCTCCTTTGCCTCCTCAGCCAAGAAAACATGATAAAACTCCT
TCGCCTCAGAAGGTAGGAAGAGATTCTCCAACTAAGCAGCATGATCCTATTATTGATAAAAAAATTTCACTACAA
ACGCACGGGCGTGAGAAACTAAATAAATTTATCGATGAAAATGAGGCCTATTTAAATTTTACAGAAGGTGCACAA
CAAACCGCCGAATTTTCATCACCAGAGAAAACACTGGATCCAACGTCGAATAGACGTTCCTTGGGACTCACTGAT
TTATCTATTGAGCATAGTCAAACTTGGGAATCTAAAGATGATTTAATGAGAGATGAACTTGAACTGTGGAAAGCA
CAACGTGAGGAAATGGAACTTGAAATAAAACAAGATAGTGGGGAAATCCAAGAGGATCTAGAAGTTGATCACATT
GATTTAGAGACCAAACAGAAACTAGACTGGGAGAACAAAAATGATTTCCGTGAAGCTGATTTGACAATAGATTCC
ACACACACAAATCCATTCCGCGATATGAATAGTGAAACCTTTCAAATTGAACAAGACCATGCTTCAAAGGAAGTA
CTACAAGAAACAGTGTCCTTGGCTCCTACAAGTACTCATCCCTCCAGAACCACTGATCAACAATCTCCGCAGAAA
TCACAAACCAGCACAAGCGCAAAGCATAAAAAGAGAGCGGCTAAACGTCGTAAGGTTTCATTAGATAATTTCGTA
TTACTCAAAGTTCTTGGTAAAGGTAATTTTGGTAAAGTTATTTTATCTAAATCAAAGAATACTGACAGGCTTTGT
GCCATAAAAGTTCTGAAGAAAGATAATATTATTCAAAATCATGACATCGAGAGTGCAAGAGCAGAAAAGAAAGTA
TTTTTGTTAGCCACAAAAACTAAACACCCATTCTTAACCAATCTATACTGCTCTTTTCAAACTGAAAACCGTATA
TATTTTGCTATGGAGTTTATTGGGGGCGGTGACTTAATGTGGCATGTTCAAAACCAAAGACTATCTGTAAGAAGG
GCCAAATTTTATGCCGCCGAGGTCTTACTGGCTCTGAAATATTTTCATGATAATGGTGTAATATACCGTGATTTG
AAGTTGGAAAACATTCTATTAACTCCAGAAGGTCATATAAAAATTGCCGATTATGGTTTGTGTAAAGATGAAATG

TGGTATGGTAATAGAACTTCTACATTTTGTGGCACACCAGAATTTATGGCTCCAGAAATTTTAAAAGAGCAAGAA
TATACCAAAGCTGTCGATTGGTGGGCATTTGGGGTGCTGCTATATCAAATGCTACTGTGCCAATCTCCATTCTCA
GGAGATGACGAAGATGAAGTTTTTAACGCTATCCTTACCGATGAACCCTTATACCCAATAGATATGGCAGGTGAG
ATTGTACAAATATTCCAAGGCCTATTAACAAAAGATCCTGAAAAGAGGTTGGGTGCTGGTCCCAGGGACGCAGAC
GAAGTTATGGAAGAACCTTTCTTCCGTAACATCAACTTTGACGATATCTTAAACCTTCGCGTGAAACCACCCTAC
ATCCCAGAAATTAAATCTCCGGAAGATACATCATATTTCGAGCAAGAATTCACCTCTGCGCCACCCACACTTACA
CCTCTGCCCTCTGTTTTGACAACGAGCCAGCAAGAAGAGTTTAGAGGATTTTCCTTTATGCCAGATGATTTGGAT
TTATGA

>YGL014W 0.78 Cold
ATGTCTACAAAAGGTTTGAAAGAAGAAATCGATGATGTACCATCAGTAGACCCTGTCGTTTCAGAAACAGTCAAT
TCTGCTTTAGAGCAGTTGCAACTAGATGATCCAGAGGAAAACGCCACCTCTAATGCATTTGCGAATAAAGTTTCT
CAAGATTCTCAATTCGCTAATGGCCCTCCGTCGCAAATGTTTCCACATCCACAAATGATGGGTGGAATGGGCTTC
ATGCCCTACTCTCAAATGATGCAGGTTCCTCATAATCCTTGTCCATTTTTTCCGCCCCCTGATTTTAATGATCCA
ACAGCACCATTGAGTAGCTCGCCCTTGAATGCAGGCGGTCCACCAATGTTATTCAAGAATGACTCACTTCCATTT
CAAATGCTGTCTTCGGGTGCTGCGGTAGCAACTCAAGGTGGACAAAATCTAAACCCATTGATAAATGACAATTCA
ATGAAGGTATTGCCAATCGCATCGGCTGATCCGTTATGGACTCATTCAAACGTACCAGGATCAGCATCTGTAGCC
ATTGAAGAAACCACCGCTACTCTACAAGAAAGCCTACCATCTAAGGGCAGGGAGTCTAATAATAAGGCTAGTTCG
TTCAGAAGACAAACTTTTCATGCTTTATCACCAACTGACCTTATCAATGCGGCCAACAATGTAACCTTGTCAAAG
GACTTCCAATCTGACATGCAGAATTTTTCTAAGGCTAAGAAACCGTCTGTAGGAGCTAACAATACTGCAAAAACC
AGAACTCAATCCATATCTTTTGATAATACTCCCTCCTCAACGTCATTTATACCCCCAACCAATAGTGTTTCTGAG
AAATTATCCGATTTCAAAATAGAAACCTCGAAGGAGGATTTGATTAATAAAACTGCACCAGCTAAAAAAGAGAGT
CCTACAACTTATGGTGCAGCATATCCATATGGGGGACCTTTACTTCAACCAAATCCTATTATGCCAGGCCACCCA
CATAATATATCCTCCCCTATCTATGGTATTAGATCACCTTTTCCTAATTCTTATGAAATGGGCGCGCAATTTCAA
CCTTTCTCTCCGATTTTAAATCCTACGAGTCATTCACTAAATGCAAATTCTCCAATTCCTCTAACCCAATCGCCA
ATTCATCTTGCACCAGTTTTAAACCCTAGTTCAAATTCTGTTGCCTTTTCAGATATGAAGAATGATGGTGGTAAG
CCCACCACCGATAACGACAAGGCGGGTCCAAATGTTAGGATGGATTTAATAAATCCTAATCTTGGGCCATCAATG
CAACCTTTCCACATATTACCTCCCCAGCAAAACACCCCCCCTCCTCCCTGGCTTTATAGCACTCCACCTCCCTTC
AACGCAATGGTTCCGCCTCATTTGTTGGCTCAAAATCATATGCCGTTAATGAATAGCGCCAATAATAAACATCAT
GGTCGTAATAACAATAGCATGTCAAGTCATAATGACAATGACAACATTGGTAATTCTAATTACAACAATAAAGAC
ACAGGTCGTTCTAACGTTGGTAAAATGAAAAATATGAAAAACAGTTATCATGGCTACTATAATAACAATAATAAT
AATAATAATAATAACAATAATAATAATAACAGTAATGCTACCAACAGCAACAGCGCGGAAAAACAACGTAAAATT
GAGGAGTCGTCGAGATTTGCGGACGCAGTTTTAGACCAATATATCGGAAGTATTCACTCATTGTGTAAAGACCAA
CATGGTTGTCGTTTTCTGCAAAAGCAGTTGGATATTCTCGGCAGTAAGGCGGCGGACGCAATTTTTGAAGAAACT
AAGGATTATACGGTTGAATTGATGACTGATTCATTCGGTAATTATTTGATCCAGAAGCTATTGGAAGAGGTTACC
ACAGAACAAAGAATCGTACTCACAAAAAATATCTTCCCCTCATTTTGTCGAAATTTCCTTAAACCCTCATGGTACT
AGGGCATTACAAAAACTCATTGAATGCATCAAAACAGATGAAGAAGCACAGATTGTTGTTGATTCTTTACGCCCT
TATACTGTCCAGTTGAGTAAGGATTTAAATGGTAATCATGTTATTCAAAAATGTTTGCAAAGGTTGAAGCCTGAA
AACTTCCAGTTTATCTTTGACGCAATCTCTGATAGCTGTATTGATATTGCTACTCATAGACACGGGTGTTGCGTT
TTGCAACGTTGTCTAGATCATGGGACTACAGAACAATGTGACAATCTGTGTGATAAGTTGCTAGCCCTTGTTGAT
AAATTAACTTTGGATCCATTTGGCAACTATGTGGTGCAATATATAATTACCAAAGAGGCTGAGAAGAACAAATAT
GATTATACGCATAAAATTGTCCACCTGTTGAAACCAAGAGCCATCGAACTTTCTATCCATAAATTTGGATCAAAT
GTGATTGAAAAAATCTTGAAGACAGCTATTGTTTCGGAGCCAATGATTCTGGAAATTTTAAATAATGGTGGCGAG
ACGGGTATTCAATCATTGTTGAATGATAGCTACGGAAATTACGTTTTACAGACAGCATTAGACATTTCTCATAAG
CAAAATGACTATCTCTATAAAAGACTATCAGAGATTGTGGCGCCTTTACTGGTGGGCCCCATAAGAAATACACCT
CATGGTAAAAGAATCATCGGAATGTTACATTTAGATTCATAG

>YNL314W 0.8 Cold
ATGGATGAATCGGTGGATCCTGTGGAGCTGCTTCTACGACTACTGATACGGCACAAACCTCATCTGAAACCATAT
GCCTACAGACAAGATAGCTGGCAAAGGGTGCTCGATGAGTACAACAGACAGACTGGGTCAAGATATAGACAATCA
AGGACGTTAAAAACCAAATTTCGTCGACTGAAGGACCTCTTCAGCGCAGATCGAGCCCAATTCTCTCCTTCCCAG
TTGAAGCTGATGGGAGCACTCTTGGACGAAGCACCAGAACATCCAAGACCAAGAACTAAATTCGGAAATGAATCA
TCTTCATCCTTATCATCATCTTCTTTCATTAAAAGTCATCCGGGGCCTGATCCGTTTCAACAATTATCATCCGCT
GAACATCCGAATAACCACAGCTCCGACGATGAGCATTCAGGCTCACAACCGCTGCCCCTGGATTCAATAACGATT
GGAATTCCGCCTACTCTTCACACAATCCCCATGATTCTGTCTAAGGATAACGACGTCGGGAAAGTCATCAAAAGC
CCTAAGATAAACAAGGGTACAAATAGGTTCAGCGAGACAGTACTGCCTCCACAAATGGCTGCTGAGCAATCGTGG
TCGGACTCTAATATGGAATTGGAAATATGTCTAGATTATCTTCACAACGAACTCGAGGTGATAAAGAAAAGGCAA
GAAGATTTTGAGTGTAAAGTTTTAAACAAGCTCAACATAATTGAGGCTCTCCTTTCACAGATGAGACCACCCAGC
CAAGGAGATAAAATATAA

>YIL016W 0.8 Cold
ATGTCGCACAACGCAATGGAACATTGGAAGAGCAAGCTGAGCAAAACAAGTACCTCGACGTACGTCCTCTTGGCC
GTGATTGCTGTTGTATTTTTGGTTACAATCAGAAGACCCAATGGCAGCAAGGGAAAAAGCAGTAAAAAGAGGGCT
TCTAAAAAGAACAAGAAAGGTAAGAATCAGTTTGAAAAAGCGCCGGTTCCTCTGACTCTAGAAGAACAAATTGAC
AATGTGTCGTTGAGATACGGCAACGAGTTGGAAGGTCGTAGCAAGGATCTAATAAACAGATTTGATGTGGAAGAT
GAAAAGGACATCTACGAGCGTAACTATTGCAACGAAATGCTTTTGAAGTTGCTGATTGAGTTAGACAGTATCGAT

TTGATTAACGTGGATGAGTCCTTAAGAAGGCCGTTGAAGGAAAAAAGGAAAGGCGTCATAAAGGAAATTCAGGCT
ATGTTAAAAAGTTTGGACTCTCTTAAATAA

>YNL182C 0.68   Cold
ATGGATGAGCAAGTTATTTTTACAACAAATACCTCAGGAACAATAGCTTCTGTACACTCATTTGAACAGATAAAT
TTGAGGCAATGCTCCACTCAATCAAGAAATAGCTGTGTTCAAGTAGGAAATAAATACCTTTTTATTGCTCAAGCA
CAAAAAGCATTAATCAATGTCTACAATCTGTCAGGTTCTTTCAAAAGAGAATCTGTTGAACAGCGCTTACCATTA
CCTGAAATCCTAAAATGTCTGGAAGTAGTTGAAAATGATGGTGTGCAGTATGATAGAATTCAAGGTGTCAATCAT
AATTTACCAGACTTCAATCTTCCGTACCTTTTACTTGGCTCCACCGAATCGGGTAAATTGTACATATGGGAGTTA
AATTCAGGGATTTTATTGAACGTGAAGCCTATGGCTCATTACCAAAGTATCACCAAGATTAAGTCCATTTTAAAC
GGCAAGTATATTATTACTTCTGGTAACGATTCGAGAGTTATTATATGGCAAACTGTTGACTTGGTATCAGCGTCC
AATGATGATCCTAAGCCTTTATGTATCCTTCACGATCATACTCTACCCGTGACAGATTTCCAAGTTTCTTCTAGT
CAAGGAAAATTTTTATCATGTACTGATACGAAACTCTTCACAGTATCTCAAGATGCTACCATTAGATGCTATGAT
TTGAGTTTAATAGGCAGCAAAAAGAAGCAGAAGGCAAACGAAAATGACGTTAGTATTGGTAAGACCCCAGTATTG
CTTGCGACATTTACAACTCCTTATTCTATCAAATCCATTGTACTGGATCCTGCTGACAGAGCATGCTATATTGGT
ACTGCGGAAGGTTGTTTTTCATTGAATTTATTTTATAAACTAAAGGGTAATGCTATCGTTAATCTGCTACAGTCC
GCCGGAGTAAACACAGTTCAAAAAGGTAGGGTTTTTTCCCTAGTGCAACGTAACTCACTAACTGGCGGCGAAAAT
GAAGATTTGGATGCACTATATGCAATGGGCCAACTTGTCTGTGAGAATGTCCTAAATTCAAATGTGTCATGCCTA
GAAATATCAATGGATGGTACATTATTATTGATCGGTGATACGGAGGGGAAAGTTTCTATTGCGGAAATTTACTCA
AAACAAATCATTAGAACTATCCAAACTTTAACTACATCACAGGATTCAGTTGGAGAAGTGACCAATCTCTTAACC
AACCCTTACAGACTCGAACGTGGAAATTTACTTTTTGAAGGAGAATCCAAAGGCAAACAACCTAGTAATAATAAT
GGTCACAATTTTATGAAGATACCAAACTTACAAAGAGTTATCTTTGATGGTAAAAACAAAGGCCATTTACACGAT
ATTTGGTATCAGATAGGAGAACCAGAAGCAGAGACAGATCCTAACCTCGCATTACCACTTAACGACTTTAATGCC
TATTTGGAGCAGGTCAAAACGCAAGAATCGATATTTTCACATATCGGTAAGGTGTCAAGCAATGTAAAAGTGATT
GACAATAAAATCGACGCCACTTCATCTTTAGACAGCAATGCCGCTAAAGATGAGGAAATTACAGAACTTAAGACC
AACATAGAAGCATTAACTCATGCCTACAAGGAGTTACGTGACATGCACGAAAAGCTGTACGAGGAACACCAACAG
ATGCTTGACAAGCAATAA

>YNR023W 0.7Cold
ATGTCCAAAGTAATGAAACCCAGCAACGGAAAAGGTTCTAGAAAGAGTTCAAAAGCAGCCACACCTGATACTAAA
AATTTCTTTCATGCAAAGAAAAAGGATCCGGTGAACCAAGATAAAGCAAATAATGCTTCTCAAATTACGCCAACA
GTGCCACATTCTCATCCATCAGATATGGTTATCCCTGACCATTTAGCCGAATTGATTCCTGAGCTGTATTCTTTC
CAACAACTGGTGGATTCCGAGAAAAGATTAGATCATTTTATTCATCTGCGAAACTTACATATGAAGCGAATGGTT
GCGCAGTGGGAGAGGTCAAAGCTATCCCAAGAGTTTCTTTATCCTCATTTAAATTTTCCAAATGTAAAATTTCTC
CGAATCTTCATTTCAAATGTTAGTGAAAACCAACCCTGGCAAATGGACACAAACAATGAGGCTGACCTTATGGCA
CTGGAGAATGCCACTTGGACGATGAGAATCGAAGGTCGATTATTGGACAATGTGCAAGCTAATGATCCAGCAAGA
GAAAAATTTAGCTCATTCATAGAATCGATAGTGGTAGACTTCAAAAATAAGGAAAACGATAATGTGCCATCGACT
AAATTTAATGCGGCCCCTGAAGAAAATGCCACCGAAGGACCCAGTGATAAGAAATTGAACTTAAACTTACCGTTA
CAATTTTCACTGCCGAATGGTGACAACTCTACTACGACTAATACCGACCAAAATAATGCCACGATGGGGGAGGAG
ACTGCCAAAAAGGATATGAGTTCTACAACTCCAAAACTAGAATCAGTGAAATGGCAGTATGACCCGAATAATCCT
GTAGACTTCGATGGTCTCGATATTAAGAGGGTAGGGTCTGAAAATGTAGAATGCACTATAAGCATCCTGCGAAAA
TCATCCCCAGAAGAGCCATTTATGAGCTACTCTCCACAACTAACCGCAATAATAGGATTGAAGAGCGGTACATCA
CATGATGCAATATTTTCCATATATAAATACATCCATTTAAATGAATTGCTTACCAATGATGAGTCTGCATTCGAA
AATTTGATGGGCAACAGGAACAATCACAATAGCAACACCAGTACTAGTAAGATGCTTGATGCAGCCTCCAGTCAA
GTTTCTATTGTAAAATTAGACACTCAGCTTATTACCTTATTACCCAGCAGTCTTAAAGAATCTTCTCCAGATACA
ATGAAATTAACGGATTTACTGTCCCTTATTAATAGTACTCACCTGCTCCCATTACAGCCAATTGAAATTGATTAC
ACAGTACGCGTTGATAAAGCTTCCACTTATGGTGAGTTGGTTCTTGATATTGAAGTACCTGATGTCAACGCTCTA
AAATTCAATAATACACAAAGAGAAAGTCAAATTGGGGCTGCTGAATTGAATGAAAATGCCAGGGAATTGGAACAA
ATCAAACCCAAGATAGCTTTACAGGACAAAGAAATAACGTCCGTTCTCTCGAATCTACACGAGAGCAACAAACGA
TACCGTTTCTTCAAGAAAATTAGTGAGGACCCAGTTAAAGCATTAAATGAATGCATTGCTTCTACCTCGAATGCT
TTGAAAGTTTTATCGGGAGATGAGGGCTATAATGAAGATATGGTAAGGCGGGCTAACTTTTACAAGGAAAATGAA
GCTATGTTGCGCGAGAATATAGAAGTCATATTATCAAATGGACGAATGTAG

>YDR083W 0.76   Cold
ATGGCCTTATTTAACGTAGAAGGTTGGTCTATTAAGACAAAAACCGTCGCTTTTGATAACAAGACTAATAAGTCT
TCAAAAGATAAAAAAAAAAATAATAGAAAAAATGGCAAACTTACAAGAGAACAGAAGTTGAAAGAAGAGACAGAG
GCTGAGTTGAAGGAACAAGTGGAAGACATTCCTTCCGAAGGATCAGTAGCCAAAGACATCCCAAAAAAAAACCAA
GAGAAAAGTGATCAAAATGAAACGAGCAAGAAACGCAAGCACGATGAAGAAGCCCCTCTGATGCAAGTAAAAGAA
AATATTGAAAAACCTACAAAGAAGCAACTAACCCCTTTACAACAAAAGATGATGGCTAAACTGACTGGTTCTAGA
TTTAGATGGATCAATGAACAACTGTATACAATTAGCTCTGATGAAGCTTTGAAATTAATAAAAGAACAACCACAA
TTATTTGACGAATATCATGATGGTTTTAGATCACAAGTGCAAGCATGGCCGGAAAATCCAGTTGATGTTTTTGTT
GACCAAATTCGTTATAGATGCATGAAACCTGTGAATGCTCCAGGTGGGTTACCAGGTCTTAAGGATAGTAAAGAA
ATAGTTATTGCTGATATGGGGTGTGGTGAAGCTCAATTAGCATTAGAAATCAACAATTTTTTCAAAAATTACAAT
AAGAAAGCGAAGAAATATTTGAAAAGACGCCATAAAGTCCACAGTTTTGATTTGAAGAAAGCTAACGAAAGAATA
ACTGTGGCAGATATTAGAAATGTGCCGCTACCAGATGAGTCCTGTACTATAGTGGTCTTCTGCCTGGCTCTAATG
GGTACAAATTTCCTCGATTTCATAAAAGAAGCTTATAGGATTTTAGCGCCAAGGGGTGAATTATGGATCGCAGAA
ATTAAATCAAGGTTTAGTGACGGCAAAGGTAATGAATTTGTAGACGCTTTGAAGCTGATGGGATTTTTTCACAAA

AAGACCTTCGACGAGAATAAGATGTTTACAAGATTCGAATTTTTCAAGCCACCTGCGGAGATTATTGAAGAGAGA
AGGCAGAAATTGGAAAGAAGACAAAAGTTTATTGAAGTTGAAACTGAGAAGGAAGAATTAGAAAAGAAAAGACGG
AAAATTGCCGAAGGAAAATGGCTCTTGAAGCCCTGTATTTATAAAAGAAGATAA

>YJR003C 0.78 Cold
ATGTTACGCTCTCTTCATTCTGCGGCTACGTTATCTAACAAGAGGTTTTACTCTCTCATATCTCATTCAAATAGA
AAGAACATAATCAAAAAGCTTCTACGTCACCCTTCTTTCGATCCAATTCGTCATCATTTGCCTGAAGATATAACC
ACCATTGACCCATACTCTCTATCGCAGAATGTTATCGAAAGTTTAAACAAATTAGAGGTTCCTAAAAAGGATGCG
GCCATGGTACACAATATGATGATAGAAAACTTAAGTGATCTTGACTACGGCGTTGCAACAATTCACTCAAATAAT
TTGCGTGATCTTGATTTAAAACCTTCACTTCCCGCCATCAAACAAATTATTAGGAATAATCCAGGAAGAGTACAA
AGCTCTTGGGAACTTTTTACACAATACAAGGCCTCAATGGAAAATGTTCCTGATGAACTGATGGAAGTGGTCTTA
GAAAAAATTATCAAGTTTGACAAGGCAGAAAAGGTTGATGGAAAAAAGAGTCTGACTTATCAAGACCTCGTTAGA
TGTCTTTATTTAATCAACCATTTTTCTTCAAATTATAATTTGCCTTCAGAGCTCGTGGAACCTATCCTTATTTAC
ATAGTGGACAACGGAATTCCCAATGTTTTAGGTTCTGTACTCAAATATAAAATACCGCTGAGCTTTTTTGATAAG
TATGTCAGTGAAATGACACAATACCAAATTTGTGAACTTTATGATTTTTATTCGCTTGATAATATAGTAGCTGAT
CCATTAGTACTACACAAGTGTCTAACTGTTCTGGGCGAAAACGAGAAAATTCAGCAAACTGAAGAAGAAAAAGAA
ATTATTAGCAAGTTGGAGGAAGAAATAGATATTGTTAAATCACAATGTCATGATAATTGGAGCCTCGAATTTCCG
AATTGGAGTGTACGCAAAACTGCAACTTCATTCGAAGAATTATTTTTAGAGATCCAAAAAAGAAACATAGACAAG
AAGGATTTCGAATTAGCTCACAAGCTGTTAAGATTAATTGGCGCTTTCAAAGGTAAAGTTTCTCTATTTTTTAAA
TTATACGACGAATATCTCTTAAAATTCAAAAATAATGAGGACGACTTAATGTTTGAAGCGTTTTTAACGCTCTGC
TGCCAGGGTTATAAAAGCAGTAATGAAAAAATGCTACAATACGCTGAAGCTTTTATTAAAGAAGACTTTGATTCC
AAACTAGAGAGTAAAATTCAAAGTGTGTTAATTGTTGCTAACGCGAAAGCAAATATTGATCTATCTCTCAAAATT
TACAATTCTAATATATCTACTGCTAAACGAGAGAAAGATAAGTACACAGATCTTGCTGAAAGTGACGTATTAACT
GAATCGCTCATTTTAGCATTCTTATCCAGAGATGATGCTGATTTTGCCAGGGTCATCTTTGATGGCGCACTAGGA
GAAAAGCTGATATCAGGGCCGACTGCTGCAAAAAAAAATAAAAAATCTCTTAGCCCAGTATGGAGAAGCGTTAGAA
ACAAAAACAAGCAAACAAGTTATGCAGACTAAGATAGAACATTATATGGAAAGCATATAA

>YER041W 0.74 Cold
ATGGGTGTCTCACAAATATGGGAATTTTTGAAGCCATATCTGCAAGATTCCAGAATTCCGTTGAGAAAGTTTGTC
ATAGACTTTAATAAATCGCAAAAAAGAGCTCCAAGAATCGCGATTGACGCATATGGATGGCTATTTGAGTGTGGA
TTTATCCAAAATATAGATATAAGCCCCAGATCTAGATCAAGATCAAGGAGTCCTACCCGTTCTCCGCGTGATAGT
GATATCGACAGTAGTCAAGAATATTATGGTAGCAGAAGTTATACAACTACAGGAAAAGCTGTGATAAACTTCATA
TCTCGATTAAAAGAACTACTGAGTTTAAACGTCGAATTCTTGCTTGTATTTGACGGAGTTATGAAACCCTCTTTC
AAAAGGAAATTTAACCACGAACAGAACGCTACTACTTGTGATGATGAGAAAGAGTACTATTCAAGCTGGGAACAG
CATGTTAAGAATCATGAAGTTTATGGCAATTGTAAAGGATTGCTAGCGCCATCTGACCCTGAGTTCATCAGTCTT
GTACGGAAATTATTAGACTTGATGAACATTTCATATGTAATCGCATGTGGGGAGGGAGAAGCCCAGTGTGTTTGG
CTGCAAGTTTCTGGGGCTGTAGATTTCATTTTAAGTAACGATTCAGATACTCTCGTATTTGGGGGAGAAAAAATC
CTGAAAAATTACTCGAAATTCTACGATGATTTTGGCCCAAGTTCAATAACTTCCCACAGTCCCAGTAGACATCAT
GATAGTAAAGAATCTTTTGTTACTGTTATAGATCTACCCAAAATTAATAAAGTAGCAGGGAAGAAGTTTGATCGC
CTGTCTCTCTTATTTTTCAGCGTGCTATTAGGTGCCGATTACAATCGTGGAGTCAAGGGTTTGGGTAAAAACAAA
TCTTTGCAGTTGGCTCAATGCGAAGATCCTAACTTTTCCATGGAATTTTATGACATTTTCAAAGATTTCAATTTA
GAAGATTTGACATCAGAAAGTCTGAGGAAGTCTCGGTATAGATTGTTTCAAAAAAGATTATATTTATACTGCAAG
GATCATTCTGTCGAGCTATTTGGAAGAAATTATCCTGTTTTATTGAATCAAGGTTCATTTGAAGGCTGGCCATCA
ACTGTCGCAATCATGCACTATTTTCACCCCATTGTCCAACCATATTTTGATGAAGAAGTTCTCAGTGATAAATAC
ATAAATATGGCTGGAAACGGACACTACAGAAACTTAAATTTCAATGAACTGAAATATTTTTTACAAAGTTTGAAT
TTGCCTCAAATTTCTAGCTTCGACAAGTGGTTTCATGATTCTATGCACGAGATGTTTCTATTAAGAGAATTTTTA
TCAATTGATGAGTCCGATAATATAGGTAAAGGTAATATGAGGATTACCGAAGAAAAAATTATGAACATAGATGGT
GGAAAGTTCCAAATACCATGTTTTAAAATACGGTATACAACATTCTTGCCTAATATACCCATTTCTTCTCAATCT
CCTTTGAAGCGCAGTAATTCTCCCAGTAGAAGCAAGAGTCCCACACGACGTCAGATGGATATAATGGAACACCCA
AATAGTCTTTGGTTACCCAAATACTTGATACCTCAATCGCATCCATTAGTAATTCAATATTATGAGACACAGCAA
CTTATACAGAAAGAAAAAGAGAAGAAAGGTAAAAAATCGAATAAGTCTCGGCTACCACAGAAGAATAACTTGGAT
GAGTTTTTAAGAAAGCATACTTCGCCGATTAAAAGTATCGGAAAGGTAGGCGAGTCGAGAAAGGAAATCCTGGAA
CCTGTGAGAAAAAGGCTTTTTGTAGATACAGACGAAGATACTAGTCTGGAAGAAATACCGGCTCCCACAAGACTG
ACCACCGTCGATGAACACAGCGATAATGACGATGATTCGTTGATTTTTGTAGATGAGATTACAAACAGCCAAAGC
GTTTTAGATAGTTCACCCGGTAAAAGAATTCGGGATCTTACACAGGACGAACAGGTTGACGTTTGGAAGGACGTA
ATAGAAATATCACCAATAAAAAAGTCAAGAACTACTAATGCTGAGAAAAATCCGCCTGAGTCTGGTTTAAAATCA
CGCTCAAGCATCACAATCAATGCTCGCCTTCAAGGTACTAAGATGTTGCCTCCAAACTTGACTGCTCCAAGGTTA
GAAAGAGAACACTCCTCTGTCCTTGACCAACTTGTCACAGACGCACAAGATACAGTCGACCGGTTTGTAGCCTGT
GACAGTGATAGCAGTAGCACTATTGAATGA

>YLR143W 0.82 Cold
ATGAAGTTTATAGCATTAATATCAGGTGGGAAGGACTCGTTCTACAATATTTTTCACTGCCTGAAGAACAACCAT
GAATTGATTGCCCTGGGAAATATATACCCAAAAGAATCAGAAGAGCAAGAATTAGACTCTTTCATGTTCCAAACA
GTAGGACATGACTTAATAGACTATTATTCTAAATGTATTGGTGTTCCACTATTTAGACGCTCAATATTACGGAAC
ACTTCTAACAACGTCGAGTTGAATTATACTGCTACTCAGGATGATGAAATTGAAGAACTATTTGAACTTCTAAGA
ACTGTCAAAGATAAAATTCCAGATTTAGAAGCTGTTAGTGTAGGTGCCATCTTATCGTCTTACCAGAGGACCAGA
GTGGAAAATGTTTGTTCCAGATTGGGTCTAGTAGTGCTAAGCTATTTATGGCAAAGGGATCAAGCCGAACTAATG

GGTGAAATGTGCCTTATGTCCAAGGATGTTAACAATGTTGAAAACGATACTAATTCAGGAAATAAATTTGACGCC
AGGATAATCAAGGTGGCAGCAATTGGATTGAACGAGAAACATTTAGGAATGTCCTTGCCTATGATGCAACCGGTG
TTACAGAAACTTAACCAACTTTATCAAGTTCATATCTGTGGAGAGGGTGGGGAGTTTGAGACAATGGTCTTGGAT
GCCCCTTTTTTCCAACATGGATACTTGGAATTAATTGATATTGTCAAATGTAGCGATGGTGAAGTTCACAATGCA
AGGTTAAAGGTAAAATTTCAACCGCGTAATTTGAGCAAAAGTTTTTTACTCAACCAATTAGATCAATTACCTGTA
CCTTCGATTTTTGGTAATAATTGGCAAGATTTAACCCAAAATTTGCCAAAGCAACAAGCTAAAACAGGAGAACAA
AGATTTGAAAATCACATGTCGAATGCTCTACCACAAACGACAATCAATAAAACCAATGATAAGTTGTACATCTCT
AACCTACAATCACGTAAAAGTGAGACGGTCGAGAAGCAAAGTGAAGATATTTTTACTGAGCTAGCCGACATTCTG
CATTCCAACCAGATTCCACGGAATCATATTTTATCTGCGTCATTATTAATTAGAGATATGTCTAATTTCGGAAAA
ATCAACAAAATATACAATGAATTTTTAGATTTGTCAAAATACGGACCTTTGCCACCATCAAGAGCATGCGTAGGT
TCGAAGTGCTTGCCAGAAGATTGTCACGTACAGTTATCAGTTGTTGTTGATGTAAAAAATACAGGCAAAGAAAAA
ATCAACAAGAATAAGGGCGGACTACATGTTCAAGGCCGTTCATATTGGGCTCCATGTAATATCGGACCATATTCC
CAAAGTACTTGGTTAAACGATGATGCAAACCAAGTCAGTTTTATCAGTGGGCAAATCGGACTAGTACCGCAGTCA
ATGGAAATATTAGGAACACCTCTCACTGACCAAATAGTTTTGGCATTACAACATTTTGATACTTTGTGTGAAACA
ATAGGAGCTCAAGAAAAGTTACTCATGACATGCTATATTTCAGACGAATCGGTTCTCGACTCTGTCATCAAAACA
TGGGCGTTTTATTGTTCAAATATGAATCATAGATCAGATCTATGGATGGACAAATCTGATGATGTTGAAAAATGC
CTAGTTCTTGTAAAGATTTCTGAATTACCTAGAGGCGCTGTTGCAGAATTTGGTGGTGTTACTTGTAAGAGATTA
ATAGTGGACGATAATGATTCCGACAAGAAAGAAAGGGAAGAGAATGATGATGTTTCAACCGTATTCCAGAAATTG
AATTTGAATATTGAAGGTTTTCATAACACTACAGTTAGTGCGTTTGGATATAATAGAAACTTTATTACGGGATTT
GTAGATAGTAGAGAGGAGTTAGAACTTATCTTGGAAAAAACCCCAAAATCAGCCCAGATTACCCTCTATTATAAT
CCCAAAGAAATCATCACTTTCCACCATCACATTGGATACTATCCGGTGGAGAAACTTTTTGATTACCGTGGTAAA
GAACACCGCTTTGGGTTGCATATTCGTTCCTAA

>YOR386W 0.73   Cold
ATGAAAAGAACGGTAATATCTTCCTCGAACGCATATGCTAGTAAGAGATCTCGGCTGGATATAGAACATGATTTT
GAACAATACCACTCGTTGAACAAAAAATATTATCCAAGGCCAATTACTAGGACAGGCGCAAATCAATTCAATAAT
AAGTCTCGGGCTAAACCAATGGAGATTGTGGAAAAACTGCAAAAGAAGCAAAAAACGTCGTTTGAGAATGTTAGC
ACTGTCATGCACTGGTTTCGAAATGATCTACGTTTATATGATAATGTGGGACTGTACAAAAGTGTTGCGCTCTTC
CAGCAATTGAGGCAAAAAAACGCAAAAGCCAAATTATATGCTGTTTATGTCATCAATGAAGATGATTGGAGAGCC
CATATGGATAGCGGATGGAAATTGATGTTTATAATGGGGGCGTTAAAAAATTTGCAGCAGTCCTTAGCCGAATTA
CACATACCTCTTCTTCTGTGGGAATTCCACACTCCAAAAAGTACCTTATCTAATTCAAAAGAGTTCGTGGAGTTT
TTCAAAGAAAAATGTATGAATGTAAGTTCAGGAACAGGTACGATAATCACTGCTAATATAGAATACCAAACAGAT
GAACTGTACCGTGATATTAGGCTGTTAGAAAATGAAGACCATAGATTGCAATTGAAATACTACCACGACTCTTGC
ATTGTTGCTCCTGGATTGATCACTACTGACAGAGGCACCAACTATTCTGTGTTTACTCCATGGTACAAAAAATGG
GTGCTATATGTAAATAATTACAAAAAGAGTACCTCTGAAATTTGTCATTTGCATATAATCGAACCATTAAAGTAC
AACGAAACTTTTGAACTGAAGCCATTCCAATATTCATTACCGGATGAATTCCTTCAATATATACCTAAATCAAAA
TGGTGCTTGCCAGACGTTTCTGAAGAAGCAGCTTTATCCAGATTAAAAGATTTCCTCGGTACAAAAAGTTCTAAG
TACAACAATGAAAAAGACATGCTATATTTGGGTGGAACCTCTGGGCTAAGTGTATATATAACTACTGGTAGGATA
AGCACAAGACTTATAGTCAACCAGGCATTTCAATCATGCAACGGACAAATAATGTCAAAAGCACTGAAAGATAAT
TCGTCCACCCAAAATTTCATCAAAGAAGTTGCATGGAGAGATTTTTACAGACATTGTATGTGCAACTGGCCATAT
ACCTCAATGGGAATGCCTTATCGATTGGACACTTTAGATATAAAATGGGAGAATAACCCTGTAGCATTTGAAAAG
TGGTGTACTGGTAATACAGGCATTCCCATAGTCGATGCCATAATGAGAAAATTACTGTACACAGGCTATATTAAT
AACAGATCTAGAATGATCACAGCTTCTTTTCTTTCTAAAAACTTATTAATAGATTGGAGATGGGGGGAACGCTGG
TTTATGAAACACTTGATAGACGGTGATTCGTCTTCAAATGTTGGTGGCTGGGGTTTTTGTTCTAGTACAGGAATT
GATGCCCAACCATATTTTAGAGTTTTTAATATGGATATACAAGCAAAAAAATATGACCCACAAATGATATTCGTC
AAACAATGGGTTCCCGAATTGATTTCTTCCGAAAATAAGCGTCCAGAAAACTATCCAAAACCTTTAGTTGATTTA
AAGCATAGTAGGGAGCGTGCTTTGAAAGTTTATAAAGACGCAATGTGA

>YPL237W 0.82   Cold
ATGTCCTCCGATTTAGCTGCTGAGTTAGGATTCGACCCTGCACTAAAGAAGAAAAAGAAGACTAAGAAGGTGATC
CCAGATGATTTTGATGCTGCCGTAAACGGCAAAGAAAATGGTTCAGGAGATGATTTATTTGCCGGATTAAAAAAG
AAAAAGAAGAAGTCCAAGAGCGTTTCTGCCGATGCTGAAGCTGAAAAAGAGCCTACTGACGACATAGCAGAAGCC
TTGGGTGAACTATCCTTGAAGAAGAAAAAGAAAAAGACAAAGGACAGCAGTGTAGACGCGTTTGAGAAAGAACTA
GCTAAAGCTGGTCTAGATAATGTGGATGCTGAAAGCAAAGAAGGCACTCCAAGTGCTAATTCCAGCATTCAACAA
GAAGTTGGCCTACCTTATTCAGAGTTATTATCTAGATTTTTCAATATTCTAAGAACTAACAATCCAGAACTTGCT
GGTGATAGAAGTGGTCCAAAGTTCAGAATTCCTCCTCCTGTTTGTTTGCGTGATGGTAAGAAGACTATTTTCTCG
AATATCCAAGATATCGCCGAAAAATTGCATAGATCTCCGGAACATTTGATTCAATATCTCTTCGCAGAATTAGGT
ACGTCCGGTTCTGTTGACGGTCAGAAAAGATTAGTCATTAAGGGTAAGTTTCAATCCAAACAAATGGAGAATGTC
TTAAGAAGATACATTTTGGAGTATGTCACTTGTAAAACTTGTAAGAGTATTAACACCGAATTGAAGAGAGAACAG
TCAAACAGACTGTTCTTTATGGTCTGTAAAAGTTGTGGTTCTACCAGATCCGTCTCTTCTATTAAAACCGGTTTC
CAAGCTACCGTTGGTAAGAGAAGGAGAATGTGA

>YDR020C 0.79   Cold
ATGGACAGAAAAGCTGTCGAAGAAAAAAGAATTGTTATTTCTATCGGTGGAGGGCATGCTACTGGTGTTGGAGCA
ATTGCACTGGATTTACAAAATACTTTCAAAAGCCTATATAACTCTATCAATATAAGGGTAATAAACCTAGATAAT
ATGATAGAAGGGAACATCAAGAGCTACAATAATAACGACTATGATTTTGATAATATCCTTAATTTGGTATATGAA
AAGCACGCTGTAACTTCTCAAAATGACATGATTCAACATGATTACGAGGACCCGATAGATTTGATAATAGTATGT

GGCTGTTATGCGCTATACGATAAACGCATCAACGAAATATCTCAATTAAAAGTATTCTTGGATAGTGATGCAGAT
AAAAGATTAATTAGTCTAATCAAGAAGAAGAACGTAGGGAGCAATGAACAACTAGCCCAGTTGATCACTGAGTAT
ATGGACCATCTGCGCCCGGAGATGCAACAATATATAGAGCCCACTAGAACTTTTGCAGATTTGATCATTCCTTCT
ACAAATGAAAATCTAGGGCGCGCAGTCCTCGTGGACGGTATAGTTAAAGCAATCGAAGATACCAAAAGCCAAATA
GAAGGCAACAATACGAACAACAAAATACGCCCACGTTTATGGGATTTCGAAGCTGAAACAATGGACTTGGAGAAA
GATAGATATTACGATCTGTCATGA

>YOR015W 0.79 Cold
ATGCCACATTTCAAAAGAGCAGCAGTATATGAAGAGCAAAAAAGAACTGGTAAATGGGGTCAACTTGTTGAAGAA
ACCAAAGATAGAATCCCCGAATATTCGAACAAAACTATAGCAAAGATAAGCCACTTGGACAATGGTTGTTTGTGG
CCTGAGATAAAGGTCTCTTTTTCTCATCATTTGAGCATACTTCAATCGATGTGTCTTCATTTTATTATTTCTATA
CTTTTCTCCAAATATATCTTCGTTTTTCTTTTCGCTTTCCTTCTTCCTTCTGCCTTTCCTCTATTTATTTTGCAC
TCCACTCTTTTCCGTAAACCTTGCCTATCTATTATTGGATTTTTGAAAACTAAAGTATAA

>YOR128C 0.82 Cold
ATGGATTCTAGAACAGTTGGTATATTAGGAGGGGGACAATTGGGACGTATGATTGTTGAGGCAGCAAACAGGCTC
AACATTAAGACGGTAATACTAGATGCTGAAAATTCTCCTGCCAAACAAATAAGCAACTCCAATGACCACGTTAAT
GGCTCCTTTTCCAATCCTCTTGATATCGAAAAACTAGCTGAAAAATGTGATGTGCTAACGATTGAGATTGAGCAT
GTTGATGTTCCTACACTAAAGAATCTTCAAGTAAAACATCCCAAATTAAAAATTTACCCTTCTCCAGAAACAATC
AGATTGATACAAGACAAATATATTCAAAAAGAGCATTTAATCAAAAATGGTATAGCAGTTACCCAAAGTGTTCCT
GTGGAACAAGCCAGTGAGACGTCCCTATTGAATGTTGGAAGAGATTTGGGTTTTCCATTCGTCTTGAAGTCGAGG
ACTTTGGCATACGATGGAAGAGGTAACTTCGTTGTAAAGAATAAGGAAATGATTCCGGAAGCTTTGGAAGTACTG
AAGGATCGTCCTTTGTACGCCGAAAAATGGGCACCATTTACTAAAGAATTAGCAGTCATGATTGTGAGATCTGTT
AACGGTTTAGTGTTTTCTTACCCAATTGTAGAGACTATCCACAAGGACAATATTTGTGACTTATGTTATGCGCCT
GCTAGAGTTCCGGACTCCGTTCAACTTAAGGCGAAGTTGTTGGCAGAAAATGCAATCAAATCTTTTCCCGGTTGT
GGTATATTTGGTGTGGAAATGTTCTATTTAGAAACAGGGGAATTGCTTATTAACGAAATTGCCCCAAGGCCTCAC
AACTCTGGACATTATACCATTGATGCTTGCGTCACTTCTCAATTTGAAGCTCATTTGAGATCAATATTGGATTTG
CCAATGCCAAAGAATTTCACATCTTTCTCCACCATTACAACGAACGCCATTATGCTAAATGTTCTTGGAGACAAA
CATACAAAAGATAAAGAGCTAGAAACTTGCGAAAGAGCATTGGCGACTCCAGGTTCCTCAGTGTACTTATATGGA
AAAGAGTCTAGACCTAACAGAAAAGTAGGTCACATAAATATTATTGCCTCCAGTATGGCGGAATGTGAACAAAGG
CTGAACTACATTACAGGTAGAACTGATATTCCAATCAAAATCTCTGTCGCTCAAAAGTTGGACTTGGAAGCAATG
GTCAAACCATTGGTTGGAATCATCATGGGATCAGACTCTGACTTGCCGGTAATGTCTGCCGCATGTGCGGTTTTA
AAAGATTTTGGCGTTCCATTTGAAGTGACAATAGTCTCTGCTCATAGAACTCCACATAGGATGTCAGCATATGCT
ATTTCCGCAAGCAAGCGTGGAATTAAAACAATTATCGCTGGAGCTGGTGGGGCTGCTCACTTGCCAGGTATGGTG
GCTGCAATGACACCACTTCCTGTCATCGGTGTGCCCGTAAAAGGTTCTTGTCTAGATGGAGTAGATTCTTTACAT
TCAATTGTGCAAATGCCTAGAGGTGTTCCAGTAGCTACCGTCGCTATTAATAATAGTACGAACGCTGCGCTGTTG
GCTGTCAGACTGCTTGGCGCTTATGATTCAAGTTATACAACGAAAATGGAACAGTTTTTATTAAAGCAAGAAGAA
GAAGTTCTTGTCAAAGCACAAAAGTTAGAAACTGTCGGTTACGAAGCTTATCTAGAAAACAAGTAA

>YEL014C 0.78 Cold
ATGACAGCACTATTCTGCCTTGAATTGAGAACTAATATATTTCTAATAATGAATGATTGTATTATTATCAATTAC
TGGAAAGGGTTCATTTTTTCTTTCCACTCGTATTTTTTTCCTTTTCGTTTTGAGTCCTCCTTAAGGGCTCATTAC
CCGGGAAAGAGAAATTATTCAGATTTTTCTGTTATACCGTTACCTTATTATATAGATGTTCGCAGTTTTCATATT
TGTGAGAGCCAACACATTATTGCATTACCGCTACAGATCCCACTTCCTTACCGTATGCTTATACGTATGTATCCA
GTTTAG

>YPL009C 0.81 Cold
ATGAAGCAAAGAATTAGTGCCCTAGATTTACTGCTGTTGGCTAGAGAGCTGAAACAGGATTTGGAAGGCTACCGA
TTGAGTAATATATATAACATTGCCGATTCCTCAAAACAATTTTTGCTGAAATTTAATAAACCAGATTCTAAACTC
AATGTTGTTGTCGATTGTGGCTTAAGAATTTACTTAACTGAATTTAGTAGGCCAATACCTCCAACTCCTTCCGGC
TTTGTTGTCAAACTAAGGAAGCATCTTAAAGCTAAACGGTTAACAGCTTTAAAGCAAGTAGACCAGGATAGAATT
CTTGTTCTACAATTTGCTGATGGCCACTTCTATTTAGTTTTGGAATTTTTCAGTGCAGGGAATGTCATTCTTCTA
GATGAGAACAGAAGAATTATGGCTTTGCAAAGAGTCGTTCTAGAGCATGAAAATAAGGTCGGACAAATTTATGAA
ATGTTTGACGAATCACTTTTTACCACAAATAACGAGTCTGCTGATGAATCCATAGAAAAAAACAGAAAGGCAGAA
TACACCTCTGAACTAGTCAACGAGTGGATAAAAGCAGTTCAAGCCAAGTATGAATCAGACATTACTGTTATTAAG
CAACTTAACATTCAAGGGAAGGAGGGCGCTAAAAAAAAAAAAAGTGAAAGTACCTTCCATCCATAAACTGCTGCTT
TCGAAAGTCCCTCATTTGTCTTCAGATTTATTATCAAAGAACCTTAAGGTATTCAATATCGACCCTTCAGAGTCT
TGTTTAAATCTTTTGGAGGAAACAGACTCACTCGCCGAATTGCTAAACAGTACTCAGTTAGAATATAATCAACTT
TTAACTACCACCGATAGAAAGGGTTACATTTTGGCCAAAAGAAATGAAAATTATATCTCTGAAAAAGATACTGCC
GATTTGGAATTTATTTATGATACATTCCATCCCTTTAAGCCATATATAAATGGGGGAGACACTGACTCTTCTTGT
ATCATTGAAGTTGAAGGTCCTTACAATAGGACGCTAGACAAATTTTTCTCAACAATAGAATCCTCTAAGTATGCT
CTACGTATACAAAATCAGGAATCGCAAGCGCAAAAGAAAATCGATGATGCTCGTGCCGAAAATGATAGAAAAATA
CAAGCTTTATTAGACGTCCAAGAACTAAATGAAAGGAAAGGCCATCTCATAATTGAAAATGCACCCTTAATAGAG
GAAGTTAAGCTTGCTGTTCAGGGTCTAATCGATCAACAAATGGACTGGAACACTATTGAGAAACTAATTAAAAGT
GAACAAAAAAAAGGCAACAGAATTGCTCAGCTTTTGAACTTGCCTTTGAACTTGAAACAGAACAAAATTAGCGTC
AAGCTTGATCTCTCTAGCAAGGAACTCAACACTTCTTCAGACGAGGACAATGAATCAGAAGGCAATACTACTGAT
AGTTCTAGTGACTCAGACTCAGAAGACATGGAGAGTTCGAAGGAGAGGAGTACAAAAAGTATGAAAAGAAAATCT

AATGAAAAGATAAACGTTACAATTGACCTTGGTTTATCAGCTTATGCCAATGCCACTGAATATTTCAATATCAAA
AAAACAAGTGCTCAAAAGCAGAAAAAAGTCGAAAAGAATGTCGGCAAGGCTATGAAGAATATTGAAGTCAAAATT
GATCAACAATTGAAAAAAAAGCTAAAAGATTCTCACAGTGTCTTAAAAAAGATCCGCACTCCTTACTTTTTTGAA
AAATATAGTTGGTTTATTTCAAGTGAAGGATTTTTGGTCATGATGGGTAAAAGTCCGGCTGAGACAGATCAAATC
TATTCAAAATATATTGAAGATGATGATATTTATATGTCCAATAGTTTTAACTCTCATGTATGGATCAAAAATCCA
GAAAAAACAGAAGTTCCACCGAACACTTTGATGCAAGCGGGTATACTATGCATGTCCTCAAGCGAGGCTTGGTCG
AAGAAGATATCTTCTTCTCCATGGTGGTGCTTTGCAAAGAATGTTAGTAAATTCGATGGCTCTGATAACTCCATT
CTGCCAGAAGGTGCCTTTCGGTTGAAAAATGAGAATGATCAAAACCATTTACCCCCTGCTCAACTTGTGATGGGT
TTTGGATTTTTATGGAAAGTGAAAACCAGTGGAAATGAGGATAATGGTGATGATGACGAAGAAGAAGAAGAAGAA
GAAGAGGAAGAAGAAGAAGAAGAAGAAGAAGAAGAAGAAGAAGAAGAAGAAGAAAAAGAAGAAGAAGAAAAAGAA
GAAGAGCAACAGCAAGATGAAGACGATAGTAACGAGGTCAATGGGTTAGAGAAGGGTGGAGATTCAAACGATAGC
ACGAAAAACAACAGTTTTGAACACGATAATCTTGAAAAAGACATCGAAAAGCATTGCACCATATCATCCGATACC
GACTCTGATTCTGGAAATGCTAAAGCCAAAAATGACAATAGCAGTACGCAGCGTATCCTTGATGAACCAGGTGTT
CCGATATCTTTAATCGAAAACATAAACAGTAACGTCCGTGGTAAAAGAGGAAAATTGAAGAAAATTCAAAAGAAA
TACGCCGACCAGGATGAAACTGAACGTCTCTTGCGTTTGGAGGCCTTGGGTACTTTGAAGGGTATCGAAAAGCAG
CAACAAAGGAAGAAAGAAGAAATAATGAAGCGAGAAGTTAGAGAAGATAGAAAGAATAAACGCGAGAAACAAAGA
AGACTACAGGCTTTAAAATTCACCAAAAAAGAGAAAGCAAGAGTGAACTATGATAAACACAAGTCCGAATTAAAA
CCATCTCTCGATAAGGGCGATGTGGTTGATGATATTATACCAGTATTTGCCCCCTGGCCTGCGCTTTTGAAATAT
AAATACAAAGTCAAAATACAACCGGGAAGTGCGAAGAAAACTAAGACTTTAACTGAAATTTTACATTATTTCAAG
AGTAGACCACTAGACGGTTCTTCAACTGACAACGAAATGGATTGGCCCCAAGAACATGAAATGATTAAAGGCTTG
AAGGAACAGGACTTGGTTCTTCTGCTCTGTGTCGACAAGTTAAAAGTAACAATCGCTGGGCAGAAATCCACCAAG
AACGGTGGTAACTCTTCGAAGAAGGGCAAAAAAAAACGCTAA

>YCL069W 0.74 Cold
ATGAATATGCTCATTGTCGGTAGAGTTGTTGCTAGTGTTGGGGGAAGCGGACTTCAAACGCTTTGCTTTGTTATT
GGTTGTACGATGGTTGGTGAAAGGTCACGTCCATTGGTGATTTCCATCCTAAGTTGTGCATTTGCTGTAGCTGCT
ATCGTTGGTCCTATAATCGGAGGTGCCTTTACAACCCATGTTACCTGGAGGTGGTGCTTCTATATCAATCTTCCT
ATCGGTGGTCTTGCCATTATTATGTTTTTACTCACATATAAGGCCGAGAATAAGGGTATACTTCAACAAATTAAA
GATGCTATAGGAACAATCTCGAGCTTTACTTTTAGTAAGTTCAGACACCAAGTTAATTTTAAAAGACTTATGAAT
GGCATAATCTTCAAGTTTGACTTCTTTGGTTTTGCCCTCTGCTCTGCAGGGCTGGTCCTTTTCCTACTGGGGCTA
ACCTTTGGTGGTAATAAATATAGTTGGAACTCTGGCCAAGTCATCGCATATTTGGTTTTGGGTGTCTTACTTTTT
ATTTTTTCATTGGTGTACGATTTCTTCTTATTCGATAAATTCAACCCGGAACCTGATAATATATCCTACAGGCCT
CTCCTTCTAAGAAGATTGGTAGCAAAACCAGCCATAATAATAATAAACATGGTAACATTTCTATTATGTACCGGT
TACAATGGGCAAATGATATACTCTGTCCAGTTTTTCCAACTTATATTTGCGTCGAGTGCATGGAAAGCCGGTCTT
CACTTGATACCAATCGTTATTACCAACGTTATTGCGGCCATTGCAAGTGGTGTGATTACCAAAAAGCTCGGTTTA
GTTAAACCACTCTTAATATTTGGAGGCGTTCTTGGGGTAATTGGAGCAGGGCTTATGACACTTATGACAAATACG
TCCACGAAGTCAACTCAAATTGGTGTTTTGCTATTACCGGGGTTTTCCCTTGGATTTGCTCTACAAGCATCGCTC
ATGAGTGCACAGCTTCAAATTACCAAAGATCGTCCAGAAGCTGCTATGGACTTTATTGAAGTAACAGCTTTCAAT
ACATTCATGAAGTCATTAGGTACAACTCTTGGTGGTGTGCTTTCAACCACTGTTTTTTCCGCCTCCTTTCACAAC
AAAGTATCACGAGCTCATCTAGAGCCTTACGAAGGAAAAACGGTTGATGACATGATTTTGTATCGTCTTCAAAAC
TACGACGGTTCTCATTCGACTATTGGAAACATTTTAAGCGACTCCATTAAGAACGTATTTTGGATGGATCTAGGG
TTTTATGCCTTAGGATTTTTGTTTTGTAGTTTTTCATCCAATAAGAAATTAATCATACCAAAAAAGGACGAGACA
CCAGAAGATAATTTAGAAGACAAGTAG

>YNL005C 0.78 Cold
ATGTGGAATCCTATTTTACTAGATACTTCTAGTTTTTCATTCCAAAAACATGTGTCTGGTGTGTTCCTTCAAGTG
CGTAATGCCACCAAAAGGGCTGCGGGCTCGAGAACAAGTATGAAGGATTCTGCAGGAAGAAGGTTAGGACCAAAA
AAATATGAAGGCCAGGATGTTTCCACAGGTGAAATTATTATGAGGCAAAGAGGAACAAAGTTTTATCCTGGAGAA
AACGTTGGTATAGGGAAAGATCATTCCATATTCGCCTTAGAACCTGGTGTTGTTCGTTACTATCTTGATCCTTTT
CATCCTAAAAGAAAATTCATTGGTGTTGCCTTGAGGCGTGATTTGAAACTGCCATCTCCTCATTTTGAGCCTACC
GTAAGGAGGTTTGGCCGTTTTGAGTTAACAAATAAAAGGGCAGCATATAAGGAAGAAAATTCTATTTCTAGAAAA
GACTATCTCGCAAAACCTAATATTTTGAAGCAGTTAGAGGTCAGGGAATCTAAAAGAAAGGAGTTACAAGACAAG
TTGAGTAAGGTTCTTCGGGATGAACTTAAGTTAGATATAAAAGACATCGAATTGGCAACGTCATACTTAATTCGT
GTAAGGGCTTCCCTGAAGAATGGATACCCCATAGAAGATGCAAGGTTCAATAGTAGATATTATTTAAAAGAAGAA
GAGCGTTTGAAGGCAAGGAGAGAAAGCTGGACGAATGAGAAATTGTCGGAGAGTCTATCCAAGATTGATGAGTGT
AGTGATCTTTTGAACTCATCTACATCTTTCAATAACAAGCTAGAGCTACACCAGTATATTTCGGAACAAGAAAAA
CAAGCTTTGAAGGCAAAACTATTAGAGGACCTAGAAAAATCCCAACACTTAGAAACCAAAAAAGACAAGAATTAT
ATTAAGGCGCTTTTCAAGGACGCTTGTAATTTTTTGACTTTATCTGAAGAAGTACATTTGCGTAGGAAATACTTA
AAGTCGGTCTTCCCCGAAACAGATAGTACGGTTGAAACCAAAAGCGGGAAGAAGTCAATTGTGTCTAGACGTTTT
GATTACACCAAAAATAAAGTTGAAGTCATTGCTAGAAGTAGGCGGGCTTTTTTGAGCAAGCTTTGA

>YNL182C 0.68 Cold
ATGGATGAGCAAGTTATTTTTACAACAAATACCTCAGGAACAATAGCTTCTGTACACTCATTTGAACAGATAAAT
TTGAGGCAATGCTCCACTCAATCAAGAAATAGCTGTGTTCAAGTAGGAAATAAATACCTTTTTATTGCTCAAGCA
CAAAAAGCATTAATCAATGTCTACAATCTGTCAGGTTCTTTCAAAAGAGAATCTGTTGAACAGCGCTTACCATTA
CCTGAAATCCTAAAATGTCTGGAAGTAGTTGAAAATGATGGTGTGCAGTATGATAGAATTCAAGGTGTCAATCAT
AATTTACCAGACTTCAATCTTCCGTACCTTTTACTTGGCTCCACCGAATCGGGTAAATTGTACATATGGAGTTA

AATTCAGGGATTTTATTGAACGTGAAGCCTATGGCTCATTACCAAAGTATCACCAAGATTAAGTCCATTTTAAAC
GGCAAGTATATTATTACTTCTGGTAACGATTCGAGAGTTATTATATGGCAAACTGTTGACTTGGTATCAGCGTCC
AATGATGATCCTAAGCCTTTATGTATCCTTCACGATCATACTCTACCCGTGACAGATTTCCAAGTTTCTTCTAGT
CAAGGAAAATTTTTATCATGTACTGATACGAAACTCTTCACAGTATCTCAAGATGCTACCATTAGATGCTATGAT
TTGAGTTTAATAGGCAGCAAAAAGAAGCAGAAGGCAAACGAAAATGACGTTAGTATTGGTAAGACCCCAGTATTG
CTTGCGACATTTACAACTCCTTATTCTATCAAATCCATTGTACTGGATCCTGCTGACAGAGCATGCTATATTGGT
ACTGCGGAAGGTTGTTTTTCATTGAATTTATTTTATAAACTAAAGGGTAATGCTATCGTTAATCTGCTACAGTCC
GCCGGAGTAAACACAGTTCAAAAAGGTAGGGTTTTTTCCCTAGTGCAACGTAACTCACTAACTGGCGGCGAAAAT
GAAGATTTGGATGCACTATATGCAATGGGCCAACTTGTCTGTGAGAATGTCCTAAATTCAAATGTGTCATGCCTA
GAAATATCAATGGATGGTACATTATTATTGATCGGTGATACGGAGGGGAAAGTTTCTATTGCGGAAATTTACTCA
AAACAAATCATTAGAACTATCCAAACTTTAACTACATCACAGGATTCAGTTGGAGAAGTGACCAATCTCTTAACC
AACCCTTACAGACTCGAACGTGGAAATTTACTTTTTGAAGGAGAATCCAAAGGCAAACAACCTAGTAATAATAAT
GGTCACAATTTTATGAAGATACCAAACTTACAAAGAGTTATCTTTGATGGTAAAAACAAAGGCCATTTACACGAT
ATTTGGTATCAGATAGGAGAACCAGAAGCAGAGACAGATCCTAACCTCGCATTACCACTTAACGACTTTAATGCC
TATTTGGAGCAGGTCAAAACGCAAGAATCGATATTTTCACATATCGGTAAGGTGTCAAGCAATGTAAAAGTGATT
GACAATAAAATCGACGCCACTTCATCTTTAGACAGCAATGCCGCTAAAGATGAGGAAATTACAGAACTTAAGACC
AACATAGAAGCATTAACTCATGCCTACAAGGAGTTACGTGACATGCACGAAAAGCTGTACGAGGAACACCAACAG
ATGCTTGACAAGCAATAA

>YNL182C 0.68 Cold
ATGGATGAGCAAGTTATTTTTACAACAAATACCTCAGGAACAATAGCTTCTGTACACTCATTTGAACAGATAAAT
TTGAGGCAATGCTCCACTCAATCAAGAAATAGCTGTGTTCAAGTAGGAAATAAATACCTTTTTATTGCTCAAGCA
CAAAAAGCATTAATCAATGTCTACAATCTGTCAGGTTCTTTCAAAAGAGAATCTGTTGAACAGCGCTTACCATTA
CCTGAAATCCTAAAATGTCTGGAAGTAGTTGAAAATGATGGTGTGCAGTATGATAGAATTCAAGGTGTCAATCAT
AATTTACCAGACTTCAATCTTCCGTACCTTTTACTTGGCTCCACCGAATCGGGTAAATTGTACATATGGGAGTTA
AATTCAGGGATTTTATTGAACGTGAAGCCTATGGCTCATTACCAAAGTATCACCAAGATTAAGTCCATTTTAAAC
GGCAAGTATATTATTACTTCTGGTAACGATTCGAGAGTTATTATATGGCAAACTGTTGACTTGGTATCAGCGTCC
AATGATGATCCTAAGCCTTTATGTATCCTTCACGATCATACTCTACCCGTGACAGATTTCCAAGTTTCTTCTAGT
CAAGGAAAATTTTTATCATGTACTGATACGAAACTCTTCACAGTATCTCAAGATGCTACCATTAGATGCTATGAT
TTGAGTTTAATAGGCAGCAAAAAGAAGCAGAAGGCAAACGAAAATGACGTTAGTATTGGTAAGACCCCAGTATTG
CTTGCGACATTTACAACTCCTTATTCTATCAAATCCATTGTACTGGATCCTGCTGACAGAGCATGCTATATTGGT
ACTGCGGAAGGTTGTTTTTCATTGAATTTATTTTATAAACTAAAGGGTAATGCTATCGTTAATCTGCTACAGTCC
GCCGGAGTAAACACAGTTCAAAAAGGTAGGGTTTTTTCCCTAGTGCAACGTAACTCACTAACTGGCGGCGAAAAT
GAAGATTTGGATGCACTATATGCAATGGGCCAACTTGTCTGTGAGAATGTCCTAAATTCAAATGTGTCATGCCTA
GAAATATCAATGGATGGTACATTATTATTGATCGGTGATACGGAGGGGAAAGTTTCTATTGCGGAAATTTACTCA
AAACAAATCATTAGAACTATCCAAACTTTAACTACATCACAGGATTCAGTTGGAGAAGTGACCAATCTCTTAACC
AACCCTTACAGACTCGAACGTGGAAATTTACTTTTTGAAGGAGAATCCAAAGGCAAACAACCTAGTAATAATAAT
GGTCACAATTTTATGAAGATACCAAACTTACAAAGAGTTATCTTTGATGGTAAAAACAAAGGCCATTTACACGAT
ATTTGGTATCAGATAGGAGAACCAGAAGCAGAGACAGATCCTAACCTCGCATTACCACTTAACGACTTTAATGCC
TATTTGGAGCAGGTCAAAACGCAAGAATCGATATTTTCACATATCGGTAAGGTGTCAAGCAATGTAAAAGTGATT
GACAATAAAATCGACGCCACTTCATCTTTAGACAGCAATGCCGCTAAAGATGAGGAAATTACAGAACTTAAGACC
AACATAGAAGCATTAACTCATGCCTACAAGGAGTTACGTGACATGCACGAAAAGCTGTACGAGGAACACCAACAG
ATGCTTGACAAGCAATAA

>YDR325W 0.75 Cold
ATGCAAGATCCTGATGGTATTGACATTAATACGAAAATCTTTAACTCAGTTGCTGAAGTATTTCAAAAGGCACAG
GGTTCTTATGCAGGACACAGGAAGCATATAGCAGTTTTGAAGAAAATTCAGTCAAAGGCTGTTGAGCAAGGCTAT
GAAGATGCTTTTAACTTTTGGTTCGATAAATTAGTTACTAAGATCCTTCCTCTGAAAAAGAATGAGATTATCGGA
GACAGGATAGTAAAGTTAGTAGCTGCATTTATAGCTTCTTTAGAAAGGGAGTTGATATTGGCCAAAAAACAAAAC
TATAAGCTCACGAATGATGAAGAAGGGATATTCTCAAGGTTCGTCGATCAGTTCATAAGACATGTTTTGCGTGGT
GTGGAAAGCCCTGACAAGAACGTCAGATTTAGAGTTTTACAGTTATTAGCCGTTATAATGGATAATATAGGGGAA
ATCGATGAATCACTTTTCAATTTATTAATATTGTCTTTAAATAAGAGGATTTATGATAGAGAACCAACGGTTAGG
ATACAGGCTGTGTTTTGTTTAACTAAATTTCAGGATGAAGAGCAAACTGAACATTTAACTGAGCTTTCTGATAAT
GAAGAAAATTTTGAAGCTACGAGAACTCTAGTTGCTTCTATCCAGAACGATCCGTCAGCTGAAGTACGGAGGGCT
GCAATGCTGAATTTGATCAATGATAATAATACTAGACCGTATATCTTGGAGAGGGCTAGAGATGTAAACATCGTT
AATAGAAGGCTCGTGTATTCGAGAATTTTGAAATCAATGGGAAGAAAGTGTTTCGATGATATTGAGCCGCATATT
TTTGATCAATTGATTGAGTGGGGTTTAGAAGATAGGGAATTATCAGTGAGAAATGCGTGTAAGAGACTCATTGCT
CATGATTGGTTAAATGCTCTGGATGGCGATTTGATAGAATTACTAGAAAAAATTGGATGTCTCAAGATCCTCAGTG
TGTGTTAAGGCTATAGAAGCACTTTTTCAATCAAGGCCAGATATATTATCTAAAATCAAATTTCCTGAAAGTATT
TGGAAAGACTTTACCGTAGAAATTGCCTTCCTCTTTCGGGCTATTTATTTGTACTGTTTGGATAATAATATAACA
GAAATGCTGGAAGAAAACTTTCCAGAAGCCTCAAAATTATCCGAGCATTTAAACCATTATATTCTTCTCAGATAT
CATCACAACGACATTTCTAATGACTCTCAGTCGCATTTTGATTATAACACTTTAGAGTTTATTATTGAGCAACTA
TCGATTGCCGCCGAAAGGTATGATTATAGCGATGAGGTTGGAAGGAGATCGATGCTTACAGTGGTACGAAATATG
CTGGCCTTAACTACACTCTCCGAACCTCTTATTAAAATTGGTATTCGTGTAATGAAAAGTCTGTCCATAAATGAA
AAAGATTTTGTAACAATGGCAATAGAAATCATTAATGATATTAGAGACGACGATATTGAAAAACAAGAACAAGAA
GAGAAAATAAAAAGCAAGAAGATTAATCGCAGAAATGAGACTTCCGTCGATGAAGAGGACGAAAACGGCACACAT
AATGACGAAGTTAACGAGGATGAAGAAGACGACAATATTTCATCCTTCCATTCTGCTGTAGAAAACTTAGTGCAG

```
GGAAACGGCAACGTATCTGAGAGTGACATAATAAATAATCTCCCACCCGAAAAGGAAGCGTCCTCAGCAACAATT
GTTCTCTGTCTTACAAGGTCATCATATATGCTAGAACTAGTTAACACACCGTTAACAGAAAACATTTTAATTGCG
TCGTTGATGGACACTTTGATCACACCAGCGGTTAGAAATACCGCGCCAAATATTAGGGAGCTTGGTGTCAAGAAC
CTTGGTTTATGTTGTCTCTTGGATGTGAAGTTGGCTATTGATAACATGTACATCTTAGGTATGTGCGTTTCGAAA
GGTAATGCATCATTAAAGTATATTGCGTTACAAGTCATTGTAGATATTTTTTCCGTACATGGGAACACTGTGGTA
GACGGAGAAGGCAAAGTTGACTCAATCTCGTTGCACAAAATATTTTACAAGGTTTTAAAGAATAACGGTTTACCG
GAATGTCAGGTGATAGCAGCGGAGGGTTTATGCAAACTATTTTTGGCAGACGTGTTCACTGATGATGATTTGTTT
GAAACGTTGGTTTTGTCATATTTTTCGCCGATAAATTCCTCAAACGAAGCGCTGGTACAGGCATTTGCCTTCTGC
ATTCCAGTCTATTGTTTTTCACATCCTGCTCATCAACAACGTATGTCTAGGACGGCTGCGGACATACTCTTAAGA
CTATGTGTTCTTTGGGACGATTTACAGAGCTCTGTAATACCTGAGGTAGACCGTGAAGCTATGCTAAAGCCTAAC
ATAATATTTCAACAGTTGCTATTTTGGACTGATCCACGTAACTTAGTTAACCAGACAGGTTCAACAAAAAAAGAT
ACAGTGCAGCTTACATTCTTGATCGATGTGCTCAAAATATACGCTCAAATTGAGAAGAAAGAAATAAAGAAGATG
ATCATCACTAATATAAACGCTATATTTCTTTCTTCTGAACAAGATTATTCTACTTTGAAAGAACTTCTTGAGTAT
TCTGACGATATTGCAGAAAATGATAATTTAGACAATGTTAGCAAAAATGCTCTGGACAAGCTAAGGAATAATTTG
AATTCGCTGATTGAAGAGATCAATGAAAGGTCAGAAACTCAGACAAAAGATGAGAACAACACTGCGAATGACCAA
TACTCGTCTATTTTGGGGAATTCATTCAATAAATCTTCAAATGACACCATAGAACACGCTGCTGATATAACTGAT
GGAAATAACACAGAATTGACTAAAACAACTGTTAATATTTCGGCAGTTGACAATACAACAGAGCAAAGTAACTCA
AGGAAAAGAACGAGATCAGAAGCGGAGCAAATTGACACATCCAAAAACCTGGAAAACATGAGTATTCAAGACACG
TCAACTGTAGCAAAAAATGTAAGTTTTGTTTTACCTGACGAGAAATCAGATGCAATGTCCATAGATGAAGAAGAT
AAGGATTCAGAGTCTTTCAGCGAGGTCTGTTAA
```

© 2014 by the authors; licensee MDPI, Basel, Switzerland. This article is an open access article distributed under the terms and conditions of the Creative Commons Attribution license (http://creativecommons.org/licenses/by/3.0/).
